# Pediatric glioma immune profiling identifies TIM3 as a therapeutic target in BRAF fusion pilocytic astrocytoma

**DOI:** 10.1172/JCI177413

**Published:** 2024-08-13

**Authors:** Shashwat Tripathi, Hinda Najem, Corey Dussold, Sebastian Pacheco, Ruochen Du, Moloud Sooreshjani, Lisa Hurley, James P. Chandler, Roger Stupp, Adam M. Sonabend, Craig M. Horbinski, Rimas V. Lukas, Joanne Xiu, Giselle Lopez, Theodore P. Nicolaides, Valerie Brown, Nitin R. Wadhwani, Sandi K. Lam, Charles David James, Ganesh Rao, Maria G. Castro, Amy B. Heimberger, Michael DeCuypere

**Affiliations:** 1 Department of Neurological Surgery,; 2 Malnati Brain Tumor Institute of the Robert H. Lurie Comprehensive Cancer Center, and; 3 Department of Pathology, Feinberg School of Medicine, Northwestern University, Chicago, Illinois, USA.; 4 Caris Life Sciences, Phoenix, Arizona, USA.; 5 Department of Pediatrics, Penn State Cancer Institute, Hershey, Pennsylvania, USA.; 6 Department of Pathology and Laboratory Medicine,; 7 Division of Neurosurgery, Ann and Robert H. Lurie Children’s Hospital of Chicago, Chicago, Illinois, USA.; 8 Department of Neurosurgery, Baylor College of Medicine, Houston Texas, USA.; 9 Department of Neurological Surgery, University of Michigan Medical School, Ann Arbor, Michigan, USA.

**Keywords:** Immunology, Oncology, Brain cancer, Cancer immunotherapy

## Abstract

Despite being the leading cause of cancer-related childhood mortality, pediatric gliomas have been relatively understudied, and the repurposing of immunotherapies has not been successful. Whole-transcriptome sequencing, single-cell sequencing, and sequential multiplex immunofluorescence were used to identify an immunotherapeutic strategy that could be applied to multiple preclinical glioma models. MAPK-driven pediatric gliomas have a higher IFN signature relative to other molecular subgroups. Single-cell sequencing identified an activated and cytotoxic microglia (MG) population designated MG-Act in BRAF-fused, MAPK-activated pilocytic astrocytoma (PA), but not in high-grade gliomas or normal brain. T cell immunoglobulin and mucin domain 3 (TIM3) was expressed on MG-Act and on the myeloid cells lining the tumor vasculature but not normal brain vasculature. TIM3 expression became upregulated on immune cells in the PA microenvironment, and anti-TIM3 reprogrammed ex vivo immune cells from human PAs to a proinflammatory cytotoxic phenotype. In a genetically engineered murine model of MAPK-driven, low-grade gliomas, anti-TIM3 treatment increased median survival over IgG- and anti–PD-1–treated mice. Single-cell RNA-Seq data during the therapeutic window of anti-TIM3 revealed enrichment of the MG-Act population. The therapeutic activity of anti-TIM3 was abrogated in mice on the CX3CR1 MG–KO background. These data support the use of anti-TIM3 in clinical trials of pediatric low-grade, MAPK-driven gliomas.

## Abstract 2

TIM3 is identified as an immune therapeutic target with relevance to MAPK activated low-grade gliomas

## Introduction

Immunological studies of adult gliomas have been numerous, whereas pediatric gliomas are relatively understudied despite the fact that they are the leading cause of cancer-related childhood mortality (1). Among the pediatric glioma immunology studies conducted to date, the most extensive utilized whole-genome sequencing, RNA-Seq, and proteomics profiling of 218 tumors spanning 7 histological types of childhood brain cancer (low-grade glioma, ependymoma, high-grade glioma, medulloblastoma, ganglioglioma, craniopharyngioma, and atypical teratoid rhabdoid tumor), with results showing diverse immune composition in tumor microenvironments (TMEs), across as well as within histologic groups (2). Results from a study of T cell populations in high- and low-grade pediatric gliomas revealed tumor-resident TCF1^+^ T cells as being located closer to the vasculature, whereas CD103^+^ T cells resided further away from the vasculature (3). This study also compared immune cell infiltrates in primary versus recurrent tumors and found fewer CD103^+^ T cells in recurrent cancer. Differences that have been highlighted in studies of pediatric versus adult brain tumor immunology include the determination of low-level expression of programmed cell death protein ligand 1 (PD-L1) as well as NKG2D ligand (4) and the lack of prognostic significance associated with immunosuppressive CD163^+^ macrophage infiltration in pediatric tumors (5, 6). As such, it has been postulated that the pediatric brain TME may reflect a failure of immune surveillance rather than the establishment of an immunosuppressive environment, as is seen in adult gliomas (7–9).

Many studies have demonstrated that the immune microenvironment of adult glioblastoma is highly immunosuppressive, thereby effectively shielding the tumor from immunological surveillance and eradication (10). Immunological reactivity, including against cancer, is a function of age. In general, there are extensive and comprehensive changes in immune reactivity that include age-related T cell anergy, exhaustion, and senescence; defects in activation of the inflammasome; and decreased innate immunity (11). Specifically, in the context of gliomas, CD8^+^ T cell recent thymic emigrants (RTEs) are at least 1 factor that accounts for the prognostic power of age in clinical outcomes in adult patients with glioblastoma. CD8^+^ T cell RTEs, which are expanded following vaccination, account for most of the tumor antigen–binding cells in the peripheral blood and are associated with clinical outcomes. Preclinical modeling in the mutant CD8β^–/–^ mice reveals an age-specific decrease in glioma host survival as well as a correlation between host survival and thymus cellular production (12). Beyond this, there may be age-dependent aspects of the brain parenchyma that contribute to tumor-related mortality (13, 14). Why cancer can arise in a pediatric setting in which immunological reactivity should be fully optimized for tumor surveillance is a paradox. Given the underlying biological propensity of immune reactivity, pediatric patients with glioma may be predisposed to respond to immune therapies. However, there have been few studies to date that have analyzed immune reactivity and prioritized potential immune therapeutic strategies in pediatric patients. The few studies that have attempted to repurpose adult brain tumor immunotherapies in pediatric patients have not been successful, highlighting the need for pediatric-specific immunotherapies (15–17).

Pilocytic astrocytomas (PAs) are the most common pediatric glioma. Surgical resection of a PA is typically the first-line treatment, and gross total resection is often curative (18, 19). However, PAs that are not amenable to gross total resection have associated long-term morbidity and mortality. The 10-year progression-free survival rate for patients with PA with a radiologically visible residual tumor is less than 50%. Other treatment options are frequently necessary for gliomas with a midline location, which can be challenging to biopsy or resect safely. Notably, some PAs undergo spontaneous regression after partial resection, which possibly suggests tumor immunoreactivity (20–24). PAs can have a single-driver BRAF rearrangement. BRAFV600-expressing melanomas are responsive to immune checkpoint inhibitor therapy, especially those with PD-L1 expression (25). PD-L1 expression and immune cell infiltration are independent of BRAF V600E mutational status (26). KIAA1549-BRAF fusion is the most common rearrangement in PAs, present in almost 70% of PAs and the focus of this study. Although tumor mutational burden (TMB) is associated with response to immune checkpoint inhibitors (ICIs) for various cancer lineages, this is not the case for adult gliomas (27–30). BRAF alterations are not typically associated with a high TMB but may still be highly immunogenic to a sufficient degree to induce spontaneous regression and/or a high propensity for response to immune therapy (31, 32). Single-cell RNA-Seq (scRNA-Seq) data of PAs also indicate that microglia (MG) may constitute a high-frequency immune cell population in these tumors (33). If and how PA MG influence tumor immunoreactivity is unknown. A thorough investigation into the immune microenvironment of PAs may yield information for the development and use of immunotherapy strategies to treat these tumors.

## Results

### Immune therapy response biomarker profiles are enriched in MAPK-driven gliomas.

**Figure 1:**
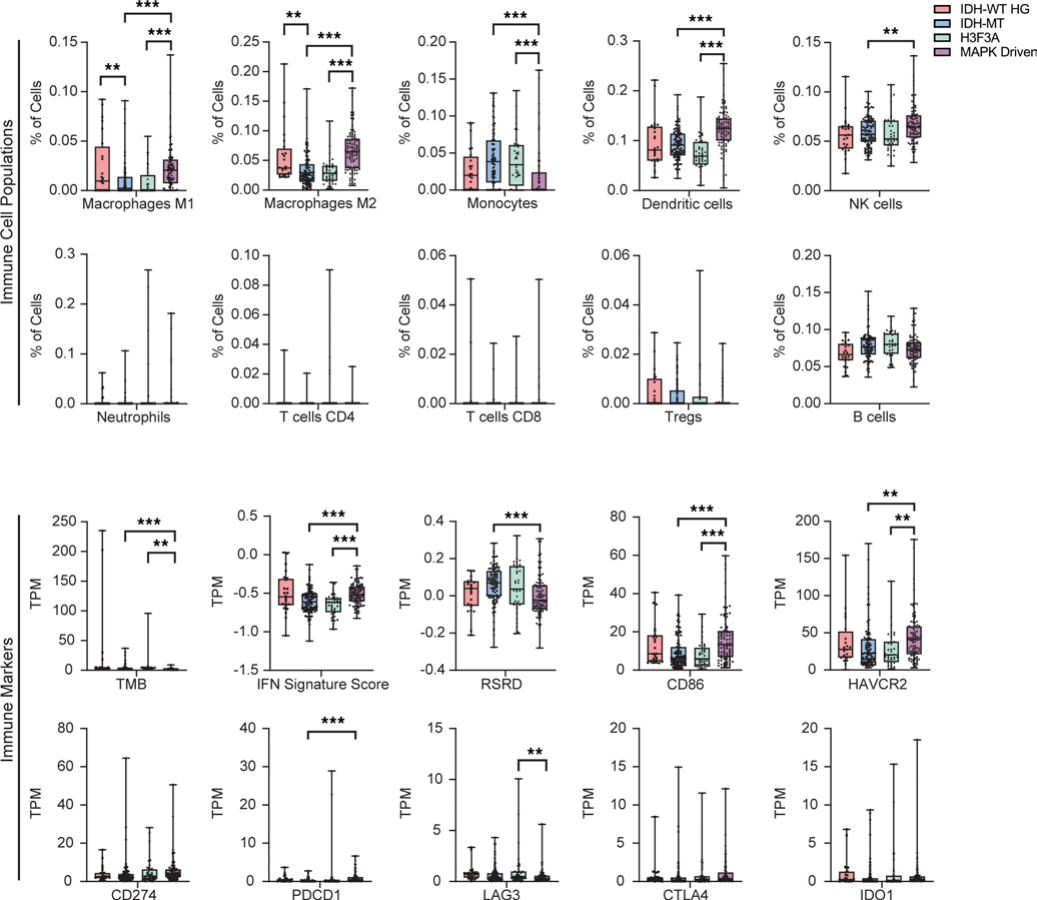
Estimated immune cell fractions in pediatric gliomas based on RNA deconvolution. *n* = 91 IDH-MT; *n* = 24 IDH-WT HG; *n* = 39 H3F3A; *n* = 96 MAPK-driven. Bulk RNA-Seq immune marker signatures of pediatric glioma molecular groups. The box indicates the median, 25%, and 75% percentiles. Whiskers extend to the minimum and maximum. Data indicate the mean ± SEM. **P < 0.01 and ***P < 0.001, by unpaired Student’s *t* test with 2-stage step-up method (with Benjamini correction).

Gliomas from 250 pediatric and young adult (<25 years of age) patients were analyzed using whole-transcriptome sequencing to examine immune microenvironment differences between molecular groups (IDH-WT high-grade [HG], H3F3A, MAPK-driven, and isocitrate dehydrogenase 1–mutant (IDH-MT). IDH-WT HG included both glioblastoma and diffuse pediatric high-grade gliomas. MAPK-driven included PAs with BRAF alterations (BRAFV600E, *n* = 30; BRAF fusion, *n* = 38; BRAF MT other, *n* = 20), diffuse low-grade gliomas with MAPK alterations (*n* = 1), and rosette-forming glioneuronal tumors (*n* = 7). Based on QuantiSeq RNA deconvolution analysis, T cells were rare in gliomas regardless of molecular type classification. In contrast, DCs and macrophages were more frequent in the MAPK-driven tumors (Figure 1). TMB and replication stress response defect (RSRD) scores were low for all glioma groups, but MAPK-driven tumors showed the highest *IFN* expression signatures. The analysis of immune markers in the MAPK-driven group revealed elevated expression of *CD86* and *HAVCR2* (T cell immunoglobulin and mucin domain 3 [TIM3]), but relatively low-level expression of *IDO1*, *PDCD1* (PD-1), *LAG3*, *CTLA4*, and *CD274* (PD-L1).

### BRAF fusion PA commonly expresses TIM3.

**Figure 2:**
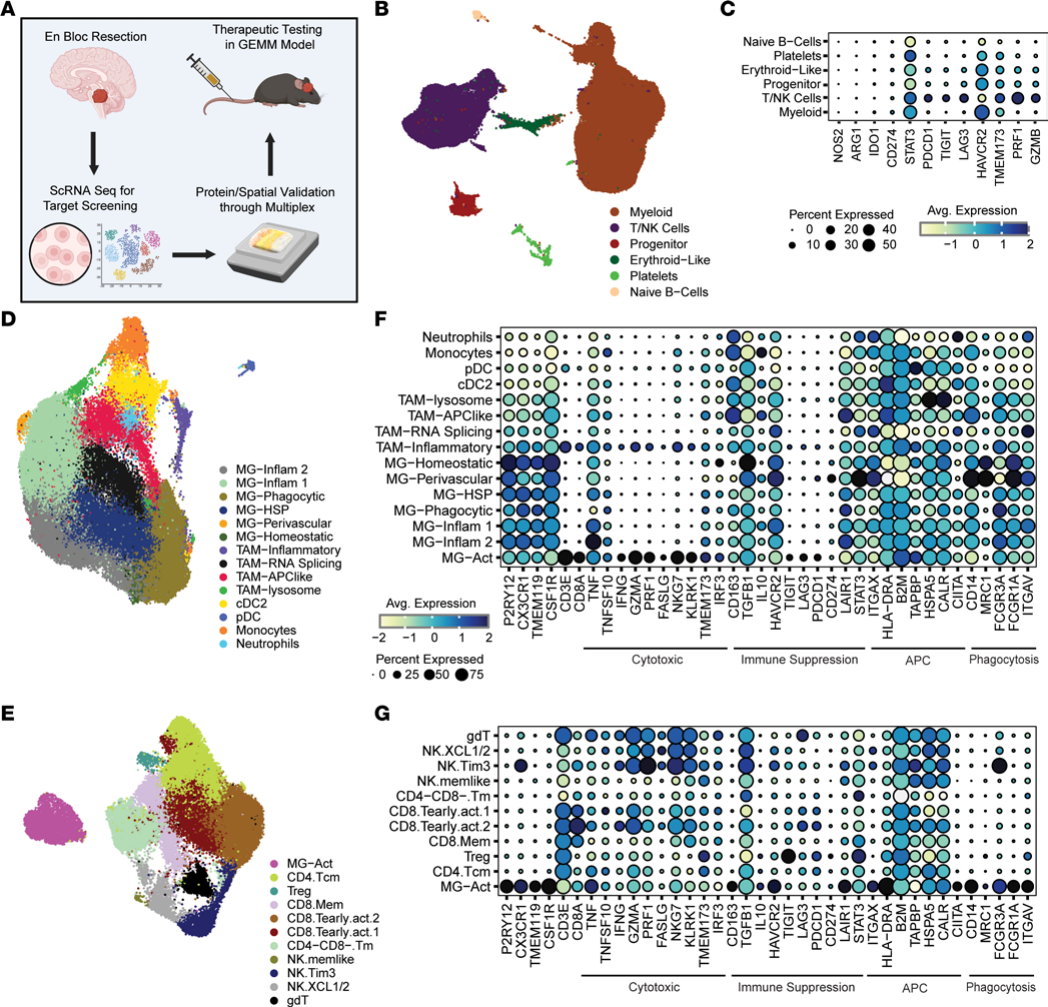
Inflammatory TME present in BRAF fusion PA. (**A**) Schematic diagram of the workflow associated with orthogonal analyses of pediatric gliomas (created with BioRender.com). (**B**) UMAP plot of glioma-infiltrating immune cells analyzed with scRNA-Seq across 16 patients (*n* = 13 BRAF fusion PAs; *n* = 3 ANBs). (**C**) Dot plot of selected immune marker genes within the general immune cell populations. Bubble size corresponds to the percentage of cells expressing a gene marker; colors indicate average (Avg.) expression. (**D**) scRNA-Seq UMAP plot of the intratumoral myeloid cells shown in brown in **B**. (**E**) scRNA-Seq UMAP plot of the intratumoral lymphoid cells shown in purple in **B**. (**F**) Dot plot of immune marker genes within the myeloid subtypes (including the MG-Act cell population from **E**) characterized on the basis of 4 distinct immunological functions: cytotoxicity, immune suppression, antigen-presenting cell (APC), and phagocytosis. Bubble size corresponds to the percentage of cells expressing the gene and colors indicate average expression. (**G**) Dot plot of immune marker genes of the cell clusters shown in **E**, broken into the 4 distinct immunological functions: cytotoxicity, immune suppression, APC, and phagocytosis. Bubble size corresponds to the percentage of cells expressing a gene marker; colors indicate average expression.

Because the BRAF fusion is expressed in 70% of PAs, we prospectively collected patients’ glioma samples to comprehensively characterize the unique immune biology of these tumors using NanoString profiling, scRNA-Seq, and sequential immunofluorescence (seqIF) multiplex staining (Figure 2A). The demographics of this cohort, WHO grade I classification, and clinical annotation are shown in Table 1. Molecular status was reported through the electronic health record (EHR). NanoString analysis of tumor direct enrichment scores (DESs) revealed increases in leukocyte- and NK-associated genes and expression of tumor necrosis factor (TNF) superfamily, IL, and antigen presentation genes relative to adjacent brain (Supplemental Figure 2, B and C; supplemental material available online with this article; https://doi.org/10.1172/JCI177413DS1). Next we profiled PAs at the single-cell level by performing scRNA-Seq on 13 tumors and 3 adjacent normal brain (ANB) specimens that were resected separately during the approach to a brain lesion. We analyzed the transcriptome of a total of 134,576 cells from a total of 16 samples. After applying the scRNA-Seq integration pipeline, we identified lymphoid, myeloid, and 1 CD45^–^ cluster (platelets) (Figure 2B and Supplemental Table 3). We detected negligible expression of *NOS2* (nitric oxide), *ARG1* (arginase), *IDO1*, and *CD274* (PD-L1) in the BRAF fusion PA (Figure 2C). T cell effector function genes such as *PRF1* (perforin) and *GZMB* (granzyme B) showed strong expression, while *PD1, TIGIT,* and *LAG3* were expressed to a lesser degree. STING (*TMEM173*) is expressed in both innate and adaptive immune cells. Among various immune targets, we found that TIM3 (*HAVCR2*) was commonly expressed across most immune cell lineages in BRAF fusion PAs, with the highest expression levels in the myeloid cell population (Figure 2C).

### Phagocytic MG and antigen presentation are present in BRAF fusion PAs.

Gene ontology (GO) enrichment analysis revealed that, of the top 50 GO pathways, 80% were related to immune activation and antigen presentation. Consistent with gene set enrichment (GSE) pathway analysis results, we confirmed that CD11c^+^ cells were interacting with either CD4^+^ or CD8^+^ T cells with LCK expression between the 2 cell populations (Supplemental Figure 3, A and B), which would be reflective of antigen presentation within the TME. Tumor-associated myeloid cells, which include cells originating from the periphery, and brain-resident MG are a dominant immune population in the glioma TME (Figure 2D). The MG are identified using 3 canonical markers (*TMEM119*, *CX3CR1*, and *P2RY12*). Using markers identified by other groups (34–38), the MG cells were further categorized into distinct subtypes: inflammatory groups 1 and 2 (*CCL4L2*, *TMEM107*, and *TNF* expression); phagocytic (*C1QA*, *TMEM176B*, and *VSIG4* expression); HSP-expressing (*HSPA1A*, *HSPA1B*, and *HSPB1*); perivascular (*LYVE1*, *FOLR2*, and *MRC1* expression); and homeostatic (*P2RY12*, *CSF1R*). Single-cell analysis also indicated the presence of innate immune cells including 4 tumor-associated macrophage (TAM) populations: lysosomal (*CSTB*, *LYZ*, and *LIPA*); inflammatory (*IFI6*, *IFIT1*, *ISG15*, *CCL4*); APC-like (*CTSD*, *MS44A4A*, *MS4A6A*); RNA splicing *(MALAT1*, *SLC1A3*, *SLC38A2*); type 2 conventional DCs (*CLEC10A*, *FCER1A*); plasmacytoid DCs (*CLEC4C*, *IL3RA*); monocytes (*VACN*, *FCN1*, *S100A8*); and neutrophils (*LY6G6D*, *JMJD1C*, *CD117*) (Figure 2D and Supplemental Table 4). To begin to elucidate the immune functional roles in the innate immune cells within the BRAF fusion PA TME, the expression of key genes involved in tumor cytotoxicity, immune suppression, antigen presentation functions, and phagocytosis were assessed. The dominant immune-suppressive mechanism genes were *TGFB1*, *STAT3*, *LAIR1*, and *HAVCR2* (Figure 2F).

### Unique CD4^+^P2RY12^+^TIM3^+^ cluster identified in BRAF fusion PA.

**Figure 3:**
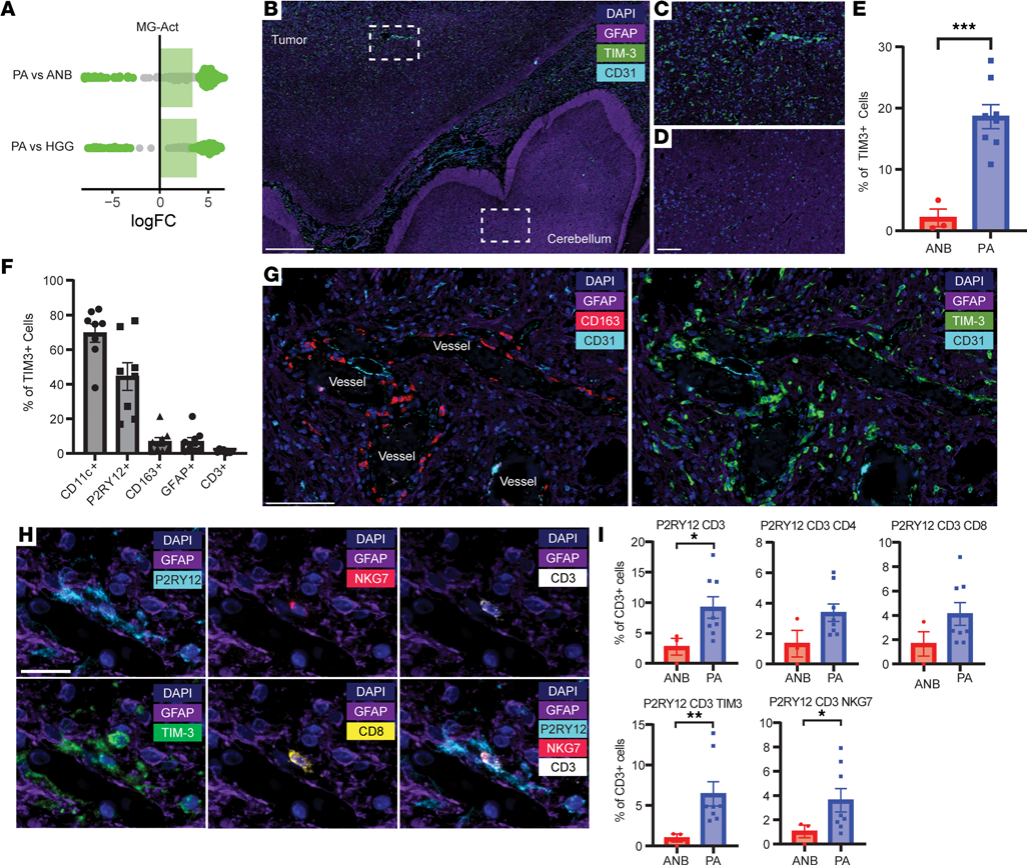
Spatial analysis of TME in BRAF fusion PA. (**A**) Strip plot showing the differential abundance of the MG-Act cell population between PA versus ANB and PA versus HGG by log (fold change [FC]). Cell types with a value of 0.1 or less are colored otherwise in gray. (**B**) Multiplex immunofluorescence imaging of the spatial distribution of TIM3 expression in BRAF fusion PA relative to ANB. Scale bar: 500 μm. (**C**) Higher-magnification image from the tumor and (**D**) image from the ANB. Scale bar: 100 μm. (**E**) Histogram of the percentage of TIM3^+^ cells based on anatomical location (*n* = 8 PAs; *n* = 3 ANBs). Each symbol represents a patient specimen. Data indicate the mean ± SEM. ***P < 0.001, by 2-tailed Student’s *t* test. (**F**) Histogram of the percentage of TIM3^+^ cells that coexpressed CD11c^+^, P2RY12^+^, CD163^+^, GFAP^+^, or CD3^+^ in PAs (*n* = 8). Data indicate the mean ± SEM. (**G**) Representative image of CD163^+^ macrophages lining the vessel walls of a BRAF fusion PA (left panel) and expressing TIM3 (right panel). Scale bar: 100 μm. (**H**) Representative image of the P2RY12^+^CD3^+^NKG7^+^ MG-Act cell population within the TME of PAs (*n* = 8). Scale bar: 20 μm. (**I**) Histogram of the percentage of MG-Act cells between ANBs (*n* = 3) and PAs (*n* = 8). Data indicate the mean ± SEM. *P < 0.05 and **P < 0.01, by 2-tailed Student’s *t* test.

When the T/NK cell population was analyzed, we identified the following clusters: *CD4^+^*, 3 *CD8^+^*, double-negative memory-like (CD4^–^CD8^–^ Tm), Tregs, 3 NK, γδ-T cells, and a unique *CD3E^+^CD4A^+^P2RY12^+^* cluster defined as MG-Act (Figure 2E and Supplemental Table 5). The CD4^+^ T cells expressed the central memory (Tcm) markers *IL7R*, *CCR7*, *TCF7*, and *CD40LG*. CD8^+^ cells were subclassified as *IL7R^+^*, *CD69^+^*, and *TCF7^+^*, and 2 early activated *GZMA/K/H^hi^GZMB^lo^* clusters were identified (CD8.Tearly.act.1 and CD.Tearly.act.2). NK cells were subclassified as *XCL1/2^+^*, *GZMB^hi^PRF1^+^TIM3^+^CXC3CR1^+^*, and memory-like given their expression of *IL7R*, *CD69*, and *TCF7*. Most T cell effector populations did not express markers of immune exhaustion such as *PD1*, *TIGIT*, and *LAG3* in the scRNA-Seq data (Figure 2G). T cells in the TME of PAs could not be identified as expressing these markers using seqIF staining. The MG-Act cell population was notable for having genes related to immune cytotoxic functions (Figure 2, F and G). The top 40 upregulated genes for the MG-Act cluster included *C1QC*, *APOE*, *C1QB*, *C1QA*, *CST3*, *APOC1*, *HLA-DRA*, *C3*, *AIF1*, *CD74*, *MARCKS*, *FTL*, *TREM2*, *SPP1*, *APOC2*, *CD68*, *TYROBP*, *HLA-DRB5*, *HLA-DPA1*, *SPI1*, *NPC2*, *CTSB*, *TMEM176B*, *SERPINA1*, *HLA-DRB1*, *FCER1G*, *IFI30*, *GSN*, *MS4A64*, *CSF1R*, *GPR34*, *LY86*, *CD14*, *VSIG4*, *HLA-DPB1*, *TUBA1B*, and *SCIN*, indicating antigen presentation capability. These MG-Act cells were enriched in the BRAF fusion PAs compared with ANB and high-grade glioma (HGG) (scRNA data obtained from Gene Expression Omnibus [GEO] GSE249263) (Figure 3A).

### TIM3-expressing immune cells are spatially localized to distinct niches of the TME.

To explore the spatial distribution of TIM3-expressing cells within the BRAF fusion PA TME, we performed seqIF. We found that TIM3 expression was dispersed throughout the TME with minimal expression found in ANB (Figure 3, B–E). CD11c^+^ and P2RY12^+^ cells were the predominant TIM3^+^ expressing population with minimal TIM3 expression on GFAP^+^ tumor cells or CD3^+^ lymphoid cells (Figure 3F). Notably, we detected TIM3 expression on myeloid cells lining the vessels in the glioma (Figure 3G), but not in ANB (Supplemental Figure 4). The scRNA-Seq data implicating cytotoxic functions of MG-Act cells were validated at the protein expression level, including for P2RY12, CD3, CD4, CD8, NKG7, and TIM3 (Figure 3, H and I). TIM3 expression on the P2RY12^+^ MG-Act cell population was dispersed throughout the TME PA but not in the ANB.

### TIM3 expression in PAd can be therapeutically manipulated.

**Figure 4:**
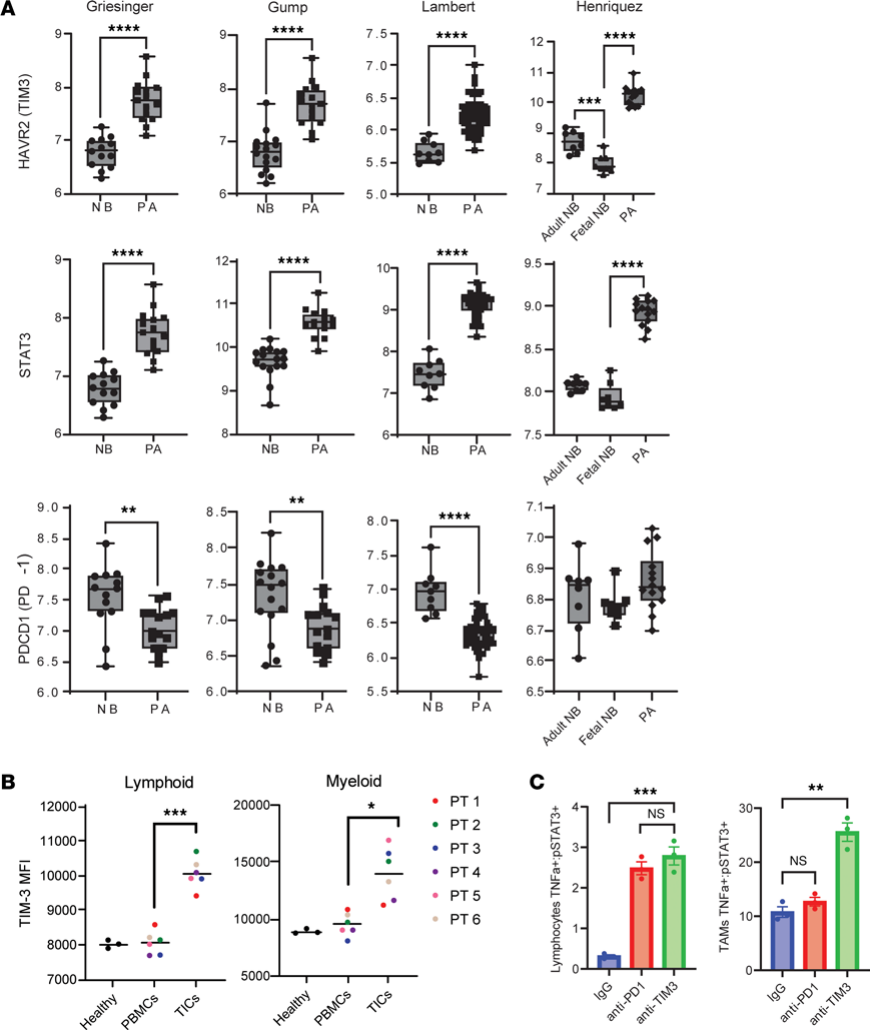
TIM3 is upregulated in TICs compared with matched PBMCs. (**A**) Comparison of *HAVCR2* (TIM3), *STAT3*, and *PDCD1* (PD-1) mRNA expression in PA and ANB samples. Data were extracted from GlioVis. mRNA expression (log_2_) of selected gene markers is shown. Full data are available in Supplemental Figure 4. **P < 0.01 and ****P < 0.0001, by Student’s *t* test or 1-way ANOVA with Tukey’s multiple-comparison test for Henriquez. (**B**) TIM3 MFI values for matched TICs and PBMCs from patients with BRAF fusion PA (*n* = 6) relative to healthy controls (*n* = 3). *P < 0.05 and ***P < 0.0001, by 2-tailed, paired Student’s *t* test. (**C**) Ratio of TNF-α^+^ to p-STAT3^+^ lymphocytes and myeloid cells after 48 hours of treatment ex vivo with either IgG, anti-TIM3, or anti–PD-1 in ex vivo PA samples (*n* = 3). **P < 0.01 and ***P < 0.001, by 1- way ANOVA with Tukey’s multiple-comparison test. Data indicate the mean ± SEM.

Four publicly available datasets were queried to evaluate TIM3 and STAT3 expression in PA tumors compared with normal brain (NB) samples (7, 39–41), showing that expression of both TIM3 and STAT3 is significantly increased in PA tumors. Additionally, the Henriquez et. al. dataset contains both fetal and adult NB, and analysis demonstrated that fetal NB had lower TIM3 and STAT3 expression than did adult NB. PDCD1 (PD-1) was expressed at lower levels in PAs compared with ANBs (Figure 4A and Supplemental Figure 5). Ex vivo flow cytometry showed increased TIM3 expression on tumor-infiltrating immune cells (TICs) in both myeloid and lymphoid compartments compared with matched PBMCs and healthy control PBMCs (Figure 4B). To evaluate whether anti–PD-1 or anti-TIM3 could reprogram the immune cells isolated from patients’ PAs, 3 ex vivo tumors were placed into single-cell suspensions and treated with antibodies. Forty-eight-hour treatment led to an increased ratio of TNF-α/phosphorylated STAT3 (p-STAT3) in lymphocytes with both anti-TIM3 and anti–PD-1 treatment. However, only anti-TIM3 increased the TNF-α/p-STAT3 ratio in the myeloid cells (Figure 4C), indicating that anti-TIM3 can drive proinflammatory responses in both the adaptive and innate arms of the immune system.

### Anti-TIM3 has a therapeutic effect in preclinical models of glioma.

**Figure 5:**
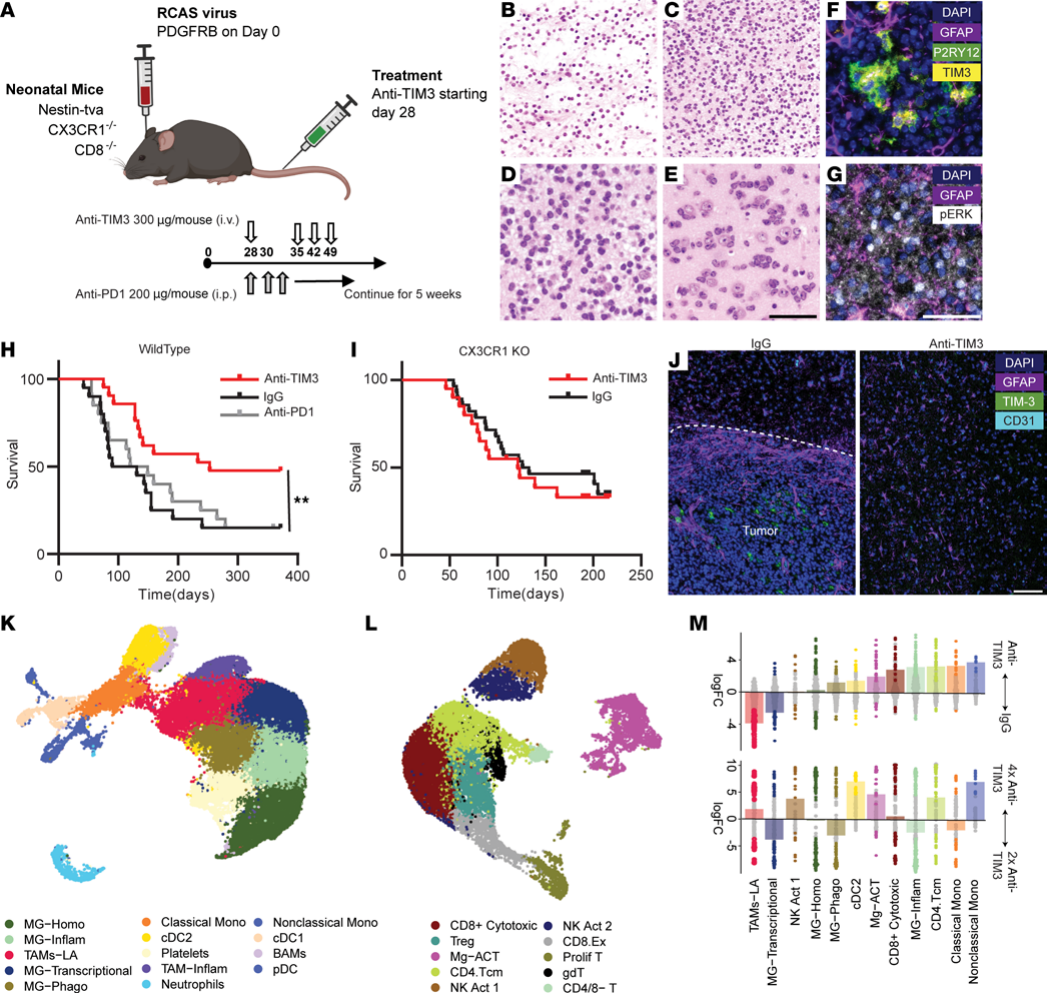
Anti-TIM3 effectively reprograms the immune environment. (**A**) Immunocompetent GEMM were treated with anti-TIM3 (300 μg/mouse) or IgG (100 μg/mouse) once per week or anti–PD-1 (200 μg/mouse) 3 times per week starting at day 28. (**B**) Histological evaluation of the GEMM displaying features of a low-grade glioma such as a loose microcystic pattern. Scale bar: 100 μm. (**C**) H&E-stained images demonstrating heterogeneity in a region of greater cellular density. Scale bar: 100 μm. (**D**) H&E-stained image demonstrating that glioma cells were monotonous and lacked mitosis (higher magnification of the image in **C**). (**E**) H&E-stained image demonstrating perineuronal satellitosis at infiltrating edges of the tumors. Scale bar: 100 μm (**B** and **C**) and 50 μm (**D** and **E**). (**F**) Immunofluorescence imaging of TIM3 expression on MG in the GEMM (*n* = 4). GFAP: purple; P2RY12: green; TIM3: yellow. (**G**) Immunofluorescence imaging p-ERK1/2 expression in the GEMM (*n* = 4). GFAP: purple; p-ERK: white. Scale bar: 50 μm (**F** and **G**). (**H**) Survival of low-grade glioma GEMM mice using Kaplan-Meier analysis. IgG: *n* = 20 mice (median survival [MS]: 110.5 days), anti–PD-1: *n* = 20 mice (MS: 134.5 days), anti-TIM3: *n* = 21 mice (MS: 253 days). Statistics (log-rank test): control versus anti–PD-1, *P* = 0.64; control versus anti-TIM3, *P* < 0.01; anti-TIM3 versus anti–PD-1, *P* = 0.02. (**I**) Survival of low-grade glioma CX3CR1-KO GEMM mice using Kaplan-Meier analysis. IgG: *n* = 20 mice (MS: 121 days), anti-TIM3: *n* = 28 mice (MS: 129.5 days). *P* = 0.51, by log-rank test for control versus anti-TIM3. (**J**) Representative immunofluorescence imaging of brains from the murine LGG model. Tumors were demarcated using H&E by a neuropathologist. Scale bar: 100 μm. (**K**) scRNA-Seq UMAP plot of the myeloid cells. *n* = 3 per group. (**L**) scRNA-Seq UMAP plot of the lymphoid cells. (**M**) Strip plot showing the differential abundance of cell types in the WT GEMM with treatment, log_2_ FC. Cell types with a *P* value of 0.1 or less are shown in gray. Cell types were ranked by the mean log_2_ FC of anti-TIM3 versus IgG. MG-Homo, MG-homeostatic; MG-Inflam, inflammatory MG; MG-Phago, phagocytic MG; Mono, monocytes.

There is no immune-competent, low-grade glioma model that has a BRAF fusion for preclinical testing (42–47). C57BL/6J mice bearing intracranial CT-2A high-grade gliomas were treated with anti-TIM3 or a control once per week or with anti–PD-1 three times per week for 3 weeks starting on day 7 after tumor implantation. Additionally, we used a low-grade genetically engineered murine model (GEMM) triggered by PDGF, which activates the MAPK pathway (48–53). Ntva^+^/BL6 mice injected with *RCAS-PDGFB* were observed for 28 days and then randomized into treatment with anti-TIM3, anti–PD-1, or IgG isotype control delivered i.v. once per week for up to 4 weeks (Figure 5A). In the CT-2A model, which shows TIM3 expression in the myeloid compartment by scRNA, the IgG control mice had a median survival of 39 days, anti–PD-1–treated mice had a median survival of 47 days, and TIM3-treated mice had a median survival of 47 days (log-rank test, *P* = 0.14; Supplemental Figure 6, A–C). To ascertain if these monotherapeutic immunotherapies would have an effect on low-grade gliomas, the *RCAS-PDGFB* GEMM glioma model was histologically characterized by a board-certified neuropathologist. The *RCAS-PDGFB* GEMM model displayed the classic features of a low-grade glioma including a loose microcystic pattern (Figure 5B), heterogeneity in cellular density (Figure 5C), the absence of mitosis (Figure 5D), and perineuronal satellitosis at the infiltrating edges of the tumors (Figure 5E). This model also shows TIM3 expression on P2RY12^+^ MG and activation of the MAPK pathway as assessed on the basis of p-ERK1/2 expression (Figure 5, F and G). In this low-grade GEMM glioma model, the median survival in the IgG control group (*n* = 20) was 110.5 days, the anti–PD-1–treated group (*n* = 20) median survival was 134.5 days (log-rank test, *P* = 0.64 vs. IgG), and the anti-TIM3–treated group (*n* = 21) was 253 days (log-rank test, *P* = 0.01 vs. IgG; Figure 5H).

### Anti-TIM3 mediates its therapeutic effect through the immune system.

Because the GEMM models have a survival time of over 90 days, in vivo depletions are not a valid strategy. As such, we used CX3CR1-KO mice to identify the immune effector cell population (54). Anti-TIM3 antibody was administered using the same schedule as for WT mice (Figure 5A). In contrast to the WT mice, the therapeutic effect of anti-TIM3 was lost in the CX3CR1-KO, since this background eliminates cytotoxic effector functions (e.g., MG-Act) and is essential for the activation of adaptive immunity (e.g., CD8^+^ cytotoxic T cells) (log-rank test, *P* = 0.33; Figure 5I). The CD8-KO background similarly eliminated the therapeutic effect of anti-TIM3 treatment (Supplemental Figure 6D).

### Anti-TIM3 enhances the MG-Act population in vivo.

During the therapeutic window, WT mice were terminated for immune assessments of the TME using scRNA-Seq after either 2 or 4 treatments with anti-TIM3 or IgG. Upon termination, the IgG-treated mice had large gliomas, while the anti-TIM3–treated group either had small gliomas or the tumors were absent (Figure 5J). The MG-Act cell population was identified in the WT GEMM MAPK-driven, low-grade glioma preclinical model (Figure 5, K and L, and Supplemental Tables 6 and 7), which became more abundant with anti-TIM3 treatment alongside other effector cell populations including CD8^+^ cytotoxic T cells (Figure 5M). Additional dosing of anti-TIM3 further increased the MG-Act cell population and T/NK cell populations (NK Act 1, CD8^+^ cytotoxic, CD4^+^ Tcm) (Figure 5M). Anti-TIM3 induced global changes in the immune response within the glioma, as reflected by increases in the phagocytic MG, cDC2, CD8 cytotoxic, inflammatory MG, CD4 Tcm, and monocyte populations.

## Discussion

To our knowledge, this is the largest cohort of PAs to date that have been immunologically characterized (33). We have previously characterized adult high-grade gliomas for potential immune therapeutic targets (28, 32, 55–58) and shown that one of the most common immune-suppressive targetable pathways was CD73/adenosine (59, 60). These prior analyses were conducted to provide prioritization of available immune therapeutic strategies and to clarify whether companion biomarkers would be needed for stratification and/or enrollment for immunotherapy clinical trials. In the current analysis, we directed our attention to identifying frequent immune-modulatory targets that may be relevant to treating pediatric patients with glioma. Using orthogonal strategies of high-dimensional immunofluorescence multiplexing and sc-Seq within the TME, we discovered that targeting TIM3 could potentially benefit patients with BRAF fusion PA and, more broadly, non-high-grade MAPK-activated gliomas. TIM3 expression was primarily observed in myeloid cells, which is consistent with findings from orthotopically implanted high-grade glioma mouse models (61–64). In such models, the combination of radiation, anti-TIM3, and anti–PD-1 was curative (61). Preclinical testing has also shown that anti-TIM3 therapy has synergistic antitumor activity when combined with adoptive immunotherapeutic agents such as CAR T cells (63).

A unique finding from this study is the discovery of a highly activated MG population that is closely associated with T/NK cells. Based on the sc-Seq data, this cell population aligns with activated MG that express TIM3. The therapeutic activity of anti-TIM3 was lost in both the CX3CR1- and CD8-KO backgrounds. The CX3CR1 KO would prevent antigen presentation and T cell activation and elimination of the CX3CR1^+^ MG-Act cell population. The CD8 KO would hinder the cytotoxic effector response but could also eliminate the MG-Act cell population. There has been no significant toxicity noted in preclinical studies involving TIM3 targeting (65, 66), and we found minimal expression of TIM3 within the ANB or on circulating PBMCs. In contrast to other immune therapeutic targets, we show that TIM3 expression is on the blood vasculature, which would provide a target for large molecules such as antibodies that are typically excluded by the blood-brain barrier (BBB).

Although there have been attempts at developing BRAF V600E–mutant glioma models, we are not aware of the existence of an immune-competent BRAF fusion PA model in which we could therapeutically test the effects of anti-TIM3 preclinically (67). There is a BRAF V637E Cre model, but mice do not succumb to tumors until after 300 days, which hampers the feasibility of testing a therapeutic agent in such a model. The group that developed this model used it in the context of seizure activity and not to evaluate glioma therapeutics (68). As such, we tested the anti-TIM3 agent in 2 preclinical models including a low-grade glioma GEMM model driven by PDGF and subsequent MAPK activation (48–53, 69) and a standard clonotype high-grade glioma, CT-2A. Another recent study evaluating monotherapeutic anti-TIM3 therapy in the CT-2A model found no therapeutic benefit (70), consistent with our results. However, anti-TIM3, but not anti–PD-1, exerts marked therapeutic effects in this preclinical low-grade glioma model. The absence of a therapeutic effect of either of these agents in high-grade gliomas may be a function of marked immune suppression and T cell exhaustion (71). We have previously shown that PD-1 expression is typically very low and infrequent in low-grade gliomas (72), probably accounting for the absence of therapeutic activity of this strategy for low-grade gliomas. Our study shows that the expression of PD-1 and PD-L1 is lower relative to TIM3 in BRAF fusion PA. As such, targeting PD-1 or PD-L1 is unlikely to be of monotherapeutic benefit in this setting. Other immune targets such as LAG3 and TIGIT were only minimally expressed in PAs on the immune cells and can also be deprioritized in this indication.

Companion biomarkers such as TMB, IFN signatures, PD-1, and PD-L1 expression have been used to identify patients who may respond to ICIs. However, with the exceptions of the IFN signature and p-ERK, these markers have not been predictive of a response to ICIs for patients with adult glioblastoma (28, 32, 73). Prior multiomics profiling has revealed that there are 2 biologically distinct groups of PAs (74). Group 1 is enriched for immune response pathways such as IFN signaling and infiltrating T cells, which aligns with the molecularly defined MAPK group in this study. Because p-ERK profiling is not yet a Clinical Laboratory Improvement Amendments–approved (CLIA-approved) biomarker that could be analyzed from commercial datasets, we relied on the published literature indicating that MAPK activation is the oncogenic mechanism for BRAF-driven gliomas (75). The marked expression of p-ERK in PAs suggests that these tumors might be responsive to ICI therapy. Although p-ERK is a biomarker for the ICI response in glioblastoma (32), it is unknown if this would be true for PAs, since so few patients have been treated with ICIs. This also serves to illustrate that immunological biomarkers will continue to rapidly evolve. Since TIM3 inhibitors are just now entering clinical trials, it is unknown if the IFN signature or p-ERK expression would predict the response to these newer agents.

Our data would support a clinical trial for treating pediatric PA patients with a TIM3-targeted therapy. There are several clinical trials evaluating anti-TIM3 strategies in solid cancers including adult recurrent high-grade gliomas (NCT03961971). Furthermore, several pharmaceutical companies have TIM3-targeting agents in development. Because TIM3 is expressed on a substantial number of bone marrow–derived immune cells such as DCs, including those that line the blood vasculature, systemic administration may be sufficient. Since PA has contrast enhancement, implying some degree of BBB disruption at baseline, there may be an additional therapeutic opportunity to modulate the brain-intrinsic MG that express TIM3 with BBB-opening ultrasound strategies that are currently in clinical trials (76, 77). If a clinical trial were to be developed for this indication, patients who have previously received anti-VEGF therapy should be excluded, since this treatment modality has been shown to downregulate TIM3 expression (62). Although we focused on the indication of anti-TIM3 in BRAF fusion PA, which accounts for the majority of pediatric gliomas, this could be further explored in high-grade, BRAF-driven gliomas such as pleomorphic xanthoastrocytoma (78). Additionally, TIM3 expression has been correlated with BRAF status in other cancers, suggesting a therapeutic benefit in non-CNS cancers (79). In summary, our analysis indicates a clinical indication for TIM3 modulation in patients with BRAF fusion PA or, more broadly, in MAPK-activated low-grade gliomas.

## Methods

### Sex as a biological variable.

Both male and female human samples were included for scRNA-Seq. For all mouse studies, male and female mice were included. Sex was not considered as a biological variable in the statistical analyses.

### Data collection.

Caris Life Sciences, a CLIA-certified laboratory, maintains a robust database of retrospectively analyzed tumor specimens. Members of the Caris Precision Oncology Alliance are able to query this database for further analysis. Study participants were deidentified before analysis, and this component of the research is exempt under the Code of Federal Regulations 45 CFR 46.101(b) (4) from 45 CFR part 46 requirements. Specimens were obtained from multiple research centers within the United States and have limited clinical annotation.

### Next-generation sequencing of DNA.

Next-generation sequencing (NGS) was performed on genomic DNA isolated from formalin-fixed paraffin-embedded (FFPE) tumor samples using the NextSeq or NovaSeq 6000 platforms (Illumina). For NextSeq-sequenced tumors, a custom-designed SureSelect XT assay was used to enrich 592 whole-gene targets (Agilent Technologies). For NovaSeq-sequenced tumors, more than 700 clinically relevant genes at high coverage and high read depth were used, along with another panel designed to enrich for an additional 20,000 genes or more at lower depth. All variants were detected with greater than 99% confidence based on allele frequency and amplicon coverage, with an average sequencing depth of coverage of more than 500 and an analytic sensitivity of 5%. Before molecular testing, tumor enrichment was achieved by harvesting targeted tissue using manual microdissection techniques. Genetic variants identified were interpreted by board-certified molecular geneticists and categorized as “pathogenic,” “likely pathogenic,” “variant of unknown significance,” “likely benign,” or “benign,” according to the American College of Medical Genetics and Genomics standards. When assessing mutation frequencies of individual genes, “pathogenic” and “likely pathogenic” were counted as mutations. The copy number alteration of each exon was determined by calculating the average depth of the sample along with the sequencing depth of each exon and comparing this calculated result with a precalibrated value.

### Tumor mutational burden assessment.

TMB was measured by counting all nonsynonymous missense, nonsense, inframe insertion/deletion, and frameshift mutations found per tumor that had not been previously described as germline alterations in dbSNP151, Genome Aggregation Database (gnomAD) databases, or benign variants identified by Caris geneticists. A cutoff point of 10 or more mutations per megabase was used based on the KEYNOTE-158 pembrolizumab trial, which showed that patients with a TMB of 10 or more mutations per megabase (mt/Mb) across several tumor types had higher response rates than did patients with a TMB of less than 10 mt/MB (80). Caris Life Sciences is a participant in the Friends of Cancer Research TMB Harmonization Project

### Whole-transcriptome sequencing.

Gene fusion detection was performed on mRNA isolated from a FFPE tumor sample using the Illumina NovaSeq platform (Illumina) and the Agilent SureSelect Human All Exon V7 bait panel (Agilent Technologies). FFPE specimens underwent pathology review to diagnose the percentage of tumor content and tumor size; a minimum of 10% of tumor content in the area for microdissection was required to enable enrichment and extraction of tumor-specific RNA. The Qiagen RNA FFPE Tissue Extraction Kit was used for extraction, and RNA quality and quantity were determined using the Agilent TapeStation. Biotinylated RNA baits were hybridized to the synthesized and purified cDNA targets, and the bait-target complexes were amplified in a post-capture PCR reaction. The resultant libraries were quantified and normalized, and the pooled libraries were denatured, diluted, and sequenced; the reference genome used was GRCh37/hg19, and analytical validation of this test demonstrated a 97% or higher positive percent agreement (PPA), 99% or higher negative percent agreement, and 99% or higher overall percent agreement with a validated comparator method. Raw data were demultiplexed by the Illumina Dragen BioIT accelerator, trimmed, and counted, and PCR duplicates were removed, followed by alignment to the human reference genome hg19 using the STAR aligner. For transcription counting, transcripts per million (TPM) values were generated using the Salmon expression pipeline. Transcriptomic signatures predictive of the response to immunotherapy (T cell–inflamed score) and the RSRD score were calculated on TPM values. Immune cell fractions were estimated using RNA deconvolution (quanTIseq). The total cell fraction consists of 10 immune cell populations and an 11th group designated as “uncharacterized cells” that includes both tumor and other stromal cells that are not 1 of the 10 cell populations adapted from ref. 81.

### Study participants at Northwestern University.

A sample of peripheral blood based on the patient’s weight was obtained at the time of initial anesthesia induction and transferred to the research laboratory for processing. No modifications were made regarding the standardized surgical resection and standard oncological principles of en bloc removal with a margin of surrounding adjacent brain if neurologically feasible (82). For the patients from whom ANB samples were obtained, adjacent brain to a benign surgical lesion was resected separately as part of the approach to create a corridor to a more deep-seated abnormality. Approximately 1 cm^3^ of the tumor was designated for scRNA-Seq analysis and processed into a single-cell suspension after enrichment for the immune cells by Percoll gradient. A second adjacent piece of tumor in continuity with the surrounding brain was processed for FFPE. This specimen was used for sequential multiplex immunohistochemical analysis.

### Tissue processing and preparation.

All tumors were graded pathologically by the study neuropathologists according to the WHO classification (83). At least 500 mg viable, non-necrotic tumor was required to obtain sufficient quantities for analysis and was processed within 1 hour of resection. ANB was obtained from study participants as part of the planned surgical approach to gain access to a low-grade, noninfiltrating glioma, epileptogenic focal cortical dysplasia (FCD), or during a planned super-total resection of adjacent regions. The ANB tissue was sent for analysis separately from the tumor. The freshly resected tissue was processed in parallel both for a single-cell suspension and for FFPE analysis. The FFPE sample was used for automated seqIF and NanoString nCounter analysis of a 770 gene panel after microdissection of the tissue (tumor area vs. ANB) and RNA isolation (Qiagen kit). For the single-cell suspension, the tissue was minced into small pieces using a scalpel, dissociated, and suspended using a Pasteur pipette in 10 mL IMDM 1X (Corning) containing 2% inactivated FBS (MilliporeSigma) and collagenase and DNase enzymes at final concentrations of 100 μg/mL and 20 units/mL, respectively. The prepared mixture was incubated for 35–40 minutes at 37°C with agitation. The tissue was filtrated through a 70 μm nylon cell strainer (BD Biosciences) and then underwent centrifugation at 4°C. The pellet was either resuspended in culture media for functional assays or in a 20 mL mixture of 5.4 mL Percoll Plus (GE Healthcare) overlaid with 12 mL 1X PBS and 0.6 mL 10X PBS (Corning) for scRNA-Seq. The tube was centrifuged at 800*g* for 10 minutes at 4°C, with 9 acceleration and 0 deceleration. After centrifugation, the immune-enriched cell pellet was collected, washed, stained with Trypan blue dye (MilliporeSigma), and counted using a Countess II FL automated cell counter in a Countess cell-counting chamber (Invitrogen, Thermo Fisher Scientific).

### scRNA-Seq.

scRNA-Seq was conducted using the chromium Next GEM Single Cell protocol (10X genomics). Post-library preparation cells were sequenced using the Illumina NovaSeq. Raw data were preprocessed and aligned using Cell Ranger to obtain the matrix and count files. The Seurat R Package using the scRNA-Seq Seurat 10X genomic workflow was then used for all subsequent analyses unless noted otherwise (84). After filtering using a mitochondrial DNA threshold of 20% and a unique molecular identifier (UMI) range of 200–15,000, a total of 186,317 cells were included for further analysis. Cells were then subjected to “log normalize,” “scale data,” and “PCA” functions. “Find clusters” and “find markers” functions were used for clustering and marker identification, and nonlinear dimensional reduction techniques were applied to visual data in uniform manifold approximation and projection (UMAP) plot format. The Harmony algorithm was used to regress batch effects (85). Cell clusters were annotated using 3 methods to produce robust cell assignments: (a) comparison against known cell markers; (b) examination of differentially expressed genes (DEGs) against the Human Protein Atlas; and (c) ScType R package, an automated cell assignment algorithm. For murine sc-Seq, human immune cell assignments and transcriptional profiles were utilized for reference mapping. The differential abundance of major cell types was assessed in partially overlapping local neighborhoods on a k nearest-neighbor graph using the novel statistical framework MiloR with the following parameters: k = 30, p = 0.2, and d = 30 (86).

### GO enrichment analysis.

The DEGs were used for GO enrichment analysis using the Bioconductor Package Cluster Profiler (87). Significantly enriched GO-BP (GO biological processes) terms were retrieved by setting the threshold of a FDR of 3; queried genes were manually selected using immunological key words. Results were displayed using bubble plots. Each bubble represents a GO term, the bubble size corresponds to the gene ratio, and the color indicates the *P* value.

### seqIF staining and imaging.

The FFPE slides were collected from the Northwestern University Nervous System Tumor Bank. Tissue slices (4 μm thickness) were prepared, mounted onto positively charged glass slides (Super Frost Plus microscope slides, Thermo Fisher Scientific), and stored at room temperature for subsequent staining analysis. For each case, 1 H&E slide was reviewed, and the tissue was segmented by a certified neuropathologist. FFPE slides were preprocessed for antigen retrieval using the PT Module (Epredia) with Dewax and HIER Buffer H (TA999-DHBH, Epredia) for 60 minutes at 102°C. Subsequently, slides were rinsed and stored in multistaining buffer (BU06, Lunaphore Technologies) until use. The protocol template was generated using COMET Control Software, and reagents were loaded onto the device to perform the seqIF protocol. A list of primary antibodies with the corresponding incubation durations is shown in Supplemental Table 1. The markers used for this analysis were as follows: CD31 (endothelial cells), GFAP (glioma tumor cells), CD3 (pan–T cells), CD4 (helper T cells), CD8 (cytotoxic T cells), P2RY12, CX3CR1, and TMEM119 (MG), CD68 (pan-monocyte/macrophage marker), CD11c (antigen-presenting cells), CD163 (macrophage scavenger receptor), CD206 (immune-suppressive macrophages), NKG2D and CD56 (NK cells), p-STAT3 (nuclear hub of immune suppression), TIM3, PD-1, and PD-L1 (immune checkpoints), HLA-DR (MHC class II), Lck (immune synapse), p-ERK1/2 (MAPK/ERK pathway predictive of the response to immune checkpoint blockade),NKG7 and granzyme A (cytotoxicity capability), and CSF1R (scavenger receptor of MG homeostasis). Two secondary antibodies were used, Alexa Fluor Plus 647 goat anti-rabbit (Thermo Fisher Scientific, 1:400 dilution) and Alexa Fluor Plus 555 goat anti-mouse (Thermo Fisher Scientific,1:200 dilution), associated with DAPI counterstain (Thermo Fisher Scientific) by dynamic incubation for 2 minutes. All antibodies were diluted in multistaining buffer (BU06, Lunaphore Technologies). For each cycle the following exposure durations were used: DAPI for 80 ms, TRITC for 2 minutes, Cy5 for 2 minutes, and primary antibody for 4 minutes. The elution step lasted 2 minutes for each cycle and was performed with elution buffer (BU07-L, Lunaphore Technologies) at 37°C. The quenching step lasted for 30 seconds and was performed with quenching buffer (BU08-L, Lunaphore Technologies). The imaging step was performed with imaging buffer (BU09, Lunaphore Technologies). The seqIF protocol in COMET resulted in a multistack OME.tiff file, in which the imaging outputs from each cycle were stitched and aligned. COMET OME.tiff contains a DAPI image, intrinsic tissue autofluorescence in TRITC and Cy5 channels, and a single fluorescent layer per marker. Visualization and analysis of the images was done using Lunaphore Viewer software, in which virtual colors were assigned for each of the markers for better interpretation. Tissue segmentation, nuclei detection, and cell quantification were then conducted using the guided workflow and Phenoplex feature after the OME.tiff files were imported into the Visiopharm software.

### Ex vivo spectral flow cytometry of matched TICs and PBMCs.

One million donor-matched PBMCs and TICs were isolated and frozen down in cell recovery media (Gibco, Thermo Fisher Scientific, 12648010). Additionally, healthy PBMCs from patients with FCD were included as a control. For Golgi inhibition and intracellular staining, cells were treated with 1× STIM cocktail (Invitrogen, Thermo Fisher Scientific, 00-4970-93) in DMEM (Gibco, Thermo Fisher Scientific, 11995-040) with 10% heat-inactivated FBS (Gibco, Thermo Fisher Scientific, 10082-147) at 37°C for 2 hours. Following stimulation, cells were washed in FACS buffer (DPBS, Corning, 21-031-CM, and 1% FBS), blocked using Fc blocker (Invitrogen, Thermo Fisher Scientific, 14-9161-73), washed an additional time, and then stained with Fixable Live/Dead (Invitrogen, Thermo Fisher Scientific, L34957) in DBPS (Corning, 21-031-CM) for 10 minutes. Cells were washed 2 additional times with FACS buffer and stained with a cocktail of surface antibodies diluted to 1:100, or, as specified by the manufacturer, the cells were stained for 30 minutes (Supplemental Table 2). Cells were then permeabilized and fixed in Foxp3 transcription factor staining buffer (eBioscience, 00-5523-08) overnight and then stained with a cocktail of intracellular antibodies diluted to 1:20, or, as specified by the manufacturer, the cells were stained for 30 minutes. Sample acquisition was performed on a Cytek Aurora (Northern Lights) and analyzed using Cytobank V7.3.0. Initial gating for singlets was performed, and live (Aqua^–^) GFAP^–^CD45^+^ cells (GFAP^–^CD45^+^) were identified (Supplemental Figure 1A). Myeloid cells (CD45^+^CD11b^+^) and lymphoid cells (CD45^+^CD11b^–^) were assessed for the indicated activation markers. Cell populations were then assessed for TIM3 expression (TIM3^+^), and MFI was analyzed and visualized using GraphPad (GraphPad Software).

### Ex vivo PA immune functional assessments.

Single-cell suspensions of newly diagnosed PA containing the preexisting immune cells in the TME were incubated for 24 hours at 38°C in a flat-bottomed tissue culture–treated plate at a concentration of 1 million cells per well in DMEM plus 10% FBS. Cells were then treated with 300 μg of either isotype control (IgG4 Fc, MedChem Express, HY-P70771), anti-TIM3 (Sabatolimab, MedChem Express, HY-P99044), or anti–PD-1 (Nivolumab, MedChem Express, HY-P9903) for another 48 hours. Flow cytometry was then performed to assess the immune functions of the immune cells (Supplemental Figure 1B) as a ratio of proinflammatory TNF-α to immune-suppressive p-STAT3 expression.

### Population-based bioinformatics.

Analyses from the Griesinger, Gump, Henriquez, and Lambert pediatric brain tumor glioma datasets (7, 39–41) were obtained from GlioVis (gliovis.bioinfo.cnio.es), with statistical analyses performed using GlioVis. Data on log_2_-transformed mRNA expression of selected markers (*HAVCR2*, *STAT3*, and *PDCD1*) were downloaded and visualized as box-and-whisker plots.

### Murine models of glioma.

Because there is no immune-competent, low-grade glioma model that has a BRAF fusion available for preclinical testing (42–47), signals of response to anti-TIM3 were evaluated in a GEMM of low-grade glioma triggered by PDGF, which activates the MAPK pathway (48–53) and CT-2A cells on a C57BL/6J background, which also activate the MAPK pathway. CT-2A cells showed MAPK activation, as demonstrated by Western blotting (Supplemental Figure 2A). The immune-competent, high-grade glioma CT-2A was implanted into C57BL/6J mice at the tumorigenic cell number of 1 × 10^5^. Mice were then randomly assigned to the control or treatment groups: (a) IgG isotype control (300 μg, i.v.); (b) anti-TIM3 (300 μg, i.v., 15 mg/kg, within the range of human dosing NCT03489343); and (c) anti–PD-1 (100 μg, i.p.). The genetic low-grade model used was RCAS/Ntv-a, which induces low-grade gliomas (69). The vector constructs were propagated in DF-1 chicken fibroblasts. Live viruses were produced by transfecting plasmid versions of RCAS vectors into DF-1 cells using FuGene6 (Roche). DF-1 cell senesce 1–2 days after injection. To transfer the gene via RCAS vectors, 2 × 10^4^ DF-1–producing cells transfected with the RCAS vectors in 1–2 μL PBS were injected into the frontal lobes of neonatal GEMM mice, which carry the Ntv-a transgene, using a 26G 10 μL Hamilton syringe. Gliomas were induced in 3 different genetic backgrounds: RCAS/Ntv-a WT, Ntv-a/CD8^−/−^, and Ntv-a/CX3CR1^−/−^. The Ntv-a/CD8^−/−^ generation is described in our prior study (88). For the RCAS- Ntv-a/CX3CR1^−/−^, RCAS-CX3CR1 vectors were created by subcloning human CX3CR1 (V249) cDNA into a gateway-compatible RCAS vector using LR recombination (Invitrogen, Thermo Fisher Scientific) and verified by sequencing. To verify that the appropriate immune cell populations were eliminated, the CX3CR1-KO and CD8-KO mice were genotyped before breeding. TIM3 expression within the tumors of these models was confirmed by sc-Seq and immunofluorescence. Mice were randomized to the following treatment groups: anti-TIM3 antibody (300 μg, i.v., once per week for 4 weeks); anti–PD-1 (200 μg, i.p., 3 times per week for 5 weeks); or the IgG control (200 μg, i.p., 3 times per week for 5 weeks) starting at approximately day 28 in the Ntv-A model. In the CX3CR1-KO and CD8-KO background mice, the IgG control was administered i.v. at 300 μg once per week for 4 weeks starting at approximately day 28, identical to the dose and schedule for anti-TIM3. The mice were observed daily for survival, and when they showed signs of neurological deficit (lethargy, hypothermia, failure to ambulate, lack of feeding, body condition score <2.0, or loss of >20% BW), they were compassionately euthanized.

### Statistics.

A 2-sided Wilcoxon rank-sum test was used to calculate *P* values for all pairwise comparisons. For multiple Student’s *t* tests, the 2-stage step-up method (with Benjamini correction) method was used and a FDR (*Q*) of less than 0.1 was used. *P* values displayed in box-and-whisker plots are reported as adjusted *P* values. All box-and-whisker plots show all individual points, with the box showing 25th percentile, median, and 75th percentile and whiskers at minimum and maximum values. GraphPad Prism, version 9.2.0 (GraphPad Software), was used to analyze data.

### Study approval.

Under protocols STU00214485, approved by the IRB of Northwestern University, and 2021-4677, approved by the Ann and Robert H. Lurie Children’s Hospital of Chicago, patients were identified with surgically resectable tumors. Pediatric patients with a presumed diagnosis of a CNS lesion based on radiographic imaging with a planned, clinically indicated surgical resection were prospectively consented by their guardian. Study participants were deidentified before analysis, and this component of the research is exempt under the Code of Federal Regulations 45 CFR 46.101(b) (4) from 45 CFR part 46 requirements. Animal experiments were conducted under Northwestern University IACUC protocol no. IS00026365.

### Data availability.

The data used to support the findings of this study are available within this article. The scRNA-Seq data that support the findings of this study have been deposited in the NCBI GEO database (https://www.ncbi.nlm.nih.gov/geo/) under accession numbers (RNA-Seq GEO GSE249263). Source data for this work are provided in the Supporting Data Values file.

## Author contributions

ST, HN, CD, ABH, and MD designed experiments. ST, HN, CD, SP, RD, MS, and LH performed the experiments. JPC, RS, AMS, SKL, CMH, NRW, LH, GR, and ABH participated in the design, preparation, and acquisition of data related to human samples or animal experiments. ST, RVL, HN, JX, GL, TPN, VB, CDJ, MGC, and ABH participated in the processing and analysis of FACS, scRNA-Seq, and Caris data. ST, HN, ABH, and MD wrote and revised the manuscript. All authors edited and reviewed the manuscript.

## Supplementary Material

Supplemental data

Unedited blot and gel images

Supporting data values

## Figures and Tables

**Table 1:**
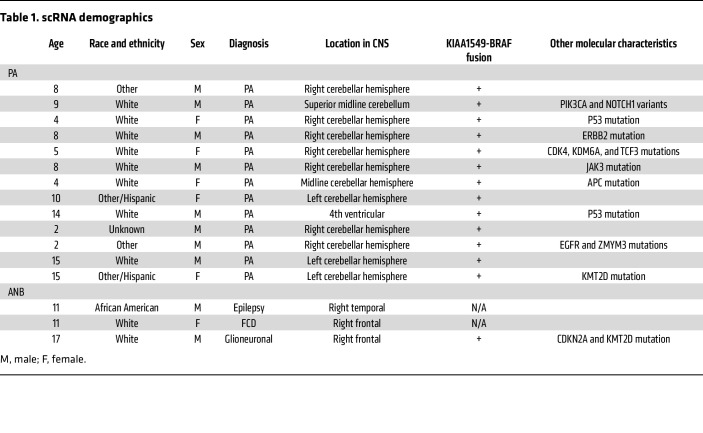
scRNA demographics

## Version 1 08/13/2024

In-Press Preview

## Version 2 10/01/2024

Electronic publication
