# Supplementary material for: Pediatric glioma immune profiling identifies TIM3 as a therapeutic target in BRAF fusion pilocytic astrocytoma
Source: J Clin Invest. 2024 Aug 13;134(19):e177413. doi: 10.1172/JCI177413 (PMC11444160; doi:10.1172/JCI177413)
Supplement: Supplemental data [file jci-134-177413-s013.pdf]

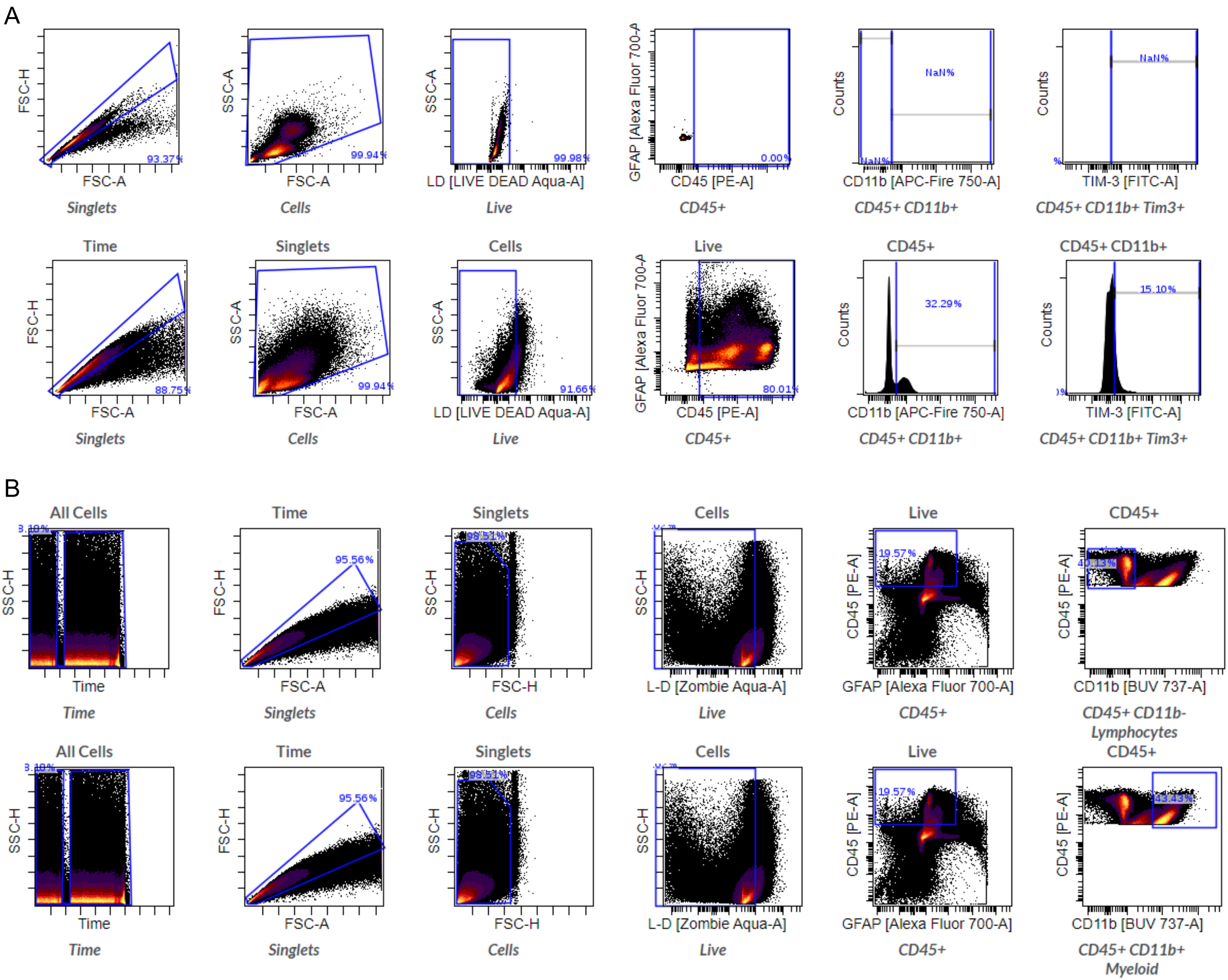

Supplementary Figure 1

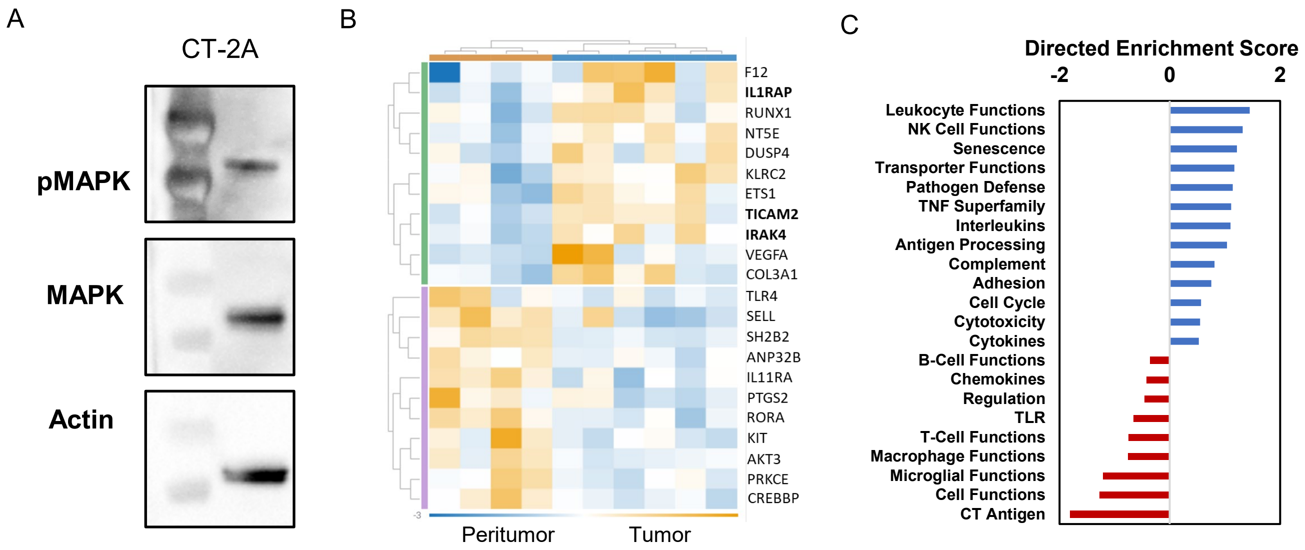

Supplementary Figure 2

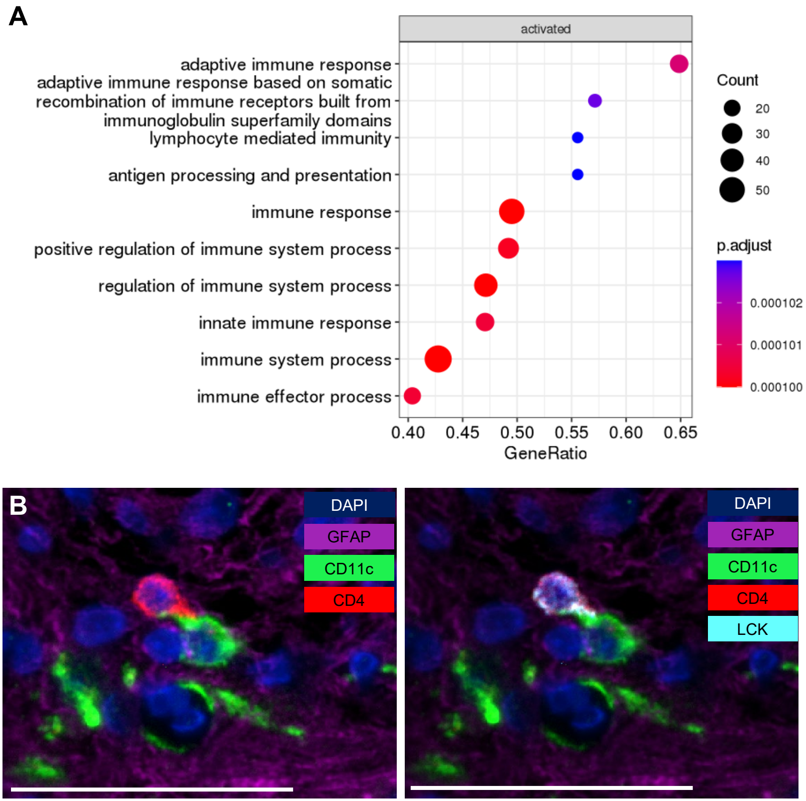

Supplementary Figure 3

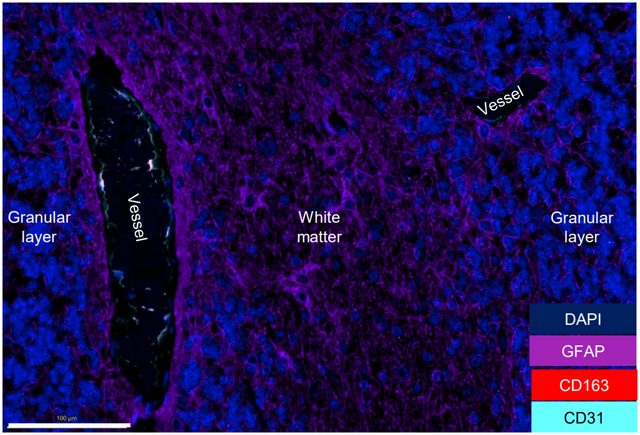

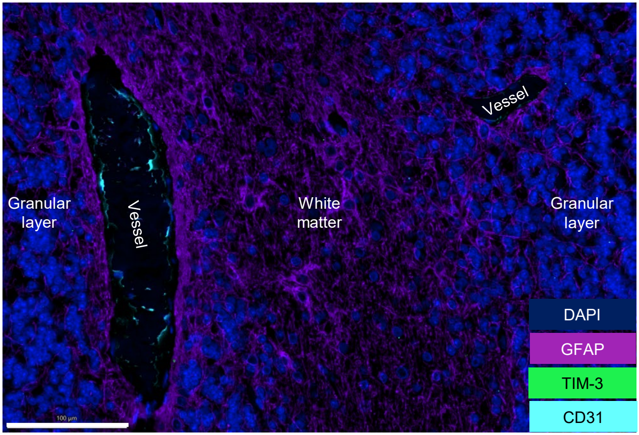

Supplementary Figure 4

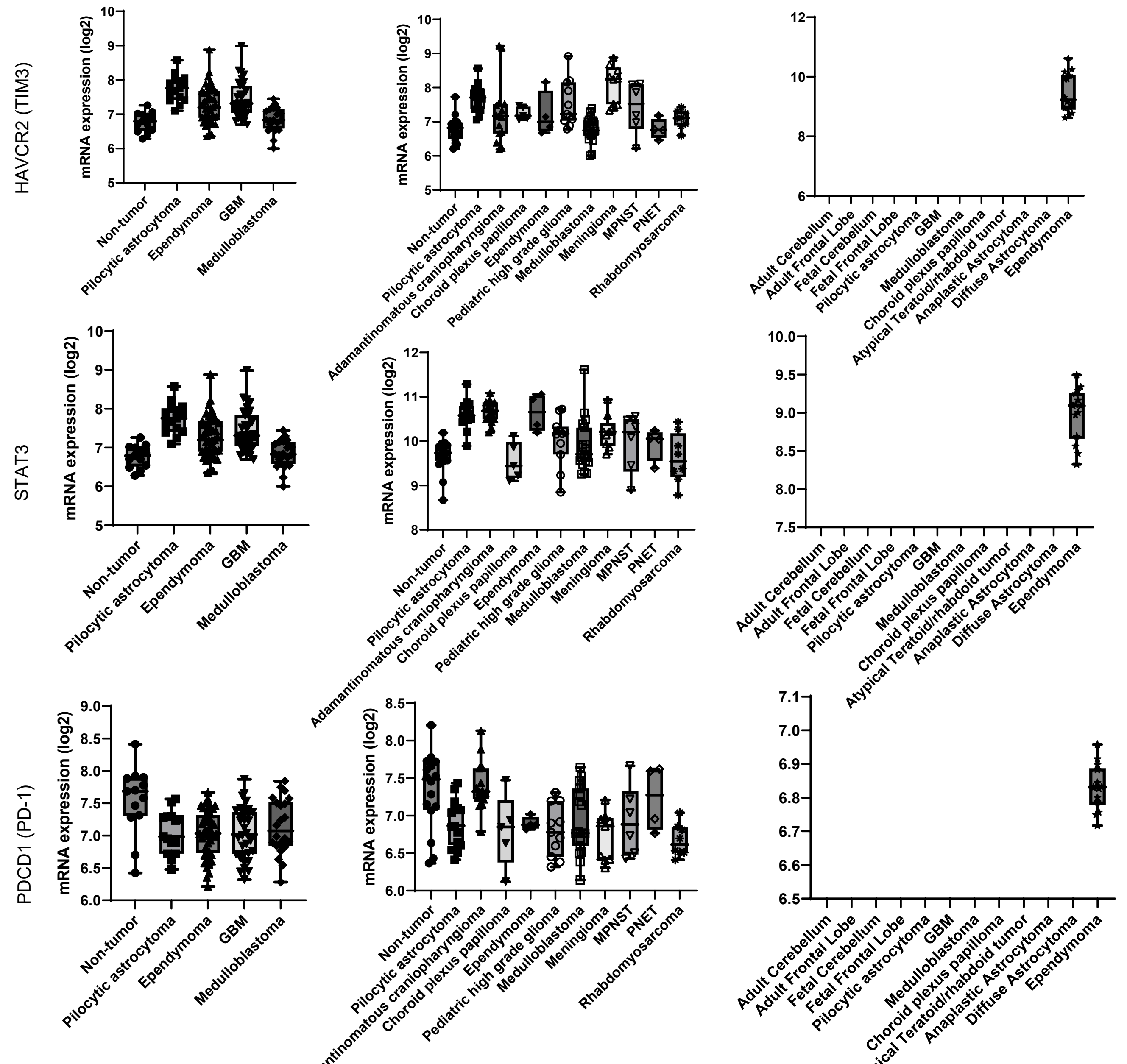

Supplementary Figure 5

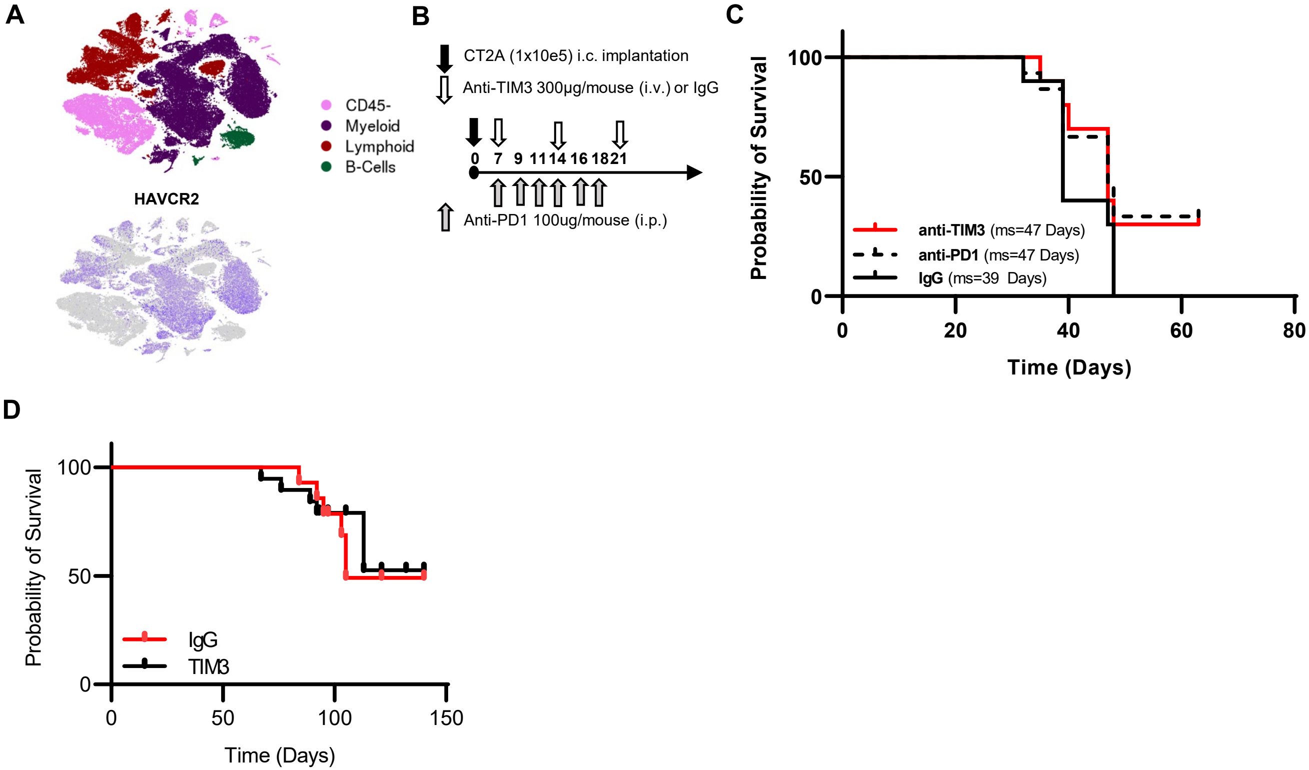

Supplementary Figure 6

Supplementary Figure 1: A) Human immunophenotyping of matched donor PBMC and TIC gating strategy: Top: Unstained pooled samples. Bottom: Representative tumor-infiltrating cells (TIC) from matched pediatric patients. FSC-H vs FSC-A to determine singlets. SSC-A vs FSC-A to determine cells. SSC-A vs LD for negative selection of live cells. The percentage of indicated subpopulation is displayed in blue. LD = Live/Dead fixable viability stain. Myeloid cells are defined as live CD45+, CD11b+. Lymphoid cells are defined as CD45+, CD11b-. B) *Ex vivo* analysis of PA samples. Top: Representative gating of lymphocytes. Bottom: Representative gating of myeloid cells. FSC-H vs FSC-A to determine singlets. SSC-A vs FSC-A to determine cells. SSC-A vs LD for negative selection of live cells. The percentage of indicated subpopulation is displayed in blue. LD = Live/Dead fixable viability stain. Myeloid cells are defined as live CD45+, CD11b+. Lymphoid cells are defined as CD45+, CD11b-
.

Supplementary Figure 2: A) Western plot of CT-2A cells showing p-MAPK expression. B) Nanostring analysis of tumor and matched peritumoral PA samples. Gene Upregulated in Tumor vs. Peritumor using unsupervised hierarchical clustering method. C) Directed Enrichment Score (DES) of tumor vs. peritumor.

Supplementary Figure 3: A) Gene Ontology analysis between BRAF-Fusion and ANB. Bubble plot depicting immune-related gene ontology (GO) analysis of intratumoral myeloid-derived cells from BRAF-Fusion compared to ANB. Each bubble represents a GO term, the bubble size corresponds to the gene ratio and the color indicates the P-value. B) Multiplex immunofluorescent imaging demonstrating an immunological synapse present in BRAF-Fusion PA. CD4: red; CD11c: green; LCK: cyan blue. Scale bars at 50 μm.

Supplementary Figure 4: Representative spatial multiplex immunofluorescence images of the adjacent normal brain showing the absence of TIM-3 and CD163 expression. The vessels and anatomical layers of the normal cerebellar cortex (granular and white matter layers) are annotated. Scale bars at 100 μm.

Supplementary Figure 5: mRNA expression of HAVCR2, STAT3, and PDCD1 of all tumor types from 3 public databases (Griesinger, Gump, and Henriquez). mRNA expression levels are displayed after the Log2 transformation.

Supplementary Figure 6: In vivo therapeutic effects of anti-TIM3 in the CT-2A glioma model. A) UMAP and feature plot showing the HAVCR2 (TIM3) expression within the CT2A model. B)

Schema of the treatment of immunocompetent C57BL/6 mice that underwent intracerebral implantation of CT-2A glioma cells. Mice were treated with either anti-TIM3 (300ug/mouse) or IgG (100ug/mouse) once per week or anti-PD1 (100 ug/mouse) 3 times weekly starting on Day 7 after implantation. C) The survival rate of high-grade glioma-bearing C57BL/6 mice implanted were estimated by the Kaplan–Meier method. IgG Control: 10 mice (MS: 39 d), anti-PD-1: 10 mice (MS: 47 d), anti-TIM3: 10 mice (MS: 47d). Statistics (log-rank test): control versus anti-PD-1 $p = 0.11$, control versus anti-TIM3 $p=0.14$, anti-PD-1 vs anti-TIM3 $p = 0.95$. D) The survival rate of low-grade glioma CD8 KO GEMM mice was estimated by the Kaplan–Meier method. IgG Control: 14 mice (MS: 105d), anti-TIM3: 19 mice (MS: undefined). Statistics (log-rank test): control versus anti-TIM3 $p = 0.66$.

Supplementary Table 1. Antibodies used for Lunaphore COMET™ multiplex immunofluorescence

Marker	Manufacturer	Catalogue number	Clone	Dilution
GFAP	Abcam	Ab68428	EPR1034Y	1/2000
CD31	Abcam	Ab225883	EPR17259	1/1500
CD3	Dako Agilent	M7254	F7.2.38	1/50
CD4	Abcam	Ab133616	EPR6855	1/500
CD8	Leica	PA0183	4B11	No dilution needed
P2YR12	Atlas Antibodies	HPA014518	Polyclonal	1/1000
CD68	Dako Agilent	M0876	PG-M1	No dilution needed
CD163	Abcam	Ab182422	EPR19518	1/600
p-STAT3	Cell signaling	9145S	(Tyr705) D3A7 XP®	1/250
NKG7	Cell signaling	84835	E6S2A	1/100
pERK1/2	Cell signaling	4370	D13.14.4E	1/1700
PD1	Abcam	Ab137132	EPR4877(2)	1/400
PDL-1	GenomeMe	IHC411-1	IHC411	1/100
Granzyme A	Thermofisher	MA5-24105	356412	1/100
TIM3	Abcam	Ab241332	EPR22241	1/100
HLA-DR	Abcam	Ab237712	CAL49	1/100
LCK	Cell signaling	2984S	D88 XP	1/100
CSF1R	Abcam	Ab183316	SP211	1/700
CD206	Abcam	Ab64693	Polyclonal	1/1000
CD11c	Abcam	Ab52632	EP1347Y	1/300
CX3CR1	Abcam	Ab8020	Polyclonal	1/100
TMEM119	Abcam	Ab185333	Polyclonal	1/150
NKG2D	Novus Bio	NB100-65956	1D11	1/500
CD56	Cell signaling	99746	E7X9M XP®	1/100
GFAP	Sigma	MAB360	GA5	1/2000
CD8	Cell signaling	98941	D4W2Z	1/500
CD4	Abcam	Ab183685	EPR19514	1/500
CD11c	Cell signaling	97585	D1V9Y	1/300
CD68	Santa Cruz	Sc-20060	KP1	1/200

P2RY12	Cell signaling	69766	E9J1J	1/100
TMEM119	Abcam	Ab209064	28-3	1/100

Supplementary Table 2. Antibodies Used For Flow Cytometry

MARKER	Color/Format	Cytek Peak Channel	Supplier	Catalog
IFN-GAMMA	Brilliant Blue 700	B9	BD Biosciences	566394
GFAP	Alexa Fluor 700	R4	Novus Biologicals	NB120-10062AF700
TNF-ALPHA	Brilliant Violet 605	V10	BioLegend	502936
TIM-3	FITC	B2	BioLegend	345022
CD45	PE	B4	BioLegend	368510
CD11B	APC-Fire 750	R7	BioLegend	301420
STAT3 (PY705)	Alexa Fluor 647	R2	BioLegend	651008
FIXABLE LIVE/DEAD	Live/Dead Fix Aqua	V7	Invitrogen	L34957

Supplementary Table 3: DEGs for Figure 2B

	p_val	avg_log2FC	pct.1	pct.2	p_val_adj	cluster	gene
APOE	0	2.250672	0.921	0.349	0	Myeloid	APOE
PLXDC2	0	2.194966	0.935	0.261	0	Myeloid	PLXDC2
CCL3	0	2.174516	0.87	0.458	0	Myeloid	CCL3
RHOB	0	2.162996	0.826	0.325	0	Myeloid	RHOB
HLA-DRA	0	2.148236	0.927	0.394	0	Myeloid	HLA-DRA
SPP1	0	2.140058	0.832	0.349	0	Myeloid	SPP1
TEX14	0	2.139177	0.758	0.351	0	Myeloid	TEX14
C1QA	0	2.115116	0.881	0.284	0	Myeloid	C1QA
CEBPD	0	2.103503	0.871	0.384	0	Myeloid	CEBPD
SERPINE1	0	2.095261	0.415	0.109	0	Myeloid	SERPINE1
C1QC	0	2.092548	0.903	0.308	0	Myeloid	C1QC
SLC1A3	0	2.088672	0.765	0.155	0	Myeloid	SLC1A3
CH25H	0	2.070111	0.472	0.14	0	Myeloid	CH25H
SGK1	0	2.066046	0.862	0.318	0	Myeloid	SGK1
APOC1	0	2.034669	0.784	0.232	0	Myeloid	APOC1
DOCK4	0	2.025271	0.897	0.259	0	Myeloid	DOCK4
CST3	0	1.983832	0.902	0.274	0	Myeloid	CST3
OLR1	0	1.977512	0.791	0.182	0	Myeloid	OLR1
C1QB	0	1.973102	0.893	0.314	0	Myeloid	C1QB
CXCL8	0	1.931676	0.505	0.149	0	Myeloid	CXCL8
CCL3L1	0	1.912223	0.741	0.306	0	Myeloid	CCL3L1
CD83	0	1.907267	0.912	0.371	0	Myeloid	CD83
HLA-DRB1	0	1.901291	0.909	0.421	0	Myeloid	HLA-DRB1
SAT1	0	1.897366	0.971	0.651	0	Myeloid	SAT1
PDK4	0	1.877223	0.612	0.166	0	Myeloid	PDK4
CD74	0	1.862844	0.991	0.806	0	Myeloid	CD74
FRMD4A	0	1.856445	0.778	0.225	0	Myeloid	FRMD4A
CSF1R	0	1.852967	0.801	0.178	0	Myeloid	CSF1R
C3	0	1.828166	0.889	0.249	0	Myeloid	C3
IL1B	0	1.804598	0.649	0.217	0	Myeloid	IL1B
RAB31	0	1.796674	0.758	0.162	0	Myeloid	RAB31
RNASET2	0	1.792564	0.901	0.42	0	Myeloid	RNASET2
TREM2	0	1.790738	0.734	0.166	0	Myeloid	TREM2
HLA-DPA1	0	1.779336	0.878	0.473	0	Myeloid	HLA-DPA1
CCL4L2	0	1.772507	0.786	0.486	0	Myeloid	CCL4L2
ARHGAP24	0	1.743484	0.724	0.16	0	Myeloid	ARHGAP24
APOC2	0	1.734765	0.64	0.208	0	Myeloid	APOC2
LRMDA	0	1.726793	0.754	0.176	0	Myeloid	LRMDA
TYROBP	0	1.70698	0.911	0.38	0	Myeloid	TYROBP
OTUD1	0	1.706387	0.564	0.147	0	Myeloid	OTUD1
CTSB	0	1.694494	0.918	0.348	0	Myeloid	CTSB
MEF2C	0	1.689744	0.742	0.166	0	Myeloid	MEF2C
CD14	0	1.689657	0.642	0.145	0	Myeloid	CD14
MAFB	0	1.678222	0.597	0.143	0	Myeloid	MAFB
IFI30	0	1.665387	0.691	0.182	0	Myeloid	IFI30

PLAUR	0	1.659903	0.537	0.142	0	Myeloid	PLAUR
IRAK3	0	1.652747	0.657	0.134	0	Myeloid	IRAK3
B3GNT5	0	1.651903	0.626	0.143	0	Myeloid	B3GNT5
SLC11A1	0	1.640793	0.636	0.125	0	Myeloid	SLC11A1
CSF2RA	0	1.640027	0.677	0.125	0	Myeloid	CSF2RA
SRGAP2	0	1.618817	0.726	0.192	0	Myeloid	SRGAP2
IER3	0	1.618063	0.771	0.261	0	Myeloid	IER3
FGD4	0	1.611243	0.683	0.154	0	Myeloid	FGD4
CLEC7A	0	1.606003	0.627	0.126	0	Myeloid	CLEC7A
ACSL1	0	1.596709	0.664	0.192	0	Myeloid	ACSL1
NR4A1	0	1.588517	0.79	0.365	0	Myeloid	NR4A1
EPB41L2	0	1.586369	0.737	0.182	0	Myeloid	EPB41L2
GPR34	0	1.585805	0.64	0.139	0	Myeloid	GPR34
FCER1G	0	1.58354	0.817	0.257	0	Myeloid	FCER1G
CD68	0	1.580537	0.706	0.156	0	Myeloid	CD68
LILRB4	0	1.578333	0.665	0.123	0	Myeloid	LILRB4
GSN	0	1.573368	0.777	0.198	0	Myeloid	GSN
C5AR1	0	1.570611	0.521	0.096	0	Myeloid	C5AR1
NLRP3	0	1.550699	0.521	0.134	0	Myeloid	NLRP3
LHFPL2	0	1.548393	0.641	0.132	0	Myeloid	LHFPL2
MS4A7	0	1.547666	0.688	0.145	0	Myeloid	MS4A7
MARCKS	0	1.540078	0.732	0.173	0	Myeloid	MARCKS
HLA-DPB1	0	1.535929	0.805	0.442	0	Myeloid	HLA-DPB1
NPC2	0	1.53233	0.805	0.297	0	Myeloid	NPC2
SLC8A1	0	1.531294	0.712	0.161	0	Myeloid	SLC8A1
FCGRT	0	1.52816	0.71	0.179	0	Myeloid	FCGRT
AIF1	0	1.52721	0.785	0.224	0	Myeloid	AIF1
HTRA1	0	1.516189	0.629	0.138	0	Myeloid	HTRA1
C3AR1	0	1.50332	0.635	0.144	0	Myeloid	C3AR1
TBXAS1	0	1.494092	0.719	0.165	0	Myeloid	TBXAS1
PLD4	0	1.492758	0.619	0.122	0	Myeloid	PLD4
SLC2A5	0	1.492144	0.619	0.121	0	Myeloid	SLC2A5
MEF2A	0	1.49204	0.786	0.258	0	Myeloid	MEF2A
HLA-DQA1	0	1.491944	0.645	0.211	0	Myeloid	HLA-DQA1
ST6GALNA(	0	1.490544	0.557	0.136	0	Myeloid	ST6GALNAC3
CD86	0	1.489248	0.637	0.126	0	Myeloid	CD86
MSR1	0	1.481348	0.579	0.122	0	Myeloid	MSR1
FTL	0	1.480334	0.974	0.897	0	Myeloid	FTL
CD163	0	1.47778	0.348	0.066	0	Myeloid	CD163
EGR1	0	1.477058	0.819	0.436	0	Myeloid	EGR1
OGFRL1	0	1.475352	0.672	0.189	0	Myeloid	OGFRL1
LPCAT2	0	1.469777	0.66	0.136	0	Myeloid	LPCAT2
P2RY12	0	1.46369	0.525	0.107	0	Myeloid	P2RY12
CXCL16	0	1.459314	0.643	0.132	0	Myeloid	CXCL16
FPR1	0	1.456569	0.63	0.132	0	Myeloid	FPR1
HSPA1A	0	1.452448	0.727	0.41	0	Myeloid	HSPA1A
JDP2	0	1.420713	0.571	0.109	0	Myeloid	JDP2

LINC00910	0	1.419842	0.536	0.252	0	Myeloid	LINC00910
HMOX1	0	1.415688	0.547	0.11	0	Myeloid	HMOX1
TRIB1	0	1.415501	0.515	0.115	0	Myeloid	TRIB1
PADI2	0	1.410733	0.581	0.142	0	Myeloid	PADI2
LDLRAD4	0	1.409978	0.822	0.396	0	Myeloid	LDLRAD4
NEAT1	0	1.405777	0.904	0.556	0	Myeloid	NEAT1
HLA-DQB1	0	1.403519	0.74	0.265	0	Myeloid	HLA-DQB1
KCNQ3	0	1.403007	0.503	0.107	0	Myeloid	KCNQ3
BASP1	0	1.400801	0.563	0.138	0	Myeloid	BASP1
AC245014.	0	1.396521	0.441	0.146	0	Myeloid	AC245014.3
HLA-DMA	0	1.394419	0.711	0.237	0	Myeloid	HLA-DMA
CYFIP1	0	1.3907	0.662	0.153	0	Myeloid	CYFIP1
SPI1	0	1.383026	0.687	0.15	0	Myeloid	SPI1
HLA-DRB5	0	1.375406	0.694	0.301	0	Myeloid	HLA-DRB5
ETS2	0	1.369148	0.64	0.165	0	Myeloid	ETS2
ADAM28	0	1.366719	0.595	0.119	0	Myeloid	ADAM28
PSAP	0	1.364772	0.895	0.498	0	Myeloid	PSAP
GRN	0	1.362501	0.669	0.177	0	Myeloid	GRN
TMEM176I	0	1.360024	0.541	0.111	0	Myeloid	TMEM176B
IL18	0	1.354643	0.591	0.115	0	Myeloid	IL18
CPVL	0	1.353774	0.588	0.117	0	Myeloid	CPVL
FCGR2A	0	1.350973	0.627	0.13	0	Myeloid	FCGR2A
ELL2	0	1.350917	0.79	0.453	0	Myeloid	ELL2
AL163541.:	0	1.346346	0.382	0.065	0	Myeloid	AL163541.1
VSIG4	0	1.344518	0.533	0.103	0	Myeloid	VSIG4
BHLHE41	0	1.343474	0.539	0.106	0	Myeloid	BHLHE41
A2M	0	1.340734	0.752	0.247	0	Myeloid	A2M
PALD1	0	1.339011	0.555	0.106	0	Myeloid	PALD1
CSF3R	0	1.33713	0.559	0.087	0	Myeloid	CSF3R
FCGR1A	0	1.335739	0.509	0.097	0	Myeloid	FCGR1A
SRGAP2B	0	1.335412	0.525	0.117	0	Myeloid	SRGAP2B
AL078590.:	0	1.322104	0.44	0.082	0	Myeloid	AL078590.2
QKI	0	1.321878	0.78	0.302	0	Myeloid	QKI
GLUL	0	1.31016	0.761	0.276	0	Myeloid	GLUL
SYNDIG1	0	1.309541	0.426	0.079	0	Myeloid	SYNDIG1
MS4A6A	0	1.303857	0.541	0.123	0	Myeloid	MS4A6A
LNCAROD	0	1.30362	0.46	0.087	0	Myeloid	LNCAROD
KLF4	0	1.301458	0.51	0.11	0	Myeloid	KLF4
ASAH1	0	1.298692	0.675	0.232	0	Myeloid	ASAH1
SPTLC2	0	1.298174	0.6	0.189	0	Myeloid	SPTLC2
SLCO2B1	0	1.294741	0.546	0.105	0	Myeloid	SLCO2B1
SERPINA1	0	1.289204	0.543	0.119	0	Myeloid	SERPINA1
TLR2	0	1.288043	0.512	0.09	0	Myeloid	TLR2
GPR183	0	1.282458	0.645	0.305	0	Myeloid	GPR183
RHBDF2	0	1.276774	0.654	0.193	0	Myeloid	RHBDF2
BCL2A1	0	1.267317	0.506	0.198	0	Myeloid	BCL2A1
CSGALNAC	0	1.255243	0.516	0.132	0	Myeloid	CSGALNACT1

MAML3	0	1.253811	0.558	0.138	0	Myeloid	MAML3
EGR2	0	1.247233	0.505	0.153	0	Myeloid	EGR2
LIMS1	0	1.245789	0.689	0.275	0	Myeloid	LIMS1
IFNGR2	0	1.2386	0.546	0.118	0	Myeloid	IFNGR2
FOS	0	1.236335	0.971	0.845	0	Myeloid	FOS
CYBB	0	1.233602	0.593	0.128	0	Myeloid	CYBB
KCTD12	0	1.230838	0.58	0.121	0	Myeloid	KCTD12
AC253572.	0	1.2294	0.33	0.121	0	Myeloid	AC253572.2
ST6GAL1	0	1.228151	0.65	0.26	0	Myeloid	ST6GAL1
TMIGD3	0	1.226478	0.396	0.073	0	Myeloid	TMIGD3
IFNGR1	0	1.226334	0.697	0.3	0	Myeloid	IFNGR1
LYN	0	1.224607	0.669	0.198	0	Myeloid	LYN
RBM47	0	1.219996	0.507	0.101	0	Myeloid	RBM47
RNF130	0	1.216938	0.655	0.19	0	Myeloid	RNF130
RAB20	0	1.213765	0.459	0.092	0	Myeloid	RAB20
CD302	0	1.206972	0.573	0.134	0	Myeloid	CD302
GRASP	0	1.20681	0.55	0.181	0	Myeloid	GRASP
SOD2	0	1.205104	0.582	0.296	0	Myeloid	SOD2
CX3CR1	0	1.203322	0.573	0.169	0	Myeloid	CX3CR1
UBE2E2	0	1.189121	0.53	0.118	0	Myeloid	UBE2E2
CEBPB	0	1.186797	0.698	0.354	0	Myeloid	CEBPB
GNAQ	0	1.186553	0.624	0.206	0	Myeloid	GNAQ
C9orf72	0	1.186009	0.576	0.184	0	Myeloid	C9orf72
RIN2	0	1.185243	0.479	0.096	0	Myeloid	RIN2
FCGBP	0	1.183882	0.382	0.115	0	Myeloid	FCGBP
RAC1	0	1.182378	0.826	0.46	0	Myeloid	RAC1
LY86	0	1.181535	0.566	0.116	0	Myeloid	LY86
NFKBID	0	1.17483	0.508	0.143	0	Myeloid	NFKBID
SCIN	0	1.17168	0.556	0.135	0	Myeloid	SCIN
LTC4S	0	1.169949	0.513	0.113	0	Myeloid	LTC4S
ABCA1	0	1.169909	0.401	0.083	0	Myeloid	ABCA1
IL13RA1	0	1.168525	0.532	0.101	0	Myeloid	IL13RA1
SRGAP1	0	1.167896	0.427	0.079	0	Myeloid	SRGAP1
ARHGAP22	0	1.165989	0.449	0.084	0	Myeloid	ARHGAP22
CCL4	0	1.165127	0.852	0.737	0	Myeloid	CCL4
HIF1A	0	1.158682	0.694	0.344	0	Myeloid	HIF1A
FMNL2	0	1.155119	0.482	0.112	0	Myeloid	FMNL2
PEAK1	0	1.151805	0.5	0.153	0	Myeloid	PEAK1
RASSF4	0	1.143693	0.488	0.105	0	Myeloid	RASSF4
IRAK2	0	1.141523	0.462	0.116	0	Myeloid	IRAK2
C1orf162	0	1.141273	0.554	0.161	0	Myeloid	C1orf162
ABCC4	0	1.140545	0.478	0.12	0	Myeloid	ABCC4
CCDC200	0	1.13814	0.251	0.068	0	Myeloid	CCDC200
LYZ	0	1.134986	0.269	0.057	0	Myeloid	LYZ
ZFHX3	0	1.134341	0.525	0.118	0	Myeloid	ZFHX3
MAP4K3	0	1.134079	0.511	0.145	0	Myeloid	MAP4K3
ITM2B	0	1.131656	0.9	0.757	0	Myeloid	ITM2B

MERTK	0	1.131645	0.409	0.074	0	Myeloid	MERTK
CCL2	0	1.129331	0.339	0.134	0	Myeloid	CCL2
AC084871.	0	1.127165	0.28	0.055	0	Myeloid	AC084871.1
KLF6	0	1.126657	0.896	0.726	0	Myeloid	KLF6
VASH1	0	1.124556	0.473	0.096	0	Myeloid	VASH1
HLA-DMB	0	1.122572	0.542	0.139	0	Myeloid	HLA-DMB
ELMO1	0	1.120743	0.757	0.387	0	Myeloid	ELMO1
PHACTR1	0	1.119125	0.417	0.087	0	Myeloid	PHACTR1
AC007952.	0	1.11675	0.395	0.195	0	Myeloid	AC007952.4
RGS1	0	1.104012	0.841	0.605	0	Myeloid	RGS1
AZIN1-AS1	0	1.103774	0.36	0.087	0	Myeloid	AZIN1-AS1
EGR3	0	1.097945	0.459	0.114	0	Myeloid	EGR3
MFSD1	0	1.09787	0.56	0.16	0	Myeloid	MFSD1
ATF3	0	1.095031	0.584	0.245	0	Myeloid	ATF3
HSPB1	0	1.093091	0.462	0.298	0	Myeloid	HSPB1
DLEU1	0	1.092694	0.543	0.175	0	Myeloid	DLEU1
CTSZ	0	1.08916	0.639	0.214	0	Myeloid	CTSZ
ATP2B1-AS	0	1.087944	0.502	0.174	0	Myeloid	ATP2B1-AS1
HAVCR2	0	1.087041	0.57	0.179	0	Myeloid	HAVCR2
FCGR3A	0	1.084326	0.605	0.206	0	Myeloid	FCGR3A
CTTNBP2	0	1.079987	0.383	0.069	0	Myeloid	CTTNBP2
SOCS6	0	1.075716	0.386	0.069	0	Myeloid	SOCS6
CFD	0	1.075514	0.361	0.058	0	Myeloid	CFD
DBI	0	1.074554	0.721	0.447	0	Myeloid	DBI
DAGLB	0	1.072197	0.432	0.092	0	Myeloid	DAGLB
PTGS2	0	1.071809	0.33	0.073	0	Myeloid	PTGS2
TM6SF1	0	1.071807	0.482	0.093	0	Myeloid	TM6SF1
MANBA	0	1.071718	0.551	0.18	0	Myeloid	MANBA
GNB4	0	1.071361	0.461	0.09	0	Myeloid	GNB4
FTH1	0	1.071323	0.984	0.935	0	Myeloid	FTH1
RGS16	0	1.067563	0.386	0.148	0	Myeloid	RGS16
GAB2	0	1.065479	0.494	0.122	0	Myeloid	GAB2
CTSH	0	1.064254	0.543	0.134	0	Myeloid	CTSH
S100A11	0	1.063722	0.831	0.504	0	Myeloid	S100A11
RGS2	0	1.063371	0.564	0.29	0	Myeloid	RGS2
BMP2K	0	1.062706	0.478	0.111	0	Myeloid	BMP2K
FMN1	0	1.060133	0.407	0.095	0	Myeloid	FMN1
ALOX5	0	1.056253	0.473	0.093	0	Myeloid	ALOX5
NHSL1	0	1.053574	0.324	0.066	0	Myeloid	NHSL1
TMEM107	0	1.051509	0.419	0.184	0	Myeloid	TMEM107
LGMN	0	1.051478	0.425	0.093	0	Myeloid	LGMN
LINC01736	0	1.048749	0.433	0.087	0	Myeloid	LINC01736
CDKN1A	0	1.04611	0.587	0.293	0	Myeloid	CDKN1A
CD81	0	1.043393	0.846	0.623	0	Myeloid	CD81
PLA2G4A	0	1.042821	0.377	0.072	0	Myeloid	PLA2G4A
PLEK	0	1.041043	0.564	0.235	0	Myeloid	PLEK
HSPA1B	0	1.037809	0.667	0.427	0	Myeloid	HSPA1B

LST1	0	1.03641	0.502	0.119	0	Myeloid	LST1
ITGAX	0	1.033723	0.415	0.091	0	Myeloid	ITGAX
BAG3	0	1.033656	0.329	0.141	0	Myeloid	BAG3
ZFP36L1	0	1.032728	0.874	0.628	0	Myeloid	ZFP36L1
SYK	0	1.029759	0.512	0.12	0	Myeloid	SYK
IL1RAP	0	1.02869	0.4	0.096	0	Myeloid	IL1RAP
NR4A3	0	1.028634	0.633	0.36	0	Myeloid	NR4A3
VSIR	0	1.027359	0.72	0.38	0	Myeloid	VSIR
PDGFB	0	1.025614	0.358	0.063	0	Myeloid	PDGFB
NAMPT	0	1.023893	0.543	0.271	0	Myeloid	NAMPT
IGSF6	0	1.022735	0.47	0.094	0	Myeloid	IGSF6
ABL2	0	1.021494	0.385	0.115	0	Myeloid	ABL2
3-Mar	0	1.01908	0.476	0.169	0	Myeloid	3-Mar
ATP8B4	0	1.016438	0.461	0.108	0	Myeloid	ATP8B4
MYO1E	0	1.016103	0.357	0.095	0	Myeloid	MYO1E
RGS10	0	1.014155	0.753	0.412	0	Myeloid	RGS10
CKB	0	1.013347	0.396	0.091	0	Myeloid	CKB
YWHAH	0	1.012689	0.648	0.262	0	Myeloid	YWHAH
USP53	0	1.011669	0.436	0.139	0	Myeloid	USP53
ALDH2	0	1.011522	0.451	0.083	0	Myeloid	ALDH2
SORL1	0	1.009644	0.706	0.322	0	Myeloid	SORL1
IGSF21	0	1.005313	0.4	0.079	0	Myeloid	IGSF21
SERPINB9	0	0.997709	0.638	0.331	0	Myeloid	SERPINB9
NAV3	0	0.996661	0.317	0.061	0	Myeloid	NAV3
CROCC	0	0.99651	0.426	0.199	0	Myeloid	CROCC
RASGEF1B	0	0.990754	0.647	0.302	0	Myeloid	RASGEF1B
GNG7	0	0.986199	0.439	0.098	0	Myeloid	GNG7
ADAP2	0	0.983652	0.439	0.083	0	Myeloid	ADAP2
SHTN1	0	0.980667	0.425	0.079	0	Myeloid	SHTN1
MAP3K8	0	0.979333	0.665	0.358	0	Myeloid	MAP3K8
PILRA	0	0.974436	0.43	0.075	0	Myeloid	PILRA
SRGAP2C	0	0.970193	0.427	0.109	0	Myeloid	SRGAP2C
LGALS9	0	0.96329	0.551	0.178	0	Myeloid	LGALS9
TAL1	0	0.957885	0.327	0.053	0	Myeloid	TAL1
FGL2	0	0.957294	0.423	0.098	0	Myeloid	FGL2
RILPL2	0	0.955881	0.551	0.244	0	Myeloid	RILPL2
ATP6V1B2	0	0.951353	0.476	0.15	0	Myeloid	ATP6V1B2
ITGAV	0	0.950218	0.453	0.135	0	Myeloid	ITGAV
AC020916.	0	0.947695	0.586	0.288	0	Myeloid	AC020916.1
ALOX5AP	0	0.94212	0.747	0.419	0	Myeloid	ALOX5AP
FSCN1	0	0.940514	0.416	0.106	0	Myeloid	FSCN1
HBEGF	0	0.939995	0.3	0.079	0	Myeloid	HBEGF
OLFML3	0	0.939345	0.416	0.092	0	Myeloid	OLFML3
SH3TC1	0	0.938833	0.416	0.091	0	Myeloid	SH3TC1
CTSS	0	0.937875	0.623	0.262	0	Myeloid	CTSS
SDCCAG8	0	0.937218	0.515	0.156	0	Myeloid	SDCCAG8
IPCEF1	0	0.936185	0.509	0.294	0	Myeloid	IPCEF1

P2RY13	0	0.935289	0.344	0.059	0	Myeloid	P2RY13
CEP170	0	0.933777	0.46	0.119	0	Myeloid	CEP170
OXR1	0	0.933381	0.436	0.166	0	Myeloid	OXR1
ABR	0	0.932251	0.54	0.202	0	Myeloid	ABR
LRRK1	0	0.932072	0.338	0.058	0	Myeloid	LRRK1
DSCAM	0	0.931985	0.3	0.056	0	Myeloid	DSCAM
ATP6V0B	0	0.931542	0.657	0.317	0	Myeloid	ATP6V0B
FHIT	0	0.931115	0.434	0.124	0	Myeloid	FHIT
WDR74	0	0.930749	0.428	0.223	0	Myeloid	WDR74
EIF4E	0	0.929983	0.435	0.203	0	Myeloid	EIF4E
AP1B1	0	0.929605	0.543	0.18	0	Myeloid	AP1B1
HDAC9	0	0.929007	0.415	0.12	0	Myeloid	HDAC9
LINC02798	0	0.927745	0.331	0.068	0	Myeloid	LINC02798
KLHL6	0	0.926538	0.432	0.141	0	Myeloid	KLHL6
SLC31A2	0	0.923553	0.377	0.078	0	Myeloid	SLC31A2
RASGEF1C	0	0.91699	0.296	0.049	0	Myeloid	RASGEF1C
CNPY3	0	0.915484	0.658	0.298	0	Myeloid	CNPY3
PELI1	0	0.912599	0.547	0.224	0	Myeloid	PELI1
GAA	0	0.910441	0.426	0.094	0	Myeloid	GAA
SKIL	0	0.909448	0.675	0.356	0	Myeloid	SKIL
SH3RF3	0	0.907677	0.33	0.068	0	Myeloid	SH3RF3
FNIP2	0	0.907235	0.371	0.097	0	Myeloid	FNIP2
SFMBT2	0	0.906236	0.639	0.314	0	Myeloid	SFMBT2
CREG1	0	0.901641	0.458	0.121	0	Myeloid	CREG1
CTTNBP2NI	0	0.896966	0.373	0.07	0	Myeloid	CTTNBP2NL
ZSWIM6	0	0.893763	0.62	0.302	0	Myeloid	ZSWIM6
TIMP2	0	0.892748	0.462	0.108	0	Myeloid	TIMP2
PICALM	0	0.88663	0.686	0.321	0	Myeloid	PICALM
AL691403.:	0	0.886491	0.423	0.146	0	Myeloid	AL691403.1
AC012447.	0	0.885068	0.336	0.13	0	Myeloid	AC012447.1
ARHGAP6	0	0.884132	0.307	0.056	0	Myeloid	ARHGAP6
CTSL	0	0.884044	0.371	0.088	0	Myeloid	CTSL
SPRED1	0	0.883068	0.319	0.066	0	Myeloid	SPRED1
SLC37A2	0	0.882606	0.305	0.053	0	Myeloid	SLC37A2
RASAL2	0	0.880882	0.354	0.077	0	Myeloid	RASAL2
TMEM119	0	0.879946	0.396	0.08	0	Myeloid	TMEM119
STAB1	0	0.878964	0.347	0.069	0	Myeloid	STAB1
LAT2	0	0.877711	0.48	0.129	0	Myeloid	LAT2
NINJ1	0	0.877	0.509	0.19	0	Myeloid	NINJ1
ERCC1	0	0.87679	0.582	0.257	0	Myeloid	ERCC1
RNF144B	0	0.875802	0.307	0.07	0	Myeloid	RNF144B
CEBPA	0	0.874331	0.357	0.071	0	Myeloid	CEBPA
AC046195.	0	0.872812	0.276	0.046	0	Myeloid	AC046195.1
AL021155.!	0	0.871046	0.281	0.096	0	Myeloid	AL021155.5
GK	0	0.868714	0.36	0.092	0	Myeloid	GK
DSE	0	0.86829	0.454	0.154	0	Myeloid	DSE
MS4A4A	0	0.867386	0.378	0.079	0	Myeloid	MS4A4A

B4GALT1	0	0.86573	0.605	0.309	0	Myeloid	B4GALT1
GABARAP	0	0.865254	0.79	0.565	0	Myeloid	GABARAP
ALCAM	0	0.864221	0.378	0.114	0	Myeloid	ALCAM
DENND3	0	0.864006	0.426	0.115	0	Myeloid	DENND3
SIGLEC10	0	0.863256	0.352	0.063	0	Myeloid	SIGLEC10
SWAP70	0	0.861767	0.409	0.098	0	Myeloid	SWAP70
AXL	0	0.858074	0.362	0.069	0	Myeloid	AXL
LAIR1	0	0.857824	0.519	0.173	0	Myeloid	LAIR1
HAMP	0	0.854185	0.365	0.103	0	Myeloid	HAMP
ADAM17	0	0.853518	0.461	0.154	0	Myeloid	ADAM17
AL136987.:	0	0.851519	0.295	0.064	0	Myeloid	AL136987.1
ITPR2	0	0.850417	0.603	0.261	0	Myeloid	ITPR2
LINC01374	0	0.850402	0.292	0.05	0	Myeloid	LINC01374
IRF8	0	0.849987	0.434	0.119	0	Myeloid	IRF8
SLC25A37	0	0.849683	0.47	0.134	0	Myeloid	SLC25A37
BIN1	0	0.847593	0.605	0.339	0	Myeloid	BIN1
SKAP2	0	0.840724	0.488	0.149	0	Myeloid	SKAP2
ICAM1	0	0.839849	0.332	0.107	0	Myeloid	ICAM1
GNA13	0	0.836225	0.604	0.298	0	Myeloid	GNA13
NFKB1	0	0.832864	0.604	0.429	0	Myeloid	NFKB1
ETV6	0	0.832854	0.551	0.228	0	Myeloid	ETV6
EPB41L3	0	0.83166	0.358	0.073	0	Myeloid	EPB41L3
DUSP1	0	0.825578	0.943	0.854	0	Myeloid	DUSP1
SSPN	0	0.823982	0.318	0.064	0	Myeloid	SSPN
MCF2L2	0	0.823365	0.366	0.099	0	Myeloid	MCF2L2
CD63	0	0.822935	0.656	0.332	0	Myeloid	CD63
AC131944.	0	0.819812	0.272	0.063	0	Myeloid	AC131944.1
KCNMA1	0	0.816495	0.271	0.045	0	Myeloid	KCNMA1
TMEM176/	0	0.81625	0.336	0.063	0	Myeloid	TMEM176A
NPL	0	0.816033	0.338	0.069	0	Myeloid	NPL
KHDRBS3	0	0.8154	0.297	0.062	0	Myeloid	KHDRBS3
KDM6B	0	0.810472	0.549	0.295	0	Myeloid	KDM6B
KLF7	0	0.808694	0.332	0.076	0	Myeloid	KLF7
DST	0	0.808438	0.352	0.079	0	Myeloid	DST
CHKA	0	0.806119	0.403	0.117	0	Myeloid	CHKA
IFITM3	0	0.804089	0.465	0.252	0	Myeloid	IFITM3
IRS2	0	0.803945	0.438	0.16	0	Myeloid	IRS2
CAPG	0	0.802937	0.556	0.218	0	Myeloid	CAPG
PHC2	0	0.798238	0.443	0.142	0	Myeloid	PHC2
STX17-AS1	0	0.795352	0.416	0.181	0	Myeloid	STX17-AS1
KLF10	0	0.795267	0.41	0.185	0	Myeloid	KLF10
FCGR1B	0	0.794642	0.286	0.045	0	Myeloid	FCGR1B
BLNK	0	0.793207	0.349	0.071	0	Myeloid	BLNK
LRP1	0	0.789844	0.353	0.065	0	Myeloid	LRP1
TOM1	0	0.787088	0.466	0.185	0	Myeloid	TOM1
RUNX1	0	0.785174	0.601	0.311	0	Myeloid	RUNX1
SLC43A2	0	0.783938	0.332	0.066	0	Myeloid	SLC43A2

Z93241.1	0	0.782644	0.322	0.17	0	Myeloid	Z93241.1
IL6R	0	0.781231	0.358	0.086	0	Myeloid	IL6R
BRI3	0	0.780666	0.521	0.22	0	Myeloid	BRI3
ENTPD1	0	0.779198	0.364	0.082	0	Myeloid	ENTPD1
MGAT1	0	0.778374	0.499	0.224	0	Myeloid	MGAT1
HCK	0	0.777921	0.343	0.061	0	Myeloid	HCK
IER5	0	0.777733	0.523	0.346	0	Myeloid	IER5
SERPINF1	0	0.776686	0.343	0.074	0	Myeloid	SERPINF1
RNASE6	0	0.776447	0.353	0.071	0	Myeloid	RNASE6
OSM	0	0.773933	0.307	0.102	0	Myeloid	OSM
TBC1D12	0	0.772373	0.31	0.054	0	Myeloid	TBC1D12
ANXA5	0	0.77158	0.634	0.322	0	Myeloid	ANXA5
GALNT2	0	0.771056	0.444	0.157	0	Myeloid	GALNT2
RTN4	0	0.768142	0.632	0.315	0	Myeloid	RTN4
HPGDS	0	0.767604	0.308	0.06	0	Myeloid	HPGDS
MLXIPL	0	0.766372	0.32	0.059	0	Myeloid	MLXIPL
SCPEP1	0	0.765496	0.391	0.103	0	Myeloid	SCPEP1
WSB1	0	0.762109	0.713	0.43	0	Myeloid	WSB1
PTPRE	0	0.762041	0.507	0.218	0	Myeloid	PTPRE
MCF2L	0	0.761589	0.29	0.053	0	Myeloid	MCF2L
TNFSF13	0	0.76114	0.341	0.07	0	Myeloid	TNFSF13
FMNL3	0	0.760385	0.439	0.147	0	Myeloid	FMNL3
FCHSD2	0	0.760366	0.474	0.196	0	Myeloid	FCHSD2
TMEM52B	0	0.760234	0.315	0.059	0	Myeloid	TMEM52B
CPM	0	0.757635	0.276	0.055	0	Myeloid	CPM
MIS18BP1	0	0.754948	0.481	0.222	0	Myeloid	MIS18BP1
MIR222HG	0	0.754148	0.291	0.071	0	Myeloid	MIR222HG
CCDC88A	0	0.753759	0.391	0.105	0	Myeloid	CCDC88A
ATP2C1	0	0.753723	0.392	0.14	0	Myeloid	ATP2C1
MTSS1	0	0.752486	0.318	0.11	0	Myeloid	MTSS1
CPED1	0	0.751185	0.278	0.049	0	Myeloid	CPED1
LPAR6	0	0.751012	0.361	0.092	0	Myeloid	LPAR6
TCF4	0	0.747541	0.37	0.09	0	Myeloid	TCF4
TSC22D2	0	0.745435	0.533	0.275	0	Myeloid	TSC22D2
REL	0	0.745222	0.749	0.488	0	Myeloid	REL
STX11	0	0.74385	0.368	0.126	0	Myeloid	STX11
FGD2	0	0.743423	0.281	0.046	0	Myeloid	FGD2
SH2B3	0	0.742706	0.369	0.111	0	Myeloid	SH2B3
TNS3	0	0.742129	0.306	0.065	0	Myeloid	TNS3
AL390957.:	0	0.740327	0.332	0.095	0	Myeloid	AL390957.1
NRP2	0	0.738789	0.261	0.061	0	Myeloid	NRP2
TGFBR1	0	0.738018	0.436	0.155	0	Myeloid	TGFBR1
RAPH1	0	0.736885	0.32	0.072	0	Myeloid	RAPH1
PPT1	0	0.735134	0.544	0.225	0	Myeloid	PPT1
1-Mar	0	0.733116	0.294	0.06	0	Myeloid	1-Mar
SNX9	0	0.732242	0.549	0.296	0	Myeloid	SNX9
STK38L	0	0.732086	0.331	0.109	0	Myeloid	STK38L

NAIP	0	0.730185	0.36	0.085	0	Myeloid	NAIP
BTG2	0	0.729155	0.745	0.536	0	Myeloid	BTG2
ENSA	0	0.728098	0.634	0.404	0	Myeloid	ENSA
SAP30	0	0.726985	0.346	0.122	0	Myeloid	SAP30
APBB1IP	0	0.72518	0.777	0.512	0	Myeloid	APBB1IP
EPS8	0	0.72518	0.374	0.108	0	Myeloid	EPS8
THEMIS2	0	0.724874	0.399	0.121	0	Myeloid	THEMIS2
TSPO	0	0.722549	0.609	0.353	0	Myeloid	TSPO
AC103591.	0	0.721673	0.265	0.082	0	Myeloid	AC103591.3
METTL7A	0	0.721563	0.33	0.066	0	Myeloid	METTL7A
PPARD	0	0.718943	0.343	0.102	0	Myeloid	PPARD
NRIP1	0	0.717006	0.42	0.142	0	Myeloid	NRIP1
MNDA	0	0.71424	0.314	0.064	0	Myeloid	MNDA
ARHGAP26	0	0.713752	0.702	0.412	0	Myeloid	ARHGAP26
BNC2	0	0.711803	0.303	0.076	0	Myeloid	BNC2
CHSY1	0	0.711005	0.342	0.117	0	Myeloid	CHSY1
APLP2	0	0.708861	0.478	0.174	0	Myeloid	APLP2
WASF2	0	0.708744	0.632	0.329	0	Myeloid	WASF2
GRINA	0	0.707068	0.428	0.144	0	Myeloid	GRINA
GADD45B	0	0.705415	0.778	0.615	0	Myeloid	GADD45B
UNC93B1	0	0.705274	0.376	0.1	0	Myeloid	UNC93B1
H1F0	0	0.702289	0.276	0.065	0	Myeloid	H1F0
RFX2	0	0.702247	0.296	0.085	0	Myeloid	RFX2
SLC16A3	0	0.701156	0.375	0.126	0	Myeloid	SLC16A3
PDPN	0	0.69979	0.272	0.051	0	Myeloid	PDPN
PRKAG2	0	0.699472	0.357	0.125	0	Myeloid	PRKAG2
MAN1A1	0	0.698454	0.43	0.192	0	Myeloid	MAN1A1
ARL5A	0	0.698182	0.506	0.227	0	Myeloid	ARL5A
NUDT3	0	0.696509	0.45	0.163	0	Myeloid	NUDT3
MTHFD1L	0	0.694706	0.332	0.093	0	Myeloid	MTHFD1L
SIPA1L1	0	0.693589	0.564	0.351	0	Myeloid	SIPA1L1
CDK2AP1	0	0.693408	0.381	0.107	0	Myeloid	CDK2AP1
TRIO	0	0.69292	0.319	0.08	0	Myeloid	TRIO
TNF	0	0.692907	0.448	0.289	0	Myeloid	TNF
PABPC4	0	0.691769	0.478	0.205	0	Myeloid	PABPC4
FAM149A	0	0.688766	0.255	0.046	0	Myeloid	FAM149A
SIPA1L2	0	0.688579	0.25	0.052	0	Myeloid	SIPA1L2
JAZF1	0	0.688396	0.507	0.227	0	Myeloid	JAZF1
SDCBP	0	0.681383	0.641	0.359	0	Myeloid	SDCBP
NFKBIA	0	0.678369	0.832	0.743	0	Myeloid	NFKBIA
WDFY4	0	0.677566	0.283	0.055	0	Myeloid	WDFY4
SRGN	0	0.677054	0.938	0.859	0	Myeloid	SRGN
HCLS1	0	0.676463	0.791	0.546	0	Myeloid	HCLS1
HERPUD1	0	0.676309	0.705	0.517	0	Myeloid	HERPUD1
PTAFR	0	0.675958	0.293	0.053	0	Myeloid	PTAFR
EBI3	0	0.675369	0.277	0.047	0	Myeloid	EBI3
RAB1A	0	0.675251	0.535	0.257	0	Myeloid	RAB1A

P4HA1	0	0.674916	0.319	0.121	0	Myeloid	P4HA1
RB1	0	0.673701	0.521	0.22	0	Myeloid	RB1
NEDD9	0	0.673395	0.517	0.324	0	Myeloid	NEDD9
CD84	0	0.672899	0.404	0.134	0	Myeloid	CD84
ATF6	0	0.672692	0.409	0.156	0	Myeloid	ATF6
CXorf21	0	0.669816	0.272	0.068	0	Myeloid	CXorf21
CTNND1	0	0.667093	0.306	0.068	0	Myeloid	CTNND1
FAM49A	0	0.666918	0.331	0.103	0	Myeloid	FAM49A
LMO2	0	0.664725	0.319	0.061	0	Myeloid	LMO2
NFIC	0	0.662779	0.4	0.135	0	Myeloid	NFIC
KLF2	0	0.662131	0.719	0.489	0	Myeloid	KLF2
MYOF	0	0.658539	0.282	0.058	0	Myeloid	MYOF
SUSD6	0	0.658137	0.435	0.177	0	Myeloid	SUSD6
SIGLEC8	0	0.656549	0.255	0.039	0	Myeloid	SIGLEC8
SYNGR2	0	0.654016	0.536	0.272	0	Myeloid	SYNGR2
TYMP	0	0.653909	0.353	0.142	0	Myeloid	TYMP
SORT1	0	0.653656	0.287	0.06	0	Myeloid	SORT1
OTULINL	0	0.651954	0.431	0.163	0	Myeloid	OTULINL
TFRC	0	0.651625	0.326	0.154	0	Myeloid	TFRC
MGST2	0	0.650073	0.342	0.08	0	Myeloid	MGST2
PLSCR1	0	0.646988	0.475	0.197	0	Myeloid	PLSCR1
SIRPA	0	0.646198	0.294	0.056	0	Myeloid	SIRPA
TTYH3	0	0.645456	0.274	0.061	0	Myeloid	TTYH3
BAZ2B	0	0.643175	0.381	0.133	0	Myeloid	BAZ2B
SELPLG	0	0.642107	0.591	0.377	0	Myeloid	SELPLG
NCOR2	0	0.636513	0.452	0.191	0	Myeloid	NCOR2
AF213884.:	0	0.635316	0.269	0.073	0	Myeloid	AF213884.3
TCF12	0	0.635124	0.56	0.286	0	Myeloid	TCF12
TLR1	0	0.632666	0.298	0.068	0	Myeloid	TLR1
TRPM2	0	0.632326	0.277	0.06	0	Myeloid	TRPM2
MAT2A	0	0.631255	0.55	0.304	0	Myeloid	MAT2A
SLC29A1	0	0.629542	0.304	0.069	0	Myeloid	SLC29A1
PRAM1	0	0.628723	0.267	0.051	0	Myeloid	PRAM1
LY96	0	0.625126	0.295	0.065	0	Myeloid	LY96
RPS6KA2	0	0.624873	0.254	0.067	0	Myeloid	RPS6KA2
AFF4	0	0.622343	0.589	0.327	0	Myeloid	AFF4
RYR1	0	0.621053	0.252	0.061	0	Myeloid	RYR1
ATP13A3	0	0.620041	0.33	0.127	0	Myeloid	ATP13A3
LPAR5	0	0.61963	0.298	0.07	0	Myeloid	LPAR5
AKR1B1	0	0.619618	0.467	0.206	0	Myeloid	AKR1B1
CTSD	0	0.61883	0.668	0.465	0	Myeloid	CTSD
SLC9A9	0	0.616708	0.428	0.184	0	Myeloid	SLC9A9
MAN2B1	0	0.616572	0.433	0.167	0	Myeloid	MAN2B1
LAPTM5	0	0.615985	0.898	0.744	0	Myeloid	LAPTM5
LINC02256	0	0.615371	0.367	0.123	0	Myeloid	LINC02256
PPP1R15A	0	0.614183	0.746	0.592	0	Myeloid	PPP1R15A
RCC2	0	0.61386	0.357	0.124	0	Myeloid	RCC2

DAB2	0	0.610749	0.253	0.049	0	Myeloid	DAB2
ARHGAP21	0	0.610192	0.297	0.078	0	Myeloid	ARHGAP21
GATM	0	0.609781	0.279	0.057	0	Myeloid	GATM
RIN3	0	0.609463	0.455	0.192	0	Myeloid	RIN3
WDR91	0	0.608795	0.274	0.071	0	Myeloid	WDR91
SERPINB6	0	0.608756	0.402	0.134	0	Myeloid	SERPINB6
SLC7A7	0	0.608609	0.27	0.046	0	Myeloid	SLC7A7
HEXA	0	0.608575	0.443	0.192	0	Myeloid	HEXA
CHD9	0	0.606652	0.475	0.211	0	Myeloid	CHD9
NME2	0	0.605828	0.667	0.439	0	Myeloid	NME2
HIF1A-AS3	0	0.605528	0.266	0.088	0	Myeloid	HIF1A-AS3
ANKS1A	0	0.605048	0.319	0.099	0	Myeloid	ANKS1A
LTBR	0	0.603716	0.283	0.053	0	Myeloid	LTBR
ARRB2	0	0.60316	0.616	0.338	0	Myeloid	ARRB2
PLEKHO1	0	0.60171	0.474	0.211	0	Myeloid	PLEKHO1
DENND5A	0	0.601432	0.258	0.069	0	Myeloid	DENND5A
IL17RA	0	0.599668	0.404	0.163	0	Myeloid	IL17RA
MCL1	0	0.599433	0.82	0.646	0	Myeloid	MCL1
CMTM6	0	0.597268	0.504	0.238	0	Myeloid	CMTM6
BEST1	0	0.596189	0.359	0.139	0	Myeloid	BEST1
PTGS1	0	0.595151	0.268	0.049	0	Myeloid	PTGS1
SPRY2	0	0.591824	0.262	0.08	0	Myeloid	SPRY2
KLHL5	0	0.591559	0.311	0.093	0	Myeloid	KLHL5
ZMIZ1	0	0.590935	0.317	0.113	0	Myeloid	ZMIZ1
CIITA	0	0.590115	0.259	0.059	0	Myeloid	CIITA
SCAMP2	0	0.589822	0.503	0.243	0	Myeloid	SCAMP2
MED13L	0	0.588162	0.481	0.237	0	Myeloid	MED13L
CALHM6	0	0.588025	0.267	0.076	0	Myeloid	CALHM6
DIAPH2	0	0.583027	0.432	0.187	0	Myeloid	DIAPH2
GAL3ST4	0	0.581692	0.256	0.052	0	Myeloid	GAL3ST4
GAS6	0	0.581173	0.267	0.059	0	Myeloid	GAS6
SNX29	0	0.58084	0.378	0.148	0	Myeloid	SNX29
GBP2	0	0.580657	0.407	0.25	0	Myeloid	GBP2
HSPH1	0	0.575032	0.461	0.349	0	Myeloid	HSPH1
PRKCD	0	0.574916	0.3	0.083	0	Myeloid	PRKCD
AKAP13	0	0.573372	0.78	0.615	0	Myeloid	AKAP13
EVI5	0	0.572925	0.259	0.058	0	Myeloid	EVI5
LAPTM4A	0	0.572852	0.513	0.251	0	Myeloid	LAPTM4A
RAB32	0	0.571028	0.254	0.049	0	Myeloid	RAB32
SPRY1	0	0.569272	0.253	0.102	0	Myeloid	SPRY1
IL6ST	0	0.568065	0.515	0.267	0	Myeloid	IL6ST
MKNK1	0	0.568016	0.328	0.113	0	Myeloid	MKNK1
CHMP4B	0	0.566985	0.343	0.127	0	Myeloid	CHMP4B
CPEB4	0	0.565283	0.294	0.101	0	Myeloid	CPEB4
STX7	0	0.564659	0.32	0.098	0	Myeloid	STX7
FLOT1	0	0.563975	0.42	0.171	0	Myeloid	FLOT1
YBX3	0	0.562068	0.402	0.152	0	Myeloid	YBX3

FILIP1L	0	0.561038	0.27	0.091	0	Myeloid	FILIP1L
PDE8A	0	0.560721	0.315	0.136	0	Myeloid	PDE8A
DISC1	0	0.559278	0.383	0.155	0	Myeloid	DISC1
CPQ	0	0.558928	0.346	0.126	0	Myeloid	CPQ
ATP6AP1	0	0.558758	0.444	0.199	0	Myeloid	ATP6AP1
ADGRG1	0	0.55835	0.344	0.128	0	Myeloid	ADGRG1
GSTM2	0	0.55744	0.296	0.093	0	Myeloid	GSTM2
PYCARD	0	0.557161	0.483	0.244	0	Myeloid	PYCARD
CYBA	0	0.556889	0.887	0.765	0	Myeloid	CYBA
LYL1	0	0.553726	0.271	0.061	0	Myeloid	LYL1
FEZ2	0	0.550688	0.304	0.098	0	Myeloid	FEZ2
TTC7A	0	0.548898	0.345	0.142	0	Myeloid	TTC7A
PLIN2	0	0.547661	0.33	0.224	0	Myeloid	PLIN2
ADAMTSL4	0	0.54535	0.336	0.143	0	Myeloid	ADAMTSL4-AS1
IER2	0	0.543972	0.834	0.71	0	Myeloid	IER2
BACH1	0	0.543618	0.381	0.151	0	Myeloid	BACH1
NUMB	0	0.542527	0.373	0.152	0	Myeloid	NUMB
TSPAN3	0	0.539297	0.328	0.1	0	Myeloid	TSPAN3
PACSIN2	0	0.536909	0.354	0.139	0	Myeloid	PACSIN2
DPYSL2	0	0.536715	0.283	0.073	0	Myeloid	DPYSL2
IRF5	0	0.536565	0.262	0.061	0	Myeloid	IRF5
RNF13	0	0.535627	0.415	0.175	0	Myeloid	RNF13
DUSP6	0	0.53545	0.288	0.132	0	Myeloid	DUSP6
RNASE2	0	0.535135	0.259	0.066	0	Myeloid	RNASE2
SOAT1	0	0.53369	0.341	0.127	0	Myeloid	SOAT1
BCL6	0	0.529542	0.333	0.14	0	Myeloid	BCL6
ADAM9	0	0.529372	0.264	0.066	0	Myeloid	ADAM9
ARHGAP12	0	0.528038	0.281	0.091	0	Myeloid	ARHGAP12
PAK1	0	0.527973	0.29	0.087	0	Myeloid	PAK1
MPP1	0	0.527663	0.266	0.079	0	Myeloid	MPP1
LCP2	0	0.527118	0.619	0.513	0	Myeloid	LCP2
ZNF267	0	0.526058	0.339	0.143	0	Myeloid	ZNF267
ABHD12	0	0.525564	0.371	0.147	0	Myeloid	ABHD12
STX4	0	0.523555	0.308	0.142	0	Myeloid	STX4
RAPGEF1	0	0.522651	0.539	0.318	0	Myeloid	RAPGEF1
FOXO3	0	0.522429	0.385	0.193	0	Myeloid	FOXO3
CTNNB1	0	0.520123	0.49	0.275	0	Myeloid	CTNNB1
CD4	0	0.519161	0.402	0.155	0	Myeloid	CD4
GRB2	0	0.519158	0.582	0.338	0	Myeloid	GRB2
PMEPA1	0	0.515998	0.356	0.153	0	Myeloid	PMEPA1
CD300A	0	0.515517	0.378	0.143	0	Myeloid	CD300A
PARVB	0	0.51473	0.294	0.103	0	Myeloid	PARVB
RAB7A	0	0.51446	0.606	0.392	0	Myeloid	RAB7A
AC022217.	0	0.512811	0.427	0.226	0	Myeloid	AC022217.3
SPECC1	0	0.512387	0.278	0.082	0	Myeloid	SPECC1
GLA	0	0.51178	0.265	0.106	0	Myeloid	GLA
COMT	0	0.511676	0.398	0.167	0	Myeloid	COMT

DIP2B	0	0.509848	0.32	0.132	0	Myeloid	DIP2B
AIG1	0	0.509422	0.281	0.079	0	Myeloid	AIG1
PLK3	0	0.509168	0.458	0.249	0	Myeloid	PLK3
RANBP2	0	0.50887	0.57	0.424	0	Myeloid	RANBP2
GPX4	0	0.50535	0.636	0.422	0	Myeloid	GPX4
SPIDR	0	0.503468	0.492	0.255	0	Myeloid	SPIDR
SEC14L1	0	0.502929	0.372	0.173	0	Myeloid	SEC14L1
RABGEF1	0	0.499552	0.396	0.228	0	Myeloid	RABGEF1
HEXB	0	0.499529	0.364	0.149	0	Myeloid	HEXB
FOSB	0	0.498719	0.769	0.602	0	Myeloid	FOSB
H2AFY	0	0.497743	0.518	0.283	0	Myeloid	H2AFY
PFKFB3	0	0.497091	0.568	0.339	0	Myeloid	PFKFB3
ZEB2	0	0.496492	0.612	0.335	0	Myeloid	ZEB2
VMP1	0	0.493527	0.454	0.227	0	Myeloid	VMP1
NFIL3	0	0.493177	0.323	0.155	0	Myeloid	NFIL3
RASSF2	0	0.490201	0.3	0.106	0	Myeloid	RASSF2
SQSTM1	0	0.487065	0.588	0.425	0	Myeloid	SQSTM1
CUX1	0	0.485768	0.399	0.182	0	Myeloid	CUX1
SBF2	0	0.484964	0.33	0.13	0	Myeloid	SBF2
ATG7	0	0.484701	0.361	0.163	0	Myeloid	ATG7
TNFSF13B	0	0.484638	0.254	0.073	0	Myeloid	TNFSF13B
PEA15	0	0.484578	0.296	0.102	0	Myeloid	PEA15
ATP6V0C	0	0.483563	0.696	0.507	0	Myeloid	ATP6V0C
RHEB	0	0.483334	0.457	0.242	0	Myeloid	RHEB
SMAP2	0	0.480328	0.77	0.584	0	Myeloid	SMAP2
PLD3	0	0.479353	0.348	0.16	0	Myeloid	PLD3
RANBP9	0	0.478073	0.416	0.22	0	Myeloid	RANBP9
TUBA1B	0	0.47648	0.809	0.559	0	Myeloid	TUBA1B
TNFRSF1A	0	0.474096	0.351	0.14	0	Myeloid	TNFRSF1A
AKR1A1	0	0.473854	0.388	0.174	0	Myeloid	AKR1A1
PDXK	0	0.469218	0.338	0.139	0	Myeloid	PDXK
POU2F2	0	0.468894	0.29	0.101	0	Myeloid	POU2F2
BST2	0	0.466954	0.55	0.362	0	Myeloid	BST2
NAGK	0	0.466563	0.298	0.106	0	Myeloid	NAGK
ENG	0	0.463734	0.299	0.088	0	Myeloid	ENG
NCOA4	0	0.46166	0.385	0.185	0	Myeloid	NCOA4
ATP6V1F	0	0.459964	0.521	0.308	0	Myeloid	ATP6V1F
NFKBIZ	0	0.459806	0.535	0.395	0	Myeloid	NFKBIZ
PTPRJ	0	0.459589	0.471	0.275	0	Myeloid	PTPRJ
FOXN3	0	0.457947	0.692	0.476	0	Myeloid	FOXN3
NAGA	0	0.456809	0.25	0.064	0	Myeloid	NAGA
DHRS7	0	0.456072	0.567	0.351	0	Myeloid	DHRS7
SPATA13	0	0.454045	0.426	0.23	0	Myeloid	SPATA13
NR4A2	0	0.45044	0.789	0.672	0	Myeloid	NR4A2
MYO9B	0	0.446204	0.486	0.282	0	Myeloid	MYO9B
DOCK8	0	0.444799	0.69	0.47	0	Myeloid	DOCK8
SPINT2	0	0.444512	0.292	0.093	0	Myeloid	SPINT2

ZFAND5	0	0.443768	0.5	0.374	0	Myeloid	ZFAND5
RHOQ	0	0.443667	0.265	0.095	0	Myeloid	RHOQ
TET2	0	0.443078	0.326	0.153	0	Myeloid	TET2
CHD7	0	0.442453	0.315	0.132	0	Myeloid	CHD7
SEC11A	0	0.441438	0.507	0.302	0	Myeloid	SEC11A
FRMD4B	0	0.441107	0.321	0.138	0	Myeloid	FRMD4B
PTTG1IP	0	0.44104	0.388	0.167	0	Myeloid	PTTG1IP
USP4	0	0.440848	0.373	0.183	0	Myeloid	USP4
NUDT16	0	0.440499	0.251	0.077	0	Myeloid	NUDT16
DLEU2	0	0.437786	0.401	0.196	0	Myeloid	DLEU2
EIF4A1	0	0.436609	0.803	0.625	0	Myeloid	EIF4A1
TMEM14C	0	0.436587	0.413	0.199	0	Myeloid	TMEM14C
GLRX	0	0.435333	0.361	0.192	0	Myeloid	GLRX
ATP6V0E1	0	0.435081	0.636	0.432	0	Myeloid	ATP6V0E1
CTNNA1	0	0.434189	0.282	0.096	0	Myeloid	CTNNA1
LIPA	0	0.432843	0.335	0.149	0	Myeloid	LIPA
CHCHD10	0	0.432337	0.484	0.277	0	Myeloid	CHCHD10
SSH2	0	0.431677	0.617	0.411	0	Myeloid	SSH2
APPL2	0	0.431459	0.276	0.105	0	Myeloid	APPL2
ITGAM	0	0.427918	0.268	0.093	0	Myeloid	ITGAM
NAA20	0	0.427868	0.295	0.127	0	Myeloid	NAA20
DNASE2	0	0.425836	0.284	0.108	0	Myeloid	DNASE2
LINC00963	0	0.425654	0.277	0.107	0	Myeloid	LINC00963
CRTAP	0	0.421258	0.33	0.145	0	Myeloid	CRTAP
MFSD12	0	0.421239	0.268	0.097	0	Myeloid	MFSD12
CNDP2	0	0.421196	0.359	0.161	0	Myeloid	CNDP2
SNAP23	0	0.419826	0.318	0.145	0	Myeloid	SNAP23
TMEM156	0	0.419491	0.299	0.134	0	Myeloid	TMEM156
CCDC50	0	0.418741	0.27	0.094	0	Myeloid	CCDC50
CACUL1	0	0.417209	0.315	0.136	0	Myeloid	CACUL1
TBC1D22A	0	0.415855	0.446	0.244	0	Myeloid	TBC1D22A
RERE	0	0.414526	0.425	0.229	0	Myeloid	RERE
MDM2	0	0.413517	0.258	0.131	0	Myeloid	MDM2
UBE2E1	0	0.413495	0.329	0.149	0	Myeloid	UBE2E1
TPP1	0	0.411274	0.343	0.152	0	Myeloid	TPP1
APMAP	0	0.406417	0.51	0.31	0	Myeloid	APMAP
P2RX4	0	0.404999	0.259	0.096	0	Myeloid	P2RX4
FKBP5	0	0.404947	0.709	0.531	0	Myeloid	FKBP5
PAG1	0	0.404653	0.437	0.247	0	Myeloid	PAG1
CTDSP1	0	0.404168	0.326	0.182	0	Myeloid	CTDSP1
TRA2B	0	0.403554	0.57	0.41	0	Myeloid	TRA2B
SPAG9	0	0.402944	0.384	0.202	0	Myeloid	SPAG9
PLCB2	0	0.402535	0.322	0.142	0	Myeloid	PLCB2
C20orf27	0	0.402443	0.285	0.112	0	Myeloid	C20orf27
STX6	0	0.402123	0.252	0.091	0	Myeloid	STX6
NCF4	0	0.399894	0.285	0.105	0	Myeloid	NCF4
NEU1	0	0.399014	0.365	0.208	0	Myeloid	NEU1

PER1	0	0.397026	0.572	0.366	0	Myeloid	PER1
MAN2A1	0	0.393782	0.372	0.202	0	Myeloid	MAN2A1
GNG5	0	0.391139	0.622	0.416	0	Myeloid	GNG5
LAP3	0	0.390897	0.393	0.222	0	Myeloid	LAP3
NANS	0	0.389599	0.314	0.143	0	Myeloid	NANS
TKT	0	0.387398	0.4	0.237	0	Myeloid	TKT
UBA7	0	0.386046	0.275	0.111	0	Myeloid	UBA7
SLC1A5	0	0.385792	0.33	0.156	0	Myeloid	SLC1A5
DDAH2	0	0.383716	0.27	0.109	0	Myeloid	DDAH2
WASHC4	0	0.381542	0.315	0.14	0	Myeloid	WASHC4
RNASEK	0	0.378557	0.709	0.544	0	Myeloid	RNASEK
LAMP2	0	0.378321	0.278	0.107	0	Myeloid	LAMP2
TAB2	0	0.376384	0.44	0.23	0	Myeloid	TAB2
JAK2	0	0.375289	0.293	0.14	0	Myeloid	JAK2
NAF1	0	0.372244	0.254	0.129	0	Myeloid	NAF1
LRCH1	0	0.370088	0.299	0.142	0	Myeloid	LRCH1
DDX3Y	0	0.367903	0.346	0.214	0	Myeloid	DDX3Y
UBE2W	0	0.367123	0.286	0.123	0	Myeloid	UBE2W
WDFY2	0	0.366995	0.289	0.134	0	Myeloid	WDFY2
ARL8B	0	0.366248	0.332	0.172	0	Myeloid	ARL8B
PIK3AP1	0	0.365624	0.298	0.127	0	Myeloid	PIK3AP1
TUT7	0	0.365525	0.325	0.172	0	Myeloid	TUT7
SAT2	0	0.363618	0.328	0.153	0	Myeloid	SAT2
ERGIC1	0	0.362799	0.344	0.168	0	Myeloid	ERGIC1
SH3BGRL	0	0.360519	0.574	0.39	0	Myeloid	SH3BGRL
FBXW11	0	0.360056	0.339	0.171	0	Myeloid	FBXW11
FAM102B	0	0.356491	0.268	0.126	0	Myeloid	FAM102B
TPM4	0	0.355524	0.424	0.272	0	Myeloid	TPM4
RGS19	0	0.355338	0.402	0.225	0	Myeloid	RGS19
ATP2A2	0	0.354719	0.302	0.159	0	Myeloid	ATP2A2
ETF1	0	0.351051	0.404	0.23	0	Myeloid	ETF1
DBNDD2	0	0.350627	0.252	0.086	0	Myeloid	DBNDD2
UBAC2	0	0.350408	0.478	0.306	0	Myeloid	UBAC2
ARAP1	0	0.348464	0.253	0.105	0	Myeloid	ARAP1
OS9	0	0.347939	0.428	0.241	0	Myeloid	OS9
FNDC3A	0	0.347132	0.349	0.183	0	Myeloid	FNDC3A
ZBTB16	0	0.34606	0.366	0.229	0	Myeloid	ZBTB16
UBE2F	0	0.345778	0.314	0.162	0	Myeloid	UBE2F
TMBIM4	0	0.345726	0.521	0.327	0	Myeloid	TMBIM4
ADRB2	0	0.344757	0.298	0.152	0	Myeloid	ADRB2
ADPGK	0	0.339738	0.314	0.147	0	Myeloid	ADPGK
ACSL3	0	0.339205	0.262	0.133	0	Myeloid	ACSL3
NSMCE2	0	0.335961	0.349	0.181	0	Myeloid	NSMCE2
TBC1D14	0	0.335819	0.276	0.135	0	Myeloid	TBC1D14
AC004687.	0	0.331939	0.283	0.149	0	Myeloid	AC004687.1
CD58	0	0.330803	0.364	0.201	0	Myeloid	CD58
ATP6V0D1	0	0.330486	0.344	0.177	0	Myeloid	ATP6V0D1

PRCP	0	0.329663	0.255	0.11	0	Myeloid	PRCP
ID2	0	0.329605	0.661	0.534	0	Myeloid	ID2
DRAM2	0	0.328538	0.265	0.115	0	Myeloid	DRAM2
RRBP1	0	0.327372	0.279	0.129	0	Myeloid	RRBP1
PTPN18	0	0.326571	0.336	0.17	0	Myeloid	PTPN18
BNIP3L	0	0.325649	0.345	0.183	0	Myeloid	BNIP3L
SVBP	0	0.325177	0.254	0.112	0	Myeloid	SVBP
CIAO2A	0	0.323533	0.345	0.181	0	Myeloid	CIAO2A
PDE4DIP	0	0.323339	0.294	0.153	0	Myeloid	PDE4DIP
MAML2	0	0.323294	0.553	0.343	0	Myeloid	MAML2
MAP2K3	0	0.321703	0.277	0.164	0	Myeloid	MAP2K3
GSTP1	0	0.317487	0.581	0.399	0	Myeloid	GSTP1
ARHGAP18	0	0.317011	0.32	0.169	0	Myeloid	ARHGAP18
JMJD1C	0	0.316864	0.595	0.408	0	Myeloid	JMJD1C
PTPN6	0	0.316181	0.412	0.244	0	Myeloid	PTPN6
MICAL1	0	0.315893	0.291	0.139	0	Myeloid	MICAL1
TLN1	0	0.313819	0.56	0.371	0	Myeloid	TLN1
TMEM219	0	0.313658	0.437	0.268	0	Myeloid	TMEM219
FAM49B	0	0.312708	0.644	0.439	0	Myeloid	FAM49B
TAOK3	0	0.312303	0.452	0.289	0	Myeloid	TAOK3
CCDC47	0	0.310106	0.312	0.149	0	Myeloid	CCDC47
ZFAND3	0	0.309746	0.503	0.314	0	Myeloid	ZFAND3
KDM4B	0	0.308917	0.273	0.143	0	Myeloid	KDM4B
ZNF106	0	0.308795	0.306	0.154	0	Myeloid	ZNF106
SLC4A7	0	0.308541	0.438	0.264	0	Myeloid	SLC4A7
UVRAG	0	0.307944	0.381	0.212	0	Myeloid	UVRAG
CLTA	0	0.307808	0.453	0.27	0	Myeloid	CLTA
SNX2	0	0.307369	0.363	0.191	0	Myeloid	SNX2
CAPZB	0	0.307316	0.76	0.601	0	Myeloid	CAPZB
CAPZA2	0	0.306995	0.414	0.235	0	Myeloid	CAPZA2
HIPK3	0	0.306033	0.278	0.138	0	Myeloid	HIPK3
YWHAE	0	0.305916	0.466	0.27	0	Myeloid	YWHAE
TNFRSF1B	0	0.30568	0.522	0.342	0	Myeloid	TNFRSF1B
FAM53B	0	0.305027	0.28	0.144	0	Myeloid	FAM53B
MGST3	0	0.304678	0.387	0.22	0	Myeloid	MGST3
FNIP1	0	0.304472	0.326	0.168	0	Myeloid	FNIP1
OAZ1	0	0.304228	0.798	0.697	0	Myeloid	OAZ1
SMS	0	0.303295	0.305	0.16	0	Myeloid	SMS
RPS6KA1	0	0.301483	0.303	0.157	0	Myeloid	RPS6KA1
PTPN1	0	0.301408	0.409	0.288	0	Myeloid	PTPN1
HSBP1	0	0.298247	0.286	0.135	0	Myeloid	HSBP1
GNG10	0	0.295548	0.279	0.134	0	Myeloid	GNG10
ABI3	0	0.294814	0.402	0.238	0	Myeloid	ABI3
STXBP2	0	0.293389	0.369	0.22	0	Myeloid	STXBP2
IFRD1	0	0.29263	0.501	0.368	0	Myeloid	IFRD1
FBXO11	0	0.292119	0.36	0.204	0	Myeloid	FBXO11
HIVEP3	0	0.291716	0.287	0.164	0	Myeloid	HIVEP3

RHOG	0	0.291375	0.515	0.343	0	Myeloid	RHOG
GRAMD1B	0	0.289596	0.297	0.164	0	Myeloid	GRAMD1B
PRDX1	0	0.289204	0.504	0.342	0	Myeloid	PRDX1
NT5C2	0	0.288853	0.273	0.138	0	Myeloid	NT5C2
MTHFD2	0	0.288178	0.352	0.206	0	Myeloid	MTHFD2
PARVG	0	0.287111	0.418	0.245	0	Myeloid	PARVG
HSP90AB1	0	0.286215	0.84	0.733	0	Myeloid	HSP90AB1
PPP1R10	0	0.285778	0.449	0.284	0	Myeloid	PPP1R10
ACTR2	0	0.284559	0.605	0.424	0	Myeloid	ACTR2
NCKAP1L	0	0.283034	0.391	0.225	0	Myeloid	NCKAP1L
PGLS	0	0.282796	0.382	0.233	0	Myeloid	PGLS
ANKRD10	0	0.282031	0.358	0.212	0	Myeloid	ANKRD10
VAMP8	0	0.281541	0.525	0.385	0	Myeloid	VAMP8
TOP1	0	0.281133	0.474	0.313	0	Myeloid	TOP1
ARPC3	0	0.278078	0.767	0.641	0	Myeloid	ARPC3
SESN1	0	0.277212	0.301	0.162	0	Myeloid	SESN1
CANX	0	0.276898	0.493	0.324	0	Myeloid	CANX
TGIF1	0	0.27421	0.257	0.134	0	Myeloid	TGIF1
RNF149	0	0.273696	0.564	0.391	0	Myeloid	RNF149
MAP2K1	0	0.272085	0.354	0.213	0	Myeloid	MAP2K1
SERPINB1	0	0.270703	0.4	0.254	0	Myeloid	SERPINB1
ATP6AP2	0	0.27041	0.402	0.246	0	Myeloid	ATP6AP2
PLCG2	0	0.269788	0.271	0.125	0	Myeloid	PLCG2
MAP4K4	0	0.269673	0.372	0.217	0	Myeloid	MAP4K4
XBP1	0	0.269507	0.494	0.353	0	Myeloid	XBP1
CLTC	0	0.268759	0.356	0.196	0	Myeloid	CLTC
EPN1	0	0.268699	0.27	0.131	0	Myeloid	EPN1
SUMF1	0	0.267869	0.265	0.135	0	Myeloid	SUMF1
MPC2	0	0.267818	0.362	0.23	0	Myeloid	MPC2
MAGT1	0	0.266914	0.258	0.132	0	Myeloid	MAGT1
MT-ND1	0	0.266881	0.899	0.81	0	Myeloid	MT-ND1
FAM53C	0	0.266737	0.304	0.179	0	Myeloid	FAM53C
HMGN3	0	0.266577	0.42	0.259	0	Myeloid	HMGN3
LAMTOR1	0	0.265877	0.4	0.247	0	Myeloid	LAMTOR1
PREX1	0	0.263507	0.592	0.415	0	Myeloid	PREX1
SNX3	0	0.262632	0.493	0.332	0	Myeloid	SNX3
PKN2	0	0.26011	0.326	0.18	0	Myeloid	PKN2
RTF2	0	0.25934	0.358	0.206	0	Myeloid	RTF2
PNPLA8	0	0.259057	0.29	0.173	0	Myeloid	PNPLA8
HOOK3	0	0.258811	0.294	0.159	0	Myeloid	HOOK3
CSNK1A1	0	0.257212	0.502	0.337	0	Myeloid	CSNK1A1
ITGB2	0	0.254677	0.74	0.566	0	Myeloid	ITGB2
ADA2	0	0.254024	0.285	0.151	0	Myeloid	ADA2
HNRNPH2	0	0.253887	0.307	0.167	0	Myeloid	HNRNPH2
LAMP1	0	0.252041	0.444	0.294	0	Myeloid	LAMP1
GNPTG	0	0.250108	0.283	0.148	0	Myeloid	GNPTG
MT2A	2.63E-273	0.36686	0.389	0.489	7.41E-269	Myeloid	MT2A

JUN	2.42E-260	0.460669	0.864	0.8	6.82E-256	Myeloid	JUN
CKS2	2.11E-254	0.257088	0.293	0.2	5.95E-250	Myeloid	CKS2
HSPD1	2.34E-229	0.326425	0.496	0.383	6.59E-225	Myeloid	HSPD1
HSP90AA1	1.63E-143	0.430065	0.853	0.793	4.57E-139	Myeloid	HSP90AA1
IFI6	2.71E-21	0.284726	0.282	0.269	7.62E-17	Myeloid	IFI6
CCL5	0	3.597543	0.732	0.127	0	T/NK Cells	CCL5
IL32	0	3.596433	0.765	0.035	0	T/NK Cells	IL32
NKG7	0	3.374087	0.532	0.04	0	T/NK Cells	NKG7
IL7R	0	3.241202	0.495	0.023	0	T/NK Cells	IL7R
CD3E	0	3.070378	0.745	0.024	0	T/NK Cells	CD3E
IFITM1	0	2.978666	0.779	0.129	0	T/NK Cells	IFITM1
FYN	0	2.907474	0.763	0.064	0	T/NK Cells	FYN
GZMK	0	2.735606	0.459	0.014	0	T/NK Cells	GZMK
CST7	0	2.685841	0.594	0.035	0	T/NK Cells	CST7
GZMA	0	2.681215	0.56	0.018	0	T/NK Cells	GZMA
CD52	0	2.63841	0.67	0.046	0	T/NK Cells	CD52
ETS1	0	2.630508	0.733	0.075	0	T/NK Cells	ETS1
CD3D	0	2.446111	0.608	0.017	0	T/NK Cells	CD3D
CD2	0	2.322717	0.579	0.017	0	T/NK Cells	CD2
CTSW	0	2.314773	0.506	0.03	0	T/NK Cells	CTSW
CD96	0	2.314297	0.593	0.021	0	T/NK Cells	CD96
SKAP1	0	2.288616	0.577	0.022	0	T/NK Cells	SKAP1
KLRB1	0	2.283722	0.359	0.011	0	T/NK Cells	KLRB1
PARP8	0	2.281168	0.713	0.216	0	T/NK Cells	PARP8
CD8A	0	2.26361	0.392	0.013	0	T/NK Cells	CD8A
CD247	0	2.254581	0.563	0.021	0	T/NK Cells	CD247
CBLB	0	2.228126	0.595	0.103	0	T/NK Cells	CBLB
TXNIP	0	2.221822	0.729	0.327	0	T/NK Cells	TXNIP
IL2RG	0	2.191607	0.624	0.054	0	T/NK Cells	IL2RG
RUNX3	0	2.158995	0.574	0.052	0	T/NK Cells	RUNX3
CEMIP2	0	2.154384	0.559	0.108	0	T/NK Cells	CEMIP2
CRIP1	0	2.143826	0.587	0.048	0	T/NK Cells	CRIP1
CD7	0	2.12684	0.521	0.131	0	T/NK Cells	CD7
TUBA4A	0	2.119934	0.517	0.026	0	T/NK Cells	TUBA4A
PTPRCAP	0	2.102571	0.441	0.02	0	T/NK Cells	PTPRCAP
STAT4	0	2.0916	0.527	0.042	0	T/NK Cells	STAT4
RAC2	0	2.046537	0.661	0.158	0	T/NK Cells	RAC2
PITPNC1	0	1.969368	0.524	0.089	0	T/NK Cells	PITPNC1
PPDPF	0	1.964635	0.727	0.268	0	T/NK Cells	PPDPF
GZMH	0	1.955212	0.341	0.012	0	T/NK Cells	GZMH
CNOT6L	0	1.954405	0.511	0.125	0	T/NK Cells	CNOT6L
BCL11B	0	1.929544	0.452	0.015	0	T/NK Cells	BCL11B
BTG1	0	1.923034	0.934	0.752	0	T/NK Cells	BTG1
PRF1	0	1.909747	0.355	0.014	0	T/NK Cells	PRF1
LCK	0	1.906157	0.53	0.015	0	T/NK Cells	LCK
TRBC2	0	1.885592	0.462	0.015	0	T/NK Cells	TRBC2
RORA	0	1.871134	0.422	0.023	0	T/NK Cells	RORA

SPOCK2	0	1.866884	0.462	0.018	0	T/NK Cells	SPOCK2
TC2N	0	1.860093	0.446	0.012	0	T/NK Cells	TC2N
ISG20	0	1.855158	0.582	0.107	0	T/NK Cells	ISG20
ITK	0	1.837302	0.43	0.016	0	T/NK Cells	ITK
SLC38A1	0	1.828253	0.477	0.031	0	T/NK Cells	SLC38A1
CYTIP	0	1.790485	0.643	0.188	0	T/NK Cells	CYTIP
LEPROTL1	0	1.78905	0.625	0.226	0	T/NK Cells	LEPROTL1
KLRD1	0	1.783466	0.287	0.013	0	T/NK Cells	KLRD1
PLAAT4	0	1.765403	0.591	0.189	0	T/NK Cells	PLAAT4
PPP1R16B	0	1.755388	0.394	0.025	0	T/NK Cells	PPP1R16B
CD8B	0	1.748202	0.301	0.009	0	T/NK Cells	CD8B
CAMK4	0	1.744345	0.392	0.015	0	T/NK Cells	CAMK4
TLE5	0	1.743256	0.628	0.21	0	T/NK Cells	TLE5
GNG2	0	1.73209	0.525	0.133	0	T/NK Cells	GNG2
SYNE2	0	1.730904	0.413	0.022	0	T/NK Cells	SYNE2
DUSP2	0	1.728496	0.738	0.402	0	T/NK Cells	DUSP2
ARL4C	0	1.718029	0.561	0.141	0	T/NK Cells	ARL4C
CXCR4	0	1.715086	0.803	0.457	0	T/NK Cells	CXCR4
LTB	0	1.71465	0.355	0.035	0	T/NK Cells	LTB
CD3G	0	1.709478	0.441	0.012	0	T/NK Cells	CD3G
S100A4	0	1.697433	0.664	0.296	0	T/NK Cells	S100A4
TENT5C	0	1.695536	0.47	0.066	0	T/NK Cells	TENT5C
CD44	0	1.691918	0.605	0.142	0	T/NK Cells	CD44
RPS27	0	1.690927	0.938	0.875	0	T/NK Cells	RPS27
TOX	0	1.682601	0.305	0.015	0	T/NK Cells	TOX
THEMIS	0	1.682506	0.351	0.012	0	T/NK Cells	THEMIS
OXNAD1	0	1.654539	0.423	0.049	0	T/NK Cells	OXNAD1
KLRK1	0	1.652738	0.362	0.009	0	T/NK Cells	KLRK1
SMCHD1	0	1.646334	0.614	0.283	0	T/NK Cells	SMCHD1
GZMM	0	1.579385	0.398	0.01	0	T/NK Cells	GZMM
ADGRE5	0	1.566285	0.522	0.144	0	T/NK Cells	ADGRE5
SEPTIN9	0	1.56171	0.516	0.099	0	T/NK Cells	SEPTIN9
PPP2R5C	0	1.559545	0.54	0.194	0	T/NK Cells	PPP2R5C
ITM2A	0	1.539305	0.396	0.016	0	T/NK Cells	ITM2A
ACAP1	0	1.527947	0.432	0.038	0	T/NK Cells	ACAP1
SEPTIN1	0	1.519266	0.445	0.044	0	T/NK Cells	SEPTIN1
TRBC1	0	1.516704	0.327	0.009	0	T/NK Cells	TRBC1
CD6	0	1.516213	0.376	0.014	0	T/NK Cells	CD6
ZFP36L2	0	1.507613	0.925	0.836	0	T/NK Cells	ZFP36L2
LIME1	0	1.505729	0.393	0.018	0	T/NK Cells	LIME1
RPS26	0	1.487321	0.897	0.813	0	T/NK Cells	RPS26
RNF125	0	1.48615	0.436	0.109	0	T/NK Cells	RNF125
CNN2	0	1.469995	0.435	0.054	0	T/NK Cells	CNN2
PTPN22	0	1.467939	0.363	0.047	0	T/NK Cells	PTPN22
ITGA4	0	1.458271	0.427	0.091	0	T/NK Cells	ITGA4
CDC42SE2	0	1.4402	0.534	0.239	0	T/NK Cells	CDC42SE2
MYL12A	0	1.439888	0.761	0.54	0	T/NK Cells	MYL12A

RPS29	0	1.438483	0.807	0.645	0	T/NK Cells	RPS29
ZAP70	0	1.438016	0.376	0.011	0	T/NK Cells	ZAP70
IQGAP2	0	1.430361	0.459	0.134	0	T/NK Cells	IQGAP2
FNBP1	0	1.430361	0.55	0.245	0	T/NK Cells	FNBP1
CD48	0	1.429705	0.453	0.071	0	T/NK Cells	CD48
HLA-A	0	1.420082	0.929	0.836	0	T/NK Cells	HLA-A
RNF19A	0	1.419081	0.425	0.162	0	T/NK Cells	RNF19A
S100A10	0	1.418122	0.553	0.121	0	T/NK Cells	S100A10
PDE7A	0	1.409638	0.429	0.126	0	T/NK Cells	PDE7A
PIP4K2A	0	1.403051	0.537	0.258	0	T/NK Cells	PIP4K2A
RPS15A	0	1.400324	0.927	0.887	0	T/NK Cells	RPS15A
TBC1D10C	0	1.398756	0.411	0.045	0	T/NK Cells	TBC1D10C
RESF1	0	1.393729	0.514	0.223	0	T/NK Cells	RESF1
RPL41	0	1.390538	0.894	0.813	0	T/NK Cells	RPL41
SPON2	0	1.389659	0.265	0.039	0	T/NK Cells	SPON2
STK17A	0	1.388718	0.622	0.308	0	T/NK Cells	STK17A
CCND3	0	1.364436	0.64	0.343	0	T/NK Cells	CCND3
AKNA	0	1.354631	0.498	0.204	0	T/NK Cells	AKNA
CRYBG1	0	1.352864	0.372	0.095	0	T/NK Cells	CRYBG1
ICOS	0	1.346219	0.266	0.008	0	T/NK Cells	ICOS
ABLIM1	0	1.334155	0.306	0.015	0	T/NK Cells	ABLIM1
INPP4B	0	1.33276	0.301	0.042	0	T/NK Cells	INPP4B
PYHIN1	0	1.330656	0.282	0.009	0	T/NK Cells	PYHIN1
CD27	0	1.329346	0.321	0.009	0	T/NK Cells	CD27
IKZF3	0	1.317956	0.311	0.015	0	T/NK Cells	IKZF3
SLC9A3R1	0	1.309186	0.4	0.064	0	T/NK Cells	SLC9A3R1
ERN1	0	1.30888	0.434	0.176	0	T/NK Cells	ERN1
CD69	0	1.308449	0.752	0.494	0	T/NK Cells	CD69
PDE4D	0	1.305999	0.347	0.088	0	T/NK Cells	PDE4D
HOPX	0	1.304017	0.289	0.016	0	T/NK Cells	HOPX
LAT	0	1.303393	0.359	0.015	0	T/NK Cells	LAT
RPL30	0	1.303328	0.928	0.894	0	T/NK Cells	RPL30
CCDC88C	0	1.303322	0.318	0.017	0	T/NK Cells	CCDC88C
CDC14A	0	1.29962	0.281	0.081	0	T/NK Cells	CDC14A
SOCS1	0	1.297406	0.428	0.136	0	T/NK Cells	SOCS1
SAMD3	0	1.287485	0.295	0.009	0	T/NK Cells	SAMD3
CALM1	0	1.287167	0.819	0.64	0	T/NK Cells	CALM1
GIMAP7	0	1.286033	0.384	0.096	0	T/NK Cells	GIMAP7
TSPYL2	0	1.285424	0.398	0.15	0	T/NK Cells	TSPYL2
CDK17	0	1.283015	0.404	0.143	0	T/NK Cells	CDK17
ANXA6	0	1.27779	0.368	0.032	0	T/NK Cells	ANXA6
ARAP2	0	1.277764	0.35	0.088	0	T/NK Cells	ARAP2
RIPOR2	0	1.27601	0.335	0.061	0	T/NK Cells	RIPOR2
PTPRC	0	1.263965	0.874	0.701	0	T/NK Cells	PTPRC
FCMR	0	1.262842	0.359	0.033	0	T/NK Cells	FCMR
RPS3	0	1.262179	0.919	0.879	0	T/NK Cells	RPS3
SCML4	0	1.25991	0.395	0.106	0	T/NK Cells	SCML4

TSC22D3	0	1.25927	0.854	0.707	0	T/NK Cells	TSC22D3
CYFIP2	0	1.255805	0.331	0.021	0	T/NK Cells	CYFIP2
SH3BGRL3	0	1.252262	0.811	0.664	0	T/NK Cells	SH3BGRL3
ICAM3	0	1.248899	0.392	0.049	0	T/NK Cells	ICAM3
IRF1	0	1.242249	0.439	0.169	0	T/NK Cells	IRF1
FLNA	0	1.2388	0.363	0.068	0	T/NK Cells	FLNA
FAM177A1	0	1.234678	0.42	0.178	0	T/NK Cells	FAM177A1
ATP8A1	0	1.22971	0.281	0.053	0	T/NK Cells	ATP8A1
ZNF831	0	1.227143	0.266	0.008	0	T/NK Cells	ZNF831
S100A6	0	1.21321	0.62	0.194	0	T/NK Cells	S100A6
FOXO1	0	1.208209	0.377	0.143	0	T/NK Cells	FOXO1
SH2D2A	0	1.206939	0.298	0.015	0	T/NK Cells	SH2D2A
PDCD4	0	1.203029	0.54	0.275	0	T/NK Cells	PDCD4
LBH	0	1.195115	0.381	0.074	0	T/NK Cells	LBH
TNFAIP3	0	1.193294	0.78	0.542	0	T/NK Cells	TNFAIP3
RPL28	0	1.192663	0.941	0.92	0	T/NK Cells	RPL28
RBM38	0	1.1906	0.396	0.118	0	T/NK Cells	RBM38
RASA2	0	1.188325	0.413	0.166	0	T/NK Cells	RASA2
LY6E	0	1.184185	0.556	0.214	0	T/NK Cells	LY6E
MDFIC	0	1.181825	0.278	0.027	0	T/NK Cells	MDFIC
PRKCQ	0	1.173622	0.281	0.032	0	T/NK Cells	PRKCQ
LINC01619	0	1.171744	0.365	0.166	0	T/NK Cells	LINC01619
RPS21	0	1.1664	0.884	0.799	0	T/NK Cells	RPS21
EVL	0	1.155543	0.703	0.496	0	T/NK Cells	EVL
SARAF	0	1.152686	0.857	0.737	0	T/NK Cells	SARAF
RPS28	0	1.149045	0.917	0.876	0	T/NK Cells	RPS28
TTC39C	0	1.137782	0.295	0.059	0	T/NK Cells	TTC39C
LYAR	0	1.133212	0.327	0.087	0	T/NK Cells	LYAR
NIBAN1	0	1.13084	0.352	0.172	0	T/NK Cells	NIBAN1
MYH9	0	1.130183	0.682	0.472	0	T/NK Cells	MYH9
CLEC2D	0	1.124554	0.327	0.078	0	T/NK Cells	CLEC2D
SYNE1	0	1.12214	0.303	0.073	0	T/NK Cells	SYNE1
RPS12	0	1.116131	0.934	0.918	0	T/NK Cells	RPS12
AHNAK	0	1.109603	0.478	0.193	0	T/NK Cells	AHNAK
GBP5	0	1.106659	0.272	0.03	0	T/NK Cells	GBP5
ARHGDIB	0	1.101967	0.836	0.695	0	T/NK Cells	ARHGDIB
RPS27A	0	1.100482	0.923	0.883	0	T/NK Cells	RPS27A
CYTOR	0	1.100474	0.301	0.073	0	T/NK Cells	CYTOR
RPL35A	0	1.098967	0.885	0.818	0	T/NK Cells	RPL35A
KLF12	0	1.096582	0.267	0.054	0	T/NK Cells	KLF12
IL2RB	0	1.093605	0.254	0.007	0	T/NK Cells	IL2RB
ZC3HAV1	0	1.087587	0.527	0.273	0	T/NK Cells	ZC3HAV1
EMB	0	1.086007	0.403	0.168	0	T/NK Cells	EMB
ARHGEF1	0	1.082161	0.526	0.299	0	T/NK Cells	ARHGEF1
CD5	0	1.080583	0.265	0.01	0	T/NK Cells	CD5
LSP1	0	1.078596	0.619	0.306	0	T/NK Cells	LSP1
SIK3	0	1.077134	0.566	0.423	0	T/NK Cells	SIK3

RBL2	0	1.072344	0.384	0.138	0	T/NK Cells	RBL2
SLFN5	0	1.06708	0.335	0.092	0	T/NK Cells	SLFN5
HCST	0	1.063948	0.709	0.53	0	T/NK Cells	HCST
EEF1D	0	1.059121	0.815	0.688	0	T/NK Cells	EEF1D
RPL23A	0	1.053578	0.863	0.776	0	T/NK Cells	RPL23A
EML4	0	1.046741	0.576	0.403	0	T/NK Cells	EML4
SUN2	0	1.045123	0.418	0.17	0	T/NK Cells	SUN2
OCIAD2	0	1.044651	0.293	0.021	0	T/NK Cells	OCIAD2
ANXA1	0	1.042953	0.613	0.346	0	T/NK Cells	ANXA1
PCNX1	0	1.042907	0.358	0.171	0	T/NK Cells	PCNX1
ARHGEF3	0	1.041231	0.286	0.07	0	T/NK Cells	ARHGEF3
STK17B	0	1.038348	0.634	0.394	0	T/NK Cells	STK17B
CDKN1B	0	1.036053	0.353	0.12	0	T/NK Cells	CDKN1B
HLA-B	0	1.035602	0.957	0.933	0	T/NK Cells	HLA-B
DENND2D	0	1.03271	0.282	0.036	0	T/NK Cells	DENND2D
HLA-F	0	1.023261	0.489	0.262	0	T/NK Cells	HLA-F
HLA-C	0	1.017063	0.913	0.822	0	T/NK Cells	HLA-C
OPTN	0	1.015873	0.267	0.014	0	T/NK Cells	OPTN
IKZF1	0	1.015415	0.637	0.421	0	T/NK Cells	IKZF1
PCED1B-AS	0	1.014438	0.537	0.336	0	T/NK Cells	PCED1B-AS1
RPSA	0	1.01317	0.823	0.718	0	T/NK Cells	RPSA
PSME1	0	1.012943	0.619	0.431	0	T/NK Cells	PSME1
PTPN4	0	1.00993	0.291	0.062	0	T/NK Cells	PTPN4
PIK3IP1	0	1.002569	0.449	0.23	0	T/NK Cells	PIK3IP1
RPS7	0	0.998778	0.904	0.86	0	T/NK Cells	RPS7
CYTH1	0	0.997232	0.473	0.296	0	T/NK Cells	CYTH1
STK4	0	0.986384	0.615	0.439	0	T/NK Cells	STK4
FKBP11	0	0.986063	0.257	0.022	0	T/NK Cells	FKBP11
SYTL3	0	0.981912	0.634	0.413	0	T/NK Cells	SYTL3
PBXIP1	0	0.981776	0.291	0.07	0	T/NK Cells	PBXIP1
RPS25	0	0.980246	0.893	0.845	0	T/NK Cells	RPS25
RPL34	0	0.979131	0.915	0.877	0	T/NK Cells	RPL34
PSMB9	0	0.978046	0.494	0.285	0	T/NK Cells	PSMB9
UBE2S	0	0.977281	0.469	0.28	0	T/NK Cells	UBE2S
RPL39	0	0.976812	0.909	0.88	0	T/NK Cells	RPL39
ABHD17A	0	0.976702	0.403	0.2	0	T/NK Cells	ABHD17A
LCP1	0	0.9746	0.637	0.433	0	T/NK Cells	LCP1
WIPF1	0	0.971783	0.538	0.329	0	T/NK Cells	WIPF1
MATK	0	0.970872	0.268	0.041	0	T/NK Cells	MATK
RPLP2	0	0.97077	0.892	0.838	0	T/NK Cells	RPLP2
HERPUD2	0	0.967056	0.335	0.123	0	T/NK Cells	HERPUD2
CCSER2	0	0.965841	0.34	0.147	0	T/NK Cells	CCSER2
B2M	0	0.961851	0.972	0.973	0	T/NK Cells	B2M
RPS4X	0	0.960869	0.897	0.855	0	T/NK Cells	RPS4X
APOBEC3G	0	0.950947	0.297	0.098	0	T/NK Cells	APOBEC3G
TOB1	0	0.949274	0.295	0.14	0	T/NK Cells	TOB1
TERF2IP	0	0.94861	0.451	0.266	0	T/NK Cells	TERF2IP

TMA7	0	0.946843	0.697	0.547	0	T/NK Cells	TMA7
FAM107B	0	0.944861	0.391	0.166	0	T/NK Cells	FAM107B
ABRACL	0	0.93892	0.377	0.147	0	T/NK Cells	ABRACL
RPL14	0	0.936087	0.896	0.851	0	T/NK Cells	RPL14
RHOH	0	0.932855	0.454	0.253	0	T/NK Cells	RHOH
LRBA	0	0.932449	0.361	0.194	0	T/NK Cells	LRBA
TNFAIP8	0	0.930169	0.341	0.141	0	T/NK Cells	TNFAIP8
LDHB	0	0.926803	0.516	0.34	0	T/NK Cells	LDHB
P2RY8	0	0.920028	0.347	0.174	0	T/NK Cells	P2RY8
RPL32	0	0.919515	0.926	0.909	0	T/NK Cells	RPL32
DDIT4	0	0.916851	0.686	0.522	0	T/NK Cells	DDIT4
NAP1L4	0	0.909908	0.386	0.208	0	T/NK Cells	NAP1L4
RPS14	0	0.907574	0.911	0.886	0	T/NK Cells	RPS14
RPL10	0	0.907538	0.945	0.948	0	T/NK Cells	RPL10
SNRPD2	0	0.905875	0.602	0.427	0	T/NK Cells	SNRPD2
IFITM2	0	0.90246	0.634	0.512	0	T/NK Cells	IFITM2
BUB3	0	0.900368	0.367	0.156	0	T/NK Cells	BUB3
DOK2	0	0.896958	0.25	0.035	0	T/NK Cells	DOK2
LIMD2	0	0.895785	0.495	0.33	0	T/NK Cells	LIMD2
RPL18A	0	0.894164	0.913	0.882	0	T/NK Cells	RPL18A
RALGAPA1	0	0.89365	0.339	0.173	0	T/NK Cells	RALGAPA1
DIAPH1	0	0.89195	0.275	0.074	0	T/NK Cells	DIAPH1
RPL36AL	0	0.89133	0.764	0.649	0	T/NK Cells	RPL36AL
RABGAP1L	0	0.890966	0.514	0.384	0	T/NK Cells	RABGAP1L
TSEN54	0	0.88813	0.268	0.056	0	T/NK Cells	TSEN54
RHOF	0	0.887496	0.263	0.037	0	T/NK Cells	RHOF
YPEL5	0	0.883093	0.522	0.362	0	T/NK Cells	YPEL5
ADD3	0	0.879577	0.259	0.055	0	T/NK Cells	ADD3
EZR	0	0.87605	0.564	0.396	0	T/NK Cells	EZR
ITGAL	0	0.875525	0.263	0.088	0	T/NK Cells	ITGAL
JAK1	0	0.875173	0.595	0.441	0	T/NK Cells	JAK1
TRAF3IP3	0	0.873556	0.344	0.153	0	T/NK Cells	TRAF3IP3
TMC8	0	0.873241	0.293	0.105	0	T/NK Cells	TMC8
VAMP2	0	0.871739	0.5	0.323	0	T/NK Cells	VAMP2
GYPC	0	0.864435	0.523	0.323	0	T/NK Cells	GYPC
UTRN	0	0.864204	0.454	0.303	0	T/NK Cells	UTRN
RPL19	0	0.861939	0.921	0.905	0	T/NK Cells	RPL19
LINC01138	0	0.861039	0.283	0.125	0	T/NK Cells	LINC01138
JUNB	0	0.857394	0.877	0.885	0	T/NK Cells	JUNB
YWHAZ	0	0.855652	0.73	0.592	0	T/NK Cells	YWHAZ
RPL7A	0	0.854414	0.906	0.869	0	T/NK Cells	RPL7A
SEPTIN7	0	0.853673	0.511	0.342	0	T/NK Cells	SEPTIN7
RPS15	0	0.852073	0.918	0.893	0	T/NK Cells	RPS15
CD47	0	0.850001	0.372	0.186	0	T/NK Cells	CD47
TOMM7	0	0.846647	0.68	0.553	0	T/NK Cells	TOMM7
HINT1	0	0.844699	0.611	0.453	0	T/NK Cells	HINT1
RPS18	0	0.84448	0.921	0.907	0	T/NK Cells	RPS18

ARHGAP9	0	0.841517	0.401	0.235	0	T/NK Cells	ARHGAP9
DDX24	0	0.841018	0.619	0.483	0	T/NK Cells	DDX24
RPL11	0	0.839044	0.922	0.901	0	T/NK Cells	RPL11
DNAJC1	0	0.835317	0.339	0.172	0	T/NK Cells	DNAJC1
RBMS1	0	0.834953	0.38	0.248	0	T/NK Cells	RBMS1
TAP1	0	0.833708	0.294	0.111	0	T/NK Cells	TAP1
RPL36	0	0.832662	0.885	0.828	0	T/NK Cells	RPL36
PAXX	0	0.831794	0.467	0.302	0	T/NK Cells	PAXX
GLCCI1	0	0.822965	0.318	0.162	0	T/NK Cells	GLCCI1
AAK1	0	0.822308	0.386	0.246	0	T/NK Cells	AAK1
RPL13	0	0.820963	0.941	0.938	0	T/NK Cells	RPL13
RGCC	0	0.819368	0.5	0.284	0	T/NK Cells	RGCC
RPL21	0	0.818476	0.815	0.739	0	T/NK Cells	RPL21
PTMA	0	0.817398	0.931	0.907	0	T/NK Cells	PTMA
SPTAN1	0	0.815507	0.412	0.249	0	T/NK Cells	SPTAN1
RPL6	0	0.811096	0.892	0.857	0	T/NK Cells	RPL6
PPP1R2	0	0.81045	0.421	0.273	0	T/NK Cells	PPP1R2
RAP1B	0	0.80987	0.549	0.419	0	T/NK Cells	RAP1B
EPB41	0	0.809713	0.329	0.186	0	T/NK Cells	EPB41
ANKRD12	0	0.809488	0.47	0.333	0	T/NK Cells	ANKRD12
PTPN7	0	0.803283	0.272	0.096	0	T/NK Cells	PTPN7
ARID4B	0	0.79859	0.467	0.337	0	T/NK Cells	ARID4B
RPLP1	0	0.798352	0.949	0.95	0	T/NK Cells	RPLP1
SSR2	0	0.792873	0.544	0.395	0	T/NK Cells	SSR2
KLF13	0	0.790078	0.353	0.193	0	T/NK Cells	KLF13
EEF1A1	0	0.788983	0.95	0.965	0	T/NK Cells	EEF1A1
TAGLN2	0	0.780393	0.502	0.326	0	T/NK Cells	TAGLN2
GMFG	0	0.778968	0.57	0.427	0	T/NK Cells	GMFG
RNF213	0	0.778714	0.546	0.442	0	T/NK Cells	RNF213
RPS5	0	0.775827	0.838	0.769	0	T/NK Cells	RPS5
RPS3A	0	0.771283	0.903	0.876	0	T/NK Cells	RPS3A
SUB1	0	0.769641	0.572	0.447	0	T/NK Cells	SUB1
SLC2A3	0	0.768535	0.571	0.386	0	T/NK Cells	SLC2A3
HMOX2	0	0.767775	0.267	0.094	0	T/NK Cells	HMOX2
RFTN1	0	0.767507	0.274	0.126	0	T/NK Cells	RFTN1
PPP3CC	0	0.765751	0.264	0.105	0	T/NK Cells	PPP3CC
CASP8	0	0.765473	0.269	0.124	0	T/NK Cells	CASP8
RPL24	0	0.762841	0.862	0.809	0	T/NK Cells	RPL24
RPL37	0	0.757341	0.899	0.864	0	T/NK Cells	RPL37
FXYD5	0	0.755393	0.682	0.56	0	T/NK Cells	FXYD5
STT3B	0	0.7553	0.325	0.182	0	T/NK Cells	STT3B
H1FX	0	0.754968	0.498	0.394	0	T/NK Cells	H1FX
RPS19	0	0.751521	0.935	0.922	0	T/NK Cells	RPS19
RPL18	0	0.750968	0.899	0.872	0	T/NK Cells	RPL18
STK10	0	0.748295	0.381	0.231	0	T/NK Cells	STK10
TSTD1	0	0.744726	0.274	0.1	0	T/NK Cells	TSTD1
FAU	0	0.743787	0.904	0.877	0	T/NK Cells	FAU

ARF6	0	0.741456	0.458	0.306	0	T/NK Cells	ARF6
NOP53	0	0.738787	0.545	0.425	0	T/NK Cells	NOP53
RPS4Y1	0	0.733522	0.471	0.42	0	T/NK Cells	RPS4Y1
BIN2	0	0.732893	0.391	0.257	0	T/NK Cells	BIN2
SIGIRR	0	0.730587	0.294	0.132	0	T/NK Cells	SIGIRR
PLP2	0	0.730485	0.296	0.133	0	T/NK Cells	PLP2
RPL10A	0	0.730008	0.823	0.762	0	T/NK Cells	RPL10A
GSTK1	0	0.725818	0.504	0.376	0	T/NK Cells	GSTK1
GSPT1	0	0.721013	0.382	0.274	0	T/NK Cells	GSPT1
KMT2E	0	0.717199	0.532	0.422	0	T/NK Cells	KMT2E
CORO1A	0	0.714422	0.712	0.627	0	T/NK Cells	CORO1A
GUK1	0	0.712163	0.538	0.402	0	T/NK Cells	GUK1
CD99	0	0.711474	0.699	0.578	0	T/NK Cells	CD99
SRPK2	0	0.710566	0.304	0.182	0	T/NK Cells	SRPK2
NSMCE3	0	0.708104	0.288	0.133	0	T/NK Cells	NSMCE3
SRSF7	0	0.705975	0.718	0.623	0	T/NK Cells	SRSF7
ARPC5L	0	0.704203	0.291	0.137	0	T/NK Cells	ARPC5L
RSBN1	0	0.703431	0.252	0.112	0	T/NK Cells	RSBN1
RPL38	0	0.700827	0.713	0.636	0	T/NK Cells	RPL38
G3BP2	0	0.697419	0.349	0.223	0	T/NK Cells	G3BP2
PACS1	0	0.697114	0.325	0.227	0	T/NK Cells	PACS1
PFN1	0	0.693844	0.857	0.829	0	T/NK Cells	PFN1
DGKZ	0	0.692113	0.277	0.123	0	T/NK Cells	DGKZ
UBALD2	0	0.690133	0.427	0.28	0	T/NK Cells	UBALD2
DUSP5	0	0.684364	0.298	0.149	0	T/NK Cells	DUSP5
ACTN4	0	0.682299	0.304	0.158	0	T/NK Cells	ACTN4
PTP4A2	0	0.681411	0.44	0.316	0	T/NK Cells	PTP4A2
SHISA5	0	0.67743	0.33	0.181	0	T/NK Cells	SHISA5
CUTA	0	0.674777	0.409	0.274	0	T/NK Cells	CUTA
CORO7	0	0.673459	0.37	0.281	0	T/NK Cells	CORO7
MZT2A	0	0.670102	0.316	0.159	0	T/NK Cells	MZT2A
VPS37B	0	0.668846	0.37	0.277	0	T/NK Cells	VPS37B
OST4	0	0.662496	0.587	0.472	0	T/NK Cells	OST4
HNRNPA1	0	0.662113	0.731	0.667	0	T/NK Cells	HNRNPA1
RPL8	0	0.661731	0.886	0.853	0	T/NK Cells	RPL8
IQGAP1	0	0.66071	0.434	0.301	0	T/NK Cells	IQGAP1
SEMA4D	0	0.65935	0.372	0.269	0	T/NK Cells	SEMA4D
RPL26	0	0.657568	0.908	0.887	0	T/NK Cells	RPL26
CCDC107	0	0.653715	0.281	0.14	0	T/NK Cells	CCDC107
RASAL3	0	0.652663	0.339	0.211	0	T/NK Cells	RASAL3
KMT2A	0	0.652497	0.315	0.203	0	T/NK Cells	KMT2A
MYL12B	0	0.649474	0.637	0.551	0	T/NK Cells	MYL12B
HERC1	0	0.647378	0.328	0.232	0	T/NK Cells	HERC1
SSBP4	0	0.647216	0.318	0.19	0	T/NK Cells	SSBP4
ARHGAP15	0	0.64671	0.611	0.56	0	T/NK Cells	ARHGAP15
RPL3	0	0.644343	0.889	0.868	0	T/NK Cells	RPL3
LDHA	0	0.639114	0.544	0.453	0	T/NK Cells	LDHA

MCUB	0	0.638026	0.305	0.173	0	T/NK Cells	MCUB
RPL13A	0	0.63793	0.795	0.738	0	T/NK Cells	RPL13A
NDUFS5	0	0.637726	0.495	0.387	0	T/NK Cells	NDUFS5
SOD1	0	0.636808	0.459	0.346	0	T/NK Cells	SOD1
CIB1	0	0.636134	0.441	0.326	0	T/NK Cells	CIB1
CLEC2B	0	0.63325	0.415	0.309	0	T/NK Cells	CLEC2B
CASK	0	0.631183	0.27	0.18	0	T/NK Cells	CASK
RSRP1	0	0.629402	0.522	0.423	0	T/NK Cells	RSRP1
RPL22	0	0.629232	0.8	0.747	0	T/NK Cells	RPL22
PRKCB	0	0.625796	0.346	0.245	0	T/NK Cells	PRKCB
SEPTIN6	0	0.624531	0.406	0.299	0	T/NK Cells	SEPTIN6
STAT1	0	0.623883	0.268	0.183	0	T/NK Cells	STAT1
RPL35	0	0.623284	0.847	0.793	0	T/NK Cells	RPL35
RPL31	0	0.622537	0.73	0.66	0	T/NK Cells	RPL31
RPS24	0	0.62157	0.915	0.9	0	T/NK Cells	RPS24
RPL9	0	0.619126	0.825	0.789	0	T/NK Cells	RPL9
C9orf78	0	0.618293	0.33	0.205	0	T/NK Cells	C9orf78
RPS2	0	0.6177	0.877	0.849	0	T/NK Cells	RPS2
PRMT2	0	0.614841	0.379	0.274	0	T/NK Cells	PRMT2
SYNRG	0	0.609184	0.291	0.191	0	T/NK Cells	SYNRG
OGA	0	0.607147	0.294	0.196	0	T/NK Cells	OGA
LITAF	0	0.605612	0.518	0.438	0	T/NK Cells	LITAF
PSMB8	0	0.602919	0.406	0.3	0	T/NK Cells	PSMB8
RPS23	0	0.602081	0.916	0.909	0	T/NK Cells	RPS23
NSD3	0	0.600298	0.353	0.251	0	T/NK Cells	NSD3
ACTG1	0	0.600186	0.82	0.79	0	T/NK Cells	ACTG1
BRD2	0	0.597375	0.54	0.464	0	T/NK Cells	BRD2
SP140	0	0.597324	0.283	0.18	0	T/NK Cells	SP140
COX7C	0	0.595927	0.666	0.592	0	T/NK Cells	COX7C
OGT	0	0.594048	0.324	0.233	0	T/NK Cells	OGT
HLA-E	0	0.593465	0.875	0.825	0	T/NK Cells	HLA-E
APRT	0	0.591109	0.48	0.366	0	T/NK Cells	APRT
AUTS2	0	0.585041	0.421	0.315	0	T/NK Cells	AUTS2
RPL17	0	0.583814	0.866	0.834	0	T/NK Cells	RPL17
RPL29	0	0.581152	0.897	0.878	0	T/NK Cells	RPL29
RPL12	0	0.579213	0.91	0.891	0	T/NK Cells	RPL12
ZBTB38	0	0.57861	0.252	0.16	0	T/NK Cells	ZBTB38
EMP3	0	0.57364	0.442	0.301	0	T/NK Cells	EMP3
NOP58	0	0.572338	0.326	0.232	0	T/NK Cells	NOP58
SRSF2	0	0.572276	0.569	0.526	0	T/NK Cells	SRSF2
MPHOSPH8	0	0.570884	0.33	0.243	0	T/NK Cells	MPHOSPH8
ANAPC16	0	0.570273	0.478	0.385	0	T/NK Cells	ANAPC16
TNFRSF14	0	0.569299	0.265	0.148	0	T/NK Cells	TNFRSF14
TRIR	0	0.567627	0.534	0.456	0	T/NK Cells	TRIR
TAPBP	0	0.56615	0.384	0.288	0	T/NK Cells	TAPBP
RPS13	0	0.565619	0.893	0.885	0	T/NK Cells	RPS13
SAP18	0	0.56482	0.496	0.426	0	T/NK Cells	SAP18

CASP4	0	0.563653	0.256	0.137	0	T/NK Cells	CASP4
MZT2B	0	0.562284	0.516	0.403	0	T/NK Cells	MZT2B
NFATC2	0	0.562102	0.372	0.293	0	T/NK Cells	NFATC2
MAZ	0	0.559931	0.25	0.127	0	T/NK Cells	MAZ
SP100	0	0.552839	0.565	0.51	0	T/NK Cells	SP100
TRAPPC10	0	0.551405	0.273	0.185	0	T/NK Cells	TRAPPC10
TMX4	0	0.548801	0.259	0.13	0	T/NK Cells	TMX4
ISG15	0	0.546915	0.281	0.185	0	T/NK Cells	ISG15
JUND	0	0.54301	0.812	0.788	0	T/NK Cells	JUND
NCOR1	0	0.540403	0.403	0.331	0	T/NK Cells	NCOR1
KIF2A	0	0.54019	0.275	0.18	0	T/NK Cells	KIF2A
AGO2	0	0.539587	0.257	0.17	0	T/NK Cells	AGO2
HNRNPL	0	0.536319	0.503	0.428	0	T/NK Cells	HNRNPL
CNBP	0	0.534746	0.594	0.516	0	T/NK Cells	CNBP
ATP5F1E	0	0.53376	0.793	0.746	0	T/NK Cells	ATP5F1E
SSR4	0	0.533537	0.576	0.49	0	T/NK Cells	SSR4
IDS	0	0.532371	0.479	0.415	0	T/NK Cells	IDS
VIM	0	0.531464	0.711	0.429	0	T/NK Cells	VIM
PRKACB	0	0.530091	0.256	0.164	0	T/NK Cells	PRKACB
RPL27	0	0.529345	0.756	0.704	0	T/NK Cells	RPL27
DDX6	0	0.528771	0.367	0.282	0	T/NK Cells	DDX6
LNPEP	0	0.528531	0.27	0.186	0	T/NK Cells	LNPEP
ANXA2	0	0.527365	0.257	0.11	0	T/NK Cells	ANXA2
CALM3	0	0.525624	0.33	0.216	0	T/NK Cells	CALM3
MIF	0	0.525214	0.649	0.539	0	T/NK Cells	MIF
RPL27A	0	0.524554	0.635	0.574	0	T/NK Cells	RPL27A
TXN	0	0.522637	0.309	0.189	0	T/NK Cells	TXN
CHIC2	0	0.521082	0.258	0.165	0	T/NK Cells	CHIC2
ECH1	0	0.520521	0.315	0.214	0	T/NK Cells	ECH1
RPL36A	0	0.519195	0.785	0.753	0	T/NK Cells	RPL36A
FOSL2	0	0.518129	0.313	0.212	0	T/NK Cells	FOSL2
PFDN5	0	0.511571	0.772	0.717	0	T/NK Cells	PFDN5
RPL5	0	0.510323	0.849	0.835	0	T/NK Cells	RPL5
RBM8A	0	0.506998	0.448	0.375	0	T/NK Cells	RBM8A
DEF6	0	0.506713	0.386	0.301	0	T/NK Cells	DEF6
GPSM3	0	0.505799	0.49	0.423	0	T/NK Cells	GPSM3
LINC01578	0	0.505738	0.545	0.491	0	T/NK Cells	LINC01578
CBX4	0	0.503725	0.253	0.152	0	T/NK Cells	CBX4
GIMAP1	0	0.503723	0.273	0.175	0	T/NK Cells	GIMAP1
SNHG7	0	0.50292	0.307	0.214	0	T/NK Cells	SNHG7
C9orf16	0	0.500371	0.42	0.338	0	T/NK Cells	C9orf16
CCNI	0	0.500177	0.631	0.581	0	T/NK Cells	CCNI
COX7A2	0	0.499723	0.459	0.374	0	T/NK Cells	COX7A2
RPL37A	0	0.498926	0.806	0.771	0	T/NK Cells	RPL37A
UQCRB	0	0.496725	0.607	0.547	0	T/NK Cells	UQCRB
MED10	0	0.495995	0.254	0.153	0	T/NK Cells	MED10
IL16	0	0.490831	0.296	0.206	0	T/NK Cells	IL16

EIF3G	0	0.489989	0.522	0.464	0	T/NK Cells	EIF3G
TMC6	0	0.48998	0.255	0.161	0	T/NK Cells	TMC6
UBA52	0	0.489302	0.807	0.772	0	T/NK Cells	UBA52
PRKCH	0	0.488465	0.661	0.585	0	T/NK Cells	PRKCH
MBNL1	0	0.486854	0.71	0.67	0	T/NK Cells	MBNL1
RACK1	0	0.486551	0.847	0.819	0	T/NK Cells	RACK1
SEC61G	0	0.485732	0.362	0.273	0	T/NK Cells	SEC61G
ATP5MG	0	0.485113	0.665	0.608	0	T/NK Cells	ATP5MG
SNU13	0	0.484527	0.415	0.338	0	T/NK Cells	SNU13
RPS8	0	0.483563	0.918	0.917	0	T/NK Cells	RPS8
SKP1	0	0.476857	0.597	0.542	0	T/NK Cells	SKP1
ATP5IF1	0	0.475957	0.422	0.349	0	T/NK Cells	ATP5IF1
POLR2L	0	0.474957	0.376	0.291	0	T/NK Cells	POLR2L
PTGER4	0	0.474865	0.462	0.369	0	T/NK Cells	PTGER4
CCDC85B	0	0.472636	0.35	0.267	0	T/NK Cells	CCDC85B
COMMD6	0	0.468414	0.61	0.545	0	T/NK Cells	COMMD6
FGFR1OP2	0	0.467717	0.281	0.204	0	T/NK Cells	FGFR1OP2
YWHAB	0	0.464798	0.603	0.574	0	T/NK Cells	YWHAB
TOMM5	0	0.460044	0.277	0.192	0	T/NK Cells	TOMM5
PSME2	0	0.458785	0.424	0.352	0	T/NK Cells	PSME2
CARD16	0	0.458064	0.256	0.175	0	T/NK Cells	CARD16
RPS16	0	0.455789	0.841	0.825	0	T/NK Cells	RPS16
POLR3GL	0	0.455085	0.261	0.171	0	T/NK Cells	POLR3GL
BTF3	0	0.454631	0.735	0.699	0	T/NK Cells	BTF3
H2AFX	0	0.45106	0.303	0.216	0	T/NK Cells	H2AFX
RPL7	0	0.447348	0.65	0.609	0	T/NK Cells	RPL7
RPS20	0	0.445488	0.528	0.488	0	T/NK Cells	RPS20
TAP2	0	0.444958	0.258	0.182	0	T/NK Cells	TAP2
COX6C	0	0.441231	0.522	0.458	0	T/NK Cells	COX6C
ARPC2	0	0.440255	0.661	0.631	0	T/NK Cells	ARPC2
UBB	0	0.438775	0.692	0.67	0	T/NK Cells	UBB
SF3A2	0	0.43421	0.276	0.194	0	T/NK Cells	SF3A2
RPS6	0	0.432049	0.887	0.882	0	T/NK Cells	RPS6
ELOB	0	0.431329	0.51	0.441	0	T/NK Cells	ELOB
NPM1	0	0.425196	0.723	0.704	0	T/NK Cells	NPM1
RPLP0	0	0.419575	0.873	0.871	0	T/NK Cells	RPLP0
CHCHD2	0	0.406918	0.616	0.571	0	T/NK Cells	CHCHD2
NACA	0	0.397877	0.849	0.83	0	T/NK Cells	NACA
EIF1	0	0.38448	0.915	0.92	0	T/NK Cells	EIF1
HMGB1	0	0.379302	0.771	0.756	0	T/NK Cells	HMGB1
EIF3K	0	0.37554	0.613	0.572	0	T/NK Cells	EIF3K
EEF1B2	0	0.352232	0.773	0.756	0	T/NK Cells	EEF1B2
MALAT1	0	0.34983	0.999	0.986	0	T/NK Cells	MALAT1
SRP14	0	0.3465	0.633	0.604	0	T/NK Cells	SRP14
TMSB10	0	0.33165	0.923	0.924	0	T/NK Cells	TMSB10
RPS9	0	0.319254	0.856	0.858	0	T/NK Cells	RPS9
DNAJB1	0	0.311426	0.746	0.632	0	T/NK Cells	DNAJB1

MYL6	0	0.300279	0.773	0.767	0	T/NK Cells	MYL6
COX4I1	0	0.286574	0.744	0.733	0	T/NK Cells	COX4I1
HSPA8	0	0.276404	0.793	0.766	0	T/NK Cells	HSPA8
FOXP1	9.65E-307	0.593747	0.52	0.463	2.71E-302	T/NK Cells	FOXP1
ARHGAP30	2.86E-303	0.440205	0.263	0.187	8.05E-299	T/NK Cells	ARHGAP30
EEF2	1.38E-299	0.283259	0.753	0.76	3.87E-295	T/NK Cells	EEF2
RNF115	6.76E-293	0.539317	0.256	0.182	1.90E-288	T/NK Cells	RNF115
LYST	3.84E-292	0.646316	0.299	0.225	1.08E-287	T/NK Cells	LYST
ATP6V1G1	6.25E-292	0.424164	0.466	0.412	1.76E-287	T/NK Cells	ATP6V1G1
RSL24D1	1.21E-290	0.436716	0.317	0.244	3.41E-286	T/NK Cells	RSL24D1
TLE4	1.84E-289	0.475011	0.28	0.201	5.17E-285	T/NK Cells	TLE4
POLR1D	9.48E-289	0.4469	0.326	0.254	2.67E-284	T/NK Cells	POLR1D
ZBTB1	4.37E-286	0.486886	0.302	0.227	1.23E-281	T/NK Cells	ZBTB1
UBE2D2	6.72E-283	0.415524	0.505	0.461	1.89E-278	T/NK Cells	UBE2D2
TNRC6B	2.16E-282	0.632282	0.367	0.298	6.07E-278	T/NK Cells	TNRC6B
GPI	1.71E-279	0.425032	0.317	0.245	4.81E-275	T/NK Cells	GPI
EIF3F	6.59E-276	0.374242	0.535	0.499	1.86E-271	T/NK Cells	EIF3F
MAT2B	3.00E-273	0.411666	0.268	0.196	8.44E-269	T/NK Cells	MAT2B
INPP4A	7.81E-270	0.520514	0.254	0.181	2.20E-265	T/NK Cells	INPP4A
TMF1	5.62E-269	0.469832	0.26	0.189	1.58E-264	T/NK Cells	TMF1
CNOT2	3.42E-267	0.561591	0.342	0.277	9.62E-263	T/NK Cells	CNOT2
MAP2K2	5.12E-267	0.432786	0.308	0.24	1.44E-262	T/NK Cells	MAP2K2
AIP	9.59E-265	0.399839	0.253	0.182	2.70E-260	T/NK Cells	AIP
PGK1	1.03E-264	0.41297	0.543	0.508	2.90E-260	T/NK Cells	PGK1
EDF1	2.29E-264	0.385029	0.518	0.477	6.46E-260	T/NK Cells	EDF1
H2AFV	5.36E-264	0.380647	0.371	0.304	1.51E-259	T/NK Cells	H2AFV
GAS5	1.20E-263	0.452765	0.467	0.421	3.37E-259	T/NK Cells	GAS5
USP36	5.40E-262	0.531949	0.35	0.279	1.52E-257	T/NK Cells	USP36
FUS	8.30E-262	0.390278	0.625	0.605	2.33E-257	T/NK Cells	FUS
UQCR11	7.41E-261	0.367768	0.535	0.487	2.08E-256	T/NK Cells	UQCR11
GLG1	2.77E-255	0.487274	0.305	0.237	7.79E-251	T/NK Cells	GLG1
MAPK1	1.56E-250	0.531197	0.319	0.254	4.39E-246	T/NK Cells	MAPK1
SMARCA2	7.22E-246	0.492621	0.372	0.309	2.03E-241	T/NK Cells	SMARCA2
KRT10	1.16E-244	0.407588	0.358	0.295	3.26E-240	T/NK Cells	KRT10
RAD21	5.91E-244	0.387678	0.308	0.238	1.66E-239	T/NK Cells	RAD21
RSBN1L	5.34E-243	0.443401	0.259	0.191	1.50E-238	T/NK Cells	RSBN1L
PAIP2	3.43E-240	0.42995	0.369	0.31	9.64E-236	T/NK Cells	PAIP2
SNHG29	6.52E-239	0.380783	0.564	0.543	1.83E-234	T/NK Cells	SNHG29
UXT	2.65E-238	0.406283	0.381	0.325	7.46E-234	T/NK Cells	UXT
RAB8B	9.87E-238	0.476304	0.27	0.203	2.78E-233	T/NK Cells	RAB8B
CTBP1	6.22E-235	0.405739	0.278	0.211	1.75E-230	T/NK Cells	CTBP1
ATXN1	1.52E-234	0.742224	0.395	0.345	4.28E-230	T/NK Cells	ATXN1
UBL5	2.56E-229	0.375487	0.501	0.463	7.22E-225	T/NK Cells	UBL5
CHURC1	5.97E-228	0.427174	0.343	0.285	1.68E-223	T/NK Cells	CHURC1
SPSB3	2.57E-224	0.424371	0.288	0.225	7.22E-220	T/NK Cells	SPSB3
C19orf53	1.38E-221	0.393452	0.373	0.316	3.87E-217	T/NK Cells	C19orf53
ANXA11	2.94E-220	0.403853	0.368	0.314	8.28E-216	T/NK Cells	ANXA11

PPP4R3A	3.88E-220	0.423061	0.264	0.199	1.09E-215	T/NK Cells	PPP4R3A
BPTF	3.25E-219	0.504432	0.388	0.336	9.14E-215	T/NK Cells	BPTF
NCL	3.19E-218	0.312842	0.611	0.596	8.98E-214	T/NK Cells	NCL
SNHG6	1.46E-217	0.420355	0.478	0.442	4.10E-213	T/NK Cells	SNHG6
ZNF292	3.48E-217	0.481934	0.261	0.197	9.79E-213	T/NK Cells	ZNF292
GTF3A	6.36E-215	0.405513	0.349	0.29	1.79E-210	T/NK Cells	GTF3A
ATF7IP	1.48E-214	0.50034	0.358	0.298	4.15E-210	T/NK Cells	ATF7IP
NSA2	4.98E-211	0.379619	0.366	0.308	1.40E-206	T/NK Cells	NSA2
ASXL1	6.84E-211	0.458988	0.271	0.209	1.92E-206	T/NK Cells	ASXL1
PABPC1	8.67E-210	0.281906	0.805	0.835	2.44E-205	T/NK Cells	PABPC1
PHC3	3.21E-204	0.455715	0.255	0.194	9.04E-200	T/NK Cells	PHC3
CUL3	4.49E-201	0.517623	0.325	0.269	1.26E-196	T/NK Cells	CUL3
SETD2	1.72E-200	0.478104	0.304	0.245	4.83E-196	T/NK Cells	SETD2
SF1	3.29E-198	0.368467	0.557	0.538	9.25E-194	T/NK Cells	SF1
CARD11	8.28E-198	0.544977	0.251	0.191	2.33E-193	T/NK Cells	CARD11
TUBB4B	1.18E-197	0.4221	0.476	0.431	3.32E-193	T/NK Cells	TUBB4B
ST3GAL1	2.26E-196	0.450826	0.284	0.224	6.35E-192	T/NK Cells	ST3GAL1
WNK1	3.91E-195	0.552987	0.347	0.296	1.10E-190	T/NK Cells	WNK1
ESYT2	7.20E-195	0.403664	0.257	0.193	2.03E-190	T/NK Cells	ESYT2
UBE2L6	7.01E-194	0.394728	0.311	0.255	1.97E-189	T/NK Cells	UBE2L6
TUBA1A	1.89E-192	0.39245	0.365	0.306	5.33E-188	T/NK Cells	TUBA1A
PRRC2C	2.07E-191	0.404252	0.536	0.519	5.81E-187	T/NK Cells	PRRC2C
NOSIP	4.51E-191	0.494587	0.296	0.245	1.27E-186	T/NK Cells	NOSIP
PPP1CB	2.20E-190	0.50992	0.304	0.248	6.18E-186	T/NK Cells	PPP1CB
KANSL1	8.78E-187	0.542606	0.315	0.259	2.47E-182	T/NK Cells	KANSL1
KAT6A	1.30E-186	0.474614	0.263	0.204	3.66E-182	T/NK Cells	KAT6A
DIP2A	9.82E-182	0.484341	0.283	0.225	2.76E-177	T/NK Cells	DIP2A
DAZAP2	4.40E-181	0.312188	0.581	0.572	1.24E-176	T/NK Cells	DAZAP2
HNRNPUL1	8.09E-180	0.391714	0.412	0.37	2.28E-175	T/NK Cells	HNRNPUL1
RAB5IF	1.04E-179	0.392235	0.274	0.22	2.93E-175	T/NK Cells	RAB5IF
LSM14A	3.75E-178	0.420794	0.3	0.247	1.06E-173	T/NK Cells	LSM14A
CD55	1.15E-177	0.38785	0.408	0.352	3.24E-173	T/NK Cells	CD55
OSBPL8	6.47E-177	0.521418	0.393	0.35	1.82E-172	T/NK Cells	OSBPL8
EIF4A2	1.78E-176	0.365028	0.517	0.495	5.00E-172	T/NK Cells	EIF4A2
PKN1	5.55E-175	0.375018	0.282	0.229	1.56E-170	T/NK Cells	PKN1
UCP2	4.36E-173	0.442148	0.393	0.354	1.23E-168	T/NK Cells	UCP2
CACYBP	1.59E-172	0.392871	0.281	0.224	4.48E-168	T/NK Cells	CACYBP
PSMA5	2.22E-172	0.369951	0.262	0.21	6.25E-168	T/NK Cells	PSMA5
RNF167	4.83E-172	0.380062	0.271	0.22	1.36E-167	T/NK Cells	RNF167
SF3A1	9.67E-172	0.350964	0.278	0.224	2.72E-167	T/NK Cells	SF3A1
IK	1.23E-165	0.371365	0.273	0.219	3.46E-161	T/NK Cells	IK
RASA3	9.57E-165	0.529278	0.411	0.369	2.69E-160	T/NK Cells	RASA3
CLK1	3.63E-164	0.377219	0.419	0.379	1.02E-159	T/NK Cells	CLK1
MSN	5.13E-163	0.332467	0.551	0.534	1.44E-158	T/NK Cells	MSN
XRN1	2.33E-162	0.44131	0.26	0.208	6.56E-158	T/NK Cells	XRN1
SURF4	1.21E-161	0.374204	0.25	0.199	3.40E-157	T/NK Cells	SURF4
SYF2	1.36E-160	0.355908	0.285	0.234	3.83E-156	T/NK Cells	SYF2

NKTR	4.84E-155	0.49744	0.287	0.238	1.36E-150	T/NK Cells	NKTR
EPS15	2.46E-150	0.475952	0.308	0.261	6.93E-146	T/NK Cells	EPS15
BHLHE40	3.60E-150	0.434217	0.329	0.278	1.01E-145	T/NK Cells	BHLHE40
GCC2	5.47E-150	0.449501	0.282	0.234	1.54E-145	T/NK Cells	GCC2
SUMO2	1.46E-149	0.266617	0.565	0.554	4.11E-145	T/NK Cells	SUMO2
RBM39	5.91E-149	0.312563	0.65	0.658	1.66E-144	T/NK Cells	RBM39
TRIM22	4.29E-148	0.468134	0.407	0.377	1.21E-143	T/NK Cells	TRIM22
COX7A2L	1.30E-146	0.353055	0.312	0.268	3.67E-142	T/NK Cells	COX7A2L
SUMO1	3.30E-145	0.320701	0.273	0.226	9.29E-141	T/NK Cells	SUMO1
C4orf3	1.92E-144	0.327843	0.356	0.315	5.41E-140	T/NK Cells	C4orf3
KDM2A	4.15E-144	0.41745	0.306	0.258	1.17E-139	T/NK Cells	KDM2A
HECA	9.53E-144	0.369574	0.281	0.229	2.68E-139	T/NK Cells	HECA
METTL26	5.65E-142	0.335823	0.255	0.207	1.59E-137	T/NK Cells	METTL26
STUB1	5.85E-141	0.334431	0.261	0.214	1.65E-136	T/NK Cells	STUB1
RPS17	8.11E-140	0.31293	0.481	0.462	2.28E-135	T/NK Cells	RPS17
SET	6.86E-137	0.302928	0.43	0.4	1.93E-132	T/NK Cells	SET
CIRBP	2.16E-135	0.268855	0.637	0.647	6.07E-131	T/NK Cells	CIRBP
GPATCH8	2.32E-135	0.4784	0.267	0.222	6.53E-131	T/NK Cells	GPATCH8
KDM4C	3.57E-135	0.431329	0.255	0.208	1.01E-130	T/NK Cells	KDM4C
SRP9	1.09E-134	0.321453	0.305	0.262	3.06E-130	T/NK Cells	SRP9
CYCS	1.50E-134	0.357475	0.402	0.369	4.22E-130	T/NK Cells	CYCS
RWDD1	1.90E-134	0.356263	0.312	0.269	5.34E-130	T/NK Cells	RWDD1
PPP1R12A	3.10E-131	0.415483	0.36	0.327	8.73E-127	T/NK Cells	PPP1R12A
YWHAQ	4.16E-131	0.330737	0.388	0.356	1.17E-126	T/NK Cells	YWHAQ
IDH2	1.48E-130	0.302763	0.267	0.221	4.17E-126	T/NK Cells	IDH2
CMPK1	4.96E-130	0.323564	0.252	0.207	1.40E-125	T/NK Cells	CMPK1
RABAC1	9.97E-128	0.356753	0.383	0.35	2.81E-123	T/NK Cells	RABAC1
ANKHD1	1.35E-125	0.39972	0.342	0.304	3.79E-121	T/NK Cells	ANKHD1
SF3B5	1.69E-125	0.323791	0.366	0.333	4.75E-121	T/NK Cells	SF3B5
DHX36	8.42E-125	0.408749	0.299	0.26	2.37E-120	T/NK Cells	DHX36
NDUFA12	1.24E-124	0.293094	0.255	0.21	3.48E-120	T/NK Cells	NDUFA12
TAF7	1.92E-123	0.33483	0.262	0.218	5.41E-119	T/NK Cells	TAF7
ADAR	1.27E-120	0.384308	0.329	0.292	3.57E-116	T/NK Cells	ADAR
PPP1R18	4.00E-120	0.351331	0.324	0.289	1.13E-115	T/NK Cells	PPP1R18
BAZ1A	5.82E-120	0.412086	0.315	0.276	1.64E-115	T/NK Cells	BAZ1A
PPP1CA	4.18E-118	0.33438	0.399	0.379	1.18E-113	T/NK Cells	PPP1CA
PARK7	1.96E-116	0.317028	0.416	0.391	5.52E-112	T/NK Cells	PARK7
JTB	2.98E-115	0.317652	0.343	0.309	8.37E-111	T/NK Cells	JTB
GIMAP4	2.04E-114	0.34172	0.348	0.31	5.75E-110	T/NK Cells	GIMAP4
SNRPB	1.17E-113	0.311108	0.365	0.336	3.29E-109	T/NK Cells	SNRPB
PHF3	2.91E-113	0.372635	0.291	0.251	8.19E-109	T/NK Cells	PHF3
NCOA2	7.89E-113	0.469185	0.26	0.218	2.22E-108	T/NK Cells	NCOA2
PSMA7	1.03E-111	0.283486	0.487	0.477	2.89E-107	T/NK Cells	PSMA7
CYLD	1.94E-109	0.348547	0.251	0.211	5.46E-105	T/NK Cells	CYLD
SCAND1	1.13E-107	0.345692	0.346	0.316	3.18E-103	T/NK Cells	SCAND1
BCL2	1.60E-107	0.653017	0.376	0.354	4.50E-103	T/NK Cells	BCL2
CLIC1	3.22E-106	0.251959	0.637	0.651	9.07E-102	T/NK Cells	CLIC1

SBDS	2.97E-105	0.342887	0.291	0.255	8.35E-101	T/NK Cells	SBDS
SERBP1	3.07E-104	0.302259	0.425	0.409	8.63E-100	T/NK Cells	SERBP1
TMEM258	3.98E-104	0.306959	0.401	0.377	1.12E-99	T/NK Cells	TMEM258
PLEKHA2	1.74E-103	0.457321	0.264	0.225	4.91E-99	T/NK Cells	PLEKHA2
NDUFB8	8.06E-102	0.302693	0.396	0.374	2.27E-97	T/NK Cells	NDUFB8
HELZ	1.40E-101	0.39714	0.254	0.216	3.94E-97	T/NK Cells	HELZ
TAGAP	9.28E-101	0.254489	0.529	0.495	2.61E-96	T/NK Cells	TAGAP
STK24	1.23E-100	0.37545	0.282	0.244	3.46E-96	T/NK Cells	STK24
EIF3H	6.09E-99	0.270032	0.478	0.469	1.71E-94	T/NK Cells	EIF3H
HMGN1	2.16E-97	0.282586	0.469	0.459	6.07E-93	T/NK Cells	HMGN1
COPS9	7.27E-97	0.284098	0.261	0.225	2.05E-92	T/NK Cells	COPS9
USP3	4.01E-96	0.403841	0.286	0.251	1.13E-91	T/NK Cells	USP3
ERP29	7.41E-96	0.285404	0.422	0.405	2.08E-91	T/NK Cells	ERP29
HNRNPA0	3.54E-95	0.301537	0.472	0.469	9.97E-91	T/NK Cells	HNRNPA0
RBM3	2.78E-94	0.275055	0.477	0.472	7.81E-90	T/NK Cells	RBM3
EMD	9.62E-94	0.323351	0.307	0.276	2.71E-89	T/NK Cells	EMD
HIGD2A	1.08E-93	0.288443	0.439	0.423	3.04E-89	T/NK Cells	HIGD2A
EPC1	6.63E-93	0.332219	0.479	0.47	1.87E-88	T/NK Cells	EPC1
PIK3R1	5.35E-91	0.385615	0.506	0.511	1.50E-86	T/NK Cells	PIK3R1
AKAP9	9.08E-89	0.405559	0.299	0.269	2.55E-84	T/NK Cells	AKAP9
ITGB1	1.08E-88	0.458938	0.365	0.353	3.03E-84	T/NK Cells	ITGB1
KHDRBS1	1.69E-87	0.285076	0.383	0.361	4.75E-83	T/NK Cells	KHDRBS1
TOMM20	5.24E-87	0.29486	0.378	0.36	1.48E-82	T/NK Cells	TOMM20
TRMT112	3.08E-86	0.303102	0.398	0.383	8.66E-82	T/NK Cells	TRMT112
TIPARP	1.08E-84	0.317147	0.265	0.228	3.04E-80	T/NK Cells	TIPARP
MIER1	4.26E-84	0.348475	0.251	0.219	1.20E-79	T/NK Cells	MIER1
FKBP8	5.16E-81	0.270619	0.39	0.375	1.45E-76	T/NK Cells	FKBP8
MACF1	1.59E-80	0.370035	0.452	0.443	4.47E-76	T/NK Cells	MACF1
MAPK1IP1l	2.89E-80	0.302096	0.328	0.303	8.14E-76	T/NK Cells	MAPK1IP1L
DYNLT1	3.72E-80	0.372974	0.326	0.301	1.05E-75	T/NK Cells	DYNLT1
ELOVL5	6.81E-80	0.354154	0.308	0.281	1.92E-75	T/NK Cells	ELOVL5
BBX	3.04E-79	0.381643	0.273	0.242	8.56E-75	T/NK Cells	BBX
EIF4B	1.27E-78	0.272827	0.387	0.371	3.58E-74	T/NK Cells	EIF4B
LSM7	4.38E-78	0.278254	0.339	0.314	1.23E-73	T/NK Cells	LSM7
TPR	1.23E-77	0.330809	0.306	0.28	3.47E-73	T/NK Cells	TPR
TGFB1	2.26E-77	0.346623	0.627	0.663	6.35E-73	T/NK Cells	TGFB1
PET100	2.66E-75	0.312634	0.256	0.226	7.49E-71	T/NK Cells	PET100
RHOC	3.06E-75	0.334548	0.277	0.252	8.60E-71	T/NK Cells	RHOC
NDFIP1	6.95E-74	0.436507	0.377	0.371	1.96E-69	T/NK Cells	NDFIP1
BZW1	1.30E-73	0.2638	0.399	0.384	3.64E-69	T/NK Cells	BZW1
SMAD7	4.54E-73	0.313554	0.268	0.236	1.28E-68	T/NK Cells	SMAD7
SMDT1	1.19E-72	0.30524	0.374	0.357	3.35E-68	T/NK Cells	SMDT1
RSF1	1.63E-71	0.345153	0.302	0.278	4.58E-67	T/NK Cells	RSF1
CSK	1.73E-68	0.27683	0.331	0.313	4.87E-64	T/NK Cells	CSK
NDUFB10	2.36E-67	0.254413	0.316	0.293	6.63E-63	T/NK Cells	NDUFB10
EIF3E	1.24E-66	0.261967	0.429	0.426	3.48E-62	T/NK Cells	EIF3E
GPBP1	3.50E-66	0.339807	0.316	0.294	9.86E-62	T/NK Cells	GPBP1

HNRNPR	1.14E-65	0.273897	0.277	0.253	3.21E-61	T/NK Cells	HNRNPR
TBCA	7.76E-64	0.277918	0.354	0.34	2.18E-59	T/NK Cells	TBCA
GRK2	1.93E-63	0.301862	0.297	0.275	5.43E-59	T/NK Cells	GRK2
SF3B2	4.34E-62	0.258751	0.394	0.386	1.22E-57	T/NK Cells	SF3B2
YTHDC1	6.87E-61	0.326385	0.295	0.274	1.93E-56	T/NK Cells	YTHDC1
UBE2I	1.39E-60	0.259917	0.301	0.28	3.91E-56	T/NK Cells	UBE2I
ITM2C	2.02E-60	0.559998	0.27	0.256	5.69E-56	T/NK Cells	ITM2C
BIRC6	4.08E-60	0.378925	0.337	0.319	1.15E-55	T/NK Cells	BIRC6
SLA	5.21E-59	0.376595	0.414	0.411	1.47E-54	T/NK Cells	SLA
POLR2A	2.32E-58	0.323074	0.288	0.266	6.53E-54	T/NK Cells	POLR2A
SRSF11	3.95E-58	0.305601	0.446	0.451	1.11E-53	T/NK Cells	SRSF11
FLI1	3.98E-58	0.309496	0.304	0.28	1.12E-53	T/NK Cells	FLI1
SND1	1.54E-57	0.342196	0.278	0.256	4.33E-53	T/NK Cells	SND1
USP34	1.06E-56	0.364362	0.312	0.293	2.99E-52	T/NK Cells	USP34
MAGOH	2.13E-56	0.25854	0.27	0.249	6.00E-52	T/NK Cells	MAGOH
VCP	1.06E-55	0.254057	0.399	0.391	2.97E-51	T/NK Cells	VCP
FP236383.3	3.03E-55	0.329179	0.427	0.512	8.53E-51	T/NK Cells	FP236383.3
GPS2	8.03E-54	0.26839	0.278	0.257	2.26E-49	T/NK Cells	GPS2
ARID1B	9.33E-54	0.413952	0.33	0.314	2.63E-49	T/NK Cells	ARID1B
MAX	2.33E-53	0.258706	0.278	0.256	6.57E-49	T/NK Cells	MAX
SNHG1	3.54E-53	0.256207	0.26	0.233	9.96E-49	T/NK Cells	SNHG1
JMJD6	3.66E-52	0.324262	0.272	0.253	1.03E-47	T/NK Cells	JMJD6
NDUFA3	4.37E-52	0.277454	0.312	0.294	1.23E-47	T/NK Cells	NDUFA3
RBIS	1.78E-51	0.260882	0.293	0.275	5.00E-47	T/NK Cells	RBIS
EIF3M	3.89E-51	0.258448	0.334	0.323	1.09E-46	T/NK Cells	EIF3M
C11orf58	2.28E-50	0.257933	0.395	0.394	6.41E-46	T/NK Cells	C11orf58
CREM	2.62E-50	0.394094	0.49	0.509	7.38E-46	T/NK Cells	CREM
MKNK2	8.02E-50	0.270284	0.265	0.244	2.26E-45	T/NK Cells	MKNK2
PSMB10	7.23E-49	0.290902	0.326	0.319	2.04E-44	T/NK Cells	PSMB10
DRAP1	1.17E-48	0.251973	0.43	0.432	3.30E-44	T/NK Cells	DRAP1
IDI1	3.14E-45	0.280741	0.29	0.272	8.82E-41	T/NK Cells	IDI1
TMEM50A	9.27E-45	0.256803	0.358	0.352	2.61E-40	T/NK Cells	TMEM50A
WDR33	3.89E-44	0.275778	0.252	0.233	1.09E-39	T/NK Cells	WDR33
PRPF4B	4.38E-44	0.29476	0.264	0.246	1.23E-39	T/NK Cells	PRPF4B
TAF1D	1.12E-43	0.289691	0.352	0.345	3.14E-39	T/NK Cells	TAF1D
ARHGAP45	1.75E-41	0.266024	0.315	0.303	4.92E-37	T/NK Cells	ARHGAP45
MAPRE2	1.18E-40	0.251537	0.347	0.333	3.32E-36	T/NK Cells	MAPRE2
OTUB1	1.37E-40	0.26142	0.277	0.264	3.85E-36	T/NK Cells	OTUB1
SRRM1	2.39E-40	0.251708	0.47	0.486	6.73E-36	T/NK Cells	SRRM1
AMD1	1.04E-39	0.281609	0.271	0.256	2.92E-35	T/NK Cells	AMD1
PLCL2	4.64E-38	0.343415	0.257	0.24	1.31E-33	T/NK Cells	PLCL2
TRAM1	1.23E-37	0.250289	0.283	0.271	3.45E-33	T/NK Cells	TRAM1
OSTF1	2.85E-36	0.255347	0.36	0.36	8.01E-32	T/NK Cells	OSTF1
TAX1BP1	1.09E-35	0.275347	0.334	0.33	3.08E-31	T/NK Cells	TAX1BP1
BCLAF1	1.63E-35	0.278328	0.385	0.388	4.59E-31	T/NK Cells	BCLAF1
TMEM123	1.40E-34	0.286892	0.273	0.263	3.95E-30	T/NK Cells	TMEM123
ARGLU1	7.59E-34	0.278756	0.451	0.467	2.14E-29	T/NK Cells	ARGLU1

GPRIN3	2.89E-32	0.355112	0.266	0.254	8.14E-28	T/NK Cells	GPRIN3
SP110	3.31E-31	0.287351	0.318	0.31	9.31E-27	T/NK Cells	SP110
MYADM	8.70E-31	0.270779	0.343	0.332	2.45E-26	T/NK Cells	MYADM
SMG1	7.73E-30	0.290855	0.26	0.248	2.17E-25	T/NK Cells	SMG1
ZBTB20	1.38E-28	0.42779	0.402	0.413	3.89E-24	T/NK Cells	ZBTB20
TUT4	9.53E-26	0.303479	0.253	0.243	2.68E-21	T/NK Cells	TUT4
TMEM259	5.33E-25	0.262531	0.294	0.292	1.50E-20	T/NK Cells	TMEM259
NR3C1	1.02E-23	0.322366	0.332	0.335	2.87E-19	T/NK Cells	NR3C1
DENND4A	8.12E-21	0.375472	0.312	0.312	2.28E-16	T/NK Cells	DENND4A
SLTM	1.05E-20	0.257401	0.331	0.335	2.95E-16	T/NK Cells	SLTM
ATRX	1.74E-19	0.279414	0.354	0.364	4.90E-15	T/NK Cells	ATRX
N4BP2L2	4.45E-11	0.272914	0.387	0.41	1.25E-06	T/NK Cells	N4BP2L2
PPP6R3	6.72E-11	0.276615	0.263	0.266	1.89E-06	T/NK Cells	PPP6R3
FP671120.4	4.74E-09	0.693312	0.404	0.424	0.000133	T/NK Cells	FP671120.4
PUM2	1.56E-08	0.267275	0.249	0.252	0.00044	T/NK Cells	PUM2
RAPGEF6	5.38E-06	0.286036	0.277	0.286	0.151468	T/NK Cells	RAPGEF6
AOAH	0.003233	0.559924	0.404	0.464	1	T/NK Cells	AOAH
STMN1	0	2.721054	0.726	0.18	0	Progenitor	STMN1
TUBB	0	2.378919	0.814	0.433	0	Progenitor	TUBB
MKI67	0	2.208329	0.502	0.013	0	Progenitor	MKI67
HIST1H4C	0	2.154539	0.613	0.335	0	Progenitor	HIST1H4C
HIST1H1E	0	2.018328	0.64	0.213	0	Progenitor	HIST1H1E
TUBA1B1	0	1.91079	0.945	0.723	0	Progenitor	TUBA1B
HMGN2	0	1.901266	0.744	0.483	0	Progenitor	HMGN2
TYMS	0	1.897578	0.424	0.014	0	Progenitor	TYMS
CENPF	0	1.897426	0.412	0.018	0	Progenitor	CENPF
UBE2C	0	1.848239	0.376	0.005	0	Progenitor	UBE2C
NUSAP1	0	1.847211	0.44	0.022	0	Progenitor	NUSAP1
RRM2	0	1.837441	0.401	0.012	0	Progenitor	RRM2
HIST1H2AI	0	1.811376	0.346	0.027	0	Progenitor	HIST1H2AI
TOP2A	0	1.786674	0.398	0.009	0	Progenitor	TOP2A
CCL21	0	1.700992	0.656	0.262	0	Progenitor	CCL2
HMGB2	0	1.69872	0.541	0.28	0	Progenitor	HMGB2
HIST1H1B	0	1.674234	0.353	0.023	0	Progenitor	HIST1H1B
H2AFZ	0	1.605513	0.695	0.475	0	Progenitor	H2AFZ
PCLAF	0	1.507619	0.374	0.013	0	Progenitor	PCLAF
TPX2	0	1.451449	0.353	0.009	0	Progenitor	TPX2
PTTG1	0	1.413149	0.354	0.056	0	Progenitor	PTTG1
ASPM	0	1.387107	0.291	0.004	0	Progenitor	ASPM
CKS1B	0	1.271317	0.353	0.05	0	Progenitor	CKS1B
AC011603.	0	1.260358	0.35	0.135	0	Progenitor	AC011603.2
CDK1	0	1.252185	0.322	0.023	0	Progenitor	CDK1
H2AFV1	0	1.240715	0.517	0.315	0	Progenitor	H2AFV
TK1	0	1.210682	0.294	0.011	0	Progenitor	TK1
TMPO	0	1.2084	0.488	0.208	0	Progenitor	TMPO
HIST1H1D	0	1.134655	0.324	0.103	0	Progenitor	HIST1H1D
KNL1	0	1.125995	0.291	0.01	0	Progenitor	KNL1

ANLN	0	1.082109	0.258	0.004	0	Progenitor	ANLN
CDKN3	0	1.050262	0.272	0.012	0	Progenitor	CDKN3
HMGB11	0	1.041693	0.767	0.759	0	Progenitor	HMGB1
LMNB1	0	1.004707	0.332	0.062	0	Progenitor	LMNB1
BIRC5	0	0.995761	0.263	0.003	0	Progenitor	BIRC5
GTSE1	0	0.99127	0.26	0.004	0	Progenitor	GTSE1
PRC1	0	0.975524	0.269	0.016	0	Progenitor	PRC1
FP236383.3	0	0.974327	0.843	0.48	0	Progenitor	FP236383.3
SMC4	0	0.967556	0.343	0.097	0	Progenitor	SMC4
PFN11	0	0.867407	0.915	0.834	0	Progenitor	PFN1
KIF22	0	0.840943	0.312	0.079	0	Progenitor	KIF22
CENPM	0	0.823841	0.253	0.027	0	Progenitor	CENPM
CKAP5	0	0.822553	0.322	0.118	0	Progenitor	CKAP5
NCAPD2	0	0.819217	0.27	0.042	0	Progenitor	NCAPD2
ACTB	0	0.80858	0.996	0.964	0	Progenitor	ACTB
MCM7	0	0.800434	0.258	0.061	0	Progenitor	MCM7
MT-ATP8	0	0.799502	0.919	0.783	0	Progenitor	MT-ATP8
GAPDH	0	0.784117	0.917	0.853	0	Progenitor	GAPDH
TACC3	0	0.735838	0.282	0.062	0	Progenitor	TACC3
CKAP2	0	0.71985	0.262	0.08	0	Progenitor	CKAP2
DTYMK	0	0.719124	0.255	0.053	0	Progenitor	DTYMK
CARHSP1	0	0.681477	0.27	0.065	0	Progenitor	CARHSP1
SMC2	0	0.666569	0.263	0.058	0	Progenitor	SMC2
G0S2	3.71E-298	0.967388	0.323	0.124	1.04E-293	Progenitor	G0S2
HIST1H1C	7.11E-294	1.033337	0.355	0.151	2.00E-289	Progenitor	HIST1H1C
ANP32E	2.20E-262	0.797663	0.336	0.145	6.18E-258	Progenitor	ANP32E
HAMP1	3.42E-257	1.108434	0.48	0.275	9.63E-253	Progenitor	HAMP
PTMA1	6.78E-252	0.576117	0.941	0.912	1.91E-247	Progenitor	PTMA
ZFP36L11	4.11E-249	0.689039	0.897	0.792	1.16E-244	Progenitor	ZFP36L1
IL1RN	1.07E-245	0.922896	0.302	0.125	3.02E-241	Progenitor	IL1RN
MT-CO1	3.99E-240	0.399213	0.987	0.982	1.12E-235	Progenitor	MT-CO1
KPNA2	4.57E-240	0.926809	0.338	0.156	1.29E-235	Progenitor	KPNA2
CKS21	9.67E-236	0.926661	0.46	0.257	2.72E-231	Progenitor	CKS2
HLA-DQB1	5.87E-232	0.707863	0.773	0.582	1.65E-227	Progenitor	HLA-DQB1
MT-CO2	7.44E-232	0.453422	0.979	0.964	2.09E-227	Progenitor	MT-CO2
MT-ND5	4.13E-229	0.638645	0.908	0.845	1.16E-224	Progenitor	MT-ND5
RPLP01	2.12E-227	0.505773	0.928	0.87	5.97E-223	Progenitor	RPLP0
SMC1A	4.07E-219	0.787459	0.361	0.177	1.14E-214	Progenitor	SMC1A
CFL1	1.25E-216	0.586908	0.862	0.831	3.52E-212	Progenitor	CFL1
CCL4L21	1.93E-211	0.444559	0.869	0.685	5.44E-207	Progenitor	CCL4L2
AC004448.	2.70E-206	0.80857	0.391	0.194	7.60E-202	Progenitor	AC004448.2
PPIA	4.78E-206	0.640457	0.812	0.799	1.35E-201	Progenitor	PPIA
DNMT1	1.19E-204	0.743067	0.36	0.18	3.36E-200	Progenitor	DNMT1
MT-ND6	1.62E-199	0.96549	0.684	0.516	4.57E-195	Progenitor	MT-ND6
FP671120.4	3.29E-198	0.434692	0.654	0.412	9.25E-194	Progenitor	FP671120.4
CALM31	2.46E-196	0.874795	0.418	0.24	6.93E-192	Progenitor	CALM3
H2AFX1	1.66E-191	0.818092	0.413	0.233	4.68E-187	Progenitor	H2AFX

NPC21	1.82E-188	0.670123	0.762	0.638	5.13E-184	Progenitor	NPC2
RPA3	1.67E-184	0.588435	0.269	0.118	4.69E-180	Progenitor	RPA3
HLA-DRB51	4.17E-183	0.583163	0.757	0.562	1.17E-178	Progenitor	HLA-DRB5
RAD211	1.33E-178	0.826125	0.422	0.251	3.73E-174	Progenitor	RAD21
PSAP1	4.18E-171	0.566854	0.871	0.765	1.18E-166	Progenitor	PSAP
LGALS1	9.10E-168	1.076427	0.371	0.209	2.56E-163	Progenitor	LGALS1
COTL1	2.03E-163	0.682547	0.715	0.639	5.70E-159	Progenitor	COTL1
DEK	4.37E-161	0.843218	0.497	0.36	1.23E-156	Progenitor	DEK
RNASE21	7.44E-157	0.845384	0.342	0.193	2.09E-152	Progenitor	RNASE2
MT-ND4L	7.79E-156	0.425984	0.934	0.907	2.19E-151	Progenitor	MT-ND4L
IDH21	2.30E-153	0.765046	0.383	0.228	6.47E-149	Progenitor	IDH2
COX8A	9.84E-152	0.840676	0.559	0.465	2.77E-147	Progenitor	COX8A
LSM4	1.72E-148	0.690202	0.363	0.207	4.83E-144	Progenitor	LSM4
DBI1	4.71E-147	0.501234	0.755	0.629	1.33E-142	Progenitor	DBI
C1QB1	1.61E-145	0.396874	0.854	0.703	4.53E-141	Progenitor	C1QB
C31	3.79E-136	0.459285	0.817	0.68	1.07E-131	Progenitor	C3
RANBP1	1.09E-133	0.721946	0.343	0.201	3.07E-129	Progenitor	RANBP1
NUDT1	7.58E-133	0.476359	0.27	0.135	2.13E-128	Progenitor	NUDT1
SAE1	2.47E-132	0.456734	0.26	0.126	6.95E-128	Progenitor	SAE1
TUBA1C	5.88E-130	0.832968	0.415	0.273	1.66E-125	Progenitor	TUBA1C
IL1B1	5.61E-124	0.294282	0.705	0.505	1.58E-119	Progenitor	IL1B
APOC21	4.25E-122	0.468667	0.672	0.496	1.19E-117	Progenitor	APOC2
FCGBP1	1.42E-121	0.562421	0.455	0.292	3.99E-117	Progenitor	FCGBP
MZT2B1	3.74E-119	0.757234	0.522	0.429	1.05E-114	Progenitor	MZT2B
ANP32B	2.65E-118	0.761364	0.489	0.381	7.47E-114	Progenitor	ANP32B
CTSB1	3.70E-117	0.455292	0.839	0.732	1.04E-112	Progenitor	CTSB
YBX1	2.18E-116	0.58142	0.677	0.633	6.13E-112	Progenitor	YBX1
HDGF	1.43E-113	0.500124	0.259	0.135	4.03E-109	Progenitor	HDGF
PKM	2.63E-112	0.725629	0.53	0.422	7.39E-108	Progenitor	PKM
RAN	5.78E-109	0.798778	0.529	0.459	1.63E-104	Progenitor	RAN
CXorf211	8.33E-108	0.764431	0.329	0.203	2.34E-103	Progenitor	CXorf21
LSM5	1.16E-107	0.493927	0.278	0.152	3.25E-103	Progenitor	LSM5
DUT	1.36E-107	0.856423	0.394	0.276	3.83E-103	Progenitor	DUT
HINT2	2.43E-107	0.395523	0.251	0.129	6.84E-103	Progenitor	HINT2
ARL6IP1	8.48E-105	0.997865	0.587	0.537	2.39E-100	Progenitor	ARL6IP1
ALYREF	1.27E-96	0.593791	0.324	0.201	3.58E-92	Progenitor	ALYREF
PPT11	2.31E-96	0.731203	0.548	0.439	6.50E-92	Progenitor	PPT1
C1QC1	1.04E-95	0.295832	0.856	0.708	2.94E-91	Progenitor	C1QC
UQCC2	2.25E-95	0.42737	0.26	0.143	6.34E-91	Progenitor	UQCC2
SIVA1	1.02E-94	0.667293	0.366	0.248	2.88E-90	Progenitor	SIVA1
PLAC8	2.03E-93	0.630163	0.34	0.217	5.70E-89	Progenitor	PLAC8
IER31	7.99E-93	0.412673	0.734	0.604	2.25E-88	Progenitor	IER3
BANF1	2.08E-92	0.625424	0.403	0.283	5.87E-88	Progenitor	BANF1
H1FX1	6.28E-92	0.567079	0.536	0.417	1.77E-87	Progenitor	H1FX
ENO1	1.28E-91	0.657729	0.542	0.462	3.59E-87	Progenitor	ENO1
VIM1	4.12E-90	0.360906	0.647	0.497	1.16E-85	Progenitor	VIM
FAM111A	4.13E-90	0.472915	0.282	0.164	1.16E-85	Progenitor	FAM111A

AP2S1	3.19E-89	0.635681	0.418	0.305	8.97E-85	Progenitor	AP2S1
SLC25A5	6.89E-89	0.647964	0.575	0.525	1.94E-84	Progenitor	SLC25A5
YWHAH1	1.02E-88	0.597824	0.617	0.522	2.88E-84	Progenitor	YWHAH
HNRNPA2B	6.93E-88	0.404476	0.771	0.789	1.95E-83	Progenitor	HNRNPA2B1
PADI21	8.38E-87	0.517604	0.553	0.437	2.36E-82	Progenitor	PADI2
CD141	9.33E-87	0.43159	0.595	0.48	2.63E-82	Progenitor	CD14
MT-CO3	3.55E-85	0.250088	0.965	0.941	9.99E-81	Progenitor	MT-CO3
PSMC3	9.87E-84	0.457441	0.28	0.166	2.78E-79	Progenitor	PSMC3
NR4A11	2.53E-83	0.324875	0.777	0.65	7.12E-79	Progenitor	NR4A1
ANAPC11	3.55E-83	0.633535	0.413	0.306	1.00E-78	Progenitor	ANAPC11
JPT1	2.01E-82	0.666748	0.376	0.268	5.65E-78	Progenitor	JPT1
FABP5	3.21E-82	0.495358	0.329	0.21	9.05E-78	Progenitor	FABP5
EGR21	8.89E-82	0.496961	0.519	0.389	2.50E-77	Progenitor	EGR2
HSP90B1	1.34E-81	0.510757	0.629	0.556	3.77E-77	Progenitor	HSP90B1
AIF11	7.98E-81	0.490265	0.677	0.603	2.25E-76	Progenitor	AIF1
PARP1	1.57E-80	0.598308	0.368	0.255	4.41E-76	Progenitor	PARP1
CDK2AP2	2.36E-80	0.436343	0.294	0.179	6.63E-76	Progenitor	CDK2AP2
GLUL1	5.80E-79	0.505139	0.678	0.604	1.63E-74	Progenitor	GLUL
TPM3	6.93E-79	0.523604	0.654	0.617	1.95E-74	Progenitor	TPM3
PRDX3	5.99E-77	0.647607	0.358	0.25	1.68E-72	Progenitor	PRDX3
CAPG1	2.39E-76	0.597401	0.52	0.445	6.74E-72	Progenitor	CAPG
PSIP1	6.35E-74	0.384745	0.279	0.168	1.79E-69	Progenitor	PSIP1
MZT2A1	8.00E-74	0.39395	0.313	0.196	2.25E-69	Progenitor	MZT2A
MCM5	1.67E-73	0.569702	0.254	0.157	4.71E-69	Progenitor	MCM5
PSME21	2.71E-72	0.614847	0.462	0.368	7.61E-68	Progenitor	PSME2
PGAM1	2.37E-70	0.592093	0.39	0.289	6.67E-66	Progenitor	PGAM1
NUCKS1	2.49E-70	0.646867	0.442	0.361	7.00E-66	Progenitor	NUCKS1
NR4A31	6.67E-69	0.393378	0.645	0.543	1.88E-64	Progenitor	NR4A3
TPI1	5.54E-68	0.529448	0.606	0.578	1.56E-63	Progenitor	TPI1
OSM1	5.95E-68	0.622567	0.34	0.239	1.68E-63	Progenitor	OSM
RBBP7	7.97E-67	0.396197	0.258	0.157	2.24E-62	Progenitor	RBBP7
DDX39A	1.28E-66	0.500535	0.391	0.289	3.60E-62	Progenitor	DDX39A
NASP	4.45E-66	0.535367	0.393	0.294	1.25E-61	Progenitor	NASP
ETFB	1.06E-64	0.412069	0.282	0.179	2.98E-60	Progenitor	ETFB
MRPL51	1.50E-64	0.426891	0.308	0.202	4.22E-60	Progenitor	MRPL51
CALR	1.57E-64	0.509651	0.612	0.552	4.42E-60	Progenitor	CALR
S100A111	1.69E-64	0.328304	0.781	0.724	4.74E-60	Progenitor	S100A11
DCTN3	5.90E-64	0.463327	0.275	0.176	1.66E-59	Progenitor	DCTN3
BTG21	2.12E-63	0.42909	0.734	0.676	5.96E-59	Progenitor	BTG2
PRKDC	1.25E-60	0.519434	0.359	0.258	3.52E-56	Progenitor	PRKDC
STX17-AS1	4.58E-60	0.71702	0.428	0.338	1.29E-55	Progenitor	STX17-AS1
BUB31	5.83E-60	0.394496	0.312	0.207	1.64E-55	Progenitor	BUB3
SCLT1	2.00E-59	0.367373	0.275	0.176	5.63E-55	Progenitor	SCLT1
TPM41	1.95E-57	0.470892	0.469	0.372	5.48E-53	Progenitor	TPM4
SNRPD1	2.21E-57	0.435819	0.306	0.207	6.23E-53	Progenitor	SNRPD1
HNRNPAB	8.73E-57	0.580479	0.374	0.282	2.46E-52	Progenitor	HNRNPAB
RHOA	1.70E-56	0.367912	0.715	0.725	4.78E-52	Progenitor	RHOA

ACTN41	3.73E-56	0.350038	0.294	0.193	1.05E-51	Progenitor	ACTN4
CALM2	1.09E-54	0.474729	0.669	0.683	3.08E-50	Progenitor	CALM2
HIF1A1	9.28E-54	0.360023	0.657	0.579	2.61E-49	Progenitor	HIF1A
EIF5A	6.49E-53	0.544508	0.419	0.337	1.83E-48	Progenitor	EIF5A
PSMB2	1.50E-52	0.466025	0.308	0.213	4.23E-48	Progenitor	PSMB2
CMC2	1.93E-52	0.329712	0.259	0.166	5.42E-48	Progenitor	CMC2
TMEM160	2.27E-52	0.339479	0.279	0.184	6.38E-48	Progenitor	TMEM160
PHPT1	2.58E-52	0.413258	0.299	0.203	7.25E-48	Progenitor	PHPT1
SCIN1	5.80E-50	0.549374	0.49	0.419	1.63E-45	Progenitor	SCIN
HP1BP3	1.93E-49	0.530937	0.448	0.377	5.44E-45	Progenitor	HP1BP3
LSM3	5.38E-49	0.336096	0.284	0.191	1.51E-44	Progenitor	LSM3
OAZ11	1.30E-48	0.300006	0.765	0.765	3.66E-44	Progenitor	OAZ1
UBE2S1	2.09E-48	0.545218	0.408	0.326	5.88E-44	Progenitor	UBE2S
PA2G4	2.22E-48	0.481376	0.374	0.288	6.24E-44	Progenitor	PA2G4
NCL1	6.37E-48	0.437333	0.626	0.599	1.79E-43	Progenitor	NCL
PSMA4	1.02E-45	0.407646	0.277	0.191	2.88E-41	Progenitor	PSMA4
CTSZ1	1.17E-45	0.510679	0.544	0.502	3.28E-41	Progenitor	CTSZ
PRKCA	1.60E-43	0.530093	0.377	0.296	4.51E-39	Progenitor	PRKCA
ALOX5AP1	3.31E-43	0.378093	0.662	0.641	9.32E-39	Progenitor	ALOX5AP
GNG51	8.59E-43	0.464951	0.567	0.555	2.42E-38	Progenitor	GNG5
HNRNPA3	3.64E-42	0.477679	0.566	0.534	1.02E-37	Progenitor	HNRNPA3
SUMO3	5.23E-41	0.403163	0.29	0.206	1.47E-36	Progenitor	SUMO3
SRRT	9.39E-41	0.294734	0.255	0.171	2.64E-36	Progenitor	SRRT
GSTP11	1.06E-40	0.470617	0.549	0.522	2.99E-36	Progenitor	GSTP1
COX5A	2.37E-40	0.511041	0.447	0.393	6.67E-36	Progenitor	COX5A
TAGLN21	2.81E-40	0.378122	0.446	0.369	7.90E-36	Progenitor	TAGLN2
APOO	8.89E-40	0.318693	0.358	0.263	2.50E-35	Progenitor	APOO
GNAI2	1.63E-39	0.389687	0.632	0.639	4.60E-35	Progenitor	GNAI2
C17orf49	3.03E-39	0.363984	0.282	0.2	8.53E-35	Progenitor	C17orf49
XPO1	4.32E-39	0.299816	0.275	0.19	1.21E-34	Progenitor	XPO1
IQGAP11	6.24E-39	0.34452	0.423	0.333	1.76E-34	Progenitor	IQGAP1
NME21	2.67E-38	0.397044	0.616	0.593	7.51E-34	Progenitor	NME2
BLOC1S1	4.08E-38	0.436295	0.418	0.347	1.15E-33	Progenitor	BLOC1S1
UQCRQ	1.92E-37	0.439466	0.399	0.33	5.39E-33	Progenitor	UQCRQ
SNRPC	2.59E-37	0.37144	0.294	0.213	7.29E-33	Progenitor	SNRPC
PPP1CA1	2.86E-36	0.519823	0.436	0.383	8.06E-32	Progenitor	PPP1CA
HNRNPU	3.09E-36	0.395769	0.619	0.587	8.70E-32	Progenitor	HNRNPU
SLC8A11	3.54E-36	0.36059	0.568	0.534	9.95E-32	Progenitor	SLC8A1
PDIA6	4.55E-36	0.44193	0.353	0.279	1.28E-31	Progenitor	PDIA6
TSPO1	5.59E-36	0.422523	0.561	0.526	1.57E-31	Progenitor	TSPO
AK2	8.82E-36	0.464326	0.285	0.208	2.48E-31	Progenitor	AK2
CCT5	1.75E-35	0.385542	0.287	0.209	4.93E-31	Progenitor	CCT5
PPM1G	2.96E-35	0.34401	0.297	0.22	8.32E-31	Progenitor	PPM1G
PIN1	2.23E-34	0.260306	0.262	0.183	6.27E-30	Progenitor	PIN1
SUZ12	5.33E-34	0.282517	0.25	0.175	1.50E-29	Progenitor	SUZ12
PDCD5	8.08E-34	0.292723	0.26	0.183	2.27E-29	Progenitor	PDCD5
ACTG11	1.19E-33	0.385752	0.805	0.797	3.35E-29	Progenitor	ACTG1

SAMHD1	1.46E-33	0.448749	0.508	0.453	4.11E-29	Progenitor	SAMHD1
TUBB4B1	1.58E-33	0.350291	0.503	0.441	4.45E-29	Progenitor	TUBB4B
MS4A4A1	1.69E-33	0.463866	0.346	0.281	4.76E-29	Progenitor	MS4A4A
TRAPPC1	3.85E-33	0.407654	0.375	0.3	1.08E-28	Progenitor	TRAPPC1
RHEB1	5.91E-33	0.42596	0.449	0.386	1.66E-28	Progenitor	RHEB
PRKAR1A	6.29E-33	0.491138	0.268	0.196	1.77E-28	Progenitor	PRKAR1A
SNRPB1	8.83E-33	0.449562	0.4	0.342	2.49E-28	Progenitor	SNRPB
SNRPG	9.14E-33	0.372036	0.377	0.307	2.57E-28	Progenitor	SNRPG
A2M1	1.22E-32	0.256097	0.642	0.588	3.42E-28	Progenitor	A2M
P4HB	1.28E-32	0.424389	0.408	0.342	3.61E-28	Progenitor	P4HB
PPIF	1.66E-32	0.407833	0.254	0.185	4.68E-28	Progenitor	PPIF
TALDO1	1.37E-31	0.430628	0.389	0.326	3.86E-27	Progenitor	TALDO1
TAF15	4.49E-31	0.377851	0.378	0.303	1.26E-26	Progenitor	TAF15
HMGA1	3.30E-30	0.365726	0.27	0.202	9.27E-26	Progenitor	HMGA1
CHCHD21	3.51E-30	0.413667	0.569	0.583	9.88E-26	Progenitor	CHCHD2
LSM2	5.41E-30	0.284731	0.258	0.188	1.52E-25	Progenitor	LSM2
SSNA1	8.03E-30	0.259231	0.262	0.189	2.26E-25	Progenitor	SSNA1
PRRC2A	1.01E-29	0.425447	0.289	0.219	2.84E-25	Progenitor	PRRC2A
AC131944.	1.13E-29	0.489783	0.266	0.203	3.17E-25	Progenitor	AC131944.1
SET1	2.69E-29	0.447155	0.455	0.406	7.56E-25	Progenitor	SET
CLTC1	2.75E-29	0.480022	0.37	0.303	7.73E-25	Progenitor	CLTC
CLIC11	3.38E-29	0.342682	0.623	0.648	9.51E-25	Progenitor	CLIC1
AURKAIP1	4.40E-29	0.347556	0.326	0.252	1.24E-24	Progenitor	AURKAIP1
GDI2	1.12E-28	0.49359	0.474	0.427	3.16E-24	Progenitor	GDI2
NONO	1.42E-28	0.392315	0.349	0.279	4.01E-24	Progenitor	NONO
NDUFB2	2.18E-28	0.433871	0.452	0.405	6.12E-24	Progenitor	NDUFB2
RPL4	3.04E-28	0.30283	0.737	0.731	8.55E-24	Progenitor	RPL4
NDUFA2	4.37E-28	0.32573	0.341	0.271	1.23E-23	Progenitor	NDUFA2
ARPC1B	9.41E-28	0.317252	0.633	0.642	2.65E-23	Progenitor	ARPC1B
QKI1	1.22E-27	0.287771	0.653	0.626	3.44E-23	Progenitor	QKI
PRDX2	1.52E-27	0.312675	0.26	0.193	4.27E-23	Progenitor	PRDX2
PSMB6	7.99E-27	0.42839	0.368	0.308	2.25E-22	Progenitor	PSMB6
CYC1	1.49E-26	0.34596	0.291	0.222	4.19E-22	Progenitor	CYC1
PLEK1	1.76E-26	0.297038	0.512	0.457	4.95E-22	Progenitor	PLEK
MIF1	2.14E-26	0.359202	0.567	0.567	6.01E-22	Progenitor	MIF
KHDRBS11	2.33E-26	0.374756	0.425	0.365	6.55E-22	Progenitor	KHDRBS1
NDUFB3	2.33E-26	0.317878	0.285	0.216	6.57E-22	Progenitor	NDUFB3
MYL61	3.54E-26	0.257362	0.724	0.77	9.97E-22	Progenitor	MYL6
CAPRIN1	5.79E-26	0.401655	0.301	0.236	1.63E-21	Progenitor	CAPRIN1
MDH1	5.87E-26	0.291947	0.286	0.218	1.65E-21	Progenitor	MDH1
LDHA1	9.82E-26	0.335099	0.509	0.475	2.76E-21	Progenitor	LDHA
ERH	1.17E-25	0.341208	0.347	0.284	3.30E-21	Progenitor	ERH
NENF	1.23E-25	0.318018	0.291	0.225	3.47E-21	Progenitor	NENF
PSMD2	1.60E-25	0.293338	0.26	0.195	4.51E-21	Progenitor	PSMD2
PSMD8	3.10E-25	0.391214	0.344	0.28	8.72E-21	Progenitor	PSMD8
DCXR	4.61E-25	0.299402	0.296	0.228	1.30E-20	Progenitor	DCXR
HNRNPF	4.32E-24	0.442756	0.472	0.441	1.22E-19	Progenitor	HNRNPF

ATP5MC3	5.39E-24	0.4387	0.455	0.426	1.52E-19	Progenitor	ATP5MC3
PTMS	5.85E-24	0.446532	0.265	0.21	1.65E-19	Progenitor	PTMS
NUTF2	3.53E-23	0.337305	0.277	0.214	9.93E-19	Progenitor	NUTF2
KPNB1	4.71E-23	0.371305	0.356	0.295	1.32E-18	Progenitor	KPNB1
HSBP11	5.69E-23	0.357598	0.298	0.236	1.60E-18	Progenitor	HSBP1
UQCRC1	8.05E-23	0.317244	0.292	0.229	2.27E-18	Progenitor	UQCRC1
HINT11	2.22E-22	0.347104	0.51	0.493	6.25E-18	Progenitor	HINT1
CORO1A1	2.63E-22	0.384421	0.625	0.649	7.40E-18	Progenitor	CORO1A
ILF2	3.07E-22	0.327439	0.337	0.278	8.64E-18	Progenitor	ILF2
NDUFS8	3.45E-22	0.319491	0.331	0.269	9.70E-18	Progenitor	NDUFS8
MRPL52	3.78E-22	0.319513	0.287	0.226	1.06E-17	Progenitor	MRPL52
ATP5MC1	4.06E-22	0.360418	0.283	0.225	1.14E-17	Progenitor	ATP5MC1
EGR31	4.36E-22	0.386698	0.398	0.347	1.23E-17	Progenitor	EGR3
EIF4G1	7.18E-22	0.365187	0.285	0.225	2.02E-17	Progenitor	EIF4G1
UCP21	7.40E-22	0.33225	0.416	0.363	2.08E-17	Progenitor	UCP2
PFKL	1.31E-21	0.296014	0.254	0.195	3.68E-17	Progenitor	PFKL
VKORC1	2.22E-21	0.304278	0.275	0.215	6.26E-17	Progenitor	VKORC1
ILF3	2.33E-21	0.300183	0.352	0.289	6.55E-17	Progenitor	ILF3
TPR1	2.73E-21	0.259522	0.347	0.285	7.69E-17	Progenitor	TPR
MLEC	3.12E-21	0.339214	0.264	0.207	8.77E-17	Progenitor	MLEC
CLTA1	4.46E-21	0.349837	0.441	0.393	1.25E-16	Progenitor	CLTA
NDUFC2	5.32E-21	0.35946	0.4	0.349	1.50E-16	Progenitor	NDUFC2
INSIG1	5.83E-21	0.355368	0.26	0.203	1.64E-16	Progenitor	INSIG1
SRP91	6.15E-21	0.336615	0.328	0.271	1.73E-16	Progenitor	SRP9
RAC21	7.99E-21	0.307574	0.349	0.284	2.25E-16	Progenitor	RAC2
NDUFB6	8.95E-21	0.252977	0.265	0.205	2.52E-16	Progenitor	NDUFB6
LSP11	9.12E-21	0.272483	0.44	0.384	2.57E-16	Progenitor	LSP1
TLN11	1.03E-20	0.389627	0.529	0.499	2.91E-16	Progenitor	TLN1
ARPC5	1.43E-20	0.374743	0.465	0.435	4.02E-16	Progenitor	ARPC5
NDUFAF3	3.06E-20	0.310498	0.316	0.255	8.61E-16	Progenitor	NDUFAF3
NDUFA6	3.38E-20	0.28865	0.31	0.251	9.50E-16	Progenitor	NDUFA6
RCC21	4.13E-20	0.412538	0.336	0.281	1.16E-15	Progenitor	RCC2
ATP5MF	1.18E-19	0.376172	0.433	0.397	3.33E-15	Progenitor	ATP5MF
COX6A1	1.30E-19	0.323685	0.559	0.592	3.66E-15	Progenitor	COX6A1
PSMB91	2.63E-19	0.365809	0.386	0.337	7.39E-15	Progenitor	PSMB9
HMGN11	3.34E-19	0.36725	0.477	0.461	9.40E-15	Progenitor	HMGN1
SUMO21	3.47E-19	0.361916	0.537	0.558	9.76E-15	Progenitor	SUMO2
CX3CR11	4.44E-19	0.326238	0.487	0.442	1.25E-14	Progenitor	CX3CR1
CKLF	6.20E-19	0.327784	0.414	0.369	1.75E-14	Progenitor	CKLF
CANX1	1.10E-18	0.432961	0.472	0.437	3.08E-14	Progenitor	CANX
GSTO1	1.15E-18	0.279181	0.277	0.219	3.23E-14	Progenitor	GSTO1
NDUFB11	2.01E-18	0.357828	0.443	0.413	5.65E-14	Progenitor	NDUFB11
SPI11	3.01E-18	0.365892	0.518	0.514	8.46E-14	Progenitor	SPI1
FSCN11	7.90E-18	0.439132	0.352	0.316	2.22E-13	Progenitor	FSCN1
PSMA2	1.16E-17	0.341057	0.327	0.28	3.27E-13	Progenitor	PSMA2
ATG3	1.71E-17	0.297922	0.258	0.203	4.80E-13	Progenitor	ATG3
HNRNPD	4.24E-17	0.349827	0.459	0.435	1.19E-12	Progenitor	HNRNPD

HADHA	5.11E-17	0.363939	0.372	0.323	1.44E-12	Progenitor	HADHA
PSMB3	6.89E-17	0.328532	0.383	0.339	1.94E-12	Progenitor	PSMB3
PARK71	1.55E-16	0.338854	0.43	0.396	4.36E-12	Progenitor	PARK7
PSMC4	2.38E-16	0.311089	0.272	0.22	6.69E-12	Progenitor	PSMC4
EMP31	3.33E-16	0.256726	0.388	0.335	9.37E-12	Progenitor	EMP3
ALCAM1	9.27E-16	0.268881	0.342	0.292	2.61E-11	Progenitor	ALCAM
PSMC5	1.92E-15	0.287794	0.285	0.235	5.42E-11	Progenitor	PSMC5
ROMO1	2.04E-15	0.254571	0.32	0.265	5.74E-11	Progenitor	ROMO1
NHP2	2.66E-15	0.275253	0.258	0.209	7.48E-11	Progenitor	NHP2
CAP1	2.87E-15	0.372463	0.536	0.527	8.06E-11	Progenitor	CAP1
NDUFA4	3.68E-15	0.354037	0.503	0.507	1.03E-10	Progenitor	NDUFA4
RBX1	2.28E-14	0.335088	0.384	0.344	6.43E-10	Progenitor	RBX1
PTTG1IP1	3.32E-14	0.427762	0.352	0.316	9.34E-10	Progenitor	PTTG1IP
HIF1A-AS3:	3.53E-14	0.386552	0.25	0.208	9.92E-10	Progenitor	HIF1A-AS3
MS4A6A1	4.58E-14	0.271984	0.444	0.406	1.29E-09	Progenitor	MS4A6A
PSMB101	7.16E-14	0.299538	0.363	0.32	2.01E-09	Progenitor	PSMB10
BAX	8.26E-14	0.301344	0.421	0.385	2.32E-09	Progenitor	BAX
RBBP4	9.69E-14	0.28237	0.295	0.247	2.73E-09	Progenitor	RBBP4
CBX3	9.82E-14	0.256777	0.313	0.266	2.76E-09	Progenitor	CBX3
YWHAE1	1.34E-13	0.344386	0.434	0.403	3.78E-09	Progenitor	YWHAE
XRCC6	1.42E-13	0.309189	0.415	0.379	4.00E-09	Progenitor	XRCC6
XRCC5	2.19E-13	0.357506	0.437	0.414	6.16E-09	Progenitor	XRCC5
PAK11	2.29E-13	0.301554	0.267	0.223	6.44E-09	Progenitor	PAK1
SF3B21	2.35E-13	0.306504	0.422	0.387	6.62E-09	Progenitor	SF3B2
TFRC1	3.93E-13	0.287142	0.311	0.269	1.11E-08	Progenitor	TFRC
PSMB81	6.99E-13	0.301233	0.365	0.326	1.97E-08	Progenitor	PSMB8
C12orf75	8.84E-13	0.2622	0.286	0.24	2.49E-08	Progenitor	C12orf75
ATP5PF	8.89E-13	0.302575	0.387	0.354	2.50E-08	Progenitor	ATP5PF
NDUFV2	9.79E-13	0.254244	0.336	0.29	2.76E-08	Progenitor	NDUFV2
NDUFA11	1.02E-12	0.313362	0.452	0.429	2.86E-08	Progenitor	NDUFA11
MICOS10	1.08E-12	0.312647	0.453	0.43	3.05E-08	Progenitor	MICOS10
TUBA1A1	1.14E-12	0.411054	0.361	0.32	3.21E-08	Progenitor	TUBA1A
NUCB1	2.16E-12	0.341379	0.349	0.305	6.08E-08	Progenitor	NUCB1
GRB21	2.21E-12	0.296677	0.524	0.503	6.21E-08	Progenitor	GRB2
DYNLL1	3.08E-12	0.3269	0.396	0.367	8.67E-08	Progenitor	DYNLL1
ATP5PB	4.61E-12	0.337611	0.376	0.341	1.30E-07	Progenitor	ATP5PB
H2AFY1	5.53E-12	0.288155	0.466	0.442	1.56E-07	Progenitor	H2AFY
PEA151	7.72E-12	0.404153	0.269	0.233	2.17E-07	Progenitor	PEA15
TM9SF2	3.86E-11	0.378964	0.358	0.322	1.09E-06	Progenitor	TM9SF2
ATP5F1B	4.96E-11	0.331902	0.52	0.526	1.39E-06	Progenitor	ATP5F1B
NAP1L1	6.81E-11	0.291739	0.431	0.412	1.92E-06	Progenitor	NAP1L1
NDUFB9	7.51E-11	0.282288	0.363	0.325	2.11E-06	Progenitor	NDUFB9
SPRED11	9.46E-11	0.319336	0.272	0.237	2.66E-06	Progenitor	SPRED1
POLR2E	1.04E-10	0.257355	0.302	0.26	2.94E-06	Progenitor	POLR2E
RALY	2.27E-10	0.318356	0.438	0.426	6.38E-06	Progenitor	RALY
SUSD3	2.57E-10	0.306596	0.257	0.221	7.24E-06	Progenitor	SUSD3
ZYX	2.75E-10	0.318098	0.444	0.421	7.74E-06	Progenitor	ZYX

STK38L1	2.83E-10	0.311575	0.293	0.259	7.96E-06	Progenitor	STK38L
APLP21	3.47E-10	0.368266	0.397	0.38	9.77E-06	Progenitor	APLP2
NDUFS7	4.31E-10	0.304117	0.344	0.309	1.21E-05	Progenitor	NDUFS7
UQCR10	5.11E-10	0.275122	0.463	0.448	1.44E-05	Progenitor	UQCR10
COPE	6.82E-10	0.287101	0.431	0.417	1.92E-05	Progenitor	COPE
OLFML31	6.91E-10	0.335725	0.337	0.312	1.94E-05	Progenitor	OLFML3
TUFM	9.10E-10	0.321332	0.341	0.308	2.56E-05	Progenitor	TUFM
TMEM258:	1.13E-09	0.270286	0.409	0.383	3.19E-05	Progenitor	TMEM258
HNRNPM	2.47E-09	0.252217	0.385	0.352	6.94E-05	Progenitor	HNRNPM
FKBP1A	2.67E-09	0.281698	0.311	0.276	7.52E-05	Progenitor	FKBP1A
YWHAQ1	5.22E-09	0.283633	0.39	0.363	0.000147	Progenitor	YWHAQ
PYCARD1	1.05E-08	0.315836	0.425	0.406	0.000297	Progenitor	PYCARD
TMEM109	1.16E-08	0.325903	0.284	0.252	0.000328	Progenitor	TMEM109
TMED9	1.67E-08	0.331359	0.364	0.343	0.000469	Progenitor	TMED9
VAMP81	2.90E-08	0.292779	0.479	0.48	0.000815	Progenitor	VAMP8
PTGES3	2.90E-08	0.302281	0.475	0.476	0.000816	Progenitor	PTGES3
KIF5B	3.52E-08	0.262816	0.374	0.345	0.000991	Progenitor	KIF5B
RTN3	6.79E-08	0.280643	0.295	0.263	0.00191	Progenitor	RTN3
ARPC21	7.47E-08	0.270794	0.587	0.64	0.002103	Progenitor	ARPC2
SRSF9	9.07E-08	0.286251	0.394	0.373	0.002553	Progenitor	SRSF9
GK1	9.66E-08	0.321731	0.298	0.274	0.002718	Progenitor	GK
CYFIP11	1.60E-07	0.283725	0.497	0.499	0.004503	Progenitor	CYFIP1
ABI31	1.63E-07	0.292362	0.374	0.349	0.004586	Progenitor	ABI3
NDUFA13	1.71E-07	0.265972	0.478	0.485	0.004803	Progenitor	NDUFA13
TMEM14C:	1.75E-07	0.323395	0.359	0.344	0.004936	Progenitor	TMEM14C
DBNL	2.47E-07	0.267521	0.293	0.26	0.006941	Progenitor	DBNL
ATP5PO	2.89E-07	0.291131	0.419	0.409	0.008132	Progenitor	ATP5PO
SPCS2	3.34E-07	0.254632	0.357	0.325	0.009394	Progenitor	SPCS2
ACTR21	4.59E-07	0.252257	0.54	0.547	0.012906	Progenitor	ACTR2
NUMA1	4.66E-07	0.273131	0.272	0.242	0.013123	Progenitor	NUMA1
LGMN1	4.74E-07	0.283285	0.338	0.318	0.013349	Progenitor	LGMN
ANP32A	6.69E-07	0.297028	0.399	0.376	0.018832	Progenitor	ANP32A
COX7B	7.90E-07	0.276103	0.411	0.393	0.022235	Progenitor	COX7B
ACTR3	7.95E-07	0.30091	0.462	0.456	0.022376	Progenitor	ACTR3
ARHGDIA	8.03E-07	0.276082	0.425	0.406	0.022601	Progenitor	ARHGDIA
BEST11	1.40E-06	0.412757	0.304	0.288	0.039398	Progenitor	BEST1
COX5B	1.47E-06	0.255549	0.476	0.482	0.041478	Progenitor	COX5B
SLC29A11	1.76E-06	0.299621	0.253	0.228	0.049561	Progenitor	SLC29A1
CNDP21	5.71E-06	0.290086	0.32	0.295	0.160652	Progenitor	CNDP2
ERP291	1.49E-05	0.264662	0.417	0.409	0.420561	Progenitor	ERP29
STAB11	2.51E-05	0.297052	0.274	0.257	0.705342	Progenitor	STAB1
RPN1	2.96E-05	0.269428	0.358	0.338	0.832038	Progenitor	RPN1
ATP5F1A	3.78E-05	0.270152	0.34	0.32	1	Progenitor	ATP5F1A
PDIA3	5.62E-05	0.259349	0.514	0.529	1	Progenitor	PDIA3
MSN1	6.64E-05	0.267312	0.522	0.539	1	Progenitor	MSN
SLC4A71	0.00014	0.269127	0.395	0.382	1	Progenitor	SLC4A7
SELENOK	0.000156	0.250234	0.452	0.445	1	Progenitor	SELENOK

GNB2	0.000229	0.258845	0.43	0.432	1	Progenitor	GNB2
MOB1A	0.000262	0.342991	0.378	0.367	1	Progenitor	MOB1A
TGOLN2	0.000311	0.299985	0.389	0.375	1	Progenitor	TGOLN2
LCP11	0.000499	0.268515	0.476	0.486	1	Progenitor	LCP1
AKR1A11	0.001299	0.261283	0.333	0.319	1	Progenitor	AKR1A1
SNX5	0.001353	0.251938	0.309	0.291	1	Progenitor	SNX5
MAN2B11	0.00518	0.29076	0.352	0.348	1	Progenitor	MAN2B1
NFIC1	0.008562	0.255131	0.321	0.315	1	Progenitor	NFIC
HBB	0	8.608517	0.977	0.033	0	Erythroid-L	HBB
HBA2	0	5.130658	0.838	0.007	0	Erythroid-L	HBA2
HBA1	0	4.422204	0.76	0.004	0	Erythroid-L	HBA1
SLC25A371	3.75E-161	1.337362	0.564	0.359	1.05E-156	Erythroid-L	SLC25A37
MTRNR2L1	1.30E-129	0.68606	0.729	0.5	3.67E-125	Erythroid-L	MTRNR2L12
XIST	1.31E-106	0.469318	0.262	0.109	3.69E-102	Erythroid-L	XIST
RPL411	2.47E-83	0.425821	0.905	0.832	6.94E-79	Erythroid-L	RPL41
CCL3L11	1.80E-70	0.737281	0.738	0.599	5.07E-66	Erythroid-L	CCL3L1
CXCL81	5.61E-59	0.807144	0.521	0.389	1.58E-54	Erythroid-L	CXCL8
TNFAIP31	2.96E-54	0.457843	0.702	0.602	8.33E-50	Erythroid-L	TNFAIP3
IL1B2	5.27E-54	0.663822	0.618	0.509	1.48E-49	Erythroid-L	IL1B
IL1A	4.13E-53	0.746579	0.303	0.185	1.16E-48	Erythroid-L	IL1A
RPL211	8.94E-51	0.418567	0.798	0.758	2.51E-46	Erythroid-L	RPL21
PPP1R15A1	1.28E-50	0.621957	0.728	0.696	3.59E-46	Erythroid-L	PPP1R15A
SERPINE11	3.65E-48	0.465195	0.457	0.315	1.03E-43	Erythroid-L	SERPINE1
TENT5C1	6.96E-46	0.47583	0.282	0.167	1.96E-41	Erythroid-L	TENT5C
BCL2A11	1.86E-43	0.743195	0.494	0.406	5.23E-39	Erythroid-L	BCL2A1
HLA-DQA2	3.95E-41	0.515208	0.273	0.169	1.11E-36	Erythroid-L	HLA-DQA2
UBB1	2.08E-40	0.637444	0.709	0.675	5.86E-36	Erythroid-L	UBB
ABL21	2.53E-38	0.724139	0.385	0.297	7.13E-34	Erythroid-L	ABL2
EGR11	4.38E-36	0.501474	0.744	0.696	1.23E-31	Erythroid-L	EGR1
SLC25A39	2.07E-35	0.840804	0.295	0.203	5.83E-31	Erythroid-L	SLC25A39
TUBB4B2	4.26E-29	0.520505	0.502	0.441	1.20E-24	Erythroid-L	TUBB4B
TNF1	1.50E-27	0.439593	0.482	0.395	4.21E-23	Erythroid-L	TNF
SRSF21	3.65E-27	0.492497	0.572	0.536	1.03E-22	Erythroid-L	SRSF2
CCL51	1.04E-26	0.319542	0.387	0.28	2.92E-22	Erythroid-L	CCL5
CH25H1	3.23E-24	0.255783	0.458	0.364	9.09E-20	Erythroid-L	CH25H
PMAIP1	7.87E-24	0.536313	0.351	0.279	2.21E-19	Erythroid-L	PMAIP1
RPS4X1	3.22E-23	0.29686	0.871	0.866	9.05E-19	Erythroid-L	RPS4X
NFKB11	7.41E-22	0.435713	0.586	0.547	2.09E-17	Erythroid-L	NFKB1
FBXO7	3.07E-21	0.546885	0.288	0.216	8.63E-17	Erythroid-L	FBXO7
MAP2K31	5.15E-21	0.430751	0.312	0.239	1.45E-16	Erythroid-L	MAP2K3
SOD21	4.43E-19	0.442053	0.527	0.49	1.25E-14	Erythroid-L	SOD2
NR4A21	9.20E-19	0.338761	0.745	0.752	2.59E-14	Erythroid-L	NR4A2
H2AFX2	3.70E-18	0.34896	0.308	0.237	1.04E-13	Erythroid-L	H2AFX
HLA-DRB52	4.33E-17	0.317747	0.601	0.567	1.22E-12	Erythroid-L	HLA-DRB5
IER32	1.53E-14	0.353949	0.624	0.607	4.31E-10	Erythroid-L	IER3
UBE2S2	5.27E-14	0.317626	0.387	0.327	1.48E-09	Erythroid-L	UBE2S
CDKN1A1	6.23E-14	0.347097	0.531	0.492	1.75E-09	Erythroid-L	CDKN1A

NFKBIZ1	2.17E-13	0.368304	0.524	0.49	6.11E-09	Erythroid-L	NFKBIZ
NPM11	2.46E-13	0.277548	0.685	0.71	6.93E-09	Erythroid-L	NPM1
EIF4A3	8.67E-13	0.4372	0.42	0.374	2.44E-08	Erythroid-L	EIF4A3
GBP21	1.90E-12	0.420179	0.403	0.356	5.34E-08	Erythroid-L	GBP2
CKB1	2.73E-12	0.400125	0.345	0.297	7.69E-08	Erythroid-L	CKB
GYPC1	4.17E-12	0.417106	0.423	0.373	1.17E-07	Erythroid-L	GYPC
KLF101	1.28E-11	0.40635	0.38	0.337	3.61E-07	Erythroid-L	KLF10
NLRP31	1.87E-10	0.317414	0.436	0.396	5.27E-06	Erythroid-L	NLRP3
SNHG5	4.47E-10	0.286734	0.418	0.379	1.26E-05	Erythroid-L	SNHG5
SIK31	1.36E-09	0.309971	0.488	0.459	3.83E-05	Erythroid-L	SIK3
SNHG11	1.63E-09	0.275537	0.288	0.239	4.59E-05	Erythroid-L	SNHG1
NR4A32	5.68E-09	0.286452	0.56	0.545	0.00016	Erythroid-L	NR4A3
ZFAS1	8.93E-09	0.328523	0.571	0.587	0.000251	Erythroid-L	ZFAS1
KDM6B1	2.09E-08	0.315073	0.481	0.467	0.000589	Erythroid-L	KDM6B
CSRNP1	2.22E-08	0.28957	0.477	0.457	0.000625	Erythroid-L	CSRNP1
BRD21	1.85E-07	0.292866	0.497	0.483	0.005193	Erythroid-L	BRD2
EIF4E1	5.38E-07	0.385788	0.38	0.36	0.015143	Erythroid-L	EIF4E
DDX3X	2.39E-06	0.307382	0.493	0.489	0.067267	Erythroid-L	DDX3X
BNIP3L1	4.15E-06	0.345814	0.326	0.293	0.116721	Erythroid-L	BNIP3L
NCOA41	1.03E-05	0.326619	0.345	0.32	0.289652	Erythroid-L	NCOA4
SRSF3	1.62E-05	0.279498	0.515	0.522	0.45608	Erythroid-L	SRSF3
FKBP81	0.008435	0.304866	0.388	0.379	1	Erythroid-L	FKBP8
SYAP1	0.008584	0.254358	0.363	0.353	1	Erythroid-L	SYAP1
IGFBP7	0	4.481193	0.412	0.022	0	Platelets	IGFBP7
SPARC	0	3.847405	0.406	0.012	0	Platelets	SPARC
SPARCL1	0	3.641212	0.387	0.004	0	Platelets	SPARCL1
IFI27	0	3.076223	0.314	0.039	0	Platelets	IFI27
TSC22D1	0	2.34529	0.34	0.045	0	Platelets	TSC22D1
TIMP3	0	2.262772	0.292	0.002	0	Platelets	TIMP3
TPM1	0	1.757128	0.279	0.055	0	Platelets	TPM1
CRIP2	0	1.713958	0.262	0.006	0	Platelets	CRIP2
ID3	0	1.705609	0.253	0.044	0	Platelets	ID3
SPTBN1	0	1.354923	0.262	0.055	0	Platelets	SPTBN1
EPAS1	7.06E-276	1.642176	0.265	0.067	1.99E-271	Platelets	EPAS1
APP	3.16E-257	1.832594	0.355	0.125	8.89E-253	Platelets	APP
HIST1H1E1	3.73E-162	1.493054	0.435	0.222	1.05E-157	Platelets	HIST1H1E
VIM2	1.80E-142	1.354578	0.681	0.499	5.08E-138	Platelets	VIM
STMN11	1.73E-139	1.304414	0.384	0.193	4.87E-135	Platelets	STMN1
TIMP1	1.07E-113	2.157008	0.334	0.173	3.00E-109	Platelets	TIMP1
IFITM31	5.88E-102	1.980402	0.518	0.395	1.66E-97	Platelets	IFITM3
GAPDH1	1.27E-100	0.664757	0.884	0.855	3.56E-96	Platelets	GAPDH
CLIC4	8.17E-87	0.997772	0.285	0.145	2.30E-82	Platelets	CLIC4
CD59	3.45E-85	1.244631	0.305	0.169	9.70E-81	Platelets	CD59
CCL22	5.29E-83	0.831753	0.439	0.271	1.49E-78	Platelets	CCL2
MT2A1	2.80E-77	1.86151	0.544	0.419	7.88E-73	Platelets	MT2A
FP236383.:	7.17E-68	0.80695	0.595	0.489	2.02E-63	Platelets	FP236383.3
LMNA	4.59E-63	0.834242	0.273	0.148	1.29E-58	Platelets	LMNA

A2M2	2.44E-62	1.000945	0.643	0.589	6.87E-58	Platelets	A2M
DSTN	1.13E-61	1.079215	0.299	0.18	3.18E-57	Platelets	DSTN
TUBB1	3.90E-61	1.056524	0.53	0.443	1.10E-56	Platelets	TUBB
ACTB1	1.31E-57	0.34	0.979	0.964	3.68E-53	Platelets	ACTB
TUBA1B2	1.19E-54	0.763724	0.765	0.728	3.35E-50	Platelets	TUBA1B
LGALS11	3.25E-51	1.201447	0.323	0.212	9.16E-47	Platelets	LGALS1
ANXA21	1.46E-50	0.868795	0.253	0.146	4.12E-46	Platelets	ANXA2
IFI61	2.74E-46	1.484448	0.38	0.277	7.72E-42	Platelets	IFI6
TAGLN22	1.12E-43	1.104579	0.445	0.37	3.16E-39	Platelets	TAGLN2
ACTN42	2.46E-41	0.710338	0.296	0.194	6.93E-37	Platelets	ACTN4
PTMS1	1.53E-34	0.777486	0.296	0.21	4.31E-30	Platelets	PTMS
ACTG12	7.72E-34	0.529466	0.761	0.798	2.17E-29	Platelets	ACTG1
PHLDA1	1.69E-32	0.784954	0.251	0.165	4.75E-28	Platelets	PHLDA1
SELENOW	5.86E-31	1.049466	0.384	0.332	1.65E-26	Platelets	SELENOW
MT-ND61	2.76E-29	0.78066	0.555	0.52	7.76E-25	Platelets	MT-ND6
SOCS3	1.45E-28	1.034721	0.26	0.186	4.09E-24	Platelets	SOCS3
FP671120.4	1.67E-28	0.484564	0.504	0.418	4.69E-24	Platelets	FP671120.4
IFITM11	1.87E-28	1.020324	0.374	0.294	5.26E-24	Platelets	IFITM1
RHOC1	7.36E-26	0.996889	0.319	0.257	2.07E-21	Platelets	RHOC
CYB5R3	2.16E-25	0.787847	0.284	0.216	6.08E-21	Platelets	CYB5R3
RGCC1	5.28E-25	0.960674	0.41	0.338	1.48E-20	Platelets	RGCC
MYL62	1.28E-21	0.532771	0.715	0.769	3.61E-17	Platelets	MYL6
ISG151	2.13E-21	0.853007	0.275	0.208	5.98E-17	Platelets	ISG15
TCF41	9.02E-21	0.86736	0.332	0.28	2.54E-16	Platelets	TCF4
ENG1	1.10E-20	1.047463	0.277	0.23	3.09E-16	Platelets	ENG
CST31	5.57E-20	0.419286	0.802	0.699	1.57E-15	Platelets	CST3
AC004448.	3.16E-19	0.496041	0.272	0.199	8.89E-15	Platelets	AC004448.2
BSG	5.78E-19	1.052824	0.376	0.348	1.63E-14	Platelets	BSG
TPM42	7.40E-19	0.733067	0.411	0.374	2.08E-14	Platelets	TPM4
MTRNR2L1	2.92E-18	0.362434	0.349	0.506	8.21E-14	Platelets	MTRNR2L12
YBX31	7.52E-18	0.594683	0.37	0.321	2.12E-13	Platelets	YBX3
MT-ND51	3.71E-17	0.425424	0.762	0.848	1.04E-12	Platelets	MT-ND5
DYNLL11	2.26E-16	0.781843	0.393	0.367	6.37E-12	Platelets	DYNLL1
FKBP1A1	4.28E-16	0.948019	0.314	0.276	1.20E-11	Platelets	FKBP1A
MIF2	1.94E-15	0.594973	0.53	0.567	5.46E-11	Platelets	MIF
ZFP36L12	1.54E-13	0.339768	0.772	0.796	4.32E-09	Platelets	ZFP36L1
HMGN21	8.07E-13	0.643617	0.485	0.491	2.27E-08	Platelets	HMGN2
NME22	2.03E-12	0.452298	0.576	0.594	5.71E-08	Platelets	NME2
TXN1	2.58E-12	0.620176	0.265	0.219	7.26E-08	Platelets	TXN
PEA152	3.35E-11	0.549333	0.278	0.233	9.44E-07	Platelets	PEA15
HSBP12	5.45E-10	0.559188	0.273	0.237	1.53E-05	Platelets	HSBP1
PSAP2	1.34E-09	0.271661	0.778	0.768	3.77E-05	Platelets	PSAP
H2AFZ1	1.39E-09	0.543152	0.471	0.481	3.91E-05	Platelets	H2AFZ
PPIA1	1.50E-09	0.280237	0.735	0.8	4.22E-05	Platelets	PPIA
CALR1	1.76E-09	0.443662	0.532	0.554	4.95E-05	Platelets	CALR
HIST1H4C1	1.26E-08	0.612399	0.358	0.343	0.000356	Platelets	HIST1H4C
MT-ATP81	5.01E-08	0.35255	0.716	0.788	0.00141	Platelets	MT-ATP8

LAPTM4A1	6.00E-08	0.61603	0.411	0.429	0.001689	Platelets	LAPTM4A
PTTG1IP2	9.15E-08	0.559655	0.331	0.317	0.002573	Platelets	PTTG1IP
STX17-AS1:	1.01E-07	0.376595	0.244	0.342	0.002837	Platelets	STX17-AS1
TUBA1A2	3.22E-07	0.837062	0.329	0.321	0.009055	Platelets	TUBA1A
CD631	3.69E-07	0.466326	0.513	0.553	0.010385	Platelets	CD63
OLFML32	4.97E-07	0.281813	0.227	0.314	0.013983	Platelets	OLFML3
YWHAH2	6.73E-07	0.445515	0.507	0.525	0.018945	Platelets	YWHAH
HSP90B11	7.17E-07	0.413245	0.521	0.559	0.020162	Platelets	HSP90B1
GSTP12	1.02E-06	0.416545	0.493	0.523	0.028792	Platelets	GSTP1
BEST12	1.68E-06	0.254581	0.215	0.29	0.047359	Platelets	BEST1
SERPINB61	3.03E-06	0.549438	0.33	0.316	0.085247	Platelets	SERPINB6
UQCRQ1	5.48E-06	0.506981	0.338	0.332	0.15432	Platelets	UQCRQ
AP2M1	9.99E-06	0.533218	0.32	0.311	0.281211	Platelets	AP2M1
ATP5PF1	1.68E-05	0.537723	0.354	0.355	0.472278	Platelets	ATP5PF
TUBA1C1	3.46E-05	0.255435	0.215	0.278	0.973374	Platelets	TUBA1C
TACC1	3.72E-05	0.7492	0.365	0.381	1	Platelets	TACC1
GSN1	3.77E-05	0.452673	0.58	0.591	1	Platelets	GSN
PNP	5.00E-05	0.255155	0.205	0.267	1	Platelets	PNP
CAMTA1	7.75E-05	0.410317	0.256	0.236	1	Platelets	CAMTA1
TPI11	0.000127	0.433597	0.515	0.579	1	Platelets	TPI1
COX7B1	0.000254	0.255662	0.317	0.395	1	Platelets	COX7B
ITGB11	0.000296	0.560169	0.342	0.356	1	Platelets	ITGB1
PKM1	0.000315	0.534007	0.4	0.426	1	Platelets	PKM
YBX11	0.000441	0.384766	0.563	0.635	1	Platelets	YBX1
NUCB11	0.000454	0.448934	0.311	0.306	1	Platelets	NUCB1
RHOA1	0.000589	0.276588	0.633	0.726	1	Platelets	RHOA
POLR2L1	0.000847	0.477876	0.314	0.313	1	Platelets	POLR2L
CCDC85B1	0.000971	0.588036	0.287	0.288	1	Platelets	CCDC85B
ELOB1	0.000973	0.487403	0.418	0.459	1	Platelets	ELOB
POMP	0.001081	0.618407	0.416	0.461	1	Platelets	POMP
DAD1	0.001161	0.252814	0.34	0.433	1	Platelets	DAD1
S100A101	0.001508	0.408248	0.25	0.231	1	Platelets	S100A10
CDC37	0.001658	0.267051	0.349	0.435	1	Platelets	CDC37
BLOC1S11	0.001722	0.26222	0.285	0.35	1	Platelets	BLOC1S1
VDAC1	0.002031	0.261796	0.229	0.278	1	Platelets	VDAC1
PSMB1	0.002511	0.254222	0.321	0.394	1	Platelets	PSMB1
MDH2	0.003125	0.253286	0.261	0.316	1	Platelets	MDH2
TALDO11	0.004522	0.297484	0.271	0.329	1	Platelets	TALDO1
SRI	0.005686	0.285529	0.246	0.3	1	Platelets	SRI
SNX31	0.006419	0.497431	0.405	0.442	1	Platelets	SNX3
SLC25A51	0.007948	0.286266	0.424	0.528	1	Platelets	SLC25A5
NDUFC1	0.008337	0.363883	0.258	0.25	1	Platelets	NDUFC1
PSMA6	0.008555	0.300278	0.262	0.314	1	Platelets	PSMA6
JCHAIN	0	4.105787	0.309	0.003	0	Naive B-Ce	JCHAIN
CD79A	0	3.846551	0.793	0.005	0	Naive B-Ce	CD79A
IGKC	0	3.804727	0.301	0.009	0	Naive B-Ce	IGKC
IGHM	0	3.722289	0.542	0.003	0	Naive B-Ce	IGHM

MS4A1	0	2.913447	0.651	0.007	0	Naive B-Ce	MS4A1
AFF3	0	2.810299	0.655	0.052	0	Naive B-Ce	AFF3
BANK1	0	2.737686	0.641	0.011	0	Naive B-Ce	BANK1
BACH2	0	2.726614	0.601	0.08	0	Naive B-Ce	BACH2
FCRL1	0	2.462315	0.501	0.001	0	Naive B-Ce	FCRL1
TCL1A	0	2.121172	0.368	0.002	0	Naive B-Ce	TCL1A
FCMR1	0	1.968095	0.6	0.113	0	Naive B-Ce	FCMR
LINC00926	0	1.966075	0.436	0.006	0	Naive B-Ce	LINC00926
P2RX5	0	1.87297	0.461	0.024	0	Naive B-Ce	P2RX5
LTB1	0	1.863759	0.577	0.114	0	Naive B-Ce	LTB
CD521	0	1.835025	0.724	0.202	0	Naive B-Ce	CD52
CD79B	0	1.834377	0.456	0.017	0	Naive B-Ce	CD79B
BLK	0	1.824696	0.439	0.007	0	Naive B-Ce	BLK
CCR7	0	1.732837	0.403	0.041	0	Naive B-Ce	CCR7
RALGPS2	0	1.720117	0.432	0.058	0	Naive B-Ce	RALGPS2
BIRC3	0	1.664487	0.433	0.081	0	Naive B-Ce	BIRC3
LY9	0	1.600561	0.379	0.046	0	Naive B-Ce	LY9
NIBAN3	0	1.581573	0.364	0.003	0	Naive B-Ce	NIBAN3
PLEKHG1	0	1.562257	0.361	0.015	0	Naive B-Ce	PLEKHG1
FCER2	0	1.530524	0.313	0	0	Naive B-Ce	FCER2
IGHD	0	1.474991	0.281	0	0	Naive B-Ce	IGHD
VPREB3	0	1.440534	0.303	0	0	Naive B-Ce	VPREB3
TLE1	0	1.439426	0.365	0.025	0	Naive B-Ce	TLE1
CXCR5	0	1.420958	0.303	0.003	0	Naive B-Ce	CXCR5
EBF1	0	1.374092	0.302	0.003	0	Naive B-Ce	EBF1
OSBPL10	0	1.280046	0.308	0.009	0	Naive B-Ce	OSBPL10
CD19	0	1.279966	0.306	0.008	0	Naive B-Ce	CD19
TNFRSF13C	0	1.270956	0.345	0.05	0	Naive B-Ce	TNFRSF13C
POU2AF1	0	1.258998	0.308	0.001	0	Naive B-Ce	POU2AF1
FCRLA	0	1.226986	0.288	0.001	0	Naive B-Ce	FCRLA
SPIB	0	1.206605	0.273	0.02	0	Naive B-Ce	SPIB
AC119396.	0	1.197432	0.287	0.027	0	Naive B-Ce	AC119396.1
PAX5	0	1.172469	0.254	0	0	Naive B-Ce	PAX5
CD22	0	1.109552	0.265	0.008	0	Naive B-Ce	CD22
CD37	2.59E-237	2.22932	0.833	0.642	7.29E-233	Naive B-Ce	CD37
RPL18A1	6.36E-209	1.317125	0.944	0.889	1.79E-204	Naive B-Ce	RPL18A
RPS51	6.82E-204	1.327397	0.902	0.786	1.92E-199	Naive B-Ce	RPS5
ISG201	1.04E-202	1.38654	0.623	0.226	2.93E-198	Naive B-Ce	ISG20
RPS231	2.56E-191	1.065909	0.961	0.911	7.21E-187	Naive B-Ce	RPS23
HVCN1	5.09E-191	1.335363	0.406	0.112	1.43E-186	Naive B-Ce	HVCN1
EZR1	2.92E-190	1.658076	0.762	0.437	8.22E-186	Naive B-Ce	EZR
RIPOR21	2.24E-187	1.267975	0.452	0.129	6.30E-183	Naive B-Ce	RIPOR2
TPD52	6.02E-181	1.128256	0.363	0.091	1.70E-176	Naive B-Ce	TPD52
RPL81	2.56E-179	1.145537	0.93	0.861	7.21E-175	Naive B-Ce	RPL8
RPS81	8.03E-179	1.002939	0.959	0.917	2.26E-174	Naive B-Ce	RPS8
SEL1L3	5.17E-178	1.142049	0.334	0.078	1.46E-173	Naive B-Ce	SEL1L3
RBM381	9.23E-176	1.550965	0.52	0.187	2.60E-171	Naive B-Ce	RBM38

IFT57	5.48E-171	0.982196	0.267	0.054	1.54E-166	Naive B-Ce IFT57
RPL191	5.46E-168	0.94458	0.961	0.909	1.54E-163	Naive B-Ce RPL19
RPL321	3.09E-164	0.996004	0.946	0.913	8.70E-160	Naive B-Ce RPL32
CD551	2.75E-160	1.710444	0.681	0.364	7.74E-156	Naive B-Ce CD55
RPL131	3.37E-160	0.872337	0.975	0.938	9.49E-156	Naive B-Ce RPL13
RPL111	1.28E-156	0.992032	0.946	0.906	3.61E-152	Naive B-Ce RPL11
RPL181	1.47E-153	0.942706	0.929	0.878	4.13E-149	Naive B-Ce RPL18
RPL341	5.97E-150	0.941732	0.944	0.886	1.68E-145	Naive B-Ce RPL34
RPS271	1.20E-149	0.990562	0.952	0.891	3.37E-145	Naive B-Ce RPS27
RPS3A1	1.49E-146	0.920264	0.944	0.882	4.18E-142	Naive B-Ce RPS3A
TXNIP1	2.07E-141	1.472822	0.734	0.428	5.83E-137	Naive B-Ce TXNIP
RPS27A1	4.13E-141	0.911558	0.946	0.893	1.16E-136	Naive B-Ce RPS27A
PRKCB1	9.31E-139	1.345926	0.582	0.269	2.62E-134	Naive B-Ce PRKCB
RPL301	3.14E-138	0.939431	0.95	0.903	8.84E-134	Naive B-Ce RPL30
RASGRP2	5.81E-138	1.068578	0.363	0.109	1.63E-133	Naive B-Ce RASGRP2
RPSA1	4.63E-137	1.112182	0.854	0.744	1.30E-132	Naive B-Ce RPSA
RPS131	7.54E-137	0.865633	0.938	0.887	2.12E-132	Naive B-Ce RPS13
BTG11	9.46E-137	1.365122	0.886	0.798	2.66E-132	Naive B-Ce BTG1
EEF1A11	6.69E-134	0.809199	0.979	0.961	1.88E-129	Naive B-Ce EEF1A1
RPL391	4.48E-133	0.933985	0.933	0.887	1.26E-128	Naive B-Ce RPL39
PIM2	8.24E-133	1.108508	0.333	0.095	2.32E-128	Naive B-Ce PIM2
RPS22	1.60E-130	0.994216	0.913	0.856	4.50E-126	Naive B-Ce RPS2
PLPP5	5.57E-130	1.1304	0.295	0.08	1.57E-125	Naive B-Ce PLPP5
RPL101	8.47E-129	0.791463	0.987	0.947	2.38E-124	Naive B-Ce RPL10
RPS181	2.52E-127	0.830316	0.963	0.91	7.10E-123	Naive B-Ce RPS18
RPS121	1.02E-125	0.83154	0.966	0.921	2.87E-121	Naive B-Ce RPS12
RPL291	1.73E-124	0.828407	0.932	0.883	4.86E-120	Naive B-Ce RPL29
RPL121	5.56E-122	0.84481	0.95	0.896	1.56E-117	Naive B-Ce RPL12
RUBCNL	8.78E-121	1.08657	0.324	0.099	2.47E-116	Naive B-Ce RUBCNL
FAU1	1.27E-119	0.810133	0.933	0.883	3.57E-115	Naive B-Ce FAU
RPL212	6.65E-118	1.101688	0.867	0.758	1.87E-113	Naive B-Ce RPL21
RPS71	5.42E-116	0.803872	0.923	0.871	1.52E-111	Naive B-Ce RPS7
RPS281	9.87E-116	0.80946	0.934	0.886	2.78E-111	Naive B-Ce RPS28
RPS151	5.56E-113	0.741401	0.945	0.899	1.56E-108	Naive B-Ce RPS15
RPS4X2	3.26E-110	0.7919	0.93	0.865	9.18E-106	Naive B-Ce RPS4X
RPL35A1	1.70E-109	0.8246	0.898	0.835	4.77E-105	Naive B-Ce RPL35A
CD40	2.52E-109	0.934785	0.287	0.086	7.08E-105	Naive B-Ce CD40
RPL7A1	1.31E-108	0.741272	0.938	0.878	3.70E-104	Naive B-Ce RPL7A
RPS15A1	1.14E-107	0.707817	0.943	0.897	3.21E-103	Naive B-Ce RPS15A
RACK11	3.72E-107	0.802471	0.883	0.826	1.05E-102	Naive B-Ce RACK1
RPL51	3.73E-107	0.827166	0.895	0.838	1.05E-102	Naive B-Ce RPL5
RPLP02	5.50E-107	0.746284	0.938	0.871	1.55E-102	Naive B-Ce RPLP0
RPS61	4.07E-106	0.751478	0.925	0.883	1.14E-101	Naive B-Ce RPS6
RPS211	5.70E-103	0.863799	0.877	0.82	1.60E-98	Naive B-Ce RPS21
RPL23A1	1.10E-102	0.815533	0.881	0.798	3.09E-98	Naive B-Ce RPL23A
RPL33	4.47E-102	0.758856	0.93	0.873	1.26E-97	Naive B-Ce RPL3
RPL281	6.41E-102	0.679951	0.951	0.925	1.80E-97	Naive B-Ce RPL28

RPLP21	7.94E-102	0.798355	0.895	0.851	2.24E-97	Naive B-Ce RPLP2
RPL221	9.47E-102	0.885858	0.84	0.76	2.67E-97	Naive B-Ce RPL22
RPL10A1	1.83E-101	0.894463	0.863	0.777	5.16E-97	Naive B-Ce RPL10A
RPL412	1.68E-99	1.142821	0.896	0.833	4.73E-95	Naive B-Ce RPL41
RPL91	2.09E-99	0.831183	0.862	0.798	5.88E-95	Naive B-Ce RPL9
RPS91	1.12E-98	0.712823	0.92	0.857	3.16E-94	Naive B-Ce RPS9
RPS31	3.51E-98	0.644782	0.954	0.889	9.88E-94	Naive B-Ce RPS3
TAGLN23	1.14E-96	1.242215	0.614	0.369	3.20E-92	Naive B-Ce TAGLN2
EEF21	5.71E-96	0.884549	0.85	0.757	1.61E-91	Naive B-Ce EEF2
RPL371	5.73E-96	0.767138	0.915	0.873	1.61E-91	Naive B-Ce RPL37
EEF1G	6.83E-96	0.880953	0.813	0.69	1.92E-91	Naive B-Ce EEF1G
LIMD21	6.97E-96	1.200066	0.611	0.37	1.96E-91	Naive B-Ce LIMD2
RPL13A1	5.78E-95	0.856535	0.842	0.752	1.63E-90	Naive B-Ce RPL13A
SELL	6.77E-94	1.169313	0.416	0.177	1.91E-89	Naive B-Ce SELL
JUND1	9.66E-90	1.042634	0.852	0.794	2.72E-85	Naive B-Ce JUND
CD72	8.56E-89	0.892752	0.252	0.078	2.41E-84	Naive B-Ce CD72
RPS251	1.26E-87	0.694068	0.905	0.857	3.54E-83	Naive B-Ce RPS25
RPL261	1.32E-87	0.629533	0.938	0.892	3.72E-83	Naive B-Ce RPL26
RPL15	1.22E-86	0.680269	0.899	0.853	3.42E-82	Naive B-Ce RPL15
TENT5C2	6.81E-86	0.94086	0.409	0.168	1.92E-81	Naive B-Ce TENT5C
SMCHD11	6.94E-86	1.00973	0.614	0.366	1.95E-81	Naive B-Ce SMCHD1
RPL61	1.17E-84	0.678211	0.919	0.865	3.28E-80	Naive B-Ce RPL6
EEF1B21	1.17E-84	0.790829	0.857	0.759	3.28E-80	Naive B-Ce EEF1B2
RPS191	1.32E-82	0.580525	0.952	0.925	3.72E-78	Naive B-Ce RPS19
SNHG71	4.24E-80	1.067519	0.465	0.236	1.19E-75	Naive B-Ce SNHG7
RPL141	7.75E-80	0.662201	0.898	0.863	2.18E-75	Naive B-Ce RPL14
RPLP11	1.23E-79	0.528141	0.979	0.95	3.45E-75	Naive B-Ce RPLP1
LAPTM51	1.70E-76	0.689454	0.893	0.849	4.79E-72	Naive B-Ce LAPTM5
CNN21	1.02E-75	0.716327	0.374	0.15	2.88E-71	Naive B-Ce CNN2
RPL361	4.13E-75	0.647871	0.878	0.842	1.16E-70	Naive B-Ce RPL36
ZCCHC7	2.72E-74	1.050597	0.379	0.17	7.64E-70	Naive B-Ce ZCCHC7
NOP531	5.88E-74	1.000315	0.632	0.454	1.65E-69	Naive B-Ce NOP53
RHOH1	1.79E-73	1.025028	0.533	0.303	5.04E-69	Naive B-Ce RHOH
EEF1D1	2.56E-73	0.812507	0.797	0.72	7.20E-69	Naive B-Ce EEF1D
RPS141	6.06E-73	0.582686	0.945	0.892	1.71E-68	Naive B-Ce RPS14
PRDM2	3.90E-71	1.008737	0.41	0.201	1.10E-66	Naive B-Ce PRDM2
TLE51	2.54E-70	0.990161	0.542	0.315	7.14E-66	Naive B-Ce TLE5
CD691	1.07E-69	1.499565	0.713	0.559	3.01E-65	Naive B-Ce CD69
IQSEC1	1.35E-69	0.701943	0.254	0.088	3.79E-65	Naive B-Ce IQSEC1
RPL171	2.45E-69	0.585209	0.911	0.842	6.90E-65	Naive B-Ce RPL17
RPS11	1.15E-67	0.804013	0.763	0.676	3.23E-63	Naive B-Ce RPS11
RPS161	3.17E-67	0.608849	0.889	0.828	8.93E-63	Naive B-Ce RPS16
HLA-DQA1:	8.21E-67	0.779124	0.73	0.505	2.31E-62	Naive B-Ce HLA-DQA1
ANXA61	2.39E-66	0.638789	0.304	0.116	6.72E-62	Naive B-Ce ANXA6
TSC22D31	4.94E-66	0.892079	0.83	0.744	1.39E-61	Naive B-Ce TSC22D3
MGAT5	4.49E-64	0.999026	0.501	0.29	1.26E-59	Naive B-Ce MGAT5
RCSD1	1.76E-63	0.985346	0.607	0.424	4.95E-59	Naive B-Ce RCSD1

IL2RG1	3.78E-63	0.596704	0.438	0.198	1.06E-58	Naive B-Ce IL2RG
EIF2AK3	1.06E-62	0.948604	0.342	0.155	2.99E-58	Naive B-Ce EIF2AK3
PTPRCAP1	1.06E-61	0.744531	0.317	0.126	2.99E-57	Naive B-Ce PTPRCAP
NCF1	1.40E-61	0.939417	0.509	0.311	3.94E-57	Naive B-Ce NCF1
PTMA2	2.82E-61	0.555652	0.941	0.913	7.93E-57	Naive B-Ce PTMA
FAM3C	4.58E-61	0.876969	0.287	0.119	1.29E-56	Naive B-Ce FAM3C
CXCR41	1.83E-60	0.709683	0.763	0.544	5.15E-56	Naive B-Ce CXCR4
HLA-DQA2:	3.86E-60	0.961272	0.357	0.169	1.09E-55	Naive B-Ce HLA-DQA2
TSTD11	5.53E-59	0.75451	0.323	0.143	1.56E-54	Naive B-Ce TSTD1
SIPA1L3	3.05E-58	0.795194	0.251	0.096	8.58E-54	Naive B-Ce SIPA1L3
CD741	1.11E-57	0.453809	0.993	0.931	3.12E-53	Naive B-Ce CD74
SLC38A11	1.62E-56	0.495363	0.337	0.143	4.54E-52	Naive B-Ce SLC38A1
CYFIP21	4.18E-56	0.555367	0.261	0.099	1.18E-51	Naive B-Ce CYFIP2
MARCKSL1	1.15E-55	0.814078	0.278	0.116	3.22E-51	Naive B-Ce MARCKSL1
CD481	7.94E-55	0.652175	0.362	0.167	2.23E-50	Naive B-Ce CD48
RPL241	2.01E-54	0.540869	0.881	0.822	5.65E-50	Naive B-Ce RPL24
IL4R	2.07E-53	1.111114	0.344	0.178	5.82E-49	Naive B-Ce IL4R
RAC22	2.06E-52	0.581549	0.513	0.285	5.80E-48	Naive B-Ce RAC2
ARRDC2	3.20E-52	0.957892	0.356	0.187	9.01E-48	Naive B-Ce ARRDC2
ADK	4.84E-52	1.101225	0.434	0.256	1.36E-47	Naive B-Ce ADK
RPL351	1.48E-51	0.579985	0.85	0.807	4.16E-47	Naive B-Ce RPL35
YBX32	8.90E-51	1.408432	0.472	0.321	2.51E-46	Naive B-Ce YBX3
PPDPF1	1.25E-50	0.631792	0.602	0.384	3.51E-46	Naive B-Ce PPDPF
SEPTIN91	5.94E-50	0.628679	0.402	0.204	1.67E-45	Naive B-Ce SEPTIN9
IRF81	1.41E-49	0.896202	0.504	0.332	3.98E-45	Naive B-Ce IRF8
TMEM243	2.36E-47	0.835756	0.393	0.225	6.64E-43	Naive B-Ce TMEM243
YWHAZ1	3.00E-47	0.694896	0.725	0.627	8.43E-43	Naive B-Ce YWHAZ
SNX21	1.26E-46	0.863296	0.47	0.307	3.55E-42	Naive B-Ce SNX2
MTRNR2L1	7.57E-46	0.976788	0.674	0.502	2.13E-41	Naive B-Ce MTRNR2L12
SH3BP5	1.48E-45	0.652906	0.269	0.122	4.17E-41	Naive B-Ce SH3BP5
STRBP	4.85E-45	0.635094	0.256	0.113	1.37E-40	Naive B-Ce STRBP
EIF11	5.50E-45	0.497224	0.931	0.919	1.55E-40	Naive B-Ce EIF1
ICAM31	1.20E-44	0.521495	0.299	0.136	3.37E-40	Naive B-Ce ICAM3
NACA1	3.07E-44	0.491956	0.865	0.835	8.63E-40	Naive B-Ce NACA
LSM71	3.99E-44	0.884228	0.477	0.32	1.12E-39	Naive B-Ce LSM7
HINT12	4.63E-43	0.735101	0.618	0.492	1.30E-38	Naive B-Ce HINT1
HLA-DPB11	9.47E-43	0.485573	0.822	0.688	2.67E-38	Naive B-Ce HLA-DPB1
UBA521	2.60E-42	0.489733	0.819	0.78	7.31E-38	Naive B-Ce UBA52
RPL37A1	3.56E-42	0.529816	0.822	0.78	1.00E-37	Naive B-Ce RPL37A
RPL42	9.35E-42	0.618017	0.778	0.731	2.63E-37	Naive B-Ce RPL4
GNG71	1.21E-41	0.774096	0.488	0.329	3.41E-37	Naive B-Ce GNG7
CHPT1	1.34E-41	0.744516	0.322	0.17	3.76E-37	Naive B-Ce CHPT1
HLA-DRA1	3.41E-41	0.368067	0.934	0.755	9.59E-37	Naive B-Ce HLA-DRA
SMIM14	1.45E-39	0.735149	0.258	0.125	4.07E-35	Naive B-Ce SMIM14
ORAI2	1.47E-39	0.615933	0.256	0.122	4.12E-35	Naive B-Ce ORAI2
CYTIP1	5.29E-39	0.59863	0.487	0.303	1.49E-34	Naive B-Ce CYTIP
RPL271	3.73E-38	0.560874	0.773	0.717	1.05E-33	Naive B-Ce RPL27

HERPUD11	5.36E-38	0.730247	0.728	0.644	1.51E-33	Naive B-Ce	HERPUD1
PFDN51	1.28E-36	0.543008	0.774	0.731	3.61E-32	Naive B-Ce	PFDN5
RPL71	1.55E-36	0.608295	0.698	0.619	4.35E-32	Naive B-Ce	RPL7
TNFAIP81	9.30E-36	0.652985	0.343	0.191	2.62E-31	Naive B-Ce	TNFAIP8
SLC25A6	2.62E-35	0.614031	0.696	0.639	7.39E-31	Naive B-Ce	SLC25A6
RPS241	3.08E-35	0.36645	0.932	0.904	8.67E-31	Naive B-Ce	RPS24
TBC1D10C1	6.53E-35	0.491661	0.282	0.138	1.84E-30	Naive B-Ce	TBC1D10C
SRPK21	3.27E-34	0.630971	0.362	0.212	9.19E-30	Naive B-Ce	SRPK2
TRBC21	4.01E-34	0.310223	0.276	0.129	1.13E-29	Naive B-Ce	TRBC2
CD831	2.97E-33	0.585687	0.889	0.738	8.37E-29	Naive B-Ce	CD83
MKNK21	8.25E-33	0.74019	0.385	0.249	2.32E-28	Naive B-Ce	MKNK2
SP1401	5.55E-32	0.679487	0.347	0.206	1.56E-27	Naive B-Ce	SP140
PIM3	1.40E-31	0.775526	0.283	0.155	3.95E-27	Naive B-Ce	PIM3
UBALD21	3.98E-31	0.871575	0.445	0.317	1.12E-26	Naive B-Ce	UBALD2
YPEL51	4.31E-31	0.732633	0.528	0.402	1.21E-26	Naive B-Ce	YPEL5
NSD31	6.27E-30	0.601189	0.416	0.276	1.76E-25	Naive B-Ce	NSD3
PDE7A1	1.69E-29	0.506291	0.349	0.203	4.75E-25	Naive B-Ce	PDE7A
RPS291	2.36E-29	0.519031	0.745	0.686	6.63E-25	Naive B-Ce	RPS29
TMEM1231	4.08E-29	0.766155	0.392	0.265	1.15E-24	Naive B-Ce	TMEM123
ATF7IP1	2.86E-28	0.7721	0.439	0.313	8.04E-24	Naive B-Ce	ATF7IP
CCNI1	4.32E-28	0.608971	0.649	0.593	1.22E-23	Naive B-Ce	CCNI
FAM107B1	3.22E-27	0.536167	0.359	0.222	9.05E-23	Naive B-Ce	FAM107B
USP12	1.64E-26	0.606706	0.251	0.137	4.61E-22	Naive B-Ce	USP12
ITPR1	1.72E-26	0.755009	0.352	0.229	4.83E-22	Naive B-Ce	ITPR1
PABPC11	2.08E-26	0.383463	0.829	0.827	5.84E-22	Naive B-Ce	PABPC1
AC007384.	3.21E-26	0.709343	0.314	0.191	9.03E-22	Naive B-Ce	AC007384.1
PRKCE	5.61E-26	0.687355	0.3	0.18	1.58E-21	Naive B-Ce	PRKCE
11-Mar	7.47E-26	0.696407	0.342	0.218	2.10E-21	Naive B-Ce	1-Mar
PPP3CC1	3.44E-25	0.504639	0.26	0.145	9.69E-21	Naive B-Ce	PPP3CC
MTSS11	4.52E-25	0.595073	0.379	0.251	1.27E-20	Naive B-Ce	MTSS1
PPM1K	2.15E-24	0.575587	0.281	0.168	6.04E-20	Naive B-Ce	PPM1K
SYPL1	2.98E-24	0.63372	0.292	0.181	8.38E-20	Naive B-Ce	SYPL1
DUSP51	3.12E-24	0.654177	0.308	0.187	8.78E-20	Naive B-Ce	DUSP5
CLEC2D1	1.81E-23	0.461739	0.255	0.141	5.10E-19	Naive B-Ce	CLEC2D
IL161	3.18E-23	0.649398	0.343	0.228	8.95E-19	Naive B-Ce	IL16
SEPTIN11	1.07E-22	0.294732	0.267	0.145	3.01E-18	Naive B-Ce	SEPTIN1
HLA-DQB11	2.40E-22	0.354923	0.739	0.587	6.76E-18	Naive B-Ce	HLA-DQB1
ERP292	2.72E-22	0.575189	0.505	0.409	7.65E-18	Naive B-Ce	ERP29
UCP22	3.34E-22	0.57537	0.471	0.363	9.40E-18	Naive B-Ce	UCP2
TNFRSF141	8.90E-22	0.4839	0.287	0.177	2.51E-17	Naive B-Ce	TNFRSF14
CCND31	5.19E-21	0.582265	0.512	0.418	1.46E-16	Naive B-Ce	CCND3
CAMK2D	5.86E-21	0.702627	0.382	0.274	1.65E-16	Naive B-Ce	CAMK2D
HNRNPA11	9.68E-21	0.443338	0.717	0.683	2.72E-16	Naive B-Ce	HNRNPA1
FOXP11	1.79E-20	0.58761	0.56	0.477	5.03E-16	Naive B-Ce	FOXP1
LYN1	2.08E-20	0.486482	0.614	0.518	5.84E-16	Naive B-Ce	LYN
SF11	2.62E-20	0.492338	0.618	0.542	7.38E-16	Naive B-Ce	SF1
CD471	2.85E-20	0.540454	0.342	0.233	8.02E-16	Naive B-Ce	CD47

SLC44A2	2.93E-20	0.526623	0.295	0.189	8.25E-16	Naive B-Ce SLC44A2
SMAP21	9.03E-20	0.438051	0.731	0.71	2.54E-15	Naive B-Ce SMAP2
SMARCB1	1.41E-19	0.566721	0.252	0.156	3.97E-15	Naive B-Ce SMARCB1
SP1001	1.44E-19	0.526919	0.593	0.523	4.05E-15	Naive B-Ce SP100
RFTN11	2.26E-19	0.53989	0.266	0.163	6.37E-15	Naive B-Ce RFTN1
EMP32	2.63E-19	0.620495	0.446	0.336	7.40E-15	Naive B-Ce EMP3
LBH1	6.27E-19	0.480723	0.256	0.152	1.77E-14	Naive B-Ce LBH
ANKRD44	1.51E-18	0.561057	0.614	0.555	4.26E-14	Naive B-Ce ANKRD44
SYNGR21	1.96E-18	0.505675	0.537	0.451	5.52E-14	Naive B-Ce SYNGR2
LSP12	2.37E-18	0.340442	0.516	0.385	6.68E-14	Naive B-Ce LSP1
VPS37B1	2.65E-18	0.503024	0.402	0.3	7.47E-14	Naive B-Ce VPS37B
SNX91	3.65E-18	0.556426	0.555	0.467	1.03E-13	Naive B-Ce SNX9
CD441	3.98E-18	0.27138	0.393	0.26	1.12E-13	Naive B-Ce CD44
NFKB2	2.48E-17	0.533794	0.254	0.163	6.98E-13	Naive B-Ce NFKB2
ANAPC161	3.76E-17	0.530159	0.488	0.408	1.06E-12	Naive B-Ce ANAPC16
OSER1	4.28E-17	0.555534	0.263	0.173	1.21E-12	Naive B-Ce OSER1
SLC2A31	6.03E-17	0.416915	0.536	0.433	1.70E-12	Naive B-Ce SLC2A3
RPL36A1	7.34E-17	0.352589	0.775	0.761	2.07E-12	Naive B-Ce RPL36A
GAS51	1.35E-16	0.505237	0.504	0.432	3.79E-12	Naive B-Ce GAS5
PPP1CC	3.51E-16	0.443997	0.294	0.203	9.86E-12	Naive B-Ce PPP1CC
SP140L	3.55E-16	0.521852	0.256	0.168	9.98E-12	Naive B-Ce SP140L
RPL381	5.05E-16	0.44278	0.679	0.655	1.42E-11	Naive B-Ce RPL38
RPL311	7.12E-16	0.383559	0.706	0.678	2.00E-11	Naive B-Ce RPL31
PLP21	9.45E-16	0.476533	0.267	0.174	2.66E-11	Naive B-Ce PLP2
PACS11	1.00E-15	0.542089	0.345	0.251	2.83E-11	Naive B-Ce PACS1
POU2F21	1.25E-15	0.57497	0.313	0.229	3.51E-11	Naive B-Ce POU2F2
BTF31	1.26E-15	0.325199	0.734	0.708	3.55E-11	Naive B-Ce BTF3
SET2	1.37E-15	0.52294	0.482	0.407	3.85E-11	Naive B-Ce SET
UVRAG1	1.86E-15	0.597159	0.407	0.326	5.22E-11	Naive B-Ce UVRAG
EIF3F1	1.96E-15	0.447109	0.561	0.508	5.50E-11	Naive B-Ce EIF3F
PLCG21	1.64E-14	0.534714	0.307	0.224	4.60E-10	Naive B-Ce PLCG2
JUNB1	1.80E-14	0.269491	0.913	0.883	5.06E-10	Naive B-Ce JUNB
MT-ATP6	1.88E-14	0.363226	0.833	0.839	5.28E-10	Naive B-Ce MT-ATP6
SELENOF	2.25E-14	0.535295	0.385	0.308	6.33E-10	Naive B-Ce SELENOF
RPS201	4.92E-14	0.500882	0.544	0.498	1.38E-09	Naive B-Ce RPS20
CALHM61	5.20E-14	0.496758	0.29	0.205	1.46E-09	Naive B-Ce CALHM6
SNHG291	5.66E-14	0.442647	0.594	0.548	1.59E-09	Naive B-Ce SNHG29
IRF11	6.70E-14	0.427877	0.331	0.237	1.88E-09	Naive B-Ce IRF1
SMDT11	7.15E-14	0.558561	0.429	0.361	2.01E-09	Naive B-Ce SMDT1
RPL27A1	7.45E-14	0.393784	0.625	0.589	2.10E-09	Naive B-Ce RPL27A
UXT1	1.09E-13	0.480236	0.407	0.339	3.05E-09	Naive B-Ce UXT
DENND4A1	1.47E-13	0.540453	0.392	0.311	4.13E-09	Naive B-Ce DENND4A
UTRN1	2.26E-13	0.454086	0.424	0.341	6.37E-09	Naive B-Ce UTRN
EIF3L	2.52E-13	0.485289	0.467	0.41	7.10E-09	Naive B-Ce EIF3L
EIF2S3	2.80E-13	0.505806	0.359	0.284	7.88E-09	Naive B-Ce EIF2S3
ADD1	2.90E-13	0.496481	0.314	0.236	8.16E-09	Naive B-Ce ADD1
ATP6V1G1	1.15E-12	0.438606	0.482	0.425	3.24E-08	Naive B-Ce ATP6V1G1

SP1101	1.26E-12	0.532082	0.385	0.312	3.55E-08	Naive B-Ce SP110
SNX32	1.59E-12	0.48227	0.489	0.441	4.47E-08	Naive B-Ce SNX3
SWAP701	1.73E-12	0.456289	0.384	0.309	4.88E-08	Naive B-Ce SWAP70
ARID5B	1.83E-12	0.423785	0.493	0.403	5.15E-08	Naive B-Ce ARID5B
TSPYL21	2.70E-12	0.371592	0.301	0.213	7.59E-08	Naive B-Ce TSPYL2
STK17A1	7.19E-12	0.433176	0.46	0.388	2.02E-07	Naive B-Ce STK17A
CRIP11	1.59E-11	0.290641	0.279	0.185	4.47E-07	Naive B-Ce CRIP1
ITSN2	6.28E-11	0.521448	0.393	0.334	1.77E-06	Naive B-Ce ITSN2
KDM4C1	6.58E-11	0.368118	0.296	0.22	1.85E-06	Naive B-Ce KDM4C
TOMM71	1.42E-10	0.349283	0.614	0.585	3.98E-06	Naive B-Ce TOMM7
GPSM31	1.46E-10	0.43313	0.486	0.439	4.09E-06	Naive B-Ce GPSM3
TUBA1A3	2.48E-10	0.525689	0.386	0.321	6.98E-06	Naive B-Ce TUBA1A
COX7C1	2.72E-10	0.329278	0.632	0.611	7.64E-06	Naive B-Ce COX7C
ALG13	2.90E-10	0.532647	0.306	0.241	8.17E-06	Naive B-Ce ALG13
TCF42	3.95E-10	0.446149	0.345	0.28	1.11E-05	Naive B-Ce TCF4
DRAM21	5.46E-10	0.493236	0.276	0.216	1.54E-05	Naive B-Ce DRAM2
SEC62	5.71E-10	0.435595	0.433	0.391	1.61E-05	Naive B-Ce SEC62
RELB	8.80E-10	0.44577	0.294	0.231	2.48E-05	Naive B-Ce RELB
PPP4R3A1	9.24E-10	0.369773	0.283	0.215	2.60E-05	Naive B-Ce PPP4R3A
EIF3H1	1.50E-09	0.352864	0.507	0.471	4.21E-05	Naive B-Ce EIF3H
LINC01619	1.63E-09	0.41461	0.286	0.216	4.58E-05	Naive B-Ce LINC01619
ZFAS11	2.12E-09	0.386276	0.603	0.587	5.96E-05	Naive B-Ce ZFAS1
PDE4B	2.26E-09	0.299993	0.55	0.482	6.35E-05	Naive B-Ce PDE4B
HNRNPK	5.06E-09	0.323328	0.597	0.579	0.000142	Naive B-Ce HNRNPK
FXYD51	5.30E-09	0.28203	0.63	0.591	0.000149	Naive B-Ce FXYD5
NUP58	6.25E-09	0.443427	0.274	0.215	0.000176	Naive B-Ce NUP58
MAPK1IP1l	6.62E-09	0.440081	0.364	0.309	0.000186	Naive B-Ce MAPK1IP1L
TAGAP1	1.11E-08	0.403445	0.542	0.504	0.000312	Naive B-Ce TAGAP
PHB2	1.13E-08	0.408965	0.374	0.319	0.000317	Naive B-Ce PHB2
SEPTIN71	1.26E-08	0.377215	0.437	0.385	0.000354	Naive B-Ce SEPTIN7
STX71	1.42E-08	0.432973	0.304	0.248	0.0004	Naive B-Ce STX7
SNHG8	1.44E-08	0.41253	0.458	0.418	0.000404	Naive B-Ce SNHG8
GRASP1	2.85E-08	0.39891	0.487	0.431	0.000803	Naive B-Ce GRASP
UBXN1	3.29E-08	0.425132	0.474	0.448	0.000926	Naive B-Ce UBXN1
CIRBP1	3.47E-08	0.301711	0.645	0.644	0.000978	Naive B-Ce CIRBP
RAB11A	4.53E-08	0.477734	0.377	0.336	0.001274	Naive B-Ce RAB11A
PARP11	5.14E-08	0.365205	0.313	0.258	0.001447	Naive B-Ce PARP1
TAPBP1	5.90E-08	0.372976	0.359	0.312	0.001661	Naive B-Ce TAPBP
CHMP1B	6.21E-08	0.742361	0.308	0.255	0.001747	Naive B-Ce CHMP1B
HNRNPA01	8.51E-08	0.366987	0.495	0.469	0.002395	Naive B-Ce HNRNPA0
ARF61	8.73E-08	0.380923	0.391	0.345	0.002456	Naive B-Ce ARF6
ASXL11	8.93E-08	0.393643	0.282	0.224	0.002512	Naive B-Ce ASXL1
ODC1	9.45E-08	0.349001	0.345	0.286	0.002659	Naive B-Ce ODC1
YBX12	1.33E-07	0.327133	0.644	0.634	0.003734	Naive B-Ce YBX1
P2RY81	1.61E-07	0.263484	0.281	0.218	0.00453	Naive B-Ce P2RY8
SNU131	1.76E-07	0.350474	0.404	0.358	0.004951	Naive B-Ce SNU13
PCBP1	2.84E-07	0.401759	0.43	0.4	0.00799	Naive B-Ce PCBP1

ELOVL51	3.15E-07	0.372515	0.333	0.288	0.00886	Naive B-Ce ELOVL5
KMT2E1	3.42E-07	0.352492	0.482	0.45	0.009636	Naive B-Ce KMT2E
LYST1	3.64E-07	0.372001	0.297	0.244	0.01024	Naive B-Ce LYST
JAK11	4.22E-07	0.326873	0.504	0.48	0.011879	Naive B-Ce JAK1
RPS171	4.47E-07	0.368714	0.493	0.466	0.012583	Naive B-Ce RPS17
TCEA1	4.66E-07	0.428917	0.348	0.306	0.013111	Naive B-Ce TCEA1
TAF1D1	5.36E-07	0.450059	0.383	0.347	0.015087	Naive B-Ce TAF1D
DAZAP21	6.00E-07	0.32509	0.577	0.574	0.016883	Naive B-Ce DAZAP2
LMBRD1	6.67E-07	0.374572	0.337	0.292	0.018764	Naive B-Ce LMBRD1
LAMTOR5	7.07E-07	0.405581	0.278	0.23	0.01988	Naive B-Ce LAMTOR5
ARID1B1	8.45E-07	0.394032	0.368	0.318	0.023785	Naive B-Ce ARID1B
RESF11	9.92E-07	0.273952	0.355	0.297	0.027923	Naive B-Ce RESF1
TUT41	1.08E-06	0.376064	0.297	0.245	0.030427	Naive B-Ce TUT4
CDK13	1.53E-06	0.392187	0.251	0.201	0.043038	Naive B-Ce CDK13
TANK	1.63E-06	0.328531	0.329	0.286	0.045786	Naive B-Ce TANK
ATP2B1	1.72E-06	0.341272	0.386	0.338	0.048436	Naive B-Ce ATP2B1
EIF3E1	2.15E-06	0.348789	0.443	0.427	0.060363	Naive B-Ce EIF3E
EHMT1	3.19E-06	0.333623	0.273	0.225	0.089873	Naive B-Ce EHMT1
PAPOLA	4.48E-06	0.364242	0.324	0.281	0.126096	Naive B-Ce PAPOLA
NCL2	5.35E-06	0.265114	0.6	0.599	0.150505	Naive B-Ce NCL
YPEL3	6.16E-06	0.325611	0.463	0.432	0.17346	Naive B-Ce YPEL3
PIK3IP11	6.73E-06	0.354807	0.329	0.286	0.18934	Naive B-Ce PIK3IP1
CYCS1	7.53E-06	0.439314	0.41	0.377	0.212015	Naive B-Ce CYCS
TAF71	1.36E-05	0.333122	0.272	0.229	0.384	Naive B-Ce TAF7
EIF4B1	1.60E-05	0.323994	0.403	0.375	0.449661	Naive B-Ce EIF4B
CSK1	1.68E-05	0.329521	0.349	0.317	0.4726	Naive B-Ce CSK
VAMP21	2.29E-05	0.29194	0.399	0.368	0.645597	Naive B-Ce VAMP2
COMMD61	4.11E-05	0.262567	0.562	0.562	1	Naive B-Ce COMMD6
BPTF1	4.99E-05	0.305175	0.377	0.349	1	Naive B-Ce BPTF
SYF21	6.64E-05	0.303298	0.283	0.247	1	Naive B-Ce SYF2
MTPN	6.99E-05	0.36796	0.333	0.309	1	Naive B-Ce MTPN
RBM8A1	8.32E-05	0.362887	0.407	0.394	1	Naive B-Ce RBM8A
PLEKHA21	8.40E-05	0.301152	0.273	0.235	1	Naive B-Ce PLEKHA2
FCHSD21	9.59E-05	0.277492	0.416	0.384	1	Naive B-Ce FCHSD2
CDC42SE1	9.98E-05	0.41756	0.392	0.376	1	Naive B-Ce CDC42SE1
AHNAK1	0.00013	0.303084	0.316	0.266	1	Naive B-Ce AHNAK
BLNK1	0.000141	0.344683	0.294	0.26	1	Naive B-Ce BLNK
SNHG61	0.000149	0.331635	0.464	0.451	1	Naive B-Ce SNHG6
PTPN61	0.000167	0.314928	0.376	0.358	1	Naive B-Ce PTPN6
SNX291	0.000182	0.380117	0.333	0.304	1	Naive B-Ce SNX29
BTBD9	0.000285	0.303779	0.251	0.215	1	Naive B-Ce BTBD9
EIF1B	0.000309	0.337334	0.337	0.316	1	Naive B-Ce EIF1B
UBE2J1	0.000321	0.348979	0.278	0.247	1	Naive B-Ce UBE2J1
DDX61	0.000357	0.282429	0.333	0.303	1	Naive B-Ce DDX6
GPBP11	0.000363	0.29973	0.327	0.299	1	Naive B-Ce GPBP1
EIF5A1	0.000426	0.311896	0.357	0.34	1	Naive B-Ce EIF5A
RBM31	0.000443	0.281265	0.473	0.473	1	Naive B-Ce RBM3

UQCRH	0.00055	0.313722	0.44	0.44	1	Naive B-Ce UQCRH
KRT101	0.000745	0.253781	0.335	0.311	1	Naive B-Ce KRT10
RPS4Y11	0.000985	0.367013	0.427	0.433	1	Naive B-Ce RPS4Y1
RSL24D11	0.001001	0.328528	0.286	0.263	1	Naive B-Ce RSL24D1
PMAIP11	0.001029	0.266184	0.311	0.28	1	Naive B-Ce PMAIP1
GTF3A1	0.001188	0.287678	0.329	0.305	1	Naive B-Ce GTF3A
TSPAN31	0.001259	0.301292	0.276	0.255	1	Naive B-Ce TSPAN3
TRIM221	0.001427	0.343648	0.395	0.385	1	Naive B-Ce TRIM22
ZBTB11	0.00148	0.325464	0.274	0.246	1	Naive B-Ce ZBTB1
RNPS1	0.001483	0.282745	0.315	0.296	1	Naive B-Ce RNPS1
KHDRBS12	0.001531	0.28501	0.378	0.367	1	Naive B-Ce KHDRBS1
NCOA3	0.001582	0.311141	0.316	0.294	1	Naive B-Ce NCOA3
WASHC41	0.001769	0.290816	0.282	0.259	1	Naive B-Ce WASHC4
BBX1	0.002062	0.324042	0.274	0.25	1	Naive B-Ce BBX
APPL1	0.00267	0.294703	0.28	0.259	1	Naive B-Ce APPL1
EIF4A21	0.002704	0.26875	0.485	0.501	1	Naive B-Ce EIF4A2
PTK2B	0.003316	0.259883	0.285	0.258	1	Naive B-Ce PTK2B
TRIO1	0.003482	0.308979	0.265	0.242	1	Naive B-Ce TRIO
RSRP11	0.004217	0.261705	0.453	0.448	1	Naive B-Ce RSRP1
PCBP2	0.005452	0.305345	0.378	0.379	1	Naive B-Ce PCBP2
RAD212	0.009949	0.263305	0.276	0.256	1	Naive B-Ce RAD21

Supplementary Table 4: DEGs for Figure 2D

	p_val	avg_log2FC	pct.1	pct.2	p_val_adj	cluster	gene
MT2A	0	1.475991	0.534	0.335	0	MG-Inflam	MT2A
CCL3L1	0	1.337038	0.918	0.675	0	MG-Inflam	CCL3L1
CCL4L2	0	1.226513	0.935	0.732	0	MG-Inflam	CCL4L2
IL1A	0	1.222393	0.474	0.163	0	MG-Inflam	IL1A
CCL3	0	1.155995	0.973	0.832	0	MG-Inflam	CCL3
IL1B	0	1.138198	0.864	0.57	0	MG-Inflam	IL1B
CCL4	0	1.112468	0.954	0.814	0	MG-Inflam	CCL4
CH25H	0	1.043404	0.7	0.388	0	MG-Inflam	CH25H
ABL2	0	0.996241	0.67	0.279	0	MG-Inflam	ABL2
TRIB1	0	0.972463	0.808	0.406	0	MG-Inflam	TRIB1
CCL2	0	0.971975	0.497	0.28	0	MG-Inflam	CCL2
EIF4E	0	0.960005	0.692	0.339	0	MG-Inflam	EIF4E
NR4A1	0	0.937665	0.969	0.724	0	MG-Inflam	NR4A1
C5AR1	0	0.91843	0.808	0.414	0	MG-Inflam	C5AR1
PLAUR	0	0.910896	0.787	0.445	0	MG-Inflam	PLAUR
RGS16	0	0.891009	0.64	0.292	0	MG-Inflam	RGS16
B3GNT5	0	0.869985	0.889	0.528	0	MG-Inflam	B3GNT5
CSRNP1	0	0.85742	0.772	0.366	0	MG-Inflam	CSRNP1
BCL2A1	0	0.830635	0.736	0.421	0	MG-Inflam	BCL2A1
NR4A2	0	0.827374	0.974	0.721	0	MG-Inflam	NR4A2
SERPINE1	0	0.815651	0.627	0.337	0	MG-Inflam	SERPINE1
SRGN	0	0.811668	0.998	0.916	0	MG-Inflam	SRGN
SPP1	0	0.781415	0.953	0.788	0	MG-Inflam	SPP1
DBI	0	0.761611	0.903	0.653	0	MG-Inflam	DBI
KDM6B	0	0.759654	0.816	0.45	0	MG-Inflam	KDM6B
CEBPB	0	0.753902	0.907	0.62	0	MG-Inflam	CEBPB
ICAM1	0	0.750832	0.547	0.252	0	MG-Inflam	ICAM1
ID2	0	0.748648	0.892	0.576	0	MG-Inflam	ID2
CDKN1A	0	0.746222	0.829	0.498	0	MG-Inflam	CDKN1A
KLF10	0	0.741678	0.648	0.322	0	MG-Inflam	KLF10
OLR1	0	0.734796	0.969	0.726	0	MG-Inflam	OLR1
HBEGF	0	0.730989	0.516	0.22	0	MG-Inflam	HBEGF
NEDD9	0	0.73043	0.797	0.414	0	MG-Inflam	NEDD9
CXCL8	0	0.72957	0.679	0.441	0	MG-Inflam	CXCL8
RHOB	0	0.724526	0.971	0.773	0	MG-Inflam	RHOB
NR4A3	0	0.721684	0.872	0.545	0	MG-Inflam	NR4A3
NFKBID	0	0.720526	0.774	0.41	0	MG-Inflam	NFKBID
PPP1R15A	0	0.718415	0.933	0.677	0	MG-Inflam	PPP1R15A
GADD45G	0	0.716969	0.376	0.154	0	MG-Inflam	GADD45G
GRASP	0	0.713022	0.811	0.453	0	MG-Inflam	GRASP
NLRP3	0	0.710898	0.758	0.433	0	MG-Inflam	NLRP3
JUND	0	0.702434	0.954	0.744	0	MG-Inflam	JUND
G0S2	0	0.696075	0.254	0.125	0	MG-Inflam	G0S2
NFKB1	0	0.687662	0.819	0.524	0	MG-Inflam	NFKB1
KLF6	0	0.670435	0.995	0.859	0	MG-Inflam	KLF6

OSM	0	0.666819	0.536	0.223	0	MG-Inflam	OSM
SPRY1	0	0.647872	0.453	0.179	0	MG-Inflam	SPRY1
MAFF	0	0.647389	0.447	0.177	0	MG-Inflam	MAFF
LCP2	0	0.634811	0.817	0.545	0	MG-Inflam	LCP2
RGCC	0	0.629089	0.428	0.22	0	MG-Inflam	RGCC
NFKBIA	0	0.616229	0.957	0.785	0	MG-Inflam	NFKBIA
SERTAD1	0	0.613555	0.607	0.287	0	MG-Inflam	SERTAD1
PNP	0	0.611321	0.491	0.22	0	MG-Inflam	PNP
TNFRSF12A	0	0.604561	0.293	0.097	0	MG-Inflam	TNFRSF12A
RAMP1	0	0.602783	0.367	0.126	0	MG-Inflam	RAMP1
DUSP2	0	0.598642	0.577	0.332	0	MG-Inflam	DUSP2
MAP3K8	0	0.597324	0.872	0.589	0	MG-Inflam	MAP3K8
ELL2	0	0.596017	0.969	0.724	0	MG-Inflam	ELL2
SPRY2	0	0.593192	0.454	0.19	0	MG-Inflam	SPRY2
REL	0	0.592224	0.931	0.682	0	MG-Inflam	REL
SNX9	0	0.59165	0.776	0.465	0	MG-Inflam	SNX9
TNFAIP8L3	0	0.586317	0.382	0.153	0	MG-Inflam	TNFAIP8L3
SGK1	0	0.580725	0.989	0.816	0	MG-Inflam	SGK1
EGR2	0	0.567868	0.723	0.424	0	MG-Inflam	EGR2
RILPL2	0	0.56739	0.784	0.465	0	MG-Inflam	RILPL2
GPR183	0	0.564709	0.852	0.568	0	MG-Inflam	GPR183
RGS1	0	0.564466	0.976	0.791	0	MG-Inflam	RGS1
PLEK	0	0.564364	0.747	0.496	0	MG-Inflam	PLEK
MIDN	0	0.563792	0.52	0.238	0	MG-Inflam	MIDN
OTUD1	0	0.557892	0.761	0.491	0	MG-Inflam	OTUD1
FTH1	0	0.551553	1	0.978	0	MG-Inflam	FTH1
MCL1	0	0.548764	0.97	0.765	0	MG-Inflam	MCL1
ZFP36	0	0.547387	0.997	0.907	0	MG-Inflam	ZFP36
FPR1	0	0.541804	0.846	0.55	0	MG-Inflam	FPR1
PDGFB	0	0.53068	0.572	0.278	0	MG-Inflam	PDGFB
MAP2K3	0	0.526442	0.491	0.197	0	MG-Inflam	MAP2K3
IRAK2	0	0.51835	0.723	0.366	0	MG-Inflam	IRAK2
TNFAIP3	0	0.517792	0.734	0.484	0	MG-Inflam	TNFAIP3
BASP1	0	0.517428	0.758	0.491	0	MG-Inflam	BASP1
JUNB	0	0.517357	0.985	0.853	0	MG-Inflam	JUNB
SYTL3	0	0.512381	0.622	0.351	0	MG-Inflam	SYTL3
ITPRIP	0	0.51082	0.519	0.23	0	MG-Inflam	ITPRIP
PTGS2	0	0.508099	0.499	0.268	0	MG-Inflam	PTGS2
ARID5B	0	0.502724	0.624	0.342	0	MG-Inflam	ARID5B
RAB20	0	0.500246	0.681	0.377	0	MG-Inflam	RAB20
VEGFA	0	0.499223	0.284	0.103	0	MG-Inflam	VEGFA
SYAP1	0	0.495482	0.568	0.309	0	MG-Inflam	SYAP1
PHLDA1	0	0.495306	0.309	0.121	0	MG-Inflam	PHLDA1
AL078590.2	0	0.49352	0.649	0.363	0	MG-Inflam	AL078590.2
FOSB	0	0.493188	0.952	0.701	0	MG-Inflam	FOSB
PANX1	0	0.491788	0.401	0.152	0	MG-Inflam	PANX1
PLIN2	0	0.488772	0.475	0.276	0	MG-Inflam	PLIN2

XBP1	0	0.486903	0.713	0.413	0	MG-Inflam XBP1
ETS2	0	0.486548	0.834	0.568	0	MG-Inflam ETS2
AC046195.	0	0.48616	0.469	0.204	0	MG-Inflam AC046195.1
CD83	0	0.48445	0.996	0.881	0	MG-Inflam CD83
NFIL3	0	0.484069	0.514	0.252	0	MG-Inflam NFIL3
SPHK1	0	0.483224	0.264	0.074	0	MG-Inflam SPHK1
DUSP1	0	0.479318	0.998	0.923	0	MG-Inflam DUSP1
EGR3	0	0.47203	0.69	0.373	0	MG-Inflam EGR3
TMIGD3	0	0.47064	0.579	0.328	0	MG-Inflam TMIGD3
CREM	0	0.469913	0.741	0.441	0	MG-Inflam CREM
KLF2	0	0.469696	0.903	0.651	0	MG-Inflam KLF2
SLC11A1	0	0.465879	0.831	0.563	0	MG-Inflam SLC11A1
STX11	0	0.465494	0.583	0.288	0	MG-Inflam STX11
PLK3	0	0.463442	0.688	0.372	0	MG-Inflam PLK3
NRP2	0	0.457973	0.44	0.194	0	MG-Inflam NRP2
AL118516.:	0	0.457562	0.498	0.241	0	MG-Inflam AL118516.1
LUCAT1	0	0.457194	0.282	0.089	0	MG-Inflam LUCAT1
NFATC1	0	0.452213	0.419	0.186	0	MG-Inflam NFATC1
FLT1	0	0.451787	0.328	0.133	0	MG-Inflam FLT1
HAVCR2	0	0.450509	0.773	0.494	0	MG-Inflam HAVCR2
MCF2L2	0	0.447692	0.576	0.289	0	MG-Inflam MCF2L2
HIF1A	0	0.44545	0.865	0.63	0	MG-Inflam HIF1A
ZNF267	0	0.441433	0.541	0.264	0	MG-Inflam ZNF267
GPR84	0	0.437511	0.339	0.129	0	MG-Inflam GPR84
SAT1	0	0.437417	0.998	0.96	0	MG-Inflam SAT1
CHSY1	0	0.436546	0.556	0.262	0	MG-Inflam CHSY1
LMNA	0	0.434124	0.263	0.107	0	MG-Inflam LMNA
OGFRL1	0	0.43252	0.851	0.606	0	MG-Inflam OGFRL1
EIF5	0	0.429821	0.847	0.58	0	MG-Inflam EIF5
CKB	0	0.428605	0.596	0.322	0	MG-Inflam CKB
CFLAR	0	0.428402	0.647	0.397	0	MG-Inflam CFLAR
IRF2BP2	0	0.42694	0.448	0.211	0	MG-Inflam IRF2BP2
ZNF331	0	0.422228	0.807	0.556	0	MG-Inflam ZNF331
GADD45B	0	0.420625	0.934	0.72	0	MG-Inflam GADD45B
C3AR1	0	0.418963	0.823	0.566	0	MG-Inflam C3AR1
SOCS3	0	0.416499	0.352	0.133	0	MG-Inflam SOCS3
SKIL	0	0.41502	0.866	0.604	0	MG-Inflam SKIL
SLC3A2	0	0.414431	0.568	0.313	0	MG-Inflam SLC3A2
MYADM	0	0.414298	0.485	0.284	0	MG-Inflam MYADM
PDE4B	0	0.414117	0.681	0.437	0	MG-Inflam PDE4B
DDIT3	0	0.410461	0.477	0.226	0	MG-Inflam DDIT3
PRXL2C	0	0.407853	0.427	0.18	0	MG-Inflam PRXL2C
CXCL16	0	0.405918	0.832	0.574	0	MG-Inflam CXCL16
TUBB4B	0	0.404401	0.629	0.354	0	MG-Inflam TUBB4B
SELENOK	0	0.403979	0.638	0.387	0	MG-Inflam SELENOK
CD69	0	0.402753	0.682	0.424	0	MG-Inflam CD69
ITGAX	0	0.400717	0.62	0.34	0	MG-Inflam ITGAX

LINC00963	0	0.397118	0.465	0.208	0	MG-Inflam	LINC00963
AF213884.:	0	0.395385	0.426	0.211	0	MG-Inflam	AF213884.3
ERCC1	0	0.391545	0.768	0.514	0	MG-Inflam	ERCC1
PDE4DIP	0	0.390626	0.47	0.228	0	MG-Inflam	PDE4DIP
STX4	0	0.390134	0.479	0.245	0	MG-Inflam	STX4
JMJD6	0	0.390013	0.431	0.196	0	MG-Inflam	JMJD6
ZFAND5	0	0.389455	0.665	0.439	0	MG-Inflam	ZFAND5
AL139807.:	0	0.387617	0.384	0.16	0	MG-Inflam	AL139807.1
BTG3	0	0.387282	0.437	0.192	0	MG-Inflam	BTG3
TNFSF9	0	0.3871	0.377	0.159	0	MG-Inflam	TNFSF9
BHLHE40	0	0.385431	0.452	0.218	0	MG-Inflam	BHLHE40
CTDSP1	0	0.384704	0.517	0.255	0	MG-Inflam	CTDSP1
PLVAP	0	0.38441	0.281	0.114	0	MG-Inflam	PLVAP
SRSF5	0	0.382096	0.817	0.562	0	MG-Inflam	SRSF5
CKS2	0	0.379525	0.442	0.238	0	MG-Inflam	CKS2
SH2B3	0	0.379142	0.576	0.293	0	MG-Inflam	SH2B3
ELOC	0	0.378362	0.586	0.337	0	MG-Inflam	ELOC
EIF4A1	0	0.376716	0.933	0.754	0	MG-Inflam	EIF4A1
H3F3B	0	0.376704	0.989	0.891	0	MG-Inflam	H3F3B
IRAK3	0	0.375893	0.841	0.588	0	MG-Inflam	IRAK3
IL1RN	0	0.375415	0.251	0.136	0	MG-Inflam	IL1RN
CEBPD	0	0.375243	0.977	0.832	0	MG-Inflam	CEBPD
AL136987.:	0	0.374362	0.459	0.235	0	MG-Inflam	AL136987.1
RRAGA	0	0.373452	0.358	0.186	0	MG-Inflam	RRAGA
SFPQ	0	0.37314	0.818	0.564	0	MG-Inflam	SFPQ
PDPN	0	0.371382	0.441	0.21	0	MG-Inflam	PDPN
RAB1A	0	0.367284	0.746	0.457	0	MG-Inflam	RAB1A
ITGAV	0	0.366893	0.652	0.38	0	MG-Inflam	ITGAV
KLHL6	0	0.364526	0.645	0.353	0	MG-Inflam	KLHL6
NAF1	0	0.360798	0.411	0.195	0	MG-Inflam	NAF1
AC131944.	0	0.359143	0.458	0.204	0	MG-Inflam	AC131944.1
UBASH3B	0	0.358419	0.365	0.144	0	MG-Inflam	UBASH3B
3-Mar	0	0.357633	0.642	0.414	0	MG-Inflam	3-Mar
ELF1	0	0.356057	0.858	0.601	0	MG-Inflam	ELF1
PTGER4	0	0.351274	0.556	0.307	0	MG-Inflam	PTGER4
NRARP	0	0.346108	0.251	0.084	0	MG-Inflam	NRARP
PER1	0	0.34266	0.776	0.496	0	MG-Inflam	PER1
GNA13	0	0.338475	0.825	0.522	0	MG-Inflam	GNA13
GBP2	0	0.337278	0.573	0.346	0	MG-Inflam	GBP2
TNF	0	0.336861	0.61	0.388	0	MG-Inflam	TNF
CHMP4B	0	0.334968	0.525	0.276	0	MG-Inflam	CHMP4B
PRKCH	0	0.33466	0.789	0.524	0	MG-Inflam	PRKCH
PMEPA1	0	0.334488	0.544	0.286	0	MG-Inflam	PMEPA1
MAP4K3	0	0.334015	0.73	0.43	0	MG-Inflam	MAP4K3
TG	0	0.333818	0.284	0.116	0	MG-Inflam	TG
TPRG1	0	0.333642	0.276	0.124	0	MG-Inflam	TPRG1
SRSF2	0	0.330345	0.714	0.464	0	MG-Inflam	SRSF2

ATP13A3	0	0.329091	0.522	0.259	0	MG-Inflam ATP13A3
CTSL	0	0.327696	0.528	0.313	0	MG-Inflam CTSL
PDK4	0	0.327516	0.783	0.549	0	MG-Inflam PDK4
SOD2	0	0.326406	0.734	0.525	0	MG-Inflam SOD2
ARL6IP1	0	0.326338	0.766	0.522	0	MG-Inflam ARL6IP1
RELB	0	0.324571	0.426	0.201	0	MG-Inflam RELB
AKAP13	0	0.322321	0.931	0.725	0	MG-Inflam AKAP13
APOC2	0	0.322011	0.777	0.589	0	MG-Inflam APOC2
RASSF5	0	0.321253	0.56	0.299	0	MG-Inflam RASSF5
KLF4	0	0.320168	0.716	0.434	0	MG-Inflam KLF4
ATF3	0	0.319773	0.776	0.513	0	MG-Inflam ATF3
LILRB4	0	0.31885	0.847	0.598	0	MG-Inflam LILRB4
TPM4	0	0.318645	0.596	0.36	0	MG-Inflam TPM4
IPCEF1	0	0.318367	0.702	0.437	0	MG-Inflam IPCEF1
PRDM1	0	0.318188	0.515	0.295	0	MG-Inflam PRDM1
AZIN1-AS1	0	0.314254	0.513	0.304	0	MG-Inflam AZIN1-AS1
GABARAPL	0	0.313923	0.483	0.257	0	MG-Inflam GABARAPL1
NRROS	0	0.313815	0.281	0.114	0	MG-Inflam NRROS
MSR1	0	0.311992	0.749	0.516	0	MG-Inflam MSR1
TGIF1	0	0.311984	0.397	0.204	0	MG-Inflam TGIF1
GLIPR2	0	0.308348	0.31	0.138	0	MG-Inflam GLIPR2
SRSF3	0	0.307877	0.724	0.473	0	MG-Inflam SRSF3
FHOD1	0	0.307467	0.267	0.128	0	MG-Inflam FHOD1
AC087286.	0	0.307299	0.32	0.137	0	MG-Inflam AC087286.2
SLC16A3	0	0.307147	0.528	0.319	0	MG-Inflam SLC16A3
TSC22D3	0	0.301039	0.87	0.662	0	MG-Inflam TSC22D3
IRS2	0	0.300697	0.614	0.372	0	MG-Inflam IRS2
TMEM52B	0	0.299453	0.48	0.253	0	MG-Inflam TMEM52B
LIMS1	0	0.299315	0.864	0.625	0	MG-Inflam LIMS1
PDE4A	0	0.299305	0.389	0.193	0	MG-Inflam PDE4A
SLC1A5	0	0.298352	0.487	0.272	0	MG-Inflam SLC1A5
DUSP6	0	0.297892	0.402	0.246	0	MG-Inflam DUSP6
FCGR1A	0	0.297403	0.681	0.445	0	MG-Inflam FCGR1A
SLC37A2	0	0.29732	0.45	0.251	0	MG-Inflam SLC37A2
TUBA1C	0	0.297092	0.448	0.245	0	MG-Inflam TUBA1C
PEAK1	0	0.295967	0.708	0.422	0	MG-Inflam PEAK1
PTPN1	0	0.294629	0.594	0.34	0	MG-Inflam PTPN1
AC079015.	0	0.292405	0.301	0.129	0	MG-Inflam AC079015.1
POMP	0	0.292054	0.693	0.445	0	MG-Inflam POMP
CLEC7A	0	0.291705	0.799	0.564	0	MG-Inflam CLEC7A
DAGLB	0	0.291278	0.614	0.365	0	MG-Inflam DAGLB
MANBA	0	0.290962	0.717	0.49	0	MG-Inflam MANBA
GPR132	0	0.290627	0.426	0.233	0	MG-Inflam GPR132
IER3	0	0.290618	0.917	0.717	0	MG-Inflam IER3
INAFM2	0	0.289237	0.374	0.184	0	MG-Inflam INAFM2
PMAIP1	0	0.288835	0.428	0.252	0	MG-Inflam PMAIP1
BZW1	0	0.288038	0.563	0.323	0	MG-Inflam BZW1

HERPUD1	0	0.286692	0.841	0.654	0	MG-Inflam	HERPUD1
EIF1	0	0.286544	0.99	0.9	0	MG-Inflam	EIF1
SLC7A1	0	0.28569	0.282	0.108	0	MG-Inflam	SLC7A1
CSF2RA	0	0.285378	0.848	0.613	0	MG-Inflam	CSF2RA
CDC14B	0	0.283662	0.259	0.118	0	MG-Inflam	CDC14B
ENSA	0	0.283504	0.812	0.568	0	MG-Inflam	ENSA
SLC2A3	0	0.283155	0.563	0.321	0	MG-Inflam	SLC2A3
RFX2	0	0.282743	0.47	0.232	0	MG-Inflam	RFX2
SPTLC2	0	0.282347	0.773	0.537	0	MG-Inflam	SPTLC2
SSPN	0	0.281177	0.465	0.263	0	MG-Inflam	SSPN
RAPGEF1	0	0.2789	0.737	0.466	0	MG-Inflam	RAPGEF1
ANXA5	0	0.278662	0.806	0.571	0	MG-Inflam	ANXA5
IFNGR2	0	0.278531	0.711	0.485	0	MG-Inflam	IFNGR2
ARID5A	0	0.277148	0.524	0.312	0	MG-Inflam	ARID5A
MYC	0	0.276693	0.278	0.134	0	MG-Inflam	MYC
PNRC1	0	0.275046	0.957	0.814	0	MG-Inflam	PNRC1
C9orf72	0	0.274711	0.751	0.511	0	MG-Inflam	C9orf72
IL1RAP	0	0.273925	0.592	0.329	0	MG-Inflam	IL1RAP
UST	0	0.273736	0.319	0.145	0	MG-Inflam	UST
CSNK1A1	0	0.271565	0.689	0.433	0	MG-Inflam	CSNK1A1
BCL6	0	0.270895	0.495	0.273	0	MG-Inflam	BCL6
CITED2	0	0.270312	0.671	0.441	0	MG-Inflam	CITED2
FABP5	0	0.267804	0.325	0.186	0	MG-Inflam	FABP5
SDCBP	0	0.267651	0.808	0.58	0	MG-Inflam	SDCBP
SELENOS	0	0.265424	0.396	0.212	0	MG-Inflam	SELENOS
UBE2F	0	0.265207	0.458	0.261	0	MG-Inflam	UBE2F
JDP2	0	0.263107	0.743	0.507	0	MG-Inflam	JDP2
CDC42SE1	0	0.262234	0.561	0.323	0	MG-Inflam	CDC42SE1
CXCR4	0	0.26184	0.598	0.411	0	MG-Inflam	CXCR4
MS4A7	0	0.261485	0.84	0.632	0	MG-Inflam	MS4A7
LINC01366	0	0.259275	0.287	0.138	0	MG-Inflam	LINC01366
SOCS6	0	0.258549	0.546	0.327	0	MG-Inflam	SOCS6
RTN4	0	0.255955	0.79	0.574	0	MG-Inflam	RTN4
DDX3X	0	0.254148	0.695	0.447	0	MG-Inflam	DDX3X
FP236383.:	0	1.299721	0.742	0.447	0	MG-Phago	FP236383.3
AC004448.	0	1.018533	0.368	0.183	0	MG-Phago	AC004448.2
FP671120.4	0	0.888485	0.534	0.391	0	MG-Phago	FP671120.4
TUBA1B	0	0.793051	0.805	0.81	0	MG-Phago	TUBA1B
C1QB	0	0.731044	0.952	0.879	0	MG-Phago	C1QB
RPLP0	0	0.641685	0.883	0.865	0	MG-Phago	RPLP0
C1QA	0	0.632681	0.918	0.872	0	MG-Phago	C1QA
AIF1	0	0.625021	0.755	0.792	0	MG-Phago	AIF1
APOC21	0	0.622988	0.681	0.63	0	MG-Phago	APOC2
PFN1	0	0.621509	0.826	0.828	0	MG-Phago	PFN1
APOC1	0	0.620607	0.817	0.775	0	MG-Phago	APOC1
NPC2	0	0.601315	0.801	0.806	0	MG-Phago	NPC2
C1QC	0	0.584527	0.958	0.889	0	MG-Phago	C1QC

RPL5	0	0.565259	0.819	0.843	0	MG-Phago	RPL5
GABARAP	0	0.560696	0.749	0.8	0	MG-Phago	GABARAP
ACTB	0	0.550784	0.989	0.967	0	MG-Phago	ACTB
ZFP36L1	0	0.541776	0.865	0.877	0	MG-Phago	ZFP36L1
CD74	0	0.515527	0.996	0.989	0	MG-Phago	CD74
MT-ATP8	0	0.511419	0.788	0.831	0	MG-Phago	MT-ATP8
TMSB4X	0	0.509176	0.985	0.958	0	MG-Phago	TMSB4X
RPL8	0	0.503662	0.847	0.855	0	MG-Phago	RPL8
RPS8	0	0.503207	0.927	0.912	0	MG-Phago	RPS8
RPL7A	0	0.497614	0.854	0.874	0	MG-Phago	RPL7A
APOE	0	0.493259	0.97	0.909	0	MG-Phago	APOE
TPT1	0	0.492403	0.972	0.952	0	MG-Phago	TPT1
RPL15	0	0.481486	0.837	0.862	0	MG-Phago	RPL15
RPL17	0	0.48076	0.804	0.844	0	MG-Phago	RPL17
RPL6	0	0.479959	0.833	0.865	0	MG-Phago	RPL6
RPL12	0	0.476032	0.883	0.895	0	MG-Phago	RPL12
RPS15	0	0.473998	0.885	0.896	0	MG-Phago	RPS15
RPL29	0	0.472522	0.873	0.882	0	MG-Phago	RPL29
NACA	0	0.467741	0.791	0.847	0	MG-Phago	NACA
RPS3	0	0.465554	0.88	0.877	0	MG-Phago	RPS3
FCER1G	0	0.463949	0.781	0.826	0	MG-Phago	FCER1G
RPL13	0	0.459098	0.954	0.932	0	MG-Phago	RPL13
RPS7	0	0.459023	0.841	0.867	0	MG-Phago	RPS7
RPS23	0	0.455839	0.917	0.907	0	MG-Phago	RPS23
OAZ1	0	0.449998	0.734	0.813	0	MG-Phago	OAZ1
RPL14	0	0.447515	0.814	0.866	0	MG-Phago	RPL14
S100A11	0	0.446656	0.794	0.84	0	MG-Phago	S100A11
RACK1	0	0.445489	0.782	0.834	0	MG-Phago	RACK1
RPL11	0	0.443915	0.895	0.902	0	MG-Phago	RPL11
RPLP1	0	0.441607	0.97	0.943	0	MG-Phago	RPLP1
RPS18	0	0.440549	0.914	0.903	0	MG-Phago	RPS18
PSAP	0	0.439797	0.873	0.901	0	MG-Phago	PSAP
RPL26	0	0.439507	0.882	0.889	0	MG-Phago	RPL26
RPS13	0	0.438738	0.882	0.887	0	MG-Phago	RPS13
RPS27A	0	0.429226	0.874	0.886	0	MG-Phago	RPS27A
RPL19	0	0.429151	0.906	0.905	0	MG-Phago	RPL19
RPS12	0	0.425779	0.933	0.911	0	MG-Phago	RPS12
GAPDH	0	0.424116	0.863	0.868	0	MG-Phago	GAPDH
RPS6	0	0.419323	0.87	0.886	0	MG-Phago	RPS6
RPS14	0	0.418427	0.887	0.885	0	MG-Phago	RPS14
RPS19	0	0.417537	0.927	0.921	0	MG-Phago	RPS19
RPS24	0	0.417101	0.897	0.903	0	MG-Phago	RPS24
RPL28	0	0.416705	0.926	0.918	0	MG-Phago	RPL28
RPS4X	0	0.408603	0.833	0.861	0	MG-Phago	RPS4X
RPL10	0	0.405425	0.962	0.943	0	MG-Phago	RPL10
RPL3	0	0.403559	0.845	0.875	0	MG-Phago	RPL3
RPS3A	0	0.402857	0.869	0.878	0	MG-Phago	RPS3A

TYROBP	0	0.402097	0.884	0.917	0	MG-Phago	TYROBP
RPL18	0	0.399583	0.849	0.881	0	MG-Phago	RPL18
FAU	0	0.397207	0.843	0.89	0	MG-Phago	FAU
RPL32	0	0.391746	0.907	0.909	0	MG-Phago	RPL32
RPS15A	0	0.391068	0.874	0.891	0	MG-Phago	RPS15A
CYBA	0	0.390003	0.85	0.896	0	MG-Phago	CYBA
FTL	0	0.38692	0.989	0.97	0	MG-Phago	FTL
RPL30	0	0.380526	0.887	0.896	0	MG-Phago	RPL30
RPL18A	0	0.376091	0.872	0.885	0	MG-Phago	RPL18A
CST3	0	0.369783	0.9	0.902	0	MG-Phago	CST3
HLA-DPA1	0	0.354427	0.908	0.87	0	MG-Phago	HLA-DPA1
RPS28	0	0.35036	0.845	0.887	0	MG-Phago	RPS28
HLA-B	0	0.333155	0.921	0.937	0	MG-Phago	HLA-B
HLA-DRB1	0	0.25045	0.933	0.903	0	MG-Phago	HLA-DRB1
RPS5	1.54E-296	0.471336	0.738	0.777	4.35E-292	MG-Phago	RPS5
HAMP	2.06E-288	0.895949	0.426	0.35	5.79E-284	MG-Phago	HAMP
C3	2.09E-288	0.39558	0.887	0.89	5.89E-284	MG-Phago	C3
TMSB10	3.79E-287	0.285414	0.905	0.93	1.07E-282	MG-Phago	TMSB10
RPS25	2.95E-282	0.362161	0.799	0.862	8.31E-278	MG-Phago	RPS25
CD81	2.39E-270	0.385047	0.797	0.858	6.73E-266	MG-Phago	CD81
ATP5MC2	3.23E-270	0.539053	0.668	0.735	9.10E-266	MG-Phago	ATP5MC2
RPL34	9.50E-260	0.323695	0.843	0.89	2.67E-255	MG-Phago	RPL34
CTSB	9.41E-257	0.311346	0.876	0.928	2.65E-252	MG-Phago	CTSB
CFL1	4.04E-255	0.395577	0.793	0.852	1.14E-250	MG-Phago	CFL1
RPL36	3.24E-240	0.36313	0.773	0.85	9.11E-236	MG-Phago	RPL36
RPS26	6.25E-238	0.392852	0.77	0.825	1.76E-233	MG-Phago	RPS26
HIST1H1E	2.98E-237	1.056299	0.287	0.206	8.40E-233	MG-Phago	HIST1H1E
ATP5F1E	1.15E-227	0.46432	0.686	0.77	3.22E-223	MG-Phago	ATP5F1E
ARPC3	4.07E-227	0.442533	0.688	0.787	1.14E-222	MG-Phago	ARPC3
PPIA	1.79E-226	0.399319	0.745	0.83	5.05E-222	MG-Phago	PPIA
TMEM176B	3.54E-224	0.676969	0.54	0.541	9.97E-220	MG-Phago	TMEM176B
SERF2	3.04E-219	0.416025	0.72	0.823	8.56E-215	MG-Phago	SERF2
NME2	1.43E-218	0.553281	0.627	0.677	4.01E-214	MG-Phago	NME2
RNASET2	2.98E-209	0.30174	0.83	0.919	8.39E-205	MG-Phago	RNASET2
COX4I1	1.57E-207	0.464059	0.674	0.756	4.42E-203	MG-Phago	COX4I1
RPL35	1.00E-188	0.372285	0.726	0.817	2.81E-184	MG-Phago	RPL35
ALOX5AP	1.05E-187	0.444264	0.698	0.759	2.97E-183	MG-Phago	ALOX5AP
TREM2	3.42E-177	0.42019	0.689	0.745	9.63E-173	MG-Phago	TREM2
PTMA	3.92E-167	0.254942	0.869	0.916	1.10E-162	MG-Phago	PTMA
RPL39	1.12E-164	0.269146	0.845	0.892	3.15E-160	MG-Phago	RPL39
FCGR3A	1.22E-162	0.639092	0.573	0.613	3.44E-158	MG-Phago	FCGR3A
B2M	1.27E-156	0.307895	0.976	0.972	3.57E-152	MG-Phago	B2M
RPL4	1.18E-155	0.484884	0.676	0.755	3.33E-151	MG-Phago	RPL4
TSPO	4.44E-152	0.554115	0.571	0.618	1.25E-147	MG-Phago	TSPO
EEF1B2	1.92E-151	0.391294	0.705	0.776	5.40E-147	MG-Phago	EEF1B2
RPLP2	1.62E-145	0.283994	0.775	0.86	4.57E-141	MG-Phago	RPLP2
RAC1	2.26E-143	0.305427	0.729	0.85	6.35E-139	MG-Phago	RAC1

ARPC1B	7.43E-142	0.490264	0.611	0.704	2.09E-137	MG-Phago	ARPC1B
MYL6	1.14E-133	0.379079	0.7	0.791	3.21E-129	MG-Phago	MYL6
HLA-E	5.89E-132	0.305807	0.748	0.852	1.66E-127	MG-Phago	HLA-E
RPL35A	3.39E-124	0.27634	0.755	0.839	9.54E-120	MG-Phago	RPL35A
RPL10A	4.48E-122	0.353668	0.695	0.783	1.26E-117	MG-Phago	RPL10A
RPL22	2.86E-111	0.360432	0.672	0.775	8.04E-107	MG-Phago	RPL22
HLA-DMA	1.06E-98	0.367141	0.661	0.724	2.99E-94	MG-Phago	HLA-DMA
MT-ND5	7.68E-98	0.392156	0.814	0.897	2.16E-93	MG-Phago	MT-ND5
UBA52	1.17E-96	0.296429	0.695	0.797	3.29E-92	MG-Phago	UBA52
RPSA	1.97E-96	0.390095	0.646	0.737	5.54E-92	MG-Phago	RPSA
EEF1G	9.40E-96	0.410422	0.629	0.711	2.65E-91	MG-Phago	EEF1G
APOO	1.19E-93	0.624295	0.314	0.275	3.34E-89	MG-Phago	APOO
RPS21	1.84E-92	0.2698	0.729	0.824	5.16E-88	MG-Phago	RPS21
EIF5A	5.18E-91	0.257825	0.244	0.366	1.46E-86	MG-Phago	EIF5A
A2M	7.96E-91	0.331035	0.703	0.764	2.24E-86	MG-Phago	A2M
PPT1	1.04E-87	0.702613	0.504	0.554	2.92E-83	MG-Phago	PPT1
ARHGDIB	4.32E-84	0.418545	0.614	0.724	1.22E-79	MG-Phago	ARHGDIB
ITGB2	9.32E-82	0.40232	0.65	0.763	2.62E-77	MG-Phago	ITGB2
PFDN5	3.20E-81	0.363269	0.636	0.75	9.00E-77	MG-Phago	PFDN5
RPL24	9.27E-78	0.259028	0.733	0.837	2.61E-73	MG-Phago	RPL24
TUBB	2.61E-76	0.798889	0.452	0.484	7.33E-72	MG-Phago	TUBB
CLTC	2.70E-72	0.267763	0.262	0.379	7.60E-68	MG-Phago	CLTC
RAN	1.61E-68	0.276196	0.338	0.489	4.53E-64	MG-Phago	RAN
SH3BGRL3	2.61E-67	0.366741	0.603	0.683	7.33E-63	MG-Phago	SH3BGRL3
HLA-DQB1	9.24E-63	0.263457	0.722	0.744	2.60E-58	MG-Phago	HLA-DQB1
RPL23A	2.96E-62	0.270819	0.701	0.8	8.33E-58	MG-Phago	RPL23A
CAPRIN1	3.77E-62	0.262776	0.188	0.274	1.06E-57	MG-Phago	CAPRIN1
RHOA	3.41E-61	0.311901	0.656	0.787	9.60E-57	MG-Phago	RHOA
CD302	1.16E-59	0.532918	0.518	0.586	3.25E-55	MG-Phago	CD302
NCL	3.26E-58	0.273488	0.453	0.633	9.17E-54	MG-Phago	NCL
FCGRT	2.24E-57	0.390347	0.618	0.733	6.32E-53	MG-Phago	FCGRT
IFI6	4.92E-57	0.318868	0.213	0.3	1.38E-52	MG-Phago	IFI6
RPS4Y1	1.36E-56	0.497138	0.423	0.432	3.82E-52	MG-Phago	RPS4Y1
MOB1A	4.05E-56	0.31413	0.308	0.438	1.14E-51	MG-Phago	MOB1A
YWHAH	5.13E-56	0.458873	0.577	0.666	1.44E-51	MG-Phago	YWHAH
MS4A6A	5.86E-54	0.433692	0.508	0.549	1.65E-49	MG-Phago	MS4A6A
UQCR10	1.81E-50	0.568397	0.434	0.478	5.10E-46	MG-Phago	UQCR10
HLA-DRB5	7.53E-50	0.345645	0.651	0.704	2.12E-45	MG-Phago	HLA-DRB5
BTF3	3.61E-49	0.359079	0.617	0.728	1.02E-44	MG-Phago	BTF3
EEF2	6.09E-49	0.324642	0.683	0.785	1.71E-44	MG-Phago	EEF2
SRSF9	7.09E-49	0.275312	0.287	0.401	2.00E-44	MG-Phago	SRSF9
HCLS1	8.27E-47	0.297753	0.67	0.821	2.33E-42	MG-Phago	HCLS1
RNASEK	2.39E-43	0.35516	0.612	0.734	6.74E-39	MG-Phago	RNASEK
TGOLN2	2.73E-43	0.295747	0.325	0.448	7.67E-39	MG-Phago	TGOLN2
NUCB1	1.01E-42	0.305475	0.253	0.349	2.83E-38	MG-Phago	NUCB1
TUFM	3.88E-42	0.287118	0.249	0.344	1.09E-37	MG-Phago	TUFM
PSMD8	4.77E-41	0.25514	0.216	0.296	1.34E-36	MG-Phago	PSMD8

BAX	1.84E-40	0.260915	0.309	0.422	5.18E-36	MG-Phago	BAX
COPE	5.31E-40	0.25832	0.332	0.458	1.49E-35	MG-Phago	COPE
PSMB3	6.64E-39	0.268025	0.275	0.377	1.87E-34	MG-Phago	PSMB3
ENO1	2.85E-38	0.293584	0.369	0.506	8.02E-34	MG-Phago	ENO1
RPL27	5.49E-38	0.310342	0.62	0.734	1.55E-33	MG-Phago	RPL27
RBBP4	7.87E-38	0.252164	0.188	0.256	2.21E-33	MG-Phago	RBBP4
CIB1	1.64E-37	0.262994	0.255	0.346	4.62E-33	MG-Phago	CIB1
ATP5PB	1.81E-37	0.267534	0.277	0.377	5.08E-33	MG-Phago	ATP5PB
HADHA	5.94E-37	0.264263	0.277	0.376	1.67E-32	MG-Phago	HADHA
CCL21	7.64E-36	0.360014	0.345	0.337	2.15E-31	MG-Phago	CCL2
PA2G4	6.47E-35	0.301863	0.224	0.303	1.82E-30	MG-Phago	PA2G4
PHB2	7.63E-35	0.261018	0.256	0.347	2.15E-30	MG-Phago	PHB2
AP2M1	1.07E-34	0.275669	0.257	0.348	3.00E-30	MG-Phago	AP2M1
SRP9	2.13E-34	0.264129	0.203	0.275	6.00E-30	MG-Phago	SRP9
ERP29	3.86E-34	0.255327	0.32	0.431	1.09E-29	MG-Phago	ERP29
CLTA	4.10E-34	0.258604	0.351	0.478	1.15E-29	MG-Phago	CLTA
RAB5C	7.89E-34	0.289892	0.335	0.459	2.22E-29	MG-Phago	RAB5C
SIVA1	9.16E-34	0.261952	0.198	0.266	2.58E-29	MG-Phago	SIVA1
ATP5F1B	1.11E-33	0.268365	0.417	0.571	3.13E-29	MG-Phago	ATP5F1B
FCGBP	1.47E-33	0.504425	0.38	0.383	4.13E-29	MG-Phago	FCGBP
HSBP1	1.57E-33	0.253829	0.224	0.301	4.41E-29	MG-Phago	HSBP1
EID1	2.64E-33	0.259552	0.304	0.41	7.43E-29	MG-Phago	EID1
ZYX	1.40E-32	0.278399	0.354	0.469	3.95E-28	MG-Phago	ZYX
ATP5MG	2.08E-32	0.415673	0.535	0.634	5.87E-28	MG-Phago	ATP5MG
GSTP1	4.15E-32	0.440618	0.517	0.597	1.17E-27	MG-Phago	GSTP1
VAMP8	8.70E-32	0.481182	0.467	0.54	2.45E-27	MG-Phago	VAMP8
KDELR1	1.22E-31	0.259769	0.292	0.395	3.42E-27	MG-Phago	KDELR1
ALDOA	1.72E-31	0.276119	0.359	0.481	4.83E-27	MG-Phago	ALDOA
BCAP31	1.81E-31	0.26297	0.353	0.48	5.09E-27	MG-Phago	BCAP31
DRAP1	2.72E-31	0.256323	0.34	0.464	7.66E-27	MG-Phago	DRAP1
SUSD3	5.78E-31	0.263423	0.199	0.264	1.63E-26	MG-Phago	SUSD3
NOP10	1.08E-30	0.263725	0.259	0.345	3.04E-26	MG-Phago	NOP10
LINC01736	1.79E-30	0.480801	0.405	0.44	5.03E-26	MG-Phago	LINC01736
SSR2	2.36E-30	0.277118	0.312	0.42	6.64E-26	MG-Phago	SSR2
TMED9	3.01E-30	0.292732	0.296	0.399	8.46E-26	MG-Phago	TMED9
H2AFV	3.68E-30	0.337687	0.234	0.313	1.04E-25	MG-Phago	H2AFV
FKBP1A	4.73E-30	0.284069	0.233	0.309	1.33E-25	MG-Phago	FKBP1A
SELENOH	5.88E-30	0.262943	0.34	0.46	1.65E-25	MG-Phago	SELENOH
MDH2	6.86E-30	0.286015	0.254	0.339	1.93E-25	MG-Phago	MDH2
ATP5F1A	7.14E-30	0.29493	0.257	0.345	2.01E-25	MG-Phago	ATP5F1A
GPSM3	2.20E-29	0.26015	0.337	0.456	6.18E-25	MG-Phago	GPSM3
POLR2E	2.87E-29	0.264182	0.216	0.287	8.08E-25	MG-Phago	POLR2E
MNDA	2.33E-28	0.252487	0.251	0.33	6.55E-24	MG-Phago	MNDA
PAIP2	9.44E-28	0.250481	0.25	0.33	2.66E-23	MG-Phago	PAIP2
EEF1D	1.90E-27	0.338831	0.591	0.72	5.34E-23	MG-Phago	EEF1D
TLN1	2.80E-27	0.278669	0.441	0.59	7.88E-23	MG-Phago	TLN1
OSTF1	5.99E-27	0.25545	0.291	0.389	1.69E-22	MG-Phago	OSTF1

FKBP8	6.45E-27	0.275685	0.299	0.399	1.82E-22	MG-Phago	FKBP8
MT-ND6	1.20E-26	0.735031	0.494	0.578	3.38E-22	MG-Phago	MT-ND6
SCIN	1.90E-26	0.432923	0.502	0.569	5.35E-22	MG-Phago	SCIN
SPI1	5.84E-26	0.35853	0.584	0.712	1.64E-21	MG-Phago	SPI1
COTL1	7.54E-26	0.363606	0.612	0.719	2.12E-21	MG-Phago	COTL1
CX3CR1	3.29E-25	0.408776	0.519	0.587	9.25E-21	MG-Phago	CX3CR1
NDUFB4	4.23E-25	0.269859	0.277	0.366	1.19E-20	MG-Phago	NDUFB4
PARP1	5.53E-25	0.314794	0.214	0.282	1.56E-20	MG-Phago	PARP1
MIF	5.75E-25	0.413014	0.483	0.553	1.62E-20	MG-Phago	MIF
TRAPPC5	1.12E-24	0.25236	0.227	0.296	3.14E-20	MG-Phago	TRAPPC5
ATP5MPL	1.18E-24	0.252122	0.25	0.327	3.33E-20	MG-Phago	ATP5MPL
RPS27L	1.44E-24	0.269202	0.27	0.353	4.05E-20	MG-Phago	RPS27L
RPL13A	2.51E-24	0.253698	0.642	0.771	7.07E-20	MG-Phago	RPL13A
UCP2	3.39E-24	0.35057	0.283	0.373	9.54E-20	MG-Phago	UCP2
PGAM1	4.21E-24	0.324851	0.227	0.297	1.19E-19	MG-Phago	PGAM1
CANX	1.25E-23	0.332533	0.388	0.519	3.51E-19	MG-Phago	CANX
ANP32B	1.42E-23	0.322549	0.302	0.398	4.00E-19	MG-Phago	ANP32B
RGS10	6.31E-23	0.290074	0.632	0.783	1.78E-18	MG-Phago	RGS10
DAD1	7.77E-23	0.282894	0.357	0.48	2.18E-18	MG-Phago	DAD1
NDUFB9	1.80E-22	0.32528	0.259	0.34	5.07E-18	MG-Phago	NDUFB9
PSME2	1.98E-22	0.305619	0.282	0.369	5.56E-18	MG-Phago	PSME2
PADI2	2.19E-22	0.264146	0.54	0.591	6.17E-18	MG-Phago	PADI2
EIF3G	2.97E-22	0.282428	0.373	0.498	8.36E-18	MG-Phago	EIF3G
CTSZ	4.46E-22	0.382821	0.554	0.661	1.25E-17	MG-Phago	CTSZ
PSMB10	4.93E-22	0.299023	0.259	0.339	1.39E-17	MG-Phago	PSMB10
TRMT112	5.38E-22	0.27158	0.31	0.407	1.51E-17	MG-Phago	TRMT112
NDUFA1	7.89E-22	0.256477	0.332	0.439	2.22E-17	MG-Phago	NDUFA1
MARCKS	8.97E-22	0.258825	0.625	0.759	2.52E-17	MG-Phago	MARCKS
MICOS10	1.53E-21	0.28397	0.372	0.498	4.30E-17	MG-Phago	MICOS10
PRR13	2.21E-21	0.274581	0.359	0.479	6.22E-17	MG-Phago	PRR13
NAA38	2.25E-21	0.260746	0.198	0.255	6.33E-17	MG-Phago	NAA38
FSCN1	2.39E-21	0.313274	0.338	0.436	6.74E-17	MG-Phago	FSCN1
PSMB6	3.39E-21	0.306739	0.26	0.34	9.55E-17	MG-Phago	PSMB6
RAB32	3.63E-21	0.265362	0.206	0.266	1.02E-16	MG-Phago	RAB32
RTRAF	5.05E-21	0.272495	0.265	0.345	1.42E-16	MG-Phago	RTRAF
ALKBH7	6.54E-21	0.273246	0.264	0.343	1.84E-16	MG-Phago	ALKBH7
UQCR11	7.19E-21	0.472082	0.438	0.508	2.02E-16	MG-Phago	UQCR11
ANP32A	1.82E-20	0.308012	0.332	0.438	5.13E-16	MG-Phago	ANP32A
GNPTG	3.02E-20	0.264005	0.231	0.296	8.50E-16	MG-Phago	GNPTG
TKT	3.26E-20	0.270271	0.322	0.42	9.17E-16	MG-Phago	TKT
SET	4.00E-20	0.358932	0.319	0.42	1.12E-15	MG-Phago	SET
HMGB1	6.86E-20	0.291209	0.641	0.79	1.93E-15	MG-Phago	HMGB1
TAF10	1.04E-19	0.273452	0.364	0.485	2.91E-15	MG-Phago	TAF10
PLAC8	1.46E-19	0.307476	0.198	0.253	4.10E-15	MG-Phago	PLAC8
NDUFAF3	2.78E-19	0.257165	0.224	0.286	7.84E-15	MG-Phago	NDUFAF3
DUT	2.96E-19	0.31716	0.238	0.307	8.32E-15	MG-Phago	DUT
BANF1	3.07E-19	0.295043	0.247	0.319	8.63E-15	MG-Phago	BANF1

UFC1	3.65E-19	0.288083	0.258	0.334	1.03E-14	MG-Phago	UFC1
UBXN1	6.44E-19	0.277891	0.365	0.485	1.81E-14	MG-Phago	UBXN1
ANAPC16	7.03E-19	0.252987	0.315	0.409	1.98E-14	MG-Phago	ANAPC16
LDHB	1.32E-18	0.345828	0.272	0.353	3.72E-14	MG-Phago	LDHB
GMFG	1.79E-18	0.264283	0.348	0.455	5.04E-14	MG-Phago	GMFG
VSIG4	2.17E-18	0.480914	0.469	0.549	6.09E-14	MG-Phago	VSIG4
PKM	2.24E-18	0.369397	0.362	0.475	6.30E-14	MG-Phago	PKM
ATP5MC1	2.82E-18	0.267379	0.199	0.255	7.94E-14	MG-Phago	ATP5MC1
HIST1H4C	4.29E-18	0.405117	0.262	0.339	1.21E-13	MG-Phago	HIST1H4C
COX6C	9.48E-18	0.311075	0.366	0.485	2.67E-13	MG-Phago	COX6C
GTF3A	1.33E-17	0.302615	0.239	0.305	3.74E-13	MG-Phago	GTF3A
EIF3H	1.53E-17	0.304161	0.377	0.5	4.30E-13	MG-Phago	EIF3H
VKORC1	1.60E-17	0.252306	0.202	0.258	4.49E-13	MG-Phago	VKORC1
SDHD	1.61E-17	0.266027	0.204	0.259	4.53E-13	MG-Phago	SDHD
C20orf27	1.97E-17	0.297238	0.233	0.298	5.53E-13	MG-Phago	C20orf27
GNAI2	1.97E-17	0.353511	0.573	0.698	5.55E-13	MG-Phago	GNAI2
C4orf3	3.54E-17	0.267478	0.258	0.333	9.96E-13	MG-Phago	C4orf3
ATP5PD	3.70E-17	0.293493	0.302	0.391	1.04E-12	MG-Phago	ATP5PD
APPL1	6.02E-17	0.283785	0.248	0.317	1.69E-12	MG-Phago	APPL1
CKLF	9.97E-17	0.292706	0.31	0.394	2.81E-12	MG-Phago	CKLF
ROMO1	1.70E-16	0.277976	0.223	0.282	4.78E-12	MG-Phago	ROMO1
SERPINB6	3.39E-16	0.318597	0.324	0.422	9.55E-12	MG-Phago	SERPINB6
TMEM176A	6.03E-16	0.47967	0.319	0.34	1.70E-11	MG-Phago	TMEM176A
CRTAP	1.39E-15	0.318316	0.27	0.345	3.91E-11	MG-Phago	CRTAP
NDUFS5	2.27E-15	0.290894	0.317	0.407	6.39E-11	MG-Phago	NDUFS5
PPP1CA	2.61E-15	0.335881	0.308	0.398	7.34E-11	MG-Phago	PPP1CA
TMEM258	3.02E-15	0.291319	0.31	0.398	8.51E-11	MG-Phago	TMEM258
C19orf53	7.64E-15	0.290217	0.263	0.333	2.15E-10	MG-Phago	C19orf53
TM9SF2	1.18E-14	0.362589	0.3	0.385	3.33E-10	MG-Phago	TM9SF2
RBX1	1.35E-14	0.314393	0.308	0.399	3.80E-10	MG-Phago	RBX1
PSME1	1.39E-14	0.295356	0.348	0.453	3.92E-10	MG-Phago	PSME1
NDUFB10	1.58E-14	0.298688	0.243	0.307	4.43E-10	MG-Phago	NDUFB10
FYB1	2.01E-14	0.28522	0.594	0.738	5.64E-10	MG-Phago	FYB1
AKR1A1	2.29E-14	0.292408	0.314	0.407	6.45E-10	MG-Phago	AKR1A1
NDUFS8	2.35E-14	0.306486	0.233	0.295	6.61E-10	MG-Phago	NDUFS8
MS4A4A	2.51E-14	0.298233	0.311	0.395	7.06E-10	MG-Phago	MS4A4A
PRDX5	3.91E-14	0.306003	0.267	0.339	1.10E-09	MG-Phago	PRDX5
NDUFB8	6.01E-14	0.315683	0.307	0.396	1.69E-09	MG-Phago	NDUFB8
NDUFS7	6.59E-14	0.322109	0.273	0.348	1.85E-09	MG-Phago	NDUFS7
CAPG	9.26E-14	0.467889	0.483	0.574	2.60E-09	MG-Phago	CAPG
CD163	1.03E-13	0.32347	0.347	0.348	2.90E-09	MG-Phago	CD163
SVBP	1.53E-13	0.275053	0.212	0.265	4.30E-09	MG-Phago	SVBP
MRPL57	2.08E-13	0.273345	0.203	0.253	5.86E-09	MG-Phago	MRPL57
CALR	5.04E-13	0.317282	0.46	0.594	1.42E-08	MG-Phago	CALR
ALDH2	5.75E-13	0.282624	0.365	0.473	1.62E-08	MG-Phago	ALDH2
ARF5	1.74E-12	0.310563	0.307	0.394	4.88E-08	MG-Phago	ARF5
KRTCAP2	2.96E-12	0.289207	0.39	0.51	8.33E-08	MG-Phago	KRTCAP2

ARPC5	3.88E-12	0.308823	0.384	0.502	1.09E-07	MG-Phago	ARPC5
SAMHD1	6.25E-12	0.509733	0.44	0.512	1.76E-07	MG-Phago	SAMHD1
ATP6V0C	7.54E-12	0.27414	0.586	0.723	2.12E-07	MG-Phago	ATP6V0C
C9orf16	1.11E-11	0.284164	0.284	0.357	3.13E-07	MG-Phago	C9orf16
ATP5ME	1.35E-11	0.263051	0.258	0.32	3.81E-07	MG-Phago	ATP5ME
MICOS13	1.67E-11	0.281797	0.212	0.262	4.71E-07	MG-Phago	MICOS13
GIMAP4	1.94E-11	0.418253	0.306	0.324	5.46E-07	MG-Phago	GIMAP4
GPX4	3.32E-11	0.363594	0.537	0.661	9.33E-07	MG-Phago	GPX4
PYCARD	5.80E-11	0.426851	0.427	0.497	1.63E-06	MG-Phago	PYCARD
LY86	1.11E-10	0.251125	0.45	0.595	3.12E-06	MG-Phago	LY86
OLFML3	2.09E-10	0.318816	0.346	0.434	5.88E-06	MG-Phago	OLFML3
EIF3K	2.95E-10	0.395087	0.495	0.6	8.30E-06	MG-Phago	EIF3K
TOMM6	3.12E-10	0.327897	0.301	0.381	8.77E-06	MG-Phago	TOMM6
SLC25A5	3.92E-10	0.306512	0.445	0.579	1.10E-05	MG-Phago	SLC25A5
ARL6IP4	4.04E-10	0.322154	0.365	0.474	1.14E-05	MG-Phago	ARL6IP4
UQCRQ	6.66E-10	0.326207	0.286	0.359	1.88E-05	MG-Phago	UQCRQ
UBL5	2.17E-09	0.303947	0.378	0.49	6.09E-05	MG-Phago	UBL5
SMDT1	3.57E-09	0.328566	0.3	0.376	0.000101	MG-Phago	SMDT1
AP2S1	3.66E-09	0.340216	0.287	0.36	0.000103	MG-Phago	AP2S1
LGALS9	5.55E-09	0.279269	0.438	0.579	0.000156	MG-Phago	LGALS9
NDUFA3	5.86E-09	0.283072	0.253	0.31	0.000165	MG-Phago	NDUFA3
H1FX	6.29E-09	0.300171	0.326	0.404	0.000177	MG-Phago	H1FX
CNBP	6.75E-09	0.340976	0.417	0.548	0.00019	MG-Phago	CNBP
HIGD2A	9.84E-09	0.308595	0.356	0.45	0.000277	MG-Phago	HIGD2A
CAP1	1.92E-08	0.325019	0.448	0.587	0.00054	MG-Phago	CAP1
DBNDD2	3.23E-08	0.285113	0.214	0.261	0.00091	MG-Phago	DBNDD2
NDUFB7	4.02E-08	0.308798	0.229	0.279	0.001131	MG-Phago	NDUFB7
AKR1B1	6.30E-08	0.308909	0.382	0.488	0.001774	MG-Phago	AKR1B1
NEDD8	6.35E-08	0.327039	0.301	0.378	0.001786	MG-Phago	NEDD8
SERPINF1	6.73E-08	0.482238	0.319	0.349	0.001894	MG-Phago	SERPINF1
RPL36AL	6.87E-08	0.327306	0.551	0.687	0.001932	MG-Phago	RPL36AL
GDI2	8.16E-08	0.417595	0.384	0.497	0.002295	MG-Phago	GDI2
HLA-DMB	1.14E-07	0.38199	0.469	0.561	0.003216	MG-Phago	HLA-DMB
EIF3F	1.32E-07	0.305479	0.411	0.53	0.003712	MG-Phago	EIF3F
LTC4S	2.41E-07	0.279196	0.422	0.536	0.006768	MG-Phago	LTC4S
TMEM14C	2.93E-07	0.349854	0.34	0.432	0.008245	MG-Phago	TMEM14C
SNHG29	3.14E-07	0.279727	0.446	0.577	0.008845	MG-Phago	SNHG29
ATP5PF	3.60E-07	0.352637	0.302	0.378	0.010139	MG-Phago	ATP5PF
PEBP1	5.59E-07	0.32427	0.325	0.41	0.015723	MG-Phago	PEBP1
ATP5F1D	5.88E-07	0.436516	0.418	0.495	0.016555	MG-Phago	ATP5F1D
BRI3	7.31E-07	0.261768	0.426	0.545	0.020564	MG-Phago	BRI3
ABI3	8.28E-07	0.366619	0.332	0.419	0.023286	MG-Phago	ABI3
SRP14	8.47E-07	0.35645	0.511	0.638	0.023844	MG-Phago	SRP14
YBX1	1.02E-06	0.355574	0.564	0.693	0.028752	MG-Phago	YBX1
SNX3	1.03E-06	0.292318	0.401	0.516	0.029049	MG-Phago	SNX3
FIS1	1.23E-06	0.318975	0.264	0.324	0.034599	MG-Phago	FIS1
SSR4	1.57E-06	0.317188	0.405	0.519	0.044194	MG-Phago	SSR4

PARK7	1.88E-06	0.373043	0.326	0.41	0.052908	MG-Phago	PARK7
ATP5MC3	3.56E-06	0.36653	0.361	0.458	0.100102	MG-Phago	ATP5MC3
GNG10	7.23E-06	0.326113	0.237	0.289	0.203471	MG-Phago	GNG10
NDUFB2	1.00E-05	0.48763	0.373	0.437	0.282476	MG-Phago	NDUFB2
YWHAB	1.48E-05	0.294445	0.464	0.609	0.417568	MG-Phago	YWHAB
TALDO1	1.96E-05	0.34891	0.308	0.381	0.550826	MG-Phago	TALDO1
ATP5MF	2.57E-05	0.348996	0.349	0.442	0.722561	MG-Phago	ATP5MF
ARL5A	2.79E-05	0.302019	0.415	0.528	0.786207	MG-Phago	ARL5A
TMEM219	3.03E-05	0.419996	0.381	0.451	0.85234	MG-Phago	TMEM219
C4orf48	3.14E-05	0.291094	0.238	0.283	0.882233	MG-Phago	C4orf48
CIAO2A	3.19E-05	0.32958	0.29	0.358	0.896975	MG-Phago	CIAO2A
CALM2	3.81E-05	0.252213	0.59	0.743	1	MG-Phago	CALM2
LAMTOR4	3.99E-05	0.399837	0.437	0.53	1	MG-Phago	LAMTOR4
H1F0	6.21E-05	0.293541	0.243	0.284	1	MG-Phago	H1F0
LAMTOR2	7.01E-05	0.325386	0.23	0.276	1	MG-Phago	LAMTOR2
ATP6V0E1	8.35E-05	0.251414	0.509	0.668	1	MG-Phago	ATP6V0E1
NENF	0.000112	0.311725	0.215	0.255	1	MG-Phago	NENF
APRT	0.000148	0.354995	0.313	0.386	1	MG-Phago	APRT
PSMB9	0.000169	0.321241	0.243	0.292	1	MG-Phago	PSMB9
ATP5IF1	0.000389	0.324812	0.3	0.365	1	MG-Phago	ATP5IF1
SYNGR2	0.000402	0.325385	0.434	0.562	1	MG-Phago	SYNGR2
SEC11A	0.000407	0.319523	0.417	0.53	1	MG-Phago	SEC11A
EDF1	0.000456	0.327972	0.398	0.506	1	MG-Phago	EDF1
SELENOW	0.000569	0.336965	0.28	0.341	1	MG-Phago	SELENOW
APMAP	0.000614	0.300953	0.42	0.533	1	MG-Phago	APMAP
TMEM119	0.000624	0.36866	0.337	0.41	1	MG-Phago	TMEM119
SPCS1	0.001291	0.352904	0.371	0.471	1	MG-Phago	SPCS1
HACD4	0.00156	0.345066	0.236	0.279	1	MG-Phago	HACD4
CSTB	0.001615	0.294222	0.356	0.436	1	MG-Phago	CSTB
POLD4	0.001638	0.333293	0.393	0.5	1	MG-Phago	POLD4
CORO1A	0.001714	0.322026	0.534	0.661	1	MG-Phago	CORO1A
NDUFA4	0.001715	0.420919	0.435	0.532	1	MG-Phago	NDUFA4
TNFSF13	0.002735	0.362041	0.29	0.354	1	MG-Phago	TNFSF13
HINT1	0.002757	0.443739	0.391	0.465	1	MG-Phago	HINT1
CTSD	0.002784	0.251509	0.568	0.693	1	MG-Phago	CTSD
COX6B1	0.002815	0.2963	0.449	0.573	1	MG-Phago	COX6B1
CHCHD2	0.004019	0.311528	0.471	0.601	1	MG-Phago	CHCHD2
PGLS	0.0059	0.369925	0.324	0.397	1	MG-Phago	PGLS
BLOC1S1	0.00667	0.421679	0.331	0.381	1	MG-Phago	BLOC1S1
BRK1	0.00833	0.402686	0.42	0.514	1	MG-Phago	BRK1
COX5A	0.008542	0.37977	0.35	0.432	1	MG-Phago	COX5A
RNASE2	0.009953	0.363125	0.229	0.266	1	MG-Phago	RNASE2
HSPA6	0	3.087428	0.478	0.151	0	MG-HSP	HSPA6
HSPA1A	0	2.505326	0.98	0.683	0	MG-HSP	HSPA1A
HSPA1B	0	2.3335	0.969	0.613	0	MG-HSP	HSPA1B
DNAJB1	0	2.269302	0.945	0.582	0	MG-HSP	DNAJB1
BAG3	0	2.144808	0.672	0.268	0	MG-HSP	BAG3

HSPH1	0	2.028112	0.838	0.395	0	MG-HSP	HSPH1
HSPB1	0	1.928514	0.716	0.417	0	MG-HSP	HSPB1
HSP90AA1	0	1.82117	0.977	0.831	0	MG-HSP	HSP90AA1
HSPD1	0	1.58065	0.753	0.45	0	MG-HSP	HSPD1
HSPE1	0	1.466829	0.741	0.431	0	MG-HSP	HSPE1
ZFAND2A	0	1.377537	0.441	0.159	0	MG-HSP	ZFAND2A
IER5	0	1.243674	0.684	0.494	0	MG-HSP	IER5
P4HA1	0	1.203564	0.583	0.272	0	MG-HSP	P4HA1
UBC	0	1.191694	0.966	0.907	0	MG-HSP	UBC
DNAJA4	0	1.101228	0.394	0.147	0	MG-HSP	DNAJA4
JUN	0	1.053242	0.953	0.848	0	MG-HSP	JUN
HSPA8	0	0.88751	0.902	0.747	0	MG-HSP	HSPA8
HSP90AB1	0	0.879215	0.905	0.828	0	MG-HSP	HSP90AB1
DNAJB4	0	0.852879	0.33	0.146	0	MG-HSP	DNAJB4
LRRC23	0	0.834426	0.267	0.132	0	MG-HSP	LRRC23
LNCAROD	0	0.802045	0.634	0.429	0	MG-HSP	LNCAROD
DNAJA1	0	0.779707	0.709	0.566	0	MG-HSP	DNAJA1
NAV3	0	0.767744	0.479	0.288	0	MG-HSP	NAV3
UBB	0	0.755648	0.758	0.661	0	MG-HSP	UBB
TECR	0	0.730996	0.513	0.368	0	MG-HSP	TECR
CHORDC1	0	0.674634	0.4	0.217	0	MG-HSP	CHORDC1
SFMBT2	0	0.647973	0.776	0.615	0	MG-HSP	SFMBT2
EGR1	0	0.645421	0.928	0.8	0	MG-HSP	EGR1
CACYBP	0	0.621031	0.359	0.2	0	MG-HSP	CACYBP
SYNDIG1	0	0.615258	0.583	0.399	0	MG-HSP	SYNDIG1
TCP1	0	0.586878	0.39	0.242	0	MG-HSP	TCP1
GNG7	0	0.582557	0.561	0.417	0	MG-HSP	GNG7
CPED1	0	0.578989	0.422	0.253	0	MG-HSP	CPED1
P2RY12	0	0.5631	0.648	0.504	0	MG-HSP	P2RY12
MRPL18	0	0.546973	0.312	0.179	0	MG-HSP	MRPL18
ATM	0	0.517438	0.504	0.369	0	MG-HSP	ATM
FRMD4A	0	0.506709	0.88	0.76	0	MG-HSP	FRMD4A
DSCAM	0	0.489069	0.425	0.278	0	MG-HSP	DSCAM
DOCK8	0	0.471166	0.774	0.675	0	MG-HSP	DOCK8
MEF2C	0	0.467601	0.828	0.727	0	MG-HSP	MEF2C
DLEU1	0	0.466073	0.658	0.523	0	MG-HSP	DLEU1
SORL1	0	0.460568	0.795	0.69	0	MG-HSP	SORL1
ANKRD44	0	0.455763	0.68	0.558	0	MG-HSP	ANKRD44
MEF2A	0	0.454081	0.863	0.772	0	MG-HSP	MEF2A
APBB1IP	0	0.435097	0.852	0.764	0	MG-HSP	APBB1IP
ST6GAL1	0	0.429735	0.763	0.63	0	MG-HSP	ST6GAL1
ARHGAP24	0	0.415915	0.82	0.707	0	MG-HSP	ARHGAP24
PLXDC2	0	0.270592	0.97	0.929	0	MG-HSP	PLXDC2
LRRK1	8.38E-302	0.493827	0.456	0.318	2.36E-297	MG-HSP	LRRK1
ARHGAP22	9.28E-298	0.42328	0.564	0.429	2.61E-293	MG-HSP	ARHGAP22
SSH2	3.98E-292	0.401114	0.709	0.601	1.12E-287	MG-HSP	SSH2
INPP5D	7.77E-292	0.436392	0.615	0.493	2.19E-287	MG-HSP	INPP5D

IL6ST	5.91E-284	0.439723	0.622	0.496	1.66E-279	MG-HSP	IL6ST
AL627171.:	3.21E-275	0.54714	0.355	0.227	9.03E-271	MG-HSP	AL627171.2
STIP1	3.93E-274	0.431016	0.323	0.201	1.11E-269	MG-HSP	STIP1
RASGEF1C	6.65E-271	0.485324	0.407	0.277	1.87E-266	MG-HSP	RASGEF1C
SNAP23	5.15E-269	0.481244	0.422	0.299	1.45E-264	MG-HSP	SNAP23
CSGALNAC⁻	1.49E-268	0.420366	0.619	0.498	4.20E-264	MG-HSP	CSGALNACT1
OXR1	2.36E-267	0.44956	0.547	0.416	6.64E-263	MG-HSP	OXR1
CX3CR11	3.89E-261	0.38032	0.683	0.554	1.10E-256	MG-HSP	CX3CR1
FGD2	8.24E-257	0.448085	0.386	0.263	2.32E-252	MG-HSP	FGD2
SLC1A3	2.02E-255	0.364813	0.85	0.75	5.68E-251	MG-HSP	SLC1A3
HS3ST4	2.63E-243	0.386231	0.326	0.205	7.40E-239	MG-HSP	HS3ST4
DIP2B	3.86E-239	0.392273	0.425	0.301	1.09E-234	MG-HSP	DIP2B
MCF2L	1.16E-230	0.414095	0.39	0.272	3.25E-226	MG-HSP	MCF2L
ADAM28	1.88E-228	0.37971	0.678	0.581	5.28E-224	MG-HSP	ADAM28
LINC01736	1.04E-220	0.355087	0.538	0.414	2.92E-216	MG-HSP	LINC01736
BLNK	2.79E-220	0.376594	0.45	0.331	7.84E-216	MG-HSP	BLNK
DNAJB6	1.69E-218	0.473984	0.53	0.431	4.75E-214	MG-HSP	DNAJB6
SELPLG	1.16E-217	0.363012	0.684	0.575	3.27E-213	MG-HSP	SELPLG
RCSD1	1.22E-217	0.414068	0.534	0.426	3.43E-213	MG-HSP	RCSD1
PICALM	2.65E-216	0.354043	0.75	0.675	7.45E-212	MG-HSP	PICALM
PALD1	6.36E-211	0.315916	0.64	0.54	1.79E-206	MG-HSP	PALD1
SYK	7.48E-210	0.365424	0.597	0.497	2.10E-205	MG-HSP	SYK
CHD9	1.08E-204	0.391574	0.562	0.46	3.03E-200	MG-HSP	CHD9
FAM135A	1.34E-204	0.407523	0.338	0.229	3.76E-200	MG-HSP	FAM135A
PWWP3A	1.82E-204	0.355202	0.28	0.177	5.11E-200	MG-HSP	PWWP3A
TGFBR2	1.65E-198	0.395295	0.575	0.477	4.64E-194	MG-HSP	TGFBR2
SLC9A9	8.96E-198	0.341085	0.521	0.412	2.52E-193	MG-HSP	SLC9A9
MYCBP2	4.29E-197	0.398022	0.567	0.473	1.21E-192	MG-HSP	MYCBP2
DISC1	2.58E-196	0.350099	0.476	0.366	7.27E-192	MG-HSP	DISC1
EPB41L2	3.84E-195	0.267928	0.806	0.725	1.08E-190	MG-HSP	EPB41L2
ATP2C1	1.80E-191	0.400679	0.483	0.377	5.07E-187	MG-HSP	ATP2C1
A2M1	4.16E-190	0.273904	0.832	0.738	1.17E-185	MG-HSP	A2M
ITPR2	1.74E-189	0.313812	0.683	0.59	4.88E-185	MG-HSP	ITPR2
ELMO1	6.65E-187	0.252113	0.814	0.747	1.87E-182	MG-HSP	ELMO1
RYR1	2.68E-184	0.351318	0.34	0.237	7.54E-180	MG-HSP	RYR1
IFNGR1	3.02E-183	0.317505	0.761	0.686	8.49E-179	MG-HSP	IFNGR1
GPR34	2.20E-182	0.305681	0.72	0.626	6.19E-178	MG-HSP	GPR34
TBXAS1	1.11E-176	0.292924	0.776	0.709	3.12E-172	MG-HSP	TBXAS1
FYB11	3.45E-174	0.275163	0.779	0.697	9.70E-170	MG-HSP	FYB1
MAP3K5	9.80E-172	0.358322	0.359	0.26	2.76E-167	MG-HSP	MAP3K5
TIAM1	1.50E-171	0.34349	0.315	0.216	4.23E-167	MG-HSP	TIAM1
JAK2	1.54E-171	0.345289	0.38	0.278	4.33E-167	MG-HSP	JAK2
HDAC9	3.54E-168	0.312404	0.504	0.4	9.97E-164	MG-HSP	HDAC9
LINC02712	1.76E-160	0.358313	0.317	0.221	4.95E-156	MG-HSP	LINC02712
LPCAT2	3.97E-156	0.289523	0.73	0.648	1.12E-151	MG-HSP	LPCAT2
MED13L	2.54E-155	0.290653	0.557	0.468	7.16E-151	MG-HSP	MED13L
APPL2	2.14E-153	0.323618	0.356	0.262	6.03E-149	MG-HSP	APPL2

FAM13A	1.52E-150	0.324841	0.289	0.199	4.27E-146	MG-HSP	FAM13A
ZBTB20	3.92E-150	0.319901	0.502	0.41	1.10E-145	MG-HSP	ZBTB20
AHSA1	8.39E-150	0.302232	0.281	0.193	2.36E-145	MG-HSP	AHSA1
FLI1	4.36E-148	0.317974	0.364	0.272	1.23E-143	MG-HSP	FLI1
KHDRBS3	4.76E-147	0.378434	0.377	0.283	1.34E-142	MG-HSP	KHDRBS3
ZFHX3	5.63E-147	0.290299	0.594	0.513	1.58E-142	MG-HSP	ZFHX3
GRID2	7.17E-147	0.441758	0.307	0.211	2.02E-142	MG-HSP	GRID2
MTRNR2L1	8.72E-147	0.306318	0.597	0.483	2.45E-142	MG-HSP	MTRNR2L12
MAF	3.20E-146	0.32538	0.342	0.251	9.00E-142	MG-HSP	MAF
DIAPH2	4.52E-145	0.309677	0.508	0.418	1.27E-140	MG-HSP	DIAPH2
CLK1	2.33E-142	0.395856	0.457	0.375	6.55E-138	MG-HSP	CLK1
SLCO2B1	3.76E-139	0.278153	0.621	0.533	1.06E-134	MG-HSP	SLCO2B1
SERPINE11	4.46E-139	0.573681	0.502	0.4	1.26E-134	MG-HSP	SERPINE1
FAM149A	2.62E-138	0.314075	0.33	0.242	7.38E-134	MG-HSP	FAM149A
FTX	4.37E-137	0.278041	0.338	0.246	1.23E-132	MG-HSP	FTX
HTRA1	1.89E-133	0.278867	0.707	0.616	5.33E-129	MG-HSP	HTRA1
FCHSD2	6.66E-133	0.252932	0.546	0.461	1.88E-128	MG-HSP	FCHSD2
ST6GALNA(	6.21E-131	0.266597	0.637	0.543	1.75E-126	MG-HSP	ST6GALNAC3
IER2	2.97E-129	0.366508	0.876	0.827	8.37E-125	MG-HSP	IER2
HSPA4	1.91E-126	0.312701	0.285	0.205	5.37E-122	MG-HSP	HSPA4
TRA2B	2.45E-126	0.396704	0.619	0.562	6.89E-122	MG-HSP	TRA2B
ENTPD1-AS	3.24E-126	0.309463	0.265	0.185	9.12E-122	MG-HSP	ENTPD1-AS1
TBC1D12	5.22E-126	0.322462	0.381	0.298	1.47E-121	MG-HSP	TBC1D12
MIR99AHG	4.71E-123	0.276265	0.284	0.202	1.33E-118	MG-HSP	MIR99AHG
TBC1D5	8.55E-123	0.278144	0.47	0.387	2.41E-118	MG-HSP	TBC1D5
1-Mar	2.82E-122	0.310562	0.367	0.281	7.95E-118	MG-HSP	1-Mar
BMP2K	3.33E-121	0.276835	0.545	0.466	9.37E-117	MG-HSP	BMP2K
ARHGAP15	3.53E-121	0.257552	0.63	0.561	9.93E-117	MG-HSP	ARHGAP15
DHRS9	7.19E-121	0.328838	0.303	0.217	2.02E-116	MG-HSP	DHRS9
ATP8B4	1.19E-120	0.280919	0.529	0.449	3.35E-116	MG-HSP	ATP8B4
AC022217.	1.25E-120	0.428163	0.489	0.416	3.53E-116	MG-HSP	AC022217.3
LINC02798	1.30E-120	0.267615	0.406	0.318	3.67E-116	MG-HSP	LINC02798
RAPGEF6	6.08E-120	0.30463	0.363	0.28	1.71E-115	MG-HSP	RAPGEF6
USP24	8.21E-120	0.28536	0.27	0.191	2.31E-115	MG-HSP	USP24
PCNX2	3.67E-119	0.296765	0.358	0.276	1.03E-114	MG-HSP	PCNX2
KANSL1	6.24E-117	0.269326	0.332	0.25	1.76E-112	MG-HSP	KANSL1
SHTN1	1.28E-115	0.288176	0.493	0.413	3.60E-111	MG-HSP	SHTN1
FEZ2	6.33E-114	0.358468	0.37	0.292	1.78E-109	MG-HSP	FEZ2
WDFY3	1.38E-112	0.268341	0.305	0.226	3.89E-108	MG-HSP	WDFY3
DLEU7	1.77E-112	0.303764	0.252	0.178	4.97E-108	MG-HSP	DLEU7
PTGES3	3.32E-112	0.268288	0.541	0.478	9.34E-108	MG-HSP	PTGES3
MAP4K4	6.10E-112	0.271003	0.439	0.361	1.72E-107	MG-HSP	MAP4K4
HECA	1.48E-111	0.355345	0.301	0.225	4.15E-107	MG-HSP	HECA
WASHC4	1.11E-109	0.30018	0.38	0.304	3.12E-105	MG-HSP	WASHC4
LPAR5	9.96E-109	0.271154	0.366	0.286	2.80E-104	MG-HSP	LPAR5
ZNF846	3.27E-107	0.288764	0.305	0.23	9.19E-103	MG-HSP	ZNF846
PKN2	4.29E-107	0.288289	0.39	0.314	1.21E-102	MG-HSP	PKN2

ZNRF2	4.93E-106	0.264799	0.332	0.255	1.39E-101	MG-HSP	ZNRF2
NCK2	1.74E-105	0.262662	0.442	0.363	4.90E-101	MG-HSP	NCK2
BNC2	1.67E-104	0.253567	0.372	0.291	4.70E-100	MG-HSP	BNC2
USP6NL	3.58E-104	0.282631	0.313	0.238	1.01E-99	MG-HSP	USP6NL
WDFY2	2.64E-103	0.266859	0.354	0.278	7.42E-99	MG-HSP	WDFY2
UVRAG	2.48E-102	0.263708	0.442	0.37	6.98E-98	MG-HSP	UVRAG
ARHGAP12	1.69E-99	0.264124	0.346	0.269	4.74E-95	MG-HSP	ARHGAP12
MPZL1	1.37E-98	0.272619	0.283	0.212	3.84E-94	MG-HSP	MPZL1
RNF213	7.77E-98	0.252137	0.513	0.442	2.19E-93	MG-HSP	RNF213
FER	1.54E-97	0.284512	0.285	0.212	4.33E-93	MG-HSP	FER
ARHGAP25	2.08E-96	0.259446	0.342	0.265	5.86E-92	MG-HSP	ARHGAP25
TAOK3	5.49E-96	0.264135	0.51	0.442	1.54E-91	MG-HSP	TAOK3
ST3GAL6	2.44E-93	0.285863	0.307	0.237	6.86E-89	MG-HSP	ST3GAL6
MBNL2	3.30E-92	0.256767	0.277	0.207	9.27E-88	MG-HSP	MBNL2
CCT4	5.37E-91	0.296829	0.402	0.34	1.51E-86	MG-HSP	CCT4
PLCL2	5.81E-91	0.265682	0.305	0.234	1.63E-86	MG-HSP	PLCL2
DCAF6	6.94E-91	0.253028	0.308	0.239	1.95E-86	MG-HSP	DCAF6
L3MBTL4	1.72E-90	0.256478	0.252	0.186	4.85E-86	MG-HSP	L3MBTL4
DIP2A	5.00E-90	0.264606	0.292	0.223	1.41E-85	MG-HSP	DIP2A
ACY3	2.71E-89	0.285746	0.259	0.191	7.62E-85	MG-HSP	ACY3
MGAT4A	4.89E-89	0.250695	0.537	0.476	1.38E-84	MG-HSP	MGAT4A
CCDC18-AS	9.34E-89	0.272453	0.374	0.306	2.63E-84	MG-HSP	CCDC18-AS1
IL17RA	4.04E-86	0.273903	0.457	0.395	1.14E-81	MG-HSP	IL17RA
LINC01374	1.92E-85	0.271545	0.35	0.282	5.41E-81	MG-HSP	LINC01374
TLN2	1.02E-84	0.266848	0.295	0.228	2.87E-80	MG-HSP	TLN2
SESN1	7.97E-83	0.26623	0.36	0.291	2.24E-78	MG-HSP	SESN1
LINC02256	1.20E-80	0.268464	0.42	0.357	3.38E-76	MG-HSP	LINC02256
STX7	1.48E-80	0.257491	0.373	0.31	4.16E-76	MG-HSP	STX7
TLR4	9.74E-72	0.351381	0.269	0.21	2.74E-67	MG-HSP	TLR4
SEMA4D	3.59E-71	0.255368	0.328	0.268	1.01E-66	MG-HSP	SEMA4D
HMOX1	3.50E-65	0.348545	0.587	0.54	9.85E-61	MG-HSP	HMOX1
SQSTM1	4.71E-56	0.315846	0.621	0.583	1.33E-51	MG-HSP	SQSTM1
RGS2	4.18E-45	0.28929	0.593	0.559	1.18E-40	MG-HSP	RGS2
SLC38A2	6.96E-45	0.282984	0.521	0.48	1.96E-40	MG-HSP	SLC38A2
DDIT4	1.25E-40	0.290338	0.564	0.528	3.52E-36	MG-HSP	DDIT4
TAGAP	6.76E-33	0.324959	0.528	0.501	1.90E-28	MG-HSP	TAGAP
OTUD11	2.77E-19	0.304467	0.574	0.562	7.80E-15	MG-HSP	OTUD1
GLA	8.00E-12	0.2656	0.276	0.264	2.25E-07	MG-HSP	GLA
MYLIP	1.57E-11	0.250674	0.364	0.351	4.41E-07	MG-HSP	MYLIP
CCDC200	0	2.220546	0.569	0.2	0	MG-Inflam	CCDC200
LINC00910	0	1.984346	0.838	0.487	0	MG-Inflam	LINC00910
C12orf57	0	1.866736	0.732	0.394	0	MG-Inflam	C12orf57
AC007952.	0	1.760732	0.69	0.347	0	MG-Inflam	AC007952.4
AC245014.	0	1.735451	0.758	0.389	0	MG-Inflam	AC245014.3
Z93241.1	0	1.680094	0.632	0.271	0	MG-Inflam	Z93241.1
AL021155.!	0	1.650145	0.604	0.228	0	MG-Inflam	AL021155.5
WDR74	0	1.62925	0.736	0.378	0	MG-Inflam	WDR74

CROCC	0	1.62872	0.717	0.378	0	MG-Inflam CROCC
TEX14	0	1.622657	0.947	0.727	0	MG-Inflam TEX14
TMEM107	0	1.619373	0.679	0.377	0	MG-Inflam TMEM107
AC253572.	0	1.564939	0.579	0.289	0	MG-Inflam AC253572.2
TNF1	0	1.399088	0.743	0.4	0	MG-Inflam TNF
IFIT2	0	1.346155	0.391	0.16	0	MG-Inflam IFIT2
AC012447.	0	1.29006	0.605	0.292	0	MG-Inflam AC012447.1
AC020916.	0	1.017547	0.825	0.547	0	MG-Inflam AC020916.1
PTCH2	0	1.005574	0.396	0.134	0	MG-Inflam PTCH2
AC103591.	0	0.865433	0.451	0.235	0	MG-Inflam AC103591.3
AC239799.	0	0.85055	0.304	0.148	0	MG-Inflam AC239799.2
NFKBIZ	0	0.82627	0.765	0.498	0	MG-Inflam NFKBIZ
NFKBIA1	0	0.815384	0.963	0.81	0	MG-Inflam NFKBIA
CREB1	0	0.773317	0.505	0.266	0	MG-Inflam CREB1
MYOSLID	0	0.766162	0.395	0.128	0	MG-Inflam MYOSLID
AC084871.	0	0.76325	0.427	0.256	0	MG-Inflam AC084871.1
TUBB4B1	0	0.753602	0.607	0.399	0	MG-Inflam TUBB4B
CH25H1	0	0.753499	0.686	0.437	0	MG-Inflam CH25H
DDIT41	0	0.732284	0.708	0.505	0	MG-Inflam DDIT4
AL136987.:	0	0.717563	0.471	0.267	0	MG-Inflam AL136987.1
LINC00623	0	0.70532	0.35	0.203	0	MG-Inflam LINC00623
PDK41	0	0.678715	0.79	0.583	0	MG-Inflam PDK4
FOS	0	0.666636	0.998	0.966	0	MG-Inflam FOS
LINC01220	0	0.660645	0.331	0.111	0	MG-Inflam LINC01220
IER21	0	0.647789	0.959	0.814	0	MG-Inflam IER2
1-Jun	0	0.633084	0.966	0.847	0	MG-Inflam JUN
AC046195.	0	0.63284	0.455	0.247	0	MG-Inflam AC046195.1
ENSA1	0	0.619206	0.804	0.607	0	MG-Inflam ENSA
SSPN1	0	0.614725	0.502	0.288	0	MG-Inflam SSPN
TAGAP1	0	0.612112	0.683	0.476	0	MG-Inflam TAGAP
TAL1	0	0.596933	0.536	0.293	0	MG-Inflam TAL1
MYLIP1	0	0.596348	0.543	0.322	0	MG-Inflam MYLIP
THUMPD3-	0	0.588776	0.433	0.223	0	MG-Inflam THUMPD3-AS1
CCL41	0	0.578597	0.969	0.833	0	MG-Inflam CCL4
AL691403.:	0	0.571887	0.603	0.393	0	MG-Inflam AL691403.1
IER5L	0	0.57164	0.44	0.258	0	MG-Inflam IER5L
CITED21	0	0.569684	0.676	0.475	0	MG-Inflam CITED2
NSUN6	0	0.565207	0.316	0.126	0	MG-Inflam NSUN6
SNHG12	0	0.558041	0.395	0.2	0	MG-Inflam SNHG12
CCL31	0	0.55419	0.976	0.853	0	MG-Inflam CCL3
PMAIP11	0	0.553748	0.467	0.273	0	MG-Inflam PMAIP1
AL390957.:	0	0.541929	0.493	0.306	0	MG-Inflam AL390957.1
EIF4A3	0	0.540688	0.549	0.367	0	MG-Inflam EIF4A3
ADRB2	0	0.538483	0.486	0.267	0	MG-Inflam ADRB2
LINC02642	0	0.533832	0.312	0.163	0	MG-Inflam LINC02642
IFNGR11	0	0.533132	0.834	0.675	0	MG-Inflam IFNGR1
LINC01091	0	0.527416	0.372	0.177	0	MG-Inflam LINC01091

EGR11	0	0.524253	0.947	0.798	0	MG-Inflam	EGR1
CXCL81	0	0.521941	0.669	0.478	0	MG-Inflam	CXCL8
KMT2E-AS1	0	0.519307	0.379	0.18	0	MG-Inflam	KMT2E-AS1
SLC38A21	0	0.519194	0.664	0.458	0	MG-Inflam	SLC38A2
AF111167.1	0	0.515088	0.307	0.113	0	MG-Inflam	AF111167.1
NEU1	0	0.506575	0.534	0.338	0	MG-Inflam	NEU1
AC091271.	0	0.505329	0.322	0.143	0	MG-Inflam	AC091271.1
KLF7	0	0.500935	0.509	0.303	0	MG-Inflam	KLF7
AC243829.	0	0.498704	0.35	0.157	0	MG-Inflam	AC243829.4
GADD45B1	0	0.497942	0.914	0.755	0	MG-Inflam	GADD45B
P2RY121	0	0.496959	0.707	0.496	0	MG-Inflam	P2RY12
IPCEF11	0	0.496679	0.691	0.479	0	MG-Inflam	IPCEF1
LINC01366	0	0.490332	0.299	0.158	0	MG-Inflam	LINC01366
SERPINB9	0	0.487732	0.782	0.614	0	MG-Inflam	SERPINB9
RGS11	0	0.482532	0.959	0.822	0	MG-Inflam	RGS1
AC243829.	0	0.481471	0.288	0.125	0	MG-Inflam	AC243829.1
HEXIM1	0	0.475802	0.275	0.122	0	MG-Inflam	HEXIM1
MIR4713H	0	0.475159	0.303	0.148	0	MG-Inflam	MIR4713HG
PEAK11	0	0.472035	0.673	0.471	0	MG-Inflam	PEAK1
MEPCE	0	0.471161	0.279	0.11	0	MG-Inflam	MEPCE
CLEC7A1	0	0.469965	0.762	0.605	0	MG-Inflam	CLEC7A
ATP2B1-AS	0	0.469506	0.669	0.475	0	MG-Inflam	ATP2B1-AS1
SQSTM11	0	0.464218	0.718	0.567	0	MG-Inflam	SQSTM1
CEP68	0	0.461958	0.361	0.205	0	MG-Inflam	CEP68
IER31	0	0.452276	0.908	0.749	0	MG-Inflam	IER3
TSC22D31	0	0.450243	0.855	0.696	0	MG-Inflam	TSC22D3
DHRS91	0	0.449996	0.364	0.208	0	MG-Inflam	DHRS9
DUSP11	0	0.445456	0.993	0.935	0	MG-Inflam	DUSP1
BTG2	0	0.441553	0.897	0.72	0	MG-Inflam	BTG2
ODC1	0	0.43976	0.435	0.263	0	MG-Inflam	ODC1
SOD21	0	0.43849	0.751	0.554	0	MG-Inflam	SOD2
AC087239.	0	0.438433	0.347	0.167	0	MG-Inflam	AC087239.1
SNHG1	0	0.436216	0.378	0.214	0	MG-Inflam	SNHG1
AC004687.	0	0.435284	0.439	0.257	0	MG-Inflam	AC004687.1
HIST2H2BF	0	0.432125	0.292	0.116	0	MG-Inflam	HIST2H2BF
RIPK1	0	0.426796	0.376	0.221	0	MG-Inflam	RIPK1
GPR341	0	0.419207	0.805	0.613	0	MG-Inflam	GPR34
PATL2	0	0.412063	0.288	0.127	0	MG-Inflam	PATL2
WSB1	0	0.410459	0.846	0.692	0	MG-Inflam	WSB1
AC020911.	0	0.404022	0.263	0.107	0	MG-Inflam	AC020911.2
CCL4L21	0	0.403002	0.919	0.765	0	MG-Inflam	CCL4L2
AZIN1-AS1	0	0.402426	0.52	0.334	0	MG-Inflam	AZIN1-AS1
MT-ATP6	0	0.398461	0.935	0.844	0	MG-Inflam	MT-ATP6
PPP1R10	0	0.39732	0.616	0.422	0	MG-Inflam	PPP1R10
FCGR1B	0	0.394763	0.435	0.262	0	MG-Inflam	FCGR1B
FAM53C	0	0.391781	0.453	0.28	0	MG-Inflam	FAM53C
AC106865.	0	0.388868	0.316	0.174	0	MG-Inflam	AC106865.2

SOCS1	0	0.38847	0.25	0.118	0	MG-Inflam	SOCS1
AC002451.	0	0.381628	0.255	0.116	0	MG-Inflam	AC002451.1
MIR222HG	0	0.38126	0.446	0.265	0	MG-Inflam	MIR222HG
AL499604.:	0	0.380825	0.313	0.162	0	MG-Inflam	AL499604.1
ADGRG1	0	0.378133	0.507	0.317	0	MG-Inflam	ADGRG1
YME1L1	0	0.376992	0.615	0.436	0	MG-Inflam	YME1L1
DNAJB41	0	0.373727	0.299	0.153	0	MG-Inflam	DNAJB4
INTS6	0	0.371416	0.471	0.3	0	MG-Inflam	INTS6
AL512603.:	0	0.370356	0.352	0.193	0	MG-Inflam	AL512603.2
AC022217.	0	0.358332	0.588	0.401	0	MG-Inflam	AC022217.3
KLF61	0	0.357509	0.975	0.883	0	MG-Inflam	KLF6
MTRNR2L1	0	0.357324	0.695	0.468	0	MG-Inflam	MTRNR2L12
C2orf76	0	0.336276	0.29	0.151	0	MG-Inflam	C2orf76
TNFAIP31	0	0.332763	0.729	0.523	0	MG-Inflam	TNFAIP3
LINC01004	0	0.326624	0.286	0.139	0	MG-Inflam	LINC01004
CCL3L11	5.99E-308	0.359019	0.88	0.718	1.68E-303	MG-Inflam	CCL3L1
AF213884.:	1.27E-307	0.388926	0.406	0.247	3.58E-303	MG-Inflam	AF213884.3
RPL41	1.81E-307	0.289825	0.927	0.801	5.08E-303	MG-Inflam	RPL41
CD831	3.41E-305	0.284078	0.982	0.901	9.58E-301	MG-Inflam	CD83
C3AR11	5.04E-301	0.3379	0.787	0.61	1.42E-296	MG-Inflam	C3AR1
SNHG25	5.74E-300	0.356083	0.327	0.186	1.61E-295	MG-Inflam	SNHG25
JUND1	8.67E-292	0.360799	0.911	0.783	2.44E-287	MG-Inflam	JUND
P2RY13	5.37E-290	0.352686	0.489	0.321	1.51E-285	MG-Inflam	P2RY13
MIR29B2CI	3.21E-289	0.2833	0.324	0.182	9.04E-285	MG-Inflam	MIR29B2CHG
GTF2A1	3.09E-287	0.285314	0.269	0.142	8.69E-283	MG-Inflam	GTF2A1
ZNF143	3.67E-285	0.270015	0.26	0.135	1.03E-280	MG-Inflam	ZNF143
MCF2L21	5.61E-285	0.407319	0.506	0.344	1.58E-280	MG-Inflam	MCF2L2
SGK11	1.80E-280	0.380692	0.952	0.848	5.06E-276	MG-Inflam	SGK1
NXF1	1.98E-277	0.313492	0.376	0.23	5.58E-273	MG-Inflam	NXF1
LPAR51	2.19E-271	0.282998	0.433	0.276	6.15E-267	MG-Inflam	LPAR5
MAFB	5.93E-270	0.49357	0.713	0.578	1.67E-265	MG-Inflam	MAFB
KLF21	1.77E-269	0.28408	0.88	0.693	4.99E-265	MG-Inflam	KLF2
ARRDC3	6.48E-267	0.301239	0.287	0.159	1.82E-262	MG-Inflam	ARRDC3
NUFIP2	1.06E-259	0.354994	0.579	0.422	2.97E-255	MG-Inflam	NUFIP2
TRA2B1	1.04E-258	0.3599	0.705	0.548	2.92E-254	MG-Inflam	TRA2B
SRSF7	1.69E-258	0.382867	0.757	0.61	4.75E-254	MG-Inflam	SRSF7
BHLHE41	1.91E-257	0.316217	0.689	0.514	5.38E-253	MG-Inflam	BHLHE41
CTTNBP2	3.26E-257	0.330508	0.525	0.36	9.17E-253	MG-Inflam	CTTNBP2
MEF2C-AS1	3.09E-253	0.255318	0.299	0.17	8.71E-249	MG-Inflam	MEF2C-AS1
CX3CR12	7.19E-250	0.289281	0.725	0.549	2.02E-245	MG-Inflam	CX3CR1
AL355881.:	1.45E-247	0.360187	0.291	0.168	4.08E-243	MG-Inflam	AL355881.1
CDK6	1.57E-246	0.285012	0.384	0.24	4.42E-242	MG-Inflam	CDK6
HSPA81	2.20E-246	0.352306	0.87	0.754	6.19E-242	MG-Inflam	HSPA8
SPATA13	2.20E-246	0.288752	0.567	0.403	6.19E-242	MG-Inflam	SPATA13
EGR21	4.53E-246	0.33991	0.66	0.48	1.27E-241	MG-Inflam	EGR2
FCGR1A1	6.76E-244	0.31408	0.654	0.485	1.90E-239	MG-Inflam	FCGR1A
SLC31A2	4.28E-243	0.390323	0.504	0.357	1.20E-238	MG-Inflam	SLC31A2

RASGEF1B	1.35E-241	0.378035	0.766	0.628	3.80E-237	MG-Inflam	RASGEF1B
EIF4A2	3.07E-240	0.31245	0.642	0.486	8.65E-236	MG-Inflam	EIF4A2
BRD2	1.60E-234	0.291837	0.606	0.446	4.51E-230	MG-Inflam	BRD2
GSTM3	2.78E-231	0.314249	0.364	0.229	7.83E-227	MG-Inflam	GSTM3
B4GALT1	8.91E-229	0.281836	0.731	0.584	2.51E-224	MG-Inflam	B4GALT1
JDP21	1.10E-228	0.289283	0.706	0.548	3.09E-224	MG-Inflam	JDP2
COPB2	6.96E-228	0.261189	0.377	0.242	1.96E-223	MG-Inflam	COPB2
AC012150.	1.54E-226	0.269043	0.288	0.167	4.33E-222	MG-Inflam	AC012150.1
CKS21	1.89E-226	0.298801	0.417	0.272	5.30E-222	MG-Inflam	CKS2
IL6ST1	4.16E-226	0.268094	0.659	0.491	1.17E-221	MG-Inflam	IL6ST
TMEM52B:	5.60E-225	0.277584	0.44	0.294	1.58E-220	MG-Inflam	TMEM52B
SKIL1	1.82E-224	0.295183	0.801	0.655	5.13E-220	MG-Inflam	SKIL
OAT	2.01E-224	0.337752	0.286	0.169	5.64E-220	MG-Inflam	OAT
LIPA	1.16E-222	0.324321	0.455	0.315	3.25E-218	MG-Inflam	LIPA
TLR41	1.25E-220	0.314085	0.328	0.202	3.52E-216	MG-Inflam	TLR4
HECA1	2.19E-219	0.29639	0.346	0.219	6.16E-215	MG-Inflam	HECA
SMAP2	5.10E-219	0.343639	0.867	0.754	1.43E-214	MG-Inflam	SMAP2
AC025164.	5.82E-218	0.31652	0.254	0.144	1.64E-213	MG-Inflam	AC025164.1
SERPINE12	1.56E-217	0.502142	0.537	0.395	4.38E-213	MG-Inflam	SERPINE1
HTRA11	3.33E-215	0.293041	0.774	0.606	9.38E-211	MG-Inflam	HTRA1
LGMN	4.34E-215	0.280296	0.558	0.404	1.22E-210	MG-Inflam	LGMN
DDX50	1.35E-214	0.25846	0.343	0.217	3.81E-210	MG-Inflam	DDX50
HSPA5	1.45E-213	0.301397	0.716	0.564	4.08E-209	MG-Inflam	HSPA5
CKB1	2.08E-212	0.305052	0.53	0.374	5.86E-208	MG-Inflam	CKB
ARMH1	2.32E-209	0.450068	0.307	0.188	6.53E-205	MG-Inflam	ARMH1
PLK31	3.96E-202	0.274362	0.589	0.436	1.12E-197	MG-Inflam	PLK3
KCTD12	7.55E-201	0.256434	0.706	0.559	2.12E-196	MG-Inflam	KCTD12
WTAP	1.41E-196	0.28247	0.55	0.409	3.96E-192	MG-Inflam	WTAP
SPTAN1	2.91E-195	0.284399	0.352	0.232	8.20E-191	MG-Inflam	SPTAN1
SRSF31	2.85E-194	0.265041	0.672	0.519	8.02E-190	MG-Inflam	SRSF3
UBE2S	5.26E-193	0.351076	0.376	0.255	1.48E-188	MG-Inflam	UBE2S
RHOB1	6.81E-192	0.348721	0.923	0.811	1.92E-187	MG-Inflam	RHOB
RPL22L1	3.78E-190	0.278225	0.587	0.443	1.06E-185	MG-Inflam	RPL22L1
IFI61	2.73E-186	0.537796	0.381	0.266	7.69E-182	MG-Inflam	IFI6
CXorf21	2.09E-181	0.269305	0.379	0.254	5.89E-177	MG-Inflam	CXorf21
AC079015.	7.84E-181	0.255375	0.265	0.16	2.21E-176	MG-Inflam	AC079015.1
SPRY11	9.32E-178	0.377576	0.355	0.236	2.62E-173	MG-Inflam	SPRY1
CCR5AS	3.80E-177	0.263816	0.308	0.198	1.07E-172	MG-Inflam	CCR5AS
TMIGD31	4.75E-176	0.307979	0.517	0.376	1.34E-171	MG-Inflam	TMIGD3
ARID5B1	8.20E-175	0.480601	0.524	0.401	2.31E-170	MG-Inflam	ARID5B
LINC01010	2.86E-172	0.331152	0.313	0.205	8.04E-168	MG-Inflam	LINC01010
SPIDR	1.15E-163	0.263513	0.606	0.473	3.24E-159	MG-Inflam	SPIDR
MAP4K31	1.32E-161	0.294546	0.623	0.493	3.71E-157	MG-Inflam	MAP4K3
RBBP6	1.16E-154	0.281262	0.462	0.342	3.27E-150	MG-Inflam	RBBP6
FOLR2	6.19E-150	0.288532	0.281	0.18	1.74E-145	MG-Inflam	FOLR2
IFI44L	6.34E-150	0.296999	0.337	0.229	1.79E-145	MG-Inflam	IFI44L
SPP11	1.52E-149	0.298803	0.919	0.818	4.29E-145	MG-Inflam	SPP1

AL138720.:	3.94E-140	0.280796	0.255	0.164	1.11E-135	MG-Inflam	AL138720.1
GADD45G1	3.00E-137	0.292668	0.298	0.2	8.45E-133	MG-Inflam	GADD45G
DUSP61	1.74E-135	0.297572	0.381	0.273	4.90E-131	MG-Inflam	DUSP6
KLHL61	6.08E-135	0.286441	0.531	0.415	1.71E-130	MG-Inflam	KLHL6
DUSP21	2.58E-91	0.28533	0.485	0.384	7.26E-87	MG-Inflam	DUSP2
PLTP	2.16E-32	0.275485	0.254	0.207	6.07E-28	MG-Inflam	PLTP
LRMDA	0	1.947621	0.854	0.746	0	TAM-RNA S	LRMDA
KCNQ3	0	1.942532	0.656	0.492	0	TAM-RNA S	KCNQ3
AUTS2	0	1.848259	0.506	0.301	0	TAM-RNA S	AUTS2
NEAT1	0	1.782138	0.93	0.902	0	TAM-RNA S	NEAT1
NHSL1	0	1.699684	0.461	0.315	0	TAM-RNA S	NHSL1
CHST11	0	1.679246	0.747	0.68	0	TAM-RNA S	CHST11
LDLRAD4	0	1.674598	0.88	0.818	0	TAM-RNA S	LDLRAD4
FRMD4A1	0	1.662237	0.865	0.772	0	TAM-RNA S	FRMD4A
ELMO11	0	1.641778	0.828	0.752	0	TAM-RNA S	ELMO1
PDE3B	0	1.637018	0.557	0.431	0	TAM-RNA S	PDE3B
ST6GALNA(	0	1.617279	0.662	0.549	0	TAM-RNA S	ST6GALNAC3
DOCK4	0	1.592091	0.949	0.894	0	TAM-RNA S	DOCK4
ZSWIM6	0	1.582839	0.709	0.614	0	TAM-RNA S	ZSWIM6
SH3RF3	0	1.57789	0.477	0.319	0	TAM-RNA S	SH3RF3
ARHGAP26	0	1.570076	0.783	0.696	0	TAM-RNA S	ARHGAP26
ANKRD11	0	1.568441	0.629	0.532	0	TAM-RNA S	ANKRD11
ABR	0	1.564723	0.64	0.532	0	TAM-RNA S	ABR
ACSL1	0	1.531318	0.685	0.663	0	TAM-RNA S	ACSL1
FOXN3	0	1.500862	0.747	0.688	0	TAM-RNA S	FOXN3
SFMBT21	0	1.492293	0.718	0.633	0	TAM-RNA S	SFMBT2
CSGALNAC	0	1.490774	0.601	0.51	0	TAM-RNA S	CSGALNACT1
ARHGAP24	0	1.46537	0.775	0.72	0	TAM-RNA S	ARHGAP24
FGD4	0	1.463395	0.739	0.679	0	TAM-RNA S	FGD4
SRGAP2B	0	1.451926	0.631	0.518	0	TAM-RNA S	SRGAP2B
ELL21	0	1.434695	0.825	0.787	0	TAM-RNA S	ELL2
CELF2	0	1.432492	0.8	0.761	0	TAM-RNA S	CELF2
ZFAND3	0	1.421683	0.607	0.496	0	TAM-RNA S	ZFAND3
SRGAP2	0	1.416179	0.786	0.721	0	TAM-RNA S	SRGAP2
MAML3	0	1.395634	0.632	0.553	0	TAM-RNA S	MAML3
RAB31	0	1.393861	0.743	0.759	0	TAM-RNA S	RAB31
PLXDC21	0	1.386764	0.949	0.934	0	TAM-RNA S	PLXDC2
AKAP131	0	1.355389	0.811	0.778	0	TAM-RNA S	AKAP13
MED13L1	0	1.355168	0.572	0.474	0	TAM-RNA S	MED13L
RAPGEF11	0	1.344141	0.602	0.535	0	TAM-RNA S	RAPGEF1
SLC8A1	0	1.343337	0.747	0.709	0	TAM-RNA S	SLC8A1
MAML2	0	1.339102	0.626	0.548	0	TAM-RNA S	MAML2
GNAQ	0	1.31419	0.673	0.62	0	TAM-RNA S	GNAQ
TCF12	0	1.286298	0.625	0.555	0	TAM-RNA S	TCF12
FKBP5	0	1.233431	0.685	0.711	0	TAM-RNA S	FKBP5
QKI	0	1.199671	0.788	0.779	0	TAM-RNA S	QKI
EPB41L21	0	1.124058	0.734	0.737	0	TAM-RNA S	EPB41L2

SLC1A31	0	1.027151	0.774	0.764	0	TAM-RNA S	SLC1A3
MT-CO1	0	0.96051	0.98	0.986	0	TAM-RNA S	MT-CO1
MT-CO3	0	0.951109	0.946	0.952	0	TAM-RNA S	MT-CO3
MALAT1	0	0.928456	1	0.988	0	TAM-RNA S	MALAT1
MT-ND4L	0	0.927353	0.908	0.927	0	TAM-RNA S	MT-ND4L
MEF2A1	0	0.920927	0.753	0.788	0	TAM-RNA S	MEF2A
MT-CO2	0	0.913288	0.963	0.971	0	TAM-RNA S	MT-CO2
MT-CYB	0	0.908734	0.949	0.961	0	TAM-RNA S	MT-CYB
MT-ND51	0	0.818515	0.847	0.883	0	TAM-RNA S	MT-ND5
ST6GAL11	3.21E-307	1.206465	0.669	0.649	9.04E-303	TAM-RNA S	ST6GAL1
UBE2E2	9.46E-307	1.230117	0.586	0.526	2.66E-302	TAM-RNA S	UBE2E2
TBC1D22A	4.87E-302	1.320616	0.536	0.44	1.37E-297	TAM-RNA S	TBC1D22A
MT-ND3	5.51E-293	0.901713	0.858	0.886	1.55E-288	TAM-RNA S	MT-ND3
ITPR21	1.60E-291	1.089087	0.627	0.602	4.50E-287	TAM-RNA S	ITPR2
ARHGAP15	3.28E-286	1.176506	0.604	0.569	9.22E-282	TAM-RNA S	ARHGAP15
MT-ND1	1.77E-284	0.812573	0.867	0.901	4.99E-280	TAM-RNA S	MT-ND1
ABCC4	1.86E-283	1.376922	0.552	0.472	5.23E-279	TAM-RNA S	ABCC4
PTPRJ	3.28E-280	1.508032	0.544	0.466	9.22E-276	TAM-RNA S	PTPRJ
SRGAP2C	1.56E-276	1.308306	0.517	0.42	4.39E-272	TAM-RNA S	SRGAP2C
RUNX1	3.93E-271	1.210879	0.633	0.598	1.11E-266	TAM-RNA S	RUNX1
FHIT	5.00E-269	1.364798	0.516	0.428	1.41E-264	TAM-RNA S	FHIT
SIK3	1.53E-267	1.300657	0.516	0.416	4.30E-263	TAM-RNA S	SIK3
SKI	1.89E-267	1.514648	0.483	0.371	5.32E-263	TAM-RNA S	SKI
PRKAG2	2.06E-263	1.394121	0.463	0.35	5.79E-259	TAM-RNA S	PRKAG2
GNA131	2.28E-258	1.125426	0.623	0.603	6.41E-254	TAM-RNA S	GNA13
SLC9A91	6.95E-256	1.271311	0.51	0.422	1.95E-251	TAM-RNA S	SLC9A9
CD832	7.23E-253	0.609555	0.903	0.913	2.03E-248	TAM-RNA S	CD83
FMNL2	6.11E-252	1.413018	0.546	0.478	1.72E-247	TAM-RNA S	FMNL2
MT-ATP81	2.08E-250	1.018876	0.814	0.823	5.86E-246	TAM-RNA S	MT-ATP8
RERE	2.75E-245	1.236435	0.506	0.419	7.74E-241	TAM-RNA S	RERE
OXR11	1.79E-243	1.402686	0.515	0.43	5.05E-239	TAM-RNA S	OXR1
IRAK31	1.32E-224	1.004765	0.647	0.657	3.71E-220	TAM-RNA S	IRAK3
DSCAM1	5.24E-222	1.364162	0.409	0.293	1.47E-217	TAM-RNA S	DSCAM
MT-ND2	7.11E-221	0.798173	0.827	0.874	2.00E-216	TAM-RNA S	MT-ND2
HS3ST41	1.05E-218	1.35811	0.34	0.215	2.94E-214	TAM-RNA S	HS3ST4
PRKCA	8.38E-218	1.278272	0.441	0.333	2.36E-213	TAM-RNA S	PRKCA
JMJD1C	1.07E-211	1.055355	0.6	0.595	3.02E-207	TAM-RNA S	JMJD1C
DIAPH21	9.64E-211	1.142127	0.497	0.427	2.71E-206	TAM-RNA S	DIAPH2
ADAMTSL4	1.46E-210	1.40573	0.43	0.33	4.10E-206	TAM-RNA S	ADAMTSL4-AS1
SSH21	5.59E-210	1.161309	0.612	0.618	1.57E-205	TAM-RNA S	SSH2
FMN1	4.16E-209	1.347962	0.483	0.402	1.17E-204	TAM-RNA S	FMN1
JAZF1	1.43E-208	1.252709	0.543	0.505	4.02E-204	TAM-RNA S	JAZF1
SNX29	1.06E-205	1.182893	0.46	0.373	2.98E-201	TAM-RNA S	SNX29
BNC21	1.84E-205	1.251034	0.405	0.296	5.18E-201	TAM-RNA S	BNC2
ZBTB201	4.09E-203	1.181874	0.489	0.419	1.15E-198	TAM-RNA S	ZBTB20
B4GALT11	1.65E-201	1.12585	0.607	0.605	4.65E-197	TAM-RNA S	B4GALT1
SDK1	3.55E-200	1.308986	0.35	0.228	1.00E-195	TAM-RNA S	SDK1

CMIP	4.99E-198	1.240534	0.439	0.347	1.40E-193	TAM-RNA S	CMIP
MGAT5	1.03E-197	1.338837	0.402	0.298	2.91E-193	TAM-RNA S	MGAT5
MT-ND4	4.96E-192	0.679576	0.839	0.893	1.40E-187	TAM-RNA S	MT-ND4
AL163541.1	2.16E-191	1.554543	0.458	0.377	6.09E-187	TAM-RNA S	AL163541.1
LYN	2.22E-190	0.979944	0.634	0.672	6.24E-186	TAM-RNA S	LYN
NR4A31	8.91E-189	1.076633	0.647	0.632	2.51E-184	TAM-RNA S	NR4A3
LIMS11	1.15E-187	0.940763	0.658	0.692	3.23E-183	TAM-RNA S	LIMS1
DIP2B1	3.68E-187	1.159478	0.412	0.313	1.04E-182	TAM-RNA S	DIP2B
SIPA1L1	1.14E-186	1.25816	0.588	0.563	3.20E-182	TAM-RNA S	SIPA1L1
FOXP1	2.37E-184	1.230435	0.506	0.465	6.68E-180	TAM-RNA S	FOXP1
LHFPL2	1.48E-178	0.949345	0.62	0.643	4.15E-174	TAM-RNA S	LHFPL2
TRIO	1.14E-176	1.117141	0.405	0.313	3.19E-172	TAM-RNA S	TRIO
ELF11	1.30E-175	0.884594	0.638	0.673	3.65E-171	TAM-RNA S	ELF1
ANKRD441	9.68E-174	1.003035	0.575	0.577	2.72E-169	TAM-RNA S	ANKRD44
PRKN	1.48E-173	1.186589	0.34	0.234	4.16E-169	TAM-RNA S	PRKN
GAB2	1.71E-173	1.11789	0.523	0.492	4.82E-169	TAM-RNA S	GAB2
EXOC4	9.55E-173	1.126799	0.407	0.317	2.69E-168	TAM-RNA S	EXOC4
WWOX	1.13E-172	1.380069	0.354	0.252	3.17E-168	TAM-RNA S	WWOX
FTX1	1.65E-168	1.096726	0.358	0.253	4.63E-164	TAM-RNA S	FTX
ARIH1	1.51E-167	1.126584	0.496	0.449	4.24E-163	TAM-RNA S	ARIH1
PALD11	3.01E-166	1.163829	0.556	0.555	8.48E-162	TAM-RNA S	PALD1
GALNT2	7.98E-166	1.143793	0.488	0.441	2.25E-161	TAM-RNA S	GALNT2
FAM53B	1.48E-165	1.188529	0.371	0.273	4.17E-161	TAM-RNA S	FAM53B
ARHGAP22	1.96E-164	1.182176	0.493	0.446	5.50E-160	TAM-RNA S	ARHGAP22
DOCK81	3.07E-164	0.901533	0.64	0.694	8.65E-160	TAM-RNA S	DOCK8
ARL15	4.62E-158	1.106774	0.339	0.238	1.30E-153	TAM-RNA S	ARL15
DYRK1A	7.21E-158	1.044112	0.361	0.262	2.03E-153	TAM-RNA S	DYRK1A
MSI2	1.69E-156	1.012643	0.253	0.149	4.76E-152	TAM-RNA S	MSI2
CAMK1D	1.07E-155	1.200608	0.463	0.411	3.01E-151	TAM-RNA S	CAMK1D
RPS6KA2	8.71E-152	1.148289	0.344	0.248	2.45E-147	TAM-RNA S	RPS6KA2
SUSD6	1.87E-151	1.128973	0.478	0.432	5.25E-147	TAM-RNA S	SUSD6
FCHSD21	2.61E-151	1.106664	0.504	0.471	7.34E-147	TAM-RNA S	FCHSD2
LINC02798	2.34E-150	1.26741	0.406	0.326	6.59E-146	TAM-RNA S	LINC02798
MBNL1	2.38E-150	0.969429	0.63	0.682	6.70E-146	TAM-RNA S	MBNL1
SRGAP1	6.30E-148	1.196715	0.47	0.424	1.77E-143	TAM-RNA S	SRGAP1
RASAL2	9.20E-148	1.140902	0.42	0.35	2.59E-143	TAM-RNA S	RASAL2
RBM47	5.71E-147	1.024676	0.527	0.505	1.61E-142	TAM-RNA S	RBM47
GLS	2.28E-145	1.107152	0.502	0.476	6.40E-141	TAM-RNA S	GLS
KMT2C	4.12E-145	0.999928	0.446	0.387	1.16E-140	TAM-RNA S	KMT2C
PPARD	1.41E-143	1.197837	0.409	0.338	3.96E-139	TAM-RNA S	PPARD
DISC11	2.70E-143	1.11799	0.439	0.379	7.61E-139	TAM-RNA S	DISC1
LNCAROD1	1.84E-141	1.120399	0.498	0.457	5.18E-137	TAM-RNA S	LNCAROD
HIF1A1	1.26E-140	0.988492	0.655	0.697	3.55E-136	TAM-RNA S	HIF1A
ETV6	4.36E-139	1.001061	0.55	0.551	1.23E-134	TAM-RNA S	ETV6
RANBP2	2.47E-138	1.207825	0.581	0.57	6.95E-134	TAM-RNA S	RANBP2
STAG1	3.34E-138	1.033092	0.458	0.413	9.41E-134	TAM-RNA S	STAG1
DLEU11	8.57E-138	1.046702	0.547	0.543	2.41E-133	TAM-RNA S	DLEU1

PAG1	3.15E-136	1.12544	0.473	0.434	8.88E-132	TAM-RNA S	PAG1
LINC01374	1.15E-135	1.132592	0.369	0.286	3.23E-131	TAM-RNA S	LINC01374
AC020916.	2.22E-135	0.961373	0.587	0.586	6.25E-131	TAM-RNA S	AC020916.1
LPP	2.73E-135	0.956162	0.516	0.501	7.69E-131	TAM-RNA S	LPP
BCAS3	7.26E-133	1.096249	0.322	0.232	2.04E-128	TAM-RNA S	BCAS3
OLR11	9.36E-132	0.625327	0.74	0.795	2.63E-127	TAM-RNA S	OLR1
ATG7	9.42E-129	1.083781	0.417	0.357	2.65E-124	TAM-RNA S	ATG7
RAD51B	2.48E-128	1.089845	0.367	0.29	6.97E-124	TAM-RNA S	RAD51B
TRAPPC9	1.84E-127	1.029212	0.33	0.244	5.17E-123	TAM-RNA S	TRAPPC9
DOCK2	3.42E-127	1.014204	0.473	0.445	9.62E-123	TAM-RNA S	DOCK2
ZFHX31	2.92E-126	0.984859	0.52	0.526	8.22E-122	TAM-RNA S	ZFHX3
MB21D2	2.46E-125	1.153807	0.318	0.233	6.91E-121	TAM-RNA S	MB21D2
FNIP2	3.20E-125	1.2475	0.423	0.367	8.99E-121	TAM-RNA S	FNIP2
SNX91	6.86E-124	1.093842	0.552	0.549	1.93E-119	TAM-RNA S	SNX9
JARID2	1.16E-123	1.091475	0.353	0.27	3.26E-119	TAM-RNA S	JARID2
MYO9B	1.54E-122	1.024706	0.503	0.485	4.33E-118	TAM-RNA S	MYO9B
MAN1A1	2.08E-122	1.16723	0.461	0.428	5.85E-118	TAM-RNA S	MAN1A1
SPTLC21	3.51E-122	0.95876	0.576	0.602	9.86E-118	TAM-RNA S	SPTLC2
PRKCH1	2.12E-121	0.904003	0.584	0.596	5.95E-117	TAM-RNA S	PRKCH
ARID1B	5.33E-120	0.993224	0.382	0.314	1.50E-115	TAM-RNA S	ARID1B
KCNMA1	9.54E-118	1.305412	0.34	0.266	2.68E-113	TAM-RNA S	KCNMA1
RASA3	2.04E-117	1.145906	0.424	0.374	5.73E-113	TAM-RNA S	RASA3
IRAK21	5.95E-114	1.077496	0.484	0.461	1.67E-109	TAM-RNA S	IRAK2
KDM6A	1.36E-112	0.928989	0.284	0.199	3.82E-108	TAM-RNA S	KDM6A
NFKB11	8.33E-112	1.235175	0.602	0.604	2.34E-107	TAM-RNA S	NFKB1
PEAK12	3.56E-111	0.980891	0.513	0.499	1.00E-106	TAM-RNA S	PEAK1
ZNF3311	4.67E-111	1.083149	0.629	0.623	1.31E-106	TAM-RNA S	ZNF331
MTSS1	2.56E-108	1.206977	0.378	0.314	7.19E-104	TAM-RNA S	MTSS1
CUX1	6.58E-108	0.997776	0.434	0.396	1.85E-103	TAM-RNA S	CUX1
SPAG9	1.81E-106	1.104712	0.421	0.381	5.10E-102	TAM-RNA S	SPAG9
PICALM1	7.77E-106	0.810386	0.615	0.691	2.19E-101	TAM-RNA S	PICALM
FBXO11	1.05E-105	1.03745	0.405	0.357	2.94E-101	TAM-RNA S	FBXO11
TNS3	2.11E-105	1.012749	0.365	0.302	5.95E-101	TAM-RNA S	TNS3
SLC11A11	4.37E-105	0.838937	0.604	0.638	1.23E-100	TAM-RNA S	SLC11A1
CSF2RA1	5.75E-104	0.752454	0.621	0.681	1.62E-99	TAM-RNA S	CSF2RA
ANKS1A	1.18E-103	1.056458	0.373	0.316	3.32E-99	TAM-RNA S	ANKS1A
PDE8A	4.27E-103	1.145525	0.373	0.311	1.20E-98	TAM-RNA S	PDE8A
IMMP2L	8.17E-99	1.103954	0.325	0.257	2.30E-94	TAM-RNA S	IMMP2L
SBF2	1.95E-96	0.962992	0.378	0.327	5.49E-92	TAM-RNA S	SBF2
REL1	2.99E-94	0.651637	0.681	0.754	8.42E-90	TAM-RNA S	REL
TANC2	5.35E-93	0.987572	0.309	0.239	1.51E-88	TAM-RNA S	TANC2
TNRC18	1.12E-92	0.989171	0.305	0.235	3.16E-88	TAM-RNA S	TNRC18
MAP4K32	2.21E-92	0.883329	0.509	0.511	6.21E-88	TAM-RNA S	MAP4K3
RASGEF1B1	7.07E-92	0.754765	0.606	0.65	1.99E-87	TAM-RNA S	RASGEF1B
PRKCE	3.24E-91	1.040796	0.273	0.2	9.12E-87	TAM-RNA S	PRKCE
SPRED1	6.32E-91	1.020651	0.372	0.315	1.78E-86	TAM-RNA S	SPRED1
MIR29B2CI	9.87E-90	0.886171	0.271	0.197	2.78E-85	TAM-RNA S	MIR29B2CHG

ATXN1	2.85E-89	0.985738	0.39	0.349	8.03E-85	TAM-RNA S	ATXN1
ADAM17	6.52E-89	0.926507	0.467	0.46	1.83E-84	TAM-RNA S	ADAM17
SMYD3	1.22E-88	1.054206	0.266	0.194	3.42E-84	TAM-RNA S	SMYD3
DPYD	5.25E-88	0.97997	0.466	0.463	1.48E-83	TAM-RNA S	DPYD
MERTK	2.20E-87	1.012816	0.434	0.407	6.20E-83	TAM-RNA S	MERTK
ATP8B41	5.03E-87	0.867464	0.468	0.461	1.42E-82	TAM-RNA S	ATP8B4
GPCPD1	5.15E-87	0.993963	0.419	0.391	1.45E-82	TAM-RNA S	GPCPD1
ZNF609	1.22E-86	1.004181	0.299	0.233	3.43E-82	TAM-RNA S	ZNF609
TBL1XR1	1.29E-86	0.958662	0.352	0.3	3.63E-82	TAM-RNA S	TBL1XR1
TSC22D2	3.54E-86	0.967946	0.518	0.534	9.95E-82	TAM-RNA S	TSC22D2
BMP2K1	2.69E-84	0.922498	0.473	0.478	7.56E-80	TAM-RNA S	BMP2K
IL1RAP1	6.81E-84	1.021041	0.426	0.398	1.92E-79	TAM-RNA S	IL1RAP
CYTH1	3.94E-83	1.021977	0.345	0.294	1.11E-78	TAM-RNA S	CYTH1
SMAP21	5.59E-83	0.561422	0.683	0.776	1.57E-78	TAM-RNA S	SMAP2
DLEU2	1.79E-82	0.909863	0.424	0.4	5.03E-78	TAM-RNA S	DLEU2
MAN2A1	8.32E-82	1.052208	0.401	0.37	2.34E-77	TAM-RNA S	MAN2A1
RFX3	1.36E-81	0.900597	0.261	0.192	3.82E-77	TAM-RNA S	RFX3
COP1	4.68E-81	0.885974	0.327	0.27	1.32E-76	TAM-RNA S	COP1
ATP9B	5.78E-81	0.861981	0.273	0.205	1.63E-76	TAM-RNA S	ATP9B
ZEB2	3.78E-80	0.900936	0.555	0.616	1.06E-75	TAM-RNA S	ZEB2
PACSIN2	4.80E-80	1.028423	0.384	0.351	1.35E-75	TAM-RNA S	PACSIN2
AKT3	5.09E-80	0.91672	0.309	0.248	1.43E-75	TAM-RNA S	AKT3
MIR99AHG	5.77E-80	0.867436	0.278	0.21	1.62E-75	TAM-RNA S	MIR99AHG
CCNL1	6.79E-80	0.70643	0.586	0.65	1.91E-75	TAM-RNA S	CCNL1
DENND1A	1.16E-79	0.888763	0.286	0.221	3.27E-75	TAM-RNA S	DENND1A
WDFY31	3.65E-79	0.853634	0.297	0.234	1.03E-74	TAM-RNA S	WDFY3
BTBD9	1.25E-77	0.954242	0.279	0.215	3.52E-73	TAM-RNA S	BTBD9
SYNDIG11	4.47E-77	1.151449	0.441	0.425	1.26E-72	TAM-RNA S	SYNDIG1
WDR70	4.94E-77	0.870697	0.307	0.248	1.39E-72	TAM-RNA S	WDR70
VTI1A	3.39E-75	0.917838	0.293	0.234	9.54E-71	TAM-RNA S	VTI1A
ATP2C11	4.07E-75	0.959628	0.415	0.391	1.14E-70	TAM-RNA S	ATP2C1
LRCH1	8.53E-75	0.9336	0.342	0.296	2.40E-70	TAM-RNA S	LRCH1
ASH1L	8.75E-75	0.906296	0.392	0.365	2.46E-70	TAM-RNA S	ASH1L
CCNH	1.61E-74	0.744691	0.65	0.728	4.54E-70	TAM-RNA S	CCNH
RIN2	4.01E-74	0.935473	0.474	0.479	1.13E-69	TAM-RNA S	RIN2
MT-ATP61	6.00E-74	0.753	0.794	0.861	1.69E-69	TAM-RNA S	MT-ATP6
INPP5D1	3.32E-72	0.901683	0.488	0.513	9.34E-68	TAM-RNA S	INPP5D
PIK3R5	5.18E-72	0.906697	0.461	0.465	1.46E-67	TAM-RNA S	PIK3R5
CDKAL1	1.31E-71	0.824917	0.255	0.192	3.70E-67	TAM-RNA S	CDKAL1
CHD2	2.02E-71	0.927325	0.453	0.46	5.70E-67	TAM-RNA S	CHD2
FAF1	4.74E-71	0.863125	0.33	0.282	1.33E-66	TAM-RNA S	FAF1
KANSL11	2.52E-70	0.873391	0.312	0.259	7.08E-66	TAM-RNA S	KANSL1
31-Mar	3.96E-70	0.963052	0.474	0.476	1.11E-65	TAM-RNA S	3-Mar
DENND4A	2.54E-69	0.913585	0.353	0.313	7.14E-65	TAM-RNA S	DENND4A
PELI1	6.14E-68	0.947912	0.52	0.549	1.73E-63	TAM-RNA S	PELI1
TBC1D51	6.34E-68	0.886866	0.412	0.399	1.78E-63	TAM-RNA S	TBC1D5
HIVEP3	1.60E-67	0.987439	0.331	0.284	4.50E-63	TAM-RNA S	HIVEP3

USP34	1.61E-67	0.861899	0.338	0.295	4.53E-63	TAM-RNA S	USP34
AFF4	2.45E-67	0.758137	0.536	0.593	6.90E-63	TAM-RNA S	AFF4
PIK3R1	4.99E-67	0.96875	0.494	0.525	1.40E-62	TAM-RNA S	PIK3R1
PDE4B1	5.63E-67	0.965017	0.493	0.504	1.58E-62	TAM-RNA S	PDE4B
MACF1	1.85E-66	0.86009	0.443	0.448	5.20E-62	TAM-RNA S	MACF1
PAN3	3.02E-66	0.806137	0.298	0.244	8.50E-62	TAM-RNA S	PAN3
MKLN1	3.54E-66	0.922027	0.368	0.338	9.97E-62	TAM-RNA S	MKLN1
FER1	1.08E-65	0.724723	0.278	0.219	3.03E-61	TAM-RNA S	FER
TTC7A	6.81E-65	1.010921	0.371	0.343	1.92E-60	TAM-RNA S	TTC7A
FOXO3	2.83E-63	1.028874	0.4	0.384	7.96E-59	TAM-RNA S	FOXO3
VPS13B	3.91E-63	0.822517	0.292	0.24	1.10E-58	TAM-RNA S	VPS13B
DOCK10	8.53E-63	0.821321	0.485	0.509	2.40E-58	TAM-RNA S	DOCK10
UBASH3B1	1.02E-62	1.127589	0.256	0.2	2.88E-58	TAM-RNA S	UBASH3B
FAM172A	4.01E-62	0.804721	0.264	0.209	1.13E-57	TAM-RNA S	FAM172A
SLC7A5	4.03E-62	1.018249	0.401	0.387	1.13E-57	TAM-RNA S	SLC7A5
DNM2	4.20E-61	0.953084	0.419	0.416	1.18E-56	TAM-RNA S	DNM2
NAV31	3.84E-60	1.001674	0.35	0.314	1.08E-55	TAM-RNA S	NAV3
PCNX21	3.99E-60	0.882625	0.325	0.285	1.12E-55	TAM-RNA S	PCNX2
WDR91	1.86E-59	0.831192	0.314	0.271	5.25E-55	TAM-RNA S	WDR91
KDM4B	3.39E-59	0.98164	0.312	0.27	9.53E-55	TAM-RNA S	KDM4B
SIPA1L2	4.89E-59	0.940365	0.294	0.247	1.38E-54	TAM-RNA S	SIPA1L2
DAGLB1	6.52E-59	0.897057	0.432	0.433	1.83E-54	TAM-RNA S	DAGLB
NCOA3	7.77E-59	0.901668	0.363	0.336	2.19E-54	TAM-RNA S	NCOA3
RREB1	1.14E-58	0.862297	0.272	0.22	3.20E-54	TAM-RNA S	RREB1
BRAF	1.37E-58	0.799025	0.289	0.239	3.86E-54	TAM-RNA S	BRAF
ADAM281	5.80E-58	0.689006	0.539	0.599	1.63E-53	TAM-RNA S	ADAM28
DTNA	8.91E-58	0.859303	0.25	0.198	2.51E-53	TAM-RNA S	DTNA
HDAC91	3.50E-57	0.966453	0.417	0.415	9.85E-53	TAM-RNA S	HDAC9
EIF4G3	3.90E-57	0.857831	0.391	0.381	1.10E-52	TAM-RNA S	EIF4G3
BIRC6	5.16E-57	0.802259	0.352	0.322	1.45E-52	TAM-RNA S	BIRC6
CPEB4	2.39E-56	0.964278	0.327	0.292	6.72E-52	TAM-RNA S	CPEB4
USP9X	4.32E-56	0.86025	0.316	0.277	1.22E-51	TAM-RNA S	USP9X
NCOR2	1.33E-55	0.845315	0.439	0.452	3.74E-51	TAM-RNA S	NCOR2
SYTL31	2.60E-55	1.104683	0.426	0.424	7.31E-51	TAM-RNA S	SYTL3
ATF6	2.04E-54	0.95674	0.409	0.409	5.73E-50	TAM-RNA S	ATF6
MRTFA	2.18E-54	0.863544	0.321	0.285	6.12E-50	TAM-RNA S	MRTFA
RYR11	9.21E-54	0.932482	0.292	0.249	2.59E-49	TAM-RNA S	RYR1
MIR222HG	1.45E-53	0.963418	0.325	0.288	4.07E-49	TAM-RNA S	MIR222HG
HNRNPC	6.06E-53	0.804637	0.528	0.612	1.71E-48	TAM-RNA S	HNRNPC
TFRC	9.46E-53	1.06943	0.354	0.323	2.66E-48	TAM-RNA S	TFRC
ZBTB16	1.08E-52	1.00309	0.381	0.365	3.03E-48	TAM-RNA S	ZBTB16
CAMK2D	1.71E-52	0.911739	0.334	0.305	4.80E-48	TAM-RNA S	CAMK2D
CPEB3	1.12E-51	0.846292	0.27	0.224	3.16E-47	TAM-RNA S	CPEB3
FBXW11	1.65E-51	0.880975	0.357	0.338	4.64E-47	TAM-RNA S	FBXW11
RAB1A1	2.62E-51	0.760748	0.492	0.538	7.36E-47	TAM-RNA S	RAB1A
IFRD1	4.23E-51	0.882963	0.475	0.502	1.19E-46	TAM-RNA S	IFRD1
IGSF21	2.45E-50	1.009937	0.4	0.4	6.88E-46	TAM-RNA S	IGSF21

CTNNB1	3.30E-50	0.97847	0.458	0.492	9.29E-46	TAM-RNA S	CTNNB1
MTHFD1L	3.83E-50	0.898195	0.351	0.331	1.08E-45	TAM-RNA S	MTHFD1L
FAM49B	2.60E-49	0.695966	0.553	0.651	7.30E-45	TAM-RNA S	FAM49B
CREBBP	6.85E-49	0.783726	0.292	0.253	1.93E-44	TAM-RNA S	CREBBP
NF1	1.07E-48	0.794985	0.324	0.294	3.02E-44	TAM-RNA S	NF1
CHKA	1.63E-48	0.895799	0.398	0.403	4.58E-44	TAM-RNA S	CHKA
HSPD11	3.06E-48	0.294533	0.329	0.507	8.61E-44	TAM-RNA S	HSPD1
OSBPL3	2.32E-47	0.808501	0.275	0.233	6.54E-43	TAM-RNA S	OSBPL3
XPR1	3.02E-47	0.807384	0.258	0.213	8.49E-43	TAM-RNA S	XPR1
SLC4A7	1.53E-46	0.848622	0.428	0.439	4.31E-42	TAM-RNA S	SLC4A7
PREX1	5.66E-46	0.767041	0.521	0.597	1.59E-41	TAM-RNA S	PREX1
PSME4	8.02E-46	0.805839	0.292	0.255	2.26E-41	TAM-RNA S	PSME4
ATP13A31	1.83E-45	0.919916	0.35	0.328	5.16E-41	TAM-RNA S	ATP13A3
KLHL62	3.14E-45	0.904159	0.424	0.432	8.83E-41	TAM-RNA S	KLHL6
ARHGAP6	6.11E-45	0.900472	0.332	0.305	1.72E-40	TAM-RNA S	ARHGAP6
MANBA1	7.09E-45	0.789944	0.502	0.555	1.99E-40	TAM-RNA S	MANBA
PTPN11	7.18E-45	0.982121	0.402	0.409	2.02E-40	TAM-RNA S	PTPN1
GRID21	7.84E-45	1.535479	0.267	0.222	2.21E-40	TAM-RNA S	GRID2
RHBDF2	1.71E-44	0.610754	0.569	0.66	4.82E-40	TAM-RNA S	RHBDF2
PADI21	6.19E-44	1.078603	0.524	0.585	1.74E-39	TAM-RNA S	PADI2
BABAM2	8.71E-44	0.82587	0.312	0.285	2.45E-39	TAM-RNA S	BABAM2
CYTH3	9.02E-44	0.838239	0.255	0.213	2.54E-39	TAM-RNA S	CYTH3
USP36	1.29E-43	0.916056	0.308	0.28	3.62E-39	TAM-RNA S	USP36
MSR11	1.52E-43	0.813482	0.524	0.583	4.29E-39	TAM-RNA S	MSR1
RAPGEF2	1.82E-42	0.842916	0.285	0.25	5.13E-38	TAM-RNA S	RAPGEF2
RCOR1	2.48E-42	0.782988	0.262	0.222	6.99E-38	TAM-RNA S	RCOR1
YWHAQ	1.07E-41	0.259374	0.228	0.363	3.02E-37	TAM-RNA S	YWHAQ
FNDC3B	1.58E-41	0.735451	0.286	0.25	4.45E-37	TAM-RNA S	FNDC3B
NAMPT	2.02E-41	0.905383	0.504	0.545	5.67E-37	TAM-RNA S	NAMPT
CAST	3.77E-41	0.261908	0.26	0.409	1.06E-36	TAM-RNA S	CAST
PPP6R3	3.82E-41	0.73988	0.297	0.267	1.08E-36	TAM-RNA S	PPP6R3
SORL11	1.23E-40	0.56758	0.602	0.713	3.47E-36	TAM-RNA S	SORL1
IPCEF12	3.40E-40	0.766161	0.486	0.51	9.56E-36	TAM-RNA S	IPCEF1
RIN3	1.45E-39	0.818148	0.428	0.457	4.08E-35	TAM-RNA S	RIN3
PTK2B	7.77E-39	0.964562	0.291	0.264	2.19E-34	TAM-RNA S	PTK2B
PFKFB3	1.01E-38	0.79465	0.513	0.572	2.85E-34	TAM-RNA S	PFKFB3
MPRIP	2.93E-38	0.905484	0.266	0.232	8.25E-34	TAM-RNA S	MPRIP
MT-ND61	5.82E-38	0.657257	0.522	0.564	1.64E-33	TAM-RNA S	MT-ND6
SPIDR1	4.03E-37	0.732756	0.456	0.494	1.13E-32	TAM-RNA S	SPIDR
CHD1	4.06E-37	0.779966	0.413	0.433	1.14E-32	TAM-RNA S	CHD1
BAZ2B	4.43E-37	0.777741	0.378	0.381	1.25E-32	TAM-RNA S	BAZ2B
ITGAV1	4.62E-37	0.842264	0.43	0.455	1.30E-32	TAM-RNA S	ITGAV
PIAS1	6.78E-37	0.754523	0.403	0.424	1.91E-32	TAM-RNA S	PIAS1
UBAP1	1.53E-35	0.782442	0.298	0.277	4.31E-31	TAM-RNA S	UBAP1
GTF2B	2.87E-35	0.273333	0.252	0.386	8.08E-31	TAM-RNA S	GTF2B
YBX3	1.80E-34	0.269328	0.269	0.411	5.06E-30	TAM-RNA S	YBX3
ABCA1	1.98E-34	0.838609	0.398	0.401	5.58E-30	TAM-RNA S	ABCA1

CTBP2	2.58E-34	0.833935	0.266	0.237	7.27E-30	TAM-RNA S	CTBP2
ALCAM	3.49E-34	1.06171	0.372	0.378	9.81E-30	TAM-RNA S	ALCAM
DDX3Y	1.01E-33	0.930154	0.348	0.346	2.84E-29	TAM-RNA S	DDX3Y
MGAT4A1	1.17E-33	0.290416	0.323	0.496	3.30E-29	TAM-RNA S	MGAT4A
KDM2A	1.22E-33	0.748638	0.285	0.261	3.43E-29	TAM-RNA S	KDM2A
NFAT5	3.02E-33	0.849836	0.355	0.352	8.51E-29	TAM-RNA S	NFAT5
COG5	5.00E-33	0.714468	0.261	0.231	1.41E-28	TAM-RNA S	COG5
STK17B	5.93E-33	0.250801	0.273	0.41	1.67E-28	TAM-RNA S	STK17B
NIPBL	7.79E-33	0.727195	0.382	0.396	2.19E-28	TAM-RNA S	NIPBL
AZIN1-AS1:	1.13E-32	1.115882	0.363	0.36	3.17E-28	TAM-RNA S	AZIN1-AS1
UBAC2	1.69E-32	0.76049	0.44	0.48	4.76E-28	TAM-RNA S	UBAC2
ETF1	1.73E-32	0.769361	0.387	0.405	4.88E-28	TAM-RNA S	ETF1
LCOR	2.60E-32	0.748651	0.262	0.234	7.31E-28	TAM-RNA S	LCOR
SH3KBP1	4.42E-32	0.778042	0.446	0.502	1.24E-27	TAM-RNA S	SH3KBP1
ARHGEF7	4.51E-32	0.789884	0.315	0.301	1.27E-27	TAM-RNA S	ARHGEF7
RBM39	8.97E-32	0.512784	0.572	0.677	2.52E-27	TAM-RNA S	RBM39
AFF1	9.08E-32	0.803991	0.315	0.302	2.56E-27	TAM-RNA S	AFF1
KLF71	1.33E-31	0.82871	0.337	0.332	3.73E-27	TAM-RNA S	KLF7
NCOA2	1.81E-31	0.723226	0.25	0.22	5.08E-27	TAM-RNA S	NCOA2
IRF8	2.66E-31	0.264271	0.297	0.444	7.49E-27	TAM-RNA S	IRF8
MAP2K1	2.94E-31	0.823318	0.351	0.354	8.26E-27	TAM-RNA S	MAP2K1
EHMT1	9.58E-31	0.763557	0.256	0.23	2.70E-26	TAM-RNA S	EHMT1
PABPC4	1.37E-30	0.284465	0.324	0.489	3.85E-26	TAM-RNA S	PABPC4
MAP3K2	2.35E-30	0.796603	0.382	0.399	6.63E-26	TAM-RNA S	MAP3K2
SSBP2	2.36E-30	0.806356	0.27	0.245	6.64E-26	TAM-RNA S	SSBP2
SUMF1	4.96E-30	0.849557	0.283	0.264	1.40E-25	TAM-RNA S	SUMF1
IFNGR21	5.05E-30	0.286488	0.373	0.559	1.42E-25	TAM-RNA S	IFNGR2
MYCBP21	6.06E-30	0.703133	0.44	0.491	1.71E-25	TAM-RNA S	MYCBP2
EML4	1.68E-29	0.72816	0.389	0.409	4.74E-25	TAM-RNA S	EML4
TMED5	1.91E-29	0.279579	0.232	0.354	5.37E-25	TAM-RNA S	TMED5
NFATC11	1.91E-29	1.010939	0.272	0.247	5.38E-25	TAM-RNA S	NFATC1
ERC1	2.13E-29	0.740252	0.268	0.245	5.99E-25	TAM-RNA S	ERC1
TET2	3.43E-29	0.776497	0.333	0.326	9.65E-25	TAM-RNA S	TET2
GSK3B	3.92E-29	0.74136	0.271	0.249	1.10E-24	TAM-RNA S	GSK3B
FOSB1	1.75E-28	0.372941	0.706	0.774	4.93E-24	TAM-RNA S	FOSB
UTRN	1.89E-28	0.774795	0.314	0.303	5.33E-24	TAM-RNA S	UTRN
AC084871.	2.46E-28	0.267024	0.196	0.286	6.91E-24	TAM-RNA S	AC084871.1
PDXK	1.40E-27	0.260372	0.231	0.346	3.95E-23	TAM-RNA S	PDXK
CHD7	2.21E-27	0.730854	0.321	0.314	6.21E-23	TAM-RNA S	CHD7
DST	2.45E-27	0.780588	0.347	0.353	6.90E-23	TAM-RNA S	DST
COMMD10	6.91E-27	0.752497	0.269	0.249	1.94E-22	TAM-RNA S	COMMD10
RAP1B	9.80E-27	0.298836	0.288	0.433	2.76E-22	TAM-RNA S	RAP1B
PDCD4	1.05E-26	0.301506	0.186	0.282	2.94E-22	TAM-RNA S	PDCD4
ERICH1	1.82E-26	0.29954	0.198	0.297	5.12E-22	TAM-RNA S	ERICH1
IL6R	2.63E-26	0.712057	0.352	0.358	7.40E-22	TAM-RNA S	IL6R
ABL21	2.95E-26	0.850512	0.384	0.385	8.31E-22	TAM-RNA S	ABL2
MAP4K41	4.73E-26	0.794563	0.36	0.373	1.33E-21	TAM-RNA S	MAP4K4

RANBP9	4.83E-26	0.804434	0.391	0.417	1.36E-21	TAM-RNA S	RANBP9
IRS21	5.21E-26	0.795654	0.407	0.44	1.47E-21	TAM-RNA S	IRS2
STX71	5.37E-26	0.284234	0.218	0.327	1.51E-21	TAM-RNA S	STX7
RLF	9.49E-26	0.730803	0.303	0.293	2.67E-21	TAM-RNA S	RLF
PAFAH1B1	1.16E-25	0.71361	0.373	0.395	3.28E-21	TAM-RNA S	PAFAH1B1
TLN21	1.26E-25	0.71724	0.259	0.236	3.55E-21	TAM-RNA S	TLN2
GNAI3	1.54E-25	0.26748	0.201	0.299	4.34E-21	TAM-RNA S	GNAI3
UBE2F1	1.62E-25	0.344748	0.213	0.321	4.56E-21	TAM-RNA S	UBE2F
TRPS1	1.89E-25	0.761127	0.263	0.242	5.31E-21	TAM-RNA S	TRPS1
PIP4K2A	2.86E-25	0.722555	0.273	0.257	8.06E-21	TAM-RNA S	PIP4K2A
CCNY	3.19E-25	0.796758	0.332	0.339	8.97E-21	TAM-RNA S	CCNY
DOCK11	3.65E-25	0.691193	0.253	0.23	1.03E-20	TAM-RNA S	DOCK11
PLA2G4A	7.02E-25	0.80626	0.366	0.377	1.98E-20	TAM-RNA S	PLA2G4A
LARP4B	8.06E-25	0.676186	0.277	0.262	2.27E-20	TAM-RNA S	LARP4B
PTPN2	9.01E-25	0.261045	0.192	0.285	2.54E-20	TAM-RNA S	PTPN2
HERC1	2.18E-24	0.664386	0.254	0.232	6.13E-20	TAM-RNA S	HERC1
THADA	3.39E-24	0.74654	0.287	0.274	9.54E-20	TAM-RNA S	THADA
FP236383.3	3.76E-24	0.293759	0.574	0.501	1.06E-19	TAM-RNA S	FP236383.3
CORO7	4.90E-24	0.253962	0.2	0.294	1.38E-19	TAM-RNA S	CORO7
RABGAP1L	6.92E-24	0.780525	0.367	0.391	1.95E-19	TAM-RNA S	RABGAP1L
GRAMD1B	1.07E-23	0.840039	0.303	0.297	3.00E-19	TAM-RNA S	GRAMD1B
ENTPD1	1.20E-23	0.272583	0.255	0.371	3.39E-19	TAM-RNA S	ENTPD1
TBC1D1	1.28E-23	0.758985	0.354	0.373	3.60E-19	TAM-RNA S	TBC1D1
YPEL5	1.43E-23	0.339874	0.257	0.376	4.01E-19	TAM-RNA S	YPEL5
ITGAX1	1.46E-23	0.906636	0.391	0.417	4.10E-19	TAM-RNA S	ITGAX
CPM	1.49E-23	0.367309	0.192	0.282	4.18E-19	TAM-RNA S	CPM
AKAP9	1.87E-23	0.25806	0.188	0.279	5.26E-19	TAM-RNA S	AKAP9
RABGEF1	2.24E-23	0.756618	0.381	0.397	6.31E-19	TAM-RNA S	RABGEF1
LINC01578	3.01E-23	0.271183	0.349	0.511	8.48E-19	TAM-RNA S	LINC01578
NCKAP1L	3.15E-23	0.256511	0.273	0.4	8.87E-19	TAM-RNA S	NCKAP1L
NSMCE2	3.20E-23	0.724765	0.342	0.35	9.02E-19	TAM-RNA S	NSMCE2
RAB7A	3.26E-23	0.787471	0.511	0.613	9.18E-19	TAM-RNA S	RAB7A
IL13RA1	3.58E-23	0.262729	0.368	0.543	1.01E-18	TAM-RNA S	IL13RA1
PSMA1	3.66E-23	0.368973	0.315	0.469	1.03E-18	TAM-RNA S	PSMA1
DENND5A	5.69E-23	0.775191	0.27	0.257	1.60E-18	TAM-RNA S	DENND5A
NUMB	8.79E-23	0.705843	0.357	0.374	2.47E-18	TAM-RNA S	NUMB
VOPP1	1.30E-22	0.314301	0.227	0.335	3.67E-18	TAM-RNA S	VOPP1
CYFIP1	1.83E-22	0.531638	0.561	0.669	5.15E-18	TAM-RNA S	CYFIP1
BACH1	1.84E-22	0.724635	0.363	0.383	5.18E-18	TAM-RNA S	BACH1
GK	2.30E-22	0.305011	0.257	0.368	6.48E-18	TAM-RNA S	GK
TTC3	3.05E-22	0.268594	0.171	0.254	8.57E-18	TAM-RNA S	TTC3
ITPR1	4.19E-22	0.749051	0.271	0.258	1.18E-17	TAM-RNA S	ITPR1
MPP1	4.24E-22	0.252973	0.186	0.272	1.19E-17	TAM-RNA S	MPP1
ATP2A2	4.40E-22	0.884193	0.304	0.302	1.24E-17	TAM-RNA S	ATP2A2
EIF4E1	4.85E-22	0.386111	0.315	0.443	1.36E-17	TAM-RNA S	EIF4E
SEPTIN6	5.41E-22	0.302213	0.21	0.308	1.52E-17	TAM-RNA S	SEPTIN6
LCP21	6.64E-22	0.824814	0.54	0.624	1.87E-17	TAM-RNA S	LCP2

HDLBP	7.26E-22	0.251109	0.188	0.275	2.04E-17	TAM-RNA S	HDLBP
YWHAZ	1.09E-21	0.274502	0.416	0.609	3.07E-17	TAM-RNA S	YWHAZ
PLCG2	1.13E-21	0.72722	0.28	0.271	3.17E-17	TAM-RNA S	PLCG2
USP15	1.45E-21	0.619474	0.449	0.515	4.08E-17	TAM-RNA S	USP15
TAOK31	2.06E-21	0.753908	0.41	0.455	5.79E-17	TAM-RNA S	TAOK3
RUNX2	2.66E-21	0.746701	0.296	0.292	7.48E-17	TAM-RNA S	RUNX2
WIPF1	3.64E-21	0.311454	0.233	0.338	1.03E-16	TAM-RNA S	WIPF1
CEP170	4.03E-21	0.638468	0.418	0.463	1.13E-16	TAM-RNA S	CEP170
ATG16L2	5.25E-21	0.255043	0.172	0.251	1.48E-16	TAM-RNA S	ATG16L2
ATP6V0A1	6.66E-21	0.69216	0.253	0.236	1.87E-16	TAM-RNA S	ATP6V0A1
BTAF1	6.33E-20	0.679435	0.283	0.275	1.78E-15	TAM-RNA S	BTAF1
11-Mar	7.44E-20	0.341959	0.208	0.3	2.09E-15	TAM-RNA S	1-Mar
TACC1	8.61E-20	0.354842	0.303	0.438	2.42E-15	TAM-RNA S	TACC1
GNA12	1.01E-19	0.758493	0.257	0.245	2.84E-15	TAM-RNA S	GNA12
TRIP12	1.86E-19	0.641691	0.303	0.303	5.23E-15	TAM-RNA S	TRIP12
PTPRE	1.93E-19	0.616185	0.451	0.511	5.44E-15	TAM-RNA S	PTPRE
PUM1	2.03E-19	0.678954	0.3	0.301	5.70E-15	TAM-RNA S	PUM1
DCTN4	2.09E-19	0.296215	0.222	0.319	5.87E-15	TAM-RNA S	DCTN4
FAM49A	2.37E-19	0.857226	0.321	0.332	6.67E-15	TAM-RNA S	FAM49A
NAF11	2.89E-19	0.256959	0.182	0.259	8.13E-15	TAM-RNA S	NAF1
SH2B31	3.13E-19	0.776046	0.348	0.371	8.81E-15	TAM-RNA S	SH2B3
DENND3	3.70E-19	0.730905	0.39	0.429	1.04E-14	TAM-RNA S	DENND3
MEF2C1	3.72E-19	0.465584	0.61	0.752	1.05E-14	TAM-RNA S	MEF2C
GAK	4.05E-19	0.709438	0.28	0.276	1.14E-14	TAM-RNA S	GAK
ARAP1	5.38E-19	0.288284	0.179	0.258	1.51E-14	TAM-RNA S	ARAP1
P4HA11	6.70E-19	0.356281	0.231	0.325	1.88E-14	TAM-RNA S	P4HA1
CHSY11	7.15E-19	0.795293	0.332	0.342	2.01E-14	TAM-RNA S	CHSY1
NFKBID1	7.24E-19	0.610565	0.457	0.512	2.04E-14	TAM-RNA S	NFKBID
RALY	1.05E-18	0.344948	0.305	0.446	2.94E-14	TAM-RNA S	RALY
RTN41	1.08E-18	0.302837	0.439	0.646	3.04E-14	TAM-RNA S	RTN4
NR3C1	1.61E-18	0.315357	0.244	0.35	4.53E-14	TAM-RNA S	NR3C1
ANKRD17	1.78E-18	0.670704	0.307	0.311	5.01E-14	TAM-RNA S	ANKRD17
USP4	2.54E-18	0.727989	0.351	0.374	7.14E-14	TAM-RNA S	USP4
NEDD91	4.69E-18	0.544076	0.476	0.52	1.32E-13	TAM-RNA S	NEDD9
EPB41L3	5.31E-18	0.304967	0.259	0.365	1.49E-13	TAM-RNA S	EPB41L3
DDX21	1.09E-17	0.314929	0.293	0.418	3.07E-13	TAM-RNA S	DDX21
ZNF207	1.25E-17	0.284439	0.263	0.375	3.53E-13	TAM-RNA S	ZNF207
NRIP1	1.43E-17	0.679324	0.381	0.422	4.03E-13	TAM-RNA S	NRIP1
SP100	1.65E-17	0.294271	0.366	0.531	4.65E-13	TAM-RNA S	SP100
NUP98	2.75E-17	0.651026	0.301	0.305	7.73E-13	TAM-RNA S	NUP98
RAB10	3.46E-17	0.311424	0.252	0.359	9.75E-13	TAM-RNA S	RAB10
FRYL	4.57E-17	0.656874	0.267	0.258	1.29E-12	TAM-RNA S	FRYL
CTNNA1	5.32E-17	0.291838	0.202	0.288	1.50E-12	TAM-RNA S	CTNNA1
VPS13D	6.30E-17	0.640394	0.262	0.254	1.77E-12	TAM-RNA S	VPS13D
SF1	8.79E-17	0.255813	0.389	0.556	2.47E-12	TAM-RNA S	SF1
RHOH	1.09E-16	0.360525	0.184	0.26	3.08E-12	TAM-RNA S	RHOH
STX111	1.29E-16	0.322445	0.267	0.375	3.63E-12	TAM-RNA S	STX11

IKZF1	1.74E-16	0.807732	0.387	0.431	4.91E-12	TAM-RNA S	IKZF1
MCF2L1	4.49E-16	0.702532	0.29	0.29	1.26E-11	TAM-RNA S	MCF2L
DNAJC3	6.10E-16	0.314989	0.209	0.292	1.72E-11	TAM-RNA S	DNAJC3
ALOX5	6.94E-16	0.372593	0.337	0.482	1.95E-11	TAM-RNA S	ALOX5
YME1L11	9.49E-16	0.337937	0.333	0.47	2.67E-11	TAM-RNA S	YME1L1
CSNK1A11	1.15E-15	0.627866	0.431	0.507	3.23E-11	TAM-RNA S	CSNK1A1
RAB2A	1.47E-15	0.325625	0.317	0.452	4.13E-11	TAM-RNA S	RAB2A
DDX17	1.96E-15	0.253902	0.367	0.524	5.53E-11	TAM-RNA S	DDX17
TMEM165	2.34E-15	0.310194	0.247	0.348	6.57E-11	TAM-RNA S	TMEM165
NFIC	2.81E-15	0.32838	0.289	0.408	7.92E-11	TAM-RNA S	NFIC
TBXAS11	3.23E-15	0.521929	0.578	0.729	9.10E-11	TAM-RNA S	TBXAS1
TTYH3	3.25E-15	0.700692	0.274	0.274	9.14E-11	TAM-RNA S	TTYH3
FRMD4B	3.31E-15	0.737489	0.308	0.321	9.31E-11	TAM-RNA S	FRMD4B
AAK1	4.61E-15	0.652501	0.257	0.25	1.30E-10	TAM-RNA S	AAK1
EPC1	4.62E-15	0.298744	0.348	0.49	1.30E-10	TAM-RNA S	EPC1
TNPO1	5.23E-15	0.3264	0.203	0.286	1.47E-10	TAM-RNA S	TNPO1
ZNF644	5.48E-15	0.664944	0.295	0.303	1.54E-10	TAM-RNA S	ZNF644
NFE2L2	6.12E-15	0.754139	0.404	0.462	1.72E-10	TAM-RNA S	NFE2L2
ZNRF21	8.67E-15	0.335366	0.194	0.272	2.44E-10	TAM-RNA S	ZNRF2
FAM149A1	1.07E-14	0.771859	0.258	0.255	3.01E-10	TAM-RNA S	FAM149A
BPTF	1.13E-14	0.346455	0.248	0.347	3.17E-10	TAM-RNA S	BPTF
ICAM11	1.40E-14	0.309875	0.25	0.338	3.95E-10	TAM-RNA S	ICAM1
USP53	1.42E-14	0.635445	0.401	0.439	3.99E-10	TAM-RNA S	USP53
ZMYM2	1.42E-14	0.608299	0.27	0.268	4.01E-10	TAM-RNA S	ZMYM2
PKN21	1.76E-14	0.661102	0.313	0.327	4.95E-10	TAM-RNA S	PKN2
ARID4B	1.76E-14	0.656335	0.322	0.341	4.97E-10	TAM-RNA S	ARID4B
ATP1B3	1.87E-14	0.885033	0.442	0.503	5.25E-10	TAM-RNA S	ATP1B3
CD58	2.16E-14	0.387788	0.264	0.371	6.08E-10	TAM-RNA S	CD58
GPR1321	2.20E-14	0.40006	0.209	0.29	6.19E-10	TAM-RNA S	GPR132
WDFY21	2.27E-14	0.674224	0.285	0.29	6.38E-10	TAM-RNA S	WDFY2
PPP3CA	3.03E-14	0.6708	0.27	0.27	8.53E-10	TAM-RNA S	PPP3CA
IST1	3.33E-14	0.264316	0.186	0.257	9.37E-10	TAM-RNA S	IST1
POLR2A	3.58E-14	0.313519	0.196	0.271	1.01E-09	TAM-RNA S	POLR2A
ABI1	3.68E-14	0.748577	0.368	0.412	1.03E-09	TAM-RNA S	ABI1
MYO1E	3.94E-14	0.940284	0.34	0.358	1.11E-09	TAM-RNA S	MYO1E
TM9SF3	4.59E-14	0.287035	0.224	0.311	1.29E-09	TAM-RNA S	TM9SF3
USP6NL1	5.59E-14	0.646904	0.254	0.249	1.57E-09	TAM-RNA S	USP6NL
TRPM2	6.37E-14	0.709269	0.274	0.277	1.79E-09	TAM-RNA S	TRPM2
PLCL21	7.26E-14	0.644114	0.251	0.244	2.04E-09	TAM-RNA S	PLCL2
CAPZA1	1.29E-13	0.347633	0.274	0.386	3.62E-09	TAM-RNA S	CAPZA1
ZNFX1	1.30E-13	0.348449	0.192	0.267	3.65E-09	TAM-RNA S	ZNFX1
MAP3K81	1.99E-13	0.495468	0.583	0.671	5.60E-09	TAM-RNA S	MAP3K8
SCAF11	2.27E-13	0.328648	0.307	0.43	6.39E-09	TAM-RNA S	SCAF11
CCDC18-AS	2.29E-13	0.357362	0.233	0.322	6.43E-09	TAM-RNA S	CCDC18-AS1
WTAP1	2.30E-13	0.354393	0.311	0.437	6.47E-09	TAM-RNA S	WTAP
RRBP1	2.63E-13	0.314519	0.207	0.284	7.40E-09	TAM-RNA S	RRBP1
IL10RA	3.40E-13	0.365452	0.278	0.388	9.57E-09	TAM-RNA S	IL10RA

AOAH	4.16E-13	0.705028	0.417	0.486	1.17E-08	TAM-RNA S	AOAH
PDGFB1	4.39E-13	0.764849	0.338	0.359	1.24E-08	TAM-RNA S	PDGFB
FNBP4	5.07E-13	0.334659	0.249	0.344	1.43E-08	TAM-RNA S	FNBP4
ADK	5.49E-13	0.656207	0.267	0.269	1.54E-08	TAM-RNA S	ADK
TRA2B2	6.93E-13	0.552619	0.489	0.576	1.95E-08	TAM-RNA S	TRA2B
EVI5	6.95E-13	0.645187	0.258	0.259	1.96E-08	TAM-RNA S	EVI5
PPP1R12A	9.67E-13	0.314186	0.244	0.337	2.72E-08	TAM-RNA S	PPP1R12A
PRRC2B	1.04E-12	0.662216	0.34	0.368	2.91E-08	TAM-RNA S	PRRC2B
IVNS1ABP	1.16E-12	0.369242	0.271	0.373	3.26E-08	TAM-RNA S	IVNS1ABP
CACUL1	1.28E-12	0.356551	0.233	0.321	3.61E-08	TAM-RNA S	CACUL1
FGD21	1.35E-12	0.300304	0.211	0.287	3.79E-08	TAM-RNA S	FGD2
CHD91	1.35E-12	0.633286	0.413	0.479	3.79E-08	TAM-RNA S	CHD9
CD86	1.52E-12	0.356146	0.458	0.65	4.27E-08	TAM-RNA S	CD86
AP3B1	3.80E-12	0.650485	0.277	0.284	1.07E-07	TAM-RNA S	AP3B1
DNAJB61	4.48E-12	0.907739	0.389	0.45	1.26E-07	TAM-RNA S	DNAJB6
AZIN1	5.98E-12	0.375202	0.233	0.319	1.68E-07	TAM-RNA S	AZIN1
OSBPL8	6.13E-12	0.396256	0.263	0.361	1.73E-07	TAM-RNA S	OSBPL8
ATRX	6.17E-12	0.340837	0.274	0.379	1.74E-07	TAM-RNA S	ATRX
NCOR1	6.22E-12	0.328045	0.247	0.342	1.75E-07	TAM-RNA S	NCOR1
GBP21	8.76E-12	0.399149	0.306	0.414	2.46E-07	TAM-RNA S	GBP2
RASGEF1C1	9.26E-12	0.805984	0.287	0.297	2.61E-07	TAM-RNA S	RASGEF1C
MAT2A	9.77E-12	0.298573	0.408	0.56	2.75E-07	TAM-RNA S	MAT2A
MICAL1	1.04E-11	0.358406	0.216	0.296	2.94E-07	TAM-RNA S	MICAL1
BCLAF1	1.15E-11	0.308108	0.29	0.4	3.24E-07	TAM-RNA S	BCLAF1
RHEB	1.17E-11	0.349461	0.336	0.465	3.28E-07	TAM-RNA S	RHEB
BCL61	2.24E-11	0.3918	0.25	0.339	6.30E-07	TAM-RNA S	BCL6
RAPH1	2.59E-11	0.711263	0.303	0.321	7.29E-07	TAM-RNA S	RAPH1
TCF25	2.82E-11	0.346018	0.352	0.495	7.94E-07	TAM-RNA S	TCF25
LRRK11	3.86E-11	0.668307	0.318	0.34	1.09E-06	TAM-RNA S	LRRK1
GOLGA4	4.80E-11	0.630824	0.295	0.309	1.35E-06	TAM-RNA S	GOLGA4
INTS61	5.38E-11	0.36486	0.243	0.33	1.51E-06	TAM-RNA S	INTS6
NEMF	5.43E-11	0.32963	0.194	0.263	1.53E-06	TAM-RNA S	NEMF
NLRP31	5.56E-11	0.7404	0.472	0.524	1.56E-06	TAM-RNA S	NLRP3
UVRAG1	6.09E-11	0.632515	0.345	0.383	1.71E-06	TAM-RNA S	UVRAG
DENND4C	9.62E-11	0.664266	0.267	0.273	2.71E-06	TAM-RNA S	DENND4C
HSPA9	1.14E-10	0.366687	0.271	0.372	3.21E-06	TAM-RNA S	HSPA9
ZMIZ1	1.79E-10	0.759665	0.3	0.318	5.05E-06	TAM-RNA S	ZMIZ1
ADAM9	2.17E-10	0.319223	0.199	0.268	6.10E-06	TAM-RNA S	ADAM9
HNRNPU	2.40E-10	0.269733	0.454	0.634	6.74E-06	TAM-RNA S	HNRNPU
ZNF2671	2.41E-10	0.340046	0.256	0.344	6.77E-06	TAM-RNA S	ZNF267
MALT1	3.30E-10	0.673336	0.261	0.265	9.29E-06	TAM-RNA S	MALT1
CCDC88A	3.78E-10	0.386328	0.289	0.399	1.06E-05	TAM-RNA S	CCDC88A
USP22	5.08E-10	0.329196	0.207	0.277	1.43E-05	TAM-RNA S	USP22
P2RX4	5.22E-10	0.356138	0.197	0.263	1.47E-05	TAM-RNA S	P2RX4
HIPK3	5.39E-10	0.663161	0.268	0.278	1.52E-05	TAM-RNA S	HIPK3
KMT2E	5.74E-10	0.644311	0.376	0.433	1.61E-05	TAM-RNA S	KMT2E
CTTNBP21	5.76E-10	0.659469	0.351	0.385	1.62E-05	TAM-RNA S	CTTNBP2

N4BP2L2	6.48E-10	0.340423	0.314	0.431	1.82E-05	TAM-RNA S	N4BP2L2
PBRM1	6.55E-10	0.296645	0.194	0.259	1.84E-05	TAM-RNA S	PBRM1
NUFIP21	1.38E-09	0.670651	0.39	0.448	3.88E-05	TAM-RNA S	NUFIP2
APBB1IP1	3.02E-09	0.453314	0.626	0.788	8.49E-05	TAM-RNA S	APBB1IP
PMEPA11	5.34E-09	0.386476	0.269	0.362	0.00015	TAM-RNA S	PMEPA1
PTPRC	5.52E-09	0.425709	0.568	0.725	0.000155	TAM-RNA S	PTPRC
JDP22	6.30E-09	0.620482	0.48	0.577	0.000177	TAM-RNA S	JDP2
ARID1A	7.34E-09	0.381705	0.189	0.251	0.000207	TAM-RNA S	ARID1A
CREM1	7.46E-09	0.283792	0.394	0.531	0.00021	TAM-RNA S	CREM
GNB1	7.57E-09	0.675879	0.406	0.487	0.000213	TAM-RNA S	GNB1
GRASP1	7.89E-09	0.438796	0.49	0.554	0.000222	TAM-RNA S	GRASP
PCM1	8.25E-09	0.326868	0.271	0.361	0.000232	TAM-RNA S	PCM1
RAB201	1.08E-08	0.670417	0.404	0.463	0.000303	TAM-RNA S	RAB20
COPA	1.42E-08	0.377464	0.254	0.341	0.0004	TAM-RNA S	COPA
RASSF2	1.56E-08	0.402717	0.229	0.305	0.000439	TAM-RNA S	RASSF2
RB1	1.65E-08	0.338644	0.385	0.531	0.000464	TAM-RNA S	RB1
UBE2D3	1.93E-08	0.528385	0.524	0.667	0.000544	TAM-RNA S	UBE2D3
SMARCA2	1.94E-08	0.419527	0.237	0.318	0.000545	TAM-RNA S	SMARCA2
RNF2131	1.94E-08	0.626998	0.394	0.457	0.000547	TAM-RNA S	RNF213
PRRC2C	2.04E-08	0.336911	0.388	0.537	0.000573	TAM-RNA S	PRRC2C
CHMP4B1	2.66E-08	0.388921	0.26	0.349	0.000749	TAM-RNA S	CHMP4B
CDK61	3.13E-08	0.345211	0.202	0.264	0.00088	TAM-RNA S	CDK6
ARID5A1	6.97E-08	0.445112	0.282	0.376	0.001961	TAM-RNA S	ARID5A
WDFY4	1.20E-07	0.595473	0.27	0.284	0.003387	TAM-RNA S	WDFY4
RSRP1	1.30E-07	0.336061	0.329	0.439	0.003669	TAM-RNA S	RSRP1
SRSF11	1.31E-07	0.354714	0.343	0.468	0.00368	TAM-RNA S	SRSF11
TSPAN14	1.52E-07	0.460456	0.249	0.331	0.004278	TAM-RNA S	TSPAN14
RNF144B	1.75E-07	0.919332	0.286	0.308	0.004928	TAM-RNA S	RNF144B
HNRNPA2B	1.76E-07	0.332986	0.661	0.828	0.00496	TAM-RNA S	HNRNPA2B1
CD55	1.82E-07	0.441241	0.273	0.36	0.005118	TAM-RNA S	CD55
STX41	1.93E-07	0.502902	0.239	0.313	0.005418	TAM-RNA S	STX4
FUS	2.07E-07	0.511491	0.495	0.621	0.005812	TAM-RNA S	FUS
TNRC6B	2.73E-07	0.645469	0.282	0.304	0.007675	TAM-RNA S	TNRC6B
SAMSN1	2.80E-07	0.593693	0.51	0.609	0.007867	TAM-RNA S	SAMSN1
ARHGAP18	2.90E-07	0.44581	0.243	0.326	0.008155	TAM-RNA S	ARHGAP18
PPP3R1	3.67E-07	0.599516	0.256	0.265	0.010315	TAM-RNA S	PPP3R1
UGCG	4.13E-07	0.442584	0.194	0.253	0.011626	TAM-RNA S	UGCG
SLC39A10	4.17E-07	0.330051	0.195	0.252	0.011725	TAM-RNA S	SLC39A10
FMNL3	4.26E-07	0.584788	0.382	0.443	0.011986	TAM-RNA S	FMNL3
TGFBR1	5.46E-07	0.407845	0.332	0.444	0.015366	TAM-RNA S	TGFBR1
RBPJ	5.56E-07	0.44557	0.259	0.344	0.015642	TAM-RNA S	RBPJ
VMP1	5.95E-07	0.59734	0.389	0.458	0.016748	TAM-RNA S	VMP1
SARNP	7.07E-07	0.40258	0.206	0.27	0.019881	TAM-RNA S	SARNP
ZFAND6	8.05E-07	0.63633	0.27	0.29	0.022664	TAM-RNA S	ZFAND6
CELF1	9.05E-07	0.573757	0.245	0.255	0.025455	TAM-RNA S	CELF1
ITPRIP1	1.08E-06	0.395143	0.241	0.313	0.030332	TAM-RNA S	ITPRIP
TUT7	1.34E-06	0.627177	0.3	0.327	0.037797	TAM-RNA S	TUT7

LUC7L3	1.49E-06	0.349735	0.219	0.284	0.041957	TAM-RNA S	LUC7L3
BBX	1.69E-06	0.38816	0.193	0.25	0.047643	TAM-RNA S	BBX
GNG71	2.11E-06	0.662344	0.378	0.443	0.059496	TAM-RNA S	GNG7
ELF2	2.88E-06	0.686056	0.282	0.307	0.081159	TAM-RNA S	ELF2
RCC2	3.05E-06	0.659973	0.32	0.36	0.085713	TAM-RNA S	RCC2
MAPKAP1	3.22E-06	0.598209	0.241	0.252	0.090532	TAM-RNA S	MAPKAP1
TPM41	3.69E-06	0.475555	0.322	0.431	0.103724	TAM-RNA S	TPM4
CTTNBP2NI	3.96E-06	0.38688	0.288	0.379	0.111499	TAM-RNA S	CTTNBP2NL
FLI11	4.53E-06	0.654496	0.268	0.287	0.127363	TAM-RNA S	FLI1
CSF3R	4.53E-06	0.331518	0.414	0.569	0.127408	TAM-RNA S	CSF3R
JAK1	4.90E-06	0.453445	0.337	0.456	0.137822	TAM-RNA S	JAK1
ROCK1	5.55E-06	0.621079	0.375	0.443	0.156136	TAM-RNA S	ROCK1
LITAF	7.31E-06	0.521399	0.337	0.454	0.205654	TAM-RNA S	LITAF
SYAP11	7.54E-06	0.735323	0.343	0.381	0.212063	TAM-RNA S	SYAP1
PUM2	8.27E-06	0.544381	0.246	0.258	0.232685	TAM-RNA S	PUM2
RBM25	1.04E-05	0.375983	0.328	0.439	0.291863	TAM-RNA S	RBM25
UBE2E1	1.32E-05	0.674101	0.298	0.331	0.371721	TAM-RNA S	UBE2E1
CNOT2	1.64E-05	0.423225	0.221	0.285	0.461208	TAM-RNA S	CNOT2
LPIN2	2.14E-05	0.41475	0.28	0.367	0.603082	TAM-RNA S	LPIN2
NFKBIZ1	2.18E-05	0.344209	0.421	0.543	0.614016	TAM-RNA S	NFKBIZ
PER11	2.51E-05	0.458082	0.474	0.579	0.707474	TAM-RNA S	PER1
DYNC1H1	2.56E-05	0.420698	0.275	0.357	0.719242	TAM-RNA S	DYNC1H1
WAC	3.12E-05	0.633402	0.286	0.317	0.876579	TAM-RNA S	WAC
SPATA131	3.19E-05	0.545514	0.372	0.43	0.897932	TAM-RNA S	SPATA13
RC3H1	3.84E-05	0.549965	0.287	0.314	1	TAM-RNA S	RC3H1
FNDC3A	3.93E-05	0.640607	0.313	0.352	1	TAM-RNA S	FNDC3A
SRSF4	4.03E-05	0.373687	0.249	0.321	1	TAM-RNA S	SRSF4
RASSF51	4.43E-05	0.445399	0.287	0.376	1	TAM-RNA S	RASSF5
STK4	4.70E-05	0.382448	0.342	0.456	1	TAM-RNA S	STK4
PHIP	5.24E-05	0.379707	0.225	0.286	1	TAM-RNA S	PHIP
NPL	6.29E-05	0.621615	0.305	0.34	1	TAM-RNA S	NPL
MKNK1	7.00E-05	0.35986	0.259	0.333	1	TAM-RNA S	MKNK1
ITFG1	8.48E-05	0.603448	0.244	0.262	1	TAM-RNA S	ITFG1
PRDM11	8.84E-05	0.425446	0.284	0.36	1	TAM-RNA S	PRDM1
NCK21	0.000101	0.624762	0.33	0.378	1	TAM-RNA S	NCK2
FNIP1	0.000109	0.597748	0.292	0.328	1	TAM-RNA S	FNIP1
RBM6	0.000123	0.581648	0.286	0.317	1	TAM-RNA S	RBM6
ATM1	0.000172	0.576894	0.341	0.392	1	TAM-RNA S	ATM
7-Mar	0.000228	0.366921	0.218	0.275	1	TAM-RNA S	7-Mar
PDS5A	0.00024	0.521421	0.265	0.288	1	TAM-RNA S	PDS5A
SMG1	0.000249	0.496157	0.239	0.253	1	TAM-RNA S	SMG1
SOAT1	0.000273	0.513887	0.266	0.346	1	TAM-RNA S	SOAT1
PPP1CB	0.000289	0.441836	0.201	0.253	1	TAM-RNA S	PPP1CB
MIS18BP1	0.000309	0.527879	0.368	0.489	1	TAM-RNA S	MIS18BP1
TRA2A	0.000359	0.575716	0.29	0.323	1	TAM-RNA S	TRA2A
PARVB	0.000396	0.653175	0.268	0.296	1	TAM-RNA S	PARVB
SETX	0.00045	0.403015	0.239	0.302	1	TAM-RNA S	SETX

PARP14	0.000451	0.42892	0.23	0.289	1	TAM-RNA S	PARP14
ETNK1	0.000464	0.411973	0.24	0.304	1	TAM-RNA S	ETNK1
WNK1	0.000485	0.555694	0.274	0.302	1	TAM-RNA S	WNK1
MAP3K51	0.000508	0.6057	0.254	0.276	1	TAM-RNA S	MAP3K5
TENT2	0.000529	0.517892	0.246	0.265	1	TAM-RNA S	TENT2
VASH1	0.00063	0.370724	0.367	0.48	1	TAM-RNA S	VASH1
ITSN2	0.000685	0.415714	0.275	0.355	1	TAM-RNA S	ITSN2
SLCO2B11	0.000724	0.542546	0.448	0.553	1	TAM-RNA S	SLCO2B1
TBC1D14	0.000776	0.655151	0.255	0.278	1	TAM-RNA S	TBC1D14
FAM102B	0.001169	0.563933	0.249	0.269	1	TAM-RNA S	FAM102B
UBL3	0.001185	0.443995	0.286	0.371	1	TAM-RNA S	UBL3
ARGLU1	0.001241	0.389127	0.367	0.485	1	TAM-RNA S	ARGLU1
RNF149	0.001403	0.399921	0.427	0.574	1	TAM-RNA S	RNF149
EPS8	0.001416	0.6508	0.324	0.378	1	TAM-RNA S	EPS8
RAP1A	0.001467	0.540161	0.434	0.552	1	TAM-RNA S	RAP1A
BAZ1A	0.001518	0.457348	0.226	0.283	1	TAM-RNA S	BAZ1A
LPCAT21	0.00154	0.433874	0.526	0.669	1	TAM-RNA S	LPCAT2
ARL8B	0.001587	0.649348	0.294	0.335	1	TAM-RNA S	ARL8B
NT5C2	0.001696	0.543651	0.252	0.274	1	TAM-RNA S	NT5C2
SLA	0.001778	0.469953	0.329	0.432	1	TAM-RNA S	SLA
RFX21	0.001797	0.547149	0.273	0.298	1	TAM-RNA S	RFX2
TANK	0.002542	0.818326	0.275	0.309	1	TAM-RNA S	TANK
JAK21	0.003396	0.566604	0.266	0.295	1	TAM-RNA S	JAK2
HIVEP2	0.003413	0.649087	0.272	0.303	1	TAM-RNA S	HIVEP2
CLK11	0.00343	0.439404	0.306	0.393	1	TAM-RNA S	CLK1
BCL2	0.00377	0.636033	0.318	0.366	1	TAM-RNA S	BCL2
CEP350	0.003796	0.574781	0.319	0.37	1	TAM-RNA S	CEP350
SENP6	0.003885	0.544155	0.282	0.318	1	TAM-RNA S	SENP6
ADAM10	0.004082	0.435043	0.278	0.355	1	TAM-RNA S	ADAM10
PHF20	0.004502	0.593085	0.336	0.398	1	TAM-RNA S	PHF20
MAPKAPK2	0.004545	0.60935	0.249	0.276	1	TAM-RNA S	MAPKAPK2
GRB2	0.004764	0.453306	0.463	0.591	1	TAM-RNA S	GRB2
MYO1F	0.005396	0.508329	0.3	0.386	1	TAM-RNA S	MYO1F
BASP11	0.005553	0.696383	0.464	0.57	1	TAM-RNA S	BASP1
AXL	0.006064	0.519227	0.286	0.368	1	TAM-RNA S	AXL
ATP1A1	0.00725	0.546498	0.319	0.415	1	TAM-RNA S	ATP1A1
MTRNR2L1	0.007329	0.811798	0.407	0.506	1	TAM-RNA S	MTRNR2L12
TLR2	0.00876	0.461608	0.395	0.52	1	TAM-RNA S	TLR2
ILF3	0.008917	0.437394	0.242	0.302	1	TAM-RNA S	ILF3
ZNF638	0.009094	0.507281	0.264	0.293	1	TAM-RNA S	ZNF638
TAB2	0.009662	0.536327	0.369	0.445	1	TAM-RNA S	TAB2
ELOVL5	0.009797	0.596684	0.23	0.286	1	TAM-RNA S	ELOVL5
SH3TC1	0.009879	0.598824	0.352	0.42	1	TAM-RNA S	SH3TC1
CD1631	0	1.584902	0.85	0.317	0	TAM-APClil	CD163
SELENOP	0	1.016063	0.369	0.146	0	TAM-APClil	SELENOP
FCGR2B	0	1.00346	0.484	0.122	0	TAM-APClil	FCGR2B
CXCR41	0	0.980193	0.765	0.443	0	TAM-APClil	CXCR4

LYZ	0	0.925335	0.665	0.245	0	TAM-APClil	LYZ
ANXA1	0	0.920853	0.642	0.326	0	TAM-APClil	ANXA1
CLEC4E	0	0.91988	0.379	0.068	0	TAM-APClil	CLEC4E
HLA-DRB11	0	0.909046	0.989	0.904	0	TAM-APClil	HLA-DRB1
HLA-DPB1	0	0.90219	0.967	0.795	0	TAM-APClil	HLA-DPB1
HLA-DPA11	0	0.900688	0.983	0.871	0	TAM-APClil	HLA-DPA1
TGFBI	0	0.894816	0.52	0.137	0	TAM-APClil	TGFBI
SAP30	0	0.861492	0.647	0.328	0	TAM-APClil	SAP30
APOC11	0	0.837808	0.947	0.774	0	TAM-APClil	APOC1
HLA-DRA	0	0.827751	0.991	0.923	0	TAM-APClil	HLA-DRA
VIM	0	0.805601	0.762	0.392	0	TAM-APClil	VIM
APOE1	0	0.791228	0.989	0.917	0	TAM-APClil	APOE
HLA-DQA1	0	0.772978	0.892	0.63	0	TAM-APClil	HLA-DQA1
F13A1	0	0.721979	0.422	0.131	0	TAM-APClil	F13A1
NAMPT1	0	0.721157	0.779	0.528	0	TAM-APClil	NAMPT
AHR	0	0.71629	0.387	0.135	0	TAM-APClil	AHR
MCTP1	0	0.709535	0.438	0.174	0	TAM-APClil	MCTP1
CST31	0	0.70869	0.985	0.897	0	TAM-APClil	CST3
ZFP36L2	0	0.707524	0.959	0.836	0	TAM-APClil	ZFP36L2
FXYD5	0	0.70739	0.837	0.552	0	TAM-APClil	FXYD5
CALHM6	0	0.695233	0.545	0.25	0	TAM-APClil	CALHM6
BTG1	0	0.659578	0.934	0.748	0	TAM-APClil	BTG1
AHNAK	0	0.654618	0.473	0.171	0	TAM-APClil	AHNAK
C1QA1	0	0.647172	0.976	0.875	0	TAM-APClil	C1QA
CD741	0	0.62265	1	0.99	0	TAM-APClil	CD74
AREG	0	0.616674	0.349	0.144	0	TAM-APClil	AREG
MS4A6A1	0	0.613341	0.812	0.525	0	TAM-APClil	MS4A6A
CHPT1	0	0.613269	0.434	0.191	0	TAM-APClil	CHPT1
ALOX15B	0	0.584709	0.284	0.093	0	TAM-APClil	ALOX15B
CD9	0	0.564656	0.37	0.136	0	TAM-APClil	CD9
FILIP1L	0	0.555551	0.504	0.256	0	TAM-APClil	FILIP1L
IQGAP2	0	0.488165	0.33	0.119	0	TAM-APClil	IQGAP2
ITGA4	0	0.425565	0.259	0.075	0	TAM-APClil	ITGA4
GAPT	0	0.389847	0.256	0.085	0	TAM-APClil	GAPT
CADM1	0	0.386146	0.278	0.107	0	TAM-APClil	CADM1
HLA-DQB11	4.50E-296	0.559762	0.918	0.729	1.27E-291	TAM-APClil	HLA-DQB1
MS4A4A1	3.46E-295	0.51892	0.612	0.364	9.75E-291	TAM-APClil	MS4A4A
GPR1831	4.81E-291	0.701025	0.827	0.634	1.35E-286	TAM-APClil	GPR183
CD14	2.99E-282	0.646828	0.843	0.63	8.41E-278	TAM-APClil	CD14
RPS181	2.10E-277	0.408132	0.982	0.901	5.92E-273	TAM-APClil	RPS18
SAT11	2.27E-276	0.439895	0.998	0.969	6.40E-272	TAM-APClil	SAT1
TXNIP	2.85E-255	0.651384	0.532	0.317	8.01E-251	TAM-APClil	TXNIP
HLA-DMA1	3.36E-255	0.461288	0.894	0.7	9.45E-251	TAM-APClil	HLA-DMA
RPL121	1.22E-251	0.380377	0.98	0.887	3.43E-247	TAM-APClil	RPL12
CFD	1.95E-250	0.530882	0.572	0.349	5.50E-246	TAM-APClil	CFD
C1orf162	5.96E-250	0.475706	0.769	0.541	1.68E-245	TAM-APClil	C1orf162
RPL321	2.45E-248	0.354669	0.987	0.904	6.90E-244	TAM-APClil	RPL32

FGL2	5.71E-247	0.502719	0.635	0.41	1.61E-242	TAM-APClil	FGL2
RPL281	8.88E-246	0.365406	0.987	0.916	2.50E-241	TAM-APClil	RPL28
APOO1	4.43E-244	0.444535	0.494	0.269	1.25E-239	TAM-APClil	APOO
TYROBP1	8.42E-236	0.33658	0.99	0.906	2.37E-231	TAM-APClil	TYROBP
RPS121	1.80E-232	0.350131	0.981	0.912	5.05E-228	TAM-APClil	RPS12
HLA-A	3.80E-232	0.453227	0.959	0.831	1.07E-227	TAM-APClil	HLA-A
RPL361	5.19E-232	0.372137	0.962	0.827	1.46E-227	TAM-APClil	RPL36
TNFSF131	3.19E-229	0.378207	0.547	0.329	8.99E-225	TAM-APClil	TNFSF13
CEBPD1	1.61E-226	0.447373	0.975	0.865	4.52E-222	TAM-APClil	CEBPD
DSE	2.19E-226	0.414244	0.667	0.442	6.17E-222	TAM-APClil	DSE
RPL261	6.76E-217	0.34396	0.972	0.882	1.90E-212	TAM-APClil	RPL26
RGS12	3.83E-215	0.405333	0.964	0.834	1.08E-210	TAM-APClil	RGS1
ATP1B31	1.22E-213	0.421602	0.702	0.487	3.43E-209	TAM-APClil	ATP1B3
AKR1B11	4.29E-212	0.404438	0.666	0.455	1.21E-207	TAM-APClil	AKR1B1
MS4A71	3.19E-208	0.393713	0.873	0.677	8.99E-204	TAM-APClil	MS4A7
CPM1	5.52E-207	0.486237	0.453	0.265	1.55E-202	TAM-APClil	CPM
EPB41L31	4.70E-205	0.375353	0.548	0.346	1.32E-200	TAM-APClil	EPB41L3
ARHGAP18	1.36E-203	0.355283	0.508	0.309	3.82E-199	TAM-APClil	ARHGAP18
METRNL	1.86E-203	0.369703	0.361	0.186	5.23E-199	TAM-APClil	METRNL
RPL291	3.47E-202	0.327825	0.977	0.874	9.75E-198	TAM-APClil	RPL29
KLF41	1.69E-200	0.521827	0.705	0.498	4.76E-196	TAM-APClil	KLF4
RPL301	3.89E-200	0.322936	0.978	0.889	1.09E-195	TAM-APClil	RPL30
HLA-DMB1	1.60E-198	0.384779	0.743	0.53	4.51E-194	TAM-APClil	HLA-DMB
NPC21	1.27E-196	0.36059	0.947	0.796	3.56E-192	TAM-APClil	NPC2
LGALS3BP	5.14E-196	0.501499	0.338	0.176	1.45E-191	TAM-APClil	LGALS3BP
HERPUD11	2.99E-195	0.478086	0.87	0.695	8.40E-191	TAM-APClil	HERPUD1
CTSS	3.05E-194	0.366283	0.804	0.612	8.57E-190	TAM-APClil	CTSS
RPL131	8.46E-193	0.301912	0.992	0.933	2.38E-188	TAM-APClil	RPL13
CHCHD10	6.74E-192	0.362737	0.682	0.471	1.90E-187	TAM-APClil	CHCHD10
RGS21	2.69E-191	0.520542	0.753	0.553	7.56E-187	TAM-APClil	RGS2
ATF31	1.36E-190	0.532904	0.775	0.573	3.83E-186	TAM-APClil	ATF3
VAMP5	2.42E-188	0.341247	0.288	0.141	6.80E-184	TAM-APClil	VAMP5
GRN	6.26E-188	0.392011	0.834	0.659	1.76E-183	TAM-APClil	GRN
RNF144B1	3.09E-187	0.440597	0.483	0.296	8.69E-183	TAM-APClil	RNF144B
RPS281	5.12E-187	0.314213	0.975	0.873	1.44E-182	TAM-APClil	RPS28
HMGB2	9.80E-187	0.388932	0.44	0.259	2.76E-182	TAM-APClil	HMGB2
ZFAND51	3.77E-186	0.593345	0.682	0.489	1.06E-181	TAM-APClil	ZFAND5
SIPA1L11	7.78E-185	0.284022	0.779	0.551	2.19E-180	TAM-APClil	SIPA1L1
CREB5	9.95E-185	0.392296	0.281	0.137	2.80E-180	TAM-APClil	CREB5
PTMA1	1.46E-184	0.306944	0.986	0.902	4.11E-180	TAM-APClil	PTMA
IQGAP1	6.05E-183	0.290293	0.479	0.286	1.70E-178	TAM-APClil	IQGAP1
UTRN1	6.84E-183	0.290593	0.489	0.293	1.93E-178	TAM-APClil	UTRN
RPL351	1.18E-182	0.329754	0.939	0.79	3.33E-178	TAM-APClil	RPL35
MSR12	5.12E-182	0.321715	0.786	0.566	1.44E-177	TAM-APClil	MSR1
PLBD1	5.61E-182	0.25726	0.269	0.128	1.58E-177	TAM-APClil	PLBD1
RPL341	5.99E-182	0.298977	0.978	0.875	1.69E-177	TAM-APClil	RPL34
RPL101	2.20E-180	0.303935	0.993	0.944	6.20E-176	TAM-APClil	RPL10

RPS4X1	6.25E-180	0.311628	0.96	0.849	1.76E-175	TAM-APClil	RPS4X
FAM110B	3.19E-179	0.289579	0.32	0.165	8.98E-175	TAM-APClil	FAM110B
RPS151	6.61E-176	0.289479	0.982	0.888	1.86E-171	TAM-APClil	RPS15
CARD16	2.00E-175	0.288977	0.324	0.169	5.63E-171	TAM-APClil	CARD16
RPS27	1.90E-174	0.3211	0.975	0.872	5.34E-170	TAM-APClil	RPS27
RACK11	2.36E-174	0.325317	0.952	0.816	6.65E-170	TAM-APClil	RACK1
RPS141	2.15E-173	0.298799	0.974	0.88	6.05E-169	TAM-APClil	RPS14
ATP2B1-AS	2.85E-173	0.435734	0.689	0.491	8.01E-169	TAM-APClil	ATP2B1-AS1
RPS15A1	3.19E-173	0.288943	0.976	0.883	8.97E-169	TAM-APClil	RPS15A
AP1S2	1.12E-172	0.303635	0.362	0.2	3.16E-168	TAM-APClil	AP1S2
RPL111	1.25E-171	0.28867	0.98	0.896	3.51E-167	TAM-APClil	RPL11
RPS251	3.42E-171	0.297966	0.962	0.843	9.62E-167	TAM-APClil	RPS25
ZBTB161	3.36E-170	0.334113	0.553	0.354	9.46E-166	TAM-APClil	ZBTB16
LTA4H	2.84E-169	0.297128	0.399	0.23	8.00E-165	TAM-APClil	LTA4H
ASAH1	7.38E-168	0.336773	0.851	0.664	2.08E-163	TAM-APClil	ASAH1
IFITM3	7.15E-167	0.430156	0.672	0.453	2.01E-162	TAM-APClil	IFITM3
EVI2B	5.18E-166	0.329798	0.594	0.401	1.46E-161	TAM-APClil	EVI2B
TKT1	4.70E-163	0.321565	0.578	0.39	1.32E-158	TAM-APClil	TKT
TLR21	3.77E-162	0.316154	0.704	0.5	1.06E-157	TAM-APClil	TLR2
SESN11	1.57E-157	0.389818	0.465	0.291	4.41E-153	TAM-APClil	SESN1
SERPINA1	7.11E-156	0.311676	0.733	0.532	2.00E-151	TAM-APClil	SERPINA1
FPR3	5.48E-155	0.268022	0.337	0.184	1.54E-150	TAM-APClil	FPR3
GSTP11	1.18E-154	0.294162	0.766	0.57	3.31E-150	TAM-APClil	GSTP1
EEF1A1	1.21E-154	0.293731	0.994	0.962	3.39E-150	TAM-APClil	EEF1A1
CD3021	1.75E-154	0.299266	0.754	0.562	4.94E-150	TAM-APClil	CD302
SGK12	1.54E-153	0.307285	0.968	0.856	4.33E-149	TAM-APClil	SGK1
DAB2	3.74E-152	0.352486	0.404	0.243	1.05E-147	TAM-APClil	DAB2
ZFP361	5.21E-152	0.345665	0.982	0.928	1.47E-147	TAM-APClil	ZFP36
RPS231	3.48E-150	0.26615	0.983	0.905	9.80E-146	TAM-APClil	RPS23
HEXA	1.00E-149	0.294129	0.619	0.432	2.82E-145	TAM-APClil	HEXA
RPL37	1.70E-149	0.276149	0.974	0.864	4.78E-145	TAM-APClil	RPL37
MGAT1	9.89E-149	0.309583	0.682	0.488	2.78E-144	TAM-APClil	MGAT1
FAU1	3.53E-148	0.261994	0.975	0.875	9.93E-144	TAM-APClil	FAU
RPL37A	8.65E-148	0.31002	0.929	0.771	2.43E-143	TAM-APClil	RPL37A
RPS131	1.29E-147	0.26655	0.969	0.881	3.64E-143	TAM-APClil	RPS13
CMSS1	1.69E-147	0.299795	0.393	0.234	4.74E-143	TAM-APClil	CMSS1
FTH11	1.26E-146	0.251689	0.998	0.983	3.54E-142	TAM-APClil	FTH1
IFITM2	1.48E-146	0.308942	0.706	0.509	4.16E-142	TAM-APClil	IFITM2
RPL18A1	6.88E-146	0.267674	0.973	0.877	1.93E-141	TAM-APClil	RPL18A
VAMP81	1.25E-145	0.272312	0.713	0.514	3.51E-141	TAM-APClil	VAMP8
BTF31	2.94E-144	0.295338	0.869	0.696	8.28E-140	TAM-APClil	BTF3
RPL141	1.22E-143	0.265321	0.966	0.849	3.43E-139	TAM-APClil	RPL14
RPL191	5.65E-142	0.255207	0.981	0.901	1.59E-137	TAM-APClil	RPL19
DUSP62	6.67E-142	0.484961	0.437	0.279	1.88E-137	TAM-APClil	DUSP6
RPL391	8.37E-142	0.259759	0.977	0.877	2.36E-137	TAM-APClil	RPL39
CTSH	1.93E-141	0.294676	0.717	0.533	5.44E-137	TAM-APClil	CTSH
PTGER41	1.93E-140	0.341454	0.545	0.364	5.43E-136	TAM-APClil	PTGER4

RPL36A	3.39E-140	0.291125	0.917	0.754	9.54E-136	TAM-APClil	RPL36A
HIGD2A1	2.27E-139	0.29002	0.601	0.421	6.38E-135	TAM-APClil	HIGD2A
RPL38	4.71E-139	0.309294	0.821	0.637	1.33E-134	TAM-APClil	RPL38
HCLS11	5.53E-139	0.288796	0.919	0.783	1.56E-134	TAM-APClil	HCLS1
RPS211	9.04E-139	0.278688	0.936	0.797	2.54E-134	TAM-APClil	RPS21
LRRK2	5.50E-137	0.271117	0.35	0.203	1.55E-132	TAM-APClil	LRRK2
ATP5MC21	1.40E-135	0.267252	0.884	0.712	3.94E-131	TAM-APClil	ATP5MC2
RPL35A1	4.32E-135	0.274624	0.943	0.815	1.22E-130	TAM-APClil	RPL35A
CLEC2B	1.79E-131	0.270484	0.467	0.303	5.03E-127	TAM-APClil	CLEC2B
FCGR2A	6.39E-130	0.278118	0.803	0.617	1.80E-125	TAM-APClil	FCGR2A
MAFB1	5.15E-128	0.457888	0.757	0.587	1.45E-123	TAM-APClil	MAFB
FCGRT1	8.97E-123	0.289588	0.858	0.701	2.52E-118	TAM-APClil	FCGRT
RPL27A	2.36E-122	0.26661	0.749	0.572	6.63E-118	TAM-APClil	RPL27A
YPEL3	2.83E-122	0.273082	0.61	0.432	7.95E-118	TAM-APClil	YPEL3
C1QC1	1.27E-121	0.338587	0.976	0.898	3.58E-117	TAM-APClil	C1QC
IL10	2.69E-121	0.42335	0.25	0.134	7.58E-117	TAM-APClil	IL10
BRI31	3.90E-121	0.261735	0.685	0.512	1.10E-116	TAM-APClil	BRI3
IL18	4.26E-121	0.295508	0.749	0.581	1.20E-116	TAM-APClil	IL18
RPS61	5.29E-121	0.264819	0.967	0.878	1.49E-116	TAM-APClil	RPS6
RPS2	3.24E-120	0.250488	0.964	0.846	9.12E-116	TAM-APClil	RPS2
HLA-C	1.08E-119	0.353287	0.952	0.822	3.03E-115	TAM-APClil	HLA-C
AC004817.	2.12E-119	0.348749	0.287	0.162	5.96E-115	TAM-APClil	AC004817.3
RASSF4	3.40E-119	0.258896	0.659	0.477	9.58E-115	TAM-APClil	RASSF4
RPL81	3.98E-118	0.251232	0.957	0.847	1.12E-113	TAM-APClil	RPL8
CD68	6.81E-118	0.276103	0.864	0.697	1.92E-113	TAM-APClil	CD68
RIPK2	8.77E-117	0.298478	0.376	0.236	2.47E-112	TAM-APClil	RIPK2
RPS29	2.68E-115	0.270245	0.814	0.644	7.55E-111	TAM-APClil	RPS29
RPL31	1.77E-113	0.25105	0.834	0.66	4.97E-109	TAM-APClil	RPL31
PNRC11	4.18E-113	0.267395	0.96	0.846	1.18E-108	TAM-APClil	PNRC1
GAS5	1.01E-112	0.26706	0.583	0.422	2.85E-108	TAM-APClil	GAS5
TAOK32	1.04E-109	0.256881	0.604	0.443	2.92E-105	TAM-APClil	TAOK3
PRKAG2-AS	1.26E-109	0.253798	0.258	0.145	3.55E-105	TAM-APClil	PRKAG2-AS1
EEF1G1	2.03E-108	0.261207	0.84	0.686	5.72E-104	TAM-APClil	EEF1G
TANK1	2.43E-108	0.264912	0.443	0.299	6.84E-104	TAM-APClil	TANK
KLF62	1.56E-107	0.282501	0.974	0.891	4.38E-103	TAM-APClil	KLF6
RHOB2	1.25E-105	0.266887	0.948	0.819	3.51E-101	TAM-APClil	RHOB
CTSD1	1.55E-101	0.265023	0.82	0.659	4.35E-97	TAM-APClil	CTSD
2-Jun	1.79E-99	0.354061	0.955	0.858	5.04E-95	TAM-APClil	JUN
RPS261	4.63E-97	0.273898	0.932	0.807	1.30E-92	TAM-APClil	RPS26
IFI30	2.52E-95	0.252645	0.832	0.682	7.09E-91	TAM-APClil	IFI30
PLTP1	1.38E-94	0.421216	0.329	0.207	3.89E-90	TAM-APClil	PLTP
NFKBIZ2	6.78E-94	0.291776	0.68	0.526	1.91E-89	TAM-APClil	NFKBIZ
TNFAIP32	3.35E-89	0.519038	0.662	0.545	9.41E-85	TAM-APClil	TNFAIP3
C1QB1	1.22E-88	0.273681	0.973	0.888	3.44E-84	TAM-APClil	C1QB
AC004448.	3.23E-84	0.440901	0.328	0.213	9.10E-80	TAM-APClil	AC004448.2
AL078590.:	9.18E-76	0.257553	0.574	0.432	2.58E-71	TAM-APClil	AL078590.2
C5AR11	5.20E-74	0.256392	0.663	0.512	1.46E-69	TAM-APClil	C5AR1

HCST	1.31E-70	0.252009	0.668	0.537	3.68E-66	TAM-APClil	HCST
IER22	2.56E-69	0.271496	0.925	0.829	7.20E-65	TAM-APClil	IER2
CXCL82	4.85E-69	0.348208	0.634	0.497	1.36E-64	TAM-APClil	CXCL8
CITED22	9.80E-42	0.304963	0.605	0.497	2.76E-37	TAM-APClil	CITED2
HLA-DRB51	2.63E-15	0.339585	0.753	0.69	7.40E-11	TAM-APClil	HLA-DRB5
AREG1	0	3.697971	0.779	0.124	0	cDC2	AREG
VIM1	0	2.285429	0.928	0.387	0	cDC2	VIM
S100A6	0	1.688561	0.709	0.159	0	cDC2	S100A6
HLA-DQA1:	0	1.590423	0.913	0.632	0	cDC2	HLA-DQA1
HLA-DPB11	0	1.582436	0.97	0.796	0	cDC2	HLA-DPB1
AFF3	0	1.556469	0.451	0.042	0	cDC2	AFF3
FCER1A	0	1.533234	0.357	0.014	0	cDC2	FCER1A
LYZ1	0	1.462113	0.725	0.247	0	cDC2	LYZ
EREG	0	1.411844	0.261	0.027	0	cDC2	EREG
HLA-DPA12	0	1.358449	0.984	0.872	0	cDC2	HLA-DPA1
HLA-DQB1:	0	1.322483	0.956	0.729	0	cDC2	HLA-DQB1
S100A10	0	1.2801	0.511	0.09	0	cDC2	S100A10
LSP1	0	1.278713	0.691	0.28	0	cDC2	LSP1
HLA-DRB12	0	1.260839	0.99	0.905	0	cDC2	HLA-DRB1
RGCC1	0	1.257457	0.627	0.259	0	cDC2	RGCC
DUSP5	0	1.234967	0.405	0.134	0	cDC2	DUSP5
IL1R2	0	1.209999	0.285	0.036	0	cDC2	IL1R2
FCGR2B1	0	1.186672	0.528	0.124	0	cDC2	FCGR2B
HLA-DRA1	0	1.183182	0.993	0.924	0	cDC2	HLA-DRA
CD44	0	1.182859	0.512	0.116	0	cDC2	CD44
CXCR42	0	1.18019	0.801	0.444	0	cDC2	CXCR4
S100A4	0	1.170435	0.648	0.27	0	cDC2	S100A4
CYTIP	0	1.131436	0.518	0.168	0	cDC2	CYTIP
IFITM21	0	1.114392	0.762	0.508	0	cDC2	IFITM2
METRNL1	0	1.10913	0.568	0.178	0	cDC2	METRNL
CRYBG1	0	1.104452	0.374	0.076	0	cDC2	CRYBG1
STK17B1	0	1.102061	0.725	0.385	0	cDC2	STK17B
FLT3	0	1.100458	0.339	0.015	0	cDC2	FLT3
CHPT11	0	1.090063	0.54	0.188	0	cDC2	CHPT1
CLEC10A	0	1.057247	0.269	0.012	0	cDC2	CLEC10A
AHNAK1	0	1.037007	0.584	0.169	0	cDC2	AHNAK
FXYD51	0	0.979086	0.841	0.555	0	cDC2	FXYD5
CALHM61	0	0.961521	0.583	0.251	0	cDC2	CALHM6
ISG20	0	0.960822	0.396	0.082	0	cDC2	ISG20
ANXA11	0	0.958337	0.69	0.327	0	cDC2	ANXA1
MS4A4E	0	0.957622	0.389	0.1	0	cDC2	MS4A4E
ANXA2	0	0.94498	0.426	0.087	0	cDC2	ANXA2
MS4A6A2	0	0.929551	0.796	0.528	0	cDC2	MS4A6A
CST32	0	0.92226	0.975	0.898	0	cDC2	CST3
GPAT3	0	0.921115	0.36	0.063	0	cDC2	GPAT3
C1orf1621	0	0.907099	0.804	0.542	0	cDC2	C1orf162
CD551	0	0.895805	0.66	0.339	0	cDC2	CD55

CNN2	0	0.890453	0.326	0.029	0	cDC2	CNN2
EMP3	0	0.878963	0.61	0.283	0	cDC2	EMP3
JAML	0	0.871903	0.329	0.029	0	cDC2	JAML
RPS182	0	0.86905	0.979	0.902	0	cDC2	RPS18
CD1632	0	0.865283	0.677	0.331	0	cDC2	CD163
GAPT1	0	0.865221	0.366	0.081	0	cDC2	GAPT
RGS22	0	0.861906	0.796	0.553	0	cDC2	RGS2
LRRFIP1	0	0.86021	0.788	0.517	0	cDC2	LRRFIP1
GPR1832	0	0.845849	0.841	0.635	0	cDC2	GPR183
AHR1	0	0.836113	0.424	0.135	0	cDC2	AHR
SAP301	0	0.824812	0.586	0.334	0	cDC2	SAP30
RPL282	0	0.813701	0.986	0.916	0	cDC2	RPL28
RPS142	0	0.803257	0.977	0.881	0	cDC2	RPS14
CKLF1	0	0.794778	0.621	0.365	0	cDC2	CKLF
RPL322	0	0.792881	0.982	0.905	0	cDC2	RPL32
RPL302	0	0.785668	0.976	0.89	0	cDC2	RPL30
RPS122	0	0.780497	0.986	0.912	0	cDC2	RPS12
LITAF1	0	0.78013	0.705	0.433	0	cDC2	LITAF
IFI301	0	0.77671	0.876	0.681	0	cDC2	IFI30
RPL18A2	0	0.773117	0.976	0.878	0	cDC2	RPL18A
TMSB101	0	0.772945	0.982	0.922	0	cDC2	TMSB10
RPL82	0	0.758657	0.958	0.848	0	cDC2	RPL8
RPL262	0	0.746651	0.97	0.883	0	cDC2	RPL26
RPL122	0	0.742078	0.972	0.889	0	cDC2	RPL12
RIPK21	0	0.739121	0.485	0.232	0	cDC2	RIPK2
RPS271	0	0.734621	0.966	0.873	0	cDC2	RPS27
APOO2	0	0.734404	0.55	0.269	0	cDC2	APOO
ARL4C	0	0.734051	0.33	0.128	0	cDC2	ARL4C
RPS4X2	0	0.731956	0.961	0.85	0	cDC2	RPS4X
PRKAR2B	0	0.710238	0.265	0.025	0	cDC2	PRKAR2B
RPS71	0	0.70804	0.959	0.857	0	cDC2	RPS7
RPL112	0	0.707821	0.975	0.897	0	cDC2	RPL11
GSTP12	0	0.707274	0.806	0.569	0	cDC2	GSTP1
RPS132	0	0.706856	0.973	0.882	0	cDC2	RPS13
RPL35A2	0	0.705737	0.943	0.816	0	cDC2	RPL35A
RPS15A2	0	0.705591	0.972	0.884	0	cDC2	RPS15A
RPS232	0	0.701964	0.981	0.906	0	cDC2	RPS23
RPL342	0	0.699521	0.968	0.876	0	cDC2	RPL34
RPS9	0	0.699471	0.962	0.856	0	cDC2	RPS9
RPL192	0	0.695833	0.975	0.902	0	cDC2	RPL19
RPL102	0	0.695096	0.993	0.944	0	cDC2	RPL10
PTMA2	0	0.695054	0.983	0.903	0	cDC2	PTMA
CD742	0	0.694365	0.999	0.99	0	cDC2	CD74
RPS212	0	0.693521	0.931	0.799	0	cDC2	RPS21
LGALS1	0	0.693482	0.452	0.197	0	cDC2	LGALS1
ARF6	0	0.692566	0.536	0.296	0	cDC2	ARF6
RPL132	0	0.69255	0.988	0.934	0	cDC2	RPL13

RPS241	0	0.691573	0.978	0.898	0	cDC2	RPS24
RPS31	0	0.691085	0.968	0.873	0	cDC2	RPS3
SEL1L3	0	0.690588	0.3	0.048	0	cDC2	SEL1L3
EEF1G2	0	0.690467	0.873	0.686	0	cDC2	EEF1G
CD1D	0	0.689908	0.296	0.051	0	cDC2	CD1D
RPS51	0	0.68072	0.914	0.762	0	cDC2	RPS5
PSTPIP2	0	0.680356	0.36	0.132	0	cDC2	PSTPIP2
CD48	0	0.678397	0.314	0.052	0	cDC2	CD48
PPDPF	0	0.677011	0.5	0.243	0	cDC2	PPDPF
RPS3A1	0	0.670083	0.963	0.872	0	cDC2	RPS3A
PPA1	0	0.66888	0.498	0.256	0	cDC2	PPA1
RPL362	0	0.6676	0.95	0.829	0	cDC2	RPL36
CDK14	0	0.664405	0.293	0.041	0	cDC2	CDK14
RPS152	0	0.662156	0.972	0.89	0	cDC2	RPS15
RPL271	0	0.652883	0.881	0.702	0	cDC2	RPL27
RPL23A1	0	0.642999	0.917	0.773	0	cDC2	RPL23A
RPS16	0	0.641404	0.938	0.824	0	cDC2	RPS16
ICAM3	0	0.637901	0.261	0.031	0	cDC2	ICAM3
RPL142	0	0.637845	0.956	0.85	0	cDC2	RPL14
RPL181	0	0.636037	0.961	0.87	0	cDC2	RPL18
RPLP21	0	0.63476	0.95	0.838	0	cDC2	RPLP2
FOSL2	0	0.633864	0.473	0.2	0	cDC2	FOSL2
RPL292	0	0.632973	0.966	0.875	0	cDC2	RPL29
RPL392	0	0.629248	0.966	0.879	0	cDC2	RPL39
RPLP11	0	0.628613	0.993	0.946	0	cDC2	RPLP1
TGFBI1	0	0.627718	0.421	0.146	0	cDC2	TGFBI
RPL371	0	0.626283	0.959	0.866	0	cDC2	RPL37
TIMP1	0	0.625561	0.41	0.181	0	cDC2	TIMP1
FYN	0	0.622374	0.252	0.039	0	cDC2	FYN
BTF32	0	0.621456	0.873	0.698	0	cDC2	BTF3
RACK12	0	0.620632	0.938	0.818	0	cDC2	RACK1
RPL10A1	0	0.618649	0.901	0.758	0	cDC2	RPL10A
SNX10	0	0.611892	0.448	0.182	0	cDC2	SNX10
RPS62	0	0.610294	0.967	0.879	0	cDC2	RPS6
SH3BP5	0	0.60859	0.337	0.109	0	cDC2	SH3BP5
RPS22	0	0.606091	0.954	0.847	0	cDC2	RPS2
RPS282	0	0.603847	0.963	0.874	0	cDC2	RPS28
EEF1B21	0	0.603092	0.9	0.755	0	cDC2	EEF1B2
CLEC4E1	0	0.600215	0.28	0.076	0	cDC2	CLEC4E
RPL36A1	0	0.599233	0.905	0.756	0	cDC2	RPL36A
RPL241	0	0.594447	0.934	0.81	0	cDC2	RPL24
RFTN1	0	0.592804	0.356	0.114	0	cDC2	RFTN1
EEF1A11	0	0.58135	0.996	0.963	0	cDC2	EEF1A1
FGR	0	0.57915	0.388	0.132	0	cDC2	FGR
RPL7A1	0	0.571579	0.963	0.865	0	cDC2	RPL7A
UBA521	0	0.570686	0.912	0.77	0	cDC2	UBA52
TES	0	0.570506	0.317	0.093	0	cDC2	TES

RPS81	0	0.567229	0.981	0.912	0	cDC2	RPS8
MCTP11	0	0.565757	0.432	0.177	0	cDC2	MCTP1
RPLP01	0	0.562133	0.964	0.864	0	cDC2	RPLP0
RPL352	0	0.560674	0.924	0.792	0	cDC2	RPL35
RPS252	0	0.553648	0.951	0.845	0	cDC2	RPS25
FAU2	0	0.543805	0.965	0.877	0	cDC2	FAU
NACA1	0	0.538272	0.944	0.83	0	cDC2	NACA
CSTA	0	0.53548	0.315	0.09	0	cDC2	CSTA
RPL33	0	0.532857	0.955	0.864	0	cDC2	RPL3
RPL61	0	0.53146	0.955	0.853	0	cDC2	RPL6
EMILIN2	0	0.522455	0.299	0.089	0	cDC2	EMILIN2
RPL151	0	0.521907	0.954	0.853	0	cDC2	RPL15
RPL171	0	0.515451	0.945	0.83	0	cDC2	RPL17
AC138123.	0	0.494577	0.34	0.13	0	cDC2	AC138123.1
RPS191	0	0.492909	0.984	0.919	0	cDC2	RPS19
ITGA41	0	0.473366	0.282	0.076	0	cDC2	ITGA4
TPT11	0	0.471515	0.992	0.954	0	cDC2	TPT1
RPL221	6.27E-307	0.561571	0.903	0.747	1.77E-302	cDC2	RPL22
FTH12	6.44E-307	0.530979	0.997	0.983	1.81E-302	cDC2	FTH1
NME21	2.24E-306	0.566589	0.864	0.657	6.30E-302	cDC2	NME2
MAP3K82	6.17E-306	0.753157	0.835	0.657	1.73E-301	cDC2	MAP3K8
DENND1B	4.80E-303	0.480223	0.26	0.089	1.35E-298	cDC2	DENND1B
PLP2	3.54E-298	0.452982	0.306	0.117	9.96E-294	cDC2	PLP2
RPS27A1	9.56E-296	0.471523	0.965	0.879	2.69E-291	cDC2	RPS27A
RPL42	2.27E-294	0.577837	0.885	0.732	6.38E-290	cDC2	RPL4
AC004817.	4.25E-294	0.661473	0.37	0.16	1.20E-289	cDC2	AC004817.3
PLAUR1	6.63E-294	0.816381	0.758	0.526	1.87E-289	cDC2	PLAUR
PRMT9	4.65E-286	0.550847	0.356	0.152	1.31E-281	cDC2	PRMT9
CTSH1	7.23E-286	0.60302	0.754	0.533	2.04E-281	cDC2	CTSH
H3F3A	3.85E-283	0.539479	0.913	0.77	1.08E-278	cDC2	H3F3A
AP1S21	1.62E-282	0.506141	0.419	0.199	4.55E-278	cDC2	AP1S2
RPS291	7.50E-281	0.685906	0.822	0.645	2.11E-276	cDC2	RPS29
BTG11	3.99E-279	0.737311	0.884	0.752	1.12E-274	cDC2	BTG1
MYL12A	1.69E-278	0.68359	0.741	0.533	4.76E-274	cDC2	MYL12A
FGL21	4.12E-278	0.689087	0.654	0.411	1.16E-273	cDC2	FGL2
RPSA1	1.45E-276	0.553467	0.878	0.711	4.07E-272	cDC2	RPSA
IFITM1	5.99E-273	1.043443	0.275	0.108	1.69E-268	cDC2	IFITM1
RPS262	1.13E-272	0.609119	0.934	0.808	3.19E-268	cDC2	RPS26
TNFAIP8	2.13E-269	0.541916	0.313	0.13	5.99E-265	cDC2	TNFAIP8
COMMD6	1.48E-268	0.568007	0.764	0.544	4.18E-264	cDC2	COMMD6
RPL51	1.40E-260	0.483314	0.941	0.833	3.94E-256	cDC2	RPL5
SIPA1L12	3.73E-260	0.475504	0.8	0.553	1.05E-255	cDC2	SIPA1L1
RAC2	1.60E-259	0.466048	0.32	0.136	4.49E-255	cDC2	RAC2
ATP1B32	7.05E-259	0.669736	0.705	0.489	1.98E-254	cDC2	ATP1B3
TAGLN2	8.25E-259	0.650445	0.536	0.305	2.32E-254	cDC2	TAGLN2
HBEGF1	3.50E-257	0.714351	0.523	0.289	9.85E-253	cDC2	HBEGF
RBFOX2	3.77E-253	0.434611	0.268	0.102	1.06E-248	cDC2	RBFOX2

BASP12	3.54E-251	0.546916	0.785	0.552	9.95E-247	cDC2	BASP1
FOXN2	4.92E-250	0.55001	0.366	0.171	1.38E-245	cDC2	FOXN2
RPS11	5.69E-248	0.512644	0.85	0.685	1.60E-243	cDC2	RPS11
RPL311	6.96E-245	0.529324	0.837	0.662	1.96E-240	cDC2	RPL31
EIF3K1	5.18E-243	0.511811	0.774	0.569	1.46E-238	cDC2	EIF3K
VEGFA1	2.12E-242	0.583216	0.326	0.143	5.95E-238	cDC2	VEGFA
OSBPL81	1.62E-241	0.558793	0.571	0.344	4.55E-237	cDC2	OSBPL8
IQGAP21	4.24E-238	0.357094	0.299	0.122	1.19E-233	cDC2	IQGAP2
GNG2	2.12E-235	0.461271	0.285	0.118	5.97E-231	cDC2	GNG2
RPL37A1	2.65E-233	0.47344	0.902	0.774	7.46E-229	cDC2	RPL37A
KYNU	2.83E-233	0.508047	0.408	0.205	7.96E-229	cDC2	KYNU
RPL27A1	4.27E-233	0.529945	0.769	0.573	1.20E-228	cDC2	RPL27A
TUBA1A	2.62E-227	0.853655	0.496	0.294	7.38E-223	cDC2	TUBA1A
CARD161	6.71E-224	0.428353	0.358	0.169	1.89E-219	cDC2	CARD16
IQGAP11	4.03E-221	0.406178	0.515	0.286	1.13E-216	cDC2	IQGAP1
COX4I11	1.64E-220	0.421035	0.889	0.733	4.63E-216	cDC2	COX4I1
HERPUD12	1.03E-219	0.648356	0.843	0.698	2.90E-215	cDC2	HERPUD1
EEF21	8.73E-219	0.451513	0.901	0.758	2.46E-214	cDC2	EEF2
RPL13A1	2.35E-218	0.461226	0.882	0.738	6.62E-214	cDC2	RPL13A
VAMP51	2.80E-216	0.449692	0.312	0.141	7.89E-212	cDC2	VAMP5
PFDN51	1.98E-211	0.428485	0.879	0.719	5.57E-207	cDC2	PFDN5
FILIP1L1	2.49E-211	0.566942	0.464	0.26	7.00E-207	cDC2	FILIP1L
RALA	3.37E-211	0.421993	0.265	0.112	9.48E-207	cDC2	RALA
HINT11	1.62E-210	0.509573	0.656	0.44	4.55E-206	cDC2	HINT1
GAPDH1	7.64E-209	0.389861	0.953	0.863	2.15E-204	cDC2	GAPDH
RPL9	2.21E-208	0.441629	0.914	0.792	6.22E-204	cDC2	RPL9
INSIG1	4.18E-207	0.75437	0.401	0.213	1.17E-202	cDC2	INSIG1
RPL7	1.59E-204	0.464306	0.791	0.608	4.47E-200	cDC2	RPL7
ATP5MG1	7.59E-203	0.442608	0.797	0.605	2.14E-198	cDC2	ATP5MG
HLA-DMA2	4.31E-202	0.453254	0.876	0.703	1.21E-197	cDC2	HLA-DMA
RPL381	4.68E-196	0.50263	0.803	0.639	1.32E-191	cDC2	RPL38
PLBD11	6.83E-192	0.305672	0.288	0.129	1.92E-187	cDC2	PLBD1
ZFAND52	2.85E-191	0.711582	0.686	0.491	8.01E-187	cDC2	ZFAND5
MIR181A1I	2.38E-189	0.391891	0.432	0.233	6.70E-185	cDC2	MIR181A1HG
MZT2B	1.06E-188	0.41108	0.604	0.391	2.97E-184	cDC2	MZT2B
ODF3B	2.26E-187	0.455651	0.388	0.205	6.37E-183	cDC2	ODF3B
SNRPD2	2.99E-183	0.419387	0.625	0.418	8.40E-179	cDC2	SNRPD2
IFITM31	9.74E-181	0.713048	0.67	0.455	2.74E-176	cDC2	IFITM3
UPP1	2.89E-179	0.348752	0.276	0.126	8.12E-175	cDC2	UPP1
ATP5F1E1	3.67E-177	0.381954	0.887	0.746	1.03E-172	cDC2	ATP5F1E
CLIC1	5.66E-177	0.447698	0.818	0.65	1.59E-172	cDC2	CLIC1
SRGN1	1.06E-176	0.393677	0.979	0.936	2.99E-172	cDC2	SRGN
BCL21	1.10E-176	0.433267	0.559	0.353	3.08E-172	cDC2	BCL2
UQCRH	5.78E-176	0.422542	0.638	0.437	1.63E-171	cDC2	UQCRH
HIGD2A2	1.28E-175	0.424807	0.627	0.421	3.60E-171	cDC2	HIGD2A
MZT2A	6.80E-174	0.335824	0.3	0.145	1.91E-169	cDC2	MZT2A
FNBP1	1.84E-173	0.420768	0.42	0.235	5.17E-169	cDC2	FNBP1

CD91	3.38E-170	0.421778	0.295	0.143	9.51E-166	cDC2	CD9
SLC25A6	3.76E-170	0.42856	0.808	0.649	1.06E-165	cDC2	SLC25A6
FP236383.3	4.47E-170	0.365104	0.745	0.494	1.26E-165	cDC2	FP236383.3
DNAJC15	8.24E-170	0.406261	0.488	0.298	2.32E-165	cDC2	DNAJC15
PLXNC1	8.68E-168	0.311548	0.286	0.136	2.44E-163	cDC2	PLXNC1
TET21	4.06E-167	0.435259	0.512	0.317	1.14E-162	cDC2	TET2
H2AFY	4.71E-167	0.429402	0.691	0.509	1.32E-162	cDC2	H2AFY
RHOH1	7.54E-167	0.585866	0.419	0.247	2.12E-162	cDC2	RHOH
ETV3	1.37E-165	0.40948	0.316	0.16	3.86E-161	cDC2	ETV3
YBX11	3.58E-165	0.377244	0.825	0.659	1.01E-160	cDC2	YBX1
RPS17	3.95E-165	0.434296	0.642	0.458	1.11E-160	cDC2	RPS17
PPIA1	9.69E-165	0.349685	0.918	0.808	2.73E-160	cDC2	PPIA
SNHG291	1.76E-164	0.433088	0.723	0.542	4.97E-160	cDC2	SNHG29
MIF1	3.67E-164	0.374593	0.734	0.53	1.03E-159	cDC2	MIF
GAS51	4.26E-163	0.440518	0.615	0.422	1.20E-158	cDC2	GAS5
PRELID1	9.96E-163	0.42646	0.442	0.264	2.80E-158	cDC2	PRELID1
DOCK5	8.86E-162	0.285196	0.342	0.175	2.49E-157	cDC2	DOCK5
PTRHD1	2.85E-161	0.311694	0.265	0.125	8.02E-157	cDC2	PTRHD1
SERP1	5.35E-160	0.389598	0.809	0.657	1.50E-155	cDC2	SERP1
PAK1	5.55E-160	0.464748	0.459	0.281	1.56E-155	cDC2	PAK1
SLC25A51	7.16E-160	0.397046	0.731	0.543	2.02E-155	cDC2	SLC25A5
TMEM123	2.21E-159	0.420896	0.435	0.258	6.23E-155	cDC2	TMEM123
TXNIP1	1.62E-158	0.718967	0.496	0.321	4.56E-154	cDC2	TXNIP
ATP2B1	1.30E-157	0.509016	0.549	0.367	3.65E-153	cDC2	ATP2B1
FBL	2.21E-156	0.336226	0.337	0.178	6.21E-152	cDC2	FBL
DUSP22	2.56E-156	0.614855	0.581	0.389	7.19E-152	cDC2	DUSP2
BHLHE401	6.37E-156	0.541402	0.447	0.273	1.79E-151	cDC2	BHLHE40
PABPC1	1.37E-155	0.366117	0.933	0.84	3.87E-151	cDC2	PABPC1
LY6E	4.55E-155	0.552108	0.375	0.202	1.28E-150	cDC2	LY6E
AC004448.	4.26E-154	0.755421	0.372	0.212	1.20E-149	cDC2	AC004448.2
EEF1D1	2.05E-153	0.369631	0.841	0.687	5.76E-149	cDC2	EEF1D
RPL23	3.05E-153	0.421524	0.713	0.539	8.59E-149	cDC2	RPL23
ATP5MC22	1.84E-152	0.351135	0.869	0.714	5.18E-148	cDC2	ATP5MC2
SMARCA21	1.41E-150	0.437313	0.481	0.304	3.95E-146	cDC2	SMARCA2
TSPAN33	6.71E-149	0.299577	0.263	0.127	1.89E-144	cDC2	TSPAN33
CHMP1B	1.57E-148	0.52946	0.436	0.264	4.42E-144	cDC2	CHMP1B
PSME11	1.86E-147	0.384058	0.61	0.423	5.24E-143	cDC2	PSME1
ZFP36L21	1.59E-145	0.500693	0.913	0.84	4.46E-141	cDC2	ZFP36L2
PTK2B1	6.41E-145	0.3102	0.433	0.257	1.80E-140	cDC2	PTK2B
HMGB21	4.87E-144	0.43927	0.431	0.261	1.37E-139	cDC2	HMGB2
CIB11	2.16E-141	0.356139	0.498	0.319	6.07E-137	cDC2	CIB1
LCP1	3.24E-141	0.435244	0.61	0.429	9.11E-137	cDC2	LCP1
TXN	5.37E-141	0.385961	0.333	0.179	1.51E-136	cDC2	TXN
SMCHD1	7.16E-141	0.433634	0.439	0.27	2.02E-136	cDC2	SMCHD1
PDE4A1	7.41E-140	0.371353	0.403	0.238	2.09E-135	cDC2	PDE4A
TLE5	3.17E-139	0.308661	0.354	0.197	8.91E-135	cDC2	TLE5
COX5A1	1.33E-138	0.364671	0.592	0.407	3.73E-134	cDC2	COX5A

PRCP	1.65E-138	0.344521	0.414	0.247	4.63E-134	cDC2	PRCP
ANP32B1	5.81E-138	0.349197	0.552	0.37	1.63E-133	cDC2	ANP32B
DNAJC4	7.93E-138	0.355428	0.353	0.201	2.23E-133	cDC2	DNAJC4
RNF144B2	2.01E-137	0.380123	0.474	0.298	5.65E-133	cDC2	RNF144B
LIMD2	2.92E-136	0.373009	0.506	0.328	8.22E-132	cDC2	LIMD2
TKT2	8.80E-136	0.342743	0.573	0.392	2.48E-131	cDC2	TKT
UTRN2	1.08E-135	0.27206	0.48	0.295	3.05E-131	cDC2	UTRN
SEC11A1	1.36E-134	0.363562	0.676	0.499	3.83E-130	cDC2	SEC11A
JARID21	1.61E-134	0.319441	0.439	0.267	4.53E-130	cDC2	JARID2
C7orf50	3.46E-134	0.297086	0.28	0.144	9.75E-130	cDC2	C7orf50
EIF3E	7.55E-134	0.363552	0.602	0.427	2.12E-129	cDC2	EIF3E
RASA2	7.67E-134	0.346616	0.299	0.158	2.16E-129	cDC2	RASA2
ERP291	9.23E-134	0.378045	0.575	0.4	2.60E-129	cDC2	ERP29
TAX1BP3	1.92E-133	0.303629	0.26	0.131	5.42E-129	cDC2	TAX1BP3
NAMPT2	2.72E-133	0.320773	0.725	0.533	7.65E-129	cDC2	NAMPT
SARAF	1.82E-132	0.367019	0.862	0.744	5.13E-128	cDC2	SARAF
RHOC	5.92E-132	0.347019	0.411	0.248	1.67E-127	cDC2	RHOC
SMDT11	1.01E-128	0.352864	0.526	0.352	2.86E-124	cDC2	SMDT1
GLO1	6.60E-128	0.333301	0.25	0.127	1.86E-123	cDC2	GLO1
APRT1	1.17E-126	0.322546	0.539	0.363	3.30E-122	cDC2	APRT
PTPN12	1.77E-126	0.292366	0.588	0.4	4.99E-122	cDC2	PTPN1
SAMHD11	6.12E-126	0.389296	0.657	0.49	1.72E-121	cDC2	SAMHD1
ATG3	2.21E-125	0.297133	0.366	0.212	6.21E-121	cDC2	ATG3
GSTK1	1.08E-124	0.332434	0.546	0.373	3.03E-120	cDC2	GSTK1
ZYX1	2.41E-124	0.319676	0.622	0.437	6.79E-120	cDC2	ZYX
LDHA	1.36E-123	0.362407	0.624	0.445	3.83E-119	cDC2	LDHA
PTEN	1.39E-123	0.338192	0.462	0.297	3.92E-119	cDC2	PTEN
COX7C	3.21E-123	0.358703	0.751	0.592	9.02E-119	cDC2	COX7C
EIF3G1	1.71E-122	0.334331	0.64	0.464	4.81E-118	cDC2	EIF3G
KLF42	1.48E-121	0.423216	0.678	0.502	4.17E-117	cDC2	KLF4
PHACTR1	1.75E-120	0.360064	0.584	0.408	4.92E-116	cDC2	PHACTR1
EVI2B1	3.59E-120	0.361671	0.577	0.404	1.01E-115	cDC2	EVI2B
VAMP82	7.19E-120	0.328425	0.692	0.517	2.02E-115	cDC2	VAMP8
FP671120.4	2.83E-119	0.292993	0.617	0.409	7.97E-115	cDC2	FP671120.4
SSR21	4.95E-119	0.320455	0.561	0.39	1.39E-114	cDC2	SSR2
STX112	9.64E-119	0.308918	0.54	0.359	2.71E-114	cDC2	STX11
PFN11	3.87E-118	0.305631	0.923	0.823	1.09E-113	cDC2	PFN1
GRN1	4.39E-118	0.328262	0.805	0.662	1.23E-113	cDC2	GRN
ENO11	4.24E-116	0.310581	0.644	0.47	1.19E-111	cDC2	ENO1
HNRNPA1	4.57E-116	0.345811	0.806	0.665	1.29E-111	cDC2	HNRNPA1
RNASE6	6.01E-115	0.475249	0.499	0.346	1.69E-110	cDC2	RNASE6
CAT	2.96E-114	0.277068	0.34	0.199	8.34E-110	cDC2	CAT
CCNI	5.68E-114	0.332038	0.735	0.581	1.60E-109	cDC2	CCNI
OGDH	2.79E-113	0.26067	0.256	0.135	7.84E-109	cDC2	OGDH
PSMA6	8.60E-113	0.326267	0.461	0.307	2.42E-108	cDC2	PSMA6
GK1	2.66E-112	0.368185	0.519	0.352	7.48E-108	cDC2	GK
OAZ11	5.86E-112	0.286281	0.911	0.792	1.65E-107	cDC2	OAZ1

CERS6	1.76E-111	0.263657	0.279	0.151	4.96E-107	cDC2	CERS6
ALDOA1	1.37E-110	0.272737	0.626	0.449	3.86E-106	cDC2	ALDOA
CREB51	1.61E-110	0.263628	0.263	0.139	4.52E-106	cDC2	CREB5
PTPRE1	2.59E-110	0.309817	0.668	0.499	7.29E-106	cDC2	PTPRE
EIF3L	3.45E-110	0.354267	0.575	0.421	9.71E-106	cDC2	EIF3L
ESYT2	5.43E-109	0.275232	0.324	0.186	1.53E-104	cDC2	ESYT2
NPM1	2.17E-108	0.305161	0.836	0.705	6.11E-104	cDC2	NPM1
COX8A	7.75E-108	0.267936	0.645	0.467	2.18E-103	cDC2	COX8A
EIF3F1	2.72E-107	0.327189	0.654	0.499	7.66E-103	cDC2	EIF3F
HIPK2	7.11E-107	0.27682	0.315	0.181	2.00E-102	cDC2	HIPK2
PSME21	4.02E-106	0.299023	0.507	0.344	1.13E-101	cDC2	PSME2
EFHD2	5.46E-106	0.328825	0.516	0.359	1.54E-101	cDC2	EFHD2
EIF3H1	9.45E-106	0.289839	0.632	0.468	2.66E-101	cDC2	EIF3H
PLAC81	1.09E-105	0.518115	0.373	0.236	3.06E-101	cDC2	PLAC8
PTGER42	6.82E-105	0.322813	0.535	0.366	1.92E-100	cDC2	PTGER4
MALT11	1.14E-104	0.280046	0.409	0.258	3.21E-100	cDC2	MALT1
SNX31	1.17E-104	0.444667	0.642	0.485	3.29E-100	cDC2	SNX3
C9orf161	5.41E-104	0.259864	0.499	0.335	1.52E-99	cDC2	C9orf16
TRAPPC51	8.85E-104	0.254964	0.429	0.275	2.49E-99	cDC2	TRAPPC5
ATP5F1D1	1.99E-103	0.271635	0.641	0.471	5.61E-99	cDC2	ATP5F1D
RILPL21	2.26E-103	0.301897	0.711	0.543	6.35E-99	cDC2	RILPL2
NOP53	4.20E-103	0.301198	0.588	0.424	1.18E-98	cDC2	NOP53
PSMB91	1.90E-101	0.331714	0.421	0.275	5.34E-97	cDC2	PSMB9
EZR	1.91E-101	0.331019	0.542	0.386	5.39E-97	cDC2	EZR
GLIPR1	4.87E-101	0.295077	0.642	0.478	1.37E-96	cDC2	GLIPR1
AC007384.	3.24E-100	0.290217	0.319	0.188	9.11E-96	cDC2	AC007384.1
SPINT2	2.80E-99	0.335965	0.425	0.285	7.87E-95	cDC2	SPINT2
HMGA1	2.95E-99	0.253898	0.352	0.216	8.31E-95	cDC2	HMGA1
UXT	4.84E-99	0.27208	0.47	0.32	1.36E-94	cDC2	UXT
NUDT16	7.54E-99	0.335524	0.382	0.244	2.12E-94	cDC2	NUDT16
RPL36AL1	6.40E-98	0.276393	0.809	0.652	1.80E-93	cDC2	RPL36AL
ZNF385A	6.42E-98	0.26789	0.319	0.19	1.81E-93	cDC2	ZNF385A
EDF11	1.76E-97	0.260747	0.64	0.476	4.96E-93	cDC2	EDF1
TOMM7	4.39E-96	0.28017	0.706	0.554	1.23E-91	cDC2	TOMM7
AKIRIN2	5.55E-96	0.327296	0.445	0.298	1.56E-91	cDC2	AKIRIN2
PPP1CB1	5.63E-96	0.287893	0.381	0.243	1.58E-91	cDC2	PPP1CB
HIVEP21	3.09E-95	0.323199	0.44	0.294	8.71E-91	cDC2	HIVEP2
HMGN1	5.86E-95	0.289369	0.61	0.456	1.65E-90	cDC2	HMGN1
INSR	1.02E-94	0.255583	0.295	0.171	2.86E-90	cDC2	INSR
LDHB1	3.12E-94	0.281403	0.481	0.33	8.78E-90	cDC2	LDHB
GPR1322	1.92E-93	0.348779	0.418	0.278	5.40E-89	cDC2	GPR132
PRDX6	3.34E-93	0.275327	0.494	0.342	9.41E-89	cDC2	PRDX6
HLA-DRB52	1.36E-92	0.668586	0.81	0.688	3.81E-88	cDC2	HLA-DRB5
SAT12	2.13E-92	0.307868	0.982	0.97	6.01E-88	cDC2	SAT1
ARHGDIB1	9.98E-92	0.273109	0.828	0.695	2.81E-87	cDC2	ARHGDIB
STK38L	1.28E-91	0.343179	0.47	0.324	3.59E-87	cDC2	STK38L
TMEM14C1	2.54E-91	0.261849	0.563	0.406	7.15E-87	cDC2	TMEM14C

EIF2AK3	8.66E-91	0.308454	0.291	0.172	2.44E-86	cDC2	EIF2AK3
HACD41	1.48E-90	0.259245	0.403	0.264	4.16E-86	cDC2	HACD4
NSA2	3.77E-90	0.252013	0.453	0.305	1.06E-85	cDC2	NSA2
SSR3	2.39E-89	0.266482	0.499	0.351	6.74E-85	cDC2	SSR3
SUB1	3.86E-89	0.283794	0.592	0.443	1.09E-84	cDC2	SUB1
JMJD1C1	8.33E-89	0.26257	0.735	0.588	2.34E-84	cDC2	JMJD1C
PRKAG2-AS	2.52E-88	0.268168	0.256	0.146	7.10E-84	cDC2	PRKAG2-AS1
PLSCR1	1.32E-87	0.294812	0.621	0.468	3.72E-83	cDC2	PLSCR1
SH3BGRL31	5.17E-87	0.289125	0.799	0.66	1.45E-82	cDC2	SH3BGRL3
MNDA1	1.39E-86	0.293686	0.447	0.307	3.90E-82	cDC2	MNDA
TAF101	2.59E-86	0.259466	0.608	0.453	7.27E-82	cDC2	TAF10
UBALD2	3.08E-85	0.372058	0.402	0.272	8.68E-81	cDC2	UBALD2
MYADM1	3.21E-85	0.30129	0.479	0.332	9.04E-81	cDC2	MYADM
HLA-DOA	6.63E-85	0.252201	0.326	0.205	1.86E-80	cDC2	HLA-DOA
CYB5R3	8.74E-85	0.253656	0.348	0.223	2.46E-80	cDC2	CYB5R3
NAP1L1	2.70E-84	0.300403	0.567	0.425	7.60E-80	cDC2	NAP1L1
ATP2B1-AS	2.50E-83	0.409865	0.635	0.495	7.03E-79	cDC2	ATP2B1-AS1
AP2S11	1.24E-82	0.252064	0.48	0.339	3.49E-78	cDC2	AP2S1
MYL12B	1.43E-81	0.26083	0.695	0.552	4.02E-77	cDC2	MYL12B
SERPINA11	1.66E-81	0.315253	0.671	0.537	4.68E-77	cDC2	SERPINA1
TLR22	2.57E-80	0.321793	0.638	0.506	7.22E-76	cDC2	TLR2
CMSS11	3.06E-80	0.277402	0.364	0.237	8.61E-76	cDC2	CMSS1
MSN	7.88E-78	0.263521	0.674	0.534	2.22E-73	cDC2	MSN
RSL24D1	7.91E-78	0.262647	0.365	0.242	2.23E-73	cDC2	RSL24D1
RPS20	2.35E-77	0.272326	0.629	0.489	6.62E-73	cDC2	RPS20
TGIF11	4.48E-77	0.271854	0.377	0.25	1.26E-72	cDC2	TGIF1
DYNLT1	1.28E-76	0.255408	0.433	0.301	3.59E-72	cDC2	DYNLT1
SNHG15	4.88E-76	0.277843	0.297	0.186	1.37E-71	cDC2	SNHG15
CXCL161	3.23E-75	0.330353	0.765	0.637	9.10E-71	cDC2	CXCL16
ARPC2	1.52E-74	0.259292	0.76	0.632	4.29E-70	cDC2	ARPC2
HPS5	3.30E-73	0.258136	0.344	0.226	9.28E-69	cDC2	HPS5
HLA-A1	3.53E-72	0.272487	0.922	0.834	9.94E-68	cDC2	HLA-A
BCL2A11	3.65E-72	0.398555	0.646	0.499	1.03E-67	cDC2	BCL2A1
DDIT42	2.19E-68	0.301669	0.664	0.527	6.17E-64	cDC2	DDIT4
ISG15	3.79E-65	0.389246	0.281	0.181	1.07E-60	cDC2	ISG15
COTL11	4.39E-62	0.279166	0.809	0.692	1.23E-57	cDC2	COTL1
GLUD1	1.14E-60	0.27699	0.376	0.264	3.21E-56	cDC2	GLUD1
NLRP32	1.51E-60	0.363693	0.629	0.515	4.25E-56	cDC2	NLRP3
GABARAPL	2.40E-60	0.253801	0.429	0.313	6.75E-56	cDC2	GABARAPL1
HDAC92	6.11E-59	0.295319	0.539	0.409	1.72E-54	cDC2	HDAC9
HLA-C1	6.39E-58	0.289006	0.921	0.825	1.80E-53	cDC2	HLA-C
TNFAIP33	2.49E-38	0.341168	0.64	0.547	7.00E-34	cDC2	TNFAIP3
RPS4Y11	9.08E-38	0.283803	0.503	0.426	2.55E-33	cDC2	RPS4Y1
ACSL3	9.12E-37	0.294919	0.34	0.258	2.57E-32	cDC2	ACSL3
PLIN21	2.86E-28	0.304943	0.409	0.326	8.05E-24	cDC2	PLIN2
S100A9	0	4.295392	0.671	0.146	0	Monocytes	S100A9
S100A8	0	4.124504	0.631	0.15	0	Monocytes	S100A8

S100A61	0	3.581808	0.866	0.165	0	Monocytes	S100A6
LYZ2	0	3.439577	0.887	0.251	0	Monocytes	LYZ
THBS1	0	3.074404	0.542	0.018	0	Monocytes	THBS1
CXCL2	0	3.058377	0.485	0.039	0	Monocytes	CXCL2
S100A41	0	3.005539	0.818	0.273	0	Monocytes	S100A4
VCAN	0	2.956464	0.62	0.018	0	Monocytes	VCAN
S100A101	0	2.863995	0.76	0.091	0	Monocytes	S100A10
TIMP11	0	2.858633	0.736	0.176	0	Monocytes	TIMP1
EREG1	0	2.745637	0.519	0.024	0	Monocytes	EREG
CXCL3	0	2.676228	0.298	0.011	0	Monocytes	CXCL3
AREG2	0	2.659718	0.726	0.139	0	Monocytes	AREG
FCN1	0	2.592979	0.477	0.006	0	Monocytes	FCN1
S100A12	0	2.487234	0.338	0.007	0	Monocytes	S100A12
VIM2	0	2.391815	0.97	0.397	0	Monocytes	VIM
CD441	0	2.225937	0.714	0.118	0	Monocytes	CD44
LGALS11	0	2.220461	0.719	0.194	0	Monocytes	LGALS1
IL1R21	0	2.046815	0.398	0.037	0	Monocytes	IL1R2
ANXA21	0	1.813374	0.646	0.088	0	Monocytes	ANXA2
IFI302	0	1.773715	0.948	0.683	0	Monocytes	IFI30
LGALS3	0	1.766337	0.521	0.039	0	Monocytes	LGALS3
SH3BGRL32	0	1.588919	0.891	0.66	0	Monocytes	SH3BGRL3
FLNA	0	1.547055	0.469	0.047	0	Monocytes	FLNA
CSTA1	0	1.52326	0.577	0.087	0	Monocytes	CSTA
PLCB1	0	1.507366	0.359	0.013	0	Monocytes	PLCB1
FNDC3B1	0	1.481802	0.565	0.243	0	Monocytes	FNDC3B
AQP9	0	1.480975	0.371	0.039	0	Monocytes	AQP9
PLAUR2	0	1.476974	0.872	0.528	0	Monocytes	PLAUR
CD52	0	1.469239	0.346	0.022	0	Monocytes	CD52
EMP31	0	1.458374	0.737	0.286	0	Monocytes	EMP3
UPP11	0	1.457624	0.568	0.12	0	Monocytes	UPP1
IFITM22	0	1.45547	0.806	0.512	0	Monocytes	IFITM2
PPDPF1	0	1.409991	0.673	0.243	0	Monocytes	PPDPF
JARID22	0	1.401501	0.608	0.265	0	Monocytes	JARID2
FGR1	0	1.393693	0.594	0.131	0	Monocytes	FGR
ATP2B11	0	1.375923	0.673	0.367	0	Monocytes	ATP2B1
CRIP1	0	1.36441	0.336	0.026	0	Monocytes	CRIP1
LSP11	0	1.340029	0.741	0.286	0	Monocytes	LSP1
SMIM25	0	1.337664	0.404	0.055	0	Monocytes	SMIM25
KYNU1	0	1.335141	0.579	0.204	0	Monocytes	KYNU
SLC16A10	0	1.317226	0.259	0.034	0	Monocytes	SLC16A10
GAPDH2	0	1.30829	0.962	0.864	0	Monocytes	GAPDH
NAMPT3	0	1.307319	0.865	0.533	0	Monocytes	NAMPT
ANXA12	0	1.221639	0.779	0.332	0	Monocytes	ANXA1
CLEC4E2	0	1.204692	0.458	0.075	0	Monocytes	CLEC4E
TKT3	0	1.200025	0.716	0.391	0	Monocytes	TKT
AHNAK2	0	1.183536	0.576	0.177	0	Monocytes	AHNAK
TYMP	0	1.176659	0.659	0.344	0	Monocytes	TYMP

AC015912.	0	1.153852	0.336	0.073	0	Monocytes	AC015912.3
EMILIN21	0	1.129476	0.466	0.088	0	Monocytes	EMILIN2
SESTD1	0	1.12669	0.336	0.047	0	Monocytes	SESTD1
CD1633	0	1.12549	0.757	0.336	0	Monocytes	CD163
CTSS1	0	1.114446	0.855	0.616	0	Monocytes	CTSS
NCF2	0	1.112915	0.482	0.125	0	Monocytes	NCF2
SERPINA12	0	1.105742	0.804	0.536	0	Monocytes	SERPINA1
BLVRB	0	1.094628	0.506	0.183	0	Monocytes	BLVRB
CFP	0	1.077861	0.296	0.007	0	Monocytes	CFP
CD481	0	1.077682	0.437	0.053	0	Monocytes	CD48
METRNL2	0	1.069626	0.576	0.185	0	Monocytes	METRNL
CSGALNAC	0	1.055677	0.423	0.084	0	Monocytes	CSGALNACT2
FTH13	0	1.047277	0.998	0.984	0	Monocytes	FTH1
RPL283	0	1.035012	0.98	0.918	0	Monocytes	RPL28
RBP7	0	0.993228	0.258	0.015	0	Monocytes	RBP7
LILRA5	0	0.98512	0.269	0.006	0	Monocytes	LILRA5
PLBD12	0	0.981835	0.396	0.129	0	Monocytes	PLBD1
MXD1	0	0.972011	0.44	0.126	0	Monocytes	MXD1
LILRB2	0	0.970216	0.328	0.059	0	Monocytes	LILRB2
TGFBI2	0	0.966684	0.505	0.149	0	Monocytes	TGFBI
TXN1	0	0.964241	0.491	0.178	0	Monocytes	TXN
TMSB102	0	0.962681	0.983	0.923	0	Monocytes	TMSB10
SH3BP51	0	0.961044	0.441	0.111	0	Monocytes	SH3BP5
PID1	0	0.953522	0.303	0.049	0	Monocytes	PID1
MCTP12	0	0.918049	0.493	0.181	0	Monocytes	MCTP1
JAML1	0	0.915375	0.358	0.034	0	Monocytes	JAML
CST33	0	0.913487	0.954	0.9	0	Monocytes	CST3
TREM1	0	0.912169	0.504	0.194	0	Monocytes	TREM1
KCNE1	0	0.909659	0.281	0.02	0	Monocytes	KCNE1
AGTRAP	0	0.908479	0.455	0.148	0	Monocytes	AGTRAP
EHD1	0	0.9048	0.27	0.036	0	Monocytes	EHD1
H3F3A1	0	0.897622	0.92	0.773	0	Monocytes	H3F3A
RPS91	0	0.890303	0.971	0.858	0	Monocytes	RPS9
GSTP13	0	0.888078	0.811	0.574	0	Monocytes	GSTP1
PFDN52	0	0.88354	0.901	0.722	0	Monocytes	PFDN5
FOSL21	0	0.880584	0.515	0.205	0	Monocytes	FOSL2
DAPK1	0	0.879029	0.319	0.041	0	Monocytes	DAPK1
S100A111	0	0.866822	0.95	0.827	0	Monocytes	S100A11
FYN1	0	0.863043	0.322	0.041	0	Monocytes	FYN
THBD	0	0.862591	0.261	0.06	0	Monocytes	THBD
KCNAB2	0	0.858433	0.35	0.05	0	Monocytes	KCNAB2
OAZ12	0	0.858334	0.935	0.793	0	Monocytes	OAZ1
SULF2	0	0.855931	0.293	0.031	0	Monocytes	SULF2
AP1S22	0	0.854939	0.501	0.201	0	Monocytes	AP1S2
RPS283	0	0.853271	0.967	0.876	0	Monocytes	RPS28
FXYD52	0	0.850559	0.826	0.561	0	Monocytes	FXYD5
CARD162	0	0.849386	0.475	0.17	0	Monocytes	CARD16

RIPOR2	0	0.842885	0.269	0.047	0	Monocytes	RIPOR2
TRAPPC52	0	0.833656	0.579	0.273	0	Monocytes	TRAPPC5
VAMP52	0	0.82799	0.413	0.142	0	Monocytes	VAMP5
FTL1	0	0.817978	0.994	0.973	0	Monocytes	FTL
SLCO3A1	0	0.809563	0.258	0.024	0	Monocytes	SLCO3A1
LDLRAD3	0	0.793084	0.255	0.018	0	Monocytes	LDLRAD3
ANKRD28	0	0.790894	0.25	0.051	0	Monocytes	ANKRD28
PLP21	0	0.789362	0.386	0.119	0	Monocytes	PLP2
SERF21	0	0.789299	0.928	0.799	0	Monocytes	SERF2
MYO1G	0	0.782959	0.275	0.026	0	Monocytes	MYO1G
BCL3	0	0.781896	0.319	0.087	0	Monocytes	BCL3
RPL83	0	0.779621	0.949	0.851	0	Monocytes	RPL8
RPS133	0	0.774411	0.959	0.884	0	Monocytes	RPS13
RAC21	0	0.768762	0.408	0.137	0	Monocytes	RAC2
LILRB3	0	0.75608	0.299	0.034	0	Monocytes	LILRB3
RPL393	0	0.747758	0.965	0.88	0	Monocytes	RPL39
ICAM31	0	0.744149	0.273	0.035	0	Monocytes	ICAM3
IQGAP22	0	0.742651	0.403	0.123	0	Monocytes	IQGAP2
RPS143	0	0.735216	0.963	0.883	0	Monocytes	RPS14
RARA	0	0.731788	0.298	0.064	0	Monocytes	RARA
RPL35A3	0	0.731243	0.937	0.819	0	Monocytes	RPL35A
RPL303	0	0.717168	0.968	0.892	0	Monocytes	RPL30
AZI2	0	0.715164	0.348	0.099	0	Monocytes	AZI2
RPL323	0	0.713918	0.967	0.907	0	Monocytes	RPL32
CD93	0	0.711474	0.302	0.063	0	Monocytes	CD93
RPL343	0	0.710023	0.962	0.878	0	Monocytes	RPL34
RPS242	0	0.707493	0.961	0.9	0	Monocytes	RPS24
RPS123	0	0.705684	0.97	0.914	0	Monocytes	RPS12
RPLP12	0	0.699789	0.982	0.947	0	Monocytes	RPLP1
RPL18A3	0	0.699577	0.962	0.88	0	Monocytes	RPL18A
RPL263	0	0.689692	0.959	0.885	0	Monocytes	RPL26
RPL113	0	0.683994	0.969	0.899	0	Monocytes	RPL11
FAU3	0	0.683567	0.962	0.878	0	Monocytes	FAU
RPS153	0	0.683438	0.964	0.892	0	Monocytes	RPS15
SEPTIN9	0	0.676784	0.317	0.081	0	Monocytes	SEPTIN9
IQSEC1	0	0.675213	0.271	0.066	0	Monocytes	IQSEC1
CNN21	0	0.67212	0.297	0.036	0	Monocytes	CNN2
RPL193	0	0.663617	0.963	0.904	0	Monocytes	RPL19
BLVRA	0	0.663504	0.256	0.015	0	Monocytes	BLVRA
RPL103	0	0.628216	0.98	0.946	0	Monocytes	RPL10
MPEG1	0	0.613728	0.299	0.081	0	Monocytes	MPEG1
UBXN11	0	0.558114	0.271	0.069	0	Monocytes	UBXN11
BRI32	3.17E-305	0.88866	0.777	0.514	8.91E-301	Monocytes	BRI3
CD552	2.62E-304	0.962834	0.66	0.345	7.36E-300	Monocytes	CD55
ATP5F1E2	1.49E-303	0.792324	0.914	0.748	4.18E-299	Monocytes	ATP5F1E
PTMA3	1.30E-302	0.622252	0.964	0.905	3.67E-298	Monocytes	PTMA
UBA522	7.06E-302	0.706811	0.92	0.773	1.99E-297	Monocytes	UBA52

RPL123	1.54E-301	0.638999	0.965	0.89	4.34E-297	Monocytes	RPL12
BTG12	5.39E-301	0.909347	0.924	0.754	1.52E-296	Monocytes	BTG1
RPS15A3	9.34E-301	0.638795	0.957	0.886	2.63E-296	Monocytes	RPS15A
AHR2	1.28E-299	0.745215	0.397	0.142	3.61E-295	Monocytes	AHR
TYROBP2	1.55E-298	0.584975	0.974	0.909	4.35E-294	Monocytes	TYROBP
SNX101	2.33E-297	0.690538	0.462	0.187	6.55E-293	Monocytes	SNX10
ISG201	4.45E-296	0.642838	0.304	0.091	1.25E-291	Monocytes	ISG20
LUCAT11	2.66E-291	1.212109	0.372	0.135	7.47E-287	Monocytes	LUCAT1
TAGLN21	1.40E-290	0.952561	0.601	0.307	3.95E-286	Monocytes	TAGLN2
GSTO1	2.27E-289	0.877268	0.52	0.243	6.39E-285	Monocytes	GSTO1
PTK2B2	8.50E-283	0.833587	0.551	0.257	2.39E-278	Monocytes	PTK2B
SIPA1L13	7.52E-282	0.95998	0.84	0.556	2.12E-277	Monocytes	SIPA1L1
ALDOA2	3.44E-281	0.972245	0.714	0.449	9.69E-277	Monocytes	ALDOA
ABRACL	4.09E-281	0.627383	0.377	0.137	1.15E-276	Monocytes	ABRACL
RPL293	3.34E-279	0.629287	0.95	0.878	9.40E-275	Monocytes	RPL29
LY6E1	3.77E-279	0.999221	0.483	0.203	1.06E-274	Monocytes	LY6E
IFITM32	8.53E-276	1.309109	0.734	0.458	2.40E-271	Monocytes	IFITM3
NDRG1	1.00E-273	0.707635	0.254	0.071	2.82E-269	Monocytes	NDRG1
G0S21	5.54E-273	1.784203	0.398	0.153	1.56E-268	Monocytes	G0S2
SAP302	1.74E-270	0.943606	0.623	0.338	4.91E-266	Monocytes	SAP30
ELOB	3.58E-270	0.773853	0.715	0.436	1.01E-265	Monocytes	ELOB
AP2S12	3.62E-268	0.759098	0.621	0.337	1.02E-263	Monocytes	AP2S1
FAM107B	1.48E-267	0.866969	0.4	0.155	4.17E-263	Monocytes	FAM107B
TNFAIP2	1.00E-265	0.794103	0.408	0.162	2.83E-261	Monocytes	TNFAIP2
TES1	9.17E-264	0.514506	0.308	0.098	2.58E-259	Monocytes	TES
ASAP1	5.97E-263	0.888682	0.483	0.214	1.68E-258	Monocytes	ASAP1
STXBP2	8.65E-263	1.073928	0.62	0.361	2.43E-258	Monocytes	STXBP2
C1orf1622	7.78E-262	0.804579	0.791	0.547	2.19E-257	Monocytes	C1orf162
ADGRE5	1.76E-261	0.801431	0.366	0.133	4.96E-257	Monocytes	ADGRE5
DPYD1	1.25E-260	0.876753	0.746	0.455	3.53E-256	Monocytes	DPYD
MYL61	1.18E-257	0.714454	0.918	0.769	3.31E-253	Monocytes	MYL6
PRELID11	1.44E-256	0.79236	0.535	0.265	4.05E-252	Monocytes	PRELID1
RPS183	6.85E-256	0.610068	0.962	0.904	1.93E-251	Monocytes	RPS18
IQGAP12	3.78E-255	0.657445	0.582	0.289	1.06E-250	Monocytes	IQGAP1
RPLP22	9.23E-255	0.64222	0.94	0.84	2.60E-250	Monocytes	RPLP2
DENND5A1	7.34E-254	1.159197	0.503	0.251	2.07E-249	Monocytes	DENND5A
TNFAIP81	2.40E-253	0.741788	0.356	0.132	6.75E-249	Monocytes	TNFAIP8
CCL20	4.78E-253	1.678065	0.326	0.118	1.34E-248	Monocytes	CCL20
C19orf38	3.95E-252	0.633308	0.352	0.131	1.11E-247	Monocytes	C19orf38
LTA4H1	6.95E-251	0.772731	0.496	0.232	1.96E-246	Monocytes	LTA4H
COX4I12	1.61E-250	0.702678	0.888	0.736	4.54E-246	Monocytes	COX4I1
CFD1	2.17E-248	0.924364	0.624	0.354	6.10E-244	Monocytes	CFD
LINC01619	3.88E-244	0.917436	0.388	0.158	1.09E-239	Monocytes	LINC01619
MX2	1.15E-243	0.781509	0.378	0.148	3.23E-239	Monocytes	MX2
ATP5MC23	4.41E-242	0.667271	0.878	0.717	1.24E-237	Monocytes	ATP5MC2
PGD	6.59E-241	0.756122	0.445	0.202	1.85E-236	Monocytes	PGD
HEBP2	4.33E-238	0.585429	0.315	0.111	1.22E-233	Monocytes	HEBP2

TALDO11	2.92E-237	0.898558	0.616	0.359	8.23E-233	Monocytes	TALDO1
RPS72	1.50E-236	0.629603	0.939	0.859	4.21E-232	Monocytes	RPS7
RPL182	5.58E-236	0.573283	0.947	0.872	1.57E-231	Monocytes	RPL18
CPD	2.41E-234	0.741547	0.281	0.092	6.78E-230	Monocytes	CPD
SOCS31	1.79E-233	0.724875	0.432	0.185	5.04E-229	Monocytes	SOCS3
FBP1	2.57E-233	0.74149	0.339	0.13	7.24E-229	Monocytes	FBP1
RPL133	3.93E-233	0.543833	0.977	0.936	1.11E-228	Monocytes	RPL13
GLIPR21	3.17E-232	0.635863	0.42	0.178	8.93E-228	Monocytes	GLIPR2
HIGD2A3	2.91E-231	0.706224	0.684	0.424	8.19E-227	Monocytes	HIGD2A
PKM1	1.87E-230	0.809728	0.692	0.446	5.25E-226	Monocytes	PKM
SERP11	1.95E-227	0.692814	0.842	0.659	5.50E-223	Monocytes	SERP1
HLA-C2	3.03E-227	0.695934	0.944	0.826	8.52E-223	Monocytes	HLA-C
RPL363	1.86E-225	0.564307	0.937	0.832	5.23E-221	Monocytes	RPL36
RACK13	7.87E-222	0.570066	0.922	0.82	2.22E-217	Monocytes	RACK1
RABGEF11	3.00E-221	0.952759	0.648	0.389	8.43E-217	Monocytes	RABGEF1
ENO12	7.11E-221	0.804877	0.714	0.472	2.00E-216	Monocytes	ENO1
GMFG1	2.28E-219	0.765422	0.672	0.427	6.42E-215	Monocytes	GMFG
MYL12A1	1.97E-217	0.726443	0.76	0.536	5.54E-213	Monocytes	MYL12A
HLA-A2	3.67E-217	0.649711	0.943	0.835	1.03E-212	Monocytes	HLA-A
POLE4	4.12E-217	0.458387	0.27	0.09	1.16E-212	Monocytes	POLE4
IFITM11	6.92E-216	0.97844	0.302	0.11	1.95E-211	Monocytes	IFITM1
PFN12	1.37E-215	0.643736	0.93	0.825	3.86E-211	Monocytes	PFN1
HMGB22	1.00E-214	0.753536	0.514	0.262	2.82E-210	Monocytes	HMGB2
MNDA2	8.91E-213	0.831693	0.553	0.307	2.51E-208	Monocytes	MNDA
RPS210	5.83E-211	0.588203	0.946	0.85	1.64E-206	Monocytes	RPS2
SLC25A61	9.51E-210	0.748623	0.814	0.652	2.68E-205	Monocytes	SLC25A6
SERPINB1	3.39E-209	0.837054	0.638	0.393	9.54E-205	Monocytes	SERPINB1
COX6B11	3.24E-208	0.65112	0.767	0.542	9.11E-204	Monocytes	COX6B1
RPL71	1.09E-204	0.690198	0.802	0.611	3.07E-200	Monocytes	RPL7
MAP2K11	1.60E-203	0.809955	0.591	0.347	4.49E-199	Monocytes	MAP2K1
EFHD21	1.44E-202	0.698024	0.604	0.36	4.04E-198	Monocytes	EFHD2
VEGFA2	2.46E-200	0.663621	0.359	0.146	6.93E-196	Monocytes	VEGFA
PLIN22	1.51E-199	1.284862	0.569	0.323	4.24E-195	Monocytes	PLIN2
ZEB21	4.38E-199	0.945269	0.789	0.606	1.23E-194	Monocytes	ZEB2
AGFG1	6.35E-197	0.657673	0.416	0.192	1.79E-192	Monocytes	AGFG1
CYTIP1	7.08E-196	0.615812	0.401	0.178	1.99E-191	Monocytes	CYTIP
MYD88	1.21E-195	0.516289	0.295	0.111	3.42E-191	Monocytes	MYD88
COX8A1	1.27E-195	0.611799	0.71	0.469	3.57E-191	Monocytes	COX8A
RPL372	2.77E-195	0.508225	0.946	0.868	7.78E-191	Monocytes	RPL37
RPS82	6.86E-195	0.519645	0.968	0.914	1.93E-190	Monocytes	RPS8
RPL222	6.61E-191	0.574467	0.891	0.75	1.86E-186	Monocytes	RPL22
CLIC11	1.14E-190	0.627724	0.833	0.653	3.21E-186	Monocytes	CLIC1
PRKCB	4.80E-188	0.685395	0.475	0.236	1.35E-183	Monocytes	PRKCB
LST1	1.47E-187	0.945713	0.7	0.496	4.13E-183	Monocytes	LST1
LCP11	4.44E-187	0.69234	0.672	0.431	1.25E-182	Monocytes	LCP1
SAMSN11	1.18E-185	0.774119	0.806	0.597	3.33E-181	Monocytes	SAMSN1
CASP4	1.51E-184	0.499079	0.324	0.132	4.26E-180	Monocytes	CASP4

RPL242	3.64E-184	0.511933	0.923	0.813	1.02E-179	Monocytes	RPL24
RPS263	5.02E-182	0.6385	0.93	0.811	1.41E-177	Monocytes	RPS26
RPS272	1.14E-181	0.484457	0.954	0.876	3.20E-177	Monocytes	RPS27
ZYX2	1.97E-181	0.582538	0.682	0.439	5.54E-177	Monocytes	ZYX
HIPK21	2.97E-181	0.662684	0.393	0.182	8.35E-177	Monocytes	HIPK2
ATP5MG2	2.92E-180	0.591555	0.805	0.609	8.22E-176	Monocytes	ATP5MG
RPL7A2	5.97E-178	0.495897	0.943	0.868	1.68E-173	Monocytes	RPL7A
EEF1D2	6.90E-177	0.560953	0.856	0.69	1.94E-172	Monocytes	EEF1D
ATP2B1-AS	5.11E-176	0.802231	0.707	0.496	1.44E-171	Monocytes	ATP2B1-AS1
HBEGF2	3.38E-174	0.685536	0.546	0.293	9.52E-170	Monocytes	HBEGF
ODF3B1	8.98E-174	0.542083	0.431	0.208	2.53E-169	Monocytes	ODF3B
BCL2A12	9.43E-174	0.952122	0.731	0.5	2.65E-169	Monocytes	BCL2A1
MEGF9	1.66E-173	0.520934	0.278	0.107	4.68E-169	Monocytes	MEGF9
EIF3K2	9.84E-172	0.593265	0.766	0.574	2.77E-167	Monocytes	EIF3K
TSPO1	2.02E-171	0.777373	0.794	0.604	5.67E-167	Monocytes	TSPO
RNASEK1	2.08E-171	0.580134	0.86	0.705	5.86E-167	Monocytes	RNASEK
STK17B2	8.47E-171	0.567921	0.649	0.394	2.38E-166	Monocytes	STK17B
RPS4X3	1.87E-170	0.505119	0.931	0.853	5.27E-166	Monocytes	RPS4X
SOD22	3.12E-170	1.309149	0.74	0.577	8.77E-166	Monocytes	SOD2
LITAF2	8.54E-170	0.675376	0.674	0.44	2.40E-165	Monocytes	LITAF
RPL143	9.42E-170	0.46509	0.939	0.853	2.65E-165	Monocytes	RPL14
PSME12	2.15E-169	0.598766	0.651	0.426	6.05E-165	Monocytes	PSME1
RPS213	2.71E-169	0.523549	0.913	0.802	7.64E-165	Monocytes	RPS21
RPS32	4.21E-169	0.472265	0.953	0.875	1.18E-164	Monocytes	RPS3
PPIF	1.17E-164	1.046004	0.424	0.226	3.28E-160	Monocytes	PPIF
HLA-B1	2.14E-164	0.442459	0.984	0.932	6.02E-160	Monocytes	HLA-B
UTRN3	5.88E-164	0.610102	0.533	0.297	1.65E-159	Monocytes	UTRN
CALHM62	6.52E-163	0.582167	0.486	0.261	1.83E-158	Monocytes	CALHM6
GUK1	3.07E-162	0.610008	0.627	0.399	8.63E-158	Monocytes	GUK1
NACA2	3.92E-161	0.49538	0.926	0.833	1.10E-156	Monocytes	NACA
COX5B	1.76E-160	0.558899	0.71	0.494	4.96E-156	Monocytes	COX5B
GABARAP1	3.92E-160	0.514172	0.916	0.786	1.10E-155	Monocytes	GABARAP
UQCR111	1.10E-159	0.575271	0.706	0.488	3.11E-155	Monocytes	UQCR11
RPL62	1.62E-159	0.519418	0.931	0.856	4.57E-155	Monocytes	RPL6
WARS	8.35E-159	0.735689	0.345	0.161	2.35E-154	Monocytes	WARS
APLP2	2.98E-158	0.674545	0.683	0.471	8.38E-154	Monocytes	APLP2
ATP5F1D2	6.88E-158	0.535264	0.697	0.473	1.94E-153	Monocytes	ATP5F1D
VAMP83	1.88E-157	0.595287	0.724	0.519	5.29E-153	Monocytes	VAMP8
STK10	2.67E-157	0.626662	0.439	0.226	7.53E-153	Monocytes	STK10
CHST15	5.57E-157	0.581452	0.297	0.124	1.57E-152	Monocytes	CHST15
LRRFIP11	3.50E-156	0.587673	0.742	0.524	9.84E-152	Monocytes	LRRFIP1
AC007384.	1.48E-154	0.635658	0.389	0.189	4.15E-150	Monocytes	AC007384.1
UQCRH1	1.91E-153	0.567338	0.656	0.44	5.37E-149	Monocytes	UQCRH
ATP6V0C1	3.79E-153	0.555469	0.85	0.691	1.07E-148	Monocytes	ATP6V0C
RIPK22	3.54E-150	0.572514	0.449	0.238	9.97E-146	Monocytes	RIPK2
STMP1	1.07E-149	0.48451	0.396	0.197	3.01E-145	Monocytes	STMP1
FES	1.22E-149	0.418859	0.276	0.114	3.43E-145	Monocytes	FES

CNIH4	5.74E-149	0.471035	0.266	0.109	1.62E-144	Monocytes	CNIH4
DMXL2	2.15E-148	0.637715	0.343	0.159	6.05E-144	Monocytes	DMXL2
SLC6A6	5.00E-147	0.462067	0.254	0.099	1.41E-142	Monocytes	SLC6A6
COTL12	5.76E-147	0.745405	0.815	0.694	1.62E-142	Monocytes	COTL1
ISG151	1.66E-146	0.850701	0.37	0.18	4.66E-142	Monocytes	ISG15
DOCK51	5.04E-146	0.474912	0.373	0.177	1.42E-141	Monocytes	DOCK5
TNIP1	2.13E-144	0.489753	0.287	0.124	5.98E-140	Monocytes	TNIP1
RPS233	4.29E-144	0.403955	0.964	0.908	1.21E-139	Monocytes	RPS23
OXSR1	9.50E-144	0.580158	0.313	0.14	2.67E-139	Monocytes	OXSR1
ZNF385A1	1.17E-143	0.482718	0.382	0.191	3.30E-139	Monocytes	ZNF385A
COX7A2	4.66E-143	0.545091	0.578	0.369	1.31E-138	Monocytes	COX7A2
BLOC1S11	5.04E-143	0.548389	0.582	0.365	1.42E-138	Monocytes	BLOC1S1
TXNIP2	9.40E-143	0.910405	0.527	0.324	2.65E-138	Monocytes	TXNIP
MS4A6A3	1.06E-142	0.627758	0.726	0.536	2.99E-138	Monocytes	MS4A6A
UBE2R2	3.86E-142	0.590947	0.466	0.264	1.09E-137	Monocytes	UBE2R2
ZFAND53	9.58E-142	0.658106	0.7	0.494	2.70E-137	Monocytes	ZFAND5
C4orf31	1.46E-141	0.606896	0.521	0.312	4.11E-137	Monocytes	C4orf3
PSMA61	4.31E-141	0.541061	0.513	0.308	1.21E-136	Monocytes	PSMA6
MIF2	4.96E-140	1.119108	0.734	0.534	1.39E-135	Monocytes	MIF
CAPNS1	9.66E-140	0.549575	0.463	0.264	2.72E-135	Monocytes	CAPNS1
TNFRSF14	1.89E-139	0.449348	0.315	0.143	5.33E-135	Monocytes	TNFRSF14
PIM3	4.95E-139	0.514501	0.307	0.138	1.39E-134	Monocytes	PIM3
NOTCH2	1.04E-138	0.468618	0.288	0.124	2.91E-134	Monocytes	NOTCH2
PSMB92	1.24E-138	0.539136	0.482	0.276	3.48E-134	Monocytes	PSMB9
ARPC31	7.54E-138	0.460525	0.886	0.764	2.12E-133	Monocytes	ARPC3
RNF144B3	1.89E-137	0.609659	0.514	0.301	5.31E-133	Monocytes	RNF144B
PRKCE1	1.22E-136	0.518996	0.395	0.199	3.44E-132	Monocytes	PRKCE
ARPC1B1	3.86E-135	0.516429	0.83	0.681	1.08E-130	Monocytes	ARPC1B
PTEN1	5.26E-135	0.563177	0.507	0.299	1.48E-130	Monocytes	PTEN
COX7C1	5.70E-135	0.475782	0.77	0.594	1.60E-130	Monocytes	COX7C
PLXNC11	1.64E-134	0.448788	0.306	0.138	4.61E-130	Monocytes	PLXNC1
PTPN121	1.97E-133	0.521338	0.346	0.168	5.55E-129	Monocytes	PTPN12
FCER1G1	2.96E-132	0.491013	0.924	0.814	8.34E-128	Monocytes	FCER1G
RPL152	1.94E-131	0.433007	0.925	0.855	5.47E-127	Monocytes	RPL15
PYCARD1	2.54E-131	0.555665	0.678	0.477	7.16E-127	Monocytes	PYCARD
SPG21	2.72E-131	0.41876	0.336	0.162	7.66E-127	Monocytes	SPG21
PRDX51	4.94E-131	0.48306	0.527	0.319	1.39E-126	Monocytes	PRDX5
RPS111	5.60E-131	0.486865	0.845	0.689	1.58E-126	Monocytes	RPS11
RPS52	6.43E-131	0.451138	0.887	0.766	1.81E-126	Monocytes	RPS5
TET22	1.64E-130	0.585205	0.529	0.32	4.61E-126	Monocytes	TET2
BTF33	8.48E-130	0.494301	0.835	0.702	2.38E-125	Monocytes	BTF3
ATP5PD1	1.72E-129	0.498291	0.575	0.367	4.85E-125	Monocytes	ATP5PD
RPL312	5.57E-129	0.452468	0.825	0.665	1.57E-124	Monocytes	RPL31
RPS3A2	6.36E-128	0.447942	0.944	0.874	1.79E-123	Monocytes	RPS3A
RPL36A2	2.11E-127	0.462614	0.876	0.76	5.95E-123	Monocytes	RPL36A
ATG31	2.47E-127	0.425069	0.403	0.214	6.95E-123	Monocytes	ATG3
RPL91	4.39E-127	0.466485	0.907	0.795	1.23E-122	Monocytes	RPL9

LAMTOR41	4.11E-126	0.526146	0.701	0.505	1.16E-121	Monocytes	LAMTOR4
GNG5	4.57E-126	0.474999	0.791	0.617	1.29E-121	Monocytes	GNG5
GSTK11	1.83E-125	0.500742	0.571	0.375	5.15E-121	Monocytes	GSTK1
RPS161	4.56E-125	0.441603	0.923	0.827	1.28E-120	Monocytes	RPS16
PGS1	4.64E-125	0.489905	0.266	0.116	1.31E-120	Monocytes	PGS1
RPL172	2.52E-123	0.410141	0.923	0.833	7.09E-119	Monocytes	RPL17
HPCAL1	5.52E-123	0.522681	0.284	0.129	1.55E-118	Monocytes	HPCAL1
HCST1	2.95E-122	0.530113	0.726	0.539	8.31E-118	Monocytes	HCST
TMEM167/	3.93E-122	0.452293	0.34	0.17	1.11E-117	Monocytes	TMEM167A
PGAM11	7.61E-122	0.509879	0.464	0.278	2.14E-117	Monocytes	PGAM1
CUX11	1.39E-121	0.548966	0.592	0.393	3.90E-117	Monocytes	CUX1
FCGR2B2	1.55E-121	0.550841	0.299	0.138	4.35E-117	Monocytes	FCGR2B
YBX12	1.23E-120	0.490615	0.805	0.663	3.47E-116	Monocytes	YBX1
RPS171	5.22E-120	0.533416	0.646	0.461	1.47E-115	Monocytes	RPS17
RILPL22	8.69E-120	0.54483	0.741	0.546	2.45E-115	Monocytes	RILPL2
MAPK6	1.04E-119	0.468721	0.264	0.118	2.92E-115	Monocytes	MAPK6
GPSM31	8.63E-119	0.498887	0.626	0.427	2.43E-114	Monocytes	GPSM3
SEC61G	1.07E-118	0.604467	0.455	0.27	3.02E-114	Monocytes	SEC61G
BNIP3L	1.43E-118	0.603617	0.531	0.339	4.04E-114	Monocytes	BNIP3L
TRPS11	7.99E-118	0.538752	0.427	0.238	2.25E-113	Monocytes	TRPS1
OGDH1	1.77E-117	0.433954	0.291	0.136	4.97E-113	Monocytes	OGDH
RPL272	2.65E-117	0.444716	0.842	0.707	7.46E-113	Monocytes	RPL27
ARPC51	8.54E-117	0.491573	0.661	0.473	2.40E-112	Monocytes	ARPC5
LRRK21	1.07E-116	0.51447	0.387	0.206	3.02E-112	Monocytes	LRRK2
FNBP11	1.94E-116	0.444953	0.427	0.239	5.45E-112	Monocytes	FNBP1
CHCHD21	2.14E-116	0.445285	0.746	0.57	6.01E-112	Monocytes	CHCHD2
RPL353	1.25E-115	0.404076	0.902	0.796	3.51E-111	Monocytes	RPL35
GAS7	7.98E-115	0.493679	0.341	0.173	2.24E-110	Monocytes	GAS7
RYBP	1.46E-114	0.466553	0.251	0.111	4.12E-110	Monocytes	RYBP
FNDC3A1	2.59E-114	0.709469	0.532	0.344	7.29E-110	Monocytes	FNDC3A
ARPC21	2.87E-114	0.439104	0.789	0.634	8.06E-110	Monocytes	ARPC2
TMEM258:	4.03E-114	0.497308	0.572	0.375	1.13E-109	Monocytes	TMEM258
FKBP1A1	8.18E-114	0.464281	0.475	0.289	2.30E-109	Monocytes	FKBP1A
TNFAIP34	8.56E-113	0.681983	0.723	0.547	2.41E-108	Monocytes	TNFAIP3
EVI2B2	1.12E-112	0.49605	0.599	0.407	3.14E-108	Monocytes	EVI2B
ATP5MC31	8.80E-112	0.443077	0.621	0.433	2.48E-107	Monocytes	ATP5MC3
C7orf501	6.99E-111	0.371586	0.302	0.146	1.97E-106	Monocytes	C7orf50
ATP1B33	2.96E-110	0.533458	0.685	0.494	8.32E-106	Monocytes	ATP1B3
RPL23A2	9.81E-110	0.408685	0.893	0.777	2.76E-105	Monocytes	RPL23A
PGLS1	3.73E-108	0.45533	0.569	0.377	1.05E-103	Monocytes	PGLS
CALM3	2.02E-107	0.419147	0.373	0.203	5.68E-103	Monocytes	CALM3
RALA1	1.04E-106	0.415477	0.253	0.115	2.94E-102	Monocytes	RALA
UBAP11	3.88E-106	0.539983	0.454	0.273	1.09E-101	Monocytes	UBAP1
NDUFA11	2.97E-105	0.414791	0.634	0.439	8.35E-101	Monocytes	NDUFA11
RPL411	3.74E-105	0.71631	0.927	0.816	1.05E-100	Monocytes	RPL41
PDE4A2	3.76E-105	0.536405	0.416	0.241	1.06E-100	Monocytes	PDE4A
FOXO31	7.05E-104	0.601032	0.565	0.38	1.98E-99	Monocytes	FOXO3

SHKBP1	7.96E-104	0.406562	0.325	0.169	2.24E-99	Monocytes	SHKBP1
TCIRG1	1.10E-103	0.485392	0.48	0.3	3.11E-99	Monocytes	TCIRG1
UBALD21	1.50E-103	0.467938	0.453	0.273	4.22E-99	Monocytes	UBALD2
RPS192	2.26E-102	0.335175	0.964	0.921	6.36E-98	Monocytes	RPS19
POR	3.78E-102	0.392315	0.264	0.125	1.06E-97	Monocytes	POR
AIF11	7.39E-102	0.4705	0.877	0.782	2.08E-97	Monocytes	AIF1
ATP6V0D1	3.00E-101	0.485341	0.518	0.338	8.45E-97	Monocytes	ATP6V0D1
TNFSF13B	4.02E-101	0.455576	0.426	0.249	1.13E-96	Monocytes	TNFSF13B
RPLP02	1.01E-100	0.364341	0.932	0.867	2.84E-96	Monocytes	RPLP0
GNAI21	1.50E-100	0.409165	0.814	0.668	4.23E-96	Monocytes	GNAI2
PSMB31	2.08E-100	0.435503	0.535	0.352	5.86E-96	Monocytes	PSMB3
EEF1A12	3.37E-100	0.36005	0.982	0.964	9.48E-96	Monocytes	EEF1A1
COX7B	4.09E-100	0.43141	0.59	0.408	1.15E-95	Monocytes	COX7B
VASP	4.15E-100	0.463074	0.407	0.241	1.17E-95	Monocytes	VASP
SLC2A31	1.00E-99	0.638123	0.581	0.38	2.82E-95	Monocytes	SLC2A3
STX10	1.68E-99	0.376369	0.298	0.15	4.72E-95	Monocytes	STX10
SSR41	1.72E-99	0.446771	0.668	0.491	4.85E-95	Monocytes	SSR4
IL101	4.21E-99	0.794554	0.277	0.137	1.19E-94	Monocytes	IL10
RPS253	5.70E-98	0.328122	0.93	0.847	1.60E-93	Monocytes	RPS25
GRINA	1.15E-97	0.445442	0.602	0.423	3.24E-93	Monocytes	GRINA
SIK31	1.32E-97	0.598383	0.594	0.418	3.71E-93	Monocytes	SIK3
SLC7A7	1.61E-97	0.443622	0.433	0.266	4.52E-93	Monocytes	SLC7A7
EIF2AK31	1.95E-97	0.552873	0.327	0.173	5.48E-93	Monocytes	EIF2AK3
PSME22	4.04E-97	0.453766	0.524	0.346	1.14E-92	Monocytes	PSME2
UQCRC1	5.29E-97	0.376801	0.411	0.242	1.49E-92	Monocytes	UQCRC1
POLR1D	2.71E-96	0.391863	0.427	0.253	7.61E-92	Monocytes	POLR1D
RNH1	3.02E-96	0.427366	0.511	0.335	8.49E-92	Monocytes	RNH1
ATP6V1G1	3.87E-96	0.416702	0.589	0.413	1.09E-91	Monocytes	ATP6V1G1
MBD2	8.48E-96	0.434214	0.299	0.155	2.39E-91	Monocytes	MBD2
APRT2	1.48E-95	0.406966	0.545	0.366	4.17E-91	Monocytes	APRT
PECAM1	4.06E-95	0.483886	0.326	0.176	1.14E-90	Monocytes	PECAM1
TPT12	8.92E-95	0.332754	0.978	0.955	2.51E-90	Monocytes	TPT1
GPI	2.42E-94	0.553953	0.402	0.241	6.82E-90	Monocytes	GPI
NDUFA14	5.54E-94	0.422744	0.593	0.412	1.56E-89	Monocytes	NDUFA1
TRAPPC1	1.25E-93	0.389317	0.47	0.292	3.51E-89	Monocytes	TRAPPC1
PRR131	1.28E-93	0.408048	0.63	0.45	3.61E-89	Monocytes	PRR13
ERP292	1.49E-93	0.391254	0.587	0.403	4.20E-89	Monocytes	ERP29
GCH1	3.71E-93	0.518759	0.339	0.188	1.04E-88	Monocytes	GCH1
MPHOSPH(	7.83E-93	0.382858	0.256	0.124	2.20E-88	Monocytes	MPHOSPH6
POLR2L	1.65E-92	0.419953	0.455	0.286	4.63E-88	Monocytes	POLR2L
SLC36A4	1.69E-92	0.359675	0.256	0.124	4.76E-88	Monocytes	SLC36A4
TOM1	2.09E-92	0.468638	0.642	0.461	5.88E-88	Monocytes	TOM1
FAM49A1	1.29E-91	0.444423	0.504	0.326	3.64E-87	Monocytes	FAM49A
UBE2D1	1.65E-91	0.430379	0.322	0.174	4.63E-87	Monocytes	UBE2D1
CRTAP1	2.96E-91	0.407131	0.496	0.325	8.34E-87	Monocytes	CRTAP
LILRA2	3.12E-91	0.383107	0.277	0.141	8.78E-87	Monocytes	LILRA2
RPL37A2	5.64E-91	0.379107	0.879	0.778	1.59E-86	Monocytes	RPL37A

ATP5MPL1	1.95E-90	0.411126	0.475	0.306	5.49E-86	Monocytes	ATP5MPL
SNRPD21	2.03E-90	0.387204	0.6	0.423	5.73E-86	Monocytes	SNRPD2
CSTB1	5.17E-90	0.676451	0.586	0.416	1.46E-85	Monocytes	CSTB
PTPRE2	4.35E-89	0.417729	0.68	0.502	1.22E-84	Monocytes	PTPRE
H2AFY1	5.15E-89	0.414737	0.676	0.513	1.45E-84	Monocytes	H2AFY
COX5A2	8.16E-88	0.381494	0.586	0.411	2.30E-83	Monocytes	COX5A
DENND1A1	1.00E-87	0.38627	0.383	0.221	2.81E-83	Monocytes	DENND1A
CASP1	1.03E-87	0.358733	0.413	0.248	2.88E-83	Monocytes	CASP1
PSMA7	3.86E-87	0.426683	0.638	0.479	1.09E-82	Monocytes	PSMA7
HCK	4.09E-87	0.533452	0.494	0.339	1.15E-82	Monocytes	HCK
HMGN2	7.90E-87	0.362714	0.659	0.486	2.22E-82	Monocytes	HMGN2
OST4	3.23E-86	0.376159	0.654	0.472	9.09E-82	Monocytes	OST4
RPL13A2	4.15E-86	0.381193	0.862	0.742	1.17E-81	Monocytes	RPL13A
RNF181	5.98E-86	0.402367	0.435	0.269	1.68E-81	Monocytes	RNF181
SNHG292	1.39E-85	0.432388	0.694	0.547	3.91E-81	Monocytes	SNHG29
AURKAIP1	1.75E-85	0.367013	0.413	0.252	4.94E-81	Monocytes	AURKAIP1
NDUFA12	1.79E-85	0.340358	0.357	0.204	5.03E-81	Monocytes	NDUFA12
DCTN3	3.04E-85	0.280325	0.285	0.148	8.54E-81	Monocytes	DCTN3
ANAPC11	3.33E-85	0.352429	0.493	0.319	9.38E-81	Monocytes	ANAPC11
RPL10A2	8.58E-85	0.383255	0.863	0.762	2.41E-80	Monocytes	RPL10A
NDUFB11	9.14E-85	0.365024	0.589	0.411	2.57E-80	Monocytes	NDUFB11
COMMD61	1.34E-84	0.366423	0.726	0.55	3.77E-80	Monocytes	COMMD6
ATP5MD	1.45E-84	0.404119	0.56	0.391	4.08E-80	Monocytes	ATP5MD
GNS	7.53E-84	0.378853	0.351	0.201	2.12E-79	Monocytes	GNS
RPL36AL2	1.41E-83	0.374216	0.803	0.656	3.97E-79	Monocytes	RPL36AL
MYO9B1	1.69E-83	0.490897	0.643	0.481	4.75E-79	Monocytes	MYO9B
UQCRB	1.77E-83	0.35892	0.716	0.552	4.98E-79	Monocytes	UQCRB
CSNK2B	3.17E-83	0.388096	0.606	0.438	8.93E-79	Monocytes	CSNK2B
AL138720.:	5.83E-83	0.450276	0.313	0.173	1.64E-78	Monocytes	AL138720.1
RPS27A2	6.06E-83	0.312909	0.948	0.882	1.71E-78	Monocytes	RPS27A
LDHA1	1.76E-82	0.607934	0.613	0.449	4.95E-78	Monocytes	LDHA
SELL	2.07E-82	0.500145	0.31	0.173	5.83E-78	Monocytes	SELL
SLC11A12	2.26E-82	0.412844	0.814	0.63	6.37E-78	Monocytes	SLC11A1
NDUFB91	3.35E-82	0.369581	0.486	0.319	9.42E-78	Monocytes	NDUFB9
RPL231	3.65E-82	0.397083	0.702	0.542	1.03E-77	Monocytes	RPL23
SMCHD11	4.66E-82	0.472825	0.436	0.274	1.31E-77	Monocytes	SMCHD1
CYTH11	1.04E-81	0.379659	0.46	0.292	2.93E-77	Monocytes	CYTH1
HLA-F	1.61E-81	0.381156	0.419	0.258	4.54E-77	Monocytes	HLA-F
SIGIRR	1.68E-81	0.294694	0.254	0.127	4.72E-77	Monocytes	SIGIRR
SUPT4H1	1.91E-81	0.350628	0.396	0.24	5.37E-77	Monocytes	SUPT4H1
NDUFB21	2.02E-81	0.333205	0.598	0.419	5.69E-77	Monocytes	NDUFB2
CHPT12	4.63E-81	0.403401	0.349	0.201	1.30E-76	Monocytes	CHPT1
CCNI1	1.19E-80	0.421523	0.729	0.584	3.36E-76	Monocytes	CCNI
GRN2	7.66E-80	0.439208	0.794	0.665	2.16E-75	Monocytes	GRN
DUSP23	1.44E-79	0.606216	0.554	0.394	4.05E-75	Monocytes	DUSP2
ITGA5	4.13E-79	0.442004	0.307	0.171	1.16E-74	Monocytes	ITGA5
PNPLA8	4.42E-79	0.458672	0.444	0.285	1.24E-74	Monocytes	PNPLA8

VMP11	5.55E-79	0.464485	0.609	0.449	1.56E-74	Monocytes	VMP1
ERN1	6.86E-79	0.430231	0.311	0.175	1.93E-74	Monocytes	ERN1
NUMB1	8.69E-79	0.534449	0.526	0.368	2.44E-74	Monocytes	NUMB
NDUFS51	1.23E-78	0.361433	0.554	0.384	3.47E-74	Monocytes	NDUFS5
CHMP1B1	1.35E-78	0.55542	0.42	0.268	3.80E-74	Monocytes	CHMP1B
TMEM123:	2.05E-78	0.411016	0.414	0.262	5.76E-74	Monocytes	TMEM123
PSMB101	2.44E-78	0.343981	0.481	0.318	6.87E-74	Monocytes	PSMB10
PCBP1	2.62E-78	0.426979	0.566	0.405	7.38E-74	Monocytes	PCBP1
ATP5PO	2.72E-78	0.34295	0.586	0.42	7.65E-74	Monocytes	ATP5PO
RAB5IF	4.88E-78	0.389277	0.361	0.217	1.37E-73	Monocytes	RAB5IF
RNF1491	9.65E-78	0.400951	0.732	0.559	2.72E-73	Monocytes	RNF149
NOP101	1.11E-77	0.388497	0.487	0.324	3.13E-73	Monocytes	NOP10
ATP6V1F	1.75E-77	0.417558	0.671	0.516	4.92E-73	Monocytes	ATP6V1F
PRKAG21	1.67E-76	0.431208	0.521	0.353	4.69E-72	Monocytes	PRKAG2
TLE51	1.74E-76	0.305032	0.347	0.2	4.90E-72	Monocytes	TLE5
MGAT11	2.36E-76	0.42159	0.654	0.494	6.65E-72	Monocytes	MGAT1
TIMM8B	4.33E-76	0.257905	0.254	0.129	1.22E-71	Monocytes	TIMM8B
MRPL33	4.48E-76	0.29878	0.319	0.181	1.26E-71	Monocytes	MRPL33
WDR83OS	4.51E-76	0.35008	0.614	0.447	1.27E-71	Monocytes	WDR83OS
ITGB21	1.30E-75	0.351523	0.836	0.737	3.67E-71	Monocytes	ITGB2
PPP1CB2	2.03E-75	0.365205	0.397	0.245	5.72E-71	Monocytes	PPP1CB
RPL382	5.77E-75	0.344207	0.776	0.643	1.62E-70	Monocytes	RPL38
MRPS24	1.60E-74	0.332742	0.324	0.186	4.51E-70	Monocytes	MRPS24
TPI1	1.94E-74	0.449705	0.759	0.608	5.45E-70	Monocytes	TPI1
EDF12	2.69E-74	0.334311	0.643	0.479	7.58E-70	Monocytes	EDF1
NAA381	3.01E-74	0.329773	0.39	0.239	8.48E-70	Monocytes	NAA38
TBCA	3.44E-74	0.338704	0.5	0.34	9.69E-70	Monocytes	TBCA
NDUFA2	5.65E-74	0.333198	0.437	0.28	1.59E-69	Monocytes	NDUFA2
GNB2	1.04E-73	0.388702	0.607	0.455	2.92E-69	Monocytes	GNB2
IFI62	1.76E-73	0.559706	0.439	0.278	4.96E-69	Monocytes	IFI6
SUB11	1.98E-73	0.358151	0.599	0.446	5.56E-69	Monocytes	SUB1
CIB12	6.64E-73	0.356145	0.48	0.323	1.87E-68	Monocytes	CIB1
ARHGDIB2	8.74E-73	0.375239	0.815	0.698	2.46E-68	Monocytes	ARHGDIB
LACTB	1.33E-72	0.283607	0.302	0.168	3.73E-68	Monocytes	LACTB
UBL51	1.37E-72	0.361429	0.623	0.463	3.87E-68	Monocytes	UBL5
MT2A1	5.25E-72	0.338436	0.56	0.383	1.48E-67	Monocytes	MT2A
C9orf162	5.32E-72	0.308403	0.501	0.338	1.50E-67	Monocytes	C9orf16
CXCL83	1.65E-71	0.70959	0.641	0.501	4.64E-67	Monocytes	CXCL8
AGO2	1.67E-71	0.354251	0.298	0.167	4.69E-67	Monocytes	AGO2
C5AR12	4.48E-71	0.477671	0.677	0.516	1.26E-66	Monocytes	C5AR1
TMA7	7.27E-71	0.355427	0.696	0.549	2.04E-66	Monocytes	TMA7
EIF11	1.37E-70	0.323693	0.965	0.923	3.86E-66	Monocytes	EIF1
PRMT91	1.61E-70	0.336241	0.286	0.158	4.54E-66	Monocytes	PRMT9
AGTPBP1	2.12E-70	0.322772	0.307	0.174	5.98E-66	Monocytes	AGTPBP1
ARF51	3.49E-70	0.323986	0.529	0.372	9.82E-66	Monocytes	ARF5
ATP5MF1	3.50E-70	0.342141	0.576	0.419	9.85E-66	Monocytes	ATP5MF
HSBP11	4.38E-70	0.380378	0.427	0.282	1.23E-65	Monocytes	HSBP1

GCA	5.49E-70	0.46167	0.382	0.244	1.54E-65	Monocytes	GCA
NEDD81	7.89E-70	0.351054	0.513	0.359	2.22E-65	Monocytes	NEDD8
NFIL31	1.04E-69	0.36989	0.481	0.318	2.94E-65	Monocytes	NFIL3
PCNX1	2.19E-69	0.422898	0.296	0.168	6.17E-65	Monocytes	PCNX1
ZNHIT1	2.28E-69	0.29261	0.335	0.198	6.41E-65	Monocytes	ZNHIT1
EIF3H2	4.06E-69	0.337726	0.62	0.471	1.14E-64	Monocytes	EIF3H
PSTPIP21	6.14E-69	0.290522	0.26	0.139	1.73E-64	Monocytes	PSTPIP2
TAF102	1.33E-68	0.361426	0.605	0.456	3.74E-64	Monocytes	TAF10
SF3B5	1.86E-68	0.369538	0.482	0.333	5.24E-64	Monocytes	SF3B5
ARL8B1	2.08E-68	0.430243	0.476	0.328	5.85E-64	Monocytes	ARL8B
NME22	2.59E-68	0.311909	0.804	0.663	7.29E-64	Monocytes	NME2
EIF4EBP1	2.79E-68	0.285658	0.283	0.158	7.86E-64	Monocytes	EIF4EBP1
SQOR	2.81E-68	0.345571	0.309	0.18	7.89E-64	Monocytes	SQOR
EIF3G2	3.49E-68	0.325261	0.623	0.468	9.82E-64	Monocytes	EIF3G
ZFAS1	3.66E-68	0.342068	0.744	0.6	1.03E-63	Monocytes	ZFAS1
AMPD2	6.99E-68	0.296303	0.301	0.171	1.97E-63	Monocytes	AMPD2
ALKBH71	1.09E-67	0.348962	0.474	0.322	3.07E-63	Monocytes	ALKBH7
ETHE1	1.39E-67	0.319032	0.267	0.148	3.90E-63	Monocytes	ETHE1
SMDT12	2.22E-67	0.326777	0.512	0.356	6.26E-63	Monocytes	SMDT1
DDT	2.60E-67	0.33518	0.275	0.155	7.32E-63	Monocytes	DDT
ANXA51	3.08E-67	0.345192	0.759	0.63	8.67E-63	Monocytes	ANXA5
ACTN4	5.05E-67	0.265372	0.268	0.146	1.42E-62	Monocytes	ACTN4
TAOK33	1.02E-66	0.381556	0.601	0.448	2.86E-62	Monocytes	TAOK3
STX113	3.25E-66	0.340711	0.524	0.363	9.14E-62	Monocytes	STX11
SLC39A11	6.11E-66	0.318141	0.267	0.147	1.72E-61	Monocytes	SLC39A11
BAZ1A1	8.05E-66	0.385706	0.419	0.275	2.27E-61	Monocytes	BAZ1A
NDUFA13	1.41E-65	0.327693	0.639	0.487	3.96E-61	Monocytes	NDUFA13
PGK1	1.86E-65	0.454941	0.642	0.51	5.23E-61	Monocytes	PGK1
SEC61B	2.81E-65	0.370886	0.617	0.468	7.92E-61	Monocytes	SEC61B
ECH1	3.80E-65	0.305035	0.341	0.209	1.07E-60	Monocytes	ECH1
NDUFA7	9.31E-65	0.278177	0.314	0.185	2.62E-60	Monocytes	NDUFA7
EEF1G3	1.30E-64	0.327736	0.82	0.691	3.66E-60	Monocytes	EEF1G
SAT13	1.45E-64	0.303253	0.991	0.97	4.07E-60	Monocytes	SAT1
GK2	4.20E-64	0.370294	0.515	0.356	1.18E-59	Monocytes	GK
CREB52	6.51E-64	0.367595	0.259	0.142	1.83E-59	Monocytes	CREB5
EIF4E2	6.88E-64	0.260431	0.253	0.137	1.94E-59	Monocytes	EIF4E2
ABHD5	7.36E-64	0.474282	0.277	0.158	2.07E-59	Monocytes	ABHD5
LIMD21	7.86E-64	0.394349	0.476	0.332	2.21E-59	Monocytes	LIMD2
ANP32B2	1.01E-63	0.315153	0.528	0.374	2.83E-59	Monocytes	ANP32B
CAST1	1.16E-63	0.287404	0.552	0.394	3.26E-59	Monocytes	CAST
H2AFV1	1.26E-63	0.257503	0.441	0.293	3.54E-59	Monocytes	H2AFV
TGIF12	1.72E-62	0.345936	0.392	0.253	4.83E-58	Monocytes	TGIF1
NDUFB71	3.15E-62	0.302959	0.407	0.265	8.87E-58	Monocytes	NDUFB7
CIAO2B	1.06E-61	0.301418	0.429	0.284	2.98E-57	Monocytes	CIAO2B
PSMB8	1.37E-61	0.309407	0.436	0.295	3.85E-57	Monocytes	PSMB8
RPL43	1.85E-61	0.313	0.835	0.736	5.21E-57	Monocytes	RPL4
SPI11	2.30E-61	0.334944	0.798	0.683	6.46E-57	Monocytes	SPI1

PTP4A2	2.67E-61	0.31641	0.452	0.314	7.50E-57	Monocytes	PTP4A2
CLIC4	4.35E-61	0.461547	0.304	0.184	1.22E-56	Monocytes	CLIC4
SHISA5	4.56E-61	0.317424	0.299	0.177	1.28E-56	Monocytes	SHISA5
RPL27A2	5.49E-61	0.321254	0.71	0.579	1.54E-56	Monocytes	RPL27A
OSBPL82	5.69E-61	0.421666	0.497	0.35	1.60E-56	Monocytes	OSBPL8
RASSF3	5.89E-61	0.319242	0.281	0.161	1.66E-56	Monocytes	RASSF3
RPS292	8.55E-61	0.343083	0.775	0.65	2.41E-56	Monocytes	RPS29
IRF7	1.86E-60	0.367255	0.344	0.216	5.22E-56	Monocytes	IRF7
IFNAR2	1.24E-59	0.26464	0.254	0.141	3.50E-55	Monocytes	IFNAR2
ADGRE2	1.70E-59	0.350044	0.344	0.214	4.79E-55	Monocytes	ADGRE2
TOMM71	1.74E-59	0.293193	0.699	0.557	4.89E-55	Monocytes	TOMM7
UXT1	1.78E-59	0.295228	0.467	0.323	5.02E-55	Monocytes	UXT
TMEM205	2.64E-59	0.28546	0.288	0.169	7.42E-55	Monocytes	TMEM205
RPSA2	2.72E-59	0.296621	0.829	0.716	7.65E-55	Monocytes	RPSA
TNFRSF1B	3.29E-59	0.365248	0.669	0.518	9.26E-55	Monocytes	TNFRSF1B
NDUFB101	4.36E-59	0.268489	0.434	0.29	1.23E-54	Monocytes	NDUFB10
SSR22	5.23E-59	0.351877	0.535	0.394	1.47E-54	Monocytes	SSR2
COPE1	5.27E-59	0.327977	0.564	0.429	1.48E-54	Monocytes	COPE
MYL12B1	1.34E-58	0.311008	0.69	0.555	3.77E-54	Monocytes	MYL12B
CKLF2	1.96E-58	0.362902	0.517	0.373	5.52E-54	Monocytes	CKLF
P4HB	2.92E-58	0.490486	0.473	0.336	8.22E-54	Monocytes	P4HB
NDUFS71	3.06E-58	0.274704	0.471	0.329	8.61E-54	Monocytes	NDUFS7
RNF7	1.11E-57	0.294144	0.429	0.29	3.11E-53	Monocytes	RNF7
UQCRQ1	2.64E-57	0.300923	0.486	0.34	7.43E-53	Monocytes	UQCRQ
P2RY8	4.84E-57	0.31128	0.288	0.17	1.36E-52	Monocytes	P2RY8
JTB	7.19E-57	0.293557	0.445	0.308	2.02E-52	Monocytes	JTB
SCAND1	2.36E-56	0.324062	0.454	0.316	6.63E-52	Monocytes	SCAND1
SRA1	3.34E-56	0.313852	0.372	0.243	9.41E-52	Monocytes	SRA1
SPAG91	3.92E-56	0.445926	0.518	0.38	1.10E-51	Monocytes	SPAG9
IGBP1	4.12E-56	0.282707	0.333	0.208	1.16E-51	Monocytes	IGBP1
FGL22	1.32E-55	0.325991	0.561	0.419	3.73E-51	Monocytes	FGL2
NDUFA6	1.62E-55	0.261174	0.38	0.247	4.57E-51	Monocytes	NDUFA6
EIF3M	4.31E-55	0.281497	0.464	0.325	1.21E-50	Monocytes	EIF3M
MMP24OS	5.59E-55	0.26149	0.365	0.235	1.57E-50	Monocytes	MMP24OS
ATP13A32	5.69E-55	0.43765	0.459	0.326	1.60E-50	Monocytes	ATP13A3
C4orf481	6.57E-55	0.282105	0.407	0.271	1.85E-50	Monocytes	C4orf48
FKBP81	1.26E-54	0.300113	0.515	0.375	3.54E-50	Monocytes	FKBP8
CD37	1.29E-54	0.314396	0.78	0.657	3.62E-50	Monocytes	CD37
ACTG1	1.72E-54	0.319542	0.872	0.789	4.84E-50	Monocytes	ACTG1
IL10RB	1.82E-54	0.262267	0.289	0.174	5.13E-50	Monocytes	IL10RB
GLRX	2.17E-54	0.337839	0.501	0.357	6.11E-50	Monocytes	GLRX
MECP2	2.38E-54	0.335934	0.301	0.184	6.69E-50	Monocytes	MECP2
SLC25A52	3.18E-54	0.315374	0.67	0.549	8.96E-50	Monocytes	SLC25A5
EIF3E1	3.90E-54	0.294353	0.568	0.432	1.10E-49	Monocytes	EIF3E
ADIPOR1	5.82E-54	0.28342	0.398	0.268	1.64E-49	Monocytes	ADIPOR1
RBIS	8.96E-54	0.279832	0.413	0.276	2.52E-49	Monocytes	RBIS
MTPN	9.34E-54	0.328147	0.46	0.329	2.63E-49	Monocytes	MTPN

TXNL4A	1.23E-53	0.264658	0.41	0.275	3.45E-49	Monocytes	TXNL4A
NDUFB41	1.30E-53	0.254674	0.485	0.344	3.67E-49	Monocytes	NDUFB4
IL1RN1	1.45E-53	0.323706	0.281	0.164	4.07E-49	Monocytes	IL1RN
FAAP20	2.02E-53	0.280271	0.363	0.235	5.69E-49	Monocytes	FAAP20
FBXL5	4.14E-53	0.359449	0.316	0.2	1.16E-48	Monocytes	FBXL5
ETFB	9.14E-53	0.256055	0.3	0.184	2.57E-48	Monocytes	ETFB
CALCOCO2	1.27E-52	0.267343	0.365	0.236	3.58E-48	Monocytes	CALCOCO2
ARL4A	1.60E-52	0.374462	0.364	0.242	4.51E-48	Monocytes	ARL4A
MAP2K2	1.93E-52	0.269225	0.364	0.238	5.42E-48	Monocytes	MAP2K2
TRIR	2.43E-52	0.307398	0.59	0.457	6.83E-48	Monocytes	TRIR
MYO1F1	2.77E-52	0.346633	0.514	0.377	7.80E-48	Monocytes	MYO1F
COX6C1	4.16E-52	0.288573	0.593	0.457	1.17E-47	Monocytes	COX6C
GPCPD11	5.82E-52	0.429404	0.519	0.389	1.64E-47	Monocytes	GPCPD1
CHMP2A	1.39E-51	0.278327	0.339	0.218	3.91E-47	Monocytes	CHMP2A
BCAT1	2.05E-51	0.276741	0.269	0.159	5.77E-47	Monocytes	BCAT1
RAB7A1	6.30E-51	0.340816	0.731	0.603	1.77E-46	Monocytes	RAB7A
CD681	1.55E-50	0.315022	0.813	0.703	4.37E-46	Monocytes	CD68
RPL21	1.88E-50	0.325426	0.868	0.743	5.28E-46	Monocytes	RPL21
RABAC1	4.08E-50	0.300914	0.489	0.353	1.15E-45	Monocytes	RABAC1
EPSTI1	6.81E-50	0.356538	0.317	0.202	1.92E-45	Monocytes	EPSTI1
COX14	8.05E-50	0.263562	0.289	0.178	2.27E-45	Monocytes	COX14
PABPC11	1.14E-49	0.292569	0.908	0.842	3.22E-45	Monocytes	PABPC1
NUDT161	3.44E-49	0.28846	0.367	0.247	9.67E-45	Monocytes	NUDT16
CFLAR1	4.50E-49	0.308763	0.609	0.46	1.27E-44	Monocytes	CFLAR
LPCAT1	2.64E-48	0.346406	0.281	0.176	7.43E-44	Monocytes	LPCAT1
BID	5.83E-48	0.348227	0.356	0.241	1.64E-43	Monocytes	BID
DNAJC151	6.65E-48	0.258698	0.43	0.303	1.87E-43	Monocytes	DNAJC15
USP3	1.27E-47	0.294917	0.377	0.254	3.59E-43	Monocytes	USP3
ACSL31	1.67E-47	0.434808	0.375	0.258	4.70E-43	Monocytes	ACSL3
DDX60L	4.35E-47	0.315365	0.277	0.172	1.22E-42	Monocytes	DDX60L
SLC43A2	7.92E-47	0.329177	0.452	0.328	2.23E-42	Monocytes	SLC43A2
OSTC	1.08E-46	0.297801	0.354	0.237	3.04E-42	Monocytes	OSTC
SMIM26	1.32E-46	0.255116	0.389	0.265	3.70E-42	Monocytes	SMIM26
GPR1323	5.37E-46	0.334998	0.403	0.282	1.51E-41	Monocytes	GPR132
GABARAPL	9.86E-46	0.292973	0.444	0.314	2.77E-41	Monocytes	GABARAPL1
FMN11	1.11E-45	0.316841	0.53	0.403	3.12E-41	Monocytes	FMN1
PILRA	1.38E-45	0.292243	0.556	0.426	3.89E-41	Monocytes	PILRA
COX7A2L	5.10E-45	0.263274	0.388	0.27	1.44E-40	Monocytes	COX7A2L
NOP531	5.91E-45	0.265358	0.555	0.428	1.66E-40	Monocytes	NOP53
UBE2L6	9.99E-45	0.288079	0.371	0.255	2.81E-40	Monocytes	UBE2L6
RSL1D1	1.17E-44	0.251186	0.44	0.316	3.28E-40	Monocytes	RSL1D1
AKIRIN21	1.19E-44	0.30858	0.426	0.302	3.35E-40	Monocytes	AKIRIN2
ZC3H12A	1.27E-44	0.342115	0.252	0.155	3.57E-40	Monocytes	ZC3H12A
IRF1	1.31E-44	0.336587	0.263	0.162	3.68E-40	Monocytes	IRF1
EIF3F2	1.35E-44	0.254549	0.627	0.503	3.79E-40	Monocytes	EIF3F
NFKBIA2	2.06E-44	0.449514	0.892	0.83	5.80E-40	Monocytes	NFKBIA
TMEM256	5.12E-44	0.255941	0.354	0.241	1.44E-39	Monocytes	TMEM256

NLRP33	2.65E-43	0.339506	0.645	0.517	7.47E-39	Monocytes	NLRP3
BASP13	4.28E-43	0.345289	0.675	0.56	1.20E-38	Monocytes	BASP1
FMNL1	4.56E-43	0.269511	0.48	0.352	1.28E-38	Monocytes	FMNL1
NAAA	5.10E-43	0.296607	0.263	0.166	1.43E-38	Monocytes	NAAA
STK24	1.84E-42	0.253149	0.356	0.241	5.18E-38	Monocytes	STK24
PITPNA	2.64E-42	0.307147	0.302	0.198	7.42E-38	Monocytes	PITPNA
CMIP1	3.64E-42	0.253954	0.475	0.349	1.03E-37	Monocytes	CMIP
NFE2L21	9.94E-42	0.283278	0.575	0.455	2.80E-37	Monocytes	NFE2L2
RBM3	2.00E-41	0.273136	0.591	0.475	5.64E-37	Monocytes	RBM3
AOAH1	3.65E-41	0.28683	0.602	0.478	1.03E-36	Monocytes	AOAH
H2AFJ	1.45E-40	0.288654	0.354	0.245	4.08E-36	Monocytes	H2AFJ
CTSH2	2.79E-40	0.253346	0.652	0.54	7.86E-36	Monocytes	CTSH
ADA2	5.33E-40	0.293038	0.391	0.282	1.50E-35	Monocytes	ADA2
PPP1CA1	1.13E-39	0.254074	0.494	0.377	3.17E-35	Monocytes	PPP1CA
NUP214	1.13E-39	0.27361	0.37	0.259	3.19E-35	Monocytes	NUP214
GRK2	1.36E-39	0.272753	0.389	0.278	3.83E-35	Monocytes	GRK2
ZFYVE16	1.55E-39	0.312794	0.263	0.169	4.35E-35	Monocytes	ZFYVE16
CORO1A1	7.70E-39	0.274161	0.734	0.633	2.17E-34	Monocytes	CORO1A
NFKB2	7.90E-39	0.287159	0.276	0.181	2.22E-34	Monocytes	NFKB2
LYST	2.29E-37	0.292713	0.331	0.225	6.45E-33	Monocytes	LYST
EPB41L32	4.37E-37	0.301784	0.464	0.355	1.23E-32	Monocytes	EPB41L3
HNRNPC1	4.56E-37	0.255146	0.727	0.602	1.28E-32	Monocytes	HNRNPC
PSEN1	1.93E-36	0.266261	0.353	0.249	5.43E-32	Monocytes	PSEN1
JAK11	2.02E-36	0.250601	0.56	0.444	5.68E-32	Monocytes	JAK1
SND1	3.85E-36	0.256153	0.361	0.255	1.08E-31	Monocytes	SND1
ITGAX2	9.85E-36	0.429638	0.521	0.412	2.77E-31	Monocytes	ITGAX
RBMS1	1.78E-35	0.263697	0.352	0.249	5.01E-31	Monocytes	RBMS1
MAP3K21	2.13E-35	0.327638	0.505	0.395	5.98E-31	Monocytes	MAP3K2
STAT1	4.08E-35	0.272489	0.273	0.181	1.15E-30	Monocytes	STAT1
QSOX1	8.24E-35	0.25202	0.297	0.203	2.32E-30	Monocytes	QSOX1
PLEC	1.12E-34	0.252217	0.362	0.256	3.14E-30	Monocytes	PLEC
RAP1B1	4.30E-34	0.285261	0.526	0.42	1.21E-29	Monocytes	RAP1B
INSIG11	4.69E-34	0.273032	0.319	0.219	1.32E-29	Monocytes	INSIG1
PLSCR11	6.02E-34	0.284754	0.579	0.472	1.69E-29	Monocytes	PLSCR1
EIF1B	7.38E-34	0.272871	0.453	0.346	2.08E-29	Monocytes	EIF1B
TOP1	1.52E-33	0.275451	0.587	0.471	4.28E-29	Monocytes	TOP1
NFKBIZ3	7.17E-33	0.264753	0.649	0.532	2.02E-28	Monocytes	NFKBIZ
USP361	2.13E-32	0.275551	0.383	0.279	5.98E-28	Monocytes	USP36
RBM471	5.24E-32	0.29204	0.62	0.503	1.47E-27	Monocytes	RBM47
FLOT1	9.30E-32	0.294327	0.513	0.417	2.62E-27	Monocytes	FLOT1
IL1B1	1.96E-27	0.460166	0.698	0.648	5.51E-23	Monocytes	IL1B
SLC25A37	3.27E-27	0.296642	0.564	0.467	9.21E-23	Monocytes	SLC25A37
NCF1	4.60E-27	0.384471	0.432	0.345	1.29E-22	Monocytes	NCF1
CSF3R1	3.44E-26	0.25814	0.647	0.556	9.69E-22	Monocytes	CSF3R
WTAP2	1.63E-24	0.280577	0.516	0.426	4.60E-20	Monocytes	WTAP
HM13	5.52E-22	0.283346	0.458	0.374	1.55E-17	Monocytes	HM13
MX1	1.76E-19	0.263718	0.302	0.229	4.95E-15	Monocytes	MX1

RPS4Y12	2.29E-15	0.265569	0.473	0.429	6.44E-11	Monocytes	RPS4Y1
CTSD2	4.49E-13	0.256421	0.76	0.665	1.26E-08	Monocytes	CTSD
AL138963.4	1.34E-11	0.314973	0.262	0.209	3.78E-07	Monocytes	AL138963.4
TUBA1A1	1.11E-07	0.293089	0.339	0.303	0.003119	Monocytes	TUBA1A
H1FX1	2.65E-07	0.416469	0.432	0.387	0.007447	Monocytes	H1FX
IL32	0	1.905928	0.452	0.014	0	TAM-Inflan	IL32
IL7R	0	1.776254	0.314	0.011	0	TAM-Inflan	IL7R
CCL5	0	1.73131	0.482	0.11	0	TAM-Inflan	CCL5
IFITM12	0	1.562824	0.486	0.11	0	TAM-Inflan	IFITM1
FYN2	0	1.516797	0.422	0.043	0	TAM-Inflan	FYN
NKG7	0	1.419021	0.269	0.028	0	TAM-Inflan	NKG7
CD3E	0	1.405714	0.368	0.007	0	TAM-Inflan	CD3E
CD521	0	1.214928	0.326	0.026	0	TAM-Inflan	CD52
CST7	0	1.08211	0.268	0.023	0	TAM-Inflan	CST7
ETS1	0	1.074531	0.333	0.056	0	TAM-Inflan	ETS1
CD247	0	0.97915	0.25	0.008	0	TAM-Inflan	CD247
CRIP11	0	0.965079	0.266	0.031	0	TAM-Inflan	CRIP1
IL2RG	0	0.892839	0.262	0.039	0	TAM-Inflan	IL2RG
RUNX3	0	0.79123	0.255	0.039	0	TAM-Inflan	RUNX3
S100A102	5.30E-216	1.002596	0.363	0.106	1.49E-211	TAM-Inflan	S100A10
ISG202	1.57E-153	0.740882	0.296	0.094	4.40E-149	TAM-Inflan	ISG20
CBLB	5.25E-145	0.783437	0.287	0.092	1.48E-140	TAM-Inflan	CBLB
LY6E2	7.74E-139	0.943078	0.466	0.207	2.18E-134	TAM-Inflan	LY6E
PITPNC1	1.92E-136	0.719428	0.259	0.08	5.41E-132	TAM-Inflan	PITPNC1
CD442	6.10E-136	0.752111	0.354	0.131	1.72E-131	TAM-Inflan	CD44
CEMIP2	1.64E-120	0.867354	0.276	0.097	4.62E-116	TAM-Inflan	CEMIP2
SEPTIN91	2.45E-113	0.632715	0.253	0.085	6.90E-109	TAM-Inflan	SEPTIN9
S100A62	7.12E-113	0.825175	0.403	0.182	2.00E-108	TAM-Inflan	S100A6
RAC22	3.44E-111	0.778934	0.339	0.142	9.68E-107	TAM-Inflan	RAC2
B2M1	1.47E-106	0.425011	0.992	0.972	4.14E-102	TAM-Inflan	B2M
PARP8	7.60E-94	0.917475	0.407	0.21	2.14E-89	TAM-Inflan	PARP8
S100A42	9.01E-91	0.884693	0.49	0.285	2.54E-86	TAM-Inflan	S100A4
S100A91	4.79E-89	0.88701	0.342	0.158	1.35E-84	TAM-Inflan	S100A9
PPDPF2	1.19E-87	0.846949	0.444	0.252	3.35E-83	TAM-Inflan	PPDPF
HLA-B2	3.63E-82	0.424613	0.979	0.933	1.02E-77	TAM-Inflan	HLA-B
IFI63	6.76E-80	0.797245	0.491	0.279	1.90E-75	TAM-Inflan	IFI6
PFN13	2.51E-74	0.495932	0.886	0.827	7.07E-70	TAM-Inflan	PFN1
S100A81	1.07E-71	0.718814	0.328	0.161	3.01E-67	TAM-Inflan	S100A8
GNG21	7.94E-70	0.577276	0.275	0.123	2.23E-65	TAM-Inflan	GNG2
RPS264	9.18E-70	0.566101	0.867	0.813	2.58E-65	TAM-Inflan	RPS26
RPL284	8.53E-68	0.406424	0.968	0.919	2.40E-63	TAM-Inflan	RPL28
CYTIP2	2.31E-67	0.634038	0.348	0.182	6.50E-63	TAM-Inflan	CYTIP
VIM3	8.99E-67	0.587988	0.628	0.41	2.53E-62	TAM-Inflan	VIM
AL138963.4	9.34E-67	0.993367	0.37	0.208	2.63E-62	TAM-Inflan	AL138963.4
PDE7A	9.04E-64	0.538383	0.261	0.118	2.54E-59	TAM-Inflan	PDE7A
AREG3	4.24E-61	0.63949	0.316	0.153	1.19E-56	TAM-Inflan	AREG
RPL412	1.20E-59	0.606354	0.861	0.818	3.37E-55	TAM-Inflan	RPL41

MTRNR2L1	1.30E-57	0.880248	0.625	0.498	3.66E-53	TAM-Inflan	MTRNR2L12
CD7	9.65E-57	0.634367	0.26	0.128	2.71E-52	TAM-Inflan	CD7
RPL173	1.46E-56	0.392261	0.875	0.835	4.10E-52	TAM-Inflan	RPL17
RPS124	1.68E-55	0.356306	0.961	0.915	4.71E-51	TAM-Inflan	RPS12
HLA-A3	5.13E-55	0.44005	0.864	0.838	1.44E-50	TAM-Inflan	HLA-A
BTG13	1.84E-54	0.60941	0.786	0.758	5.17E-50	TAM-Inflan	BTG1
RPS33	1.00E-52	0.376987	0.922	0.877	2.82E-48	TAM-Inflan	RPS3
RPLP03	1.92E-52	0.36002	0.909	0.868	5.40E-48	TAM-Inflan	RPLP0
RPS15A4	6.81E-52	0.376808	0.933	0.887	1.92E-47	TAM-Inflan	RPS15A
ADGRE51	3.88E-51	0.470554	0.274	0.138	1.09E-46	TAM-Inflan	ADGRE5
TXNIP3	4.12E-50	0.784164	0.47	0.327	1.16E-45	TAM-Inflan	TXNIP
HLA-C3	1.12E-49	0.431843	0.851	0.83	3.16E-45	TAM-Inflan	HLA-C
RPL18A4	3.67E-49	0.355479	0.934	0.881	1.03E-44	TAM-Inflan	RPL18A
ARL4C1	3.56E-48	0.47382	0.265	0.135	1.00E-43	TAM-Inflan	ARL4C
IFITM33	4.15E-47	0.573797	0.597	0.463	1.17E-42	TAM-Inflan	IFITM3
RPL304	5.88E-47	0.364625	0.938	0.893	1.66E-42	TAM-Inflan	RPL30
IQGAP23	4.18E-46	0.44178	0.255	0.129	1.17E-41	TAM-Inflan	IQGAP2
TPT13	8.93E-44	0.259631	0.982	0.955	2.51E-39	TAM-Inflan	TPT1
RPS273	1.82E-43	0.394777	0.92	0.877	5.12E-39	TAM-Inflan	RPS27
DNAJB11	5.49E-43	0.336657	0.783	0.634	1.54E-38	TAM-Inflan	DNAJB1
RPS184	1.06E-42	0.291823	0.956	0.905	2.99E-38	TAM-Inflan	RPS18
LEPROTL1	3.11E-40	0.608722	0.35	0.224	8.76E-36	TAM-Inflan	LEPROTL1
PTMA4	2.15E-39	0.296286	0.918	0.906	6.06E-35	TAM-Inflan	PTMA
AHNAK3	1.14E-38	0.418879	0.321	0.187	3.21E-34	TAM-Inflan	AHNAK
MX11	3.07E-37	0.627066	0.356	0.229	8.65E-33	TAM-Inflan	MX1
SH3BGRL33	1.43E-36	0.483829	0.709	0.666	4.03E-32	TAM-Inflan	SH3BGRL3
DUSP24	1.53E-36	0.463173	0.545	0.396	4.29E-32	TAM-Inflan	DUSP2
RASA21	1.53E-36	0.482243	0.282	0.163	4.32E-32	TAM-Inflan	RASA2
SMCHD12	2.38E-36	0.590017	0.398	0.277	6.70E-32	TAM-Inflan	SMCHD1
ISG152	2.10E-35	0.465919	0.305	0.184	5.92E-31	TAM-Inflan	ISG15
TLE52	3.21E-35	0.502938	0.322	0.202	9.04E-31	TAM-Inflan	TLE5
PPP2R5C	1.09E-33	0.536257	0.308	0.19	3.06E-29	TAM-Inflan	PPP2R5C
LSP12	7.94E-32	0.450526	0.423	0.297	2.23E-27	TAM-Inflan	LSP1
PLAAT4	9.73E-30	0.48398	0.292	0.186	2.74E-25	TAM-Inflan	PLAAT4
MYL12A2	1.26E-29	0.559948	0.595	0.542	3.54E-25	TAM-Inflan	MYL12A
IFI44L1	1.38E-29	0.481233	0.363	0.242	3.89E-25	TAM-Inflan	IFI44L
ARHGDIB3	4.60E-29	0.39623	0.709	0.701	1.29E-24	TAM-Inflan	ARHGDIB
IRF11	4.85E-29	0.441828	0.268	0.164	1.37E-24	TAM-Inflan	IRF1
RPL84	1.59E-27	0.266506	0.875	0.853	4.49E-23	TAM-Inflan	RPL8
RPS73	1.63E-27	0.269156	0.887	0.861	4.58E-23	TAM-Inflan	RPS7
RPSA3	2.39E-27	0.376467	0.737	0.719	6.71E-23	TAM-Inflan	RPSA
RPL23A3	2.53E-26	0.315736	0.806	0.78	7.12E-22	TAM-Inflan	RPL23A
TAGLN22	3.74E-26	0.504076	0.416	0.314	1.05E-21	TAM-Inflan	TAGLN2
SLC2A32	1.47E-25	0.3542	0.507	0.384	4.13E-21	TAM-Inflan	SLC2A3
FNBP12	1.84E-25	0.451644	0.347	0.242	5.18E-21	TAM-Inflan	FNBP1
RPS92	3.39E-25	0.263788	0.88	0.86	9.53E-21	TAM-Inflan	RPS9
RPL10A3	3.57E-23	0.295032	0.779	0.765	1.00E-18	TAM-Inflan	RPL10A

FAM107B1	6.31E-23	0.403207	0.255	0.161	1.78E-18	TAM-Inflan	FAM107B
TNFAIP35	8.22E-23	0.345019	0.652	0.55	2.31E-18	TAM-Inflan	TNFAIP3
HIST1H1E1	3.28E-22	0.515586	0.318	0.22	9.24E-18	TAM-Inflan	HIST1H1E
CALM1	7.85E-22	0.388464	0.652	0.644	2.21E-17	TAM-Inflan	CALM1
GAPDH3	5.78E-21	0.270393	0.877	0.867	1.63E-16	TAM-Inflan	GAPDH
LCP12	7.52E-21	0.487831	0.511	0.437	2.12E-16	TAM-Inflan	LCP1
AKNA	2.89E-20	0.375866	0.296	0.204	8.13E-16	TAM-Inflan	AKNA
SELL1	3.69E-20	0.458072	0.259	0.175	1.04E-15	TAM-Inflan	SELL
CDC42SE2	4.47E-20	0.391305	0.33	0.237	1.26E-15	TAM-Inflan	CDC42SE2
RPS53	9.77E-20	0.278181	0.79	0.769	2.75E-15	TAM-Inflan	RPS5
CXCR43	1.30E-19	0.266028	0.566	0.459	3.67E-15	TAM-Inflan	CXCR4
ZC3HAV1	9.46E-19	0.343701	0.364	0.273	2.66E-14	TAM-Inflan	ZC3HAV1
MYH9	2.63E-18	0.443324	0.529	0.47	7.40E-14	TAM-Inflan	MYH9
IFITM23	3.42E-18	0.440115	0.563	0.52	9.63E-14	TAM-Inflan	IFITM2
STAT11	5.61E-18	0.462947	0.261	0.182	1.58E-13	TAM-Inflan	STAT1
RPL44	1.71E-16	0.273879	0.752	0.739	4.82E-12	TAM-Inflan	RPL4
ERN11	1.78E-15	0.346117	0.253	0.178	5.01E-11	TAM-Inflan	ERN1
CORO1A2	1.96E-15	0.337024	0.662	0.635	5.53E-11	TAM-Inflan	CORO1A
FCGBP1	2.64E-15	0.373092	0.457	0.381	7.42E-11	TAM-Inflan	FCGBP
CD691	2.82E-15	0.313578	0.579	0.493	7.94E-11	TAM-Inflan	CD69
TYMP1	2.93E-15	0.391759	0.424	0.352	8.25E-11	TAM-Inflan	TYMP
PSME23	3.96E-15	0.419846	0.421	0.35	1.11E-10	TAM-Inflan	PSME2
LDHA2	6.56E-15	0.453265	0.5	0.453	1.85E-10	TAM-Inflan	LDHA
MIF3	7.64E-15	0.365329	0.576	0.539	2.15E-10	TAM-Inflan	MIF
EEF1D3	3.65E-14	0.30418	0.681	0.695	1.03E-09	TAM-Inflan	EEF1D
RPL211	1.02E-13	0.251616	0.768	0.746	2.88E-09	TAM-Inflan	RPL21
LYZ3	1.14E-13	0.591778	0.331	0.269	3.21E-09	TAM-Inflan	LYZ
PIP4K2A1	2.28E-13	0.393823	0.33	0.257	6.41E-09	TAM-Inflan	PIP4K2A
H1FX2	3.89E-13	0.439485	0.453	0.387	1.09E-08	TAM-Inflan	H1FX
EIF2AK2	4.92E-13	0.425561	0.273	0.205	1.38E-08	TAM-Inflan	EIF2AK2
LRBA	1.20E-12	0.341657	0.262	0.192	3.39E-08	TAM-Inflan	LRBA
TIMP12	1.30E-12	0.351619	0.258	0.191	3.65E-08	TAM-Inflan	TIMP1
PTPRC1	4.21E-12	0.305513	0.686	0.715	1.18E-07	TAM-Inflan	PTPRC
LGALS12	4.91E-12	0.341517	0.276	0.208	1.38E-07	TAM-Inflan	LGALS1
PSME13	5.27E-12	0.37717	0.474	0.432	1.48E-07	TAM-Inflan	PSME1
PDCD41	8.14E-12	0.35469	0.342	0.274	2.29E-07	TAM-Inflan	PDCD4
HCST2	2.16E-11	0.319982	0.566	0.544	6.07E-07	TAM-Inflan	HCST
CALR1	2.36E-11	0.346684	0.598	0.567	6.64E-07	TAM-Inflan	CALR
CCND3	4.29E-11	0.367886	0.41	0.347	1.21E-06	TAM-Inflan	CCND3
PSMB93	1.53E-10	0.345995	0.339	0.281	4.32E-06	TAM-Inflan	PSMB9
UBE2S1	1.78E-10	0.326061	0.333	0.271	5.02E-06	TAM-Inflan	UBE2S
RHOH2	2.44E-10	0.281229	0.319	0.254	6.86E-06	TAM-Inflan	RHOH
RESF1	3.75E-10	0.405729	0.285	0.222	1.06E-05	TAM-Inflan	RESF1
FXYD53	6.25E-10	0.257329	0.581	0.568	1.76E-05	TAM-Inflan	FXYD5
ARMH11	8.97E-10	0.603874	0.258	0.204	2.52E-05	TAM-Inflan	ARMH1
ARHGAP9	1.13E-09	0.370963	0.294	0.237	3.19E-05	TAM-Inflan	ARHGAP9
PRKCH2	2.71E-09	0.265164	0.635	0.595	7.62E-05	TAM-Inflan	PRKCH

SERPINA13	3.02E-09	0.316832	0.566	0.543	8.49E-05	TAM-Inflan	SERPINA1
RNF2132	4.74E-09	0.380917	0.484	0.452	0.000133	TAM-Inflan	RNF213
HLA-F1	8.04E-09	0.307625	0.318	0.262	0.000226	TAM-Inflan	HLA-F
HLA-DQA2	1.15E-08	0.319509	0.271	0.214	0.000322	TAM-Inflan	HLA-DQA2
WIPF11	1.77E-08	0.365937	0.379	0.331	0.000497	TAM-Inflan	WIPF1
UTRN4	2.56E-08	0.291781	0.359	0.303	0.000719	TAM-Inflan	UTRN
STK17A	2.65E-08	0.362319	0.359	0.309	0.000745	TAM-Inflan	STK17A
LDHB2	5.40E-08	0.364939	0.384	0.336	0.001521	TAM-Inflan	LDHB
ARHGEF1	6.56E-08	0.32941	0.35	0.301	0.001845	TAM-Inflan	ARHGEF1
ADAR	2.21E-07	0.394735	0.338	0.295	0.00623	TAM-Inflan	ADAR
GTF3A1	3.18E-07	0.333731	0.337	0.291	0.008948	TAM-Inflan	GTF3A
BRD21	3.52E-07	0.307984	0.496	0.468	0.009908	TAM-Inflan	BRD2
EPSTI11	7.01E-07	0.287248	0.251	0.205	0.019726	TAM-Inflan	EPSTI1
PARP141	3.57E-06	0.352147	0.323	0.284	0.100546	TAM-Inflan	PARP14
G3BP2	7.48E-06	0.259978	0.266	0.224	0.21041	TAM-Inflan	G3BP2
SIK32	2.81E-05	0.252231	0.447	0.423	0.790694	TAM-Inflan	SIK3
OAS1	7.36E-05	0.323893	0.279	0.245	1	TAM-Inflan	OAS1
PRELID12	7.89E-05	0.267323	0.307	0.272	1	TAM-Inflan	PRELID1
RABGAP1L	8.99E-05	0.273051	0.416	0.389	1	TAM-Inflan	RABGAP1L
SP110	0.000177	0.267551	0.351	0.317	1	TAM-Inflan	SP110
RAD21	0.000194	0.263665	0.266	0.231	1	TAM-Inflan	RAD21
EVL	0.000245	0.285493	0.512	0.505	1	TAM-Inflan	EVL
SMDT13	0.000275	0.277016	0.385	0.36	1	TAM-Inflan	SMDT1
GSPT1	0.000438	0.295274	0.3	0.273	1	TAM-Inflan	GSPT1
UBE2L61	0.000731	0.27488	0.287	0.258	1	TAM-Inflan	UBE2L6
ENO13	0.000945	0.296283	0.48	0.479	1	TAM-Inflan	ENO1
RPS4Y13	0.000999	0.275777	0.43	0.43	1	TAM-Inflan	RPS4Y1
SSR23	0.002064	0.268201	0.407	0.398	1	TAM-Inflan	SSR2
PKM2	0.003125	0.278905	0.457	0.453	1	TAM-Inflan	PKM
TECR1	0.007516	0.277933	0.4	0.39	1	TAM-Inflan	TECR
AFF31	0	3.053503	0.412	0.059	0	Neutrophil	AFF3
CDK141	2.33E-305	2.130733	0.369	0.051	6.56E-301	Neutrophil	CDK14
SIPA1L14	6.54E-170	2.232521	0.832	0.563	1.84E-165	Neutrophil	SIPA1L1
DAPK11	1.25E-152	1.653697	0.264	0.048	3.52E-148	Neutrophil	DAPK1
MALAT11	9.02E-151	1.084667	1	0.989	2.54E-146	Neutrophil	MALAT1
AKAP132	2.62E-136	1.603254	0.88	0.78	7.38E-132	Neutrophil	AKAP13
FMN12	6.39E-115	2.034872	0.664	0.405	1.80E-110	Neutrophil	FMN1
UTRN5	1.03E-114	1.86156	0.591	0.302	2.91E-110	Neutrophil	UTRN
PRKCE2	1.37E-114	1.975532	0.49	0.203	3.84E-110	Neutrophil	PRKCE
AREG4	3.23E-114	1.687976	0.465	0.153	9.08E-110	Neutrophil	AREG
PLXDC22	2.13E-112	1.223912	0.939	0.935	5.98E-108	Neutrophil	PLXDC2
JARID23	2.27E-110	2.20971	0.56	0.273	6.39E-106	Neutrophil	JARID2
NEAT11	1.87E-107	1.460575	0.939	0.904	5.26E-103	Neutrophil	NEAT1
MS4A4E1	2.43E-100	1.788873	0.351	0.112	6.84E-96	Neutrophil	MS4A4E
PHLPP1	2.10E-98	1.520446	0.3	0.085	5.92E-94	Neutrophil	PHLPP1
CD443	7.93E-95	2.073822	0.381	0.133	2.23E-90	Neutrophil	CD44
CELF21	6.24E-92	1.478768	0.812	0.763	1.76E-87	Neutrophil	CELF2

DPYD2	3.79E-89	1.717959	0.664	0.462	1.07E-84	Neutrophil	DPYD
FOXP11	1.98E-87	1.70263	0.659	0.466	5.57E-83	Neutrophil	FOXP1
MCTP13	2.64E-87	1.920635	0.442	0.188	7.42E-83	Neutrophil	MCTP1
SLC8A11	1.92E-85	1.518325	0.778	0.711	5.41E-81	Neutrophil	SLC8A1
LRMDA1	1.63E-84	1.381743	0.796	0.753	4.60E-80	Neutrophil	LRMDA
PTK2B3	9.84E-83	1.948318	0.504	0.264	2.77E-78	Neutrophil	PTK2B
FOXN31	2.89E-81	1.327745	0.768	0.692	8.14E-77	Neutrophil	FOXN3
ELMO12	7.46E-81	1.331703	0.798	0.757	2.10E-76	Neutrophil	ELMO1
JMJD1C2	1.04E-78	1.751261	0.711	0.595	2.93E-74	Neutrophil	JMJD1C
SRGAP11	5.79E-78	1.810394	0.614	0.426	1.63E-73	Neutrophil	SRGAP1
TRPS12	7.72E-76	1.731557	0.476	0.242	2.17E-71	Neutrophil	TRPS1
ARHGAP15	8.23E-75	1.464624	0.687	0.57	2.31E-70	Neutrophil	ARHGAP15
DOCK52	1.57E-74	1.585488	0.411	0.181	4.43E-70	Neutrophil	DOCK5
MT-CO11	1.80E-71	0.980867	0.977	0.986	5.07E-67	Neutrophil	MT-CO1
MBNL11	1.93E-70	1.499713	0.743	0.679	5.43E-66	Neutrophil	MBNL1
CAMK1D1	2.15E-70	1.668008	0.594	0.414	6.04E-66	Neutrophil	CAMK1D
LDLRAD41	3.05E-70	1.147331	0.84	0.822	8.59E-66	Neutrophil	LDLRAD4
FKBP51	1.36E-69	1.516891	0.75	0.709	3.84E-65	Neutrophil	FKBP5
KYNU2	4.89E-69	1.880523	0.431	0.214	1.37E-64	Neutrophil	KYNU
FGD41	3.85E-66	1.25373	0.747	0.682	1.08E-61	Neutrophil	FGD4
MT-CO21	4.64E-65	1.025247	0.95	0.971	1.31E-60	Neutrophil	MT-CO2
ATP1B34	5.56E-65	2.020595	0.647	0.498	1.56E-60	Neutrophil	ATP1B3
NAMPT4	1.19E-64	1.771865	0.683	0.542	3.35E-60	Neutrophil	NAMPT
TET23	1.35E-64	1.633404	0.526	0.325	3.81E-60	Neutrophil	TET2
ARHGAP26	3.08E-64	1.160997	0.764	0.701	8.67E-60	Neutrophil	ARHGAP26
MT-ND4L1	1.21E-60	0.97822	0.904	0.926	3.42E-56	Neutrophil	MT-ND4L
ZFAND31	2.41E-59	1.424543	0.631	0.502	6.78E-55	Neutrophil	ZFAND3
GNAQ1	4.18E-59	1.267001	0.706	0.623	1.18E-54	Neutrophil	GNAQ
RNF144B4	7.65E-59	1.96006	0.496	0.305	2.15E-54	Neutrophil	RNF144B
ETV61	4.17E-58	1.485369	0.652	0.55	1.17E-53	Neutrophil	ETV6
IQGAP24	1.25E-57	1.384855	0.316	0.129	3.52E-53	Neutrophil	IQGAP2
CERS61	3.91E-57	1.46744	0.347	0.156	1.10E-52	Neutrophil	CERS6
CHST111	9.57E-57	1.37871	0.719	0.684	2.69E-52	Neutrophil	CHST11
ZEB22	1.46E-55	1.465839	0.689	0.611	4.12E-51	Neutrophil	ZEB2
MT-CO31	9.68E-53	0.926122	0.935	0.952	2.72E-48	Neutrophil	MT-CO3
AHR3	1.58E-52	1.589506	0.33	0.148	4.43E-48	Neutrophil	AHR
MT-ATP82	5.17E-52	1.182199	0.841	0.822	1.45E-47	Neutrophil	MT-ATP8
ACSL11	2.16E-50	1.517613	0.719	0.664	6.07E-46	Neutrophil	ACSL1
SIK33	2.74E-50	1.387248	0.575	0.422	7.71E-46	Neutrophil	SIK3
DENND1A2	7.49E-50	1.418149	0.409	0.224	2.11E-45	Neutrophil	DENND1A
MAML31	3.85E-49	1.386248	0.638	0.558	1.08E-44	Neutrophil	MAML3
TAOK34	1.32E-48	1.604332	0.572	0.451	3.72E-44	Neutrophil	TAOK3
RAPGEF12	4.56E-47	1.424909	0.638	0.539	1.28E-42	Neutrophil	RAPGEF1
ZSWIM61	8.37E-47	1.147992	0.692	0.619	2.36E-42	Neutrophil	ZSWIM6
FCHSD22	1.13E-46	1.673146	0.588	0.473	3.18E-42	Neutrophil	FCHSD2
PRKAG22	1.40E-46	1.55189	0.504	0.356	3.95E-42	Neutrophil	PRKAG2
RFTN11	2.55E-46	1.382732	0.286	0.124	7.18E-42	Neutrophil	RFTN1

RAB311	1.03E-45	1.200732	0.745	0.758	2.90E-41	Neutrophil	RAB31
CYTH12	4.97E-45	1.448193	0.462	0.296	1.40E-40	Neutrophil	CYTH1
LRRFIP12	1.28E-44	1.535977	0.619	0.529	3.59E-40	Neutrophil	LRRFIP1
SMCHD13	1.41E-44	1.619205	0.442	0.277	3.97E-40	Neutrophil	SMCHD1
SSH22	1.57E-44	1.370305	0.655	0.617	4.43E-40	Neutrophil	SSH2
IRAK32	4.66E-44	1.081761	0.698	0.656	1.31E-39	Neutrophil	IRAK3
NHSL11	4.86E-44	1.752601	0.479	0.323	1.37E-39	Neutrophil	NHSL1
MT-ND11	1.47E-43	0.843688	0.863	0.899	4.12E-39	Neutrophil	MT-ND1
ZBTB202	2.82E-41	1.208047	0.546	0.423	7.92E-37	Neutrophil	ZBTB20
ASAP11	7.81E-41	1.30998	0.386	0.221	2.20E-36	Neutrophil	ASAP1
HIPK22	1.41E-40	1.424377	0.347	0.187	3.96E-36	Neutrophil	HIPK2
MT-CYB1	1.77E-40	0.757607	0.947	0.96	4.98E-36	Neutrophil	MT-CYB
ADAM171	2.05E-40	1.417318	0.558	0.46	5.78E-36	Neutrophil	ADAM17
ANKRD111	1.53E-39	1.356859	0.611	0.538	4.32E-35	Neutrophil	ANKRD11
MT-ND52	2.39E-39	0.874674	0.846	0.88	6.72E-35	Neutrophil	MT-ND5
INSR1	4.73E-39	1.410267	0.333	0.176	1.33E-34	Neutrophil	INSR
CD1634	5.48E-39	1.166931	0.524	0.346	1.54E-34	Neutrophil	CD163
HDAC93	8.04E-39	1.587478	0.532	0.415	2.26E-34	Neutrophil	HDAC9
ARID1B1	1.30E-38	1.242632	0.462	0.317	3.66E-34	Neutrophil	ARID1B
MT-ND31	1.40E-37	0.897943	0.857	0.885	3.94E-33	Neutrophil	MT-ND3
ITSN1	1.43E-37	1.392212	0.353	0.2	4.02E-33	Neutrophil	ITSN1
ZBTB162	3.20E-37	1.481083	0.495	0.365	9.01E-33	Neutrophil	ZBTB16
CCNH1	1.80E-36	1.08728	0.728	0.723	5.06E-32	Neutrophil	CCNH
PTPN13	2.30E-36	1.534186	0.515	0.408	6.46E-32	Neutrophil	PTPN1
FAM49A2	9.23E-36	1.441976	0.456	0.33	2.60E-31	Neutrophil	FAM49A
ATP2B12	9.82E-36	1.56579	0.493	0.375	2.76E-31	Neutrophil	ATP2B1
MT-ND21	1.54E-35	0.817264	0.838	0.871	4.32E-31	Neutrophil	MT-ND2
FOXO32	2.32E-35	1.455576	0.498	0.384	6.52E-31	Neutrophil	FOXO3
NFKB12	5.85E-35	1.767162	0.653	0.604	1.65E-30	Neutrophil	NFKB1
MT-ND41	8.09E-35	0.811343	0.865	0.89	2.28E-30	Neutrophil	MT-ND4
VIM4	2.32E-34	0.897839	0.588	0.412	6.52E-30	Neutrophil	VIM
KDM6A1	4.51E-34	1.236085	0.35	0.204	1.27E-29	Neutrophil	KDM6A
ARIH11	8.91E-34	1.200482	0.543	0.451	2.51E-29	Neutrophil	ARIH1
MAN1A11	1.51E-33	1.390036	0.527	0.429	4.25E-29	Neutrophil	MAN1A1
CD553	1.94E-33	1.31521	0.482	0.353	5.45E-29	Neutrophil	CD55
TRIO1	1.87E-32	1.243112	0.446	0.318	5.27E-28	Neutrophil	TRIO
MED13L2	2.67E-32	1.102341	0.56	0.48	7.51E-28	Neutrophil	MED13L
RERE1	2.17E-31	1.210935	0.515	0.424	6.10E-27	Neutrophil	RERE
PTEN2	4.15E-31	1.385037	0.426	0.304	1.17E-26	Neutrophil	PTEN
OSBPL83	8.45E-31	1.38898	0.467	0.354	2.38E-26	Neutrophil	OSBPL8
TBC1D9	3.66E-30	1.739433	0.333	0.2	1.03E-25	Neutrophil	TBC1D9
PDE4B2	3.75E-30	1.240545	0.58	0.502	1.05E-25	Neutrophil	PDE4B
TBC1D22A1	1.71E-29	1.147891	0.529	0.446	4.82E-25	Neutrophil	TBC1D22A
STK17B3	5.09E-29	1.148092	0.504	0.401	1.43E-24	Neutrophil	STK17B
TMEM131	1.07E-28	1.090709	0.336	0.202	3.02E-24	Neutrophil	TMEM131
DOCK41	3.94E-28	0.91964	0.84	0.898	1.11E-23	Neutrophil	DOCK4
ELF12	5.61E-28	0.912256	0.672	0.671	1.58E-23	Neutrophil	ELF1

MIR181A1I	6.14E-28	1.429622	0.365	0.242	1.73E-23	Neutrophil	MIR181A1HG
THADA1	5.07E-27	1.247939	0.393	0.274	1.43E-22	Neutrophil	THADA
TANC21	7.00E-27	1.191186	0.369	0.242	1.97E-22	Neutrophil	TANC2
GAB21	1.44E-26	1.161909	0.549	0.493	4.06E-22	Neutrophil	GAB2
SRGAP21	1.56E-26	0.862084	0.692	0.726	4.38E-22	Neutrophil	SRGAP2
GRK3	1.58E-26	1.172346	0.3	0.176	4.45E-22	Neutrophil	GRK3
BTBD91	1.98E-26	1.12752	0.344	0.218	5.58E-22	Neutrophil	BTBD9
CUX12	2.17E-26	1.317852	0.481	0.398	6.11E-22	Neutrophil	CUX1
CMIP2	6.64E-26	1.31181	0.446	0.352	1.87E-21	Neutrophil	CMIP
QKI1	8.65E-26	0.708009	0.74	0.78	2.43E-21	Neutrophil	QKI
SMARCA22	1.67E-25	1.391282	0.412	0.312	4.70E-21	Neutrophil	SMARCA2
RBM472	3.02E-25	1.203348	0.552	0.506	8.51E-21	Neutrophil	RBM47
MAP2K12	3.74E-25	1.334994	0.442	0.353	1.05E-20	Neutrophil	MAP2K1
AHNAK4	9.56E-25	1.100371	0.317	0.188	2.69E-20	Neutrophil	AHNAK
PSTPIP22	1.78E-24	1.309275	0.255	0.142	5.02E-20	Neutrophil	PSTPIP2
NLRP34	1.97E-24	1.379991	0.58	0.52	5.55E-20	Neutrophil	NLRP3
BCL22	4.51E-24	1.20819	0.453	0.362	1.27E-19	Neutrophil	BCL2
SPIDR2	8.82E-24	1.033074	0.54	0.491	2.48E-19	Neutrophil	SPIDR
MAN2A11	1.23E-23	1.265364	0.456	0.372	3.45E-19	Neutrophil	MAN2A1
PTPRE3	1.80E-23	1.007492	0.549	0.507	5.05E-19	Neutrophil	PTPRE
NR4A32	3.02E-23	1.038124	0.667	0.633	8.50E-19	Neutrophil	NR4A3
CTNNB11	5.90E-23	1.261795	0.523	0.49	1.66E-18	Neutrophil	CTNNB1
TP53BP2	6.95E-23	1.216097	0.3	0.188	1.96E-18	Neutrophil	TP53BP2
PRKCB1	2.23E-22	1.063306	0.353	0.242	6.27E-18	Neutrophil	PRKCB
SUMF11	4.98E-22	1.098971	0.369	0.264	1.40E-17	Neutrophil	SUMF1
SYTL32	5.53E-22	1.195758	0.501	0.424	1.56E-17	Neutrophil	SYTL3
RUNX21	8.19E-22	1.22631	0.387	0.291	2.30E-17	Neutrophil	RUNX2
ATP9B1	8.73E-22	1.09086	0.319	0.209	2.46E-17	Neutrophil	ATP9B
UBAP12	1.16E-21	1.144518	0.378	0.278	3.28E-17	Neutrophil	UBAP1
RHOQ	1.46E-21	1.245721	0.364	0.264	4.10E-17	Neutrophil	RHOQ
RIN31	1.63E-21	1.075309	0.498	0.455	4.58E-17	Neutrophil	RIN3
TBC1D52	2.10E-21	1.070952	0.47	0.399	5.91E-17	Neutrophil	TBC1D5
RASA22	3.19E-21	1.175514	0.272	0.164	8.96E-17	Neutrophil	RASA2
KMT2C1	4.61E-21	0.968514	0.465	0.391	1.30E-16	Neutrophil	KMT2C
SFMBT22	6.23E-21	0.848395	0.639	0.639	1.75E-16	Neutrophil	SFMBT2
PPP1CB3	3.67E-20	1.154577	0.347	0.249	1.03E-15	Neutrophil	PPP1CB
MRTFA1	5.37E-20	1.059137	0.378	0.287	1.51E-15	Neutrophil	MRTFA
EXOC41	5.69E-20	1.082603	0.406	0.322	1.60E-15	Neutrophil	EXOC4
VPS13B1	8.16E-20	0.984365	0.345	0.243	2.30E-15	Neutrophil	VPS13B
PDE3B1	8.22E-20	1.143388	0.498	0.439	2.31E-15	Neutrophil	PDE3B
ELL22	1.20E-19	0.850802	0.725	0.79	3.37E-15	Neutrophil	ELL2
FNDC3B2	1.61E-19	1.267902	0.347	0.252	4.53E-15	Neutrophil	FNDC3B
ALCAM1	1.84E-19	1.178363	0.454	0.377	5.17E-15	Neutrophil	ALCAM
FP236383.3	2.13E-19	0.535659	0.701	0.504	5.99E-15	Neutrophil	FP236383.3
ESYT21	2.32E-19	1.316365	0.294	0.192	6.53E-15	Neutrophil	ESYT2
ANKRD442	2.42E-19	0.948772	0.572	0.577	6.80E-15	Neutrophil	ANKRD44
PDE8A1	2.69E-19	1.145309	0.403	0.314	7.56E-15	Neutrophil	PDE8A

MAP3K83	5.01E-19	0.991594	0.658	0.665	1.41E-14	Neutrophil	MAP3K8
SRGAP2B1	6.80E-19	0.919988	0.551	0.525	1.91E-14	Neutrophil	SRGAP2B
ITPR11	6.99E-19	1.035545	0.353	0.259	1.97E-14	Neutrophil	ITPR1
RASGEF1B2	8.31E-19	0.784884	0.647	0.647	2.34E-14	Neutrophil	RASGEF1B
REL2	9.04E-19	0.740872	0.709	0.75	2.54E-14	Neutrophil	REL
SPAG92	1.59E-18	1.070337	0.449	0.383	4.48E-14	Neutrophil	SPAG9
DOCK82	2.08E-18	0.808526	0.639	0.69	5.85E-14	Neutrophil	DOCK8
LYN1	3.63E-18	0.914559	0.624	0.669	1.02E-13	Neutrophil	LYN
DLEU21	5.17E-18	1.008922	0.457	0.401	1.45E-13	Neutrophil	DLEU2
ZNF3312	1.01E-17	1.117045	0.636	0.624	2.83E-13	Neutrophil	ZNF331
FTX2	1.25E-17	0.913121	0.353	0.259	3.51E-13	Neutrophil	FTX
TANK2	1.81E-17	1.365072	0.376	0.307	5.10E-13	Neutrophil	TANK
LRRK22	1.83E-17	1.024797	0.305	0.211	5.15E-13	Neutrophil	LRRK2
NFE2L22	1.83E-17	1.105766	0.485	0.458	5.16E-13	Neutrophil	NFE2L2
GABPB1	3.24E-17	1.150587	0.267	0.175	9.12E-13	Neutrophil	GABPB1
KDM4C	4.62E-17	0.919997	0.305	0.211	1.30E-12	Neutrophil	KDM4C
PIK3R51	4.84E-17	0.999019	0.496	0.464	1.36E-12	Neutrophil	PIK3R5
FAM172A1	6.42E-17	0.815669	0.309	0.212	1.81E-12	Neutrophil	FAM172A
SAMSN12	7.48E-17	1.207472	0.577	0.603	2.10E-12	Neutrophil	SAMSN1
AOAH2	8.17E-17	1.131224	0.498	0.481	2.30E-12	Neutrophil	AOAH
PAN31	1.32E-16	0.889292	0.337	0.247	3.70E-12	Neutrophil	PAN3
GNA132	1.53E-16	0.814407	0.594	0.604	4.29E-12	Neutrophil	GNA13
BASP14	2.64E-16	1.137153	0.569	0.563	7.44E-12	Neutrophil	BASP1
VTI1A1	2.70E-16	0.926741	0.325	0.237	7.60E-12	Neutrophil	VTI1A
TLR23	3.79E-16	1.082719	0.519	0.512	1.07E-11	Neutrophil	TLR2
HIVEP22	6.89E-16	1.212893	0.375	0.301	1.94E-11	Neutrophil	HIVEP2
SNX92	8.84E-16	1.045834	0.56	0.549	2.49E-11	Neutrophil	SNX9
RASSF31	9.38E-16	1.050276	0.252	0.163	2.64E-11	Neutrophil	RASSF3
AFF11	9.99E-16	1.020188	0.376	0.302	2.81E-11	Neutrophil	AFF1
MAP4K33	1.08E-15	0.809217	0.533	0.511	3.04E-11	Neutrophil	MAP4K3
GAS71	1.08E-15	1.118789	0.267	0.177	3.05E-11	Neutrophil	GAS7
SUSD61	1.15E-15	0.894487	0.471	0.435	3.24E-11	Neutrophil	SUSD6
CD833	1.48E-15	0.508154	0.879	0.912	4.17E-11	Neutrophil	CD83
MYO9B2	1.77E-15	0.961301	0.509	0.486	4.98E-11	Neutrophil	MYO9B
ARL151	2.28E-15	1.026642	0.328	0.245	6.40E-11	Neutrophil	ARL15
USP341	2.66E-15	0.958141	0.369	0.298	7.50E-11	Neutrophil	USP34
HNRNPC2	2.78E-15	0.918969	0.569	0.606	7.83E-11	Neutrophil	HNRNPC
STK38L1	3.09E-15	1.387434	0.397	0.33	8.71E-11	Neutrophil	STK38L
ATXN11	3.95E-15	1.054974	0.407	0.352	1.11E-10	Neutrophil	ATXN1
FNDC3A2	4.67E-15	1.113065	0.404	0.349	1.31E-10	Neutrophil	FNDC3A
LIMS12	4.75E-15	0.731687	0.659	0.69	1.34E-10	Neutrophil	LIMS1
NFAT51	4.80E-15	1.049766	0.412	0.351	1.35E-10	Neutrophil	NFAT5
NCOA21	5.12E-15	1.008929	0.305	0.221	1.44E-10	Neutrophil	NCOA2
FRYL1	5.69E-15	0.947919	0.339	0.258	1.60E-10	Neutrophil	FRYL
AGO21	8.15E-15	1.141984	0.253	0.17	2.29E-10	Neutrophil	AGO2
SBF21	8.36E-15	0.918015	0.393	0.329	2.35E-10	Neutrophil	SBF2
CDC42SE21	8.85E-15	1.051943	0.317	0.238	2.49E-10	Neutrophil	CDC42SE2

ADAMTSL4	8.92E-15	0.916238	0.404	0.336	2.51E-10	Neutrophil	ADAMTSL4-AS1
MKLN11	9.61E-15	0.881751	0.406	0.34	2.70E-10	Neutrophil	MKLN1
TNFAIP36	1.21E-14	1.085142	0.588	0.551	3.40E-10	Neutrophil	TNFAIP3
DYRK1A1	1.47E-14	0.932301	0.344	0.268	4.13E-10	Neutrophil	DYRK1A
CYTIP3	2.48E-14	1.046551	0.267	0.184	6.98E-10	Neutrophil	CYTIP
FNBP13	3.37E-14	1.060476	0.32	0.244	9.47E-10	Neutrophil	FNBP1
FOSL22	3.71E-14	1.001433	0.297	0.213	1.04E-09	Neutrophil	FOSL2
WWOX1	4.62E-14	1.15665	0.334	0.258	1.30E-09	Neutrophil	WWOX
SSBP21	5.51E-14	1.187603	0.323	0.246	1.55E-09	Neutrophil	SSBP2
LPP1	6.11E-14	0.784645	0.513	0.502	1.72E-09	Neutrophil	LPP
EPB41L33	6.82E-14	1.105466	0.406	0.358	1.92E-09	Neutrophil	EPB41L3
BCAS31	8.02E-14	0.929239	0.317	0.238	2.26E-09	Neutrophil	BCAS3
GLS1	8.59E-14	1.02533	0.49	0.478	2.42E-09	Neutrophil	GLS
RIC1	1.30E-13	0.972337	0.322	0.244	3.65E-09	Neutrophil	RIC1
ELF21	1.83E-13	1.081106	0.365	0.305	5.15E-09	Neutrophil	ELF2
PLAUR3	2.62E-13	0.717067	0.58	0.537	7.37E-09	Neutrophil	PLAUR
B4GALT12	2.64E-13	0.77183	0.591	0.605	7.42E-09	Neutrophil	B4GALT1
PELI11	2.67E-13	1.24902	0.538	0.547	7.50E-09	Neutrophil	PELI1
CHD21	4.31E-13	0.895839	0.473	0.459	1.21E-08	Neutrophil	CHD2
FNIP21	4.63E-13	1.081922	0.418	0.37	1.30E-08	Neutrophil	FNIP2
ROCK11	5.20E-13	0.992235	0.454	0.438	1.46E-08	Neutrophil	ROCK1
DISC12	5.28E-13	0.875976	0.426	0.383	1.48E-08	Neutrophil	DISC1
DENND4A1	6.26E-13	0.975245	0.373	0.315	1.76E-08	Neutrophil	DENND4A
RUNX11	7.23E-13	0.792743	0.588	0.601	2.03E-08	Neutrophil	RUNX1
UVRAG2	8.51E-13	0.933186	0.417	0.381	2.40E-08	Neutrophil	UVRAG
FBXW111	9.79E-13	0.941227	0.389	0.339	2.75E-08	Neutrophil	FBXW11
ST3GAL1	1.25E-12	0.948526	0.302	0.228	3.52E-08	Neutrophil	ST3GAL1
SMAP22	1.33E-12	0.592127	0.701	0.77	3.73E-08	Neutrophil	SMAP2
PAG11	1.40E-12	0.947622	0.46	0.437	3.93E-08	Neutrophil	PAG1
IMMP2L1	1.41E-12	1.058859	0.327	0.261	3.96E-08	Neutrophil	IMMP2L
KDM2A1	1.48E-12	0.946898	0.328	0.262	4.16E-08	Neutrophil	KDM2A
MT-ND62	1.67E-12	0.703874	0.568	0.561	4.69E-08	Neutrophil	MT-ND6
FMNL21	1.72E-12	1.003317	0.495	0.482	4.84E-08	Neutrophil	FMNL2
BIRC61	1.77E-12	0.859429	0.376	0.323	4.98E-08	Neutrophil	BIRC6
FBXL17	2.25E-12	0.859108	0.261	0.184	6.34E-08	Neutrophil	FBXL17
FBXO111	2.78E-12	0.976524	0.4	0.36	7.83E-08	Neutrophil	FBXO11
KDM4B1	3.06E-12	0.986858	0.336	0.272	8.61E-08	Neutrophil	KDM4B
CSGALNAC	3.99E-12	1.00839	0.505	0.517	1.12E-07	Neutrophil	CSGALNACT1
COP11	4.30E-12	0.905587	0.336	0.274	1.21E-07	Neutrophil	COP1
DOCK101	1.46E-11	0.867171	0.502	0.508	4.10E-07	Neutrophil	DOCK10
CCNL11	1.75E-11	0.618842	0.596	0.646	4.92E-07	Neutrophil	CCNL1
RIPK23	1.80E-11	1.077365	0.308	0.244	5.05E-07	Neutrophil	RIPK2
MAP3K22	2.22E-11	0.977721	0.423	0.398	6.24E-07	Neutrophil	MAP3K2
LITAF3	3.41E-11	1.042647	0.459	0.446	9.59E-07	Neutrophil	LITAF
ARHGAP24	4.09E-11	0.861869	0.636	0.725	1.15E-06	Neutrophil	ARHGAP24
RABGEF12	5.42E-11	1.019579	0.428	0.396	1.53E-06	Neutrophil	RABGEF1
UBAC21	6.00E-11	0.766177	0.482	0.478	1.69E-06	Neutrophil	UBAC2

CCDC26	6.29E-11	0.970373	0.253	0.182	1.77E-06	Neutrophil	CCDC26
UBE2E21	6.84E-11	0.825804	0.504	0.53	1.92E-06	Neutrophil	UBE2E2
CMSS12	7.50E-11	1.244612	0.303	0.243	2.11E-06	Neutrophil	CMSS1
ANKRD12	7.68E-11	0.867658	0.381	0.337	2.16E-06	Neutrophil	ANKRD12
LRBA1	9.95E-11	0.767991	0.263	0.193	2.80E-06	Neutrophil	LRBA
RREB11	1.02E-10	0.817839	0.291	0.223	2.88E-06	Neutrophil	RREB1
ATG71	1.13E-10	0.963229	0.395	0.361	3.18E-06	Neutrophil	ATG7
FAM102B1	1.24E-10	0.906175	0.325	0.267	3.48E-06	Neutrophil	FAM102B
KIF13B	1.27E-10	1.154589	0.266	0.202	3.57E-06	Neutrophil	KIF13B
HERC11	1.36E-10	0.881199	0.297	0.233	3.84E-06	Neutrophil	HERC1
KANSL12	1.41E-10	0.844694	0.322	0.262	3.97E-06	Neutrophil	KANSL1
MACF11	3.25E-10	0.772036	0.457	0.447	9.15E-06	Neutrophil	MACF1
RANBP21	3.57E-10	0.810149	0.565	0.57	1.01E-05	Neutrophil	RANBP2
IPMK	4.11E-10	0.813333	0.253	0.186	1.16E-05	Neutrophil	IPMK
TRAPPC91	4.46E-10	0.888164	0.309	0.249	1.25E-05	Neutrophil	TRAPPC9
RAD51B1	4.48E-10	0.960012	0.344	0.295	1.26E-05	Neutrophil	RAD51B
DSE1	5.44E-10	0.979197	0.457	0.454	1.53E-05	Neutrophil	DSE
GPRIN3	5.62E-10	0.952705	0.314	0.262	1.58E-05	Neutrophil	GPRIN3
SNX291	5.67E-10	0.840263	0.406	0.378	1.60E-05	Neutrophil	SNX29
MANBA2	5.89E-10	0.902415	0.515	0.552	1.66E-05	Neutrophil	MANBA
FHIT1	6.83E-10	0.929299	0.443	0.433	1.92E-05	Neutrophil	FHIT
CHD11	1.16E-09	0.888762	0.443	0.432	3.25E-05	Neutrophil	CHD1
PIP4K2A2	1.81E-09	0.919942	0.308	0.258	5.09E-05	Neutrophil	PIP4K2A
PFKFB31	1.85E-09	0.885304	0.533	0.568	5.22E-05	Neutrophil	PFKFB3
RBM391	1.96E-09	0.600041	0.594	0.671	5.51E-05	Neutrophil	RBM39
BABAM21	4.20E-09	0.802621	0.331	0.286	0.000118	Neutrophil	BABAM2
UBE2D31	4.48E-09	0.730012	0.583	0.658	0.000126	Neutrophil	UBE2D3
RCOR11	4.50E-09	0.836644	0.28	0.225	0.000127	Neutrophil	RCOR1
FNIP11	4.87E-09	0.922311	0.359	0.326	0.000137	Neutrophil	FNIP1
FAM53B1	5.14E-09	0.926543	0.325	0.279	0.000145	Neutrophil	FAM53B
MSR13	5.34E-09	0.631753	0.551	0.579	0.00015	Neutrophil	MSR1
ATP8B42	5.72E-09	0.948462	0.457	0.461	0.000161	Neutrophil	ATP8B4
ZNF6091	5.73E-09	0.792195	0.292	0.237	0.000161	Neutrophil	ZNF609
CFLAR2	5.83E-09	0.728993	0.473	0.465	0.000164	Neutrophil	CFLAR
MTRNR2L1	5.97E-09	0.637827	0.313	0.501	0.000168	Neutrophil	MTRNR2L12
CIITA	6.58E-09	0.892687	0.308	0.259	0.000185	Neutrophil	CIITA
FBXW7	8.86E-09	0.838719	0.261	0.205	0.000249	Neutrophil	FBXW7
DTNA1	1.84E-08	0.836549	0.257	0.201	0.000519	Neutrophil	DTNA
TBXAS12	1.94E-08	0.653546	0.613	0.72	0.000546	Neutrophil	TBXAS1
NIPBL1	2.12E-08	0.772415	0.401	0.395	0.000598	Neutrophil	NIPBL
MEF2A2	2.14E-08	0.543639	0.672	0.787	0.000601	Neutrophil	MEF2A
TOP11	2.66E-08	0.901702	0.459	0.474	0.000748	Neutrophil	TOP1
RHOH3	2.83E-08	1.352313	0.297	0.254	0.000797	Neutrophil	RHOH
ARL8B2	2.92E-08	0.963688	0.356	0.332	0.000822	Neutrophil	ARL8B
ZMIZ11	3.37E-08	0.736314	0.355	0.317	0.000948	Neutrophil	ZMIZ1
ERC11	3.56E-08	0.775887	0.294	0.246	0.001001	Neutrophil	ERC1
RFX31	4.28E-08	0.753129	0.252	0.196	0.001204	Neutrophil	RFX3

CDKAL11	4.29E-08	0.748187	0.252	0.196	0.001206	Neutrophil	CDKAL1
PCNX22	4.58E-08	0.746334	0.33	0.288	0.001289	Neutrophil	PCNX2
COG51	4.92E-08	0.700222	0.283	0.232	0.001384	Neutrophil	COG5
KAT6A	6.14E-08	0.773959	0.258	0.206	0.001727	Neutrophil	KAT6A
ZFC3H1	7.16E-08	0.801853	0.316	0.276	0.002016	Neutrophil	ZFC3H1
USP9X1	8.41E-08	0.755286	0.32	0.279	0.002367	Neutrophil	USP9X
DST1	8.42E-08	0.883971	0.373	0.352	0.00237	Neutrophil	DST
ACSL32	8.98E-08	1.198969	0.303	0.261	0.002526	Neutrophil	ACSL3
XPR11	9.78E-08	0.734315	0.269	0.216	0.002751	Neutrophil	XPR1
CSF2RA2	1.13E-07	0.596414	0.607	0.677	0.003171	Neutrophil	CSF2RA
ANKS1A1	1.21E-07	0.770358	0.348	0.319	0.003417	Neutrophil	ANKS1A
UBR5	1.38E-07	0.871385	0.26	0.211	0.003888	Neutrophil	UBR5
MALT12	1.56E-07	0.979617	0.303	0.264	0.004403	Neutrophil	MALT1
DOCK21	1.83E-07	0.756671	0.435	0.447	0.005146	Neutrophil	DOCK2
DIP2B2	2.22E-07	0.824731	0.348	0.319	0.006246	Neutrophil	DIP2B
FAF11	2.22E-07	0.789831	0.32	0.285	0.006252	Neutrophil	FAF1
KMT2E1	2.39E-07	0.804473	0.418	0.429	0.006712	Neutrophil	KMT2E
FILIP1L2	2.82E-07	0.858508	0.309	0.27	0.007927	Neutrophil	FILIP1L
PPP3CA1	2.85E-07	0.851206	0.306	0.27	0.00801	Neutrophil	PPP3CA
VMP12	3.08E-07	0.752353	0.437	0.454	0.008656	Neutrophil	VMP1
ABCA11	3.25E-07	1.008249	0.407	0.401	0.009136	Neutrophil	ABCA1
PREX11	3.47E-07	0.746254	0.521	0.592	0.009767	Neutrophil	PREX1
RNF1492	3.79E-07	0.697414	0.509	0.565	0.010662	Neutrophil	RNF149
USP151	4.40E-07	0.677365	0.474	0.51	0.012393	Neutrophil	USP15
MYO1E1	4.74E-07	0.39649	0.221	0.358	0.013346	Neutrophil	MYO1E
ADGRE21	5.30E-07	0.880703	0.261	0.217	0.01492	Neutrophil	ADGRE2
EIF4G31	5.72E-07	0.786279	0.39	0.382	0.016093	Neutrophil	EIF4G3
USP362	5.92E-07	0.960452	0.316	0.281	0.016664	Neutrophil	USP36
ASH1L1	6.04E-07	0.724776	0.381	0.367	0.017004	Neutrophil	ASH1L
PTPRC2	7.07E-07	0.600808	0.597	0.716	0.019896	Neutrophil	PTPRC
MB21D21	7.19E-07	0.834409	0.28	0.238	0.020219	Neutrophil	MB21D2
GPCPD12	7.26E-07	0.830535	0.4	0.393	0.020422	Neutrophil	GPCPD1
WDR701	7.69E-07	0.751032	0.291	0.251	0.021644	Neutrophil	WDR70
MAML21	9.38E-07	0.747132	0.509	0.553	0.026382	Neutrophil	MAML2
STK101	1.12E-06	0.813835	0.274	0.232	0.031534	Neutrophil	STK10
TNRC6B1	1.20E-06	0.831299	0.327	0.303	0.033668	Neutrophil	TNRC6B
EHMT11	1.20E-06	0.722919	0.272	0.231	0.033703	Neutrophil	EHMT1
DYM	1.71E-06	0.766942	0.253	0.214	0.048057	Neutrophil	DYM
HIPK31	2.10E-06	0.744074	0.308	0.277	0.058995	Neutrophil	HIPK3
MPRIP1	2.10E-06	0.896857	0.272	0.234	0.059148	Neutrophil	MPRIP
SH2B32	2.39E-06	0.701574	0.378	0.369	0.06737	Neutrophil	SH2B3
RBPJ1	2.42E-06	0.853095	0.345	0.338	0.068202	Neutrophil	RBPJ
TNRC181	2.46E-06	0.779059	0.277	0.239	0.069307	Neutrophil	TNRC18
JAZF11	2.61E-06	0.796501	0.468	0.508	0.073385	Neutrophil	JAZF1
IGSF211	3.21E-06	0.267958	0.25	0.401	0.090186	Neutrophil	IGSF21
MBNL21	3.52E-06	0.724157	0.258	0.218	0.098917	Neutrophil	MBNL2
LCOR1	3.57E-06	0.834251	0.272	0.235	0.100338	Neutrophil	LCOR

PICALM2	3.76E-06	0.594361	0.583	0.687	0.105911	Neutrophil	PICALM
STAG11	5.38E-06	0.669611	0.407	0.416	0.151337	Neutrophil	STAG1
ADAM282	5.63E-06	0.583157	0.532	0.596	0.158375	Neutrophil	ADAM28
PIK3R11	5.67E-06	0.854284	0.481	0.524	0.159652	Neutrophil	PIK3R1
CCNY1	5.88E-06	0.733123	0.348	0.338	0.165566	Neutrophil	CCNY
RPS6KA3	5.92E-06	0.82721	0.351	0.341	0.166437	Neutrophil	RPS6KA3
PHF201	5.98E-06	0.870539	0.386	0.394	0.168269	Neutrophil	PHF20
ABR1	6.02E-06	0.638892	0.495	0.54	0.169526	Neutrophil	ABR
FAM49B1	6.26E-06	0.554525	0.552	0.645	0.176164	Neutrophil	FAM49B
AUTS21	6.43E-06	0.913235	0.337	0.315	0.180997	Neutrophil	AUTS2
TMEM1232	6.45E-06	0.263631	0.16	0.267	0.181486	Neutrophil	TMEM123
STX42	6.48E-06	0.306699	0.191	0.309	0.182297	Neutrophil	STX4
TCF121	6.82E-06	0.662564	0.507	0.56	0.191769	Neutrophil	TCF12
NSMCE21	7.31E-06	0.748486	0.359	0.349	0.205568	Neutrophil	NSMCE2
SLC7A51	7.47E-06	0.928731	0.387	0.388	0.210079	Neutrophil	SLC7A5
TRAK1	8.96E-06	0.650623	0.252	0.212	0.252076	Neutrophil	TRAK1
BACH11	1.08E-05	0.795442	0.375	0.381	0.303217	Neutrophil	BACH1
PRKCH3	1.09E-05	0.551723	0.547	0.596	0.305495	Neutrophil	PRKCH
SPPL3	1.26E-05	0.712049	0.257	0.221	0.354283	Neutrophil	SPPL3
PSME41	1.27E-05	0.661157	0.289	0.257	0.358393	Neutrophil	PSME4
RGCC2	1.41E-05	0.655825	0.323	0.276	0.39626	Neutrophil	RGCC
IQGAP13	1.51E-05	0.713854	0.317	0.297	0.424871	Neutrophil	IQGAP1
RABGAP1L1	1.59E-05	0.829668	0.378	0.39	0.446736	Neutrophil	RABGAP1L
PADI22	1.70E-05	0.341184	0.397	0.582	0.477473	Neutrophil	PADI2
NUMB2	1.88E-05	0.765046	0.367	0.373	0.530104	Neutrophil	NUMB
TBC1D11	1.94E-05	0.776711	0.364	0.371	0.547218	Neutrophil	TBC1D1
RNF2133	2.37E-05	0.843453	0.428	0.453	0.666946	Neutrophil	RNF213
PPP6R31	2.40E-05	0.713982	0.294	0.269	0.674844	Neutrophil	PPP6R3
NUP981	2.89E-05	0.771671	0.317	0.305	0.81385	Neutrophil	NUP98
CSNK1A12	3.46E-05	0.64005	0.454	0.502	0.974527	Neutrophil	CSNK1A1
STX72	4.30E-05	0.260568	0.202	0.321	1	Neutrophil	STX7
FGL23	4.80E-05	0.397059	0.272	0.424	1	Neutrophil	FGL2
MT-ATP62	5.04E-05	0.519814	0.787	0.857	1	Neutrophil	MT-ATP6
AP3B11	5.46E-05	0.703275	0.3	0.283	1	Neutrophil	AP3B1
SRGAP2C1	5.89E-05	0.663274	0.409	0.427	1	Neutrophil	SRGAP2C
DDX24	5.99E-05	0.310861	0.325	0.493	1	Neutrophil	DDX24
GRASP2	6.08E-05	0.412735	0.538	0.55	1	Neutrophil	GRASP
SND11	7.07E-05	0.72834	0.281	0.258	1	Neutrophil	SND1
CTBP21	9.52E-05	0.782812	0.263	0.239	1	Neutrophil	CTBP2
PRCP1	0.000112	0.324068	0.163	0.256	1	Neutrophil	PRCP
ITGAX3	0.00013	0.809152	0.393	0.416	1	Neutrophil	ITGAX
SETD2	0.00017	0.68072	0.267	0.246	1	Neutrophil	SETD2
FAM149A2	0.00018	0.263223	0.165	0.256	1	Neutrophil	FAM149A
CLK12	0.000186	0.685923	0.375	0.387	1	Neutrophil	CLK1
VAPA	0.000193	0.307892	0.32	0.49	1	Neutrophil	VAPA
JAK12	0.000216	0.771545	0.406	0.448	1	Neutrophil	JAK1
DENND5A2	0.000236	0.920521	0.275	0.258	1	Neutrophil	DENND5A

MTSS11	0.000249	0.280645	0.221	0.319	1	Neutrophil	MTSS1
CTTNBP2NI	0.000275	0.650728	0.362	0.373	1	Neutrophil	CTTNBP2NL
NUFIP22	0.000303	0.655396	0.418	0.444	1	Neutrophil	NUFIP2
PSEN11	0.000303	0.778319	0.269	0.252	1	Neutrophil	PSEN1
HSPH11	0.000322	0.313209	0.333	0.462	1	Neutrophil	HSPH1
RRBP11	0.000336	0.342651	0.184	0.28	1	Neutrophil	RRBP1
RBM61	0.000338	0.637957	0.319	0.315	1	Neutrophil	RBM6
IFRD11	0.000346	0.633124	0.459	0.501	1	Neutrophil	IFRD1
YWHAQ1	0.000358	0.308789	0.233	0.355	1	Neutrophil	YWHAQ
AC020916.	0.000383	0.572969	0.526	0.586	1	Neutrophil	AC020916.1
BAZ2B1	0.000384	0.684924	0.369	0.381	1	Neutrophil	BAZ2B
GSK3B1	0.000401	0.615725	0.269	0.25	1	Neutrophil	GSK3B
PPARD1	0.000421	0.744027	0.339	0.343	1	Neutrophil	PPARD
GTF2B1	0.000431	0.255326	0.257	0.378	1	Neutrophil	GTF2B
ANXA13	0.000437	0.796465	0.358	0.344	1	Neutrophil	ANXA1
ARID4B1	0.000466	0.695263	0.333	0.34	1	Neutrophil	ARID4B
PDE4DIP1	0.000484	0.28764	0.199	0.294	1	Neutrophil	PDE4DIP
PNPLA81	0.00049	0.853076	0.294	0.29	1	Neutrophil	PNPLA8
CUL3	0.000508	0.702205	0.283	0.272	1	Neutrophil	CUL3
JAK22	0.000536	0.79213	0.297	0.293	1	Neutrophil	JAK2
NCOA31	0.000541	0.642661	0.337	0.338	1	Neutrophil	NCOA3
RAB7A2	0.000587	0.720833	0.515	0.607	1	Neutrophil	RAB7A
NF11	0.000587	0.646438	0.303	0.296	1	Neutrophil	NF1
CLASP2	0.000617	0.604685	0.25	0.227	1	Neutrophil	CLASP2
RCC21	0.00069	0.310514	0.241	0.358	1	Neutrophil	RCC2
LINC02256	0.00072	0.279491	0.246	0.367	1	Neutrophil	LINC02256
ATP2B1-AS	0.00073	0.317023	0.348	0.503	1	Neutrophil	ATP2B1-AS1
INPP5D2	0.000742	0.599416	0.454	0.511	1	Neutrophil	INPP5D
EZR1	0.00077	0.909041	0.369	0.394	1	Neutrophil	EZR
ETF11	0.000785	0.666279	0.378	0.404	1	Neutrophil	ETF1
SEMA4D1	0.000805	0.286882	0.187	0.278	1	Neutrophil	SEMA4D
DNAJC31	0.000822	0.279528	0.193	0.287	1	Neutrophil	DNAJC3
MAP4K42	0.000838	0.347759	0.255	0.373	1	Neutrophil	MAP4K4
STAB1	0.000862	0.319544	0.236	0.348	1	Neutrophil	STAB1
IFNGR22	0.000876	0.263925	0.373	0.547	1	Neutrophil	IFNGR2
BTAF11	0.000882	0.625722	0.286	0.275	1	Neutrophil	BTAF1
ATP6V1B2	0.000955	0.412131	0.325	0.477	1	Neutrophil	ATP6V1B2
SP1001	0.001005	0.339323	0.348	0.521	1	Neutrophil	SP100
BRAF1	0.001012	0.645567	0.261	0.242	1	Neutrophil	BRAF
NKTR	0.001032	0.63233	0.257	0.241	1	Neutrophil	NKTR
THEMIS2	0.001102	0.29939	0.271	0.4	1	Neutrophil	THEMIS2
RNF13	0.001159	0.324769	0.278	0.416	1	Neutrophil	RNF13
CPQ	0.001168	0.709066	0.336	0.347	1	Neutrophil	CPQ
APLP21	0.001257	0.27193	0.323	0.479	1	Neutrophil	APLP2
AMD1	0.001292	0.282451	0.174	0.262	1	Neutrophil	AMD1
TUT71	0.00136	0.262384	0.222	0.326	1	Neutrophil	TUT7
SYK1	0.001367	0.308161	0.347	0.513	1	Neutrophil	SYK

RFX22	0.001634	0.293373	0.207	0.297	1	Neutrophil:	RFX2
TRIP121	0.001754	0.585058	0.303	0.303	1	Neutrophil:	TRIP12
KCNMA11	0.001854	0.431419	0.185	0.271	1	Neutrophil:	KCNMA1
BBX1	0.001881	0.666226	0.258	0.246	1	Neutrophil:	BBX
MGAT51	0.001977	0.62759	0.313	0.305	1	Neutrophil:	MGAT5
SEPTIN61	0.002189	0.358193	0.207	0.302	1	Neutrophil:	SEPTIN6
MORF4L2	0.002239	0.250749	0.221	0.321	1	Neutrophil:	MORF4L2
SNRNP70	0.002253	0.261971	0.264	0.384	1	Neutrophil:	SNRNP70
SYAP12	0.002258	0.770074	0.365	0.379	1	Neutrophil:	SYAP1
CREBBP1	0.00238	0.604559	0.264	0.256	1	Neutrophil:	CREBBP
INTS62	0.00253	0.264743	0.227	0.325	1	Neutrophil:	INTS6
DNAJB62	0.002645	0.430678	0.305	0.447	1	Neutrophil:	DNAJB6
KCNQ31	0.002678	0.586007	0.344	0.504	1	Neutrophil:	KCNQ3
PRDM12	0.002979	0.255285	0.253	0.355	1	Neutrophil:	PRDM1
ITGB1	0.003013	0.280714	0.246	0.359	1	Neutrophil:	ITGB1
ETNK11	0.003014	0.275891	0.208	0.301	1	Neutrophil:	ETNK1
NFKBIZ4	0.003127	0.529936	0.498	0.536	1	Neutrophil:	NFKBIZ
PUM21	0.003167	0.616967	0.266	0.258	1	Neutrophil:	PUM2
PRKN1	0.003269	0.604264	0.257	0.241	1	Neutrophil:	PRKN
SRSF111	0.003625	0.2533	0.32	0.461	1	Neutrophil:	SRSF11
CHD92	0.00367	0.253463	0.33	0.476	1	Neutrophil:	CHD9
GALNT21	0.003696	0.672955	0.401	0.445	1	Neutrophil:	GALNT2
PIAS11	0.003901	0.669204	0.378	0.423	1	Neutrophil:	PIAS1
ERGIC1	0.003941	0.292571	0.24	0.344	1	Neutrophil:	ERGIC1
ITGAV2	0.004121	0.316507	0.327	0.454	1	Neutrophil:	ITGAV
PARVG	0.004494	0.310326	0.291	0.419	1	Neutrophil:	PARVG
CDK62	0.004509	0.27442	0.185	0.26	1	Neutrophil:	CDK6
DAGLB2	0.004717	0.713324	0.39	0.433	1	Neutrophil:	DAGLB
TRA2A1	0.005398	0.563131	0.314	0.321	1	Neutrophil:	TRA2A
GPR1324	0.005569	0.9797	0.28	0.285	1	Neutrophil:	GPR132
SARNP1	0.006268	0.269802	0.188	0.267	1	Neutrophil:	SARNP
IDS	0.006302	0.298007	0.299	0.425	1	Neutrophil:	IDS
12-Mar	0.006519	0.807834	0.288	0.294	1	Neutrophil:	1-Mar
VPS13D1	0.006865	0.56142	0.261	0.255	1	Neutrophil:	VPS13D
ZFAND54	0.007182	0.463621	0.454	0.5	1	Neutrophil:	ZFAND5
MAPKAP11	0.007408	0.646814	0.257	0.251	1	Neutrophil:	MAPKAP1
DDX171	0.007499	0.271052	0.358	0.515	1	Neutrophil:	DDX17
MGAT12	0.007548	0.366715	0.35	0.5	1	Neutrophil:	MGAT1
NXF11	0.007569	0.299378	0.179	0.251	1	Neutrophil:	NXF1
AZIN1-AS1:	0.007613	0.927139	0.342	0.36	1	Neutrophil:	AZIN1-AS1
PACSIN21	0.008022	0.680587	0.33	0.354	1	Neutrophil:	PACSIN2
MYH91	0.008351	0.282145	0.331	0.472	1	Neutrophil:	MYH9
UBL31	0.008504	0.718575	0.334	0.365	1	Neutrophil:	UBL3
ERICH11	0.009835	0.398773	0.205	0.291	1	Neutrophil:	ERICH1
LGALS13	0	2.470753	0.787	0.206	0	TAM-lysos(	LGALS1
GPNMB	0	2.242795	0.616	0.038	0	TAM-lysos(	GPNMB
LGALS31	8.03E-307	1.612416	0.4	0.051	2.26E-302	TAM-lysos(	LGALS3

NUPR1	1.95E-223	1.729698	0.337	0.049	5.47E-219	TAM-lysosc	NUPR1
ANXA22	3.82E-220	1.321714	0.499	0.101	1.08E-215	TAM-lysosc	ANXA2
CD92	1.13E-179	1.294233	0.559	0.147	3.17E-175	TAM-lysosc	CD9
FTL2	4.90E-157	1.40999	1	0.974	1.38E-152	TAM-lysosc	FTL
S100A103	1.11E-151	1.579813	0.446	0.108	3.12E-147	TAM-lysosc	S100A10
CSTB2	5.85E-142	1.886721	0.82	0.418	1.65E-137	TAM-lysosc	CSTB
VIM5	1.79E-131	1.984779	0.813	0.411	5.03E-127	TAM-lysosc	VIM
GAPDH4	3.97E-128	2.072306	0.989	0.866	1.12E-123	TAM-lysosc	GAPDH
CD63	2.77E-115	1.103986	0.908	0.655	7.80E-111	TAM-lysosc	CD63
MIF4	2.33E-114	2.207538	0.836	0.538	6.56E-110	TAM-lysosc	MIF
CTSD3	1.49E-106	1.467651	0.905	0.666	4.20E-102	TAM-lysosc	CTSD
ENO14	1.82E-99	1.409879	0.79	0.477	5.11E-95	TAM-lysosc	ENO1
PLIN23	2.85E-97	1.816409	0.684	0.327	8.01E-93	TAM-lysosc	PLIN2
LPL	1.26E-95	1.030383	0.363	0.101	3.53E-91	TAM-lysosc	LPL
CD682	2.33E-89	0.962227	0.917	0.705	6.56E-85	TAM-lysosc	CD68
EMP32	6.24E-89	1.101325	0.644	0.297	1.76E-84	TAM-lysosc	EMP3
ALDOA3	4.03E-85	1.392595	0.76	0.455	1.13E-80	TAM-lysosc	ALDOA
FTH14	1.78E-84	1.125377	1	0.984	5.02E-80	TAM-lysosc	FTH1
IFI303	5.98E-84	1.173741	0.914	0.689	1.68E-79	TAM-lysosc	IFI30
PPDPF3	9.25E-84	1.046155	0.586	0.253	2.60E-79	TAM-lysosc	PPDPF
S100A112	1.05E-81	0.842162	0.966	0.83	2.96E-77	TAM-lysosc	S100A11
LDHA3	1.31E-81	1.676722	0.751	0.451	3.67E-77	TAM-lysosc	LDHA
SH3BGRL34	1.88E-80	0.998017	0.891	0.666	5.29E-76	TAM-lysosc	SH3BGRL3
PKM3	6.98E-80	1.062856	0.758	0.451	1.96E-75	TAM-lysosc	PKM
ERO1A	2.51E-78	0.948627	0.362	0.118	7.05E-74	TAM-lysosc	ERO1A
GPI1	1.31E-76	1.126287	0.538	0.244	3.68E-72	TAM-lysosc	GPI
ATF5	2.82E-76	0.839988	0.354	0.113	7.93E-72	TAM-lysosc	ATF5
ATP6V1F1	4.56E-76	0.942023	0.801	0.519	1.28E-71	TAM-lysosc	ATP6V1F
BRI33	1.36E-75	0.95796	0.822	0.52	3.83E-71	TAM-lysosc	BRI3
P4HB1	3.72E-74	1.396027	0.635	0.338	1.05E-69	TAM-lysosc	P4HB
SDF2L1	1.12E-73	1.043801	0.423	0.156	3.15E-69	TAM-lysosc	SDF2L1
SPP12	1.35E-71	1.963492	0.935	0.832	3.81E-67	TAM-lysosc	SPP1
FABP51	8.22E-70	1.297723	0.506	0.222	2.31E-65	TAM-lysosc	FABP5
LSP13	1.21E-69	1.055425	0.607	0.297	3.39E-65	TAM-lysosc	LSP1
TXN2	8.76E-69	1.079638	0.457	0.185	2.47E-64	TAM-lysosc	TXN
TPI11	1.31E-67	1.114725	0.855	0.61	3.68E-63	TAM-lysosc	TPI1
SEC61G1	1.68E-67	1.038167	0.566	0.273	4.74E-63	TAM-lysosc	SEC61G
HM131	1.05E-63	0.96193	0.661	0.375	2.96E-59	TAM-lysosc	HM13
ADM	9.56E-63	1.288982	0.356	0.13	2.69E-58	TAM-lysosc	ADM
ATP6V0C2	1.01E-62	0.751706	0.899	0.695	2.84E-58	TAM-lysosc	ATP6V0C
PGK11	2.19E-62	1.086689	0.783	0.512	6.16E-58	TAM-lysosc	PGK1
VEGFB	1.11E-59	0.781304	0.55	0.268	3.13E-55	TAM-lysosc	VEGFB
CTSL1	3.78E-59	1.350552	0.653	0.37	1.06E-54	TAM-lysosc	CTSL
SERF22	2.34E-56	0.692399	0.935	0.801	6.59E-52	TAM-lysosc	SERF2
TAGLN23	2.53E-56	0.723603	0.608	0.314	7.12E-52	TAM-lysosc	TAGLN2
APOC12	1.32E-55	0.915789	0.933	0.783	3.72E-51	TAM-lysosc	APOC1
PLP22	4.08E-55	0.547971	0.342	0.125	1.15E-50	TAM-lysosc	PLP2

C4orf32	3.02E-54	0.872183	0.587	0.316	8.50E-50	TAM-lysos(	C4orf3
EIF4EBP11	5.88E-54	0.668931	0.393	0.16	1.65E-49	TAM-lysos(	EIF4EBP1
MT2A2	5.89E-54	1.861469	0.661	0.387	1.66E-49	TAM-lysos(	MT2A
S100A63	8.02E-54	0.450361	0.448	0.184	2.26E-49	TAM-lysos(	S100A6
FAM20C	7.48E-53	0.548006	0.337	0.124	2.11E-48	TAM-lysos(	FAM20C
MANF	2.74E-52	0.778679	0.353	0.139	7.70E-48	TAM-lysos(	MANF
GSTO11	1.24E-51	0.610467	0.517	0.249	3.49E-47	TAM-lysos(	GSTO1
METRNL3	4.11E-51	0.855265	0.427	0.195	1.16E-46	TAM-lysos(	METRNL
PDIA6	2.49E-50	0.967785	0.545	0.287	7.00E-46	TAM-lysos(	PDIA6
SNHG121	3.12E-50	1.698157	0.469	0.226	8.78E-46	TAM-lysos(	SNHG12
RNF187	1.35E-49	0.53553	0.33	0.126	3.79E-45	TAM-lysos(	RNF187
VDAC1	2.09E-49	0.687366	0.561	0.299	5.87E-45	TAM-lysos(	VDAC1
PGAM12	3.80E-48	0.648046	0.547	0.282	1.07E-43	TAM-lysos(	PGAM1
PPP1R14B	5.10E-48	0.55216	0.466	0.218	1.43E-43	TAM-lysos(	PPP1R14B
SLC3A21	1.69E-47	0.829559	0.642	0.381	4.75E-43	TAM-lysos(	SLC3A2
UPP12	1.86E-47	0.613608	0.337	0.132	5.23E-43	TAM-lysos(	UPP1
GUK11	2.08E-47	0.73928	0.656	0.403	5.86E-43	TAM-lysos(	GUK1
HPCAL11	5.28E-47	0.380643	0.342	0.132	1.49E-42	TAM-lysos(	HPCAL1
RALA2	1.30E-46	0.618469	0.31	0.118	3.65E-42	TAM-lysos(	RALA
RGCC3	3.42E-46	1.240584	0.527	0.275	9.62E-42	TAM-lysos(	RGCC
MYL62	5.06E-46	0.610054	0.914	0.772	1.42E-41	TAM-lysos(	MYL6
MPP11	8.27E-46	0.516583	0.527	0.265	2.33E-41	TAM-lysos(	MPP1
CLIC12	2.24E-45	0.664493	0.861	0.657	6.31E-41	TAM-lysos(	CLIC1
TYMP2	2.32E-45	0.707908	0.608	0.351	6.52E-41	TAM-lysos(	TYMP
BNIP3L1	6.35E-45	0.835489	0.593	0.343	1.79E-40	TAM-lysos(	BNIP3L
VKORC11	9.57E-45	0.593739	0.49	0.245	2.69E-40	TAM-lysos(	VKORC1
MGAT13	1.45E-44	0.823979	0.734	0.498	4.09E-40	TAM-lysos(	MGAT1
BCAP311	2.99E-44	0.605561	0.7	0.453	8.40E-40	TAM-lysos(	BCAP31
TMEM70	6.00E-44	0.511661	0.455	0.213	1.69E-39	TAM-lysos(	TMEM70
CALR2	5.27E-42	0.980162	0.774	0.566	1.48E-37	TAM-lysos(	CALR
GBE1	1.21E-41	0.667111	0.369	0.163	3.39E-37	TAM-lysos(	GBE1
FBP11	1.44E-41	0.600043	0.326	0.135	4.06E-37	TAM-lysos(	FBP1
SSR31	1.62E-41	0.693139	0.601	0.357	4.57E-37	TAM-lysos(	SSR3
GNG51	4.31E-41	0.519738	0.843	0.62	1.21E-36	TAM-lysos(	GNG5
SMIM3	2.13E-40	0.580216	0.4	0.185	6.00E-36	TAM-lysos(	SMIM3
GYPC	9.03E-40	0.61156	0.57	0.322	2.54E-35	TAM-lysos(	GYPC
ACTG11	1.20E-39	0.631278	0.929	0.79	3.39E-35	TAM-lysos(	ACTG1
SSR42	1.79E-39	0.724859	0.734	0.494	5.04E-35	TAM-lysos(	SSR4
MITF	1.93E-39	0.446821	0.416	0.195	5.43E-35	TAM-lysos(	MITF
ZYX3	3.21E-39	0.632308	0.684	0.444	9.02E-35	TAM-lysos(	ZYX
RNASEK2	6.56E-39	0.548623	0.887	0.708	1.85E-34	TAM-lysos(	RNASEK
TMED2	9.30E-39	0.728299	0.471	0.247	2.62E-34	TAM-lysos(	TMED2
ANXA52	1.18E-38	0.574289	0.831	0.633	3.32E-34	TAM-lysos(	ANXA5
PPIB	1.51E-38	0.908548	0.808	0.63	4.25E-34	TAM-lysos(	PPIB
AP3S1	2.84E-38	0.389211	0.296	0.117	7.99E-34	TAM-lysos(	AP3S1
KDELR2	5.73E-38	0.550624	0.541	0.3	1.61E-33	TAM-lysos(	KDELR2
TMEM51	8.17E-38	0.455368	0.339	0.147	2.30E-33	TAM-lysos(	TMEM51

RNH11	1.90E-37	0.492551	0.578	0.339	5.35E-33	TAM-lysos(	RNH1
FGR2	2.05E-37	0.422395	0.335	0.143	5.76E-33	TAM-lysos(	FGR
DYNLL1	3.18E-37	0.544636	0.614	0.37	8.94E-33	TAM-lysos(	DYNLL1
GALM	5.05E-37	0.320129	0.259	0.097	1.42E-32	TAM-lysos(	GALM
RPL413	7.57E-37	0.666497	0.951	0.818	2.13E-32	TAM-lysos(	RPL41
SLC31A1	9.48E-37	0.330584	0.291	0.117	2.67E-32	TAM-lysos(	SLC31A1
HSP90B1	1.10E-36	1.397793	0.783	0.585	3.09E-32	TAM-lysos(	HSP90B1
CCDC107	1.22E-36	0.40431	0.325	0.138	3.43E-32	TAM-lysos(	CCDC107
CREG1	2.05E-36	0.579693	0.695	0.457	5.77E-32	TAM-lysos(	CREG1
FAM162A	2.46E-36	0.665133	0.397	0.196	6.92E-32	TAM-lysos(	FAM162A
DSTN	6.24E-36	0.401443	0.365	0.166	1.76E-31	TAM-lysos(	DSTN
CNIH41	1.50E-35	0.359779	0.28	0.112	4.21E-31	TAM-lysos(	CNIH4
RNF1811	2.36E-35	0.529204	0.501	0.272	6.65E-31	TAM-lysos(	RNF181
PSMA71	4.15E-35	0.536226	0.709	0.482	1.17E-30	TAM-lysos(	PSMA7
RAC23	4.26E-35	0.485449	0.326	0.144	1.20E-30	TAM-lysos(	RAC2
DCXR	5.30E-35	0.526626	0.441	0.231	1.49E-30	TAM-lysos(	DCXR
CD151	1.46E-34	0.384458	0.272	0.109	4.10E-30	TAM-lysos(	CD151
PDIA4	1.80E-34	0.758981	0.321	0.145	5.07E-30	TAM-lysos(	PDIA4
PCBP11	1.91E-34	0.597024	0.628	0.409	5.39E-30	TAM-lysos(	PCBP1
YBX13	2.16E-34	0.46639	0.859	0.666	6.09E-30	TAM-lysos(	YBX1
SEC61B1	5.78E-34	0.671139	0.698	0.47	1.63E-29	TAM-lysos(	SEC61B
ARL4C2	3.02E-33	0.54577	0.309	0.136	8.51E-29	TAM-lysos(	ARL4C
MT1X	6.31E-33	1.899812	0.317	0.151	1.78E-28	TAM-lysos(	MT1X
NOP102	7.09E-33	0.443468	0.557	0.327	1.99E-28	TAM-lysos(	NOP10
CNBP1	9.65E-33	0.611566	0.737	0.52	2.72E-28	TAM-lysos(	CNBP
DHRS3	1.28E-32	0.40523	0.411	0.204	3.59E-28	TAM-lysos(	DHRS3
RPN1	1.62E-32	0.528419	0.596	0.367	4.55E-28	TAM-lysos(	RPN1
SLC16A31	1.62E-32	0.636288	0.591	0.374	4.56E-28	TAM-lysos(	SLC16A3
OSTC1	2.05E-32	0.647401	0.439	0.24	5.76E-28	TAM-lysos(	OSTC
DERL2	6.86E-32	0.35877	0.323	0.145	1.93E-27	TAM-lysos(	DERL2
TRAM1	8.09E-32	0.542505	0.489	0.274	2.28E-27	TAM-lysos(	TRAM1
TRMT1121	8.22E-32	0.477116	0.619	0.387	2.31E-27	TAM-lysos(	TRMT112
LIPA1	8.91E-32	0.5476	0.556	0.333	2.51E-27	TAM-lysos(	LIPA
CANX1	1.40E-31	0.650215	0.713	0.491	3.95E-27	TAM-lysos(	CANX
PLTP2	2.28E-31	0.58193	0.409	0.212	6.43E-27	TAM-lysos(	PLTP
HINT12	4.08E-31	0.455446	0.69	0.449	1.15E-26	TAM-lysos(	HINT1
UBE2M	4.96E-31	0.438893	0.372	0.182	1.39E-26	TAM-lysos(	UBE2M
CALM11	1.23E-30	0.625675	0.811	0.643	3.46E-26	TAM-lysos(	CALM1
SH3GLB1	1.71E-30	0.43987	0.466	0.255	4.80E-26	TAM-lysos(	SH3GLB1
TIMP13	1.75E-30	0.814654	0.379	0.191	4.93E-26	TAM-lysos(	TIMP1
CD99	2.23E-30	0.513887	0.794	0.586	6.28E-26	TAM-lysos(	CD99
CTSB1	3.98E-30	0.514343	0.966	0.918	1.12E-25	TAM-lysos(	CTSB
GPX41	4.19E-30	0.526071	0.811	0.635	1.18E-25	TAM-lysos(	GPX4
SPCS2	5.65E-30	0.470413	0.55	0.332	1.59E-25	TAM-lysos(	SPCS2
CHCHD22	9.07E-30	0.425721	0.794	0.574	2.55E-25	TAM-lysos(	CHCHD2
FCGR2B3	1.41E-29	0.680777	0.305	0.142	3.97E-25	TAM-lysos(	FCGR2B
C7orf502	2.66E-29	0.326867	0.325	0.149	7.47E-25	TAM-lysos(	C7orf50

FERMT3	2.89E-29	0.474772	0.517	0.3	8.13E-25	TAM-lysosc	FERMT3
PTMA5	3.89E-29	0.413896	0.981	0.906	1.10E-24	TAM-lysosc	PTMA
RPL104	6.11E-29	0.403732	1	0.946	1.72E-24	TAM-lysosc	RPL10
COX7A21	6.97E-29	0.442416	0.589	0.373	1.96E-24	TAM-lysosc	COX7A2
DAD11	7.71E-29	0.531162	0.661	0.454	2.17E-24	TAM-lysosc	DAD1
PDIA3	8.63E-29	0.699056	0.746	0.544	2.43E-24	TAM-lysosc	PDIA3
PTMS	9.17E-29	0.413569	0.45	0.247	2.58E-24	TAM-lysosc	PTMS
OAZ13	1.07E-28	0.383471	0.94	0.797	3.01E-24	TAM-lysosc	OAZ1
GLDN	1.58E-28	1.020117	0.377	0.209	4.44E-24	TAM-lysosc	GLDN
HSD17B4	1.59E-28	0.456984	0.323	0.155	4.47E-24	TAM-lysosc	HSD17B4
CDK2AP2	1.81E-28	0.559189	0.342	0.172	5.08E-24	TAM-lysosc	CDK2AP2
MYDGF	1.82E-28	0.660272	0.556	0.362	5.11E-24	TAM-lysosc	MYDGF
BAG1	1.86E-28	0.352491	0.374	0.186	5.24E-24	TAM-lysosc	BAG1
ASAH11	2.07E-28	0.515244	0.838	0.674	5.83E-24	TAM-lysosc	ASAH1
EIF12	2.10E-28	0.504844	0.984	0.924	5.91E-24	TAM-lysosc	EIF1
LMAN1	2.75E-28	0.602105	0.432	0.238	7.74E-24	TAM-lysosc	LMAN1
HDLBP1	3.16E-28	0.451144	0.476	0.268	8.89E-24	TAM-lysosc	HDLBP
PSMD81	4.47E-28	0.357951	0.496	0.279	1.26E-23	TAM-lysosc	PSMD8
RAB13	4.99E-28	0.39246	0.275	0.122	1.41E-23	TAM-lysosc	RAB13
TMEM2582	5.44E-28	0.615812	0.607	0.379	1.53E-23	TAM-lysosc	TMEM258
BLOC1S2	6.18E-28	0.340882	0.309	0.145	1.74E-23	TAM-lysosc	BLOC1S2
COPE2	1.03E-27	0.51807	0.642	0.431	2.89E-23	TAM-lysosc	COPE
ATP6V1G1	1.17E-27	0.498219	0.623	0.416	3.30E-23	TAM-lysosc	ATP6V1G1
TMSB103	1.20E-27	0.481081	0.982	0.925	3.39E-23	TAM-lysosc	TMSB10
TMED91	4.66E-27	0.461675	0.589	0.377	1.31E-22	TAM-lysosc	TMED9
CORO1C	6.63E-27	0.385188	0.425	0.233	1.86E-22	TAM-lysosc	CORO1C
CIAO2B1	1.22E-26	0.350783	0.496	0.287	3.42E-22	TAM-lysosc	CIAO2B
ANXA14	1.80E-26	0.584771	0.563	0.343	5.08E-22	TAM-lysosc	ANXA1
LAMTOR5	1.88E-26	0.356778	0.436	0.24	5.28E-22	TAM-lysosc	LAMTOR5
PFKL	2.32E-26	0.371821	0.4	0.215	6.52E-22	TAM-lysosc	PFKL
CSNK2B1	2.44E-26	0.423267	0.653	0.442	6.86E-22	TAM-lysosc	CSNK2B
EIF5A1	2.71E-26	0.395279	0.552	0.341	7.62E-22	TAM-lysosc	EIF5A
ZNF385A2	2.72E-26	0.448742	0.374	0.195	7.66E-22	TAM-lysosc	ZNF385A
PFN14	3.22E-26	0.442919	0.931	0.827	9.07E-22	TAM-lysosc	PFN1
COX6A1	3.42E-26	0.399823	0.801	0.61	9.63E-22	TAM-lysosc	COX6A1
GRINA1	3.50E-26	0.450794	0.642	0.427	9.84E-22	TAM-lysosc	GRINA
HLA-A4	3.53E-26	0.489664	0.942	0.838	9.92E-22	TAM-lysosc	HLA-A
ARHGDIA	6.59E-26	0.428345	0.621	0.407	1.85E-21	TAM-lysosc	ARHGDIA
UBA523	7.25E-26	0.370416	0.922	0.776	2.04E-21	TAM-lysosc	UBA52
NCF21	1.15E-25	0.390668	0.287	0.134	3.24E-21	TAM-lysosc	NCF2
NDUFAF31	1.17E-25	0.357714	0.478	0.273	3.29E-21	TAM-lysosc	NDUFAF3
PLD3	1.18E-25	0.47163	0.55	0.347	3.33E-21	TAM-lysosc	PLD3
PSMA62	1.22E-25	0.434822	0.522	0.313	3.44E-21	TAM-lysosc	PSMA6
CCNI2	1.30E-25	0.537839	0.776	0.587	3.66E-21	TAM-lysosc	CCNI
FAU4	1.52E-25	0.392202	0.982	0.88	4.27E-21	TAM-lysosc	FAU
PRDX1	3.69E-25	0.500334	0.691	0.503	1.04E-20	TAM-lysosc	PRDX1
HSPA51	4.11E-25	1.291651	0.762	0.584	1.16E-20	TAM-lysosc	HSPA5

CARD19	4.97E-25	0.363422	0.314	0.151	1.40E-20	TAM-lysos(	CARD19
RPL85	5.14E-25	0.373785	0.963	0.853	1.45E-20	TAM-lysos(	RPL8
RHOC1	6.39E-25	0.430197	0.439	0.254	1.80E-20	TAM-lysos(	RHOC
BCKDK	8.51E-25	0.347453	0.282	0.134	2.39E-20	TAM-lysos(	BCKDK
TYROBP3	1.10E-24	0.312502	0.961	0.91	3.10E-20	TAM-lysos(	TYROBP
RAB5IF1	1.18E-24	0.385763	0.397	0.22	3.33E-20	TAM-lysos(	RAB5IF
BCAT11	1.39E-24	0.326881	0.326	0.161	3.90E-20	TAM-lysos(	BCAT1
PPP4C	2.90E-24	0.360043	0.496	0.297	8.15E-20	TAM-lysos(	PPP4C
SSR24	3.05E-24	0.591681	0.582	0.397	8.59E-20	TAM-lysos(	SSR2
CALU	3.84E-24	0.319196	0.279	0.131	1.08E-19	TAM-lysos(	CALU
RPS27L1	4.35E-24	0.453942	0.54	0.335	1.22E-19	TAM-lysos(	RPS27L
RHOA1	5.73E-24	0.376339	0.892	0.76	1.61E-19	TAM-lysos(	RHOA
BLVRB1	6.15E-24	0.381897	0.36	0.191	1.73E-19	TAM-lysos(	BLVRB
KDELR11	6.90E-24	0.434844	0.571	0.373	1.94E-19	TAM-lysos(	KDELR1
LRRC59	7.31E-24	0.261898	0.337	0.171	2.06E-19	TAM-lysos(	LRRC59
H2AFY2	7.35E-24	0.397021	0.705	0.517	2.07E-19	TAM-lysos(	H2AFY
CADM11	7.75E-24	0.289369	0.254	0.116	2.18E-19	TAM-lysos(	CADM1
SNX32	8.61E-24	0.375796	0.705	0.492	2.42E-19	TAM-lysos(	SNX3
SEC11C	1.34E-23	0.614878	0.279	0.138	3.77E-19	TAM-lysos(	SEC11C
UBL52	2.04E-23	0.401405	0.672	0.467	5.75E-19	TAM-lysos(	UBL5
FAM50A	2.39E-23	0.316307	0.434	0.243	6.72E-19	TAM-lysos(	FAM50A
UCP21	4.31E-23	0.502347	0.545	0.354	1.21E-18	TAM-lysos(	UCP2
LAGE3	5.11E-23	0.290975	0.314	0.159	1.44E-18	TAM-lysos(	LAGE3
ARF1	6.25E-23	0.391747	0.737	0.541	1.76E-18	TAM-lysos(	ARF1
ELOC1	6.32E-23	0.356628	0.617	0.403	1.78E-18	TAM-lysos(	ELOC
TMBIM6	6.97E-23	0.434416	0.827	0.655	1.96E-18	TAM-lysos(	TMBIM6
NANS	8.95E-23	0.32841	0.515	0.313	2.52E-18	TAM-lysos(	NANS
PGD1	1.20E-22	0.399099	0.377	0.208	3.36E-18	TAM-lysos(	PGD
ATP5MC32	1.21E-22	0.413892	0.637	0.437	3.41E-18	TAM-lysos(	ATP5MC3
MGST3	1.25E-22	0.441517	0.573	0.386	3.51E-18	TAM-lysos(	MGST3
GSDME	2.38E-22	0.383726	0.261	0.127	6.70E-18	TAM-lysos(	GSDME
EMD	2.39E-22	0.325925	0.467	0.277	6.72E-18	TAM-lysos(	EMD
RPL36AL3	2.77E-22	0.434169	0.85	0.659	7.80E-18	TAM-lysos(	RPL36AL
OST41	3.38E-22	0.42822	0.67	0.476	9.52E-18	TAM-lysos(	OST4
COPZ1	3.84E-22	0.314471	0.316	0.163	1.08E-17	TAM-lysos(	COPZ1
AURKAIP11	4.24E-22	0.260427	0.446	0.256	1.19E-17	TAM-lysos(	AURKAIP1
WARS1	4.45E-22	0.43766	0.314	0.165	1.25E-17	TAM-lysos(	WARS
SELENOW1	4.77E-22	0.38831	0.524	0.328	1.34E-17	TAM-lysos(	SELENOW
RAN1	5.36E-22	0.38561	0.656	0.458	1.51E-17	TAM-lysos(	RAN
PRDX61	7.12E-22	0.311981	0.549	0.348	2.00E-17	TAM-lysos(	PRDX6
SCARB2	7.32E-22	0.295859	0.397	0.223	2.06E-17	TAM-lysos(	SCARB2
NDUFB92	8.06E-22	0.339587	0.519	0.322	2.27E-17	TAM-lysos(	NDUFB9
ATP5MD1	8.53E-22	0.381749	0.601	0.395	2.40E-17	TAM-lysos(	ATP5MD
LITAF4	1.02E-21	0.376211	0.66	0.445	2.88E-17	TAM-lysos(	LITAF
TNFRSF12A	1.10E-21	0.631068	0.291	0.149	3.11E-17	TAM-lysos(	TNFRSF12A
ARL8A	2.40E-21	0.348245	0.354	0.194	6.75E-17	TAM-lysos(	ARL8A
ATP5PF1	2.99E-21	0.373935	0.556	0.362	8.40E-17	TAM-lysos(	ATP5PF

ARPC22	4.14E-21	0.420885	0.785	0.637	1.16E-16	TAM-lysos(	ARPC2
SEC61A1	4.16E-21	0.415286	0.321	0.171	1.17E-16	TAM-lysos(	SEC61A1
TMEM167/	5.47E-21	0.323636	0.325	0.174	1.54E-16	TAM-lysos(	TMEM167A
NPL1	5.94E-21	0.274155	0.536	0.336	1.67E-16	TAM-lysos(	NPL
EEF1A13	6.01E-21	0.290885	0.996	0.964	1.69E-16	TAM-lysos(	EEF1A1
CLEC2B1	7.23E-21	0.623525	0.492	0.311	2.03E-16	TAM-lysos(	CLEC2B
CD444	8.58E-21	0.34564	0.272	0.134	2.41E-16	TAM-lysos(	CD44
UBE2B	8.87E-21	0.397373	0.543	0.35	2.50E-16	TAM-lysos(	UBE2B
ISG153	1.07E-20	0.796673	0.332	0.185	3.00E-16	TAM-lysos(	ISG15
P4HA12	1.14E-20	0.462787	0.503	0.318	3.21E-16	TAM-lysos(	P4HA1
MIR155HG	1.16E-20	0.344265	0.257	0.123	3.27E-16	TAM-lysos(	MIR155HG
SIRPA	1.88E-20	0.303989	0.476	0.293	5.28E-16	TAM-lysos(	SIRPA
GABARAP2	1.97E-20	0.32835	0.929	0.789	5.54E-16	TAM-lysos(	GABARAP
TALDO12	2.24E-20	0.377957	0.549	0.366	6.30E-16	TAM-lysos(	TALDO1
HMOX11	2.60E-20	0.926032	0.73	0.546	7.32E-16	TAM-lysos(	HMOX1
NDUFA61	3.85E-20	0.276746	0.427	0.249	1.08E-15	TAM-lysos(	NDUFA6
C19orf531	4.94E-20	0.256658	0.513	0.318	1.39E-15	TAM-lysos(	C19orf53
VEGFA3	8.95E-20	0.40477	0.291	0.151	2.52E-15	TAM-lysos(	VEGFA
B2M2	9.55E-20	0.308469	0.998	0.972	2.69E-15	TAM-lysos(	B2M
UQCR112	1.06E-19	0.376983	0.691	0.493	2.97E-15	TAM-lysos(	UQCR11
MLF2	1.09E-19	0.339012	0.462	0.282	3.08E-15	TAM-lysos(	MLF2
CYP27A1	1.49E-19	0.390566	0.282	0.146	4.20E-15	TAM-lysos(	CYP27A1
COX8A2	1.87E-19	0.388928	0.672	0.474	5.26E-15	TAM-lysos(	COX8A
NPC22	1.91E-19	0.327927	0.94	0.804	5.36E-15	TAM-lysos(	NPC2
ELOB1	1.91E-19	0.337371	0.628	0.443	5.38E-15	TAM-lysos(	ELOB
KRT10	2.25E-19	0.282033	0.474	0.294	6.32E-15	TAM-lysos(	KRT10
TXNL1	2.44E-19	0.306914	0.346	0.194	6.86E-15	TAM-lysos(	TXNL1
FCER1G2	2.49E-19	0.455729	0.905	0.816	7.02E-15	TAM-lysos(	FCER1G
NECTIN2	2.71E-19	0.269151	0.279	0.142	7.62E-15	TAM-lysos(	NECTIN2
EIF4A11	2.84E-19	0.369042	0.919	0.802	8.00E-15	TAM-lysos(	EIF4A1
PAIP21	3.10E-19	0.282063	0.496	0.313	8.71E-15	TAM-lysos(	PAIP2
EDF13	3.27E-19	0.333315	0.667	0.483	9.20E-15	TAM-lysos(	EDF1
TNFRSF141	3.66E-19	0.341751	0.282	0.148	1.03E-14	TAM-lysos(	TNFRSF14
SELENOH1	3.78E-19	0.300075	0.621	0.435	1.06E-14	TAM-lysos(	SELENOH
SAR1A	4.80E-19	0.266595	0.27	0.135	1.35E-14	TAM-lysos(	SAR1A
BANF11	5.52E-19	0.254997	0.487	0.304	1.55E-14	TAM-lysos(	BANF1
LMAN2	6.54E-19	0.31582	0.483	0.308	1.84E-14	TAM-lysos(	LMAN2
CAPG1	6.73E-19	0.451198	0.713	0.555	1.89E-14	TAM-lysos(	CAPG
COX20	7.88E-19	0.321225	0.3	0.159	2.22E-14	TAM-lysos(	COX20
APOE2	8.00E-19	0.405501	0.965	0.921	2.25E-14	TAM-lysos(	APOE
NDUFAB1	8.43E-19	0.284089	0.384	0.225	2.37E-14	TAM-lysos(	NDUFAB1
GHITM	9.31E-19	0.395343	0.437	0.272	2.62E-14	TAM-lysos(	GHITM
UGCG1	9.41E-19	0.355219	0.416	0.248	2.65E-14	TAM-lysos(	UGCG
MEA1	9.67E-19	0.276221	0.349	0.197	2.72E-14	TAM-lysos(	MEA1
PRDX2	9.75E-19	0.274981	0.317	0.172	2.74E-14	TAM-lysos(	PRDX2
SQSTM12	9.91E-19	0.347429	0.758	0.587	2.79E-14	TAM-lysos(	SQSTM1
POMP1	1.24E-18	0.367624	0.691	0.511	3.48E-14	TAM-lysos(	POMP

MCRIP1	1.34E-18	0.286394	0.31	0.167	3.76E-14	TAM-lysoso	MCRIP1
HLA-B3	1.48E-18	0.395915	0.981	0.934	4.16E-14	TAM-lysoso	HLA-B
CXCL162	1.63E-18	0.388521	0.792	0.642	4.58E-14	TAM-lysoso	CXCL16
RER1	1.94E-18	0.2893	0.446	0.275	5.46E-14	TAM-lysoso	RER1
TMED51	1.95E-18	0.294312	0.531	0.345	5.49E-14	TAM-lysoso	TMED5
HLA-C4	2.00E-18	0.401415	0.937	0.829	5.63E-14	TAM-lysoso	HLA-C
MFSD12	2.91E-18	0.278505	0.434	0.267	8.20E-14	TAM-lysoso	MFSD12
SRSF91	3.84E-18	0.268429	0.557	0.377	1.08E-13	TAM-lysoso	SRSF9
TMEM9B	4.57E-18	0.257481	0.446	0.269	1.29E-13	TAM-lysoso	TMEM9B
SPCS3	4.57E-18	0.354521	0.407	0.25	1.29E-13	TAM-lysoso	SPCS3
MBOAT7	4.68E-18	0.25478	0.254	0.129	1.32E-13	TAM-lysoso	MBOAT7
ARL6IP11	4.92E-18	0.351622	0.771	0.587	1.38E-13	TAM-lysoso	ARL6IP1
ARHGAP18	4.96E-18	0.291753	0.494	0.319	1.40E-13	TAM-lysoso	ARHGAP18
ANAPC111	5.21E-18	0.257452	0.506	0.323	1.47E-13	TAM-lysoso	ANAPC11
MYO1E2	5.41E-18	0.449615	0.54	0.356	1.52E-13	TAM-lysoso	MYO1E
SYNGR21	5.96E-18	0.336573	0.718	0.535	1.68E-13	TAM-lysoso	SYNGR2
ATP6V1B2	6.81E-18	0.332463	0.677	0.475	1.92E-13	TAM-lysoso	ATP6V1B2
DRAP11	8.92E-18	0.332675	0.623	0.438	2.51E-13	TAM-lysoso	DRAP1
ARPC52	1.05E-17	0.319815	0.672	0.477	2.96E-13	TAM-lysoso	ARPC5
IFITM34	1.19E-17	0.518868	0.64	0.464	3.35E-13	TAM-lysoso	IFITM3
HOMER3	1.19E-17	0.260742	0.291	0.156	3.36E-13	TAM-lysoso	HOMER3
PSME14	1.40E-17	0.304885	0.623	0.431	3.94E-13	TAM-lysoso	PSME1
SDF4	1.44E-17	0.300495	0.43	0.265	4.04E-13	TAM-lysoso	SDF4
WDR1	1.63E-17	0.284323	0.513	0.337	4.57E-13	TAM-lysoso	WDR1
CALM31	1.70E-17	0.334761	0.358	0.207	4.77E-13	TAM-lysoso	CALM3
PLEKHB2	1.81E-17	0.314333	0.416	0.253	5.09E-13	TAM-lysoso	PLEKHB2
MYL12A3	1.83E-17	0.345001	0.713	0.542	5.16E-13	TAM-lysoso	MYL12A
NDUFB42	2.54E-17	0.299602	0.522	0.347	7.14E-13	TAM-lysoso	NDUFB4
MLX	2.59E-17	0.263356	0.404	0.242	7.28E-13	TAM-lysoso	MLX
TWF2	2.81E-17	0.333133	0.478	0.313	7.89E-13	TAM-lysoso	TWF2
SELENOS1	3.14E-17	0.45201	0.407	0.261	8.83E-13	TAM-lysoso	SELENOS
ZNHIT11	4.28E-17	0.27422	0.353	0.201	1.21E-12	TAM-lysoso	ZNHIT1
ATP6AP1	4.69E-17	0.324505	0.614	0.443	1.32E-12	TAM-lysoso	ATP6AP1
PSMD7	4.70E-17	0.260026	0.402	0.247	1.32E-12	TAM-lysoso	PSMD7
DNAJB111	5.29E-17	0.325049	0.358	0.21	1.49E-12	TAM-lysoso	DNAJB11
IRAK1	5.60E-17	0.277467	0.3	0.167	1.57E-12	TAM-lysoso	IRAK1
DDT1	5.81E-17	0.29346	0.287	0.158	1.63E-12	TAM-lysoso	DDT
SPI12	5.98E-17	0.349173	0.838	0.686	1.68E-12	TAM-lysoso	SPI1
NAGK	6.01E-17	0.349985	0.455	0.297	1.69E-12	TAM-lysoso	NAGK
PSMA2	6.93E-17	0.252604	0.457	0.283	1.95E-12	TAM-lysoso	PSMA2
SSBP1	7.20E-17	0.267693	0.522	0.341	2.03E-12	TAM-lysoso	SSBP1
SF3B6	7.98E-17	0.261966	0.397	0.243	2.25E-12	TAM-lysoso	SF3B6
RPS284	8.05E-17	0.263833	0.979	0.878	2.26E-12	TAM-lysoso	RPS28
TMBIM1	8.35E-17	0.373683	0.427	0.272	2.35E-12	TAM-lysoso	TMBIM1
PRDX52	1.36E-16	0.281811	0.494	0.324	3.83E-12	TAM-lysoso	PRDX5
TUBA1C1	1.40E-16	0.300923	0.464	0.299	3.95E-12	TAM-lysoso	TUBA1C
JTB1	1.40E-16	0.295819	0.483	0.311	3.95E-12	TAM-lysoso	JTB

ALKBH72	1.43E-16	0.272635	0.501	0.326	4.01E-12	TAM-lysos(	ALKBH7
ATOX1	1.92E-16	0.291492	0.383	0.231	5.40E-12	TAM-lysos(	ATOX1
SOD1	1.94E-16	0.354604	0.515	0.346	5.45E-12	TAM-lysos(	SOD1
PDXK1	2.27E-16	0.274633	0.508	0.337	6.39E-12	TAM-lysos(	PDXK
PIM31	2.47E-16	0.314502	0.265	0.143	6.96E-12	TAM-lysos(	PIM3
SPG211	2.52E-16	0.257779	0.298	0.166	7.10E-12	TAM-lysos(	SPG21
LMNA1	2.77E-16	0.445068	0.272	0.149	7.80E-12	TAM-lysos(	LMNA
SEM1	3.97E-16	0.30661	0.529	0.358	1.12E-11	TAM-lysos(	SEM1
ASAP12	4.23E-16	0.275297	0.37	0.221	1.19E-11	TAM-lysos(	ASAP1
SIVA11	5.90E-16	0.347053	0.397	0.252	1.66E-11	TAM-lysos(	SIVA1
RPL273	6.26E-16	0.285653	0.875	0.71	1.76E-11	TAM-lysos(	RPL27
PSAP1	6.48E-16	0.306626	0.944	0.895	1.82E-11	TAM-lysos(	PSAP
CD1635	7.19E-16	0.336184	0.534	0.346	2.02E-11	TAM-lysos(	CD163
LY6E3	7.35E-16	0.562792	0.342	0.21	2.07E-11	TAM-lysos(	LY6E
CTDNEP1	7.60E-16	0.255559	0.48	0.308	2.14E-11	TAM-lysos(	CTDNEP1
BZW11	1.00E-15	0.359078	0.556	0.387	2.82E-11	TAM-lysos(	BZW1
RPS4Y14	1.20E-15	0.32448	0.614	0.429	3.38E-11	TAM-lysos(	RPS4Y1
CD300A	1.27E-15	0.330337	0.54	0.377	3.57E-11	TAM-lysos(	CD300A
AP2S13	1.46E-15	0.25923	0.513	0.344	4.11E-11	TAM-lysos(	AP2S1
COX6B12	1.53E-15	0.294929	0.728	0.547	4.29E-11	TAM-lysos(	COX6B1
DDOST	1.53E-15	0.280419	0.52	0.357	4.30E-11	TAM-lysos(	DDOST
MCUB	1.75E-15	0.254485	0.302	0.171	4.92E-11	TAM-lysos(	MCUB
CAST2	1.80E-15	0.307317	0.582	0.398	5.08E-11	TAM-lysos(	CAST
GNB21	2.04E-15	0.301902	0.63	0.458	5.74E-11	TAM-lysos(	GNB2
TCIRG11	2.07E-15	0.253445	0.466	0.304	5.82E-11	TAM-lysos(	TCIRG1
PSMF1	2.14E-15	0.26896	0.386	0.235	6.03E-11	TAM-lysos(	PSMF1
COX5B1	2.24E-15	0.261547	0.667	0.499	6.29E-11	TAM-lysos(	COX5B
TMEM176/	2.35E-15	0.334139	0.504	0.335	6.61E-11	TAM-lysos(	TMEM176A
PTGES31	2.59E-15	0.295904	0.663	0.486	7.29E-11	TAM-lysos(	PTGES3
OSBPL84	3.01E-15	0.439357	0.515	0.354	8.46E-11	TAM-lysos(	OSBPL8
SQOR1	3.06E-15	0.258763	0.319	0.183	8.61E-11	TAM-lysos(	SQOR
HAMP1	3.06E-15	0.771376	0.52	0.364	8.61E-11	TAM-lysos(	HAMP
MPC2	3.42E-15	0.351001	0.529	0.361	9.62E-11	TAM-lysos(	MPC2
SNRPD22	3.70E-15	0.273064	0.61	0.427	1.04E-10	TAM-lysos(	SNRPD2
VDAC2	3.99E-15	0.328756	0.571	0.402	1.12E-10	TAM-lysos(	VDAC2
PSMA5	6.18E-15	0.260234	0.347	0.209	1.74E-10	TAM-lysos(	PSMA5
CIB13	9.51E-15	0.255618	0.492	0.327	2.68E-10	TAM-lysos(	CIB1
PFDN53	1.03E-14	0.266596	0.869	0.726	2.91E-10	TAM-lysos(	PFDN5
GLUL	1.03E-14	0.610982	0.882	0.76	2.91E-10	TAM-lysos(	GLUL
ANXA111	1.08E-14	0.290257	0.469	0.318	3.03E-10	TAM-lysos(	ANXA11
TUBB1	1.12E-14	0.29114	0.642	0.476	3.15E-10	TAM-lysos(	TUBB
RABAC11	1.43E-14	0.32905	0.522	0.356	4.02E-10	TAM-lysos(	RABAC1
SND12	1.50E-14	0.254621	0.406	0.257	4.22E-10	TAM-lysos(	SND1
KRTCAP21	1.52E-14	0.28665	0.654	0.485	4.27E-10	TAM-lysos(	KRTCAP2
RPL92	1.57E-14	0.280589	0.914	0.797	4.42E-10	TAM-lysos(	RPL9
TPP1	1.61E-14	0.304529	0.51	0.342	4.54E-10	TAM-lysos(	TPP1
RPS54	1.93E-14	0.28006	0.887	0.769	5.44E-10	TAM-lysos(	RPS5

G0S22	1.95E-14	0.80686	0.277	0.159	5.49E-10	TAM-lysoso	G0S2
SDCBP1	2.24E-14	0.303972	0.794	0.64	6.29E-10	TAM-lysoso	SDCBP
EIF2S2	2.48E-14	0.254156	0.409	0.26	6.99E-10	TAM-lysoso	EIF2S2
UBE2R21	2.89E-14	0.25962	0.416	0.268	8.14E-10	TAM-lysoso	UBE2R2
HMGN11	3.32E-14	0.295118	0.637	0.462	9.33E-10	TAM-lysoso	HMGN1
BRK11	3.59E-14	0.269106	0.663	0.494	1.01E-09	TAM-lysoso	BRK1
ATP5PD2	3.74E-14	0.258882	0.536	0.372	1.05E-09	TAM-lysoso	ATP5PD
SF3B51	4.02E-14	0.331769	0.481	0.336	1.13E-09	TAM-lysoso	SF3B5
RPL212	4.18E-14	0.32443	0.892	0.745	1.18E-09	TAM-lysoso	RPL21
PEA15	4.19E-14	0.273155	0.437	0.295	1.18E-09	TAM-lysoso	PEA15
SKP1	4.47E-14	0.26829	0.714	0.548	1.26E-09	TAM-lysoso	SKP1
EGLN1	4.71E-14	0.25618	0.259	0.143	1.32E-09	TAM-lysoso	EGLN1
TMED10	5.29E-14	0.262696	0.596	0.431	1.49E-09	TAM-lysoso	TMED10
DARS	6.52E-14	0.257624	0.411	0.26	1.83E-09	TAM-lysoso	DARS
GTF2A2	6.54E-14	0.278721	0.351	0.214	1.84E-09	TAM-lysoso	GTF2A2
MYADM2	7.37E-14	0.316936	0.496	0.338	2.07E-09	TAM-lysoso	MYADM
RPS93	8.34E-14	0.261327	0.954	0.86	2.35E-09	TAM-lysoso	RPS9
CDV3	8.34E-14	0.279607	0.556	0.396	2.35E-09	TAM-lysoso	CDV3
LAPTM4A	9.08E-14	0.29206	0.683	0.512	2.55E-09	TAM-lysoso	LAPTM4A
ACADVL	1.49E-13	0.257715	0.439	0.296	4.19E-09	TAM-lysoso	ACADVL
UFM1	2.65E-13	0.273811	0.362	0.229	7.46E-09	TAM-lysoso	UFM1
FKBP82	2.71E-13	0.255882	0.536	0.378	7.62E-09	TAM-lysoso	FKBP8
UBALD22	3.15E-13	0.310404	0.422	0.278	8.86E-09	TAM-lysoso	UBALD2
C18orf32	3.67E-13	0.289738	0.407	0.265	1.03E-08	TAM-lysoso	C18orf32
COX7A2L1	3.81E-13	0.275981	0.414	0.272	1.07E-08	TAM-lysoso	COX7A2L
PHLDA11	5.52E-13	0.278724	0.291	0.171	1.55E-08	TAM-lysoso	PHLDA1
MAFB2	5.69E-13	0.326841	0.746	0.596	1.60E-08	TAM-lysoso	MAFB
MYL12B2	6.97E-13	0.277975	0.718	0.558	1.96E-08	TAM-lysoso	MYL12B
TOMM20	8.31E-13	0.261791	0.529	0.366	2.34E-08	TAM-lysoso	TOMM20
SRP141	1.08E-12	0.251157	0.772	0.612	3.04E-08	TAM-lysoso	SRP14
ANP32B3	1.69E-12	0.256223	0.538	0.377	4.77E-08	TAM-lysoso	ANP32B
AL138963.4	1.89E-12	0.441188	0.333	0.21	5.32E-08	TAM-lysoso	AL138963.4
ZNF706	1.98E-12	0.279192	0.446	0.305	5.57E-08	TAM-lysoso	ZNF706
TREM21	2.55E-12	0.509548	0.771	0.733	7.16E-08	TAM-lysoso	TREM2
NINJ1	2.61E-12	0.335452	0.656	0.508	7.34E-08	TAM-lysoso	NINJ1
CYTIP4	2.78E-12	0.297625	0.302	0.184	7.81E-08	TAM-lysoso	CYTIP
SAP18	4.84E-12	0.255929	0.589	0.429	1.36E-07	TAM-lysoso	SAP18
C1orf43	5.08E-12	0.294392	0.543	0.381	1.43E-07	TAM-lysoso	C1orf43
CCPG1	5.22E-12	0.293074	0.25	0.147	1.47E-07	TAM-lysoso	CCPG1
RTN3	5.67E-12	0.325616	0.446	0.305	1.59E-07	TAM-lysoso	RTN3
MRPS36	1.46E-11	0.252853	0.295	0.18	4.11E-07	TAM-lysoso	MRPS36
APLP22	1.52E-11	0.278675	0.637	0.477	4.27E-07	TAM-lysoso	APLP2
EIF1B1	1.66E-11	0.271079	0.494	0.348	4.68E-07	TAM-lysoso	EIF1B
CACYBP1	2.00E-11	0.321832	0.346	0.223	5.64E-07	TAM-lysoso	CACYBP
DAB21	2.04E-11	0.26876	0.381	0.252	5.74E-07	TAM-lysoso	DAB2
SAP303	2.75E-11	0.290301	0.478	0.345	7.75E-07	TAM-lysoso	SAP30
PRNP	2.87E-11	0.382132	0.471	0.335	8.09E-07	TAM-lysoso	PRNP

ADIPOR11	2.88E-11	0.279475	0.4	0.271	8.10E-07	TAM-lysos(	ADIPOR1
NPM11	4.85E-11	0.279992	0.847	0.71	1.37E-06	TAM-lysos(	NPM1
ITGB11	5.34E-11	0.295746	0.497	0.357	1.50E-06	TAM-lysos(	ITGB1
MAP1LC3B	1.82E-10	0.270505	0.573	0.423	5.12E-06	TAM-lysos(	MAP1LC3B
ACTB1	2.22E-10	0.30228	0.993	0.971	6.24E-06	TAM-lysos(	ACTB
HK2	2.42E-10	0.7041	0.342	0.248	6.80E-06	TAM-lysos(	HK2
IFI64	3.95E-10	0.556325	0.395	0.282	1.11E-05	TAM-lysos(	IFI6
STAT12	5.76E-10	0.251512	0.284	0.183	1.62E-05	TAM-lysos(	STAT1
GLRX1	2.22E-09	0.315176	0.49	0.361	6.25E-05	TAM-lysos(	GLRX
CEBPA	2.25E-09	0.30051	0.474	0.356	6.33E-05	TAM-lysos(	CEBPA
SLC2A33	3.60E-09	0.472938	0.492	0.385	0.000101	TAM-lysos(	SLC2A3
ALOX5AP1	4.97E-09	0.379622	0.852	0.746	0.00014	TAM-lysos(	ALOX5AP
PTTG1IP	8.84E-09	0.27868	0.513	0.387	0.000249	TAM-lysos(	PTTG1IP
LGMN1	2.00E-08	0.423732	0.527	0.425	0.000562	TAM-lysos(	LGMN
HIF1A-AS3	4.78E-08	0.268081	0.372	0.266	0.001345	TAM-lysos(	HIF1A-AS3
ZFAND2A1	1.88E-07	0.632408	0.296	0.2	0.005281	TAM-lysos(	ZFAND2A
CPM2	4.56E-07	0.262456	0.37	0.275	0.012835	TAM-lysos(	CPM
HSPB11	4.85E-07	0.62039	0.566	0.461	0.013649	TAM-lysos(	HSPB1
GADD45G2	5.83E-07	0.260181	0.303	0.213	0.016411	TAM-lysos(	GADD45G
SERP12	1.44E-06	0.272233	0.806	0.664	0.040583	TAM-lysos(	SERP1
PTGDS	0	5.421953	0.403	0.005	0	pDC	PTGDS
GZMB	0	4.422474	0.701	0.005	0	pDC	GZMB
JCHAIN	0	3.475483	0.628	0.001	0	pDC	JCHAIN
IRF4	0	3.453368	0.705	0.015	0	pDC	IRF4
CLIC3	0	3.442581	0.682	0.007	0	pDC	CLIC3
IGKC	0	2.859325	0.529	0.01	0	pDC	IGKC
CXCR3	0	2.756509	0.622	0.006	0	pDC	CXCR3
AREG5	0	2.688956	0.8	0.152	0	pDC	AREG
LILRA4	0	2.661	0.641	0.038	0	pDC	LILRA4
RASD1	0	2.586797	0.586	0.039	0	pDC	RASD1
TCL1A	0	2.43741	0.308	0	0	pDC	TCL1A
SEL1L31	0	2.409655	0.667	0.057	0	pDC	SEL1L3
DERL3	0	2.355903	0.656	0.029	0	pDC	DERL3
FAM160A1	0	2.11518	0.558	0.006	0	pDC	FAM160A1
MZB1	0	1.948041	0.548	0.001	0	pDC	MZB1
AFF32	0	1.945325	0.648	0.059	0	pDC	AFF3
TPM2	0	1.881107	0.522	0.018	0	pDC	TPM2
RHEX	0	1.834581	0.58	0.011	0	pDC	RHEX
LRRC26	0	1.827875	0.524	0	0	pDC	LRRC26
TSPAN13	0	1.777348	0.554	0.025	0	pDC	TSPAN13
LIME1	0	1.729459	0.554	0.009	0	pDC	LIME1
SULF21	0	1.71997	0.586	0.036	0	pDC	SULF2
SPIB	0	1.71743	0.556	0.024	0	pDC	SPIB
SMPD3	0	1.69473	0.55	0.002	0	pDC	SMPD3
SCT	0	1.675536	0.495	0.001	0	pDC	SCT
EGLN3	0	1.664583	0.493	0.013	0	pDC	EGLN3
PTPRS	0	1.620036	0.52	0.007	0	pDC	PTPRS

LTB	0	1.607589	0.401	0.026	0	pDC	LTB
MYBL2	0	1.593869	0.435	0.002	0	pDC	MYBL2
PPP1R16B	0	1.589683	0.463	0.014	0	pDC	PPP1R16B
RAB11FIP1	0	1.583879	0.582	0.085	0	pDC	RAB11FIP1
CRIP12	0	1.581613	0.51	0.032	0	pDC	CRIP1
LINC01478	0	1.559385	0.391	0.002	0	pDC	LINC01478
ZFAT	0	1.503429	0.501	0.038	0	pDC	ZFAT
CLEC4C	0	1.481849	0.484	0.001	0	pDC	CLEC4C
COBLL1	0	1.464561	0.465	0.001	0	pDC	COBLL1
NIBAN3	0	1.445805	0.478	0.002	0	pDC	NIBAN3
PTCRA	0	1.432899	0.52	0.063	0	pDC	PTCRA
MAP1A	0	1.425039	0.484	0.002	0	pDC	MAP1A
SPON2	0	1.37815	0.463	0.033	0	pDC	SPON2
TRAF4	0	1.371871	0.486	0.031	0	pDC	TRAF4
BCL11A	0	1.359151	0.465	0.008	0	pDC	BCL11A
LAMP5	0	1.351763	0.446	0.003	0	pDC	LAMP5
PHEX	0	1.310769	0.369	0.005	0	pDC	PHEX
LINC00996	0	1.286519	0.427	0.032	0	pDC	LINC00996
VASH2	0	1.278613	0.363	0.001	0	pDC	VASH2
SEMA7A	0	1.27295	0.45	0.047	0	pDC	SEMA7A
P2RY14	0	1.234723	0.437	0.013	0	pDC	P2RY14
AC023590.	0	1.226448	0.348	0.007	0	pDC	AC023590.1
VIPR2	0	1.224385	0.27	0	0	pDC	VIPR2
CUX2	0	1.176629	0.355	0.004	0	pDC	CUX2
CCDC69	0	1.150236	0.486	0.063	0	pDC	CCDC69
PPP1R14A	0	1.116112	0.301	0.003	0	pDC	PPP1R14A
CYFIP2	0	1.105787	0.418	0.011	0	pDC	CYFIP2
LINC01226	0	1.088594	0.308	0.003	0	pDC	LINC01226
PACSIN1	0	1.081352	0.414	0.001	0	pDC	PACSIN1
ZBTB18	0	1.064129	0.367	0.023	0	pDC	ZBTB18
JAML2	0	1.045524	0.439	0.041	0	pDC	JAML
VAV3	0	0.999649	0.35	0.031	0	pDC	VAV3
EPHB1	0	0.984314	0.361	0.008	0	pDC	EPHB1
LY9	0	0.979939	0.316	0.003	0	pDC	LY9
PLXNA4	0	0.970554	0.282	0.002	0	pDC	PLXNA4
IL2RG1	0	0.967167	0.397	0.041	0	pDC	IL2RG
PTPRCAP	0	0.960657	0.28	0.01	0	pDC	PTPRCAP
COL24A1	0	0.954196	0.291	0.001	0	pDC	COL24A1
SLC38A1	0	0.920279	0.363	0.019	0	pDC	SLC38A1
IGHM	0	0.917599	0.297	0.001	0	pDC	IGHM
SEPTIN1	0	0.898649	0.38	0.034	0	pDC	SEPTIN1
FLT31	0	0.884742	0.352	0.029	0	pDC	FLT3
ADAM19	0	0.876905	0.308	0.012	0	pDC	ADAM19
MDFIC	0	0.872486	0.363	0.02	0	pDC	MDFIC
FLNB	0	0.867381	0.346	0.021	0	pDC	FLNB
P2RX1	0	0.853991	0.323	0.014	0	pDC	P2RX1
MCOLN2	0	0.834947	0.306	0.019	0	pDC	MCOLN2

FUT7	0	0.832887	0.285	0.002	0	pDC		FUT7
SCAMP5	0	0.814462	0.314	0.001	0	pDC		SCAMP5
AC097375.	0	0.806215	0.312	0	0	pDC		AC097375.1
LINC02812	0	0.784651	0.314	0.012	0	pDC		LINC02812
TLR9	0	0.767435	0.308	0.002	0	pDC		TLR9
CBFA2T3	0	0.737357	0.299	0.019	0	pDC		CBFA2T3
SMIM5	0	0.729014	0.278	0.002	0	pDC		SMIM5
COL26A1	0	0.723503	0.263	0.001	0	pDC		COL26A1
ST3GAL4	0	0.721537	0.308	0.008	0	pDC		ST3GAL4
DACH1	0	0.716433	0.251	0.003	0	pDC		DACH1
DNASE1L3	0	0.712024	0.289	0.001	0	pDC		DNASE1L3
SHD	0	0.687447	0.251	0.001	0	pDC		SHD
ATP2A3	0	0.674031	0.301	0.015	0	pDC		ATP2A3
SLAMF7	0	0.667863	0.257	0.011	0	pDC		SLAMF7
SCN9A	0	0.662597	0.251	0.001	0	pDC		SCN9A
TMEM8B	0	0.660953	0.282	0.012	0	pDC		TMEM8B
AC007381.	0	0.655041	0.255	0.001	0	pDC		AC007381.1
CCDC183	0	0.654305	0.257	0.006	0	pDC		CCDC183
ADA	0	0.649017	0.287	0.011	0	pDC		ADA
CIB2	0	0.632902	0.297	0.006	0	pDC		CIB2
CD36	0	0.587148	0.255	0.015	0	pDC		CD36
PLP23	4.72E-305	2.076236	0.641	0.124	1.33E-300	pDC		PLP2
ANTXR2	7.44E-293	0.741972	0.291	0.024	2.09E-288	pDC		ANTXR2
CDKN2D	9.79E-267	0.702614	0.304	0.029	2.76E-262	pDC		CDKN2D
SEPTIN92	6.18E-262	1.134395	0.527	0.086	1.74E-257	pDC		SEPTIN9
ACAP1	3.12E-259	0.688818	0.301	0.029	8.79E-255	pDC		ACAP1
TSPYL2	1.26E-256	2.142842	0.654	0.145	3.55E-252	pDC		TSPYL2
IL3RA	6.68E-255	2.175298	0.701	0.178	1.88E-250	pDC		IL3RA
CXXC5	3.95E-248	0.979457	0.414	0.056	1.11E-243	pDC		CXXC5
NUCB2	1.60E-247	1.053168	0.459	0.069	4.51E-243	pDC		NUCB2
P2RY6	2.11E-246	1.218381	0.41	0.056	5.94E-242	pDC		P2RY6
RFTN12	2.14E-246	1.527785	0.611	0.123	6.01E-242	pDC		RFTN1
SOX4	4.73E-241	2.278935	0.622	0.136	1.33E-236	pDC		SOX4
IRF71	4.17E-240	2.991656	0.722	0.217	1.17E-235	pDC		IRF7
APP	1.09E-237	1.931644	0.648	0.155	3.05E-233	pDC		APP
DSTN1	2.17E-236	1.93803	0.656	0.165	6.11E-232	pDC		DSTN
PIM2	3.59E-233	0.905803	0.369	0.048	1.01E-228	pDC		PIM2
LPIN1	3.67E-225	1.109719	0.427	0.064	1.03E-220	pDC		LPIN1
NOTCH4	3.81E-224	0.818446	0.318	0.037	1.07E-219	pDC		NOTCH4
SIDT1	9.06E-223	0.992927	0.446	0.071	2.55E-218	pDC		SIDT1
RUBCN	5.21E-222	1.804411	0.599	0.137	1.46E-217	pDC		RUBCN
DUSP51	2.13E-221	1.980333	0.618	0.144	5.99E-217	pDC		DUSP5
CD522	9.42E-216	0.821819	0.282	0.03	2.65E-211	pDC		CD52
RBM38	1.36E-213	1.525812	0.537	0.111	3.84E-209	pDC		RBM38
CYB561A3	2.15E-211	1.303153	0.539	0.112	6.05E-207	pDC		CYB561A3
STAT4	1.05E-208	0.853842	0.299	0.034	2.96E-204	pDC		STAT4
CERS62	1.21E-208	1.66925	0.637	0.155	3.42E-204	pDC		CERS6

MPEG11	1.16E-199	1.067215	0.469	0.086	3.27E-195	pDC	MPEG1
IGF2R	2.86E-199	1.33476	0.544	0.114	8.05E-195	pDC	IGF2R
DAPK12	2.92E-197	0.923675	0.348	0.048	8.21E-193	pDC	DAPK1
S100A64	1.48E-195	1.705783	0.675	0.183	4.17E-191	pDC	S100A6
PPP1R14B1	5.57E-193	2.572114	0.665	0.217	1.57E-188	pDC	PPP1R14B
CHAF1A	8.96E-193	0.750723	0.323	0.043	2.52E-188	pDC	CHAF1A
POLB	4.26E-192	1.459155	0.537	0.12	1.20E-187	pDC	POLB
CSF2RB	5.36E-191	0.766423	0.321	0.043	1.51E-186	pDC	CSF2RB
ISG203	1.21E-187	1.459438	0.478	0.095	3.40E-183	pDC	ISG20
MED26	7.10E-180	1.233073	0.554	0.129	2.00E-175	pDC	MED26
PFKFB2	8.59E-170	0.878567	0.408	0.074	2.42E-165	pDC	PFKFB2
TXNDC5	1.02E-169	0.876634	0.399	0.072	2.88E-165	pDC	TXNDC5
CD2AP	1.41E-169	1.582811	0.656	0.195	3.96E-165	pDC	CD2AP
BCL2L11	8.72E-167	1.074513	0.478	0.101	2.45E-162	pDC	BCL2L11
ADD3	9.45E-165	0.585704	0.312	0.045	2.66E-160	pDC	ADD3
ITM2C	1.36E-164	2.228159	0.675	0.258	3.83E-160	pDC	ITM2C
MIR4435-2	4.46E-162	0.905604	0.437	0.086	1.25E-157	pDC	MIR4435-2HG
ARL4C3	1.94E-161	1.418241	0.546	0.135	5.45E-157	pDC	ARL4C
RAC24	2.03E-158	1.237575	0.546	0.143	5.72E-154	pDC	RAC2
TXN3	2.15E-158	1.657922	0.611	0.184	6.04E-154	pDC	TXN
IQGAP25	1.39E-157	1.064152	0.537	0.129	3.90E-153	pDC	IQGAP2
TGFBI3	5.97E-157	1.105988	0.594	0.156	1.68E-152	pDC	TGFBI
OPN3	9.55E-157	0.844415	0.352	0.059	2.69E-152	pDC	OPN3
ZNF10	1.69E-155	1.148441	0.42	0.085	4.74E-151	pDC	ZNF10
IDH3A	1.75E-155	0.801618	0.395	0.074	4.94E-151	pDC	IDH3A
PLAC82	8.26E-149	1.641884	0.669	0.24	2.32E-144	pDC	PLAC8
CORO71	9.38E-147	1.895666	0.713	0.286	2.64E-142	pDC	CORO7
GLT1D1	4.73E-145	0.707034	0.301	0.047	1.33E-140	pDC	GLT1D1
TNFRSF21	2.05E-141	0.901533	0.473	0.109	5.78E-137	pDC	TNFRSF21
PDE7A1	2.54E-141	1.093681	0.49	0.119	7.15E-137	pDC	PDE7A
ATP13A2	4.86E-141	0.653136	0.282	0.043	1.37E-136	pDC	ATP13A2
CNN22	2.25E-140	0.487611	0.282	0.042	6.34E-136	pDC	CNN2
C12orf75	3.34E-140	1.727295	0.65	0.247	9.40E-136	pDC	C12orf75
HYOU1	4.60E-140	0.837991	0.401	0.082	1.29E-135	pDC	HYOU1
UGCG2	3.79E-139	1.533178	0.658	0.247	1.07E-134	pDC	UGCG
SELL2	8.86E-138	1.331707	0.569	0.175	2.49E-133	pDC	SELL
SMIM31	1.88E-137	1.46721	0.584	0.184	5.30E-133	pDC	SMIM3
SERPINF11	2.04E-136	1.609857	0.741	0.341	5.73E-132	pDC	SERPINF1
N4BP2L1	2.25E-136	1.064535	0.476	0.12	6.34E-132	pDC	N4BP2L1
EZR2	1.48E-133	1.770877	0.781	0.391	4.16E-129	pDC	EZR
C12orf45	1.20E-129	0.713431	0.342	0.065	3.39E-125	pDC	C12orf45
SNHG7	1.25E-129	1.322089	0.614	0.213	3.53E-125	pDC	SNHG7
PRMT92	1.03E-127	1.142144	0.554	0.16	2.91E-123	pDC	PRMT9
GNA15	9.51E-127	1.410433	0.58	0.193	2.67E-122	pDC	GNA15
TLE53	8.29E-126	1.197895	0.594	0.202	2.33E-121	pDC	TLE5
TAGLN24	3.72E-125	1.676206	0.703	0.314	1.05E-120	pDC	TAGLN2
SLC15A4	3.40E-124	1.367567	0.652	0.241	9.56E-120	pDC	SLC15A4

CDYL	8.50E-123	1.11673	0.586	0.182	2.39E-118	pDC	CDYL
TCF4	6.04E-122	1.700775	0.741	0.368	1.70E-117	pDC	TCF4
CBX4	1.27E-121	1.111983	0.512	0.151	3.59E-117	pDC	CBX4
GAPT2	2.18E-121	0.779783	0.406	0.093	6.15E-117	pDC	GAPT
SERPINF2	4.87E-120	0.601898	0.299	0.054	1.37E-115	pDC	SERPINF2
SEC61B2	1.77E-119	2.066614	0.741	0.47	4.97E-115	pDC	SEC61B
INPP4A	7.13E-117	1.248656	0.569	0.183	2.01E-112	pDC	INPP4A
CCDC186	2.55E-116	0.736103	0.344	0.072	7.16E-112	pDC	CCDC186
SEPHS1	3.28E-116	0.70419	0.306	0.058	9.24E-112	pDC	SEPHS1
CCDC50	3.57E-116	1.448523	0.645	0.268	1.00E-111	pDC	CCDC50
HIVEP1	1.88E-115	1.035448	0.512	0.148	5.30E-111	pDC	HIVEP1
KCNAB21	3.76E-115	0.717566	0.304	0.057	1.06E-110	pDC	KCNAB2
ST6GALNA(	1.61E-114	0.557666	0.306	0.058	4.52E-110	pDC	ST6GALNAC4
IRF81	1.03E-113	1.739958	0.747	0.432	2.90E-109	pDC	IRF8
FCHSD23	2.55E-112	1.8332	0.815	0.472	7.18E-108	pDC	FCHSD2
GAB1	1.92E-111	0.978675	0.452	0.119	5.41E-107	pDC	GAB1
AGPAT5	2.01E-111	0.89246	0.42	0.106	5.65E-107	pDC	AGPAT5
SMC6	3.40E-110	0.655	0.325	0.067	9.58E-106	pDC	SMC6
FYTTD1	6.63E-108	1.046156	0.539	0.178	1.86E-103	pDC	FYTTD1
MAP4K1	1.87E-106	0.583154	0.329	0.069	5.25E-102	pDC	MAP4K1
LSP14	6.05E-106	1.145221	0.703	0.297	1.70E-101	pDC	LSP1
CXCR44	2.96E-105	1.549107	0.794	0.459	8.34E-101	pDC	CXCR4
KMO	3.56E-104	0.502981	0.293	0.056	1.00E-99	pDC	KMO
TSEN54	3.71E-103	0.585831	0.278	0.053	1.04E-98	pDC	TSEN54
NSMCE3	2.45E-102	0.986069	0.45	0.13	6.89E-98	pDC	NSMCE3
DCPS	5.00E-101	0.575465	0.316	0.068	1.41E-96	pDC	DCPS
BANP	9.49E-101	0.985877	0.51	0.162	2.67E-96	pDC	BANP
PBX3	5.95E-100	0.872155	0.446	0.127	1.67E-95	pDC	PBX3
NUP210	2.57E-99	0.528438	0.304	0.063	7.23E-95	pDC	NUP210
RPS125	5.21E-99	1.160671	0.964	0.915	1.47E-94	pDC	RPS12
ZC3HAV11	9.57E-99	1.432612	0.643	0.273	2.69E-94	pDC	ZC3HAV1
ERN12	3.36E-98	1.202008	0.531	0.177	9.46E-94	pDC	ERN1
PPM1K	9.18E-98	0.901164	0.507	0.164	2.58E-93	pDC	PPM1K
SETBP1	1.44E-97	0.707697	0.316	0.069	4.06E-93	pDC	SETBP1
MSL2	1.75E-97	0.804894	0.389	0.099	4.94E-93	pDC	MSL2
VEGFB1	4.65E-96	1.256805	0.607	0.268	1.31E-91	pDC	VEGFB
A1BG	1.33E-95	0.570781	0.348	0.083	3.74E-91	pDC	A1BG
RPS3A3	3.53E-95	1.181581	0.932	0.876	9.93E-91	pDC	RPS3A
PHB	2.16E-94	1.171266	0.522	0.191	6.08E-90	pDC	PHB
NR3C11	3.55E-94	1.701834	0.673	0.341	9.99E-90	pDC	NR3C1
RABGAP1L:	2.82E-93	1.596158	0.73	0.388	7.94E-89	pDC	RABGAP1L
RPS83	7.02E-93	0.96828	0.964	0.915	1.97E-88	pDC	RPS8
RPS285	3.60E-92	1.097295	0.921	0.878	1.01E-87	pDC	RPS28
JMY	1.10E-90	0.841697	0.31	0.072	3.09E-86	pDC	JMY
RPS15A5	9.27E-90	0.992753	0.945	0.888	2.61E-85	pDC	RPS15A
RPS234	6.47E-89	0.909708	0.938	0.909	1.82E-84	pDC	RPS23
PAXX	1.96E-88	1.32011	0.626	0.302	5.52E-84	pDC	PAXX

AHI1	4.82E-88	0.754571	0.469	0.146	1.36E-83	pDC	AHI1
YPEL51	1.24E-87	1.283553	0.692	0.366	3.48E-83	pDC	YPEL5
RRBP12	1.70E-87	1.323971	0.626	0.277	4.79E-83	pDC	RRBP1
VIM6	4.95E-87	0.897469	0.82	0.411	1.39E-82	pDC	VIM
IGFLR1	5.26E-87	1.002662	0.512	0.192	1.48E-82	pDC	IGFLR1
CORO1C1	1.15E-84	1.00697	0.584	0.232	3.24E-80	pDC	CORO1C
PRKCB2	4.23E-83	0.995965	0.59	0.241	1.19E-78	pDC	PRKCB
AP3S11	2.41E-82	0.673078	0.397	0.117	6.78E-78	pDC	AP3S1
HINT13	1.08E-81	1.271048	0.709	0.449	3.04E-77	pDC	HINT1
SNRNP25	1.92E-81	0.428475	0.299	0.071	5.40E-77	pDC	SNRNP25
IFNAR21	2.20E-81	0.694912	0.45	0.143	6.18E-77	pDC	IFNAR2
SEPTIN11	1.26E-80	0.444971	0.274	0.061	3.55E-76	pDC	SEPTIN11
RPLP04	3.16E-80	1.00421	0.921	0.869	8.90E-76	pDC	RPLP0
ATG101	4.48E-80	0.81536	0.439	0.146	1.26E-75	pDC	ATG101
NUTM2B-A	9.00E-80	0.8601	0.503	0.181	2.53E-75	pDC	NUTM2B-AS1
RPL373	1.38E-79	0.896354	0.911	0.87	3.89E-75	pDC	RPL37
ARID3B	1.46E-79	0.585212	0.251	0.053	4.09E-75	pDC	ARID3B
RIPOR21	1.46E-79	0.553528	0.251	0.053	4.11E-75	pDC	RIPOR2
PARK71	1.78E-79	1.189847	0.673	0.391	5.02E-75	pDC	PARK7
RPL63	1.09E-78	0.958846	0.913	0.858	3.08E-74	pDC	RPL6
ERP293	1.40E-78	1.161909	0.679	0.407	3.94E-74	pDC	ERP29
RPS55	1.47E-77	0.939473	0.89	0.769	4.14E-73	pDC	RPS5
ACTN41	2.49E-77	0.736913	0.45	0.148	7.01E-73	pDC	ACTN4
ETV31	3.82E-77	0.905255	0.473	0.166	1.07E-72	pDC	ETV3
RPS74	1.78E-76	0.90347	0.909	0.861	5.00E-72	pDC	RPS7
RPS274	4.38E-76	0.903054	0.936	0.878	1.23E-71	pDC	RPS27
MAPKAPK2	5.10E-76	0.984216	0.603	0.272	1.43E-71	pDC	MAPKAPK2
REPIN1	6.15E-76	0.501301	0.293	0.072	1.73E-71	pDC	REPIN1
SPCS11	7.89E-76	1.317806	0.69	0.449	2.22E-71	pDC	SPCS1
ARID3A	1.92E-75	0.730759	0.38	0.112	5.41E-71	pDC	ARID3A
RPLP13	2.38E-75	0.754834	0.975	0.948	6.71E-71	pDC	RPLP1
EIF2AK4	9.03E-75	0.908461	0.52	0.201	2.54E-70	pDC	EIF2AK4
CLN8	1.57E-74	1.035713	0.548	0.231	4.42E-70	pDC	CLN8
PTRHD11	4.14E-74	0.594528	0.412	0.13	1.16E-69	pDC	PTRHD1
MYL12A4	6.00E-74	1.255897	0.741	0.542	1.69E-69	pDC	MYL12A
STMN1	8.78E-74	0.748011	0.518	0.196	2.47E-69	pDC	STMN1
ZHX2	1.28E-72	0.923185	0.382	0.12	3.59E-68	pDC	ZHX2
RPL134	4.12E-72	0.691706	0.972	0.937	1.16E-67	pDC	RPL13
SAP130	5.37E-72	0.567593	0.255	0.059	1.51E-67	pDC	SAP130
PLD4	1.26E-71	1.160718	0.803	0.618	3.55E-67	pDC	PLD4
RNF126	3.31E-71	0.677706	0.369	0.112	9.31E-67	pDC	RNF126
PARP10	3.41E-71	0.727513	0.429	0.147	9.59E-67	pDC	PARP10
RPL114	1.01E-70	0.812603	0.943	0.901	2.84E-66	pDC	RPL11
ZDHHC17	1.01E-70	0.480364	0.291	0.074	2.85E-66	pDC	ZDHHC17
PI4KA	1.60E-70	0.879398	0.488	0.184	4.50E-66	pDC	PI4KA
CARD11	1.80E-70	0.86628	0.514	0.194	5.07E-66	pDC	CARD11
CYSTM1	1.84E-70	0.564877	0.348	0.099	5.19E-66	pDC	CYSTM1

TRAF3	3.84E-70	0.783805	0.41	0.135	1.08E-65	pDC	TRAF3
ABRACL1	4.57E-70	0.661355	0.42	0.142	1.29E-65	pDC	ABRACL
SOCS11	8.42E-70	0.722604	0.412	0.135	2.37E-65	pDC	SOCS1
CCDC138	1.25E-69	1.163884	0.389	0.125	3.51E-65	pDC	CCDC138
TENT4A	2.06E-69	0.707128	0.321	0.09	5.80E-65	pDC	TENT4A
MDN1	2.32E-69	0.573277	0.316	0.086	6.53E-65	pDC	MDN1
S100A43	3.41E-68	1.006148	0.618	0.287	9.60E-64	pDC	S100A4
RPL194	4.59E-68	0.762537	0.947	0.905	1.29E-63	pDC	RPL19
SLC7A52	7.83E-68	1.233564	0.707	0.387	2.20E-63	pDC	SLC7A5
RPL344	8.49E-68	0.805981	0.926	0.88	2.39E-63	pDC	RPL34
OGT	1.52E-67	0.811797	0.563	0.236	4.27E-63	pDC	OGT
RPS4X4	1.22E-66	0.973501	0.892	0.855	3.42E-62	pDC	RPS4X
UTRN6	2.72E-66	1.072664	0.635	0.302	7.65E-62	pDC	UTRN
SEC61G2	2.77E-66	0.864714	0.573	0.274	7.80E-62	pDC	SEC61G
RPL285	3.77E-66	0.685163	0.966	0.919	1.06E-61	pDC	RPL28
TMIGD2	7.14E-66	0.450545	0.285	0.074	2.01E-61	pDC	TMIGD2
RELL1	7.66E-66	0.794244	0.397	0.133	2.16E-61	pDC	RELL1
NCF11	2.23E-65	0.88332	0.667	0.346	6.28E-61	pDC	NCF1
RPS144	3.34E-65	0.782639	0.928	0.885	9.39E-61	pDC	RPS14
RPS27A3	7.45E-65	0.77458	0.924	0.883	2.10E-60	pDC	RPS27A
HSP90B11	9.85E-65	1.042469	0.807	0.585	2.77E-60	pDC	HSP90B1
ZNF3313	5.51E-64	0.987232	0.883	0.622	1.55E-59	pDC	ZNF331
RNF11	5.93E-64	0.661104	0.408	0.141	1.67E-59	pDC	RNF11
RASGRP2	6.05E-64	0.420336	0.278	0.073	1.70E-59	pDC	RASGRP2
RPS185	7.26E-64	0.693336	0.949	0.905	2.04E-59	pDC	RPS18
SUB12	1.03E-63	1.009847	0.692	0.449	2.89E-59	pDC	SUB1
RPLP23	2.55E-63	0.766317	0.904	0.843	7.18E-59	pDC	RPLP2
DDX241	3.12E-63	1.008031	0.752	0.49	8.77E-59	pDC	DDX24
GPR65	1.01E-62	1.124482	0.52	0.24	2.83E-58	pDC	GPR65
SLC35E1	1.93E-62	0.537827	0.34	0.103	5.42E-58	pDC	SLC35E1
CD482	2.00E-62	0.439231	0.255	0.063	5.63E-58	pDC	CD48
SRP142	3.45E-62	1.074266	0.764	0.612	9.70E-58	pDC	SRP14
RPSA4	4.29E-62	0.899746	0.862	0.718	1.21E-57	pDC	RPSA
ALG2	4.46E-62	0.566111	0.352	0.112	1.25E-57	pDC	ALG2
NOP58	5.78E-62	0.819092	0.527	0.23	1.63E-57	pDC	NOP58
RPL305	6.21E-62	0.753641	0.943	0.894	1.75E-57	pDC	RPL30
CLINT1	9.13E-62	0.753705	0.514	0.22	2.57E-57	pDC	CLINT1
RPL324	1.09E-61	0.714076	0.934	0.909	3.06E-57	pDC	RPL32
PIM32	2.19E-61	0.650321	0.406	0.142	6.16E-57	pDC	PIM3
RPS214	4.18E-61	0.79833	0.881	0.804	1.18E-56	pDC	RPS21
RPL105	5.30E-61	0.680292	0.979	0.946	1.49E-56	pDC	RPL10
KLF13	1.06E-60	0.742288	0.478	0.193	2.98E-56	pDC	KLF13
RPL7A3	1.30E-60	0.777108	0.924	0.87	3.67E-56	pDC	RPL7A
RPS34	1.84E-60	0.725085	0.938	0.877	5.19E-56	pDC	RPS3
RPS94	2.50E-60	0.746654	0.932	0.86	7.05E-56	pDC	RPS9
SSR43	2.72E-60	0.989138	0.715	0.495	7.67E-56	pDC	SSR4
CAT1	3.70E-60	0.717315	0.49	0.204	1.04E-55	pDC	CAT

MARCKSL1	7.61E-60	0.565108	0.272	0.074	2.14E-55	pDC	MARCKSL1
PPDPF4	1.12E-59	0.786471	0.554	0.254	3.16E-55	pDC	PPDPF
RPS134	1.34E-59	0.788895	0.917	0.886	3.78E-55	pDC	RPS13
BCL31	1.92E-59	0.541255	0.31	0.092	5.41E-55	pDC	BCL3
NAPSA	3.16E-59	0.692601	0.399	0.143	8.88E-55	pDC	NAPSA
EEF1A14	6.25E-59	0.791677	0.972	0.964	1.76E-54	pDC	EEF1A1
RPL223	1.34E-58	0.808674	0.849	0.754	3.78E-54	pDC	RPL22
UCP22	1.68E-58	0.94409	0.631	0.354	4.73E-54	pDC	UCP2
RNF138	2.26E-58	0.594374	0.418	0.153	6.37E-54	pDC	RNF138
CNOT6L	6.83E-58	0.588034	0.359	0.119	1.92E-53	pDC	CNOT6L
RALA3	8.43E-58	0.565801	0.359	0.118	2.37E-53	pDC	RALA
RPL10A4	9.78E-58	0.802991	0.879	0.765	2.75E-53	pDC	RPL10A
RPS215	4.85E-57	0.818972	0.911	0.852	1.37E-52	pDC	RPS2
SLC44A2	5.68E-57	0.577752	0.476	0.19	1.60E-52	pDC	SLC44A2
FBXW5	7.40E-57	0.671903	0.414	0.158	2.08E-52	pDC	FBXW5
CEP128	8.10E-57	0.518877	0.265	0.073	2.28E-52	pDC	CEP128
CDK2AP21	1.40E-56	0.635881	0.442	0.171	3.94E-52	pDC	CDK2AP2
DDIT43	1.51E-56	0.980493	0.764	0.532	4.26E-52	pDC	DDIT4
RPL394	1.75E-56	0.751326	0.924	0.883	4.93E-52	pDC	RPL39
IRF2BP21	4.15E-56	0.789889	0.573	0.274	1.17E-51	pDC	IRF2BP2
RPL310	4.74E-56	0.666046	0.93	0.868	1.33E-51	pDC	RPL3
RPS63	3.89E-55	0.638269	0.945	0.883	1.09E-50	pDC	RPS6
SLC20A1	4.39E-55	0.895579	0.471	0.198	1.24E-50	pDC	SLC20A1
SMIM14	4.48E-55	0.549335	0.408	0.15	1.26E-50	pDC	SMIM14
RPL183	9.06E-55	0.644244	0.909	0.874	2.55E-50	pDC	RPL18
RPL35A4	1.43E-54	0.772944	0.883	0.822	4.02E-50	pDC	RPL35A
MED30	2.33E-54	0.494679	0.335	0.111	6.55E-50	pDC	MED30
RAP1GAP2	7.62E-54	0.710895	0.329	0.109	2.14E-49	pDC	RAP1GAP2
IQSEC11	1.14E-53	0.490957	0.257	0.071	3.20E-49	pDC	IQSEC1
RPS6KA4	2.18E-53	0.612435	0.469	0.197	6.13E-49	pDC	RPS6KA4
TMEM141	2.36E-53	0.41989	0.314	0.1	6.63E-49	pDC	TMEM141
NADK	4.73E-53	0.409175	0.31	0.098	1.33E-48	pDC	NADK
NACA3	7.02E-53	0.791967	0.885	0.835	1.97E-48	pDC	NACA
RPL18A5	1.04E-52	0.69374	0.928	0.882	2.92E-48	pDC	RPL18A
GNA121	1.05E-52	0.798744	0.537	0.244	2.96E-48	pDC	GNA12
SPN	1.92E-52	0.558763	0.323	0.108	5.39E-48	pDC	SPN
BAG11	2.23E-52	0.603176	0.446	0.186	6.28E-48	pDC	BAG1
RNF5	7.63E-52	0.487408	0.363	0.13	2.15E-47	pDC	RNF5
PTMA6	8.01E-52	0.57968	0.947	0.906	2.25E-47	pDC	PTMA
P4HB2	1.10E-51	0.816709	0.603	0.338	3.10E-47	pDC	P4HB
CTDP1	1.14E-51	0.632908	0.263	0.078	3.21E-47	pDC	CTDP1
GRASP3	1.59E-51	0.901604	0.803	0.548	4.46E-47	pDC	GRASP
PPIB1	1.95E-51	0.788133	0.8	0.631	5.48E-47	pDC	PPIB
LDHB3	3.23E-51	0.743604	0.607	0.336	9.09E-47	pDC	LDHB
STIM1	4.57E-51	0.57857	0.306	0.098	1.29E-46	pDC	STIM1
MX12	4.76E-51	0.988331	0.501	0.229	1.34E-46	pDC	MX1
SINHCAF	9.13E-51	0.433279	0.31	0.1	2.57E-46	pDC	SINHCAF

RPL294	9.54E-51	0.620068	0.934	0.88	2.68E-46	pDC	RPL29
SLC1A4	1.87E-50	0.754079	0.357	0.128	5.25E-46	pDC	SLC1A4
TSTD1	2.07E-50	0.44794	0.301	0.096	5.82E-46	pDC	TSTD1
EEF1B22	2.22E-50	0.712615	0.879	0.761	6.26E-46	pDC	EEF1B2
PAFAH1B2	3.90E-50	0.531299	0.268	0.08	1.10E-45	pDC	PAFAH1B2
FADS1	4.52E-50	0.689337	0.31	0.103	1.27E-45	pDC	FADS1
OSTC2	1.17E-49	0.620809	0.514	0.239	3.29E-45	pDC	OSTC
GNL3	1.28E-49	0.667455	0.323	0.111	3.59E-45	pDC	GNL3
RPL23A4	1.67E-49	0.69931	0.864	0.78	4.69E-45	pDC	RPL23A
SMARCB1	2.88E-49	0.56707	0.414	0.166	8.11E-45	pDC	SMARCB1
SIVA12	5.48E-49	0.74558	0.516	0.251	1.54E-44	pDC	SIVA1
AKNA1	5.87E-49	0.614694	0.473	0.204	1.65E-44	pDC	AKNA
RPL52	7.33E-49	0.621981	0.909	0.838	2.06E-44	pDC	RPL5
ISCA1	8.58E-49	0.459975	0.268	0.082	2.41E-44	pDC	ISCA1
QDPR	1.16E-48	0.454958	0.263	0.08	3.27E-44	pDC	QDPR
RGS23	1.51E-48	1.006762	0.758	0.563	4.25E-44	pDC	RGS2
DUSP221	2.96E-48	0.625992	0.403	0.161	8.32E-44	pDC	DUSP22
RALGAPA1	3.32E-48	0.726193	0.423	0.17	9.36E-44	pDC	RALGAPA1
GPR1833	3.46E-48	0.895383	0.839	0.644	9.72E-44	pDC	GPR183
COMMD62	3.63E-48	0.862559	0.743	0.554	1.02E-43	pDC	COMMD6
OGDH2	4.56E-48	0.668797	0.369	0.139	1.28E-43	pDC	OGDH
UBE2J1	5.27E-48	0.714757	0.537	0.272	1.48E-43	pDC	UBE2J1
TIMM13	1.03E-47	0.466621	0.374	0.139	2.89E-43	pDC	TIMM13
RUNX22	2.48E-47	0.722365	0.584	0.29	6.98E-43	pDC	RUNX2
NGLY1	3.54E-47	0.61132	0.408	0.165	9.96E-43	pDC	NGLY1
KPNA2	4.23E-47	0.521739	0.386	0.152	1.19E-42	pDC	KPNA2
YWHAZ1	6.66E-47	0.754449	0.783	0.595	1.87E-42	pDC	YWHAZ
NDRG11	6.72E-47	0.505889	0.253	0.076	1.89E-42	pDC	NDRG1
UBE2S2	7.62E-47	0.661504	0.554	0.271	2.14E-42	pDC	UBE2S
KAT2B	2.87E-46	0.54771	0.297	0.101	8.08E-42	pDC	KAT2B
ATP1B35	5.47E-46	1.20847	0.728	0.498	1.54E-41	pDC	ATP1B3
ABHD15	6.04E-46	0.407349	0.265	0.083	1.70E-41	pDC	ABHD15
FAU5	1.37E-45	0.585117	0.911	0.881	3.87E-41	pDC	FAU
DCK	1.40E-45	0.628685	0.459	0.205	3.95E-41	pDC	DCK
RPL274	1.51E-45	0.677679	0.832	0.71	4.24E-41	pDC	RPL27
RACK14	1.96E-45	0.615996	0.883	0.823	5.50E-41	pDC	RACK1
PABPC12	2.31E-45	0.626141	0.904	0.844	6.49E-41	pDC	PABPC1
MAD1L1	2.39E-45	0.715777	0.403	0.165	6.73E-41	pDC	MAD1L1
TMEM268	2.65E-45	0.363656	0.285	0.093	7.46E-41	pDC	TMEM268
AHCY	3.45E-45	0.427273	0.342	0.126	9.70E-41	pDC	AHCY
SNRPN	3.61E-45	0.366866	0.295	0.097	1.02E-40	pDC	SNRPN
RPL144	7.16E-45	0.586449	0.9	0.855	2.02E-40	pDC	RPL14
RPS112	7.63E-45	0.657682	0.826	0.693	2.15E-40	pDC	RPS11
MALT13	9.90E-45	0.873589	0.533	0.263	2.79E-40	pDC	MALT1
PMEPA12	4.36E-44	0.868651	0.601	0.355	1.23E-39	pDC	PMEPA1
OFD1	5.69E-44	0.715995	0.49	0.233	1.60E-39	pDC	OFD1
SND13	6.35E-44	0.616198	0.524	0.257	1.79E-39	pDC	SND1

ANKRD112	7.66E-44	0.884084	0.773	0.537	2.16E-39	pDC	ANKRD11
PRXL2A	8.46E-44	0.433006	0.276	0.091	2.38E-39	pDC	PRXL2A
EEF22	1.11E-43	0.650495	0.864	0.764	3.14E-39	pDC	EEF2
GORASP2	1.54E-43	0.435715	0.342	0.127	4.35E-39	pDC	GORASP2
DNAJB9	1.85E-43	0.454625	0.321	0.116	5.21E-39	pDC	DNAJB9
RPL37A3	2.21E-43	0.631335	0.879	0.78	6.21E-39	pDC	RPL37A
RPL93	2.47E-43	0.702043	0.866	0.797	6.94E-39	pDC	RPL9
CHCHD23	5.66E-43	0.801571	0.732	0.574	1.59E-38	pDC	CHCHD2
GLCCI1	5.88E-43	0.546774	0.391	0.159	1.66E-38	pDC	GLCCI1
PARVB1	1.65E-42	0.649505	0.567	0.293	4.64E-38	pDC	PARVB
GRSF1	2.78E-42	0.573238	0.423	0.189	7.83E-38	pDC	GRSF1
RPL45	6.06E-42	0.600214	0.887	0.738	1.71E-37	pDC	RPL4
THEMIS21	7.12E-42	0.715598	0.641	0.398	2.00E-37	pDC	THEMIS2
SPINT21	7.89E-42	0.580522	0.552	0.291	2.22E-37	pDC	SPINT2
EEF1G4	1.16E-41	0.628788	0.832	0.694	3.25E-37	pDC	EEF1G
STRBP	1.44E-41	0.590777	0.327	0.121	4.05E-37	pDC	STRBP
NIN	1.90E-41	0.528952	0.391	0.162	5.33E-37	pDC	NIN
ZNF791	1.95E-41	0.542304	0.327	0.121	5.48E-37	pDC	ZNF791
RPL153	2.02E-41	0.536893	0.919	0.857	5.68E-37	pDC	RPL15
STK17B4	2.41E-41	0.604498	0.699	0.4	6.78E-37	pDC	STK17B
RPL414	2.64E-41	1.142303	0.894	0.819	7.43E-37	pDC	RPL41
MRPS6	4.09E-41	0.340347	0.293	0.101	1.15E-36	pDC	MRPS6
TAP1	4.12E-41	0.484114	0.295	0.106	1.16E-36	pDC	TAP1
NPIPB5	8.89E-41	0.579874	0.253	0.082	2.50E-36	pDC	NPIPB5
RPL354	9.98E-41	0.581748	0.881	0.798	2.81E-36	pDC	RPL35
RPL72	1.04E-40	0.682175	0.758	0.616	2.92E-36	pDC	RPL7
RPS254	1.27E-40	0.555962	0.898	0.849	3.56E-36	pDC	RPS25
HNRNPA11	1.81E-40	0.685436	0.788	0.671	5.08E-36	pDC	HNRNPA1
DANCR	1.86E-40	0.388629	0.31	0.111	5.22E-36	pDC	DANCR
RPS193	2.64E-40	0.480863	0.949	0.922	7.44E-36	pDC	RPS19
NR4A33	5.79E-40	0.762293	0.851	0.632	1.63E-35	pDC	NR4A3
GUK12	8.02E-40	0.728294	0.637	0.404	2.26E-35	pDC	GUK1
DAAM1	1.03E-39	0.474779	0.318	0.119	2.89E-35	pDC	DAAM1
RUBCNL	1.15E-39	0.455985	0.346	0.133	3.23E-35	pDC	RUBCNL
SUSD1	1.28E-39	0.417528	0.31	0.113	3.61E-35	pDC	SUSD1
PPIA2	1.36E-39	0.568609	0.892	0.813	3.82E-35	pDC	PPIA
FOSL23	1.63E-39	0.691906	0.454	0.212	4.57E-35	pDC	FOSL2
UBL7	1.78E-39	0.493792	0.365	0.151	5.01E-35	pDC	UBL7
EIF4A31	2.68E-39	0.76925	0.633	0.391	7.53E-35	pDC	EIF4A3
SEPTIN62	4.95E-39	0.606371	0.556	0.3	1.39E-34	pDC	SEPTIN6
VDAC3	5.46E-39	0.417359	0.365	0.148	1.54E-34	pDC	VDAC3
CD554	6.16E-39	0.592289	0.624	0.353	1.73E-34	pDC	CD55
RPL124	6.82E-39	0.519661	0.936	0.892	1.92E-34	pDC	RPL12
RPL13A3	6.82E-39	0.634394	0.845	0.745	1.92E-34	pDC	RPL13A
S100A104	1.47E-38	0.304011	0.306	0.109	4.13E-34	pDC	S100A10
EEF1D4	1.55E-38	0.655379	0.805	0.694	4.37E-34	pDC	EEF1D
CSNK1E	1.81E-38	0.465874	0.323	0.125	5.10E-34	pDC	CSNK1E

USP363	2.52E-38	0.789098	0.529	0.28	7.08E-34	pDC	USP36
CD47	2.52E-38	0.552012	0.401	0.182	7.09E-34	pDC	CD47
RPS154	2.64E-38	0.458999	0.934	0.894	7.42E-34	pDC	RPS15
ZFP36L22	2.72E-38	0.714906	0.932	0.843	7.65E-34	pDC	ZFP36L2
ESYT22	4.20E-38	0.557272	0.425	0.192	1.18E-33	pDC	ESYT2
SLC9A7	5.07E-38	0.467626	0.261	0.09	1.43E-33	pDC	SLC9A7
CREB3L2	5.36E-38	0.621959	0.429	0.199	1.51E-33	pDC	CREB3L2
HVCN1	1.18E-37	0.46311	0.346	0.14	3.31E-33	pDC	HVCN1
SELENOS2	2.23E-37	0.629153	0.503	0.26	6.26E-33	pDC	SELENOS
CBLB1	2.47E-37	0.404129	0.27	0.094	6.95E-33	pDC	CBLB
TXNL4A1	2.68E-37	0.580142	0.522	0.278	7.55E-33	pDC	TXNL4A
ATP5F1E3	2.83E-37	0.59365	0.845	0.752	7.98E-33	pDC	ATP5F1E
RPL36A3	5.01E-37	0.578423	0.858	0.763	1.41E-32	pDC	RPL36A
DNAJC1	5.88E-37	0.523662	0.391	0.166	1.65E-32	pDC	DNAJC1
ARPC5L	7.11E-37	0.468811	0.331	0.132	2.00E-32	pDC	ARPC5L
ITGAE	7.32E-37	0.415624	0.299	0.112	2.06E-32	pDC	ITGAE
RPL243	7.80E-37	0.550363	0.873	0.816	2.20E-32	pDC	RPL24
ODC11	1.02E-36	0.568439	0.537	0.286	2.86E-32	pDC	ODC1
RNF1493	1.51E-36	0.682382	0.769	0.563	4.24E-32	pDC	RNF149
IL16	1.69E-36	0.630879	0.429	0.209	4.74E-32	pDC	IL16
MAN2B1	3.94E-36	0.665432	0.667	0.432	1.11E-31	pDC	MAN2B1
FBXO34	5.64E-36	0.629931	0.361	0.154	1.59E-31	pDC	FBXO34
RPL264	1.25E-35	0.485112	0.913	0.887	3.53E-31	pDC	RPL26
HERPUD13	1.44E-35	0.709585	0.86	0.704	4.04E-31	pDC	HERPUD1
ZMYM21	1.74E-35	0.647814	0.512	0.267	4.90E-31	pDC	ZMYM2
AHNAK5	1.77E-35	0.458362	0.427	0.188	4.97E-31	pDC	AHNAK
NRP1	1.83E-35	0.488692	0.299	0.115	5.16E-31	pDC	NRP1
SFT2D2	2.43E-35	0.432694	0.416	0.19	6.83E-31	pDC	SFT2D2
PDIA41	2.64E-35	0.422162	0.35	0.145	7.44E-31	pDC	PDIA4
PPP6R1	3.42E-35	0.41631	0.338	0.139	9.63E-31	pDC	PPP6R1
SRPRA	4.51E-35	0.523333	0.497	0.255	1.27E-30	pDC	SRPRA
HIST1H1C	4.57E-35	0.653938	0.357	0.151	1.28E-30	pDC	HIST1H1C
RPL313	5.64E-35	0.666743	0.762	0.67	1.59E-30	pDC	RPL31
MAP3K23	6.11E-35	0.672246	0.635	0.397	1.72E-30	pDC	MAP3K2
EIF4EBP3	8.56E-35	0.315284	0.261	0.093	2.41E-30	pDC	EIF4EBP3
RBIS1	9.50E-35	0.5008	0.516	0.279	2.67E-30	pDC	RBIS
PAIP1	1.16E-34	0.351289	0.335	0.136	3.26E-30	pDC	PAIP1
FAM177A1	1.18E-34	0.748911	0.374	0.175	3.32E-30	pDC	FAM177A1
ZDHHC4	1.28E-34	0.309265	0.268	0.097	3.61E-30	pDC	ZDHHC4
LINC-PINT	2.31E-34	0.452675	0.265	0.098	6.51E-30	pDC	LINC-PINT
ASPH	2.44E-34	0.464515	0.312	0.125	6.86E-30	pDC	ASPH
AKAP133	2.67E-34	0.717002	0.879	0.78	7.52E-30	pDC	AKAP13
MNAT1	3.96E-34	0.403022	0.263	0.095	1.12E-29	pDC	MNAT1
CCDC57	4.69E-34	0.367763	0.293	0.114	1.32E-29	pDC	CCDC57
BID1	5.33E-34	0.485808	0.488	0.243	1.50E-29	pDC	BID
PPM1G	5.70E-34	0.454919	0.435	0.212	1.61E-29	pDC	PPM1G
LSM5	6.60E-34	0.358487	0.359	0.153	1.86E-29	pDC	LSM5

PPP1R2	2.38E-33	0.627627	0.514	0.276	6.70E-29	pDC	PPP1R2
ANXA112	3.70E-33	0.622231	0.537	0.318	1.04E-28	pDC	ANXA11
SRSF6	4.25E-33	0.446134	0.397	0.183	1.19E-28	pDC	SRSF6
CDK17	6.43E-33	0.629842	0.325	0.138	1.81E-28	pDC	CDK17
GRAMD4	6.43E-33	0.566574	0.35	0.153	1.81E-28	pDC	GRAMD4
MYD881	7.38E-33	0.434298	0.293	0.115	2.08E-28	pDC	MYD88
TMED3	8.74E-33	0.28603	0.255	0.092	2.46E-28	pDC	TMED3
REXO2	1.02E-32	0.332245	0.253	0.093	2.88E-28	pDC	REXO2
AP1S23	1.50E-32	0.474503	0.425	0.208	4.23E-28	pDC	AP1S2
CD164	1.83E-32	0.685432	0.62	0.423	5.15E-28	pDC	CD164
BTAF12	2.63E-32	0.577703	0.514	0.274	7.39E-28	pDC	BTAF1
DGKZ	3.25E-32	0.299382	0.301	0.121	9.15E-28	pDC	DGKZ
CREM2	3.63E-32	0.822143	0.694	0.521	1.02E-27	pDC	CREM
RAPGEF21	7.02E-32	0.876334	0.469	0.251	1.98E-27	pDC	RAPGEF2
RPS265	7.39E-32	0.730869	0.909	0.814	2.08E-27	pDC	RPS26
PMF1	7.46E-32	0.319246	0.276	0.106	2.10E-27	pDC	PMF1
ANKRD121	9.11E-32	0.623785	0.58	0.336	2.56E-27	pDC	ANKRD12
AGPAT2	1.04E-31	0.362491	0.295	0.117	2.94E-27	pDC	AGPAT2
RNASE61	1.30E-31	0.614655	0.569	0.352	3.65E-27	pDC	RNASE6
GAS6	1.33E-31	0.543304	0.49	0.266	3.74E-27	pDC	GAS6
POLR2A1	1.35E-31	0.631726	0.488	0.265	3.81E-27	pDC	POLR2A
SNHG293	1.98E-31	0.65041	0.709	0.55	5.57E-27	pDC	SNHG29
SEC11C1	2.21E-31	0.387113	0.325	0.138	6.22E-27	pDC	SEC11C
TEX2	2.33E-31	0.306001	0.289	0.113	6.55E-27	pDC	TEX2
PPP2R5C1	2.59E-31	0.463698	0.403	0.191	7.28E-27	pDC	PPP2R5C
RPS293	3.58E-31	0.634695	0.779	0.653	1.01E-26	pDC	RPS29
ZNF7061	3.65E-31	0.586923	0.524	0.305	1.03E-26	pDC	ZNF706
UBALD23	3.75E-31	0.516804	0.499	0.277	1.06E-26	pDC	UBALD2
BRD4	5.03E-31	0.572275	0.62	0.376	1.41E-26	pDC	BRD4
PSMA21	5.04E-31	0.487593	0.516	0.283	1.42E-26	pDC	PSMA2
CHMP1B2	6.11E-31	0.725149	0.497	0.271	1.72E-26	pDC	CHMP1B
LINC02245	6.35E-31	0.374654	0.386	0.174	1.79E-26	pDC	LINC02245
NHP2	6.43E-31	0.440442	0.431	0.213	1.81E-26	pDC	NHP2
SP1101	1.10E-30	0.620346	0.544	0.317	3.11E-26	pDC	SP110
HNRNPUL1	1.92E-30	0.59	0.603	0.374	5.40E-26	pDC	HNRNPUL1
LRCH3	2.28E-30	0.536559	0.323	0.14	6.42E-26	pDC	LRCH3
RPS243	2.39E-30	0.416799	0.953	0.901	6.72E-26	pDC	RPS24
DNAJC41	2.82E-30	0.417918	0.42	0.207	7.94E-26	pDC	DNAJC4
HLA-DQA2:	2.97E-30	0.57923	0.425	0.214	8.34E-26	pDC	HLA-DQA2
RABL6	3.12E-30	0.439922	0.335	0.148	8.79E-26	pDC	RABL6
ST14	3.46E-30	0.374101	0.323	0.137	9.73E-26	pDC	ST14
RPL213	3.61E-30	0.698718	0.809	0.746	1.02E-25	pDC	RPL21
DTNBP1	3.69E-30	0.447687	0.304	0.128	1.04E-25	pDC	DTNBP1
SMCHD14	5.52E-30	0.784488	0.497	0.277	1.55E-25	pDC	SMCHD1
C17orf49	5.56E-30	0.40944	0.41	0.202	1.56E-25	pDC	C17orf49
EDF14	9.58E-30	0.54983	0.684	0.483	2.70E-25	pDC	EDF1
GATAD2A	1.27E-29	0.507706	0.333	0.148	3.58E-25	pDC	GATAD2A

VAMP84	2.32E-29	0.613123	0.694	0.524	6.54E-25	pDC	VAMP8
TNIP11	2.93E-29	0.350844	0.301	0.127	8.24E-25	pDC	TNIP1
OST42	5.40E-29	0.568127	0.658	0.476	1.52E-24	pDC	OST4
ADGRE52	5.67E-29	0.417254	0.323	0.139	1.59E-24	pDC	ADGRE5
JTB2	5.67E-29	0.50125	0.529	0.311	1.60E-24	pDC	JTB
SERF23	5.91E-29	0.455095	0.864	0.802	1.66E-24	pDC	SERF2
CPNE3	6.54E-29	0.405274	0.359	0.167	1.84E-24	pDC	CPNE3
PQBP1	8.19E-29	0.401234	0.397	0.193	2.30E-24	pDC	PQBP1
UNC93B1	8.61E-29	0.567812	0.59	0.375	2.42E-24	pDC	UNC93B1
ALKBH5	9.62E-29	0.353168	0.299	0.126	2.71E-24	pDC	ALKBH5
RANBP22	1.01E-28	0.622542	0.766	0.569	2.85E-24	pDC	RANBP2
SEC61A11	1.60E-28	0.468832	0.363	0.171	4.51E-24	pDC	SEC61A1
TM9SF21	1.96E-28	0.487704	0.59	0.367	5.51E-24	pDC	TM9SF2
RNASEH2B	2.12E-28	0.320472	0.344	0.155	5.96E-24	pDC	RNASEH2B
STAT13	2.26E-28	0.640711	0.367	0.182	6.35E-24	pDC	STAT1
WDFY41	2.66E-28	0.556312	0.503	0.282	7.47E-24	pDC	WDFY4
RPL174	2.70E-28	0.484127	0.881	0.836	7.60E-24	pDC	RPL17
NME4	2.83E-28	0.301574	0.335	0.151	7.95E-24	pDC	NME4
H2AFV2	2.85E-28	0.494186	0.507	0.296	8.01E-24	pDC	H2AFV
SNRPD23	3.59E-28	0.520099	0.631	0.427	1.01E-23	pDC	SNRPD2
EDEM1	4.05E-28	0.523701	0.335	0.153	1.14E-23	pDC	EDEM1
GLO11	4.85E-28	0.32411	0.306	0.132	1.36E-23	pDC	GLO1
MEF2D	5.27E-28	0.410685	0.382	0.185	1.48E-23	pDC	MEF2D
RPL232	7.88E-28	0.599405	0.707	0.546	2.22E-23	pDC	RPL23
STX17-AS1	8.24E-28	0.58074	0.658	0.414	2.32E-23	pDC	STX17-AS1
CYTIP5	8.42E-28	0.42074	0.384	0.183	2.37E-23	pDC	CYTIP
ATP5MG3	9.49E-28	0.522989	0.745	0.614	2.67E-23	pDC	ATP5MG
ARHGEF71	1.09E-27	0.709904	0.514	0.301	3.06E-23	pDC	ARHGEF7
TRAM11	2.24E-27	0.420471	0.488	0.274	6.30E-23	pDC	TRAM1
COX7A2L2	2.60E-27	0.403363	0.486	0.272	7.31E-23	pDC	COX7A2L
RPS162	3.06E-27	0.44141	0.892	0.829	8.61E-23	pDC	RPS16
DIPK2A	3.08E-27	0.431099	0.359	0.171	8.66E-23	pDC	DIPK2A
COX7A22	3.27E-27	0.462957	0.584	0.374	9.20E-23	pDC	COX7A2
EIF3M1	4.37E-27	0.48484	0.548	0.328	1.23E-22	pDC	EIF3M
HEBP21	4.91E-27	0.298786	0.278	0.116	1.38E-22	pDC	HEBP2
PSME15	5.74E-27	0.531926	0.62	0.431	1.62E-22	pDC	PSME1
SSR32	8.69E-27	0.485567	0.567	0.357	2.44E-22	pDC	SSR3
ANAPC161	9.53E-27	0.482145	0.601	0.39	2.68E-22	pDC	ANAPC16
MPG	1.08E-26	0.295185	0.306	0.134	3.03E-22	pDC	MPG
RIPOR1	1.34E-26	0.375102	0.287	0.123	3.77E-22	pDC	RIPOR1
CUX13	1.42E-26	0.573236	0.622	0.397	3.99E-22	pDC	CUX1
SUPT5H	2.19E-26	0.380718	0.389	0.191	6.15E-22	pDC	SUPT5H
RPS172	4.84E-26	0.547217	0.662	0.466	1.36E-21	pDC	RPS17
NFATC2IP	7.00E-26	0.391868	0.278	0.12	1.97E-21	pDC	NFATC2IP
RPS10	1.19E-25	0.315535	0.27	0.114	3.36E-21	pDC	RPS10
PCBP2	1.57E-25	0.498387	0.59	0.395	4.42E-21	pDC	PCBP2
NAPA	2.30E-25	0.460544	0.573	0.358	6.48E-21	pDC	NAPA

DDB1	3.09E-25	0.35938	0.355	0.172	8.70E-21	pDC	DDB1
TTC31	3.74E-25	0.516361	0.444	0.247	1.05E-20	pDC	TTC3
UBL53	5.03E-25	0.470768	0.669	0.467	1.41E-20	pDC	UBL5
HPCAL12	6.55E-25	0.410512	0.295	0.133	1.84E-20	pDC	HPCAL1
CCND31	8.21E-25	0.414322	0.584	0.347	2.31E-20	pDC	CCND3
RPS201	1.38E-24	0.514564	0.671	0.494	3.89E-20	pDC	RPS20
ARHGAP17	1.83E-24	0.398511	0.338	0.161	5.14E-20	pDC	ARHGAP17
PHYKPL	1.86E-24	0.395313	0.397	0.204	5.24E-20	pDC	PHYKPL
HINT2	1.92E-24	0.271236	0.287	0.127	5.41E-20	pDC	HINT2
APRT3	2.33E-24	0.452324	0.577	0.37	6.54E-20	pDC	APRT
RBBP41	2.96E-24	0.378722	0.446	0.241	8.32E-20	pDC	RBBP4
SAP181	3.23E-24	0.434032	0.622	0.429	9.09E-20	pDC	SAP18
CD991	3.53E-24	0.521731	0.732	0.586	9.92E-20	pDC	CD99
PDE4B3	3.55E-24	0.607606	0.737	0.502	9.99E-20	pDC	PDE4B
EIF3L1	3.88E-24	0.485482	0.62	0.428	1.09E-19	pDC	EIF3L
COQ7	3.99E-24	0.388345	0.289	0.129	1.12E-19	pDC	COQ7
RAP1GDS1	4.62E-24	0.367513	0.318	0.148	1.30E-19	pDC	RAP1GDS1
MANF1	5.61E-24	0.286495	0.304	0.139	1.58E-19	pDC	MANF
MOB1B	5.71E-24	0.578527	0.403	0.218	1.61E-19	pDC	MOB1B
MED10	6.96E-24	0.292576	0.325	0.151	1.96E-19	pDC	MED10
DCTN31	7.47E-24	0.270018	0.323	0.151	2.10E-19	pDC	DCTN3
DDX18	1.01E-23	0.483824	0.524	0.319	2.84E-19	pDC	DDX18
KCTD5	1.06E-23	0.274629	0.295	0.132	2.98E-19	pDC	KCTD5
TMEM258:	1.12E-23	0.433169	0.58	0.38	3.15E-19	pDC	TMEM258
AKAP91	1.28E-23	0.480164	0.471	0.272	3.60E-19	pDC	AKAP9
TBC1D8	1.37E-23	0.523798	0.372	0.19	3.84E-19	pDC	TBC1D8
DBNL	1.58E-23	0.473957	0.48	0.281	4.44E-19	pDC	DBNL
CENPC	1.59E-23	0.361857	0.365	0.181	4.48E-19	pDC	CENPC
SNAP29	1.67E-23	0.316857	0.27	0.118	4.69E-19	pDC	SNAP29
ZNF787	1.81E-23	0.350058	0.263	0.115	5.09E-19	pDC	ZNF787
SSR25	2.07E-23	0.441221	0.584	0.397	5.83E-19	pDC	SSR2
TGIF2	2.74E-23	0.344382	0.306	0.143	7.72E-19	pDC	TGIF2
TRABD	3.48E-23	0.467229	0.48	0.282	9.79E-19	pDC	TRABD
CCDC1071	4.23E-23	0.318825	0.299	0.138	1.19E-18	pDC	CCDC107
SAMHD12	4.42E-23	0.440908	0.696	0.497	1.24E-18	pDC	SAMHD1
ADI1	6.94E-23	0.320538	0.344	0.171	1.95E-18	pDC	ADI1
MX21	7.04E-23	0.326118	0.323	0.153	1.98E-18	pDC	MX2
UBA524	8.46E-23	0.404782	0.856	0.776	2.38E-18	pDC	UBA52
IKZF11	1.09E-22	0.455625	0.645	0.427	3.07E-18	pDC	IKZF1
GLG1	1.22E-22	0.44882	0.431	0.238	3.44E-18	pDC	GLG1
LINC01374	1.28E-22	0.582613	0.503	0.291	3.60E-18	pDC	LINC01374
PECAM11	1.38E-22	0.37689	0.355	0.179	3.89E-18	pDC	PECAM1
UXT2	1.53E-22	0.461326	0.52	0.326	4.30E-18	pDC	UXT
NDUFA71	1.57E-22	0.317733	0.361	0.188	4.42E-18	pDC	NDUFA7
CLASP21	1.83E-22	0.431391	0.416	0.226	5.16E-18	pDC	CLASP2
GGA2	1.89E-22	0.350306	0.365	0.191	5.33E-18	pDC	GGA2
GTF3A2	2.14E-22	0.391901	0.493	0.291	6.02E-18	pDC	GTF3A

PDPK1	2.27E-22	0.292041	0.263	0.117	6.38E-18	pDC	PDPK1
SPTY2D1	2.28E-22	0.394671	0.265	0.12	6.42E-18	pDC	SPTY2D1
IDS1	2.32E-22	0.50317	0.622	0.423	6.52E-18	pDC	IDS
ATP6V1G1:	2.33E-22	0.457903	0.597	0.417	6.56E-18	pDC	ATP6V1G1
TOX4	2.96E-22	0.312998	0.338	0.168	8.33E-18	pDC	TOX4
TRIM22	3.52E-22	0.549833	0.569	0.387	9.91E-18	pDC	TRIM22
SNX93	3.84E-22	0.406464	0.758	0.548	1.08E-17	pDC	SNX9
TATDN3	5.18E-22	0.31332	0.295	0.139	1.46E-17	pDC	TATDN3
COX5A3	5.48E-22	0.539431	0.592	0.415	1.54E-17	pDC	COX5A
ARID2	8.83E-22	0.369223	0.348	0.175	2.48E-17	pDC	ARID2
HIST1H4C1	1.00E-21	0.460053	0.529	0.323	2.82E-17	pDC	HIST1H4C
JOSD1	1.53E-21	0.385744	0.327	0.164	4.29E-17	pDC	JOSD1
MYL63	2.38E-21	0.367182	0.862	0.772	6.71E-17	pDC	MYL6
SET1	2.90E-21	0.442581	0.592	0.399	8.17E-17	pDC	SET
ALYREF	3.30E-21	0.315053	0.378	0.2	9.30E-17	pDC	ALYREF
RPL27A3	3.71E-21	0.483917	0.722	0.582	1.05E-16	pDC	RPL27A
ADD1	5.36E-21	0.36055	0.418	0.232	1.51E-16	pDC	ADD1
TOMM72	6.19E-21	0.479365	0.711	0.56	1.74E-16	pDC	TOMM7
COX7C2	6.54E-21	0.48816	0.709	0.599	1.84E-16	pDC	COX7C
SRSF71	6.59E-21	0.400758	0.794	0.63	1.85E-16	pDC	SRSF7
SPCS31	7.20E-21	0.391391	0.435	0.25	2.03E-16	pDC	SPCS3
EIF3F3	7.47E-21	0.435497	0.682	0.505	2.10E-16	pDC	EIF3F
SELENOF	8.73E-21	0.391695	0.512	0.318	2.46E-16	pDC	SELENOF
PGD2	8.88E-21	0.33222	0.382	0.208	2.50E-16	pDC	PGD
NDUFB6	8.92E-21	0.360381	0.386	0.21	2.51E-16	pDC	NDUFB6
COX4I13	9.46E-21	0.38607	0.826	0.74	2.66E-16	pDC	COX4I1
ANKLE2	9.74E-21	0.368707	0.323	0.16	2.74E-16	pDC	ANKLE2
SNRPF	1.05E-20	0.305864	0.376	0.196	2.96E-16	pDC	SNRPF
TBCA1	1.09E-20	0.403022	0.539	0.344	3.06E-16	pDC	TBCA
TLK1	1.11E-20	0.360565	0.297	0.144	3.13E-16	pDC	TLK1
TMA71	1.12E-20	0.483316	0.701	0.552	3.15E-16	pDC	TMA7
FIP1L1	1.19E-20	0.330263	0.327	0.163	3.33E-16	pDC	FIP1L1
SRSF92	1.19E-20	0.411792	0.567	0.377	3.35E-16	pDC	SRSF9
TMED21	1.24E-20	0.333201	0.433	0.247	3.49E-16	pDC	TMED2
TNRC6B2	1.25E-20	0.413971	0.499	0.302	3.51E-16	pDC	TNRC6B
SERINC3	1.40E-20	0.329976	0.456	0.262	3.95E-16	pDC	SERINC3
NARF	1.43E-20	0.403709	0.257	0.119	4.01E-16	pDC	NARF
OSBP	1.49E-20	0.309847	0.274	0.128	4.20E-16	pDC	OSBP
NSMCE1	1.50E-20	0.350739	0.323	0.163	4.21E-16	pDC	NSMCE1
FBL1	1.50E-20	0.318029	0.352	0.185	4.22E-16	pDC	FBL
CTBP1	1.59E-20	0.360582	0.386	0.212	4.48E-16	pDC	CTBP1
HMGN12	1.70E-20	0.424324	0.633	0.462	4.78E-16	pDC	HMGN1
APEX1	1.74E-20	0.404772	0.499	0.313	4.90E-16	pDC	APEX1
NDUFS52	1.76E-20	0.459791	0.569	0.388	4.95E-16	pDC	NDUFS5
ZNF428	1.88E-20	0.301516	0.325	0.164	5.29E-16	pDC	ZNF428
MDM4	2.22E-20	0.414224	0.369	0.198	6.23E-16	pDC	MDM4
ATAD2B	3.15E-20	0.33993	0.333	0.17	8.86E-16	pDC	ATAD2B

ARMH12	3.34E-20	0.308843	0.389	0.204	9.40E-16	pDC	ARMH1
SNU13	4.97E-20	0.344529	0.537	0.339	1.40E-15	pDC	SNU13
NUDT22	5.43E-20	0.29876	0.306	0.151	1.53E-15	pDC	NUDT22
NDUFB111	5.93E-20	0.407993	0.594	0.415	1.67E-15	pDC	NDUFB11
PFN15	6.08E-20	0.334796	0.904	0.827	1.71E-15	pDC	PFN1
PSMA63	7.02E-20	0.438685	0.495	0.313	1.98E-15	pDC	PSMA6
SCARB21	8.53E-20	0.341375	0.401	0.223	2.40E-15	pDC	SCARB2
ATP5MPL2	9.17E-20	0.360306	0.505	0.31	2.58E-15	pDC	ATP5MPL
STAT2	9.92E-20	0.35577	0.268	0.127	2.79E-15	pDC	STAT2
NDUFA9	9.95E-20	0.279718	0.276	0.13	2.80E-15	pDC	NDUFA9
LSM7	1.02E-19	0.394156	0.503	0.314	2.88E-15	pDC	LSM7
H2AFX	1.04E-19	0.371197	0.372	0.205	2.92E-15	pDC	H2AFX
CD371	1.18E-19	0.450647	0.752	0.66	3.31E-15	pDC	CD37
TOMM5	1.28E-19	0.320137	0.352	0.188	3.61E-15	pDC	TOMM5
UNC119	1.46E-19	0.265054	0.272	0.129	4.10E-15	pDC	UNC119
PTPN21	2.40E-19	0.379336	0.456	0.278	6.76E-15	pDC	PTPN2
RSL24D11	2.96E-19	0.365614	0.425	0.247	8.33E-15	pDC	RSL24D1
UBE2E3	3.66E-19	0.377851	0.382	0.217	1.03E-14	pDC	UBE2E3
BAIAP2	4.61E-19	0.382993	0.384	0.215	1.30E-14	pDC	BAIAP2
RAB4B	5.01E-19	0.330635	0.331	0.175	1.41E-14	pDC	RAB4B
TPT14	6.14E-19	0.296632	0.977	0.956	1.73E-14	pDC	TPT1
FAAP201	6.21E-19	0.308405	0.423	0.238	1.75E-14	pDC	FAAP20
BHLHE402	6.41E-19	0.473931	0.467	0.28	1.80E-14	pDC	BHLHE40
ARID1B2	6.71E-19	0.474073	0.507	0.318	1.89E-14	pDC	ARID1B
NBDY	6.75E-19	0.316607	0.437	0.255	1.90E-14	pDC	NBDY
TNFSF91	9.55E-19	0.639175	0.367	0.217	2.69E-14	pDC	TNFSF9
NRDC	1.15E-18	0.403772	0.435	0.255	3.23E-14	pDC	NRDC
ARFGAP3	1.23E-18	0.341011	0.355	0.193	3.46E-14	pDC	ARFGAP3
RBM31	1.60E-18	0.458909	0.635	0.478	4.50E-14	pDC	RBM3
NFX1	1.73E-18	0.341015	0.372	0.204	4.88E-14	pDC	NFX1
TP53I13	1.74E-18	0.306584	0.374	0.203	4.91E-14	pDC	TP53I13
PSMB4	1.94E-18	0.281921	0.301	0.151	5.46E-14	pDC	PSMB4
COX141	1.98E-18	0.275877	0.338	0.181	5.57E-14	pDC	COX14
MLF21	2.41E-18	0.314278	0.467	0.282	6.77E-14	pDC	MLF2
NSUN2	2.46E-18	0.330931	0.253	0.12	6.93E-14	pDC	NSUN2
USP241	3.56E-18	0.332533	0.367	0.202	1.00E-13	pDC	USP24
SF3B52	4.02E-18	0.357815	0.52	0.336	1.13E-13	pDC	SF3B5
KRT101	5.28E-18	0.341315	0.478	0.294	1.48E-13	pDC	KRT10
ANAPC5	5.55E-18	0.256491	0.408	0.229	1.56E-13	pDC	ANAPC5
CNOT21	6.05E-18	0.400342	0.459	0.28	1.70E-13	pDC	CNOT2
TRAPPC11	7.15E-18	0.267828	0.49	0.297	2.01E-13	pDC	TRAPPC1
IFI35	7.49E-18	0.309316	0.295	0.151	2.11E-13	pDC	IFI35
HNRNPA0	8.48E-18	0.415474	0.631	0.474	2.39E-13	pDC	HNRNPA0
SERP13	9.98E-18	0.349286	0.775	0.664	2.81E-13	pDC	SERP1
MAPK1IP1l	1.21E-17	0.335875	0.484	0.304	3.42E-13	pDC	MAPK1IP1L
TMEM109	1.41E-17	0.346169	0.444	0.273	3.96E-13	pDC	TMEM109
TOB2	1.46E-17	0.263771	0.274	0.135	4.10E-13	pDC	TOB2

RNF139	1.53E-17	0.28912	0.251	0.121	4.32E-13	pDC	RNF139
RNF71	1.61E-17	0.320786	0.476	0.293	4.53E-13	pDC	RNF7
PRDX62	2.37E-17	0.285878	0.537	0.349	6.68E-13	pDC	PRDX6
ZBTB1	2.52E-17	0.361502	0.399	0.231	7.09E-13	pDC	ZBTB1
EIF4B	2.72E-17	0.330268	0.563	0.376	7.65E-13	pDC	EIF4B
COX6C2	2.75E-17	0.304027	0.641	0.46	7.73E-13	pDC	COX6C
UPF3A	2.79E-17	0.311059	0.291	0.148	7.85E-13	pDC	UPF3A
USP7	2.97E-17	0.319433	0.287	0.148	8.36E-13	pDC	USP7
CD4	3.11E-17	0.367584	0.569	0.401	8.74E-13	pDC	CD4
UBE2M1	3.27E-17	0.261344	0.335	0.183	9.19E-13	pDC	UBE2M
COMMD10	3.50E-17	0.534453	0.418	0.25	9.85E-13	pDC	COMMD10
SUPT3H	3.51E-17	0.299586	0.327	0.174	9.88E-13	pDC	SUPT3H
PIP4K2A3	3.65E-17	0.28731	0.439	0.257	1.03E-12	pDC	PIP4K2A
DDX27	5.94E-17	0.271473	0.31	0.164	1.67E-12	pDC	DDX27
SAP30BP	6.17E-17	0.313587	0.412	0.238	1.74E-12	pDC	SAP30BP
PSMB102	6.96E-17	0.317712	0.505	0.322	1.96E-12	pDC	PSMB10
HIST1H2AC	7.63E-17	0.310571	0.301	0.158	2.15E-12	pDC	HIST1H2AC
RO60	7.68E-17	0.319325	0.268	0.135	2.16E-12	pDC	RO60
CDC42SE22	7.88E-17	0.39621	0.41	0.237	2.22E-12	pDC	CDC42SE2
SLC25A53	8.09E-17	0.389198	0.69	0.551	2.28E-12	pDC	SLC25A5
PA2G41	9.16E-17	0.290938	0.465	0.286	2.58E-12	pDC	PA2G4
PIN1	9.17E-17	0.290517	0.316	0.17	2.58E-12	pDC	PIN1
ATG12	9.75E-17	0.335489	0.287	0.151	2.74E-12	pDC	ATG12
PPP4R3B	1.00E-16	0.28687	0.342	0.189	2.81E-12	pDC	PPP4R3B
XBP11	1.10E-16	0.45728	0.658	0.493	3.09E-12	pDC	XBP1
CCNI3	1.11E-16	0.401772	0.696	0.588	3.13E-12	pDC	CCNI
H1FX3	1.21E-16	0.854631	0.541	0.388	3.40E-12	pDC	H1FX
RPL86	1.22E-16	0.284597	0.915	0.853	3.42E-12	pDC	RPL8
HMGN21	1.22E-16	0.345306	0.658	0.49	3.45E-12	pDC	HMGN2
RPL364	1.41E-16	0.297987	0.892	0.834	3.96E-12	pDC	RPL36
ATP5MC33	1.46E-16	0.372474	0.605	0.438	4.12E-12	pDC	ATP5MC3
TCEA1	1.62E-16	0.319845	0.484	0.307	4.57E-12	pDC	TCEA1
TPM42	1.64E-16	0.310935	0.618	0.422	4.62E-12	pDC	TPM4
TAPBP	1.74E-16	0.369379	0.456	0.288	4.90E-12	pDC	TAPBP
FERMT31	1.86E-16	0.326875	0.473	0.301	5.22E-12	pDC	FERMT3
METRNL4	2.08E-16	0.273589	0.357	0.196	5.85E-12	pDC	METRNL
NDUFB102	2.44E-16	0.30778	0.471	0.294	6.87E-12	pDC	NDUFB10
MYL12B3	2.77E-16	0.427414	0.673	0.558	7.80E-12	pDC	MYL12B
SLC66A2	2.81E-16	0.344609	0.348	0.195	7.92E-12	pDC	SLC66A2
PIK3CG	3.14E-16	0.316676	0.28	0.145	8.83E-12	pDC	PIK3CG
RPL36AL4	3.17E-16	0.320834	0.792	0.659	8.91E-12	pDC	RPL36AL
NDUFA41	3.89E-16	0.309316	0.688	0.511	1.09E-11	pDC	NDUFA4
NADSYN1	3.89E-16	0.351807	0.27	0.138	1.10E-11	pDC	NADSYN1
JARID24	4.02E-16	0.366454	0.45	0.274	1.13E-11	pDC	JARID2
KLC1	5.17E-16	0.316555	0.293	0.154	1.45E-11	pDC	KLC1
EIF2S21	5.78E-16	0.310612	0.427	0.26	1.63E-11	pDC	EIF2S2
TMEM14B	6.00E-16	0.280756	0.452	0.279	1.69E-11	pDC	TMEM14B

ERGIC3	6.27E-16	0.325656	0.425	0.266	1.76E-11	pDC	ERGIC3
CEBPZ	6.42E-16	0.383869	0.297	0.16	1.81E-11	pDC	CEBPZ
PAN32	7.10E-16	0.305293	0.416	0.247	2.00E-11	pDC	PAN3
ANAPC112	7.71E-16	0.284673	0.505	0.323	2.17E-11	pDC	ANAPC11
NOP532	7.98E-16	0.428045	0.609	0.431	2.25E-11	pDC	NOP53
RPL22L11	8.89E-16	0.35032	0.648	0.462	2.50E-11	pDC	RPL22L1
GPAA1	9.34E-16	0.268198	0.318	0.173	2.63E-11	pDC	GPAA1
MVP	1.05E-15	0.285598	0.282	0.149	2.96E-11	pDC	MVP
CCS	1.07E-15	0.331223	0.338	0.192	3.01E-11	pDC	CCS
SLU7	1.10E-15	0.251955	0.251	0.126	3.08E-11	pDC	SLU7
ADAR1	1.13E-15	0.419064	0.456	0.295	3.18E-11	pDC	ADAR
BRD22	1.55E-15	0.392894	0.637	0.468	4.35E-11	pDC	BRD2
UBE2L62	1.57E-15	0.384905	0.414	0.258	4.41E-11	pDC	UBE2L6
FDFT1	1.71E-15	0.255896	0.412	0.243	4.81E-11	pDC	FDFT1
EIF3H3	1.77E-15	0.318984	0.656	0.475	4.99E-11	pDC	EIF3H
MACO1	1.87E-15	0.272339	0.268	0.139	5.27E-11	pDC	MACO1
HMGA11	1.98E-15	0.29337	0.38	0.222	5.57E-11	pDC	HMGA1
MORF4L21	2.25E-15	0.370698	0.495	0.32	6.33E-11	pDC	MORF4L2
PAIP22	2.73E-15	0.288459	0.486	0.313	7.68E-11	pDC	PAIP2
IRF12	3.20E-15	0.366974	0.299	0.165	9.01E-11	pDC	IRF1
NDUFV2	3.32E-15	0.371106	0.454	0.297	9.34E-11	pDC	NDUFV2
IDI1	3.69E-15	0.413247	0.431	0.276	1.04E-10	pDC	IDI1
USP9Y	4.00E-15	0.385845	0.272	0.145	1.13E-10	pDC	USP9Y
PDCD42	4.13E-15	0.334577	0.433	0.275	1.16E-10	pDC	PDCD4
BTF34	4.92E-15	0.334508	0.786	0.706	1.39E-10	pDC	BTF3
CCDC59	5.21E-15	0.270532	0.355	0.204	1.47E-10	pDC	CCDC59
LSM4	5.31E-15	0.264756	0.374	0.221	1.49E-10	pDC	LSM4
NDUFA111	5.62E-15	0.338434	0.622	0.443	1.58E-10	pDC	NDUFA11
H3F3A2	6.23E-15	0.31292	0.845	0.776	1.75E-10	pDC	H3F3A
IFRD12	6.28E-15	0.400414	0.658	0.5	1.77E-10	pDC	IFRD1
SUMO2	7.80E-15	0.339895	0.679	0.559	2.19E-10	pDC	SUMO2
AMZ2	8.30E-15	0.337415	0.34	0.193	2.34E-10	pDC	AMZ2
UBE2D2	9.20E-15	0.347388	0.618	0.467	2.59E-10	pDC	UBE2D2
CYBA1	9.36E-15	0.270875	0.93	0.886	2.63E-10	pDC	CYBA
SEC63	9.40E-15	0.307439	0.359	0.213	2.65E-10	pDC	SEC63
STUB1	9.85E-15	0.293363	0.367	0.215	2.77E-10	pDC	STUB1
RBM4	1.09E-14	0.284323	0.344	0.2	3.07E-10	pDC	RBM4
SNHG5	1.09E-14	0.288148	0.567	0.39	3.07E-10	pDC	SNHG5
TMED101	1.17E-14	0.354	0.599	0.431	3.29E-10	pDC	TMED10
DNAJC7	1.30E-14	0.329897	0.363	0.217	3.66E-10	pDC	DNAJC7
TOP2B	1.61E-14	0.253903	0.257	0.136	4.54E-10	pDC	TOP2B
TAF15	1.85E-14	0.269709	0.482	0.307	5.20E-10	pDC	TAF15
EIF3E2	2.10E-14	0.374445	0.592	0.435	5.91E-10	pDC	EIF3E
ATP5F1A1	2.18E-14	0.370314	0.478	0.327	6.15E-10	pDC	ATP5F1A
RNF220	2.31E-14	0.272066	0.253	0.132	6.49E-10	pDC	RNF220
HIGD2A4	2.53E-14	0.281195	0.605	0.43	7.11E-10	pDC	HIGD2A
PSMB32	2.87E-14	0.272589	0.531	0.356	8.06E-10	pDC	PSMB3

PCMTD1	3.03E-14	0.311432	0.384	0.23	8.53E-10	pDC	PCMTD1
GHITM1	3.19E-14	0.26062	0.433	0.272	8.98E-10	pDC	GHITM
NCL1	3.40E-14	0.311324	0.735	0.596	9.58E-10	pDC	NCL
ILF2	3.50E-14	0.262554	0.452	0.292	9.85E-10	pDC	ILF2
FPGS	3.51E-14	0.278055	0.295	0.164	9.87E-10	pDC	FPGS
HDAC2	3.86E-14	0.267152	0.297	0.163	1.09E-09	pDC	HDAC2
SPCS21	3.87E-14	0.335721	0.493	0.332	1.09E-09	pDC	SPCS2
LCP13	4.02E-14	0.304352	0.611	0.437	1.13E-09	pDC	LCP1
NSA21	4.20E-14	0.284108	0.482	0.311	1.18E-09	pDC	NSA2
RBBP61	5.63E-14	0.355009	0.52	0.358	1.58E-09	pDC	RBBP6
KRTCAP22	9.62E-14	0.312227	0.635	0.485	2.71E-09	pDC	KRTCAP2
NOP56	1.00E-13	0.290569	0.276	0.152	2.83E-09	pDC	NOP56
SCAND11	1.15E-13	0.27314	0.486	0.319	3.22E-09	pDC	SCAND1
OSER1	1.39E-13	0.275715	0.306	0.174	3.92E-09	pDC	OSER1
JUND2	1.47E-13	0.407095	0.873	0.8	4.12E-09	pDC	JUND
SEC31A	1.62E-13	0.306483	0.38	0.237	4.57E-09	pDC	SEC31A
CAPRIN11	2.08E-13	0.323657	0.403	0.256	5.84E-09	pDC	CAPRIN1
COPE3	2.12E-13	0.299745	0.59	0.432	5.96E-09	pDC	COPE
ITCH	2.15E-13	0.287835	0.372	0.226	6.06E-09	pDC	ITCH
G3BP21	2.37E-13	0.269377	0.361	0.224	6.66E-09	pDC	G3BP2
CCDC88A1	2.52E-13	0.397152	0.55	0.391	7.10E-09	pDC	CCDC88A
C11orf58	2.57E-13	0.310575	0.546	0.398	7.22E-09	pDC	C11orf58
SLC25A62	2.76E-13	0.330585	0.749	0.656	7.77E-09	pDC	SLC25A6
TDG	3.22E-13	0.268989	0.255	0.137	9.07E-09	pDC	TDG
TAF103	3.24E-13	0.309104	0.611	0.46	9.12E-09	pDC	TAF10
SMG11	3.80E-13	0.309432	0.408	0.251	1.07E-08	pDC	SMG1
PLAAT3	3.95E-13	0.29221	0.423	0.275	1.11E-08	pDC	PLAAT3
DCAF5	4.12E-13	0.265918	0.265	0.146	1.16E-08	pDC	DCAF5
CYCS	4.18E-13	0.277297	0.55	0.372	1.18E-08	pDC	CYCS
UBB1	4.35E-13	0.349106	0.777	0.675	1.22E-08	pDC	UBB
KDM5A	4.47E-13	0.262615	0.352	0.212	1.26E-08	pDC	KDM5A
AC087286.	5.86E-13	0.291467	0.321	0.186	1.65E-08	pDC	AC087286.2
ARF4	7.86E-13	0.327033	0.631	0.464	2.21E-08	pDC	ARF4
XRN1	7.97E-13	0.390199	0.344	0.21	2.24E-08	pDC	XRN1
SERBP1	8.09E-13	0.268273	0.563	0.413	2.28E-08	pDC	SERBP1
PSME24	8.25E-13	0.304086	0.512	0.351	2.32E-08	pDC	PSME2
NPM12	8.31E-13	0.287583	0.792	0.711	2.34E-08	pDC	NPM1
ROMO11	9.66E-13	0.260843	0.418	0.269	2.72E-08	pDC	ROMO1
NAP1L11	1.03E-12	0.318469	0.588	0.431	2.89E-08	pDC	NAP1L1
POLR2L1	1.92E-12	0.271811	0.444	0.29	5.40E-08	pDC	POLR2L
PSMB81	2.19E-12	0.259019	0.446	0.298	6.16E-08	pDC	PSMB8
COPA1	2.79E-12	0.322892	0.495	0.334	7.85E-08	pDC	COPA
RABAC12	2.79E-12	0.297672	0.527	0.356	7.86E-08	pDC	RABAC1
TRIR1	3.12E-12	0.282917	0.605	0.46	8.79E-08	pDC	TRIR
NDUFB81	3.41E-12	0.271604	0.544	0.378	9.58E-08	pDC	NDUFB8
EIF2S3	3.50E-12	0.269072	0.454	0.3	9.84E-08	pDC	EIF2S3
ADK1	3.61E-12	0.291709	0.418	0.269	1.02E-07	pDC	ADK

SNX33	3.74E-12	0.265066	0.633	0.492	1.05E-07	pDC	SNX3
ITPR12	3.84E-12	0.296333	0.406	0.258	1.08E-07	pDC	ITPR1
MKNK2	3.93E-12	0.274387	0.391	0.247	1.11E-07	pDC	MKNK2
MXD4	4.30E-12	0.347816	0.452	0.303	1.21E-07	pDC	MXD4
UVRAG3	5.40E-12	0.342512	0.531	0.38	1.52E-07	pDC	UVRAG
ASXL2	5.99E-12	0.463093	0.278	0.162	1.69E-07	pDC	ASXL2
PPHLN1	7.14E-12	0.269363	0.314	0.188	2.01E-07	pDC	PPHLN1
ALOX5AP2	7.79E-12	0.263223	0.813	0.747	2.19E-07	pDC	ALOX5AP
RNMT	9.03E-12	0.257456	0.355	0.219	2.54E-07	pDC	RNMT
RPL383	9.12E-12	0.288901	0.752	0.647	2.57E-07	pDC	RPL38
SH3BGRL3!	9.43E-12	0.26412	0.762	0.667	2.65E-07	pDC	SH3BGRL3
SELENOH2	1.49E-11	0.289774	0.588	0.436	4.19E-07	pDC	SELENOH
PHC3	1.50E-11	0.265689	0.323	0.196	4.21E-07	pDC	PHC3
MAP2K31	1.56E-11	0.275648	0.42	0.276	4.40E-07	pDC	MAP2K3
PRDX53	1.95E-11	0.269118	0.48	0.324	5.48E-07	pDC	PRDX5
TRIM44	2.42E-11	0.273126	0.431	0.285	6.81E-07	pDC	TRIM44
PHF14	3.27E-11	0.277567	0.28	0.161	9.21E-07	pDC	PHF14
ARIH12	3.31E-11	0.332284	0.601	0.451	9.32E-07	pDC	ARIH1
DMXL1	3.52E-11	0.29545	0.28	0.164	9.89E-07	pDC	DMXL1
GNAS	4.39E-11	0.277198	0.786	0.716	1.23E-06	pDC	GNAS
SNRNP200	6.26E-11	0.30294	0.297	0.182	1.76E-06	pDC	SNRNP200
YTHDC1	7.36E-11	0.295555	0.41	0.278	2.07E-06	pDC	YTHDC1
KDM4B2	7.58E-11	0.317369	0.41	0.272	2.13E-06	pDC	KDM4B
EIF4G2	7.75E-11	0.317385	0.639	0.511	2.18E-06	pDC	EIF4G2
SCFD1	9.29E-11	0.306214	0.259	0.151	2.61E-06	pDC	SCFD1
ALG13	1.05E-10	0.325909	0.376	0.244	2.95E-06	pDC	ALG13
NDUFB43	1.08E-10	0.254914	0.49	0.347	3.03E-06	pDC	NDUFB4
ZCCHC7	1.11E-10	0.275155	0.259	0.149	3.11E-06	pDC	ZCCHC7
DYNLL11	1.20E-10	0.272605	0.527	0.371	3.36E-06	pDC	DYNLL1
MKLN12	1.20E-10	0.306	0.484	0.339	3.38E-06	pDC	MKLN1
NSD3	1.41E-10	0.316359	0.384	0.249	3.98E-06	pDC	NSD3
LRBA2	1.60E-10	0.302759	0.312	0.193	4.50E-06	pDC	LRBA
EPB41	2.46E-10	0.293805	0.304	0.187	6.93E-06	pDC	EPB41
SMDT14	2.64E-10	0.253483	0.505	0.36	7.42E-06	pDC	SMDT1
FBXW112	2.91E-10	0.328682	0.476	0.339	8.20E-06	pDC	FBXW11
SH2B33	2.92E-10	0.30055	0.516	0.369	8.21E-06	pDC	SH2B3
HNRNPL	3.14E-10	0.27531	0.569	0.429	8.84E-06	pDC	HNRNPL
DDX211	3.27E-10	0.279101	0.556	0.409	9.20E-06	pDC	DDX21
EID11	3.28E-10	0.272131	0.529	0.388	9.23E-06	pDC	EID1
BCLAF11	3.50E-10	0.324276	0.535	0.392	9.86E-06	pDC	BCLAF1
PFKL1	3.92E-10	0.254824	0.333	0.215	1.10E-05	pDC	PFKL
PCNX11	4.76E-10	0.339601	0.28	0.171	1.34E-05	pDC	PCNX1
HECTD1	6.60E-10	0.298873	0.34	0.219	1.86E-05	pDC	HECTD1
GPBP1	6.96E-10	0.256426	0.425	0.298	1.96E-05	pDC	GPBP1
ARF61	8.31E-10	0.264245	0.435	0.306	2.34E-05	pDC	ARF6
ARID4B2	9.40E-10	0.284255	0.478	0.339	2.65E-05	pDC	ARID4B
AUTS22	9.84E-10	0.301827	0.461	0.314	2.77E-05	pDC	AUTS2

CMSS13	1.03E-09	0.701504	0.355	0.243	2.89E-05	pDC	CMSS1
IFI44L2	1.07E-09	0.367748	0.359	0.243	3.02E-05	pDC	IFI44L
BPTF1	1.26E-09	0.29188	0.469	0.34	3.54E-05	pDC	BPTF
FOXP12	1.54E-09	0.266157	0.62	0.467	4.35E-05	pDC	FOXP1
SLC7A11	1.66E-09	0.324533	0.257	0.154	4.66E-05	pDC	SLC7A1
CDC37	1.90E-09	0.266086	0.571	0.444	5.35E-05	pDC	CDC37
PRPF4B	1.94E-09	0.258123	0.369	0.249	5.47E-05	pDC	PRPF4B
FNBP14	2.71E-09	0.28605	0.367	0.243	7.63E-05	pDC	FNBP1
SRSF10	3.54E-09	0.257559	0.55	0.403	9.97E-05	pDC	SRSF10
HDAC94	4.11E-09	0.26801	0.571	0.415	0.000116	pDC	HDAC9
RASA23	8.03E-09	0.255473	0.265	0.164	0.000226	pDC	RASA2
JUNB1	2.14E-08	0.2616	0.926	0.888	0.000602	pDC	JUNB
RBMX	2.47E-08	0.251596	0.55	0.417	0.000694	pDC	RBMX
HM132	2.58E-08	0.250537	0.501	0.376	0.000726	pDC	HM13
ISG154	6.12E-08	0.374755	0.278	0.185	0.001722	pDC	ISG15
INTS63	7.78E-08	0.266387	0.439	0.324	0.00219	pDC	INTS6
ZRANB2	1.34E-07	0.255826	0.321	0.216	0.003778	pDC	ZRANB2
CDC73	3.33E-07	0.25118	0.338	0.231	0.009376	pDC	CDC73
SETD21	5.15E-07	0.273069	0.344	0.246	0.014502	pDC	SETD2
MAN1A12	6.91E-07	0.291292	0.554	0.429	0.019452	pDC	MAN1A1
MBD5	8.11E-06	0.266935	0.285	0.199	0.228297	pDC	MBD5
H3F3B1	3.84E-05	0.306742	0.945	0.917	1	pDC	H3F3B
WWOX2	0.000287	0.323727	0.335	0.258	1	pDC	WWOX
FOXO33	0.000318	0.256077	0.469	0.384	1	pDC	FOXO3
FP671120.4	0.003743	0.851356	0.478	0.419	1	pDC	FP671120.4
PDGFA	9.35E-305	2.145749	0.686	0.093	2.63E-300	MG-Home	PDGFA
TNFRSF12A	3.48E-221	1.962976	0.733	0.148	9.79E-217	MG-Home	TNFRSF12A
LYVE1	1.72E-218	1.824774	0.513	0.068	4.85E-214	MG-Home	LYVE1
MATK	1.11E-195	1.18462	0.365	0.039	3.12E-191	MG-Home	MATK
MRC1	1.44E-181	1.922986	0.55	0.093	4.05E-177	MG-Home	MRC1
XIRP1	1.27E-171	1.568573	0.33	0.036	3.57E-167	MG-Home	XIRP1
NAB2	9.97E-162	1.486723	0.591	0.122	2.81E-157	MG-Home	NAB2
RNASE1	1.62E-159	1.788755	0.396	0.054	4.57E-155	MG-Home	RNASE1
FOLR21	1.97E-135	1.754265	0.686	0.193	5.56E-131	MG-Home	FOLR2
CH25H2	8.82E-120	1.874932	0.965	0.47	2.48E-115	MG-Home	CH25H
TMIGD32	4.77E-119	1.560326	0.89	0.394	1.34E-114	MG-Home	TMIGD3
FHOD11	6.34E-119	1.380395	0.604	0.164	1.78E-114	MG-Home	FHOD1
ABCG2	9.31E-118	0.831833	0.261	0.032	2.62E-113	MG-Home	ABCG2
DHRS92	2.76E-99	1.434835	0.682	0.228	7.78E-95	MG-Home	DHRS9
PLVAP1	8.36E-99	1.394875	0.55	0.158	2.35E-94	MG-Home	PLVAP
SOCS61	2.42E-93	1.414021	0.821	0.384	6.81E-89	MG-Home	SOCS6
FCGR1B1	4.76E-92	1.361407	0.714	0.284	1.34E-87	MG-Home	FCGR1B
ACTG12	6.57E-92	1.378721	0.978	0.791	1.85E-87	MG-Home	ACTG1
NAA20	2.39E-83	1.264134	0.692	0.294	6.74E-79	MG-Home	NAA20
FCGR1A2	1.05E-81	1.39985	0.868	0.508	2.94E-77	MG-Home	FCGR1A
P2RY122	2.53E-80	1.40817	0.884	0.524	7.12E-76	MG-Home	P2RY12
SERPINE13	3.71E-79	1.628951	0.824	0.414	1.04E-74	MG-Home	SERPINE1

HTRA12	1.92E-78	1.256755	0.928	0.628	5.40E-74	MG-Home(	HTRA1
IFNGR12	6.47E-77	1.30808	0.925	0.696	1.82E-72	MG-Home(	IFNGR1
ITM2B	1.88E-75	0.885489	1	0.9	5.30E-71	MG-Home(	ITM2B
AL138963.4	1.96E-75	1.349645	0.594	0.209	5.51E-71	MG-Home(	AL138963.4
BIN1	7.78E-75	1.319536	0.899	0.604	2.19E-70	MG-Home(	BIN1
RGL1	1.25E-73	1.125916	0.459	0.131	3.53E-69	MG-Home(	RGL1
NCK22	4.00E-72	1.065421	0.77	0.373	1.12E-67	MG-Home(	NCK2
RASL11A	1.38E-71	1.099419	0.362	0.087	3.87E-67	MG-Home(	RASL11A
PLD41	7.66E-71	1.161967	0.906	0.618	2.15E-66	MG-Home(	PLD4
PDGFB2	4.40E-70	1.304305	0.73	0.356	1.24E-65	MG-Home(	PDGFB
IL3RA1	1.48E-67	1.037743	0.528	0.18	4.18E-63	MG-Home(	IL3RA
SDK11	6.77E-67	1.206058	0.604	0.235	1.90E-62	MG-Home(	SDK1
SPRY21	1.90E-66	1.118531	0.638	0.26	5.35E-62	MG-Home(	SPRY2
A2M2	3.79E-66	1.07869	0.947	0.751	1.07E-61	MG-Home(	A2M
MTRNR2L1	1.08E-65	1.19808	0.947	0.498	3.03E-61	MG-Home(	MTRNR2L12
ADGRG11	8.00E-65	1.1227	0.717	0.342	2.25E-60	MG-Home(	ADGRG1
PTPN6	1.73E-64	1.149936	0.752	0.41	4.86E-60	MG-Home(	PTPN6
CD992	2.42E-64	1.162774	0.871	0.586	6.82E-60	MG-Home(	CD99
BATF3	1.20E-63	0.854988	0.355	0.094	3.38E-59	MG-Home(	BATF3
MKNK11	1.92E-63	1.006173	0.695	0.327	5.41E-59	MG-Home(	MKNK1
LONRF1	3.55E-63	1.011034	0.362	0.096	9.99E-59	MG-Home(	LONRF1
C3AR12	6.62E-62	1.209969	0.896	0.634	1.86E-57	MG-Home(	C3AR1
LPL1	2.61E-61	0.986458	0.374	0.102	7.34E-57	MG-Home(	LPL
GADD45G3	4.66E-60	1.472833	0.547	0.213	1.31E-55	MG-Home(	GADD45G
SPHK11	1.26E-59	1.076595	0.406	0.124	3.56E-55	MG-Home(	SPHK1
GNAS1	1.61E-59	0.912555	0.953	0.715	4.53E-55	MG-Home(	GNAS
IPCEF13	1.79E-59	1.015741	0.884	0.507	5.02E-55	MG-Home(	IPCEF1
BHLHE411	2.41E-59	1.038772	0.862	0.538	6.79E-55	MG-Home(	BHLHE41
SRC	6.34E-58	0.787933	0.333	0.088	1.78E-53	MG-Home(	SRC
RAMP11	5.15E-57	1.081595	0.513	0.19	1.45E-52	MG-Home(	RAMP1
SLC30A1	7.33E-57	0.869655	0.336	0.092	2.06E-52	MG-Home(	SLC30A1
CD276	7.37E-57	0.954393	0.45	0.155	2.07E-52	MG-Home(	CD276
GPR342	1.47E-55	1.129508	0.903	0.639	4.14E-51	MG-Home(	GPR34
PLK32	3.16E-55	1.03546	0.796	0.457	8.88E-51	MG-Home(	PLK3
SPRED11	9.01E-55	0.942072	0.679	0.318	2.54E-50	MG-Home(	SPRED1
BMPR2	8.67E-54	1.028664	0.566	0.24	2.44E-49	MG-Home(	BMPR2
SLC44A21	1.89E-52	1.017372	0.487	0.19	5.32E-48	MG-Home(	SLC44A2
SLC16A32	2.17E-52	1.137575	0.686	0.374	6.10E-48	MG-Home(	SLC16A3
ITM2C1	2.28E-52	1.045838	0.569	0.259	6.43E-48	MG-Home(	ITM2C
CSF1R	3.77E-52	0.882244	0.953	0.8	1.06E-47	MG-Home(	CSF1R
FOXP2	3.53E-51	0.826354	0.374	0.112	9.94E-47	MG-Home(	FOXP2
STAMBPL1	3.57E-51	0.865199	0.437	0.155	1.00E-46	MG-Home(	STAMBPL1
PDPN1	5.25E-50	1.042416	0.579	0.271	1.48E-45	MG-Home(	PDPN
LINC01141	3.47E-49	0.770656	0.399	0.131	9.77E-45	MG-Home(	LINC01141
DDAH2	1.14E-48	0.959456	0.569	0.269	3.20E-44	MG-Home(	DDAH2
SH3TC11	2.05E-48	0.942105	0.72	0.415	5.78E-44	MG-Home(	SH3TC1
CDK63	6.40E-48	0.932382	0.572	0.259	1.80E-43	MG-Home(	CDK6

GSTM31	8.22E-48	1.022116	0.55	0.247	2.31E-43	MG-Home(	GSTM3
IRAK22	1.12E-47	0.822127	0.808	0.461	3.15E-43	MG-Home(	IRAK2
OLR12	1.65E-47	0.731723	0.975	0.791	4.65E-43	MG-Home(	OLR1
DDX5	2.61E-47	0.661693	0.991	0.902	7.35E-43	MG-Home(	DDX5
CBFB	3.73E-47	0.934771	0.481	0.198	1.05E-42	MG-Home(	CBFB
CKB2	4.63E-47	1.079147	0.717	0.395	1.30E-42	MG-Home(	CKB
PTPRJ1	2.88E-46	0.736586	0.799	0.47	8.10E-42	MG-Home(	PTPRJ
ST6GAL12	6.29E-46	0.810814	0.906	0.649	1.77E-41	MG-Home(	ST6GAL1
CTTNBP22	1.66E-44	0.996902	0.679	0.382	4.66E-40	MG-Home(	CTTNBP2
SPTLC22	2.01E-44	0.883492	0.843	0.6	5.65E-40	MG-Home(	SPTLC2
BCL2L1	3.29E-44	0.983266	0.462	0.192	9.26E-40	MG-Home(	BCL2L1
TNFAIP8L3	6.75E-44	1.041923	0.506	0.214	1.90E-39	MG-Home(	TNFAIP8L3
MIDN1	8.68E-44	0.976152	0.613	0.314	2.44E-39	MG-Home(	MIDN
NRROS1	1.45E-43	0.877425	0.415	0.159	4.09E-39	MG-Home(	NRROS
PRRG4	1.48E-43	0.853908	0.409	0.154	4.16E-39	MG-Home(	PRRG4
PCBP12	2.00E-43	0.892555	0.689	0.409	5.64E-39	MG-Home(	PCBP1
ARRDC2	3.56E-43	1.009156	0.469	0.2	1.00E-38	MG-Home(	ARRDC2
MBD4	3.92E-43	0.88017	0.513	0.226	1.10E-38	MG-Home(	MBD4
ZYX4	1.43E-42	0.996395	0.708	0.445	4.03E-38	MG-Home(	ZYX
GAREM1	7.66E-42	0.81167	0.478	0.2	2.15E-37	MG-Home(	GAREM1
PLD31	2.35E-41	0.904157	0.629	0.347	6.62E-37	MG-Home(	PLD3
NUDT3	5.13E-41	0.845088	0.717	0.449	1.44E-36	MG-Home(	NUDT3
ERF	3.89E-40	0.793584	0.456	0.196	1.09E-35	MG-Home(	ERF
LGMN2	5.76E-40	0.840282	0.726	0.424	1.62E-35	MG-Home(	LGMN
RHOB3	6.18E-40	0.821045	0.972	0.826	1.74E-35	MG-Home(	RHOB
PANX11	1.61E-39	0.839005	0.497	0.219	4.53E-35	MG-Home(	PANX1
ARID5A2	4.65E-39	0.972247	0.635	0.369	1.31E-34	MG-Home(	ARID5A
TWF21	8.77E-39	0.842763	0.582	0.313	2.47E-34	MG-Home(	TWF2
ACTB2	1.01E-38	0.604038	0.997	0.971	2.85E-34	MG-Home(	ACTB
LILRB41	1.05E-38	0.751268	0.884	0.664	2.95E-34	MG-Home(	LILRB4
SLC15A2	1.61E-38	0.653017	0.358	0.127	4.52E-34	MG-Home(	SLC15A2
TAL11	1.99E-38	0.93503	0.616	0.326	5.60E-34	MG-Home(	TAL1
SLC1A32	4.40E-38	0.632286	0.953	0.764	1.24E-33	MG-Home(	SLC1A3
TGFB1	4.45E-38	0.842483	0.846	0.674	1.25E-33	MG-Home(	TGFB1
NBL1	7.24E-38	0.885171	0.412	0.172	2.04E-33	MG-Home(	NBL1
LCP22	7.65E-38	0.848948	0.843	0.618	2.15E-33	MG-Home(	LCP2
SGK13	9.16E-38	0.858206	0.978	0.862	2.58E-33	MG-Home(	SGK1
SPP13	1.36E-37	0.934402	0.965	0.832	3.82E-33	MG-Home(	SPP1
VASP1	4.79E-37	0.845223	0.5	0.245	1.35E-32	MG-Home(	VASP
IL1RAP2	5.33E-37	0.735161	0.698	0.399	1.50E-32	MG-Home(	IL1RAP
SERTAD11	1.49E-36	0.806433	0.657	0.372	4.20E-32	MG-Home(	SERTAD1
JDP23	5.04E-36	0.814386	0.83	0.57	1.42E-31	MG-Home(	JDP2
UBE2F2	7.40E-36	0.797562	0.575	0.313	2.08E-31	MG-Home(	UBE2F
CDKN1A1	8.03E-36	0.872443	0.849	0.587	2.26E-31	MG-Home(	CDKN1A
ACY31	1.08E-35	0.791205	0.459	0.201	3.03E-31	MG-Home(	ACY3
TMIGD21	1.63E-35	0.564131	0.252	0.075	4.58E-31	MG-Home(	TMIGD2
CD53	2.30E-35	0.747216	0.855	0.671	6.47E-31	MG-Home(	CD53

PEA151	2.68E-35	0.827304	0.553	0.295	7.54E-31	MG-Home(	PEA15
RAB1A2	3.50E-35	0.691706	0.78	0.534	9.85E-31	MG-Home(	RAB1A
XIST	3.75E-35	0.632599	0.321	0.109	1.05E-30	MG-Home(	XIST
SLC39A1	4.79E-35	0.898728	0.456	0.219	1.35E-30	MG-Home(	SLC39A1
CLIC13	5.38E-35	0.717515	0.855	0.658	1.51E-30	MG-Home(	CLIC1
SLC1A51	1.34E-34	0.83752	0.579	0.329	3.77E-30	MG-Home(	SLC1A5
VASH11	1.37E-34	0.800972	0.733	0.472	3.87E-30	MG-Home(	VASH1
PMEPA13	2.12E-34	0.77138	0.623	0.355	5.96E-30	MG-Home(	PMEPA1
RGS13	2.30E-34	0.834537	0.959	0.841	6.48E-30	MG-Home(	RGS1
SFPQ1	4.47E-34	0.702839	0.855	0.632	1.26E-29	MG-Home(	SFPQ
CSF2RA3	6.59E-34	0.68706	0.896	0.676	1.86E-29	MG-Home(	CSF2RA
ENPP1	1.00E-33	0.819119	0.286	0.096	2.82E-29	MG-Home(	ENPP1
FHL3	1.21E-33	0.714809	0.39	0.164	3.40E-29	MG-Home(	FHL3
TBC1D16	1.84E-33	0.716682	0.462	0.211	5.18E-29	MG-Home(	TBC1D16
FKBP52	1.89E-33	0.641119	0.912	0.708	5.33E-29	MG-Home(	FKBP5
ATP6V1B2.	6.41E-33	0.853574	0.723	0.475	1.80E-28	MG-Home(	ATP6V1B2
DIP2A1	6.95E-33	0.742019	0.484	0.232	1.96E-28	MG-Home(	DIP2A
CDC42BPB	7.72E-33	0.644349	0.324	0.12	2.17E-28	MG-Home(	CDC42BPB
RAP1A1	1.12E-32	0.705057	0.767	0.544	3.15E-28	MG-Home(	RAP1A
CD631	1.69E-32	0.684525	0.855	0.655	4.74E-28	MG-Home(	CD63
VDAC11	2.86E-32	0.763717	0.541	0.3	8.06E-28	MG-Home(	VDAC1
SIPA1L21	3.14E-32	0.775622	0.5	0.249	8.83E-28	MG-Home(	SIPA1L2
LAMP1	4.42E-32	0.676165	0.692	0.443	1.24E-27	MG-Home(	LAMP1
GNAQ2	1.30E-31	0.610452	0.843	0.623	3.65E-27	MG-Home(	GNAQ
AHDC1	2.04E-31	0.555706	0.255	0.082	5.75E-27	MG-Home(	AHDC1
TM6SF1	2.20E-31	0.762296	0.714	0.481	6.19E-27	MG-Home(	TM6SF1
AC012150.	3.31E-31	0.729142	0.412	0.183	9.30E-27	MG-Home(	AC012150.1
LTC4S1	3.43E-31	0.752342	0.752	0.512	9.64E-27	MG-Home(	LTC4S
SMAD7	4.91E-31	0.876124	0.481	0.242	1.38E-26	MG-Home(	SMAD7
ST6GALNA(	6.51E-31	0.543158	0.843	0.556	1.83E-26	MG-Home(	ST6GALNAC3
GPN3	7.11E-31	0.772456	0.465	0.231	2.00E-26	MG-Home(	GPN3
PLEKHO2	7.88E-31	0.718988	0.453	0.22	2.22E-26	MG-Home(	PLEKHO2
WDR11	8.97E-31	0.741848	0.566	0.337	2.53E-26	MG-Home(	WDR1
SIGLEC10	9.42E-31	0.795133	0.591	0.351	2.65E-26	MG-Home(	SIGLEC10
PXDC1	1.59E-30	0.672732	0.333	0.132	4.47E-26	MG-Home(	PXDC1
CAPZB	4.22E-30	0.567187	0.918	0.759	1.19E-25	MG-Home(	CAPZB
TRIB11	6.37E-30	0.881202	0.774	0.514	1.79E-25	MG-Home(	TRIB1
TPM3	6.82E-30	0.69089	0.836	0.636	1.92E-25	MG-Home(	TPM3
MEF2C2	7.17E-30	0.624541	0.918	0.742	2.02E-25	MG-Home(	MEF2C
LAPTM5	7.33E-30	0.525201	0.975	0.898	2.06E-25	MG-Home(	LAPTM5
GSN	1.08E-29	0.648513	0.937	0.776	3.04E-25	MG-Home(	GSN
SLC2A5	1.21E-29	0.728224	0.83	0.618	3.40E-25	MG-Home(	SLC2A5
GNL1	1.36E-29	0.544762	0.261	0.089	3.84E-25	MG-Home(	GNL1
CXCL163	1.56E-29	0.693311	0.846	0.643	4.38E-25	MG-Home(	CXCL16
PLEKHO1	2.25E-29	0.769783	0.695	0.473	6.34E-25	MG-Home(	PLEKHO1
FAM20C1	1.36E-28	0.690021	0.314	0.125	3.83E-24	MG-Home(	FAM20C
GRASP4	4.45E-28	0.81167	0.783	0.549	1.25E-23	MG-Home(	GRASP

PHC2	5.82E-28	0.77301	0.657	0.443	1.64E-23	MG-Homeo	PHC2
EIF13	7.33E-28	0.436868	0.994	0.924	2.06E-23	MG-Homeo	EIF1
C2	2.56E-27	0.658718	0.311	0.125	7.20E-23	MG-Homeo	C2
IL6R1	3.38E-27	0.660727	0.591	0.357	9.50E-23	MG-Homeo	IL6R
MYADM3	3.70E-27	0.950229	0.566	0.338	1.04E-22	MG-Homeo	MYADM
ID21	1.02E-26	0.671978	0.877	0.66	2.87E-22	MG-Homeo	ID2
RPL214	1.08E-26	0.560897	0.947	0.746	3.05E-22	MG-Homeo	RPL21
DAGLB3	1.22E-26	0.671394	0.67	0.432	3.44E-22	MG-Homeo	DAGLB
AFDN	1.25E-26	0.522093	0.252	0.088	3.53E-22	MG-Homeo	AFDN
SMAP23	1.36E-26	0.616243	0.921	0.769	3.82E-22	MG-Homeo	SMAP2
RTTN	2.06E-26	0.868247	0.327	0.136	5.79E-22	MG-Homeo	RTTN
FSCN11	2.50E-26	0.722086	0.654	0.415	7.02E-22	MG-Homeo	FSCN1
AC010378.	3.07E-26	0.544309	0.264	0.095	8.65E-22	MG-Homeo	AC010378.2
RHBDF21	3.42E-26	0.607559	0.827	0.653	9.62E-22	MG-Homeo	RHBDF2
IFNGR23	3.46E-26	0.725478	0.758	0.545	9.74E-22	MG-Homeo	IFNGR2
SUSD62	3.66E-26	0.713075	0.651	0.435	1.03E-21	MG-Homeo	SUSD6
C12orf751	4.25E-26	0.727355	0.469	0.248	1.20E-21	MG-Homeo	C12orf75
TICAM1	4.98E-26	0.603692	0.396	0.19	1.40E-21	MG-Homeo	TICAM1
KLF101	2.81E-25	0.831764	0.635	0.409	7.90E-21	MG-Homeo	KLF10
CD811	2.89E-25	0.46857	0.981	0.845	8.12E-21	MG-Homeo	CD81
ARHGAP12	3.50E-25	0.655816	0.506	0.28	9.85E-21	MG-Homeo	ARHGAP12
ANXA53	3.56E-25	0.630993	0.824	0.633	1.00E-20	MG-Homeo	ANXA5
MTSS12	3.71E-25	0.834799	0.541	0.317	1.05E-20	MG-Homeo	MTSS1
KDELR21	4.11E-25	0.627892	0.509	0.3	1.16E-20	MG-Homeo	KDELR2
LINC02642	4.65E-25	0.613354	0.396	0.183	1.31E-20	MG-Homeo	LINC02642
SHTN11	4.97E-25	0.620958	0.651	0.424	1.40E-20	MG-Homeo	SHTN1
IER5L1	7.13E-25	0.784824	0.509	0.283	2.01E-20	MG-Homeo	IER5L
PGK12	7.16E-25	0.592427	0.72	0.513	2.01E-20	MG-Homeo	PGK1
TMBIM11	9.51E-25	0.728966	0.478	0.272	2.68E-20	MG-Homeo	TMBIM1
PPP1R18	1.54E-24	0.712998	0.497	0.293	4.33E-20	MG-Homeo	PPP1R18
RGS101	1.82E-24	0.516576	0.918	0.753	5.12E-20	MG-Homeo	RGS10
NFKBID2	2.19E-24	0.675149	0.742	0.508	6.17E-20	MG-Homeo	NFKBID
SH3GL1	3.14E-24	0.659549	0.349	0.161	8.85E-20	MG-Homeo	SH3GL1
SLC15A41	3.18E-24	0.717589	0.453	0.242	8.94E-20	MG-Homeo	SLC15A4
CABIN1	4.19E-24	0.690939	0.34	0.157	1.18E-19	MG-Homeo	CABIN1
ZMIZ12	4.89E-24	0.676437	0.535	0.316	1.38E-19	MG-Homeo	ZMIZ1
EIF4A12	7.07E-24	0.524863	0.947	0.802	1.99E-19	MG-Homeo	EIF4A1
SELENOS3	1.06E-23	0.682946	0.465	0.261	2.99E-19	MG-Homeo	SELENOS
CSGALNAC	1.45E-23	0.447574	0.761	0.516	4.07E-19	MG-Homeo	CSGALNACT1
TNFRSF211	1.60E-23	0.651185	0.277	0.11	4.50E-19	MG-Homeo	TNFRSF21
PSAP2	3.21E-23	0.46515	0.965	0.895	9.02E-19	MG-Homeo	PSAP
RPL415	3.51E-23	0.394855	0.987	0.818	9.89E-19	MG-Homeo	RPL41
NCOR21	4.03E-23	0.678672	0.645	0.451	1.13E-18	MG-Homeo	NCOR2
NLRP35	5.00E-23	0.612017	0.748	0.52	1.41E-18	MG-Homeo	NLRP3
SF11	6.26E-23	0.574797	0.739	0.544	1.76E-18	MG-Homeo	SF1
RHEB1	7.23E-23	0.622342	0.664	0.456	2.03E-18	MG-Homeo	RHEB
RUNX12	1.23E-22	0.553636	0.789	0.6	3.45E-18	MG-Homeo	RUNX1

MAP1S	1.60E-22	0.661196	0.406	0.215	4.49E-18	MG-Home(	MAP1S
ITGAX4	1.67E-22	0.561814	0.638	0.415	4.69E-18	MG-Home(	ITGAX
WASF2	2.00E-22	0.555245	0.802	0.631	5.63E-18	MG-Home(	WASF2
PFN16	3.18E-22	0.487095	0.956	0.827	8.95E-18	MG-Home(	PFN1
KIAA1211L	3.32E-22	0.571588	0.28	0.118	9.34E-18	MG-Home(	KIAA1211L
ARHGAP61	4.46E-22	0.642449	0.525	0.306	1.26E-17	MG-Home(	ARHGAP6
UBASH3B2	4.86E-22	0.581871	0.403	0.203	1.37E-17	MG-Home(	UBASH3B
PDE3B2	5.25E-22	0.558779	0.664	0.439	1.48E-17	MG-Home(	PDE3B
DLGAP4	5.72E-22	0.591222	0.314	0.142	1.61E-17	MG-Home(	DLGAP4
HMGA12	6.57E-22	0.707831	0.412	0.222	1.85E-17	MG-Home(	HMGA1
SLCO2B12	6.84E-22	0.627453	0.739	0.546	1.92E-17	MG-Home(	SLCO2B1
BCOR	6.90E-22	0.571645	0.267	0.11	1.94E-17	MG-Home(	BCOR
CEP1701	7.63E-22	0.624728	0.66	0.459	2.15E-17	MG-Home(	CEP170
LHFPL21	7.71E-22	0.494771	0.846	0.641	2.17E-17	MG-Home(	LHFPL2
RHOC2	8.68E-22	0.673783	0.447	0.255	2.44E-17	MG-Home(	RHOC
RASSF8	1.50E-21	0.542444	0.305	0.133	4.22E-17	MG-Home(	RASSF8
FMNL31	1.59E-21	0.55933	0.642	0.439	4.46E-17	MG-Home(	FMNL3
TTYH31	1.91E-21	0.656498	0.472	0.273	5.38E-17	MG-Home(	TTYH3
ARHGDIA1	2.22E-21	0.562168	0.607	0.408	6.24E-17	MG-Home(	ARHGDIA
PDK42	2.51E-21	0.77621	0.799	0.612	7.05E-17	MG-Home(	PDK4
EBI3	3.45E-21	0.672891	0.472	0.277	9.70E-17	MG-Home(	EBI3
EML41	4.29E-21	0.587033	0.604	0.407	1.21E-16	MG-Home(	EML4
PRNP1	4.44E-21	0.601802	0.535	0.336	1.25E-16	MG-Home(	PRNP
JUND3	4.66E-21	0.482486	0.969	0.8	1.31E-16	MG-Home(	JUND
GAL3ST4	5.17E-21	0.614465	0.45	0.255	1.45E-16	MG-Home(	GAL3ST4
BCAR3	5.22E-21	0.541892	0.343	0.163	1.47E-16	MG-Home(	BCAR3
SLC25A25	7.63E-21	0.565612	0.355	0.177	2.15E-16	MG-Home(	SLC25A25
CHSY12	7.97E-21	0.614009	0.553	0.341	2.24E-16	MG-Home(	CHSY1
ARPC23	8.03E-21	0.472138	0.84	0.637	2.26E-16	MG-Home(	ARPC2
OLFML31	8.59E-21	0.760175	0.604	0.416	2.42E-16	MG-Home(	OLFML3
TMBIM61	1.38E-20	0.528131	0.824	0.655	3.89E-16	MG-Home(	TMBIM6
PEAK13	2.20E-20	0.546076	0.708	0.499	6.19E-16	MG-Home(	PEAK1
CAP11	3.45E-20	0.504775	0.745	0.558	9.71E-16	MG-Home(	CAP1
KIF21B	3.80E-20	0.603116	0.28	0.123	1.07E-15	MG-Home(	KIF21B
GABARAPL	4.67E-20	0.648679	0.607	0.432	1.31E-15	MG-Home(	GABARAPL2
PPP2CA	5.56E-20	0.525748	0.541	0.362	1.56E-15	MG-Home(	PPP2CA
ANKH	5.75E-20	0.456876	0.327	0.151	1.62E-15	MG-Home(	ANKH
B3GNT51	5.98E-20	0.551302	0.83	0.625	1.68E-15	MG-Home(	B3GNT5
SGMS1	6.76E-20	0.522634	0.355	0.178	1.90E-15	MG-Home(	SGMS1
ARRB2	1.27E-19	0.518122	0.796	0.615	3.58E-15	MG-Home(	ARRB2
RNASET21	1.30E-19	0.414675	0.972	0.901	3.66E-15	MG-Home(	RNASET2
LIMS13	2.45E-19	0.453977	0.852	0.689	6.90E-15	MG-Home(	LIMS1
CCL4L22	2.56E-19	0.656418	0.943	0.786	7.20E-15	MG-Home(	CCL4L2
DDX39A	3.43E-19	0.548811	0.497	0.308	9.64E-15	MG-Home(	DDX39A
ATP6V0B	3.87E-19	0.461674	0.833	0.657	1.09E-14	MG-Home(	ATP6V0B
GNB4	4.46E-19	0.509512	0.66	0.46	1.25E-14	MG-Home(	GNB4
RAN2	5.92E-19	0.522999	0.638	0.458	1.66E-14	MG-Home(	RAN

PTMS1	6.02E-19	0.577917	0.431	0.248	1.70E-14	MG-Home(	PTMS
CSTB3	8.33E-19	0.529424	0.616	0.42	2.34E-14	MG-Home(	CSTB
ABI11	9.79E-19	0.563513	0.588	0.408	2.76E-14	MG-Home(	ABI1
PIH1D1	1.03E-18	0.573668	0.443	0.268	2.89E-14	MG-Home(	PIH1D1
KLHL5	1.13E-18	0.624672	0.494	0.31	3.19E-14	MG-Home(	KLHL5
CD683	1.46E-18	0.485476	0.852	0.706	4.11E-14	MG-Home(	CD68
GNB22	1.47E-18	0.557202	0.623	0.459	4.14E-14	MG-Home(	GNB2
AC046195.	2.35E-18	0.611535	0.475	0.275	6.61E-14	MG-Home(	AC046195.1
CTSB2	2.45E-18	0.39599	0.972	0.918	6.90E-14	MG-Home(	CTSB
ABI2	2.50E-18	0.497345	0.264	0.118	7.03E-14	MG-Home(	ABI2
CDK2AP1	3.25E-18	0.694393	0.544	0.38	9.13E-14	MG-Home(	CDK2AP1
HNRNPK	3.39E-18	0.509467	0.767	0.592	9.54E-14	MG-Home(	HNRNPK
RASA31	3.50E-18	0.495064	0.569	0.376	9.84E-14	MG-Home(	RASA3
OTUD12	4.25E-18	0.511988	0.799	0.563	1.20E-13	MG-Home(	OTUD1
USP221	4.80E-18	0.539251	0.453	0.272	1.35E-13	MG-Home(	USP22
ARHGEF40	4.85E-18	0.694189	0.371	0.205	1.37E-13	MG-Home(	ARHGEF40
CRK	6.00E-18	0.443497	0.336	0.171	1.69E-13	MG-Home(	CRK
SETD3	7.26E-18	0.557742	0.336	0.177	2.04E-13	MG-Home(	SETD3
CEBPB1	7.57E-18	0.557902	0.877	0.697	2.13E-13	MG-Home(	CEBPB
LINC02712	1.01E-17	0.56692	0.418	0.235	2.83E-13	MG-Home(	LINC02712
RAC11	1.21E-17	0.422813	0.937	0.826	3.41E-13	MG-Home(	RAC1
FAM53B2	1.36E-17	0.501034	0.459	0.279	3.83E-13	MG-Home(	FAM53B
CD84	1.44E-17	0.577009	0.579	0.404	4.06E-13	MG-Home(	CD84
ERCC11	2.29E-17	0.556393	0.739	0.582	6.45E-13	MG-Home(	ERCC1
ARMCX3	2.81E-17	0.568065	0.387	0.223	7.91E-13	MG-Home(	ARMCX3
MIOS	3.07E-17	0.470806	0.362	0.195	8.64E-13	MG-Home(	MIOS
ZBTB7A	3.19E-17	0.541687	0.34	0.183	8.98E-13	MG-Home(	ZBTB7A
SMURF1	3.63E-17	0.477515	0.346	0.18	1.02E-12	MG-Home(	SMURF1
RBM8A	3.74E-17	0.498183	0.55	0.377	1.05E-12	MG-Home(	RBM8A
ORMDL2	3.76E-17	0.551061	0.292	0.147	1.06E-12	MG-Home(	ORMDL2
ODC12	7.35E-17	0.506052	0.472	0.287	2.07E-12	MG-Home(	ODC1
PPARG	8.06E-17	0.512223	0.314	0.155	2.27E-12	MG-Home(	PPARG
EHD4	8.09E-17	0.571585	0.358	0.198	2.28E-12	MG-Home(	EHD4
SON	8.20E-17	0.47572	0.84	0.703	2.31E-12	MG-Home(	SON
JAZF12	9.20E-17	0.51187	0.679	0.507	2.59E-12	MG-Home(	JAZF1
HIVEP31	9.49E-17	0.54866	0.469	0.287	2.67E-12	MG-Home(	HIVEP3
RPN11	1.07E-16	0.492379	0.538	0.368	3.02E-12	MG-Home(	RPN1
ARF11	1.12E-16	0.474447	0.698	0.542	3.16E-12	MG-Home(	ARF1
MAPKAPK2	1.15E-16	0.522514	0.443	0.273	3.25E-12	MG-Home(	MAPKAPK2
ADRM1	1.25E-16	0.513226	0.415	0.253	3.51E-12	MG-Home(	ADRM1
KPNA4	1.41E-16	0.50186	0.45	0.278	3.97E-12	MG-Home(	KPNA4
KLF22	1.68E-16	0.772045	0.849	0.718	4.73E-12	MG-Home(	KLF2
GPRIN31	1.68E-16	0.6654	0.428	0.262	4.73E-12	MG-Home(	GPRIN3
TLN22	1.90E-16	0.500437	0.412	0.237	5.34E-12	MG-Home(	TLN2
RNF122	2.05E-16	0.499154	0.343	0.182	5.76E-12	MG-Home(	RNF122
CAMSAP1	2.32E-16	0.496375	0.264	0.123	6.53E-12	MG-Home(	CAMSAP1
FEZ21	2.75E-16	0.507831	0.475	0.303	7.72E-12	MG-Home(	FEZ2

MYL64	4.02E-16	0.478927	0.903	0.772	1.13E-11	MG-Homeo	MYL6
INKA1	4.40E-16	0.46958	0.261	0.122	1.24E-11	MG-Homeo	INKA1
NFIC1	4.50E-16	0.611858	0.553	0.399	1.26E-11	MG-Homeo	NFIC
CSRNP11	4.70E-16	0.631327	0.66	0.475	1.32E-11	MG-Homeo	CSRNP1
HNRNPDL	5.74E-16	0.444402	0.849	0.698	1.61E-11	MG-Homeo	HNRNPDL
ADAMTSL2	6.17E-16	0.508403	0.27	0.13	1.74E-11	MG-Homeo	ADAMTSL2
LYL1	7.06E-16	0.573744	0.425	0.27	1.99E-11	MG-Homeo	LYL1
MAP3K11	8.93E-16	0.467901	0.318	0.169	2.51E-11	MG-Homeo	MAP3K11
EFHD22	9.37E-16	0.489805	0.531	0.366	2.64E-11	MG-Homeo	EFHD2
RIN21	9.39E-16	0.468142	0.651	0.478	2.64E-11	MG-Homeo	RIN2
LPAR1	9.74E-16	0.497822	0.381	0.214	2.74E-11	MG-Homeo	LPAR1
ATP6AP11	1.06E-15	0.535045	0.607	0.443	2.99E-11	MG-Homeo	ATP6AP1
RASGEF1C2	1.44E-15	0.433627	0.481	0.296	4.06E-11	MG-Homeo	RASGEF1C
FCGRT2	1.51E-15	0.42994	0.858	0.709	4.24E-11	MG-Homeo	FCGRT
UST1	1.54E-15	0.539141	0.352	0.191	4.34E-11	MG-Homeo	UST
NEDD92	1.55E-15	0.45547	0.73	0.516	4.36E-11	MG-Homeo	NEDD9
SYNDIG12	1.57E-15	0.502593	0.623	0.426	4.42E-11	MG-Homeo	SYNDIG1
DDX3X1	1.83E-15	0.467373	0.676	0.514	5.15E-11	MG-Homeo	DDX3X
EGR31	1.91E-15	0.519379	0.654	0.458	5.39E-11	MG-Homeo	EGR3
MFSD121	2.17E-15	0.581456	0.421	0.267	6.11E-11	MG-Homeo	MFSD12
ACTR3	2.54E-15	0.487352	0.623	0.472	7.15E-11	MG-Homeo	ACTR3
MT-ATP63	2.65E-15	0.354255	0.95	0.857	7.47E-11	MG-Homeo	MT-ATP6
COPB1	3.25E-15	0.461227	0.403	0.245	9.14E-11	MG-Homeo	COPB1
TBXAS13	3.37E-15	0.349889	0.865	0.718	9.49E-11	MG-Homeo	TBXAS1
SKIL2	5.26E-15	0.414636	0.846	0.674	1.48E-10	MG-Homeo	SKIL
PRXL2C1	6.27E-15	0.575198	0.399	0.246	1.76E-10	MG-Homeo	PRXL2C
CDV31	7.50E-15	0.471568	0.553	0.397	2.11E-10	MG-Homeo	CDV3
EGLN2	8.33E-15	0.434748	0.261	0.128	2.34E-10	MG-Homeo	EGLN2
UBE2J11	8.52E-15	0.484317	0.431	0.273	2.40E-10	MG-Homeo	UBE2J1
VAPA1	9.95E-15	0.496877	0.642	0.488	2.80E-10	MG-Homeo	VAPA
UBE2N	1.11E-14	0.514598	0.431	0.281	3.11E-10	MG-Homeo	UBE2N
NFATC12	1.25E-14	0.485374	0.415	0.248	3.53E-10	MG-Homeo	NFATC1
GAA	2.54E-14	0.424713	0.591	0.425	7.14E-10	MG-Homeo	GAA
SPRY12	2.81E-14	0.678046	0.421	0.252	7.91E-10	MG-Homeo	SPRY1
PRKCH4	2.99E-14	0.316514	0.786	0.595	8.42E-10	MG-Homeo	PRKCH
SLC3A22	3.00E-14	0.575809	0.535	0.382	8.43E-10	MG-Homeo	SLC3A2
CMTM6	3.44E-14	0.428395	0.654	0.503	9.67E-10	MG-Homeo	CMTM6
PALD12	4.71E-14	0.33802	0.723	0.554	1.33E-09	MG-Homeo	PALD1
YBX14	7.33E-14	0.358807	0.786	0.667	2.06E-09	MG-Homeo	YBX1
CSNK1E1	9.61E-14	0.424327	0.252	0.126	2.70E-09	MG-Homeo	CSNK1E
TNFRSF10D	1.24E-13	0.445979	0.296	0.156	3.48E-09	MG-Homeo	TNFRSF10D
KLF63	1.37E-13	0.433915	0.975	0.896	3.87E-09	MG-Homeo	KLF6
SH3GLB11	1.39E-13	0.46354	0.403	0.255	3.92E-09	MG-Homeo	SH3GLB1
GPCPD13	1.69E-13	0.404617	0.553	0.392	4.77E-09	MG-Homeo	GPCPD1
KMT5A	1.87E-13	0.438582	0.255	0.128	5.26E-09	MG-Homeo	KMT5A
HOMER31	1.88E-13	0.467814	0.289	0.156	5.29E-09	MG-Homeo	HOMER3
LIPA2	1.92E-13	0.433316	0.487	0.334	5.40E-09	MG-Homeo	LIPA

ITGA51	2.23E-13	0.599949	0.311	0.174	6.26E-09	MG-Home(	ITGA5
USP41	2.35E-13	0.468565	0.519	0.372	6.61E-09	MG-Home(	USP4
CALR3	2.71E-13	0.41749	0.72	0.567	7.63E-09	MG-Home(	CALR
VPS37B	3.02E-13	0.426162	0.431	0.278	8.49E-09	MG-Home(	VPS37B
IL17RA1	3.06E-13	0.459114	0.557	0.403	8.61E-09	MG-Home(	IL17RA
PIM33	3.09E-13	0.488496	0.274	0.143	8.69E-09	MG-Home(	PIM3
HNRNPF	3.50E-13	0.486178	0.585	0.456	9.85E-09	MG-Home(	HNRNPF
SH2B34	4.23E-13	0.446731	0.525	0.369	1.19E-08	MG-Home(	SH2B3
BCL23	4.29E-13	0.437513	0.528	0.362	1.21E-08	MG-Home(	BCL2
SGTA	4.75E-13	0.38955	0.258	0.133	1.34E-08	MG-Home(	SGTA
FUS1	5.02E-13	0.320942	0.767	0.612	1.41E-08	MG-Home(	FUS
SLC25A32	5.14E-13	0.477595	0.321	0.185	1.45E-08	MG-Home(	SLC25A32
SIGLEC8	6.01E-13	0.514415	0.403	0.255	1.69E-08	MG-Home(	SIGLEC8
GATM	6.68E-13	0.458412	0.428	0.278	1.88E-08	MG-Home(	GATM
PIEZO1	6.70E-13	0.4536	0.333	0.194	1.88E-08	MG-Home(	PIEZO1
PNP1	6.81E-13	0.659684	0.437	0.292	1.92E-08	MG-Home(	PNP
TSC22D32	7.93E-13	0.533348	0.83	0.718	2.23E-08	MG-Home(	TSC22D3
CD834	7.97E-13	0.274093	0.997	0.912	2.24E-08	MG-Home(	CD83
EIF4E3	8.28E-13	0.431383	0.623	0.434	2.33E-08	MG-Home(	EIF4E
REST	1.02E-12	0.445918	0.28	0.153	2.88E-08	MG-Home(	REST
GABARAPL	1.04E-12	0.538503	0.465	0.318	2.94E-08	MG-Home(	GABARAPL1
CASS4	1.12E-12	0.562494	0.374	0.232	3.15E-08	MG-Home(	CASS4
LRRK12	1.14E-12	0.422128	0.494	0.338	3.20E-08	MG-Home(	LRRK1
BCAP312	1.18E-12	0.409675	0.594	0.454	3.32E-08	MG-Home(	BCAP31
CTTNBP2NI	1.20E-12	0.430924	0.522	0.372	3.37E-08	MG-Home(	CTTNBP2NL
DDX39B	1.27E-12	0.414282	0.528	0.381	3.56E-08	MG-Home(	DDX39B
SKI1	1.29E-12	0.257488	0.553	0.378	3.64E-08	MG-Home(	SKI
CRTC2	1.37E-12	0.433065	0.27	0.145	3.84E-08	MG-Home(	CRTC2
SUN2	1.38E-12	0.523735	0.289	0.165	3.89E-08	MG-Home(	SUN2
CSNK1D	1.43E-12	0.456826	0.487	0.347	4.02E-08	MG-Home(	CSNK1D
C5orf15	1.46E-12	0.422593	0.277	0.152	4.10E-08	MG-Home(	C5orf15
HNRNPM	1.55E-12	0.483551	0.491	0.363	4.36E-08	MG-Home(	HNRNPM
TMOD1	1.64E-12	0.389513	0.299	0.164	4.62E-08	MG-Home(	TMOD1
FGD22	1.68E-12	0.455508	0.428	0.281	4.74E-08	MG-Home(	FGD2
CEP681	1.79E-12	0.483029	0.371	0.227	5.04E-08	MG-Home(	CEP68
RAPGEF61	2.10E-12	0.49036	0.437	0.292	5.90E-08	MG-Home(	RAPGEF6
RELA	2.13E-12	0.394266	0.292	0.166	6.01E-08	MG-Home(	RELA
AL139807.:	2.65E-12	0.561759	0.358	0.22	7.45E-08	MG-Home(	AL139807.1
TP53I11	2.85E-12	0.436257	0.289	0.16	8.02E-08	MG-Home(	TP53I11
AC079015.	3.10E-12	0.50614	0.308	0.175	8.71E-08	MG-Home(	AC079015.1
BCL7B	3.56E-12	0.464569	0.333	0.205	1.00E-07	MG-Home(	BCL7B
OFD11	4.27E-12	0.565582	0.368	0.234	1.20E-07	MG-Home(	OFD1
GDI21	4.44E-12	0.3499	0.619	0.474	1.25E-07	MG-Home(	GDI2
B4GALT4	4.61E-12	0.418421	0.267	0.144	1.30E-07	MG-Home(	B4GALT4
FYTTD11	4.72E-12	0.493293	0.305	0.179	1.33E-07	MG-Home(	FYTTD1
CTBP22	5.35E-12	0.354547	0.384	0.239	1.51E-07	MG-Home(	CTBP2
XBP12	5.70E-12	0.470344	0.638	0.493	1.60E-07	MG-Home(	XBP1

CD1641	6.46E-12	0.389405	0.563	0.424	1.82E-07	MG-Homeo	CD164
MEF2A3	6.92E-12	0.288169	0.909	0.785	1.95E-07	MG-Homeo	MEF2A
ZSWIM62	7.27E-12	0.313442	0.767	0.619	2.05E-07	MG-Homeo	ZSWIM6
NECTIN21	9.80E-12	0.474197	0.261	0.142	2.76E-07	MG-Homeo	NECTIN2
DGKA	9.89E-12	0.464177	0.283	0.16	2.78E-07	MG-Homeo	DGKA
TMPO	9.90E-12	0.389767	0.365	0.224	2.79E-07	MG-Homeo	TMPO
TRAK11	1.19E-11	0.435573	0.346	0.212	3.36E-07	MG-Homeo	TRAK1
TMEM1191	1.28E-11	0.37646	0.553	0.395	3.60E-07	MG-Homeo	TMEM119
CREG11	1.39E-11	0.446157	0.594	0.458	3.92E-07	MG-Homeo	CREG1
ARRDC31	1.44E-11	0.495545	0.305	0.177	4.04E-07	MG-Homeo	ARRDC3
PITPNA1	1.70E-11	0.370695	0.333	0.201	4.79E-07	MG-Homeo	PITPNA
PER12	2.01E-11	0.308776	0.733	0.571	5.66E-07	MG-Homeo	PER1
GNB11	2.40E-11	0.335965	0.61	0.481	6.76E-07	MG-Homeo	GNB1
TMX1	2.71E-11	0.347267	0.399	0.265	7.62E-07	MG-Homeo	TMX1
MCL11	3.20E-11	0.348889	0.959	0.82	9.00E-07	MG-Homeo	MCL1
KDM6B1	3.24E-11	0.361549	0.745	0.548	9.12E-07	MG-Homeo	KDM6B
HLA-E1	3.33E-11	0.320829	0.953	0.831	9.37E-07	MG-Homeo	HLA-E
MAPK1IP1L	3.48E-11	0.429147	0.437	0.305	9.80E-07	MG-Homeo	MAPK1IP1L
PAFAH1B11	3.59E-11	0.360953	0.525	0.393	1.01E-06	MG-Homeo	PAFAH1B1
SH3BP2	3.73E-11	0.412987	0.349	0.221	1.05E-06	MG-Homeo	SH3BP2
ETS21	4.38E-11	0.362091	0.802	0.64	1.23E-06	MG-Homeo	ETS2
USP12	4.67E-11	0.384156	0.274	0.153	1.31E-06	MG-Homeo	USP12
HMGN13	4.96E-11	0.371159	0.585	0.463	1.40E-06	MG-Homeo	HMGN1
AKT31	5.15E-11	0.376448	0.393	0.251	1.45E-06	MG-Homeo	AKT3
SLC20A11	5.51E-11	0.436696	0.327	0.199	1.55E-06	MG-Homeo	SLC20A1
DNAJB12	5.80E-11	0.391158	0.318	0.195	1.63E-06	MG-Homeo	DNAJB12
DOK1	6.58E-11	0.44272	0.318	0.195	1.85E-06	MG-Homeo	DOK1
SMAD3	7.01E-11	0.415761	0.349	0.219	1.97E-06	MG-Homeo	SMAD3
BZW12	7.45E-11	0.461587	0.509	0.388	2.10E-06	MG-Homeo	BZW1
KIAA0232	8.62E-11	0.384381	0.277	0.158	2.43E-06	MG-Homeo	KIAA0232
AKIRIN1	9.47E-11	0.478695	0.324	0.204	2.66E-06	MG-Homeo	AKIRIN1
ALAS1	1.05E-10	0.381357	0.267	0.151	2.94E-06	MG-Homeo	ALAS1
NCOA32	1.26E-10	0.304378	0.481	0.338	3.53E-06	MG-Homeo	NCOA3
MERTK1	1.34E-10	0.365734	0.55	0.409	3.78E-06	MG-Homeo	MERTK
ELK3	1.41E-10	0.52114	0.318	0.199	3.96E-06	MG-Homeo	ELK3
CCR1	1.43E-10	0.506255	0.327	0.207	4.02E-06	MG-Homeo	CCR1
TNFRSF1A	1.44E-10	0.426487	0.478	0.35	4.05E-06	MG-Homeo	TNFRSF1A
SRSF21	1.45E-10	0.348689	0.67	0.532	4.09E-06	MG-Homeo	SRSF2
MAP4K43	1.50E-10	0.308864	0.516	0.372	4.23E-06	MG-Homeo	MAP4K4
PDXK2	1.58E-10	0.408736	0.462	0.338	4.44E-06	MG-Homeo	PDXK
RTN42	1.63E-10	0.339579	0.774	0.632	4.57E-06	MG-Homeo	RTN4
FMNL11	1.76E-10	0.450646	0.484	0.356	4.94E-06	MG-Homeo	FMNL1
CYFIP11	1.87E-10	0.33982	0.783	0.661	5.26E-06	MG-Homeo	CYFIP1
RNF145	2.13E-10	0.433525	0.374	0.248	5.99E-06	MG-Homeo	RNF145
CALM12	2.15E-10	0.362912	0.777	0.643	6.05E-06	MG-Homeo	CALM1
SCAMP2	2.18E-10	0.393782	0.619	0.503	6.14E-06	MG-Homeo	SCAMP2
IL6ST2	2.37E-10	0.383505	0.657	0.514	6.67E-06	MG-Homeo	IL6ST

APBB1IP2	2.50E-10	0.308274	0.903	0.777	7.04E-06	MG-Home(	APBB1IP
ARHGAP31	2.92E-10	0.431214	0.33	0.212	8.21E-06	MG-Home(	ARHGAP31
MED13L3	3.11E-10	0.254587	0.626	0.48	8.76E-06	MG-Home(	MED13L
LCP14	3.49E-10	0.377189	0.569	0.437	9.81E-06	MG-Home(	LCP1
CDC371	3.74E-10	0.350353	0.563	0.444	1.05E-05	MG-Home(	CDC37
ARGLU11	4.25E-10	0.318606	0.601	0.477	1.20E-05	MG-Home(	ARGLU1
CDC42SE11	4.29E-10	0.36388	0.513	0.387	1.21E-05	MG-Home(	CDC42SE1
YWHAE	4.49E-10	0.388412	0.582	0.466	1.26E-05	MG-Home(	YWHAE
GLIPR11	4.49E-10	0.367959	0.613	0.486	1.26E-05	MG-Home(	GLIPR1
HAVCR21	5.20E-10	0.35388	0.704	0.569	1.46E-05	MG-Home(	HAVCR2
KPNB1	5.22E-10	0.36982	0.431	0.309	1.47E-05	MG-Home(	KPNB1
JUNB2	5.89E-10	0.416124	0.969	0.888	1.66E-05	MG-Home(	JUNB
CCL22	5.98E-10	0.419919	0.519	0.338	1.68E-05	MG-Home(	CCL2
ARL6IP12	6.02E-10	0.349437	0.714	0.588	1.69E-05	MG-Home(	ARL6IP1
DEGS1	6.56E-10	0.360061	0.434	0.311	1.85E-05	MG-Home(	DEGS1
SIRPA1	6.74E-10	0.382315	0.418	0.294	1.90E-05	MG-Home(	SIRPA
ITGB5	7.81E-10	0.36962	0.258	0.146	2.20E-05	MG-Home(	ITGB5
EIF4G21	8.12E-10	0.301039	0.635	0.512	2.29E-05	MG-Home(	EIF4G2
RASSF1	8.92E-10	0.386697	0.252	0.144	2.51E-05	MG-Home(	RASSF1
INPPL1	9.27E-10	0.378053	0.261	0.153	2.61E-05	MG-Home(	INPPL1
TNFRSF1B1	9.42E-10	0.334588	0.66	0.522	2.65E-05	MG-Home(	TNFRSF1B
RRP7A	9.94E-10	0.346666	0.289	0.175	2.80E-05	MG-Home(	RRP7A
MAST3	1.03E-09	0.432545	0.289	0.177	2.89E-05	MG-Home(	MAST3
EIF3A	1.11E-09	0.350653	0.494	0.374	3.13E-05	MG-Home(	EIF3A
ILKAP	1.25E-09	0.339916	0.264	0.154	3.51E-05	MG-Home(	ILKAP
ITGAM	1.32E-09	0.373598	0.387	0.267	3.71E-05	MG-Home(	ITGAM
PTTG1IP1	1.35E-09	0.364064	0.509	0.387	3.79E-05	MG-Home(	PTTG1IP
AL078590.:	1.38E-09	0.352445	0.601	0.44	3.88E-05	MG-Home(	AL078590.2
FPR11	1.48E-09	0.386818	0.752	0.629	4.15E-05	MG-Home(	FPR1
CX3CR13	1.70E-09	0.369996	0.711	0.573	4.77E-05	MG-Home(	CX3CR1
PDIA31	1.71E-09	0.269414	0.67	0.545	4.81E-05	MG-Home(	PDIA3
TIMP2	1.72E-09	0.380758	0.569	0.461	4.85E-05	MG-Home(	TIMP2
POGZ	1.77E-09	0.369495	0.336	0.221	4.99E-05	MG-Home(	POGZ
SLC25A63	1.78E-09	0.345507	0.792	0.656	5.02E-05	MG-Home(	SLC25A6
ORMDL1	1.82E-09	0.453953	0.443	0.334	5.12E-05	MG-Home(	ORMDL1
SSBP4	1.86E-09	0.428985	0.299	0.191	5.22E-05	MG-Home(	SSBP4
ARHGEF72	1.87E-09	0.396397	0.428	0.302	5.27E-05	MG-Home(	ARHGEF7
CYTH31	2.15E-09	0.348073	0.34	0.215	6.05E-05	MG-Home(	CYTH3
PRRC2A	2.25E-09	0.316212	0.355	0.237	6.33E-05	MG-Home(	PRRC2A
LINC01480	2.31E-09	0.43653	0.255	0.146	6.50E-05	MG-Home(	LINC01480
KLF72	2.65E-09	0.322209	0.465	0.332	7.45E-05	MG-Home(	KLF7
RABEP1	2.73E-09	0.336175	0.346	0.227	7.68E-05	MG-Home(	RABEP1
CPSF6	2.97E-09	0.327858	0.343	0.226	8.34E-05	MG-Home(	CPSF6
TRIM28	3.44E-09	0.388347	0.343	0.23	9.68E-05	MG-Home(	TRIM28
NEK6	3.56E-09	0.374711	0.261	0.155	0.0001	MG-Home(	NEK6
ARL8A1	3.61E-09	0.413387	0.302	0.195	0.000102	MG-Home(	ARL8A
GPR137B	3.84E-09	0.352177	0.28	0.169	0.000108	MG-Home(	GPR137B

SELPLG1	3.87E-09	0.327689	0.73	0.591	0.000109	MG-Home(	SELPLG
LUZP1	4.26E-09	0.3747	0.28	0.171	0.00012	MG-Home(	LUZP1
CEBPD2	4.53E-09	0.348782	0.943	0.871	0.000127	MG-Home(	CEBPD
TMED52	4.93E-09	0.333445	0.465	0.345	0.000139	MG-Home(	TMED5
CHD71	5.32E-09	0.2899	0.443	0.314	0.00015	MG-Home(	CHD7
PAG12	5.53E-09	0.35657	0.563	0.436	0.000156	MG-Home(	PAG1
SAT2	5.61E-09	0.476532	0.434	0.328	0.000158	MG-Home(	SAT2
MGST31	5.81E-09	0.397338	0.497	0.386	0.000163	MG-Home(	MGST3
OS9	6.42E-09	0.328745	0.538	0.428	0.000181	MG-Home(	OS9
HNRNPA12	6.57E-09	0.288881	0.811	0.671	0.000185	MG-Home(	HNRNPA1
HNRNPA3	6.72E-09	0.334508	0.642	0.553	0.000189	MG-Home(	HNRNPA3
SMG12	7.24E-09	0.356811	0.368	0.252	0.000204	MG-Home(	SMG1
PSMA11	7.61E-09	0.353677	0.566	0.459	0.000214	MG-Home(	PSMA1
ASAP13	7.63E-09	0.369641	0.336	0.222	0.000215	MG-Home(	ASAP1
RNH12	8.04E-09	0.413162	0.44	0.34	0.000226	MG-Home(	RNH1
ATP6V0C3	9.42E-09	0.288145	0.814	0.695	0.000265	MG-Home(	ATP6V0C
ZMYND11	9.63E-09	0.376148	0.296	0.187	0.000271	MG-Home(	ZMYND11
BCAT12	1.14E-08	0.374616	0.267	0.162	0.000322	MG-Home(	BCAT1
LASP1	1.34E-08	0.408352	0.314	0.208	0.000376	MG-Home(	LASP1
SPNS2	1.41E-08	0.39131	0.277	0.173	0.000396	MG-Home(	SPNS2
ALDOA4	1.42E-08	0.269167	0.582	0.457	0.0004	MG-Home(	ALDOA
ABCC41	1.59E-08	0.313869	0.604	0.477	0.000447	MG-Home(	ABCC4
FAM133B	1.88E-08	0.434074	0.355	0.25	0.000529	MG-Home(	FAM133B
SRSF32	2.10E-08	0.33265	0.664	0.54	0.000591	MG-Home(	SRSF3
BAIAP21	2.13E-08	0.361088	0.324	0.215	0.000599	MG-Home(	BAIAP2
RPN2	2.17E-08	0.338359	0.569	0.459	0.000611	MG-Home(	RPN2
PGM2	2.18E-08	0.377643	0.264	0.166	0.000613	MG-Home(	PGM2
SPIN1	2.27E-08	0.321291	0.274	0.17	0.000638	MG-Home(	SPIN1
TP53I131	2.31E-08	0.373683	0.308	0.204	0.00065	MG-Home(	TP53I13
YWHAG	2.37E-08	0.396633	0.336	0.229	0.000668	MG-Home(	YWHAG
NFE2L23	2.57E-08	0.25021	0.579	0.458	0.000723	MG-Home(	NFE2L2
NIPA2	2.59E-08	0.30184	0.277	0.173	0.000729	MG-Home(	NIPA2
MYL12B4	2.62E-08	0.326095	0.657	0.558	0.000737	MG-Home(	MYL12B
MAGED2	2.68E-08	0.413354	0.299	0.197	0.000754	MG-Home(	MAGED2
DUSP12	2.80E-08	0.35365	0.978	0.943	0.000789	MG-Home(	DUSP1
RGL2	3.11E-08	0.382143	0.252	0.156	0.000874	MG-Home(	RGL2
WDR911	3.22E-08	0.324093	0.39	0.274	0.000907	MG-Home(	WDR91
AFTPH	3.42E-08	0.356925	0.34	0.232	0.000963	MG-Home(	AFTPH
MCF2L3	3.46E-08	0.3595	0.406	0.289	0.000972	MG-Home(	MCF2L
GDI1	3.46E-08	0.35325	0.358	0.252	0.000974	MG-Home(	GDI1
PPP4C1	3.76E-08	0.363556	0.399	0.298	0.001059	MG-Home(	PPP4C
LMO2	3.81E-08	0.349166	0.428	0.318	0.001072	MG-Home(	LMO2
OTULINL	3.88E-08	0.361938	0.538	0.43	0.001091	MG-Home(	OTULINL
NDRG2	3.90E-08	0.402451	0.314	0.212	0.001098	MG-Home(	NDRG2
RBM32	4.00E-08	0.369825	0.579	0.478	0.001125	MG-Home(	RBM3
KDM2B	4.01E-08	0.405814	0.28	0.18	0.001129	MG-Home(	KDM2B
RYR12	4.02E-08	0.370617	0.365	0.252	0.001131	MG-Home(	RYR1

BSG	4.10E-08	0.356664	0.456	0.353	0.001152	MG-Home	BSG
YWHAZ2	4.90E-08	0.254946	0.704	0.596	0.001378	MG-Home	YWHAZ
PRKAB1	4.92E-08	0.338253	0.321	0.214	0.001383	MG-Home	PRKAB1
CLDND1	5.04E-08	0.346379	0.258	0.162	0.001418	MG-Home	CLDND1
TM9SF31	5.36E-08	0.294189	0.415	0.305	0.001508	MG-Home	TM9SF3
EWSR1	6.02E-08	0.279405	0.528	0.431	0.001693	MG-Home	EWSR1
NDFIP1	6.34E-08	0.314809	0.481	0.379	0.001783	MG-Home	NDFIP1
NOL7	6.65E-08	0.354255	0.459	0.357	0.001872	MG-Home	NOL7
GAS61	7.05E-08	0.377976	0.371	0.267	0.001985	MG-Home	GAS6
PTGS1	7.07E-08	0.406009	0.365	0.268	0.001988	MG-Home	PTGS1
KLF3	7.37E-08	0.427887	0.374	0.273	0.002073	MG-Home	KLF3
ATP6V1F2	7.49E-08	0.273663	0.645	0.52	0.002109	MG-Home	ATP6V1F
PRMT1	7.70E-08	0.310316	0.362	0.259	0.002168	MG-Home	PRMT1
USP531	7.88E-08	0.414604	0.55	0.436	0.002217	MG-Home	USP53
PSMD82	8.45E-08	0.340887	0.381	0.28	0.002379	MG-Home	PSMD8
UBQLN1	8.59E-08	0.316937	0.305	0.204	0.002418	MG-Home	UBQLN1
MAPRE1	8.60E-08	0.366991	0.355	0.258	0.00242	MG-Home	MAPRE1
AUP1	8.67E-08	0.352059	0.513	0.417	0.00244	MG-Home	AUP1
TRIP122	8.75E-08	0.320813	0.409	0.302	0.002461	MG-Home	TRIP12
EMD1	9.05E-08	0.406012	0.374	0.278	0.002547	MG-Home	EMD
DHX15	1.03E-07	0.323037	0.305	0.205	0.002892	MG-Home	DHX15
LINC00963	1.03E-07	0.349736	0.387	0.277	0.002897	MG-Home	LINC00963
EIF1B2	1.10E-07	0.380537	0.447	0.348	0.003081	MG-Home	EIF1B
ARPC4	1.12E-07	0.317188	0.475	0.379	0.003152	MG-Home	ARPC4
FERMT32	1.29E-07	0.350811	0.406	0.301	0.003621	MG-Home	FERMT3
Mar-71	1.29E-07	0.33495	0.371	0.271	0.00364	MG-Home	7-Mar
ARF62	1.34E-07	0.361704	0.406	0.307	0.003777	MG-Home	ARF6
DYNLL12	1.51E-07	0.340001	0.475	0.371	0.004235	MG-Home	DYNLL1
EIF4G1	1.70E-07	0.332851	0.343	0.245	0.004788	MG-Home	EIF4G1
ACSL33	1.84E-07	0.288999	0.365	0.261	0.005168	MG-Home	ACSL3
GSTM2	1.87E-07	0.335309	0.399	0.296	0.005264	MG-Home	GSTM2
ATF7IP2	1.93E-07	0.340515	0.274	0.176	0.005419	MG-Home	ATF7IP2
PSMA72	2.26E-07	0.330693	0.585	0.483	0.006363	MG-Home	PSMA7
BID2	2.36E-07	0.366279	0.343	0.244	0.00664	MG-Home	BID
SH3BP1	2.42E-07	0.292414	0.336	0.235	0.006817	MG-Home	SH3BP1
CYTL1	2.50E-07	0.318384	0.311	0.206	0.007046	MG-Home	CYTL1
MXD41	2.64E-07	0.324714	0.409	0.303	0.007426	MG-Home	MXD4
AHCYL1	2.91E-07	0.341146	0.324	0.228	0.008179	MG-Home	AHCYL1
E2F4	2.92E-07	0.395191	0.267	0.178	0.008212	MG-Home	E2F4
SEC62	3.02E-07	0.349271	0.503	0.411	0.008484	MG-Home	SEC62
PITPNB	3.20E-07	0.339204	0.277	0.183	0.008995	MG-Home	PITPNB
AKIRIN22	3.44E-07	0.365129	0.399	0.305	0.009692	MG-Home	AKIRIN2
CDC14B1	4.10E-07	0.282019	0.252	0.156	0.011547	MG-Home	CDC14B
EIF4H	4.12E-07	0.295941	0.465	0.372	0.011598	MG-Home	EIF4H
DAZAP1	4.34E-07	0.312383	0.277	0.186	0.012216	MG-Home	DAZAP1
MGAT4A2	4.44E-07	0.34073	0.569	0.484	0.012491	MG-Home	MGAT4A
MAGT1	4.93E-07	0.305013	0.358	0.257	0.013871	MG-Home	MAGT1

DENND4C1	5.01E-07	0.28322	0.377	0.272	0.014093	MG-Homeo	DENND4C
ZFP362	5.09E-07	0.429307	0.965	0.931	0.014312	MG-Homeo	ZFP36
FLOT11	5.13E-07	0.370053	0.506	0.42	0.014424	MG-Homeo	FLOT1
RNFT1	5.21E-07	0.316834	0.252	0.162	0.014672	MG-Homeo	RNFT1
BAG6	5.69E-07	0.314594	0.28	0.19	0.016005	MG-Homeo	BAG6
VPS29	5.70E-07	0.346605	0.469	0.381	0.016036	MG-Homeo	VPS29
RASAL21	5.73E-07	0.340571	0.45	0.354	0.016134	MG-Homeo	RASAL2
TSPAN3	5.93E-07	0.338728	0.418	0.328	0.016696	MG-Homeo	TSPAN3
TCEAL3	6.61E-07	0.391286	0.289	0.2	0.018591	MG-Homeo	TCEAL3
ZNF7062	7.91E-07	0.34561	0.399	0.306	0.022261	MG-Homeo	ZNF706
SYPL1	8.04E-07	0.350742	0.289	0.203	0.022625	MG-Homeo	SYPL1
ZNF800	8.60E-07	0.328666	0.299	0.208	0.024192	MG-Homeo	ZNF800
EIF4A32	8.96E-07	0.393987	0.494	0.392	0.025206	MG-Homeo	EIF4A3
PDS5A1	9.06E-07	0.266181	0.387	0.286	0.025499	MG-Homeo	PDS5A
RAP1B2	1.01E-06	0.272748	0.509	0.423	0.02839	MG-Homeo	RAP1B
GSPT11	1.04E-06	0.297198	0.368	0.273	0.029367	MG-Homeo	GSPT1
RER11	1.12E-06	0.383226	0.358	0.276	0.031605	MG-Homeo	RER1
DENND11	1.14E-06	0.305748	0.252	0.164	0.032053	MG-Homeo	DENND11
RGS19	1.14E-06	0.338319	0.484	0.401	0.032189	MG-Homeo	RGS19
TMBIM4	1.31E-06	0.287944	0.613	0.521	0.036887	MG-Homeo	TMBIM4
M6PR	1.48E-06	0.300891	0.434	0.348	0.041647	MG-Homeo	M6PR
NPTN	1.48E-06	0.286627	0.321	0.229	0.041721	MG-Homeo	NPTN
CGGBP1	1.50E-06	0.322361	0.311	0.22	0.042267	MG-Homeo	CGGBP1
HMOX12	1.71E-06	0.278543	0.667	0.546	0.048034	MG-Homeo	HMOX1
AC131944.	1.86E-06	0.298327	0.384	0.272	0.052329	MG-Homeo	AC131944.1
TMEM259	2.06E-06	0.301559	0.384	0.298	0.058082	MG-Homeo	TMEM259
MRC2	2.09E-06	0.454379	0.258	0.175	0.058707	MG-Homeo	MRC2
SPCS32	2.14E-06	0.341684	0.336	0.25	0.060308	MG-Homeo	SPCS3
SFT2D1	2.19E-06	0.320593	0.393	0.309	0.061563	MG-Homeo	SFT2D1
DPP7	2.32E-06	0.272752	0.516	0.437	0.065319	MG-Homeo	DPP7
UFM11	2.37E-06	0.326805	0.314	0.229	0.066807	MG-Homeo	UFM1
SSB	2.59E-06	0.292614	0.396	0.308	0.072833	MG-Homeo	SSB
MICAL11	2.65E-06	0.301445	0.377	0.29	0.074623	MG-Homeo	MICAL1
RCOR12	2.92E-06	0.284189	0.314	0.225	0.08205	MG-Homeo	RCOR1
CEP3501	3.01E-06	0.339001	0.447	0.367	0.084762	MG-Homeo	CEP350
PUF60	3.27E-06	0.343769	0.318	0.238	0.092079	MG-Homeo	PUF60
VSIG41	3.27E-06	0.288683	0.629	0.533	0.092125	MG-Homeo	VSIG4
WBP2	3.38E-06	0.311585	0.355	0.272	0.095231	MG-Homeo	WBP2
RBMX1	3.39E-06	0.277615	0.509	0.418	0.09533	MG-Homeo	RBMX
TOR1AIP1	3.56E-06	0.309604	0.308	0.222	0.100203	MG-Homeo	TOR1AIP1
ZNF2672	3.57E-06	0.29385	0.434	0.338	0.100484	MG-Homeo	ZNF267
IST11	3.65E-06	0.253381	0.343	0.252	0.10273	MG-Homeo	IST1
BTBD1	4.00E-06	0.27151	0.318	0.226	0.112526	MG-Homeo	BTBD1
TMEM9B1	4.12E-06	0.322875	0.352	0.27	0.115946	MG-Homeo	TMEM9B
SEC14L1	4.12E-06	0.334647	0.465	0.372	0.11604	MG-Homeo	SEC14L1
BTG31	4.21E-06	0.327143	0.349	0.258	0.118507	MG-Homeo	BTG3
EIF1AX	4.60E-06	0.357998	0.396	0.314	0.129326	MG-Homeo	EIF1AX

DDOST1	4.62E-06	0.278177	0.437	0.357	0.130026	MG-Home(	DDOST
CBX3	4.65E-06	0.288124	0.355	0.266	0.130845	MG-Home(	CBX3
MYDGF1	4.78E-06	0.272552	0.453	0.363	0.134622	MG-Home(	MYDGF
IVNS1ABP1	4.89E-06	0.336344	0.45	0.366	0.137532	MG-Home(	IVNS1ABP
KHDRBS1	4.93E-06	0.282239	0.443	0.36	0.138793	MG-Home(	KHDRBS1
KLF9	5.18E-06	0.324551	0.252	0.169	0.14584	MG-Home(	KLF9
CHD93	5.72E-06	0.291497	0.553	0.475	0.160942	MG-Home(	CHD9
PIP5K1B	5.88E-06	0.271521	0.261	0.173	0.165566	MG-Home(	PIP5K1B
MPRIP2	5.93E-06	0.319306	0.321	0.234	0.166967	MG-Home(	MPRIP
TLR42	6.26E-06	0.271422	0.308	0.219	0.176249	MG-Home(	TLR4
DUSP63	6.35E-06	0.341279	0.387	0.288	0.178723	MG-Home(	DUSP6
PLA2G4A1	6.54E-06	0.25955	0.475	0.376	0.183887	MG-Home(	PLA2G4A
MPP12	7.24E-06	0.360867	0.349	0.266	0.203718	MG-Home(	MPP1
CSNK2B2	8.05E-06	0.364378	0.509	0.443	0.226635	MG-Home(	CSNK2B
ILK	8.24E-06	0.292608	0.308	0.229	0.231924	MG-Home(	ILK
MTCH1	8.32E-06	0.273913	0.44	0.361	0.233993	MG-Home(	MTCH1
WAPL	8.46E-06	0.366346	0.346	0.268	0.238028	MG-Home(	WAPL
MEF2D1	9.88E-06	0.308203	0.264	0.186	0.278053	MG-Home(	MEF2D
AGO3	1.03E-05	0.32918	0.308	0.227	0.288536	MG-Home(	AGO3
POMP2	1.11E-05	0.281497	0.588	0.512	0.312266	MG-Home(	POMP
IPO7	1.14E-05	0.291972	0.321	0.241	0.320887	MG-Home(	IPO7
LIMD22	1.18E-05	0.386211	0.418	0.336	0.332037	MG-Home(	LIMD2
GOLPH3	1.21E-05	0.278356	0.305	0.223	0.341862	MG-Home(	GOLPH3
IL13RA11	1.22E-05	0.289986	0.601	0.532	0.344133	MG-Home(	IL13RA1
GRINA2	1.23E-05	0.305807	0.5	0.428	0.346686	MG-Home(	GRINA
CCT5	1.44E-05	0.276808	0.299	0.22	0.406531	MG-Home(	CCT5
SPPL2A	1.52E-05	0.304637	0.314	0.233	0.427462	MG-Home(	SPPL2A
PANK2	1.57E-05	0.334587	0.274	0.198	0.440778	MG-Home(	PANK2
HM133	1.65E-05	0.268861	0.453	0.376	0.464086	MG-Home(	HM13
SDF41	1.73E-05	0.280884	0.34	0.266	0.486648	MG-Home(	SDF4
UBE2I	1.79E-05	0.280783	0.355	0.28	0.502915	MG-Home(	UBE2I
SNX5	1.83E-05	0.30734	0.412	0.336	0.513988	MG-Home(	SNX5
NOP103	1.87E-05	0.290768	0.406	0.328	0.526607	MG-Home(	NOP10
DNASE2	1.97E-05	0.278554	0.362	0.283	0.553101	MG-Home(	DNASE2
ST8SIA4	1.98E-05	0.380938	0.352	0.276	0.556733	MG-Home(	ST8SIA4
SMS	1.98E-05	0.301948	0.384	0.305	0.557499	MG-Home(	SMS
SERINC1	2.37E-05	0.259761	0.456	0.382	0.665945	MG-Home(	SERINC1
GPS2	2.52E-05	0.292956	0.336	0.261	0.709373	MG-Home(	GPS2
ASXL1	2.56E-05	0.276943	0.286	0.207	0.719795	MG-Home(	ASXL1
ATXN2L	2.72E-05	0.263923	0.314	0.236	0.764565	MG-Home(	ATXN2L
H1F01	2.98E-05	0.349521	0.365	0.276	0.838153	MG-Home(	H1F0
PRDX63	3.53E-05	0.306184	0.418	0.349	0.993327	MG-Home(	PRDX6
DDX212	3.66E-05	0.254124	0.491	0.409	1	MG-Home(	DDX21
EVI2A	3.89E-05	0.322932	0.384	0.311	1	MG-Home(	EVI2A
SCAF4	4.28E-05	0.272479	0.264	0.191	1	MG-Home(	SCAF4
USP11	4.49E-05	0.323296	0.267	0.195	1	MG-Home(	USP11
AHSA11	4.70E-05	0.326058	0.28	0.206	1	MG-Home(	AHSA1

PLCL22	4.85E-05	0.31833	0.321	0.245	1	MG-Home(	PLCL2
MT2A3	4.95E-05	1.059978	0.459	0.388	1	MG-Home(	MT2A
CYTH4	4.98E-05	0.262106	0.311	0.236	1	MG-Home(	CYTH4
PARVG1	5.00E-05	0.279427	0.478	0.418	1	MG-Home(	PARVG
TRAM12	5.80E-05	0.283813	0.346	0.275	1	MG-Home(	TRAM1
DELE1	5.92E-05	0.366572	0.286	0.217	1	MG-Home(	DELE1
CEBPA1	6.00E-05	0.364576	0.425	0.357	1	MG-Home(	CEBPA
USP6NL2	6.34E-05	0.251916	0.324	0.249	1	MG-Home(	USP6NL
VCP	6.80E-05	0.304365	0.459	0.395	1	MG-Home(	VCP
PABPN1	7.24E-05	0.297142	0.525	0.47	1	MG-Home(	PABPN1
NAGA	7.59E-05	0.274738	0.318	0.25	1	MG-Home(	NAGA
EIF2S22	8.17E-05	0.261991	0.33	0.26	1	MG-Home(	EIF2S2
EMP33	8.31E-05	0.441466	0.374	0.299	1	MG-Home(	EMP3
MRPL14	8.38E-05	0.288016	0.264	0.196	1	MG-Home(	MRPL14
PRKCB3	8.43E-05	0.264637	0.318	0.243	1	MG-Home(	PRKCB
GGNBP2	8.91E-05	0.269235	0.39	0.323	1	MG-Home(	GGNBP2
PFDN2	0.000106	0.319268	0.346	0.284	1	MG-Home(	PFDN2
HNRNPAB	0.000117	0.298698	0.368	0.305	1	MG-Home(	HNRNPAB
JMJD61	0.000118	0.352754	0.33	0.259	1	MG-Home(	JMJD6
SPCS22	0.000129	0.250192	0.396	0.333	1	MG-Home(	SPCS2
PPP2R2A	0.000133	0.289549	0.302	0.234	1	MG-Home(	PPP2R2A
IRF2BP22	0.000135	0.350251	0.346	0.275	1	MG-Home(	IRF2BP2
TMEM52B2	0.000142	0.29116	0.387	0.314	1	MG-Home(	TMEM52B
MAP2K32	0.000147	0.269275	0.349	0.276	1	MG-Home(	MAP2K3
EHBP1L1	0.000152	0.294661	0.274	0.209	1	MG-Home(	EHBP1L1
PNPLA2	0.000154	0.295182	0.399	0.342	1	MG-Home(	PNPLA2
DAB22	0.000156	0.321126	0.321	0.252	1	MG-Home(	DAB2
UBA2	0.000167	0.298858	0.264	0.2	1	MG-Home(	UBA2
KTN1	0.000197	0.28397	0.519	0.462	1	MG-Home(	KTN1
WASHC1	0.000202	0.281226	0.264	0.202	1	MG-Home(	WASHC1
FLT11	0.000235	0.280618	0.258	0.185	1	MG-Home(	FLT1
CCDC85B	0.000238	0.290813	0.327	0.268	1	MG-Home(	CCDC85B
ABHD11	0.00027	0.281799	0.264	0.199	1	MG-Home(	ABHD11
TRIM8	0.000278	0.304731	0.286	0.223	1	MG-Home(	TRIM8
ACADVL1	0.000334	0.269943	0.355	0.296	1	MG-Home(	ACADVL
ETNK12	0.000409	0.295872	0.358	0.3	1	MG-Home(	ETNK1
VDAC21	0.000412	0.269787	0.459	0.403	1	MG-Home(	VDAC2
SAFB	0.000416	0.259226	0.274	0.214	1	MG-Home(	SAFB
HNRNPH3	0.000484	0.26719	0.393	0.339	1	MG-Home(	HNRNPH3
ALOX51	0.000571	0.252039	0.522	0.472	1	MG-Home(	ALOX5
SLTM	0.000642	0.264494	0.393	0.341	1	MG-Home(	SLTM
IMP3	0.000728	0.260597	0.255	0.199	1	MG-Home(	IMP3
METTL7A	0.000731	0.265895	0.387	0.33	1	MG-Home(	METTL7A
PLEKHM2	0.000788	0.253103	0.299	0.245	1	MG-Home(	PLEKHM2
P2RX41	0.000853	0.30473	0.318	0.259	1	MG-Home(	P2RX4
MRPL54	0.00103	0.254831	0.264	0.211	1	MG-Home(	MRPL54
PMS1	0.001167	0.287636	0.267	0.21	1	MG-Home(	PMS1

PTBP1	0.001274	0.285532	0.296	0.246	1	MG-Home	PTBP1
CTSC	0.001808	0.265996	0.638	0.591	1	MG-Home	CTSC
NORAD	0.002524	0.271722	0.274	0.226	1	MG-Home	NORAD
METAP2	0.002572	0.26687	0.277	0.229	1	MG-Home	METAP2
VEGFB2	0.003135	0.259165	0.314	0.269	1	MG-Home	VEGFB
SNRPB	0.003163	0.259555	0.371	0.336	1	MG-Home	SNRPB
P2RY131	0.005962	0.295836	0.387	0.344	1	MG-Home	P2RY13
SOCS32	0	3.935983	0.966	0.19	0	MG-Periva	SOCS3
CCL8	0	3.438968	0.543	0.034	0	MG-Periva	CCL8
TNFRSF10D	0	3.014877	0.921	0.154	0	MG-Periva	TNFRSF10D
XIRP11	0	2.641222	0.57	0.035	0	MG-Periva	XIRP1
PDGFA1	0	2.640801	0.845	0.093	0	MG-Periva	PDGFA
LYVE11	0	2.371926	0.785	0.068	0	MG-Periva	LYVE1
PIM1	0	2.362105	0.743	0.064	0	MG-Periva	PIM1
MRC11	0	2.336635	0.789	0.093	0	MG-Periva	MRC1
HAS1	0	1.977944	0.389	0.019	0	MG-Periva	HAS1
MATK1	0	1.550448	0.491	0.039	0	MG-Periva	MATK
DACT1	0	1.480371	0.453	0.021	0	MG-Periva	DACT1
CISH	0	1.013306	0.377	0.022	0	MG-Periva	CISH
RGL11	8.62E-273	2.133977	0.819	0.13	2.42E-268	MG-Periva	RGL1
JAM3	2.30E-272	0.977118	0.279	0.014	6.48E-268	MG-Periva	JAM3
THBD1	1.73E-235	2.120155	0.555	0.065	4.88E-231	MG-Periva	THBD
SPHK12	6.23E-218	1.942884	0.717	0.124	1.75E-213	MG-Periva	SPHK1
RAMP12	2.52E-216	2.177832	0.864	0.19	7.09E-212	MG-Periva	RAMP1
FLT12	1.49E-201	2.696432	0.811	0.184	4.20E-197	MG-Periva	FLT1
PLVAP2	2.95E-194	1.884475	0.77	0.157	8.30E-190	MG-Periva	PLVAP
TNFRSF12A	2.48E-192	1.771255	0.758	0.148	6.96E-188	MG-Periva	TNFRSF12A
RNASE11	6.00E-184	1.708951	0.46	0.054	1.69E-179	MG-Periva	RNASE1
BATF	6.31E-183	0.873586	0.325	0.028	1.77E-178	MG-Periva	BATF
TPD52	3.33E-182	1.620959	0.521	0.073	9.36E-178	MG-Periva	TPD52
HIC1	1.97E-172	1.030407	0.358	0.036	5.54E-168	MG-Periva	HIC1
CCL23	1.27E-171	3.140542	0.97	0.337	3.58E-167	MG-Periva	CCL2
AC072022.	1.84E-169	0.80534	0.313	0.028	5.19E-165	MG-Periva	AC072022.2
CDKN1A2	6.16E-161	2.534361	0.996	0.586	1.73E-156	MG-Periva	CDKN1A
PLXNA2	4.67E-159	1.170634	0.464	0.064	1.31E-154	MG-Periva	PLXNA2
CA8	1.13E-158	1.067013	0.294	0.027	3.19E-154	MG-Periva	CA8
FCGR1B2	3.57E-158	2.15644	0.879	0.284	1.00E-153	MG-Periva	FCGR1B
PANX12	3.06E-154	1.653353	0.826	0.218	8.61E-150	MG-Periva	PANX1
BATF31	4.08E-153	1.385077	0.547	0.093	1.15E-148	MG-Periva	BATF3
FOLR22	8.22E-147	1.594953	0.781	0.193	2.31E-142	MG-Periva	FOLR2
SPRED12	2.92E-146	1.78009	0.94	0.317	8.21E-142	MG-Periva	SPRED1
FHOD12	1.16E-141	1.452939	0.702	0.164	3.27E-137	MG-Periva	FHOD1
BCL62	1.47E-141	1.927691	0.917	0.331	4.14E-137	MG-Periva	BCL6
CH25H3	1.61E-140	2.514249	0.981	0.471	4.52E-136	MG-Periva	CH25H
DHRS93	2.38E-140	2.099741	0.804	0.228	6.71E-136	MG-Periva	DHRS9
PIM34	2.61E-140	1.534035	0.649	0.142	7.35E-136	MG-Periva	PIM3
PEX5	3.78E-138	1.335424	0.404	0.057	1.06E-133	MG-Periva	PEX5

HOMER1	4.39E-136	1.576014	0.589	0.114	1.24E-131	MG-Periva:	HOMER1
BCL32	8.72E-136	1.099766	0.521	0.092	2.45E-131	MG-Periva:	BCL3
HLX	2.48E-135	1.114517	0.472	0.076	6.98E-131	MG-Periva:	HLX
HLX-AS1	2.97E-135	0.874092	0.358	0.045	8.34E-131	MG-Periva:	HLX-AS1
NAB21	2.44E-133	1.174509	0.608	0.122	6.85E-129	MG-Periva:	NAB2
ARID5A3	1.97E-130	1.897754	0.917	0.368	5.55E-126	MG-Periva:	ARID5A
SLC2A34	8.71E-129	2.075582	0.94	0.384	2.45E-124	MG-Periva:	SLC2A3
IL1RAP3	2.28E-125	1.733517	0.94	0.398	6.41E-121	MG-Periva:	IL1RAP
SDK12	1.23E-123	1.55151	0.804	0.234	3.47E-119	MG-Periva:	SDK1
TNFSF18	9.14E-121	1.779885	0.419	0.066	2.57E-116	MG-Periva:	TNFSF18
CEMIP21	1.94E-119	1.269904	0.509	0.098	5.45E-115	MG-Periva:	CEMIP2
FCGR1A3	2.18E-117	1.941513	0.951	0.508	6.13E-113	MG-Periva:	FCGR1A
GADD45G4	3.19E-116	2.090423	0.732	0.212	8.99E-112	MG-Periva:	GADD45G
SNHG151	6.28E-116	1.405337	0.694	0.19	1.77E-111	MG-Periva:	SNHG15
MT2A4	3.46E-115	3.434518	0.86	0.387	9.73E-111	MG-Periva:	MT2A
MIDN2	1.93E-110	1.626785	0.849	0.313	5.43E-106	MG-Periva:	MIDN
B3GNT52	5.92E-110	1.870826	0.989	0.625	1.67E-105	MG-Periva:	B3GNT5
CTSL2	1.50E-108	2.228437	0.872	0.37	4.22E-104	MG-Periva:	CTSL
RUNX13	1.89E-107	1.737454	0.977	0.6	5.31E-103	MG-Periva:	RUNX1
PRR5	1.32E-103	0.992282	0.491	0.1	3.72E-99	MG-Periva:	PRR5
TXLNA	7.65E-102	1.070941	0.423	0.078	2.15E-97	MG-Periva:	TXLNA
ABCG21	1.25E-101	0.642296	0.268	0.032	3.53E-97	MG-Periva:	ABCG2
LCP23	3.42E-100	1.582287	0.985	0.618	9.62E-96	MG-Periva:	LCP2
DIP2C	1.41E-99	1.175581	0.551	0.129	3.98E-95	MG-Periva:	DIP2C
AC046195.	1.90E-99	1.574847	0.781	0.274	5.34E-95	MG-Periva:	AC046195.1
CSRNP12	9.18E-99	1.544157	0.928	0.475	2.58E-94	MG-Periva:	CSRNP1
RHOU	4.71E-98	0.902768	0.302	0.043	1.33E-93	MG-Periva:	RHOU
NCK23	6.36E-98	1.425618	0.883	0.373	1.79E-93	MG-Periva:	NCK2
IFI16	1.11E-97	1.631364	0.894	0.435	3.12E-93	MG-Periva:	IFI16
XIST1	1.86E-97	0.871447	0.513	0.109	5.25E-93	MG-Periva:	XIST
SH2B2	2.46E-96	0.705265	0.253	0.031	6.91E-92	MG-Periva:	SH2B2
ARRDC32	2.24E-95	1.370163	0.63	0.176	6.30E-91	MG-Periva:	ARRDC3
GAREM11	1.51E-94	1.172903	0.679	0.199	4.25E-90	MG-Periva:	GAREM1
ITPKC	1.59E-94	0.868097	0.298	0.043	4.48E-90	MG-Periva:	ITPKC
ELOC2	1.19E-93	1.666811	0.868	0.403	3.35E-89	MG-Periva:	ELOC
AC079015.	3.89E-93	1.248334	0.623	0.174	1.09E-88	MG-Periva:	AC079015.1
CHSY13	1.51E-92	1.412332	0.838	0.34	4.24E-88	MG-Periva:	CHSY1
AL139807.:	2.68E-91	1.296266	0.694	0.219	7.53E-87	MG-Periva:	AL139807.1
SRC1	1.72E-89	0.849173	0.434	0.088	4.85E-85	MG-Periva:	SRC
KLF102	8.93E-89	1.493072	0.891	0.409	2.51E-84	MG-Periva:	KLF10
GOLGA41	4.92E-88	1.683648	0.762	0.306	1.38E-83	MG-Periva:	GOLGA4
PPARD2	7.51E-88	1.296017	0.83	0.342	2.11E-83	MG-Periva:	PPARD
AC010378.	1.78E-87	0.803611	0.453	0.095	5.01E-83	MG-Periva:	AC010378.2
NEDD93	1.47E-86	1.476333	0.947	0.516	4.14E-82	MG-Periva:	NEDD9
TMEM701	1.62E-86	1.310385	0.66	0.214	4.57E-82	MG-Periva:	TMEM70
TP53BP21	2.63E-86	1.323389	0.619	0.187	7.40E-82	MG-Periva:	TP53BP2
BMPR21	1.09E-85	1.254505	0.706	0.239	3.07E-81	MG-Periva:	BMPR2

SBNO2	1.42E-84	0.85463	0.457	0.103	4.00E-80	MG-Periva:	SBNO2
LINC01814	3.29E-84	0.851901	0.325	0.055	9.27E-80	MG-Periva:	LINC01814
SPRY22	1.34E-83	1.29788	0.74	0.26	3.78E-79	MG-Periva:	SPRY2
LMNB1	2.12E-83	0.675684	0.294	0.046	5.95E-79	MG-Periva:	LMNB1
AL138963.4	8.18E-81	1.370451	0.657	0.209	2.30E-76	MG-Periva:	AL138963.4
MCF2L22	1.08E-80	1.33791	0.834	0.365	3.04E-76	MG-Periva:	MCF2L2
UBASH3B3	5.19E-79	1.174164	0.638	0.203	1.46E-74	MG-Periva:	UBASH3B
PTPN22	1.07E-78	1.23731	0.725	0.278	3.02E-74	MG-Periva:	PTPN2
ZMIZ13	2.71E-78	1.208775	0.777	0.315	7.63E-74	MG-Periva:	ZMIZ1
PDPN2	1.63E-77	1.254465	0.713	0.271	4.60E-73	MG-Periva:	PDPN
AP000331.	1.52E-76	0.915735	0.438	0.102	4.29E-72	MG-Periva:	AP000331.1
JAG1	1.69E-76	0.863588	0.317	0.056	4.75E-72	MG-Periva:	JAG1
PXDC11	1.72E-76	1.006395	0.498	0.131	4.85E-72	MG-Periva:	PXDC1
MOB3C	3.09E-76	0.76565	0.343	0.066	8.70E-72	MG-Periva:	MOB3C
UST2	3.67E-76	1.330655	0.604	0.191	1.03E-71	MG-Periva:	UST
KDM6B2	1.80E-75	1.298261	0.955	0.547	5.07E-71	MG-Periva:	KDM6B
BHLHE403	9.51E-74	1.367057	0.721	0.28	2.68E-69	MG-Periva:	BHLHE40
RAPGEF22	1.01E-73	1.171384	0.698	0.251	2.84E-69	MG-Periva:	RAPGEF2
PRKAR1B	1.11E-73	0.899016	0.483	0.125	3.12E-69	MG-Periva:	PRKAR1B
SEH1L	1.87E-73	0.765728	0.423	0.098	5.26E-69	MG-Periva:	SEH1L
VEGFA4	2.29E-73	1.440609	0.532	0.151	6.45E-69	MG-Periva:	VEGFA
FAM157C	3.16E-73	0.645497	0.279	0.046	8.90E-69	MG-Periva:	FAM157C
STAT3	3.56E-73	1.114763	0.891	0.45	1.00E-68	MG-Periva:	STAT3
HIVEP32	4.05E-73	1.198604	0.725	0.286	1.14E-68	MG-Periva:	HIVEP3
NECTIN22	7.96E-73	0.874994	0.509	0.142	2.24E-68	MG-Periva:	NECTIN2
STX43	5.28E-72	1.472736	0.751	0.307	1.49E-67	MG-Periva:	STX4
PNP2	6.68E-72	1.628388	0.713	0.292	1.88E-67	MG-Periva:	PNP
IL3RA2	9.29E-72	1.071085	0.589	0.18	2.62E-67	MG-Periva:	IL3RA
FAM20C2	1.48E-71	0.844538	0.479	0.125	4.15E-67	MG-Periva:	FAM20C
PRNP2	2.93E-71	1.250797	0.766	0.335	8.25E-67	MG-Periva:	PRNP
DOT1L	3.46E-70	0.887903	0.453	0.116	9.73E-66	MG-Periva:	DOT1L
CD2761	7.53E-70	0.879638	0.532	0.155	2.12E-65	MG-Periva:	CD276
CYTOR	9.92E-70	0.903796	0.328	0.065	2.79E-65	MG-Periva:	CYTOR
MPP13	6.92E-69	1.18401	0.687	0.265	1.95E-64	MG-Periva:	MPP1
FPR12	8.27E-69	1.218793	0.958	0.629	2.33E-64	MG-Periva:	FPR1
CPEB41	9.34E-69	1.107792	0.736	0.293	2.63E-64	MG-Periva:	CPEB4
ZSWIM63	1.58E-68	1.17102	0.936	0.619	4.44E-64	MG-Periva:	ZSWIM6
IL6ST3	2.33E-68	1.303705	0.894	0.514	6.56E-64	MG-Periva:	IL6ST
SLC1A33	4.98E-68	1.170436	0.989	0.764	1.40E-63	MG-Periva:	SLC1A3
PELI12	2.90E-66	1.284724	0.906	0.546	8.16E-62	MG-Periva:	PELI1
RNF19A	5.43E-66	1.004811	0.528	0.158	1.53E-61	MG-Periva:	RNF19A
FURIN	6.02E-66	0.647557	0.294	0.056	1.70E-61	MG-Periva:	FURIN
NFATC13	6.30E-66	1.146737	0.668	0.248	1.77E-61	MG-Periva:	NFATC1
NCS1	1.10E-65	0.666119	0.26	0.044	3.09E-61	MG-Periva:	NCS1
GRAMD41	5.59E-65	1.010983	0.506	0.153	1.57E-60	MG-Periva:	GRAMD4
13-Mar	6.19E-65	1.370973	0.694	0.293	1.74E-60	MG-Periva:	1-Mar
LINC01686	9.47E-65	0.597711	0.268	0.047	2.67E-60	MG-Periva:	LINC01686

DDX213	1.36E-64	1.170973	0.815	0.408	3.84E-60	MG-Periva:	DDX21
SIPA1L22	1.52E-64	1.042883	0.664	0.249	4.27E-60	MG-Periva:	SIPA1L2
ID22	2.78E-64	1.282905	0.955	0.66	7.81E-60	MG-Periva:	ID2
TNFAIP8L3:	3.59E-64	1.143991	0.623	0.214	1.01E-59	MG-Periva:	TNFAIP8L3
LUCAT12	1.01E-63	0.989896	0.491	0.14	2.85E-59	MG-Periva:	LUCAT1
TJP1	1.28E-63	0.559977	0.264	0.046	3.61E-59	MG-Periva:	TJP1
IL4R	1.35E-63	0.975178	0.6	0.21	3.79E-59	MG-Periva:	IL4R
CD141	3.21E-63	1.653402	0.94	0.641	9.03E-59	MG-Periva:	CD14
GADD45B2	2.10E-62	1.188257	1	0.777	5.91E-58	MG-Periva:	GADD45B
UBE2F3	9.88E-62	1.118638	0.717	0.313	2.78E-57	MG-Periva:	UBE2F
KLHL63	1.79E-61	1.011755	0.864	0.43	5.04E-57	MG-Periva:	KLHL6
ABL22	1.83E-61	1.048829	0.83	0.383	5.14E-57	MG-Periva:	ABL2
SERTAD12	7.32E-61	1.124382	0.8	0.372	2.06E-56	MG-Periva:	SERTAD1
SFPQ2	8.19E-61	1.018881	0.951	0.632	2.30E-56	MG-Periva:	SFPQ
BCL2L12	1.16E-60	0.778554	0.574	0.191	3.27E-56	MG-Periva:	BCL2L1
IRF13	1.52E-60	1.173891	0.513	0.164	4.26E-56	MG-Periva:	IRF1
CEBPB2	2.45E-60	1.036613	0.985	0.697	6.89E-56	MG-Periva:	CEBPB
FOXP21	1.15E-59	0.675059	0.438	0.112	3.24E-55	MG-Periva:	FOXP2
FLVCR2	1.45E-59	0.592243	0.268	0.051	4.09E-55	MG-Periva:	FLVCR2
DDX51	1.68E-59	0.814678	1	0.902	4.72E-55	MG-Periva:	DDX5
KLF16	2.13E-59	0.640528	0.313	0.067	5.98E-55	MG-Periva:	KLF16
NRROS2	2.50E-59	0.833475	0.509	0.159	7.04E-55	MG-Periva:	NRROS
CCR11	2.55E-59	1.182311	0.57	0.206	7.17E-55	MG-Periva:	CCR1
PRKCH5	6.42E-59	1.04573	0.936	0.594	1.81E-54	MG-Periva:	PRKCH
ZNF460	1.23E-58	0.746571	0.336	0.077	3.47E-54	MG-Periva:	ZNF460
ACVR1B	1.31E-58	0.564868	0.287	0.058	3.69E-54	MG-Periva:	ACVR1B
MAFK	1.80E-58	0.559045	0.268	0.051	5.06E-54	MG-Periva:	MAFK
CEBPD3	2.28E-58	0.961254	0.992	0.871	6.41E-54	MG-Periva:	CEBPD
LAP3	3.56E-58	1.122889	0.785	0.392	1.00E-53	MG-Periva:	LAP3
FNIP22	6.60E-58	1.257906	0.751	0.37	1.86E-53	MG-Periva:	FNIP2
NDST2	6.69E-58	0.767431	0.392	0.104	1.88E-53	MG-Periva:	NDST2
SUSD63	7.01E-58	1.027798	0.838	0.434	1.97E-53	MG-Periva:	SUSD6
PGS11	7.53E-58	0.87315	0.426	0.12	2.12E-53	MG-Periva:	PGS1
CHST112	3.13E-57	0.995533	0.958	0.684	8.80E-53	MG-Periva:	CHST11
MTRNR2L1	3.52E-57	1.192535	0.955	0.498	9.92E-53	MG-Periva:	MTRNR2L12
NAB1	5.23E-57	0.715155	0.37	0.093	1.47E-52	MG-Periva:	NAB1
MAT2A1	6.51E-57	0.981903	0.932	0.549	1.83E-52	MG-Periva:	MAT2A
PPRC1	1.58E-56	0.675104	0.392	0.103	4.44E-52	MG-Periva:	PPRC1
ETS22	3.70E-56	1.04619	0.936	0.639	1.04E-51	MG-Periva:	ETS2
XBP13	8.16E-56	1.248671	0.853	0.493	2.30E-51	MG-Periva:	XBP1
ZFP363	1.29E-55	0.900924	1	0.931	3.62E-51	MG-Periva:	ZFP36
SOCS62	1.96E-55	0.978037	0.804	0.385	5.52E-51	MG-Periva:	SOCS6
STK102	4.28E-55	0.897858	0.608	0.231	1.20E-50	MG-Periva:	STK10
LINC02669	2.60E-54	0.741807	0.332	0.078	7.32E-50	MG-Periva:	LINC02669
SLC15A21	3.19E-54	0.731888	0.442	0.127	8.98E-50	MG-Periva:	SLC15A2
CDK64	5.83E-54	0.92037	0.653	0.259	1.64E-49	MG-Periva:	CDK6
NEK61	1.30E-53	0.953034	0.479	0.154	3.66E-49	MG-Periva:	NEK6

HIVEP23	1.45E-53	0.948633	0.691	0.3	4.07E-49	MG-Periva:	HIVEP2
AC008892.	1.94E-53	0.876568	0.347	0.086	5.45E-49	MG-Periva:	AC008892.1
MGAT52	3.03E-53	0.873571	0.706	0.304	8.54E-49	MG-Periva:	MGAT5
BTG21	3.58E-53	1.003924	0.97	0.744	1.01E-48	MG-Periva:	BTG2
CD632	6.18E-53	1.015132	0.936	0.655	1.74E-48	MG-Periva:	CD63
ADAMTS17	7.86E-53	0.807226	0.294	0.064	2.21E-48	MG-Periva:	ADAMTS17
HNRNPM1	1.45E-52	0.857674	0.751	0.362	4.07E-48	MG-Periva:	HNRNPM
MKNK12	4.08E-52	0.987459	0.706	0.327	1.15E-47	MG-Periva:	MKNK1
LONRF3	1.35E-51	0.857058	0.423	0.123	3.80E-47	MG-Periva:	LONRF3
CDC42BPB:	3.37E-51	0.775557	0.411	0.12	9.49E-47	MG-Periva:	CDC42BPB
ZNF410	3.79E-51	0.805998	0.445	0.141	1.07E-46	MG-Periva:	ZNF410
SH3GL11	7.78E-51	0.768814	0.487	0.161	2.19E-46	MG-Periva:	SH3GL1
MAPKAPK2	1.31E-50	0.843851	0.649	0.273	3.68E-46	MG-Periva:	MAPKAPK2
LINC01141	1.58E-50	0.685624	0.442	0.131	4.44E-46	MG-Periva:	LINC01141
GNAQ3	2.97E-50	0.887042	0.943	0.623	8.36E-46	MG-Periva:	GNAQ
GAB3	3.99E-50	0.809429	0.555	0.206	1.12E-45	MG-Periva:	GAB3
EML42	4.25E-50	0.956647	0.785	0.407	1.20E-45	MG-Periva:	EML4
RBM473	7.47E-50	0.926664	0.879	0.506	2.10E-45	MG-Periva:	RBM47
ITGAX5	1.01E-49	1.110484	0.789	0.414	2.85E-45	MG-Periva:	ITGAX
RNF1221	2.98E-49	0.720956	0.525	0.182	8.39E-45	MG-Periva:	RNF122
BZW13	3.06E-49	0.875742	0.755	0.387	8.61E-45	MG-Periva:	BZW1
DDAH21	1.48E-48	0.841937	0.634	0.269	4.16E-44	MG-Periva:	DDAH2
TMIGD33	2.11E-48	0.927225	0.796	0.395	5.93E-44	MG-Periva:	TMIGD3
ABI12	2.43E-48	0.88221	0.781	0.408	6.85E-44	MG-Periva:	ABI1
TRIB12	2.44E-48	1.077345	0.898	0.513	6.87E-44	MG-Periva:	TRIB1
LINC00513	2.54E-48	0.73783	0.366	0.101	7.16E-44	MG-Periva:	LINC00513
GNB41	3.43E-48	1.049565	0.808	0.46	9.64E-44	MG-Periva:	GNB4
METRNL5	6.05E-48	0.774327	0.543	0.195	1.70E-43	MG-Periva:	METRNL
SLC1A52	1.27E-47	0.904377	0.698	0.329	3.57E-43	MG-Periva:	SLC1A5
RASA32	2.42E-47	0.902197	0.743	0.376	6.80E-43	MG-Periva:	RASA3
MAP2K33	7.42E-47	0.969674	0.626	0.276	2.09E-42	MG-Periva:	MAP2K3
GSTM32	9.62E-47	1.005631	0.592	0.247	2.71E-42	MG-Periva:	GSTM3
SPACA6	1.96E-46	0.6623	0.317	0.081	5.52E-42	MG-Periva:	SPACA6
AL512625.:	2.12E-46	0.709777	0.374	0.107	5.97E-42	MG-Periva:	AL512625.3
KPNA41	2.76E-46	0.762243	0.638	0.277	7.76E-42	MG-Periva:	KPNA4
LIMS14	2.90E-46	0.85081	0.97	0.689	8.17E-42	MG-Periva:	LIMS1
ELL23	5.17E-46	0.80016	0.992	0.789	1.46E-41	MG-Periva:	ELL2
CCNL12	5.94E-46	0.846145	0.928	0.644	1.67E-41	MG-Periva:	CCNL1
PLK33	1.71E-45	0.90354	0.83	0.457	4.81E-41	MG-Periva:	PLK3
AC012150.	4.49E-45	0.782435	0.506	0.183	1.26E-40	MG-Periva:	AC012150.1
IRAK23	6.33E-45	0.787753	0.842	0.461	1.78E-40	MG-Periva:	IRAK2
DDX3X2	7.63E-45	0.853183	0.868	0.513	2.15E-40	MG-Periva:	DDX3X
CEP682	8.01E-45	0.81119	0.566	0.226	2.25E-40	MG-Periva:	CEP68
STOM	9.80E-45	1.125734	0.687	0.357	2.76E-40	MG-Periva:	STOM
H3F3A3	2.56E-44	0.811929	0.974	0.776	7.20E-40	MG-Periva:	H3F3A
MTSS13	3.23E-44	1.048739	0.672	0.317	9.10E-40	MG-Periva:	MTSS1
AC131944.	3.71E-44	0.996033	0.634	0.271	1.04E-39	MG-Periva:	AC131944.1

ATP13A33	4.15E-44	1.05791	0.675	0.329	1.17E-39	MG-Periva:	ATP13A3
NBL11	4.28E-44	0.775344	0.475	0.172	1.21E-39	MG-Periva:	NBL1
ARID5B2	1.24E-43	1.087121	0.777	0.417	3.50E-39	MG-Periva:	ARID5B
CD531	1.28E-43	0.85404	0.943	0.671	3.61E-39	MG-Periva:	CD53
SPATA2L	1.77E-43	0.625868	0.279	0.069	4.97E-39	MG-Periva:	SPATA2L
ABCB4	2.14E-43	0.593058	0.302	0.077	6.02E-39	MG-Periva:	ABCB4
HIST2H3PS	3.34E-43	0.499264	0.291	0.071	9.40E-39	MG-Periva:	HIST2H3PS2
NFIL32	3.65E-43	0.948833	0.675	0.322	1.03E-38	MG-Periva:	NFIL3
PEA152	4.68E-43	0.844493	0.634	0.295	1.32E-38	MG-Periva:	PEA15
SLC39A12	6.68E-43	0.798516	0.536	0.219	1.88E-38	MG-Periva:	SLC39A1
HK1	6.82E-43	0.70985	0.536	0.209	1.92E-38	MG-Periva:	HK1
SLC2A51	7.08E-43	0.932999	0.94	0.618	1.99E-38	MG-Periva:	SLC2A5
IL6R-AS1	8.07E-43	0.803657	0.291	0.074	2.27E-38	MG-Periva:	IL6R-AS1
CBFB1	1.30E-42	0.678299	0.525	0.198	3.65E-38	MG-Periva:	CBFB
MAFF1	1.62E-42	0.79141	0.6	0.249	4.56E-38	MG-Periva:	MAFF
ZNF2673	2.68E-42	0.956798	0.687	0.338	7.53E-38	MG-Periva:	ZNF267
PTPN14	4.74E-42	0.907574	0.762	0.408	1.33E-37	MG-Periva:	PTPN1
SLC16A33	5.60E-42	0.929287	0.725	0.374	1.58E-37	MG-Periva:	SLC16A3
TLR24	7.69E-42	0.920281	0.826	0.511	2.16E-37	MG-Periva:	TLR2
COQ71	9.68E-42	0.882007	0.392	0.129	2.72E-37	MG-Periva:	COQ7
STAMBPL1	9.80E-42	0.693358	0.449	0.155	2.76E-37	MG-Periva:	STAMBPL1
ADGRG12	1.71E-41	0.805714	0.717	0.343	4.82E-37	MG-Periva:	ADGRG1
LILRB42	2.26E-41	0.86614	0.936	0.664	6.35E-37	MG-Periva:	LILRB4
SEC14L11	2.70E-41	0.873579	0.732	0.371	7.60E-37	MG-Periva:	SEC14L1
YBX31	8.74E-41	0.938816	0.74	0.401	2.46E-36	MG-Periva:	YBX3
FOSL1	1.34E-40	0.605414	0.317	0.089	3.76E-36	MG-Periva:	FOSL1
KIF21B1	1.38E-40	0.74677	0.385	0.123	3.87E-36	MG-Periva:	KIF21B
SLC3A23	3.53E-40	0.828132	0.74	0.381	9.94E-36	MG-Periva:	SLC3A2
PPP1R3B	4.49E-40	0.544732	0.272	0.069	1.26E-35	MG-Periva:	PPP1R3B
CD993	1.14E-39	0.780203	0.898	0.586	3.22E-35	MG-Periva:	CD99
VASP2	1.43E-39	0.722747	0.57	0.245	4.04E-35	MG-Periva:	VASP
NOTCH1	5.02E-39	0.569224	0.287	0.076	1.41E-34	MG-Periva:	NOTCH1
GCA1	6.21E-39	0.826632	0.558	0.247	1.75E-34	MG-Periva:	GCA
SPP14	8.33E-39	1.055456	0.977	0.832	2.34E-34	MG-Periva:	SPP1
CLIC14	9.70E-39	0.792483	0.928	0.658	2.73E-34	MG-Periva:	CLIC1
ACTG13	1.32E-38	0.946537	0.951	0.791	3.73E-34	MG-Periva:	ACTG1
ERF1	2.02E-38	0.664508	0.498	0.196	5.69E-34	MG-Periva:	ERF
PPIF1	3.01E-38	0.912055	0.536	0.23	8.47E-34	MG-Periva:	PPIF
ARHGEF73	3.03E-38	0.783148	0.638	0.301	8.53E-34	MG-Periva:	ARHGEF7
MIR22HG	3.59E-38	0.653187	0.343	0.107	1.01E-33	MG-Periva:	MIR22HG
BIN11	4.49E-38	0.796358	0.906	0.604	1.26E-33	MG-Periva:	BIN1
EIF4A13	4.55E-38	0.683821	0.977	0.802	1.28E-33	MG-Periva:	EIF4A1
GPCPD14	7.36E-38	0.947801	0.717	0.392	2.07E-33	MG-Periva:	GPCPD1
CCL4L23	8.18E-38	1.077609	0.974	0.786	2.30E-33	MG-Periva:	CCL4L2
ANXA54	1.05E-37	0.786201	0.928	0.633	2.96E-33	MG-Periva:	ANXA5
PHC21	1.09E-37	0.799215	0.785	0.442	3.08E-33	MG-Periva:	PHC2
LDHA4	1.70E-37	0.890439	0.789	0.452	4.79E-33	MG-Periva:	LDHA

CSNK1D1	2.05E-37	0.710999	0.683	0.347	5.77E-33	MG-Periva:	CSNK1D
TFRC1	2.22E-37	0.910179	0.672	0.325	6.26E-33	MG-Periva:	TFRC
VAPA2	3.16E-37	0.744346	0.815	0.487	8.88E-33	MG-Periva:	VAPA
LONRF11	4.21E-37	0.535544	0.328	0.097	1.19E-32	MG-Periva:	LONRF1
BCOR1	9.44E-37	0.58864	0.347	0.11	2.66E-32	MG-Periva:	BCOR
RAB5A	1.40E-36	0.671324	0.596	0.277	3.93E-32	MG-Periva:	RAB5A
NAA201	1.84E-36	0.794079	0.608	0.294	5.19E-32	MG-Periva:	NAA20
PAG13	5.40E-36	0.736184	0.77	0.436	1.52E-31	MG-Periva:	PAG1
VPS37B1	7.68E-36	0.613327	0.608	0.277	2.16E-31	MG-Periva:	VPS37B
C3AR13	8.48E-36	0.867247	0.898	0.634	2.39E-31	MG-Periva:	C3AR1
TPM31	8.64E-36	0.745981	0.917	0.636	2.43E-31	MG-Periva:	TPM3
PPP1R181	9.22E-36	0.788768	0.6	0.293	2.59E-31	MG-Periva:	PPP1R18
LPL2	9.57E-36	0.72543	0.332	0.102	2.69E-31	MG-Periva:	LPL
FOSL24	1.02E-35	0.78406	0.513	0.213	2.88E-31	MG-Periva:	FOSL2
RAB202	1.44E-35	0.83739	0.792	0.458	4.05E-31	MG-Periva:	RAB20
GRASP5	1.59E-35	0.725553	0.909	0.549	4.47E-31	MG-Periva:	GRASP
ANKH1	2.69E-35	0.543871	0.423	0.151	7.57E-31	MG-Periva:	ANKH
KIFC3	2.74E-35	0.69272	0.306	0.093	7.72E-31	MG-Periva:	KIFC3
SLC44A22	3.82E-35	0.673118	0.475	0.19	1.08E-30	MG-Periva:	SLC44A2
OSM1	5.54E-35	0.875575	0.642	0.306	1.56E-30	MG-Periva:	OSM
VMP13	5.72E-35	0.779518	0.766	0.453	1.61E-30	MG-Periva:	VMP1
TLN23	8.90E-35	0.647809	0.547	0.237	2.50E-30	MG-Periva:	TLN2
EIF4E4	1.05E-34	0.917824	0.766	0.434	2.96E-30	MG-Periva:	EIF4E
CEP1702	1.89E-34	0.659357	0.796	0.459	5.31E-30	MG-Periva:	CEP170
SH3TC12	3.63E-34	0.708234	0.74	0.415	1.02E-29	MG-Periva:	SH3TC1
BTG32	3.74E-34	0.787946	0.562	0.257	1.05E-29	MG-Periva:	BTG3
GABARAPL	4.60E-34	0.704505	0.642	0.317	1.29E-29	MG-Periva:	GABARAPL1
SRGAP12	9.70E-34	0.762834	0.751	0.426	2.73E-29	MG-Periva:	SRGAP1
SESN2	1.09E-33	0.583025	0.275	0.079	3.06E-29	MG-Periva:	SESN2
PEAK14	1.12E-33	0.668431	0.857	0.499	3.15E-29	MG-Periva:	PEAK1
ELF4	1.48E-33	0.481868	0.283	0.082	4.16E-29	MG-Periva:	ELF4
PLAUR4	1.53E-33	0.845536	0.883	0.536	4.30E-29	MG-Periva:	PLAUR
IPCEF14	2.04E-33	0.608269	0.891	0.508	5.73E-29	MG-Periva:	IPCEF1
ASAP14	7.02E-33	0.829155	0.502	0.221	1.98E-28	MG-Periva:	ASAP1
RHEB2	1.02E-32	0.715409	0.758	0.456	2.88E-28	MG-Periva:	RHEB
KLF64	1.21E-32	0.653075	0.996	0.896	3.41E-28	MG-Periva:	KLF6
SLC7A53	1.26E-32	0.591747	0.74	0.387	3.55E-28	MG-Periva:	SLC7A5
ARHGAP62	1.46E-32	0.771947	0.615	0.306	4.10E-28	MG-Periva:	ARHGAP6
SLC20A12	1.46E-32	0.634086	0.475	0.199	4.12E-28	MG-Periva:	SLC20A1
AL357522.:	1.51E-32	0.539889	0.257	0.071	4.26E-28	MG-Periva:	AL357522.1
EIF4G11	2.88E-32	0.583861	0.54	0.244	8.11E-28	MG-Periva:	EIF4G1
PPP3R11	4.10E-32	0.6023	0.566	0.264	1.15E-27	MG-Periva:	PPP3R1
AP5B1	4.26E-32	0.466756	0.275	0.081	1.20E-27	MG-Periva:	AP5B1
GOLPH31	5.31E-32	0.656091	0.502	0.223	1.49E-27	MG-Periva:	GOLPH3
ICAM12	6.44E-32	0.724669	0.664	0.331	1.81E-27	MG-Periva:	ICAM1
LINC01353	6.83E-32	0.534047	0.317	0.103	1.92E-27	MG-Periva:	LINC01353
RBMS3	8.01E-32	0.635514	0.332	0.108	2.25E-27	MG-Periva:	RBMS3

HINT3	9.52E-32	0.429075	0.275	0.081	2.68E-27	MG-Periva:	HINT3
ERCC12	1.00E-31	0.7212	0.868	0.582	2.82E-27	MG-Periva:	ERCC1
ATF61	1.16E-31	0.724011	0.725	0.408	3.26E-27	MG-Periva:	ATF6
PDE4DIP2	1.28E-31	0.740277	0.596	0.293	3.59E-27	MG-Periva:	PDE4DIP
EHD41	1.57E-31	0.592414	0.472	0.198	4.41E-27	MG-Periva:	EHD4
AC022509.	2.31E-31	0.658838	0.358	0.127	6.49E-27	MG-Periva:	AC022509.3
MTHFD2	2.88E-31	0.902275	0.623	0.351	8.10E-27	MG-Periva:	MTHFD2
LINC00963	5.58E-31	0.633485	0.581	0.276	1.57E-26	MG-Periva:	LINC00963
NOLC1	5.86E-31	0.559186	0.396	0.152	1.65E-26	MG-Periva:	NOLC1
CTTNBP23	6.15E-31	0.79762	0.683	0.382	1.73E-26	MG-Periva:	CTTNBP2
PLEKHO21	7.02E-31	0.568542	0.502	0.22	1.98E-26	MG-Periva:	PLEKHO2
SNX94	7.17E-31	0.640256	0.864	0.548	2.02E-26	MG-Periva:	SNX9
SWAP70	8.01E-31	0.647057	0.717	0.408	2.25E-26	MG-Periva:	SWAP70
RAB1A3	9.14E-31	0.565333	0.872	0.534	2.57E-26	MG-Periva:	RAB1A
DLGAP41	1.14E-30	0.558397	0.385	0.142	3.20E-26	MG-Periva:	DLGAP4
PCNX12	1.46E-30	0.685058	0.423	0.171	4.10E-26	MG-Periva:	PCNX1
MPRIP3	2.30E-30	0.651538	0.513	0.233	6.47E-26	MG-Periva:	MPRIP
RASL11A1	2.62E-30	0.562441	0.287	0.087	7.38E-26	MG-Periva:	RASL11A
HMGA13	2.93E-30	0.714314	0.494	0.222	8.24E-26	MG-Periva:	HMGA1
UBE2D32	2.99E-30	0.750018	0.875	0.657	8.42E-26	MG-Periva:	UBE2D3
IFNGR24	3.64E-30	0.650575	0.853	0.545	1.02E-25	MG-Periva:	IFNGR2
LINC01128	3.82E-30	0.527066	0.336	0.117	1.08E-25	MG-Periva:	LINC01128
ZYX5	5.74E-30	0.713538	0.732	0.445	1.62E-25	MG-Periva:	ZYX
SERPINE14	8.21E-30	0.711263	0.77	0.414	2.31E-25	MG-Periva:	SERPINE1
YWHAG1	8.43E-30	0.572475	0.509	0.229	2.37E-25	MG-Periva:	YWHAG
AC015912.	1.24E-29	0.564332	0.264	0.08	3.50E-25	MG-Periva:	AC015912.3
PCBP13	1.81E-29	0.620832	0.721	0.409	5.09E-25	MG-Periva:	PCBP1
UPP13	2.39E-29	0.644808	0.362	0.133	6.71E-25	MG-Periva:	UPP1
C5AR13	2.58E-29	0.7856	0.838	0.52	7.25E-25	MG-Periva:	C5AR1
ATP1A11	4.07E-29	0.635522	0.717	0.408	1.14E-24	MG-Periva:	ATP1A1
HNRNPF1	4.89E-29	0.575628	0.755	0.455	1.38E-24	MG-Periva:	HNRNPF
SKIL3	1.21E-28	0.610997	0.921	0.674	3.41E-24	MG-Periva:	SKIL
MYO1E3	1.24E-28	0.685941	0.66	0.356	3.49E-24	MG-Periva:	MYO1E
APP1	2.22E-28	0.77115	0.389	0.157	6.24E-24	MG-Periva:	APP
OTUD13	2.89E-28	0.790806	0.849	0.563	8.14E-24	MG-Periva:	OTUD1
WDR12	3.84E-28	0.558107	0.626	0.337	1.08E-23	MG-Periva:	WDR1
PISD	4.13E-28	0.443771	0.325	0.114	1.16E-23	MG-Periva:	PISD
KBTBD2	4.43E-28	0.521166	0.366	0.14	1.25E-23	MG-Periva:	KBTBD2
GPR137B1	4.54E-28	0.679099	0.408	0.169	1.28E-23	MG-Periva:	GPR137B
PFKFB32	5.25E-28	0.714444	0.838	0.567	1.48E-23	MG-Periva:	PFKFB3
UBE2J12	5.73E-28	0.635012	0.551	0.273	1.61E-23	MG-Periva:	UBE2J1
MALAT12	6.01E-28	0.668065	1	0.989	1.69E-23	MG-Periva:	MALAT1
MT1X1	7.09E-28	1.259836	0.37	0.151	2.00E-23	MG-Periva:	MT1X
HECTD2	8.09E-28	0.624077	0.4	0.161	2.28E-23	MG-Periva:	HECTD2
PDE3B3	1.29E-27	0.548083	0.751	0.439	3.62E-23	MG-Periva:	PDE3B
CEBPA2	1.82E-27	0.858367	0.623	0.356	5.13E-23	MG-Periva:	CEBPA
ITGB51	1.89E-27	0.520514	0.377	0.145	5.32E-23	MG-Periva:	ITGB5

PTTG1IP2	1.91E-27	0.694508	0.657	0.387	5.37E-23	MG-Periva:	PTTG1IP
LINC01480	2.10E-27	1.001465	0.37	0.146	5.91E-23	MG-Periva:	LINC01480
TTYH32	3.07E-27	0.531669	0.558	0.273	8.65E-23	MG-Periva:	TTYH3
IL6R2	4.31E-27	0.68413	0.634	0.357	1.21E-22	MG-Periva:	IL6R
CD841	4.45E-27	0.630821	0.698	0.403	1.25E-22	MG-Periva:	CD84
SLC25A251	6.09E-27	0.609029	0.419	0.177	1.71E-22	MG-Periva:	SLC25A25
ST6GALNA(	6.23E-27	0.53547	0.864	0.556	1.75E-22	MG-Periva:	ST6GALNAC3
NET1	6.66E-27	0.706437	0.366	0.144	1.87E-22	MG-Periva:	NET1
SLC30A11	6.79E-27	0.500244	0.279	0.092	1.91E-22	MG-Periva:	SLC30A1
ARID3A1	7.55E-27	0.509824	0.317	0.113	2.12E-22	MG-Periva:	ARID3A
ARFGAP31	9.33E-27	0.528316	0.442	0.193	2.62E-22	MG-Periva:	ARFGAP3
NOTCH21	9.43E-27	0.521703	0.343	0.129	2.65E-22	MG-Periva:	NOTCH2
RHOH4	1.15E-26	0.77571	0.517	0.254	3.22E-22	MG-Periva:	RHOH
SPTLC23	1.41E-26	0.597482	0.875	0.6	3.97E-22	MG-Periva:	SPTLC2
CHCHD7	1.64E-26	0.797549	0.483	0.239	4.62E-22	MG-Periva:	CHCHD7
ZBTB46	1.71E-26	0.479882	0.291	0.098	4.82E-22	MG-Periva:	ZBTB46
FCGR2A1	2.11E-26	0.636463	0.887	0.627	5.94E-22	MG-Periva:	FCGR2A
OLR13	2.89E-26	0.509238	0.996	0.791	8.13E-22	MG-Periva:	OLR1
JUNB3	3.02E-26	0.551423	0.992	0.888	8.49E-22	MG-Periva:	JUNB
TBC1D2B	3.57E-26	0.592375	0.396	0.167	1.00E-21	MG-Periva:	TBC1D2B
MCL12	5.32E-26	0.569236	0.992	0.82	1.50E-21	MG-Periva:	MCL1
ARHGAP31	5.38E-26	0.551496	0.46	0.211	1.51E-21	MG-Periva:	ARHGAP31
NANS1	6.51E-26	0.602776	0.585	0.313	1.83E-21	MG-Periva:	NANS
BHLHE412	7.81E-26	0.785268	0.804	0.538	2.20E-21	MG-Periva:	BHLHE41
LPAR11	8.41E-26	0.53421	0.472	0.214	2.37E-21	MG-Periva:	LPAR1
NFE2L24	1.08E-25	0.595321	0.721	0.458	3.04E-21	MG-Periva:	NFE2L2
JDP24	1.35E-25	0.635429	0.834	0.57	3.79E-21	MG-Periva:	JDP2
BCL24	1.61E-25	0.546157	0.66	0.362	4.52E-21	MG-Periva:	BCL2
CDC372	1.82E-25	0.522906	0.74	0.443	5.11E-21	MG-Periva:	CDC37
TNFRSF212	1.86E-25	0.492992	0.309	0.11	5.22E-21	MG-Periva:	TNFRSF21
ZDHHC14	2.54E-25	0.547821	0.404	0.172	7.14E-21	MG-Periva:	ZDHHC14
CCL3L12	2.59E-25	0.751428	0.966	0.74	7.29E-21	MG-Periva:	CCL3L1
PTPN61	2.66E-25	0.666684	0.675	0.411	7.48E-21	MG-Periva:	PTPN6
AFDN1	2.74E-25	0.450467	0.268	0.089	7.71E-21	MG-Periva:	AFDN
RTN43	3.54E-25	0.549176	0.913	0.631	9.96E-21	MG-Periva:	RTN4
R3HDM4	3.96E-25	0.52096	0.283	0.1	1.11E-20	MG-Periva:	R3HDM4
PRRG41	4.29E-25	0.511438	0.377	0.155	1.21E-20	MG-Periva:	PRRG4
EIF51	4.32E-25	0.653876	0.898	0.651	1.21E-20	MG-Periva:	EIF5
DDX39A1	4.46E-25	0.551034	0.585	0.307	1.25E-20	MG-Periva:	DDX39A
HRH2	4.88E-25	0.598625	0.328	0.127	1.37E-20	MG-Periva:	HRH2
CDK171	5.42E-25	0.442276	0.355	0.138	1.53E-20	MG-Periva:	CDK17
ANKRD10	5.65E-25	0.679803	0.615	0.358	1.59E-20	MG-Periva:	ANKRD10
RBM7	7.40E-25	0.45094	0.313	0.116	2.08E-20	MG-Periva:	RBM7
ARHGDIA2	8.99E-25	0.551502	0.691	0.408	2.53E-20	MG-Periva:	ARHGDIA
LPP2	1.20E-24	0.547167	0.785	0.502	3.38E-20	MG-Periva:	LPP
DIP2A2	1.30E-24	0.519901	0.494	0.233	3.67E-20	MG-Periva:	DIP2A
UBE2N1	1.30E-24	0.556656	0.536	0.281	3.67E-20	MG-Periva:	UBE2N

IPO71	1.36E-24	0.513124	0.494	0.24	3.83E-20	MG-Periva:	IPO7
SMG13	1.45E-24	0.633374	0.502	0.251	4.07E-20	MG-Periva:	SMG1
PPARG1	1.51E-24	0.53025	0.381	0.155	4.24E-20	MG-Periva:	PPARG
ITM2C2	1.61E-24	0.557899	0.517	0.26	4.54E-20	MG-Periva:	ITM2C
CAMSAP11	1.91E-24	0.494572	0.325	0.123	5.36E-20	MG-Periva:	CAMSAP1
ATP6V1B2:	2.80E-24	0.671447	0.743	0.475	7.89E-20	MG-Periva:	ATP6V1B2
ARHGEF40:	3.27E-24	0.558384	0.442	0.204	9.20E-20	MG-Periva:	ARHGEF40
ABCA12	3.58E-24	0.720354	0.687	0.4	1.01E-19	MG-Periva:	ABCA1
PDXK3	4.04E-24	0.600687	0.619	0.338	1.14E-19	MG-Periva:	PDXK
UGCG3	4.05E-24	0.644045	0.498	0.248	1.14E-19	MG-Periva:	UGCG
NRIP11	5.90E-24	0.64313	0.683	0.419	1.66E-19	MG-Periva:	NRIP1
SORCS1	5.95E-24	0.615617	0.275	0.095	1.67E-19	MG-Periva:	SORCS1
SSPN2	7.17E-24	0.737741	0.574	0.317	2.02E-19	MG-Periva:	SSPN
TAL12	7.27E-24	0.543328	0.611	0.326	2.04E-19	MG-Periva:	TAL1
CDC42SE12	7.68E-24	0.576162	0.657	0.386	2.16E-19	MG-Periva:	CDC42SE1
PER2	8.20E-24	0.493044	0.287	0.103	2.31E-19	MG-Periva:	PER2
METAP1	8.51E-24	0.369737	0.268	0.091	2.39E-19	MG-Periva:	METAP1
ANKRD113	8.90E-24	0.511137	0.808	0.538	2.50E-19	MG-Periva:	ANKRD11
FHL31	1.14E-23	0.550366	0.381	0.164	3.21E-19	MG-Periva:	FHL3
SRSF22	1.15E-23	0.574666	0.804	0.531	3.24E-19	MG-Periva:	SRSF2
ARGLU12	1.17E-23	0.529189	0.758	0.476	3.29E-19	MG-Periva:	ARGLU1
ABI21	1.47E-23	0.397213	0.313	0.118	4.15E-19	MG-Periva:	ABI2
RHOB4	1.49E-23	0.568167	0.981	0.826	4.18E-19	MG-Periva:	RHOB
LGMN3	1.65E-23	0.483567	0.725	0.424	4.65E-19	MG-Periva:	LGMN
SGK14	2.29E-23	0.575803	0.985	0.862	6.44E-19	MG-Periva:	SGK1
TGIF13	2.33E-23	0.682277	0.506	0.256	6.56E-19	MG-Periva:	TGIF1
UAP1	2.71E-23	0.430948	0.343	0.135	7.63E-19	MG-Periva:	UAP1
PURB	4.34E-23	0.441128	0.317	0.124	1.22E-18	MG-Periva:	PURB
LINC01091	5.58E-23	0.472818	0.457	0.204	1.57E-18	MG-Periva:	LINC01091
SLC11A2	7.31E-23	0.525307	0.453	0.22	2.06E-18	MG-Periva:	SLC11A2
TENT4A1	8.25E-23	0.433073	0.26	0.09	2.32E-18	MG-Periva:	TENT4A
GTPBP4	9.44E-23	0.39528	0.347	0.142	2.66E-18	MG-Periva:	GTPBP4
USP32	1.04E-22	0.473255	0.426	0.189	2.93E-18	MG-Periva:	USP32
TPD52L2	1.04E-22	0.427104	0.362	0.154	2.93E-18	MG-Periva:	TPD52L2
P2RY123	1.26E-22	0.641002	0.796	0.524	3.54E-18	MG-Periva:	P2RY12
RFX23	1.46E-22	0.651744	0.547	0.295	4.10E-18	MG-Periva:	RFX2
PHF1	1.60E-22	0.35272	0.279	0.1	4.50E-18	MG-Periva:	PHF1
SGMS11	1.68E-22	0.550141	0.4	0.178	4.74E-18	MG-Periva:	SGMS1
TBC1D141	1.92E-22	0.580682	0.525	0.276	5.39E-18	MG-Periva:	TBC1D14
TRAK12	3.33E-22	0.460197	0.449	0.211	9.37E-18	MG-Periva:	TRAK1
ARHGAP12	4.08E-22	0.600095	0.525	0.28	1.15E-17	MG-Periva:	ARHGAP12
HNRNPAB1	4.09E-22	0.494906	0.558	0.304	1.15E-17	MG-Periva:	HNRNPAB
QSOX11	5.14E-22	0.477596	0.426	0.205	1.45E-17	MG-Periva:	QSOX1
ASXL11	7.90E-22	0.524486	0.434	0.206	2.22E-17	MG-Periva:	ASXL1
NCOA7	8.41E-22	0.882109	0.381	0.177	2.37E-17	MG-Periva:	NCOA7
TLNRD1	8.75E-22	0.40622	0.264	0.095	2.46E-17	MG-Periva:	TLNRD1
HBEGF3	9.87E-22	0.600841	0.574	0.3	2.78E-17	MG-Periva:	HBEGF

RASAL22	1.32E-21	0.690179	0.608	0.354	3.73E-17	MG-Periva:	RASAL2
MAP1S1	1.44E-21	0.615432	0.438	0.215	4.04E-17	MG-Periva:	MAP1S
CXCL164	1.45E-21	0.539947	0.872	0.643	4.07E-17	MG-Periva:	CXCL16
ALAS11	1.54E-21	0.539795	0.351	0.151	4.33E-17	MG-Periva:	ALAS1
PGK13	2.02E-21	0.528111	0.77	0.513	5.69E-17	MG-Periva:	PGK1
SLC10A3	2.23E-21	0.406273	0.264	0.095	6.26E-17	MG-Periva:	SLC10A3
SAMSN13	2.29E-21	0.695841	0.834	0.602	6.45E-17	MG-Periva:	SAMSN1
ADAM91	2.59E-21	0.411724	0.517	0.263	7.28E-17	MG-Periva:	ADAM9
TWSG1	2.76E-21	0.457387	0.328	0.133	7.77E-17	MG-Periva:	TWSG1
FAM133B1	3.26E-21	0.485855	0.487	0.249	9.18E-17	MG-Periva:	FAM133B
MB21D22	5.75E-21	0.453076	0.479	0.238	1.62E-16	MG-Periva:	MB21D2
LIMD23	6.10E-21	0.740392	0.562	0.336	1.72E-16	MG-Periva:	LIMD2
RANBP91	7.02E-21	0.557927	0.679	0.415	1.98E-16	MG-Periva:	RANBP9
GAS62	7.96E-21	0.520007	0.506	0.267	2.24E-16	MG-Periva:	GAS6
NUDT31	1.10E-20	0.56849	0.694	0.449	3.10E-16	MG-Periva:	NUDT3
IVNS1ABP2	1.16E-20	0.647211	0.611	0.366	3.26E-16	MG-Periva:	IVNS1ABP
CCNT1	1.29E-20	0.573705	0.404	0.191	3.62E-16	MG-Periva:	CCNT1
SH3GLB12	1.29E-20	0.667636	0.475	0.255	3.63E-16	MG-Periva:	SH3GLB1
SLC12A6	1.40E-20	0.511845	0.34	0.145	3.93E-16	MG-Periva:	SLC12A6
MTCH11	1.46E-20	0.488923	0.611	0.36	4.10E-16	MG-Periva:	MTCH1
PTK2	1.85E-20	0.354499	0.332	0.136	5.21E-16	MG-Periva:	PTK2
PRELID13	1.98E-20	0.514328	0.509	0.272	5.58E-16	MG-Periva:	PRELID1
GLS2	2.42E-20	0.491493	0.725	0.477	6.82E-16	MG-Periva:	GLS
HNRNPA2E	2.67E-20	0.460885	0.966	0.816	7.51E-16	MG-Periva:	HNRNPA2B1
FGR3	2.80E-20	0.552901	0.336	0.144	7.87E-16	MG-Periva:	FGR
KPNB11	3.32E-20	0.542172	0.54	0.309	9.34E-16	MG-Periva:	KPNB1
SRSF51	4.04E-20	0.535125	0.879	0.63	1.14E-15	MG-Periva:	SRSF5
IRF2BP23	4.09E-20	0.553882	0.509	0.274	1.15E-15	MG-Periva:	IRF2BP2
MFSD122	4.14E-20	0.45837	0.498	0.267	1.17E-15	MG-Periva:	MFSD12
SLC7A12	5.02E-20	0.498689	0.351	0.154	1.41E-15	MG-Periva:	SLC7A1
CRY1	5.47E-20	0.439697	0.415	0.197	1.54E-15	MG-Periva:	CRY1
ANKRD22	5.53E-20	0.571047	0.404	0.194	1.56E-15	MG-Periva:	ANKRD22
PRRC2A1	6.30E-20	0.393767	0.468	0.236	1.77E-15	MG-Periva:	PRRC2A
AFTPH1	6.83E-20	0.482498	0.457	0.231	1.92E-15	MG-Periva:	AFTPH
TMED53	8.33E-20	0.468386	0.592	0.345	2.34E-15	MG-Periva:	TMED5
AC008957.	8.35E-20	0.515654	0.4	0.192	2.35E-15	MG-Periva:	AC008957.1
PPP4R1	8.43E-20	0.36666	0.291	0.115	2.37E-15	MG-Periva:	PPP4R1
ACTR31	8.79E-20	0.454824	0.736	0.472	2.47E-15	MG-Periva:	ACTR3
RIPOR11	9.78E-20	0.493552	0.298	0.123	2.75E-15	MG-Periva:	RIPOR1
RHBDF22	1.29E-19	0.501106	0.883	0.653	3.62E-15	MG-Periva:	RHBDF2
USP532	1.29E-19	0.584496	0.698	0.436	3.64E-15	MG-Periva:	USP53
COLGALT1	1.49E-19	0.497116	0.385	0.185	4.18E-15	MG-Periva:	COLGALT1
NFKBID3	1.64E-19	0.547442	0.766	0.508	4.62E-15	MG-Periva:	NFKBID
CCL32	2.23E-19	0.685223	0.977	0.87	6.29E-15	MG-Periva:	CCL3
EIF14	2.29E-19	0.37039	0.996	0.924	6.43E-15	MG-Periva:	EIF1
RASSF52	2.43E-19	0.46919	0.63	0.369	6.83E-15	MG-Periva:	RASSF5
LRRC591	2.87E-19	0.377345	0.374	0.172	8.07E-15	MG-Periva:	LRRC59

BAZ1A2	3.08E-19	0.517246	0.502	0.278	8.65E-15	MG-Perivas	BAZ1A
SOCS12	3.29E-19	0.658139	0.317	0.136	9.26E-15	MG-Perivas	SOCS1
ST6GAL13	5.00E-19	0.410343	0.868	0.65	1.41E-14	MG-Perivas	ST6GAL1
CHD12	5.74E-19	0.416549	0.694	0.431	1.62E-14	MG-Perivas	CHD1
DNAJC2	5.81E-19	0.433622	0.347	0.155	1.63E-14	MG-Perivas	DNAJC2
USP9X2	6.41E-19	0.578506	0.502	0.278	1.80E-14	MG-Perivas	USP9X
KCNQ32	6.58E-19	0.308504	0.785	0.502	1.85E-14	MG-Perivas	KCNQ3
PAFAH1B1	7.66E-19	0.408235	0.649	0.393	2.16E-14	MG-Perivas	PAFAH1B1
HTRA13	8.28E-19	0.535715	0.887	0.628	2.33E-14	MG-Perivas	HTRA1
SERTAD2	8.71E-19	0.50566	0.392	0.188	2.45E-14	MG-Perivas	SERTAD2
LUZP11	8.92E-19	0.422754	0.37	0.171	2.51E-14	MG-Perivas	LUZP1
PLD42	9.51E-19	0.486799	0.864	0.618	2.68E-14	MG-Perivas	PLD4
ZFAND32	1.00E-18	0.411276	0.774	0.502	2.82E-14	MG-Perivas	ZFAND3
TNFRSF1B2	1.01E-18	0.483533	0.789	0.521	2.85E-14	MG-Perivas	TNFRSF1B
CEBPZ1	1.09E-18	0.377064	0.355	0.16	3.07E-14	MG-Perivas	CEBPZ
AKT32	1.28E-18	0.372047	0.494	0.251	3.60E-14	MG-Perivas	AKT3
DHX151	1.32E-18	0.438441	0.411	0.204	3.73E-14	MG-Perivas	DHX15
IKZF12	1.42E-18	0.560956	0.668	0.428	4.00E-14	MG-Perivas	IKZF1
DNTTIP2	1.49E-18	0.489953	0.513	0.29	4.19E-14	MG-Perivas	DNTTIP2
SPIDR3	1.52E-18	0.531526	0.725	0.491	4.26E-14	MG-Perivas	SPIDR
DGKA1	1.55E-18	0.411713	0.351	0.16	4.35E-14	MG-Perivas	DGKA
HAVCR22	1.59E-18	0.675311	0.781	0.569	4.49E-14	MG-Perivas	HAVCR2
RAP1B3	1.62E-18	0.440165	0.66	0.423	4.57E-14	MG-Perivas	RAP1B
FERMT33	1.64E-18	0.551318	0.528	0.301	4.61E-14	MG-Perivas	FERMT3
MAP3K111	1.70E-18	0.475358	0.358	0.169	4.79E-14	MG-Perivas	MAP3K11
POMP3	1.93E-18	0.467488	0.766	0.511	5.43E-14	MG-Perivas	POMP
HNRNPK1	2.07E-18	0.457526	0.808	0.592	5.84E-14	MG-Perivas	HNRNPK
RABEP11	2.24E-18	0.398225	0.449	0.226	6.30E-14	MG-Perivas	RABEP1
IRF2BPL	2.61E-18	0.4316	0.268	0.107	7.33E-14	MG-Perivas	IRF2BPL
BACH12	2.83E-18	0.537692	0.619	0.381	7.97E-14	MG-Perivas	BACH1
SMAD31	3.35E-18	0.471234	0.434	0.219	9.42E-14	MG-Perivas	SMAD3
EIF3A1	4.02E-18	0.466505	0.604	0.373	1.13E-13	MG-Perivas	EIF3A
OTUD4	4.38E-18	0.482119	0.355	0.166	1.23E-13	MG-Perivas	OTUD4
ALPK3	4.47E-18	0.586117	0.298	0.126	1.26E-13	MG-Perivas	ALPK3
SHTN12	4.69E-18	0.441401	0.683	0.424	1.32E-13	MG-Perivas	SHTN1
TMEM87B	5.16E-18	0.385639	0.298	0.125	1.45E-13	MG-Perivas	TMEM87B
PLSCR12	6.03E-18	0.481009	0.717	0.475	1.70E-13	MG-Perivas	PLSCR1
RASSF81	6.64E-18	0.460687	0.309	0.133	1.87E-13	MG-Perivas	RASSF8
LAMP11	7.62E-18	0.455887	0.683	0.443	2.14E-13	MG-Perivas	LAMP1
NR3C12	9.62E-18	0.398435	0.585	0.342	2.71E-13	MG-Perivas	NR3C1
SPRY13	9.63E-18	0.614964	0.472	0.252	2.71E-13	MG-Perivas	SPRY1
ARPC24	9.75E-18	0.488596	0.883	0.637	2.74E-13	MG-Perivas	ARPC2
GLUL1	1.03E-17	0.641785	0.936	0.761	2.90E-13	MG-Perivas	GLUL
ZMYND111	1.11E-17	0.390465	0.389	0.187	3.14E-13	MG-Perivas	ZMYND11
IDI11	1.12E-17	0.463367	0.502	0.277	3.14E-13	MG-Perivas	IDI1
SMAD71	1.17E-17	0.351034	0.468	0.242	3.29E-13	MG-Perivas	SMAD7
CREM3	1.29E-17	0.440805	0.777	0.521	3.62E-13	MG-Perivas	CREM

KLF91	1.32E-17	0.408248	0.362	0.169	3.73E-13	MG-Periva:	KLF9
NUP982	1.45E-17	0.380435	0.536	0.304	4.07E-13	MG-Periva:	NUP98
RGS14	1.53E-17	0.516643	0.977	0.841	4.30E-13	MG-Periva:	RGS1
SIGLEC101	1.58E-17	0.480742	0.585	0.351	4.43E-13	MG-Periva:	SIGLEC10
CKB3	1.78E-17	0.483998	0.657	0.395	5.01E-13	MG-Periva:	CKB
ACSL4	1.91E-17	0.457597	0.449	0.24	5.39E-13	MG-Periva:	ACSL4
HIST2H2BF	1.96E-17	0.386482	0.321	0.14	5.52E-13	MG-Periva:	HIST2H2BF
SIGLEC9	2.17E-17	0.468606	0.351	0.169	6.10E-13	MG-Periva:	SIGLEC9
TARS	2.42E-17	0.351321	0.257	0.103	6.81E-13	MG-Periva:	TARS
A2M3	2.42E-17	0.525377	0.925	0.751	6.82E-13	MG-Periva:	A2M
DENND4C2	2.43E-17	0.433863	0.498	0.272	6.83E-13	MG-Periva:	DENND4C
WLS	3.32E-17	0.330111	0.26	0.103	9.35E-13	MG-Periva:	WLS
PRDX64	3.42E-17	0.538777	0.562	0.349	9.62E-13	MG-Periva:	PRDX6
YWHAZ3	4.16E-17	0.447201	0.823	0.595	1.17E-12	MG-Periva:	YWHAZ
RUBCNL1	5.09E-17	0.541779	0.302	0.134	1.43E-12	MG-Periva:	RUBCNL
ZNF8001	5.37E-17	0.390124	0.411	0.207	1.51E-12	MG-Periva:	ZNF800
RAN3	5.93E-17	0.392385	0.713	0.458	1.67E-12	MG-Periva:	RAN
BCL7B1	5.94E-17	0.4096	0.404	0.205	1.67E-12	MG-Periva:	BCL7B
FBXL51	6.14E-17	0.427792	0.4	0.203	1.73E-12	MG-Periva:	FBXL5
RBM33	6.30E-17	0.45652	0.743	0.478	1.77E-12	MG-Periva:	RBM3
CD300C	6.94E-17	0.441585	0.291	0.128	1.95E-12	MG-Periva:	CD300C
SMURF11	7.07E-17	0.42899	0.374	0.18	1.99E-12	MG-Periva:	SMURF1
ELF13	7.12E-17	0.404984	0.902	0.67	2.00E-12	MG-Periva:	ELF1
EBI31	7.67E-17	0.452035	0.494	0.277	2.16E-12	MG-Periva:	EBI3
FYTTD12	8.01E-17	0.336828	0.37	0.179	2.25E-12	MG-Periva:	FYTTD1
COPB11	8.15E-17	0.425405	0.453	0.245	2.29E-12	MG-Periva:	COPB1
JAZF13	8.45E-17	0.500169	0.747	0.507	2.38E-12	MG-Periva:	JAZF1
AP003086.	1.05E-16	0.497041	0.423	0.224	2.95E-12	MG-Periva:	AP003086.1
H3F3B2	1.09E-16	0.278932	0.996	0.917	3.07E-12	MG-Periva:	H3F3B
RHOC3	1.09E-16	0.554179	0.453	0.255	3.08E-12	MG-Periva:	RHOC
PIK3R12	1.11E-16	0.508353	0.747	0.523	3.14E-12	MG-Periva:	PIK3R1
CALM13	1.23E-16	0.544833	0.834	0.643	3.47E-12	MG-Periva:	CALM1
ITPRIP2	1.24E-16	0.420703	0.543	0.307	3.50E-12	MG-Periva:	ITPRIP
FKBP53	1.62E-16	0.368465	0.928	0.708	4.57E-12	MG-Periva:	FKBP5
FUS2	1.67E-16	0.447776	0.838	0.612	4.71E-12	MG-Periva:	FUS
CAPZB1	1.82E-16	0.41277	0.94	0.759	5.13E-12	MG-Periva:	CAPZB
TIPARP	2.01E-16	0.568639	0.43	0.231	5.64E-12	MG-Periva:	TIPARP
ITPRIPL2	2.19E-16	0.388703	0.26	0.108	6.17E-12	MG-Periva:	ITPRIPL2
AKIRIN11	2.51E-16	0.370415	0.4	0.204	7.07E-12	MG-Periva:	AKIRIN1
CCL42	2.69E-16	0.752045	0.977	0.852	7.58E-12	MG-Periva:	CCL4
RCOR13	2.84E-16	0.364019	0.43	0.224	7.99E-12	MG-Periva:	RCOR1
STAU1	3.60E-16	0.358254	0.404	0.21	1.01E-11	MG-Periva:	STAU1
SF12	3.78E-16	0.3781	0.77	0.544	1.06E-11	MG-Periva:	SF1
SLC38A22	3.80E-16	0.494772	0.713	0.486	1.07E-11	MG-Periva:	SLC38A2
CEP3502	4.27E-16	0.433694	0.589	0.366	1.20E-11	MG-Periva:	CEP350
MAPRE11	5.13E-16	0.402948	0.46	0.258	1.44E-11	MG-Periva:	MAPRE1
EGR22	5.14E-16	0.571081	0.774	0.504	1.45E-11	MG-Periva:	EGR2

PRKCB4	5.30E-16	0.406992	0.449	0.242	1.49E-11	MG-Periva:	PRKCB
SERPINB91	5.79E-16	0.392791	0.891	0.637	1.63E-11	MG-Periva:	SERPINB9
AGO31	6.94E-16	0.366445	0.43	0.227	1.95E-11	MG-Periva:	AGO3
USP121	7.71E-16	0.38834	0.325	0.153	2.17E-11	MG-Periva:	USP12
SBDS	9.18E-16	0.377675	0.464	0.259	2.58E-11	MG-Periva:	SBDS
C21	1.04E-15	0.377882	0.287	0.125	2.93E-11	MG-Periva:	C2
TUT72	1.18E-15	0.486328	0.547	0.325	3.32E-11	MG-Periva:	TUT7
CSF2RA4	1.28E-15	0.377133	0.898	0.676	3.59E-11	MG-Periva:	CSF2RA
TPM43	1.39E-15	0.302392	0.679	0.423	3.91E-11	MG-Periva:	TPM4
RCC22	1.41E-15	0.350324	0.592	0.357	3.96E-11	MG-Periva:	RCC2
TMBIM12	1.53E-15	0.421911	0.479	0.272	4.29E-11	MG-Periva:	TMBIM1
DUSP52	1.53E-15	0.575593	0.313	0.146	4.30E-11	MG-Periva:	DUSP5
ZNF276	1.66E-15	0.314209	0.275	0.119	4.67E-11	MG-Periva:	ZNF276
AC008592.	1.82E-15	0.315751	0.264	0.111	5.13E-11	MG-Periva:	AC008592.1
WDR912	1.95E-15	0.364294	0.491	0.274	5.49E-11	MG-Periva:	WDR91
GNAI31	2.02E-15	0.411386	0.498	0.292	5.67E-11	MG-Periva:	GNAI3
ZNF2071	2.21E-15	0.342077	0.6	0.367	6.23E-11	MG-Periva:	ZNF207
SETD31	2.32E-15	0.360903	0.355	0.177	6.51E-11	MG-Periva:	SETD3
ITGA52	2.59E-15	0.577266	0.347	0.174	7.28E-11	MG-Periva:	ITGA5
ADM1	2.68E-15	0.42907	0.294	0.131	7.53E-11	MG-Periva:	ADM
GSPT12	3.06E-15	0.318396	0.487	0.273	8.62E-11	MG-Periva:	GSPT1
N4BP2L11	3.24E-15	0.51148	0.272	0.121	9.12E-11	MG-Periva:	N4BP2L1
LITAF5	3.28E-15	0.395912	0.687	0.446	9.22E-11	MG-Periva:	LITAF
SLC25A321	5.58E-15	0.27328	0.374	0.185	1.57E-10	MG-Periva:	SLC25A32
KCMF1	5.79E-15	0.366039	0.325	0.159	1.63E-10	MG-Periva:	KCMF1
NLRP36	6.06E-15	0.411109	0.755	0.52	1.71E-10	MG-Periva:	NLRP3
BCLAF12	6.27E-15	0.345007	0.619	0.392	1.76E-10	MG-Periva:	BCLAF1
PPP6R32	8.01E-15	0.314982	0.479	0.269	2.26E-10	MG-Periva:	PPP6R3
CCDC2001	8.57E-15	0.590295	0.453	0.251	2.41E-10	MG-Periva:	CCDC200
MAN1A13	1.06E-14	0.318991	0.672	0.429	2.98E-10	MG-Periva:	MAN1A1
ASXL21	1.11E-14	0.323246	0.336	0.163	3.11E-10	MG-Periva:	ASXL2
TMEM65	1.11E-14	0.34088	0.37	0.188	3.13E-10	MG-Periva:	TMEM65
WBP4	1.19E-14	0.415436	0.294	0.138	3.34E-10	MG-Periva:	WBP4
ATP2A21	1.31E-14	0.328748	0.517	0.301	3.68E-10	MG-Periva:	ATP2A2
REL3	1.33E-14	0.504688	0.921	0.749	3.74E-10	MG-Periva:	REL
SMU1	1.35E-14	0.30157	0.408	0.217	3.79E-10	MG-Periva:	SMU1
SPAG93	1.44E-14	0.300519	0.619	0.383	4.05E-10	MG-Periva:	SPAG9
CD835	1.54E-14	0.3223	1	0.912	4.34E-10	MG-Periva:	CD83
MIR222HG	1.55E-14	0.449556	0.494	0.29	4.36E-10	MG-Periva:	MIR222HG
AL390957.:	1.72E-14	0.400844	0.551	0.332	4.85E-10	MG-Periva:	AL390957.1
RTTN1	1.94E-14	0.535847	0.294	0.136	5.45E-10	MG-Periva:	RTTN
CDV32	1.94E-14	0.38508	0.615	0.397	5.47E-10	MG-Periva:	CDV3
PITPNB1	1.99E-14	0.363901	0.358	0.183	5.59E-10	MG-Periva:	PITPNB
GK3	2.00E-14	0.435326	0.589	0.36	5.62E-10	MG-Periva:	GK
PNPLA82	2.35E-14	0.378842	0.498	0.289	6.62E-10	MG-Periva:	PNPLA8
ATXN2L1	2.80E-14	0.41043	0.419	0.236	7.89E-10	MG-Periva:	ATXN2L
LAPTM51	2.87E-14	0.317312	0.989	0.898	8.08E-10	MG-Periva:	LAPTM5

U2AF2	3.80E-14	0.302096	0.321	0.156	1.07E-09	MG-Periva:	U2AF2
FMN13	3.82E-14	0.498817	0.623	0.406	1.08E-09	MG-Periva:	FMN1
ADRM11	3.83E-14	0.388898	0.434	0.253	1.08E-09	MG-Periva:	ADRM1
PFDN21	3.93E-14	0.396921	0.483	0.284	1.10E-09	MG-Periva:	PFDN2
MAN2A12	4.08E-14	0.499429	0.57	0.372	1.15E-09	MG-Periva:	MAN2A1
ZBTB7A1	4.13E-14	0.418533	0.351	0.183	1.16E-09	MG-Periva:	ZBTB7A
SH2B35	4.19E-14	0.352068	0.596	0.369	1.18E-09	MG-Periva:	SH2B3
CSNK1A13	4.81E-14	0.394296	0.721	0.501	1.35E-09	MG-Periva:	CSNK1A1
RNF1494	5.25E-14	0.433214	0.777	0.564	1.48E-09	MG-Periva:	RNF149
AHI11	5.28E-14	0.573676	0.302	0.147	1.49E-09	MG-Periva:	AHI1
AL135905.:	5.29E-14	0.301691	0.396	0.21	1.49E-09	MG-Periva:	AL135905.2
DUSP13	5.36E-14	0.33367	0.996	0.943	1.51E-09	MG-Periva:	DUSP1
CSF1R1	5.43E-14	0.428282	0.962	0.801	1.53E-09	MG-Periva:	CSF1R
GDI11	5.65E-14	0.418771	0.434	0.252	1.59E-09	MG-Periva:	GDI1
PACSIN22	6.93E-14	0.453696	0.547	0.353	1.95E-09	MG-Periva:	PACSIN2
RPS101	7.80E-14	0.333202	0.257	0.114	2.20E-09	MG-Periva:	RPS10
HAT1	7.90E-14	0.292866	0.291	0.136	2.22E-09	MG-Periva:	HAT1
RLIM	8.65E-14	0.341287	0.472	0.273	2.43E-09	MG-Periva:	RLIM
DTX2	9.51E-14	0.367574	0.309	0.153	2.68E-09	MG-Periva:	DTX2
ECD	1.02E-13	0.332446	0.415	0.227	2.88E-09	MG-Periva:	ECD
GNA133	1.20E-13	0.288663	0.838	0.603	3.38E-09	MG-Periva:	GNA13
ISG20L2	1.23E-13	0.374153	0.358	0.186	3.47E-09	MG-Periva:	ISG20L2
AC253572.	1.34E-13	0.408302	0.551	0.329	3.77E-09	MG-Periva:	AC253572.2
RAB5C1	1.35E-13	0.3591	0.649	0.433	3.79E-09	MG-Periva:	RAB5C
ITGA9	1.35E-13	0.362616	0.283	0.132	3.79E-09	MG-Periva:	ITGA9
KAT6A1	1.36E-13	0.304609	0.385	0.205	3.83E-09	MG-Periva:	KAT6A
KHDRBS11	1.36E-13	0.366565	0.562	0.36	3.84E-09	MG-Periva:	KHDRBS1
MAML22	1.46E-13	0.525569	0.751	0.552	4.10E-09	MG-Periva:	MAML2
RBM14	1.47E-13	0.282051	0.306	0.147	4.13E-09	MG-Periva:	RBM14
SNRPB1	1.53E-13	0.404371	0.525	0.336	4.30E-09	MG-Periva:	SNRPB
PRXL2C2	1.56E-13	0.376088	0.434	0.246	4.40E-09	MG-Periva:	PRXL2C
FBXW113	1.75E-13	0.290649	0.558	0.339	4.91E-09	MG-Periva:	FBXW11
KCTD51	1.76E-13	0.386362	0.279	0.132	4.96E-09	MG-Periva:	KCTD5
TBC1D161	1.93E-13	0.306342	0.396	0.211	5.42E-09	MG-Periva:	TBC1D16
DDX39B1	1.99E-13	0.358279	0.589	0.381	5.61E-09	MG-Periva:	DDX39B
NCOR22	2.81E-13	0.338479	0.679	0.451	7.90E-09	MG-Periva:	NCOR2
CHD72	3.12E-13	0.356477	0.521	0.314	8.78E-09	MG-Periva:	CHD7
ATP11A	3.14E-13	0.462547	0.351	0.184	8.85E-09	MG-Periva:	ATP11A
TWF22	3.87E-13	0.400827	0.509	0.313	1.09E-08	MG-Periva:	TWF2
EIF1B3	4.13E-13	0.326099	0.555	0.348	1.16E-08	MG-Periva:	EIF1B
RBMX2	4.31E-13	0.318054	0.638	0.417	1.21E-08	MG-Periva:	RBMX
PTBP11	4.44E-13	0.370912	0.426	0.246	1.25E-08	MG-Periva:	PTBP1
SLC30A7	6.03E-13	0.417185	0.257	0.121	1.70E-08	MG-Periva:	SLC30A7
MAGT11	6.23E-13	0.355078	0.442	0.257	1.75E-08	MG-Periva:	MAGT1
EHBP1L11	6.27E-13	0.473754	0.37	0.208	1.77E-08	MG-Periva:	EHBP1L1
IER5L2	6.35E-13	0.403927	0.475	0.283	1.79E-08	MG-Periva:	IER5L
SGTA1	6.78E-13	0.297509	0.279	0.133	1.91E-08	MG-Periva:	SGTA

ETV62	7.13E-13	0.354888	0.758	0.55	2.01E-08	MG-Periva:	ETV6
ITGAV3	7.31E-13	0.436918	0.657	0.453	2.06E-08	MG-Periva:	ITGAV
CPSF61	7.61E-13	0.327401	0.404	0.226	2.14E-08	MG-Periva:	CPSF6
GNAS2	7.79E-13	0.370644	0.921	0.715	2.19E-08	MG-Periva:	GNAS
TP53I111	7.80E-13	0.326162	0.317	0.16	2.20E-08	MG-Periva:	TP53I11
USP111	8.04E-13	0.346907	0.362	0.194	2.26E-08	MG-Periva:	USP11
QSER1	8.19E-13	0.266596	0.257	0.116	2.31E-08	MG-Periva:	QSER1
CLTC1	8.34E-13	0.308756	0.558	0.355	2.35E-08	MG-Periva:	CLTC
KMT5A1	8.81E-13	0.372932	0.268	0.128	2.48E-08	MG-Periva:	KMT5A
ARF63	8.95E-13	0.363751	0.502	0.307	2.52E-08	MG-Periva:	ARF6
ST3GAL11	9.18E-13	0.316986	0.408	0.228	2.58E-08	MG-Periva:	ST3GAL1
PDK43	9.96E-13	0.536861	0.796	0.612	2.80E-08	MG-Periva:	PDK4
GPN31	1.08E-12	0.394382	0.408	0.231	3.04E-08	MG-Periva:	GPN3
HNRNPA31	1.18E-12	0.34034	0.762	0.552	3.32E-08	MG-Periva:	HNRNPA3
MED14	1.26E-12	0.280383	0.253	0.115	3.55E-08	MG-Periva:	MED14
ARF12	1.29E-12	0.309433	0.774	0.542	3.62E-08	MG-Periva:	ARF1
TM6SF11	1.33E-12	0.374031	0.679	0.482	3.75E-08	MG-Periva:	TM6SF1
AEBP2	1.40E-12	0.268015	0.26	0.12	3.95E-08	MG-Periva:	AEBP2
HNRNPU1	1.49E-12	0.32455	0.804	0.621	4.21E-08	MG-Periva:	HNRNPU
TWF1	1.50E-12	0.322412	0.264	0.125	4.21E-08	MG-Periva:	TWF1
GPR343	1.51E-12	0.512994	0.83	0.64	4.25E-08	MG-Periva:	GPR34
ANKLE21	1.64E-12	0.280588	0.317	0.16	4.60E-08	MG-Periva:	ANKLE2
EWSR11	1.65E-12	0.339552	0.634	0.431	4.63E-08	MG-Periva:	EWSR1
DENND5A3	1.69E-12	0.334822	0.442	0.257	4.75E-08	MG-Periva:	DENND5A
GABARAPL	1.73E-12	0.448131	0.626	0.432	4.87E-08	MG-Periva:	GABARAPL2
LTBP3	1.78E-12	0.358834	0.253	0.119	5.01E-08	MG-Periva:	LTBP3
GATAD2A1	1.96E-12	0.311705	0.298	0.149	5.50E-08	MG-Periva:	GATAD2A
CDC731	2.35E-12	0.288132	0.408	0.231	6.60E-08	MG-Periva:	CDC73
MAP4K44	2.64E-12	0.299815	0.589	0.372	7.43E-08	MG-Periva:	MAP4K4
JMJD62	2.87E-12	0.337995	0.445	0.259	8.08E-08	MG-Periva:	JMJD6
PSMD12	2.89E-12	0.368391	0.336	0.181	8.13E-08	MG-Periva:	PSMD12
SLC11A13	3.11E-12	0.352476	0.849	0.635	8.74E-08	MG-Periva:	SLC11A1
MAPK1IP1L	3.57E-12	0.3108	0.494	0.305	1.00E-07	MG-Periva:	MAPK1IP1L
XPR12	4.23E-12	0.354247	0.389	0.216	1.19E-07	MG-Periva:	XPR1
KBTBD8	4.72E-12	0.346118	0.283	0.141	1.33E-07	MG-Periva:	KBTBD8
CRK1	5.15E-12	0.331888	0.325	0.171	1.45E-07	MG-Periva:	CRK
ELK31	5.44E-12	0.37998	0.358	0.199	1.53E-07	MG-Periva:	ELK3
TMEM117	5.62E-12	0.299354	0.325	0.167	1.58E-07	MG-Periva:	TMEM117
RRBP13	5.65E-12	0.348388	0.457	0.279	1.59E-07	MG-Periva:	RRBP1
C16orf72	5.70E-12	0.281419	0.343	0.182	1.60E-07	MG-Periva:	C16orf72
RAB21	5.99E-12	0.283692	0.389	0.22	1.68E-07	MG-Periva:	RAB21
MAP4K34	6.05E-12	0.357487	0.725	0.511	1.70E-07	MG-Periva:	MAP4K3
LINC01970	6.21E-12	0.454347	0.306	0.157	1.75E-07	MG-Periva:	LINC01970
RBM8A1	6.46E-12	0.321446	0.577	0.377	1.82E-07	MG-Periva:	RBM8A
RELA1	7.67E-12	0.332428	0.313	0.166	2.16E-07	MG-Periva:	RELA
CTDSP11	8.07E-12	0.277197	0.528	0.325	2.27E-07	MG-Periva:	CTDSP1
OFD12	8.16E-12	0.394672	0.404	0.234	2.30E-07	MG-Periva:	OFD1

CTSC1	8.42E-12	0.51935	0.755	0.59	2.37E-07	MG-Periva:	CTSC
NONO	9.76E-12	0.270343	0.468	0.281	2.75E-07	MG-Periva:	NONO
SKI2	1.01E-11	0.291643	0.574	0.378	2.85E-07	MG-Periva:	SKI
RAP1A2	1.20E-11	0.307327	0.751	0.544	3.39E-07	MG-Periva:	RAP1A
CDC14B2	1.30E-11	0.312275	0.306	0.156	3.65E-07	MG-Periva:	CDC14B
EIF4G22	1.33E-11	0.327961	0.706	0.511	3.74E-07	MG-Periva:	EIF4G2
ARRB21	1.37E-11	0.349358	0.83	0.616	3.85E-07	MG-Periva:	ARRB2
GSTM21	1.39E-11	0.32394	0.483	0.296	3.91E-07	MG-Periva:	GSTM2
ATF32	1.46E-11	0.334384	0.815	0.584	4.11E-07	MG-Periva:	ATF3
FMNL12	1.48E-11	0.286362	0.562	0.355	4.16E-07	MG-Periva:	FMNL1
FAR2	1.58E-11	0.280114	0.392	0.22	4.46E-07	MG-Periva:	FAR2
EMD2	1.84E-11	0.327871	0.449	0.278	5.18E-07	MG-Periva:	EMD
PDGFB3	1.87E-11	0.333104	0.555	0.357	5.25E-07	MG-Periva:	PDGFB
EIF2S1	1.91E-11	0.297623	0.268	0.134	5.36E-07	MG-Periva:	EIF2S1
CYTH41	1.97E-11	0.374799	0.4	0.236	5.53E-07	MG-Periva:	CYTH4
ACADVL2	2.00E-11	0.376649	0.46	0.296	5.62E-07	MG-Periva:	ACADVL
SOD23	2.13E-11	0.434902	0.8	0.581	6.00E-07	MG-Periva:	SOD2
SARNP2	2.33E-11	0.275105	0.442	0.266	6.54E-07	MG-Periva:	SARNP
RALY1	2.38E-11	0.351227	0.619	0.436	6.70E-07	MG-Periva:	RALY
SELENOS4	2.97E-11	0.385949	0.426	0.261	8.35E-07	MG-Periva:	SELENOS
ADAM172	3.38E-11	0.324594	0.668	0.46	9.52E-07	MG-Periva:	ADAM17
FAM102B2	3.50E-11	0.310812	0.445	0.267	9.84E-07	MG-Periva:	FAM102B
KDELR22	3.60E-11	0.322905	0.479	0.301	1.01E-06	MG-Periva:	KDELR2
STK38L2	3.89E-11	0.281001	0.536	0.33	1.10E-06	MG-Periva:	STK38L
VSIG42	4.07E-11	0.409351	0.732	0.533	1.15E-06	MG-Periva:	VSIG4
EIF3I	4.45E-11	0.343263	0.426	0.266	1.25E-06	MG-Periva:	EIF3I
KLHL51	4.98E-11	0.338327	0.487	0.31	1.40E-06	MG-Periva:	KLHL5
HIPK32	5.18E-11	0.369985	0.445	0.277	1.46E-06	MG-Periva:	HIPK3
ODC13	6.00E-11	0.299106	0.468	0.287	1.69E-06	MG-Periva:	ODC1
ALOX52	6.28E-11	0.53746	0.634	0.472	1.77E-06	MG-Periva:	ALOX5
LHFPL22	6.28E-11	0.255515	0.86	0.641	1.77E-06	MG-Periva:	LHFPL2
YTHDF3	6.64E-11	0.306396	0.396	0.235	1.87E-06	MG-Periva:	YTHDF3
NPL2	7.65E-11	0.329048	0.517	0.337	2.15E-06	MG-Periva:	NPL
CDK2AP11	7.77E-11	0.431727	0.558	0.38	2.19E-06	MG-Periva:	CDK2AP1
SRRT	8.03E-11	0.259906	0.291	0.151	2.26E-06	MG-Periva:	SRRT
UFM12	9.83E-11	0.305972	0.385	0.229	2.77E-06	MG-Periva:	UFM1
CTNNA11	1.01E-10	0.270543	0.449	0.282	2.83E-06	MG-Periva:	CTNNA1
DNAJC5	1.05E-10	0.270499	0.283	0.146	2.94E-06	MG-Periva:	DNAJC5
SEC16A	1.17E-10	0.300111	0.279	0.146	3.28E-06	MG-Periva:	SEC16A
AGO22	1.24E-10	0.273536	0.313	0.17	3.48E-06	MG-Periva:	AGO2
PTPN122	1.29E-10	0.300525	0.317	0.173	3.63E-06	MG-Periva:	PTPN12
ARMCX31	1.36E-10	0.293414	0.381	0.223	3.84E-06	MG-Periva:	ARMCX3
PPP2CA1	1.39E-10	0.299007	0.543	0.362	3.91E-06	MG-Periva:	PPP2CA
ZNF577	1.39E-10	0.305092	0.294	0.153	3.92E-06	MG-Periva:	ZNF577
ITGB12	1.54E-10	0.258487	0.54	0.357	4.33E-06	MG-Periva:	ITGB1
CUL31	1.59E-10	0.259907	0.442	0.271	4.47E-06	MG-Periva:	CUL3
CUL1	1.73E-10	0.250874	0.381	0.224	4.88E-06	MG-Periva:	CUL1

FKBP1A2	1.77E-10	0.309507	0.457	0.293	4.99E-06	MG-Periva:	FKBP1A
SUN21	1.83E-10	0.310444	0.306	0.165	5.14E-06	MG-Periva:	SUN2
LTC4S2	1.84E-10	0.322064	0.725	0.512	5.18E-06	MG-Periva:	LTC4S
EIF1AX1	1.91E-10	0.267702	0.491	0.314	5.39E-06	MG-Periva:	EIF1AX
C12orf752	1.98E-10	0.273014	0.419	0.249	5.56E-06	MG-Periva:	C12orf75
SAT14	2.03E-10	0.38878	1	0.97	5.70E-06	MG-Periva:	SAT1
RNF1451	2.03E-10	0.410471	0.404	0.248	5.71E-06	MG-Periva:	RNF145
AC002451.	2.16E-10	0.269252	0.272	0.135	6.07E-06	MG-Periva:	AC002451.1
PTMS2	2.16E-10	0.309202	0.408	0.248	6.08E-06	MG-Periva:	PTMS
GEM	2.16E-10	0.426001	0.264	0.132	6.09E-06	MG-Periva:	GEM
SLC66A21	2.18E-10	0.286744	0.343	0.195	6.14E-06	MG-Periva:	SLC66A2
FBXO112	2.34E-10	0.256383	0.543	0.36	6.59E-06	MG-Periva:	FBXO11
RPS6KA31	2.36E-10	0.317328	0.513	0.34	6.63E-06	MG-Periva:	RPS6KA3
GCH11	2.42E-10	0.350761	0.343	0.192	6.82E-06	MG-Periva:	GCH1
LYST1	2.66E-10	0.397369	0.385	0.228	7.49E-06	MG-Periva:	LYST
NCOA33	2.84E-10	0.399488	0.509	0.338	7.99E-06	MG-Periva:	NCOA3
NUMB3	2.85E-10	0.280719	0.551	0.372	8.03E-06	MG-Periva:	NUMB
ACSL34	3.29E-10	0.288627	0.426	0.261	9.27E-06	MG-Periva:	ACSL3
POGZ1	3.33E-10	0.286754	0.374	0.221	9.36E-06	MG-Periva:	POGZ
DNM21	3.36E-10	0.260177	0.604	0.416	9.45E-06	MG-Periva:	DNM2
NUDT4	3.50E-10	0.405751	0.325	0.18	9.84E-06	MG-Periva:	NUDT4
FPR31	3.61E-10	0.294492	0.34	0.192	1.02E-05	MG-Periva:	FPR3
PMEPA14	3.69E-10	0.319901	0.536	0.355	1.04E-05	MG-Periva:	PMEPA1
ATP6V0A1:	3.89E-10	0.309096	0.396	0.237	1.10E-05	MG-Periva:	ATP6V0A1
NCL2	3.91E-10	0.271434	0.8	0.597	1.10E-05	MG-Periva:	NCL
TRIP123	4.06E-10	0.28807	0.472	0.302	1.14E-05	MG-Periva:	TRIP12
SEC24B	4.07E-10	0.286828	0.298	0.161	1.15E-05	MG-Periva:	SEC24B
RNF168	4.70E-10	0.280036	0.291	0.156	1.32E-05	MG-Periva:	RNF168
PTPRE4	4.73E-10	0.43802	0.668	0.507	1.33E-05	MG-Periva:	PTPRE
EP400	5.03E-10	0.300942	0.291	0.158	1.41E-05	MG-Periva:	EP400
TCF122	5.35E-10	0.289271	0.74	0.559	1.50E-05	MG-Periva:	TCF12
GPRIN32	5.37E-10	0.406464	0.415	0.262	1.51E-05	MG-Periva:	GPRIN3
AL136987.:	5.46E-10	0.30657	0.468	0.295	1.54E-05	MG-Periva:	AL136987.1
TMEM52B:	5.48E-10	0.268217	0.491	0.314	1.54E-05	MG-Periva:	TMEM52B
IFITM10	6.00E-10	0.379573	0.389	0.237	1.69E-05	MG-Periva:	IFITM10
PRKAB11	6.67E-10	0.297689	0.366	0.214	1.88E-05	MG-Periva:	PRKAB1
RNH13	7.00E-10	0.346077	0.502	0.34	1.97E-05	MG-Periva:	RNH1
SON1	7.67E-10	0.258433	0.917	0.702	2.16E-05	MG-Periva:	SON
YTHDF2	7.86E-10	0.287558	0.389	0.236	2.21E-05	MG-Periva:	YTHDF2
ILF31	7.93E-10	0.260269	0.46	0.297	2.23E-05	MG-Periva:	ILF3
TGFB11	7.94E-10	0.325926	0.815	0.674	2.23E-05	MG-Periva:	TGFB1
E2F41	9.07E-10	0.255661	0.317	0.178	2.55E-05	MG-Periva:	E2F4
ADGRE22	9.22E-10	0.363453	0.366	0.217	2.59E-05	MG-Periva:	ADGRE2
NPTN1	9.86E-10	0.313478	0.377	0.229	2.77E-05	MG-Periva:	NPTN
CCNY2	1.02E-09	0.305861	0.494	0.338	2.87E-05	MG-Periva:	CCNY
XRN2	1.06E-09	0.299192	0.445	0.288	2.98E-05	MG-Periva:	XRN2
ING1	1.08E-09	0.329674	0.279	0.15	3.03E-05	MG-Periva:	ING1

DNAJA2	1.27E-09	0.278519	0.336	0.196	3.58E-05	MG-Periva:	DNAJA2
SAMD4B	1.30E-09	0.253893	0.283	0.153	3.66E-05	MG-Periva:	SAMD4B
SRSF41	1.36E-09	0.286543	0.475	0.316	3.82E-05	MG-Periva:	SRSF4
HMOX13	1.37E-09	0.452931	0.743	0.546	3.86E-05	MG-Periva:	HMOX1
NOL71	1.42E-09	0.335014	0.521	0.357	3.98E-05	MG-Periva:	NOL7
MBD6	1.43E-09	0.281398	0.26	0.136	4.01E-05	MG-Periva:	MBD6
FUNDC2	1.46E-09	0.304642	0.423	0.264	4.10E-05	MG-Periva:	FUNDC2
ARL8A2	1.52E-09	0.284552	0.336	0.195	4.28E-05	MG-Periva:	ARL8A
ARHGEF2	1.54E-09	0.361944	0.343	0.204	4.33E-05	MG-Periva:	ARHGEF2
HNRNPH31	1.57E-09	0.276934	0.509	0.338	4.42E-05	MG-Periva:	HNRNPH3
RAB8B	1.69E-09	0.253908	0.347	0.203	4.74E-05	MG-Periva:	RAB8B
RRAGA1	1.71E-09	0.411254	0.377	0.232	4.81E-05	MG-Periva:	RRAGA
AL078590.:	1.79E-09	0.320327	0.645	0.44	5.05E-05	MG-Periva:	AL078590.2
ARPC5L1	1.85E-09	0.261577	0.253	0.132	5.21E-05	MG-Periva:	ARPC5L
SZRD1	1.93E-09	0.275396	0.302	0.171	5.44E-05	MG-Periva:	SZRD1
BAIAP22	1.96E-09	0.317393	0.362	0.215	5.52E-05	MG-Periva:	BAIAP2
NT5C21	2.17E-09	0.280224	0.43	0.272	6.10E-05	MG-Periva:	NT5C2
DELE11	2.33E-09	0.275428	0.362	0.217	6.56E-05	MG-Periva:	DELE1
ELL	2.96E-09	0.250564	0.302	0.17	8.33E-05	MG-Periva:	ELL
EIF4H1	3.11E-09	0.254773	0.551	0.372	8.76E-05	MG-Periva:	EIF4H
SPIN11	3.33E-09	0.257864	0.302	0.17	9.36E-05	MG-Periva:	SPIN1
ILF21	3.49E-09	0.257518	0.449	0.292	9.81E-05	MG-Periva:	ILF2
ARL8B3	3.80E-09	0.322867	0.491	0.332	0.000107	MG-Periva:	ARL8B
SH3BP21	4.45E-09	0.287089	0.362	0.221	0.000125	MG-Periva:	SH3BP2
KLF43	4.50E-09	0.329164	0.694	0.51	0.000127	MG-Periva:	KLF4
PNPLA21	4.63E-09	0.281589	0.502	0.342	0.00013	MG-Periva:	PNPLA2
JAK13	4.82E-09	0.357873	0.623	0.447	0.000136	MG-Periva:	JAK1
TCEAL31	4.90E-09	0.267082	0.34	0.2	0.000138	MG-Periva:	TCEAL3
MANF2	5.07E-09	0.265992	0.26	0.14	0.000143	MG-Periva:	MANF
ALDOA5	5.15E-09	0.259047	0.626	0.457	0.000145	MG-Periva:	ALDOA
SLCO2B13	6.74E-09	0.266137	0.728	0.546	0.00019	MG-Periva:	SLCO2B1
HCK1	7.45E-09	0.287807	0.513	0.343	0.00021	MG-Periva:	HCK
PUF601	7.79E-09	0.301184	0.374	0.238	0.000219	MG-Periva:	PUF60
AC243829.	7.97E-09	0.27095	0.317	0.183	0.000224	MG-Periva:	AC243829.4
BCAP313	8.41E-09	0.349728	0.611	0.454	0.000237	MG-Periva:	BCAP31
VDAC12	9.03E-09	0.302368	0.445	0.3	0.000254	MG-Periva:	VDAC1
AFF12	1.04E-08	0.314159	0.46	0.302	0.000293	MG-Periva:	AFF1
MERTK2	1.06E-08	0.266146	0.577	0.409	0.000298	MG-Periva:	MERTK
CASS41	1.07E-08	0.33542	0.374	0.233	0.000302	MG-Periva:	CASS4
GNA151	1.11E-08	0.282937	0.325	0.195	0.000312	MG-Periva:	GNA15
EGR32	1.14E-08	0.296624	0.657	0.458	0.00032	MG-Periva:	EGR3
MACF12	1.14E-08	0.308881	0.604	0.447	0.000322	MG-Periva:	MACF1
MRC21	1.16E-08	0.262862	0.306	0.174	0.000326	MG-Periva:	MRC2
RAPH11	1.23E-08	0.269598	0.479	0.319	0.000345	MG-Periva:	RAPH1
PSMA12	1.28E-08	0.298377	0.63	0.459	0.000361	MG-Periva:	PSMA1
DEK	1.32E-08	0.272725	0.536	0.38	0.000371	MG-Periva:	DEK
PLD32	1.33E-08	0.270905	0.513	0.348	0.000374	MG-Periva:	PLD3

NAIP	1.47E-08	0.351459	0.509	0.359	0.000413	MG-Periva:	NAIP
MAP1LC3B	1.47E-08	0.274243	0.589	0.423	0.000414	MG-Periva:	MAP1LC3B
REST1	1.64E-08	0.298891	0.272	0.153	0.000461	MG-Periva:	REST
LIPA3	1.70E-08	0.255516	0.498	0.334	0.000478	MG-Periva:	LIPA
PGM21	1.85E-08	0.253512	0.287	0.166	0.00052	MG-Periva:	PGM2
ZFYVE161	1.99E-08	0.326126	0.294	0.172	0.00056	MG-Periva:	ZFYVE16
KDM2B1	2.04E-08	0.346261	0.302	0.18	0.000574	MG-Periva:	KDM2B
OAT1	2.07E-08	0.288799	0.313	0.185	0.000583	MG-Periva:	OAT
SRSF61	2.18E-08	0.255558	0.309	0.184	0.000613	MG-Periva:	SRSF6
TMOD11	2.25E-08	0.273092	0.291	0.164	0.000634	MG-Periva:	TMOD1
SUB13	2.28E-08	0.267827	0.608	0.45	0.000641	MG-Periva:	SUB1
Mar-72	2.42E-08	0.253712	0.415	0.271	0.000682	MG-Periva:	7-Mar
ORMDL11	2.53E-08	0.308604	0.483	0.334	0.000712	MG-Periva:	ORMDL1
RBMS11	2.85E-08	0.254355	0.389	0.251	0.000803	MG-Periva:	RBMS1
SDCBP2	4.13E-08	0.38421	0.819	0.641	0.001162	MG-Periva:	SDCBP
PIEZO11	4.44E-08	0.297003	0.321	0.195	0.001251	MG-Periva:	PIEZO1
HIVEP11	7.74E-08	0.258727	0.264	0.15	0.002179	MG-Periva:	HIVEP1
P3H2	8.99E-08	0.332633	0.268	0.153	0.00253	MG-Periva:	P3H2
MCUB1	9.67E-08	0.337709	0.287	0.171	0.002722	MG-Periva:	MCUB
TUBA1C2	1.22E-07	0.307415	0.438	0.299	0.00343	MG-Periva:	TUBA1C
HERC4	1.22E-07	0.286953	0.291	0.172	0.003434	MG-Periva:	HERC4
GNB23	1.52E-07	0.262376	0.608	0.459	0.00428	MG-Periva:	GNB2
TXN4	1.98E-07	0.578493	0.294	0.186	0.005567	MG-Periva:	TXN
CCNK	1.98E-07	0.296797	0.302	0.185	0.00557	MG-Periva:	CCNK
RGL21	2.40E-07	0.275128	0.264	0.156	0.00676	MG-Periva:	RGL2
WDFY32	2.42E-07	0.255338	0.37	0.237	0.006806	MG-Periva:	WDFY3
PLA2G4A2	2.47E-07	0.306662	0.521	0.376	0.006943	MG-Periva:	PLA2G4A
ACER3	2.81E-07	0.271313	0.37	0.238	0.00791	MG-Periva:	ACER3
MSR14	4.01E-07	0.338237	0.728	0.579	0.011285	MG-Periva:	MSR1
PPP4R2	4.15E-07	0.256181	0.34	0.218	0.011678	MG-Periva:	PPP4R2
IFNGR13	4.37E-07	0.268591	0.834	0.697	0.012306	MG-Periva:	IFNGR1
JARID25	4.51E-07	0.277547	0.404	0.275	0.01268	MG-Periva:	JARID2
MBD41	5.29E-07	0.279832	0.351	0.226	0.014893	MG-Periva:	MBD4
ELMSAN1	5.91E-07	0.353339	0.283	0.175	0.01662	MG-Periva:	ELMSAN1
SLC25A371	8.23E-07	0.304883	0.604	0.469	0.023162	MG-Periva:	SLC25A37
SURF4	2.24E-06	0.271496	0.306	0.2	0.062951	MG-Periva:	SURF4
FLOT12	2.75E-06	0.284657	0.551	0.42	0.077263	MG-Periva:	FLOT1
RGS24	6.39E-06	0.3649	0.698	0.564	0.179879	MG-Periva:	RGS2
AC091271.	6.84E-06	0.252533	0.268	0.167	0.192529	MG-Periva:	AC091271.1
CHSY3	7.89E-06	0.281655	0.253	0.158	0.221882	MG-Periva:	CHSY3
AKIRIN23	1.02E-05	0.255598	0.415	0.305	0.285714	MG-Periva:	AKIRIN2
AL392172.:	1.50E-05	0.259583	0.306	0.204	0.422725	MG-Periva:	AL392172.1
SSH1	4.77E-05	0.256837	0.336	0.243	1	MG-Periva:	SSH1
HAMP2	6.38E-05	1.338593	0.449	0.365	1	MG-Periva:	HAMP
LY96	0.000477	0.310247	0.381	0.294	1	MG-Periva:	LY96

Supplementary Table 5: DEGs for Figure 2E

	p_val	avg_log2FC	pct.1	pct.2	p_val_adj	cluster	gene
CD8A	0	1.770838	0.85	0.261	0	CD8.Tearly	CD8A
CD8B	0	1.578215	0.705	0.186	0	CD8.Tearly	CD8B
GZMK	0	1.520261	0.822	0.355	0	CD8.Tearly	GZMK
CCL5	0	1.38054	0.99	0.659	0	CD8.Tearly	CCL5
GZMH	0	1.220283	0.656	0.251	0	CD8.Tearly	GZMH
GZMA	0	1.026231	0.844	0.479	0	CD8.Tearly	GZMA
DUSP4	0	0.980893	0.453	0.187	0	CD8.Tearly	DUSP4
CCL4	0	0.920698	0.917	0.652	0	CD8.Tearly	CCL4
CRTAM	0	0.902432	0.271	0.094	0	CD8.Tearly	CRTAM
CST7	0	0.859239	0.86	0.518	0	CD8.Tearly	CST7
TUBA4A	0	0.817548	0.682	0.469	0	CD8.Tearly	TUBA4A
CXCR4	0	0.723926	0.94	0.763	0	CD8.Tearly	CXCR4
RGS1	0	0.717126	0.794	0.542	0	CD8.Tearly	RGS1
DUSP2	0	0.711047	0.921	0.686	0	CD8.Tearly	DUSP2
CD3D	0	0.663925	0.81	0.551	0	CD8.Tearly	CD3D
ITM2C	0	0.649604	0.437	0.222	0	CD8.Tearly	ITM2C
IL32	0	0.619536	0.923	0.72	0	CD8.Tearly	IL32
CYTOR	0	0.610671	0.474	0.252	0	CD8.Tearly	CYTOR
KLRK1	0	0.599375	0.563	0.304	0	CD8.Tearly	KLRK1
CD3E	0	0.564815	0.912	0.698	0	CD8.Tearly	CD3E
RPS27	0	0.540247	0.995	0.922	0	CD8.Tearly	RPS27
ZFP36L2	0	0.531647	0.983	0.909	0	CD8.Tearly	ZFP36L2
BTG1	0	0.523946	0.991	0.918	0	CD8.Tearly	BTG1
RPS29	0	0.509131	0.915	0.776	0	CD8.Tearly	RPS29
HLA-A	0	0.471565	0.982	0.914	0	CD8.Tearly	HLA-A
TMSB4X	0	0.449655	0.995	0.93	0	CD8.Tearly	TMSB4X
HLA-B	0	0.434327	0.995	0.947	0	CD8.Tearly	HLA-B
RPS15A	0	0.389832	0.995	0.907	0	CD8.Tearly	RPS15A
NKG7	0	0.332155	0.839	0.445	0	CD8.Tearly	NKG7
B2M	0	0.311483	1	0.964	0	CD8.Tearly	B2M
RPS3	1.44E-298	0.362351	0.994	0.898	4.05E-294	CD8.Tearly	RPS3
PPDPF	8.64E-296	0.510066	0.858	0.69	2.43E-291	CD8.Tearly	PPDPF
RPL23A	1.05E-291	0.403729	0.956	0.836	2.95E-287	CD8.Tearly	RPL23A
TERF2IP	1.47E-278	0.639665	0.599	0.408	4.15E-274	CD8.Tearly	TERF2IP
PTPRCAP	6.82E-272	0.713601	0.599	0.397	1.92E-267	CD8.Tearly	PTPRCAP
CTSW	1.56E-270	0.33363	0.712	0.447	4.38E-266	CD8.Tearly	CTSW
H3F3B	7.11E-267	0.490558	0.974	0.879	2.00E-262	CD8.Tearly	H3F3B
IFNG	8.66E-266	0.817423	0.281	0.12	2.44E-261	CD8.Tearly	IFNG
SH3BGRL3	1.94E-264	0.448648	0.919	0.78	5.46E-260	CD8.Tearly	SH3BGRL3
RPL28	6.47E-260	0.316511	0.995	0.926	1.82E-255	CD8.Tearly	RPL28
ATP5F1E	2.41E-259	0.430611	0.897	0.763	6.79E-255	CD8.Tearly	ATP5F1E
ITM2A	1.42E-258	0.666969	0.541	0.355	4.00E-254	CD8.Tearly	ITM2A
UBC	5.49E-251	0.363086	0.967	0.867	1.55E-246	CD8.Tearly	UBC
TNFSF9	1.43E-246	0.767676	0.288	0.134	4.02E-242	CD8.Tearly	TNFSF9
GAPDH	3.71E-245	0.454208	0.924	0.794	1.04E-240	CD8.Tearly	GAPDH

SRSF2	1.08E-243	0.58281	0.69	0.535	3.05E-239	CD8.Tearly	SRSF2
LAG3	8.14E-242	0.503623	0.292	0.132	2.29E-237	CD8.Tearly	LAG3
EIF1	1.13E-239	0.376243	0.98	0.896	3.18E-235	CD8.Tearly	EIF1
SRSF7	2.14E-238	0.503918	0.825	0.687	6.03E-234	CD8.Tearly	SRSF7
CD3G	1.88E-235	0.512114	0.591	0.399	5.30E-231	CD8.Tearly	CD3G
COTL1	2.22E-234	0.56955	0.645	0.459	6.24E-230	CD8.Tearly	COTL1
ZNF683	6.52E-233	0.550335	0.276	0.121	1.83E-228	CD8.Tearly	ZNF683
RPL7A	8.69E-230	0.321005	0.987	0.883	2.44E-225	CD8.Tearly	RPL7A
GZMM	3.21E-228	0.517328	0.546	0.355	9.02E-224	CD8.Tearly	GZMM
PLAAT4	5.15E-227	0.47618	0.718	0.554	1.45E-222	CD8.Tearly	PLAAT4
HCST	2.23E-225	0.415632	0.836	0.673	6.27E-221	CD8.Tearly	HCST
ARHGDIB	1.05E-221	0.371019	0.914	0.814	2.95E-217	CD8.Tearly	ARHGDIB
CLEC2B	1.05E-221	0.56889	0.552	0.377	2.95E-217	CD8.Tearly	CLEC2B
CD2	1.41E-221	0.442318	0.725	0.538	3.98E-217	CD8.Tearly	CD2
CITED2	3.34E-221	0.652108	0.633	0.455	9.39E-217	CD8.Tearly	CITED2
JMJD6	1.10E-218	0.57406	0.401	0.235	3.10E-214	CD8.Tearly	JMJD6
HERPUD1	1.36E-215	0.551896	0.652	0.476	3.82E-211	CD8.Tearly	HERPUD1
RUNX3	5.76E-211	0.41826	0.714	0.535	1.62E-206	CD8.Tearly	RUNX3
HLA-DPB1	8.13E-210	0.298969	0.552	0.335	2.29E-205	CD8.Tearly	HLA-DPB1
JUNB	9.92E-209	0.430857	0.956	0.854	2.79E-204	CD8.Tearly	JUNB
LSP1	1.69E-208	0.446228	0.736	0.585	4.75E-204	CD8.Tearly	LSP1
SUB1	2.56E-193	0.471928	0.683	0.541	7.19E-189	CD8.Tearly	SUB1
CD27	1.34E-190	0.536443	0.447	0.285	3.77E-186	CD8.Tearly	CD27
PNRC1	1.19E-189	0.401395	0.903	0.77	3.35E-185	CD8.Tearly	PNRC1
APOBEC3G	1.74E-189	0.52072	0.422	0.261	4.90E-185	CD8.Tearly	APOBEC3G
RPS21	2.24E-187	0.299694	0.972	0.859	6.31E-183	CD8.Tearly	RPS21
FABP5	1.59E-186	0.570747	0.279	0.143	4.48E-182	CD8.Tearly	FABP5
RPS28	2.24E-182	0.277299	0.99	0.897	6.30E-178	CD8.Tearly	RPS28
CD99	1.75E-179	0.368985	0.809	0.667	4.92E-175	CD8.Tearly	CD99
UBE2S	1.93E-179	0.540034	0.589	0.434	5.44E-175	CD8.Tearly	UBE2S
CXCR6	2.10E-177	0.606482	0.313	0.171	5.90E-173	CD8.Tearly	CXCR6
RPS26	8.87E-174	0.383463	0.965	0.877	2.50E-169	CD8.Tearly	RPS26
RPS7	1.43E-170	0.270393	0.985	0.881	4.03E-166	CD8.Tearly	RPS7
TENT5C	3.42E-169	0.408404	0.6	0.432	9.62E-165	CD8.Tearly	TENT5C
TMA7	2.11E-161	0.368787	0.802	0.667	5.94E-157	CD8.Tearly	TMA7
SH2D1A	7.63E-161	0.493264	0.32	0.184	2.15E-156	CD8.Tearly	SH2D1A
GUK1	8.34E-160	0.417566	0.646	0.507	2.35E-155	CD8.Tearly	GUK1
CFL1	3.28E-159	0.353002	0.906	0.788	9.22E-155	CD8.Tearly	CFL1
RPS25	1.13E-154	0.26437	0.976	0.869	3.17E-150	CD8.Tearly	RPS25
LIME1	1.72E-153	0.448981	0.508	0.361	4.84E-149	CD8.Tearly	LIME1
CALM1	5.27E-152	0.347147	0.898	0.797	1.48E-147	CD8.Tearly	CALM1
LEPROTL1	3.24E-151	0.363708	0.738	0.593	9.13E-147	CD8.Tearly	LEPROTL1
RBM38	1.13E-148	0.454816	0.51	0.363	3.19E-144	CD8.Tearly	RBM38
GABARAPL	2.00E-148	0.451188	0.352	0.216	5.63E-144	CD8.Tearly	GABARAPL1
IDH2	8.25E-148	0.430084	0.372	0.237	2.32E-143	CD8.Tearly	IDH2
ZFP36	1.49E-144	0.280951	0.962	0.853	4.18E-140	CD8.Tearly	ZFP36
ACTG1	3.98E-144	0.377979	0.909	0.795	1.12E-139	CD8.Tearly	ACTG1

LYST	5.34E-143	0.333242	0.414	0.266	1.50E-138	CD8.Tearly	LYST
FAU	3.43E-142	0.252747	0.985	0.881	9.64E-138	CD8.Tearly	FAU
MYL6	3.82E-138	0.307321	0.871	0.746	1.07E-133	CD8.Tearly	MYL6
NR4A2	1.16E-133	0.313519	0.786	0.626	3.25E-129	CD8.Tearly	NR4A2
CD69	3.92E-133	0.330141	0.866	0.719	1.10E-128	CD8.Tearly	CD69
TUBB4B	8.80E-132	0.531658	0.568	0.449	2.48E-127	CD8.Tearly	TUBB4B
TOMM7	5.56E-131	0.32578	0.784	0.65	1.57E-126	CD8.Tearly	TOMM7
CYTIP	1.03E-128	0.345216	0.737	0.616	2.91E-124	CD8.Tearly	CYTIP
TRBC2	1.57E-128	0.424208	0.567	0.433	4.43E-124	CD8.Tearly	TRBC2
CHCHD2	6.05E-127	0.340642	0.713	0.588	1.70E-122	CD8.Tearly	CHCHD2
RGCC	1.14E-125	0.486433	0.612	0.468	3.22E-121	CD8.Tearly	RGCC
RPL36AL	4.38E-125	0.281921	0.869	0.734	1.23E-120	CD8.Tearly	RPL36AL
ANXA1	1.56E-123	0.349178	0.721	0.582	4.40E-119	CD8.Tearly	ANXA1
BUB3	3.41E-123	0.413318	0.468	0.338	9.60E-119	CD8.Tearly	BUB3
UQCRB	1.00E-122	0.340588	0.705	0.579	2.82E-118	CD8.Tearly	UQCRB
ZYX	3.98E-122	0.418408	0.469	0.343	1.12E-117	CD8.Tearly	ZYX
STK17A	6.83E-121	0.299291	0.72	0.594	1.92E-116	CD8.Tearly	STK17A
PFN1	1.21E-120	0.294998	0.944	0.832	3.41E-116	CD8.Tearly	PFN1
LINC01871	2.05E-120	0.460789	0.291	0.175	5.76E-116	CD8.Tearly	LINC01871
OST4	1.36E-119	0.316221	0.688	0.558	3.82E-115	CD8.Tearly	OST4
RBFOX2	5.07E-118	0.605826	0.259	0.15	1.43E-113	CD8.Tearly	RBFOX2
CALM2	1.41E-116	0.333289	0.716	0.597	3.96E-112	CD8.Tearly	CALM2
TSC22D3	5.02E-116	0.312845	0.933	0.832	1.41E-111	CD8.Tearly	TSC22D3
PTP4A2	5.64E-116	0.382924	0.534	0.413	1.59E-111	CD8.Tearly	PTP4A2
PIK3R1	7.08E-116	0.37249	0.603	0.478	1.99E-111	CD8.Tearly	PIK3R1
SNRPD2	2.45E-113	0.31887	0.691	0.576	6.89E-109	CD8.Tearly	SNRPD2
ID2	2.45E-113	0.398977	0.652	0.527	6.91E-109	CD8.Tearly	ID2
RAC2	5.01E-112	0.298895	0.761	0.633	1.41E-107	CD8.Tearly	RAC2
OAZ1	2.43E-111	0.299815	0.789	0.662	6.84E-107	CD8.Tearly	OAZ1
SRP14	4.43E-110	0.300609	0.722	0.608	1.25E-105	CD8.Tearly	SRP14
HMGB2	7.36E-109	0.420037	0.41	0.29	2.07E-104	CD8.Tearly	HMGB2
CNN2	1.30E-108	0.349895	0.533	0.408	3.65E-104	CD8.Tearly	CNN2
YPEL5	7.37E-106	0.333979	0.617	0.495	2.07E-101	CD8.Tearly	YPEL5
MYL12B	1.17E-105	0.299116	0.725	0.612	3.29E-101	CD8.Tearly	MYL12B
S100A6	1.38E-102	0.264929	0.724	0.59	3.90E-98	CD8.Tearly	S100A6
CXCR3	1.45E-102	0.358192	0.285	0.176	4.08E-98	CD8.Tearly	CXCR3
SIT1	2.28E-101	0.353736	0.307	0.196	6.42E-97	CD8.Tearly	SIT1
SRRT	1.33E-100	0.436037	0.313	0.206	3.73E-96	CD8.Tearly	SRRT
LAT	7.73E-98	0.343046	0.447	0.333	2.17E-93	CD8.Tearly	LAT
LCP1	2.19E-96	0.25355	0.731	0.61	6.17E-92	CD8.Tearly	LCP1
CORO1A	1.70E-95	0.302887	0.789	0.689	4.77E-91	CD8.Tearly	CORO1A
AKIRIN2	1.99E-95	0.368307	0.335	0.229	5.61E-91	CD8.Tearly	AKIRIN2
XCL1	2.79E-94	0.560011	0.279	0.173	7.85E-90	CD8.Tearly	XCL1
IER5L	7.21E-94	0.477224	0.273	0.175	2.03E-89	CD8.Tearly	IER5L
BTG3	4.56E-93	0.368667	0.305	0.202	1.28E-88	CD8.Tearly	BTG3
CRIP1	6.82E-92	0.314136	0.682	0.56	1.92E-87	CD8.Tearly	CRIP1
TRIR	1.29E-91	0.308246	0.616	0.51	3.62E-87	CD8.Tearly	TRIR

UBL5	6.14E-91	0.309141	0.585	0.477	1.73E-86	CD8.Tearly UBL5
ATP5MG	3.81E-90	0.266986	0.748	0.641	1.07E-85	CD8.Tearly ATP5MG
CD52	8.33E-90	0.278297	0.767	0.643	2.34E-85	CD8.Tearly CD52
LCK	7.14E-89	0.275543	0.616	0.506	2.01E-84	CD8.Tearly LCK
MYADM	1.11E-88	0.46177	0.426	0.319	3.12E-84	CD8.Tearly MYADM
RESF1	3.10E-87	0.279412	0.604	0.488	8.73E-83	CD8.Tearly RESF1
ARF6	2.62E-86	0.30835	0.544	0.433	7.37E-82	CD8.Tearly ARF6
HNRNPLL	2.80E-86	0.307505	0.307	0.205	7.89E-82	CD8.Tearly HNRNPLL
DUSP5	3.19E-86	0.354591	0.385	0.273	8.99E-82	CD8.Tearly DUSP5
CLIC1	2.80E-85	0.264718	0.727	0.611	7.89E-81	CD8.Tearly CLIC1
RPL27A	5.71E-85	0.274797	0.723	0.61	1.61E-80	CD8.Tearly RPL27A
GABARAPL	1.21E-84	0.344264	0.428	0.328	3.42E-80	CD8.Tearly GABARAPL2
VAMP2	2.23E-84	0.291288	0.586	0.476	6.27E-80	CD8.Tearly VAMP2
LGALS1	3.37E-84	0.453305	0.281	0.185	9.49E-80	CD8.Tearly LGALS1
CCL4L2	4.00E-84	0.313207	0.52	0.388	1.13E-79	CD8.Tearly CCL4L2
ALDOA	3.40E-83	0.350199	0.486	0.383	9.55E-79	CD8.Tearly ALDOA
SAP18	3.70E-83	0.302965	0.575	0.474	1.04E-78	CD8.Tearly SAP18
HINT1	2.04E-81	0.273443	0.692	0.588	5.73E-77	CD8.Tearly HINT1
PTGER4	7.15E-81	0.283611	0.551	0.437	2.01E-76	CD8.Tearly PTGER4
ODC1	2.38E-79	0.32394	0.374	0.274	6.70E-75	CD8.Tearly ODC1
NSMCE3	4.35E-79	0.355524	0.364	0.266	1.22E-74	CD8.Tearly NSMCE3
RAB5IF	8.15E-78	0.347227	0.348	0.252	2.29E-73	CD8.Tearly RAB5IF
H2AFX	2.77E-77	0.365961	0.379	0.281	7.81E-73	CD8.Tearly H2AFX
GMFG	3.40E-77	0.270637	0.645	0.549	9.56E-73	CD8.Tearly GMFG
RPL38	6.56E-77	0.250368	0.793	0.69	1.85E-72	CD8.Tearly RPL38
COX7A2	1.69E-74	0.282911	0.533	0.438	4.75E-70	CD8.Tearly COX7A2
MZT2A	2.30E-74	0.298582	0.393	0.294	6.47E-70	CD8.Tearly MZT2A
DDX24	9.38E-74	0.250571	0.697	0.597	2.64E-69	CD8.Tearly DDX24
TBCC	4.67E-73	0.31342	0.257	0.171	1.31E-68	CD8.Tearly TBCC
LYAR	8.56E-73	0.271685	0.409	0.304	2.41E-68	CD8.Tearly LYAR
GYPC	1.57E-72	0.253145	0.601	0.5	4.42E-68	CD8.Tearly GYPC
ZBTB38	1.65E-72	0.292513	0.326	0.231	4.64E-68	CD8.Tearly ZBTB38
APOO	4.00E-72	0.401997	0.299	0.205	1.13E-67	CD8.Tearly APOO
PSMB9	3.19E-71	0.282606	0.567	0.473	8.96E-67	CD8.Tearly PSMB9
ATP5IF1	1.71E-70	0.299223	0.493	0.402	4.81E-66	CD8.Tearly ATP5IF1
YWHAB	7.60E-70	0.258227	0.671	0.584	2.14E-65	CD8.Tearly YWHAB
CYCS	2.90E-69	0.33256	0.475	0.381	8.17E-65	CD8.Tearly CYCS
C12orf75	2.37E-68	0.299102	0.299	0.211	6.65E-64	CD8.Tearly C12orf75
BRD2	1.45E-67	0.291782	0.608	0.52	4.07E-63	CD8.Tearly BRD2
HERPUD2	5.92E-67	0.266789	0.409	0.314	1.66E-62	CD8.Tearly HERPUD2
NDUFA1	6.01E-67	0.2759	0.485	0.395	1.69E-62	CD8.Tearly NDUFA1
GPI	1.09E-66	0.270663	0.388	0.297	3.06E-62	CD8.Tearly GPI
NDUFS5	5.96E-66	0.262092	0.567	0.474	1.68E-61	CD8.Tearly NDUFS5
TMX4	3.50E-65	0.274223	0.331	0.239	9.86E-61	CD8.Tearly TMX4
FAM102A	7.92E-65	0.259046	0.295	0.207	2.23E-60	CD8.Tearly FAM102A
PARK7	2.47E-63	0.28313	0.483	0.397	6.95E-59	CD8.Tearly PARK7
TSPYL2	8.48E-63	0.288957	0.469	0.378	2.39E-58	CD8.Tearly TSPYL2

JPT1	5.80E-62	0.30864	0.317	0.234	1.63E-57	CD8.Tearly	JPT1
GBP5	5.99E-62	0.295571	0.343	0.252	1.69E-57	CD8.Tearly	GBP5
CDKN1B	1.16E-61	0.273602	0.426	0.333	3.27E-57	CD8.Tearly	CDKN1B
IFI27L2	1.51E-61	0.277343	0.258	0.178	4.24E-57	CD8.Tearly	IFI27L2
CAP1	1.52E-58	0.261304	0.522	0.439	4.28E-54	CD8.Tearly	CAP1
FKBP8	2.97E-57	0.255924	0.456	0.372	8.37E-53	CD8.Tearly	FKBP8
RAB1B	8.58E-57	0.257658	0.307	0.226	2.42E-52	CD8.Tearly	RAB1B
H2AFZ	2.46E-56	0.328819	0.53	0.453	6.93E-52	CD8.Tearly	H2AFZ
SELENOK	3.14E-56	0.275278	0.496	0.41	8.84E-52	CD8.Tearly	SELENOK
PIM3	1.70E-55	0.295058	0.251	0.176	4.80E-51	CD8.Tearly	PIM3
CALM3	1.75E-55	0.273297	0.394	0.312	4.93E-51	CD8.Tearly	CALM3
SLC2A4RG	1.38E-54	0.278635	0.275	0.198	3.89E-50	CD8.Tearly	SLC2A4RG
MARCKSL1	1.96E-54	0.295337	0.282	0.205	5.52E-50	CD8.Tearly	MARCKSL1
MRPL10	3.61E-54	0.255298	0.251	0.176	1.02E-49	CD8.Tearly	MRPL10
TUBA1A	3.28E-52	0.309688	0.428	0.347	9.22E-48	CD8.Tearly	TUBA1A
HIST1H4C	5.19E-52	0.299356	0.43	0.349	1.46E-47	CD8.Tearly	HIST1H4C
METRNL	8.55E-52	0.305625	0.283	0.205	2.40E-47	CD8.Tearly	METRNL
EVI2B	8.23E-50	0.254773	0.375	0.296	2.32E-45	CD8.Tearly	EVI2B
PRDX6	1.22E-47	0.260815	0.37	0.297	3.43E-43	CD8.Tearly	PRDX6
SLC25A5	2.99E-47	0.26089	0.519	0.447	8.42E-43	CD8.Tearly	SLC25A5
ARPC5L	1.45E-44	0.251358	0.348	0.275	4.09E-40	CD8.Tearly	ARPC5L
BAX	2.25E-42	0.324938	0.413	0.344	6.34E-38	CD8.Tearly	BAX
IL7R	0	1.594624	0.848	0.41	0	CD4.Tcm	IL7R
KLRB1	0	1.235314	0.614	0.298	0	CD4.Tcm	KLRB1
AQP3	0	1.036942	0.408	0.12	0	CD4.Tcm	AQP3
LTB	0	0.998978	0.589	0.298	0	CD4.Tcm	LTB
TOB1	0	0.981091	0.515	0.242	0	CD4.Tcm	TOB1
CD40LG	0	0.971931	0.333	0.065	0	CD4.Tcm	CD40LG
GPR183	0	0.912832	0.505	0.207	0	CD4.Tcm	GPR183
TNFAIP3	0	0.867217	0.938	0.742	0	CD4.Tcm	TNFAIP3
S100A4	0	0.861442	0.87	0.614	0	CD4.Tcm	S100A4
NFKBIA	0	0.793368	0.905	0.706	0	CD4.Tcm	NFKBIA
CDKN1A	0	0.780925	0.426	0.209	0	CD4.Tcm	CDKN1A
ANXA11	0	0.779634	0.798	0.568	0	CD4.Tcm	ANXA1
VIM	0	0.762504	0.871	0.673	0	CD4.Tcm	VIM
ZFP36L21	0	0.739315	0.986	0.91	0	CD4.Tcm	ZFP36L2
FTH1	0	0.710874	0.99	0.907	0	CD4.Tcm	FTH1
FXYD5	0	0.694406	0.843	0.644	0	CD4.Tcm	FXYD5
PDE4D	0	0.680107	0.552	0.298	0	CD4.Tcm	PDE4D
RPLP1	0	0.675197	0.997	0.937	0	CD4.Tcm	RPLP1
JUNB1	0	0.669554	0.976	0.853	0	CD4.Tcm	JUNB
SPOCK2	0	0.668093	0.684	0.408	0	CD4.Tcm	SPOCK2
TPT1	0	0.637015	0.987	0.907	0	CD4.Tcm	TPT1
TSC22D31	0	0.636255	0.958	0.829	0	CD4.Tcm	TSC22D3
RPLP0	0	0.631745	0.967	0.85	0	CD4.Tcm	RPLP0
CD4	0	0.609436	0.289	0.102	0	CD4.Tcm	CD4
ZFP361	0	0.59862	0.969	0.855	0	CD4.Tcm	ZFP36

CXCR41	0	0.574778	0.939	0.769	0	CD4.Tcm	CXCR4
RPL32	0	0.553855	0.991	0.91	0	CD4.Tcm	RPL32
EEF1A1	0	0.521428	0.993	0.939	0	CD4.Tcm	EEF1A1
RPL10	0	0.491051	0.995	0.933	0	CD4.Tcm	RPL10
RPS4X	0	0.485562	0.978	0.877	0	CD4.Tcm	RPS4X
RPL12	0	0.479623	0.982	0.893	0	CD4.Tcm	RPL12
RPS13	0	0.478809	0.978	0.873	0	CD4.Tcm	RPS13
RPL8	0	0.473314	0.971	0.865	0	CD4.Tcm	RPL8
RPL34	0	0.472958	0.985	0.898	0	CD4.Tcm	RPL34
BTG11	0	0.464595	0.99	0.921	0	CD4.Tcm	BTG1
RPL11	0	0.462545	0.989	0.906	0	CD4.Tcm	RPL11
RPS18	0	0.460161	0.987	0.906	0	CD4.Tcm	RPS18
RPL39	0	0.455041	0.986	0.891	0	CD4.Tcm	RPL39
RPS12	0	0.450851	0.993	0.92	0	CD4.Tcm	RPS12
RPL14	0	0.447434	0.978	0.877	0	CD4.Tcm	RPL14
RPL29	0	0.433735	0.978	0.877	0	CD4.Tcm	RPL29
RPL36	0	0.432602	0.97	0.864	0	CD4.Tcm	RPL36
RPL18	0	0.432307	0.98	0.879	0	CD4.Tcm	RPL18
RPS19	0	0.429341	0.991	0.921	0	CD4.Tcm	RPS19
RPS3A	0	0.425288	0.975	0.886	0	CD4.Tcm	RPS3A
RPL18A	0	0.425165	0.985	0.896	0	CD4.Tcm	RPL18A
RPS8	0	0.41942	0.986	0.902	0	CD4.Tcm	RPS8
RPS251	0	0.418076	0.976	0.872	0	CD4.Tcm	RPS25
RPL37	0	0.417223	0.979	0.879	0	CD4.Tcm	RPL37
RPL30	0	0.398783	0.99	0.913	0	CD4.Tcm	RPL30
RPL13	0	0.381652	0.993	0.929	0	CD4.Tcm	RPL13
RPS23	0	0.381433	0.987	0.899	0	CD4.Tcm	RPS23
RPL19	0	0.375834	0.988	0.904	0	CD4.Tcm	RPL19
RPL281	0	0.361913	0.994	0.928	0	CD4.Tcm	RPL28
RPS14	7.54E-307	0.386177	0.985	0.893	2.12E-302	CD4.Tcm	RPS14
SARAF	1.25E-306	0.468006	0.939	0.838	3.53E-302	CD4.Tcm	SARAF
RPS27A	1.40E-301	0.365797	0.989	0.906	3.95E-297	CD4.Tcm	RPS27A
RPL9	1.77E-293	0.461291	0.924	0.801	4.99E-289	CD4.Tcm	RPL9
PTGER2	3.40E-292	0.682515	0.349	0.156	9.56E-288	CD4.Tcm	PTGER2
PTMA	1.51E-282	0.369195	0.986	0.917	4.26E-278	CD4.Tcm	PTMA
CD521	1.40E-281	0.508387	0.845	0.628	3.93E-277	CD4.Tcm	CD52
S100A10	2.15E-280	0.60322	0.726	0.512	6.04E-276	CD4.Tcm	S100A10
MAF	4.58E-279	0.621659	0.362	0.17	1.29E-274	CD4.Tcm	MAF
JUN	3.17E-276	0.646759	0.913	0.771	8.92E-272	CD4.Tcm	JUN
RPS2	4.39E-276	0.42703	0.954	0.859	1.24E-271	CD4.Tcm	RPS2
RPLP2	5.47E-274	0.365566	0.97	0.873	1.54E-269	CD4.Tcm	RPLP2
DDIT4	8.12E-274	0.626543	0.83	0.651	2.29E-269	CD4.Tcm	DDIT4
RPS16	5.16E-269	0.428392	0.932	0.819	1.45E-264	CD4.Tcm	RPS16
RPS6	2.35E-267	0.395363	0.963	0.869	6.63E-263	CD4.Tcm	RPS6
FOS	8.03E-262	0.579076	0.929	0.796	2.26E-257	CD4.Tcm	FOS
FLT3LG	8.71E-262	0.587356	0.381	0.188	2.45E-257	CD4.Tcm	FLT3LG
DUSP51	4.36E-261	0.666243	0.462	0.258	1.23E-256	CD4.Tcm	DUSP5

PBXIP1	3.69E-258	0.523387	0.462	0.25	1.04E-253	CD4.Tcm	PBXIP1
RPL35A	2.72E-255	0.351482	0.969	0.865	7.66E-251	CD4.Tcm	RPL35A
RPL10A	2.76E-250	0.420367	0.918	0.799	7.77E-246	CD4.Tcm	RPL10A
RPL6	1.32E-245	0.362421	0.965	0.874	3.72E-241	CD4.Tcm	RPL6
RPL5	2.19E-244	0.393615	0.938	0.827	6.15E-240	CD4.Tcm	RPL5
CREM	1.09E-243	0.656782	0.649	0.452	3.07E-239	CD4.Tcm	CREM
RPL22	1.25E-237	0.392914	0.911	0.774	3.51E-233	CD4.Tcm	RPL22
RORA	7.29E-235	0.395412	0.598	0.38	2.05E-230	CD4.Tcm	RORA
RPL3	1.30E-234	0.386088	0.963	0.871	3.66E-230	CD4.Tcm	RPL3
RPSA	4.41E-232	0.416599	0.912	0.801	1.24E-227	CD4.Tcm	RPSA
RPS15	4.61E-227	0.304752	0.985	0.902	1.30E-222	CD4.Tcm	RPS15
RPS15A1	5.30E-227	0.303503	0.989	0.911	1.49E-222	CD4.Tcm	RPS15A
RPL36A	1.72E-226	0.418029	0.898	0.758	4.84E-222	CD4.Tcm	RPL36A
RPL36AL1	6.08E-226	0.413242	0.883	0.736	1.71E-221	CD4.Tcm	RPL36AL
RPL24	1.51E-225	0.343756	0.952	0.84	4.25E-221	CD4.Tcm	RPL24
EEF1B2	9.86E-223	0.418554	0.877	0.747	2.77E-218	CD4.Tcm	EEF1B2
CD28	1.17E-222	0.410925	0.276	0.119	3.29E-218	CD4.Tcm	CD28
RPL35	2.12E-220	0.356803	0.942	0.824	5.97E-216	CD4.Tcm	RPL35
SOCS3	6.82E-220	0.527034	0.296	0.137	1.92E-215	CD4.Tcm	SOCS3
ERN1	9.32E-219	0.461123	0.599	0.393	2.62E-214	CD4.Tcm	ERN1
RPS281	9.62E-217	0.318655	0.986	0.901	2.71E-212	CD4.Tcm	RPS28
EIF11	1.34E-214	0.327214	0.977	0.9	3.77E-210	CD4.Tcm	EIF1
LEPROTL11	4.16E-214	0.462357	0.77	0.59	1.17E-209	CD4.Tcm	LEPROTL1
RPS31	1.76E-213	0.314933	0.986	0.903	4.96E-209	CD4.Tcm	RPS3
RPL17	1.81E-213	0.350823	0.946	0.847	5.09E-209	CD4.Tcm	RPL17
TRADD	5.59E-210	0.513556	0.29	0.136	1.57E-205	CD4.Tcm	TRADD
PABPC1	2.33E-209	0.401533	0.892	0.785	6.56E-205	CD4.Tcm	PABPC1
RPL26	1.10E-207	0.307922	0.98	0.891	3.11E-203	CD4.Tcm	RPL26
EEF1G	1.92E-206	0.445908	0.81	0.665	5.39E-202	CD4.Tcm	EEF1G
RPS5	9.28E-205	0.352258	0.933	0.815	2.61E-200	CD4.Tcm	RPS5
PBX4	8.01E-200	0.334677	0.384	0.203	2.25E-195	CD4.Tcm	PBX4
RACK1	1.26E-199	0.348815	0.938	0.825	3.54E-195	CD4.Tcm	RACK1
S100A61	1.54E-197	0.469075	0.764	0.585	4.33E-193	CD4.Tcm	S100A6
SNX9	4.56E-197	0.402483	0.411	0.229	1.28E-192	CD4.Tcm	SNX9
FAU1	7.21E-192	0.288534	0.981	0.885	2.03E-187	CD4.Tcm	FAU
LDHB	2.04E-185	0.463869	0.662	0.481	5.74E-181	CD4.Tcm	LDHB
RPS211	9.35E-183	0.315277	0.969	0.864	2.63E-178	CD4.Tcm	RPS21
RPL4	4.44E-178	0.407217	0.833	0.69	1.25E-173	CD4.Tcm	RPL4
EEF2	1.05E-177	0.386449	0.853	0.728	2.97E-173	CD4.Tcm	EEF2
TENT5C1	2.83E-176	0.426964	0.623	0.432	7.96E-172	CD4.Tcm	TENT5C
BHLHE40	1.45E-171	0.543367	0.467	0.295	4.07E-167	CD4.Tcm	BHLHE40
RPS71	6.08E-168	0.281652	0.977	0.886	1.71E-163	CD4.Tcm	RPS7
TXNIP	5.36E-167	0.520822	0.833	0.704	1.51E-162	CD4.Tcm	TXNIP
RPL41	4.97E-165	0.378028	0.959	0.878	1.40E-160	CD4.Tcm	RPL41
DUSP1	3.07E-156	0.351118	0.946	0.816	8.64E-152	CD4.Tcm	DUSP1
RPL7	4.52E-154	0.37129	0.767	0.622	1.27E-149	CD4.Tcm	RPL7
RPL15	7.88E-154	0.317771	0.934	0.824	2.22E-149	CD4.Tcm	RPL15

RPL7A1	8.87E-154	0.264985	0.976	0.889	2.49E-149	CD4.Tcm	RPL7A
NPM1	2.61E-153	0.350634	0.837	0.696	7.33E-149	CD4.Tcm	NPM1
CSRNP1	3.33E-150	0.458178	0.559	0.396	9.38E-146	CD4.Tcm	CSRNP1
RPS9	1.76E-147	0.293713	0.938	0.836	4.96E-143	CD4.Tcm	RPS9
NACA	5.10E-147	0.293161	0.943	0.826	1.44E-142	CD4.Tcm	NACA
CD691	7.27E-147	0.411962	0.862	0.725	2.05E-142	CD4.Tcm	CD69
CRIP11	8.21E-147	0.441157	0.719	0.556	2.31E-142	CD4.Tcm	CRIP1
S100A11	3.24E-146	0.397969	0.613	0.427	9.11E-142	CD4.Tcm	S100A11
CD21	7.70E-145	0.3736	0.715	0.546	2.17E-140	CD4.Tcm	CD2
NDUFS51	9.11E-145	0.409458	0.624	0.464	2.56E-140	CD4.Tcm	NDUFS5
FKBP11	2.80E-143	0.429407	0.378	0.228	7.87E-139	CD4.Tcm	FKBP11
UBA52	3.91E-143	0.290118	0.901	0.784	1.10E-138	CD4.Tcm	UBA52
ABRACL	1.06E-140	0.412799	0.507	0.345	2.97E-136	CD4.Tcm	ABRACL
TMEM123	5.80E-136	0.37711	0.394	0.244	1.63E-131	CD4.Tcm	TMEM123
H3F3B1	2.20E-134	0.269027	0.967	0.884	6.19E-130	CD4.Tcm	H3F3B
ICOS	7.10E-134	0.406276	0.387	0.237	2.00E-129	CD4.Tcm	ICOS
RPL13A	4.49E-129	0.293112	0.887	0.772	1.26E-124	CD4.Tcm	RPL13A
TESPA1	4.09E-128	0.317131	0.275	0.149	1.15E-123	CD4.Tcm	TESPA1
ARL4C	5.99E-128	0.355729	0.692	0.529	1.68E-123	CD4.Tcm	ARL4C
SNHG16	2.61E-127	0.414951	0.317	0.188	7.34E-123	CD4.Tcm	SNHG16
PIM2	8.35E-127	0.490696	0.331	0.199	2.35E-122	CD4.Tcm	PIM2
JUND	1.81E-126	0.333732	0.894	0.793	5.09E-122	CD4.Tcm	JUND
TAGLN2	7.25E-126	0.388174	0.625	0.472	2.04E-121	CD4.Tcm	TAGLN2
RPL21	8.83E-126	0.317495	0.892	0.796	2.48E-121	CD4.Tcm	RPL21
SLC2A3	1.26E-124	0.326438	0.699	0.54	3.55E-120	CD4.Tcm	SLC2A3
SERP1	2.14E-124	0.367909	0.659	0.506	6.03E-120	CD4.Tcm	SERP1
DNAJB9	7.66E-124	0.394929	0.311	0.183	2.15E-119	CD4.Tcm	DNAJB9
BTF3	2.80E-120	0.300849	0.847	0.708	7.89E-116	CD4.Tcm	BTF3
MIF	4.29E-120	0.320975	0.768	0.62	1.21E-115	CD4.Tcm	MIF
RCAN3	1.03E-119	0.310012	0.324	0.188	2.89E-115	CD4.Tcm	RCAN3
MGAT4A	1.87E-119	0.373055	0.461	0.315	5.27E-115	CD4.Tcm	MGAT4A
LDHA	1.01E-118	0.372269	0.66	0.515	2.85E-114	CD4.Tcm	LDHA
SNHG29	2.12E-118	0.348647	0.684	0.536	5.96E-114	CD4.Tcm	SNHG29
RPL31	3.41E-118	0.296194	0.836	0.705	9.59E-114	CD4.Tcm	RPL31
CERK	5.31E-118	0.285731	0.255	0.138	1.49E-113	CD4.Tcm	CERK
RPL23A1	7.98E-118	0.251791	0.946	0.842	2.24E-113	CD4.Tcm	RPL23A
PLP2	2.77E-116	0.359947	0.412	0.268	7.79E-112	CD4.Tcm	PLP2
CALM11	3.86E-115	0.256707	0.909	0.797	1.09E-110	CD4.Tcm	CALM1
CDKN1B1	4.92E-114	0.368645	0.471	0.325	1.39E-109	CD4.Tcm	CDKN1B
RPL381	8.65E-113	0.30443	0.818	0.688	2.43E-108	CD4.Tcm	RPL38
PPP1R15A	1.24E-111	0.360495	0.703	0.553	3.48E-107	CD4.Tcm	PPP1R15A
RGCC1	7.60E-111	0.32555	0.627	0.469	2.14E-106	CD4.Tcm	RGCC
NFKBIZ	1.12E-109	0.387966	0.515	0.367	3.14E-105	CD4.Tcm	NFKBIZ
HNRNPA1	1.17E-108	0.288886	0.831	0.706	3.30E-104	CD4.Tcm	HNRNPA1
YWHAQ	1.61E-107	0.333532	0.503	0.36	4.54E-103	CD4.Tcm	YWHAQ
TCF7	1.99E-106	0.259438	0.31	0.182	5.59E-102	CD4.Tcm	TCF7
G3BP2	2.76E-106	0.364043	0.459	0.322	7.77E-102	CD4.Tcm	G3BP2

UBALD2	3.85E-105	0.356967	0.539	0.4	1.08E-100	CD4.Tcm	UBALD2
APRT	3.96E-105	0.334846	0.592	0.453	1.12E-100	CD4.Tcm	APRT
CD5	5.86E-104	0.317366	0.371	0.24	1.65E-99	CD4.Tcm	CD5
PFDN5	7.38E-103	0.252775	0.869	0.749	2.08E-98	CD4.Tcm	PFDN5
PER1	1.53E-102	0.291865	0.478	0.335	4.30E-98	CD4.Tcm	PER1
COX7C	4.72E-102	0.277659	0.778	0.639	1.33E-97	CD4.Tcm	COX7C
HINT11	8.35E-101	0.296159	0.721	0.585	2.35E-96	CD4.Tcm	HINT1
SEC61B	1.74E-100	0.334699	0.566	0.423	4.90E-96	CD4.Tcm	SEC61B
TOMM71	2.27E-99	0.267985	0.784	0.655	6.39E-95	CD4.Tcm	TOMM7
HSPA5	2.98E-99	0.293966	0.698	0.551	8.38E-95	CD4.Tcm	HSPA5
RPL27	1.77E-98	0.264695	0.85	0.733	4.99E-94	CD4.Tcm	RPL27
PTGER41	4.06E-97	0.321611	0.573	0.435	1.14E-92	CD4.Tcm	PTGER4
CIB1	4.37E-96	0.33034	0.551	0.414	1.23E-91	CD4.Tcm	CIB1
ARL4A	3.54E-95	0.378139	0.254	0.15	9.95E-91	CD4.Tcm	ARL4A
EIF3E	2.68E-93	0.325304	0.534	0.404	7.53E-89	CD4.Tcm	EIF3E
PRDM1	4.80E-90	0.305679	0.463	0.326	1.35E-85	CD4.Tcm	PRDM1
ANP32B	2.30E-89	0.316274	0.506	0.374	6.47E-85	CD4.Tcm	ANP32B
SEC61G	1.15E-87	0.332306	0.464	0.338	3.25E-83	CD4.Tcm	SEC61G
KLF2	1.25E-87	0.438377	0.564	0.448	3.51E-83	CD4.Tcm	KLF2
S1PR4	2.81E-87	0.320749	0.273	0.167	7.90E-83	CD4.Tcm	S1PR4
NOP53	6.40E-86	0.280427	0.645	0.521	1.80E-81	CD4.Tcm	NOP53
PPIB	1.10E-83	0.26031	0.736	0.602	3.09E-79	CD4.Tcm	PPIB
PRKCQ-AS1	2.36E-83	0.252956	0.257	0.155	6.65E-79	CD4.Tcm	PRKCQ-AS1
CITED21	9.63E-80	0.323368	0.602	0.468	2.71E-75	CD4.Tcm	CITED2
RAN	8.43E-79	0.281372	0.571	0.442	2.37E-74	CD4.Tcm	RAN
SNHG6	5.11E-78	0.304103	0.578	0.454	1.44E-73	CD4.Tcm	SNHG6
TOMM5	8.52E-78	0.32002	0.366	0.255	2.40E-73	CD4.Tcm	TOMM5
GAS5	2.87E-77	0.290619	0.566	0.443	8.08E-73	CD4.Tcm	GAS5
XBP1	1.73E-76	0.290411	0.461	0.339	4.86E-72	CD4.Tcm	XBP1
ATP6V1G1	8.69E-76	0.274078	0.566	0.442	2.45E-71	CD4.Tcm	ATP6V1G1
OCIAD2	9.80E-76	0.272793	0.387	0.271	2.76E-71	CD4.Tcm	OCIAD2
PEBP1	1.41E-75	0.270685	0.452	0.33	3.96E-71	CD4.Tcm	PEBP1
CD48	4.93E-75	0.273968	0.555	0.428	1.39E-70	CD4.Tcm	CD48
TXN	2.00E-74	0.303827	0.4	0.286	5.62E-70	CD4.Tcm	TXN
SSR2	5.35E-74	0.258109	0.644	0.519	1.50E-69	CD4.Tcm	SSR2
H1FX	1.71E-73	0.403438	0.597	0.474	4.80E-69	CD4.Tcm	H1FX
CCDC107	2.39E-73	0.278479	0.369	0.26	6.72E-69	CD4.Tcm	CCDC107
HNRNPUL1	2.83E-73	0.253836	0.508	0.389	7.97E-69	CD4.Tcm	HNRNPUL1
HNRNPA0	4.32E-73	0.28	0.571	0.447	1.22E-68	CD4.Tcm	HNRNPA0
PNP	7.56E-73	0.274856	0.28	0.182	2.13E-68	CD4.Tcm	PNP
SMDT1	1.74E-71	0.294138	0.467	0.351	4.90E-67	CD4.Tcm	SMDT1
TMEM173	1.03E-70	0.289025	0.289	0.192	2.89E-66	CD4.Tcm	TMEM173
PIM1	2.51E-70	0.333864	0.311	0.213	7.06E-66	CD4.Tcm	PIM1
RPS20	5.36E-70	0.260797	0.62	0.506	1.51E-65	CD4.Tcm	RPS20
ZC3H12A	1.14E-67	0.28733	0.286	0.191	3.19E-63	CD4.Tcm	ZC3H12A
RSL1D1	4.26E-67	0.293437	0.394	0.29	1.20E-62	CD4.Tcm	RSL1D1
MYADM1	1.82E-66	0.289645	0.431	0.321	5.12E-62	CD4.Tcm	MYADM

TOMM20	4.29E-66	0.25049	0.469	0.355	1.21E-61	CD4.Tcm	TOMM20
CORO1B	4.23E-65	0.30805	0.287	0.195	1.19E-60	CD4.Tcm	CORO1B
MZT2A1	1.89E-64	0.251829	0.403	0.295	5.31E-60	CD4.Tcm	MZT2A
NOSIP	4.53E-61	0.273856	0.378	0.277	1.27E-56	CD4.Tcm	NOSIP
RPL22L1	1.94E-54	0.267566	0.45	0.349	5.46E-50	CD4.Tcm	RPL22L1
AL118516.:	1.32E-48	0.257475	0.28	0.199	3.72E-44	CD4.Tcm	AL118516.1
GPR65	1.28E-45	0.268457	0.31	0.23	3.61E-41	CD4.Tcm	GPR65
HSPA1B	0	2.50845	0.695	0.36	0	CD8.Tearly	HSPA1B
HSPA1A	0	2.497301	0.618	0.326	0	CD8.Tearly	HSPA1A
RPL301	5.55E-210	0.398815	0.985	0.921	1.56E-205	CD8.Tearly	RPL30
RPL282	1.42E-169	0.353259	0.986	0.935	4.01E-165	CD8.Tearly	RPL28
IL321	7.11E-168	0.525132	0.886	0.749	2.00E-163	CD8.Tearly	IL32
RPS15A2	2.03E-164	0.351715	0.983	0.919	5.72E-160	CD8.Tearly	RPS15A
DNAJB1	5.71E-164	1.411007	0.756	0.745	1.61E-159	CD8.Tearly	DNAJB1
RPS151	1.10E-151	0.355317	0.968	0.912	3.09E-147	CD8.Tearly	RPS15
B2M1	3.69E-137	0.28178	0.995	0.968	1.04E-132	CD8.Tearly	B2M
RPL111	2.92E-136	0.334987	0.975	0.915	8.21E-132	CD8.Tearly	RPL11
FAU2	2.44E-133	0.364972	0.958	0.897	6.86E-129	CD8.Tearly	FAU
RPS282	5.61E-132	0.335147	0.973	0.91	1.58E-127	CD8.Tearly	RPS28
RPS121	2.28E-129	0.301927	0.979	0.928	6.42E-125	CD8.Tearly	RPS12
HSP90AA1	1.74E-128	0.994435	0.797	0.801	4.88E-124	CD8.Tearly	HSP90AA1
ATP5F1E1	2.73E-125	0.460658	0.839	0.787	7.69E-121	CD8.Tearly	ATP5F1E
RPS191	1.20E-119	0.345433	0.98	0.929	3.36E-115	CD8.Tearly	RPS19
CD3E1	6.66E-118	0.441359	0.828	0.734	1.87E-113	CD8.Tearly	CD3E
RPL191	4.65E-117	0.302693	0.972	0.914	1.31E-112	CD8.Tearly	RPL19
CD22	1.66E-116	0.631438	0.663	0.568	4.66E-112	CD8.Tearly	CD2
RPL321	1.91E-112	0.295694	0.973	0.919	5.36E-108	CD8.Tearly	RPL32
RPL391	4.22E-111	0.317522	0.966	0.902	1.19E-106	CD8.Tearly	RPL39
RPS261	8.45E-110	0.445376	0.935	0.892	2.38E-105	CD8.Tearly	RPS26
RPS252	8.95E-110	0.32607	0.944	0.886	2.52E-105	CD8.Tearly	RPS25
SH3BGRL3:	2.20E-109	0.426818	0.866	0.803	6.20E-105	CD8.Tearly	SH3BGRL3
RPL18A1	1.51E-103	0.29462	0.965	0.907	4.26E-99	CD8.Tearly	RPL18A
RPL361	2.30E-102	0.316606	0.926	0.879	6.46E-98	CD8.Tearly	RPL36
RPL7A2	1.54E-101	0.31475	0.96	0.899	4.33E-97	CD8.Tearly	RPL7A
RPS72	6.16E-99	0.30511	0.954	0.897	1.73E-94	CD8.Tearly	RPS7
CCL51	4.98E-98	0.53588	0.821	0.721	1.40E-93	CD8.Tearly	CCL5
RPS27A1	9.14E-97	0.276993	0.969	0.916	2.57E-92	CD8.Tearly	RPS27A
RPS212	1.82E-96	0.318684	0.932	0.878	5.12E-92	CD8.Tearly	RPS21
RPL101	2.07E-96	0.282079	0.987	0.939	5.84E-92	CD8.Tearly	RPL10
RPS24	8.63E-96	0.280812	0.963	0.909	2.43E-91	CD8.Tearly	RPS24
RPS32	1.37E-95	0.289468	0.973	0.912	3.84E-91	CD8.Tearly	RPS3
AL627171.:	7.17E-94	0.824035	0.302	0.185	2.02E-89	CD8.Tearly	AL627171.2
HSPE1	1.08E-92	1.064846	0.505	0.435	3.05E-88	CD8.Tearly	HSPE1
S100A62	4.54E-92	0.529045	0.677	0.612	1.28E-87	CD8.Tearly	S100A6
RPL141	1.87E-91	0.306806	0.939	0.891	5.25E-87	CD8.Tearly	RPL14
RPS271	9.92E-89	0.279472	0.98	0.932	2.79E-84	CD8.Tearly	RPS27
RPL121	1.34E-85	0.316851	0.954	0.904	3.77E-81	CD8.Tearly	RPL12

MYL12A	4.92E-85	0.403759	0.802	0.755	1.39E-80	CD8.Tearly MYL12A
PPDPF1	1.08E-84	0.435318	0.767	0.722	3.05E-80	CD8.Tearly PPDPF
RPL341	1.81E-84	0.25828	0.965	0.908	5.09E-80	CD8.Tearly RPL34
RPS131	2.79E-83	0.29503	0.948	0.886	7.84E-79	CD8.Tearly RPS13
RPL291	3.08E-83	0.280209	0.952	0.89	8.67E-79	CD8.Tearly RPL29
RPS231	1.16E-81	0.250587	0.966	0.909	3.26E-77	CD8.Tearly RPS23
HSPH1	1.63E-80	0.903315	0.434	0.347	4.59E-76	CD8.Tearly HSPH1
RPS181	1.38E-78	0.260498	0.97	0.915	3.87E-74	CD8.Tearly RPS18
RPLP21	2.37E-78	0.280958	0.935	0.886	6.67E-74	CD8.Tearly RPLP2
RPL81	3.84E-77	0.299309	0.927	0.88	1.08E-72	CD8.Tearly RPL8
GIMAP7	6.36E-77	0.661076	0.467	0.373	1.79E-72	CD8.Tearly GIMAP7
RPL181	7.87E-73	0.263493	0.948	0.892	2.21E-68	CD8.Tearly RPL18
TMA71	1.20E-72	0.449244	0.721	0.694	3.36E-68	CD8.Tearly TMA7
GZMK1	1.20E-67	0.546781	0.556	0.446	3.37E-63	CD8.Tearly GZMK
RPL35A1	1.29E-67	0.262789	0.932	0.879	3.63E-63	CD8.Tearly RPL35A
RPL61	1.23E-66	0.263441	0.933	0.886	3.46E-62	CD8.Tearly RPL6
CD522	2.72E-66	0.415168	0.724	0.663	7.64E-62	CD8.Tearly CD52
RPL351	6.31E-59	0.274864	0.875	0.843	1.78E-54	CD8.Tearly RPL35
HSPD1	1.93E-57	0.832965	0.432	0.373	5.43E-53	CD8.Tearly HSPD1
CD3D1	3.81E-57	0.392335	0.674	0.6	1.07E-52	CD8.Tearly CD3D
CD8B1	4.48E-54	0.677127	0.379	0.29	1.26E-49	CD8.Tearly CD8B
S100A41	4.89E-52	0.418403	0.703	0.659	1.38E-47	CD8.Tearly S100A4
RPL36AL2	1.10E-48	0.332412	0.779	0.762	3.09E-44	CD8.Tearly RPL36AL
PLAAT41	2.03E-47	0.471657	0.617	0.587	5.71E-43	CD8.Tearly PLAAT4
THEMIS	7.03E-45	0.445008	0.42	0.342	1.98E-40	CD8.Tearly THEMIS
INPP4B	2.48E-42	0.329575	0.374	0.291	6.99E-38	CD8.Tearly INPP4B
LINC00861	1.85E-41	0.56773	0.296	0.225	5.21E-37	CD8.Tearly LINC00861
EEF1D	1.29E-40	0.264707	0.826	0.813	3.62E-36	CD8.Tearly EEF1D
RPS291	1.66E-40	0.28446	0.826	0.804	4.66E-36	CD8.Tearly RPS29
IFITM1	1.81E-40	0.315555	0.806	0.775	5.08E-36	CD8.Tearly IFITM1
RPL221	1.25E-39	0.25604	0.817	0.798	3.51E-35	CD8.Tearly RPL22
PTPRCAP1	2.85E-36	0.57425	0.486	0.435	8.03E-32	CD8.Tearly PTPRCAP
RPS4Y1	2.78E-35	0.435371	0.515	0.465	7.82E-31	CD8.Tearly RPS4Y1
S100A101	1.19E-34	0.398981	0.574	0.551	3.34E-30	CD8.Tearly S100A10
ATP5MC2	1.83E-32	0.304472	0.716	0.707	5.14E-28	CD8.Tearly ATP5MC2
MYL12B1	4.10E-32	0.358056	0.637	0.637	1.15E-27	CD8.Tearly MYL12B
PFDN51	6.55E-32	0.265424	0.775	0.772	1.84E-27	CD8.Tearly PFDN5
GIMAP4	2.35E-31	0.503442	0.39	0.343	6.60E-27	CD8.Tearly GIMAP4
RESF11	2.38E-31	0.434017	0.536	0.511	6.71E-27	CD8.Tearly RESF1
RAC21	4.98E-30	0.304626	0.673	0.66	1.40E-25	CD8.Tearly RAC2
PSMB91	6.22E-29	0.416819	0.514	0.491	1.75E-24	CD8.Tearly PSMB9
GIMAP5	7.80E-29	0.52771	0.295	0.242	2.19E-24	CD8.Tearly GIMAP5
CD8A1	1.36E-28	0.408942	0.447	0.384	3.82E-24	CD8.Tearly CD8A
CLIC11	1.39E-28	0.308388	0.647	0.635	3.91E-24	CD8.Tearly CLIC1
TOMM72	1.73E-28	0.294113	0.68	0.68	4.88E-24	CD8.Tearly TOMM7
GZMA1	1.27E-27	0.317386	0.61	0.553	3.56E-23	CD8.Tearly GZMA
TC2N	1.38E-27	0.36076	0.48	0.441	3.89E-23	CD8.Tearly TC2N

COX7C1	1.80E-27	0.308585	0.662	0.667	5.06E-23	CD8.Tearly	COX7C
CACYBP	2.60E-27	0.603421	0.317	0.276	7.31E-23	CD8.Tearly	CACYBP
HCST1	5.11E-26	0.276571	0.719	0.707	1.44E-21	CD8.Tearly	HCST
TRAF3IP3	1.84E-25	0.44016	0.38	0.339	5.17E-21	CD8.Tearly	TRAF3IP3
OST41	3.14E-25	0.36318	0.585	0.587	8.84E-21	CD8.Tearly	OST4
MIF1	3.84E-25	0.334406	0.645	0.649	1.08E-20	CD8.Tearly	MIF
MDFIC	4.72E-25	0.404313	0.323	0.272	1.33E-20	CD8.Tearly	MDFIC
LY6E	5.38E-25	0.392494	0.565	0.555	1.51E-20	CD8.Tearly	LY6E
UQCR11	4.94E-24	0.371102	0.537	0.535	1.39E-19	CD8.Tearly	UQCR11
SEPTIN1	1.46E-23	0.404625	0.463	0.442	4.11E-19	CD8.Tearly	SEPTIN1
PLP21	9.43E-23	0.428891	0.334	0.291	2.65E-18	CD8.Tearly	PLP2
HSPB1	1.36E-22	0.434005	0.322	0.28	3.83E-18	CD8.Tearly	HSPB1
PSME1	4.15E-22	0.320415	0.615	0.62	1.17E-17	CD8.Tearly	PSME1
CD3G1	1.12E-21	0.377005	0.468	0.438	3.15E-17	CD8.Tearly	CD3G
TRIM22	1.10E-20	0.414739	0.427	0.405	3.09E-16	CD8.Tearly	TRIM22
LINC01934	1.65E-20	0.280921	0.263	0.211	4.64E-16	CD8.Tearly	LINC01934
CD991	2.20E-20	0.251713	0.693	0.699	6.19E-16	CD8.Tearly	CD99
HSPA8	2.14E-19	0.345914	0.791	0.793	6.03E-15	CD8.Tearly	HSPA8
SIT11	4.27E-19	0.406985	0.257	0.216	1.20E-14	CD8.Tearly	SIT1
TECR	2.77E-18	0.48702	0.397	0.382	7.79E-14	CD8.Tearly	TECR
RCSD1	2.83E-18	0.418955	0.414	0.398	7.95E-14	CD8.Tearly	RCSD1
COX6B1	1.75E-17	0.327387	0.528	0.534	4.93E-13	CD8.Tearly	COX6B1
SNRPD21	1.98E-17	0.299626	0.59	0.603	5.58E-13	CD8.Tearly	SNRPD2
HINT12	8.56E-17	0.265179	0.605	0.612	2.41E-12	CD8.Tearly	HINT1
SSR4	2.59E-16	0.300624	0.562	0.578	7.30E-12	CD8.Tearly	SSR4
GIMAP1	6.55E-16	0.444306	0.297	0.27	1.84E-11	CD8.Tearly	GIMAP1
CD271	1.54E-14	0.372745	0.345	0.318	4.32E-10	CD8.Tearly	CD27
COMMD6	2.78E-14	0.27525	0.592	0.613	7.83E-10	CD8.Tearly	COMMD6
CD37	6.63E-14	0.262951	0.62	0.65	1.87E-09	CD8.Tearly	CD37
GMFG1	1.46E-13	0.292674	0.552	0.573	4.10E-09	CD8.Tearly	GMFG
HSP90AB1	3.97E-13	0.611309	0.677	0.735	1.12E-08	CD8.Tearly	HSP90AB1
ISG15	1.52E-12	0.447188	0.305	0.277	4.27E-08	CD8.Tearly	ISG15
MZT2B	3.42E-12	0.313637	0.505	0.517	9.61E-08	CD8.Tearly	MZT2B
PCED1B-AS	4.83E-12	0.282389	0.526	0.539	1.36E-07	CD8.Tearly	PCED1B-AS1
GSTK1	6.19E-12	0.301572	0.494	0.505	1.74E-07	CD8.Tearly	GSTK1
ELOB	8.74E-12	0.322569	0.494	0.512	2.46E-07	CD8.Tearly	ELOB
TRBC21	1.07E-11	0.321975	0.468	0.462	3.01E-07	CD8.Tearly	TRBC2
UQCRB1	9.54E-11	0.271344	0.583	0.61	2.68E-06	CD8.Tearly	UQCRB
LCK1	9.90E-11	0.25955	0.522	0.531	2.78E-06	CD8.Tearly	LCK
KRTCAP2	1.29E-10	0.320366	0.455	0.469	3.64E-06	CD8.Tearly	KRTCAP2
APRT1	4.43E-10	0.324977	0.466	0.482	1.25E-05	CD8.Tearly	APRT
MX1	6.10E-10	0.380373	0.259	0.232	1.72E-05	CD8.Tearly	MX1
CKLF	5.02E-09	0.341586	0.369	0.365	0.000141	CD8.Tearly	CKLF
ANAPC16	3.46E-08	0.276375	0.464	0.48	0.000974	CD8.Tearly	ANAPC16
RBL2	4.00E-08	0.322081	0.384	0.384	0.001127	CD8.Tearly	RBL2
DENND2D	5.89E-08	0.3225	0.294	0.28	0.001657	CD8.Tearly	DENND2D
EDF1	2.12E-06	0.267963	0.488	0.522	0.059561	CD8.Tearly	EDF1

GZMH1	2.16E-06	0.303082	0.354	0.339	0.060714	CD8.Tearly GZMH
ATP5MD	2.55E-06	0.32124	0.383	0.397	0.071637	CD8.Tearly ATP5MD
CD481	3.10E-06	0.28679	0.436	0.455	0.087134	CD8.Tearly CD48
FP671120.4	3.70E-06	0.271324	0.412	0.402	0.103977	CD8.Tearly FP671120.4
SOD1	6.07E-06	0.287717	0.437	0.462	0.170913	CD8.Tearly SOD1
SAMD3	6.98E-06	0.287864	0.302	0.294	0.196372	CD8.Tearly SAMD3
SMDT11	2.16E-05	0.315289	0.363	0.375	0.60708	CD8.Tearly SMDT1
TBC1D10C	3.55E-05	0.2524	0.401	0.412	0.998522	CD8.Tearly TBC1D10C
IFI6	0.000184	0.280138	0.283	0.273	1	CD8.Tearly IFI6
OCIAD21	0.000264	0.288867	0.292	0.294	1	CD8.Tearly OCIAD2
DRAP1	0.000272	0.265089	0.409	0.433	1	CD8.Tearly DRAP1
SLFN5	0.000496	0.279393	0.33	0.336	1	CD8.Tearly SLFN5
SYNRG	0.000573	0.258898	0.29	0.291	1	CD8.Tearly SYNRG
CHCHD10	0.000613	0.306097	0.264	0.266	1	CD8.Tearly CHCHD10
TOMM6	0.000722	0.315392	0.334	0.35	1	CD8.Tearly TOMM6
UBB	0.00079	0.314652	0.648	0.698	1	CD8.Tearly UBB
C4orf3	0.001003	0.292789	0.341	0.358	1	CD8.Tearly C4orf3
ATP5F1D	0.001037	0.261206	0.431	0.466	1	CD8.Tearly ATP5F1D
SHISA5	0.001774	0.278388	0.321	0.331	1	CD8.Tearly SHISA5
ARL6IP4	0.002036	0.257689	0.39	0.417	1	CD8.Tearly ARL6IP4
TSTD1	0.002145	0.30076	0.271	0.274	1	CD8.Tearly TSTD1
ZBTB381	0.002639	0.337467	0.251	0.252	1	CD8.Tearly ZBTB38
TMEM258	0.002738	0.266796	0.379	0.404	1	CD8.Tearly TMEM258
NDUFA3	0.002937	0.319003	0.302	0.313	1	CD8.Tearly NDUFA3
UQCR10	0.003804	0.290713	0.384	0.411	1	CD8.Tearly UQCR10
PPP1R18	0.009945	0.25993	0.313	0.325	1	CD8.Tearly PPP1R18
CNOT6L	0	2.164156	0.662	0.491	0	CD4-CD8-.1CNOT6L
SIK3	0	2.110321	0.71	0.547	0	CD4-CD8-.1SIK3
ATXN1	0	2.016418	0.573	0.372	0	CD4-CD8-.1ATXN1
CHST11	0	1.849896	0.659	0.532	0	CD4-CD8-.1CHST11
ARHGAP15	0	1.799635	0.71	0.598	0	CD4-CD8-.1ARHGAP15
PITPNC1	0	1.771014	0.616	0.512	0	CD4-CD8-.1PITPNC1
CDC42SE2	0	1.722362	0.637	0.52	0	CD4-CD8-.1CDC42SE2
PRKCH	0	1.700742	0.745	0.651	0	CD4-CD8-.1PRKCH
FYN	0	1.698488	0.828	0.755	0	CD4-CD8-.1FYN
CBLB	0	1.672405	0.678	0.584	0	CD4-CD8-.1CBLB
MT-ATP8	0	1.661847	0.77	0.687	0	CD4-CD8-.1MT-ATP8
PARP8	0	1.629192	0.764	0.706	0	CD4-CD8-.1PARP8
CELF2	0	1.623366	0.721	0.643	0	CD4-CD8-.1CELF2
EML4	0	1.562375	0.655	0.566	0	CD4-CD8-.1EML4
MT-ND3	0	1.535181	0.86	0.832	0	CD4-CD8-.1MT-ND3
MBNL1	0	1.510072	0.728	0.708	0	CD4-CD8-.1MBNL1
MT-CO1	0	1.457356	0.98	0.979	0	CD4-CD8-.1MT-CO1
MT-CO3	0	1.36779	0.924	0.92	0	CD4-CD8-.1MT-CO3
MT-ND2	0	1.340903	0.838	0.82	0	CD4-CD8-.1MT-ND2
MT-CO2	0	1.337471	0.957	0.957	0	CD4-CD8-.1MT-CO2
MT-ND1	0	1.328868	0.828	0.806	0	CD4-CD8-.1MT-ND1

MT-ND4L	0	1.327001	0.874	0.865	0	CD4-CD8-.1	MT-ND4L
MT-CYB	0	1.304084	0.941	0.936	0	CD4-CD8-.1	MT-CYB
MT-ND5	0	1.242419	0.763	0.764	0	CD4-CD8-.1	MT-ND5
MALAT1	0	1.236757	0.999	0.999	0	CD4-CD8-.1	MALAT1
MT-ND4	0	1.176821	0.805	0.809	0	CD4-CD8-.1	MT-ND4
SMCHD1	1.70E-305	1.544961	0.668	0.607	4.77E-301	CD4-CD8-.1	SMCHD1
ANKRD44	1.53E-304	1.55176	0.618	0.523	4.32E-300	CD4-CD8-.1	ANKRD44
PPP1R16B	1.41E-295	1.786587	0.539	0.376	3.98E-291	CD4-CD8-.1	PPP1R16B
LRBA	7.35E-284	1.668446	0.507	0.342	2.07E-279	CD4-CD8-.1	LRBA
CYTH1	3.51E-282	1.755742	0.572	0.46	9.86E-278	CD4-CD8-.1	CYTH1
FOXP1	1.91E-268	1.625084	0.599	0.51	5.37E-264	CD4-CD8-.1	FOXP1
FNBP1	2.30E-262	1.472995	0.611	0.542	6.47E-258	CD4-CD8-.1	FNBP1
LINC01619	9.96E-248	1.823284	0.499	0.347	2.80E-243	CD4-CD8-.1	LINC01619
BCL11B	1.64E-241	1.558263	0.549	0.44	4.63E-237	CD4-CD8-.1	BCL11B
RABGAP1L	1.95E-238	1.533374	0.58	0.505	5.47E-234	CD4-CD8-.1	RABGAP1L
STAT4	4.74E-236	1.549442	0.593	0.518	1.33E-231	CD4-CD8-.1	STAT4
MT-ATP6	3.32E-226	1.151787	0.8	0.817	9.34E-222	CD4-CD8-.1	MT-ATP6
UTRN	3.65E-224	1.482364	0.543	0.443	1.03E-219	CD4-CD8-.1	UTRN
SKAP1	8.97E-220	1.451868	0.61	0.573	2.52E-215	CD4-CD8-.1	SKAP1
PIP4K2A	2.04E-218	1.572894	0.588	0.53	5.75E-214	CD4-CD8-.1	PIP4K2A
MSI2	2.16E-213	1.595088	0.342	0.177	6.08E-209	CD4-CD8-.1	MSI2
ARID1B	6.19E-208	1.557144	0.454	0.314	1.74E-203	CD4-CD8-.1	ARID1B
AC016831.	3.27E-204	1.573329	0.263	0.113	9.21E-200	CD4-CD8-.1	AC016831.7
PCNX1	4.92E-204	1.609411	0.474	0.343	1.38E-199	CD4-CD8-.1	PCNX1
CDC14A	1.39E-202	1.886222	0.416	0.263	3.92E-198	CD4-CD8-.1	CDC14A
CEMIP2	9.25E-200	1.498349	0.597	0.554	2.60E-195	CD4-CD8-.1	CEMIP2
ANKRD11	5.61E-197	1.500191	0.514	0.418	1.58E-192	CD4-CD8-.1	ANKRD11
ZBTB20	9.22E-195	1.642945	0.496	0.39	2.60E-190	CD4-CD8-.1	ZBTB20
PPP2R5C	4.87E-193	1.303229	0.582	0.534	1.37E-188	CD4-CD8-.1	PPP2R5C
FOXO1	3.09E-192	1.692719	0.479	0.363	8.69E-188	CD4-CD8-.1	FOXO1
KANSL1	2.01E-189	1.557611	0.436	0.3	5.66E-185	CD4-CD8-.1	KANSL1
ARL15	1.86E-188	1.595315	0.335	0.182	5.22E-184	CD4-CD8-.1	ARL15
FOXN3	1.52E-183	1.408262	0.541	0.47	4.27E-179	CD4-CD8-.1	FOXN3
MGAT5	2.69E-182	1.771732	0.386	0.245	7.56E-178	CD4-CD8-.1	MGAT5
KDM6A	1.38E-175	1.515057	0.35	0.205	3.89E-171	CD4-CD8-.1	KDM6A
TNRC6B	8.66E-174	1.498545	0.464	0.355	2.44E-169	CD4-CD8-.1	TNRC6B
IQGAP2	1.22E-164	1.395399	0.518	0.452	3.44E-160	CD4-CD8-.1	IQGAP2
PTPRC	7.81E-161	0.840189	0.776	0.887	2.20E-156	CD4-CD8-.1	PTPRC
SYTL3	9.21E-158	1.277423	0.629	0.635	2.59E-153	CD4-CD8-.1	SYTL3
PDE3B	1.24E-157	1.788722	0.458	0.36	3.50E-153	CD4-CD8-.1	PDE3B
KLF12	3.45E-153	1.547195	0.378	0.253	9.72E-149	CD4-CD8-.1	KLF12
ARIH1	2.68E-150	1.428158	0.477	0.405	7.55E-146	CD4-CD8-.1	ARIH1
ELMO1	1.26E-148	1.39041	0.452	0.35	3.55E-144	CD4-CD8-.1	ELMO1
ARHGAP26	1.01E-142	1.393844	0.467	0.38	2.85E-138	CD4-CD8-.1	ARHGAP26
MAML2	6.65E-142	1.661818	0.424	0.324	1.87E-137	CD4-CD8-.1	MAML2
SLC38A1	3.47E-141	1.322342	0.514	0.472	9.76E-137	CD4-CD8-.1	SLC38A1
CDK17	8.06E-141	1.435472	0.47	0.395	2.27E-136	CD4-CD8-.1	CDK17

VPS13B	2.27E-140	1.404288	0.357	0.233	6.39E-136	CD4-CD8-.1	VPS13B
DENND4A	1.10E-137	1.518928	0.406	0.3	3.10E-133	CD4-CD8-.1	DENND4A
DOCK10	2.89E-137	1.386054	0.486	0.424	8.14E-133	CD4-CD8-.1	DOCK10
FP236383.3	8.20E-135	1.340899	0.571	0.408	2.31E-130	CD4-CD8-.1	FP236383.3
THEMIS1	4.06E-132	1.536703	0.433	0.341	1.14E-127	CD4-CD8-.1	THEMIS
TMEM181	1.78E-129	1.341725	0.302	0.179	5.02E-125	CD4-CD8-.1	TMEM181
STAG1	2.18E-129	1.431817	0.411	0.317	6.15E-125	CD4-CD8-.1	STAG1
JARID2	2.86E-127	1.449219	0.351	0.239	8.05E-123	CD4-CD8-.1	JARID2
CMIP	4.29E-127	1.525485	0.355	0.24	1.21E-122	CD4-CD8-.1	CMIP
CAMK4	6.63E-127	1.445595	0.458	0.383	1.86E-122	CD4-CD8-.1	CAMK4
ZFAND3	1.73E-126	1.403914	0.394	0.291	4.87E-122	CD4-CD8-.1	ZFAND3
RNF19A	1.77E-125	1.43088	0.486	0.417	4.99E-121	CD4-CD8-.1	RNF19A
EXOC4	3.62E-124	1.353026	0.362	0.251	1.02E-119	CD4-CD8-.1	EXOC4
PDE7A	4.87E-123	1.403608	0.478	0.422	1.37E-118	CD4-CD8-.1	PDE7A
FKBP5	3.64E-118	1.270359	0.552	0.561	1.02E-113	CD4-CD8-.1	FKBP5
NEAT1	1.11E-116	1.21965	0.545	0.517	3.13E-112	CD4-CD8-.1	NEAT1
KMT2C	4.16E-116	1.340067	0.4	0.312	1.17E-111	CD4-CD8-.1	KMT2C
SLFN12L	4.40E-116	1.333455	0.286	0.17	1.24E-111	CD4-CD8-.1	SLFN12L
FTX	7.72E-115	1.325771	0.278	0.164	2.17E-110	CD4-CD8-.1	FTX
RORA1	1.31E-114	1.503161	0.472	0.416	3.68E-110	CD4-CD8-.1	RORA
AAK1	1.45E-114	1.293358	0.448	0.378	4.08E-110	CD4-CD8-.1	AAK1
BCAS3	1.85E-114	1.43663	0.285	0.172	5.22E-110	CD4-CD8-.1	BCAS3
AKAP13	4.16E-114	1.01546	0.595	0.624	1.17E-109	CD4-CD8-.1	AKAP13
FAM172A	2.83E-113	1.331023	0.325	0.214	7.97E-109	CD4-CD8-.1	FAM172A
TOX	3.74E-112	1.77981	0.387	0.294	1.05E-107	CD4-CD8-.1	TOX
DOCK2	7.31E-112	1.342534	0.401	0.313	2.06E-107	CD4-CD8-.1	DOCK2
PAN3	1.18E-111	1.316881	0.316	0.208	3.33E-107	CD4-CD8-.1	PAN3
ITK	1.82E-111	1.366581	0.475	0.424	5.12E-107	CD4-CD8-.1	ITK
INPP4B1	5.41E-111	1.486777	0.385	0.29	1.52E-106	CD4-CD8-.1	INPP4B
RUNX1	1.60E-110	1.427863	0.37	0.272	4.50E-106	CD4-CD8-.1	RUNX1
PRKCB	2.16E-109	1.38951	0.414	0.337	6.07E-105	CD4-CD8-.1	PRKCB
ANK3	8.45E-109	1.55541	0.256	0.148	2.38E-104	CD4-CD8-.1	ANK3
MACF1	4.51E-108	1.190364	0.486	0.448	1.27E-103	CD4-CD8-.1	MACF1
SYNE2	1.74E-107	1.281938	0.46	0.407	4.88E-103	CD4-CD8-.1	SYNE2
HERC1	2.06E-105	1.269153	0.402	0.318	5.80E-101	CD4-CD8-.1	HERC1
ATP8A1	2.69E-105	1.432444	0.365	0.271	7.57E-101	CD4-CD8-.1	ATP8A1
RNF213	1.62E-104	1.106927	0.542	0.547	4.57E-100	CD4-CD8-.1	RNF213
CDKAL1	4.33E-104	1.316896	0.284	0.178	1.22E-99	CD4-CD8-.1	CDKAL1
MT-ND6	3.48E-103	1.435398	0.462	0.398	9.80E-99	CD4-CD8-.1	MT-ND6
IKZF1	5.35E-103	1.045444	0.592	0.643	1.50E-98	CD4-CD8-.1	IKZF1
PACS1	7.98E-103	1.40053	0.395	0.316	2.25E-98	CD4-CD8-.1	PACS1
PTPRJ	8.79E-103	1.434021	0.352	0.259	2.47E-98	CD4-CD8-.1	PTPRJ
SMYD3	1.24E-102	1.529411	0.333	0.236	3.48E-98	CD4-CD8-.1	SMYD3
LPP	1.21E-99	1.326267	0.404	0.334	3.41E-95	CD4-CD8-.1	LPP
JMJD1C	9.75E-97	1.301499	0.441	0.393	2.74E-92	CD4-CD8-.1	JMJD1C
INPP5D	1.34E-95	1.362406	0.438	0.387	3.76E-91	CD4-CD8-.1	INPP5D
ZHX2	4.68E-95	1.34797	0.269	0.168	1.32E-90	CD4-CD8-.1	ZHX2

DYRK1A	1.21E-94	1.296523	0.317	0.221	3.41E-90	CD4-CD8-.1	DYRK1A
SKI	2.50E-93	1.413046	0.316	0.222	7.02E-89	CD4-CD8-.1	SKI
KAT2B	1.43E-92	1.299586	0.257	0.158	4.03E-88	CD4-CD8-.1	KAT2B
TRAPPC9	1.95E-92	1.297415	0.285	0.188	5.48E-88	CD4-CD8-.1	TRAPPC9
MYH9	5.21E-92	0.881636	0.612	0.69	1.47E-87	CD4-CD8-.1	MYH9
BIRC6	7.16E-91	1.271338	0.395	0.329	2.01E-86	CD4-CD8-.1	BIRC6
NIBAN1	1.04E-90	1.478525	0.408	0.345	2.93E-86	CD4-CD8-.1	NIBAN1
FBXW7	1.64E-89	1.269422	0.302	0.209	4.62E-85	CD4-CD8-.1	FBXW7
WWOX	2.97E-89	1.536743	0.276	0.18	8.37E-85	CD4-CD8-.1	WWOX
RNF38	1.25E-88	1.258942	0.288	0.192	3.52E-84	CD4-CD8-.1	RNF38
ARID4B	1.92E-87	1.182679	0.477	0.465	5.39E-83	CD4-CD8-.1	ARID4B
ATP9B	3.28E-87	1.157501	0.268	0.171	9.24E-83	CD4-CD8-.1	ATP9B
RAPGEF1	1.26E-85	1.274729	0.374	0.301	3.55E-81	CD4-CD8-.1	RAPGEF1
COP1	1.58E-84	1.213036	0.293	0.202	4.45E-80	CD4-CD8-.1	COP1
DOCK8	3.98E-84	1.147113	0.482	0.469	1.12E-79	CD4-CD8-.1	DOCK8
ARAP2	4.91E-84	1.286205	0.404	0.343	1.38E-79	CD4-CD8-.1	ARAP2
ABCC1	8.03E-83	1.268722	0.273	0.181	2.26E-78	CD4-CD8-.1	ABCC1
USP36	5.46E-82	1.345246	0.399	0.344	1.54E-77	CD4-CD8-.1	USP36
TBL1XR1	8.68E-82	1.329138	0.294	0.207	2.44E-77	CD4-CD8-.1	TBL1XR1
LINC01934	1.87E-81	1.46138	0.295	0.207	5.26E-77	CD4-CD8-.1	LINC01934
NCOA2	7.12E-81	1.303835	0.333	0.251	2.00E-76	CD4-CD8-.1	NCOA2
CLASP1	2.07E-80	1.159827	0.275	0.183	5.82E-76	CD4-CD8-.1	CLASP1
BTBD9	2.22E-80	1.37197	0.289	0.202	6.25E-76	CD4-CD8-.1	BTBD9
FP671120.4	1.79E-79	1.365663	0.472	0.395	5.03E-75	CD4-CD8-.1	FP671120.4
PRKCA	8.45E-78	1.361548	0.266	0.179	2.38E-73	CD4-CD8-.1	PRKCA
SIPA1L1	3.73E-77	1.373104	0.376	0.312	1.05E-72	CD4-CD8-.1	SIPA1L1
STIM1	1.63E-76	1.265068	0.291	0.207	4.59E-72	CD4-CD8-.1	STIM1
AKNA	2.06E-76	1.075838	0.497	0.498	5.79E-72	CD4-CD8-.1	AKNA
OSBPL8	1.13E-75	1.330209	0.423	0.389	3.19E-71	CD4-CD8-.1	OSBPL8
RASA3	3.10E-74	1.355746	0.437	0.408	8.72E-70	CD4-CD8-.1	RASA3
DPYD	3.19E-73	1.333309	0.377	0.322	8.98E-69	CD4-CD8-.1	DPYD
PRKCQ	1.28E-72	1.206983	0.343	0.273	3.61E-68	CD4-CD8-.1	PRKCQ
SFMBT2	2.85E-71	1.316024	0.361	0.295	8.01E-67	CD4-CD8-.1	SFMBT2
USP34	3.94E-70	1.172997	0.365	0.305	1.11E-65	CD4-CD8-.1	USP34
MAP3K5	3.95E-70	1.271152	0.291	0.213	1.11E-65	CD4-CD8-.1	MAP3K5
STK39	1.04E-68	1.164968	0.265	0.183	2.92E-64	CD4-CD8-.1	STK39
EPB41	1.50E-68	1.213569	0.375	0.324	4.21E-64	CD4-CD8-.1	EPB41
TNIK	1.62E-68	1.268056	0.304	0.229	4.55E-64	CD4-CD8-.1	TNIK
MED13L	1.65E-68	1.200722	0.289	0.212	4.65E-64	CD4-CD8-.1	MED13L
ANKRD12	1.68E-68	1.094678	0.471	0.47	4.74E-64	CD4-CD8-.1	ANKRD12
NLRC5	4.48E-68	1.161616	0.283	0.203	1.26E-63	CD4-CD8-.1	NLRC5
GATAD2B	1.10E-67	1.176505	0.254	0.173	3.09E-63	CD4-CD8-.1	GATAD2B
FBXL17	1.47E-67	1.267549	0.258	0.178	4.14E-63	CD4-CD8-.1	FBXL17
CAMK1D	1.33E-66	1.394423	0.3	0.228	3.73E-62	CD4-CD8-.1	CAMK1D
PSME4	1.60E-65	1.221094	0.286	0.212	4.52E-61	CD4-CD8-.1	PSME4
VTI1A	6.01E-64	1.207716	0.266	0.189	1.69E-59	CD4-CD8-.1	VTI1A
PIK3R5	9.07E-64	1.182745	0.373	0.323	2.55E-59	CD4-CD8-.1	PIK3R5

SSH2	1.91E-63	1.160758	0.43	0.41	5.36E-59	CD4-CD8-.1	SSH2
BRAF	9.79E-63	1.208564	0.292	0.22	2.75E-58	CD4-CD8-.1	BRAF
PLEKHA2	3.91E-62	1.272004	0.32	0.257	1.10E-57	CD4-CD8-.1	PLEKHA2
FAF1	5.54E-62	1.216433	0.315	0.251	1.56E-57	CD4-CD8-.1	FAF1
ZC3HAV1	1.21E-61	1.047797	0.497	0.531	3.40E-57	CD4-CD8-.1	ZC3HAV1
RALGAPA1	6.33E-61	1.235276	0.376	0.334	1.78E-56	CD4-CD8-.1	RALGAPA1
NKTR	3.42E-59	1.132621	0.338	0.28	9.63E-55	CD4-CD8-.1	NKTR
RASA2	5.99E-58	1.08616	0.427	0.411	1.68E-53	CD4-CD8-.1	RASA2
UBR5	1.41E-56	1.097671	0.264	0.194	3.98E-52	CD4-CD8-.1	UBR5
CRYBG1	3.33E-56	1.266364	0.397	0.369	9.36E-52	CD4-CD8-.1	CRYBG1
WNK1	1.34E-55	1.206309	0.378	0.343	3.78E-51	CD4-CD8-.1	WNK1
CREBBP	8.48E-55	1.10488	0.274	0.207	2.39E-50	CD4-CD8-.1	CREBBP
KAT6A	1.32E-54	1.145133	0.314	0.256	3.72E-50	CD4-CD8-.1	KAT6A
BCL2	1.48E-54	1.58667	0.392	0.374	4.16E-50	CD4-CD8-.1	BCL2
MLLT3	1.72E-54	1.21516	0.252	0.183	4.85E-50	CD4-CD8-.1	MLLT3
CD96	4.85E-54	0.938265	0.537	0.6	1.36E-49	CD4-CD8-.1	CD96
ANKRD17	6.90E-54	1.097687	0.274	0.208	1.94E-49	CD4-CD8-.1	ANKRD17
TBC1D5	1.00E-53	1.173144	0.33	0.278	2.83E-49	CD4-CD8-.1	TBC1D5
STK10	1.81E-53	1.086275	0.4	0.379	5.10E-49	CD4-CD8-.1	STK10
CHD2	3.13E-53	1.08575	0.407	0.391	8.80E-49	CD4-CD8-.1	CHD2
KMT2E	3.25E-53	0.954449	0.492	0.537	9.13E-49	CD4-CD8-.1	KMT2E
SH3KBP1	1.28E-52	1.05698	0.439	0.448	3.60E-48	CD4-CD8-.1	SH3KBP1
ASH1L	1.96E-52	1.142341	0.338	0.292	5.52E-48	CD4-CD8-.1	ASH1L
PPP3CA	1.14E-51	1.215697	0.287	0.227	3.22E-47	CD4-CD8-.1	PPP3CA
RICTOR	1.28E-49	1.066116	0.275	0.212	3.61E-45	CD4-CD8-.1	RICTOR
GNG2	2.17E-49	1.086725	0.488	0.529	6.11E-45	CD4-CD8-.1	GNG2
RNF125	4.92E-48	1.107683	0.43	0.437	1.39E-43	CD4-CD8-.1	RNF125
ATF7IP	6.18E-48	1.103764	0.379	0.356	1.74E-43	CD4-CD8-.1	ATF7IP
CASK	9.11E-47	1.18458	0.312	0.265	2.56E-42	CD4-CD8-.1	CASK
USP9X	1.57E-46	1.087188	0.261	0.201	4.40E-42	CD4-CD8-.1	USP9X
TBC1D22A	2.68E-46	1.114222	0.282	0.226	7.53E-42	CD4-CD8-.1	TBC1D22A
ADK	5.08E-46	1.13326	0.276	0.221	1.43E-41	CD4-CD8-.1	ADK
CD44	5.21E-46	1.031924	0.533	0.615	1.47E-41	CD4-CD8-.1	CD44
GPCPD1	2.62E-43	1.246442	0.308	0.265	7.36E-39	CD4-CD8-.1	GPCPD1
NEK7	2.81E-42	1.161282	0.268	0.215	7.91E-38	CD4-CD8-.1	NEK7
ZSWIM6	3.91E-42	1.155195	0.309	0.265	1.10E-37	CD4-CD8-.1	ZSWIM6
CEP85L	9.95E-42	1.077437	0.255	0.199	2.80E-37	CD4-CD8-.1	CEP85L
TC2N1	1.39E-41	1.041948	0.43	0.448	3.90E-37	CD4-CD8-.1	TC2N
STK4	1.42E-41	0.900761	0.533	0.625	3.99E-37	CD4-CD8-.1	STK4
PPP6R3	1.52E-41	1.063364	0.302	0.258	4.27E-37	CD4-CD8-.1	PPP6R3
SLC4A7	8.28E-41	1.145349	0.293	0.247	2.33E-36	CD4-CD8-.1	SLC4A7
AOAH	1.07E-40	1.28073	0.408	0.403	3.00E-36	CD4-CD8-.1	AOAH
BACH2	2.69E-40	1.365607	0.275	0.226	7.57E-36	CD4-CD8-.1	BACH2
OGT	2.69E-40	1.020872	0.35	0.32	7.58E-36	CD4-CD8-.1	OGT
TCF12	8.11E-40	1.083983	0.298	0.255	2.28E-35	CD4-CD8-.1	TCF12
PBX41	1.93E-39	1.28753	0.279	0.233	5.42E-35	CD4-CD8-.1	PBX4
INPP4A	3.34E-39	1.058985	0.293	0.249	9.39E-35	CD4-CD8-.1	INPP4A

SPIDR	3.99E-39	1.125144	0.283	0.237	1.12E-34	CD4-CD8-.1	SPIDR
VAV3	4.51E-39	1.173951	0.27	0.216	1.27E-34	CD4-CD8-.1	VAV3
PAG1	1.13E-38	1.143184	0.285	0.24	3.19E-34	CD4-CD8-.1	PAG1
TSPAN5	2.00E-38	1.196829	0.276	0.23	5.63E-34	CD4-CD8-.1	TSPAN5
CCNH	4.94E-38	0.840974	0.579	0.685	1.39E-33	CD4-CD8-.1	CCNH
NFAT5	6.82E-38	1.074045	0.287	0.242	1.92E-33	CD4-CD8-.1	NFAT5
CCSER2	4.79E-37	1.065503	0.354	0.338	1.35E-32	CD4-CD8-.1	CCSER2
PPP1CB	2.27E-36	1.164014	0.326	0.301	6.39E-32	CD4-CD8-.1	PPP1CB
NIPBL	3.03E-36	1.017597	0.354	0.34	8.52E-32	CD4-CD8-.1	NIPBL
CARD11	3.82E-36	1.046187	0.289	0.247	1.07E-31	CD4-CD8-.1	CARD11
RIPOR2	4.09E-36	1.153284	0.353	0.332	1.15E-31	CD4-CD8-.1	RIPOR2
RTN4	4.41E-36	0.256493	0.169	0.303	1.24E-31	CD4-CD8-.1	RTN4
ZNF831	2.41E-35	1.087273	0.3	0.262	6.79E-31	CD4-CD8-.1	ZNF831
CUL3	1.40E-34	1.083502	0.34	0.323	3.94E-30	CD4-CD8-.1	CUL3
CD247	2.04E-34	0.814974	0.506	0.571	5.74E-30	CD4-CD8-.1	CD247
KIF13B	8.41E-34	1.112776	0.263	0.22	2.37E-29	CD4-CD8-.1	KIF13B
HIVEP2	9.14E-34	1.089539	0.283	0.246	2.57E-29	CD4-CD8-.1	HIVEP2
SLC7A5	1.17E-33	1.213194	0.365	0.363	3.28E-29	CD4-CD8-.1	SLC7A5
CLEC2D	2.77E-33	1.018504	0.344	0.325	7.80E-29	CD4-CD8-.1	CLEC2D
ZCCHC7	7.38E-33	1.071538	0.265	0.225	2.08E-28	CD4-CD8-.1	ZCCHC7
P2RY8	7.78E-33	1.12359	0.355	0.346	2.19E-28	CD4-CD8-.1	P2RY8
PAK2	1.89E-32	0.283935	0.199	0.341	5.32E-28	CD4-CD8-.1	PAK2
SUCLG2	3.82E-32	1.093834	0.275	0.238	1.08E-27	CD4-CD8-.1	SUCLG2
DDX6	4.84E-32	0.273871	0.229	0.384	1.36E-27	CD4-CD8-.1	DDX6
ATP1A1	3.06E-31	0.333222	0.16	0.28	8.62E-27	CD4-CD8-.1	ATP1A1
ARHGAP45	5.78E-31	0.260085	0.195	0.33	1.63E-26	CD4-CD8-.1	ARHGAP45
CCND3	7.18E-31	0.913681	0.535	0.654	2.02E-26	CD4-CD8-.1	CCND3
SRSF10	1.08E-30	0.284433	0.239	0.4	3.05E-26	CD4-CD8-.1	SRSF10
PUM2	1.30E-30	0.986428	0.28	0.245	3.66E-26	CD4-CD8-.1	PUM2
WTAP	1.70E-30	0.308366	0.216	0.365	4.79E-26	CD4-CD8-.1	WTAP
PTPN22	2.29E-30	1.077651	0.366	0.362	6.44E-26	CD4-CD8-.1	PTPN22
ARHGEF7	6.03E-30	1.159992	0.282	0.248	1.70E-25	CD4-CD8-.1	ARHGEF7
ATXN7	1.04E-29	0.943395	0.259	0.22	2.92E-25	CD4-CD8-.1	ATXN7
RBM6	2.30E-29	1.009936	0.274	0.239	6.47E-25	CD4-CD8-.1	RBM6
TMEM259	2.95E-29	0.310666	0.181	0.309	8.30E-25	CD4-CD8-.1	TMEM259
RAB7A	3.53E-29	0.315942	0.234	0.391	9.94E-25	CD4-CD8-.1	RAB7A
ESYT2	3.55E-29	1.041653	0.283	0.253	9.98E-25	CD4-CD8-.1	ESYT2
KDM4C	4.94E-29	0.981951	0.283	0.252	1.39E-24	CD4-CD8-.1	KDM4C
USP15	9.10E-29	0.92114	0.421	0.461	2.56E-24	CD4-CD8-.1	USP15
PRDM2	1.12E-28	0.950571	0.256	0.217	3.15E-24	CD4-CD8-.1	PRDM2
IDS	1.15E-28	0.333968	0.308	0.501	3.22E-24	CD4-CD8-.1	IDS
MECP2	2.23E-28	1.006908	0.262	0.227	6.27E-24	CD4-CD8-.1	MECP2
KDM6B	3.26E-28	0.259907	0.167	0.281	9.17E-24	CD4-CD8-.1	KDM6B
LDLRAD4	8.56E-28	1.166262	0.368	0.364	2.41E-23	CD4-CD8-.1	LDLRAD4
ATP1B3	1.66E-27	1.307473	0.402	0.422	4.67E-23	CD4-CD8-.1	ATP1B3
CCDC88C	2.01E-27	0.972134	0.33	0.316	5.67E-23	CD4-CD8-.1	CCDC88C
KTN1	5.85E-27	0.283576	0.211	0.347	1.64E-22	CD4-CD8-.1	KTN1

ETS1	6.60E-27	0.666077	0.609	0.749	1.86E-22	CD4-CD8-.1	ETS1
GLS	1.35E-26	1.015406	0.362	0.371	3.80E-22	CD4-CD8-.1	GLS
TAX1BP1	2.42E-26	0.301955	0.212	0.35	6.80E-22	CD4-CD8-.1	TAX1BP1
ATP11B	3.25E-26	0.955166	0.271	0.24	9.14E-22	CD4-CD8-.1	ATP11B
CD55	4.37E-26	0.296818	0.267	0.426	1.23E-21	CD4-CD8-.1	CD55
AUTS2	4.87E-26	1.08293	0.398	0.424	1.37E-21	CD4-CD8-.1	AUTS2
TENT5C2	8.37E-26	0.250594	0.308	0.49	2.36E-21	CD4-CD8-.1	TENT5C
SRPK2	1.34E-25	1.066739	0.314	0.303	3.76E-21	CD4-CD8-.1	SRPK2
PRKACB	1.84E-25	0.337621	0.161	0.268	5.18E-21	CD4-CD8-.1	PRKACB
SEPTIN2	1.91E-25	0.273218	0.172	0.285	5.38E-21	CD4-CD8-.1	SEPTIN2
RPS6KA3	1.03E-24	1.027027	0.32	0.312	2.90E-20	CD4-CD8-.1	RPS6KA3
RFTN1	1.10E-24	1.008286	0.293	0.271	3.10E-20	CD4-CD8-.1	RFTN1
MKLN1	1.13E-24	0.94583	0.25	0.217	3.17E-20	CD4-CD8-.1	MKLN1
MYCBP2	1.52E-24	0.952911	0.363	0.374	4.27E-20	CD4-CD8-.1	MYCBP2
NUP98	1.61E-24	0.970074	0.293	0.273	4.53E-20	CD4-CD8-.1	NUP98
GPRIN3	3.66E-24	1.092318	0.285	0.264	1.03E-19	CD4-CD8-.1	GPRIN3
EIF3A	9.19E-24	0.340701	0.197	0.32	2.59E-19	CD4-CD8-.1	EIF3A
ARHGAP4	1.69E-23	0.349752	0.187	0.304	4.75E-19	CD4-CD8-.1	ARHGAP4
HSPA9	3.40E-23	0.336391	0.202	0.327	9.56E-19	CD4-CD8-.1	HSPA9
RBM39	4.07E-23	0.638869	0.535	0.664	1.15E-18	CD4-CD8-.1	RBM39
GRB2	4.37E-23	0.343912	0.204	0.328	1.23E-18	CD4-CD8-.1	GRB2
PICALM	6.25E-23	0.915978	0.3	0.283	1.76E-18	CD4-CD8-.1	PICALM
FLNA	6.46E-23	0.408671	0.237	0.379	1.82E-18	CD4-CD8-.1	FLNA
URI1	1.71E-22	0.293814	0.159	0.259	4.80E-18	CD4-CD8-.1	URI1
TIPARP	1.81E-22	0.309938	0.172	0.277	5.09E-18	CD4-CD8-.1	TIPARP
TGFB1	2.23E-22	0.436022	0.405	0.656	6.27E-18	CD4-CD8-.1	TGFB1
CCDC91	5.32E-22	1.037994	0.267	0.245	1.50E-17	CD4-CD8-.1	CCDC91
PBXIP11	6.70E-22	0.270184	0.192	0.304	1.88E-17	CD4-CD8-.1	PBXIP1
TNFAIP31	1.07E-21	0.553642	0.699	0.79	3.00E-17	CD4-CD8-.1	TNFAIP3
RAPGEF6	1.63E-21	0.932533	0.292	0.275	4.58E-17	CD4-CD8-.1	RAPGEF6
CFLAR	2.35E-21	0.339867	0.266	0.419	6.62E-17	CD4-CD8-.1	CFLAR
HELZ	3.22E-21	0.933809	0.273	0.252	9.07E-17	CD4-CD8-.1	HELZ
SF1	3.27E-21	0.300076	0.367	0.582	9.20E-17	CD4-CD8-.1	SF1
PRKX	3.28E-21	1.019227	0.253	0.227	9.24E-17	CD4-CD8-.1	PRKX
ABLIM1	5.55E-21	1.089486	0.311	0.305	1.56E-16	CD4-CD8-.1	ABLIM1
SNRNP70	6.59E-21	0.384519	0.195	0.312	1.86E-16	CD4-CD8-.1	SNRNP70
HIF1A	7.98E-21	0.286004	0.193	0.302	2.24E-16	CD4-CD8-.1	HIF1A
CYLD	8.18E-21	0.346236	0.163	0.262	2.30E-16	CD4-CD8-.1	CYLD
KDM2A	9.95E-21	0.946967	0.311	0.305	2.80E-16	CD4-CD8-.1	KDM2A
TMF1	1.23E-20	0.341456	0.17	0.272	3.46E-16	CD4-CD8-.1	TMF1
RASA1	2.26E-20	1.044312	0.3	0.293	6.37E-16	CD4-CD8-.1	RASA1
AMD1	3.81E-20	0.317306	0.178	0.283	1.07E-15	CD4-CD8-.1	AMD1
MIER1	4.00E-20	0.321516	0.165	0.263	1.12E-15	CD4-CD8-.1	MIER1
ARHGEF3	4.04E-20	1.008037	0.296	0.284	1.14E-15	CD4-CD8-.1	ARHGEF3
YPEL51	7.96E-20	0.271369	0.352	0.544	2.24E-15	CD4-CD8-.1	YPEL5
RANBP2	8.44E-20	0.951989	0.394	0.428	2.37E-15	CD4-CD8-.1	RANBP2
CHIC2	8.51E-20	0.347315	0.169	0.269	2.39E-15	CD4-CD8-.1	CHIC2

OFD1	1.34E-19	0.365667	0.162	0.258	3.77E-15	CD4-CD8-.1	OFD1
MAP3K2	1.37E-19	1.011581	0.285	0.275	3.85E-15	CD4-CD8-.1	MAP3K2
OGA	1.70E-19	0.881066	0.302	0.293	4.79E-15	CD4-CD8-.1	OGA
SND1	2.13E-19	0.962117	0.287	0.277	5.99E-15	CD4-CD8-.1	SND1
SMG1	2.49E-19	0.868487	0.275	0.258	7.00E-15	CD4-CD8-.1	SMG1
FRYL	2.93E-19	0.94952	0.257	0.236	8.24E-15	CD4-CD8-.1	FRYL
SMAP1	3.63E-19	0.382665	0.164	0.26	1.02E-14	CD4-CD8-.1	SMAP1
PDE4D1	3.91E-19	1.071107	0.341	0.348	1.10E-14	CD4-CD8-.1	PDE4D
AGO2	4.55E-19	0.96677	0.271	0.256	1.28E-14	CD4-CD8-.1	AGO2
EPS15	1.01E-18	0.968023	0.31	0.308	2.85E-14	CD4-CD8-.1	EPS15
ADGRE5	1.40E-18	0.959191	0.453	0.531	3.95E-14	CD4-CD8-.1	ADGRE5
SETD2	1.60E-18	0.96732	0.306	0.304	4.49E-14	CD4-CD8-.1	SETD2
PLCL2	2.23E-18	0.964273	0.272	0.256	6.28E-14	CD4-CD8-.1	PLCL2
ZNF292	2.54E-18	0.894935	0.274	0.259	7.15E-14	CD4-CD8-.1	ZNF292
ATP2B1	2.87E-18	1.035332	0.263	0.246	8.07E-14	CD4-CD8-.1	ATP2B1
DDX21	3.28E-18	0.35432	0.182	0.283	9.23E-14	CD4-CD8-.1	DDX21
MCUB	3.99E-18	0.439956	0.204	0.318	1.12E-13	CD4-CD8-.1	MCUB
TSPAN14	4.89E-18	0.356321	0.178	0.278	1.37E-13	CD4-CD8-.1	TSPAN14
KLF13	9.07E-18	0.363613	0.239	0.367	2.55E-13	CD4-CD8-.1	KLF13
PTBP3	3.34E-17	0.354482	0.163	0.254	9.40E-13	CD4-CD8-.1	PTBP3
PTPN7	5.39E-17	0.379935	0.184	0.283	1.52E-12	CD4-CD8-.1	PTPN7
SAMHD1	5.82E-17	0.446439	0.243	0.371	1.64E-12	CD4-CD8-.1	SAMHD1
SEPTIN6	7.78E-17	0.409481	0.274	0.423	2.19E-12	CD4-CD8-.1	SEPTIN6
ZAP70	1.15E-16	0.38952	0.256	0.391	3.23E-12	CD4-CD8-.1	ZAP70
C5orf56	1.52E-16	0.359881	0.164	0.253	4.28E-12	CD4-CD8-.1	C5orf56
CAST	1.95E-16	0.442691	0.244	0.372	5.48E-12	CD4-CD8-.1	CAST
SLC2A31	2.36E-16	0.355218	0.396	0.593	6.63E-12	CD4-CD8-.1	SLC2A3
DIP2A	3.44E-16	0.90708	0.288	0.282	9.68E-12	CD4-CD8-.1	DIP2A
TGFBR2	4.45E-16	0.964533	0.363	0.394	1.25E-11	CD4-CD8-.1	TGFBR2
ZNF644	6.06E-16	0.92589	0.287	0.284	1.70E-11	CD4-CD8-.1	ZNF644
SAMSN1	4.33E-15	0.834714	0.441	0.513	1.22E-10	CD4-CD8-.1	SAMSN1
RAB2A	5.49E-15	0.417319	0.235	0.359	1.55E-10	CD4-CD8-.1	RAB2A
FAM49B	6.16E-15	0.883672	0.388	0.442	1.73E-10	CD4-CD8-.1	FAM49B
TRA2A	7.45E-15	0.945605	0.289	0.288	2.10E-10	CD4-CD8-.1	TRA2A
IFI16	8.44E-15	0.418652	0.272	0.412	2.37E-10	CD4-CD8-.1	IFI16
TUT4	1.10E-14	0.851351	0.262	0.251	3.10E-10	CD4-CD8-.1	TUT4
IQGAP1	1.31E-14	0.442982	0.297	0.452	3.69E-10	CD4-CD8-.1	IQGAP1
SPOCK21	1.80E-14	0.332467	0.32	0.48	5.06E-10	CD4-CD8-.1	SPOCK2
PREX1	1.94E-14	0.835019	0.38	0.423	5.45E-10	CD4-CD8-.1	PREX1
PHF3	2.40E-14	0.388704	0.202	0.303	6.76E-10	CD4-CD8-.1	PHF3
SYNE1	2.87E-14	0.864985	0.301	0.303	8.07E-10	CD4-CD8-.1	SYNE1
NFKB1	3.13E-14	0.969732	0.38	0.415	8.80E-10	CD4-CD8-.1	NFKB1
ARID5B	3.60E-14	0.943509	0.352	0.377	1.01E-09	CD4-CD8-.1	ARID5B
YWHAE	6.79E-14	0.390371	0.167	0.251	1.91E-09	CD4-CD8-.1	YWHAE
LYST1	7.80E-14	0.957639	0.297	0.299	2.19E-09	CD4-CD8-.1	LYST
PTPN2	1.01E-13	0.422227	0.176	0.265	2.84E-09	CD4-CD8-.1	PTPN2
YME1L1	1.06E-13	0.407293	0.238	0.356	2.98E-09	CD4-CD8-.1	YME1L1

NDFIP1	1.40E-13	0.512372	0.261	0.392	3.95E-09	CD4-CD8-.1	NDFIP1
OXNAD1	1.65E-13	0.890673	0.382	0.428	4.65E-09	CD4-CD8-.1	OXNAD1
FYB1	2.06E-13	0.272847	0.427	0.639	5.80E-09	CD4-CD8-.1	FYB1
ALG13	2.21E-13	0.409248	0.17	0.256	6.21E-09	CD4-CD8-.1	ALG13
NEDD9	2.82E-13	0.898947	0.304	0.311	7.93E-09	CD4-CD8-.1	NEDD9
ARHGEF1	3.20E-13	0.372293	0.36	0.547	9.01E-09	CD4-CD8-.1	ARHGEF1
SNRNP200	9.65E-13	0.3879	0.172	0.254	2.72E-08	CD4-CD8-.1	SNRNP200
SEMA4D	2.24E-12	0.914836	0.343	0.375	6.30E-08	CD4-CD8-.1	SEMA4D
GOLGA4	3.00E-12	0.843027	0.264	0.261	8.45E-08	CD4-CD8-.1	GOLGA4
GNA13	3.08E-12	0.459442	0.189	0.278	8.66E-08	CD4-CD8-.1	GNA13
CHD1	3.55E-12	0.874987	0.319	0.339	9.98E-08	CD4-CD8-.1	CHD1
DDX39B	5.48E-12	0.38745	0.205	0.3	1.54E-07	CD4-CD8-.1	DDX39B
INTS6	6.89E-12	0.505297	0.204	0.3	1.94E-07	CD4-CD8-.1	INTS6
ELL2	1.04E-11	0.813891	0.38	0.423	2.91E-07	CD4-CD8-.1	ELL2
PNISR	1.41E-11	0.418571	0.238	0.348	3.97E-07	CD4-CD8-.1	PNISR
BPTF	1.45E-11	0.848399	0.352	0.392	4.09E-07	CD4-CD8-.1	BPTF
USP47	1.75E-11	0.84241	0.25	0.245	4.91E-07	CD4-CD8-.1	USP47
ACAP1	2.00E-11	0.443834	0.302	0.449	5.64E-07	CD4-CD8-.1	ACAP1
TNFAIP8	2.17E-11	1.081127	0.316	0.344	6.09E-07	CD4-CD8-.1	TNFAIP8
RBMS1	2.76E-11	1.122415	0.342	0.385	7.77E-07	CD4-CD8-.1	RBMS1
CYTOR1	2.90E-11	0.432089	0.218	0.312	8.15E-07	CD4-CD8-.1	CYTOR
NFATC2	3.61E-11	0.877313	0.341	0.376	1.02E-06	CD4-CD8-.1	NFATC2
TAOK3	3.71E-11	0.899032	0.28	0.285	1.04E-06	CD4-CD8-.1	TAOK3
IFRD1	4.58E-11	0.502195	0.26	0.38	1.29E-06	CD4-CD8-.1	IFRD1
USP3	5.20E-11	0.868202	0.279	0.287	1.46E-06	CD4-CD8-.1	USP3
TACC1	6.00E-11	0.417806	0.188	0.273	1.69E-06	CD4-CD8-.1	TACC1
GCC2	6.29E-11	0.398187	0.203	0.292	1.77E-06	CD4-CD8-.1	GCC2
RBM25	6.37E-11	0.413837	0.287	0.424	1.79E-06	CD4-CD8-.1	RBM25
CSNK1A1	7.04E-11	0.464517	0.234	0.343	1.98E-06	CD4-CD8-.1	CSNK1A1
MYO9B	1.42E-10	0.853682	0.267	0.271	3.99E-06	CD4-CD8-.1	MYO9B
FOSL2	2.16E-10	0.400704	0.227	0.324	6.06E-06	CD4-CD8-.1	FOSL2
NR4A3	2.38E-10	0.40318	0.228	0.324	6.69E-06	CD4-CD8-.1	NR4A3
HSPH11	6.35E-10	0.553179	0.264	0.37	1.79E-05	CD4-CD8-.1	HSPH1
ZNF331	7.44E-10	0.672975	0.526	0.634	2.09E-05	CD4-CD8-.1	ZNF331
LNPEP	1.32E-09	0.421414	0.197	0.279	3.71E-05	CD4-CD8-.1	LNPEP
SMARCA2	1.42E-09	0.849665	0.339	0.376	3.99E-05	CD4-CD8-.1	SMARCA2
DDX241	1.55E-09	0.301539	0.437	0.642	4.37E-05	CD4-CD8-.1	DDX24
NASP	1.85E-09	0.454269	0.209	0.301	5.19E-05	CD4-CD8-.1	NASP
SCML4	2.17E-09	0.843447	0.355	0.4	6.09E-05	CD4-CD8-.1	SCML4
GPATCH8	3.34E-09	0.884486	0.262	0.267	9.39E-05	CD4-CD8-.1	GPATCH8
PTK2B	3.90E-09	0.844764	0.257	0.261	0.00011	CD4-CD8-.1	PTK2B
ANKHD1	4.57E-09	0.826764	0.314	0.345	0.000129	CD4-CD8-.1	ANKHD1
ADD3	4.93E-09	0.476575	0.189	0.268	0.000139	CD4-CD8-.1	ADD3
SLTM	5.06E-09	0.475232	0.238	0.343	0.000143	CD4-CD8-.1	SLTM
HECA	5.36E-09	0.840584	0.271	0.282	0.000151	CD4-CD8-.1	HECA
NFE2L2	1.18E-08	0.477763	0.219	0.31	0.000333	CD4-CD8-.1	NFE2L2
PIAS1	1.50E-08	0.846758	0.274	0.287	0.000423	CD4-CD8-.1	PIAS1

MAPK1	1.59E-08	0.438	0.233	0.33	0.000447	CD4-CD8-.1	MAPK1
CYFIP2	2.21E-08	0.439511	0.243	0.343	0.000621	CD4-CD8-.1	CYFIP2
WDR33	2.29E-08	0.49536	0.184	0.261	0.000646	CD4-CD8-.1	WDR33
XRN1	3.13E-08	0.767652	0.256	0.26	0.000881	CD4-CD8-.1	XRN1
SMAP2	3.45E-08	0.646111	0.484	0.616	0.00097	CD4-CD8-.1	SMAP2
RSF1	3.52E-08	0.459298	0.222	0.313	0.000991	CD4-CD8-.1	RSF1
AKAP9	3.78E-08	0.826607	0.284	0.301	0.001064	CD4-CD8-.1	AKAP9
PPP1R10	5.44E-08	0.491416	0.207	0.291	0.001531	CD4-CD8-.1	PPP1R10
BCLAF1	5.53E-08	0.482026	0.278	0.399	0.001556	CD4-CD8-.1	BCLAF1
SYNRG1	7.61E-08	0.499198	0.214	0.301	0.002142	CD4-CD8-.1	SYNRG
RUNX31	9.16E-08	0.620007	0.472	0.587	0.002578	CD4-CD8-.1	RUNX3
STAG2	1.36E-07	0.84086	0.25	0.257	0.00384	CD4-CD8-.1	STAG2
FNBP4	1.63E-07	0.444016	0.234	0.326	0.004596	CD4-CD8-.1	FNBP4
TAF15	2.13E-07	0.489768	0.222	0.31	0.005985	CD4-CD8-.1	TAF15
MALT1	2.46E-07	0.871201	0.248	0.255	0.006925	CD4-CD8-.1	MALT1
TCF25	2.56E-07	0.782855	0.373	0.44	0.007202	CD4-CD8-.1	TCF25
FAM107B	3.00E-07	0.633234	0.283	0.405	0.008433	CD4-CD8-.1	FAM107B
SPTAN1	3.30E-07	0.516489	0.298	0.427	0.009284	CD4-CD8-.1	SPTAN1
TRA2B	3.41E-07	0.431105	0.301	0.427	0.009602	CD4-CD8-.1	TRA2B
PYHIN1	3.97E-07	0.842556	0.272	0.283	0.011157	CD4-CD8-.1	PYHIN1
ACTN4	5.36E-07	0.444592	0.228	0.314	0.015079	CD4-CD8-.1	ACTN4
GLCCI1	6.70E-07	0.910578	0.292	0.321	0.01886	CD4-CD8-.1	GLCCI1
CCNY	8.35E-07	0.894896	0.248	0.261	0.023495	CD4-CD8-.1	CCNY
DNAJB6	8.45E-07	0.829292	0.354	0.417	0.02377	CD4-CD8-.1	DNAJB6
YTHDC1	1.17E-06	0.460114	0.221	0.304	0.032961	CD4-CD8-.1	YTHDC1
TRAPPC10	1.19E-06	0.81483	0.26	0.275	0.0336	CD4-CD8-.1	TRAPPC10
WAC	1.55E-06	0.831379	0.265	0.282	0.043539	CD4-CD8-.1	WAC
IVNS1ABP	2.07E-06	0.527316	0.236	0.326	0.058308	CD4-CD8-.1	IVNS1ABP
PAFAH1B1	2.24E-06	0.823531	0.297	0.332	0.062979	CD4-CD8-.1	PAFAH1B1
ITGA4	2.32E-06	0.820847	0.369	0.435	0.065403	CD4-CD8-.1	ITGA4
PRPF4B	3.79E-06	0.476832	0.201	0.272	0.106651	CD4-CD8-.1	PRPF4B
CD6	3.85E-06	0.828923	0.331	0.382	0.10843	CD4-CD8-.1	CD6
ASXL1	4.01E-06	0.803224	0.258	0.273	0.112822	CD4-CD8-.1	ASXL1
NFKBIZ1	5.53E-06	0.485454	0.298	0.409	0.155614	CD4-CD8-.1	NFKBIZ
TNFRSF1B	6.21E-06	0.516829	0.257	0.355	0.174643	CD4-CD8-.1	TNFRSF1B
SRRM2	7.36E-06	0.594519	0.475	0.611	0.207039	CD4-CD8-.1	SRRM2
FUS	8.02E-06	0.591704	0.489	0.642	0.225699	CD4-CD8-.1	FUS
PHF20	1.08E-05	0.851408	0.315	0.36	0.304746	CD4-CD8-.1	PHF20
PDE4B	1.28E-05	0.823754	0.383	0.462	0.360587	CD4-CD8-.1	PDE4B
ITGAL	2.11E-05	0.807812	0.25	0.265	0.593344	CD4-CD8-.1	ITGAL
PTPN1	2.26E-05	0.536881	0.213	0.288	0.63686	CD4-CD8-.1	PTPN1
MYO1F	2.67E-05	0.803603	0.244	0.258	0.75269	CD4-CD8-.1	MYO1F
APBB1IP	2.71E-05	0.482776	0.367	0.528	0.762844	CD4-CD8-.1	APBB1IP
ILF3	2.72E-05	0.506475	0.208	0.281	0.766133	CD4-CD8-.1	ILF3
NCK2	3.28E-05	0.861693	0.31	0.358	0.923871	CD4-CD8-.1	NCK2
IPCEF1	3.43E-05	0.557604	0.216	0.291	0.963755	CD4-CD8-.1	IPCEF1
RHOH	3.66E-05	0.818663	0.382	0.463	1	CD4-CD8-.1	RHOH

SLA	4.26E-05	0.797197	0.355	0.422	1	CD4-CD8-.1	SLA
IKZF3	4.50E-05	0.739252	0.287	0.314	1	CD4-CD8-.1	IKZF3
DHX36	7.96E-05	0.543914	0.228	0.308	1	CD4-CD8-.1	DHX36
AHNAK	0.000127	0.437282	0.357	0.494	1	CD4-CD8-.1	AHNAK
BBX	0.000128	0.837945	0.253	0.275	1	CD4-CD8-.1	BBX
ITSN2	0.000155	0.774959	0.285	0.319	1	CD4-CD8-.1	ITSN2
PRRC2B	0.000186	0.750605	0.264	0.288	1	CD4-CD8-.1	PRRC2B
SNX91	0.000192	0.964127	0.248	0.267	1	CD4-CD8-.1	SNX9
RNF149	0.000194	0.811481	0.34	0.408	1	CD4-CD8-.1	RNF149
NSD3	0.000257	0.587297	0.264	0.364	1	CD4-CD8-.1	NSD3
ATM	0.000274	0.75497	0.286	0.321	1	CD4-CD8-.1	ATM
N4BP2L2	0.000293	0.751919	0.333	0.393	1	CD4-CD8-.1	N4BP2L2
IL6ST	0.000345	0.500707	0.199	0.26	1	CD4-CD8-.1	IL6ST
DDX17	0.000414	0.726417	0.356	0.429	1	CD4-CD8-.1	DDX17
ZEB2	0.000428	0.974382	0.282	0.314	1	CD4-CD8-.1	ZEB2
ERN11	0.000455	0.845611	0.367	0.442	1	CD4-CD8-.1	ERN1
REL	0.000506	0.496174	0.356	0.488	1	CD4-CD8-.1	REL
TLE4	0.000556	0.790873	0.257	0.283	1	CD4-CD8-.1	TLE4
HERPUD21	0.000566	0.560656	0.255	0.346	1	CD4-CD8-.1	HERPUD2
DIAPH1	0.000592	0.75156	0.255	0.278	1	CD4-CD8-.1	DIAPH1
CASP8	0.00065	0.727696	0.251	0.271	1	CD4-CD8-.1	CASP8
RESF12	0.000689	0.461619	0.381	0.531	1	CD4-CD8-.1	RESF1
SMAD7	0.000816	0.532357	0.209	0.275	1	CD4-CD8-.1	SMAD7
PFKFB3	0.000838	0.865336	0.29	0.328	1	CD4-CD8-.1	PFKFB3
VPS13C	0.001037	0.708662	0.285	0.321	1	CD4-CD8-.1	VPS13C
ELOVL5	0.001117	0.827122	0.276	0.313	1	CD4-CD8-.1	ELOVL5
RAB8B	0.001175	0.807033	0.248	0.273	1	CD4-CD8-.1	RAB8B
STK17B	0.001191	0.5474	0.492	0.653	1	CD4-CD8-.1	STK17B
DYNC1H1	0.001232	0.756917	0.287	0.327	1	CD4-CD8-.1	DYNC1H1
VPS37B	0.001845	0.851402	0.32	0.376	1	CD4-CD8-.1	VPS37B
PRRC2C	0.002068	0.635577	0.422	0.551	1	CD4-CD8-.1	PRRC2C
JAK1	0.002483	0.599072	0.461	0.612	1	CD4-CD8-.1	JAK1
AFF4	0.002546	0.574403	0.242	0.32	1	CD4-CD8-.1	AFF4
SRSF4	0.003593	0.546971	0.229	0.299	1	CD4-CD8-.1	SRSF4
PDCD4	0.003926	0.490269	0.397	0.558	1	CD4-CD8-.1	PDCD4
FLI1	0.004579	0.849648	0.27	0.309	1	CD4-CD8-.1	FLI1
SP100	0.005445	0.622063	0.444	0.581	1	CD4-CD8-.1	SP100
MBP	0.00681	0.56122	0.291	0.391	1	CD4-CD8-.1	MBP
ELF1	0.007506	0.415759	0.449	0.636	1	CD4-CD8-.1	ELF1
APOE	0	4.158267	0.932	0.165	0	MG-Act	APOE
SPP1	0	3.958265	0.83	0.178	0	MG-Act	SPP1
C1QC	0	3.708666	0.909	0.121	0	MG-Act	C1QC
HLA-DRA	0	3.602555	0.926	0.209	0	MG-Act	HLA-DRA
C1QB	0	3.527303	0.898	0.131	0	MG-Act	C1QB
CCL3	0	3.499811	0.862	0.32	0	MG-Act	CCL3
C1QA	0	3.429294	0.878	0.103	0	MG-Act	C1QA
CCL3L1	0	3.31838	0.713	0.152	0	MG-Act	CCL3L1

CD74	0	3.176903	0.995	0.744	0	MG-Act	CD74
C3	0	3.124975	0.858	0.057	0	MG-Act	C3
CST3	0	3.029568	0.862	0.085	0	MG-Act	CST3
IL1B	0	2.987373	0.595	0.067	0	MG-Act	IL1B
PLXDC2	0	2.946637	0.851	0.082	0	MG-Act	PLXDC2
APOC1	0	2.90765	0.768	0.075	0	MG-Act	APOC1
CD83	0	2.88535	0.85	0.204	0	MG-Act	CD83
DOCK4	0	2.799468	0.825	0.083	0	MG-Act	DOCK4
SGK1	0	2.792923	0.792	0.16	0	MG-Act	SGK1
RHOB	0	2.709677	0.768	0.18	0	MG-Act	RHOB
HLA-DRB1	0	2.663689	0.896	0.258	0	MG-Act	HLA-DRB1
APOC2	0	2.567792	0.608	0.063	0	MG-Act	APOC2
SERPINE1	0	2.542739	0.421	0.032	0	MG-Act	SERPINE1
CXCL8	0	2.531664	0.502	0.051	0	MG-Act	CXCL8
IER3	0	2.513227	0.729	0.101	0	MG-Act	IER3
CCL4L21	0	2.439058	0.812	0.37	0	MG-Act	CCL4L2
HLA-DRB5	0	2.41016	0.751	0.145	0	MG-Act	HLA-DRB5
CTSB	0	2.385721	0.87	0.182	0	MG-Act	CTSB
TEX14	0	2.37828	0.752	0.27	0	MG-Act	TEX14
CEBPD	0	2.376213	0.818	0.258	0	MG-Act	CEBPD
HLA-DPA1	0	2.364124	0.884	0.335	0	MG-Act	HLA-DPA1
OLR1	0	2.355531	0.705	0.038	0	MG-Act	OLR1
SLC1A3	0	2.325889	0.727	0.03	0	MG-Act	SLC1A3
CSF1R	0	2.265269	0.725	0.024	0	MG-Act	CSF1R
TREM2	0	2.224649	0.69	0.026	0	MG-Act	TREM2
PDK4	0	2.224317	0.562	0.044	0	MG-Act	PDK4
CH25H	0	2.179239	0.469	0.058	0	MG-Act	CH25H
SAT1	0	2.172592	0.938	0.553	0	MG-Act	SAT1
NPC2	0	2.131118	0.774	0.146	0	MG-Act	NPC2
GSN	0	2.108908	0.717	0.045	0	MG-Act	GSN
AIF1	0	2.107464	0.739	0.075	0	MG-Act	AIF1
EGR1	0	2.085376	0.827	0.301	0	MG-Act	EGR1
MAFB	0	2.083761	0.588	0.033	0	MG-Act	MAFB
HLA-DQB1	0	2.071295	0.716	0.103	0	MG-Act	HLA-DQB1
MARCKS	0	2.060761	0.695	0.027	0	MG-Act	MARCKS
LRMDA	0	1.998677	0.67	0.037	0	MG-Act	LRMDA
RNASET2	0	1.979912	0.869	0.305	0	MG-Act	RNASET2
FRMD4A	0	1.957228	0.726	0.098	0	MG-Act	FRMD4A
IFI30	0	1.950926	0.614	0.043	0	MG-Act	IFI30
ARHGAP24	0	1.94669	0.648	0.031	0	MG-Act	ARHGAP24
RAB31	0	1.91852	0.657	0.031	0	MG-Act	RAB31
NR4A1	0	1.900469	0.722	0.228	0	MG-Act	NR4A1
FCER1G	0	1.891476	0.77	0.115	0	MG-Act	FCER1G
A2M	0	1.883973	0.727	0.097	0	MG-Act	A2M
TYROBP	0	1.883775	0.898	0.242	0	MG-Act	TYROBP
CD14	0	1.881314	0.524	0.014	0	MG-Act	CD14
PSAP	0	1.855727	0.877	0.374	0	MG-Act	PSAP

OTUD1	0	1.85101	0.475	0.04	0	MG-Act	OTUD1
EPB41L2	0	1.849002	0.675	0.042	0	MG-Act	EPB41L2
MEF2C	0	1.839179	0.63	0.025	0	MG-Act	MEF2C
SLC8A1	0	1.825174	0.622	0.026	0	MG-Act	SLC8A1
CD68	0	1.823144	0.646	0.031	0	MG-Act	CD68
FCGBP	0	1.809339	0.42	0.022	0	MG-Act	FCGBP
HLA-DQA1	0	1.807677	0.664	0.086	0	MG-Act	HLA-DQA1
HLA-DPB1	0	1.795381	0.829	0.33	0	MG-Act	HLA-DPB1
FTL	0	1.787042	0.993	0.865	0	MG-Act	FTL
ACSL1	0	1.767435	0.623	0.081	0	MG-Act	ACSL1
B3GNT5	0	1.760087	0.535	0.028	0	MG-Act	B3GNT5
FGD4	0	1.724486	0.595	0.035	0	MG-Act	FGD4
GPR34	0	1.718765	0.611	0.033	0	MG-Act	GPR34
PLAUR	0	1.697186	0.422	0.041	0	MG-Act	PLAUR
PADI2	0	1.690282	0.516	0.02	0	MG-Act	PADI2
HLA-DMA	0	1.671496	0.665	0.105	0	MG-Act	HLA-DMA
MS4A7	0	1.670749	0.583	0.021	0	MG-Act	MS4A7
CLEC7A	0	1.667717	0.541	0.02	0	MG-Act	CLEC7A
LPCAT2	0	1.655576	0.58	0.017	0	MG-Act	LPCAT2
IRAK3	0	1.652509	0.577	0.029	0	MG-Act	IRAK3
BCL2A1	0	1.63699	0.506	0.104	0	MG-Act	BCL2A1
FPR1	0	1.635136	0.544	0.013	0	MG-Act	FPR1
SLC11A1	0	1.6336	0.543	0.02	0	MG-Act	SLC11A1
LHFPL2	0	1.619557	0.571	0.02	0	MG-Act	LHFPL2
PLD4	0	1.617519	0.566	0.011	0	MG-Act	PLD4
LILRB4	0	1.600711	0.573	0.01	0	MG-Act	LILRB4
SPI1	0	1.596058	0.597	0.02	0	MG-Act	SPI1
HTRA1	0	1.592901	0.539	0.019	0	MG-Act	HTRA1
CSF2RA	0	1.590514	0.577	0.018	0	MG-Act	CSF2RA
MEF2A	0	1.587051	0.725	0.134	0	MG-Act	MEF2A
SLC2A5	0	1.581686	0.559	0.011	0	MG-Act	SLC2A5
GLUL	0	1.572988	0.694	0.144	0	MG-Act	GLUL
NLRP3	0	1.553709	0.467	0.047	0	MG-Act	NLRP3
SRGAP2	0	1.5535	0.633	0.077	0	MG-Act	SRGAP2
TBXAS1	0	1.550979	0.633	0.05	0	MG-Act	TBXAS1
SCIN	0	1.544451	0.523	0.016	0	MG-Act	SCIN
EGR2	0	1.543022	0.455	0.048	0	MG-Act	EGR2
CYFIP1	0	1.538868	0.577	0.026	0	MG-Act	CYFIP1
OGFRL1	0	1.538504	0.621	0.084	0	MG-Act	OGFRL1
CXCL16	0	1.5211	0.558	0.025	0	MG-Act	CXCL16
SERPINA1	0	1.520298	0.524	0.017	0	MG-Act	SERPINA1
GRN	0	1.515423	0.597	0.058	0	MG-Act	GRN
MSR1	0	1.514479	0.491	0.017	0	MG-Act	MSR1
C5AR1	0	1.509887	0.416	0.011	0	MG-Act	C5AR1
P2RY12	0	1.507863	0.519	0.017	0	MG-Act	P2RY12
FCGRT	0	1.505494	0.605	0.057	0	MG-Act	FCGRT
ST6GALNA(	0	1.501031	0.507	0.035	0	MG-Act	ST6GALNAC3

CD86	0	1.490693	0.522	0.013	0	MG-Act	CD86
TMEM176I	0	1.483859	0.479	0.012	0	MG-Act	TMEM176B
CPVL	0	1.482636	0.533	0.013	0	MG-Act	CPVL
VSIG4	0	1.47941	0.456	0.012	0	MG-Act	VSIG4
HSPA1A1	0	1.477982	0.789	0.31	0	MG-Act	HSPA1A
QKI	0	1.459778	0.726	0.176	0	MG-Act	QKI
BASP1	0	1.457486	0.464	0.027	0	MG-Act	BASP1
HMOX1	0	1.45595	0.497	0.019	0	MG-Act	HMOX1
MS4A6A	0	1.454442	0.484	0.019	0	MG-Act	MS4A6A
FCGR2A	0	1.448605	0.53	0.015	0	MG-Act	FCGR2A
C3AR1	0	1.435559	0.568	0.049	0	MG-Act	C3AR1
ETS2	0	1.435023	0.566	0.059	0	MG-Act	ETS2
TRIB1	0	1.422692	0.442	0.021	0	MG-Act	TRIB1
KCNQ3	0	1.417396	0.462	0.02	0	MG-Act	KCNQ3
CYBB	0	1.40581	0.519	0.015	0	MG-Act	CYBB
IFITM3	0	1.397949	0.587	0.189	0	MG-Act	IFITM3
CX3CR1	0	1.393681	0.533	0.072	0	MG-Act	CX3CR1
CD163	0	1.383834	0.287	0.009	0	MG-Act	CD163
KCTD12	0	1.383025	0.527	0.018	0	MG-Act	KCTD12
LINC00910	0	1.378803	0.576	0.207	0	MG-Act	LINC00910
CTSZ	0	1.353785	0.599	0.095	0	MG-Act	CTSZ
HSPA6	0	1.351707	0.253	0.062	0	MG-Act	HSPA6
IL18	0	1.341659	0.524	0.02	0	MG-Act	IL18
KLF4	0	1.327659	0.436	0.022	0	MG-Act	KLF4
AC245014.	0	1.325028	0.457	0.093	0	MG-Act	AC245014.3
SYNDIG1	0	1.314785	0.4	0.011	0	MG-Act	SYNDIG1
JDP2	0	1.302643	0.479	0.015	0	MG-Act	JDP2
FCGR1A	0	1.295003	0.446	0.009	0	MG-Act	FCGR1A
LNCAROD	0	1.2949	0.424	0.01	0	MG-Act	LNCAROD
ADAM28	0	1.294794	0.521	0.021	0	MG-Act	ADAM28
BHLHE41	0	1.286599	0.463	0.015	0	MG-Act	BHLHE41
SLCO2B1	0	1.279497	0.482	0.012	0	MG-Act	SLCO2B1
FMNL2	0	1.278466	0.446	0.019	0	MG-Act	FMNL2
LYN	0	1.276145	0.591	0.086	0	MG-Act	LYN
LDLRAD41	0	1.274082	0.798	0.313	0	MG-Act	LDLRAD4
PALD1	0	1.272954	0.484	0.013	0	MG-Act	PALD1
FCGR3A	0	1.269073	0.612	0.106	0	MG-Act	FCGR3A
RAC1	0	1.268654	0.809	0.37	0	MG-Act	RAC1
SOD2	0	1.257374	0.623	0.227	0	MG-Act	SOD2
AC007952.	0	1.25184	0.494	0.16	0	MG-Act	AC007952.4
NEAT11	0	1.242839	0.866	0.479	0	MG-Act	NEAT1
HAMP	0	1.238887	0.329	0.013	0	MG-Act	HAMP
LY86	0	1.2366	0.5	0.018	0	MG-Act	LY86
TUBA1B	0	1.236533	0.844	0.456	0	MG-Act	TUBA1B
ASAH1	0	1.221516	0.628	0.14	0	MG-Act	ASAH1
CD302	0	1.220665	0.498	0.035	0	MG-Act	CD302
ELL21	0	1.219369	0.771	0.376	0	MG-Act	ELL2

TLR2	0	1.196606	0.45	0.014	0	MG-Act	TLR2
S100A111	0	1.191122	0.848	0.417	0	MG-Act	S100A11
CCL41	0	1.188919	0.927	0.685	0	MG-Act	CCL4
AL078590.:	0	1.184541	0.382	0.014	0	MG-Act	AL078590.2
UBE2E2	0	1.179501	0.472	0.024	0	MG-Act	UBE2E2
EGR3	0	1.170428	0.386	0.022	0	MG-Act	EGR3
MAML3	0	1.168953	0.51	0.051	0	MG-Act	MAML3
HLA-DMB	0	1.159387	0.487	0.044	0	MG-Act	HLA-DMB
GRASP	0	1.154102	0.488	0.094	0	MG-Act	GRASP
CSF3R	0	1.154041	0.459	0.005	0	MG-Act	CSF3R
RHBDF2	0	1.151964	0.587	0.103	0	MG-Act	RHBDF2
RNF130	0	1.146925	0.585	0.093	0	MG-Act	RNF130
IFNGR2	0	1.141686	0.471	0.025	0	MG-Act	IFNGR2
RBM47	0	1.139981	0.413	0.012	0	MG-Act	RBM47
YWHAH	0	1.139973	0.614	0.152	0	MG-Act	YWHAH
RIN2	0	1.126299	0.406	0.011	0	MG-Act	RIN2
SPTLC2	0	1.125344	0.568	0.11	0	MG-Act	SPTLC2
IL1A	0	1.123781	0.251	0.01	0	MG-Act	IL1A
LTC4S	0	1.119175	0.454	0.033	0	MG-Act	LTC4S
FOS1	0	1.112567	0.978	0.803	0	MG-Act	FOS
ZFHX3	0	1.112018	0.463	0.026	0	MG-Act	ZFHX3
AZIN1-AS1	0	1.112013	0.36	0.029	0	MG-Act	AZIN1-AS1
RGS16	0	1.105487	0.388	0.084	0	MG-Act	RGS16
AL163541.:	0	1.102251	0.312	0.011	0	MG-Act	AL163541.1
IFNGR1	0	1.100962	0.687	0.228	0	MG-Act	IFNGR1
RASSF4	0	1.100782	0.442	0.026	0	MG-Act	RASSF4
LIMS1	0	1.100176	0.635	0.185	0	MG-Act	LIMS1
LINC01736	0	1.097886	0.4	0.011	0	MG-Act	LINC01736
TMEM107	0	1.093754	0.45	0.133	0	MG-Act	TMEM107
IL13RA1	0	1.091978	0.442	0.013	0	MG-Act	IL13RA1
C1orf162	0	1.08994	0.511	0.079	0	MG-Act	C1orf162
SRGAP2B	0	1.081609	0.427	0.035	0	MG-Act	SRGAP2B
CD81	0	1.078394	0.885	0.565	0	MG-Act	CD81
NAV3	0	1.078185	0.339	0.009	0	MG-Act	NAV3
RAB20	0	1.07333	0.362	0.014	0	MG-Act	RAB20
GPR1831	0	1.072292	0.607	0.224	0	MG-Act	GPR183
AC084871.	0	1.069289	0.272	0.011	0	MG-Act	AC084871.1
ARHGAP22	0	1.067693	0.397	0.009	0	MG-Act	ARHGAP22
LGALS9	0	1.065213	0.537	0.1	0	MG-Act	LGALS9
CTSH	0	1.064426	0.48	0.044	0	MG-Act	CTSH
DLEU1	0	1.062944	0.52	0.102	0	MG-Act	DLEU1
CKB	0	1.062361	0.371	0.014	0	MG-Act	CKB
MYO1E	0	1.059021	0.337	0.022	0	MG-Act	MYO1E
MAP4K3	0	1.057354	0.479	0.065	0	MG-Act	MAP4K3
CDKN1A1	0	1.052195	0.593	0.211	0	MG-Act	CDKN1A
PLEK	0	1.046113	0.534	0.152	0	MG-Act	PLEK
RGS11	0	1.044201	0.887	0.563	0	MG-Act	RGS1

ZFP36L1	0	1.044162	0.873	0.549	0	MG-Act	ZFP36L1
DBI	0	1.041182	0.701	0.363	0	MG-Act	DBI
IGSF6	0	1.039689	0.404	0.012	0	MG-Act	IGSF6
PTGS2	0	1.03786	0.285	0.009	0	MG-Act	PTGS2
ST6GAL1	0	1.037658	0.609	0.179	0	MG-Act	ST6GAL1
ABL2	0	1.035206	0.355	0.048	0	MG-Act	ABL2
GNAQ	0	1.031002	0.574	0.119	0	MG-Act	GNAQ
FTH11	0	1.03061	0.995	0.914	0	MG-Act	FTH1
PHACTR1	0	1.030108	0.349	0.009	0	MG-Act	PHACTR1
IRAK2	0	1.026759	0.4	0.039	0	MG-Act	IRAK2
TMIGD3	0	1.025154	0.331	0.01	0	MG-Act	TMIGD3
PEAK1	0	1.023627	0.46	0.072	0	MG-Act	PEAK1
CEBPB	0	1.023123	0.675	0.291	0	MG-Act	CEBPB
SYK	0	1.017891	0.448	0.033	0	MG-Act	SYK
HAVCR2	0	1.0162	0.529	0.098	0	MG-Act	HAVCR2
ATF3	0	1.012596	0.518	0.157	0	MG-Act	ATF3
ALOX5	0	1.007746	0.393	0.011	0	MG-Act	ALOX5
NR4A31	0	1.001961	0.619	0.276	0	MG-Act	NR4A3
HIF1A1	0	0.998451	0.63	0.249	0	MG-Act	HIF1A
ABCC4	0	0.998045	0.42	0.046	0	MG-Act	ABCC4
ABCA1	0	0.996714	0.308	0.012	0	MG-Act	ABCA1
TMEM119	0	0.99619	0.359	0.008	0	MG-Act	TMEM119
KLF6	0	0.992033	0.916	0.687	0	MG-Act	KLF6
MERTK	0	0.988363	0.36	0.009	0	MG-Act	MERTK
CSGALNAC	0	0.980868	0.472	0.067	0	MG-Act	CSGALNACT1
SRGAP1	0	0.979493	0.371	0.015	0	MG-Act	SRGAP1
LST1	0	0.978845	0.455	0.04	0	MG-Act	LST1
GNB4	0	0.974794	0.393	0.014	0	MG-Act	GNB4
NFKBID	0	0.968498	0.408	0.057	0	MG-Act	NFKBID
OLFML3	0	0.964329	0.352	0.01	0	MG-Act	OLFML3
HSPA1B1	0	0.964312	0.767	0.356	0	MG-Act	HSPA1B
VASH1	0	0.961835	0.399	0.024	0	MG-Act	VASH1
CTTNBP2	0	0.957128	0.345	0.008	0	MG-Act	CTTNBP2
MFSD1	0	0.954815	0.498	0.081	0	MG-Act	MFSD1
C9orf72	0	0.954051	0.493	0.112	0	MG-Act	C9orf72
BMP2K	0	0.94993	0.415	0.032	0	MG-Act	BMP2K
FMN1	0	0.948848	0.386	0.034	0	MG-Act	FMN1
FSCN1	0	0.945559	0.352	0.024	0	MG-Act	FSCN1
ITM2B	0	0.943273	0.923	0.728	0	MG-Act	ITM2B
CAPG	0	0.942472	0.54	0.13	0	MG-Act	CAPG
BAG3	0	0.941611	0.371	0.1	0	MG-Act	BAG3
TM6SF1	0	0.940761	0.413	0.019	0	MG-Act	TM6SF1
LGMN	0	0.940519	0.344	0.013	0	MG-Act	LGMN
PPT1	0	0.938057	0.531	0.13	0	MG-Act	PPT1
SHTN1	0	0.93725	0.362	0.011	0	MG-Act	SHTN1
GNG7	0	0.937081	0.389	0.02	0	MG-Act	GNG7
3-Mar	0	0.929978	0.472	0.103	0	MG-Act	3-Mar

ALOX5AP	0	0.928388	0.749	0.343	0	MG-Act	ALOX5AP
IGSF21	0	0.927139	0.335	0.009	0	MG-Act	IGSF21
ALDH2	0	0.9187	0.374	0.008	0	MG-Act	ALDH2
TIMP2	0	0.915043	0.401	0.022	0	MG-Act	TIMP2
FGL2	0	0.912585	0.367	0.029	0	MG-Act	FGL2
ITGAV	0	0.911923	0.426	0.059	0	MG-Act	ITGAV
GAB2	0	0.909362	0.436	0.045	0	MG-Act	GAB2
MANBA	0	0.909281	0.502	0.105	0	MG-Act	MANBA
CREG1	0	0.908591	0.42	0.046	0	MG-Act	CREG1
SOCS6	0	0.90767	0.331	0.011	0	MG-Act	SOCS6
ADAP2	0	0.898451	0.37	0.008	0	MG-Act	ADAP2
ATP2B1-AS	0	0.89722	0.47	0.117	0	MG-Act	ATP2B1-AS1
HDAC9	0	0.896833	0.396	0.05	0	MG-Act	HDAC9
SORL1	0	0.894712	0.693	0.248	0	MG-Act	SORL1
OXR1	0	0.892493	0.439	0.101	0	MG-Act	OXR1
IL1RAP	0	0.891073	0.347	0.029	0	MG-Act	IL1RAP
GABARAP	0	0.889782	0.824	0.505	0	MG-Act	GABARAP
NAMPT	0	0.880519	0.531	0.206	0	MG-Act	NAMPT
LAT2	0	0.879873	0.433	0.05	0	MG-Act	LAT2
NHSL1	0	0.878945	0.296	0.014	0	MG-Act	NHSL1
RGS2	0	0.877179	0.585	0.245	0	MG-Act	RGS2
DSE	0	0.875291	0.42	0.08	0	MG-Act	DSE
PLA2G4A	0	0.874955	0.307	0.008	0	MG-Act	PLA2G4A
CTSS	0	0.874432	0.606	0.187	0	MG-Act	CTSS
PILRA	0	0.865848	0.365	0.014	0	MG-Act	PILRA
VSIR	0	0.863999	0.708	0.324	0	MG-Act	VSIR
BIN1	0	0.863707	0.641	0.276	0	MG-Act	BIN1
CNPY3	0	0.856755	0.637	0.225	0	MG-Act	CNPY3
SERPINB9	0	0.852715	0.645	0.277	0	MG-Act	SERPINB9
LAIR1	0	0.851656	0.489	0.105	0	MG-Act	LAIR1
RASGEF1B	0	0.849157	0.62	0.219	0	MG-Act	RASGEF1B
SH3RF3	0	0.84825	0.29	0.012	0	MG-Act	SH3RF3
RGS10	0	0.847737	0.738	0.358	0	MG-Act	RGS10
ITGAX	0	0.842625	0.353	0.03	0	MG-Act	ITGAX
ATP8B4	0	0.842602	0.398	0.039	0	MG-Act	ATP8B4
IER5	0	0.839558	0.626	0.308	0	MG-Act	IER5
ELMO11	0	0.837882	0.714	0.32	0	MG-Act	ELMO1
AP1B1	0	0.83728	0.488	0.097	0	MG-Act	AP1B1
STAB1	0	0.837193	0.284	0.004	0	MG-Act	STAB1
SLC25A37	0	0.836004	0.413	0.055	0	MG-Act	SLC25A37
TCF4	0	0.835333	0.341	0.013	0	MG-Act	TCF4
MS4A4A	0	0.831362	0.29	0.007	0	MG-Act	MS4A4A
SDCCAG8	0	0.828992	0.477	0.085	0	MG-Act	SDCCAG8
HLA-DQA2	0	0.827705	0.311	0.031	0	MG-Act	HLA-DQA2
RASAL2	0	0.827495	0.312	0.01	0	MG-Act	RASAL2
ATP6V0B	0	0.826342	0.641	0.245	0	MG-Act	ATP6V0B
BLNK	0	0.824899	0.316	0.006	0	MG-Act	BLNK

DSCAM	0	0.824846	0.292	0.012	0	MG-Act	DSCAM
EIF4E	0	0.823542	0.437	0.149	0	MG-Act	EIF4E
DAGLB	0	0.822998	0.369	0.033	0	MG-Act	DAGLB
NINJ1	0	0.819237	0.481	0.119	0	MG-Act	NINJ1
CFD	0	0.817905	0.31	0.012	0	MG-Act	CFD
LINC02798	0	0.812693	0.303	0.014	0	MG-Act	LINC02798
ENTPD1	0	0.806641	0.324	0.016	0	MG-Act	ENTPD1
KLF10	0	0.806105	0.428	0.135	0	MG-Act	KLF10
PELI1	0	0.804434	0.528	0.154	0	MG-Act	PELI1
SCPEP1	0	0.803712	0.363	0.034	0	MG-Act	SCPEP1
TNF	0	0.803058	0.569	0.239	0	MG-Act	TNF
RILPL2	0	0.802331	0.528	0.179	0	MG-Act	RILPL2
IRF8	0	0.801097	0.376	0.042	0	MG-Act	IRF8
AL136987.:	0	0.800299	0.281	0.02	0	MG-Act	AL136987.1
PICALM1	0	0.800292	0.659	0.24	0	MG-Act	PICALM
CEP170	0	0.798375	0.39	0.042	0	MG-Act	CEP170
ATP6V1B2	0	0.795248	0.418	0.083	0	MG-Act	ATP6V1B2
LRRK1	0	0.792882	0.294	0.004	0	MG-Act	LRRK1
ALCAM	0	0.789571	0.331	0.046	0	MG-Act	ALCAM
SSPN	0	0.789028	0.291	0.005	0	MG-Act	SSPN
SIGLEC10	0	0.788782	0.305	0.006	0	MG-Act	SIGLEC10
AL021155.!	0	0.787853	0.306	0.06	0	MG-Act	AL021155.5
ERCC1	0	0.785453	0.56	0.191	0	MG-Act	ERCC1
CTTNBP2NI	0	0.781586	0.305	0.008	0	MG-Act	CTTNBP2NL
SERPINF1	0	0.778916	0.283	0.008	0	MG-Act	SERPINF1
KLHL6	0	0.776761	0.416	0.089	0	MG-Act	KLHL6
TYMP	0	0.776397	0.407	0.097	0	MG-Act	TYMP
CD63	0	0.772046	0.639	0.256	0	MG-Act	CD63
USP53	0	0.771985	0.402	0.08	0	MG-Act	USP53
EPB41L3	0	0.769785	0.31	0.009	0	MG-Act	EPB41L3
MLXIPL	0	0.769266	0.283	0.003	0	MG-Act	MLXIPL
ABR	0	0.76911	0.489	0.127	0	MG-Act	ABR
TMEM176/	0	0.767375	0.281	0.006	0	MG-Act	TMEM176A
AXL	0	0.765983	0.314	0.009	0	MG-Act	AXL
AL691403.:	0	0.765703	0.404	0.091	0	MG-Act	AL691403.1
CEBPA	0	0.762022	0.308	0.015	0	MG-Act	CEBPA
SWAP70	0	0.761768	0.339	0.022	0	MG-Act	SWAP70
TMEM52B	0	0.761262	0.279	0.005	0	MG-Act	TMEM52B
TNFSF13	0	0.760949	0.298	0.008	0	MG-Act	TNFSF13
SLC31A2	0	0.760709	0.318	0.022	0	MG-Act	SLC31A2
SH3TC1	0	0.760264	0.366	0.036	0	MG-Act	SH3TC1
WDR74	0	0.759003	0.476	0.187	0	MG-Act	WDR74
P2RY13	0	0.758454	0.279	0.006	0	MG-Act	P2RY13
RNASE6	0	0.758314	0.31	0.012	0	MG-Act	RNASE6
ITPR2	0	0.757449	0.577	0.179	0	MG-Act	ITPR2
ADAM17	0	0.751198	0.423	0.088	0	MG-Act	ADAM17
FHIT	0	0.74967	0.392	0.06	0	MG-Act	FHIT

ICAM1	0	0.749567	0.304	0.057	0	MG-Act	ICAM1
KCNMA1	0	0.748449	0.26	0.007	0	MG-Act	KCNMA1
FNIP2	0	0.744718	0.34	0.038	0	MG-Act	FNIP2
DST	0	0.741233	0.296	0.011	0	MG-Act	DST
SKIL	0	0.740906	0.675	0.304	0	MG-Act	SKIL
RFX2	0	0.740272	0.272	0.026	0	MG-Act	RFX2
GK	0	0.732482	0.293	0.03	0	MG-Act	GK
DENND3	0	0.732395	0.371	0.051	0	MG-Act	DENND3
GAA	0	0.730493	0.354	0.033	0	MG-Act	GAA
RNF144B	0	0.727186	0.26	0.018	0	MG-Act	RNF144B
PDGFB	0	0.727022	0.291	0.013	0	MG-Act	PDGFB
MCF2L2	0	0.725702	0.334	0.043	0	MG-Act	MCF2L2
DUSP11	0	0.724298	0.971	0.826	0	MG-Act	DUSP1
RASGEF1C	0	0.723083	0.265	0.005	0	MG-Act	RASGEF1C
TSPO	0	0.722121	0.637	0.289	0	MG-Act	TSPO
CCDC88A	0	0.720473	0.351	0.038	0	MG-Act	CCDC88A
HPGDS	0	0.718475	0.262	0.006	0	MG-Act	HPGDS
ZSWIM61	0	0.715407	0.6	0.231	0	MG-Act	ZSWIM6
1-Mar	0	0.715021	0.26	0.006	0	MG-Act	1-Mar
TAL1	0	0.713529	0.262	0.004	0	MG-Act	TAL1
AC012447.	0	0.712781	0.359	0.095	0	MG-Act	AC012447.1
CTSL	0	0.711054	0.308	0.028	0	MG-Act	CTSL
CPM	0	0.708838	0.253	0.008	0	MG-Act	CPM
CHKA	0	0.706936	0.386	0.059	0	MG-Act	CHKA
HCK	0	0.704601	0.293	0.005	0	MG-Act	HCK
TNS3	0	0.700993	0.256	0.006	0	MG-Act	TNS3
SKAP2	0	0.698992	0.442	0.083	0	MG-Act	SKAP2
SRGAP2C	0	0.698469	0.373	0.048	0	MG-Act	SRGAP2C
LRP1	0	0.695714	0.281	0.005	0	MG-Act	LRP1
LINC01374	0	0.695419	0.251	0.005	0	MG-Act	LINC01374
AC020916.	0	0.692197	0.578	0.233	0	MG-Act	AC020916.1
KDM6B1	0	0.688392	0.555	0.234	0	MG-Act	KDM6B
ETV6	0	0.687339	0.519	0.152	0	MG-Act	ETV6
MAP3K8	0	0.687108	0.653	0.301	0	MG-Act	MAP3K8
AL390957.:	0	0.686439	0.322	0.053	0	MG-Act	AL390957.1
ARL5A	0	0.685699	0.514	0.164	0	MG-Act	ARL5A
MIR222HG	0	0.683462	0.264	0.025	0	MG-Act	MIR222HG
LMO2	0	0.682994	0.276	0.006	0	MG-Act	LMO2
UNC93B1	0	0.682435	0.35	0.046	0	MG-Act	UNC93B1
KLF7	0	0.681152	0.293	0.025	0	MG-Act	KLF7
SLC29A1	0	0.674116	0.275	0.009	0	MG-Act	SLC29A1
CDK2AP1	0	0.673617	0.348	0.043	0	MG-Act	CDK2AP1
NPL	0	0.672714	0.282	0.019	0	MG-Act	NPL
TOM1	0	0.670853	0.452	0.136	0	MG-Act	TOM1
KHDRBS3	0	0.667886	0.259	0.012	0	MG-Act	KHDRBS3
TGFBR1	0	0.667244	0.406	0.092	0	MG-Act	TGFBR1
RTN41	0	0.665268	0.607	0.249	0	MG-Act	RTN4

EBI3	0	0.655563	0.256	0.004	0	MG-Act	EBI3
EPS8	0	0.653609	0.346	0.047	0	MG-Act	EPS8
MNDA	0	0.651715	0.266	0.007	0	MG-Act	MNDA
MCF2L	0	0.649921	0.251	0.01	0	MG-Act	MCF2L
APLP2	0	0.649529	0.426	0.093	0	MG-Act	APLP2
FMNL3	0	0.648474	0.409	0.089	0	MG-Act	FMNL3
BRI3	0	0.647035	0.507	0.161	0	MG-Act	BRI3
PHC2	0	0.643956	0.407	0.084	0	MG-Act	PHC2
IRS2	0	0.639809	0.423	0.114	0	MG-Act	IRS2
SERPINB6	0	0.638975	0.373	0.067	0	MG-Act	SERPINB6
TBC1D12	0	0.637284	0.256	0.004	0	MG-Act	TBC1D12
GRINA	0	0.636396	0.402	0.086	0	MG-Act	GRINA
SYNGR2	0	0.634857	0.543	0.212	0	MG-Act	SYNGR2
ANXA5	0	0.634829	0.602	0.247	0	MG-Act	ANXA5
MIS18BP1	0	0.63261	0.488	0.168	0	MG-Act	MIS18BP1
ATP2C1	0	0.628661	0.376	0.086	0	MG-Act	ATP2C1
MGAT1	0	0.628499	0.496	0.174	0	MG-Act	MGAT1
METTL7A	0	0.623721	0.283	0.013	0	MG-Act	METTL7A
TSC22D2	0	0.620836	0.537	0.222	0	MG-Act	TSC22D2
TRIO	0	0.61988	0.274	0.02	0	MG-Act	TRIO
RAPH1	0	0.619567	0.267	0.015	0	MG-Act	RAPH1
SFMBT21	0	0.619151	0.632	0.264	0	MG-Act	SFMBT2
B4GALT1	0	0.614871	0.59	0.261	0	MG-Act	B4GALT1
GALNT2	0	0.612909	0.428	0.101	0	MG-Act	GALNT2
NUDT3	0	0.60916	0.434	0.103	0	MG-Act	NUDT3
LPAR6	0	0.60845	0.307	0.039	0	MG-Act	LPAR6
FCHSD2	0	0.605294	0.448	0.13	0	MG-Act	FCHSD2
GNA131	0	0.604349	0.564	0.232	0	MG-Act	GNA13
SAP30	0	0.603646	0.316	0.075	0	MG-Act	SAP30
CALHM6	0	0.600888	0.253	0.024	0	MG-Act	CALHM6
NAIP	0	0.600174	0.301	0.032	0	MG-Act	NAIP
IL6R	0	0.598558	0.291	0.032	0	MG-Act	IL6R
MGST2	0	0.597178	0.302	0.022	0	MG-Act	MGST2
CTNND1	0	0.595444	0.26	0.015	0	MG-Act	CTNND1
MAN2B1	0	0.594261	0.403	0.106	0	MG-Act	MAN2B1
YBX3	0	0.593836	0.352	0.076	0	MG-Act	YBX3
STX11	0	0.592078	0.329	0.071	0	MG-Act	STX11
RB1	0	0.586763	0.493	0.147	0	MG-Act	RB1
AF213884.:	0	0.584768	0.268	0.036	0	MG-Act	AF213884.3
MTHFD1L	0	0.584747	0.292	0.039	0	MG-Act	MTHFD1L
P4HA1	0	0.583109	0.335	0.083	0	MG-Act	P4HA1
SLC43A2	0	0.576903	0.261	0.014	0	MG-Act	SLC43A2
NRIP1	0	0.572981	0.371	0.084	0	MG-Act	NRIP1
TLR1	0	0.572809	0.255	0.021	0	MG-Act	TLR1
CD84	0	0.572575	0.383	0.087	0	MG-Act	CD84
LY96	0	0.570427	0.263	0.018	0	MG-Act	LY96
RUNX11	0	0.560112	0.591	0.246	0	MG-Act	RUNX1

NFIC	0	0.558078	0.363	0.069	0	MG-Act	NFIC
BNC2	0	0.556934	0.282	0.034	0	MG-Act	BNC2
DPYSL2	0	0.552271	0.263	0.021	0	MG-Act	DPYSL2
ARHGAP21	0	0.550964	0.274	0.031	0	MG-Act	ARHGAP21
WASF2	0	0.549952	0.617	0.269	0	MG-Act	WASF2
CMTM6	0	0.546251	0.502	0.177	0	MG-Act	CMTM6
SNX92	0	0.543575	0.556	0.23	0	MG-Act	SNX9
LAPTM4A	0	0.543351	0.51	0.193	0	MG-Act	LAPTM4A
ATF6	0	0.541921	0.394	0.105	0	MG-Act	ATF6
RCC2	0	0.539368	0.31	0.064	0	MG-Act	RCC2
BEST1	0	0.535851	0.322	0.089	0	MG-Act	BEST1
PTPRE	0	0.535214	0.458	0.16	0	MG-Act	PTPRE
PLSCR1	0	0.533716	0.456	0.142	0	MG-Act	PLSCR1
CHSY1	0	0.533198	0.305	0.07	0	MG-Act	CHSY1
BAZ2B	0	0.532554	0.358	0.079	0	MG-Act	BAZ2B
STK38L	0	0.528204	0.278	0.054	0	MG-Act	STK38L
MAN1A1	0	0.527206	0.434	0.146	0	MG-Act	MAN1A1
LPAR5	0	0.526345	0.268	0.03	0	MG-Act	LPAR5
PABPC4	0	0.522889	0.45	0.153	0	MG-Act	PABPC4
HEXA	0	0.521661	0.424	0.135	0	MG-Act	HEXA
PDE8A	0	0.520905	0.326	0.087	0	MG-Act	PDE8A
TSPAN3	0	0.517893	0.294	0.04	0	MG-Act	TSPAN3
PEA15	0	0.515136	0.276	0.042	0	MG-Act	PEA15
PPARD	0	0.513957	0.295	0.049	0	MG-Act	PPARD
AKR1B1	0	0.51327	0.456	0.152	0	MG-Act	AKR1B1
FILIP1L	0	0.51008	0.258	0.047	0	MG-Act	FILIP1L
FAM49A	0	0.505792	0.307	0.058	0	MG-Act	FAM49A
AKR1A1	0	0.504609	0.394	0.121	0	MG-Act	AKR1A1
RHEB	0	0.502763	0.476	0.182	0	MG-Act	RHEB
SCAMP2	0	0.502141	0.508	0.195	0	MG-Act	SCAMP2
SH2B3	0	0.50098	0.316	0.06	0	MG-Act	SH2B3
GLA	0	0.49941	0.275	0.062	0	MG-Act	GLA
CPEB4	0	0.493817	0.272	0.051	0	MG-Act	CPEB4
RAB1A	0	0.492291	0.529	0.206	0	MG-Act	RAB1A
DISC1	0	0.488196	0.391	0.107	0	MG-Act	DISC1
OTULINL	0	0.487305	0.409	0.112	0	MG-Act	OTULINL
FLOT1	0	0.484785	0.394	0.111	0	MG-Act	FLOT1
TNFSF13B	0	0.482838	0.262	0.039	0	MG-Act	TNFSF13B
CD41	0	0.481646	0.386	0.109	0	MG-Act	CD4
ATP6AP1	0	0.480816	0.437	0.152	0	MG-Act	ATP6AP1
JAZF1	0	0.480723	0.486	0.173	0	MG-Act	JAZF1
CHD9	0	0.47991	0.465	0.158	0	MG-Act	CHD9
TMEM14C	0	0.479441	0.423	0.146	0	MG-Act	TMEM14C
AC103591.	0	0.477694	0.253	0.053	0	MG-Act	AC103591.3
IL17RA	0	0.475644	0.384	0.115	0	MG-Act	IL17RA
SLC16A3	0	0.473369	0.304	0.076	0	MG-Act	SLC16A3
ENG	0	0.472364	0.266	0.037	0	MG-Act	ENG

ABHD12	0	0.471433	0.37	0.098	0	MG-Act	ABHD12
SUSD6	0	0.471111	0.429	0.132	0	MG-Act	SUSD6
COMT	0	0.470326	0.391	0.118	0	MG-Act	COMT
CNDP2	0	0.465848	0.367	0.108	0	MG-Act	CNDP2
SPECC1	0	0.464548	0.263	0.036	0	MG-Act	SPECC1
NCOR2	0	0.463961	0.419	0.132	0	MG-Act	NCOR2
ANKS1A	0	0.462905	0.295	0.056	0	MG-Act	ANKS1A
RASSF2	0	0.461272	0.275	0.058	0	MG-Act	RASSF2
PRKAG2	0	0.456072	0.328	0.086	0	MG-Act	PRKAG2
CHMP4B	0	0.454537	0.321	0.081	0	MG-Act	CHMP4B
AIG1	0	0.453116	0.258	0.03	0	MG-Act	AIG1
RIN3	0	0.452689	0.448	0.143	0	MG-Act	RIN3
STX7	0	0.452501	0.289	0.053	0	MG-Act	STX7
NAGK	0	0.443848	0.295	0.065	0	MG-Act	NAGK
PLEKHO1	0	0.443512	0.456	0.166	0	MG-Act	PLEKHO1
MKNK1	0	0.439787	0.299	0.07	0	MG-Act	MKNK1
THEMIS2	0	0.437662	0.319	0.078	0	MG-Act	THEMIS2
PRKCD	0	0.434968	0.259	0.042	0	MG-Act	PRKCD
C20orf27	0	0.433426	0.294	0.074	0	MG-Act	C20orf27
KLHL5	0	0.432023	0.288	0.05	0	MG-Act	KLHL5
HEXB	0	0.431725	0.35	0.1	0	MG-Act	HEXB
DIAPH2	0	0.431145	0.423	0.138	0	MG-Act	DIAPH2
PACSIN2	0	0.428828	0.348	0.087	0	MG-Act	PACSIN2
ZMIZ1	0	0.428759	0.286	0.067	0	MG-Act	ZMIZ1
ADAMTSL4	0	0.424524	0.363	0.105	0	MG-Act	ADAMTSL4-AS1
FEZ2	0	0.422892	0.285	0.061	0	MG-Act	FEZ2
MTSS1	0	0.422806	0.279	0.067	0	MG-Act	MTSS1
DIP2B	0	0.421244	0.329	0.09	0	MG-Act	DIP2B
ARHGAP12	0	0.418924	0.266	0.056	0	MG-Act	ARHGAP12
PTTG1IP	0	0.418751	0.361	0.106	0	MG-Act	PTTG1IP
BACH1	0	0.417841	0.355	0.096	0	MG-Act	BACH1
TNFRSF1A	0	0.417515	0.352	0.095	0	MG-Act	TNFRSF1A
TTC7A	0	0.41411	0.324	0.097	0	MG-Act	TTC7A
POU2F2	0	0.412473	0.288	0.06	0	MG-Act	POU2F2
ATP13A3	0	0.408538	0.287	0.079	0	MG-Act	ATP13A3
DNASE2	0	0.406932	0.278	0.072	0	MG-Act	DNASE2
NUMB	0	0.405402	0.364	0.103	0	MG-Act	NUMB
ADGRG1	0	0.392813	0.322	0.092	0	MG-Act	ADGRG1
CACUL1	0	0.389618	0.326	0.093	0	MG-Act	CACUL1
RNF13	0	0.388148	0.406	0.132	0	MG-Act	RNF13
CTNNA1	0	0.382171	0.256	0.048	0	MG-Act	CTNNA1
CPQ	0	0.381261	0.33	0.086	0	MG-Act	CPQ
LINC02256	0	0.380581	0.34	0.085	0	MG-Act	LINC02256
RHOQ	0	0.380393	0.272	0.06	0	MG-Act	RHOQ
GSTM2	0	0.379821	0.274	0.059	0	MG-Act	GSTM2
SLC9A9	0	0.376849	0.412	0.14	0	MG-Act	SLC9A9
CD300A	0	0.374965	0.347	0.102	0	MG-Act	CD300A

SPINT2	0	0.37297	0.262	0.051	0	MG-Act	SPINT2
LINC00963	0	0.370457	0.275	0.071	0	MG-Act	LINC00963
SNX29	0	0.36826	0.349	0.104	0	MG-Act	SNX29
PMEPA1	0	0.361186	0.36	0.112	0	MG-Act	PMEPA1
PARVB	0	0.353944	0.261	0.061	0	MG-Act	PARVB
SOAT1	0	0.3467	0.314	0.09	0	MG-Act	SOAT1
SBF2	0	0.345213	0.317	0.089	0	MG-Act	SBF2
NCF4	0	0.318672	0.272	0.067	0	MG-Act	NCF4
P2RX4	9.56E-307	0.330287	0.252	0.063	2.69E-302	MG-Act	P2RX4
AC253572.	8.40E-305	0.860862	0.284	0.083	2.36E-300	MG-Act	AC253572.2
ATG7	1.08E-304	0.364464	0.368	0.119	3.05E-300	MG-Act	ATG7
SDCBP	3.72E-301	0.578032	0.642	0.306	1.05E-296	MG-Act	SDCBP
PDXK	1.11E-298	0.392206	0.307	0.091	3.12E-294	MG-Act	PDXK
LAPTM5	5.13E-298	0.636636	0.926	0.709	1.44E-293	MG-Act	LAPTM5
CUX1	8.96E-298	0.34856	0.387	0.13	2.52E-293	MG-Act	CUX1
MFSD12	1.61E-297	0.30084	0.251	0.064	4.54E-293	MG-Act	MFSD12
MED13L1	3.06E-296	0.379002	0.487	0.189	8.61E-292	MG-Act	MED13L
CHD7	8.50E-292	0.303565	0.312	0.093	2.39E-287	MG-Act	CHD7
NME2	2.46E-291	0.667425	0.702	0.372	6.93E-287	MG-Act	NME2
ENSA	2.78E-290	0.669868	0.695	0.36	7.83E-286	MG-Act	ENSA
WSB1	8.52E-290	0.612722	0.713	0.382	2.40E-285	MG-Act	WSB1
PYCARD	6.41E-289	0.512057	0.489	0.197	1.80E-284	MG-Act	PYCARD
ARHGAP26	1.14E-288	0.531988	0.699	0.353	3.22E-284	MG-Act	ARHGAP26
FOXO3	3.23E-287	0.38268	0.424	0.154	9.08E-283	MG-Act	FOXO3
IL6ST1	1.57E-285	0.432032	0.532	0.22	4.41E-281	MG-Act	IL6ST
NCOA4	4.41E-285	0.42452	0.393	0.139	1.24E-280	MG-Act	NCOA4
ZNF267	6.93E-285	0.387008	0.323	0.101	1.95E-280	MG-Act	ZNF267
VMP1	1.04E-279	0.37196	0.469	0.182	2.93E-275	MG-Act	VMP1
NANS	1.58E-278	0.407202	0.316	0.1	4.45E-274	MG-Act	NANS
APPL2	2.44E-276	0.31759	0.27	0.077	6.87E-272	MG-Act	APPL2
WASHC4	7.37E-276	0.345543	0.328	0.105	2.07E-271	MG-Act	WASHC4
STX17-AS1	5.67E-275	0.545368	0.351	0.121	1.60E-270	MG-Act	STX17-AS1
APBB1IP1	1.06E-273	0.597397	0.79	0.476	2.97E-269	MG-Act	APBB1IP
DLEU2	1.57E-273	0.279037	0.405	0.146	4.41E-269	MG-Act	DLEU2
HSPB11	6.93E-273	1.020467	0.54	0.255	1.95E-268	MG-Act	HSPB1
LAMP2	3.89E-272	0.316019	0.261	0.073	1.09E-267	MG-Act	LAMP2
IPCEF11	5.94E-272	0.723576	0.538	0.252	1.67E-267	MG-Act	IPCEF1
REL1	1.02E-271	0.608988	0.762	0.438	2.88E-267	MG-Act	REL
PLD3	1.56E-267	0.353598	0.351	0.12	4.38E-263	MG-Act	PLD3
BCL6	2.01E-267	0.354258	0.319	0.102	5.66E-263	MG-Act	BCL6
H2AFY	8.74E-267	0.459291	0.525	0.225	2.46E-262	MG-Act	H2AFY
LIPA	9.32E-267	0.377562	0.34	0.114	2.62E-262	MG-Act	LIPA
LAP3	2.74E-266	0.463306	0.448	0.178	7.71E-262	MG-Act	LAP3
NFKBIA1	5.05E-266	0.738738	0.92	0.724	1.42E-261	MG-Act	NFKBIA
UBE2E1	7.72E-265	0.330373	0.333	0.11	2.17E-260	MG-Act	UBE2E1
BST2	2.79E-263	0.61961	0.64	0.324	7.84E-259	MG-Act	BST2
SSH1	7.84E-262	0.287599	0.251	0.07	2.21E-257	MG-Act	SSH1

FRMD4B	1.49E-261	0.316702	0.313	0.1	4.19E-257	MG-Act	FRMD4B
PLCB2	1.88E-261	0.350704	0.325	0.107	5.29E-257	MG-Act	PLCB2
CRTAP	1.43E-260	0.329584	0.319	0.103	4.02E-256	MG-Act	CRTAP
NFKB11	8.24E-258	0.816167	0.674	0.38	2.32E-253	MG-Act	NFKB1
DDAH2	1.13E-257	0.287762	0.256	0.073	3.18E-253	MG-Act	DDAH2
UBA7	1.24E-257	0.283306	0.278	0.084	3.49E-253	MG-Act	UBA7
ZEB21	2.22E-257	0.303659	0.612	0.274	6.26E-253	MG-Act	ZEB2
TMEM156	6.27E-257	0.291841	0.325	0.108	1.76E-252	MG-Act	TMEM156
SVBP	4.39E-256	0.30677	0.26	0.075	1.24E-251	MG-Act	SVBP
TPP1	8.24E-256	0.335611	0.337	0.115	2.32E-251	MG-Act	TPP1
SPATA13	6.87E-254	0.39002	0.471	0.193	1.93E-249	MG-Act	SPATA13
TAB2	1.49E-252	0.338151	0.454	0.18	4.19E-248	MG-Act	TAB2
NFIL3	5.30E-252	0.405634	0.343	0.121	1.49E-247	MG-Act	NFIL3
MAT2A	1.19E-251	0.509563	0.554	0.255	3.36E-247	MG-Act	MAT2A
OAS1	1.33E-250	0.395891	0.285	0.09	3.76E-246	MG-Act	OAS1
TCF121	2.72E-248	0.356664	0.524	0.229	7.66E-244	MG-Act	TCF12
GRB21	2.46E-247	0.442117	0.594	0.28	6.91E-243	MG-Act	GRB2
AL138963.4	4.41E-246	0.72212	0.379	0.152	1.24E-241	MG-Act	AL138963.4
GBP2	5.99E-246	0.604901	0.478	0.212	1.68E-241	MG-Act	GBP2
CROCC	1.13E-245	0.580274	0.412	0.164	3.17E-241	MG-Act	CROCC
AFF41	1.49E-244	0.440392	0.585	0.279	4.20E-240	MG-Act	AFF4
NSMCE2	1.79E-243	0.286587	0.382	0.142	5.03E-239	MG-Act	NSMCE2
PLK3	1.09E-242	0.397223	0.48	0.205	3.06E-238	MG-Act	PLK3
GADD45B	6.41E-242	0.646861	0.841	0.567	1.80E-237	MG-Act	GADD45B
PPP1R15A1	1.45E-241	0.697316	0.826	0.553	4.08E-237	MG-Act	PPP1R15A
GNG10	3.36E-238	0.348866	0.299	0.099	9.46E-234	MG-Act	GNG10
NAA20	1.89E-237	0.276419	0.285	0.092	5.32E-233	MG-Act	NAA20
SNAP23	7.16E-237	0.350173	0.331	0.116	2.01E-232	MG-Act	SNAP23
Z93241.1	6.64E-236	0.656598	0.373	0.147	1.87E-231	MG-Act	Z93241.1
DUSP6	1.73E-234	0.414216	0.291	0.097	4.86E-230	MG-Act	DUSP6
WDFY2	6.25E-233	0.263628	0.295	0.097	1.76E-228	MG-Act	WDFY2
GLRX	8.59E-230	0.413813	0.406	0.164	2.42E-225	MG-Act	GLRX
HCLS1	1.19E-228	0.556447	0.809	0.513	3.34E-224	MG-Act	HCLS1
HSBP1	2.72E-228	0.288692	0.274	0.088	7.67E-224	MG-Act	HSBP1
SNX2	5.88E-228	0.301426	0.383	0.147	1.65E-223	MG-Act	SNX2
UBE2W	6.04E-226	0.25678	0.269	0.086	1.70E-221	MG-Act	UBE2W
SLC1A5	7.87E-226	0.315324	0.341	0.125	2.21E-221	MG-Act	SLC1A5
ZNF106	1.89E-225	0.279884	0.331	0.118	5.30E-221	MG-Act	ZNF106
RANBP9	4.01E-225	0.359981	0.432	0.179	1.13E-220	MG-Act	RANBP9
SPIDR1	2.20E-224	0.262954	0.492	0.213	6.18E-220	MG-Act	SPIDR
ARAP1	1.01E-223	0.258645	0.253	0.078	2.84E-219	MG-Act	ARAP1
TBC1D14	5.44E-223	0.278551	0.31	0.108	1.53E-218	MG-Act	TBC1D14
JAK2	2.08E-222	0.276035	0.307	0.107	5.86E-218	MG-Act	JAK2
TET2	2.94E-222	0.321878	0.317	0.112	8.27E-218	MG-Act	TET2
TFRC	1.46E-221	0.523453	0.302	0.108	4.10E-217	MG-Act	TFRC
CIAO2A	3.23E-220	0.342864	0.362	0.139	9.09E-216	MG-Act	CIAO2A
IER2	7.02E-219	0.641254	0.892	0.672	1.97E-214	MG-Act	IER2

CYBA	9.96E-219	0.492764	0.921	0.739	2.80E-214	MG-Act	CYBA
ARRB2	1.26E-217	0.416844	0.608	0.302	3.56E-213	MG-Act	ARRB2
IFI44L	2.76E-216	0.480316	0.37	0.148	7.76E-212	MG-Act	IFI44L
SEC14L1	1.04E-214	0.334377	0.35	0.134	2.92E-210	MG-Act	SEC14L1
IFI61	1.40E-213	0.909447	0.485	0.249	3.93E-209	MG-Act	IFI6
ATP6V1F	3.24E-208	0.422276	0.551	0.267	9.13E-204	MG-Act	ATP6V1F
ERGIC1	7.76E-208	0.269548	0.35	0.133	2.18E-203	MG-Act	ERGIC1
DRAM2	2.21E-205	0.256751	0.259	0.086	6.23E-201	MG-Act	DRAM2
SPAG9	2.56E-201	0.256083	0.382	0.155	7.21E-197	MG-Act	SPAG9
AC022217.	3.80E-201	0.38723	0.449	0.199	1.07E-196	MG-Act	AC022217.3
BNIP3L	1.82E-197	0.286053	0.365	0.146	5.13E-193	MG-Act	BNIP3L
NEU1	5.55E-197	0.349132	0.415	0.181	1.56E-192	MG-Act	NEU1
ARL8B	7.55E-197	0.307442	0.337	0.131	2.12E-192	MG-Act	ARL8B
SEC11A	4.24E-196	0.385878	0.536	0.261	1.19E-191	MG-Act	SEC11A
MTHFD2	1.43E-194	0.347214	0.4	0.17	4.02E-190	MG-Act	MTHFD2
YWHAE1	1.46E-194	0.285976	0.475	0.214	4.10E-190	MG-Act	YWHAE
ADRB2	3.36E-194	0.253104	0.343	0.133	9.46E-190	MG-Act	ADRB2
ABI3	5.31E-193	0.317362	0.455	0.201	1.49E-188	MG-Act	ABI3
STX4	7.92E-192	0.33719	0.291	0.108	2.23E-187	MG-Act	STX4
PFKFB31	1.46E-191	0.329894	0.585	0.293	4.11E-187	MG-Act	PFKFB3
CHCHD101	1.67E-189	0.349813	0.504	0.238	4.70E-185	MG-Act	CHCHD10
CAPZA2	2.98E-189	0.283259	0.438	0.194	8.38E-185	MG-Act	CAPZA2
GNG5	1.25E-188	0.404221	0.664	0.364	3.50E-184	MG-Act	GNG5
NEDD91	1.53E-188	0.437895	0.548	0.282	4.31E-184	MG-Act	NEDD9
RAB7A1	7.15E-188	0.436294	0.631	0.342	2.01E-183	MG-Act	RAB7A
PRDX1	8.76E-188	0.411591	0.575	0.294	2.46E-183	MG-Act	PRDX1
PTPN18	1.22E-187	0.275676	0.339	0.134	3.43E-183	MG-Act	PTPN18
ATP2A2	9.44E-187	0.2515	0.314	0.12	2.66E-182	MG-Act	ATP2A2
MDM2	1.77E-185	0.31245	0.276	0.101	4.99E-181	MG-Act	MDM2
ETF1	5.26E-184	0.308325	0.424	0.189	1.48E-179	MG-Act	ETF1
KLF21	2.53E-183	0.42423	0.757	0.437	7.13E-179	MG-Act	KLF2
NFKBIZ2	3.34E-183	0.447082	0.647	0.366	9.39E-179	MG-Act	NFKBIZ
CD58	4.29E-183	0.269983	0.391	0.167	1.21E-178	MG-Act	CD58
MPC2	4.12E-182	0.357275	0.429	0.194	1.16E-177	MG-Act	MPC2
CLTC	5.21E-182	0.270514	0.361	0.148	1.47E-177	MG-Act	CLTC
CTNNB1	7.99E-182	0.33383	0.486	0.229	2.25E-177	MG-Act	CTNNB1
OS9	4.52E-181	0.318113	0.44	0.199	1.27E-176	MG-Act	OS9
BTG2	7.66E-181	0.556246	0.748	0.478	2.16E-176	MG-Act	BTG2
ATP6V0D1	8.78E-181	0.252765	0.341	0.137	2.47E-176	MG-Act	ATP6V0D1
CTSD	3.21E-178	0.471305	0.714	0.429	9.03E-174	MG-Act	CTSD
NAF1	3.29E-178	0.294334	0.278	0.104	9.26E-174	MG-Act	NAF1
RGS19	1.33E-177	0.325643	0.43	0.194	3.74E-173	MG-Act	RGS19
CLTA	1.92E-176	0.31691	0.474	0.222	5.41E-172	MG-Act	CLTA
1-Jun	1.92E-172	0.492357	0.941	0.782	5.42E-168	MG-Act	JUN
TM9SF2	2.71E-172	0.284745	0.409	0.182	7.61E-168	MG-Act	TM9SF2
HSP90AA1:	3.21E-172	0.556391	0.936	0.784	9.04E-168	MG-Act	HSP90AA1
TMEM219	7.91E-171	0.326541	0.483	0.233	2.23E-166	MG-Act	TMEM219

GPX4	4.42E-170	0.404545	0.662	0.379	1.24E-165	MG-Act	GPX4
SRGN	2.95E-169	0.453407	0.958	0.857	8.30E-165	MG-Act	SRGN
FOSB	6.41E-169	0.386287	0.821	0.558	1.80E-164	MG-Act	FOSB
TMBIM4	1.74E-168	0.33009	0.564	0.29	4.90E-164	MG-Act	TMBIM4
EIF4A1	1.75E-168	0.453213	0.839	0.581	4.92E-164	MG-Act	EIF4A1
DHRS7	8.26E-168	0.356264	0.604	0.322	2.33E-163	MG-Act	DHRS7
MCL1	8.82E-167	0.450129	0.844	0.612	2.48E-162	MG-Act	MCL1
PRDX3	1.91E-166	0.273927	0.298	0.118	5.38E-162	MG-Act	PRDX3
SIPA1L11	6.43E-164	0.402966	0.546	0.293	1.81E-159	MG-Act	SIPA1L1
ATP6V0C	6.83E-164	0.435657	0.744	0.468	1.92E-159	MG-Act	ATP6V0C
SELPLG	2.47E-163	0.438145	0.622	0.358	6.94E-159	MG-Act	SELPLG
RAPGEF11	7.89E-163	0.259646	0.546	0.281	2.22E-158	MG-Act	RAPGEF1
PLIN2	6.50E-160	0.48852	0.413	0.199	1.83E-155	MG-Act	PLIN2
HSPH12	9.55E-159	0.452235	0.588	0.33	2.69E-154	MG-Act	HSPH1
SSH21	2.54E-158	0.269095	0.672	0.381	7.14E-154	MG-Act	SSH2
SNX3	2.89E-158	0.313187	0.555	0.285	8.13E-154	MG-Act	SNX3
SQSTM1	2.74E-156	0.456869	0.663	0.396	7.72E-152	MG-Act	SQSTM1
RABGEF1	3.70E-156	0.290826	0.409	0.192	1.04E-151	MG-Act	RABGEF1
MTRNR2L1	6.46E-155	0.672725	0.711	0.519	1.82E-150	MG-Act	MTRNR2L12
APMAP	7.89E-154	0.266225	0.545	0.282	2.22E-149	MG-Act	APMAP
LAMTOR1	3.81E-153	0.289406	0.44	0.211	1.07E-148	MG-Act	LAMTOR1
HERPUD11	4.27E-153	0.506772	0.741	0.488	1.20E-148	MG-Act	HERPUD1
HSP90AB1:	6.23E-149	0.395323	0.906	0.706	1.75E-144	MG-Act	HSP90AB1
ATP6V0E1	6.77E-146	0.368842	0.669	0.397	1.91E-141	MG-Act	ATP6V0E1
TLN1	5.32E-143	0.276072	0.595	0.324	1.50E-138	MG-Act	TLN1
DDX3Y	7.26E-139	0.312695	0.395	0.194	2.04E-134	MG-Act	DDX3Y
SH3BGRL	3.37E-138	0.326941	0.621	0.356	9.49E-134	MG-Act	SH3BGRL
CKS2	2.00E-137	0.346531	0.323	0.148	5.62E-133	MG-Act	CKS2
GDI2	7.13E-136	0.281056	0.534	0.283	2.00E-131	MG-Act	GDI2
CANX	4.00E-132	0.27388	0.521	0.277	1.13E-127	MG-Act	CANX
NR4A21	7.24E-129	0.395681	0.856	0.638	2.04E-124	MG-Act	NR4A2
RHOG	2.51E-127	0.268088	0.571	0.315	7.06E-123	MG-Act	RHOG
TUBB	3.19E-126	0.332661	0.534	0.293	8.98E-122	MG-Act	TUBB
VAMP8	1.23E-122	0.31031	0.618	0.358	3.47E-118	MG-Act	VAMP8
COTL11	4.13E-120	0.263288	0.755	0.47	1.16E-115	MG-Act	COTL1
ZFAND2A	3.11E-114	0.286795	0.266	0.12	8.75E-110	MG-Act	ZFAND2A
ACTB	3.24E-114	0.289091	0.996	0.936	9.13E-110	MG-Act	ACTB
HSPD11	3.87E-111	0.286371	0.592	0.355	1.09E-106	MG-Act	HSPD1
TRA2B1	5.74E-106	0.266714	0.632	0.387	1.61E-101	MG-Act	TRA2B
PNP1	4.29E-104	0.266893	0.353	0.183	1.21E-99	MG-Act	PNP
ARL6IP1	2.63E-103	0.267569	0.638	0.391	7.41E-99	MG-Act	ARL6IP1
AKAP131	5.13E-101	0.376269	0.797	0.6	1.44E-96	MG-Act	AKAP13
RNASEK	2.43E-98	0.313364	0.751	0.513	6.85E-94	MG-Act	RNASEK
CAPZB	4.00E-94	0.281968	0.808	0.574	1.13E-89	MG-Act	CAPZB
ZFAND5	6.81E-90	0.3559	0.565	0.354	1.92E-85	MG-Act	ZFAND5
HSP90B1	1.55E-88	0.280895	0.706	0.462	4.35E-84	MG-Act	HSP90B1
MT-ATP61	1.71E-81	0.282702	0.937	0.801	4.80E-77	MG-Act	MT-ATP6

ARPC3	1.13E-78	0.26597	0.826	0.616	3.18E-74	MG-Act	ARPC3
LCP2	1.51E-78	0.338033	0.724	0.51	4.26E-74	MG-Act	LCP2
OAZ11	4.59E-74	0.273523	0.857	0.671	1.29E-69	MG-Act	OAZ1
RHOA	4.65E-71	0.253616	0.834	0.618	1.31E-66	MG-Act	RHOA
MT2A	1.15E-13	0.496049	0.602	0.476	3.24E-09	MG-Act	MT2A
LEF1	0	2.079348	0.619	0.078	0	CD8.Mem	LEF1
KLRC2	0	1.773533	0.351	0.072	0	CD8.Mem	KLRC2
TCF71	0	1.541775	0.616	0.173	0	CD8.Mem	TCF7
CCR7	0	1.398208	0.332	0.052	0	CD8.Mem	CCR7
NELL2	0	0.946457	0.265	0.05	0	CD8.Mem	NELL2
TXK	0	0.915189	0.425	0.141	0	CD8.Mem	TXK
RPL322	0	0.680468	0.983	0.921	0	CD8.Mem	RPL32
KLF22	1.63E-306	1.291992	0.735	0.449	4.59E-302	CD8.Mem	KLF2
SELL	6.80E-291	1.382437	0.43	0.169	1.91E-286	CD8.Mem	SELL
RPL222	5.63E-278	0.750858	0.914	0.791	1.58E-273	CD8.Mem	RPL22
RPS132	7.10E-263	0.662089	0.97	0.887	2.00E-258	CD8.Mem	RPS13
RPL192	8.78E-263	0.558042	0.982	0.916	2.47E-258	CD8.Mem	RPL19
RPL342	7.08E-257	0.581976	0.976	0.91	1.99E-252	CD8.Mem	RPL34
RPL112	7.08E-248	0.55756	0.981	0.917	1.99E-243	CD8.Mem	RPL11
RPS122	2.23E-246	0.58412	0.984	0.93	6.28E-242	CD8.Mem	RPS12
RPS3A1	4.74E-240	0.667443	0.972	0.898	1.33E-235	CD8.Mem	RPS3A
RPL18A2	1.17E-237	0.535143	0.978	0.908	3.28E-233	CD8.Mem	RPL18A
RPL82	2.94E-232	0.560613	0.964	0.879	8.27E-228	CD8.Mem	RPL8
BACH21	2.29E-231	0.796374	0.481	0.211	6.46E-227	CD8.Mem	BACH2
RPS51	2.61E-231	0.709412	0.927	0.831	7.35E-227	CD8.Mem	RPS5
RPL131	5.40E-230	0.508773	0.987	0.938	1.52E-225	CD8.Mem	RPL13
PABPC11	3.14E-222	0.662	0.911	0.797	8.83E-218	CD8.Mem	PABPC1
RPS232	9.39E-220	0.524887	0.98	0.911	2.64E-215	CD8.Mem	RPS23
EEF1A11	2.44E-219	0.577199	0.988	0.947	6.86E-215	CD8.Mem	EEF1A1
LDLRAP1	5.41E-212	0.714673	0.319	0.112	1.52E-207	CD8.Mem	LDLRAP1
RPL302	2.57E-208	0.457805	0.984	0.924	7.23E-204	CD8.Mem	RPL30
LTB1	1.31E-207	0.864807	0.608	0.334	3.68E-203	CD8.Mem	LTB
RPL292	3.33E-206	0.517069	0.97	0.891	9.37E-202	CD8.Mem	RPL29
RPS61	1.06E-200	0.608632	0.954	0.882	2.99E-196	CD8.Mem	RPS6
RPL51	2.11E-200	0.646066	0.937	0.842	5.93E-196	CD8.Mem	RPL5
RPS27A2	9.30E-198	0.467162	0.981	0.918	2.62E-193	CD8.Mem	RPS27A
RPS213	5.23E-193	0.489473	0.96	0.878	1.47E-188	CD8.Mem	RPS21
IL7R1	9.02E-191	0.480292	0.821	0.468	2.54E-186	CD8.Mem	IL7R
RPS283	2.88E-187	0.454126	0.979	0.912	8.10E-183	CD8.Mem	RPS28
RPS81	1.39E-186	0.576146	0.974	0.914	3.92E-182	CD8.Mem	RPS8
RPL371	6.36E-184	0.489975	0.965	0.893	1.79E-179	CD8.Mem	RPL37
RPS141	4.34E-179	0.470716	0.974	0.905	1.22E-174	CD8.Mem	RPS14
CD7	6.25E-178	0.921833	0.735	0.503	1.76E-173	CD8.Mem	CD7
NUCB2	8.81E-177	0.795738	0.442	0.214	2.48E-172	CD8.Mem	NUCB2
RPL142	4.90E-164	0.465448	0.966	0.891	1.38E-159	CD8.Mem	RPL14
RPL392	1.33E-162	0.455597	0.968	0.905	3.73E-158	CD8.Mem	RPL39
TNFSF8	1.07E-159	0.63569	0.398	0.181	3.01E-155	CD8.Mem	TNFSF8

EEF1B21	2.07E-156	0.613268	0.876	0.764	5.81E-152	CD8.Mem	EEF1B2
RPL182	6.80E-155	0.44575	0.964	0.893	1.91E-150	CD8.Mem	RPL18
RPL102	2.13E-152	0.393403	0.985	0.942	5.98E-148	CD8.Mem	RPL10
NOSIP1	5.64E-152	0.910735	0.484	0.281	1.59E-147	CD8.Mem	NOSIP
RPS182	5.93E-149	0.44424	0.977	0.917	1.67E-144	CD8.Mem	RPS18
RPS4X1	8.70E-149	0.477736	0.97	0.891	2.45E-144	CD8.Mem	RPS4X
CD551	7.71E-148	0.761626	0.609	0.391	2.17E-143	CD8.Mem	CD55
TMEM123:	6.83E-147	0.683302	0.474	0.257	1.92E-142	CD8.Mem	TMEM123
RIPOR21	1.39E-145	0.651552	0.545	0.317	3.91E-141	CD8.Mem	RIPOR2
LDHB1	1.72E-137	0.720112	0.681	0.503	4.85E-133	CD8.Mem	LDHB
RPL35A2	1.90E-136	0.422302	0.956	0.879	5.35E-132	CD8.Mem	RPL35A
RACK11	3.72E-136	0.458309	0.932	0.84	1.05E-131	CD8.Mem	RACK1
NACA1	8.06E-135	0.467039	0.933	0.842	2.27E-130	CD8.Mem	NACA
RPSA1	8.12E-135	0.495727	0.911	0.816	2.29E-130	CD8.Mem	RPSA
RPL362	6.42E-132	0.402267	0.958	0.878	1.81E-127	CD8.Mem	RPL36
RPL122	2.52E-131	0.408761	0.97	0.905	7.08E-127	CD8.Mem	RPL12
RPL62	4.43E-130	0.407691	0.964	0.886	1.25E-125	CD8.Mem	RPL6
RPL211	1.05E-128	0.59162	0.902	0.808	2.94E-124	CD8.Mem	RPL21
NDFIP11	7.37E-125	0.850665	0.543	0.364	2.07E-120	CD8.Mem	NDFIP1
SOCS31	2.74E-124	0.736069	0.326	0.155	7.70E-120	CD8.Mem	SOCS3
RPS91	1.63E-121	0.44831	0.933	0.85	4.60E-117	CD8.Mem	RPS9
RPLP22	6.99E-120	0.382786	0.965	0.886	1.97E-115	CD8.Mem	RPLP2
AREG	1.07E-119	0.458935	0.422	0.217	3.01E-115	CD8.Mem	AREG
RPL10A1	2.33E-118	0.455089	0.906	0.816	6.54E-114	CD8.Mem	RPL10A
ZNF6831	1.90E-117	1.018788	0.302	0.143	5.36E-113	CD8.Mem	ZNF683
RPL33	4.71E-117	0.443248	0.951	0.884	1.32E-112	CD8.Mem	RPL3
IFITM11	5.87E-113	0.59408	0.916	0.768	1.65E-108	CD8.Mem	IFITM1
RPL91	8.84E-113	0.50848	0.901	0.819	2.49E-108	CD8.Mem	RPL9
EEF1G1	5.56E-108	0.5409	0.794	0.685	1.57E-103	CD8.Mem	EEF1G
PRKCQ-AS1	1.96E-106	0.671053	0.325	0.163	5.52E-102	CD8.Mem	PRKCQ-AS1
RPS73	5.82E-106	0.334714	0.967	0.899	1.64E-101	CD8.Mem	RPS7
RPS33	3.17E-105	0.328636	0.977	0.915	8.93E-101	CD8.Mem	RPS3
RPL42	5.58E-105	0.558753	0.81	0.711	1.57E-100	CD8.Mem	RPL4
RPS253	1.56E-104	0.35546	0.959	0.887	4.39E-100	CD8.Mem	RPS25
SATB1	2.43E-102	0.572024	0.301	0.148	6.82E-98	CD8.Mem	SATB1
TXNIP1	1.91E-101	0.445334	0.869	0.718	5.37E-97	CD8.Mem	TXNIP
TPT11	4.05E-101	0.390377	0.973	0.918	1.14E-96	CD8.Mem	TPT1
RPS152	1.87E-100	0.312308	0.975	0.914	5.25E-96	CD8.Mem	RPS15
RPL261	1.81E-99	0.314287	0.97	0.903	5.09E-95	CD8.Mem	RPL26
PIK3IP1	5.38E-95	0.537103	0.613	0.436	1.51E-90	CD8.Mem	PIK3IP1
TMSB10	8.46E-94	0.353931	0.971	0.919	2.38E-89	CD8.Mem	TMSB10
RPS272	2.72E-92	0.300424	0.985	0.934	7.65E-88	CD8.Mem	RPS27
FCMR	2.93E-91	0.550839	0.522	0.345	8.24E-87	CD8.Mem	FCMR
RASGRP2	4.75E-89	0.538942	0.356	0.198	1.34E-84	CD8.Mem	RASGRP2
TMEM243	1.01E-88	0.516754	0.386	0.225	2.85E-84	CD8.Mem	TMEM243
RPS22	8.07E-87	0.464748	0.937	0.872	2.27E-82	CD8.Mem	RPS2
RPS161	9.78E-87	0.383309	0.916	0.835	2.75E-82	CD8.Mem	RPS16

ITPKB	1.55E-86	0.444685	0.349	0.19	4.36E-82	CD8.Mem	ITPKB
RPS241	1.33E-84	0.284698	0.977	0.91	3.74E-80	CD8.Mem	RPS24
RPS15A3	3.15E-84	0.289413	0.98	0.922	8.88E-80	CD8.Mem	RPS15A
NOP531	6.23E-82	0.511237	0.671	0.535	1.75E-77	CD8.Mem	NOP53
GYPC1	1.98E-79	0.492069	0.666	0.511	5.58E-75	CD8.Mem	GYPC
EEF21	9.05E-79	0.454865	0.836	0.746	2.55E-74	CD8.Mem	EEF2
EEF1D1	1.37E-78	0.34671	0.9	0.808	3.86E-74	CD8.Mem	EEF1D
RPL151	3.91E-77	0.344513	0.924	0.839	1.10E-72	CD8.Mem	RPL15
FAU3	6.42E-75	0.269191	0.97	0.898	1.81E-70	CD8.Mem	FAU
RPL71	9.44E-73	0.471353	0.745	0.643	2.66E-68	CD8.Mem	RPL7
SERINC5	1.55E-68	0.4259	0.296	0.162	4.35E-64	CD8.Mem	SERINC5
PFDN52	3.16E-67	0.352426	0.854	0.766	8.89E-63	CD8.Mem	PFDN5
SNHG8	1.84E-66	0.451654	0.541	0.392	5.17E-62	CD8.Mem	SNHG8
RPL171	1.27E-65	0.304087	0.937	0.86	3.57E-61	CD8.Mem	RPL17
MCUB1	4.16E-63	0.401358	0.441	0.294	1.17E-58	CD8.Mem	MCUB
EIF3E1	5.40E-63	0.456936	0.554	0.419	1.52E-58	CD8.Mem	EIF3E
PIM11	1.43E-62	0.502902	0.356	0.222	4.02E-58	CD8.Mem	PIM1
RPLP01	3.16E-62	0.318567	0.934	0.868	8.89E-58	CD8.Mem	RPLP0
PCED1B-AS	6.33E-62	0.465656	0.656	0.528	1.78E-57	CD8.Mem	PCED1B-AS1
RPL271	3.28E-61	0.342823	0.845	0.748	9.22E-57	CD8.Mem	RPL27
FXYD51	6.49E-61	0.357641	0.805	0.672	1.82E-56	CD8.Mem	FXYD5
GAS51	1.04E-59	0.464379	0.581	0.457	2.93E-55	CD8.Mem	GAS5
SNHG291	1.36E-59	0.444271	0.667	0.556	3.83E-55	CD8.Mem	SNHG29
RPL382	1.16E-56	0.32815	0.815	0.705	3.26E-52	CD8.Mem	RPL38
GADD45A	1.46E-56	0.530778	0.268	0.157	4.10E-52	CD8.Mem	GADD45A
EIF3L	2.18E-56	0.405789	0.501	0.37	6.12E-52	CD8.Mem	EIF3L
UBA521	3.84E-56	0.286955	0.893	0.799	1.08E-51	CD8.Mem	UBA52
MAML21	4.15E-56	0.306951	0.474	0.324	1.17E-51	CD8.Mem	MAML2
DGKA	2.07E-54	0.364658	0.302	0.18	5.81E-50	CD8.Mem	DGKA
APBA2	4.30E-54	0.327326	0.307	0.182	1.21E-49	CD8.Mem	APBA2
ATM1	8.19E-54	0.387327	0.443	0.306	2.31E-49	CD8.Mem	ATM
NPM11	7.52E-51	0.330466	0.812	0.716	2.12E-46	CD8.Mem	NPM1
FLT3LG1	1.48E-50	0.422135	0.337	0.217	4.16E-46	CD8.Mem	FLT3LG
RPL37A	4.85E-49	0.284664	0.886	0.799	1.37E-44	CD8.Mem	RPL37A
CD272	4.85E-48	0.315725	0.452	0.31	1.37E-43	CD8.Mem	CD27
ANP32B1	1.76E-46	0.384939	0.51	0.391	4.94E-42	CD8.Mem	ANP32B
AKT3	6.27E-46	0.300235	0.273	0.164	1.76E-41	CD8.Mem	AKT3
GTF3A	1.46E-45	0.389861	0.461	0.339	4.10E-41	CD8.Mem	GTF3A
EIF2S3	1.83E-45	0.376073	0.355	0.242	5.15E-41	CD8.Mem	EIF2S3
PIM21	5.64E-44	0.458076	0.326	0.216	1.59E-39	CD8.Mem	PIM2
LIMD2	1.35E-42	0.296135	0.621	0.484	3.79E-38	CD8.Mem	LIMD2
TESPA11	3.64E-42	0.3168	0.269	0.165	1.02E-37	CD8.Mem	TESPA1
BTF31	6.57E-42	0.276264	0.822	0.728	1.85E-37	CD8.Mem	BTF3
RPL311	9.01E-41	0.267946	0.807	0.724	2.53E-36	CD8.Mem	RPL31
RPL36A1	1.53E-39	0.27185	0.858	0.779	4.31E-35	CD8.Mem	RPL36A
COX4I1	1.58E-39	0.262877	0.827	0.737	4.44E-35	CD8.Mem	COX4I1
TSC22D32	3.28E-39	0.286775	0.921	0.849	9.23E-35	CD8.Mem	TSC22D3

RCAN31	9.06E-39	0.290611	0.315	0.206	2.55E-34	CD8.Mem	RCAN3
ADD31	5.41E-38	0.281204	0.362	0.25	1.52E-33	CD8.Mem	ADD3
TLE5	6.49E-38	0.277821	0.727	0.619	1.82E-33	CD8.Mem	TLE5
SNHG61	7.00E-38	0.349268	0.58	0.47	1.97E-33	CD8.Mem	SNHG6
HNRNPA01	3.22E-37	0.343305	0.568	0.464	9.05E-33	CD8.Mem	HNRNPA0
UXT	5.30E-37	0.339232	0.481	0.373	1.49E-32	CD8.Mem	UXT
RAPGEF61	1.52E-36	0.350237	0.377	0.268	4.26E-32	CD8.Mem	RAPGEF6
VAV31	3.23E-36	0.295538	0.318	0.215	9.09E-32	CD8.Mem	VAV3
KLF3	5.68E-36	0.335759	0.315	0.214	1.60E-31	CD8.Mem	KLF3
HSPB12	5.83E-36	0.286038	0.392	0.276	1.64E-31	CD8.Mem	HSPB1
RNF138	8.13E-36	0.311107	0.315	0.215	2.29E-31	CD8.Mem	RNF138
EIF3H	1.61E-35	0.295494	0.573	0.47	4.53E-31	CD8.Mem	EIF3H
PRKCA1	1.69E-35	0.276437	0.278	0.181	4.76E-31	CD8.Mem	PRKCA
JUNB2	2.54E-35	0.252891	0.938	0.872	7.16E-31	CD8.Mem	JUNB
SEMA4D1	3.03E-35	0.260996	0.48	0.363	8.54E-31	CD8.Mem	SEMA4D
C12orf57	3.48E-35	0.300999	0.645	0.539	9.78E-31	CD8.Mem	C12orf57
IL6ST2	1.88E-33	0.378303	0.344	0.246	5.29E-29	CD8.Mem	IL6ST
EIF4B	1.01E-32	0.327092	0.483	0.379	2.84E-28	CD8.Mem	EIF4B
GPCPD11	3.84E-32	0.275468	0.363	0.262	1.08E-27	CD8.Mem	GPCPD1
RPS17	9.67E-32	0.299565	0.566	0.474	2.72E-27	CD8.Mem	RPS17
NAP1L4	2.31E-31	0.254666	0.488	0.378	6.51E-27	CD8.Mem	NAP1L4
NME21	6.55E-30	0.298331	0.507	0.399	1.84E-25	CD8.Mem	NME2
BIRC2	1.51E-29	0.292522	0.252	0.169	4.26E-25	CD8.Mem	BIRC2
STK38	4.82E-28	0.271459	0.298	0.208	1.36E-23	CD8.Mem	STK38
XCL11	2.48E-27	0.467986	0.274	0.19	6.98E-23	CD8.Mem	XCL1
RPL23	7.67E-27	0.26585	0.582	0.493	2.16E-22	CD8.Mem	RPL23
RSL1D11	3.38E-26	0.285067	0.394	0.304	9.50E-22	CD8.Mem	RSL1D1
PRMT2	3.47E-26	0.257626	0.466	0.372	9.78E-22	CD8.Mem	PRMT2
FBL	6.56E-26	0.28657	0.305	0.222	1.84E-21	CD8.Mem	FBL
RGS101	1.79E-23	0.250269	0.488	0.391	5.04E-19	CD8.Mem	RGS10
TUBA1A1	6.96E-23	0.272647	0.445	0.358	1.96E-18	CD8.Mem	TUBA1A
ATP5PO	1.02E-22	0.258107	0.46	0.372	2.87E-18	CD8.Mem	ATP5PO
HMGN3	4.20E-21	0.281144	0.317	0.241	1.18E-16	CD8.Mem	HMGN3
VSIR1	1.69E-20	0.260162	0.444	0.358	4.75E-16	CD8.Mem	VSIR
DCXR	1.86E-20	0.276089	0.295	0.222	5.23E-16	CD8.Mem	DCXR
SNHG7	3.14E-20	0.255808	0.381	0.301	8.83E-16	CD8.Mem	SNHG7
ST13	7.77E-20	0.251495	0.39	0.314	2.19E-15	CD8.Mem	ST13
TAGLN21	4.77E-18	0.273764	0.568	0.497	1.34E-13	CD8.Mem	TAGLN2
IFITM2	5.36E-18	0.251295	0.712	0.628	1.51E-13	CD8.Mem	IFITM2
LYAR1	1.99E-09	0.359854	0.357	0.325	5.59E-05	CD8.Mem	LYAR
GNLY	0	3.327123	0.913	0.193	0	NK.Tim3	GNLY
GZMB	0	3.192233	0.909	0.127	0	NK.Tim3	GZMB
FGFBP2	0	2.923747	0.777	0.05	0	NK.Tim3	FGFBP2
PRF1	0	2.658286	0.914	0.317	0	NK.Tim3	PRF1
NKG71	0	2.636045	0.991	0.501	0	NK.Tim3	NKG7
SPON2	0	2.597985	0.806	0.229	0	NK.Tim3	SPON2
CLIC3	0	2.248804	0.719	0.169	0	NK.Tim3	CLIC3

FCGR3A1	0	2.217069	0.713	0.123	0	NK.Tim3	FCGR3A
KLRD1	0	2.040083	0.876	0.247	0	NK.Tim3	KLRD1
EFHD2	0	1.981324	0.823	0.295	0	NK.Tim3	EFHD2
KLRF1	0	1.980697	0.51	0.024	0	NK.Tim3	KLRF1
ADGRG11	0	1.828082	0.586	0.085	0	NK.Tim3	ADGRG1
AREG1	0	1.781594	0.528	0.213	0	NK.Tim3	AREG
FCER1G1	0	1.703142	0.605	0.157	0	NK.Tim3	FCER1G
S1PR5	0	1.694913	0.494	0.036	0	NK.Tim3	S1PR5
TTC38	0	1.507706	0.464	0.05	0	NK.Tim3	TTC38
SH2D1B	0	1.506307	0.379	0.021	0	NK.Tim3	SH2D1B
TYROBP1	0	1.489161	0.741	0.283	0	NK.Tim3	TYROBP
PLAC8	0	1.461638	0.501	0.131	0	NK.Tim3	PLAC8
CTSW1	0	1.460969	0.887	0.48	0	NK.Tim3	CTSW
CST71	0	1.421991	0.936	0.571	0	NK.Tim3	CST7
IGFBP7	0	1.351725	0.306	0.012	0	NK.Tim3	IGFBP7
CX3CR11	0	1.349709	0.466	0.098	0	NK.Tim3	CX3CR1
PRSS23	0	1.341869	0.364	0.015	0	NK.Tim3	PRSS23
PTPN12	0	1.331954	0.426	0.072	0	NK.Tim3	PTPN12
IFITM21	0	1.279824	0.892	0.617	0	NK.Tim3	IFITM2
FGR	0	1.272236	0.398	0.055	0	NK.Tim3	FGR
PLEK1	0	1.270915	0.571	0.167	0	NK.Tim3	PLEK
CD2471	0	1.265559	0.847	0.544	0	NK.Tim3	CD247
C1orf21	0	1.264434	0.442	0.067	0	NK.Tim3	C1orf21
ABHD17A	0	1.231048	0.741	0.38	0	NK.Tim3	ABHD17A
CD71	0	1.190683	0.843	0.499	0	NK.Tim3	CD7
MCTP2	0	1.16909	0.515	0.152	0	NK.Tim3	MCTP2
ITGB2	0	1.160392	0.841	0.553	0	NK.Tim3	ITGB2
OSBPL5	0	1.145371	0.319	0.027	0	NK.Tim3	OSBPL5
RAP1GAP2	0	1.121709	0.36	0.057	0	NK.Tim3	RAP1GAP2
GSAP	0	1.11606	0.352	0.076	0	NK.Tim3	GSAP
BIN2	0	1.112278	0.711	0.369	0	NK.Tim3	BIN2
FLNA1	0	1.096519	0.678	0.342	0	NK.Tim3	FLNA
TBX21	0	1.07829	0.543	0.207	0	NK.Tim3	TBX21
HSH2D	0	1.075427	0.397	0.109	0	NK.Tim3	HSH2D
ZEB22	0	1.042696	0.667	0.286	0	NK.Tim3	ZEB2
LINC00299	0	1.012607	0.311	0.054	0	NK.Tim3	LINC00299
CYBA1	0	1.005678	0.924	0.748	0	NK.Tim3	CYBA
LPCAT1	0	0.95223	0.379	0.108	0	NK.Tim3	LPCAT1
ITGAM	0	0.949763	0.309	0.068	0	NK.Tim3	ITGAM
CEP78	0	0.942719	0.317	0.064	0	NK.Tim3	CEP78
PFN11	0	0.921297	0.979	0.849	0	NK.Tim3	PFN1
HLA-C	0	0.732435	0.985	0.908	0	NK.Tim3	HLA-C
IFITM12	3.99E-306	0.833715	0.95	0.767	1.12E-301	NK.Tim3	IFITM1
PXN	1.06E-304	0.972919	0.465	0.163	2.99E-300	NK.Tim3	PXN
TPST2	1.21E-302	0.987264	0.475	0.17	3.41E-298	NK.Tim3	TPST2
MTSS11	4.13E-298	0.927284	0.302	0.075	1.16E-293	NK.Tim3	MTSS1
GK5	2.73E-284	0.835726	0.251	0.054	7.69E-280	NK.Tim3	GK5

GZMH2	7.80E-284	1.211265	0.658	0.32	2.19E-279	NK.Tim3	GZMH
FCRL6	9.81E-282	0.836104	0.388	0.118	2.76E-277	NK.Tim3	FCRL6
NCR3	7.00E-269	0.891466	0.313	0.085	1.97E-264	NK.Tim3	NCR3
RAP1B	9.81E-257	0.893137	0.787	0.533	2.76E-252	NK.Tim3	RAP1B
TXK1	2.92E-256	0.869305	0.419	0.145	8.23E-252	NK.Tim3	TXK
LYN1	1.45E-237	0.857134	0.372	0.124	4.07E-233	NK.Tim3	LYN
SYNE11	4.85E-236	0.915016	0.591	0.283	1.37E-231	NK.Tim3	SYNE1
ARL4C1	5.69E-234	0.889722	0.814	0.544	1.60E-229	NK.Tim3	ARL4C
HOPX	9.97E-234	1.076111	0.564	0.27	2.80E-229	NK.Tim3	HOPX
APMAP1	2.76E-230	0.905626	0.588	0.291	7.78E-226	NK.Tim3	APMAP
TLE51	8.17E-227	0.825838	0.835	0.614	2.30E-222	NK.Tim3	TLE5
GZMM1	1.73E-220	0.864059	0.676	0.379	4.86E-216	NK.Tim3	GZMM
SELPLG1	1.80E-220	0.934485	0.653	0.368	5.07E-216	NK.Tim3	SELPLG
GFOD1	1.14E-219	0.762232	0.348	0.115	3.21E-215	NK.Tim3	GFOD1
UBE2F	1.68E-215	0.848057	0.385	0.145	4.72E-211	NK.Tim3	UBE2F
TMSB101	2.12E-215	0.575349	0.992	0.918	5.98E-211	NK.Tim3	TMSB10
METRNL1	9.24E-215	0.910031	0.481	0.205	2.60E-210	NK.Tim3	METRNL
CD300A1	1.87E-212	0.866612	0.334	0.114	5.27E-208	NK.Tim3	CD300A
KLRK11	6.69E-209	0.768455	0.665	0.341	1.88E-204	NK.Tim3	KLRK1
KLF23	1.19E-206	0.646174	0.815	0.448	3.34E-202	NK.Tim3	KLF2
UCP2	4.50E-206	0.937738	0.644	0.376	1.27E-201	NK.Tim3	UCP2
GZMA2	4.65E-203	0.771046	0.836	0.541	1.31E-198	NK.Tim3	GZMA
EMP3	1.37E-200	0.837898	0.696	0.425	3.85E-196	NK.Tim3	EMP3
MYL12A1	1.46E-197	0.718104	0.912	0.751	4.10E-193	NK.Tim3	MYL12A
RAC22	8.48E-196	0.753371	0.837	0.649	2.39E-191	NK.Tim3	RAC2
LITAF	1.07E-195	0.75926	0.77	0.501	3.01E-191	NK.Tim3	LITAF
CCL42	1.68E-190	0.97743	0.922	0.697	4.73E-186	NK.Tim3	CCL4
HLA-E	5.40E-190	0.541535	0.965	0.869	1.52E-185	NK.Tim3	HLA-E
SPN	5.47E-190	0.861158	0.453	0.204	1.54E-185	NK.Tim3	SPN
CDC42SE1	8.51E-188	0.850699	0.615	0.349	2.39E-183	NK.Tim3	CDC42SE1
HIPK2	7.26E-186	0.850901	0.322	0.115	2.04E-181	NK.Tim3	HIPK2
MYO1F1	2.35E-181	0.780847	0.5	0.24	6.62E-177	NK.Tim3	MYO1F
B2M2	2.15E-180	0.40747	0.996	0.97	6.05E-176	NK.Tim3	B2M
ARPC2	1.50E-175	0.704292	0.833	0.65	4.22E-171	NK.Tim3	ARPC2
IGF2R	8.21E-175	0.750575	0.455	0.208	2.31E-170	NK.Tim3	IGF2R
MAPK11	4.29E-174	0.809587	0.561	0.302	1.21E-169	NK.Tim3	MAPK1
ICAM2	1.65E-171	0.789698	0.352	0.14	4.64E-167	NK.Tim3	ICAM2
CD47	6.04E-168	0.757099	0.609	0.356	1.70E-163	NK.Tim3	CD47
ADAM8	3.63E-165	0.686688	0.315	0.117	1.02E-160	NK.Tim3	ADAM8
CMC1	8.31E-162	0.88991	0.43	0.202	2.34E-157	NK.Tim3	CMC1
C1orf1621	8.74E-161	0.791089	0.302	0.113	2.46E-156	NK.Tim3	C1orf162
ABI31	9.87E-161	0.758242	0.447	0.214	2.78E-156	NK.Tim3	ABI3
PPP1CA	8.12E-159	0.765239	0.62	0.385	2.29E-154	NK.Tim3	PPP1CA
SH3BP5	1.86E-158	0.714514	0.305	0.115	5.22E-154	NK.Tim3	SH3BP5
GSTP1	1.36E-157	0.749272	0.618	0.364	3.83E-153	NK.Tim3	GSTP1
SUN2	6.14E-156	0.779546	0.644	0.403	1.73E-151	NK.Tim3	SUN2
IL2RB	1.87E-155	0.804741	0.476	0.239	5.27E-151	NK.Tim3	IL2RB

RAB29	2.98E-155	0.675149	0.314	0.122	8.40E-151	NK.Tim3	RAB29
SLC9A3R1	8.13E-155	0.751348	0.626	0.384	2.29E-150	NK.Tim3	SLC9A3R1
CTSD1	5.56E-154	0.724835	0.682	0.445	1.56E-149	NK.Tim3	CTSD
RIN31	4.92E-153	0.716826	0.374	0.162	1.39E-148	NK.Tim3	RIN3
RASGRP21	2.17E-151	0.7204	0.422	0.196	6.11E-147	NK.Tim3	RASGRP2
TGFBR3	5.41E-151	0.68538	0.385	0.166	1.52E-146	NK.Tim3	TGFBR3
TFDP2	1.40E-149	0.663774	0.333	0.135	3.94E-145	NK.Tim3	TFDP2
AOAH1	1.22E-148	0.552057	0.662	0.386	3.44E-144	NK.Tim3	AOAH
HLA-B1	7.20E-148	0.403728	0.994	0.955	2.03E-143	NK.Tim3	HLA-B
LIMD21	1.10E-147	0.710791	0.703	0.481	3.09E-143	NK.Tim3	LIMD2
VAV32	3.35E-147	0.617283	0.445	0.207	9.44E-143	NK.Tim3	VAV3
GNG21	1.39E-140	0.625408	0.736	0.51	3.91E-136	NK.Tim3	GNG2
MBP1	2.03E-139	0.719018	0.595	0.365	5.71E-135	NK.Tim3	MBP
PTGDR	4.01E-139	0.68248	0.347	0.148	1.13E-134	NK.Tim3	PTGDR
PLEKHF1	2.36E-138	0.628079	0.289	0.112	6.63E-134	NK.Tim3	PLEKHF1
HLA-A1	4.76E-138	0.405627	0.986	0.925	1.34E-133	NK.Tim3	HLA-A
CARD111	5.99E-137	0.729765	0.463	0.237	1.69E-132	NK.Tim3	CARD11
CALM12	1.58E-136	0.508466	0.928	0.812	4.44E-132	NK.Tim3	CALM1
PYHIN11	2.44E-136	0.635452	0.507	0.267	6.86E-132	NK.Tim3	PYHIN1
FNDC3B	4.63E-135	0.627703	0.333	0.14	1.30E-130	NK.Tim3	FNDC3B
CFL11	3.09E-134	0.528419	0.924	0.807	8.68E-130	NK.Tim3	CFL1
TGFB11	9.80E-132	0.585215	0.814	0.614	2.76E-127	NK.Tim3	TGFB1
SLC44A2	5.59E-131	0.695441	0.386	0.185	1.57E-126	NK.Tim3	SLC44A2
SYTL1	6.80E-131	0.723825	0.417	0.21	1.91E-126	NK.Tim3	SYTL1
MATK	1.16E-130	0.737836	0.476	0.254	3.26E-126	NK.Tim3	MATK
SLC15A4	3.08E-130	0.636541	0.256	0.097	8.67E-126	NK.Tim3	SLC15A4
CCND31	6.11E-130	0.51922	0.814	0.629	1.72E-125	NK.Tim3	CCND3
MAP3K81	5.14E-129	0.754531	0.553	0.324	1.44E-124	NK.Tim3	MAP3K8
HCST2	4.67E-127	0.56861	0.862	0.699	1.31E-122	NK.Tim3	HCST
MYO1G	6.20E-127	0.641198	0.401	0.196	1.75E-122	NK.Tim3	MYO1G
IL2RG	4.31E-126	0.618689	0.788	0.613	1.21E-121	NK.Tim3	IL2RG
KLHDC4	1.62E-124	0.571408	0.288	0.116	4.56E-120	NK.Tim3	KLHDC4
YES1	2.12E-123	0.593577	0.325	0.139	5.97E-119	NK.Tim3	YES1
ZAP701	2.92E-123	0.630677	0.583	0.362	8.21E-119	NK.Tim3	ZAP70
RASSF1	6.42E-123	0.675532	0.428	0.222	1.81E-118	NK.Tim3	RASSF1
CDKN2D	3.37E-121	0.596901	0.261	0.102	9.49E-117	NK.Tim3	CDKN2D
KLRB11	7.06E-121	0.510703	0.612	0.342	1.99E-116	NK.Tim3	KLRB1
CLIC12	4.71E-120	0.588023	0.803	0.626	1.33E-115	NK.Tim3	CLIC1
PLCG2	1.10E-118	0.50039	0.257	0.099	3.11E-114	NK.Tim3	PLCG2
G6PD	3.57E-118	0.576298	0.264	0.106	1.01E-113	NK.Tim3	G6PD
PSMB10	3.73E-118	0.662245	0.524	0.313	1.05E-113	NK.Tim3	PSMB10
C5orf561	5.36E-115	0.59795	0.438	0.23	1.51E-110	NK.Tim3	C5orf56
ACTB1	1.12E-112	0.506916	0.992	0.939	3.16E-108	NK.Tim3	ACTB
DOK2	5.70E-111	0.64289	0.44	0.237	1.60E-106	NK.Tim3	DOK2
AGTRAP	2.21E-109	0.606159	0.318	0.147	6.21E-105	NK.Tim3	AGTRAP
CEBPD1	3.77E-107	0.309825	0.551	0.302	1.06E-102	NK.Tim3	CEBPD
PCBP1	2.34E-104	0.682455	0.571	0.383	6.59E-100	NK.Tim3	PCBP1

ARPC5L1	1.32E-103	0.613595	0.478	0.279	3.73E-99	NK.Tim3	ARPC5L
NCALD	2.23E-103	0.527799	0.35	0.168	6.27E-99	NK.Tim3	NCALD
SERPINB1	4.55E-103	0.646747	0.421	0.238	1.28E-98	NK.Tim3	SERPINB1
C12orf751	1.18E-102	0.591742	0.408	0.218	3.33E-98	NK.Tim3	C12orf75
NFATC21	2.48E-102	0.516037	0.571	0.358	6.97E-98	NK.Tim3	NFATC2
TRBC1	3.18E-102	0.737748	0.512	0.315	8.94E-98	NK.Tim3	TRBC1
UBB1	1.14E-100	0.502406	0.841	0.682	3.22E-96	NK.Tim3	UBB
PPP1R181	8.40E-100	0.603521	0.506	0.311	2.36E-95	NK.Tim3	PPP1R18
CHST12	8.64E-100	0.544459	0.429	0.232	2.43E-95	NK.Tim3	CHST12
JAK11	8.88E-99	0.561132	0.748	0.585	2.50E-94	NK.Tim3	JAK1
LY6E1	7.38E-98	0.66456	0.715	0.546	2.08E-93	NK.Tim3	LY6E
SH3BGRL32	1.73E-97	0.494298	0.917	0.804	4.86E-93	NK.Tim3	SH3BGRL3
CD992	1.08E-95	0.464089	0.84	0.689	3.04E-91	NK.Tim3	CD99
AGTPBP1	2.19E-95	0.508601	0.288	0.132	6.17E-91	NK.Tim3	AGTPBP1
BHLHE401	4.77E-95	0.627652	0.512	0.316	1.34E-90	NK.Tim3	BHLHE40
LSP11	9.67E-95	0.484363	0.778	0.608	2.72E-90	NK.Tim3	LSP1
DIP2A1	1.09E-94	0.5987	0.461	0.271	3.06E-90	NK.Tim3	DIP2A
ITGAL1	4.81E-94	0.530766	0.442	0.251	1.35E-89	NK.Tim3	ITGAL
PSME11	1.04E-93	0.520187	0.76	0.61	2.91E-89	NK.Tim3	PSME1
CD631	1.54E-93	0.593466	0.478	0.285	4.35E-89	NK.Tim3	CD63
MAPRE2	2.92E-93	0.579812	0.529	0.335	8.22E-89	NK.Tim3	MAPRE2
GPR651	5.26E-92	0.594341	0.416	0.234	1.48E-87	NK.Tim3	GPR65
AC092821.	5.74E-92	0.597454	0.394	0.218	1.62E-87	NK.Tim3	AC092821.3
SERTAD1	7.50E-92	0.676432	0.533	0.349	2.11E-87	NK.Tim3	SERTAD1
SASH3	1.77E-91	0.54744	0.411	0.232	4.98E-87	NK.Tim3	SASH3
FOSL21	9.11E-90	0.578066	0.492	0.301	2.56E-85	NK.Tim3	FOSL2
IQGAP21	5.56E-89	0.409566	0.648	0.447	1.57E-84	NK.Tim3	IQGAP2
KLF131	6.80E-89	0.539864	0.529	0.341	1.91E-84	NK.Tim3	KLF13
GNPTAB	5.16E-87	0.513612	0.382	0.206	1.45E-82	NK.Tim3	GNPTAB
GRASP1	3.12E-86	0.704192	0.27	0.127	8.77E-82	NK.Tim3	GRASP
FMNL1	4.77E-86	0.546569	0.47	0.29	1.34E-81	NK.Tim3	FMNL1
MIEN1	1.68E-85	0.513206	0.322	0.164	4.72E-81	NK.Tim3	MIEN1
SH2D2A	2.79E-85	0.550744	0.472	0.286	7.85E-81	NK.Tim3	SH2D2A
PTPRE1	2.75E-84	0.526069	0.344	0.182	7.75E-80	NK.Tim3	PTPRE
STK381	6.06E-84	0.459503	0.376	0.204	1.71E-79	NK.Tim3	STK38
SAMD31	2.15E-83	0.50452	0.472	0.283	6.06E-79	NK.Tim3	SAMD3
MSN	4.99E-83	0.487664	0.707	0.541	1.40E-78	NK.Tim3	MSN
PKN1	2.77E-82	0.507912	0.448	0.271	7.79E-78	NK.Tim3	PKN1
DSTN	5.15E-82	0.550664	0.374	0.21	1.45E-77	NK.Tim3	DSTN
CSK	6.25E-82	0.556084	0.492	0.32	1.76E-77	NK.Tim3	CSK
TES	6.42E-82	0.530009	0.379	0.213	1.81E-77	NK.Tim3	TES
CTBP1	2.33E-81	0.539393	0.441	0.267	6.56E-77	NK.Tim3	CTBP1
GPATCH81	2.99E-81	0.461635	0.435	0.255	8.41E-77	NK.Tim3	GPATCH8
HMGN31	6.81E-80	0.552423	0.402	0.237	1.92E-75	NK.Tim3	HMGN3
H3F3A	1.85E-78	0.429139	0.834	0.697	5.20E-74	NK.Tim3	H3F3A
NDUFB2	1.89E-78	0.531788	0.527	0.354	5.31E-74	NK.Tim3	NDUFB2
FBXW5	9.07E-78	0.553317	0.367	0.21	2.55E-73	NK.Tim3	FBXW5

OSTF1	3.78E-77	0.501859	0.522	0.349	1.06E-72	NK.Tim3	OSTF1
AKNA1	1.12E-76	0.429981	0.666	0.487	3.14E-72	NK.Tim3	AKNA
HAVCR21	6.84E-76	0.474342	0.276	0.135	1.93E-71	NK.Tim3	HAVCR2
PTPN6	1.06E-75	0.586424	0.381	0.224	2.99E-71	NK.Tim3	PTPN6
ACTG11	1.90E-75	0.484439	0.902	0.815	5.34E-71	NK.Tim3	ACTG1
PTPN181	2.46E-75	0.527134	0.287	0.147	6.92E-71	NK.Tim3	PTPN18
EIF3G	3.18E-75	0.561457	0.665	0.512	8.94E-71	NK.Tim3	EIF3G
SRPK21	5.40E-75	0.40422	0.472	0.293	1.52E-70	NK.Tim3	SRPK2
IRF1	5.88E-75	0.578693	0.599	0.429	1.65E-70	NK.Tim3	IRF1
SIRT2	1.07E-73	0.444072	0.346	0.191	3.02E-69	NK.Tim3	SIRT2
KLF31	1.28E-73	0.485632	0.37	0.212	3.60E-69	NK.Tim3	KLF3
LGALS11	1.34E-73	0.693169	0.351	0.196	3.76E-69	NK.Tim3	LGALS1
PRR5	1.37E-73	0.46586	0.294	0.151	3.85E-69	NK.Tim3	PRR5
PRMT21	2.18E-73	0.492352	0.541	0.368	6.13E-69	NK.Tim3	PRMT2
RUNX32	3.43E-73	0.384168	0.741	0.563	9.66E-69	NK.Tim3	RUNX3
DBI1	4.25E-73	0.436269	0.569	0.388	1.19E-68	NK.Tim3	DBI
CD38	6.44E-73	0.484243	0.283	0.142	1.81E-68	NK.Tim3	CD38
CD53	7.73E-73	0.468158	0.732	0.586	2.18E-68	NK.Tim3	CD53
RHOC	9.46E-73	0.62358	0.423	0.267	2.66E-68	NK.Tim3	RHOC
IER21	3.24E-71	0.441445	0.838	0.686	9.12E-67	NK.Tim3	IER2
PPIA	4.87E-71	0.400863	0.881	0.762	1.37E-66	NK.Tim3	PPIA
IFITM31	5.62E-71	0.578362	0.376	0.222	1.58E-66	NK.Tim3	IFITM3
CCDC69	1.10E-70	0.461804	0.282	0.145	3.09E-66	NK.Tim3	CCDC69
TAP1	2.74E-70	0.501967	0.444	0.284	7.70E-66	NK.Tim3	TAP1
DENND2D1	6.00E-70	0.505778	0.434	0.271	1.69E-65	NK.Tim3	DENND2D
ARPC4	1.44E-69	0.510845	0.502	0.344	4.04E-65	NK.Tim3	ARPC4
RIPOR22	1.07E-68	0.340735	0.508	0.323	3.00E-64	NK.Tim3	RIPOR2
MT2A1	1.61E-68	0.651679	0.634	0.48	4.52E-64	NK.Tim3	MT2A
TRAPPC101	3.16E-68	0.402164	0.429	0.263	8.89E-64	NK.Tim3	TRAPPC10
MIDN	3.20E-68	0.524875	0.422	0.263	9.00E-64	NK.Tim3	MIDN
ADGRE51	9.95E-68	0.350025	0.684	0.511	2.80E-63	NK.Tim3	ADGRE5
ZBTB16	1.10E-67	0.553827	0.373	0.221	3.11E-63	NK.Tim3	ZBTB16
CTSC	6.67E-67	0.435174	0.591	0.422	1.88E-62	NK.Tim3	CTSC
DHRS71	1.93E-66	0.526617	0.497	0.342	5.44E-62	NK.Tim3	DHRS7
ITPRIP	1.16E-65	0.500386	0.348	0.201	3.27E-61	NK.Tim3	ITPRIP
CORO1A1	1.81E-65	0.418097	0.824	0.704	5.10E-61	NK.Tim3	CORO1A
ANXA6	2.72E-64	0.450837	0.518	0.358	7.64E-60	NK.Tim3	ANXA6
SIPA1	1.45E-63	0.474443	0.384	0.236	4.09E-59	NK.Tim3	SIPA1
CD164	1.66E-63	0.479882	0.465	0.314	4.66E-59	NK.Tim3	CD164
SCLT1	7.04E-63	0.41871	0.278	0.147	1.98E-58	NK.Tim3	SCLT1
RHOA1	2.06E-62	0.398721	0.762	0.633	5.81E-58	NK.Tim3	RHOA
SAMHD11	3.37E-62	0.45226	0.509	0.346	9.49E-58	NK.Tim3	SAMHD1
PDIA3	1.15E-61	0.480348	0.636	0.491	3.23E-57	NK.Tim3	PDIA3
PSMB92	2.61E-61	0.446593	0.628	0.485	7.33E-57	NK.Tim3	PSMB9
TADA3	4.01E-61	0.450359	0.265	0.142	1.13E-56	NK.Tim3	TADA3
ARPC5	1.02E-60	0.469165	0.476	0.325	2.86E-56	NK.Tim3	ARPC5
TMBIM1	2.69E-60	0.492873	0.27	0.147	7.58E-56	NK.Tim3	TMBIM1

VASP	3.70E-60	0.471009	0.339	0.203	1.04E-55	NK.Tim3	VASP
CDK2AP2	4.97E-60	0.450706	0.329	0.19	1.40E-55	NK.Tim3	CDK2AP2
BSG	8.39E-60	0.446663	0.479	0.33	2.36E-55	NK.Tim3	BSG
ADD32	5.22E-59	0.427195	0.398	0.249	1.47E-54	NK.Tim3	ADD3
NDUFA12	7.09E-59	0.467568	0.388	0.246	2.00E-54	NK.Tim3	NDUFA12
CAPNS1	1.27E-58	0.462402	0.4	0.254	3.56E-54	NK.Tim3	CAPNS1
S100A42	2.19E-58	0.377025	0.821	0.653	6.18E-54	NK.Tim3	S100A4
ARHGAP9	3.15E-58	0.450422	0.543	0.392	8.87E-54	NK.Tim3	ARHGAP9
NDUFB7	8.54E-58	0.486192	0.374	0.237	2.40E-53	NK.Tim3	NDUFB7
PTPN4	1.12E-57	0.38027	0.435	0.282	3.14E-53	NK.Tim3	PTPN4
ITGB7	1.31E-57	0.460355	0.304	0.175	3.68E-53	NK.Tim3	ITGB7
TNFRSF1B1	2.50E-57	0.434727	0.488	0.334	7.03E-53	NK.Tim3	TNFRSF1B
RNF126	4.72E-57	0.430249	0.332	0.197	1.33E-52	NK.Tim3	RNF126
RPS6KA1	2.24E-56	0.433612	0.264	0.145	6.30E-52	NK.Tim3	RPS6KA1
SYNE21	2.36E-56	0.310963	0.572	0.402	6.64E-52	NK.Tim3	SYNE2
ORAI1	3.33E-56	0.438243	0.36	0.221	9.38E-52	NK.Tim3	ORAI1
NCOA1	4.31E-56	0.325063	0.302	0.169	1.21E-51	NK.Tim3	NCOA1
POLR2G	1.53E-55	0.405589	0.359	0.221	4.30E-51	NK.Tim3	POLR2G
GNB2	1.58E-55	0.442854	0.507	0.363	4.44E-51	NK.Tim3	GNB2
ARPC31	3.34E-55	0.361058	0.759	0.63	9.39E-51	NK.Tim3	ARPC3
IQGAP11	1.95E-54	0.404575	0.574	0.424	5.49E-50	NK.Tim3	IQGAP1
CYTH4	2.05E-54	0.330895	0.258	0.138	5.78E-50	NK.Tim3	CYTH4
SERF2	3.00E-54	0.310658	0.894	0.787	8.45E-50	NK.Tim3	SERF2
DDX39A	8.37E-54	0.437069	0.372	0.236	2.36E-49	NK.Tim3	DDX39A
FAM49B1	1.48E-53	0.326462	0.581	0.426	4.17E-49	NK.Tim3	FAM49B
PLAAT42	5.33E-53	0.407698	0.705	0.583	1.50E-48	NK.Tim3	PLAAT4
YPEL3	9.86E-53	0.438081	0.574	0.432	2.78E-48	NK.Tim3	YPEL3
WDR1	9.93E-53	0.467461	0.469	0.336	2.80E-48	NK.Tim3	WDR1
PSMB8	1.87E-52	0.430662	0.533	0.397	5.27E-48	NK.Tim3	PSMB8
CFLAR1	2.70E-52	0.384416	0.545	0.392	7.59E-48	NK.Tim3	CFLAR
CTDSP1	1.11E-51	0.439078	0.293	0.172	3.13E-47	NK.Tim3	CTDSP1
RAB5IF1	1.84E-51	0.478381	0.399	0.265	5.17E-47	NK.Tim3	RAB5IF
ATM2	1.03E-50	0.368893	0.453	0.307	2.91E-46	NK.Tim3	ATM
PPP3CC	1.70E-50	0.342719	0.397	0.256	4.79E-46	NK.Tim3	PPP3CC
BTN3A2	2.33E-50	0.382121	0.359	0.225	6.57E-46	NK.Tim3	BTN3A2
CISD3	2.54E-50	0.413106	0.265	0.15	7.16E-46	NK.Tim3	CISD3
TLN11	3.28E-50	0.427811	0.486	0.344	9.23E-46	NK.Tim3	TLN1
HIVEP3	5.44E-50	0.347715	0.265	0.148	1.53E-45	NK.Tim3	HIVEP3
SDF4	9.95E-50	0.437208	0.357	0.23	2.80E-45	NK.Tim3	SDF4
PAM	1.09E-49	0.282737	0.29	0.165	3.07E-45	NK.Tim3	PAM
ISG20	3.22E-49	0.341753	0.72	0.573	9.07E-45	NK.Tim3	ISG20
CYC1	5.30E-49	0.392789	0.266	0.153	1.49E-44	NK.Tim3	CYC1
XCL2	6.77E-49	0.839161	0.271	0.153	1.90E-44	NK.Tim3	XCL2
UTRN1	7.99E-49	0.29239	0.597	0.445	2.25E-44	NK.Tim3	UTRN
ZNF276	1.96E-48	0.355479	0.262	0.148	5.51E-44	NK.Tim3	ZNF276
ACTR3	2.04E-48	0.411753	0.547	0.417	5.73E-44	NK.Tim3	ACTR3
TCF251	2.34E-48	0.368509	0.561	0.424	6.58E-44	NK.Tim3	TCF25

P2RY81	4.70E-48	0.312151	0.486	0.338	1.32E-43	NK.Tim3	P2RY8
TBC1D10C1	4.82E-48	0.393154	0.54	0.402	1.36E-43	NK.Tim3	TBC1D10C
XBP11	5.10E-48	0.444078	0.488	0.354	1.43E-43	NK.Tim3	XBP1
HLA-F	7.08E-48	0.347898	0.624	0.48	1.99E-43	NK.Tim3	HLA-F
STK101	9.21E-48	0.295061	0.523	0.372	2.59E-43	NK.Tim3	STK10
CAP11	1.16E-47	0.404685	0.575	0.45	3.26E-43	NK.Tim3	CAP1
TRAPPC1	2.40E-47	0.377247	0.442	0.308	6.76E-43	NK.Tim3	TRAPPC1
JAZF11	3.32E-47	0.287751	0.326	0.198	9.34E-43	NK.Tim3	JAZF1
CDC42	8.72E-47	0.370503	0.614	0.483	2.45E-42	NK.Tim3	CDC42
IFNG1	9.32E-47	0.555035	0.262	0.149	2.62E-42	NK.Tim3	IFNG
LASP1	9.47E-47	0.37374	0.306	0.186	2.66E-42	NK.Tim3	LASP1
SLA2	2.07E-46	0.349374	0.317	0.191	5.82E-42	NK.Tim3	SLA2
PTPRA	5.29E-46	0.335773	0.331	0.204	1.49E-41	NK.Tim3	PTPRA
RNPEPL1	6.81E-46	0.398587	0.362	0.238	1.92E-41	NK.Tim3	RNPEPL1
PAXX	1.23E-45	0.395125	0.583	0.459	3.47E-41	NK.Tim3	PAXX
BZW1	3.70E-45	0.402033	0.521	0.39	1.04E-40	NK.Tim3	BZW1
TNFRSF14	7.28E-45	0.404074	0.383	0.257	2.05E-40	NK.Tim3	TNFRSF14
PRR13	7.59E-45	0.380749	0.555	0.426	2.14E-40	NK.Tim3	PRR13
SEPTIN7	7.97E-45	0.324524	0.639	0.503	2.24E-40	NK.Tim3	SEPTIN7
ADD1	9.41E-45	0.383349	0.363	0.239	2.65E-40	NK.Tim3	ADD1
NAPA	2.01E-44	0.372854	0.439	0.31	5.66E-40	NK.Tim3	NAPA
MAP2K2	1.06E-43	0.390808	0.426	0.3	2.99E-39	NK.Tim3	MAP2K2
ARHGEF11	1.83E-43	0.325715	0.649	0.517	5.15E-39	NK.Tim3	ARHGEF1
TPM3	3.18E-43	0.344527	0.692	0.574	8.94E-39	NK.Tim3	TPM3
LAMP1	4.44E-43	0.40698	0.408	0.285	1.25E-38	NK.Tim3	LAMP1
RNF166	7.94E-43	0.356458	0.353	0.232	2.23E-38	NK.Tim3	RNF166
DDOST	1.29E-42	0.389438	0.435	0.31	3.63E-38	NK.Tim3	DDOST
ARPC1B	1.73E-42	0.346521	0.659	0.539	4.88E-38	NK.Tim3	ARPC1B
PSME2	1.90E-42	0.417142	0.538	0.417	5.34E-38	NK.Tim3	PSME2
GLIPR2	5.44E-42	0.393248	0.301	0.188	1.53E-37	NK.Tim3	GLIPR2
TPI1	5.73E-42	0.354684	0.621	0.491	1.61E-37	NK.Tim3	TPI1
INPP4A1	6.97E-42	0.285054	0.374	0.245	1.96E-37	NK.Tim3	INPP4A
RALY	1.08E-41	0.321509	0.537	0.403	3.03E-37	NK.Tim3	RALY
CYFIP21	1.31E-41	0.328985	0.456	0.323	3.68E-37	NK.Tim3	CYFIP2
MAFF	1.92E-41	0.427533	0.298	0.187	5.41E-37	NK.Tim3	MAFF
RNASEK1	2.34E-41	0.334828	0.659	0.53	6.59E-37	NK.Tim3	RNASEK
CCDC88C1	2.79E-41	0.272003	0.448	0.309	7.85E-37	NK.Tim3	CCDC88C
ACTN41	4.07E-41	0.32674	0.43	0.295	1.15E-36	NK.Tim3	ACTN4
YWHAZ	3.97E-40	0.259189	0.818	0.725	1.12E-35	NK.Tim3	YWHAZ
NFATC3	5.07E-40	0.254303	0.353	0.228	1.43E-35	NK.Tim3	NFATC3
DCXR1	6.52E-40	0.363015	0.337	0.22	1.83E-35	NK.Tim3	DCXR
DGKZ	9.04E-40	0.362067	0.391	0.269	2.54E-35	NK.Tim3	DGKZ
APOL6	2.42E-39	0.279742	0.273	0.164	6.80E-35	NK.Tim3	APOL6
SCP2	4.97E-39	0.342857	0.361	0.246	1.40E-34	NK.Tim3	SCP2
KCNAB2	9.07E-39	0.30741	0.326	0.211	2.55E-34	NK.Tim3	KCNAB2
RAB8A	9.40E-39	0.349119	0.276	0.171	2.65E-34	NK.Tim3	RAB8A
RGS191	9.47E-39	0.391052	0.322	0.213	2.67E-34	NK.Tim3	RGS19

RC3H1	1.08E-38	0.299292	0.336	0.219	3.04E-34	NK.Tim3	RC3H1
CLASP11	2.47E-38	0.290657	0.299	0.187	6.94E-34	NK.Tim3	CLASP1
TAGLN22	4.72E-38	0.32478	0.623	0.494	1.33E-33	NK.Tim3	TAGLN2
PPP2R5A	7.20E-38	0.287082	0.314	0.2	2.03E-33	NK.Tim3	PPP2R5A
RBM391	8.37E-38	0.263984	0.747	0.643	2.35E-33	NK.Tim3	RBM39
PRDX5	1.89E-37	0.389451	0.39	0.276	5.31E-33	NK.Tim3	PRDX5
ARF61	2.67E-37	0.340541	0.573	0.45	7.50E-33	NK.Tim3	ARF6
GNAI2	3.48E-37	0.318714	0.67	0.556	9.80E-33	NK.Tim3	GNAI2
RBM381	5.09E-37	0.306793	0.523	0.387	1.43E-32	NK.Tim3	RBM38
RPA2	5.44E-37	0.341605	0.268	0.167	1.53E-32	NK.Tim3	RPA2
CD552	1.58E-36	0.283914	0.536	0.399	4.45E-32	NK.Tim3	CD55
PRELID1	2.45E-36	0.349132	0.389	0.276	6.88E-32	NK.Tim3	PRELID1
S1PR41	3.30E-36	0.336484	0.286	0.181	9.27E-32	NK.Tim3	S1PR4
RAB5C	6.07E-36	0.327876	0.389	0.275	1.71E-31	NK.Tim3	RAB5C
HMOX2	8.39E-36	0.311983	0.377	0.26	2.36E-31	NK.Tim3	HMOX2
RBBP4	8.95E-36	0.317537	0.367	0.255	2.52E-31	NK.Tim3	RBBP4
SUPT4H1	1.25E-35	0.383438	0.3	0.198	3.52E-31	NK.Tim3	SUPT4H1
SORL11	1.44E-35	0.286509	0.411	0.288	4.05E-31	NK.Tim3	SORL1
RAB8B1	1.54E-35	0.276011	0.38	0.263	4.35E-31	NK.Tim3	RAB8B
UBE2E3	3.11E-35	0.300522	0.25	0.152	8.74E-31	NK.Tim3	UBE2E3
DIAPH11	5.19E-35	0.278658	0.387	0.268	1.46E-30	NK.Tim3	DIAPH1
PLCL21	5.96E-35	0.265226	0.368	0.25	1.68E-30	NK.Tim3	PLCL2
APOBEC3G	1.01E-34	0.319711	0.412	0.289	2.85E-30	NK.Tim3	APOBEC3G
DECR1	1.11E-34	0.294616	0.326	0.217	3.11E-30	NK.Tim3	DECR1
GLRX1	1.28E-34	0.352744	0.283	0.183	3.60E-30	NK.Tim3	GLRX
RHOF	2.08E-34	0.343852	0.37	0.255	5.87E-30	NK.Tim3	RHOF
DCTN3	2.16E-34	0.364672	0.341	0.235	6.09E-30	NK.Tim3	DCTN3
ACAA2	2.30E-34	0.280166	0.256	0.158	6.46E-30	NK.Tim3	ACAA2
ARHGAP30	4.60E-34	0.278074	0.371	0.256	1.30E-29	NK.Tim3	ARHGAP30
NT5C	4.94E-34	0.325961	0.267	0.169	1.39E-29	NK.Tim3	NT5C
TSC22D4	4.95E-34	0.327226	0.322	0.217	1.39E-29	NK.Tim3	TSC22D4
TAP2	5.17E-34	0.305926	0.363	0.251	1.46E-29	NK.Tim3	TAP2
TBCB	1.13E-33	0.329106	0.361	0.252	3.18E-29	NK.Tim3	TBCB
TCIRG1	1.23E-33	0.348901	0.318	0.213	3.46E-29	NK.Tim3	TCIRG1
CARD16	1.44E-33	0.312069	0.36	0.249	4.04E-29	NK.Tim3	CARD16
SFT2D1	2.24E-33	0.324537	0.252	0.158	6.29E-29	NK.Tim3	SFT2D1
LINC00861	3.79E-33	0.256312	0.34	0.226	1.07E-28	NK.Tim3	LINC00861
TWF2	8.39E-33	0.346728	0.329	0.229	2.36E-28	NK.Tim3	TWF2
FKBP111	9.84E-33	0.314023	0.362	0.25	2.77E-28	NK.Tim3	FKBP11
LMAN2	1.19E-32	0.32638	0.395	0.286	3.36E-28	NK.Tim3	LMAN2
BANF1	1.20E-32	0.353045	0.326	0.224	3.38E-28	NK.Tim3	BANF1
ATP5PB	1.91E-32	0.339356	0.414	0.304	5.37E-28	NK.Tim3	ATP5PB
ETFA	2.69E-32	0.300384	0.285	0.186	7.57E-28	NK.Tim3	ETFA
ARIH2	3.85E-32	0.290311	0.319	0.215	1.08E-27	NK.Tim3	ARIH2
SH3GLB1	6.25E-32	0.31814	0.286	0.188	1.76E-27	NK.Tim3	SH3GLB1
FAM117A	7.71E-32	0.297889	0.278	0.181	2.17E-27	NK.Tim3	FAM117A
BST21	1.43E-31	0.307926	0.47	0.351	4.01E-27	NK.Tim3	BST2

TRAF3IP31	1.60E-31	0.292834	0.452	0.337	4.50E-27	NK.Tim3	TRAF3IP3
EIF5A	2.45E-31	0.346768	0.429	0.322	6.88E-27	NK.Tim3	EIF5A
AP2M1	2.92E-31	0.328961	0.364	0.26	8.23E-27	NK.Tim3	AP2M1
UHMK1	3.64E-31	0.328491	0.252	0.161	1.03E-26	NK.Tim3	UHMK1
MTPN	3.88E-31	0.305669	0.35	0.247	1.09E-26	NK.Tim3	MTPN
TAPBP	1.16E-30	0.287997	0.489	0.377	3.26E-26	NK.Tim3	TAPBP
AP2S1	1.75E-30	0.276261	0.299	0.2	4.91E-26	NK.Tim3	AP2S1
CYBC1	3.95E-30	0.295707	0.258	0.166	1.11E-25	NK.Tim3	CYBC1
FCMR1	4.90E-30	0.302928	0.467	0.351	1.38E-25	NK.Tim3	FCMR
RASSF5	5.31E-30	0.311989	0.414	0.308	1.49E-25	NK.Tim3	RASSF5
STOM	5.83E-30	0.302908	0.329	0.228	1.64E-25	NK.Tim3	STOM
POLR2L	7.49E-30	0.318465	0.475	0.369	2.11E-25	NK.Tim3	POLR2L
GNG51	8.19E-30	0.282032	0.502	0.389	2.30E-25	NK.Tim3	GNG5
LRP10	8.99E-30	0.33159	0.275	0.184	2.53E-25	NK.Tim3	LRP10
ARF1	1.03E-29	0.26879	0.618	0.507	2.89E-25	NK.Tim3	ARF1
SHISA51	1.43E-29	0.298603	0.435	0.323	4.02E-25	NK.Tim3	SHISA5
ACADVL	1.73E-29	0.299548	0.294	0.198	4.88E-25	NK.Tim3	ACADVL
PTP4A21	1.77E-29	0.273291	0.544	0.433	4.99E-25	NK.Tim3	PTP4A2
TRIR1	2.03E-29	0.277559	0.639	0.527	5.70E-25	NK.Tim3	TRIR
P4HB	2.07E-29	0.296921	0.456	0.348	5.83E-25	NK.Tim3	P4HB
CAST1	2.23E-29	0.280262	0.464	0.35	6.26E-25	NK.Tim3	CAST
CALR	3.76E-29	0.327554	0.617	0.511	1.06E-24	NK.Tim3	CALR
ZBTB7A	7.68E-29	0.302387	0.296	0.201	2.16E-24	NK.Tim3	ZBTB7A
CCDC85B	1.06E-28	0.31377	0.448	0.344	2.99E-24	NK.Tim3	CCDC85B
PSMA2	1.24E-28	0.289528	0.367	0.268	3.49E-24	NK.Tim3	PSMA2
HDAC1	1.50E-28	0.263685	0.291	0.196	4.21E-24	NK.Tim3	HDAC1
MAT2B	3.71E-28	0.300298	0.361	0.262	1.04E-23	NK.Tim3	MAT2B
LCP11	4.29E-28	0.262222	0.731	0.631	1.21E-23	NK.Tim3	LCP1
LYAR2	6.04E-28	0.294413	0.435	0.32	1.70E-23	NK.Tim3	LYAR
HNRNPK	8.10E-28	0.289143	0.648	0.552	2.28E-23	NK.Tim3	HNRNPK
CAPN2	2.10E-27	0.293903	0.339	0.24	5.90E-23	NK.Tim3	CAPN2
CSNK2B	2.45E-27	0.267929	0.474	0.374	6.88E-23	NK.Tim3	CSNK2B
DUSP21	2.50E-27	0.287283	0.821	0.733	7.04E-23	NK.Tim3	DUSP2
SQSTM11	2.60E-27	0.25873	0.526	0.418	7.31E-23	NK.Tim3	SQSTM1
FERMT3	3.04E-27	0.29381	0.341	0.246	8.55E-23	NK.Tim3	FERMT3
RAB2A1	3.19E-27	0.287769	0.439	0.338	8.99E-23	NK.Tim3	RAB2A
LNPEP1	8.15E-27	0.252603	0.364	0.263	2.29E-22	NK.Tim3	LNPEP
PHB	1.00E-26	0.306271	0.276	0.189	2.82E-22	NK.Tim3	PHB
CNOT2	1.56E-26	0.277051	0.437	0.335	4.39E-22	NK.Tim3	CNOT2
RNF167	1.98E-26	0.288145	0.363	0.265	5.57E-22	NK.Tim3	RNF167
LAMTOR11	2.88E-26	0.277868	0.323	0.229	8.09E-22	NK.Tim3	LAMTOR1
VAMP21	2.92E-26	0.251731	0.6	0.493	8.22E-22	NK.Tim3	VAMP2
PSMA4	3.17E-26	0.26901	0.26	0.174	8.93E-22	NK.Tim3	PSMA4
NDUFV1	3.97E-26	0.298156	0.343	0.249	1.12E-21	NK.Tim3	NDUFV1
PRKACB1	6.57E-26	0.28103	0.345	0.25	1.85E-21	NK.Tim3	PRKACB
TPM4	7.86E-26	0.339809	0.324	0.234	2.21E-21	NK.Tim3	TPM4
COPZ1	9.35E-26	0.294045	0.251	0.168	2.63E-21	NK.Tim3	COPZ1

C17orf49	1.05E-25	0.313681	0.278	0.192	2.96E-21	NK.Tim3	C17orf49
VAMP81	1.55E-25	0.250375	0.482	0.38	4.37E-21	NK.Tim3	VAMP8
IMP3	2.56E-25	0.296257	0.271	0.186	7.22E-21	NK.Tim3	IMP3
UBE2M	2.69E-25	0.301916	0.28	0.194	7.56E-21	NK.Tim3	UBE2M
OTUB1	1.28E-24	0.261813	0.363	0.271	3.61E-20	NK.Tim3	OTUB1
SELL1	2.45E-24	0.271626	0.271	0.183	6.89E-20	NK.Tim3	SELL
SSNA1	6.71E-24	0.252646	0.303	0.216	1.89E-19	NK.Tim3	SSNA1
MRPL101	7.24E-24	0.286649	0.271	0.187	2.04E-19	NK.Tim3	MRPL10
SIGIRR	7.89E-24	0.294105	0.379	0.288	2.22E-19	NK.Tim3	SIGIRR
PPP4C	7.91E-24	0.280002	0.32	0.233	2.23E-19	NK.Tim3	PPP4C
SRSF9	8.86E-24	0.285167	0.454	0.364	2.49E-19	NK.Tim3	SRSF9
MAP1LC3B	1.06E-23	0.277719	0.49	0.389	2.99E-19	NK.Tim3	MAP1LC3B
PSMB1	1.20E-23	0.267217	0.465	0.37	3.37E-19	NK.Tim3	PSMB1
ID21	1.60E-23	0.276171	0.648	0.548	4.50E-19	NK.Tim3	ID2
TAF10	3.27E-23	0.280415	0.501	0.416	9.21E-19	NK.Tim3	TAF10
SERINC3	5.43E-23	0.25571	0.318	0.23	1.53E-18	NK.Tim3	SERINC3
SLC25A51	1.33E-22	0.27093	0.555	0.457	3.73E-18	NK.Tim3	SLC25A5
COPE	1.38E-22	0.250031	0.479	0.382	3.88E-18	NK.Tim3	COPE
HNRNPF	1.72E-22	0.26549	0.492	0.406	4.85E-18	NK.Tim3	HNRNPF
CRY1	1.79E-22	0.265432	0.25	0.172	5.05E-18	NK.Tim3	CRY1
MAZ	2.93E-22	0.264588	0.331	0.244	8.24E-18	NK.Tim3	MAZ
UFC1	7.73E-22	0.283593	0.394	0.309	2.17E-17	NK.Tim3	UFC1
BLOC1S1	1.13E-21	0.274167	0.375	0.288	3.18E-17	NK.Tim3	BLOC1S1
MLLT6	1.56E-21	0.250704	0.254	0.176	4.39E-17	NK.Tim3	MLLT6
MYLIP	2.42E-21	0.298537	0.386	0.297	6.82E-17	NK.Tim3	MYLIP
ECH1	2.68E-21	0.254983	0.398	0.309	7.55E-17	NK.Tim3	ECH1
RNF181	2.85E-21	0.251562	0.264	0.186	8.02E-17	NK.Tim3	RNF181
NUCB1	2.04E-20	0.281428	0.321	0.24	5.74E-16	NK.Tim3	NUCB1
MOB1A	5.00E-20	0.253429	0.337	0.256	1.41E-15	NK.Tim3	MOB1A
TMED9	1.84E-19	0.252575	0.334	0.253	5.18E-15	NK.Tim3	TMED9
PPP2R1A	1.10E-18	0.266025	0.351	0.272	3.10E-14	NK.Tim3	PPP2R1A
UBE2V1	1.43E-18	0.259174	0.377	0.297	4.01E-14	NK.Tim3	UBE2V1
DNAJC8	7.29E-18	0.255867	0.324	0.247	2.05E-13	NK.Tim3	DNAJC8
TMEM179I	3.08E-16	0.250893	0.258	0.191	8.67E-12	NK.Tim3	TMEM179B
NEU11	9.04E-16	0.262089	0.27	0.202	2.54E-11	NK.Tim3	NEU1
DDIT3	1.59E-13	0.268025	0.263	0.202	4.46E-09	NK.Tim3	DDIT3
AREG2	0	2.977898	0.724	0.208	0	NK.XCL1/2	AREG
IGFBP2	0	2.347116	0.46	0.055	0	NK.XCL1/2	IGFBP2
XCL21	0	2.21239	0.516	0.143	0	NK.XCL1/2	XCL2
KRT86	0	2.063174	0.407	0.033	0	NK.XCL1/2	KRT86
KLRC1	0	2.038953	0.517	0.092	0	NK.XCL1/2	KLRC1
XCL12	0	1.994706	0.554	0.179	0	NK.XCL1/2	XCL1
MCTP21	0	1.876846	0.557	0.156	0	NK.XCL1/2	MCTP2
TYROBP2	0	1.747508	0.782	0.289	0	NK.XCL1/2	TYROBP
KLRD11	0	1.701738	0.758	0.263	0	NK.XCL1/2	KLRD1
TXK2	0	1.698287	0.536	0.144	0	NK.XCL1/2	TXK
NCAM1	0	1.647853	0.325	0.02	0	NK.XCL1/2	NCAM1

CD72	0	1.607705	0.797	0.507	0	NK.XCL1/2	CD7
KRT81	0	1.48513	0.282	0.008	0	NK.XCL1/2	KRT81
KLRC21	0	1.483056	0.389	0.078	0	NK.XCL1/2	KLRC2
TMIGD2	0	1.413983	0.388	0.068	0	NK.XCL1/2	TMIGD2
TRDC	0	1.333221	0.32	0.043	0	NK.XCL1/2	TRDC
SH2D1B1	0	1.317306	0.31	0.031	0	NK.XCL1/2	SH2D1B
KIR2DL4	0	1.309062	0.266	0.017	0	NK.XCL1/2	KIR2DL4
FCER1G2	0	1.275426	0.554	0.167	0	NK.XCL1/2	FCER1G
SPRY2	0	1.228229	0.284	0.052	0	NK.XCL1/2	SPRY2
KLRF11	0	1.198483	0.308	0.043	0	NK.XCL1/2	KLRF1
CLIC31	1.00E-294	1.558832	0.529	0.187	2.82E-290	NK.XCL1/2	CLIC3
CMC11	4.16E-261	1.748746	0.509	0.202	1.17E-256	NK.XCL1/2	CMC1
IL2RB1	4.81E-257	1.352937	0.564	0.239	1.35E-252	NK.XCL1/2	IL2RB
NKG72	5.14E-254	1.15122	0.828	0.517	1.45E-249	NK.XCL1/2	NKG7
CTSW2	3.30E-252	1.310083	0.773	0.493	9.28E-248	NK.XCL1/2	CTSW
ATP8B41	6.11E-252	1.22646	0.291	0.067	1.72E-247	NK.XCL1/2	ATP8B4
PLCG21	7.14E-232	1.178722	0.345	0.097	2.01E-227	NK.XCL1/2	PLCG2
GNLY1	8.42E-228	1.860373	0.548	0.223	2.37E-223	NK.XCL1/2	GNLY
AOAH2	3.61E-185	1.20516	0.691	0.39	1.01E-180	NK.XCL1/2	AOAH
SYK1	5.26E-181	0.971436	0.258	0.069	1.48E-176	NK.XCL1/2	SYK
CD381	1.97E-171	1.145635	0.378	0.14	5.54E-167	NK.XCL1/2	CD38
MAFF1	2.23E-171	1.228726	0.431	0.182	6.28E-167	NK.XCL1/2	MAFF
GFOD11	3.95E-158	1.084082	0.339	0.119	1.11E-153	NK.XCL1/2	GFOD1
MAPK12	3.13E-155	1.11449	0.563	0.307	8.79E-151	NK.XCL1/2	MAPK1
MAP3K82	5.64E-154	1.172068	0.582	0.326	1.59E-149	NK.XCL1/2	MAP3K8
MATK1	5.58E-148	1.068704	0.502	0.257	1.57E-143	NK.XCL1/2	MATK
KLRB12	3.71E-134	0.823131	0.636	0.346	1.04E-129	NK.XCL1/2	KLRB1
C1orf211	4.60E-131	1.011026	0.256	0.083	1.29E-126	NK.XCL1/2	C1orf21
KLRK12	1.68E-116	0.846785	0.592	0.35	4.74E-112	NK.XCL1/2	KLRK1
HIP1	1.24E-115	0.895878	0.251	0.086	3.48E-111	NK.XCL1/2	HIP1
METRNL2	1.56E-113	0.969288	0.429	0.212	4.39E-109	NK.XCL1/2	METRNL
ZBTB161	3.53E-111	1.189679	0.434	0.221	9.92E-107	NK.XCL1/2	ZBTB16
LYN2	1.33E-109	0.890015	0.319	0.131	3.74E-105	NK.XCL1/2	LYN
HIPK21	2.88E-109	1.135434	0.298	0.12	8.10E-105	NK.XCL1/2	HIPK2
CD2472	2.74E-103	0.948436	0.722	0.556	7.71E-99	NK.XCL1/2	CD247
RIN32	6.73E-101	1.043488	0.355	0.167	1.89E-96	NK.XCL1/2	RIN3
PRF11	9.79E-92	0.684874	0.566	0.344	2.75E-87	NK.XCL1/2	PRF1
YES11	8.78E-88	0.93831	0.314	0.143	2.47E-83	NK.XCL1/2	YES1
IFITM22	5.53E-87	0.876571	0.712	0.631	1.55E-82	NK.XCL1/2	IFITM2
ITM2C1	2.35E-86	1.579311	0.426	0.262	6.62E-82	NK.XCL1/2	ITM2C
APBA21	6.09E-86	0.976947	0.364	0.183	1.71E-81	NK.XCL1/2	APBA2
HSH2D1	7.53E-85	0.792011	0.278	0.12	2.12E-80	NK.XCL1/2	HSH2D
GSTP11	3.57E-84	0.928949	0.54	0.372	1.00E-79	NK.XCL1/2	GSTP1
CLDND1	2.26E-80	0.837741	0.417	0.239	6.35E-76	NK.XCL1/2	CLDND1
ITGAE	1.16E-77	0.942597	0.353	0.189	3.27E-73	NK.XCL1/2	ITGAE
EPAS1	5.36E-77	0.87689	0.281	0.128	1.51E-72	NK.XCL1/2	EPAS1
PTGDR1	6.78E-76	0.705369	0.32	0.153	1.91E-71	NK.XCL1/2	PTGDR

HOPX1	7.24E-73	0.839036	0.454	0.281	2.04E-68	NK.XCL1/2	HOPX
FOSL22	1.78E-71	0.832856	0.474	0.305	5.00E-67	NK.XCL1/2	FOSL2
IFITM13	4.68E-71	0.692534	0.803	0.778	1.32E-66	NK.XCL1/2	IFITM1
CD632	2.31E-69	0.744702	0.46	0.289	6.49E-65	NK.XCL1/2	CD63
ARHGAP91	6.35E-65	0.728449	0.543	0.394	1.79E-60	NK.XCL1/2	ARHGAP9
DUSP22	4.35E-61	0.671174	0.78	0.736	1.22E-56	NK.XCL1/2	DUSP2
NR4A22	1.26E-57	0.787582	0.729	0.658	3.53E-53	NK.XCL1/2	NR4A2
RASA21	1.40E-55	0.773405	0.55	0.406	3.93E-51	NK.XCL1/2	RASA2
VAV33	1.57E-54	0.873415	0.364	0.215	4.43E-50	NK.XCL1/2	VAV3
TCIRG11	1.91E-54	0.693527	0.355	0.213	5.39E-50	NK.XCL1/2	TCIRG1
SRGN1	6.16E-54	0.543881	0.88	0.867	1.73E-49	NK.XCL1/2	SRGN
VPS37B1	2.70E-53	0.835357	0.503	0.363	7.58E-49	NK.XCL1/2	VPS37B
GRASP2	3.10E-53	0.774716	0.256	0.13	8.73E-49	NK.XCL1/2	GRASP
CLASP12	3.44E-52	0.786967	0.325	0.187	9.68E-48	NK.XCL1/2	CLASP1
ABCB1	2.59E-50	0.65791	0.275	0.148	7.30E-46	NK.XCL1/2	ABCB1
GNPTAB1	8.62E-50	0.756729	0.348	0.211	2.42E-45	NK.XCL1/2	GNPTAB
UBASH3B	4.63E-49	0.910621	0.266	0.142	1.30E-44	NK.XCL1/2	UBASH3B
CNOT21	8.77E-48	0.809718	0.466	0.336	2.47E-43	NK.XCL1/2	CNOT2
ERGIC11	2.20E-47	0.609696	0.273	0.151	6.19E-43	NK.XCL1/2	ERGIC1
CCL43	3.30E-44	0.593087	0.806	0.706	9.29E-40	NK.XCL1/2	CCL4
IFITM32	1.47E-43	0.608649	0.36	0.225	4.13E-39	NK.XCL1/2	IFITM3
CEBPD2	1.84E-43	0.511827	0.464	0.311	5.18E-39	NK.XCL1/2	CEBPD
CEMIP21	5.25E-43	0.666589	0.649	0.554	1.48E-38	NK.XCL1/2	CEMIP2
AUTS21	9.27E-42	0.69993	0.537	0.416	2.61E-37	NK.XCL1/2	AUTS2
SLA21	4.80E-40	0.658981	0.315	0.193	1.35E-35	NK.XCL1/2	SLA2
PLCB1	7.24E-39	0.780709	0.252	0.142	2.04E-34	NK.XCL1/2	PLCB1
REL2	1.23E-38	0.63247	0.577	0.468	3.45E-34	NK.XCL1/2	REL
ZEB23	9.16E-38	0.707565	0.434	0.304	2.58E-33	NK.XCL1/2	ZEB2
PLCL22	2.42E-37	0.686147	0.37	0.252	6.80E-33	NK.XCL1/2	PLCL2
PIK3R11	3.05E-36	0.629206	0.6	0.501	8.58E-32	NK.XCL1/2	PIK3R1
TGFB12	4.91E-35	0.517741	0.694	0.624	1.38E-30	NK.XCL1/2	TGFB1
TBCD	1.15E-34	0.602293	0.325	0.214	3.22E-30	NK.XCL1/2	TBCD
RASSF11	1.45E-34	0.512104	0.345	0.23	4.08E-30	NK.XCL1/2	RASSF1
CST72	2.14E-33	0.351	0.715	0.588	6.02E-29	NK.XCL1/2	CST7
TOX1	3.33E-33	0.377702	0.427	0.299	9.37E-29	NK.XCL1/2	TOX
SYTL31	8.24E-33	0.46558	0.704	0.63	2.32E-28	NK.XCL1/2	SYTL3
IER22	3.50E-32	0.465798	0.742	0.693	9.86E-28	NK.XCL1/2	IER2
IFRD11	1.96E-30	0.699379	0.453	0.362	5.51E-26	NK.XCL1/2	IFRD1
GZMB1	1.10E-29	0.376004	0.287	0.171	3.10E-25	NK.XCL1/2	GZMB
PHYKPL	1.43E-29	0.55411	0.308	0.208	4.02E-25	NK.XCL1/2	PHYKPL
CHST121	6.05E-29	0.503939	0.348	0.24	1.70E-24	NK.XCL1/2	CHST12
CTSD2	2.13E-28	0.481241	0.541	0.456	6.00E-24	NK.XCL1/2	CTSD
CCND2	2.17E-27	0.510968	0.295	0.198	6.10E-23	NK.XCL1/2	CCND2
NCALD1	8.12E-27	0.805241	0.268	0.175	2.29E-22	NK.XCL1/2	NCALD
APOBEC3G	2.81E-26	0.506402	0.393	0.292	7.91E-22	NK.XCL1/2	APOBEC3G
LITAF1	4.42E-26	0.498954	0.584	0.515	1.24E-21	NK.XCL1/2	LITAF
TIPARP1	1.36E-25	0.602638	0.353	0.26	3.82E-21	NK.XCL1/2	TIPARP

NR4A11	6.78E-25	0.370387	0.384	0.275	1.91E-20	NK.XCL1/2	NR4A1
UBB2	1.73E-24	0.416978	0.722	0.691	4.87E-20	NK.XCL1/2	UBB
DDIT41	1.77E-24	0.529928	0.702	0.685	4.99E-20	NK.XCL1/2	DDIT4
JAK12	1.83E-24	0.445059	0.647	0.593	5.16E-20	NK.XCL1/2	JAK1
PLEKHA21	2.30E-24	0.581789	0.355	0.259	6.47E-20	NK.XCL1/2	PLEKHA2
GLIPR21	6.96E-24	0.424331	0.28	0.191	1.96E-19	NK.XCL1/2	GLIPR2
BST22	1.36E-23	0.457898	0.442	0.354	3.82E-19	NK.XCL1/2	BST2
RALGAPA1	2.15E-23	0.530368	0.421	0.335	6.06E-19	NK.XCL1/2	RALGAPA1
RANBP21	2.31E-23	0.593386	0.498	0.42	6.49E-19	NK.XCL1/2	RANBP2
GPATCH82	3.36E-23	0.606951	0.353	0.263	9.45E-19	NK.XCL1/2	GPATCH8
RAB8B2	6.70E-23	0.585798	0.354	0.266	1.89E-18	NK.XCL1/2	RAB8B
TPST21	8.02E-23	0.464914	0.273	0.186	2.26E-18	NK.XCL1/2	TPST2
SFMBT22	2.03E-22	0.523187	0.393	0.298	5.72E-18	NK.XCL1/2	SFMBT2
APMAP2	1.32E-21	0.453673	0.396	0.306	3.73E-17	NK.XCL1/2	APMAP
ZFP362	3.36E-21	0.397888	0.856	0.879	9.44E-17	NK.XCL1/2	ZFP36
RBM382	1.63E-20	0.495369	0.465	0.392	4.60E-16	NK.XCL1/2	RBM38
ADGRE52	4.21E-20	0.43009	0.579	0.519	1.19E-15	NK.XCL1/2	ADGRE5
JUND1	5.08E-20	0.482173	0.791	0.813	1.43E-15	NK.XCL1/2	JUND
NFKB12	1.27E-19	0.489469	0.49	0.408	3.58E-15	NK.XCL1/2	NFKB1
PRKX1	2.38E-19	0.532374	0.308	0.227	6.68E-15	NK.XCL1/2	PRKX
PTPN221	3.39E-19	0.43127	0.44	0.359	9.54E-15	NK.XCL1/2	PTPN22
EIF3G1	1.12E-18	0.503419	0.547	0.521	3.15E-14	NK.XCL1/2	EIF3G
AGO21	3.31E-18	0.475542	0.332	0.254	9.32E-14	NK.XCL1/2	AGO2
XAF1	8.87E-18	0.501313	0.296	0.219	2.50E-13	NK.XCL1/2	XAF1
IRF7	1.47E-17	0.450237	0.281	0.206	4.15E-13	NK.XCL1/2	IRF7
ZNF3311	3.40E-17	0.40039	0.665	0.62	9.56E-13	NK.XCL1/2	ZNF331
SCML41	3.45E-17	0.454448	0.46	0.391	9.70E-13	NK.XCL1/2	SCML4
TPI11	6.51E-17	0.393394	0.547	0.497	1.83E-12	NK.XCL1/2	TPI1
PDE7A1	6.90E-17	0.400012	0.493	0.426	1.94E-12	NK.XCL1/2	PDE7A
GADD45B1	2.06E-16	0.413728	0.647	0.594	5.78E-12	NK.XCL1/2	GADD45B
UTRN2	8.37E-16	0.54731	0.509	0.452	2.35E-11	NK.XCL1/2	UTRN
HMGN32	1.36E-15	0.376885	0.314	0.244	3.83E-11	NK.XCL1/2	HMGN3
PITPNC11	1.78E-15	0.47329	0.574	0.521	5.01E-11	NK.XCL1/2	PITPNC1
NCL	2.45E-15	0.30677	0.638	0.61	6.91E-11	NK.XCL1/2	NCL
ALOX5AP1	3.25E-15	0.402426	0.451	0.383	9.13E-11	NK.XCL1/2	ALOX5AP
RBM392	3.89E-15	0.358776	0.676	0.648	1.10E-10	NK.XCL1/2	RBM39
ELL22	6.60E-15	0.405524	0.487	0.415	1.86E-10	NK.XCL1/2	ELL2
NR4A32	6.67E-15	0.519994	0.38	0.309	1.88E-10	NK.XCL1/2	NR4A3
FAM49B2	1.21E-14	0.458047	0.492	0.433	3.41E-10	NK.XCL1/2	FAM49B
CMIP1	2.26E-14	0.610153	0.316	0.25	6.35E-10	NK.XCL1/2	CMIP
PIP4K2A1	3.03E-14	0.424266	0.587	0.534	8.51E-10	NK.XCL1/2	PIP4K2A
PREX11	3.45E-14	0.511944	0.469	0.415	9.71E-10	NK.XCL1/2	PREX1
ACTN42	3.68E-14	0.41258	0.367	0.301	1.03E-09	NK.XCL1/2	ACTN4
JARID21	9.02E-14	0.463697	0.315	0.249	2.54E-09	NK.XCL1/2	JARID2
MBP2	9.99E-14	0.435596	0.438	0.376	2.81E-09	NK.XCL1/2	MBP
EVL	1.18E-13	0.278472	0.708	0.703	3.32E-09	NK.XCL1/2	EVL
FAM177A1	1.39E-13	0.452711	0.465	0.418	3.90E-09	NK.XCL1/2	FAM177A1

PRDX51	4.55E-13	0.345607	0.343	0.28	1.28E-08	NK.XCL1/2	PRDX5
SOCS1	4.99E-13	0.385425	0.475	0.426	1.40E-08	NK.XCL1/2	SOCS1
HELZ1	5.12E-13	0.456853	0.314	0.251	1.44E-08	NK.XCL1/2	HELZ
DOK21	6.17E-13	0.375119	0.31	0.247	1.74E-08	NK.XCL1/2	DOK2
CDK171	1.03E-12	0.457366	0.46	0.401	2.90E-08	NK.XCL1/2	CDK17
SQSTM12	1.22E-12	0.404517	0.479	0.422	3.42E-08	NK.XCL1/2	SQSTM1
ADD11	1.47E-12	0.43626	0.303	0.244	4.13E-08	NK.XCL1/2	ADD1
SLC38A11	1.60E-12	0.413022	0.523	0.474	4.51E-08	NK.XCL1/2	SLC38A1
H2AFX1	1.85E-12	0.421148	0.359	0.3	5.20E-08	NK.XCL1/2	H2AFX
LSP12	2.99E-12	0.2761	0.632	0.618	8.41E-08	NK.XCL1/2	LSP1
FYN1	3.47E-12	0.260675	0.785	0.762	9.77E-08	NK.XCL1/2	FYN
SKIL1	5.99E-12	0.406359	0.397	0.341	1.69E-07	NK.XCL1/2	SKIL
LCP12	1.26E-11	0.322523	0.647	0.637	3.53E-07	NK.XCL1/2	LCP1
CD961	1.26E-11	0.316757	0.628	0.591	3.55E-07	NK.XCL1/2	CD96
SRSF21	1.39E-11	0.389992	0.583	0.569	3.91E-07	NK.XCL1/2	SRSF2
GABARAPL	1.81E-11	0.355083	0.303	0.243	5.08E-07	NK.XCL1/2	GABARAPL1
DDX171	1.85E-11	0.334846	0.47	0.419	5.21E-07	NK.XCL1/2	DDX17
STK17A1	2.14E-11	0.265656	0.641	0.621	6.01E-07	NK.XCL1/2	STK17A
MAT2A1	3.43E-11	0.400312	0.339	0.284	9.66E-07	NK.XCL1/2	MAT2A
SLA1	3.75E-11	0.450957	0.456	0.412	1.05E-06	NK.XCL1/2	SLA
HSPA51	6.79E-11	0.402364	0.597	0.579	1.91E-06	NK.XCL1/2	HSPA5
SMYD31	9.20E-11	0.412609	0.301	0.244	2.59E-06	NK.XCL1/2	SMYD3
SMARCA21	9.46E-11	0.388767	0.416	0.37	2.66E-06	NK.XCL1/2	SMARCA2
CDC42SE11	1.09E-10	0.31919	0.414	0.363	3.08E-06	NK.XCL1/2	CDC42SE1
LY6E2	1.21E-10	0.338349	0.579	0.555	3.40E-06	NK.XCL1/2	LY6E
MACF11	1.33E-10	0.409425	0.488	0.45	3.75E-06	NK.XCL1/2	MACF1
TRAPPC102	1.42E-10	0.391009	0.325	0.271	3.99E-06	NK.XCL1/2	TRAPPC10
SNTB2	1.47E-10	0.285701	0.26	0.2	4.12E-06	NK.XCL1/2	SNTB2
CBLB1	2.89E-10	0.302497	0.63	0.593	8.13E-06	NK.XCL1/2	CBLB
SEPTIN71	3.57E-10	0.289314	0.548	0.51	1.00E-05	NK.XCL1/2	SEPTIN7
MT-ND41	3.59E-10	0.30303	0.802	0.808	1.01E-05	NK.XCL1/2	MT-ND4
UBE2S1	4.00E-10	0.407802	0.498	0.467	1.12E-05	NK.XCL1/2	UBE2S
PPM1B	9.73E-10	0.379408	0.28	0.228	2.74E-05	NK.XCL1/2	PPM1B
AKNA2	1.17E-09	0.394754	0.531	0.496	3.28E-05	NK.XCL1/2	AKNA
CFLAR2	1.21E-09	0.32441	0.444	0.4	3.39E-05	NK.XCL1/2	CFLAR
KAT6B	1.61E-09	0.297408	0.275	0.221	4.54E-05	NK.XCL1/2	KAT6B
CARD112	2.02E-09	0.71961	0.295	0.249	5.69E-05	NK.XCL1/2	CARD11
GLCCI11	2.11E-09	0.298716	0.373	0.315	5.92E-05	NK.XCL1/2	GLCCI1
PYHIN12	7.13E-09	0.417838	0.33	0.28	0.000201	NK.XCL1/2	PYHIN1
BPTF1	8.08E-09	0.340513	0.426	0.386	0.000227	NK.XCL1/2	BPTF
PRKCH1	1.93E-08	0.373158	0.673	0.661	0.000544	NK.XCL1/2	PRKCH
NDUFS8	4.60E-08	0.307039	0.284	0.239	0.001295	NK.XCL1/2	NDUFS8
PGK1	7.63E-08	0.336953	0.551	0.542	0.002146	NK.XCL1/2	PGK1
BZW11	1.25E-07	0.287957	0.423	0.398	0.003512	NK.XCL1/2	BZW1
RHOC1	1.59E-07	0.262853	0.322	0.275	0.004475	NK.XCL1/2	RHOC
GPR652	1.75E-07	0.358635	0.284	0.244	0.004918	NK.XCL1/2	GPR65
BRD21	2.53E-07	0.306273	0.556	0.539	0.007119	NK.XCL1/2	BRD2

MX11	2.61E-07	0.352708	0.275	0.234	0.007342	NK.XCL1/2	MX1
PRKACB2	2.86E-07	0.286485	0.299	0.254	0.008038	NK.XCL1/2	PRKACB
HSP90AB1:	2.86E-07	0.320021	0.726	0.728	0.00806	NK.XCL1/2	HSP90AB1
PSTPIP1	3.32E-07	0.29269	0.253	0.211	0.009345	NK.XCL1/2	PSTPIP1
RAP1GDS1	4.87E-07	0.261256	0.291	0.243	0.013703	NK.XCL1/2	RAP1GDS1
NDUFV11	6.59E-07	0.269617	0.296	0.253	0.01855	NK.XCL1/2	NDUFV1
SERTAD11	7.47E-07	0.321843	0.393	0.359	0.021021	NK.XCL1/2	SERTAD1
CASK1	7.60E-07	0.289872	0.314	0.268	0.02138	NK.XCL1/2	CASK
ATP8A11	7.87E-07	0.262436	0.326	0.279	0.022143	NK.XCL1/2	ATP8A1
IFI161	9.06E-07	0.318391	0.42	0.395	0.025486	NK.XCL1/2	IFI16
MTRNR2L1	9.08E-07	0.291113	0.59	0.537	0.02555	NK.XCL1/2	MTRNR2L12
GRK2	9.82E-07	0.297236	0.332	0.295	0.027622	NK.XCL1/2	GRK2
BRAF1	1.39E-06	0.337101	0.265	0.227	0.039083	NK.XCL1/2	BRAF
MIDN1	1.40E-06	0.299061	0.309	0.272	0.039392	NK.XCL1/2	MIDN
BIN21	3.09E-06	0.268184	0.421	0.389	0.08698	NK.XCL1/2	BIN2
SYNE12	3.37E-06	0.532822	0.33	0.302	0.094734	NK.XCL1/2	SYNE1
IRF11	3.76E-06	0.297858	0.464	0.438	0.105878	NK.XCL1/2	IRF1
PTK2B1	4.30E-06	0.30441	0.296	0.259	0.12103	NK.XCL1/2	PTK2B
DDX3X	5.57E-06	0.300794	0.466	0.442	0.156807	NK.XCL1/2	DDX3X
RC3H11	5.75E-06	0.31429	0.261	0.225	0.161902	NK.XCL1/2	RC3H1
CD553	7.95E-06	0.343872	0.429	0.407	0.223687	NK.XCL1/2	CD55
RFTN11	8.38E-06	0.306247	0.309	0.272	0.235719	NK.XCL1/2	RFTN1
OSTF11	1.08E-05	0.250312	0.381	0.359	0.30296	NK.XCL1/2	OSTF1
DNAJA1	1.12E-05	0.322411	0.561	0.559	0.31442	NK.XCL1/2	DNAJA1
INPP5D1	1.17E-05	0.362413	0.417	0.391	0.329046	NK.XCL1/2	INPP5D
PPP3CA1	1.23E-05	0.355539	0.267	0.232	0.34477	NK.XCL1/2	PPP3CA
RNF1251	2.10E-05	0.255637	0.46	0.435	0.591143	NK.XCL1/2	RNF125
CTNNB11	2.27E-05	0.254816	0.291	0.255	0.639934	NK.XCL1/2	CTNNB1
YPEL52	2.52E-05	0.292287	0.525	0.522	0.710262	NK.XCL1/2	YPEL5
C5orf562	4.29E-05	0.314806	0.272	0.241	1	NK.XCL1/2	C5orf56
DNM2	6.04E-05	0.325876	0.318	0.291	1	NK.XCL1/2	DNM2
STAT41	8.16E-05	0.276343	0.537	0.526	1	NK.XCL1/2	STAT4
SIPA11	8.34E-05	0.252324	0.272	0.244	1	NK.XCL1/2	SIPA1
SSH22	8.36E-05	0.421541	0.429	0.411	1	NK.XCL1/2	SSH2
IQGAP22	8.41E-05	0.304256	0.477	0.458	1	NK.XCL1/2	IQGAP2
TSPAN51	0.000143	0.259022	0.267	0.234	1	NK.XCL1/2	TSPAN5
DIP2A2	0.000144	0.364405	0.307	0.281	1	NK.XCL1/2	DIP2A
SKI1	0.000173	0.304931	0.261	0.231	1	NK.XCL1/2	SKI
ATP1B31	0.000196	0.37694	0.434	0.419	1	NK.XCL1/2	ATP1B3
RABGAP1L:	0.000234	0.31536	0.524	0.513	1	NK.XCL1/2	RABGAP1L
DNAJB61	0.000262	0.332858	0.418	0.409	1	NK.XCL1/2	DNAJB6
KLF132	0.000269	0.307285	0.37	0.352	1	NK.XCL1/2	KLF13
LPP1	0.000336	0.320951	0.362	0.341	1	NK.XCL1/2	LPP
SYAP1	0.000358	0.276205	0.327	0.302	1	NK.XCL1/2	SYAP1
CCL52	0.000363	0.33263	0.696	0.734	1	NK.XCL1/2	CCL5
CDK13	0.000641	0.283212	0.27	0.246	1	NK.XCL1/2	CDK13
TNRC6B1	0.000693	0.350635	0.38	0.366	1	NK.XCL1/2	TNRC6B

LDHA1	0.00071	0.31005	0.526	0.544	1	NK.XCL1/2	LDHA
RASGEF1B1	0.000807	0.379164	0.285	0.261	1	NK.XCL1/2	RASGEF1B
CALR1	0.000977	0.256683	0.512	0.517	1	NK.XCL1/2	CALR
FGD3	0.001	0.255653	0.265	0.241	1	NK.XCL1/2	FGD3
ARF4	0.001081	0.267384	0.342	0.326	1	NK.XCL1/2	ARF4
H2AFZ1	0.001297	0.270572	0.474	0.47	1	NK.XCL1/2	H2AFZ
MAPRE21	0.003009	0.316638	0.363	0.346	1	NK.XCL1/2	MAPRE2
CCND32	0.003509	0.26079	0.627	0.641	1	NK.XCL1/2	CCND3
ATP11B1	0.004095	0.280071	0.263	0.242	1	NK.XCL1/2	ATP11B
VPS13B1	0.005728	0.289892	0.263	0.246	1	NK.XCL1/2	VPS13B
HSPE11	0.008334	0.430581	0.437	0.444	1	NK.XCL1/2	HSPE1
RNF115	0.008997	0.25825	0.271	0.256	1	NK.XCL1/2	RNF115
TRDV2	0	3.362272	0.874	0.012	0	gdT	TRDV2
TRGV9	0	2.591139	0.837	0.058	0	gdT	TRGV9
KLRC11	0	1.658873	0.625	0.089	0	gdT	KLRC1
TRDC1	0	1.416262	0.47	0.037	0	gdT	TRDC
KLRB13	0	1.4128	0.845	0.338	0	gdT	KLRB1
KLRG1	0	1.260514	0.735	0.215	0	gdT	KLRG1
KLRD12	0	1.242391	0.817	0.263	0	gdT	KLRD1
NKG73	0	1.148202	0.959	0.513	0	gdT	NKG7
IGFBP21	0	1.145998	0.356	0.062	0	gdT	IGFBP2
HOPX2	0	1.080082	0.718	0.27	0	gdT	HOPX
CD160	0	0.945031	0.308	0.044	0	gdT	CD160
ZBTB162	0	0.887353	0.643	0.212	0	gdT	ZBTB16
IL12RB2	0	0.804624	0.352	0.071	0	gdT	IL12RB2
NCR31	3.19E-262	0.763815	0.366	0.087	8.97E-258	gdT	NCR3
CTSW3	1.57E-245	0.863987	0.907	0.488	4.41E-241	gdT	CTSW
CST73	9.61E-227	0.825691	0.937	0.579	2.70E-222	gdT	CST7
DUSP23	2.08E-225	0.998662	0.942	0.729	5.86E-221	gdT	DUSP2
HPGD	5.09E-223	0.60616	0.261	0.054	1.43E-218	gdT	HPGD
GZMA3	2.90E-177	0.741673	0.908	0.544	8.17E-173	gdT	GZMA
MATK2	5.13E-172	0.692634	0.595	0.254	1.44E-167	gdT	MATK
CCL53	8.98E-171	0.644821	0.991	0.721	2.53E-166	gdT	CCL5
IL322	9.20E-168	0.708547	0.967	0.756	2.59E-163	gdT	IL32
GZMM2	1.22E-160	0.640892	0.747	0.382	3.43E-156	gdT	GZMM
KLRK13	1.15E-155	0.667581	0.716	0.346	3.25E-151	gdT	KLRK1
XCL13	1.92E-154	1.060754	0.458	0.185	5.41E-150	gdT	XCL1
TRBC11	5.59E-154	0.738063	0.647	0.313	1.57E-149	gdT	TRBC1
TRAC	2.00E-143	0.616148	0.493	0.205	5.62E-139	gdT	TRAC
GZMK2	1.12E-142	0.743831	0.786	0.444	3.16E-138	gdT	GZMK
LINC01871	1.40E-142	0.633725	0.471	0.188	3.93E-138	gdT	LINC01871
TBX211	4.44E-137	0.600482	0.507	0.216	1.25E-132	gdT	TBX21
RPL411	2.97E-136	0.642419	0.983	0.89	8.34E-132	gdT	RPL41
LAG31	1.47E-132	0.567671	0.409	0.157	4.14E-128	gdT	LAG3
PRF12	8.19E-132	0.48194	0.682	0.34	2.31E-127	gdT	PRF1
CCL44	1.82E-130	0.788313	0.927	0.701	5.12E-126	gdT	CCL4
B3GNT2	1.54E-122	0.5399	0.405	0.162	4.33E-118	gdT	B3GNT2

HLA-C1	1.44E-121	0.426092	0.993	0.909	4.05E-117	gdT	HLA-C
RPS273	2.16E-120	0.43729	0.997	0.935	6.08E-116	gdT	RPS27
HLA-B2	6.32E-119	0.388248	0.997	0.956	1.78E-114	gdT	HLA-B
MT2A2	5.05E-114	0.92187	0.75	0.478	1.42E-109	gdT	MT2A
APOBEC3G	4.14E-112	0.571317	0.568	0.285	1.17E-107	gdT	APOBEC3G
RPL212	1.41E-111	0.516316	0.957	0.808	3.96E-107	gdT	RPL21
GABARAPL	3.73E-111	0.547471	0.5	0.235	1.05E-106	gdT	GABARAPL1
EEF1A12	1.31E-109	0.385855	0.997	0.948	3.68E-105	gdT	EEF1A1
RPL37A1	1.98E-108	0.501776	0.957	0.799	5.57E-104	gdT	RPL37A
CD3E2	8.29E-108	0.498203	0.955	0.736	2.33E-103	gdT	CD3E
RPS242	1.19E-107	0.401613	0.996	0.912	3.34E-103	gdT	RPS24
RPLP11	2.15E-105	0.366184	0.999	0.946	6.06E-101	gdT	RPLP1
RPL23A2	9.52E-104	0.446397	0.979	0.857	2.68E-99	gdT	RPL23A
HCST3	2.70E-103	0.510391	0.931	0.699	7.58E-99	gdT	HCST
MAZ1	1.47E-96	0.499958	0.485	0.239	4.14E-92	gdT	MAZ
CD3D2	2.50E-96	0.489765	0.876	0.596	7.04E-92	gdT	CD3D
ZNF6832	4.01E-96	0.793623	0.346	0.146	1.13E-91	gdT	ZNF683
ABHD17A1	1.95E-94	0.494892	0.669	0.391	5.49E-90	gdT	ABHD17A
ARPC5L2	4.17E-93	0.465518	0.544	0.28	1.17E-88	gdT	ARPC5L
IFNG-AS1	2.54E-90	0.2973	0.258	0.091	7.15E-86	gdT	IFNG-AS1
EFHD21	4.98E-90	0.539369	0.569	0.317	1.40E-85	gdT	EFHD2
TRAT1	6.73E-90	0.410442	0.372	0.16	1.89E-85	gdT	TRAT1
SATB11	2.86E-89	0.428638	0.354	0.151	8.05E-85	gdT	SATB1
CD523	1.10E-86	0.475915	0.895	0.66	3.09E-82	gdT	CD52
SLC7A51	3.52E-86	0.388449	0.621	0.352	9.91E-82	gdT	SLC7A5
CNN21	7.38E-86	0.527984	0.685	0.424	2.08E-81	gdT	CNN2
PTMS	4.52E-85	0.368343	0.268	0.103	1.27E-80	gdT	PTMS
RPS4Y11	5.29E-85	0.486227	0.756	0.458	1.49E-80	gdT	RPS4Y1
LYAR3	2.28E-84	0.463678	0.579	0.316	6.40E-80	gdT	LYAR
CD73	2.01E-83	0.370346	0.817	0.507	5.66E-79	gdT	CD7
ABCB11	2.40E-83	0.305055	0.341	0.145	6.75E-79	gdT	ABCB1
RPL283	5.40E-83	0.332369	0.997	0.939	1.52E-78	gdT	RPL28
CASP4	1.72E-82	0.442817	0.478	0.246	4.83E-78	gdT	CASP4
RPS15A4	2.55E-82	0.335323	0.998	0.923	7.17E-78	gdT	RPS15A
ACTG12	6.15E-81	0.510494	0.953	0.814	1.73E-76	gdT	ACTG1
RPL372	1.55E-79	0.349314	0.989	0.894	4.35E-75	gdT	RPL37
RPS210	3.64E-77	0.371597	0.978	0.873	1.02E-72	gdT	RPS2
RPS74	1.36E-76	0.332702	0.994	0.9	3.84E-72	gdT	RPS7
CD993	2.70E-75	0.414961	0.902	0.689	7.59E-71	gdT	CD99
RPS292	5.60E-75	0.378226	0.943	0.8	1.58E-70	gdT	RPS29
CD3G2	1.50E-74	0.405031	0.707	0.429	4.21E-70	gdT	CD3G
LDHA2	1.34E-73	0.565538	0.762	0.534	3.76E-69	gdT	LDHA
ITPRIP1	2.23E-73	0.352663	0.411	0.201	6.26E-69	gdT	ITPRIP
IER5L1	2.13E-72	0.544846	0.379	0.188	5.98E-68	gdT	IER5L
RPS142	4.64E-72	0.330184	0.993	0.907	1.31E-67	gdT	RPS14
UBB3	1.82E-70	0.389299	0.886	0.684	5.13E-66	gdT	UBB
RPL193	9.99E-70	0.312099	0.996	0.917	2.81E-65	gdT	RPL19

RPS123	1.38E-69	0.300149	0.998	0.931	3.88E-65	gdT	RPS12
RPL132	1.41E-69	0.300979	0.999	0.939	3.97E-65	gdT	RPL13
MAP3K83	3.26E-69	0.39727	0.579	0.327	9.18E-65	gdT	MAP3K8
MAPK13	6.41E-69	0.277664	0.555	0.308	1.81E-64	gdT	MAPK1
LBH	1.45E-68	0.425527	0.621	0.37	4.07E-64	gdT	LBH
ACTB2	2.22E-68	0.339319	0.998	0.94	6.24E-64	gdT	ACTB
PIK3AP1	9.86E-68	0.25622	0.263	0.109	2.77E-63	gdT	PIK3AP1
IL2RG1	1.14E-67	0.412497	0.849	0.614	3.20E-63	gdT	IL2RG
NR4A23	1.70E-67	0.558343	0.851	0.653	4.79E-63	gdT	NR4A2
MYL12A2	1.85E-67	0.384702	0.943	0.753	5.20E-63	gdT	MYL12A
MT1X	4.17E-67	0.349715	0.343	0.163	1.17E-62	gdT	MT1X
CCDC1071	6.34E-67	0.436117	0.494	0.271	1.78E-62	gdT	CCDC107
RPSA2	1.03E-66	0.359312	0.964	0.816	2.89E-62	gdT	RPSA
PTPN61	2.76E-66	0.405404	0.427	0.225	7.76E-62	gdT	PTPN6
CD2473	1.19E-65	0.280636	0.797	0.553	3.35E-61	gdT	CD247
TUBA4A1	5.29E-65	0.451548	0.745	0.506	1.49E-60	gdT	TUBA4A
HNRNPA11	7.03E-65	0.408657	0.913	0.722	1.98E-60	gdT	HNRNPA1
PLEK2	1.25E-64	0.30763	0.378	0.185	3.51E-60	gdT	PLEK
TERF2IP1	1.41E-64	0.412693	0.68	0.44	3.98E-60	gdT	TERF2IP
RAC23	1.95E-64	0.409417	0.856	0.652	5.50E-60	gdT	RAC2
RPL18A3	2.34E-64	0.297046	0.995	0.91	6.59E-60	gdT	RPL18A
RPS214	7.11E-64	0.319034	0.984	0.88	2.00E-59	gdT	RPS21
UBE2S2	7.72E-64	0.461292	0.692	0.459	2.17E-59	gdT	UBE2S
RPS34	8.00E-64	0.306869	0.997	0.916	2.25E-59	gdT	RPS3
RPL310	2.41E-63	0.344299	0.987	0.884	6.79E-59	gdT	RPL3
HLA-A2	4.41E-63	0.285629	0.993	0.926	1.24E-58	gdT	HLA-A
TPST22	7.56E-63	0.315325	0.367	0.182	2.13E-58	gdT	TPST2
S100A43	4.65E-62	0.330761	0.907	0.653	1.31E-57	gdT	S100A4
PSME21	2.50E-61	0.464784	0.639	0.415	7.02E-57	gdT	PSME2
RPS82	7.27E-61	0.284939	0.996	0.915	2.05E-56	gdT	RPS8
LCP13	8.82E-61	0.383886	0.851	0.627	2.48E-56	gdT	LCP1
RPL7A3	9.23E-61	0.29502	0.995	0.902	2.60E-56	gdT	RPL7A
RPL262	1.11E-60	0.301133	0.997	0.904	3.12E-56	gdT	RPL26
FKBP112	1.23E-60	0.333463	0.456	0.248	3.47E-56	gdT	FKBP11
PSME12	1.50E-60	0.410028	0.824	0.61	4.21E-56	gdT	PSME1
SERTAD12	2.15E-60	0.457824	0.573	0.351	6.06E-56	gdT	SERTAD1
GYG1	7.13E-60	0.350788	0.348	0.173	2.00E-55	gdT	GYG1
RPS284	3.41E-59	0.282395	0.993	0.914	9.61E-55	gdT	RPS28
RPL35A3	4.71E-59	0.305774	0.985	0.881	1.33E-54	gdT	RPL35A
RPL63	7.03E-59	0.308651	0.991	0.888	1.98E-54	gdT	RPL6
LAT1	2.16E-58	0.311264	0.596	0.348	6.08E-54	gdT	LAT
PTMA1	2.27E-58	0.297454	0.993	0.928	6.40E-54	gdT	PTMA
NOSIP2	3.52E-58	0.388841	0.492	0.288	9.91E-54	gdT	NOSIP
CD273	4.00E-58	0.309732	0.54	0.311	1.12E-53	gdT	CD27
RPL343	2.19E-57	0.286122	0.991	0.911	6.16E-53	gdT	RPL34
CXCR42	3.73E-57	0.389519	0.928	0.797	1.05E-52	gdT	CXCR4
ITM2C2	6.03E-57	0.52045	0.442	0.262	1.70E-52	gdT	ITM2C

PRDX2	1.09E-56	0.341944	0.429	0.237	3.08E-52	gdT	PRDX2
CXCR61	5.53E-56	0.36534	0.375	0.194	1.56E-51	gdT	CXCR6
PFN12	8.06E-56	0.328075	0.982	0.852	2.27E-51	gdT	PFN1
EEF1D2	6.19E-55	0.314463	0.961	0.808	1.74E-50	gdT	EEF1D
RPLP23	7.99E-55	0.304789	0.988	0.887	2.25E-50	gdT	RPLP2
SPOCK22	2.87E-54	0.265946	0.714	0.451	8.09E-50	gdT	SPOCK2
JUND2	2.96E-54	0.446968	0.928	0.807	8.32E-50	gdT	JUND
VAMP5	5.36E-54	0.303306	0.348	0.179	1.51E-49	gdT	VAMP5
RAB5IF2	8.76E-54	0.339738	0.463	0.265	2.46E-49	gdT	RAB5IF
RPL36A2	1.49E-53	0.356092	0.94	0.778	4.20E-49	gdT	RPL36A
RPS27A3	1.61E-53	0.282151	0.997	0.919	4.53E-49	gdT	RPS27A
DUSP41	2.12E-53	0.483962	0.421	0.238	5.97E-49	gdT	DUSP4
LY9	7.63E-53	0.268793	0.31	0.151	2.15E-48	gdT	LY9
SH2D1A1	9.75E-53	0.27121	0.39	0.206	2.74E-48	gdT	SH2D1A
PSMB93	1.13E-52	0.366368	0.705	0.485	3.17E-48	gdT	PSMB9
MARCKSL1	1.89E-52	0.34567	0.394	0.214	5.32E-48	gdT	MARCKSL1
IFNGR11	2.89E-52	0.38729	0.465	0.269	8.14E-48	gdT	IFNGR1
MIDN2	3.39E-52	0.349662	0.458	0.265	9.53E-48	gdT	MIDN
GZMB2	1.44E-51	0.392265	0.332	0.169	4.04E-47	gdT	GZMB
RPS3A2	1.46E-51	0.257278	0.99	0.899	4.09E-47	gdT	RPS3A
SH3BGRL3:	1.90E-51	0.314312	0.953	0.804	5.34E-47	gdT	SH3BGRL3
CTDNEP1	2.47E-51	0.303821	0.441	0.251	6.94E-47	gdT	CTDNEP1
TUBB4B1	2.70E-51	0.361411	0.689	0.466	7.59E-47	gdT	TUBB4B
TRIR2	5.23E-51	0.320546	0.752	0.524	1.47E-46	gdT	TRIR
RPL10A2	6.99E-51	0.309664	0.964	0.816	1.97E-46	gdT	RPL10A
SLC9A3R11	1.17E-50	0.302925	0.616	0.39	3.31E-46	gdT	SLC9A3R1
ARF62	1.46E-50	0.355824	0.669	0.448	4.11E-46	gdT	ARF6
RPL393	3.55E-50	0.263492	0.994	0.906	9.98E-46	gdT	RPL39
CD300A2	6.67E-50	0.296862	0.259	0.122	1.88E-45	gdT	CD300A
PNRC11	8.23E-50	0.365483	0.941	0.793	2.32E-45	gdT	PNRC1
SSR21	1.09E-49	0.324509	0.76	0.534	3.06E-45	gdT	SSR2
RPS162	1.44E-49	0.290309	0.972	0.835	4.05E-45	gdT	RPS16
SEC61G1	2.80E-49	0.294655	0.568	0.353	7.89E-45	gdT	SEC61G
TLE52	5.32E-49	0.346114	0.828	0.618	1.50E-44	gdT	TLE5
GYPC2	1.49E-48	0.324703	0.747	0.512	4.18E-44	gdT	GYPC
PTPN71	2.14E-48	0.259111	0.457	0.263	6.02E-44	gdT	PTPN7
RPL152	1.63E-47	0.288206	0.976	0.839	4.58E-43	gdT	RPL15
CFL12	2.37E-47	0.314666	0.955	0.808	6.66E-43	gdT	CFL1
RAB9A	5.55E-47	0.275996	0.285	0.142	1.56E-42	gdT	RAB9A
UBE2L6	6.46E-47	0.315939	0.494	0.303	1.82E-42	gdT	UBE2L6
SLC25A6	7.14E-47	0.327649	0.819	0.608	2.01E-42	gdT	SLC25A6
UBA522	8.74E-47	0.303078	0.951	0.8	2.46E-42	gdT	UBA52
CISD31	1.10E-46	0.250463	0.298	0.151	3.10E-42	gdT	CISD3
RPS92	1.34E-46	0.274833	0.973	0.851	3.76E-42	gdT	RPS9
ICAM3	2.06E-46	0.300113	0.598	0.383	5.80E-42	gdT	ICAM3
POLR2E	4.95E-46	0.274456	0.398	0.225	1.39E-41	gdT	POLR2E
PPIA1	5.59E-46	0.327855	0.936	0.762	1.57E-41	gdT	PPIA

NFKBIA2	1.31E-45	0.342711	0.902	0.738	3.70E-41	gdT	NFKBIA
PPIB1	1.56E-45	0.332299	0.821	0.619	4.40E-41	gdT	PPIB
LCK2	2.32E-45	0.290626	0.741	0.52	6.54E-41	gdT	LCK
H2AFX2	5.27E-45	0.272518	0.491	0.294	1.48E-40	gdT	H2AFX
CKLF1	6.12E-45	0.292583	0.563	0.357	1.72E-40	gdT	CKLF
RPL272	1.32E-44	0.309682	0.917	0.748	3.72E-40	gdT	RPL27
ADRB21	1.86E-44	0.290141	0.29	0.15	5.24E-40	gdT	ADRB2
RGCC2	1.90E-44	0.612948	0.672	0.492	5.34E-40	gdT	RGCC
BZW12	1.18E-43	0.2847	0.599	0.39	3.32E-39	gdT	BZW1
RNF1671	1.31E-43	0.279722	0.444	0.264	3.70E-39	gdT	RNF167
RPL352	1.53E-43	0.277924	0.97	0.841	4.30E-39	gdT	RPL35
RPLP02	1.83E-43	0.263608	0.985	0.868	5.15E-39	gdT	RPLP0
SLC25A52	6.28E-43	0.333625	0.651	0.455	1.77E-38	gdT	SLC25A5
ARL4C2	8.17E-43	0.29902	0.774	0.551	2.30E-38	gdT	ARL4C
HSPA52	1.95E-42	0.31649	0.782	0.571	5.49E-38	gdT	HSPA5
FBXW51	2.09E-42	0.267628	0.375	0.213	5.89E-38	gdT	FBXW5
GHITM	2.68E-42	0.272667	0.454	0.274	7.53E-38	gdT	GHITM
PTP4A22	3.38E-42	0.265459	0.635	0.431	9.52E-38	gdT	PTP4A2
CRTAM1	4.07E-42	0.260287	0.258	0.128	1.15E-37	gdT	CRTAM
CYCS1	5.66E-42	0.277873	0.6	0.393	1.59E-37	gdT	CYCS
IGFLR1	1.96E-41	0.284253	0.284	0.149	5.51E-37	gdT	IGFLR1
CSRNP11	3.41E-41	0.351987	0.617	0.419	9.60E-37	gdT	CSRNP1
ZYX1	4.01E-41	0.279379	0.562	0.362	1.13E-36	gdT	ZYX
SELENOH	4.20E-41	0.273557	0.488	0.303	1.18E-36	gdT	SELENOH
RPL13A1	4.52E-41	0.272426	0.938	0.788	1.27E-36	gdT	RPL13A
RAB291	6.41E-41	0.253996	0.254	0.129	1.80E-36	gdT	RAB29
GABARAPL	1.54E-40	0.266464	0.542	0.341	4.34E-36	gdT	GABARAPL2
CD482	2.25E-40	0.286022	0.656	0.444	6.32E-36	gdT	CD48
CALM13	5.32E-40	0.266391	0.941	0.814	1.50E-35	gdT	CALM1
CLIC13	1.09E-39	0.277049	0.835	0.628	3.06E-35	gdT	CLIC1
ZC3H12A1	1.28E-39	0.320851	0.354	0.203	3.60E-35	gdT	ZC3H12A
ARF11	1.48E-39	0.26824	0.715	0.505	4.17E-35	gdT	ARF1
GNAS	1.59E-39	0.305111	0.806	0.641	4.48E-35	gdT	GNAS
SNRPD22	1.84E-39	0.264707	0.798	0.593	5.17E-35	gdT	SNRPD2
RPL52	2.05E-39	0.253381	0.974	0.843	5.78E-35	gdT	RPL5
RPL312	2.11E-39	0.283554	0.901	0.723	5.93E-35	gdT	RPL31
CSK1	2.19E-39	0.255314	0.506	0.323	6.17E-35	gdT	CSK
EEF22	2.84E-39	0.277547	0.912	0.745	8.00E-35	gdT	EEF2
ABRACL1	3.86E-39	0.259412	0.571	0.368	1.09E-34	gdT	ABRACL
CTSD3	4.81E-39	0.279386	0.665	0.45	1.35E-34	gdT	CTSD
DYNLL1	5.56E-39	0.275027	0.537	0.349	1.56E-34	gdT	DYNLL1
BIN22	1.30E-38	0.266329	0.575	0.383	3.65E-34	gdT	BIN2
LITAF2	1.77E-38	0.270849	0.708	0.509	4.97E-34	gdT	LITAF
GAPDH1	1.98E-38	0.308408	0.944	0.818	5.58E-34	gdT	GAPDH
H3F3A1	4.18E-38	0.295688	0.877	0.698	1.18E-33	gdT	H3F3A
RPL231	6.98E-38	0.279064	0.704	0.491	1.96E-33	gdT	RPL23
P4HB1	7.02E-38	0.254418	0.536	0.347	1.98E-33	gdT	P4HB

IFRD12	8.60E-38	0.292191	0.539	0.359	2.42E-33	gdT	IFRD1
GUK11	1.08E-37	0.250967	0.736	0.529	3.05E-33	gdT	GUK1
COX7C2	1.62E-37	0.265924	0.856	0.658	4.56E-33	gdT	COX7C
SSR41	3.89E-37	0.251252	0.777	0.567	1.09E-32	gdT	SSR4
ATP5MG1	8.86E-37	0.279935	0.831	0.657	2.49E-32	gdT	ATP5MG
UQCRH	9.22E-37	0.268062	0.628	0.43	2.59E-32	gdT	UQCRH
DNAAF2	1.01E-36	0.270168	0.275	0.149	2.84E-32	gdT	DNAAF2
RPS201	1.76E-36	0.285782	0.722	0.519	4.96E-32	gdT	RPS20
UCP21	3.01E-36	0.426815	0.552	0.386	8.47E-32	gdT	UCP2
SEC61B1	3.27E-36	0.274423	0.627	0.443	9.19E-32	gdT	SEC61B
PPP1CA1	5.26E-36	0.292586	0.571	0.392	1.48E-31	gdT	PPP1CA
HMGN2	1.51E-35	0.289965	0.656	0.461	4.25E-31	gdT	HMGN2
EEF1B22	2.16E-35	0.268029	0.936	0.765	6.08E-31	gdT	EEF1B2
TNF1	2.23E-35	0.314284	0.425	0.268	6.28E-31	gdT	TNF
COX4I11	3.12E-35	0.25286	0.911	0.736	8.78E-31	gdT	COX4I1
IFNG2	3.32E-35	0.297586	0.278	0.15	9.35E-31	gdT	IFNG
HNRNPL	4.45E-35	0.261413	0.693	0.495	1.25E-30	gdT	HNRNPL
REL3	4.82E-35	0.261815	0.662	0.464	1.36E-30	gdT	REL
GLRX2	8.28E-35	0.256134	0.319	0.184	2.33E-30	gdT	GLRX
NINJ11	1.59E-34	0.281041	0.275	0.153	4.48E-30	gdT	NINJ1
DUSP12	1.79E-34	0.307657	0.935	0.837	5.03E-30	gdT	DUSP1
EEF1G2	2.19E-34	0.252787	0.876	0.685	6.16E-30	gdT	EEF1G
AL135905.:	5.15E-34	0.271449	0.291	0.164	1.45E-29	gdT	AL135905.2
TAP11	6.44E-34	0.291196	0.445	0.287	1.81E-29	gdT	TAP1
EIF3L1	7.05E-34	0.253225	0.556	0.372	1.98E-29	gdT	EIF3L
PHB2	1.10E-33	0.250733	0.458	0.296	3.09E-29	gdT	PHB2
SELL2	3.83E-33	0.262219	0.313	0.183	1.08E-28	gdT	SELL
NDUFS52	1.59E-32	0.277148	0.679	0.487	4.46E-28	gdT	NDUFS5
CD692	6.17E-32	0.2979	0.882	0.746	1.74E-27	gdT	CD69
SRGN2	3.23E-31	0.257708	0.96	0.864	9.09E-27	gdT	SRGN
PTGER42	4.10E-31	0.266845	0.641	0.454	1.15E-26	gdT	PTGER4
LIMD22	4.68E-29	0.265578	0.659	0.487	1.32E-24	gdT	LIMD2
MAFF2	1.45E-28	0.282792	0.312	0.189	4.09E-24	gdT	MAFF
ZFP36L22	2.24E-28	0.325969	0.979	0.923	6.31E-24	gdT	ZFP36L2
TXNIP2	2.40E-25	0.270433	0.874	0.723	6.75E-21	gdT	TXNIP
CTLA4	0	2.676633	0.741	0.099	0	Treg	CTLA4
FOXP3	0	2.510993	0.59	0.007	0	Treg	FOXP3
TNFRSF4	0	1.961706	0.428	0.035	0	Treg	TNFRSF4
TNFRSF18	0	1.816864	0.435	0.04	0	Treg	TNFRSF18
RTKN2	0	1.627334	0.371	0.023	0	Treg	RTKN2
BATF	1.46E-301	2.390347	0.682	0.123	4.11E-297	Treg	BATF
TIGIT	3.19E-275	1.92092	0.609	0.102	8.99E-271	Treg	TIGIT
IL2RA	1.31E-234	1.13438	0.259	0.021	3.67E-230	Treg	IL2RA
LAIR2	2.14E-192	1.154421	0.281	0.03	6.03E-188	Treg	LAIR2
LINC01943	5.20E-184	1.13532	0.265	0.028	1.46E-179	Treg	LINC01943
AL136456.:	2.91E-181	1.433463	0.378	0.055	8.18E-177	Treg	AL136456.1
ICA1	1.36E-176	1.112017	0.252	0.026	3.82E-172	Treg	ICA1

TBC1D4	9.25E-174	1.715344	0.545	0.121	2.60E-169	Treg	TBC1D4
IKZF2	1.99E-142	1.347513	0.462	0.098	5.59E-138	Treg	IKZF2
STAM	7.44E-142	1.521219	0.467	0.105	2.09E-137	Treg	STAM
ICOS1	1.94E-139	1.612889	0.73	0.26	5.47E-135	Treg	ICOS
SLAMF1	1.94E-132	1.400533	0.467	0.108	5.46E-128	Treg	SLAMF1
PELI11	1.87E-121	1.735839	0.597	0.189	5.28E-117	Treg	PELI1
TNFRSF9	1.45E-119	1.166886	0.263	0.039	4.07E-115	Treg	TNFRSF9
IL323	8.75E-119	1.552764	0.979	0.762	2.46E-114	Treg	IL32
PHLDA1	4.05E-116	1.78289	0.503	0.141	1.14E-111	Treg	PHLDA1
DUSP42	1.02E-114	1.899716	0.657	0.241	2.87E-110	Treg	DUSP4
MAST4	2.37E-102	1.05902	0.254	0.042	6.66E-98	Treg	MAST4
CARD161	2.74E-96	1.562597	0.622	0.252	7.70E-92	Treg	CARD16
CORO1B1	3.28E-85	1.392765	0.551	0.209	9.24E-81	Treg	CORO1B
HLA-A3	1.11E-83	0.88828	0.986	0.928	3.11E-79	Treg	HLA-A
DNPH1	1.63E-82	1.027304	0.355	0.093	4.58E-78	Treg	DNPH1
CD274	2.21E-82	1.382066	0.677	0.316	6.21E-78	Treg	CD27
PMAIP1	1.36E-79	1.672079	0.568	0.234	3.83E-75	Treg	PMAIP1
LTB2	2.70E-77	1.518486	0.716	0.35	7.60E-73	Treg	LTB
CD42	1.10E-76	0.946382	0.432	0.134	3.10E-72	Treg	CD4
PHACTR2	4.82E-73	0.988095	0.524	0.193	1.36E-68	Treg	PHACTR2
ACP5	4.43E-70	0.818897	0.304	0.078	1.25E-65	Treg	ACP5
DUSP16	4.59E-67	0.946273	0.41	0.133	1.29E-62	Treg	DUSP16
PRDM11	7.81E-61	1.195396	0.664	0.349	2.20E-56	Treg	PRDM1
ARID5B1	1.08E-58	0.916688	0.705	0.37	3.05E-54	Treg	ARID5B
SAT11	4.46E-54	1.095894	0.856	0.59	1.25E-49	Treg	SAT1
SIRPG	2.41E-53	0.955686	0.421	0.161	6.79E-49	Treg	SIRPG
S100A44	4.03E-53	0.9397	0.895	0.661	1.13E-48	Treg	S100A4
SNX93	2.86E-52	0.960832	0.558	0.261	8.06E-48	Treg	SNX9
RHBDD2	3.93E-51	0.922141	0.414	0.162	1.11E-46	Treg	RHBDD2
MIR4435-2	2.00E-50	0.983821	0.485	0.207	5.64E-46	Treg	MIR4435-2HG
GAPDH2	5.70E-49	0.973086	0.963	0.821	1.60E-44	Treg	GAPDH
MYL61	1.00E-47	0.700224	0.954	0.771	2.82E-43	Treg	MYL6
PHTF2	7.71E-47	0.870489	0.428	0.175	2.17E-42	Treg	PHTF2
BTG31	5.93E-46	1.025375	0.483	0.222	1.67E-41	Treg	BTG3
FAS	9.93E-46	0.77292	0.277	0.086	2.79E-41	Treg	FAS
UCP22	5.35E-45	1.002298	0.657	0.389	1.51E-40	Treg	UCP2
B2M3	7.36E-45	0.436756	0.998	0.971	2.07E-40	Treg	B2M
RGS12	1.00E-44	0.999384	0.856	0.595	2.82E-40	Treg	RGS1
CD281	2.14E-44	0.752781	0.38	0.147	6.03E-40	Treg	CD28
PLCL1	1.88E-42	0.652828	0.256	0.078	5.30E-38	Treg	PLCL1
ARHGDIB1	2.36E-42	0.608091	0.961	0.834	6.65E-38	Treg	ARHGDIB
BIRC3	5.30E-42	0.878486	0.343	0.13	1.49E-37	Treg	BIRC3
HLA-B3	6.38E-42	0.572134	0.995	0.957	1.80E-37	Treg	HLA-B
CTSC1	1.07E-41	1.250432	0.664	0.429	3.01E-37	Treg	CTSC
GBP51	2.94E-40	0.913445	0.524	0.269	8.26E-36	Treg	GBP5
UGP2	2.83E-38	0.92275	0.444	0.211	7.97E-34	Treg	UGP2
CALM31	2.26E-37	0.813615	0.574	0.327	6.37E-33	Treg	CALM3

PIM22	3.09E-37	0.876474	0.462	0.221	8.68E-33	Treg	PIM2
SPOCK23	4.87E-37	0.777482	0.728	0.459	1.37E-32	Treg	SPOCK2
TOX2	9.16E-37	0.541319	0.579	0.301	2.58E-32	Treg	TOX
SOD11	9.99E-35	0.852424	0.691	0.456	2.81E-30	Treg	SOD1
GADD45A1	2.40E-34	1.019317	0.366	0.163	6.75E-30	Treg	GADD45A
IGFLR11	4.93E-34	0.783223	0.352	0.152	1.39E-29	Treg	IGFLR1
C9orf16	8.13E-34	0.83874	0.638	0.417	2.29E-29	Treg	C9orf16
SAMSN11	9.06E-34	0.816496	0.735	0.502	2.55E-29	Treg	SAMSN1
ZBTB382	6.59E-33	0.674558	0.49	0.249	1.85E-28	Treg	ZBTB38
GBP21	2.12E-32	0.745477	0.465	0.238	5.97E-28	Treg	GBP2
CNIH1	2.17E-32	0.898921	0.348	0.157	6.10E-28	Treg	CNIH1
EPSTI1	3.84E-32	0.756621	0.355	0.156	1.08E-27	Treg	EPSTI1
CD741	4.31E-32	0.373595	0.936	0.769	1.21E-27	Treg	CD74
RBPJ	5.06E-32	0.880237	0.455	0.239	1.42E-27	Treg	RBPJ
FKBP1A	1.03E-31	0.704673	0.449	0.231	2.91E-27	Treg	FKBP1A
SCAND1	1.19E-31	0.76268	0.574	0.343	3.36E-27	Treg	SCAND1
BCL3	3.05E-31	0.691502	0.261	0.098	8.58E-27	Treg	BCL3
ZC3H7A	6.78E-31	0.690445	0.297	0.122	1.91E-26	Treg	ZC3H7A
LGALS3	1.34E-30	0.800051	0.32	0.137	3.78E-26	Treg	LGALS3
NDUFV2	9.55E-30	0.752881	0.49	0.276	2.69E-25	Treg	NDUFV2
CNST	7.62E-29	0.610227	0.364	0.168	2.15E-24	Treg	CNST
BACH11	9.87E-29	0.649193	0.293	0.121	2.78E-24	Treg	BACH1
USP151	1.02E-28	0.66674	0.689	0.454	2.86E-24	Treg	USP15
NSD31	1.03E-28	0.569209	0.586	0.35	2.90E-24	Treg	NSD3
FXYD52	2.25E-28	0.585209	0.851	0.68	6.34E-24	Treg	FXYD5
UBC1	2.28E-28	0.569811	0.973	0.888	6.43E-24	Treg	UBC
ATP5MC21	8.19E-28	0.560495	0.879	0.706	2.31E-23	Treg	ATP5MC2
STAT3	1.09E-27	0.679012	0.593	0.363	3.06E-23	Treg	STAT3
SIT12	4.01E-27	0.626487	0.423	0.218	1.13E-22	Treg	SIT1
TMSB4X1	4.04E-27	0.431333	0.989	0.944	1.14E-22	Treg	TMSB4X
TMSB102	2.06E-26	0.492806	0.993	0.922	5.78E-22	Treg	TMSB10
PPM1G	3.38E-26	0.642535	0.442	0.241	9.52E-22	Treg	PPM1G
HNRNPLL1	5.34E-26	0.590212	0.43	0.225	1.50E-21	Treg	HNRNPLL
NAMPT1	5.50E-26	0.64254	0.446	0.238	1.55E-21	Treg	NAMPT
ARPC1B1	6.64E-26	0.594018	0.728	0.545	1.87E-21	Treg	ARPC1B
GADD45G	1.40E-25	0.772042	0.256	0.106	3.93E-21	Treg	GADD45G
PKM	1.57E-25	0.765011	0.549	0.348	4.42E-21	Treg	PKM
HLA-DRB11	1.67E-25	0.461046	0.57	0.323	4.71E-21	Treg	HLA-DRB1
ICAM31	2.81E-25	0.604234	0.597	0.39	7.91E-21	Treg	ICAM3
PBXIP12	3.82E-25	0.689661	0.492	0.289	1.07E-20	Treg	PBXIP1
SUB11	4.59E-25	0.576356	0.755	0.57	1.29E-20	Treg	SUB1
SRP141	1.47E-24	0.532531	0.817	0.631	4.13E-20	Treg	SRP14
ACTB3	4.31E-24	0.485797	0.998	0.942	1.21E-19	Treg	ACTB
RNF187	6.90E-24	0.613787	0.359	0.184	1.94E-19	Treg	RNF187
CD3D3	1.22E-23	0.490557	0.799	0.606	3.42E-19	Treg	CD3D
IL2RG2	2.64E-23	0.589821	0.794	0.622	7.43E-19	Treg	IL2RG
SUMO2	3.56E-23	0.546462	0.73	0.563	1.00E-18	Treg	SUMO2

PRDX21	6.35E-23	0.625077	0.428	0.243	1.79E-18	Treg	PRDX2
PFN13	2.14E-22	0.436443	0.947	0.856	6.02E-18	Treg	PFN1
OAZ12	5.32E-22	0.506956	0.824	0.689	1.50E-17	Treg	OAZ1
FOXO11	1.73E-21	0.387468	0.593	0.374	4.87E-17	Treg	FOXO1
BUB31	2.77E-21	0.572631	0.561	0.364	7.79E-17	Treg	BUB3
CLIC14	3.80E-21	0.523138	0.815	0.634	1.07E-16	Treg	CLIC1
FCMR2	3.88E-21	0.53185	0.554	0.356	1.09E-16	Treg	FCMR
PLSCR3	4.53E-21	0.450048	0.307	0.147	1.28E-16	Treg	PLSCR3
ITM2A1	5.69E-21	0.621306	0.579	0.394	1.60E-16	Treg	ITM2A
SMC4	5.91E-21	0.494364	0.291	0.137	1.66E-16	Treg	SMC4
ISG201	8.37E-21	0.590083	0.737	0.58	2.36E-16	Treg	ISG20
THADA	1.15E-20	0.600579	0.295	0.141	3.23E-16	Treg	THADA
ALDOA1	1.24E-20	0.561657	0.597	0.404	3.48E-16	Treg	ALDOA
PAK21	1.31E-20	0.492071	0.522	0.322	3.70E-16	Treg	PAK2
TNFRSF1B2	3.35E-20	0.590064	0.524	0.342	9.43E-16	Treg	TNFRSF1B
COX5A	9.64E-20	0.63089	0.508	0.341	2.71E-15	Treg	COX5A
NIBAN11	1.44E-19	0.570521	0.533	0.35	4.05E-15	Treg	NIBAN1
PARK71	1.92E-19	0.645178	0.584	0.414	5.42E-15	Treg	PARK7
CXCR62	2.52E-19	0.657481	0.366	0.2	7.09E-15	Treg	CXCR6
COX8A	3.01E-19	0.603852	0.618	0.446	8.46E-15	Treg	COX8A
ZEB1	7.73E-19	0.448346	0.352	0.185	2.17E-14	Treg	ZEB1
SAMHD12	1.01E-18	0.477664	0.535	0.354	2.85E-14	Treg	SAMHD1
LDHB2	2.32E-18	0.522806	0.67	0.514	6.53E-14	Treg	LDHB
CCNI	2.74E-18	0.519126	0.787	0.629	7.70E-14	Treg	CCNI
RAC24	3.22E-18	0.457785	0.796	0.659	9.05E-14	Treg	RAC2
CYTOR2	5.49E-18	0.434874	0.492	0.299	1.54E-13	Treg	CYTOR
CD23	5.51E-18	0.461882	0.748	0.577	1.55E-13	Treg	CD2
RSBN1	5.59E-18	0.539771	0.416	0.25	1.57E-13	Treg	RSBN1
RAB11FIP1	6.49E-18	0.471067	0.27	0.13	1.82E-13	Treg	RAB11FIP1
SH3BGRL34	1.91E-17	0.379504	0.938	0.809	5.39E-13	Treg	SH3BGRL3
ACTG13	2.37E-17	0.452681	0.915	0.819	6.68E-13	Treg	ACTG1
SKAP11	2.76E-17	0.303658	0.748	0.575	7.76E-13	Treg	SKAP1
PVT1	3.01E-17	0.441199	0.275	0.137	8.47E-13	Treg	PVT1
SYNGR21	5.30E-17	0.645753	0.403	0.245	1.49E-12	Treg	SYNGR2
SLC25A3	1.31E-16	0.508784	0.641	0.487	3.69E-12	Treg	SLC25A3
STK17B1	1.85E-16	0.505522	0.773	0.633	5.21E-12	Treg	STK17B
SERF21	1.95E-16	0.390959	0.924	0.792	5.47E-12	Treg	SERF2
RAB9A1	2.84E-16	0.626449	0.275	0.146	7.98E-12	Treg	RAB9A
ZNRF2	4.37E-16	0.339528	0.32	0.172	1.23E-11	Treg	ZNRF2
CUTA	4.95E-16	0.513551	0.57	0.407	1.39E-11	Treg	CUTA
PSMB81	5.74E-16	0.481967	0.563	0.404	1.62E-11	Treg	PSMB8
COX5B	6.70E-16	0.435231	0.611	0.449	1.88E-11	Treg	COX5B
CLEC2D1	7.29E-16	0.526318	0.492	0.325	2.05E-11	Treg	CLEC2D
CD524	7.64E-16	0.459116	0.805	0.669	2.15E-11	Treg	CD52
NDUFA4	8.36E-16	0.4895	0.643	0.505	2.35E-11	Treg	NDUFA4
TLE53	8.56E-16	0.466458	0.771	0.626	2.41E-11	Treg	TLE5
ITGA41	9.27E-16	0.474242	0.593	0.425	2.61E-11	Treg	ITGA4

EID1	9.90E-16	0.509809	0.542	0.38	2.79E-11	Treg	EID1
YWHAB1	1.08E-15	0.47981	0.73	0.602	3.03E-11	Treg	YWHAB
RHOH1	1.18E-15	0.435058	0.618	0.452	3.33E-11	Treg	RHOH
TRAPPC11	1.25E-15	0.471645	0.469	0.314	3.53E-11	Treg	TRAPPC1
LIMD23	1.49E-15	0.503996	0.652	0.493	4.20E-11	Treg	LIMD2
IQGAP12	1.63E-15	0.509874	0.593	0.432	4.60E-11	Treg	IQGAP1
RNF19A1	1.86E-15	0.428441	0.595	0.423	5.25E-11	Treg	RNF19A
H3F3A2	2.83E-15	0.442838	0.828	0.704	7.97E-11	Treg	H3F3A
YPEL31	2.92E-15	0.451409	0.604	0.439	8.21E-11	Treg	YPEL3
GLRX3	3.16E-15	0.502808	0.327	0.188	8.88E-11	Treg	GLRX
CD382	3.27E-15	0.471381	0.281	0.149	9.21E-11	Treg	CD38
GMFG2	5.23E-15	0.441947	0.7	0.569	1.47E-10	Treg	GMFG
LSP13	5.28E-15	0.445204	0.764	0.617	1.48E-10	Treg	LSP1
IRF12	5.46E-15	0.50959	0.6	0.437	1.54E-10	Treg	IRF1
TSPAN141	5.61E-15	0.460823	0.419	0.265	1.58E-10	Treg	TSPAN14
ENO1	6.48E-15	0.430927	0.59	0.431	1.82E-10	Treg	ENO1
TMEM173:	6.65E-15	0.420611	0.359	0.209	1.87E-10	Treg	TMEM173
PIK3IP11	6.89E-15	0.471662	0.611	0.447	1.94E-10	Treg	PIK3IP1
SQSTM13	1.02E-14	0.529565	0.588	0.422	2.86E-10	Treg	SQSTM1
NDUFA13	1.05E-14	0.434153	0.627	0.482	2.97E-10	Treg	NDUFA13
IL6ST3	1.13E-14	0.473187	0.403	0.251	3.17E-10	Treg	IL6ST
MAN1A2	1.19E-14	0.391311	0.307	0.17	3.35E-10	Treg	MAN1A2
LINC01578	1.21E-14	0.488306	0.682	0.543	3.40E-10	Treg	LINC01578
PTPRCAP2	1.62E-14	0.405002	0.622	0.439	4.55E-10	Treg	PTPRCAP
ELOB1	2.21E-14	0.338472	0.684	0.508	6.23E-10	Treg	ELOB
TNIK1	2.29E-14	0.357566	0.389	0.236	6.44E-10	Treg	TNIK
CSGALNAC	2.38E-14	0.418958	0.259	0.136	6.69E-10	Treg	CSGALNACT2
EIF3F	2.47E-14	0.390988	0.682	0.533	6.95E-10	Treg	EIF3F
EIF3K	2.58E-14	0.394782	0.753	0.611	7.27E-10	Treg	EIF3K
SH3BGRL1	2.75E-14	0.459622	0.531	0.383	7.73E-10	Treg	SH3BGRL
RACK12	3.24E-14	0.330261	0.938	0.846	9.11E-10	Treg	RACK1
RAB11B	4.23E-14	0.436783	0.407	0.26	1.19E-09	Treg	RAB11B
CYCS2	4.68E-14	0.51159	0.551	0.4	1.32E-09	Treg	CYCS
RPL153	4.92E-14	0.299384	0.954	0.844	1.39E-09	Treg	RPL15
BCL2L11	5.82E-14	0.42018	0.279	0.152	1.64E-09	Treg	BCL2L11
SELL3	6.95E-14	0.431759	0.327	0.187	1.96E-09	Treg	SELL
ZNF2921	7.63E-14	0.406397	0.412	0.259	2.15E-09	Treg	ZNF292
RAP1GDS1	8.48E-14	0.333629	0.4	0.243	2.39E-09	Treg	RAP1GDS1
HLA-DMA1	1.04E-13	0.419474	0.295	0.163	2.93E-09	Treg	HLA-DMA
CSNK2B1	1.08E-13	0.402963	0.533	0.378	3.05E-09	Treg	CSNK2B
CDKN1B2	1.21E-13	0.528851	0.506	0.351	3.41E-09	Treg	CDKN1B
TRAC1	1.51E-13	0.456627	0.359	0.216	4.26E-09	Treg	TRAC
DAD1	1.76E-13	0.427331	0.54	0.383	4.95E-09	Treg	DAD1
IL10RA	2.37E-13	0.476465	0.492	0.34	6.68E-09	Treg	IL10RA
SUSD3	2.48E-13	0.360692	0.279	0.154	6.97E-09	Treg	SUSD3
UBE2D3	2.76E-13	0.463586	0.709	0.574	7.76E-09	Treg	UBE2D3
FAM53B	3.64E-13	0.333545	0.263	0.141	1.02E-08	Treg	FAM53B

ATP5MC3	5.44E-13	0.450256	0.542	0.405	1.53E-08	Treg	ATP5MC3
NFKBIZ3	6.09E-13	0.523974	0.547	0.394	1.71E-08	Treg	NFKBIZ
HINT13	6.38E-13	0.393692	0.737	0.61	1.80E-08	Treg	HINT1
COTL12	6.43E-13	0.413342	0.634	0.498	1.81E-08	Treg	COTL1
NOP58	6.46E-13	0.478779	0.462	0.324	1.82E-08	Treg	NOP58
LINC01934	8.05E-13	0.400372	0.352	0.215	2.26E-08	Treg	LINC01934
PPDPF2	8.42E-13	0.348514	0.879	0.725	2.37E-08	Treg	PPDPF
PSIP1	8.71E-13	0.38478	0.382	0.239	2.45E-08	Treg	PSIP1
NDUFB8	1.05E-12	0.424775	0.538	0.394	2.95E-08	Treg	NDUFB8
MRPL54	1.45E-12	0.40603	0.364	0.227	4.08E-08	Treg	MRPL54
CD581	1.57E-12	0.387347	0.318	0.189	4.43E-08	Treg	CD58
CFL13	1.66E-12	0.287406	0.922	0.813	4.68E-08	Treg	CFL1
UHRF2	2.03E-12	0.403345	0.293	0.168	5.71E-08	Treg	UHRF2
CCNDBP1	3.47E-12	0.377149	0.334	0.204	9.75E-08	Treg	CCNDBP1
VAMP51	3.72E-12	0.420873	0.309	0.185	1.05E-07	Treg	VAMP5
C12orf571	4.04E-12	0.37623	0.689	0.545	1.14E-07	Treg	C12orf57
CORO1A2	4.77E-12	0.346369	0.819	0.71	1.34E-07	Treg	CORO1A
ANP32B2	5.29E-12	0.41794	0.535	0.398	1.49E-07	Treg	ANP32B
PFKL	6.31E-12	0.277582	0.254	0.139	1.78E-07	Treg	PFKL
TAGLN23	6.62E-12	0.383143	0.652	0.5	1.86E-07	Treg	TAGLN2
CACYBP1	6.69E-12	0.533167	0.414	0.279	1.88E-07	Treg	CACYBP
CUL31	7.87E-12	0.338502	0.469	0.323	2.21E-07	Treg	CUL3
PSME22	1.19E-11	0.445867	0.558	0.423	3.34E-07	Treg	PSME2
UBXN1	1.46E-11	0.340609	0.581	0.435	4.11E-07	Treg	UBXN1
GATA3	1.61E-11	0.453733	0.368	0.236	4.53E-07	Treg	GATA3
TLK1	1.75E-11	0.282881	0.343	0.211	4.92E-07	Treg	TLK1
COX4I12	1.83E-11	0.316504	0.879	0.742	5.14E-07	Treg	COX4I1
TANK	2.04E-11	0.382435	0.375	0.244	5.73E-07	Treg	TANK
HIST1H1C	2.50E-11	0.504933	0.261	0.15	7.03E-07	Treg	HIST1H1C
CD3E3	2.52E-11	0.278673	0.874	0.744	7.10E-07	Treg	CD3E
GALM	2.53E-11	0.337897	0.316	0.192	7.13E-07	Treg	GALM
NDUFS7	2.57E-11	0.433173	0.387	0.255	7.24E-07	Treg	NDUFS7
ATP5IF11	2.74E-11	0.380918	0.561	0.421	7.72E-07	Treg	ATP5IF1
CLTB	3.10E-11	0.32034	0.346	0.215	8.72E-07	Treg	CLTB
ATP5F1D1	3.21E-11	0.365337	0.597	0.46	9.03E-07	Treg	ATP5F1D
MTHFD21	3.21E-11	0.515055	0.307	0.193	9.04E-07	Treg	MTHFD2
PSMB94	3.78E-11	0.383183	0.613	0.493	1.06E-06	Treg	PSMB9
TENT5C3	4.61E-11	0.404751	0.609	0.468	1.30E-06	Treg	TENT5C
BAX1	5.14E-11	0.474579	0.497	0.357	1.45E-06	Treg	BAX
CSTB	5.16E-11	0.395745	0.439	0.3	1.45E-06	Treg	CSTB
VAV34	5.31E-11	0.385977	0.348	0.221	1.50E-06	Treg	VAV3
PSMB3	5.69E-11	0.40859	0.426	0.3	1.60E-06	Treg	PSMB3
SUMO1	8.91E-11	0.337326	0.407	0.271	2.51E-06	Treg	SUMO1
CREM1	8.95E-11	0.483302	0.627	0.489	2.52E-06	Treg	CREM
UBE2B	9.23E-11	0.331388	0.469	0.335	2.60E-06	Treg	UBE2B
KLF61	9.64E-11	0.35353	0.84	0.71	2.71E-06	Treg	KLF6
CTSZ1	1.03E-10	0.400895	0.254	0.148	2.89E-06	Treg	CTSZ

COPZ11	1.08E-10	0.34774	0.284	0.172	3.03E-06	Treg	COPZ1
RAP1A	1.13E-10	0.306885	0.549	0.405	3.17E-06	Treg	RAP1A
H1FX1	1.16E-10	0.513344	0.636	0.496	3.28E-06	Treg	H1FX
CLPP	1.22E-10	0.382246	0.341	0.218	3.44E-06	Treg	CLPP
MIF2	1.35E-10	0.385874	0.764	0.647	3.80E-06	Treg	MIF
CASP1	1.50E-10	0.356571	0.263	0.155	4.21E-06	Treg	CASP1
COX6A1	1.52E-10	0.279061	0.707	0.557	4.27E-06	Treg	COX6A1
C17orf491	1.53E-10	0.335847	0.311	0.195	4.30E-06	Treg	C17orf49
CHMP2A	1.73E-10	0.35661	0.275	0.165	4.86E-06	Treg	CHMP2A
SNX5	1.82E-10	0.341481	0.295	0.181	5.11E-06	Treg	SNX5
ATP5F1E2	1.89E-10	0.259194	0.904	0.792	5.31E-06	Treg	ATP5F1E
HNRNPK1	2.71E-10	0.360797	0.682	0.557	7.63E-06	Treg	HNRNPK
ARPC41	2.81E-10	0.308533	0.485	0.353	7.92E-06	Treg	ARPC4
AGTRAP1	3.00E-10	0.422904	0.261	0.157	8.45E-06	Treg	AGTRAP
PPP1CA2	3.39E-10	0.402217	0.517	0.398	9.52E-06	Treg	PPP1CA
PRDX11	3.82E-10	0.439277	0.446	0.322	1.08E-05	Treg	PRDX1
ATP5MG2	3.82E-10	0.305064	0.76	0.663	1.08E-05	Treg	ATP5MG
COX6C	3.99E-10	0.336771	0.636	0.521	1.12E-05	Treg	COX6C
PRNP	4.25E-10	0.383106	0.325	0.21	1.19E-05	Treg	PRNP
PGAM1	4.46E-10	0.412389	0.43	0.308	1.26E-05	Treg	PGAM1
R3HDM4	5.19E-10	0.343788	0.311	0.197	1.46E-05	Treg	R3HDM4
NCOA3	5.87E-10	0.256209	0.314	0.195	1.65E-05	Treg	NCOA3
IFI62	5.98E-10	0.523828	0.398	0.273	1.68E-05	Treg	IFI6
ARPC32	6.13E-10	0.314745	0.755	0.637	1.73E-05	Treg	ARPC3
EIF3H1	6.77E-10	0.339326	0.604	0.476	1.91E-05	Treg	EIF3H
S100A63	7.08E-10	0.339718	0.76	0.618	1.99E-05	Treg	S100A6
TSC22D33	7.63E-10	0.279715	0.945	0.853	2.15E-05	Treg	TSC22D3
TMEM258:	7.83E-10	0.32053	0.533	0.399	2.20E-05	Treg	TMEM258
COX6B11	7.98E-10	0.268559	0.652	0.532	2.25E-05	Treg	COX6B1
WDR83OS	8.40E-10	0.327853	0.499	0.374	2.36E-05	Treg	WDR83OS
PSMA5	9.15E-10	0.310605	0.387	0.261	2.57E-05	Treg	PSMA5
GYPC3	9.44E-10	0.299094	0.648	0.521	2.65E-05	Treg	GYPC
CCND21	9.91E-10	0.369837	0.314	0.201	2.79E-05	Treg	CCND2
PPP4R2	1.05E-09	0.352844	0.311	0.198	2.95E-05	Treg	PPP4R2
CYTIP1	1.10E-09	0.331298	0.762	0.641	3.08E-05	Treg	CYTIP
OTUB11	1.17E-09	0.340462	0.398	0.275	3.30E-05	Treg	OTUB1
MAP3K51	1.22E-09	0.252199	0.339	0.22	3.44E-05	Treg	MAP3K5
ISG151	1.27E-09	0.596715	0.398	0.279	3.57E-05	Treg	ISG15
CXCR31	1.43E-09	0.326834	0.314	0.198	4.03E-05	Treg	CXCR3
RASGRP1	1.61E-09	0.252462	0.334	0.214	4.54E-05	Treg	RASGRP1
APOO1	2.06E-09	0.341349	0.35	0.225	5.80E-05	Treg	APOO
UBE2N	2.17E-09	0.294503	0.375	0.255	6.10E-05	Treg	UBE2N
RGS102	2.23E-09	0.335282	0.531	0.397	6.26E-05	Treg	RGS10
TPI12	2.24E-09	0.320132	0.636	0.498	6.31E-05	Treg	TPI1
CDV3	2.73E-09	0.322585	0.419	0.298	7.67E-05	Treg	CDV3
CIRBP	2.73E-09	0.366573	0.762	0.635	7.68E-05	Treg	CIRBP
WDR11	2.73E-09	0.353753	0.465	0.343	7.69E-05	Treg	WDR1

CMTM3	2.87E-09	0.338711	0.309	0.199	8.06E-05	Treg	CMTM3
HBP1	3.40E-09	0.301433	0.297	0.188	9.55E-05	Treg	HBP1
SNHG292	3.42E-09	0.323939	0.682	0.563	9.62E-05	Treg	SNHG29
PARP1	3.96E-09	0.376487	0.336	0.224	0.000112	Treg	PARP1
NR3C1	4.02E-09	0.570435	0.435	0.33	0.000113	Treg	NR3C1
SUMO3	4.22E-09	0.335169	0.263	0.163	0.000119	Treg	SUMO3
ARID4A	4.52E-09	0.298786	0.307	0.193	0.000127	Treg	ARID4A
ARPC21	4.85E-09	0.261422	0.764	0.66	0.000136	Treg	ARPC2
RPS27L	4.99E-09	0.447829	0.416	0.303	0.00014	Treg	RPS27L
SNX17	5.00E-09	0.314239	0.318	0.207	0.000141	Treg	SNX17
SLC25A61	5.00E-09	0.319775	0.725	0.616	0.000141	Treg	SLC25A6
DGKA1	5.05E-09	0.286559	0.297	0.188	0.000142	Treg	DGKA
RPS4Y12	5.54E-09	0.364484	0.597	0.469	0.000156	Treg	RPS4Y1
BSG1	6.08E-09	0.324646	0.458	0.337	0.000171	Treg	BSG
TMEM219:	6.54E-09	0.36533	0.371	0.258	0.000184	Treg	TMEM219
ATP5PO1	6.99E-09	0.313542	0.499	0.377	0.000197	Treg	ATP5PO
COX7C3	7.03E-09	0.261481	0.783	0.665	0.000198	Treg	COX7C
GSTK11	7.37E-09	0.344719	0.611	0.502	0.000207	Treg	GSTK1
HIGD2A	7.39E-09	0.317045	0.547	0.437	0.000208	Treg	HIGD2A
PSMB6	8.80E-09	0.354887	0.387	0.275	0.000248	Treg	PSMB6
PAM1	9.11E-09	0.265529	0.277	0.171	0.000256	Treg	PAM
CIB11	9.12E-09	0.339798	0.563	0.439	0.000257	Treg	CIB1
WSB11	1.02E-08	0.501516	0.54	0.416	0.000288	Treg	WSB1
CHCHD21	1.05E-08	0.32548	0.712	0.614	0.000296	Treg	CHCHD2
NDUFA11	1.11E-08	0.360901	0.513	0.401	0.000311	Treg	NDUFA11
PAIP2	1.20E-08	0.325329	0.485	0.367	0.000337	Treg	PAIP2
MAT2B1	1.25E-08	0.344046	0.378	0.267	0.000351	Treg	MAT2B
ETFB	1.34E-08	0.287343	0.261	0.162	0.000376	Treg	ETFB
UQCRB2	1.49E-08	0.26203	0.735	0.605	0.000418	Treg	UQCRB
RNASEK2	1.74E-08	0.328742	0.659	0.537	0.00049	Treg	RNASEK
EMP31	2.22E-08	0.340467	0.554	0.44	0.000624	Treg	EMP3
SARAF1	2.24E-08	0.260927	0.931	0.856	0.00063	Treg	SARAF
UBE2D2	2.34E-08	0.264356	0.622	0.504	0.000659	Treg	UBE2D2
CHURC1	2.37E-08	0.372217	0.458	0.342	0.000666	Treg	CHURC1
G3BP21	2.47E-08	0.368542	0.471	0.347	0.000694	Treg	G3BP2
CFLAR3	2.71E-08	0.268128	0.533	0.4	0.000763	Treg	CFLAR
IVNS1ABP1	2.74E-08	0.412056	0.426	0.315	0.000771	Treg	IVNS1ABP
MX12	2.75E-08	0.41302	0.343	0.234	0.000773	Treg	MX1
EEF23	2.83E-08	0.256438	0.863	0.751	0.000796	Treg	EEF2
ISCU	2.89E-08	0.319067	0.421	0.307	0.000814	Treg	ISCU
NDUFC1	2.93E-08	0.293161	0.272	0.174	0.000825	Treg	NDUFC1
TUT7	3.11E-08	0.301716	0.261	0.164	0.000876	Treg	TUT7
JPT11	3.68E-08	0.296487	0.357	0.251	0.001036	Treg	JPT1
PLIN21	3.82E-08	0.703727	0.314	0.221	0.001074	Treg	PLIN2
RANBP1	3.94E-08	0.351566	0.284	0.187	0.001107	Treg	RANBP1
IRF9	3.98E-08	0.345674	0.346	0.24	0.001119	Treg	IRF9
PPP1R182	3.99E-08	0.374022	0.43	0.322	0.001123	Treg	PPP1R18

MAP4K1	4.07E-08	0.262784	0.286	0.185	0.001144	Treg	MAP4K1
NOP10	4.08E-08	0.343134	0.362	0.255	0.001149	Treg	NOP10
TAP12	4.20E-08	0.350657	0.407	0.293	0.001182	Treg	TAP1
GLCCI12	4.22E-08	0.394921	0.423	0.316	0.001186	Treg	GLCCI1
SOCS32	4.73E-08	0.363707	0.261	0.167	0.001331	Treg	SOCS3
ADD33	4.84E-08	0.297161	0.366	0.257	0.001361	Treg	ADD3
CMTM7	5.15E-08	0.276156	0.336	0.226	0.001448	Treg	CMTM7
ARL6IP11	5.18E-08	0.332215	0.533	0.416	0.001457	Treg	ARL6IP1
UFD1	5.26E-08	0.263123	0.256	0.16	0.00148	Treg	UFD1
CSK2	5.38E-08	0.341449	0.435	0.33	0.001513	Treg	CSK
AP2M11	5.89E-08	0.301828	0.373	0.265	0.001657	Treg	AP2M1
MRFAP1	6.11E-08	0.306343	0.316	0.217	0.00172	Treg	MRFAP1
HNRNPR	6.70E-08	0.27418	0.387	0.276	0.001884	Treg	HNRNPR
ATP5MF	6.95E-08	0.311818	0.444	0.34	0.001955	Treg	ATP5MF
LAMTOR5	7.31E-08	0.26857	0.304	0.203	0.002056	Treg	LAMTOR5
TNFRSF141	7.68E-08	0.35801	0.368	0.264	0.002162	Treg	TNFRSF14
ANXA7	8.03E-08	0.261728	0.32	0.216	0.00226	Treg	ANXA7
EDF11	8.66E-08	0.255351	0.625	0.517	0.002435	Treg	EDF1
GABARAP1	9.42E-08	0.285455	0.652	0.538	0.002652	Treg	GABARAP
SMAP21	1.00E-07	0.260528	0.732	0.599	0.002824	Treg	SMAP2
ARHGEF6	1.05E-07	0.251698	0.275	0.178	0.00296	Treg	ARHGEF6
TMEM59	1.11E-07	0.31873	0.519	0.406	0.003113	Treg	TMEM59
COX14	1.15E-07	0.314854	0.323	0.224	0.00325	Treg	COX14
TALDO1	1.40E-07	0.270124	0.334	0.229	0.003938	Treg	TALDO1
PPP1CC	1.40E-07	0.283208	0.339	0.237	0.003953	Treg	PPP1CC
NAP1L41	1.42E-07	0.298084	0.499	0.385	0.003996	Treg	NAP1L4
NT5C3A	1.45E-07	0.292658	0.256	0.164	0.004072	Treg	NT5C3A
ANXA111	1.52E-07	0.301074	0.474	0.366	0.004267	Treg	ANXA11
DOK22	1.76E-07	0.273603	0.357	0.248	0.004952	Treg	DOK2
ERH	1.89E-07	0.293916	0.396	0.286	0.00532	Treg	ERH
AQP31	2.07E-07	0.282798	0.272	0.175	0.005836	Treg	AQP3
LAT3	2.09E-07	0.348933	0.449	0.357	0.005869	Treg	LAT
AKIRIN21	2.10E-07	0.30615	0.352	0.251	0.005903	Treg	AKIRIN2
MRPS24	2.13E-07	0.278421	0.265	0.174	0.005983	Treg	MRPS24
NDUFB11	2.20E-07	0.291046	0.526	0.413	0.006195	Treg	NDUFB11
CRIP12	2.38E-07	0.267618	0.696	0.586	0.006705	Treg	CRIP1
LY6E3	2.49E-07	0.386412	0.643	0.555	0.007017	Treg	LY6E
HMGN21	2.72E-07	0.29984	0.568	0.468	0.007652	Treg	HMGN2
SRI	2.76E-07	0.353401	0.357	0.256	0.007756	Treg	SRI
KIF5B	3.14E-07	0.261935	0.391	0.282	0.008842	Treg	KIF5B
ATP6V1F1	3.76E-07	0.277744	0.403	0.296	0.010585	Treg	ATP6V1F
HSPB13	4.40E-07	0.304724	0.391	0.284	0.01239	Treg	HSPB1
PSMG2	4.53E-07	0.313361	0.284	0.193	0.012747	Treg	PSMG2
POLD4	5.35E-07	0.287028	0.426	0.321	0.015044	Treg	POLD4
C4orf31	5.53E-07	0.266896	0.465	0.355	0.015563	Treg	C4orf3
ATP5PB1	6.65E-07	0.313728	0.405	0.309	0.018704	Treg	ATP5PB
ARL6IP5	6.79E-07	0.288258	0.595	0.492	0.019103	Treg	ARL6IP5

PFDN2	7.11E-07	0.356799	0.265	0.181	0.020014	Treg	PFDN2
RHOG1	7.18E-07	0.308122	0.442	0.341	0.020212	Treg	RHOG
TMPO	7.51E-07	0.288625	0.254	0.166	0.021127	Treg	TMPO
STAT1	8.26E-07	0.365731	0.371	0.267	0.023255	Treg	STAT1
LCK3	1.38E-06	0.255214	0.613	0.529	0.038959	Treg	LCK
OST42	1.48E-06	0.252929	0.703	0.586	0.04153	Treg	OST4
NDUFA7	1.49E-06	0.278933	0.27	0.186	0.042027	Treg	NDUFA7
CAST2	1.54E-06	0.266197	0.465	0.356	0.043404	Treg	CAST
PIM31	1.57E-06	0.343186	0.277	0.192	0.044107	Treg	PIM3
MDH1	1.71E-06	0.28309	0.311	0.221	0.048221	Treg	MDH1
ECH11	1.75E-06	0.266273	0.412	0.314	0.04911	Treg	ECH1
MZT2A2	1.89E-06	0.294874	0.41	0.315	0.053176	Treg	MZT2A
NDUFB4	2.01E-06	0.303826	0.407	0.314	0.056434	Treg	NDUFB4
TNFSF12	2.19E-06	0.328015	0.33	0.242	0.061618	Treg	TNFSF12
BRK1	2.42E-06	0.266415	0.458	0.367	0.067958	Treg	BRK1
FDFT1	2.44E-06	0.250834	0.291	0.203	0.06864	Treg	FDFT1
EIF3I	2.47E-06	0.309731	0.339	0.246	0.069444	Treg	EIF3I
COX16	2.63E-06	0.27012	0.261	0.177	0.073911	Treg	COX16
RHOC2	2.95E-06	0.417235	0.357	0.276	0.082885	Treg	RHOC
PPP2CA	3.32E-06	0.256604	0.428	0.337	0.09342	Treg	PPP2CA
PSMC5	4.13E-06	0.267036	0.341	0.247	0.116234	Treg	PSMC5
JTB	4.42E-06	0.25187	0.442	0.341	0.12435	Treg	JTB
PDCD6	4.49E-06	0.259338	0.327	0.235	0.12643	Treg	PDCD6
TSTD11	5.04E-06	0.282139	0.362	0.273	0.141808	Treg	TSTD1
COX17	5.80E-06	0.302506	0.3	0.215	0.163302	Treg	COX17
MALT11	7.19E-06	0.306541	0.339	0.253	0.202174	Treg	MALT1
DCTN31	7.87E-06	0.250321	0.327	0.24	0.221369	Treg	DCTN3
MRPL52	8.00E-06	0.282897	0.259	0.18	0.224985	Treg	MRPL52
MPC21	8.21E-06	0.312438	0.297	0.218	0.230888	Treg	MPC2
TMED91	8.59E-06	0.295434	0.343	0.257	0.241718	Treg	TMED9
LRPAP1	1.06E-05	0.288027	0.291	0.215	0.297476	Treg	LRPAP1
PSMA21	1.15E-05	0.300803	0.352	0.273	0.322621	Treg	PSMA2
UBALD21	1.20E-05	0.311594	0.508	0.426	0.338503	Treg	UBALD2
AP001011.	1.43E-05	0.254142	0.279	0.198	0.403078	Treg	AP001011.1
RCAN32	1.71E-05	0.268391	0.297	0.213	0.482278	Treg	RCAN3
NEDD92	1.88E-05	0.275079	0.396	0.309	0.527879	Treg	NEDD9
NDUFS81	2.54E-05	0.25573	0.323	0.24	0.71439	Treg	NDUFS8
EIF5A1	3.55E-05	0.281007	0.412	0.328	1	Treg	EIF5A
S100A102	3.86E-05	0.293638	0.622	0.553	1	Treg	S100A10
LGALS12	6.44E-05	0.370987	0.277	0.205	1	Treg	LGALS1
CMTM61	7.29E-05	0.270102	0.286	0.211	1	Treg	CMTM6
PRDX52	7.83E-05	0.290232	0.357	0.282	1	Treg	PRDX5
DPP7	8.07E-05	0.289975	0.41	0.333	1	Treg	DPP7
TACC11	0.000107	0.271546	0.334	0.262	1	Treg	TACC1
AIP	0.000133	0.252703	0.325	0.252	1	Treg	AIP
LAPTM4A1	0.000138	0.308124	0.293	0.226	1	Treg	LAPTM4A
TMEM256	0.000188	0.253136	0.27	0.204	1	Treg	TMEM256

SERPINB91	0.000237	0.32173	0.394	0.315	1	Treg	SERPINB9
LIME11	0.00025	0.302816	0.449	0.393	1	Treg	LIME1
UBE2L61	0.00029	0.260373	0.378	0.31	1	Treg	UBE2L6
TCEA1	0.00082	0.294026	0.375	0.306	1	Treg	TCEA1
KIT	0	2.673309	0.689	0.005	0	NK.memlik	KIT
PCDH9	0	2.066996	0.405	0.003	0	NK.memlik	PCDH9
IL1R1	3.62E-278	1.47121	0.311	0.005	1.02E-273	NK.memlik	IL1R1
PPP1R9A	1.25E-254	1.658016	0.324	0.006	3.53E-250	NK.memlik	PPP1R9A
AFF3	8.26E-204	2.713698	0.568	0.024	2.32E-199	NK.memlik	AFF3
SPINK2	1.11E-158	1.174885	0.257	0.006	3.12E-154	NK.memlik	SPINK2
TNFRSF181	1.94E-91	1.74825	0.527	0.044	5.47E-87	NK.memlik	TNFRSF18
TOX21	3.58E-91	1.627477	0.378	0.023	1.01E-86	NK.memlik	TOX2
AL136456.:	5.98E-90	3.381565	0.595	0.058	1.68E-85	NK.memlik	AL136456.1
TNFRSF41	1.35E-87	1.651938	0.486	0.039	3.79E-83	NK.memlik	TNFRSF4
SCN1B	7.57E-80	1.524512	0.27	0.013	2.13E-75	NK.memlik	SCN1B
AREG3	1.21E-62	3.196152	0.932	0.231	3.39E-58	NK.memlik	AREG
COL9A2	5.27E-61	0.867949	0.284	0.019	1.48E-56	NK.memlik	COL9A2
IL4I1	1.19E-60	1.874673	0.419	0.041	3.35E-56	NK.memlik	IL4I1
TLE1	1.08E-54	1.473385	0.514	0.066	3.05E-50	NK.memlik	TLE1
SOX4	1.11E-53	1.441694	0.378	0.038	3.12E-49	NK.memlik	SOX4
LINC00299	3.25E-44	1.936773	0.473	0.069	9.15E-40	NK.memlik	LINC00299
SVIL	2.30E-41	1.622869	0.351	0.041	6.48E-37	NK.memlik	SVIL
SSBP2	4.10E-37	1.74806	0.514	0.094	1.15E-32	NK.memlik	SSBP2
PLPP1	3.44E-32	1.3414	0.365	0.055	9.68E-28	NK.memlik	PLPP1
KRT861	2.07E-30	1.328529	0.338	0.05	5.82E-26	NK.memlik	KRT86
DOCK5	4.17E-30	1.117393	0.392	0.065	1.17E-25	NK.memlik	DOCK5
FRY	1.25E-27	0.671417	0.257	0.032	3.52E-23	NK.memlik	FRY
TMIGD21	7.80E-27	1.303888	0.419	0.082	2.19E-22	NK.memlik	TMIGD2
NFKB13	1.24E-26	1.774779	0.892	0.41	3.49E-22	NK.memlik	NFKB1
LST11	1.37E-25	1.606988	0.405	0.084	3.85E-21	NK.memlik	LST1
IL18R1	1.17E-23	1.216045	0.419	0.09	3.30E-19	NK.memlik	IL18R1
AGPAT5	2.61E-23	1.229389	0.432	0.096	7.35E-19	NK.memlik	AGPAT5
NRIP11	6.15E-22	1.193378	0.459	0.114	1.73E-17	NK.memlik	NRIP1
PRMT9	6.82E-22	1.350858	0.5	0.133	1.92E-17	NK.memlik	PRMT9
XCL22	5.31E-21	2.250996	0.527	0.16	1.49E-16	NK.memlik	XCL2
TIMP1	7.02E-21	1.262612	0.473	0.123	1.97E-16	NK.memlik	TIMP1
GRAMD2B	7.29E-21	1.038798	0.297	0.054	2.05E-16	NK.memlik	GRAMD2B
GRASP3	6.44E-20	1.518036	0.486	0.135	1.81E-15	NK.memlik	GRASP
FURIN	2.23E-19	0.940778	0.324	0.065	6.27E-15	NK.memlik	FURIN
CCR6	3.41E-19	1.488629	0.365	0.083	9.60E-15	NK.memlik	CCR6
CCNG2	6.32E-19	0.953872	0.297	0.057	1.78E-14	NK.memlik	CCNG2
HIPK22	1.25E-18	1.258887	0.459	0.127	3.51E-14	NK.memlik	HIPK2
TNFRSF25	2.16E-18	1.118422	0.378	0.094	6.08E-14	NK.memlik	TNFRSF25
KCTD9	2.92E-18	0.623192	0.284	0.053	8.23E-14	NK.memlik	KCTD9
SPAG1	3.32E-18	0.751262	0.27	0.05	9.35E-14	NK.memlik	SPAG1
IL7R2	9.76E-18	1.290358	0.892	0.494	2.75E-13	NK.memlik	IL7R
LTBP3	1.09E-17	0.839397	0.324	0.071	3.06E-13	NK.memlik	LTBP3

NFKBIA3	2.04E-17	1.381449	0.946	0.745	5.73E-13	NK.memlik NFKBIA
CAT	2.46E-17	1.042249	0.419	0.116	6.91E-13	NK.memlik CAT
TCF72	1.19E-16	1.277118	0.568	0.206	3.35E-12	NK.memlik TCF7
IRAK31	2.07E-16	1.20417	0.351	0.087	5.82E-12	NK.memlik IRAK3
ANKRD28	2.70E-16	1.444738	0.473	0.147	7.60E-12	NK.memlik ANKRD28
AC018816.	2.83E-16	0.991904	0.311	0.071	7.96E-12	NK.memlik AC018816.1
ATP8B42	4.80E-16	1.040086	0.324	0.077	1.35E-11	NK.memlik ATP8B4
NCOA7	4.84E-16	1.16697	0.514	0.174	1.36E-11	NK.memlik NCOA7
XCL14	1.03E-15	1.660272	0.541	0.196	2.91E-11	NK.memlik XCL1
TRDC2	1.32E-15	0.879956	0.27	0.056	3.73E-11	NK.memlik TRDC
COQ8A	3.81E-15	0.943499	0.284	0.064	1.07E-10	NK.memlik COQ8A
AHR	1.95E-14	0.919725	0.419	0.125	5.49E-10	NK.memlik AHR
SKAP21	2.34E-14	1.045662	0.405	0.12	6.59E-10	NK.memlik SKAP2
ZFP36L11	2.67E-14	1.337776	0.865	0.583	7.51E-10	NK.memlik ZFP36L1
SEC11A1	3.87E-14	0.98396	0.649	0.289	1.09E-09	NK.memlik SEC11A
RUNX2	5.54E-14	1.234943	0.541	0.203	1.56E-09	NK.memlik RUNX2
IFNGR21	6.49E-14	0.666565	0.297	0.072	1.83E-09	NK.memlik IFNGR2
ARHGAP10	1.02E-13	0.842403	0.324	0.084	2.86E-09	NK.memlik ARHGAP10
MAP3K84	1.21E-13	1.196941	0.703	0.338	3.42E-09	NK.memlik MAP3K8
KLRB14	1.68E-13	1.321584	0.757	0.359	4.72E-09	NK.memlik KLRB1
ZMIZ11	4.94E-13	1.011731	0.324	0.089	1.39E-08	NK.memlik ZMIZ1
ICAM11	5.01E-13	1.025354	0.311	0.083	1.41E-08	NK.memlik ICAM1
MPG	1.03E-12	0.944964	0.446	0.16	2.89E-08	NK.memlik MPG
ZFAND51	1.18E-12	1.204355	0.689	0.376	3.31E-08	NK.memlik ZFAND5
NR4A12	1.74E-12	1.014342	0.635	0.28	4.89E-08	NK.memlik NR4A1
CITED4	2.42E-12	0.538763	0.27	0.066	6.81E-08	NK.memlik CITED4
STAM1	2.60E-12	0.734131	0.365	0.109	7.31E-08	NK.memlik STAM
CD75	3.14E-12	1.101936	0.824	0.52	8.84E-08	NK.memlik CD7
SLC16A31	4.46E-12	0.709684	0.338	0.1	1.25E-07	NK.memlik SLC16A3
ZBTB163	7.75E-12	0.847589	0.554	0.23	2.18E-07	NK.memlik ZBTB16
RIPK2	1.67E-11	0.949712	0.297	0.085	4.70E-07	NK.memlik RIPK2
IL4R	2.11E-11	0.843462	0.324	0.097	5.94E-07	NK.memlik IL4R
AHI1	2.21E-11	1.028518	0.324	0.097	6.21E-07	NK.memlik AHI1
FXYD53	2.48E-11	1.152671	0.851	0.682	6.99E-07	NK.memlik FXYD5
TSC22D34	4.51E-11	0.88514	0.973	0.854	1.27E-06	NK.memlik TSC22D3
PHLDA11	6.17E-11	1.304789	0.405	0.145	1.74E-06	NK.memlik PHLDA1
TPT12	6.90E-11	0.669181	1	0.922	1.94E-06	NK.memlik TPT1
REEP3	8.86E-11	0.766599	0.324	0.1	2.49E-06	NK.memlik REEP3
EEF1A13	1.18E-10	0.661253	0.973	0.95	3.33E-06	NK.memlik EEF1A1
XYLT1	1.27E-10	0.895296	0.405	0.146	3.56E-06	NK.memlik XYLT1
JMY	2.78E-10	1.417268	0.5	0.229	7.81E-06	NK.memlik JMY
UNC93B11	5.36E-10	0.621851	0.27	0.078	1.51E-05	NK.memlik UNC93B1
PABPC12	6.54E-10	0.721191	0.959	0.805	1.84E-05	NK.memlik PABPC1
ANKRD10	7.13E-10	0.748176	0.486	0.203	2.01E-05	NK.memlik ANKRD10
ATP10A	8.16E-10	0.575898	0.311	0.097	2.29E-05	NK.memlik ATP10A
KDM1A	1.75E-09	0.538319	0.284	0.087	4.92E-05	NK.memlik KDM1A
DUSP13	1.81E-09	0.91534	0.959	0.841	5.10E-05	NK.memlik DUSP1

SELENOS	2.07E-09	0.639764	0.419	0.163	5.83E-05	NK.memlik SELENOS
FOS2	2.11E-09	0.859316	0.986	0.821	5.93E-05	NK.memlik FOS
MAFF3	2.35E-09	0.840388	0.459	0.194	6.62E-05	NK.memlik MAFF
TTN	3.12E-09	0.550202	0.257	0.074	8.79E-05	NK.memlik TTN
PRR51	3.28E-09	0.850911	0.392	0.16	9.23E-05	NK.memlik PRR5
FOSB1	3.83E-09	1.131923	0.851	0.585	0.000108	NK.memlik FOSB
JUND3	4.65E-09	0.919395	0.932	0.812	0.000131	NK.memlik JUND
PIM23	5.08E-09	1.099481	0.486	0.224	0.000143	NK.memlik PIM2
B4GALT5	5.41E-09	0.603808	0.27	0.083	0.000152	NK.memlik B4GALT5
PRNP1	7.46E-09	0.863352	0.473	0.211	0.00021	NK.memlik PRNP
TYROBP3	7.79E-09	0.52399	0.676	0.312	0.000219	NK.memlik TYROBP
NSMCE1	9.42E-09	0.639646	0.392	0.154	0.000265	NK.memlik NSMCE1
NCALD2	1.12E-08	0.754989	0.446	0.179	0.000316	NK.memlik NCALD
GSN1	1.19E-08	0.559461	0.338	0.116	0.000335	NK.memlik GSN
ST3GAL1	1.94E-08	0.670554	0.568	0.284	0.000547	NK.memlik ST3GAL1
SNHG293	2.88E-08	0.821364	0.743	0.564	0.00081	NK.memlik SNHG29
HNRNPA02	2.99E-08	0.897887	0.716	0.471	0.000843	NK.memlik HNRNPA0
HMGN33	3.74E-08	0.599124	0.527	0.246	0.001051	NK.memlik HMGN3
WDFY21	4.33E-08	0.562702	0.324	0.118	0.001218	NK.memlik WDFY2
FCER1G3	4.91E-08	0.636304	0.446	0.185	0.001383	NK.memlik FCER1G
DNAAF21	7.45E-08	0.823317	0.365	0.154	0.002096	NK.memlik DNAAF2
LAT21	7.73E-08	0.539585	0.27	0.09	0.002174	NK.memlik LAT2
PER11	8.18E-08	0.763315	0.635	0.362	0.002301	NK.memlik PER1
EPAS11	8.90E-08	0.654599	0.351	0.134	0.002504	NK.memlik EPAS1
BACH22	9.06E-08	0.759834	0.486	0.231	0.002549	NK.memlik BACH2
EEF1G3	1.31E-07	0.677649	0.865	0.692	0.003688	NK.memlik EEF1G
BCL61	1.50E-07	1.031877	0.311	0.125	0.004229	NK.memlik BCL6
AC253572.	1.78E-07	0.855243	0.284	0.104	0.005009	NK.memlik AC253572.2
HDAC91	2.18E-07	0.607658	0.257	0.087	0.006144	NK.memlik HDAC9
RIN33	2.20E-07	0.524017	0.405	0.175	0.006186	NK.memlik RIN3
PABPC41	2.77E-07	0.764341	0.405	0.184	0.007793	NK.memlik PABPC4
SSH11	2.87E-07	0.509903	0.257	0.089	0.008088	NK.memlik SSH1
OTUD5	2.95E-07	0.699831	0.351	0.147	0.008302	NK.memlik OTUD5
NPLOC4	3.15E-07	0.458744	0.284	0.104	0.008855	NK.memlik NPLOC4
CTNNB12	3.35E-07	0.867025	0.486	0.256	0.009423	NK.memlik CTNNB1
AQP32	3.44E-07	0.865639	0.392	0.176	0.009691	NK.memlik AQP3
PRPF6	3.56E-07	0.730117	0.405	0.183	0.010029	NK.memlik PRPF6
LTB3	4.73E-07	1.262875	0.554	0.354	0.013301	NK.memlik LTB
ZCCHC2	5.74E-07	0.49021	0.297	0.111	0.016165	NK.memlik ZCCHC2
HIPK1	5.80E-07	0.67185	0.392	0.171	0.016308	NK.memlik HIPK1
SEC14L11	6.09E-07	1.137081	0.351	0.156	0.017148	NK.memlik SEC14L1
RPL83	6.26E-07	0.447503	0.932	0.886	0.017628	NK.memlik RPL8
CHKA1	7.91E-07	0.685683	0.257	0.094	0.022253	NK.memlik CHKA
BTBD11	8.07E-07	0.569893	0.284	0.106	0.022715	NK.memlik BTBD11
NFKB2	8.94E-07	0.442296	0.297	0.113	0.025145	NK.memlik NFKB2
AC020916.	9.24E-07	0.573112	0.527	0.27	0.025992	NK.memlik AC020916.1
H2AFY1	9.92E-07	0.828178	0.473	0.256	0.027925	NK.memlik H2AFY

TMEM243:	1.04E-06	0.71565	0.473	0.237	0.029385	NK.memlik	TMEM243
RPS3A3	1.16E-06	0.414719	0.986	0.903	0.032517	NK.memlik	RPS3A
MOB3A	1.17E-06	0.388418	0.324	0.13	0.032822	NK.memlik	MOB3A
SEPTIN11	1.27E-06	0.49594	0.257	0.094	0.035864	NK.memlik	SEPTIN11
CASP3	1.32E-06	0.521924	0.311	0.125	0.037012	NK.memlik	CASP3
ELK3	1.46E-06	0.700994	0.27	0.105	0.041055	NK.memlik	ELK3
RACK13	1.97E-06	0.46602	0.919	0.847	0.055357	NK.memlik	RACK1
POR	2.29E-06	0.633865	0.257	0.096	0.064324	NK.memlik	POR
RPL92	2.43E-06	0.518485	0.905	0.825	0.068371	NK.memlik	RPL9
PLCB11	2.62E-06	0.446553	0.351	0.146	0.073858	NK.memlik	PLCB1
MDFIC1	2.86E-06	0.559792	0.514	0.278	0.080454	NK.memlik	MDFIC
AC012447.	3.06E-06	0.780665	0.297	0.123	0.086099	NK.memlik	AC012447.1
MAML31	3.07E-06	0.788792	0.257	0.1	0.086298	NK.memlik	MAML3
MGAT51	3.09E-06	0.413298	0.514	0.26	0.086888	NK.memlik	MGAT5
SYPL1	3.23E-06	0.392023	0.311	0.126	0.090829	NK.memlik	SYPL1
LRRFIP2	3.31E-06	0.4203	0.27	0.103	0.09306	NK.memlik	LRRFIP2
RPS93	3.35E-06	0.417749	0.959	0.856	0.094263	NK.memlik	RPS9
TMEM123:	3.94E-06	0.853384	0.486	0.273	0.110788	NK.memlik	TMEM123
AC245014.	4.18E-06	0.580975	0.311	0.132	0.117519	NK.memlik	AC245014.3
ABCC11	4.32E-06	0.678322	0.392	0.191	0.121624	NK.memlik	ABCC1
MIS18BP11	4.41E-06	0.736872	0.392	0.201	0.124166	NK.memlik	MIS18BP1
HTATSF1	4.72E-06	0.609434	0.297	0.125	0.13273	NK.memlik	HTATSF1
PFKFB32	4.81E-06	0.650321	0.554	0.323	0.135416	NK.memlik	PFKFB3
ADI1	4.96E-06	0.596205	0.257	0.101	0.139553	NK.memlik	ADI1
RPL53	5.06E-06	0.488719	0.919	0.849	0.142302	NK.memlik	RPL5
CSRNP12	5.18E-06	0.633024	0.689	0.427	0.145749	NK.memlik	CSRNP1
RHOB1	5.23E-06	0.659503	0.473	0.242	0.14727	NK.memlik	RHOB
ERGIC3	5.23E-06	0.53821	0.432	0.22	0.147286	NK.memlik	ERGIC3
IFNGR12	5.29E-06	0.689356	0.514	0.276	0.148801	NK.memlik	IFNGR1
ITGB1	5.60E-06	0.605353	0.595	0.365	0.157651	NK.memlik	ITGB1
LINC00910	6.67E-06	0.892225	0.446	0.246	0.187547	NK.memlik	LINC00910
EEF24	7.67E-06	0.607018	0.851	0.752	0.215818	NK.memlik	EEF2
ID22	7.84E-06	0.828842	0.757	0.554	0.220492	NK.memlik	ID2
GNAQ1	8.53E-06	0.460823	0.365	0.167	0.239913	NK.memlik	GNAQ
RPL72	8.76E-06	0.569771	0.838	0.65	0.246531	NK.memlik	RPL7
RNF1301	8.81E-06	0.60265	0.324	0.145	0.247916	NK.memlik	RNF130
AC007952.	8.89E-06	0.752253	0.392	0.195	0.250101	NK.memlik	AC007952.4
CDC14A1	8.95E-06	0.262669	0.527	0.28	0.251814	NK.memlik	CDC14A
IFITM33	8.96E-06	0.661438	0.446	0.231	0.251979	NK.memlik	IFITM3
BCL31	9.04E-06	0.524622	0.257	0.1	0.254247	NK.memlik	BCL3
SERTAD2	9.92E-06	0.419519	0.27	0.109	0.279064	NK.memlik	SERTAD2
SOCS11	1.15E-05	0.733829	0.649	0.427	0.323343	NK.memlik	SOCS1
SESN1	1.28E-05	0.753273	0.338	0.16	0.359912	NK.memlik	SESN1
RPL154	1.32E-05	0.482455	0.905	0.845	0.372338	NK.memlik	RPL15
RERE	1.50E-05	0.707558	0.405	0.214	0.422405	NK.memlik	RERE
TTC7A1	1.56E-05	0.513552	0.284	0.121	0.437969	NK.memlik	TTC7A
PCNX4	1.60E-05	0.721953	0.297	0.134	0.450521	NK.memlik	PCNX4

RAB11FIP1	1.65E-05	0.619414	0.297	0.132	0.465627	NK.memlik	RAB11FIP1
CCDC57	2.18E-05	0.333298	0.297	0.127	0.612785	NK.memlik	CCDC57
TEX141	2.23E-05	0.851557	0.541	0.321	0.628385	NK.memlik	TEX14
TNFAIP32	2.42E-05	0.58107	0.919	0.78	0.682224	NK.memlik	TNFAIP3
GADD45A2	2.48E-05	0.817451	0.338	0.165	0.699158	NK.memlik	GADD45A
ZBED4	2.89E-05	0.480612	0.284	0.124	0.813251	NK.memlik	ZBED4
MED30	3.16E-05	0.488966	0.324	0.153	0.888844	NK.memlik	MED30
ELL23	3.24E-05	0.608978	0.649	0.418	0.911717	NK.memlik	ELL2
MED13L2	3.30E-05	0.675972	0.419	0.221	0.928767	NK.memlik	MED13L
RPL223	3.45E-05	0.405607	0.878	0.8	0.972151	NK.memlik	RPL22
CRY11	3.47E-05	0.513145	0.365	0.176	0.977511	NK.memlik	CRY1
BHLHE402	3.64E-05	0.770229	0.514	0.328	1	NK.memlik	BHLHE40
COA4	4.39E-05	0.319067	0.257	0.106	1	NK.memlik	COA4
ARL4A1	4.45E-05	0.61542	0.338	0.17	1	NK.memlik	ARL4A
IER23	4.62E-05	0.583384	0.892	0.695	1	NK.memlik	IER2
LINC02256	4.73E-05	0.541118	0.257	0.112	1	NK.memlik	LINC02256
ERCC11	4.76E-05	0.41497	0.432	0.23	1	NK.memlik	ERCC1
PGLS	4.97E-05	0.542875	0.419	0.226	1	NK.memlik	PGLS
RPLP12	5.03E-05	0.410348	0.986	0.949	1	NK.memlik	RPLP1
MAPK8	5.11E-05	0.442506	0.324	0.152	1	NK.memlik	MAPK8
PPP1R15A2	5.49E-05	0.49549	0.824	0.582	1	NK.memlik	PPP1R15A
RELB	5.53E-05	0.409612	0.338	0.163	1	NK.memlik	RELB
CACUL11	5.56E-05	0.415188	0.27	0.118	1	NK.memlik	CACUL1
ILK	5.66E-05	0.361742	0.311	0.145	1	NK.memlik	ILK
SQSTM14	5.73E-05	0.363803	0.662	0.424	1	NK.memlik	SQSTM1
ESYT21	6.46E-05	0.470898	0.459	0.256	1	NK.memlik	ESYT2
PHACTR21	6.92E-05	0.316293	0.392	0.197	1	NK.memlik	PHACTR2
ERGIC12	7.30E-05	0.454653	0.324	0.156	1	NK.memlik	ERGIC1
BST23	7.51E-05	0.627602	0.554	0.358	1	NK.memlik	BST2
PTOV1	7.60E-05	0.422475	0.284	0.131	1	NK.memlik	PTOV1
SNHG25	7.90E-05	0.451473	0.378	0.192	1	NK.memlik	SNHG25
NFKBIZ4	8.22E-05	0.669757	0.608	0.396	1	NK.memlik	NFKBIZ
FLOT11	8.26E-05	0.524803	0.297	0.141	1	NK.memlik	FLOT1
OSTC	8.83E-05	0.463897	0.446	0.246	1	NK.memlik	OSTC
NINJ12	9.40E-05	0.620197	0.311	0.158	1	NK.memlik	NINJ1
NME3	0.000114	0.353796	0.459	0.259	1	NK.memlik	NME3
HSP90AB13	0.000116	0.495007	0.838	0.727	1	NK.memlik	HSP90AB1
RPS6KA31	0.000137	0.547662	0.514	0.312	1	NK.memlik	RPS6KA3
LYN3	0.000164	0.351237	0.297	0.14	1	NK.memlik	LYN
RPS233	0.000167	0.321901	0.946	0.916	1	NK.memlik	RPS23
CHD71	0.00017	0.626693	0.257	0.116	1	NK.memlik	CHD7
IMPDH2	0.000174	0.375095	0.284	0.134	1	NK.memlik	IMPDH2
SVIP	0.000183	0.326491	0.284	0.132	1	NK.memlik	SVIP
TUBB1	0.000185	0.426043	0.527	0.318	1	NK.memlik	TUBB
RPS83	0.000188	0.310223	0.973	0.918	1	NK.memlik	RPS8
RPL10A3	0.000189	0.430377	0.905	0.822	1	NK.memlik	RPL10A
RAB11B1	0.000189	0.446027	0.459	0.261	1	NK.memlik	RAB11B

CD831	0.0002	0.739882	0.459	0.273	1	NK.memlik	CD83
HNRNPA12	0.000209	0.474512	0.838	0.73	1	NK.memlik	HNRNPA1
TPM41	0.000218	0.528968	0.419	0.239	1	NK.memlik	TPM4
CRTAP1	0.000236	0.308151	0.27	0.126	1	NK.memlik	CRTAP
ZFP363	0.000255	0.503082	0.919	0.877	1	NK.memlik	ZFP36
UGCG	0.000266	0.414524	0.27	0.127	1	NK.memlik	UGCG
RALBP1	0.000266	0.427277	0.351	0.186	1	NK.memlik	RALBP1
IRF2BP2	0.000268	0.501226	0.324	0.173	1	NK.memlik	IRF2BP2
DCAF11	0.000268	0.366352	0.257	0.121	1	NK.memlik	DCAF11
RPS133	0.00027	0.303996	0.919	0.893	1	NK.memlik	RPS13
OCIAD1	0.000297	0.377724	0.473	0.278	1	NK.memlik	OCIAD1
31-Mar	0.000303	0.582739	0.284	0.142	1	NK.memlik	3-Mar
TESPA12	0.000304	0.435662	0.338	0.173	1	NK.memlik	TESPA1
RBMS11	0.000309	0.724196	0.541	0.38	1	NK.memlik	RBMS1
CCNI1	0.000318	0.44199	0.77	0.631	1	NK.memlik	CCNI
STK24	0.00032	0.376625	0.473	0.281	1	NK.memlik	STK24
PNRC12	0.000324	0.422798	0.919	0.799	1	NK.memlik	PNRC1
YWHAH1	0.000361	0.257318	0.378	0.201	1	NK.memlik	YWHAH
TDG	0.000367	0.511527	0.27	0.134	1	NK.memlik	TDG
GLUL1	0.00039	0.459667	0.365	0.203	1	NK.memlik	GLUL
CD811	0.000406	0.676234	0.73	0.599	1	NK.memlik	CD81
ARID1B1	0.000411	0.601188	0.514	0.33	1	NK.memlik	ARID1B
DDX3X1	0.000413	0.574259	0.635	0.443	1	NK.memlik	DDX3X
RPS243	0.000434	0.298633	0.973	0.915	1	NK.memlik	RPS24
TRBC12	0.000466	0.63468	0.5	0.327	1	NK.memlik	TRBC1
ADAM10	0.00047	0.726447	0.405	0.242	1	NK.memlik	ADAM10
SLC25A39	0.0005	0.290718	0.297	0.152	1	NK.memlik	SLC25A39
INPP4A2	0.000509	0.526109	0.419	0.253	1	NK.memlik	INPP4A
SMAP22	0.000525	0.531261	0.743	0.601	1	NK.memlik	SMAP2
BIRC31	0.000528	0.646169	0.27	0.132	1	NK.memlik	BIRC3
RPL143	0.000577	0.300453	0.932	0.896	1	NK.memlik	RPL14
LTA4H	0.00058	0.254688	0.257	0.122	1	NK.memlik	LTA4H
TCIRG12	0.000589	0.34582	0.392	0.219	1	NK.memlik	TCIRG1
NAP1L1	0.00059	0.468817	0.541	0.368	1	NK.memlik	NAP1L1
RASA22	0.000594	0.373186	0.608	0.412	1	NK.memlik	RASA2
TGIF1	0.000626	0.337934	0.27	0.134	1	NK.memlik	TGIF1
EIF2AK1	0.000652	0.500349	0.257	0.127	1	NK.memlik	EIF2AK1
LPXN	0.000672	0.414137	0.473	0.302	1	NK.memlik	LPXN
IRS21	0.000684	0.516527	0.284	0.147	1	NK.memlik	IRS2
RORA2	0.000695	0.672859	0.581	0.422	1	NK.memlik	RORA
FNDC3B1	0.000698	0.347642	0.297	0.152	1	NK.memlik	FNDC3B
PABPN1	0.000734	0.406112	0.554	0.37	1	NK.memlik	PABPN1
THEM4	0.000757	0.26223	0.27	0.131	1	NK.memlik	THEM4
RPL323	0.00078	0.262887	0.946	0.926	1	NK.memlik	RPL32
RPS52	0.000783	0.382427	0.865	0.838	1	NK.memlik	RPS5
UXT1	0.000854	0.430131	0.568	0.381	1	NK.memlik	UXT
CCT4	0.000896	0.389872	0.5	0.31	1	NK.memlik	CCT4

UBASH3B1	0.000903	0.473979	0.284	0.148	1	NK.memlik	UBASH3B
TMEM167/	0.000922	0.329063	0.257	0.126	1	NK.memlik	TMEM167A
ELOVL51	0.000956	0.319109	0.486	0.308	1	NK.memlik	ELOVL5
NORAD	0.000956	0.485395	0.284	0.149	1	NK.memlik	NORAD
SYNCRIP	0.000965	0.394748	0.365	0.21	1	NK.memlik	SYNCRIP
ETV61	0.000969	0.417104	0.338	0.191	1	NK.memlik	ETV6
WDR741	0.000999	0.44178	0.365	0.218	1	NK.memlik	WDR74
ARHGEF71	0.001044	0.513565	0.405	0.251	1	NK.memlik	ARHGEF7
Z93241.11	0.001056	0.575073	0.311	0.17	1	NK.memlik	Z93241.1
COMMD1	0.001074	0.330591	0.311	0.167	1	NK.memlik	COMMD1
DUSP161	0.001083	0.375037	0.27	0.136	1	NK.memlik	DUSP16
TMEM165	0.001146	0.263564	0.338	0.182	1	NK.memlik	TMEM165
CNST1	0.001175	0.442591	0.311	0.17	1	NK.memlik	CNST
HSH2D2	0.001193	0.570766	0.257	0.127	1	NK.memlik	HSH2D
SMARCA22	0.001219	0.602667	0.527	0.372	1	NK.memlik	SMARCA2
PDE4A	0.001233	0.375032	0.284	0.148	1	NK.memlik	PDE4A
MEF2A1	0.001233	0.476766	0.351	0.197	1	NK.memlik	MEF2A
GRAMD1A	0.001271	0.31951	0.365	0.207	1	NK.memlik	GRAMD1A
MMP24OS	0.001335	0.465351	0.392	0.228	1	NK.memlik	MMP24OS
RPL293	0.001389	0.279202	0.959	0.897	1	NK.memlik	RPL29
G3BP1	0.001444	0.489538	0.365	0.224	1	NK.memlik	G3BP1
CAPG1	0.001524	0.460385	0.311	0.173	1	NK.memlik	CAPG
NME22	0.001539	0.439753	0.581	0.407	1	NK.memlik	NME2
SF3B4	0.001576	0.403766	0.324	0.183	1	NK.memlik	SF3B4
NR1D2	0.001586	0.430534	0.351	0.206	1	NK.memlik	NR1D2
RPL133	0.001599	0.255454	0.986	0.941	1	NK.memlik	RPL13
EIF3L2	0.001602	0.504306	0.527	0.38	1	NK.memlik	EIF3L
SNRNP701	0.001625	0.442484	0.459	0.299	1	NK.memlik	SNRNP70
NOP532	0.001636	0.354419	0.73	0.545	1	NK.memlik	NOP53
SLTM1	0.001648	0.365049	0.5	0.331	1	NK.memlik	SLTM
HIVEP21	0.001681	0.387821	0.419	0.25	1	NK.memlik	HIVEP2
TAGLN24	0.001711	0.474323	0.676	0.502	1	NK.memlik	TAGLN2
HDLBP	0.001732	0.375719	0.297	0.158	1	NK.memlik	HDLBP
ODF2L	0.001781	0.559294	0.351	0.208	1	NK.memlik	ODF2L
ZNF2922	0.001783	0.354377	0.432	0.26	1	NK.memlik	ZNF292
STX16	0.001827	0.304956	0.324	0.177	1	NK.memlik	STX16
CANX1	0.001902	0.311802	0.473	0.303	1	NK.memlik	CANX
AC079793.	0.001959	0.621882	0.284	0.159	1	NK.memlik	AC079793.1
NR4A33	0.001962	0.569131	0.473	0.312	1	NK.memlik	NR4A3
ITGB71	0.002013	0.348317	0.324	0.182	1	NK.memlik	ITGB7
SET	0.002023	0.399883	0.581	0.429	1	NK.memlik	SET
CTR9	0.002174	0.475487	0.297	0.17	1	NK.memlik	CTR9
PRKCSH	0.002335	0.321822	0.311	0.175	1	NK.memlik	PRKCSH
RPL43	0.002375	0.356355	0.824	0.718	1	NK.memlik	RPL4
CMTM62	0.002417	0.3987	0.351	0.211	1	NK.memlik	CMTM6
TRAF5	0.002431	0.3587	0.297	0.164	1	NK.memlik	TRAF5
VPS13B2	0.002487	0.331185	0.405	0.247	1	NK.memlik	VPS13B

FPGS	0.002576	0.445571	0.257	0.135	1	NK.memlik FPGS
NDUFAF3	0.002627	0.307854	0.365	0.214	1	NK.memlik NDUFAF3
RTN42	0.002674	0.635548	0.446	0.287	1	NK.memlik RTN4
AGFG1	0.002703	0.286777	0.257	0.135	1	NK.memlik AGFG1
HSP90B11	0.002737	0.519998	0.622	0.488	1	NK.memlik HSP90B1
ABCB12	0.00282	0.297059	0.284	0.154	1	NK.memlik ABCB1
SMAD2	0.002869	0.309054	0.324	0.183	1	NK.memlik SMAD2
TM9SF3	0.003042	0.447579	0.365	0.225	1	NK.memlik TM9SF3
EPB41L4A-.	0.003098	0.319535	0.257	0.136	1	NK.memlik EPB41L4A-AS1
PAPOLA	0.003216	0.294032	0.432	0.278	1	NK.memlik PAPOLA
GOLPH3	0.003229	0.282506	0.378	0.23	1	NK.memlik GOLPH3
CITED22	0.003235	0.661486	0.622	0.494	1	NK.memlik CITED2
PRDX53	0.003246	0.399022	0.432	0.283	1	NK.memlik PRDX5
RASA11	0.00335	0.382936	0.446	0.293	1	NK.memlik RASA1
NFATC1	0.003395	0.287359	0.27	0.148	1	NK.memlik NFATC1
CTSW4	0.003486	0.517367	0.649	0.506	1	NK.memlik CTSW
PCBP2	0.003489	0.289723	0.527	0.346	1	NK.memlik PCBP2
SFPQ	0.003555	0.357826	0.743	0.578	1	NK.memlik SFPQ
SMARCC2	0.00358	0.351686	0.297	0.17	1	NK.memlik SMARCC2
NCL1	0.003581	0.364529	0.716	0.611	1	NK.memlik NCL
AL138963.4	0.003682	0.395288	0.311	0.176	1	NK.memlik AL138963.4
CROCC1	0.003731	0.591583	0.324	0.19	1	NK.memlik CROCC
USP7	0.003761	0.435117	0.257	0.142	1	NK.memlik USP7
NECAP2	0.004033	0.289717	0.27	0.151	1	NK.memlik NECAP2
UBB4	0.004052	0.28212	0.838	0.692	1	NK.memlik UBB
GATA31	0.004181	0.432888	0.378	0.237	1	NK.memlik GATA3
RPSA3	0.004246	0.253236	0.932	0.823	1	NK.memlik RPSA
SRGN3	0.004304	0.612747	0.905	0.868	1	NK.memlik SRGN
ILF31	0.00435	0.253225	0.432	0.272	1	NK.memlik ILF3
DDX3Y1	0.004453	0.271408	0.365	0.215	1	NK.memlik DDX3Y
SELENOH1	0.004592	0.29612	0.459	0.31	1	NK.memlik SELENOH
TRIM44	0.004615	0.258609	0.27	0.15	1	NK.memlik TRIM44
SPCS3	0.004619	0.383771	0.378	0.238	1	NK.memlik SPCS3
CHMP1B	0.004645	0.509816	0.351	0.226	1	NK.memlik CHMP1B
CYCS3	0.004902	0.554695	0.541	0.402	1	NK.memlik CYCS
HSPB14	0.004902	0.705119	0.405	0.285	1	NK.memlik HSPB1
RPL64	0.004927	0.25784	0.959	0.892	1	NK.memlik RPL6
PEBP11	0.004948	0.339077	0.5	0.353	1	NK.memlik PEBP1
BOD1L1	0.004975	0.446289	0.405	0.264	1	NK.memlik BOD1L1
ALKBH7	0.004995	0.359217	0.392	0.244	1	NK.memlik ALKBH7
CUX11	0.005201	0.487696	0.27	0.157	1	NK.memlik CUX1
PIM32	0.005203	0.31505	0.324	0.192	1	NK.memlik PIM3
ANP32B3	0.005277	0.305657	0.581	0.399	1	NK.memlik ANP32B
TXK3	0.005299	0.316874	0.284	0.162	1	NK.memlik TXK
SMYD32	0.005488	0.317547	0.392	0.247	1	NK.memlik SMYD3
UBE3A	0.005556	0.586926	0.351	0.229	1	NK.memlik UBE3A
RPL344	0.005817	0.275668	0.973	0.915	1	NK.memlik RPL34

TIPARP2	0.00597	0.385689	0.405	0.264	1	NK.memlik TIPARP
HSPA53	0.006046	0.273041	0.743	0.579	1	NK.memlik HSPA5
IFITM23	0.006244	0.6578	0.757	0.634	1	NK.memlik IFITM2
CTSA	0.006273	0.431365	0.257	0.148	1	NK.memlik CTSA
MAML22	0.006315	0.276026	0.5	0.335	1	NK.memlik MAML2
HNRNPC	0.006473	0.290924	0.689	0.532	1	NK.memlik HNRNPC
CPSF6	0.006691	0.412403	0.338	0.208	1	NK.memlik CPSF6
AKT31	0.006776	0.536707	0.284	0.172	1	NK.memlik AKT3
DDIT42	0.006786	0.520997	0.757	0.686	1	NK.memlik DDIT4
MAN1A11	0.006818	0.31813	0.297	0.177	1	NK.memlik MAN1A1
ARGLU1	0.006829	0.324138	0.622	0.451	1	NK.memlik ARGLU1
GAS52	0.006914	0.277675	0.635	0.466	1	NK.memlik GAS5
VDAC2	0.00693	0.266268	0.446	0.3	1	NK.memlik VDAC2
PTGDR2	0.007073	0.266812	0.284	0.16	1	NK.memlik PTGDR
RPL313	0.007116	0.263646	0.811	0.73	1	NK.memlik RPL31
CKLF2	0.007153	0.494913	0.5	0.365	1	NK.memlik CKLF
RPL314	0.007189	0.265034	0.946	0.889	1	NK.memlik RPL3
GTF2F1	0.007604	0.440488	0.27	0.159	1	NK.memlik GTF2F1
STAG21	0.007738	0.3927	0.392	0.256	1	NK.memlik STAG2
SATB12	0.008401	0.376558	0.27	0.159	1	NK.memlik SATB1
BZW13	0.008477	0.463999	0.527	0.398	1	NK.memlik BZW1
NUMA1	0.008502	0.27146	0.297	0.179	1	NK.memlik NUMA1
CIRBP1	0.008777	0.360933	0.757	0.636	1	NK.memlik CIRBP
PNPLA2	0.008839	0.273767	0.419	0.284	1	NK.memlik PNPLA2
ITPR1	0.009071	0.280964	0.27	0.159	1	NK.memlik ITPR1
CDK2AP21	0.009135	0.293859	0.324	0.199	1	NK.memlik CDK2AP2
DHRS72	0.009162	0.540335	0.473	0.352	1	NK.memlik DHRS7
PPP1R101	0.009269	0.365973	0.419	0.281	1	NK.memlik PPP1R10
NACA2	0.009595	0.275588	0.878	0.848	1	NK.memlik NACA
PTGER21	0.009633	0.409243	0.311	0.194	1	NK.memlik PTGER2

Supplementary Table 6: DEGs for Figure 5K

	p_val	avg_log2FC	pct.1	pct.2	p_val_adj	cluster	gene
Nav2	0	2.539209	0.866	0.545	0	MG-Homo	Nav2
Csmd3	0	2.655197	0.654	0.337	0	MG-Homo	Csmd3
Nav3	0	2.339172	0.724	0.408	0	MG-Homo	Nav3
Plcl1	0	2.724035	0.718	0.414	0	MG-Homo	Plcl1
Rapgef5	0	2.102347	0.814	0.54	0	MG-Homo	Rapgef5
4933406I1	0	1.976345	0.703	0.442	0	MG-Homo	4933406I18Rik
Maml3	0	2.478869	0.749	0.492	0	MG-Homo	Maml3
Plxna4	0	2.725398	0.46	0.204	0	MG-Homo	Plxna4
Magi1	0	2.677692	0.468	0.214	0	MG-Homo	Magi1
Atp8a2	0	2.169546	0.62	0.376	0	MG-Homo	Atp8a2
Large1	0	2.133728	0.651	0.409	0	MG-Homo	Large1
Tanc2	0	1.96902	0.931	0.69	0	MG-Homo	Tanc2
Ldlrad4	0	2.191243	0.834	0.594	0	MG-Homo	Ldlrad4
Chn2	0	2.272715	0.551	0.312	0	MG-Homo	Chn2
Mir99ahg	0	2.010764	0.744	0.506	0	MG-Homo	Mir99ahg
Slc24a3	0	2.559683	0.414	0.178	0	MG-Homo	Slc24a3
Cacnb2	0	2.291388	0.536	0.304	0	MG-Homo	Cacnb2
Snx29	0	2.192841	0.718	0.487	0	MG-Homo	Snx29
Plxdc2	0	2.058391	0.951	0.725	0	MG-Homo	Plxdc2
Pag1	0	1.903396	0.827	0.603	0	MG-Homo	Pag1
Fhit	0	2.522337	0.446	0.224	0	MG-Homo	Fhit
Klf12	0	2.540174	0.401	0.18	0	MG-Homo	Klf12
Nfia	0	2.229186	0.578	0.358	0	MG-Homo	Nfia
Prkn	0	2.366923	0.457	0.241	0	MG-Homo	Prkn
Frmd4a	0	1.915255	0.816	0.6	0	MG-Homo	Frmd4a
Pde3b	0	1.905062	0.82	0.61	0	MG-Homo	Pde3b
Gm2629	0	1.814451	0.619	0.411	0	MG-Homo	Gm2629
Arhgap22	0	2.269731	0.498	0.294	0	MG-Homo	Arhgap22
Numb	0	1.678061	0.76	0.556	0	MG-Homo	Numb
Dock4	0	2.0869	0.862	0.662	0	MG-Homo	Dock4
Ptprm	0	2.296291	0.403	0.205	0	MG-Homo	Ptprm
Agmo	0	1.91625	0.538	0.341	0	MG-Homo	Agmo
Maml2	0	2.085426	0.646	0.451	0	MG-Homo	Maml2
Inpp4b	0	2.025056	0.659	0.466	0	MG-Homo	Inpp4b
Gm10790	0	1.56537	0.655	0.462	0	MG-Homo	Gm10790
Gm26917	0	2.664325	0.586	0.393	0	MG-Homo	Gm26917
Zfhx3	0	1.829228	0.885	0.693	0	MG-Homo	Zfhx3
Ank2	0	1.991714	0.481	0.296	0	MG-Homo	Ank2
Prkca	0	1.984682	0.49	0.305	0	MG-Homo	Prkca
P3h2	0	1.984945	0.509	0.324	0	MG-Homo	P3h2
Mertk	0	1.63361	0.877	0.693	0	MG-Homo	Mertk
Ophn1	0	1.774437	0.751	0.567	0	MG-Homo	Ophn1

Fat3	0	2.520875	0.348	0.165	0	MG-Homo	Fat3
Tmcc3	0	1.866074	0.822	0.642	0	MG-Homo	Tmcc3
Dst	0	1.804522	0.744	0.565	0	MG-Homo	Dst
Rptor	0	1.963731	0.523	0.344	0	MG-Homo	Rptor
Fgf13	0	2.466868	0.393	0.214	0	MG-Homo	Fgf13
Ttc28	0	2.070294	0.49	0.311	0	MG-Homo	Ttc28
Bbs9	0	2.023896	0.416	0.237	0	MG-Homo	Bbs9
Ankrd44	0	1.850396	0.683	0.506	0	MG-Homo	Ankrd44
Kcnma1	0	2.524561	0.34	0.164	0	MG-Homo	Kcnma1
Srgap2	0	1.928911	0.915	0.742	0	MG-Homo	Srgap2
Tmem135	0	1.899463	0.524	0.351	0	MG-Homo	Tmem135
Hdac9	0	1.814017	0.484	0.312	0	MG-Homo	Hdac9
Slco2b1	0	1.377635	0.777	0.607	0	MG-Homo	Slco2b1
Cables1	0	2.214274	0.382	0.213	0	MG-Homo	Cables1
Mgat4a	0	1.945359	0.519	0.351	0	MG-Homo	Mgat4a
Tgfbr1	0	1.567039	0.926	0.758	0	MG-Homo	Tgfbr1
Elmo1	0	2.198214	0.9	0.733	0	MG-Homo	Elmo1
Ppp1r9a	0	1.811917	0.518	0.352	0	MG-Homo	Ppp1r9a
Plcl2	0	1.877878	0.638	0.472	0	MG-Homo	Plcl2
Slc8a1	0	2.112336	0.846	0.68	0	MG-Homo	Slc8a1
8030442B0	0	2.115338	0.336	0.172	0	MG-Homo	8030442B05Rik
Rreb1	0	1.843166	0.695	0.531	0	MG-Homo	Rreb1
Dip2b	0	1.622296	0.724	0.561	0	MG-Homo	Dip2b
Ifitm10	0	1.592814	0.448	0.287	0	MG-Homo	Ifitm10
Mgat5	0	1.89599	0.503	0.343	0	MG-Homo	Mgat5
Itga9	0	1.945424	0.424	0.264	0	MG-Homo	Itga9
Pard3b	0	2.428818	0.28	0.121	0	MG-Homo	Pard3b
Fbxl17	0	1.998788	0.517	0.358	0	MG-Homo	Fbxl17
Zeb1	0	1.846142	0.459	0.303	0	MG-Homo	Zeb1
Ubash3b	0	1.980057	0.607	0.451	0	MG-Homo	Ubash3b
Zswim6	0	1.870471	0.62	0.464	0	MG-Homo	Zswim6
Zfp710	0	1.897433	0.604	0.45	0	MG-Homo	Zfp710
Smyd3	0	2.139389	0.421	0.268	0	MG-Homo	Smyd3
Zdhhc14	0	2.000245	0.426	0.273	0	MG-Homo	Zdhhc14
Slc9a9	0	1.653979	0.766	0.616	0	MG-Homo	Slc9a9
Lrch1	0	1.705929	0.675	0.526	0	MG-Homo	Lrch1
Hivep3	0	1.777031	0.554	0.406	0	MG-Homo	Hivep3
Sipa1l2	0	1.880238	0.508	0.36	0	MG-Homo	Sipa1l2
0610040J0	0	1.630554	0.55	0.403	0	MG-Homo	0610040J01Rik
Epb41l2	0	1.706576	0.859	0.713	0	MG-Homo	Epb41l2
Arl15	0	1.934155	0.438	0.292	0	MG-Homo	Arl15
Nos1ap	0	2.385578	0.259	0.115	0	MG-Homo	Nos1ap
Pvt1	0	2.231689	0.372	0.229	0	MG-Homo	Pvt1
Bach2	0	1.872937	0.457	0.316	0	MG-Homo	Bach2

E230029C(	0	1.572242	0.655	0.514	0	MG-Homo	E230029C05Rik
Fchsd2	0	1.541754	0.635	0.495	0	MG-Homo	Fchsd2
Wwox	0	2.06544	0.45	0.31	0	MG-Homo	Wwox
Specc1	0	1.746314	0.519	0.379	0	MG-Homo	Specc1
Arid1b	0	1.905645	0.486	0.346	0	MG-Homo	Arid1b
Ssh2	0	1.501241	0.838	0.701	0	MG-Homo	Ssh2
Runx1	0	1.872633	0.707	0.57	0	MG-Homo	Runx1
Nuak1	0	1.901021	0.412	0.275	0	MG-Homo	Nuak1
Zbtb20	0	1.717293	0.629	0.493	0	MG-Homo	Zbtb20
Btbd9	0	1.873358	0.518	0.382	0	MG-Homo	Btbd9
Pan3	0	1.751779	0.523	0.388	0	MG-Homo	Pan3
Tbc1d5	0	1.752498	0.611	0.476	0	MG-Homo	Tbc1d5
Soga1	0	1.57971	0.496	0.362	0	MG-Homo	Soga1
Ppm1h	0	1.847319	0.615	0.481	0	MG-Homo	Ppm1h
Tbc1d16	0	1.770574	0.455	0.321	0	MG-Homo	Tbc1d16
Mctp1	0	1.413825	0.454	0.32	0	MG-Homo	Mctp1
Pitpnc1	0	1.873142	0.53	0.397	0	MG-Homo	Pitpnc1
Tbc1d22a	0	1.933196	0.446	0.313	0	MG-Homo	Tbc1d22a
Trio	0	1.903631	0.407	0.274	0	MG-Homo	Trio
Lrmda	0	2.219886	0.832	0.701	0	MG-Homo	Lrmda
Adap2	0	1.482403	0.682	0.552	0	MG-Homo	Adap2
Ski	0	2.017996	0.496	0.367	0	MG-Homo	Ski
Pld1	0	1.739323	0.428	0.3	0	MG-Homo	Pld1
Zcchc7	0	1.838196	0.433	0.305	0	MG-Homo	Zcchc7
Dleu2	0	1.5698	0.729	0.601	0	MG-Homo	Dleu2
Gnaq	0	1.707807	0.628	0.5	0	MG-Homo	Gnaq
Ikzf1	0	1.595165	0.733	0.607	0	MG-Homo	Ikzf1
Rasgrp3	0	1.579673	0.521	0.395	0	MG-Homo	Rasgrp3
Chd7	0	1.708505	0.529	0.405	0	MG-Homo	Chd7
Exoc4	0	1.691914	0.548	0.426	0	MG-Homo	Exoc4
Itpr2	0	1.660858	0.513	0.391	0	MG-Homo	Itpr2
Arhgap39	0	1.672794	0.493	0.371	0	MG-Homo	Arhgap39
Inpp5d	0	1.61021	0.897	0.777	0	MG-Homo	Inpp5d
Frmd4b	0	1.404778	0.713	0.593	0	MG-Homo	Frmd4b
Mef2a	0	1.627876	0.838	0.718	0	MG-Homo	Mef2a
Lhfpl2	0	1.622831	0.481	0.361	0	MG-Homo	Lhfpl2
Fli1	0	1.686589	0.672	0.554	0	MG-Homo	Fli1
Foxn3	0	1.514474	0.823	0.705	0	MG-Homo	Foxn3
Diaph2	0	1.770671	0.651	0.533	0	MG-Homo	Diaph2
Slc12a6	0	1.581587	0.494	0.377	0	MG-Homo	Slc12a6
Ccnd3	0	1.665885	0.622	0.505	0	MG-Homo	Ccnd3
Dock8	0	1.555411	0.733	0.617	0	MG-Homo	Dock8
Cadm1	0	1.933961	0.494	0.379	0	MG-Homo	Cadm1
Gab2	0	1.527481	0.786	0.673	0	MG-Homo	Gab2

Rere	0	1.640826	0.537	0.426	0	MG-Homo	Rere
Mef2c	0	1.414147	0.819	0.708	0	MG-Homo	Mef2c
Asap1	0	1.581582	0.644	0.533	0	MG-Homo	Asap1
Macf1	0	1.521811	0.766	0.657	0	MG-Homo	Macf1
Phf14	0	1.578181	0.491	0.383	0	MG-Homo	Phf14
Dennd4a	0	1.409189	0.704	0.596	0	MG-Homo	Dennd4a
Tns3	0	1.653241	0.62	0.513	0	MG-Homo	Tns3
Dock2	0	1.915226	0.781	0.675	0	MG-Homo	Dock2
Rap1gds1	0	1.477401	0.683	0.578	0	MG-Homo	Rap1gds1
Rnf180	0	1.53934	0.506	0.403	0	MG-Homo	Rnf180
Dock10	0	1.336017	0.695	0.593	0	MG-Homo	Dock10
Jmjd1c	0	1.535614	0.627	0.525	0	MG-Homo	Jmjd1c
Fam172a	0	1.635899	0.492	0.39	0	MG-Homo	Fam172a
Pip4k2a	0	1.563464	0.74	0.639	0	MG-Homo	Pip4k2a
Atp8a1	0	1.610014	0.59	0.49	0	MG-Homo	Atp8a1
Bmp2k	0	1.419902	0.714	0.614	0	MG-Homo	Bmp2k
Ivns1abp	0	1.30869	0.561	0.463	0	MG-Homo	Ivns1abp
Qk	0	1.449669	0.888	0.791	0	MG-Homo	Qk
Nrip1	0	1.317705	0.592	0.496	0	MG-Homo	Nrip1
Mitf	0	1.631634	0.529	0.436	0	MG-Homo	Mitf
Camk2d	0	1.384304	0.612	0.52	0	MG-Homo	Camk2d
St3gal6	0	1.129921	0.623	0.531	0	MG-Homo	St3gal6
Map4k4	0	1.360213	0.569	0.478	0	MG-Homo	Map4k4
Arhgap5	0	1.203275	0.612	0.521	0	MG-Homo	Arhgap5
Clasp2	0	1.446813	0.519	0.429	0	MG-Homo	Clasp2
Celf2	0	1.45145	0.681	0.592	0	MG-Homo	Celf2
Wdfy3	0	1.433236	0.517	0.429	0	MG-Homo	Wdfy3
Zfand3	0	1.625883	0.505	0.421	0	MG-Homo	Zfand3
Ptprj	0	1.545279	0.711	0.629	0	MG-Homo	Ptprj
Pik3r1	0	1.392204	0.563	0.481	0	MG-Homo	Pik3r1
Zeb2	0	1.279752	0.827	0.748	0	MG-Homo	Zeb2
Etv6	0	1.435267	0.652	0.574	0	MG-Homo	Etv6
Ppp3ca	0	1.422811	0.615	0.537	0	MG-Homo	Ppp3ca
Mbnl1	0	1.494671	0.768	0.691	0	MG-Homo	Mbnl1
Kansl1	0	1.381264	0.563	0.486	0	MG-Homo	Kansl1
Hpgds	0	1.082416	0.68	0.608	0	MG-Homo	Hpgds
Chd9	0	1.220869	0.792	0.723	0	MG-Homo	Chd9
Prkcb	0	1.53445	0.574	0.508	0	MG-Homo	Prkcb
Picalm	0	1.406925	0.679	0.618	0	MG-Homo	Picalm
Mycbp2	0	1.269982	0.591	0.53	0	MG-Homo	Mycbp2
Entpd1	0	1.140369	0.712	0.656	0	MG-Homo	Entpd1
Apbb1ip	0	1.129837	0.788	0.741	0	MG-Homo	Apbb1ip
Wnk1	0	1.241621	0.647	0.601	0	MG-Homo	Wnk1
Lair1	0	0.786165	0.776	0.73	0	MG-Homo	Lair1

Tbxas1	0	1.170546	0.629	0.589	0	MG-Homo	Tbxas1
Tgfbr2	0	0.986252	0.673	0.643	0	MG-Homo	Tgfbr2
Sirpa	0	0.67377	0.854	0.828	0	MG-Homo	Sirpa
Lyn	0	1.064406	0.834	0.809	0	MG-Homo	Lyn
Malat1	0	1.139688	0.998	0.974	0	MG-Homo	Malat1
Fam49b	0	1.144641	0.755	0.744	0	MG-Homo	Fam49b
Rrbp1	0	0.703198	0.773	0.775	0	MG-Homo	Rrbp1
Sh3kbp1	2.14E-304	1.505979	0.515	0.433	5.23E-300	MG-Homo	Sh3kbp1
1-Mar	5.92E-301	1.469139	0.51	0.42	1.45E-296	MG-Homo	1-Mar
Smad3	4.51E-298	2.10611	0.324	0.186	1.10E-293	MG-Homo	Smad3
Med13l	5.27E-298	1.850977	0.394	0.264	1.29E-293	MG-Homo	Med13l
Sdk1	2.33E-297	2.326931	0.295	0.157	5.71E-293	MG-Homo	Sdk1
Prkce	4.07E-292	2.181632	0.304	0.166	9.96E-288	MG-Homo	Prkce
Smap2	1.68E-290	0.985304	0.66	0.644	4.12E-286	MG-Homo	Smap2
Apba1	4.24E-290	1.806616	0.409	0.282	1.04E-285	MG-Homo	Apba1
Tcf12	5.87E-290	1.795578	0.403	0.277	1.44E-285	MG-Homo	Tcf12
Pwwp2a	1.50E-287	1.758989	0.379	0.248	3.66E-283	MG-Homo	Pwwp2a
Sipa1l1	6.97E-286	2.013826	0.321	0.184	1.71E-281	MG-Homo	Sipa1l1
Nek7	7.17E-286	1.493603	0.499	0.416	1.76E-281	MG-Homo	Nek7
Cacna1d	2.01E-282	1.955571	0.377	0.25	4.93E-278	MG-Homo	Cacna1d
Cttnbp2nl	2.94E-278	1.265415	0.55	0.485	7.19E-274	MG-Homo	Cttnbp2nl
Prex1	2.18E-275	1.358883	0.546	0.497	5.33E-271	MG-Homo	Prex1
Tcf4	1.26E-272	1.370734	0.527	0.462	3.09E-268	MG-Homo	Tcf4
Peli2	1.30E-271	2.106444	0.318	0.188	3.18E-267	MG-Homo	Peli2
Mvb12b	3.17E-271	1.789766	0.389	0.268	7.77E-267	MG-Homo	Mvb12b
Cux1	2.45E-268	1.641398	0.473	0.391	6.01E-264	MG-Homo	Cux1
Stag1	6.18E-267	1.631365	0.437	0.332	1.51E-262	MG-Homo	Stag1
Itgb5	1.49E-263	0.562133	0.849	0.784	3.66E-259	MG-Homo	Itgb5
Nf1	1.20E-262	1.73728	0.413	0.305	2.93E-258	MG-Homo	Nf1
Camk1d	4.30E-258	0.681329	0.777	0.815	1.05E-253	MG-Homo	Camk1d
Cdkal1	6.42E-258	1.949473	0.321	0.194	1.57E-253	MG-Homo	Cdkal1
Nbea	8.88E-258	2.166677	0.251	0.126	2.18E-253	MG-Homo	Nbea
Vav2	1.63E-256	1.957715	0.36	0.242	3.98E-252	MG-Homo	Vav2
Gtdc1	1.87E-256	1.803498	0.374	0.253	4.58E-252	MG-Homo	Gtdc1
P2ry12	3.36E-256	0.532993	0.861	0.746	8.22E-252	MG-Homo	P2ry12
Lncpint	3.37E-256	1.713094	0.43	0.329	8.26E-252	MG-Homo	Lncpint
Ankrd11	1.13E-255	1.305343	0.537	0.493	2.77E-251	MG-Homo	Ankrd11
Whrn	1.82E-254	1.817539	0.329	0.2	4.45E-250	MG-Homo	Whrn
Sh3pxd2a	6.14E-253	1.770869	0.364	0.242	1.50E-248	MG-Homo	Sh3pxd2a
Col27a1	9.77E-252	1.760027	0.346	0.219	2.39E-247	MG-Homo	Col27a1
Mbnl2	4.37E-248	1.354013	0.5	0.431	1.07E-243	MG-Homo	Mbnl2
Msi2	3.27E-244	1.84696	0.336	0.213	8.00E-240	MG-Homo	Msi2
Fgd2	1.53E-243	1.005751	0.604	0.574	3.74E-239	MG-Homo	Fgd2
Ints6l	2.62E-242	1.474688	0.437	0.334	6.42E-238	MG-Homo	Ints6l

E2f3	8.14E-242	2.067063	0.289	0.167	1.99E-237	MG-Homo	E2f3
Atxn7l1	1.13E-240	1.838581	0.37	0.258	2.76E-236	MG-Homo	Atxn7l1
Cmip	1.72E-240	1.606968	0.453	0.371	4.21E-236	MG-Homo	Cmip
Retreg1	3.81E-240	1.394365	0.505	0.445	9.34E-236	MG-Homo	Retreg1
Foxp1	8.51E-240	1.292102	0.517	0.454	2.08E-235	MG-Homo	Foxp1
Fkbp5	1.39E-236	1.654845	0.405	0.294	3.41E-232	MG-Homo	Fkbp5
Fto	5.03E-236	1.88812	0.35	0.236	1.23E-231	MG-Homo	Fto
Vti1a	1.33E-234	1.660155	0.43	0.339	3.27E-230	MG-Homo	Vti1a
Luc7l2	3.32E-233	1.032779	0.588	0.576	8.14E-229	MG-Homo	Luc7l2
Phyhd1	6.92E-233	0.994565	0.593	0.541	1.69E-228	MG-Homo	Phyhd1
Uvrag	5.43E-231	1.320453	0.497	0.437	1.33E-226	MG-Homo	Uvrag
Fyb	2.12E-229	0.740534	0.718	0.73	5.20E-225	MG-Homo	Fyb
Atxn1	1.76E-227	1.85387	0.323	0.204	4.30E-223	MG-Homo	Atxn1
Abca9	6.23E-227	1.123851	0.529	0.462	1.53E-222	MG-Homo	Abca9
Siglech	8.27E-227	0.59852	0.755	0.645	2.02E-222	MG-Homo	Siglech
Tnrc6b	9.45E-225	1.481887	0.44	0.352	2.31E-220	MG-Homo	Tnrc6b
Bin1	1.85E-223	0.752727	0.695	0.682	4.54E-219	MG-Homo	Bin1
Extl3	7.90E-222	1.561242	0.424	0.334	1.93E-217	MG-Homo	Extl3
Dnmt3a	9.56E-222	1.599597	0.431	0.346	2.34E-217	MG-Homo	Dnmt3a
Parp8	1.06E-221	1.659171	0.387	0.284	2.60E-217	MG-Homo	Parp8
Snx24	1.15E-221	1.54019	0.467	0.394	2.81E-217	MG-Homo	Snx24
Rnf169	2.36E-221	1.536382	0.422	0.327	5.79E-217	MG-Homo	Rnf169
Arhgap17	2.25E-220	1.322657	0.505	0.457	5.50E-216	MG-Homo	Arhgap17
Dip2c	8.30E-218	2.128559	0.266	0.151	2.03E-213	MG-Homo	Dip2c
Anks1	4.46E-217	1.763963	0.344	0.234	1.09E-212	MG-Homo	Anks1
Fam102b	2.50E-216	1.49602	0.415	0.313	6.11E-212	MG-Homo	Fam102b
Igf1r	2.51E-211	1.782976	0.32	0.206	6.16E-207	MG-Homo	Igf1r
Phf21a	1.25E-210	1.543236	0.43	0.348	3.07E-206	MG-Homo	Phf21a
Skap2	1.87E-208	1.141391	0.564	0.567	4.57E-204	MG-Homo	Skap2
Dyrk1a	1.89E-207	1.851637	0.304	0.193	4.63E-203	MG-Homo	Dyrk1a
Apbb2	4.54E-207	2.01539	0.314	0.204	1.11E-202	MG-Homo	Apbb2
Herc2	2.70E-204	1.261175	0.48	0.416	6.61E-200	MG-Homo	Herc2
Ncoa2	1.30E-201	1.702028	0.369	0.273	3.18E-197	MG-Homo	Ncoa2
Dock1	2.56E-200	1.825281	0.293	0.183	6.26E-196	MG-Homo	Dock1
Rnf216	1.37E-199	1.454492	0.429	0.351	3.36E-195	MG-Homo	Rnf216
Usp24	2.14E-199	1.606813	0.356	0.253	5.24E-195	MG-Homo	Usp24
Abca1	2.98E-199	1.246198	0.548	0.512	7.30E-195	MG-Homo	Abca1
Apc	8.80E-199	1.471066	0.414	0.329	2.15E-194	MG-Homo	Apc
Oxr1	1.52E-198	1.697667	0.375	0.28	3.71E-194	MG-Homo	Oxr1
Sbf2	9.45E-198	1.347993	0.425	0.337	2.32E-193	MG-Homo	Sbf2
Itga6	2.12E-197	1.211873	0.482	0.41	5.19E-193	MG-Homo	Itga6
Abhd12	3.51E-193	0.627119	0.748	0.756	8.60E-189	MG-Homo	Abhd12
Spire1	7.23E-193	1.648892	0.347	0.248	1.77E-188	MG-Homo	Spire1
Fnbp1	3.20E-191	1.382131	0.451	0.389	7.84E-187	MG-Homo	Fnbp1

Rtn4rl1	8.54E-191	1.36069	0.419	0.332	2.09E-186	MG-Homo	Rtn4rl1
Ube2h	8.59E-191	1.546196	0.386	0.295	2.10E-186	MG-Homo	Ube2h
Bcas3	3.10E-190	1.933653	0.314	0.211	7.60E-186	MG-Homo	Bcas3
Slc7a8	2.65E-187	1.191805	0.503	0.455	6.48E-183	MG-Homo	Slc7a8
A830008E2	7.44E-187	1.563128	0.364	0.263	1.82E-182	MG-Homo	A830008E24Rik
Tbc1d9	1.02E-186	1.408893	0.437	0.367	2.49E-182	MG-Homo	Tbc1d9
Rad51b	3.39E-186	1.31072	0.501	0.441	8.30E-182	MG-Homo	Rad51b
Mbd5	2.09E-185	1.689001	0.337	0.239	5.12E-181	MG-Homo	Mbd5
Pid1	4.71E-185	1.095287	0.479	0.404	1.15E-180	MG-Homo	Pid1
Asph	7.28E-185	1.230369	0.477	0.417	1.78E-180	MG-Homo	Asph
2610203C2	3.74E-184	1.750025	0.3	0.196	9.16E-180	MG-Homo	2610203C22Rik
Dgkd	1.86E-183	1.645538	0.373	0.286	4.56E-179	MG-Homo	Dgkd
Fars2	2.59E-183	1.884243	0.317	0.219	6.35E-179	MG-Homo	Fars2
Tet3	3.04E-183	1.703543	0.349	0.256	7.45E-179	MG-Homo	Tet3
Dennd1a	5.83E-183	1.282718	0.421	0.341	1.43E-178	MG-Homo	Dennd1a
Cerk	3.90E-182	1.412786	0.432	0.366	9.56E-178	MG-Homo	Cerk
Tcf7l2	5.10E-182	1.830988	0.311	0.211	1.25E-177	MG-Homo	Tcf7l2
Git2	4.35E-180	1.118963	0.513	0.481	1.07E-175	MG-Homo	Git2
Rcsd1	1.35E-179	1.436469	0.432	0.366	3.31E-175	MG-Homo	Rcsd1
Vps13b	6.27E-179	1.573524	0.366	0.278	1.54E-174	MG-Homo	Vps13b
Plcg2	1.52E-177	1.237914	0.483	0.444	3.72E-173	MG-Homo	Plcg2
St3gal3	5.55E-177	1.846872	0.29	0.19	1.36E-172	MG-Homo	St3gal3
Hdac8	8.32E-176	1.918871	0.271	0.17	2.04E-171	MG-Homo	Hdac8
Zfp652	2.91E-175	1.266957	0.453	0.391	7.13E-171	MG-Homo	Zfp652
Exoc6b	5.88E-175	1.717406	0.309	0.21	1.44E-170	MG-Homo	Exoc6b
Snx13	1.53E-173	1.563856	0.372	0.289	3.76E-169	MG-Homo	Snx13
Gm26542	1.84E-173	1.693897	0.309	0.209	4.51E-169	MG-Homo	Gm26542
Son	2.13E-173	0.812063	0.631	0.656	5.22E-169	MG-Homo	Son
Disc1	4.34E-173	1.929484	0.257	0.157	1.06E-168	MG-Homo	Disc1
Wdfy2	4.50E-172	1.470083	0.375	0.29	1.10E-167	MG-Homo	Wdfy2
Man1a	5.85E-172	1.44202	0.431	0.371	1.43E-167	MG-Homo	Man1a
Ankrd17	1.10E-170	1.548401	0.384	0.309	2.70E-166	MG-Homo	Ankrd17
Insr	4.81E-170	1.758828	0.274	0.174	1.18E-165	MG-Homo	Insr
Tbl1xr1	3.20E-169	1.579605	0.378	0.3	7.84E-165	MG-Homo	Tbl1xr1
Acap2	1.85E-168	1.101742	0.51	0.488	4.53E-164	MG-Homo	Acap2
AW554918	4.86E-166	1.881915	0.256	0.158	1.19E-161	MG-Homo	AW554918
Cdk6	1.01E-164	1.812436	0.291	0.195	2.47E-160	MG-Homo	Cdk6
Kmt2c	1.25E-163	1.465986	0.378	0.299	3.06E-159	MG-Homo	Kmt2c
Rock2	3.18E-163	1.287579	0.435	0.377	7.79E-159	MG-Homo	Rock2
Wasf2	3.13E-162	0.874034	0.6	0.622	7.66E-158	MG-Homo	Wasf2
Nsd1	3.79E-160	1.300906	0.435	0.381	9.27E-156	MG-Homo	Nsd1
Csf3r	4.56E-160	0.897137	0.533	0.506	1.12E-155	MG-Homo	Csf3r
Lpcat2	5.87E-160	0.41912	0.824	0.796	1.44E-155	MG-Homo	Lpcat2
Kcnk13	1.25E-158	1.540581	0.382	0.31	3.05E-154	MG-Homo	Kcnk13

Stard9	5.70E-158	1.379706	0.398	0.328	1.40E-153	MG-Homo	Stard9
Mkln1	6.61E-158	1.391444	0.403	0.338	1.62E-153	MG-Homo	Mkln1
Chd2	1.14E-157	1.595575	0.34	0.256	2.79E-153	MG-Homo	Chd2
Fmnl3	4.08E-157	1.183325	0.462	0.412	1.00E-152	MG-Homo	Fmnl3
Arhgap25	7.59E-157	1.560573	0.373	0.301	1.86E-152	MG-Homo	Arhgap25
Zmiz1	1.31E-156	1.174076	0.507	0.497	3.20E-152	MG-Homo	Zmiz1
Fam193a	1.23E-155	1.628492	0.326	0.24	3.01E-151	MG-Homo	Fam193a
Lrrk1	8.43E-155	1.315563	0.431	0.379	2.06E-150	MG-Homo	Lrrk1
Rapgef1	4.04E-154	1.482559	0.382	0.314	9.88E-150	MG-Homo	Rapgef1
Crebbp	5.86E-154	1.474097	0.387	0.32	1.43E-149	MG-Homo	Crebbp
Pik3ap1	3.23E-153	1.08807	0.526	0.527	7.90E-149	MG-Homo	Pik3ap1
Vrk2	6.32E-153	1.563368	0.358	0.282	1.55E-148	MG-Homo	Vrk2
Thada	1.24E-152	1.754996	0.283	0.193	3.04E-148	MG-Homo	Thada
Blnk	3.57E-152	1.037655	0.513	0.488	8.74E-148	MG-Homo	Blnk
Il6ra	8.06E-151	1.068449	0.506	0.481	1.97E-146	MG-Homo	Il6ra
Immp2l	6.59E-150	1.928082	0.305	0.219	1.61E-145	MG-Homo	Immp2l
Cdk19	1.52E-149	1.605217	0.323	0.239	3.72E-145	MG-Homo	Cdk19
Gtf2h2	1.07E-148	1.137465	0.455	0.403	2.61E-144	MG-Homo	Gtf2h2
Mlxip	1.48E-147	1.66212	0.309	0.224	3.62E-143	MG-Homo	Mlxip
Ulk2	4.28E-146	1.24468	0.423	0.362	1.05E-141	MG-Homo	Ulk2
Nipbl	8.50E-146	1.196303	0.47	0.446	2.08E-141	MG-Homo	Nipbl
Arhgap45	2.18E-145	0.808773	0.607	0.62	5.34E-141	MG-Homo	Arhgap45
Cnot2	5.40E-145	1.461402	0.379	0.314	1.32E-140	MG-Homo	Cnot2
Pnpla7	1.63E-144	1.318107	0.424	0.375	3.98E-140	MG-Homo	Pnpla7
Man1c1	1.80E-144	1.352812	0.462	0.433	4.41E-140	MG-Homo	Man1c1
Smg6	4.88E-144	1.635644	0.323	0.243	1.19E-139	MG-Homo	Smg6
Stxbp5	5.24E-144	1.663737	0.282	0.193	1.28E-139	MG-Homo	Stxbp5
Map2k5	1.98E-143	1.768376	0.28	0.194	4.84E-139	MG-Homo	Map2k5
Cfh	4.02E-142	0.700131	0.628	0.611	9.85E-138	MG-Homo	Cfh
Eif4g3	6.91E-142	1.291141	0.446	0.414	1.69E-137	MG-Homo	Eif4g3
Fndc3b	2.11E-140	1.672202	0.323	0.245	5.18E-136	MG-Homo	Fndc3b
Map3k3	7.06E-138	1.45447	0.355	0.283	1.73E-133	MG-Homo	Map3k3
Cog5	3.30E-137	1.668177	0.28	0.195	8.08E-133	MG-Homo	Cog5
Lims1	5.13E-137	1.199165	0.478	0.465	1.26E-132	MG-Homo	Lims1
Lcorl	6.93E-137	1.653794	0.294	0.211	1.70E-132	MG-Homo	Lcorl
Sik2	7.78E-137	1.583876	0.297	0.212	1.90E-132	MG-Homo	Sik2
Wdfy4	1.96E-136	1.33606	0.354	0.28	4.80E-132	MG-Homo	Wdfy4
Mllt10	2.22E-136	1.536222	0.342	0.269	5.43E-132	MG-Homo	Mllt10
B4galt1	3.20E-136	1.221921	0.447	0.412	7.85E-132	MG-Homo	B4galt1
Zfp407	3.38E-136	1.799318	0.267	0.182	8.28E-132	MG-Homo	Zfp407
Arsb	4.75E-135	1.099023	0.477	0.436	1.16E-130	MG-Homo	Arsb
Tacc1	1.33E-133	1.065808	0.501	0.494	3.26E-129	MG-Homo	Tacc1
Mtmr3	9.50E-133	1.517356	0.319	0.242	2.33E-128	MG-Homo	Mtmr3
Myo1f	1.01E-131	0.781257	0.601	0.635	2.48E-127	MG-Homo	Myo1f

Rhobtb1	2.25E-131	1.466696	0.312	0.23	5.50E-127	MG-Homo	Rhobtb1
Zfp292	5.24E-131	1.382567	0.371	0.308	1.28E-126	MG-Homo	Zfp292
Ambra1	5.76E-131	1.576536	0.33	0.259	1.41E-126	MG-Homo	Ambra1
Rtn1	1.24E-130	1.625653	0.312	0.232	3.03E-126	MG-Homo	Rtn1
Ash1l	4.65E-130	1.410002	0.375	0.315	1.14E-125	MG-Homo	Ash1l
Osbpl11	6.48E-130	1.355888	0.395	0.34	1.59E-125	MG-Homo	Osbpl11
Vsir	2.54E-129	0.521758	0.718	0.732	6.23E-125	MG-Homo	Vsir
Arhgap12	5.17E-129	1.462798	0.33	0.255	1.27E-124	MG-Homo	Arhgap12
Slmap	3.38E-128	1.472984	0.333	0.26	8.27E-124	MG-Homo	Slmap
Irak2	5.08E-128	1.199872	0.447	0.419	1.24E-123	MG-Homo	Irak2
Atp9b	1.51E-127	1.49457	0.337	0.269	3.70E-123	MG-Homo	Atp9b
Ssbp2	3.20E-127	1.563932	0.316	0.241	7.83E-123	MG-Homo	Ssbp2
Cradd	6.19E-127	1.880334	0.262	0.182	1.51E-122	MG-Homo	Cradd
Cdk14	1.65E-126	1.509362	0.307	0.229	4.03E-122	MG-Homo	Cdk14
Garnl3	3.14E-125	1.568097	0.266	0.183	7.69E-121	MG-Homo	Garnl3
Strn3	1.87E-124	1.439799	0.348	0.282	4.58E-120	MG-Homo	Strn3
Arhgap24	4.77E-124	2.035928	0.268	0.19	1.17E-119	MG-Homo	Arhgap24
Map3k5	9.59E-124	1.412745	0.326	0.252	2.35E-119	MG-Homo	Map3k5
Mylip	1.85E-123	0.941147	0.513	0.492	4.53E-119	MG-Homo	Mylip
Pou2f2	2.71E-123	0.92389	0.531	0.528	6.64E-119	MG-Homo	Pou2f2
Map4k3	6.61E-122	1.56111	0.282	0.203	1.62E-117	MG-Homo	Map4k3
Gna12	1.16E-121	0.974459	0.522	0.526	2.85E-117	MG-Homo	Gna12
Golm1	1.66E-121	0.746021	0.546	0.509	4.06E-117	MG-Homo	Golm1
Agps	3.40E-121	1.300633	0.381	0.328	8.33E-117	MG-Homo	Agps
Akap13	1.31E-120	1.012088	0.497	0.499	3.20E-116	MG-Homo	Akap13
Rbm39	3.50E-120	0.559891	0.697	0.761	8.57E-116	MG-Homo	Rbm39
Fbxl20	3.12E-118	1.522402	0.308	0.236	7.64E-114	MG-Homo	Fbxl20
Lpin2	1.08E-116	1.117846	0.454	0.432	2.64E-112	MG-Homo	Lpin2
Rffl	1.35E-116	1.692123	0.284	0.211	3.30E-112	MG-Homo	Rffl
Capn3	1.91E-116	1.203295	0.397	0.343	4.67E-112	MG-Homo	Capn3
Phkb	2.00E-116	1.592799	0.28	0.204	4.90E-112	MG-Homo	Phkb
Ext1	4.98E-116	1.564081	0.289	0.213	1.22E-111	MG-Homo	Ext1
Bank1	6.13E-116	1.517274	0.266	0.185	1.50E-111	MG-Homo	Bank1
Snd1	1.88E-114	1.414096	0.383	0.342	4.62E-110	MG-Homo	Snd1
Baz2b	1.97E-114	1.221533	0.416	0.381	4.83E-110	MG-Homo	Baz2b
Man1a2	2.92E-114	1.180973	0.416	0.376	7.15E-110	MG-Homo	Man1a2
Abl1	1.45E-113	1.471672	0.314	0.246	3.56E-109	MG-Homo	Abl1
Lrba	2.24E-113	1.420564	0.268	0.188	5.48E-109	MG-Homo	Lrba
Foxo3	4.50E-113	1.534841	0.301	0.23	1.10E-108	MG-Homo	Foxo3
Nfkb1	8.25E-113	1.098056	0.474	0.472	2.02E-108	MG-Homo	Nfkb1
Setd5	1.53E-112	1.332492	0.369	0.319	3.75E-108	MG-Homo	Setd5
Smarca2	3.08E-112	1.383839	0.346	0.286	7.55E-108	MG-Homo	Smarca2
Dis3l2	7.29E-111	1.723953	0.255	0.181	1.79E-106	MG-Homo	Dis3l2
Tmem164	1.41E-110	1.475691	0.337	0.277	3.46E-106	MG-Homo	Tmem164

Atxn7	3.21E-110	1.57407	0.285	0.215	7.85E-106	MG-Homo	Atxn7
Smg1	5.87E-109	1.355731	0.357	0.304	1.44E-104	MG-Homo	Smg1
Rbm6	1.83E-108	1.478897	0.32	0.257	4.47E-104	MG-Homo	Rbm6
Celf1	2.82E-108	1.369391	0.356	0.305	6.92E-104	MG-Homo	Celf1
Sdccag8	1.11E-107	1.557751	0.313	0.249	2.73E-103	MG-Homo	Sdccag8
Tnks	1.24E-106	1.648201	0.253	0.18	3.04E-102	MG-Homo	Tnks
Cyfip1	1.75E-106	0.57065	0.662	0.706	4.29E-102	MG-Homo	Cyfip1
Usp34	8.64E-106	1.436341	0.323	0.262	2.12E-101	MG-Homo	Usp34
Tbl1x	1.76E-105	1.567311	0.318	0.258	4.30E-101	MG-Homo	Tbl1x
Plekhm3	1.93E-105	1.59378	0.28	0.212	4.72E-101	MG-Homo	Plekhm3
Mapk14	4.33E-105	1.182701	0.419	0.393	1.06E-100	MG-Homo	Mapk14
Ddx17	9.04E-105	1.250111	0.376	0.333	2.21E-100	MG-Homo	Ddx17
Cask	2.60E-104	1.233175	0.36	0.301	6.38E-100	MG-Homo	Cask
Slc1a3	3.02E-104	1.025549	0.443	0.407	7.40E-100	MG-Homo	Slc1a3
Lcor	8.01E-104	1.478762	0.294	0.227	1.96E-99	MG-Homo	Lcor
Nlrp1b	1.18E-103	1.554848	0.308	0.247	2.88E-99	MG-Homo	Nlrp1b
Sfmbt1	2.55E-103	1.563984	0.282	0.215	6.25E-99	MG-Homo	Sfmbt1
Tcf20	5.31E-103	1.635584	0.271	0.202	1.30E-98	MG-Homo	Tcf20
Rbm47	1.73E-102	1.207598	0.419	0.396	4.24E-98	MG-Homo	Rbm47
Atp2c1	2.49E-101	1.154219	0.42	0.392	6.10E-97	MG-Homo	Atp2c1
Arih1	5.36E-101	1.355969	0.339	0.286	1.31E-96	MG-Homo	Arih1
Chd6	8.20E-101	1.515282	0.261	0.19	2.01E-96	MG-Homo	Chd6
Nedd4l	2.79E-100	1.670799	0.265	0.198	6.84E-96	MG-Homo	Nedd4l
Eml4	1.83E-99	1.304891	0.371	0.329	4.47E-95	MG-Homo	Eml4
Cx3cr1	4.10E-99	0.359446	0.872	0.829	1.00E-94	MG-Homo	Cx3cr1
Tspan14	6.35E-99	1.122334	0.425	0.404	1.56E-94	MG-Homo	Tspan14
Cflar	2.95E-98	1.064769	0.445	0.436	7.24E-94	MG-Homo	Cflar
Cyth4	1.89E-97	0.641831	0.623	0.672	4.64E-93	MG-Homo	Cyth4
Sall1	1.33E-96	0.88248	0.463	0.431	3.25E-92	MG-Homo	Sall1
Smurf2	1.44E-96	1.447187	0.293	0.23	3.54E-92	MG-Homo	Smurf2
Fbxo11	2.93E-96	1.555139	0.289	0.226	7.17E-92	MG-Homo	Fbxo11
Ubn2	2.58E-95	1.333001	0.355	0.31	6.32E-91	MG-Homo	Ubn2
AU020206	2.69E-95	1.046358	0.485	0.495	6.58E-91	MG-Homo	AU020206
Trappc9	1.28E-94	1.559935	0.296	0.237	3.14E-90	MG-Homo	Trappc9
Ap3b1	2.58E-94	1.266495	0.387	0.36	6.32E-90	MG-Homo	Ap3b1
Dapp1	2.61E-94	1.183587	0.391	0.357	6.40E-90	MG-Homo	Dapp1
Pald1	3.10E-94	1.314173	0.339	0.288	7.58E-90	MG-Homo	Pald1
Cnot4	4.79E-94	1.378934	0.346	0.301	1.17E-89	MG-Homo	Cnot4
Sppl3	5.99E-94	1.23538	0.383	0.351	1.47E-89	MG-Homo	Sppl3
Tjp1	3.28E-93	1.242971	0.321	0.26	8.02E-89	MG-Homo	Tjp1
Rab31	1.31E-92	1.315153	0.37	0.334	3.22E-88	MG-Homo	Rab31
Xist	8.50E-92	1.278123	0.407	0.373	2.08E-87	MG-Homo	Xist
Kif21b	1.71E-91	1.297726	0.333	0.282	4.19E-87	MG-Homo	Kif21b
Mtus1	4.59E-91	1.086203	0.429	0.411	1.12E-86	MG-Homo	Mtus1

Kansl1l	6.22E-91	1.563018	0.259	0.195	1.52E-86	MG-Homo	Kansl1l
Mdm4	2.59E-90	1.466162	0.281	0.221	6.35E-86	MG-Homo	Mdm4
Ppp1r12a	4.00E-90	1.047265	0.431	0.42	9.79E-86	MG-Homo	Ppp1r12a
Phactr2	6.80E-90	1.589934	0.283	0.223	1.67E-85	MG-Homo	Phactr2
Pik3cd	8.97E-90	1.242887	0.404	0.387	2.20E-85	MG-Homo	Pik3cd
Tubgcp5	2.45E-89	1.252942	0.341	0.292	6.00E-85	MG-Homo	Tubgcp5
Wdr44	3.96E-89	1.465196	0.282	0.221	9.70E-85	MG-Homo	Wdr44
Lifr	9.10E-89	1.491921	0.26	0.195	2.23E-84	MG-Homo	Lifr
Slc16a6	2.14E-88	1.272884	0.348	0.3	5.24E-84	MG-Homo	Slc16a6
Rbm26	2.37E-88	1.24428	0.342	0.294	5.80E-84	MG-Homo	Rbm26
Rhoh	7.18E-88	1.052513	0.43	0.415	1.76E-83	MG-Homo	Rhoh
Lpp	7.78E-88	1.470782	0.327	0.281	1.91E-83	MG-Homo	Lpp
Rassf2	8.08E-88	1.259809	0.355	0.315	1.98E-83	MG-Homo	Rassf2
Cds1	8.41E-88	1.490426	0.255	0.191	2.06E-83	MG-Homo	Cds1
Heatr5a	1.01E-87	1.209216	0.372	0.336	2.47E-83	MG-Homo	Heatr5a
Rab8b	1.52E-87	1.164689	0.401	0.38	3.73E-83	MG-Homo	Rab8b
Babam2	3.87E-87	1.366235	0.364	0.333	9.48E-83	MG-Homo	Babam2
Srrm2	1.03E-85	0.67736	0.595	0.661	2.51E-81	MG-Homo	Srrm2
Scmh1	1.29E-85	1.508098	0.262	0.2	3.16E-81	MG-Homo	Scmh1
Ppp1r21	3.99E-85	1.268538	0.364	0.331	9.78E-81	MG-Homo	Ppp1r21
Tbc1d14	9.19E-85	1.391529	0.321	0.274	2.25E-80	MG-Homo	Tbc1d14
Gm5086	1.68E-84	1.370045	0.299	0.243	4.11E-80	MG-Homo	Gm5086
Aftph	3.78E-84	1.193325	0.388	0.363	9.26E-80	MG-Homo	Aftph
Tnrc6a	9.08E-84	1.514882	0.259	0.199	2.22E-79	MG-Homo	Tnrc6a
Dock11	1.09E-83	1.357988	0.338	0.298	2.67E-79	MG-Homo	Dock11
Stk3	1.41E-83	1.546197	0.254	0.193	3.46E-79	MG-Homo	Stk3
Rbm33	2.30E-83	1.499955	0.251	0.189	5.64E-79	MG-Homo	Rbm33
Fnip2	3.32E-82	1.826165	0.25	0.191	8.12E-78	MG-Homo	Fnip2
Gng2	5.44E-82	1.23998	0.395	0.379	1.33E-77	MG-Homo	Gng2
Map4	6.31E-82	1.053504	0.442	0.445	1.54E-77	MG-Homo	Map4
Myo9a	1.03E-81	1.317116	0.349	0.314	2.52E-77	MG-Homo	Myo9a
Clint1	2.32E-81	0.961289	0.468	0.488	5.69E-77	MG-Homo	Clint1
Lrp5	4.48E-81	1.542308	0.263	0.206	1.10E-76	MG-Homo	Lrp5
Itgam	6.29E-81	0.572107	0.614	0.645	1.54E-76	MG-Homo	Itgam
Zfp148	8.00E-80	1.340251	0.325	0.283	1.96E-75	MG-Homo	Zfp148
Tns1	9.76E-80	1.540523	0.261	0.203	2.39E-75	MG-Homo	Tns1
Itch	2.88E-79	1.199897	0.365	0.334	7.05E-75	MG-Homo	Itch
Fam168a	7.08E-79	1.418703	0.294	0.243	1.73E-74	MG-Homo	Fam168a
Arid1a	1.25E-78	1.128937	0.399	0.386	3.05E-74	MG-Homo	Arid1a
Zfp638	1.39E-78	1.340569	0.311	0.266	3.40E-74	MG-Homo	Zfp638
Rnf111	5.91E-78	1.481281	0.27	0.216	1.45E-73	MG-Homo	Rnf111
Wipf1	1.38E-77	1.265785	0.392	0.381	3.38E-73	MG-Homo	Wipf1
A630001G2	1.61E-77	1.347873	0.305	0.258	3.94E-73	MG-Homo	A630001G21Rik
Phc2	3.63E-77	1.274972	0.366	0.344	8.88E-73	MG-Homo	Phc2

Cop1	9.01E-77	1.442909	0.261	0.205	2.21E-72	MG-Homo	Cop1
Nfatc2	2.53E-76	1.520089	0.25	0.192	6.18E-72	MG-Homo	Nfatc2
Suclg2	5.62E-76	1.514051	0.274	0.224	1.38E-71	MG-Homo	Suclg2
Lrp1	2.33E-74	0.624313	0.569	0.603	5.71E-70	MG-Homo	Lrp1
Fbxw11	1.45E-73	1.482927	0.285	0.238	3.54E-69	MG-Homo	Fbxw11
Pik3r5	1.60E-73	1.408603	0.286	0.237	3.91E-69	MG-Homo	Pik3r5
Smad7	3.41E-73	1.28681	0.35	0.315	8.34E-69	MG-Homo	Smad7
Arglu1	4.28E-73	1.130814	0.379	0.359	1.05E-68	MG-Homo	Arglu1
Ppp6r3	8.59E-73	1.378212	0.281	0.231	2.10E-68	MG-Homo	Ppp6r3
Nckap1l	1.01E-71	0.929922	0.463	0.486	2.48E-67	MG-Homo	Nckap1l
Tln2	2.11E-71	1.454312	0.252	0.198	5.17E-67	MG-Homo	Tln2
Eri3	1.86E-70	1.5484	0.282	0.238	4.54E-66	MG-Homo	Eri3
Ubr3	4.96E-70	1.376825	0.263	0.212	1.21E-65	MG-Homo	Ubr3
Pacsin2	7.58E-70	1.25662	0.363	0.344	1.86E-65	MG-Homo	Pacsin2
Peli1	7.26E-69	1.113197	0.399	0.394	1.78E-64	MG-Homo	Peli1
Rsrc1	9.48E-69	1.386881	0.303	0.264	2.32E-64	MG-Homo	Rsrc1
Snrnp70	1.19E-68	0.862034	0.486	0.519	2.92E-64	MG-Homo	Snrnp70
Atg10	1.91E-67	1.57881	0.257	0.208	4.69E-63	MG-Homo	Atg10
Mon2	4.22E-67	1.316845	0.297	0.255	1.03E-62	MG-Homo	Mon2
Acss1	6.66E-67	1.41359	0.266	0.218	1.63E-62	MG-Homo	Acss1
Fmnl2	1.01E-66	1.414589	0.33	0.299	2.47E-62	MG-Homo	Fmnl2
AC149090.	1.97E-66	0.906325	0.443	0.445	4.83E-62	MG-Homo	AC149090.1
Rps6ka3	9.02E-66	1.419191	0.269	0.223	2.21E-61	MG-Homo	Rps6ka3
Tec	9.76E-66	1.347307	0.316	0.284	2.39E-61	MG-Homo	Tec
Ythdf3	1.14E-65	1.388063	0.252	0.202	2.80E-61	MG-Homo	Ythdf3
Pias1	7.20E-65	1.05238	0.397	0.395	1.76E-60	MG-Homo	Pias1
Fnip1	1.29E-63	1.196504	0.346	0.323	3.17E-59	MG-Homo	Fnip1
Ptprc	1.75E-63	0.646834	0.536	0.586	4.28E-59	MG-Homo	Ptprc
Hpcal1	1.96E-63	0.288254	0.164	0.285	4.80E-59	MG-Homo	Hpcal1
Kif13b	2.42E-63	1.421847	0.264	0.22	5.92E-59	MG-Homo	Kif13b
Ptpra	2.87E-63	0.870737	0.47	0.501	7.02E-59	MG-Homo	Ptpra
Cep170	6.31E-63	1.278655	0.308	0.272	1.55E-58	MG-Homo	Cep170
Pros1	1.76E-62	0.798665	0.468	0.477	4.30E-58	MG-Homo	Pros1
Ubr5	2.59E-62	1.390545	0.272	0.229	6.35E-58	MG-Homo	Ubr5
Snx30	3.11E-62	1.344569	0.279	0.237	7.62E-58	MG-Homo	Snx30
Ccr5	9.72E-62	0.786434	0.491	0.499	2.38E-57	MG-Homo	Ccr5
Aim2	1.88E-61	1.075359	0.379	0.37	4.60E-57	MG-Homo	Aim2
Ndufs4	8.80E-61	0.26566	0.193	0.325	2.15E-56	MG-Homo	Ndufs4
Pacs1	2.77E-60	1.468949	0.253	0.207	6.78E-56	MG-Homo	Pacs1
Herc1	5.63E-60	1.257247	0.286	0.246	1.38E-55	MG-Homo	Herc1
Atad2b	8.16E-60	1.382238	0.266	0.223	2.00E-55	MG-Homo	Atad2b
Kat6a	2.61E-59	1.292639	0.286	0.247	6.39E-55	MG-Homo	Kat6a
Rapgef2	3.51E-59	1.396222	0.25	0.203	8.59E-55	MG-Homo	Rapgef2
Gpatch8	9.32E-59	1.299366	0.288	0.251	2.28E-54	MG-Homo	Gpatch8

Nfatc1	2.32E-57	1.279327	0.311	0.282	5.68E-53	MG-Homo	Nfatc1
Itgav	2.56E-57	1.111493	0.34	0.316	6.27E-53	MG-Homo	Itgav
Trip12	3.31E-57	1.235478	0.32	0.295	8.11E-53	MG-Homo	Trip12
Nfat5	5.29E-57	1.30044	0.279	0.24	1.29E-52	MG-Homo	Nfat5
Notch2	7.99E-57	1.097291	0.346	0.325	1.96E-52	MG-Homo	Notch2
Ptk2b	9.90E-57	1.149962	0.363	0.352	2.43E-52	MG-Homo	Ptk2b
Larp4b	1.06E-56	1.128561	0.355	0.344	2.59E-52	MG-Homo	Larp4b
Tmcc1	4.86E-56	0.917512	0.375	0.358	1.19E-51	MG-Homo	Tmcc1
Nsmce2	8.41E-56	1.407466	0.307	0.28	2.06E-51	MG-Homo	Nsmce2
Asxl1	1.34E-55	1.32516	0.265	0.225	3.29E-51	MG-Homo	Asxl1
Abcc5	2.38E-55	1.329252	0.259	0.217	5.84E-51	MG-Homo	Abcc5
Tox4	2.49E-55	0.273574	0.175	0.294	6.10E-51	MG-Homo	Tox4
Dpy19l4	3.46E-55	1.265193	0.298	0.267	8.48E-51	MG-Homo	Dpy19l4
Tnrc6c	7.65E-55	1.326825	0.287	0.254	1.87E-50	MG-Homo	Tnrc6c
Il6st	7.88E-55	1.027913	0.373	0.363	1.93E-50	MG-Homo	Il6st
Ccny	1.13E-54	1.390285	0.273	0.237	2.77E-50	MG-Homo	Ccny
Lrrc8d	1.69E-54	1.310285	0.304	0.277	4.14E-50	MG-Homo	Lrrc8d
Pum2	2.53E-54	1.19296	0.325	0.303	6.21E-50	MG-Homo	Pum2
Gsap	4.02E-54	1.239613	0.314	0.289	9.84E-50	MG-Homo	Gsap
Mbtd1	4.38E-54	1.376693	0.266	0.229	1.07E-49	MG-Homo	Mbtd1
Itsn2	1.09E-53	1.203622	0.336	0.32	2.66E-49	MG-Homo	Itsn2
Fcho2	1.61E-52	0.997682	0.389	0.393	3.95E-48	MG-Homo	Fcho2
Srsf6	2.77E-52	0.255075	0.189	0.311	6.78E-48	MG-Homo	Srsf6
Evl	4.30E-52	0.864853	0.459	0.495	1.05E-47	MG-Homo	Evl
Ube2r2	1.18E-51	0.306799	0.156	0.262	2.88E-47	MG-Homo	Ube2r2
Zbtb7a	2.14E-51	0.27918	0.184	0.301	5.25E-47	MG-Homo	Zbtb7a
St6gal1	2.56E-51	1.438972	0.261	0.224	6.27E-47	MG-Homo	St6gal1
Fam117b	3.20E-51	1.289163	0.28	0.247	7.83E-47	MG-Homo	Fam117b
Fus	4.73E-51	0.721885	0.496	0.546	1.16E-46	MG-Homo	Fus
Trim8	5.86E-51	0.261482	0.152	0.253	1.44E-46	MG-Homo	Trim8
Trim44	6.96E-51	1.292224	0.273	0.239	1.71E-46	MG-Homo	Trim44
Cd180	1.10E-50	0.742836	0.49	0.525	2.70E-46	MG-Homo	Cd180
Rin3	4.35E-50	1.238939	0.27	0.233	1.06E-45	MG-Homo	Rin3
Zfp644	5.38E-50	1.220074	0.3	0.274	1.32E-45	MG-Homo	Zfp644
Stard3	5.62E-50	0.25523	0.21	0.337	1.38E-45	MG-Homo	Stard3
Kdm3b	8.78E-50	1.223145	0.293	0.264	2.15E-45	MG-Homo	Kdm3b
Arl10	1.25E-49	0.275396	0.159	0.263	3.06E-45	MG-Homo	Arl10
Ccdc93	1.90E-49	1.319864	0.257	0.22	4.66E-45	MG-Homo	Ccdc93
Znrf1	2.51E-49	1.344472	0.255	0.218	6.16E-45	MG-Homo	Znrf1
Huwe1	2.73E-49	1.253801	0.289	0.261	6.67E-45	MG-Homo	Huwe1
Abr	3.76E-49	1.318712	0.327	0.314	9.21E-45	MG-Homo	Abr
Psme4	5.11E-49	1.321224	0.253	0.216	1.25E-44	MG-Homo	Psme4
Mia3	5.18E-49	0.293373	0.15	0.25	1.27E-44	MG-Homo	Mia3
Plxdc1	1.44E-48	1.333568	0.268	0.235	3.52E-44	MG-Homo	Plxdc1

Eef2k	3.30E-48	1.236128	0.278	0.245	8.08E-44	MG-Homo	Eef2k
Phip	3.34E-48	1.20786	0.288	0.258	8.18E-44	MG-Homo	Phip
Ube2f	5.76E-48	0.342119	0.181	0.296	1.41E-43	MG-Homo	Ube2f
Chd4	1.08E-47	0.294438	0.213	0.34	2.64E-43	MG-Homo	Chd4
Setd2	1.21E-47	1.214045	0.298	0.274	2.97E-43	MG-Homo	Setd2
Pmepa1	1.53E-47	0.493782	0.585	0.61	3.76E-43	MG-Homo	Pmepa1
Sf1	8.58E-47	0.258093	0.221	0.352	2.10E-42	MG-Homo	Sf1
Ttr	8.75E-47	0.372552	0.539	0.506	2.14E-42	MG-Homo	Ttr
Gsk3b	1.80E-46	1.075482	0.342	0.332	4.41E-42	MG-Homo	Gsk3b
Srsf7	2.71E-46	0.26128	0.298	0.464	6.64E-42	MG-Homo	Srsf7
Pik3cb	2.75E-46	1.273192	0.272	0.242	6.73E-42	MG-Homo	Pik3cb
Slc38a10	3.21E-46	0.259139	0.18	0.29	7.87E-42	MG-Homo	Slc38a10
Erbin	3.90E-46	1.171371	0.32	0.305	9.55E-42	MG-Homo	Erbin
Rb1	9.88E-46	1.328161	0.252	0.217	2.42E-41	MG-Homo	Rb1
Tnfaip8	1.41E-45	0.270888	0.222	0.35	3.45E-41	MG-Homo	Tnfaip8
Lrch3	1.74E-45	1.260528	0.279	0.251	4.26E-41	MG-Homo	Lrch3
Usp15	1.80E-45	1.197926	0.286	0.259	4.40E-41	MG-Homo	Usp15
Mark2	2.01E-45	1.331867	0.267	0.237	4.93E-41	MG-Homo	Mark2
mt-Nd3	2.34E-45	0.27862	0.174	0.278	5.73E-41	MG-Homo	mt-Nd3
Rab5a	4.08E-45	0.270516	0.157	0.255	9.99E-41	MG-Homo	Rab5a
Crlf3	5.01E-45	0.95719	0.398	0.416	1.23E-40	MG-Homo	Crlf3
Tnrc18	1.01E-44	1.193485	0.303	0.282	2.46E-40	MG-Homo	Tnrc18
Sh3gl1	1.05E-44	0.355247	0.188	0.302	2.58E-40	MG-Homo	Sh3gl1
Il10ra	1.13E-44	0.519636	0.613	0.691	2.77E-40	MG-Homo	Il10ra
Ppp2r5c	1.30E-44	1.176197	0.33	0.321	3.17E-40	MG-Homo	Ppp2r5c
Irf2	6.45E-44	0.920312	0.416	0.445	1.58E-39	MG-Homo	Irf2
Arl8b	9.40E-44	0.26147	0.219	0.344	2.30E-39	MG-Homo	Arl8b
Nr3c1	1.27E-43	1.039902	0.372	0.379	3.10E-39	MG-Homo	Nr3c1
Osbpl8	3.00E-43	1.239416	0.279	0.252	7.36E-39	MG-Homo	Osbpl8
Nsd3	1.02E-42	1.090379	0.332	0.323	2.50E-38	MG-Homo	Nsd3
Hacd2	1.11E-42	0.325154	0.164	0.264	2.73E-38	MG-Homo	Hacd2
Dnm2	1.12E-42	0.808609	0.469	0.523	2.74E-38	MG-Homo	Dnm2
Zc3hav1	2.44E-42	0.309598	0.161	0.258	5.98E-38	MG-Homo	Zc3hav1
Vmp1	3.38E-42	0.322022	0.189	0.3	8.28E-38	MG-Homo	Vmp1
Ppcdc	9.35E-42	0.910464	0.389	0.396	2.29E-37	MG-Homo	Ppcdc
Senp2	1.09E-41	1.062269	0.339	0.334	2.67E-37	MG-Homo	Senp2
Cebpz	1.11E-41	0.291141	0.199	0.313	2.72E-37	MG-Homo	Cebpz
Rasa4	1.48E-41	0.304211	0.239	0.372	3.62E-37	MG-Homo	Rasa4
Dnajc13	1.51E-41	1.234831	0.281	0.259	3.69E-37	MG-Homo	Dnajc13
Dync1i2	1.53E-41	0.330016	0.166	0.266	3.75E-37	MG-Homo	Dync1i2
Arhgap31	1.70E-41	1.155545	0.323	0.311	4.16E-37	MG-Homo	Arhgap31
Ifnar1	2.35E-41	0.274231	0.161	0.256	5.75E-37	MG-Homo	Ifnar1
Dennd1b	2.68E-41	1.185134	0.26	0.23	6.56E-37	MG-Homo	Dennd1b
Ncoa3	4.03E-41	1.128147	0.336	0.334	9.87E-37	MG-Homo	Ncoa3

Ascc3	4.90E-41	1.258537	0.254	0.224	1.20E-36	MG-Homo	Ascc3
Rbm25	6.42E-41	0.999656	0.382	0.4	1.57E-36	MG-Homo	Rbm25
Taz	6.63E-41	0.362645	0.167	0.266	1.62E-36	MG-Homo	Taz
Kif5b	1.38E-40	0.258177	0.225	0.349	3.38E-36	MG-Homo	Kif5b
Dnajc1	2.38E-40	1.240763	0.297	0.282	5.84E-36	MG-Homo	Dnajc1
Hdlbp	3.68E-40	0.335393	0.204	0.318	9.01E-36	MG-Homo	Hdlbp
Ube2e1	1.32E-39	0.430037	0.176	0.28	3.22E-35	MG-Homo	Ube2e1
Ythdf2	1.70E-39	0.359333	0.169	0.268	4.17E-35	MG-Homo	Ythdf2
Cdk13	1.79E-39	1.2259	0.289	0.271	4.39E-35	MG-Homo	Cdk13
Tnfrsf11a	1.91E-39	0.979571	0.374	0.381	4.67E-35	MG-Homo	Tnfrsf11a
Cr1l	2.73E-39	0.36715	0.166	0.263	6.69E-35	MG-Homo	Cr1l
Mgat1	3.92E-39	0.302124	0.192	0.299	9.61E-35	MG-Homo	Mgat1
Copa	1.59E-38	0.287818	0.198	0.308	3.89E-34	MG-Homo	Copa
Akap10	1.69E-38	1.249711	0.257	0.23	4.15E-34	MG-Homo	Akap10
Bmpr2	2.21E-38	1.165575	0.27	0.244	5.42E-34	MG-Homo	Bmpr2
Sgpl1	2.43E-38	0.284266	0.228	0.352	5.94E-34	MG-Homo	Sgpl1
Ndfip2	3.00E-38	0.32196	0.181	0.283	7.36E-34	MG-Homo	Ndfip2
Mrtfa	1.44E-37	1.262444	0.256	0.23	3.53E-33	MG-Homo	Mrtfa
Dtnbp1	2.11E-37	0.380664	0.199	0.31	5.17E-33	MG-Homo	Dtnbp1
Vgll4	3.18E-37	1.160331	0.299	0.285	7.80E-33	MG-Homo	Vgll4
Znrf2	3.77E-37	1.196088	0.332	0.335	9.24E-33	MG-Homo	Znrf2
Tab2	4.65E-37	1.021262	0.345	0.347	1.14E-32	MG-Homo	Tab2
Tgs1	4.70E-37	0.345443	0.195	0.302	1.15E-32	MG-Homo	Tgs1
Elk3	5.21E-37	1.110097	0.342	0.345	1.28E-32	MG-Homo	Elk3
Iffo1	5.40E-37	1.192694	0.264	0.239	1.32E-32	MG-Homo	Iffo1
Adam10	5.80E-37	0.894937	0.4	0.424	1.42E-32	MG-Homo	Adam10
Hipk2	6.45E-37	1.175875	0.3	0.288	1.58E-32	MG-Homo	Hipk2
Cops4	7.34E-37	0.397929	0.164	0.258	1.80E-32	MG-Homo	Cops4
Rap2a	1.51E-36	0.31871	0.199	0.306	3.71E-32	MG-Homo	Rap2a
Vav1	1.54E-36	0.771607	0.454	0.51	3.78E-32	MG-Homo	Vav1
Stag2	2.12E-36	1.217492	0.277	0.259	5.18E-32	MG-Homo	Stag2
Slc7a7	2.34E-36	1.013022	0.366	0.38	5.73E-32	MG-Homo	Slc7a7
Prcp	2.80E-36	0.315542	0.256	0.393	6.85E-32	MG-Homo	Prcp
Basp1	3.92E-36	0.404596	0.643	0.698	9.60E-32	MG-Homo	Basp1
Stambpl1	4.63E-36	1.187236	0.26	0.234	1.13E-31	MG-Homo	Stambpl1
Tnpo1	1.34E-35	1.101919	0.314	0.309	3.29E-31	MG-Homo	Tnpo1
Pten	3.10E-35	0.846126	0.416	0.453	7.60E-31	MG-Homo	Pten
Aff1	4.43E-35	1.249771	0.257	0.234	1.09E-30	MG-Homo	Aff1
Ptgs1	5.07E-35	0.387685	0.608	0.633	1.24E-30	MG-Homo	Ptgs1
Mknk1	6.72E-35	0.931962	0.382	0.4	1.64E-30	MG-Homo	Mknk1
Pla2g4a	7.93E-35	1.274223	0.263	0.243	1.94E-30	MG-Homo	Pla2g4a
Lpcat1	1.97E-34	0.380243	0.163	0.254	4.81E-30	MG-Homo	Lpcat1
Cd86	2.37E-34	0.592733	0.529	0.602	5.81E-30	MG-Homo	Cd86
Rubcnl	3.05E-34	1.17948	0.261	0.238	7.47E-30	MG-Homo	Rubcnl

Ddi2	3.55E-34	1.237589	0.269	0.251	8.69E-30	MG-Homo	Ddi2
Skil	4.08E-34	1.088094	0.343	0.348	1.00E-29	MG-Homo	Skil
Rab1a	1.12E-33	0.323249	0.25	0.38	2.74E-29	MG-Homo	Rab1a
Tlr7	1.42E-33	0.980807	0.351	0.356	3.48E-29	MG-Homo	Tlr7
Gdi1	1.43E-33	0.356498	0.17	0.26	3.51E-29	MG-Homo	Gdi1
Rsf1	3.17E-33	1.198399	0.263	0.244	7.77E-29	MG-Homo	Rsf1
5031439G(	4.03E-33	0.384529	0.168	0.258	9.86E-29	MG-Homo	5031439G07Rik
Ube2j1	6.21E-33	0.360377	0.211	0.32	1.52E-28	MG-Homo	Ube2j1
Arl4c	9.74E-33	0.338476	0.223	0.336	2.39E-28	MG-Homo	Arl4c
Kctd12	1.98E-32	0.57676	0.561	0.631	4.84E-28	MG-Homo	Kctd12
Dnajb6	6.78E-32	0.353625	0.229	0.346	1.66E-27	MG-Homo	Dnajb6
Slc15a3	8.24E-32	0.445444	0.168	0.255	2.02E-27	MG-Homo	Slc15a3
Cebpg	9.24E-32	0.353021	0.237	0.355	2.26E-27	MG-Homo	Cebpg
Atf7ip	1.05E-31	1.130072	0.287	0.274	2.58E-27	MG-Homo	Atf7ip
Pkig	4.17E-31	0.740913	0.462	0.522	1.02E-26	MG-Homo	Pkig
Klf13	4.30E-31	0.277582	0.225	0.333	1.05E-26	MG-Homo	Klf13
R3hdm2	5.11E-31	1.248791	0.261	0.244	1.25E-26	MG-Homo	R3hdm2
P2rx7	5.61E-31	0.754572	0.37	0.379	1.37E-26	MG-Homo	P2rx7
Vps4b	6.19E-31	0.366728	0.167	0.255	1.52E-26	MG-Homo	Vps4b
Rin2	1.80E-30	0.874006	0.385	0.41	4.42E-26	MG-Homo	Rin2
Clk1	3.76E-30	0.974846	0.342	0.352	9.21E-26	MG-Homo	Clk1
Pepd	4.74E-30	0.469372	0.179	0.272	1.16E-25	MG-Homo	Pepd
Yy1	6.79E-30	0.434293	0.167	0.253	1.66E-25	MG-Homo	Yy1
Acer3	7.40E-30	0.806863	0.429	0.477	1.81E-25	MG-Homo	Acer3
Il13ra1	7.75E-30	0.373757	0.199	0.298	1.90E-25	MG-Homo	Il13ra1
Myo5a	1.12E-29	0.36871	0.183	0.272	2.73E-25	MG-Homo	Myo5a
Rnpep	1.20E-29	0.380574	0.257	0.383	2.94E-25	MG-Homo	Rnpep
Gtf2i	1.22E-29	1.152217	0.264	0.248	2.99E-25	MG-Homo	Gtf2i
Sort1	1.30E-29	0.993583	0.307	0.301	3.18E-25	MG-Homo	Sort1
Otulinl	2.19E-29	0.425187	0.613	0.702	5.36E-25	MG-Homo	Otulinl
Abi1	2.37E-29	0.961851	0.361	0.384	5.79E-25	MG-Homo	Abi1
Rgmb	2.55E-29	0.352886	0.18	0.269	6.24E-25	MG-Homo	Rgmb
Setd3	4.07E-29	1.071557	0.331	0.343	9.97E-25	MG-Homo	Setd3
Birc6	4.29E-29	1.012865	0.334	0.344	1.05E-24	MG-Homo	Birc6
Zfr	4.48E-29	1.068152	0.306	0.305	1.10E-24	MG-Homo	Zfr
Gls	5.30E-29	1.162241	0.277	0.266	1.30E-24	MG-Homo	Gls
Dmxl1	9.29E-29	1.133722	0.253	0.233	2.27E-24	MG-Homo	Dmxl1
Cd84	1.72E-28	0.627679	0.497	0.568	4.21E-24	MG-Homo	Cd84
Susd6	5.71E-28	1.144277	0.305	0.306	1.40E-23	MG-Homo	Susd6
Atf6	7.15E-28	1.141613	0.274	0.263	1.75E-23	MG-Homo	Atf6
Bach1	9.04E-28	0.403092	0.181	0.269	2.21E-23	MG-Homo	Bach1
Card9	1.21E-27	0.437732	0.191	0.283	2.96E-23	MG-Homo	Card9
Taf15	1.24E-27	1.032127	0.31	0.311	3.05E-23	MG-Homo	Taf15
Snx18	1.49E-27	0.810743	0.399	0.429	3.64E-23	MG-Homo	Snx18

Zmynd11	4.98E-27	1.062147	0.291	0.285	1.22E-22	MG-Homo	Zmynd11
Elavl1	6.07E-27	0.445263	0.197	0.294	1.49E-22	MG-Homo	Elavl1
Taok3	1.19E-26	1.004689	0.352	0.376	2.93E-22	MG-Homo	Taok3
Tmod3	2.17E-26	0.437719	0.181	0.269	5.32E-22	MG-Homo	Tmod3
Trim30a	2.26E-26	0.339705	0.238	0.346	5.54E-22	MG-Homo	Trim30a
Fez2	4.06E-26	0.374264	0.204	0.299	9.94E-22	MG-Homo	Fez2
Sptlc2	4.60E-26	0.443715	0.226	0.336	1.13E-21	MG-Homo	Sptlc2
Fam91a1	4.66E-26	0.99997	0.296	0.29	1.14E-21	MG-Homo	Fam91a1
Atp6v0a1	7.27E-26	0.917913	0.342	0.355	1.78E-21	MG-Homo	Atp6v0a1
Usp12	1.03E-25	1.284268	0.254	0.244	2.53E-21	MG-Homo	Usp12
Tbc1d23	1.56E-25	1.126611	0.262	0.251	3.82E-21	MG-Homo	Tbc1d23
Zmynd8	1.59E-25	1.137059	0.275	0.268	3.90E-21	MG-Homo	Zmynd8
Capza1	2.05E-25	0.331667	0.288	0.425	5.02E-21	MG-Homo	Capza1
Havcr2	3.58E-25	0.860449	0.355	0.371	8.76E-21	MG-Homo	Havcr2
U2surp	7.54E-25	0.457188	0.183	0.27	1.85E-20	MG-Homo	U2surp
Ldb1	8.03E-25	0.410548	0.222	0.326	1.97E-20	MG-Homo	Ldb1
Plxnb2	9.14E-25	0.258587	0.321	0.463	2.24E-20	MG-Homo	Plxnb2
mt-Cytb	1.96E-24	0.402262	0.913	0.959	4.80E-20	MG-Homo	mt-Cytb
Mark3	3.13E-24	1.123028	0.259	0.249	7.67E-20	MG-Homo	Mark3
Pds5a	4.18E-24	1.113722	0.254	0.241	1.02E-19	MG-Homo	Pds5a
Ncor2	5.18E-24	1.083301	0.289	0.289	1.27E-19	MG-Homo	Ncor2
Csde1	5.99E-24	0.438497	0.198	0.29	1.47E-19	MG-Homo	Csde1
Lcp2	8.59E-24	0.802854	0.413	0.465	2.10E-19	MG-Homo	Lcp2
Lmbrd1	1.26E-23	0.434371	0.193	0.283	3.08E-19	MG-Homo	Lmbrd1
Rsu1	1.58E-23	0.469692	0.235	0.344	3.87E-19	MG-Homo	Rsu1
Gns	1.84E-23	0.362532	0.628	0.712	4.51E-19	MG-Homo	Gns
Tmem104	2.12E-23	1.161318	0.271	0.269	5.20E-19	MG-Homo	Tmem104
Anxa3	2.74E-23	0.466816	0.542	0.611	6.71E-19	MG-Homo	Anxa3
Rgl1	5.11E-23	1.084979	0.27	0.263	1.25E-18	MG-Homo	Rgl1
Chic2	7.14E-23	0.450096	0.218	0.317	1.75E-18	MG-Homo	Chic2
Gpr34	7.45E-23	0.383992	0.644	0.674	1.82E-18	MG-Homo	Gpr34
Snx10	8.28E-23	0.507669	0.187	0.273	2.03E-18	MG-Homo	Snx10
Ggnbp2	8.75E-23	0.456801	0.198	0.287	2.14E-18	MG-Homo	Ggnbp2
Tm9sf2	9.79E-23	0.324025	0.304	0.441	2.40E-18	MG-Homo	Tm9sf2
Atrx	9.97E-23	1.040938	0.308	0.316	2.44E-18	MG-Homo	Atrx
Hist1h1e	1.05E-22	0.299805	0.214	0.3	2.57E-18	MG-Homo	Hist1h1e
Tcirg1	2.36E-22	0.676839	0.464	0.533	5.78E-18	MG-Homo	Tcirg1
Wsb1	2.50E-22	0.933544	0.35	0.376	6.13E-18	MG-Homo	Wsb1
Nfkbiz	3.10E-22	0.439677	0.194	0.272	7.59E-18	MG-Homo	Nfkbiz
Rhoq	4.68E-22	1.118296	0.262	0.254	1.15E-17	MG-Homo	Rhoq
Ptpro	6.25E-22	0.908903	0.326	0.334	1.53E-17	MG-Homo	Ptpro
Cbl	1.27E-21	0.8801	0.352	0.377	3.11E-17	MG-Homo	Cbl
Wdr26	1.92E-21	0.95408	0.335	0.358	4.70E-17	MG-Homo	Wdr26
Washc4	2.31E-21	1.046414	0.273	0.27	5.65E-17	MG-Homo	Washc4

Herc4	2.39E-21	1.00448	0.269	0.262	5.84E-17	MG-Homo	Herc4
Ptpre	3.38E-21	0.295582	0.257	0.364	8.27E-17	MG-Homo	Ptpre
Hps3	3.40E-21	1.009786	0.3	0.305	8.32E-17	MG-Homo	Hps3
Eps15	3.41E-21	0.393442	0.175	0.251	8.36E-17	MG-Homo	Eps15
Chchd3	4.67E-21	1.20953	0.258	0.254	1.14E-16	MG-Homo	Chchd3
Gnb1	6.73E-21	0.298915	0.358	0.518	1.65E-16	MG-Homo	Gnb1
Map2k1	7.55E-21	0.534472	0.209	0.302	1.85E-16	MG-Homo	Map2k1
E330020D1	8.71E-21	0.320959	0.195	0.271	2.13E-16	MG-Homo	E330020D12Rik
Pum1	9.79E-21	1.131153	0.256	0.25	2.40E-16	MG-Homo	Pum1
Pon3	1.04E-20	0.45015	0.193	0.276	2.55E-16	MG-Homo	Pon3
Nipa2	1.55E-20	0.490358	0.189	0.271	3.79E-16	MG-Homo	Nipa2
Srsf10	3.42E-20	0.474525	0.184	0.263	8.37E-16	MG-Homo	Srsf10
Dennd5a	3.69E-20	1.035571	0.28	0.281	9.03E-16	MG-Homo	Dennd5a
Eif4a2	5.64E-20	0.361395	0.26	0.372	1.38E-15	MG-Homo	Eif4a2
Rcbtb2	6.31E-20	1.088811	0.298	0.308	1.55E-15	MG-Homo	Rcbtb2
Pnisr	6.32E-20	0.953618	0.318	0.331	1.55E-15	MG-Homo	Pnisr
Pnn	7.66E-20	0.493866	0.198	0.283	1.87E-15	MG-Homo	Pnn
Gng12	9.21E-20	0.53824	0.222	0.317	2.26E-15	MG-Homo	Gng12
Tcf25	1.77E-19	0.396382	0.284	0.41	4.32E-15	MG-Homo	Tcf25
Rtn3	1.98E-19	0.740799	0.433	0.502	4.84E-15	MG-Homo	Rtn3
Zfp36l1	2.50E-19	0.573896	0.573	0.647	6.13E-15	MG-Homo	Zfp36l1
Resf1	2.64E-19	0.444796	0.192	0.272	6.46E-15	MG-Homo	Resf1
Wdr33	2.85E-19	1.011964	0.307	0.322	6.97E-15	MG-Homo	Wdr33
Dhx9	4.02E-19	1.034982	0.291	0.298	9.83E-15	MG-Homo	Dhx9
Gspt1	5.47E-19	0.444393	0.237	0.337	1.34E-14	MG-Homo	Gspt1
Ikbkb	9.65E-19	0.465289	0.179	0.253	2.36E-14	MG-Homo	Ikbkb
Zup1	1.13E-18	1.00443	0.283	0.286	2.78E-14	MG-Homo	Zup1
Akap8l	1.25E-18	1.01582	0.305	0.318	3.06E-14	MG-Homo	Akap8l
Rabep1	2.46E-18	1.125381	0.258	0.257	6.02E-14	MG-Homo	Rabep1
Naa35	2.64E-18	0.460703	0.191	0.269	6.47E-14	MG-Homo	Naa35
Slc12a9	3.25E-18	0.419794	0.254	0.359	7.95E-14	MG-Homo	Slc12a9
Nfam1	4.24E-18	0.316799	0.266	0.372	1.04E-13	MG-Homo	Nfam1
N4bp1	4.52E-18	0.488792	0.181	0.254	1.11E-13	MG-Homo	N4bp1
Tor1aip1	7.16E-18	0.342597	0.317	0.452	1.75E-13	MG-Homo	Tor1aip1
Mindy2	1.59E-17	0.473227	0.191	0.268	3.90E-13	MG-Homo	Mindy2
Jak1	1.86E-17	0.895195	0.374	0.421	4.55E-13	MG-Homo	Jak1
Arid4a	1.92E-17	1.001954	0.294	0.305	4.70E-13	MG-Homo	Arid4a
Rasa3	2.01E-17	1.052663	0.269	0.271	4.92E-13	MG-Homo	Rasa3
Kdm2a	2.47E-17	1.036143	0.278	0.284	6.04E-13	MG-Homo	Kdm2a
Apobec3	3.46E-17	0.315188	0.324	0.453	8.47E-13	MG-Homo	Apobec3
Hnrnpul1	3.52E-17	0.510928	0.219	0.309	8.62E-13	MG-Homo	Hnrnpul1
Usp9x	4.13E-17	1.04003	0.263	0.263	1.01E-12	MG-Homo	Usp9x
Cryl1	5.69E-17	1.089237	0.264	0.266	1.39E-12	MG-Homo	Cryl1
Tspan7	7.81E-17	0.275358	0.301	0.414	1.91E-12	MG-Homo	Tspan7

Usp2	8.83E-17	0.326108	0.267	0.366	2.16E-12	MG-Homo	Usp2
Srrm1	9.62E-17	0.379653	0.307	0.437	2.36E-12	MG-Homo	Srrm1
Cmklr1	1.12E-16	1.117526	0.268	0.272	2.74E-12	MG-Homo	Cmklr1
Rlim	1.35E-16	0.496179	0.191	0.267	3.31E-12	MG-Homo	Rlim
Numa1	2.11E-16	0.484942	0.218	0.303	5.16E-12	MG-Homo	Numa1
Xrn2	2.33E-16	0.506852	0.195	0.274	5.69E-12	MG-Homo	Xrn2
Nktr	2.95E-16	0.972317	0.292	0.303	7.22E-12	MG-Homo	Nktr
Npc1	3.34E-16	0.516296	0.19	0.265	8.18E-12	MG-Homo	Npc1
Stx7	3.53E-16	0.307089	0.361	0.515	8.64E-12	MG-Homo	Stx7
Pak2	6.54E-16	0.35622	0.302	0.427	1.60E-11	MG-Homo	Pak2
Sel1l	7.73E-16	0.568621	0.18	0.252	1.89E-11	MG-Homo	Sel1l
Ogt	1.05E-15	0.958147	0.262	0.262	2.56E-11	MG-Homo	Ogt
Tra2a	1.69E-15	0.832287	0.361	0.402	4.14E-11	MG-Homo	Tra2a
Add1	1.91E-15	0.509166	0.185	0.257	4.67E-11	MG-Homo	Add1
Pdgfb	2.25E-15	1.101025	0.27	0.274	5.52E-11	MG-Homo	Pdgfb
Kras	2.46E-15	0.486557	0.185	0.256	6.03E-11	MG-Homo	Kras
Pik3cg	2.98E-15	0.94948	0.286	0.295	7.29E-11	MG-Homo	Pik3cg
Csnk1a1	3.19E-15	0.420623	0.281	0.394	7.81E-11	MG-Homo	Csnk1a1
mt-Nd2	1.14E-14	0.395268	0.706	0.823	2.79E-10	MG-Homo	mt-Nd2
Bptf	1.37E-14	0.994811	0.276	0.286	3.37E-10	MG-Homo	Bptf
Rsrc2	1.80E-14	0.475829	0.219	0.303	4.41E-10	MG-Homo	Rsrc2
Creb1	3.16E-14	1.074686	0.252	0.253	7.74E-10	MG-Homo	Creb1
St3gal5	3.82E-14	0.742873	0.346	0.368	9.37E-10	MG-Homo	St3gal5
Phf20l1	4.36E-14	0.896534	0.293	0.307	1.07E-09	MG-Homo	Phf20l1
Gm4951	8.32E-14	0.394506	0.199	0.264	2.04E-09	MG-Homo	Gm4951
Top1	8.75E-14	0.462915	0.263	0.365	2.14E-09	MG-Homo	Top1
1700017B0	1.16E-13	0.373852	0.272	0.37	2.85E-09	MG-Homo	1700017B05Rik
Elf2	1.18E-13	1.035462	0.3	0.324	2.88E-09	MG-Homo	Elf2
Abi3	1.23E-13	0.416895	0.516	0.585	3.01E-09	MG-Homo	Abi3
Map11	1.28E-13	0.509641	0.257	0.356	3.13E-09	MG-Homo	Map11
Pkn1	1.38E-13	0.835093	0.358	0.406	3.39E-09	MG-Homo	Pkn1
Rbbp6	1.96E-13	0.557111	0.208	0.286	4.80E-09	MG-Homo	Rbbp6
Aff4	2.97E-13	0.963535	0.275	0.286	7.27E-09	MG-Homo	Aff4
Lnpep	3.31E-13	0.767364	0.359	0.401	8.12E-09	MG-Homo	Lnpep
Snta1	3.74E-13	0.951788	0.262	0.266	9.15E-09	MG-Homo	Snta1
Ptbp3	4.24E-13	0.663476	0.423	0.501	1.04E-08	MG-Homo	Ptbp3
Scarb2	4.83E-13	0.263654	0.381	0.528	1.18E-08	MG-Homo	Scarb2
Brd4	4.93E-13	0.873628	0.344	0.385	1.21E-08	MG-Homo	Brd4
Clip1	6.43E-13	1.048358	0.261	0.27	1.58E-08	MG-Homo	Clip1
Slc38a2	8.85E-13	0.415352	0.207	0.278	2.17E-08	MG-Homo	Slc38a2
Mafg	9.11E-13	0.558212	0.192	0.261	2.23E-08	MG-Homo	Mafg
Ccdc50	1.53E-12	0.782544	0.348	0.387	3.75E-08	MG-Homo	Ccdc50
Gtpbp2	2.52E-12	0.55734	0.21	0.286	6.18E-08	MG-Homo	Gtpbp2
Prkcd	2.67E-12	0.250212	0.402	0.554	6.54E-08	MG-Homo	Prkcd

Dpp8	2.93E-12	1.01963	0.254	0.261	7.18E-08	MG-Homo	Dpp8
Mia2	3.08E-12	0.503151	0.261	0.359	7.55E-08	MG-Homo	Mia2
Hps4	5.42E-12	0.855583	0.327	0.359	1.33E-07	MG-Homo	Hps4
Actr2	1.02E-11	0.303511	0.38	0.529	2.50E-07	MG-Homo	Actr2
Il21r	1.06E-11	0.411426	0.29	0.398	2.60E-07	MG-Homo	Il21r
Ubl3	1.37E-11	0.447433	0.321	0.449	3.36E-07	MG-Homo	Ubl3
Abcg1	1.53E-11	0.690852	0.213	0.289	3.76E-07	MG-Homo	Abcg1
Golgb1	2.28E-11	0.56124	0.227	0.31	5.58E-07	MG-Homo	Golgb1
Rbm5	4.11E-11	0.918234	0.295	0.318	1.01E-06	MG-Homo	Rbm5
Arap1	4.79E-11	0.654511	0.188	0.254	1.17E-06	MG-Homo	Arap1
Dnajc7	5.14E-11	0.47963	0.282	0.388	1.26E-06	MG-Homo	Dnajc7
Maf	5.81E-11	0.489802	0.427	0.472	1.42E-06	MG-Homo	Maf
Fbrsl1	6.68E-11	0.868455	0.318	0.349	1.64E-06	MG-Homo	Fbrsl1
Ptp4a2	6.80E-11	0.322732	0.377	0.526	1.67E-06	MG-Homo	Ptp4a2
Nrp1	6.95E-11	0.795525	0.354	0.391	1.70E-06	MG-Homo	Nrp1
Hnrnpc	8.41E-11	0.603691	0.194	0.261	2.06E-06	MG-Homo	Hnrnpc
Neat1	1.03E-10	0.973181	0.234	0.314	2.52E-06	MG-Homo	Neat1
Atp2b1	1.12E-10	0.629428	0.406	0.471	2.75E-06	MG-Homo	Atp2b1
Ogdh	1.77E-10	0.600816	0.211	0.285	4.33E-06	MG-Homo	Ogdh
Abcc3	3.54E-10	0.833132	0.289	0.308	8.66E-06	MG-Homo	Abcc3
Ubap2l	4.55E-10	0.536186	0.233	0.315	1.11E-05	MG-Homo	Ubap2l
Fndc3a	4.62E-10	0.886034	0.287	0.308	1.13E-05	MG-Homo	Fndc3a
Kmt2e	4.74E-10	0.738362	0.361	0.411	1.16E-05	MG-Homo	Kmt2e
Cul1	6.46E-10	0.909296	0.296	0.322	1.58E-05	MG-Homo	Cul1
Ddx50	8.64E-10	0.594473	0.194	0.259	2.12E-05	MG-Homo	Ddx50
Sla	9.78E-10	0.841037	0.317	0.35	2.39E-05	MG-Homo	Sla
Ubac2	1.51E-09	0.93296	0.296	0.325	3.69E-05	MG-Homo	Ubac2
Mtdh	1.57E-09	0.340679	0.593	0.712	3.84E-05	MG-Homo	Mtdh
Rsrp1	1.80E-09	0.349737	0.599	0.718	4.41E-05	MG-Homo	Rsrp1
Snx9	1.86E-09	0.637367	0.209	0.28	4.55E-05	MG-Homo	Snx9
Prpf38b	2.28E-09	0.636165	0.205	0.274	5.58E-05	MG-Homo	Prpf38b
Edem1	3.03E-09	0.552183	0.256	0.345	7.42E-05	MG-Homo	Edem1
Gna15	3.24E-09	0.765292	0.345	0.388	7.95E-05	MG-Homo	Gna15
Lpxn	6.11E-09	0.570897	0.207	0.274	0.00015	MG-Homo	Lpxn
Samsn1	6.30E-09	0.361851	0.199	0.257	0.000154	MG-Homo	Samsn1
Ankhd1	7.03E-09	1.007228	0.245	0.256	0.000172	MG-Homo	Ankhd1
Tnfrsf21	1.00E-08	0.495059	0.265	0.355	0.000245	MG-Homo	Tnfrsf21
Nmt1	1.07E-08	0.520975	0.273	0.371	0.000263	MG-Homo	Nmt1
Rab10	1.11E-08	0.541543	0.279	0.379	0.000271	MG-Homo	Rab10
Gm43305	1.31E-08	0.399247	0.335	0.436	0.000321	MG-Homo	Gm43305
Abcd2	1.66E-08	0.960289	0.258	0.274	0.000406	MG-Homo	Abcd2
Spag9	2.73E-08	0.87207	0.285	0.311	0.000668	MG-Homo	Spag9
Stau1	3.19E-08	1.007723	0.242	0.255	0.00078	MG-Homo	Stau1
Slc6a6	3.90E-08	0.519999	0.293	0.398	0.000956	MG-Homo	Slc6a6

Myo9b	4.17E-08	0.975851	0.286	0.316	0.001021	MG-Homo	Myo9b
Scaf11	5.09E-08	0.488771	0.273	0.365	0.001246	MG-Homo	Scaf11
Stat3	6.32E-08	0.480673	0.296	0.399	0.001549	MG-Homo	Stat3
Rap1a	6.49E-08	0.268219	0.412	0.563	0.001589	MG-Homo	Rap1a
Zfand5	7.36E-08	0.627835	0.271	0.361	0.001803	MG-Homo	Zfand5
Cd2ap	7.61E-08	0.794377	0.327	0.37	0.001863	MG-Homo	Cd2ap
Taok1	8.55E-08	0.897725	0.267	0.286	0.002094	MG-Homo	Taok1
Hnrnph1	8.97E-08	0.841116	0.297	0.326	0.002197	MG-Homo	Hnrnph1
Zfand6	1.63E-07	0.878891	0.284	0.311	0.003986	MG-Homo	Zfand6
Dgkz	1.95E-07	0.69294	0.232	0.308	0.004771	MG-Homo	Dgkz
Ubn1	2.38E-07	0.648299	0.198	0.26	0.005839	MG-Homo	Ubn1
Lpar6	2.52E-07	0.513683	0.233	0.303	0.006174	MG-Homo	Lpar6
Wac	2.66E-07	0.867754	0.292	0.322	0.006524	MG-Homo	Wac
Elf4	2.95E-07	1.011487	0.25	0.267	0.007224	MG-Homo	Elf4
Btg2	3.67E-07	0.292787	0.514	0.64	0.008998	MG-Homo	Btg2
Ccnl1	4.51E-07	0.876517	0.314	0.355	0.01105	MG-Homo	Ccnl1
Ecscr	4.76E-07	0.364454	0.243	0.304	0.011669	MG-Homo	Ecscr
Tra2b	5.44E-07	0.5083	0.301	0.404	0.013333	MG-Homo	Tra2b
Plek	5.81E-07	0.584888	0.554	0.679	0.014219	MG-Homo	Plek
Stx8	6.22E-07	1.09519	0.26	0.285	0.015232	MG-Homo	Stx8
Dpysl2	6.36E-07	0.970376	0.263	0.284	0.015566	MG-Homo	Dpysl2
Sgk1	6.67E-07	0.339374	0.321	0.403	0.016345	MG-Homo	Sgk1
Wwp2	7.31E-07	0.706619	0.195	0.255	0.017898	MG-Homo	Wwp2
Glul	1.08E-06	0.415423	0.655	0.76	0.026456	MG-Homo	Glul
Rasal3	1.94E-06	0.631207	0.211	0.273	0.047558	MG-Homo	Rasal3
Sesn1	2.08E-06	0.82234	0.284	0.309	0.050984	MG-Homo	Sesn1
Ptpn1	2.27E-06	0.370577	0.383	0.518	0.055511	MG-Homo	Ptpn1
Nlrc5	2.48E-06	0.692618	0.197	0.252	0.060684	MG-Homo	Nlrc5
Leng8	2.51E-06	0.924503	0.256	0.276	0.061544	MG-Homo	Leng8
Gapvd1	2.69E-06	0.903394	0.257	0.279	0.06582	MG-Homo	Gapvd1
Rab3il1	2.87E-06	0.379487	0.334	0.44	0.070276	MG-Homo	Rab3il1
Hnrnph3	3.46E-06	0.616406	0.211	0.273	0.084653	MG-Homo	Hnrnph3
Xiap	3.95E-06	0.930033	0.251	0.271	0.096651	MG-Homo	Xiap
Nisch	4.42E-06	0.736485	0.322	0.365	0.108214	MG-Homo	Nisch
Gpr183	5.09E-06	0.432327	0.282	0.359	0.124694	MG-Homo	Gpr183
Rapgef6	5.29E-06	0.840027	0.265	0.287	0.129567	MG-Homo	Rapgef6
Slc12a2	6.66E-06	0.832585	0.259	0.277	0.163154	MG-Homo	Slc12a2
Ncor1	8.43E-06	0.703604	0.361	0.427	0.206487	MG-Homo	Ncor1
Cdc42se2	9.99E-06	0.675365	0.222	0.288	0.24466	MG-Homo	Cdc42se2
Btbd1	1.18E-05	0.649623	0.213	0.275	0.290148	MG-Homo	Btbd1
Tnfrsf1b	1.45E-05	0.465037	0.285	0.371	0.355757	MG-Homo	Tnfrsf1b
Acin1	1.64E-05	0.574565	0.271	0.356	0.401364	MG-Homo	Acin1
Fubp1	2.32E-05	0.612509	0.209	0.267	0.567528	MG-Homo	Fubp1
P2ry13	3.25E-05	0.362241	0.345	0.44	0.795447	MG-Homo	P2ry13

Lpcat3	4.20E-05	0.524058	0.267	0.345	1	MG-Homo	Lpcat3
Itfg1	4.69E-05	0.658216	0.212	0.27	1	MG-Homo	Itfg1
Hck	4.99E-05	0.327848	0.437	0.583	1	MG-Homo	Hck
Arhgef2	5.21E-05	0.641494	0.232	0.299	1	MG-Homo	Arhgef2
Syk	5.46E-05	0.756179	0.314	0.36	1	MG-Homo	Syk
Khdrbs1	5.54E-05	0.552867	0.293	0.387	1	MG-Homo	Khdrbs1
P4ha1	6.00E-05	0.523246	0.3	0.392	1	MG-Homo	P4ha1
Fam168b	6.64E-05	0.640741	0.25	0.325	1	MG-Homo	Fam168b
Oxct1	9.75E-05	0.663883	0.377	0.449	1	MG-Homo	Oxct1
Scamp5	0.000104	0.893594	0.243	0.263	1	MG-Homo	Scamp5
Tial1	0.00014	0.715624	0.199	0.252	1	MG-Homo	Tial1
Kdm7a	0.000155	0.836877	0.245	0.264	1	MG-Homo	Kdm7a
Tmem131	0.000174	0.802825	0.281	0.316	1	MG-Homo	Tmem131
Sipa1	0.000199	0.597825	0.275	0.357	1	MG-Homo	Sipa1
Lacc1	0.00023	0.641641	0.234	0.299	1	MG-Homo	Lacc1
Ube3a	0.00024	0.838488	0.258	0.285	1	MG-Homo	Ube3a
Arhgap30	0.000243	0.592135	0.266	0.344	1	MG-Homo	Arhgap30
Pcmtd1	0.000259	0.915223	0.234	0.253	1	MG-Homo	Pcmtd1
Sf3b1	0.000295	0.372655	0.383	0.511	1	MG-Homo	Sf3b1
Smox	0.000346	0.621143	0.235	0.297	1	MG-Homo	Smox
Arhgef1	0.000379	0.598686	0.233	0.294	1	MG-Homo	Arhgef1
Jun	0.000505	0.303006	0.583	0.65	1	MG-Homo	Jun
Mbp	0.000572	0.427732	0.275	0.329	1	MG-Homo	Mbp
Dnajc5	0.000784	0.654179	0.212	0.266	1	MG-Homo	Dnajc5
Elf1	0.000788	0.766664	0.321	0.378	1	MG-Homo	Elf1
Slc29a3	0.000987	0.519795	0.399	0.477	1	MG-Homo	Slc29a3
Ube2k	0.001041	0.918712	0.257	0.289	1	MG-Homo	Ube2k
Cept1	0.001068	0.686792	0.23	0.291	1	MG-Homo	Cept1
Larp1	0.001303	0.65503	0.255	0.327	1	MG-Homo	Larp1
Ythdc1	0.001321	0.697211	0.217	0.273	1	MG-Homo	Ythdc1
Ep300	0.001406	0.836356	0.276	0.313	1	MG-Homo	Ep300
Rp2	0.001512	0.659056	0.245	0.313	1	MG-Homo	Rp2
Cd33	0.001512	0.419872	0.468	0.563	1	MG-Homo	Cd33
mt-Nd4l	0.001534	0.311686	0.729	0.845	1	MG-Homo	mt-Nd4l
Arid5b	0.001873	0.920982	0.297	0.341	1	MG-Homo	Arid5b
Ankrd12	0.001977	0.931616	0.235	0.258	1	MG-Homo	Ankrd12
Smchd1	0.002232	0.678436	0.216	0.27	1	MG-Homo	Smchd1
Rock1	0.002896	0.8146	0.278	0.317	1	MG-Homo	Rock1
Adam17	0.003455	0.56546	0.303	0.392	1	MG-Homo	Adam17
Hectd1	0.003535	0.650973	0.229	0.287	1	MG-Homo	Hectd1
Prrc2c	0.003544	0.562607	0.299	0.385	1	MG-Homo	Prrc2c
Kpna4	0.004282	0.578975	0.387	0.469	1	MG-Homo	Kpna4
Wls	0.005029	0.688903	0.261	0.332	1	MG-Homo	Wls
Mthfs	0.005052	1.074513	0.251	0.285	1	MG-Homo	Mthfs

Synj1	0.005713	0.789272	0.271	0.308	1	MG-Homo	Synj1
Fkbp15	0.006623	0.795794	0.295	0.345	1	MG-Homo	Fkbp15
Zdhhc20	0.006672	0.785881	0.29	0.336	1	MG-Homo	Zdhhc20
Gramd1a	0.006901	0.951637	0.227	0.251	1	MG-Homo	Gramd1a
Gna13	0.007203	0.629738	0.294	0.38	1	MG-Homo	Gna13
Kcnk6	0.007387	0.814375	0.239	0.264	1	MG-Homo	Kcnk6
Senp6	0.007702	0.812046	0.264	0.299	1	MG-Homo	Senp6
Tm6sf1	0.008387	0.505558	0.304	0.385	1	MG-Homo	Tm6sf1
Gm26740	0.009902	0.765181	0.277	0.315	1	MG-Homo	Gm26740
Tmem119	0	1.343659	0.944	0.59	0	MG-Inflam	Tmem119
Fcrls	0	1.429258	0.923	0.579	0	MG-Inflam	Fcrls
Gpr341	0	1.409235	0.951	0.618	0	MG-Inflam	Gpr34
Siglech1	0	1.169129	0.935	0.615	0	MG-Inflam	Siglech
Ecscr1	0	1.636829	0.565	0.245	0	MG-Inflam	Ecscr
Olfml3	0	1.219104	0.961	0.645	0	MG-Inflam	Olfml3
Slc2a5	0	1.590974	0.535	0.233	0	MG-Inflam	Slc2a5
Golm11	0	1.253653	0.766	0.47	0	MG-Inflam	Golm1
Csmd31	0	0.416457	0.635	0.348	0	MG-Inflam	Csmd3
Adgrg1	0	1.301102	0.636	0.35	0	MG-Inflam	Adgrg1
Rnase4	0	1.266154	0.83	0.545	0	MG-Inflam	Rnase4
Sparc	0	1.213216	0.99	0.708	0	MG-Inflam	Sparc
P2ry131	0	1.331162	0.661	0.38	0	MG-Inflam	P2ry13
Nav31	0	0.486545	0.701	0.42	0	MG-Inflam	Nav3
Ltc4s	0	1.041688	0.857	0.584	0	MG-Inflam	Ltc4s
Serpine2	0	0.781681	0.82	0.551	0	MG-Inflam	Serpine2
P2ry121	0	1.507128	0.986	0.726	0	MG-Inflam	P2ry12
F11r	0	1.059537	0.814	0.558	0	MG-Inflam	F11r
Fscn1	0	0.973316	0.568	0.313	0	MG-Inflam	Fscn1
Susd3	0	1.185968	0.682	0.439	0	MG-Inflam	Susd3
Crybb1	0	0.740567	0.717	0.476	0	MG-Inflam	Crybb1
Ifitm101	0	1.031513	0.519	0.278	0	MG-Inflam	Ifitm10
Ctsl	0	0.55523	0.958	0.717	0	MG-Inflam	Ctsl
Ptgs11	0	0.862637	0.832	0.592	0	MG-Inflam	Ptgs1
Numb1	0	0.733426	0.789	0.555	0	MG-Inflam	Numb
Cd164	0	1.245735	0.636	0.408	0	MG-Inflam	Cd164
Gm107901	0	0.748566	0.688	0.46	0	MG-Inflam	Gm10790
Pmp22	0	1.032286	0.54	0.322	0	MG-Inflam	Pmp22
Cd81	0	0.798367	0.992	0.778	0	MG-Inflam	Cd81
Cd37	0	0.914384	0.831	0.618	0	MG-Inflam	Cd37
Trem2	0	0.754529	0.982	0.769	0	MG-Inflam	Trem2
Cd9	0	0.512295	0.932	0.72	0	MG-Inflam	Cd9
Vsir1	0	1.093442	0.909	0.697	0	MG-Inflam	Vsir
Selplg	0	1.477663	0.988	0.776	0	MG-Inflam	Selplg
Plxdc21	0	0.275293	0.943	0.732	0	MG-Inflam	Plxdc2

Slco2b11	0	0.684804	0.815	0.604	0	MG-Inflam	Slco2b1
Cfh1	0	0.794613	0.792	0.582	0	MG-Inflam	Cfh
Tmem173	0	1.016387	0.67	0.461	0	MG-Inflam	Tmem173
Hpgd	0	0.733044	0.71	0.501	0	MG-Inflam	Hpgd
Tanc21	0	0.335427	0.907	0.7	0	MG-Inflam	Tanc2
St3gal61	0	0.708684	0.72	0.515	0	MG-Inflam	St3gal6
Gal3st4	0	1.093946	0.528	0.325	0	MG-Inflam	Gal3st4
Rhob	0	0.541022	0.896	0.696	0	MG-Inflam	Rhob
Scoc	0	0.984254	0.632	0.433	0	MG-Inflam	Scoc
Pmepa11	0	0.802656	0.774	0.576	0	MG-Inflam	Pmepa1
Sall11	0	0.81962	0.601	0.407	0	MG-Inflam	Sall1
Trf	0	0.686919	0.899	0.708	0	MG-Inflam	Trf
Phyhd11	0	0.713121	0.707	0.522	0	MG-Inflam	Phyhd1
Cd68	0	0.557055	0.915	0.732	0	MG-Inflam	Cd68
Usp21	0	1.068102	0.503	0.321	0	MG-Inflam	Usp2
Fcgr3	0	0.527656	0.934	0.755	0	MG-Inflam	Fcgr3
Lpcat21	0	0.75065	0.952	0.774	0	MG-Inflam	Lpcat2
Basp11	0	0.674725	0.839	0.661	0	MG-Inflam	Basp1
Ifngr1	0	0.842273	0.903	0.726	0	MG-Inflam	Ifngr1
Selenop	0	0.44509	0.967	0.791	0	MG-Inflam	Selenop
Otulinl1	0	0.838172	0.833	0.66	0	MG-Inflam	Otulinl
Cx3cr11	0	1.01067	0.982	0.81	0	MG-Inflam	Cx3cr1
Zfhx31	0	0.4012	0.871	0.7	0	MG-Inflam	Zfhx3
Itgb51	0	0.597496	0.94	0.769	0	MG-Inflam	Itgb5
Tgfbr11	0	0.541513	0.931	0.761	0	MG-Inflam	Tgfbr1
Abi31	0	0.796623	0.717	0.547	0	MG-Inflam	Abi3
Tcn2	0	0.891187	0.647	0.48	0	MG-Inflam	Tcn2
Sft2d1	0	0.895811	0.783	0.618	0	MG-Inflam	Sft2d1
Scamp2	0	0.76605	0.847	0.686	0	MG-Inflam	Scamp2
Cmtm6	0	0.956755	0.586	0.433	0	MG-Inflam	Cmtm6
Marcks	0	0.719416	0.922	0.771	0	MG-Inflam	Marcks
Ckb	0	0.610881	0.797	0.646	0	MG-Inflam	Ckb
Ywhah	0	0.773586	0.732	0.583	0	MG-Inflam	Ywhah
Cd53	0	0.779607	0.757	0.609	0	MG-Inflam	Cd53
Arhgap451	0	0.755155	0.743	0.595	0	MG-Inflam	Arhgap45
C1qa	0	0.453	0.999	0.852	0	MG-Inflam	C1qa
C1qc	0	0.533763	0.998	0.854	0	MG-Inflam	C1qc
Rgs10	0	0.643579	0.926	0.785	0	MG-Inflam	Rgs10
Abhd121	0	0.522086	0.87	0.734	0	MG-Inflam	Abhd12
C1qb	0	0.48049	0.999	0.871	0	MG-Inflam	C1qb
Mtdh1	0	0.693255	0.8	0.672	0	MG-Inflam	Mtdh
Rsrp11	0	0.714208	0.805	0.678	0	MG-Inflam	Rsrp1
Unc93b1	0	0.588183	0.966	0.846	0	MG-Inflam	Unc93b1
Sirpa1	0	0.547666	0.931	0.814	0	MG-Inflam	Sirpa

Lgmn	0	0.700904	0.996	0.885	0	MG-Inflam	Lgmn
Csf1r	0	0.950952	0.997	0.892	0	MG-Inflam	Csf1r
Ctsd	0	0.609427	0.998	0.895	0	MG-Inflam	Ctsd
Serinc3	0	0.898784	0.984	0.883	0	MG-Inflam	Serinc3
Laptm5	0	0.769016	0.983	0.884	0	MG-Inflam	Laptm5
Itm2b	0	0.464303	0.998	0.949	0	MG-Inflam	Itm2b
Hexb	0	0.946093	0.999	0.968	0	MG-Inflam	Hexb
Cst3	0	1.309744	1	0.985	0	MG-Inflam	Cst3
Calm2	2.62E-305	0.659988	0.768	0.653	6.41E-301	MG-Inflam	Calm2
Arhgap51	3.54E-304	0.681518	0.686	0.51	8.66E-300	MG-Inflam	Arhgap5
Tmem59	4.50E-300	0.675466	0.753	0.632	1.10E-295	MG-Inflam	Tmem59
Sox4	1.32E-296	1.26559	0.343	0.173	3.24E-292	MG-Inflam	Sox4
Ccr51	7.20E-292	0.73632	0.65	0.47	1.76E-287	MG-Inflam	Ccr5
Pld4	5.29E-289	0.527685	0.879	0.733	1.29E-284	MG-Inflam	Pld4
Saraf	6.49E-287	0.838469	0.624	0.482	1.59E-282	MG-Inflam	Saraf
Hsp90b1	9.48E-287	0.540195	0.851	0.723	2.32E-282	MG-Inflam	Hsp90b1
Qk1	1.86E-285	0.331309	0.898	0.792	4.56E-281	MG-Inflam	Qk
Cd331	1.04E-284	0.753913	0.669	0.525	2.54E-280	MG-Inflam	Cd33
Ctsf	2.20E-284	0.960255	0.537	0.37	5.38E-280	MG-Inflam	Ctsf
Itm2c	4.82E-281	0.585953	0.807	0.667	1.18E-276	MG-Inflam	Itm2c
Lag3	1.08E-280	0.5904	0.737	0.509	2.65E-276	MG-Inflam	Lag3
Apbb1ip1	3.50E-278	0.456234	0.84	0.733	8.56E-274	MG-Inflam	Apbb1ip
Cyth41	5.44E-278	0.609032	0.756	0.647	1.33E-273	MG-Inflam	Cyth4
Ctss	5.98E-278	0.35451	0.998	0.922	1.46E-273	MG-Inflam	Ctss
Mertk1	1.86E-277	0.343728	0.862	0.7	4.55E-273	MG-Inflam	Mertk
Itgam1	1.86E-275	0.612695	0.755	0.619	4.55E-271	MG-Inflam	Itgam
Arhgef40	6.74E-273	1.219087	0.318	0.157	1.65E-268	MG-Inflam	Arhgef40
Bin11	1.26E-269	0.533386	0.792	0.665	3.09E-265	MG-Inflam	Bin1
Ivns1abp1	1.41E-268	0.711943	0.615	0.455	3.46E-264	MG-Inflam	Ivns1abp
Ctsa	9.35E-268	0.454784	0.921	0.762	2.29E-263	MG-Inflam	Ctsa
Man2b1	1.74E-266	0.523876	0.85	0.712	4.27E-262	MG-Inflam	Man2b1
Syngr1	5.23E-266	0.658999	0.645	0.45	1.28E-261	MG-Inflam	Syngr1
Arsb1	2.99E-263	0.722915	0.588	0.417	7.32E-259	MG-Inflam	Arsb
Ly86	1.62E-262	0.336841	0.992	0.9	3.97E-258	MG-Inflam	Ly86
Rtn4rl11	3.81E-260	0.843864	0.493	0.32	9.33E-256	MG-Inflam	Rtn4rl1
Laptm4a	4.66E-259	0.518862	0.845	0.718	1.14E-254	MG-Inflam	Laptm4a
Rapgef51	3.79E-258	0.273621	0.756	0.557	9.28E-254	MG-Inflam	Rapgef5
Rnf13	1.33E-256	0.684688	0.689	0.566	3.27E-252	MG-Inflam	Rnf13
Jam2	2.18E-256	1.380787	0.255	0.115	5.33E-252	MG-Inflam	Jam2
Gng10	2.69E-255	0.587933	0.765	0.61	6.60E-251	MG-Inflam	Gng10
Comt	4.41E-255	0.710036	0.683	0.554	1.08E-250	MG-Inflam	Comt
Grn	3.61E-254	0.365808	0.976	0.834	8.84E-250	MG-Inflam	Grn
Tmem50a	4.54E-249	0.597412	0.761	0.651	1.11E-244	MG-Inflam	Tmem50a
Hpgds1	9.31E-249	0.542134	0.742	0.599	2.28E-244	MG-Inflam	Hpgds

Lair11	1.63E-247	0.463676	0.857	0.716	3.98E-243	MG-Inflam	Lair1
Frmd4a1	1.06E-241	0.313235	0.77	0.613	2.58E-237	MG-Inflam	Frmd4a
Lpar61	1.11E-241	1.095326	0.422	0.267	2.72E-237	MG-Inflam	Lpar6
Pag11	4.04E-241	0.315704	0.771	0.618	9.89E-237	MG-Inflam	Pag1
Pdia3	2.25E-237	0.507617	0.828	0.7	5.52E-233	MG-Inflam	Pdia3
Glul1	4.13E-237	0.426848	0.85	0.723	1.01E-232	MG-Inflam	Glul
Nrip11	1.24E-233	0.596937	0.639	0.49	3.04E-229	MG-Inflam	Nrip1
Ldhb	2.60E-231	0.611864	0.681	0.517	6.37E-227	MG-Inflam	Ldhb
Itga61	3.81E-231	0.725032	0.555	0.399	9.32E-227	MG-Inflam	Itga6
Abca91	9.69E-231	0.659071	0.605	0.45	2.37E-226	MG-Inflam	Abca9
Pla2g15	2.20E-230	0.785117	0.583	0.447	5.38E-226	MG-Inflam	Pla2g15
Sgk11	2.31E-230	0.712735	0.534	0.362	5.66E-226	MG-Inflam	Sgk1
Tspan71	2.32E-226	0.799408	0.524	0.371	5.67E-222	MG-Inflam	Tspan7
4933406I1	6.51E-226	0.444352	0.638	0.46	1.59E-221	MG-Inflam	4933406I18Rik
Gtf2h21	4.55E-225	0.780933	0.536	0.39	1.11E-220	MG-Inflam	Gtf2h2
Dip2b1	1.08E-224	0.46003	0.706	0.568	2.65E-220	MG-Inflam	Dip2b
Tmbim6	3.40E-224	0.452864	0.877	0.764	8.33E-220	MG-Inflam	Tmbim6
Entpd11	1.04E-214	0.450249	0.764	0.648	2.55E-210	MG-Inflam	Entpd1
Mafb	2.59E-211	0.411356	0.851	0.689	6.33E-207	MG-Inflam	Mafb
0610040J0	5.28E-210	0.591451	0.559	0.405	1.29E-205	MG-Inflam	0610040J01Rik
Cyfip11	1.79E-209	0.486599	0.782	0.684	4.38E-205	MG-Inflam	Cyfip1
Smap21	1.14E-206	0.481208	0.739	0.63	2.78E-202	MG-Inflam	Smap2
St3gal51	4.26E-202	0.789576	0.489	0.342	1.04E-197	MG-Inflam	St3gal5
E330020D1	9.60E-200	0.975757	0.38	0.236	2.35E-195	MG-Inflam	E330020D12Rik
Chd91	2.26E-196	0.364159	0.81	0.721	5.53E-192	MG-Inflam	Chd9
Ctc1	4.26E-196	0.953372	0.45	0.32	1.04E-191	MG-Inflam	Ctc1
Gns1	9.80E-196	0.476264	0.789	0.681	2.40E-191	MG-Inflam	Gns
Atp6v0b	1.77E-195	0.434313	0.863	0.774	4.33E-191	MG-Inflam	Atp6v0b
Zfp69	4.55E-192	1.079492	0.309	0.176	1.11E-187	MG-Inflam	Zfp69
P2ry6	5.90E-191	0.534487	0.748	0.639	1.44E-186	MG-Inflam	P2ry6
Adora3	2.29E-190	1.027414	0.392	0.258	5.60E-186	MG-Inflam	Adora3
Gpr1831	2.20E-189	0.795604	0.467	0.324	5.38E-185	MG-Inflam	Gpr183
Daglb	8.87E-188	0.64551	0.615	0.5	2.17E-183	MG-Inflam	Daglb
Camk1	1.99E-186	0.607494	0.661	0.545	4.89E-182	MG-Inflam	Camk1
Mef2c1	3.67E-186	0.277309	0.826	0.71	9.00E-182	MG-Inflam	Mef2c
Cxxc5	7.01E-184	0.869105	0.412	0.275	1.72E-179	MG-Inflam	Cxxc5
Rnaset2b	9.34E-183	0.86512	0.524	0.416	2.29E-178	MG-Inflam	Rnaset2b
Bin2	2.30E-180	0.55994	0.679	0.57	5.64E-176	MG-Inflam	Bin2
1700017B0	6.53E-179	0.854386	0.459	0.334	1.60E-174	MG-Inflam	1700017B05Rik
Ppcdc1	6.71E-179	0.756737	0.502	0.375	1.64E-174	MG-Inflam	Ppcdc
Bsg	3.27E-174	0.444998	0.76	0.666	8.01E-170	MG-Inflam	Bsg
Tgfb1	3.75E-172	0.531193	0.698	0.609	9.20E-168	MG-Inflam	Tgfb1
Tmed9	2.28E-171	0.716966	0.563	0.467	5.59E-167	MG-Inflam	Tmed9
Il10ra1	3.00E-170	0.432525	0.762	0.662	7.34E-166	MG-Inflam	Il10ra

Pros11	8.61E-168	0.625992	0.576	0.457	2.11E-163	MG-Inflam	Pros1
Lrba1	9.24E-168	0.871911	0.312	0.182	2.26E-163	MG-Inflam	Lrba
Edem2	4.81E-167	0.8676	0.469	0.362	1.18E-162	MG-Inflam	Edem2
8030442B0	4.46E-166	0.637993	0.316	0.18	1.09E-161	MG-Inflam	8030442B05Rik
Hexa	7.82E-166	0.398299	0.83	0.708	1.91E-161	MG-Inflam	Hexa
Clic1	2.89E-165	0.425171	0.852	0.744	7.08E-161	MG-Inflam	Clic1
Orai1	2.16E-164	0.711224	0.564	0.471	5.29E-160	MG-Inflam	Orai1
Fam102b1	5.08E-162	0.665922	0.444	0.31	1.25E-157	MG-Inflam	Fam102b
Adrb2	5.91E-161	0.679008	0.478	0.347	1.45E-156	MG-Inflam	Adrb2
Ppib	3.83E-160	0.477773	0.764	0.66	9.39E-156	MG-Inflam	Ppib
Lpcat31	3.31E-158	0.879947	0.428	0.314	8.11E-154	MG-Inflam	Lpcat3
A830008E2	1.54E-157	0.713403	0.394	0.26	3.77E-153	MG-Inflam	A830008E24Rik
Syngr2	3.37E-155	0.456993	0.656	0.556	8.26E-151	MG-Inflam	Syngr2
Tmem176b	5.44E-155	0.361503	0.763	0.606	1.33E-150	MG-Inflam	Tmem176b
Fgd21	1.34E-154	0.493395	0.663	0.564	3.29E-150	MG-Inflam	Fgd2
Rpn2	3.56E-152	0.623751	0.602	0.513	8.73E-148	MG-Inflam	Rpn2
Agmo1	5.19E-151	0.388361	0.498	0.353	1.27E-146	MG-Inflam	Agmo
Slc3a2	3.64E-150	0.574852	0.627	0.53	8.90E-146	MG-Inflam	Slc3a2
Fchsd21	7.86E-150	0.381265	0.622	0.5	1.92E-145	MG-Inflam	Fchsd2
Col27a11	1.22E-149	0.704121	0.35	0.221	2.99E-145	MG-Inflam	Col27a1
Gm26291	2.50E-148	0.393025	0.564	0.426	6.12E-144	MG-Inflam	Gm2629
Tjp11	2.07E-145	0.74404	0.376	0.251	5.06E-141	MG-Inflam	Tjp1
Csnk1e	2.25E-144	0.765834	0.472	0.369	5.51E-140	MG-Inflam	Csnk1e
Son1	8.46E-142	0.447986	0.709	0.641	2.07E-137	MG-Inflam	Son
Commd8	3.70E-140	0.872799	0.422	0.321	9.05E-136	MG-Inflam	Commd8
Sgce	3.01E-139	0.881469	0.352	0.238	7.36E-135	MG-Inflam	Sgce
Pip4p1	6.56E-139	0.684273	0.534	0.452	1.61E-134	MG-Inflam	Pip4p1
Rnf167	1.62E-138	0.971751	0.359	0.254	3.96E-134	MG-Inflam	Rnf167
Cmtm7	2.72E-138	0.528652	0.666	0.591	6.67E-134	MG-Inflam	Cmtm7
Snta11	2.17E-137	0.86456	0.361	0.248	5.32E-133	MG-Inflam	Snta1
Asah1	8.93E-136	0.449339	0.752	0.661	2.19E-131	MG-Inflam	Asah1
Rnf130	3.04E-135	0.443043	0.739	0.665	7.45E-131	MG-Inflam	Rnf130
Alox5ap	5.98E-135	0.271281	0.765	0.63	1.46E-130	MG-Inflam	Alox5ap
Gna151	1.50E-134	0.711844	0.466	0.365	3.68E-130	MG-Inflam	Gna15
Tpst2	1.37E-133	0.577968	0.584	0.505	3.36E-129	MG-Inflam	Tpst2
Tnfaip8l2	8.89E-133	0.684389	0.537	0.455	2.18E-128	MG-Inflam	Tnfaip8l2
Maf2	9.28E-133	0.476504	0.568	0.446	2.27E-128	MG-Inflam	Maf
Tmem86a	9.40E-133	0.607222	0.563	0.458	2.30E-128	MG-Inflam	Tmem86a
Plod1	2.10E-132	0.894314	0.393	0.291	5.15E-128	MG-Inflam	Plod1
Bbs91	2.74E-132	0.502161	0.376	0.248	6.70E-128	MG-Inflam	Bbs9
Cd300c2	6.86E-132	0.383753	0.82	0.713	1.68E-127	MG-Inflam	Cd300c2
Sec11c	8.45E-130	0.657941	0.548	0.47	2.07E-125	MG-Inflam	Sec11c
Rbm391	1.29E-129	0.367472	0.798	0.741	3.16E-125	MG-Inflam	Rbm39
Slc7a81	3.74E-129	0.49212	0.554	0.447	9.15E-125	MG-Inflam	Slc7a8

Eng	7.77E-129	1.003487	0.304	0.2	1.90E-124	MG-Inflam	Eng
Ddost	8.78E-129	0.616428	0.572	0.489	2.15E-124	MG-Inflam	Ddost
Slc46a1	6.60E-128	1.122317	0.294	0.195	1.62E-123	MG-Inflam	Slc46a1
Prkca1	1.60E-126	0.377911	0.446	0.317	3.93E-122	MG-Inflam	Prkca
Inka1	3.96E-126	1.133904	0.283	0.185	9.69E-122	MG-Inflam	Inka1
Mlxipl	1.33E-125	0.852862	0.3	0.192	3.27E-121	MG-Inflam	Mlxipl
Ndfip1	1.00E-124	0.499225	0.637	0.567	2.45E-120	MG-Inflam	Ndfip1
Rps6ka1	7.43E-124	0.595611	0.554	0.477	1.82E-119	MG-Inflam	Rps6ka1
Gpr155	4.10E-123	0.987518	0.263	0.161	1.01E-118	MG-Inflam	Gpr155
Whrn1	1.70E-122	0.607526	0.32	0.204	4.17E-118	MG-Inflam	Whrn
Plekho1	4.69E-122	0.515518	0.638	0.569	1.15E-117	MG-Inflam	Plekho1
Xist1	5.32E-122	0.44468	0.491	0.358	1.30E-117	MG-Inflam	Xist
Atraid	7.09E-122	0.755561	0.47	0.388	1.74E-117	MG-Inflam	Atraid
Cttnbp2nl1	2.97E-120	0.432345	0.579	0.481	7.28E-116	MG-Inflam	Cttnbp2nl
Snn	3.12E-120	0.98041	0.291	0.191	7.64E-116	MG-Inflam	Snn
Asph1	4.45E-120	0.479621	0.519	0.411	1.09E-115	MG-Inflam	Asph
Cyb561a3	1.39E-119	0.856985	0.383	0.286	3.40E-115	MG-Inflam	Cyb561a3
Dock101	6.96E-119	0.287528	0.692	0.596	1.70E-114	MG-Inflam	Dock10
Bmyc	1.16E-116	0.75831	0.455	0.37	2.83E-112	MG-Inflam	Bmyc
Slc29a31	2.11E-116	0.59693	0.536	0.451	5.17E-112	MG-Inflam	Slc29a3
Kctd121	9.15E-116	0.341766	0.699	0.605	2.24E-111	MG-Inflam	Kctd12
Soga11	3.11E-115	0.480251	0.478	0.368	7.62E-111	MG-Inflam	Soga1
Arl6ip1	8.97E-114	0.479956	0.661	0.594	2.20E-109	MG-Inflam	Arl6ip1
Rasgrp31	3.81E-113	0.437083	0.508	0.4	9.32E-109	MG-Inflam	Rasgrp3
Cd14	9.17E-113	0.30937	0.649	0.53	2.25E-108	MG-Inflam	Cd14
Ang	2.05E-112	0.858415	0.394	0.302	5.03E-108	MG-Inflam	Ang
Canx	9.71E-112	0.483817	0.647	0.584	2.38E-107	MG-Inflam	Canx
Havcr21	1.12E-111	0.660275	0.448	0.353	2.75E-107	MG-Inflam	Havcr2
Il6ra1	6.25E-111	0.489488	0.557	0.473	1.53E-106	MG-Inflam	Il6ra
Wasf21	9.59E-108	0.392119	0.665	0.61	2.35E-103	MG-Inflam	Wasf2
Erp29	1.12E-107	0.395665	0.758	0.681	2.74E-103	MG-Inflam	Erp29
Iffo11	4.25E-106	0.778837	0.327	0.228	1.04E-101	MG-Inflam	Iffo1
Pdia6	5.45E-106	0.544963	0.567	0.488	1.33E-101	MG-Inflam	Pdia6
Csf3r1	1.46E-105	0.337711	0.583	0.498	3.58E-101	MG-Inflam	Csf3r
Cd302	4.77E-104	0.787252	0.419	0.337	1.17E-99	MG-Inflam	Cd302
Cd34	2.68E-103	0.938443	0.284	0.19	6.57E-99	MG-Inflam	Cd34
Capn31	7.51E-102	0.542627	0.44	0.336	1.84E-97	MG-Inflam	Capn3
Mylip1	4.54E-101	0.414083	0.579	0.48	1.11E-96	MG-Inflam	Mylip
Adap21	6.61E-101	0.285017	0.648	0.562	1.62E-96	MG-Inflam	Adap2
Abhd6	7.72E-101	0.988564	0.297	0.21	1.89E-96	MG-Inflam	Abhd6
Sesn11	7.73E-101	0.685882	0.384	0.29	1.89E-96	MG-Inflam	Sesn1
P3h21	9.20E-100	0.311156	0.452	0.339	2.25E-95	MG-Inflam	P3h2
Pnp	1.91E-98	0.515379	0.547	0.471	4.67E-94	MG-Inflam	Pnp
Eef2k1	2.12E-98	0.695791	0.332	0.236	5.19E-94	MG-Inflam	Eef2k

Mfap3	1.27E-97	0.874836	0.327	0.241	3.10E-93	MG-Inflam	Mfap3
Lrrc3	1.59E-97	0.944974	0.259	0.171	3.89E-93	MG-Inflam	Lrrc3
Adam101	6.90E-96	0.544023	0.484	0.408	1.69E-91	MG-Inflam	Adam10
Adam15	2.60E-95	0.742571	0.431	0.358	6.38E-91	MG-Inflam	Adam15
Tpp1	2.90E-95	0.53233	0.543	0.462	7.11E-91	MG-Inflam	Tpp1
Tgfbr21	5.84E-94	0.293866	0.711	0.637	1.43E-89	MG-Inflam	Tgfbr2
Ntpcr	7.81E-94	0.511361	0.559	0.482	1.91E-89	MG-Inflam	Ntpcr
Stard31	8.28E-93	0.791886	0.38	0.303	2.03E-88	MG-Inflam	Stard3
Lrp11	1.04E-92	0.394021	0.664	0.585	2.54E-88	MG-Inflam	Lrp1
P4ha11	1.65E-92	0.638492	0.442	0.364	4.04E-88	MG-Inflam	P4ha1
Garnl31	1.54E-91	0.710682	0.275	0.183	3.77E-87	MG-Inflam	Garnl3
Asb2	3.07E-91	0.883958	0.281	0.195	7.53E-87	MG-Inflam	Asb2
Bank11	6.50E-90	0.54214	0.28	0.184	1.59E-85	MG-Inflam	Bank1
AC149090.	3.01E-89	0.518942	0.509	0.433	7.37E-85	MG-Inflam	AC149090.1
Ociad1	3.46E-88	0.607902	0.498	0.445	8.47E-84	MG-Inflam	Ociad1
B4galt4	1.26E-87	0.941769	0.273	0.191	3.08E-83	MG-Inflam	B4galt4
Il6st1	1.82E-87	0.58422	0.433	0.352	4.47E-83	MG-Inflam	Il6st
Anxa31	2.77E-87	0.35948	0.666	0.587	6.78E-83	MG-Inflam	Anxa3
Mgll	3.56E-87	0.682993	0.263	0.175	8.71E-83	MG-Inflam	Mgll
Plxnb21	4.33E-87	0.575591	0.494	0.429	1.06E-82	MG-Inflam	Plxnb2
Nptn	5.13E-86	0.45302	0.587	0.541	1.26E-81	MG-Inflam	Nptn
Ergic3	7.68E-86	0.580289	0.522	0.47	1.88E-81	MG-Inflam	Ergic3
Tacc11	1.14E-85	0.407946	0.551	0.485	2.78E-81	MG-Inflam	Tacc1
Lhfpl21	1.67E-85	0.369744	0.464	0.367	4.10E-81	MG-Inflam	Lhfpl2
Aup1	1.78E-85	0.514065	0.582	0.531	4.36E-81	MG-Inflam	Aup1
Rnaset2a	2.05E-85	0.79531	0.399	0.329	5.02E-81	MG-Inflam	Rnaset2a
Srsf71	4.91E-85	0.570739	0.488	0.426	1.20E-80	MG-Inflam	Srsf7
Fam91a11	9.52E-85	0.682344	0.361	0.278	2.33E-80	MG-Inflam	Fam91a1
Fmnl31	4.05E-84	0.443868	0.49	0.408	9.91E-80	MG-Inflam	Fmnl3
Rab3il11	4.39E-84	0.562328	0.48	0.411	1.08E-79	MG-Inflam	Rab3il1
Tram1	1.49E-81	0.684579	0.434	0.374	3.65E-77	MG-Inflam	Tram1
Tmem273	1.97E-81	0.787513	0.286	0.204	4.81E-77	MG-Inflam	Tmem273
Serinc1	8.30E-81	0.720354	0.406	0.343	2.03E-76	MG-Inflam	Serinc1
Snx181	1.80E-80	0.520352	0.482	0.413	4.40E-76	MG-Inflam	Snx18
Plod3	2.88E-80	0.723544	0.388	0.32	7.06E-76	MG-Inflam	Plod3
Rrbp11	5.31E-80	0.26964	0.823	0.766	1.30E-75	MG-Inflam	Rrbp1
Spcs2	5.42E-80	0.510456	0.55	0.497	1.33E-75	MG-Inflam	Spcs2
Tspan4	2.84E-79	0.891547	0.314	0.24	6.96E-75	MG-Inflam	Tspan4
Sort11	7.66E-79	0.629628	0.37	0.29	1.87E-74	MG-Inflam	Sort1
Adprh	4.58E-78	0.80613	0.361	0.297	1.12E-73	MG-Inflam	Adprh
Adrb1	9.96E-78	0.770204	0.255	0.173	2.44E-73	MG-Inflam	Adrb1
Ccng2	1.28E-77	0.924387	0.25	0.174	3.13E-73	MG-Inflam	Ccng2
Ypel3	2.55E-77	0.371656	0.633	0.573	6.24E-73	MG-Inflam	Ypel3
Tm9sf21	5.46E-77	0.580179	0.465	0.409	1.34E-72	MG-Inflam	Tm9sf2

Map111	8.21E-77	0.7064	0.396	0.329	2.01E-72	MG-Inflam	Map11
P4hb	2.60E-76	0.502072	0.537	0.484	6.36E-72	MG-Inflam	P4hb
Tmed10	7.08E-75	0.426219	0.606	0.563	1.73E-70	MG-Inflam	Tmed10
Mfng	8.85E-75	0.908971	0.284	0.212	2.17E-70	MG-Inflam	Mfng
Man1a21	5.41E-73	0.517771	0.442	0.373	1.32E-68	MG-Inflam	Man1a2
Rhobtb11	1.23E-72	0.539783	0.316	0.231	3.02E-68	MG-Inflam	Rhobtb1
Git21	3.01E-72	0.404794	0.535	0.478	7.38E-68	MG-Inflam	Git2
Cask1	4.11E-72	0.517669	0.381	0.299	1.01E-67	MG-Inflam	Cask
Selenos	4.97E-72	0.670348	0.425	0.372	1.22E-67	MG-Inflam	Selenos
Tmed7	1.67E-71	0.719154	0.38	0.32	4.10E-67	MG-Inflam	Tmed7
Rpn1	3.59E-71	0.687219	0.411	0.355	8.80E-67	MG-Inflam	Rpn1
Arf1	1.04E-70	0.420029	0.597	0.556	2.55E-66	MG-Inflam	Arf1
Scamp51	4.10E-70	0.690271	0.321	0.248	1.00E-65	MG-Inflam	Scamp5
Tmed5	6.37E-70	0.646751	0.426	0.374	1.56E-65	MG-Inflam	Tmed5
Itgb2	1.14E-69	0.363362	0.632	0.582	2.78E-65	MG-Inflam	Itgb2
Slc16a61	1.77E-69	0.495977	0.375	0.296	4.34E-65	MG-Inflam	Slc16a6
Slc35b2	9.06E-69	0.726728	0.383	0.327	2.22E-64	MG-Inflam	Slc35b2
Rab14	1.41E-68	0.425388	0.589	0.555	3.45E-64	MG-Inflam	Rab14
Arl101	2.95E-68	0.84182	0.301	0.235	7.22E-64	MG-Inflam	Arl10
Rabac1	7.97E-68	0.432749	0.56	0.519	1.95E-63	MG-Inflam	Rabac1
Atp6ap1	1.05E-67	0.474611	0.545	0.504	2.57E-63	MG-Inflam	Atp6ap1
Slc40a1	2.37E-67	0.372257	0.292	0.211	5.80E-63	MG-Inflam	Slc40a1
Cysltr1	7.51E-67	0.848248	0.25	0.179	1.84E-62	MG-Inflam	Cysltr1
Atp6v0a2	1.26E-66	0.658271	0.363	0.296	3.08E-62	MG-Inflam	Atp6v0a2
4632427E1	4.57E-66	0.783054	0.252	0.181	1.12E-61	MG-Inflam	4632427E13Rik
Tecr	6.36E-66	0.496295	0.511	0.47	1.56E-61	MG-Inflam	Tecr
Capza2	1.43E-65	0.310186	0.71	0.67	3.50E-61	MG-Inflam	Capza2
Itgb1	4.47E-65	0.513099	0.482	0.434	1.09E-60	MG-Inflam	Itgb1
Snrnp701	7.02E-65	0.402741	0.547	0.507	1.72E-60	MG-Inflam	Snrnp70
Phf141	1.42E-64	0.354608	0.46	0.391	3.47E-60	MG-Inflam	Phf14
Tspan141	3.14E-64	0.418801	0.462	0.398	7.68E-60	MG-Inflam	Tspan14
Tm6sf11	3.36E-64	0.503637	0.428	0.361	8.22E-60	MG-Inflam	Tm6sf1
Zfp706	3.98E-64	0.460845	0.537	0.499	9.75E-60	MG-Inflam	Zfp706
Tmem37	7.16E-64	0.737297	0.338	0.273	1.75E-59	MG-Inflam	Tmem37
Bcap31	8.71E-64	0.532579	0.483	0.444	2.13E-59	MG-Inflam	Bcap31
Card91	1.00E-63	0.776645	0.32	0.258	2.45E-59	MG-Inflam	Card9
Ints6l1	1.48E-63	0.439527	0.415	0.34	3.64E-59	MG-Inflam	Ints6l
Ifnar2	1.89E-63	0.338707	0.675	0.634	4.64E-59	MG-Inflam	Ifnar2
Atp2c11	1.91E-63	0.494138	0.446	0.388	4.68E-59	MG-Inflam	Atp2c1
Slc1a31	4.44E-63	0.360771	0.482	0.401	1.09E-58	MG-Inflam	Slc1a3
Mknk11	5.25E-63	0.509869	0.443	0.389	1.29E-58	MG-Inflam	Mknk1
Klf3	1.13E-62	0.465914	0.464	0.407	2.77E-58	MG-Inflam	Klf3
Fcgr1	1.45E-62	0.306171	0.654	0.573	3.54E-58	MG-Inflam	Fcgr1
Snx17	6.95E-62	0.689335	0.388	0.34	1.70E-57	MG-Inflam	Snx17

Abcc31	1.36E-61	0.596253	0.362	0.294	3.34E-57	MG-Inflam	Abcc3
Pycard	4.45E-61	0.347616	0.626	0.563	1.09E-56	MG-Inflam	Pycard
Pwwp2a1	5.73E-61	0.446978	0.335	0.259	1.40E-56	MG-Inflam	Pwwp2a
Ifi27	2.64E-60	0.61905	0.39	0.329	6.46E-56	MG-Inflam	Ifi27
Stambpl11	6.79E-60	0.679696	0.297	0.228	1.66E-55	MG-Inflam	Stambpl1
Blnk1	5.78E-59	0.304981	0.548	0.483	1.42E-54	MG-Inflam	Blnk
Nckap1l1	7.95E-59	0.400252	0.516	0.476	1.95E-54	MG-Inflam	Nckap1l
Mtus11	8.14E-59	0.389112	0.466	0.405	1.99E-54	MG-Inflam	Mtus1
Tspan3	1.31E-58	0.503869	0.441	0.389	3.20E-54	MG-Inflam	Tspan3
Rgmb1	5.24E-58	0.762565	0.305	0.244	1.28E-53	MG-Inflam	Rgmb
Slc44a2	1.01E-56	0.718251	0.275	0.211	2.48E-52	MG-Inflam	Slc44a2
Pald11	1.11E-56	0.482871	0.355	0.287	2.72E-52	MG-Inflam	Pald1
Tmem63a	4.12E-56	0.760781	0.284	0.222	1.01E-51	MG-Inflam	Tmem63a
Pou2f21	2.27E-55	0.282199	0.573	0.521	5.56E-51	MG-Inflam	Pou2f2
Hps41	3.69E-55	0.511026	0.403	0.345	9.04E-51	MG-Inflam	Hps4
Fermt3	5.12E-55	0.368298	0.593	0.565	1.25E-50	MG-Inflam	Fermt3
Slc50a1	1.08E-54	0.712082	0.365	0.321	2.64E-50	MG-Inflam	Slc50a1
Ptp4a3	1.08E-54	0.713543	0.333	0.28	2.65E-50	MG-Inflam	Ptp4a3
Tnfrsf1a	1.26E-54	0.497045	0.481	0.449	3.09E-50	MG-Inflam	Tnfrsf1a
Twf2	5.45E-54	0.537758	0.46	0.424	1.34E-49	MG-Inflam	Twf2
Naa351	8.59E-54	0.766477	0.303	0.247	2.10E-49	MG-Inflam	Naa35
Cd300a	1.02E-53	0.439291	0.487	0.434	2.51E-49	MG-Inflam	Cd300a
Ulk21	2.48E-53	0.392375	0.427	0.363	6.07E-49	MG-Inflam	Ulk2
Retreg11	5.07E-53	0.262435	0.503	0.447	1.24E-48	MG-Inflam	Retreg1
Reep5	5.50E-53	0.355196	0.607	0.575	1.35E-48	MG-Inflam	Reep5
Slc29a1	6.05E-53	0.719024	0.325	0.272	1.48E-48	MG-Inflam	Slc29a1
Mgat11	1.23E-52	0.676247	0.327	0.272	3.01E-48	MG-Inflam	Mgat1
Pkig1	1.25E-52	0.376942	0.54	0.506	3.07E-48	MG-Inflam	Pkig
Sec62	1.92E-52	0.615656	0.424	0.388	4.70E-48	MG-Inflam	Sec62
Selenof	4.83E-52	0.303788	0.665	0.62	1.18E-47	MG-Inflam	Selenof
Lamp2	1.88E-51	0.271659	0.693	0.649	4.61E-47	MG-Inflam	Lamp2
Acap21	8.27E-51	0.320411	0.526	0.485	2.02E-46	MG-Inflam	Acap2
Rtn4	4.83E-50	0.335879	0.605	0.577	1.18E-45	MG-Inflam	Rtn4
Gm265421	1.57E-49	0.486412	0.282	0.216	3.84E-45	MG-Inflam	Gm26542
St6galnac4	2.17E-49	0.668251	0.347	0.299	5.31E-45	MG-Inflam	St6galnac4
Gcnt1	5.39E-49	0.673781	0.257	0.197	1.32E-44	MG-Inflam	Gcnt1
Ppfia4	6.18E-49	0.358371	0.544	0.489	1.51E-44	MG-Inflam	Ppfia4
Arrb2	7.07E-49	0.358772	0.555	0.527	1.73E-44	MG-Inflam	Arrb2
Tubgcp51	1.16E-48	0.501674	0.351	0.291	2.85E-44	MG-Inflam	Tubgcp5
Manf	1.77E-48	0.475889	0.444	0.4	4.32E-44	MG-Inflam	Manf
Slc12a91	9.96E-48	0.56866	0.381	0.334	2.44E-43	MG-Inflam	Slc12a9
Sipa11	4.22E-47	0.529359	0.382	0.336	1.03E-42	MG-Inflam	Sipa1
Pten1	6.71E-47	0.393644	0.478	0.441	1.64E-42	MG-Inflam	Pten
Tmem30a	2.97E-46	0.577607	0.407	0.373	7.28E-42	MG-Inflam	Tmem30a

Mpc1	7.42E-46	0.399548	0.508	0.48	1.82E-41	MG-Inflam	Mpc1
Zfp6521	9.50E-46	0.32194	0.447	0.393	2.33E-41	MG-Inflam	Zfp652
Tspan13	2.04E-45	0.402266	0.417	0.359	4.99E-41	MG-Inflam	Tspan13
H2-M3	5.99E-45	0.56098	0.44	0.414	1.47E-40	MG-Inflam	H2-M3
Spcs1	8.39E-45	0.405737	0.532	0.506	2.06E-40	MG-Inflam	Spcs1
Herc21	1.97E-44	0.307967	0.473	0.419	4.83E-40	MG-Inflam	Herc2
Nucb1	1.98E-44	0.555373	0.412	0.378	4.84E-40	MG-Inflam	Nucb1
Cebpg1	2.12E-44	0.60565	0.368	0.328	5.19E-40	MG-Inflam	Cebpg
Vkorc1	5.25E-44	0.489542	0.451	0.42	1.29E-39	MG-Inflam	Vkorc1
Ncstn	7.55E-44	0.521497	0.434	0.404	1.85E-39	MG-Inflam	Ncstn
Papss1	2.16E-43	0.67664	0.283	0.232	5.29E-39	MG-Inflam	Papss1
Il16	4.84E-43	0.659119	0.309	0.261	1.19E-38	MG-Inflam	Il16
Txndc15	7.53E-43	0.680535	0.328	0.287	1.84E-38	MG-Inflam	Txndc15
Parvg	1.07E-42	0.680301	0.3	0.251	2.63E-38	MG-Inflam	Parvg
Ptpra1	1.10E-42	0.322811	0.524	0.491	2.69E-38	MG-Inflam	Ptpra
Evl1	1.17E-42	0.311819	0.516	0.484	2.87E-38	MG-Inflam	Evl
Tnfrsf211	3.81E-42	0.497766	0.377	0.332	9.33E-38	MG-Inflam	Tnfrsf21
Rtn11	1.70E-41	0.266953	0.301	0.236	4.16E-37	MG-Inflam	Rtn1
Mlec	3.37E-41	0.617613	0.337	0.295	8.25E-37	MG-Inflam	Mlec
Taz1	1.91E-40	0.703478	0.287	0.242	4.68E-36	MG-Inflam	Taz
Cul11	1.64E-39	0.503667	0.354	0.311	4.02E-35	MG-Inflam	Cul1
Tsc22d4	2.91E-39	0.449122	0.442	0.417	7.12E-35	MG-Inflam	Tsc22d4
Acp2	4.56E-39	0.554732	0.38	0.346	1.12E-34	MG-Inflam	Acp2
Pttg1ip	4.69E-39	0.701208	0.309	0.27	1.15E-34	MG-Inflam	Pttg1ip
Rnasek	6.24E-39	0.336494	0.576	0.553	1.53E-34	MG-Inflam	Rnasek
Rasal31	1.95E-38	0.616132	0.301	0.255	4.76E-34	MG-Inflam	Rasal3
Cnpy3	5.63E-38	0.663984	0.331	0.295	1.38E-33	MG-Inflam	Cnpy3
Emc3	9.37E-38	0.744337	0.277	0.236	2.30E-33	MG-Inflam	Emc3
Tmco1	1.21E-37	0.663634	0.344	0.312	2.97E-33	MG-Inflam	Tmco1
Cldnd1	1.71E-37	0.748969	0.261	0.218	4.18E-33	MG-Inflam	Cldnd1
Calr	4.08E-37	0.330327	0.526	0.489	9.99E-33	MG-Inflam	Calr
Cd82	1.40E-36	0.508681	0.404	0.376	3.44E-32	MG-Inflam	Cd82
Tuba1a	1.92E-36	0.412688	0.297	0.245	4.69E-32	MG-Inflam	Tuba1a
Vav11	2.83E-36	0.324759	0.513	0.498	6.92E-32	MG-Inflam	Vav1
Dad1	5.85E-35	0.324727	0.551	0.533	1.43E-30	MG-Inflam	Dad1
Rogdi	9.77E-35	0.460238	0.403	0.37	2.39E-30	MG-Inflam	Rogdi
Kcnk61	1.95E-34	0.567693	0.297	0.253	4.78E-30	MG-Inflam	Kcnk6
Rtn31	2.77E-34	0.285071	0.506	0.487	6.79E-30	MG-Inflam	Rtn3
Tmed2	2.82E-34	0.419303	0.482	0.467	6.91E-30	MG-Inflam	Tmed2
Pdgfb1	1.21E-33	0.370018	0.315	0.266	2.97E-29	MG-Inflam	Pdgfb
Spint1	4.12E-33	0.450501	0.368	0.323	1.01E-28	MG-Inflam	Spint1
B230219D2	1.42E-32	0.583834	0.328	0.295	3.47E-28	MG-Inflam	B230219D22Rik
Ttc14	1.55E-32	0.572811	0.312	0.273	3.80E-28	MG-Inflam	Ttc14
Hnrnph31	3.37E-32	0.573231	0.297	0.257	8.25E-28	MG-Inflam	Hnrnph3

Lman1	3.51E-32	0.746959	0.277	0.242	8.60E-28	MG-Inflam	Lman1
Dtx4	3.90E-32	0.586239	0.25	0.204	9.54E-28	MG-Inflam	Dtx4
Hnrnpk	4.61E-32	0.299899	0.563	0.552	1.13E-27	MG-Inflam	Hnrnpk
Pdia4	5.80E-32	0.561968	0.351	0.32	1.42E-27	MG-Inflam	Pdia4
Pcbp1	5.87E-32	0.304298	0.546	0.527	1.44E-27	MG-Inflam	Pcbp1
Tm9sf3	1.70E-31	0.472686	0.401	0.38	4.17E-27	MG-Inflam	Tm9sf3
Emc7	1.85E-31	0.538137	0.372	0.352	4.54E-27	MG-Inflam	Emc7
Cryl11	1.89E-31	0.464384	0.302	0.259	4.63E-27	MG-Inflam	Cryl1
Ndfip21	2.58E-31	0.610751	0.297	0.26	6.31E-27	MG-Inflam	Ndfip2
Cnpy2	2.77E-31	0.603009	0.334	0.308	6.78E-27	MG-Inflam	Cnpy2
Cnot8	3.09E-31	0.67813	0.291	0.258	7.58E-27	MG-Inflam	Cnot8
Hsd17b11	4.47E-31	0.678561	0.298	0.265	1.10E-26	MG-Inflam	Hsd17b11
Dnm21	2.44E-30	0.27129	0.525	0.512	5.98E-26	MG-Inflam	Dnm2
Pon31	2.86E-30	0.619887	0.293	0.256	7.00E-26	MG-Inflam	Pon3
Atp6ap2	3.42E-30	0.356656	0.494	0.478	8.39E-26	MG-Inflam	Atp6ap2
Ppt1	3.75E-30	0.342192	0.47	0.45	9.18E-26	MG-Inflam	Ppt1
Rhoh1	3.76E-30	0.266792	0.448	0.412	9.21E-26	MG-Inflam	Rhoh
Arglu11	8.66E-30	0.391514	0.388	0.358	2.12E-25	MG-Inflam	Arglu1
Arpc4	1.11E-29	0.26225	0.618	0.599	2.71E-25	MG-Inflam	Arpc4
2610203C2	1.23E-29	0.324626	0.257	0.206	3.01E-25	MG-Inflam	2610203C22Rik
Hnrnph11	1.56E-29	0.420423	0.351	0.315	3.82E-25	MG-Inflam	Hnrnph1
Herpud1	1.68E-29	0.388628	0.414	0.383	4.11E-25	MG-Inflam	Herpud1
Xbp1	5.03E-29	0.508553	0.397	0.378	1.23E-24	MG-Inflam	Xbp1
Fkbp2	6.23E-29	0.449282	0.424	0.406	1.52E-24	MG-Inflam	Fkbp2
Zeb2os	2.44E-28	0.477972	0.377	0.351	5.97E-24	MG-Inflam	Zeb2os
Sema4d	3.12E-28	0.357328	0.458	0.441	7.63E-24	MG-Inflam	Sema4d
Idh2	3.54E-28	0.545821	0.325	0.296	8.66E-24	MG-Inflam	Idh2
Dapp11	5.76E-28	0.349468	0.391	0.358	1.41E-23	MG-Inflam	Dapp1
Lpin21	1.16E-27	0.284799	0.458	0.431	2.84E-23	MG-Inflam	Lpin2
Ndufc2	1.62E-27	0.305873	0.516	0.506	3.96E-23	MG-Inflam	Ndufc2
Cebpd	4.21E-27	0.376805	0.319	0.277	1.03E-22	MG-Inflam	Cebpd
Selenok	9.68E-27	0.250328	0.595	0.59	2.37E-22	MG-Inflam	Selenok
Scarb21	1.37E-26	0.294145	0.517	0.501	3.36E-22	MG-Inflam	Scarb2
Arl8b1	1.81E-26	0.500946	0.343	0.319	4.43E-22	MG-Inflam	Arl8b
Ighm	1.92E-26	0.416004	0.401	0.376	4.69E-22	MG-Inflam	Ighm
Nagpa	5.78E-26	0.706805	0.289	0.264	1.41E-21	MG-Inflam	Nagpa
Emc10	6.84E-26	0.553617	0.358	0.343	1.67E-21	MG-Inflam	Emc10
Rp21	1.67E-25	0.507188	0.325	0.297	4.10E-21	MG-Inflam	Rp2
Tm2d2	1.92E-25	0.579962	0.329	0.31	4.71E-21	MG-Inflam	Tm2d2
Gm50861	2.18E-25	0.354554	0.289	0.246	5.34E-21	MG-Inflam	Gm5086
Iscu	1.18E-24	0.30877	0.478	0.471	2.89E-20	MG-Inflam	Iscu
Psenen	1.35E-24	0.329824	0.506	0.505	3.30E-20	MG-Inflam	Psenen
2-Mar	1.83E-24	0.573239	0.324	0.306	4.49E-20	MG-Inflam	2-Mar
Ldb11	3.07E-24	0.517693	0.328	0.305	7.51E-20	MG-Inflam	Ldb1

Itgav1	3.78E-24	0.353419	0.349	0.315	9.25E-20	MG-Inflam	Itgav
Slc46a3	5.96E-24	0.551891	0.263	0.229	1.46E-19	MG-Inflam	Slc46a3
Nars	8.04E-24	0.507825	0.353	0.336	1.97E-19	MG-Inflam	Nars
Il13ra11	1.12E-23	0.527909	0.304	0.276	2.75E-19	MG-Inflam	Il13ra1
Yipf4	3.15E-23	0.671204	0.259	0.233	7.71E-19	MG-Inflam	Yipf4
Srsf9	4.02E-23	0.351602	0.478	0.475	9.85E-19	MG-Inflam	Srsf9
Fez21	4.74E-23	0.501881	0.306	0.278	1.16E-18	MG-Inflam	Fez2
Zfp90	1.07E-22	0.489245	0.271	0.238	2.61E-18	MG-Inflam	Zfp90
Ostc	1.38E-22	0.528995	0.348	0.334	3.38E-18	MG-Inflam	Ostc
Kif21b1	3.09E-22	0.335907	0.32	0.285	7.57E-18	MG-Inflam	Kif21b
Sar1b	4.40E-22	0.591895	0.293	0.272	1.08E-17	MG-Inflam	Sar1b
Akr1b10	4.81E-22	0.665163	0.26	0.232	1.18E-17	MG-Inflam	Akr1b10
Clptm1	6.47E-22	0.52548	0.339	0.323	1.58E-17	MG-Inflam	Clptm1
Stt3a	7.32E-22	0.581485	0.297	0.276	1.79E-17	MG-Inflam	Stt3a
Lmo2	8.69E-22	0.330313	0.464	0.458	2.13E-17	MG-Inflam	Lmo2
Ddrgk1	1.76E-21	0.455851	0.406	0.401	4.30E-17	MG-Inflam	Ddrgk1
Krtcap2	1.82E-21	0.385161	0.448	0.444	4.46E-17	MG-Inflam	Krtcap2
Cnbp	2.32E-21	0.254272	0.526	0.523	5.69E-17	MG-Inflam	Cnbp
Sppl31	3.03E-21	0.316482	0.375	0.353	7.43E-17	MG-Inflam	Sppl3
Gtpbp21	4.20E-21	0.530426	0.295	0.269	1.03E-16	MG-Inflam	Gtpbp2
Npc11	7.05E-21	0.579687	0.274	0.248	1.73E-16	MG-Inflam	Npc1
Leprot	8.02E-21	0.479485	0.351	0.341	1.96E-16	MG-Inflam	Leprot
Sypl	1.50E-20	0.530237	0.31	0.292	3.66E-16	MG-Inflam	Sypl
Nfam11	1.92E-20	0.371193	0.37	0.351	4.69E-16	MG-Inflam	Nfam1
Arhgap4	3.85E-20	0.486716	0.293	0.267	9.43E-16	MG-Inflam	Arhgap4
Gmip	3.87E-20	0.38543	0.395	0.383	9.48E-16	MG-Inflam	Gmip
Glmp	4.16E-20	0.304663	0.481	0.469	1.02E-15	MG-Inflam	Glmp
Zdhhc201	4.78E-20	0.405824	0.346	0.325	1.17E-15	MG-Inflam	Zdhhc20
Lpxn1	7.19E-20	0.495128	0.285	0.259	1.76E-15	MG-Inflam	Lpxn
Gspt11	7.75E-20	0.457782	0.335	0.317	1.90E-15	MG-Inflam	Gspt1
Arhgap121	9.76E-20	0.288716	0.297	0.263	2.39E-15	MG-Inflam	Arhgap12
Adam171	1.23E-19	0.38426	0.385	0.375	3.01E-15	MG-Inflam	Adam17
M6pr	1.49E-19	0.323878	0.469	0.466	3.64E-15	MG-Inflam	M6pr
Tra2a1	1.70E-19	0.342008	0.405	0.394	4.15E-15	MG-Inflam	Tra2a
Fyco1	1.96E-19	0.50798	0.254	0.224	4.81E-15	MG-Inflam	Fyco1
Tmem33	1.98E-19	0.577136	0.294	0.276	4.85E-15	MG-Inflam	Tmem33
Il21r1	4.77E-19	0.346501	0.392	0.377	1.17E-14	MG-Inflam	Il21r
Supt20	6.23E-19	0.531616	0.254	0.227	1.53E-14	MG-Inflam	Supt20
Smox1	1.04E-18	0.442193	0.306	0.283	2.54E-14	MG-Inflam	Smox
Sik21	1.62E-18	0.332202	0.255	0.221	3.96E-14	MG-Inflam	Sik2
Tmem109	1.81E-18	0.634285	0.261	0.24	4.44E-14	MG-Inflam	Tmem109
Rubcnl1	6.27E-18	0.386293	0.268	0.238	1.54E-13	MG-Inflam	Rubcnl
Ccni	1.38E-17	0.327073	0.425	0.42	3.38E-13	MG-Inflam	Ccni
Arl1	1.51E-17	0.59336	0.26	0.241	3.70E-13	MG-Inflam	Arl1

Elk31	2.10E-17	0.298949	0.359	0.341	5.15E-13	MG-Inflam	Elk3
Gusb	4.55E-17	0.266464	0.508	0.5	1.11E-12	MG-Inflam	Gusb
Tmed3	5.08E-17	0.404415	0.393	0.39	1.24E-12	MG-Inflam	Tmed3
Lrp10	5.82E-17	0.516468	0.316	0.306	1.43E-12	MG-Inflam	Lrp10
Pon2	6.53E-17	0.467621	0.327	0.316	1.60E-12	MG-Inflam	Pon2
Srpr	7.93E-17	0.459816	0.345	0.338	1.94E-12	MG-Inflam	Srpr
Rassf21	8.31E-17	0.26479	0.342	0.318	2.04E-12	MG-Inflam	Rassf2
Man2b2	1.19E-16	0.506239	0.313	0.3	2.92E-12	MG-Inflam	Man2b2
Fbrsl11	1.20E-16	0.346092	0.356	0.341	2.93E-12	MG-Inflam	Fbrsl1
Os9	1.23E-16	0.356995	0.43	0.431	3.01E-12	MG-Inflam	Os9
Sla1	3.55E-16	0.301482	0.36	0.342	8.69E-12	MG-Inflam	Sla
Slc35c2	3.86E-16	0.551742	0.288	0.274	9.46E-12	MG-Inflam	Slc35c2
Aim21	4.43E-16	0.276712	0.384	0.369	1.09E-11	MG-Inflam	Aim2
Sun2	4.45E-16	0.486907	0.279	0.259	1.09E-11	MG-Inflam	Sun2
Samsn11	5.36E-16	0.277717	0.272	0.243	1.31E-11	MG-Inflam	Samsn1
Rgs19	1.23E-15	0.442007	0.343	0.337	3.01E-11	MG-Inflam	Rgs19
Abl11	1.35E-15	0.257255	0.282	0.253	3.30E-11	MG-Inflam	Abl1
Vapa	2.17E-15	0.277334	0.453	0.461	5.32E-11	MG-Inflam	Vapa
Edem11	2.42E-15	0.387139	0.339	0.328	5.94E-11	MG-Inflam	Edem1
Plxdc11	3.17E-15	0.37489	0.262	0.237	7.76E-11	MG-Inflam	Plxdc1
Cebpzos	3.93E-15	0.473014	0.333	0.324	9.62E-11	MG-Inflam	Cebpzos
Tlr71	5.08E-15	0.280664	0.369	0.352	1.25E-10	MG-Inflam	Tlr7
Cebpz1	8.96E-15	0.524168	0.303	0.291	2.19E-10	MG-Inflam	Cebpz
Naglu	9.90E-15	0.482587	0.313	0.3	2.43E-10	MG-Inflam	Naglu
Hfe	1.07E-14	0.503287	0.28	0.263	2.63E-10	MG-Inflam	Hfe
Srsf2	1.20E-14	0.284198	0.447	0.451	2.93E-10	MG-Inflam	Srsf2
Gdi11	1.61E-14	0.490733	0.261	0.242	3.95E-10	MG-Inflam	Gdi1
Akap8l1	2.53E-14	0.345344	0.329	0.313	6.21E-10	MG-Inflam	Akap8l
Derl1	3.28E-14	0.423004	0.361	0.361	8.03E-10	MG-Inflam	Derl1
Heatr5a1	3.63E-14	0.277023	0.355	0.34	8.89E-10	MG-Inflam	Heatr5a
Slc12a21	4.31E-14	0.296047	0.296	0.27	1.05E-09	MG-Inflam	Slc12a2
Itch1	4.36E-14	0.271715	0.352	0.337	1.07E-09	MG-Inflam	Itch
Clec4a3	7.01E-14	0.388238	0.306	0.287	1.72E-09	MG-Inflam	Clec4a3
Mcfd2	7.89E-14	0.499623	0.292	0.281	1.93E-09	MG-Inflam	Mcfd2
Ssr2	9.00E-14	0.457121	0.336	0.333	2.20E-09	MG-Inflam	Ssr2
Clec4a2	1.14E-13	0.439777	0.279	0.258	2.79E-09	MG-Inflam	Clec4a2
Washc41	1.34E-13	0.409309	0.286	0.268	3.28E-09	MG-Inflam	Washc4
Tmem14c	1.66E-13	0.289595	0.438	0.445	4.07E-09	MG-Inflam	Tmem14c
Slc39a7	2.11E-13	0.546875	0.25	0.235	5.16E-09	MG-Inflam	Slc39a7
Ptpre1	2.96E-13	0.313488	0.353	0.344	7.26E-09	MG-Inflam	Ptpre
Gtf2i1	3.23E-13	0.340164	0.269	0.247	7.91E-09	MG-Inflam	Gtf2i
Tm2d1	4.41E-13	0.526847	0.287	0.28	1.08E-08	MG-Inflam	Tm2d1
Tnfrsf1b1	4.73E-13	0.264854	0.364	0.355	1.16E-08	MG-Inflam	Tnfrsf1b
Sppl2a	6.78E-13	0.33975	0.387	0.39	1.66E-08	MG-Inflam	Sppl2a

Fam173a	9.34E-13	0.445834	0.34	0.341	2.29E-08	MG-Inflam	Fam173a
Ebp	1.31E-12	0.583463	0.268	0.26	3.21E-08	MG-Inflam	Ebp
Pik3cg1	1.37E-12	0.306349	0.307	0.29	3.37E-08	MG-Inflam	Pik3cg
Leng81	1.84E-12	0.372391	0.286	0.27	4.50E-08	MG-Inflam	Leng8
Wdr441	1.88E-12	0.283563	0.252	0.228	4.60E-08	MG-Inflam	Wdr44
Il10rb	2.17E-12	0.272694	0.458	0.463	5.32E-08	MG-Inflam	Il10rb
Numa11	3.06E-12	0.420235	0.298	0.287	7.49E-08	MG-Inflam	Numa1
Unc50	4.55E-12	0.536388	0.28	0.274	1.11E-07	MG-Inflam	Unc50
Mtch1	5.40E-12	0.380542	0.345	0.345	1.32E-07	MG-Inflam	Mtch1
Pnisr1	8.57E-12	0.296408	0.336	0.328	2.10E-07	MG-Inflam	Pnisr
Cers2	1.13E-11	0.508258	0.296	0.293	2.76E-07	MG-Inflam	Cers2
Tmem208	1.14E-11	0.524224	0.297	0.296	2.79E-07	MG-Inflam	Tmem208
Plgrkt	1.41E-11	0.350215	0.344	0.341	3.46E-07	MG-Inflam	Plgrkt
Wls1	1.49E-11	0.336294	0.326	0.319	3.65E-07	MG-Inflam	Wls
Cklf	1.93E-11	0.550878	0.26	0.25	4.72E-07	MG-Inflam	Cklf
Prcp1	2.32E-11	0.384887	0.365	0.37	5.67E-07	MG-Inflam	Prcp
Ube2j11	2.72E-11	0.421497	0.308	0.3	6.65E-07	MG-Inflam	Ube2j1
Tspan31	3.34E-11	0.538672	0.294	0.293	8.18E-07	MG-Inflam	Tspan31
Ccdc12	3.36E-11	0.273505	0.446	0.457	8.22E-07	MG-Inflam	Ccdc12
Cr1l1	9.54E-11	0.493023	0.255	0.245	2.34E-06	MG-Inflam	Cr1l
Ppp1r14b	1.11E-10	0.330257	0.373	0.375	2.73E-06	MG-Inflam	Ppp1r14b
Rnf7	1.56E-10	0.359174	0.369	0.379	3.81E-06	MG-Inflam	Rnf7
Sdf2l1	1.90E-10	0.253135	0.407	0.402	4.64E-06	MG-Inflam	Sdf2l1
Lyl1	4.90E-10	0.441488	0.252	0.24	1.20E-05	MG-Inflam	Lyl1
Rer1	4.94E-10	0.342592	0.402	0.419	1.21E-05	MG-Inflam	Rer1
Dnajc3	5.05E-10	0.313368	0.383	0.39	1.24E-05	MG-Inflam	Dnajc3
Tmem243	7.93E-10	0.315488	0.377	0.384	1.94E-05	MG-Inflam	Tmem243
Zmynd111	1.54E-09	0.274058	0.295	0.284	3.78E-05	MG-Inflam	Zmynd11
Tifab	1.85E-09	0.410206	0.31	0.307	4.54E-05	MG-Inflam	Tifab
Tlr13	2.21E-09	0.459351	0.25	0.239	5.40E-05	MG-Inflam	Tlr13
Naxe	2.31E-09	0.483977	0.294	0.296	5.66E-05	MG-Inflam	Naxe
Sec14l1	2.68E-09	0.31957	0.313	0.306	6.56E-05	MG-Inflam	Sec14l1
1810026B0	3.38E-09	0.378164	0.278	0.268	8.29E-05	MG-Inflam	1810026B05Rik
Pdlim4	4.14E-09	0.449126	0.27	0.264	0.000101	MG-Inflam	Pdlim4
Higd2a	4.99E-09	0.301638	0.406	0.419	0.000122	MG-Inflam	Higd2a
Arl6ip5	5.59E-09	0.353178	0.371	0.384	0.000137	MG-Inflam	Arl6ip5
Kpnb1	6.40E-09	0.465173	0.257	0.251	0.000157	MG-Inflam	Kpnb1
Dram2	1.05E-08	0.326633	0.365	0.375	0.000256	MG-Inflam	Dram2
Casp8	1.19E-08	0.287216	0.338	0.338	0.00029	MG-Inflam	Casp8
Vgll41	2.41E-08	0.273575	0.295	0.286	0.00059	MG-Inflam	Vgll4
Hmox2	3.57E-08	0.289419	0.407	0.426	0.000875	MG-Inflam	Hmox2
Alg5	4.90E-08	0.527661	0.256	0.254	0.0012	MG-Inflam	Alg5
Itfg11	4.92E-08	0.393928	0.265	0.259	0.001204	MG-Inflam	Itfg1
Pura	5.19E-08	0.341912	0.333	0.338	0.00127	MG-Inflam	Pura

A630001G2	8.33E-08	0.251987	0.275	0.264	0.002039	MG-Inflam	A630001G21Rik
Atxn7l3b	1.75E-07	0.443099	0.302	0.307	0.00428	MG-Inflam	Atxn7l3b
Tmbim4	2.11E-07	0.279455	0.42	0.442	0.005171	MG-Inflam	Tmbim4
Cryba4	2.25E-07	0.362473	0.281	0.272	0.005509	MG-Inflam	Cryba4
Sec11a	2.95E-07	0.4468	0.295	0.303	0.007225	MG-Inflam	Sec11a
Rhoq1	6.50E-07	0.281403	0.262	0.254	0.015912	MG-Inflam	Rhoq
Senp21	9.68E-07	0.257979	0.333	0.335	0.023699	MG-Inflam	Senp2
Fuca1	1.07E-06	0.255015	0.42	0.437	0.026086	MG-Inflam	Fuca1
Yif1b	1.36E-06	0.491838	0.271	0.277	0.0332	MG-Inflam	Yif1b
Rbm51	1.78E-06	0.276515	0.313	0.314	0.043564	MG-Inflam	Rbm5
Hvcn1	1.93E-06	0.374234	0.305	0.31	0.047246	MG-Inflam	Hvcn1
Dnase2a	2.49E-06	0.314118	0.377	0.392	0.060908	MG-Inflam	Dnase2a
Wdr331	4.03E-06	0.255307	0.318	0.32	0.098582	MG-Inflam	Wdr33
Dcakd	5.28E-06	0.311298	0.258	0.253	0.129241	MG-Inflam	Dcakd
Cirbp	6.56E-06	0.34519	0.29	0.291	0.160538	MG-Inflam	Cirbp
Ythdf21	2.28E-05	0.417729	0.25	0.251	0.557452	MG-Inflam	Ythdf2
Tmem9b	2.37E-05	0.424078	0.285	0.295	0.580985	MG-Inflam	Tmem9b
Tgs11	3.70E-05	0.393862	0.279	0.284	0.906287	MG-Inflam	Tgs1
Naga	6.00E-05	0.354253	0.31	0.321	1	MG-Inflam	Naga
Kdelr1	6.12E-05	0.366926	0.329	0.349	1	MG-Inflam	Kdelr1
Maf1	8.54E-05	0.425573	0.254	0.259	1	MG-Inflam	Maf1
Csk	0.000127	0.278498	0.344	0.363	1	MG-Inflam	Csk
0610010K1	0.000199	0.474159	0.255	0.262	1	MG-Inflam	0610010K14Rik
Ciao2b	0.000209	0.257564	0.208	0.253	1	MG-Inflam	Ciao2b
Sec13	0.000231	0.419935	0.276	0.289	1	MG-Inflam	Sec13
Creb3	0.000242	0.480487	0.248	0.256	1	MG-Inflam	Creb3
Map2k11	0.000297	0.259152	0.281	0.287	1	MG-Inflam	Map2k1
Reep3	0.000349	0.273878	0.363	0.385	1	MG-Inflam	Reep3
Rsrc21	0.000532	0.298742	0.283	0.289	1	MG-Inflam	Rsrc2
Mydgf	0.000537	0.435985	0.26	0.271	1	MG-Inflam	Mydgf
Ssbp4	0.00058	0.300527	0.311	0.326	1	MG-Inflam	Ssbp4
Ltbr	0.000619	0.471031	0.243	0.252	1	MG-Inflam	Ltbr
Scaf111	0.000713	0.267457	0.335	0.351	1	MG-Inflam	Scaf11
Pabpn1	0.000797	0.284314	0.335	0.356	1	MG-Inflam	Pabpn1
Evi2a	0.000906	0.356431	0.285	0.297	1	MG-Inflam	Evi2a
Fbxw4	0.001085	0.256381	0.308	0.32	1	MG-Inflam	Fbxw4
Pnn1	0.001354	0.354297	0.262	0.27	1	MG-Inflam	Pnn
Mfsd5	0.001532	0.476005	0.247	0.258	1	MG-Inflam	Mfsd5
Tubb2a	0.002031	0.286518	0.255	0.259	1	MG-Inflam	Tubb2a
Hp1bp3	0.002139	0.308622	0.265	0.272	1	MG-Inflam	Hp1bp3
Gps2	0.003154	0.446822	0.268	0.283	1	MG-Inflam	Gps2
Tex261	0.004071	0.4504	0.244	0.255	1	MG-Inflam	Tex261
BC031181	0.00497	0.309978	0.324	0.35	1	MG-Inflam	BC031181
Slc25a11	0.008104	0.398962	0.273	0.292	1	MG-Inflam	Slc25a11

Lrpap1	0.00865	0.301021	0.327	0.349	1	MG-Inflam	Lrpap1
Cst7	0	2.980614	0.705	0.109	0	TAMs-LA	Cst7
Pld3	0	1.641991	0.852	0.361	0	TAMs-LA	Pld3
Hebp1	0	1.439158	0.676	0.191	0	TAMs-LA	Hebp1
Axl	0	1.222308	0.685	0.201	0	TAMs-LA	Axl
Cstb	0	1.544818	0.796	0.319	0	TAMs-LA	Cstb
Mif	0	1.449146	0.833	0.372	0	TAMs-LA	Mif
Lgals3bp	0	1.677021	0.926	0.473	0	TAMs-LA	Lgals3bp
Itgax	0	2.278414	0.556	0.109	0	TAMs-LA	Itgax
Bcl2a1b	0	0.90173	0.818	0.38	0	TAMs-LA	Bcl2a1b
Lap3	0	0.944436	0.736	0.302	0	TAMs-LA	Lap3
Cxcl16	0	1.047846	0.665	0.244	0	TAMs-LA	Cxcl16
Hspe1	0	0.845541	0.79	0.37	0	TAMs-LA	Hspe1
Glmp1	0	0.806944	0.823	0.413	0	TAMs-LA	Glmp
Cd83	0	0.832634	0.834	0.426	0	TAMs-LA	Cd83
Cd63	0	1.685393	0.975	0.568	0	TAMs-LA	Cd63
Slc11a1	0	0.792856	0.845	0.442	0	TAMs-LA	Slc11a1
Syngr11	0	0.952936	0.823	0.423	0	TAMs-LA	Syngr1
Prdx1	0	1.495672	0.892	0.494	0	TAMs-LA	Prdx1
Tspo	0	0.485192	0.794	0.396	0	TAMs-LA	Tspo
Dnase2a1	0	0.668956	0.731	0.333	0	TAMs-LA	Dnase2a
Akr1a1	0	1.024813	0.913	0.516	0	TAMs-LA	Akr1a1
Npm1	0	0.796815	0.887	0.494	0	TAMs-LA	Npm1
Ly9	0	1.318585	0.536	0.148	0	TAMs-LA	Ly9
Gpi1	0	0.60112	0.789	0.402	0	TAMs-LA	Gpi1
Uba52	0	0.734234	0.794	0.408	0	TAMs-LA	Uba52
Rpl22l1	0	0.753829	0.832	0.446	0	TAMs-LA	Rpl22l1
Pkm	0	0.961689	0.84	0.454	0	TAMs-LA	Pkm
Cox5a	0	0.587727	0.796	0.411	0	TAMs-LA	Cox5a
Rtcb	0	0.812323	0.642	0.257	0	TAMs-LA	Rtcb
Fcgr4	0	1.155902	0.574	0.19	0	TAMs-LA	Fcgr4
Psmb6	0	0.77196	0.711	0.327	0	TAMs-LA	Psmb6
Tubb5	0	0.66016	0.801	0.418	0	TAMs-LA	Tubb5
Vkorc11	0	0.482831	0.753	0.371	0	TAMs-LA	Vkorc1
Naglu1	0	0.727124	0.63	0.248	0	TAMs-LA	Naglu
Renbp	0	0.877387	0.603	0.221	0	TAMs-LA	Renbp
Bst2	0	0.670148	0.921	0.54	0	TAMs-LA	Bst2
Tpi1	0	0.995288	0.646	0.266	0	TAMs-LA	Tpi1
Eif3k	0	0.470152	0.815	0.435	0	TAMs-LA	Eif3k
Eef1g	0	0.62107	0.712	0.332	0	TAMs-LA	Eef1g
Ifi27l2a	0	0.339848	0.713	0.334	0	TAMs-LA	Ifi27l2a
Ccl3	0	1.959277	0.518	0.14	0	TAMs-LA	Ccl3
Nme2	0	0.799946	0.928	0.55	0	TAMs-LA	Nme2
Atp5mpl	0	0.631663	0.746	0.368	0	TAMs-LA	Atp5mpl

Gde1	0	0.976406	0.57	0.193	0	TAMs-LA	Gde1
Ccl4	0	2.11189	0.515	0.139	0	TAMs-LA	Ccl4
Creg1	0	1.091011	0.923	0.548	0	TAMs-LA	Creg1
Lpl	0	2.700108	0.46	0.085	0	TAMs-LA	Lpl
Rpl31	0	0.62533	0.852	0.478	0	TAMs-LA	Rpl31
Gm2a	0	0.513274	0.763	0.389	0	TAMs-LA	Gm2a
Nsa2	0	0.525959	0.772	0.399	0	TAMs-LA	Nsa2
Rtraf	0	0.540918	0.742	0.369	0	TAMs-LA	Rtraf
Sqstm1	0	0.929716	0.837	0.464	0	TAMs-LA	Sqstm1
Gatm	0	0.728844	0.716	0.344	0	TAMs-LA	Gatm
Aldoa	0	1.083645	0.917	0.545	0	TAMs-LA	Aldoa
Selenow	0	0.485116	0.782	0.411	0	TAMs-LA	Selenow
C3ar1	0	0.662764	0.747	0.376	0	TAMs-LA	C3ar1
Aprt	0	0.862777	0.595	0.224	0	TAMs-LA	Aprt
Hint1	0	0.616434	0.848	0.477	0	TAMs-LA	Hint1
Tpp11	0	0.574528	0.792	0.422	0	TAMs-LA	Tpp1
Il1a	0	1.037138	0.595	0.225	0	TAMs-LA	Il1a
Atp6v1f	0	0.651717	0.879	0.509	0	TAMs-LA	Atp6v1f
Ptms	0	0.621029	0.843	0.473	0	TAMs-LA	Ptms
C1qbp	0	0.816408	0.595	0.225	0	TAMs-LA	C1qbp
Timp2	0	1.198298	0.922	0.552	0	TAMs-LA	Timp2
Sdf4	0	0.596665	0.833	0.463	0	TAMs-LA	Sdf4
Gaa	0	0.952435	0.563	0.193	0	TAMs-LA	Gaa
Mfsd1	0	0.647185	0.661	0.292	0	TAMs-LA	Mfsd1
Vegfb	0	0.787451	0.585	0.216	0	TAMs-LA	Vegfb
Edf1	0	0.500542	0.781	0.413	0	TAMs-LA	Edf1
Eef1d	0	0.501343	0.811	0.445	0	TAMs-LA	Eef1d
Tmem86a1	0	0.563007	0.788	0.423	0	TAMs-LA	Tmem86a
Tomm20	0	0.538404	0.719	0.354	0	TAMs-LA	Tomm20
Psme2	0	0.423062	0.836	0.471	0	TAMs-LA	Psme2
Lrpap11	0	0.666793	0.658	0.294	0	TAMs-LA	Lrpap1
Pgam1	0	0.831391	0.664	0.3	0	TAMs-LA	Pgam1
Atp5d	0	0.465746	0.842	0.48	0	TAMs-LA	Atp5d
Ninj1	0	0.801239	0.643	0.281	0	TAMs-LA	Ninj1
Sdhb	0	0.531832	0.711	0.35	0	TAMs-LA	Sdhb
Ubxn1	0	0.492327	0.7	0.339	0	TAMs-LA	Ubxn1
Scpep1	0	0.587356	0.687	0.327	0	TAMs-LA	Scpep1
Ftl1-ps1	0	1.569214	0.532	0.172	0	TAMs-LA	Ftl1-ps1
Rac2	0	0.252159	0.769	0.409	0	TAMs-LA	Rac2
Rps17	0	0.571081	0.876	0.516	0	TAMs-LA	Rps17
Rpl23a	0	0.465563	0.817	0.457	0	TAMs-LA	Rpl23a
Ldha	0	0.678025	0.755	0.396	0	TAMs-LA	Ldha
Gltp	0	0.596017	0.69	0.331	0	TAMs-LA	Gltp
Gusb1	0	0.627193	0.808	0.45	0	TAMs-LA	Gusb

Ndufa1	0	0.688897	0.641	0.283	0	TAMs-LA	Ndufa1
Csf1	0	1.837852	0.453	0.096	0	TAMs-LA	Csf1
Atp6v1g1	0	0.682382	0.881	0.524	0	TAMs-LA	Atp6v1g1
Bax	0	0.640193	0.672	0.316	0	TAMs-LA	Bax
Eif5a	0	0.490995	0.8	0.444	0	TAMs-LA	Eif5a
Lag31	0	0.822569	0.849	0.493	0	TAMs-LA	Lag3
Cotl1	0	0.526225	0.822	0.466	0	TAMs-LA	Cotl1
Sec61b	0	0.375938	0.771	0.416	0	TAMs-LA	Sec61b
C4b	0	0.982603	0.538	0.183	0	TAMs-LA	C4b
Dpp7	0	1.39154	0.476	0.121	0	TAMs-LA	Dpp7
Sdf2l11	0	0.661046	0.707	0.352	0	TAMs-LA	Sdf2l1
Taf10	0	0.438357	0.673	0.319	0	TAMs-LA	Taf10
Atp5g1	0	0.430014	0.774	0.42	0	TAMs-LA	Atp5g1
Eif3i	0	0.579007	0.632	0.278	0	TAMs-LA	Eif3i
Cox6b1	0	0.501231	0.864	0.51	0	TAMs-LA	Cox6b1
Ndufb5	0	0.439608	0.679	0.326	0	TAMs-LA	Ndufb5
Atp6v0e	0	0.511084	0.869	0.516	0	TAMs-LA	Atp6v0e
Cops9	0	0.526584	0.671	0.319	0	TAMs-LA	Cops9
Akr1b3	0	0.662556	0.621	0.269	0	TAMs-LA	Akr1b3
Atp5b	0	0.474494	0.84	0.488	0	TAMs-LA	Atp5b
Dtnbp11	0	0.666178	0.593	0.241	0	TAMs-LA	Dtnbp1
Tkt	0	0.416263	0.73	0.378	0	TAMs-LA	Tkt
Il2rg	0	1.129055	0.525	0.174	0	TAMs-LA	Il2rg
P2rx4	0	0.571272	0.622	0.271	0	TAMs-LA	P2rx4
Lipa	0	1.033553	0.602	0.251	0	TAMs-LA	Lipa
Tmed31	0	0.496083	0.692	0.341	0	TAMs-LA	Tmed3
Sec61g	0	0.427655	0.738	0.388	0	TAMs-LA	Sec61g
Psme1	0	0.623142	0.908	0.558	0	TAMs-LA	Psme1
Mlf2	0	0.47816	0.689	0.339	0	TAMs-LA	Mlf2
Echs1	0	0.862888	0.536	0.187	0	TAMs-LA	Echs1
H2-T22	0	0.557057	0.648	0.299	0	TAMs-LA	H2-T22
Pomp	0	0.476657	0.852	0.503	0	TAMs-LA	Pomp
Uqcr11	0	0.394561	0.733	0.384	0	TAMs-LA	Uqcr11
Abcg11	0	0.834024	0.576	0.227	0	TAMs-LA	Abcg1
Micos13	0	0.565436	0.62	0.271	0	TAMs-LA	Micos13
Ap2m1	0	0.523081	0.723	0.375	0	TAMs-LA	Ap2m1
Mt1	0	0.637787	0.806	0.459	0	TAMs-LA	Mt1
Cox5b	0	0.434198	0.846	0.499	0	TAMs-LA	Cox5b
Bag1	0	0.636607	0.609	0.262	0	TAMs-LA	Bag1
Ifi30	0	0.48227	0.896	0.549	0	TAMs-LA	Ifi30
Ndufa2	0	0.381172	0.737	0.39	0	TAMs-LA	Ndufa2
Slc25a3	0	0.620114	0.911	0.565	0	TAMs-LA	Slc25a3
Atp13a2	0	0.669404	0.575	0.229	0	TAMs-LA	Atp13a2
Os91	0	0.374666	0.728	0.382	0	TAMs-LA	Os9

Uqcrq	0	0.409356	0.738	0.392	0	TAMs-LA	Uqcrq
H2-Q7	0	0.629027	0.782	0.437	0	TAMs-LA	H2-Q7
Ppp1r14b1	0	0.510408	0.671	0.326	0	TAMs-LA	Ppp1r14b
Polr1d	0	0.412219	0.722	0.377	0	TAMs-LA	Polr1d
Atox1	0	0.363248	0.892	0.547	0	TAMs-LA	Atox1
Vdac2	0	0.47519	0.837	0.492	0	TAMs-LA	Vdac2
Slc25a5	0	0.683616	0.892	0.547	0	TAMs-LA	Slc25a5
Eif4a1	0	0.666712	0.873	0.529	0	TAMs-LA	Eif4a1
Ndufv3	0	0.467533	0.671	0.328	0	TAMs-LA	Ndufv3
Pycard1	0	0.624983	0.867	0.524	0	TAMs-LA	Pycard
Pgls	0	0.425943	0.673	0.331	0	TAMs-LA	Pgls
Hvcn11	0	0.478066	0.603	0.261	0	TAMs-LA	Hvcn1
Cndp2	0	0.633228	0.619	0.277	0	TAMs-LA	Cndp2
Med28	0	0.468354	0.654	0.313	0	TAMs-LA	Med28
AI413582	0	0.524108	0.624	0.283	0	TAMs-LA	AI413582
Tbca	0	0.393618	0.722	0.381	0	TAMs-LA	Tbca
Anxa5	0	0.674132	0.599	0.259	0	TAMs-LA	Anxa5
Uqcrh	0	0.553485	0.893	0.553	0	TAMs-LA	Uqcrh
Eif3f	0	0.53253	0.884	0.544	0	TAMs-LA	Eif3f
Rpl4	0	0.569252	0.893	0.553	0	TAMs-LA	Rpl4
Rps15	0	0.46126	0.863	0.523	0	TAMs-LA	Rps15
CAAA0114	0	0.471067	0.697	0.357	0	TAMs-LA	CAAA01147332.1
Rnh1	0	0.53672	0.643	0.304	0	TAMs-LA	Rnh1
Lyz2	0	0.267646	0.972	0.633	0	TAMs-LA	Lyz2
Pgk1	0	0.893169	0.551	0.213	0	TAMs-LA	Pgk1
Wdr83os	0	0.378172	0.681	0.343	0	TAMs-LA	Wdr83os
Gm11808	0	0.633273	0.598	0.26	0	TAMs-LA	Gm11808
0610012G0	0	0.636118	0.589	0.251	0	TAMs-LA	0610012G03Rik
Csf2ra	0	0.443463	0.711	0.373	0	TAMs-LA	Csf2ra
Gba	0	0.816081	0.528	0.191	0	TAMs-LA	Gba
Pebp1	0	0.444682	0.653	0.316	0	TAMs-LA	Pebp1
Sdcbp	0	0.472181	0.823	0.486	0	TAMs-LA	Sdcbp
Hmox1	0	2.682096	0.499	0.162	0	TAMs-LA	Hmox1
Btf3	0	0.486088	0.904	0.568	0	TAMs-LA	Btf3
Rps27l	0	0.327447	0.65	0.314	0	TAMs-LA	Rps27l
Fabp5	0	2.811812	0.431	0.095	0	TAMs-LA	Fabp5
Man2b21	0	0.470509	0.59	0.255	0	TAMs-LA	Man2b2
Psmb8	0	0.446001	0.891	0.556	0	TAMs-LA	Psmb8
Pabpc1	0	0.67437	0.912	0.578	0	TAMs-LA	Pabpc1
Csnk2b	0	0.426307	0.626	0.292	0	TAMs-LA	Csnk2b
Ssr4	0	0.766156	0.933	0.6	0	TAMs-LA	Ssr4
Plaur	0	1.020985	0.463	0.13	0	TAMs-LA	Plaur
Psmb10	0	0.399083	0.671	0.338	0	TAMs-LA	Psmb10
Uqcr10	0	0.396049	0.672	0.339	0	TAMs-LA	Uqcr10

2410006H1	0	0.44087	0.776	0.443	0	TAMs-LA	2410006H16Rik
Tlr2	0	0.896051	0.564	0.231	0	TAMs-LA	Tlr2
Atp6v1c1	0	0.552102	0.601	0.268	0	TAMs-LA	Atp6v1c1
Bri3	0	0.68686	0.906	0.574	0	TAMs-LA	Bri3
Cebpa	0	0.528164	0.63	0.298	0	TAMs-LA	Cebpa
Ndufs3	0	0.461132	0.607	0.275	0	TAMs-LA	Ndufs3
Hnrnpa1	0	0.373018	0.672	0.341	0	TAMs-LA	Hnrnpa1
Atp5o	0	0.309394	0.666	0.335	0	TAMs-LA	Atp5o
Sirt2	0	0.445517	0.601	0.27	0	TAMs-LA	Sirt2
Tmem256	0	0.571677	0.606	0.275	0	TAMs-LA	Tmem256
Cryba41	0	0.729284	0.557	0.227	0	TAMs-LA	Cryba4
Ncl	0	0.457851	0.643	0.313	0	TAMs-LA	Ncl
Srp14	0	0.393942	0.653	0.323	0	TAMs-LA	Srp14
Nucb11	0	0.366974	0.666	0.336	0	TAMs-LA	Nucb1
Mrpl23	0	0.401619	0.629	0.299	0	TAMs-LA	Mrpl23
Ndufb8	0	0.358739	0.68	0.351	0	TAMs-LA	Ndufb8
Mdh2	0	0.350277	0.64	0.311	0	TAMs-LA	Mdh2
Esd	0	0.794335	0.611	0.282	0	TAMs-LA	Esd
Phb2	0	0.489002	0.589	0.26	0	TAMs-LA	Phb2
Atxn10	0	0.322197	0.642	0.314	0	TAMs-LA	Atxn10
Blvrb	0	0.668989	0.596	0.268	0	TAMs-LA	Blvrb
Slc25a39	0	0.453032	0.584	0.256	0	TAMs-LA	Slc25a39
Cebpzos1	0	0.383447	0.607	0.279	0	TAMs-LA	Cebpzos
Ggh	0	0.577887	0.559	0.232	0	TAMs-LA	Ggh
Plbd2	0	0.606092	0.552	0.225	0	TAMs-LA	Plbd2
Rps25	0	0.585408	0.921	0.594	0	TAMs-LA	Rps25
Swi5	0	0.363837	0.663	0.336	0	TAMs-LA	Swi5
Capns1	0	0.477637	0.634	0.307	0	TAMs-LA	Capns1
Gpr137b	0	0.888452	0.513	0.186	0	TAMs-LA	Gpr137b
Colgalt1	0	0.45106	0.613	0.287	0	TAMs-LA	Colgalt1
Eif3a	0	0.429986	0.625	0.299	0	TAMs-LA	Eif3a
Vps29	0	0.527485	0.599	0.273	0	TAMs-LA	Vps29
Cct2	0	0.455824	0.581	0.255	0	TAMs-LA	Cct2
Rack1	0	0.618804	0.939	0.614	0	TAMs-LA	Rack1
1110038B1	0	0.961033	0.478	0.153	0	TAMs-LA	1110038B12Rik
Rpl5	0	0.454651	0.896	0.572	0	TAMs-LA	Rpl5
Pold4	0	0.45892	0.653	0.329	0	TAMs-LA	Pold4
Cox7b	0	0.376262	0.648	0.324	0	TAMs-LA	Cox7b
Tmem160	0	0.499012	0.574	0.25	0	TAMs-LA	Tmem160
Hmgn1	0	0.352409	0.607	0.284	0	TAMs-LA	Hmgn1
Grcc10	0	0.447557	0.865	0.542	0	TAMs-LA	Grcc10
Tent5c	0	0.610727	0.498	0.176	0	TAMs-LA	Tent5c
Bola2	0	0.795433	0.507	0.185	0	TAMs-LA	Bola2
Timm13	0	0.423468	0.616	0.294	0	TAMs-LA	Timm13

Ak2	0	0.551252	0.551	0.23	0	TAMs-LA	Ak2
Hif1a	0	0.700989	0.546	0.225	0	TAMs-LA	Hif1a
Ran	0	0.477232	0.634	0.313	0	TAMs-LA	Ran
Gpr84	0	0.946983	0.498	0.178	0	TAMs-LA	Gpr84
Igbp1	0	0.710953	0.502	0.182	0	TAMs-LA	Igbp1
Rpl36al	0	0.454014	0.924	0.605	0	TAMs-LA	Rpl36al
Psma5	0	0.516289	0.585	0.266	0	TAMs-LA	Psma5
Atp6v1b2	0	0.457617	0.613	0.295	0	TAMs-LA	Atp6v1b2
Cd52	0	0.681461	0.949	0.631	0	TAMs-LA	Cd52
Apoc1	0	3.082775	0.367	0.05	0	TAMs-LA	Apoc1
Txndc17	0	0.280497	0.585	0.268	0	TAMs-LA	Txndc17
Nme1	0	0.724088	0.536	0.22	0	TAMs-LA	Nme1
H2-Oa	0	0.714681	0.601	0.285	0	TAMs-LA	H2-Oa
C3	0	1.541962	0.419	0.103	0	TAMs-LA	C3
Atp6v1a	0	0.575051	0.541	0.226	0	TAMs-LA	Atp6v1a
Pea15a	0	0.345291	0.601	0.286	0	TAMs-LA	Pea15a
Napa	0	0.443602	0.572	0.257	0	TAMs-LA	Napa
Cuedc2	0	0.407313	0.572	0.257	0	TAMs-LA	Cuedc2
Dgkz1	0	0.363374	0.564	0.25	0	TAMs-LA	Dgkz
Sys1	0	0.408733	0.574	0.26	0	TAMs-LA	Sys1
Lat2	0	0.452448	0.586	0.272	0	TAMs-LA	Lat2
Gabarap	0	0.763808	0.947	0.633	0	TAMs-LA	Gabarap
Gng121	0	0.325568	0.569	0.257	0	TAMs-LA	Gng12
Hspd1	0	0.742762	0.498	0.187	0	TAMs-LA	Hspd1
Tbc1d10a	0	0.768533	0.473	0.162	0	TAMs-LA	Tbc1d10a
Trappc2l	0	0.460536	0.557	0.247	0	TAMs-LA	Trappc2l
Rpl36a	0	0.67348	0.92	0.61	0	TAMs-LA	Rpl36a
Ndufs5	0	0.346518	0.602	0.292	0	TAMs-LA	Ndufs5
Mrpl17	0	0.528544	0.533	0.224	0	TAMs-LA	Mrpl17
Ndufab1	0	0.344007	0.602	0.293	0	TAMs-LA	Ndufab1
Cpq	0	0.359992	0.591	0.282	0	TAMs-LA	Cpq
Gapdh	0	0.761782	0.895	0.587	0	TAMs-LA	Gapdh
Ndufa8	0	0.311447	0.591	0.283	0	TAMs-LA	Ndufa8
Svbp	0	0.500553	0.524	0.217	0	TAMs-LA	Svbp
Spint11	0	0.404349	0.592	0.286	0	TAMs-LA	Spint1
Cdc37	0	0.336733	0.59	0.284	0	TAMs-LA	Cdc37
Glb1	0	0.619173	0.495	0.189	0	TAMs-LA	Glb1
Psmd8	0	0.5102	0.583	0.277	0	TAMs-LA	Psmd8
Uqcrfs1	0	0.286778	0.584	0.278	0	TAMs-LA	Uqcrfs1
Ndufc1	0	0.459899	0.547	0.243	0	TAMs-LA	Ndufc1
Uqcc2	0	0.430622	0.549	0.245	0	TAMs-LA	Uqcc2
Uap1l1	0	1.109351	0.436	0.132	0	TAMs-LA	Uap1l1
Aph1a	0	0.384171	0.559	0.256	0	TAMs-LA	Aph1a
Scn1b	0	0.49963	0.486	0.184	0	TAMs-LA	Scn1b

Arl8a	0	0.449362	0.576	0.274	0	TAMs-LA	Arl8a
Cdk4	0	0.513687	0.513	0.212	0	TAMs-LA	Cdk4
Cnpy21	0	0.260999	0.57	0.269	0	TAMs-LA	Cnpy2
Soat1	0	0.471864	0.547	0.247	0	TAMs-LA	Soat1
Ube2v1	0	0.412568	0.545	0.245	0	TAMs-LA	Ube2v1
Il18bp	0	1.602305	0.409	0.109	0	TAMs-LA	Il18bp
Ube2l3	0	0.347587	0.565	0.265	0	TAMs-LA	Ube2l3
Tor3a	0	0.330924	0.55	0.251	0	TAMs-LA	Tor3a
Cct5	0	0.455507	0.529	0.23	0	TAMs-LA	Cct5
Eef1b2	0	0.600329	0.937	0.639	0	TAMs-LA	Eef1b2
Nudt9	0	0.394744	0.522	0.224	0	TAMs-LA	Nudt9
Eif3e	0	0.352079	0.564	0.266	0	TAMs-LA	Eif3e
Plin2	0	1.023387	0.481	0.184	0	TAMs-LA	Plin2
Atp6v1d	0	0.399319	0.538	0.242	0	TAMs-LA	Atp6v1d
Atf3	0	0.283515	0.566	0.271	0	TAMs-LA	Atf3
Atp5g2	0	0.684943	0.943	0.648	0	TAMs-LA	Atp5g2
Romo1	0	0.422474	0.525	0.231	0	TAMs-LA	Romo1
Psmb4	0	0.420713	0.537	0.243	0	TAMs-LA	Psmb4
Tmem9b1	0	0.296615	0.546	0.252	0	TAMs-LA	Tmem9b
Igsf8	0	0.607034	0.484	0.19	0	TAMs-LA	Igsf8
Sin3b	0	0.399033	0.526	0.233	0	TAMs-LA	Sin3b
Cenpx	0	0.36534	0.534	0.241	0	TAMs-LA	Cenpx
Tmem106a	0	0.662816	0.483	0.19	0	TAMs-LA	Tmem106a
Necap2	0	0.381241	0.548	0.256	0	TAMs-LA	Necap2
Rpl12	0	0.698374	0.95	0.658	0	TAMs-LA	Rpl12
Rps6	0	0.541551	0.94	0.648	0	TAMs-LA	Rps6
Sdc3	0	0.478477	0.51	0.219	0	TAMs-LA	Sdc3
Plekhm2	0	0.3567	0.527	0.236	0	TAMs-LA	Plekhm2
Myo1e	0	0.758205	0.406	0.116	0	TAMs-LA	Myo1e
Fuca2	0	0.442698	0.503	0.213	0	TAMs-LA	Fuca2
Alas1	0	0.642649	0.474	0.184	0	TAMs-LA	Alas1
Psmc3	0	0.345864	0.544	0.255	0	TAMs-LA	Psmc3
Aplp2	0	0.508819	0.527	0.238	0	TAMs-LA	Aplp2
Ndufa12	0	0.425227	0.511	0.222	0	TAMs-LA	Ndufa12
Gsto1	0	1.277611	0.39	0.101	0	TAMs-LA	Gsto1
Nudc	0	0.296242	0.529	0.241	0	TAMs-LA	Nudc
Cd300lf	0	1.256474	0.394	0.106	0	TAMs-LA	Cd300lf
Rab20	0	0.703733	0.437	0.15	0	TAMs-LA	Rab20
Cyp4v3	0	0.390435	0.491	0.204	0	TAMs-LA	Cyp4v3
Id2	0	0.872612	0.437	0.15	0	TAMs-LA	Id2
Eif4b	0	0.342638	0.535	0.248	0	TAMs-LA	Eif4b
Snrpd2	0	0.35851	0.512	0.226	0	TAMs-LA	Snrpd2
Rrp1	0	0.391262	0.52	0.234	0	TAMs-LA	Rrp1
Hscb	0	0.87749	0.415	0.129	0	TAMs-LA	Hscb

Sdhd	0	0.375038	0.522	0.238	0	TAMs-LA	Sdhd
Ccl5	0	0.900209	0.405	0.121	0	TAMs-LA	Ccl5
Cpd	0	0.824676	0.419	0.135	0	TAMs-LA	Cpd
Slc15a31	0	0.603631	0.484	0.2	0	TAMs-LA	Slc15a3
Rasgef1b	0	0.480645	0.458	0.176	0	TAMs-LA	Rasgef1b
Tspan41	0	0.290969	0.493	0.211	0	TAMs-LA	Tspan4
Acaa1a	0	0.347041	0.51	0.228	0	TAMs-LA	Acaa1a
Mydgf1	0	0.333962	0.512	0.23	0	TAMs-LA	Mydgf
Epb41l3	0	0.870806	0.38	0.098	0	TAMs-LA	Epb41l3
Naca	0	0.532901	0.948	0.666	0	TAMs-LA	Naca
Naa20	0	0.326113	0.518	0.237	0	TAMs-LA	Naa20
Gars	0	0.600333	0.457	0.176	0	TAMs-LA	Gars
Kxd1	0	0.456884	0.488	0.207	0	TAMs-LA	Kxd1
Hcar2	0	1.752366	0.344	0.063	0	TAMs-LA	Hcar2
Mrpl12	0	0.59694	0.447	0.167	0	TAMs-LA	Mrpl12
Ranbp1	0	0.434942	0.513	0.233	0	TAMs-LA	Ranbp1
Rpl14	0	0.684881	0.959	0.679	0	TAMs-LA	Rpl14
Vcam1	0	0.872985	0.384	0.105	0	TAMs-LA	Vcam1
Yif1b1	0	0.329131	0.516	0.237	0	TAMs-LA	Yif1b
Snrpf	0	0.311114	0.513	0.234	0	TAMs-LA	Snrpf
Amdhd2	0	0.85801	0.416	0.137	0	TAMs-LA	Amdhd2
Aif1	0	0.660082	0.945	0.666	0	TAMs-LA	Aif1
Aurkaip1	0	0.311355	0.503	0.225	0	TAMs-LA	Aurkaip1
Ctdnep1	0	0.538818	0.459	0.181	0	TAMs-LA	Ctdnep1
Tnfsf12	0	0.571002	0.475	0.197	0	TAMs-LA	Tnfsf12
Use1	0	0.376789	0.497	0.22	0	TAMs-LA	Use1
Cct8	0	0.43452	0.498	0.221	0	TAMs-LA	Cct8
Banf1	0	0.389737	0.509	0.232	0	TAMs-LA	Banf1
Hpse	0	1.841443	0.347	0.07	0	TAMs-LA	Hpse
Arpc1b	0	0.457467	0.943	0.666	0	TAMs-LA	Arpc1b
Commd4	0	0.362404	0.491	0.215	0	TAMs-LA	Commd4
Hcfc1r1	0	0.322139	0.505	0.229	0	TAMs-LA	Hcfc1r1
Prxl2b	0	0.704367	0.425	0.15	0	TAMs-LA	Prxl2b
Mbd3	0	0.33566	0.489	0.214	0	TAMs-LA	Mbd3
Zfas1	0	0.767214	0.428	0.154	0	TAMs-LA	Zfas1
Adh5	0	0.498771	0.459	0.185	0	TAMs-LA	Adh5
Fbl	0	0.361661	0.495	0.221	0	TAMs-LA	Fbl
Tcp1	0	0.435655	0.488	0.214	0	TAMs-LA	Tcp1
Elovl1	0	0.434056	0.48	0.207	0	TAMs-LA	Elovl1
Mrpl20	0	0.366036	0.496	0.223	0	TAMs-LA	Mrpl20
Ndufb2	0	0.354285	0.483	0.21	0	TAMs-LA	Ndufb2
Cops6	0	0.46205	0.471	0.198	0	TAMs-LA	Cops6
Spg21	0	0.452226	0.478	0.206	0	TAMs-LA	Spg21
Ccdc86	0	0.879588	0.404	0.132	0	TAMs-LA	Ccdc86

Nceh1	0	1.219739	0.363	0.092	0	TAMs-LA	Nceh1
Denr	0	0.400679	0.463	0.192	0	TAMs-LA	Denr
Fundc2	0	0.81096	0.395	0.125	0	TAMs-LA	Fundc2
Naa38	0	0.384762	0.475	0.206	0	TAMs-LA	Naa38
Dcxr	0	0.52405	0.444	0.175	0	TAMs-LA	Dcxr
Pa2g4	0	0.428559	0.471	0.202	0	TAMs-LA	Pa2g4
Aimp1	0	0.397285	0.467	0.199	0	TAMs-LA	Aimp1
Rpl38	0	0.473983	0.934	0.666	0	TAMs-LA	Rpl38
Ptpmt1	0	0.407782	0.45	0.184	0	TAMs-LA	Ptpmt1
Scamp3	0	0.321067	0.464	0.199	0	TAMs-LA	Scamp3
Ier3	0	1.37657	0.381	0.116	0	TAMs-LA	Ier3
Mpeg1	0	0.594917	0.932	0.667	0	TAMs-LA	Mpeg1
Ccrl2	0	1.15843	0.382	0.118	0	TAMs-LA	Ccrl2
Gnptg	0	0.433066	0.43	0.166	0	TAMs-LA	Gnptg
Npl	0	0.566321	0.422	0.16	0	TAMs-LA	Npl
Crlf2	0	0.572585	0.505	0.243	0	TAMs-LA	Crlf2
Abhd17a	0	0.372346	0.458	0.197	0	TAMs-LA	Abhd17a
Sat1	0	0.744881	0.955	0.695	0	TAMs-LA	Sat1
Gng5	0	0.488829	0.945	0.685	0	TAMs-LA	Gng5
Eef2	0	0.605584	0.947	0.687	0	TAMs-LA	Eef2
Rpl7	0	0.523682	0.96	0.701	0	TAMs-LA	Rpl7
Hsp90ab1	0	0.694218	0.965	0.706	0	TAMs-LA	Hsp90ab1
Zmat2	0	0.339159	0.453	0.194	0	TAMs-LA	Zmat2
Ndufa10	0	0.314832	0.45	0.193	0	TAMs-LA	Ndufa10
Cd91	0	1.051854	0.973	0.716	0	TAMs-LA	Cd9
Hspa8	0	0.646394	0.964	0.707	0	TAMs-LA	Hspa8
Akirin1	0	0.407657	0.446	0.19	0	TAMs-LA	Akirin1
Rragc	0	0.462084	0.442	0.186	0	TAMs-LA	Rragc
Nutf2	0	0.446664	0.434	0.178	0	TAMs-LA	Nutf2
Timm8b	0	0.322115	0.451	0.195	0	TAMs-LA	Timm8b
Lgals3	0	1.227208	0.411	0.157	0	TAMs-LA	Lgals3
Prdx3	0	0.514533	0.422	0.168	0	TAMs-LA	Prdx3
Cd72	0	1.248612	0.357	0.104	0	TAMs-LA	Cd72
Ucp2	0	0.503377	0.947	0.694	0	TAMs-LA	Ucp2
Oxa1l	0	0.282335	0.439	0.186	0	TAMs-LA	Oxa1l
Chst1	0	1.601594	0.303	0.051	0	TAMs-LA	Chst1
Cmc1	0	0.343961	0.436	0.184	0	TAMs-LA	Cmc1
Eif3l	0	0.404138	0.44	0.188	0	TAMs-LA	Eif3l
Neu1	0	0.40393	0.42	0.17	0	TAMs-LA	Neu1
Sars	0	0.375898	0.429	0.18	0	TAMs-LA	Sars
Ctsl1	0	0.716294	0.968	0.719	0	TAMs-LA	Ctsl
Plaat3	0	0.369746	0.424	0.175	0	TAMs-LA	Plaat3
Hexa1	0	0.593188	0.94	0.692	0	TAMs-LA	Hexa
Polr2g	0	0.387602	0.443	0.195	0	TAMs-LA	Polr2g

Slamf8	0	0.653053	0.411	0.163	0	TAMs-LA	Slamf8
Ly6a	0	0.327966	0.475	0.227	0	TAMs-LA	Ly6a
Xlr	0	0.446803	0.392	0.145	0	TAMs-LA	Xlr
Eprs	0	0.443019	0.423	0.176	0	TAMs-LA	Eprs
Rexo2	0	0.396646	0.441	0.194	0	TAMs-LA	Rexo2
Phb	0	0.313129	0.437	0.19	0	TAMs-LA	Phb
Ndufs6	0	0.400548	0.425	0.178	0	TAMs-LA	Ndufs6
Asna1	0	0.625894	0.382	0.137	0	TAMs-LA	Asna1
Ppp1r15a	0	0.556432	0.401	0.158	0	TAMs-LA	Ppp1r15a
Rpl22	0	0.545991	0.958	0.715	0	TAMs-LA	Rpl22
Rps18	0	0.648844	0.97	0.728	0	TAMs-LA	Rps18
Mpdu1	0	0.369841	0.412	0.17	0	TAMs-LA	Mpdu1
Gbp2	0	0.640501	0.412	0.171	0	TAMs-LA	Gbp2
Mrps36	0	0.528968	0.391	0.151	0	TAMs-LA	Mrps36
Slc31a1	0	0.335269	0.414	0.174	0	TAMs-LA	Slc31a1
Fblim1	0	0.934836	0.325	0.085	0	TAMs-LA	Fblim1
Atp6v0c	0	1.015848	0.984	0.744	0	TAMs-LA	Atp6v0c
Mpv17l2	0	0.48095	0.388	0.149	0	TAMs-LA	Mpv17l2
Gclm	0	1.041049	0.406	0.168	0	TAMs-LA	Gclm
Plk3	0	0.576002	0.373	0.135	0	TAMs-LA	Plk3
Mrpl54	0	0.450944	0.401	0.164	0	TAMs-LA	Mrpl54
Pfkl	0	0.520248	0.38	0.144	0	TAMs-LA	Pfkl
Mien1	0	0.356054	0.406	0.17	0	TAMs-LA	Mien1
Mmp14	0	0.444037	0.398	0.162	0	TAMs-LA	Mmp14
Ptrhd1	0	0.591588	0.37	0.134	0	TAMs-LA	Ptrhd1
Rgs1	0	0.567115	0.441	0.206	0	TAMs-LA	Rgs1
Eif3g	0	0.488492	0.388	0.153	0	TAMs-LA	Eif3g
Mea1	0	0.371574	0.403	0.168	0	TAMs-LA	Mea1
Trappc1	0	0.341148	0.409	0.174	0	TAMs-LA	Trappc1
Cfl1	0	0.58079	0.968	0.733	0	TAMs-LA	Cfl1
Nagk	0	0.429475	0.391	0.157	0	TAMs-LA	Nagk
Pkib	0	0.256875	0.405	0.171	0	TAMs-LA	Pkib
Glrx5	0	0.363309	0.386	0.152	0	TAMs-LA	Glrx5
Gfer	0	0.362757	0.401	0.167	0	TAMs-LA	Gfer
Rpl7a	0	0.698194	0.968	0.734	0	TAMs-LA	Rpl7a
Pes1	0	0.532519	0.372	0.138	0	TAMs-LA	Pes1
Cd681	0	0.684962	0.96	0.727	0	TAMs-LA	Cd68
Rps28	0	0.673835	0.959	0.726	0	TAMs-LA	Rps28
Tnfaip3	0	0.345921	0.406	0.174	0	TAMs-LA	Tnfaip3
Arl11	0	0.61208	0.364	0.132	0	TAMs-LA	Arl11
C2	0	1.769654	0.313	0.081	0	TAMs-LA	C2
Ddt	0	0.483451	0.37	0.139	0	TAMs-LA	Ddt
Impdh2	0	0.570887	0.371	0.141	0	TAMs-LA	Impdh2
Polr2f	0	0.34059	0.395	0.165	0	TAMs-LA	Polr2f

Selenoh	0	0.607666	0.356	0.127	0	TAMs-LA	Selenoh
1700003F1	0	1.462273	0.282	0.053	0	TAMs-LA	1700003F12Rik
Rapsn	0	0.669854	0.338	0.11	0	TAMs-LA	Rapsn
Ethe1	0	0.84997	0.334	0.106	0	TAMs-LA	Ethe1
Rab32	0	0.354039	0.393	0.165	0	TAMs-LA	Rab32
Nhp2	0	0.573057	0.37	0.142	0	TAMs-LA	Nhp2
Psmd2	0	0.46601	0.396	0.168	0	TAMs-LA	Psmd2
Apoe	0	1.785585	0.983	0.755	0	TAMs-LA	Apoe
Cfb	0	1.150292	0.302	0.074	0	TAMs-LA	Cfb
Oas1a	0	0.592359	0.355	0.128	0	TAMs-LA	Oas1a
Rpl27	0	0.509347	0.973	0.746	0	TAMs-LA	Rpl27
Cant1	0	0.312313	0.382	0.156	0	TAMs-LA	Cant1
Sod2	0	0.380236	0.386	0.16	0	TAMs-LA	Sod2
Rpl35	0	0.758545	0.969	0.745	0	TAMs-LA	Rpl35
Hint2	0	0.311916	0.376	0.153	0	TAMs-LA	Hint2
Phgdh	0	0.428971	0.381	0.159	0	TAMs-LA	Phgdh
Amz1	0	0.351678	0.374	0.152	0	TAMs-LA	Amz1
Acadl	0	0.37959	0.378	0.158	0	TAMs-LA	Acadl
Snhg12	0	0.48959	0.362	0.142	0	TAMs-LA	Snhg12
AB124611	0	0.561036	0.358	0.138	0	TAMs-LA	AB124611
Slirp	0	0.570156	0.354	0.134	0	TAMs-LA	Slirp
Cd22	0	1.440018	0.273	0.054	0	TAMs-LA	Cd22
Spns1	0	0.317457	0.367	0.148	0	TAMs-LA	Spns1
Atp1a3	0	0.815154	0.304	0.085	0	TAMs-LA	Atp1a3
Rpl15	0	0.665537	0.976	0.757	0	TAMs-LA	Rpl15
Cln5	0	0.510892	0.347	0.128	0	TAMs-LA	Cln5
Cd36	0	0.981351	0.308	0.09	0	TAMs-LA	Cd36
Mtln	0	0.443385	0.354	0.136	0	TAMs-LA	Mtln
Got1	0	0.667998	0.323	0.106	0	TAMs-LA	Got1
Commd9	0	0.71071	0.316	0.099	0	TAMs-LA	Commd9
Zbp1	0	0.254329	0.368	0.151	0	TAMs-LA	Zbp1
Npnt	0	0.254083	0.342	0.126	0	TAMs-LA	Npnt
Igf1	0	1.320382	0.306	0.09	0	TAMs-LA	Igf1
Creld2	0	0.502223	0.362	0.146	0	TAMs-LA	Creld2
Nrp2	0	0.362439	0.346	0.13	0	TAMs-LA	Nrp2
As3mt	0	0.440388	0.339	0.124	0	TAMs-LA	As3mt
Gyg	0	0.59832	0.339	0.125	0	TAMs-LA	Gyg
Qars	0	0.48276	0.345	0.131	0	TAMs-LA	Qars
Cox8a	0	0.457357	0.969	0.755	0	TAMs-LA	Cox8a
Psat1	0	1.462242	0.262	0.049	0	TAMs-LA	Psat1
Rpl3	0	0.744941	0.978	0.765	0	TAMs-LA	Rpl3
Trem21	0	0.508119	0.984	0.771	0	TAMs-LA	Trem2
Mcoln1	0	0.363046	0.342	0.13	0	TAMs-LA	Mcoln1
Acot13	0	0.326737	0.351	0.139	0	TAMs-LA	Acot13

Anxa4	0	0.701426	0.336	0.125	0	TAMs-LA	Anxa4
Arl5c	0	0.632039	0.322	0.111	0	TAMs-LA	Arl5c
Ctla2b	0	1.174288	0.281	0.07	0	TAMs-LA	Ctla2b
Cd274	0	0.784876	0.319	0.108	0	TAMs-LA	Cd274
Ctsa1	0	0.716785	0.968	0.757	0	TAMs-LA	Ctsa
Rpl13a	0	0.652355	0.976	0.765	0	TAMs-LA	Rpl13a
Gm1673	0	1.697763	0.252	0.042	0	TAMs-LA	Gm1673
Rpl8	0	0.45587	0.977	0.767	0	TAMs-LA	Rpl8
Slc2a1	0	0.396295	0.347	0.138	0	TAMs-LA	Slc2a1
Rps19	0	0.689774	0.981	0.772	0	TAMs-LA	Rps19
Rpl24	0	0.566976	0.971	0.762	0	TAMs-LA	Rpl24
Ipo5	0	0.405843	0.343	0.135	0	TAMs-LA	Ipo5
Slc48a1	0	1.073302	0.339	0.131	0	TAMs-LA	Slc48a1
Mrpl11	0	0.451668	0.339	0.131	0	TAMs-LA	Mrpl11
Guca1a	0	0.836547	0.298	0.09	0	TAMs-LA	Guca1a
Smim12	0	0.40658	0.347	0.139	0	TAMs-LA	Smim12
Capg	0	0.767674	0.322	0.115	0	TAMs-LA	Capg
Tlr12	0	0.503911	0.327	0.121	0	TAMs-LA	Tlr12
Clcn7	0	0.48771	0.331	0.125	0	TAMs-LA	Clcn7
Clta	0	0.657025	0.969	0.763	0	TAMs-LA	Clta
Srm	0	0.576768	0.33	0.125	0	TAMs-LA	Srm
Bhlhe41	0	0.46923	0.318	0.113	0	TAMs-LA	Bhlhe41
Tceal9	0	0.712224	0.301	0.096	0	TAMs-LA	Tceal9
Rpl29	0	0.616754	0.978	0.773	0	TAMs-LA	Rpl29
Rpl36	0	0.492961	0.971	0.766	0	TAMs-LA	Rpl36
Coa5	0	0.538406	0.325	0.121	0	TAMs-LA	Coa5
Gnptab	0	0.390731	0.318	0.114	0	TAMs-LA	Gnptab
Ccl2	0	0.993035	0.328	0.124	0	TAMs-LA	Ccl2
Gm10076	0	0.785898	0.306	0.102	0	TAMs-LA	Gm10076
Fdx2	0	0.472442	0.322	0.119	0	TAMs-LA	Fdx2
Rilpl2	0	1.001709	0.286	0.083	0	TAMs-LA	Rilpl2
Npc2	0	0.881857	0.987	0.784	0	TAMs-LA	Npc2
Rplp2	0	0.551919	0.976	0.774	0	TAMs-LA	Rplp2
Dapk3	0	0.361163	0.326	0.125	0	TAMs-LA	Dapk3
Srrd	0	0.595477	0.301	0.1	0	TAMs-LA	Srrd
Rps2	0	0.845966	0.991	0.79	0	TAMs-LA	Rps2
Glrx	0	0.588125	0.327	0.127	0	TAMs-LA	Glrx
Cacna1a	0	0.427076	0.289	0.089	0	TAMs-LA	Cacna1a
Cox4i1	0	0.584929	0.976	0.777	0	TAMs-LA	Cox4i1
Plxna4os1	0	0.379327	0.311	0.113	0	TAMs-LA	Plxna4os1
Bckdk	0	0.3465	0.321	0.124	0	TAMs-LA	Bckdk
Speg	0	1.049341	0.251	0.055	0	TAMs-LA	Speg
Cenpb	0	0.457634	0.318	0.122	0	TAMs-LA	Cenpb
Rpl10a	0	0.916496	0.986	0.79	0	TAMs-LA	Rpl10a

Gm2000	0	0.644226	0.3	0.105	0	TAMs-LA	Gm2000
Bola3	0	0.463318	0.306	0.111	0	TAMs-LA	Bola3
Bcl2a1a	0	0.583065	0.305	0.11	0	TAMs-LA	Bcl2a1a
Paox	0	0.654756	0.291	0.096	0	TAMs-LA	Paox
Rps14	0	0.490583	0.979	0.784	0	TAMs-LA	Rps14
Coq7	0	1.082654	0.251	0.058	0	TAMs-LA	Coq7
Mpst	0	0.689523	0.28	0.088	0	TAMs-LA	Mpst
Tufm	0	0.435194	0.308	0.116	0	TAMs-LA	Tufm
Rspo1	0	1.076063	0.251	0.061	0	TAMs-LA	Rspo1
Meis3	0	0.743441	0.264	0.075	0	TAMs-LA	Meis3
Klc4	0	0.772115	0.266	0.078	0	TAMs-LA	Klc4
Lamp1	0	0.774945	0.986	0.799	0	TAMs-LA	Lamp1
Rps26	0	0.69343	0.983	0.797	0	TAMs-LA	Rps26
Arl2	0	0.415175	0.287	0.103	0	TAMs-LA	Arl2
Tuba1c	0	0.712352	0.277	0.094	0	TAMs-LA	Tuba1c
Rpsa	0	0.623707	0.992	0.809	0	TAMs-LA	Rpsa
Rpl6	0	0.603175	0.986	0.804	0	TAMs-LA	Rpl6
Rpl39	0	0.569337	0.979	0.797	0	TAMs-LA	Rpl39
Pqlc2	0	0.617417	0.274	0.093	0	TAMs-LA	Pqlc2
Rps3	0	0.562302	0.984	0.805	0	TAMs-LA	Rps3
Rhoc	0	0.803705	0.261	0.084	0	TAMs-LA	Rhoc
Cryzl2	0	0.630695	0.256	0.08	0	TAMs-LA	Cryzl2
Rpl26	0	0.568266	0.981	0.805	0	TAMs-LA	Rpl26
Nelfe	0	0.452209	0.273	0.099	0	TAMs-LA	Nelfe
Rplp0	0	0.716529	0.992	0.819	0	TAMs-LA	Rplp0
Bnip3	0	0.662122	0.269	0.097	0	TAMs-LA	Bnip3
Il1b	0	0.914068	0.252	0.081	0	TAMs-LA	Il1b
Rpl18	0	0.471646	0.985	0.816	0	TAMs-LA	Rpl18
Rpl10	0	0.523598	0.986	0.817	0	TAMs-LA	Rpl10
Siglecf	0	0.654237	0.255	0.087	0	TAMs-LA	Siglecf
Acot9	0	0.611595	0.251	0.085	0	TAMs-LA	Acot9
Rpl28	0	0.547413	0.984	0.819	0	TAMs-LA	Rpl28
Rpl17	0	0.535978	0.983	0.818	0	TAMs-LA	Rpl17
Rps21	0	0.527116	0.975	0.812	0	TAMs-LA	Rps21
Rpl34	0	0.488068	0.984	0.822	0	TAMs-LA	Rpl34
Rps5	0	0.586926	0.99	0.831	0	TAMs-LA	Rps5
Rps13	0	0.521169	0.987	0.828	0	TAMs-LA	Rps13
Ctsz	0	1.181933	0.992	0.835	0	TAMs-LA	Ctsz
Grn1	0	0.711646	0.986	0.834	0	TAMs-LA	Grn
Rps15a	0	0.585359	0.987	0.836	0	TAMs-LA	Rps15a
Rps23	0	0.499572	0.985	0.834	0	TAMs-LA	Rps23
Cyba	0	0.673197	0.991	0.841	0	TAMs-LA	Cyba
Rpl37a	0	0.571525	0.983	0.834	0	TAMs-LA	Rpl37a
Rpl37	0	0.483349	0.98	0.831	0	TAMs-LA	Rpl37

Rpl32	0	0.728096	0.99	0.847	0	TAMs-LA	Rpl32
C1qa1	0	0.584342	0.994	0.855	0	TAMs-LA	C1qa
C1qc1	0	0.520632	0.995	0.857	0	TAMs-LA	C1qc
Rps12	0	0.654327	0.988	0.85	0	TAMs-LA	Rps12
Rps7	0	0.531931	0.985	0.849	0	TAMs-LA	Rps7
Rpl9	0	0.473514	0.991	0.858	0	TAMs-LA	Rpl9
Rpl11	0	0.547524	0.991	0.859	0	TAMs-LA	Rpl11
Rps20	0	0.794659	0.993	0.864	0	TAMs-LA	Rps20
Rpl23	0	0.655071	0.994	0.865	0	TAMs-LA	Rpl23
Rps4x	0	0.45555	0.99	0.863	0	TAMs-LA	Rps4x
Rps16	0	0.575394	0.991	0.865	0	TAMs-LA	Rps16
Rps3a1	0	0.410267	0.992	0.867	0	TAMs-LA	Rps3a1
C1qb1	0	0.539864	0.997	0.873	0	TAMs-LA	C1qb
Rpl19	0	0.724889	0.997	0.874	0	TAMs-LA	Rpl19
Rpl35a	0	0.45383	0.99	0.869	0	TAMs-LA	Rpl35a
H2-K1	0	0.550135	0.983	0.864	0	TAMs-LA	H2-K1
Rpl27a	0	0.68463	0.992	0.874	0	TAMs-LA	Rpl27a
Rps10	0	0.556886	0.994	0.876	0	TAMs-LA	Rps10
Rps11	0	0.587803	0.994	0.88	0	TAMs-LA	Rps11
Rpl21	0	0.663669	0.989	0.876	0	TAMs-LA	Rpl21
Tpt1	0	0.404325	0.994	0.882	0	TAMs-LA	Tpt1
Rps8	0	0.595969	0.994	0.886	0	TAMs-LA	Rps8
Rpl30	0	0.468863	0.99	0.883	0	TAMs-LA	Rpl30
Rpl18a	0	0.51872	0.992	0.886	0	TAMs-LA	Rpl18a
Rpl41	0	0.711378	0.995	0.889	0	TAMs-LA	Rpl41
Ctsb	0	1.333049	0.997	0.891	0	TAMs-LA	Ctsb
H2-D1	0	0.654729	0.993	0.89	0	TAMs-LA	H2-D1
Ctsd1	0	1.059328	0.998	0.896	0	TAMs-LA	Ctsd
Rps27a	0	0.550375	0.994	0.894	0	TAMs-LA	Rps27a
B2m	0	0.57672	0.993	0.896	0	TAMs-LA	B2m
Rps9	0	0.451522	0.994	0.899	0	TAMs-LA	Rps9
Tyrobp	0	0.564058	0.998	0.903	0	TAMs-LA	Tyrobp
Ftl1	0	1.53685	1	0.912	0	TAMs-LA	Ftl1
Rpl13	0	0.501232	0.995	0.908	0	TAMs-LA	Rpl13
Rplp1	0	0.527405	0.995	0.909	0	TAMs-LA	Rplp1
Rps24	0	0.484817	0.995	0.919	0	TAMs-LA	Rps24
Ctss1	0	0.522764	0.993	0.924	0	TAMs-LA	Ctss
Fau	0	0.53975	0.998	0.93	0	TAMs-LA	Fau
Eef1a1	0	0.736543	0.999	0.935	0	TAMs-LA	Eef1a1
Psap	0	0.827178	0.997	0.938	0	TAMs-LA	Psap
Itm2b1	0	0.418112	0.997	0.95	0	TAMs-LA	Itm2b
Fth1	0	1.144251	1	0.976	0	TAMs-LA	Fth1
Ccl12	4.86E-308	0.509771	0.672	0.385	1.19E-303	TAMs-LA	Ccl12
Tomm6	1.15E-307	0.4276	0.828	0.487	2.82E-303	TAMs-LA	Tomm6

Serbp1	4.78E-307	0.357944	0.696	0.361	1.17E-302	TAMs-LA	Serbp1
Crtap	6.65E-307	0.445569	0.302	0.114	1.63E-302	TAMs-LA	Crtap
Ppia	9.41E-307	0.370006	0.993	0.864	2.31E-302	TAMs-LA	Ppia
Ybx1	1.14E-306	0.442053	0.867	0.526	2.80E-302	TAMs-LA	Ybx1
Ten1	2.17E-306	0.336541	0.392	0.165	5.30E-302	TAMs-LA	Ten1
Ndufb6	2.53E-306	0.322735	0.495	0.227	6.20E-302	TAMs-LA	Ndufb6
Sra1	2.94E-306	0.328122	0.397	0.169	7.19E-302	TAMs-LA	Sra1
Lgals9	5.60E-306	0.43707	0.833	0.5	1.37E-301	TAMs-LA	Lgals9
Tgif1	5.93E-306	0.421475	0.349	0.142	1.45E-301	TAMs-LA	Tgif1
Psmb1	9.33E-306	0.385767	0.693	0.359	2.29E-301	TAMs-LA	Psmb1
Atp5c1	1.36E-305	0.430653	0.806	0.457	3.32E-301	TAMs-LA	Atp5c1
Ube2m	1.52E-305	0.343058	0.567	0.274	3.73E-301	TAMs-LA	Ube2m
Ap1s1	1.88E-305	0.342079	0.349	0.141	4.61E-301	TAMs-LA	Ap1s1
Mrpl27	2.07E-305	0.438776	0.325	0.128	5.08E-301	TAMs-LA	Mrpl27
Ppt11	3.12E-305	0.327422	0.751	0.404	7.65E-301	TAMs-LA	Ppt1
Lamtor1	3.49E-305	0.397744	0.723	0.385	8.55E-301	TAMs-LA	Lamtor1
Stxbp2	2.62E-304	0.360011	0.358	0.146	6.40E-300	TAMs-LA	Stxbp2
Odc1	4.47E-304	0.454972	0.279	0.104	1.09E-299	TAMs-LA	Odc1
Ddhd1	1.49E-303	0.428339	0.302	0.116	3.64E-299	TAMs-LA	Ddhd1
Chchd5	1.58E-303	0.4877	0.255	0.09	3.88E-299	TAMs-LA	Chchd5
Cct4	3.04E-303	0.327136	0.563	0.269	7.45E-299	TAMs-LA	Cct4
Cuta	3.36E-303	0.326423	0.702	0.362	8.22E-299	TAMs-LA	Cuta
Gm5617	5.50E-303	0.439165	0.298	0.113	1.35E-298	TAMs-LA	Gm5617
Slc25a1	1.64E-302	0.446194	0.281	0.104	4.01E-298	TAMs-LA	Slc25a1
Cox6a1	1.68E-302	0.396589	0.814	0.467	4.11E-298	TAMs-LA	Cox6a1
Aldh2	1.94E-302	0.499897	0.501	0.237	4.75E-298	TAMs-LA	Aldh2
Cmtm3	3.47E-302	0.294029	0.536	0.251	8.49E-298	TAMs-LA	Cmtm3
Rbm3	1.17E-301	0.46285	0.835	0.49	2.86E-297	TAMs-LA	Rbm3
Tbcb	2.46E-301	0.356851	0.494	0.228	6.03E-297	TAMs-LA	Tbcb
Ormdl2	3.93E-301	0.361693	0.388	0.164	9.62E-297	TAMs-LA	Ormdl2
Fam50a	4.31E-301	0.363689	0.364	0.152	1.06E-296	TAMs-LA	Fam50a
Cln8	5.65E-301	0.326072	0.389	0.164	1.38E-296	TAMs-LA	Cln8
Cyb5r3	1.10E-300	0.499228	0.306	0.119	2.70E-296	TAMs-LA	Cyb5r3
Cd74	2.01E-300	0.572037	0.944	0.748	4.93E-296	TAMs-LA	Cd74
Cct3	3.41E-300	0.305594	0.396	0.168	8.35E-296	TAMs-LA	Cct3
Ndufb9	3.55E-300	0.322805	0.753	0.404	8.70E-296	TAMs-LA	Ndufb9
Dctpp1	3.91E-300	0.334448	0.35	0.142	9.57E-296	TAMs-LA	Dctpp1
Hspa9	8.35E-300	0.33753	0.401	0.173	2.05E-295	TAMs-LA	Hspa9
Eif4e2	1.05E-299	0.277797	0.487	0.222	2.56E-295	TAMs-LA	Eif4e2
Clptm1l	1.65E-299	0.257553	0.459	0.204	4.05E-295	TAMs-LA	Clptm1l
Plekho2	6.88E-299	0.304609	0.404	0.176	1.69E-294	TAMs-LA	Plekho2
Ldhb1	1.12E-298	0.388593	0.84	0.492	2.74E-294	TAMs-LA	Ldhb
Mrps24	1.32E-298	0.279911	0.519	0.242	3.23E-294	TAMs-LA	Mrps24
Serpine21	1.70E-298	0.518126	0.851	0.549	4.16E-294	TAMs-LA	Serpine2

Mrpl51	2.58E-298	0.396838	0.369	0.155	6.32E-294	TAMs-LA	Mrpl51
Ssbp41	4.76E-298	0.288566	0.581	0.281	1.17E-293	TAMs-LA	Ssbp4
Tomm7	5.81E-298	0.344654	0.667	0.343	1.42E-293	TAMs-LA	Tomm7
Rnaseh2c	5.75E-297	0.272882	0.441	0.195	1.41E-292	TAMs-LA	Rnaseh2c
Glrx3	6.70E-297	0.295381	0.509	0.237	1.64E-292	TAMs-LA	Glrx3
Rfc2	1.01E-296	0.341782	0.37	0.155	2.48E-292	TAMs-LA	Rfc2
Coro1c	1.05E-296	0.345474	0.389	0.167	2.58E-292	TAMs-LA	Coro1c
Fcer1g	1.16E-296	0.344947	0.995	0.891	2.83E-292	TAMs-LA	Fcer1g
Ufc1	1.25E-296	0.279815	0.49	0.225	3.06E-292	TAMs-LA	Ufc1
Idh3b	2.16E-296	0.258547	0.51	0.237	5.28E-292	TAMs-LA	Idh3b
Atp6v0d1	5.17E-296	0.356876	0.646	0.33	1.27E-291	TAMs-LA	Atp6v0d1
Pacc1	5.19E-296	0.309181	0.4	0.17	1.27E-291	TAMs-LA	Pacc1
Vdac1	7.65E-296	0.285818	0.486	0.223	1.87E-291	TAMs-LA	Vdac1
Gnl3	1.61E-295	0.459508	0.256	0.091	3.94E-291	TAMs-LA	Gnl3
Nrbf2	2.33E-295	0.384046	0.3	0.115	5.70E-291	TAMs-LA	Nrbf2
Gnpda1	2.54E-295	0.363422	0.329	0.131	6.23E-291	TAMs-LA	Gnpda1
Eif3m	3.86E-295	0.282228	0.525	0.245	9.44E-291	TAMs-LA	Eif3m
Gadd45gip	5.18E-295	0.328334	0.359	0.149	1.27E-290	TAMs-LA	Gadd45gip1
Anp32b	7.23E-295	0.291622	0.532	0.251	1.77E-290	TAMs-LA	Anp32b
Mrto4	3.30E-294	0.441808	0.281	0.105	8.08E-290	TAMs-LA	Mrto4
Mvp	3.30E-294	0.350372	0.379	0.161	8.09E-290	TAMs-LA	Mvp
Armc5	4.70E-294	0.407868	0.261	0.094	1.15E-289	TAMs-LA	Armc5
Pdlim41	6.14E-294	0.318964	0.492	0.227	1.50E-289	TAMs-LA	Pdlim4
Coa3	1.21E-293	0.258276	0.526	0.247	2.97E-289	TAMs-LA	Coa3
Sec131	1.65E-292	0.29372	0.523	0.247	4.04E-288	TAMs-LA	Sec13
Sf3a1	2.14E-292	0.313534	0.331	0.133	5.24E-288	TAMs-LA	Sf3a1
Gnl2	3.52E-292	0.303721	0.36	0.149	8.62E-288	TAMs-LA	Gnl2
St13	5.03E-292	0.278583	0.494	0.229	1.23E-287	TAMs-LA	St13
Prkcsh	5.14E-292	0.264711	0.393	0.168	1.26E-287	TAMs-LA	Prkcsh
Taldo1	8.04E-292	0.330101	0.786	0.435	1.97E-287	TAMs-LA	Taldo1
Eif3h	1.50E-291	0.376669	0.793	0.44	3.67E-287	TAMs-LA	Eif3h
H2-Q4	1.78E-291	0.377935	0.726	0.396	4.35E-287	TAMs-LA	H2-Q4
Rabggtb	2.16E-291	0.310088	0.312	0.123	5.29E-287	TAMs-LA	Rabggtb
Eif3b	4.02E-291	0.374187	0.347	0.144	9.84E-287	TAMs-LA	Eif3b
Atp5j2	9.31E-291	0.396112	0.817	0.474	2.28E-286	TAMs-LA	Atp5j2
Mybbp1a	1.41E-290	0.344416	0.323	0.13	3.46E-286	TAMs-LA	Mybbp1a
Rab5c	1.53E-290	0.326844	0.648	0.33	3.75E-286	TAMs-LA	Rab5c
Ciao2a	3.91E-290	0.307098	0.473	0.216	9.57E-286	TAMs-LA	Ciao2a
Park7	4.32E-290	0.328216	0.701	0.365	1.06E-285	TAMs-LA	Park7
Pfdn1	1.44E-289	0.264432	0.384	0.164	3.52E-285	TAMs-LA	Pfdn1
Prelid1	1.97E-289	0.33299	0.598	0.296	4.82E-285	TAMs-LA	Prelid1
Chchd2	1.98E-289	0.3579	0.646	0.332	4.84E-285	TAMs-LA	Chchd2
Ubl5	2.17E-289	0.372869	0.784	0.436	5.31E-285	TAMs-LA	Ubl5
Ptpn6	1.09E-288	0.401253	0.809	0.468	2.67E-284	TAMs-LA	Ptpn6

Slamf9	2.05E-288	0.333456	0.407	0.179	5.01E-284	TAMs-LA	Slamf9
Iah1	6.36E-288	0.430343	0.311	0.124	1.56E-283	TAMs-LA	Iah1
Nectin2	9.15E-288	0.342582	0.261	0.094	2.24E-283	TAMs-LA	Nectin2
Mrpl36	9.98E-288	0.306538	0.39	0.168	2.44E-283	TAMs-LA	Mrpl36
Socs3	1.52E-287	0.36634	0.465	0.223	3.73E-283	TAMs-LA	Socs3
Mrpl30	7.97E-287	0.253105	0.524	0.247	1.95E-282	TAMs-LA	Mrpl30
Hpf1	9.43E-287	0.274452	0.347	0.143	2.31E-282	TAMs-LA	Hpf1
Rps29	1.20E-286	0.462828	0.978	0.836	2.94E-282	TAMs-LA	Rps29
Pigt	1.62E-286	0.251085	0.461	0.209	3.97E-282	TAMs-LA	Pigt
Slc31a2	2.47E-286	0.405484	0.432	0.195	6.04E-282	TAMs-LA	Slc31a2
Gpx4	4.93E-286	0.406776	0.87	0.554	1.21E-281	TAMs-LA	Gpx4
Cct7	5.81E-285	0.280846	0.488	0.226	1.42E-280	TAMs-LA	Cct7
Mrpl34	2.87E-284	0.274167	0.361	0.151	7.02E-280	TAMs-LA	Mrpl34
Nfe2l2	9.41E-284	0.407915	0.779	0.448	2.30E-279	TAMs-LA	Nfe2l2
Fkbp8	1.12E-283	0.29339	0.579	0.285	2.75E-279	TAMs-LA	Fkbp8
Litaf	2.19E-283	0.297271	0.673	0.352	5.35E-279	TAMs-LA	Litaf
Ndufb7	6.06E-283	0.26804	0.654	0.331	1.48E-278	TAMs-LA	Ndufb7
Psmb5	8.53E-283	0.37694	0.599	0.3	2.09E-278	TAMs-LA	Psmb5
Chmp6	1.40E-282	0.386507	0.29	0.112	3.42E-278	TAMs-LA	Chmp6
Snhg1	1.70E-282	0.403247	0.317	0.128	4.17E-278	TAMs-LA	Snhg1
Prelid3b	5.02E-282	0.367	0.313	0.125	1.23E-277	TAMs-LA	Prelid3b
M6pr1	8.11E-282	0.362993	0.761	0.418	1.99E-277	TAMs-LA	M6pr
Lsm4	9.95E-282	0.254451	0.483	0.224	2.44E-277	TAMs-LA	Lsm4
Eci2	6.30E-281	0.275772	0.319	0.128	1.54E-276	TAMs-LA	Eci2
Ogfr	1.72E-280	0.304882	0.391	0.17	4.20E-276	TAMs-LA	Ogfr
Psmc5	2.31E-280	0.27849	0.44	0.199	5.67E-276	TAMs-LA	Psmc5
Ctsc	3.61E-280	0.404394	0.938	0.67	8.83E-276	TAMs-LA	Ctsc
Aco2	3.92E-280	0.252465	0.452	0.206	9.61E-276	TAMs-LA	Aco2
Morrbid	2.41E-279	0.332377	0.265	0.1	5.89E-275	TAMs-LA	Morrbid
Cd48	2.48E-279	0.342837	0.685	0.36	6.08E-275	TAMs-LA	Cd48
1810058I2	4.11E-279	0.266442	0.487	0.229	1.01E-274	TAMs-LA	1810058I24Rik
Txnl1	9.38E-278	0.285484	0.468	0.217	2.30E-273	TAMs-LA	Txnl1
Cln3	1.76E-277	0.25315	0.507	0.24	4.30E-273	TAMs-LA	Cln3
Chchd1	2.11E-277	0.34268	0.395	0.174	5.17E-273	TAMs-LA	Chchd1
Nfkbib	2.54E-277	0.343589	0.361	0.154	6.22E-273	TAMs-LA	Nfkbib
Vps28	7.98E-277	0.283141	0.69	0.361	1.95E-272	TAMs-LA	Vps28
Bscl2	1.07E-276	0.251905	0.456	0.209	2.62E-272	TAMs-LA	Bscl2
Cox7a2l	6.70E-276	0.371154	0.746	0.413	1.64E-271	TAMs-LA	Cox7a2l
Rex1bd	9.38E-276	0.311844	0.448	0.207	2.30E-271	TAMs-LA	Rex1bd
Rbm42	1.11E-275	0.295713	0.474	0.221	2.71E-271	TAMs-LA	Rbm42
Dnajc2	1.19E-275	0.308297	0.34	0.142	2.92E-271	TAMs-LA	Dnajc2
Lamtor2	8.25E-275	0.282347	0.606	0.303	2.02E-270	TAMs-LA	Lamtor2
Dap	3.02E-274	0.420266	0.321	0.132	7.39E-270	TAMs-LA	Dap
Ergic31	8.63E-274	0.298551	0.775	0.429	2.11E-269	TAMs-LA	Ergic3

Chmp4b	1.45E-273	0.341083	0.762	0.419	3.55E-269	TAMs-LA	Chmp4b
Mrpl28	2.34E-273	0.357706	0.31	0.125	5.74E-269	TAMs-LA	Mrpl28
Psmc2	2.35E-273	0.310275	0.416	0.187	5.75E-269	TAMs-LA	Psmc2
Gpr108	4.52E-273	0.272691	0.427	0.193	1.11E-268	TAMs-LA	Gpr108
Fam162a	8.30E-273	0.38937	0.303	0.122	2.03E-268	TAMs-LA	Fam162a
Hsd17b10	1.75E-272	0.327367	0.312	0.127	4.28E-268	TAMs-LA	Hsd17b10
Gnas	1.77E-272	0.437246	0.924	0.698	4.34E-268	TAMs-LA	Gnas
Hras	3.18E-272	0.273733	0.318	0.13	7.79E-268	TAMs-LA	Hras
Rrp7a	4.32E-272	0.380429	0.265	0.1	1.06E-267	TAMs-LA	Rrp7a
Rap2b	6.66E-272	0.565896	0.276	0.108	1.63E-267	TAMs-LA	Rap2b
Rnasek1	1.14E-271	0.36514	0.842	0.51	2.80E-267	TAMs-LA	Rnasek
Myc	1.92E-271	0.709056	0.25	0.094	4.71E-267	TAMs-LA	Myc
Mrps7	2.00E-271	0.282178	0.335	0.139	4.90E-267	TAMs-LA	Mrps7
S100a1	3.32E-271	0.484496	0.355	0.156	8.14E-267	TAMs-LA	S100a1
Lrrc59	4.45E-271	0.331299	0.305	0.123	1.09E-266	TAMs-LA	Lrrc59
Rab5if	8.82E-271	0.332209	0.674	0.353	2.16E-266	TAMs-LA	Rab5if
Psmc4	1.64E-270	0.291303	0.392	0.173	4.02E-266	TAMs-LA	Psmc4
Ndufs8	4.09E-270	0.292011	0.545	0.269	1.00E-265	TAMs-LA	Ndufs8
Mrpl41	4.48E-270	0.384774	0.28	0.109	1.10E-265	TAMs-LA	Mrpl41
Uqcrb	4.80E-270	0.337476	0.709	0.38	1.18E-265	TAMs-LA	Uqcrb
Ap2s1	4.94E-270	0.270379	0.542	0.263	1.21E-265	TAMs-LA	Ap2s1
Apex1	2.15E-269	0.350316	0.318	0.13	5.27E-265	TAMs-LA	Apex1
Pgd	1.57E-268	0.334896	0.506	0.244	3.84E-264	TAMs-LA	Pgd
Arhgdia	3.85E-268	0.401039	0.819	0.49	9.44E-264	TAMs-LA	Arhgdia
Ctnnb1	3.94E-268	0.274836	0.61	0.311	9.64E-264	TAMs-LA	Ctnnb1
Glipr1	1.33E-267	0.470153	0.25	0.093	3.25E-263	TAMs-LA	Glipr1
Psmd12	1.57E-267	0.290558	0.385	0.17	3.84E-263	TAMs-LA	Psmd12
Lamtor3	1.74E-267	0.318815	0.356	0.153	4.27E-263	TAMs-LA	Lamtor3
Atp6ap21	2.85E-267	0.357277	0.766	0.433	6.99E-263	TAMs-LA	Atp6ap2
Snrpe	7.42E-267	0.258627	0.621	0.311	1.82E-262	TAMs-LA	Snrpe
Tomm5	1.00E-266	0.307483	0.39	0.173	2.45E-262	TAMs-LA	Tomm5
Fgl2	1.11E-266	0.280353	0.495	0.239	2.71E-262	TAMs-LA	Fgl2
Higd2a1	1.88E-266	0.282481	0.696	0.371	4.60E-262	TAMs-LA	Higd2a
Dbndd2	3.55E-266	0.297477	0.33	0.139	8.69E-262	TAMs-LA	Dbndd2
Llph	4.40E-266	0.256398	0.444	0.205	1.08E-261	TAMs-LA	Llph
Fcgr31	5.04E-266	0.396975	0.957	0.753	1.23E-261	TAMs-LA	Fcgr3
Fis1	5.60E-266	0.305935	0.764	0.423	1.37E-261	TAMs-LA	Fis1
Bloc1s1	8.79E-266	0.277786	0.428	0.196	2.15E-261	TAMs-LA	Bloc1s1
Txndc9	8.97E-266	0.278919	0.381	0.168	2.20E-261	TAMs-LA	Txndc9
Tmem179b	3.73E-265	0.267856	0.706	0.372	9.13E-261	TAMs-LA	Tmem179b
Cope	7.66E-265	0.299401	0.66	0.344	1.88E-260	TAMs-LA	Cope
Hmga1	1.10E-264	0.453881	0.295	0.119	2.70E-260	TAMs-LA	Hmga1
Slc35f6	3.93E-264	0.307546	0.289	0.114	9.62E-260	TAMs-LA	Slc35f6
Psma7	6.47E-264	0.292094	0.701	0.369	1.59E-259	TAMs-LA	Psma7

Stoml2	1.44E-263	0.320636	0.325	0.136	3.54E-259	TAMs-LA	Stoml2
Tomm40	2.09E-263	0.328087	0.316	0.131	5.12E-259	TAMs-LA	Tomm40
Tlr1	8.22E-263	0.340651	0.26	0.099	2.01E-258	TAMs-LA	Tlr1
Imp4	2.06E-262	0.353562	0.296	0.119	5.05E-258	TAMs-LA	Imp4
Pfdn5	3.46E-262	0.38629	0.922	0.655	8.47E-258	TAMs-LA	Pfdn5
Prmt1	5.41E-262	0.284288	0.388	0.173	1.32E-257	TAMs-LA	Prmt1
Wsb2	1.80E-261	0.295256	0.296	0.12	4.42E-257	TAMs-LA	Wsb2
Atp5e	3.00E-261	0.382139	0.901	0.612	7.34E-257	TAMs-LA	Atp5e
Fuca11	8.14E-261	0.282367	0.717	0.388	1.99E-256	TAMs-LA	Fuca1
Psma2	1.08E-260	0.297726	0.62	0.317	2.64E-256	TAMs-LA	Psma2
Selenof1	2.29E-260	0.373572	0.894	0.583	5.61E-256	TAMs-LA	Selenof
Tmem258	2.73E-260	0.28896	0.689	0.365	6.68E-256	TAMs-LA	Tmem258
Nudt14	1.62E-259	0.323922	0.255	0.097	3.96E-255	TAMs-LA	Nudt14
Blvra	1.76E-259	0.289137	0.364	0.159	4.32E-255	TAMs-LA	Blvra
Dohh	3.11E-259	0.423485	0.262	0.101	7.61E-255	TAMs-LA	Dohh
Smim3	4.68E-259	0.252603	0.304	0.124	1.15E-254	TAMs-LA	Smim3
Icam1	1.02E-258	0.321404	0.59	0.305	2.50E-254	TAMs-LA	Icam1
Mrpl52	1.15E-258	0.307127	0.764	0.427	2.82E-254	TAMs-LA	Mrpl52
Prdx4	1.32E-258	0.281799	0.365	0.16	3.23E-254	TAMs-LA	Prdx4
Mfsd11	2.07E-258	0.26724	0.406	0.183	5.06E-254	TAMs-LA	Mfsd11
Psma6	4.16E-258	0.282538	0.492	0.237	1.02E-253	TAMs-LA	Psma6
Tuba1b	4.96E-258	0.335342	0.626	0.329	1.21E-253	TAMs-LA	Tuba1b
Vcp	1.23E-257	0.315285	0.653	0.347	3.02E-253	TAMs-LA	Vcp
Psmb3	1.48E-257	0.319766	0.676	0.359	3.62E-253	TAMs-LA	Psmb3
Serf2	1.58E-257	0.368975	0.962	0.728	3.87E-253	TAMs-LA	Serf2
Gipc1	1.73E-257	0.264163	0.275	0.108	4.24E-253	TAMs-LA	Gipc1
Dynlt3	5.20E-257	0.250095	0.262	0.101	1.27E-252	TAMs-LA	Dynlt3
Atp5j	1.11E-256	0.356332	0.829	0.499	2.71E-252	TAMs-LA	Atp5j
Mrpl2	2.84E-256	0.299909	0.312	0.13	6.96E-252	TAMs-LA	Mrpl2
Eif1ad	3.61E-256	0.334815	0.255	0.097	8.85E-252	TAMs-LA	Eif1ad
Cox7a2	1.52E-255	0.28831	0.756	0.416	3.71E-251	TAMs-LA	Cox7a2
Atp5l	1.56E-255	0.33723	0.865	0.522	3.81E-251	TAMs-LA	Atp5l
Ssr21	6.74E-254	0.279012	0.579	0.293	1.65E-249	TAMs-LA	Ssr2
Milr1	3.43E-253	0.31817	0.325	0.138	8.41E-249	TAMs-LA	Milr1
Slc3a21	4.24E-253	0.383438	0.826	0.498	1.04E-248	TAMs-LA	Slc3a2
Adrm1	1.42E-252	0.365249	0.339	0.146	3.48E-248	TAMs-LA	Adrm1
Eno1	5.80E-252	0.342587	0.525	0.265	1.42E-247	TAMs-LA	Eno1
Epb41l4aos	1.50E-251	0.414217	0.27	0.108	3.67E-247	TAMs-LA	Epb41l4aos
Psen2	1.80E-251	0.393111	0.286	0.117	4.41E-247	TAMs-LA	Psen2
Atp1b3	2.11E-251	0.305855	0.718	0.396	5.16E-247	TAMs-LA	Atp1b3
Timm10	9.24E-251	0.396078	0.252	0.097	2.26E-246	TAMs-LA	Timm10
Calr1	1.62E-250	0.431474	0.756	0.452	3.97E-246	TAMs-LA	Calr
Psmb2	3.85E-250	0.279929	0.632	0.33	9.43E-246	TAMs-LA	Psmb2
Lamtor4	1.51E-249	0.27364	0.727	0.4	3.69E-245	TAMs-LA	Lamtor4

Sh3bgrl3	2.24E-249	0.293345	0.961	0.716	5.49E-245	TAMs-LA	Sh3bgrl3
Tomm22	4.74E-249	0.251071	0.575	0.289	1.16E-244	TAMs-LA	Tomm22
Yif1a	9.09E-249	0.290281	0.292	0.119	2.23E-244	TAMs-LA	Yif1a
Pdia41	4.79E-248	0.262151	0.563	0.286	1.17E-243	TAMs-LA	Pdia4
Spcs11	4.84E-248	0.306839	0.797	0.462	1.18E-243	TAMs-LA	Spcs1
Pmpcb	6.67E-248	0.287334	0.283	0.115	1.63E-243	TAMs-LA	Pmpcb
Tex264	2.85E-247	0.268555	0.329	0.141	6.98E-243	TAMs-LA	Tex264
Cdc34	4.60E-247	0.372184	0.291	0.12	1.13E-242	TAMs-LA	Cdc34
Ddost1	8.27E-247	0.338211	0.781	0.455	2.03E-242	TAMs-LA	Ddost
Atp5k	1.09E-246	0.271185	0.399	0.184	2.68E-242	TAMs-LA	Atp5k
Timm17a	3.66E-246	0.257867	0.331	0.143	8.97E-242	TAMs-LA	Timm17a
Adipor1	1.47E-245	0.266218	0.713	0.398	3.59E-241	TAMs-LA	Adipor1
Sf3b4	4.69E-244	0.286966	0.369	0.167	1.15E-239	TAMs-LA	Sf3b4
Cd300c21	8.42E-244	0.35728	0.942	0.694	2.06E-239	TAMs-LA	Cd300c2
Efhd2	1.01E-243	0.386711	0.881	0.577	2.48E-239	TAMs-LA	Efhd2
Acvrl1	1.03E-241	0.368486	0.294	0.123	2.53E-237	TAMs-LA	Acvrl1
Nipsnap3b	1.13E-241	0.337247	0.286	0.118	2.76E-237	TAMs-LA	Nipsnap3b
C1galt1c1	2.03E-241	0.294786	0.302	0.127	4.97E-237	TAMs-LA	C1galt1c1
H2-Aa	5.63E-241	0.413044	0.826	0.583	1.38E-236	TAMs-LA	H2-Aa
Eif1a	1.56E-239	0.306383	0.304	0.129	3.83E-235	TAMs-LA	Eif1a
Tgm2	2.79E-239	0.514717	0.26	0.105	6.83E-235	TAMs-LA	Tgm2
Pih1d1	1.67E-238	0.313324	0.266	0.107	4.10E-234	TAMs-LA	Pih1d1
Hnrnpab	6.95E-237	0.276939	0.583	0.303	1.70E-232	TAMs-LA	Hnrnpab
Psmd3	1.72E-236	0.256539	0.335	0.147	4.20E-232	TAMs-LA	Psmd3
Pcna	2.25E-235	0.307768	0.263	0.106	5.51E-231	TAMs-LA	Pcna
Mrpl55	3.20E-235	0.266628	0.274	0.112	7.84E-231	TAMs-LA	Mrpl55
Coq10b	4.78E-235	0.342834	0.27	0.111	1.17E-230	TAMs-LA	Coq10b
Ssrp1	6.76E-235	0.284146	0.325	0.142	1.66E-230	TAMs-LA	Ssrp1
Hdgf	1.57E-234	0.294996	0.336	0.149	3.85E-230	TAMs-LA	Hdgf
Dad11	3.00E-234	0.317372	0.817	0.49	7.35E-230	TAMs-LA	Dad1
Nfkbid	3.03E-233	0.498802	0.276	0.117	7.43E-229	TAMs-LA	Nfkbid
Ppfia41	4.55E-233	0.325727	0.761	0.454	1.11E-228	TAMs-LA	Ppfia4
Med29	1.55E-232	0.272257	0.265	0.107	3.80E-228	TAMs-LA	Med29
Fos	4.11E-232	0.324499	0.713	0.445	1.01E-227	TAMs-LA	Fos
Ctsh	6.15E-231	0.353413	0.955	0.762	1.50E-226	TAMs-LA	Ctsh
Tmem126a	7.82E-231	0.262467	0.279	0.116	1.91E-226	TAMs-LA	Tmem126a
Coro1b	6.62E-230	0.298775	0.753	0.427	1.62E-225	TAMs-LA	Coro1b
Irf8	8.11E-230	0.287134	0.873	0.562	1.99E-225	TAMs-LA	Irf8
Atp5g3	1.74E-229	0.329773	0.866	0.55	4.26E-225	TAMs-LA	Atp5g3
Sdc4	2.80E-229	0.418555	0.273	0.115	6.86E-225	TAMs-LA	Sdc4
Trappc6a	5.56E-229	0.311007	0.291	0.124	1.36E-224	TAMs-LA	Trappc6a
Tmem251	4.91E-228	0.432962	0.25	0.1	1.20E-223	TAMs-LA	Tmem251
Rps27	6.32E-228	0.378833	0.948	0.757	1.55E-223	TAMs-LA	Rps27
Tmem14c1	4.17E-226	0.272092	0.717	0.399	1.02E-221	TAMs-LA	Tmem14c

Ubb	1.24E-225	0.296158	0.986	0.847	3.03E-221	TAMs-LA	Ubb
Cox7c	2.20E-225	0.320828	0.849	0.532	5.38E-221	TAMs-LA	Cox7c
Cd811	8.63E-225	0.373608	0.979	0.784	2.11E-220	TAMs-LA	Cd81
Cox6c	2.40E-224	0.299289	0.858	0.535	5.88E-220	TAMs-LA	Cox6c
Wdr1	1.48E-223	0.252897	0.611	0.328	3.63E-219	TAMs-LA	Wdr1
Ufm1	2.44E-223	0.256759	0.289	0.124	5.96E-219	TAMs-LA	Ufm1
Stat1	1.19E-221	0.263949	0.757	0.457	2.92E-217	TAMs-LA	Stat1
P4hb1	1.17E-219	0.333811	0.758	0.448	2.87E-215	TAMs-LA	P4hb
Camk11	4.73E-218	0.296547	0.837	0.517	1.16E-213	TAMs-LA	Camk1
Zbtb8os	1.90E-217	0.276485	0.251	0.102	4.66E-213	TAMs-LA	Zbtb8os
Atp5a1	7.95E-217	0.270279	0.747	0.429	1.95E-212	TAMs-LA	Atp5a1
Ndufa7	1.24E-216	0.252701	0.779	0.452	3.04E-212	TAMs-LA	Ndufa7
Ndufa13	2.00E-216	0.301378	0.812	0.494	4.89E-212	TAMs-LA	Ndufa13
2610001J0	2.41E-216	0.25802	0.253	0.103	5.91E-212	TAMs-LA	2610001J05Rik
Exosc4	2.84E-216	0.253581	0.25	0.103	6.96E-212	TAMs-LA	Exosc4
Actb	1.34E-215	0.250741	0.999	0.952	3.29E-211	TAMs-LA	Actb
Bak1	3.29E-213	0.254391	0.271	0.115	8.05E-209	TAMs-LA	Bak1
Tmem242	1.90E-212	0.267467	0.251	0.104	4.66E-208	TAMs-LA	Tmem242
Mrpl40	6.75E-212	0.276952	0.255	0.106	1.65E-207	TAMs-LA	Mrpl40
H2-Ab1	8.99E-210	0.26284	0.869	0.612	2.20E-205	TAMs-LA	H2-Ab1
Fbxo6	9.59E-210	0.250243	0.287	0.125	2.35E-205	TAMs-LA	Fbxo6
Cat	2.27E-208	0.454589	0.341	0.16	5.56E-204	TAMs-LA	Cat
Lilrb4a	1.61E-204	0.799123	0.307	0.149	3.95E-200	TAMs-LA	Lilrb4a
Spcs21	1.84E-204	0.256599	0.776	0.46	4.51E-200	TAMs-LA	Spcs2
Itm2c1	3.13E-202	0.304342	0.916	0.651	7.67E-198	TAMs-LA	Itm2c
Dusp1	4.06E-202	0.268634	0.595	0.353	9.95E-198	TAMs-LA	Dusp1
Plau	4.06E-202	0.637676	0.299	0.136	9.95E-198	TAMs-LA	Plau
H2-Eb1	5.81E-202	0.287226	0.803	0.562	1.42E-197	TAMs-LA	H2-Eb1
Pla2g7	7.85E-202	0.439403	0.303	0.14	1.92E-197	TAMs-LA	Pla2g7
Atp6ap11	5.41E-201	0.268533	0.782	0.465	1.32E-196	TAMs-LA	Atp6ap1
Nfkbia	1.42E-198	0.301736	0.734	0.451	3.48E-194	TAMs-LA	Nfkbia
Man2b11	6.74E-198	0.328221	0.924	0.702	1.65E-193	TAMs-LA	Man2b1
Elob	6.15E-197	0.262094	0.8	0.482	1.51E-192	TAMs-LA	Elob
Cyb5a	3.98E-196	0.335436	0.651	0.359	9.75E-192	TAMs-LA	Cyb5a
Vamp8	1.25E-195	0.291318	0.867	0.553	3.06E-191	TAMs-LA	Vamp8
Ier2	5.13E-195	0.25242	0.555	0.324	1.26E-190	TAMs-LA	Ier2
Tapbp	5.73E-195	0.253538	0.781	0.459	1.40E-190	TAMs-LA	Tapbp
Zfp36	1.04E-189	0.259643	0.885	0.692	2.55E-185	TAMs-LA	Zfp36
Ly861	6.37E-176	0.307902	0.978	0.903	1.56E-171	TAMs-LA	Ly86
Bsg1	2.23E-170	0.25389	0.906	0.643	5.47E-166	TAMs-LA	Bsg
Arpc3	1.15E-168	0.262273	0.901	0.6	2.82E-164	TAMs-LA	Arpc3
H2-T23	4.00E-157	0.252743	0.924	0.682	9.79E-153	TAMs-LA	H2-T23
Ubc	1.38E-155	0.259393	0.936	0.735	3.37E-151	TAMs-LA	Ubc
Erp291	2.09E-149	0.257491	0.911	0.657	5.11E-145	TAMs-LA	Erp29

Egr1	0	2.06585	0.872	0.316	0	MG-Transcı	Egr1
Fos1	0	1.617094	0.839	0.433	0	MG-Transcı	Fos
Ier21	0	1.929286	0.693	0.31	0	MG-Transcı	Ier2
Ier5	0	1.584201	0.881	0.501	0	MG-Transcı	Ier5
Btg21	0	1.651771	0.944	0.573	0	MG-Transcı	Btg2
Socs31	0	2.161726	0.577	0.213	0	MG-Transcı	Socs3
1-Jun	0	1.641251	0.948	0.595	0	MG-Transcı	Jun
Junb	0	1.779403	0.982	0.656	0	MG-Transcı	Junb
Crybb11	0	0.90804	0.785	0.474	0	MG-Transcı	Crybb1
Tmem1191	0	0.672536	0.914	0.607	0	MG-Transcı	Tmem119
Zfp361	0	1.62361	0.988	0.681	0	MG-Transcı	Zfp36
Siglech2	0	0.603336	0.927	0.627	0	MG-Transcı	Siglech
Gpr342	0	0.638925	0.932	0.632	0	MG-Transcı	Gpr34
Olfml31	0	0.686844	0.954	0.657	0	MG-Transcı	Olfml3
Cd831	0	1.004709	0.742	0.448	0	MG-Transcı	Cd83
Mir142hg	0	1.107352	0.847	0.558	0	MG-Transcı	Mir142hg
Dusp11	0	1.22623	0.64	0.352	0	MG-Transcı	Dusp1
Rhob1	0	1.48649	0.977	0.692	0	MG-Transcı	Rhob
Nfkbia1	0	1.166807	0.739	0.457	0	MG-Transcı	Nfkbia
Jund	0	1.377189	0.934	0.656	0	MG-Transcı	Jund
Ltc4s1	0	0.62691	0.858	0.593	0	MG-Transcı	Ltc4s
Sparc1	0	0.790852	0.98	0.719	0	MG-Transcı	Sparc
Il1a1	0	1.36026	0.504	0.246	0	MG-Transcı	Il1a
Ctsl2	0	0.656731	0.975	0.723	0	MG-Transcı	Ctsl
P2ry122	0	0.555179	0.98	0.736	0	MG-Transcı	P2ry12
Dennd4a1	0	0.63675	0.828	0.585	0	MG-Transcı	Dennd4a
Kctd122	0	0.839238	0.828	0.59	0	MG-Transcı	Kctd12
Gng101	0	0.654064	0.841	0.605	0	MG-Transcı	Gng10
Mafb1	0	0.908768	0.913	0.686	0	MG-Transcı	Mafb
Sgk12	0	1.126895	0.584	0.361	0	MG-Transcı	Sgk1
Basp12	0	0.591005	0.878	0.662	0	MG-Transcı	Basp1
Trem22	0	0.553796	0.99	0.775	0	MG-Transcı	Trem2
Ubc1	0	1.016475	0.949	0.737	0	MG-Transcı	Ubc
Cd812	0	0.621314	0.996	0.785	0	MG-Transcı	Cd81
Zfp36l11	0	0.802696	0.818	0.608	0	MG-Transcı	Zfp36l1
Plek1	0	0.620767	0.84	0.632	0	MG-Transcı	Plek
Fcgr32	0	0.539449	0.961	0.757	0	MG-Transcı	Fcgr3
Glul2	0	0.730875	0.91	0.719	0	MG-Transcı	Glul
Ifngr11	0	0.49806	0.917	0.73	0	MG-Transcı	Ifngr1
Selplg1	0	0.571795	0.97	0.786	0	MG-Transcı	Selplg
Selenop1	0	0.498958	0.969	0.797	0	MG-Transcı	Selenop
Cx3cr12	0	0.563786	0.987	0.816	0	MG-Transcı	Cx3cr1
Marcks1	0	0.524443	0.944	0.773	0	MG-Transcı	Marcks
Rgs101	0	0.522905	0.952	0.786	0	MG-Transcı	Rgs10

C1qa2	0	0.45109	1	0.857	0	MG-Transci	C1qa
C1qc2	0	0.468758	1	0.859	0	MG-Transci	C1qc
H3f3b	0	1.064416	0.991	0.86	0	MG-Transci	H3f3b
C1qb2	0	0.462345	1	0.876	0	MG-Transci	C1qb
Lgmn1	0	0.485573	0.999	0.888	0	MG-Transci	Lgmn
Laptm51	0	0.440769	0.991	0.886	0	MG-Transci	Laptm5
Serinc31	0	0.472451	0.99	0.886	0	MG-Transci	Serinc3
Csf1r1	0	0.521333	0.998	0.896	0	MG-Transci	Csf1r
Ly862	0	0.463778	0.997	0.902	0	MG-Transci	Ly86
Ctss2	0	0.515186	0.999	0.924	0	MG-Transci	Ctss
Hexb1	0	0.580341	1	0.968	0	MG-Transci	Hexb
Cst31	0	0.755663	1	0.986	0	MG-Transci	Cst3
Ccr52	1.54E-305	0.728514	0.709	0.468	3.76E-301	MG-Transci	Ccr5
Itm2b2	1.25E-298	0.396906	0.999	0.951	3.06E-294	MG-Transci	Itm2b
Adgrg11	4.13E-291	0.659025	0.622	0.362	1.01E-286	MG-Transci	Adgrg1
Ddx5	3.04E-288	0.526814	0.911	0.77	7.45E-284	MG-Transci	Ddx5
Unc93b11	1.96E-277	0.393755	0.979	0.849	4.81E-273	MG-Transci	Unc93b1
Ckb1	2.71E-273	0.534983	0.846	0.644	6.64E-269	MG-Transci	Ckb
F11r1	8.84E-272	0.545939	0.809	0.568	2.17E-267	MG-Transci	F11r
Pnrc1	2.24E-265	0.77107	0.68	0.475	5.49E-261	MG-Transci	Pnrc1
Cd371	3.89E-265	0.514775	0.847	0.623	9.53E-261	MG-Transci	Cd37
Susd31	1.65E-264	0.657772	0.674	0.449	4.04E-260	MG-Transci	Susd3
Ccl121	9.64E-259	0.738847	0.629	0.397	2.36E-254	MG-Transci	Ccl12
Srsf5	2.04E-258	0.631575	0.765	0.579	5.00E-254	MG-Transci	Srsf5
Cd141	7.72E-258	0.653767	0.746	0.521	1.89E-253	MG-Transci	Cd14
Itgb52	2.85E-254	0.419948	0.955	0.773	6.98E-250	MG-Transci	Itgb5
Lpcat22	4.77E-252	0.412053	0.957	0.779	1.17E-247	MG-Transci	Lpcat2
Serpine22	1.56E-247	0.472653	0.834	0.558	3.82E-243	MG-Transci	Serpine2
Ldhb2	3.93E-246	0.578945	0.746	0.513	9.64E-242	MG-Transci	Ldhb
Atf31	1.12E-243	0.817629	0.492	0.288	2.73E-239	MG-Transci	Atf3
Lag32	3.06E-243	0.523206	0.779	0.511	7.49E-239	MG-Transci	Lag3
Scamp21	3.84E-242	0.471135	0.864	0.689	9.41E-238	MG-Transci	Scamp2
Ecscr2	6.85E-236	0.645571	0.486	0.267	1.68E-231	MG-Transci	Ecscr
Ppp1r15a1	8.52E-236	1.45564	0.335	0.172	2.09E-231	MG-Transci	Ppp1r15a
Rnase41	4.24E-235	0.486985	0.79	0.56	1.04E-230	MG-Transci	Rnase4
Fscn11	1.86E-232	0.501971	0.54	0.325	4.56E-228	MG-Transci	Fscn1
Fcrls1	1.08E-220	0.434967	0.841	0.603	2.64E-216	MG-Transci	Fcrls
Cd92	1.08E-216	0.359265	0.945	0.725	2.64E-212	MG-Transci	Cd9
Vsir2	2.28E-216	0.382264	0.888	0.707	5.58E-212	MG-Transci	Vsir
St3gal62	2.64E-215	0.447936	0.749	0.518	6.47E-211	MG-Transci	St3gal6
Tmem1731	8.04E-211	0.585724	0.669	0.468	1.97E-206	MG-Transci	Tmem173
P2ry132	3.28E-205	0.514796	0.615	0.397	8.02E-201	MG-Transci	P2ry13
Hpgd1	5.54E-201	0.48142	0.732	0.505	1.36E-196	MG-Transci	Hpgd
Cebpb	8.36E-201	0.423853	0.726	0.516	2.05E-196	MG-Transci	Cebpb

Adrb21	1.70E-199	0.757147	0.535	0.343	4.16E-195	MG-Transcı	Adrb2
Dusp6	2.19E-199	0.808804	0.537	0.36	5.36E-195	MG-Transcı	Dusp6
Ctsf1	3.80E-198	0.592086	0.576	0.371	9.30E-194	MG-Transcı	Ctsf
Fosb	3.47E-194	0.990811	0.295	0.148	8.50E-190	MG-Transcı	Fosb
Sft2d11	4.57E-194	0.450643	0.8	0.621	1.12E-189	MG-Transcı	Sft2d1
Il10ra2	5.59E-192	0.446791	0.831	0.656	1.37E-187	MG-Transcı	Il10ra
Sall12	1.09E-188	0.492268	0.626	0.41	2.67E-184	MG-Transcı	Sall1
Txnip	1.24E-188	0.586343	0.694	0.521	3.04E-184	MG-Transcı	Txnip
Bin21	1.57E-188	0.510006	0.745	0.564	3.84E-184	MG-Transcı	Bin2
Trf1	3.59E-188	0.358782	0.906	0.714	8.79E-184	MG-Transcı	Trf
Ptgs12	7.01E-188	0.394401	0.829	0.601	1.72E-183	MG-Transcı	Ptgs1
Pld41	5.40E-187	0.396331	0.919	0.732	1.32E-182	MG-Transcı	Pld4
Irf2bpl	5.26E-186	1.11259	0.302	0.157	1.29E-181	MG-Transcı	Irf2bpl
Herpud11	1.44E-184	0.72201	0.545	0.366	3.53E-180	MG-Transcı	Herpud1
Saraf1	1.82E-184	0.526166	0.667	0.481	4.47E-180	MG-Transcı	Saraf
Slc2a51	2.68E-182	0.566152	0.447	0.256	6.56E-178	MG-Transcı	Slc2a5
Bin12	1.19E-179	0.374208	0.843	0.663	2.91E-175	MG-Transcı	Bin1
Ctc11	4.01E-179	0.654836	0.498	0.318	9.83E-175	MG-Transcı	Ctc1
Itm2c2	3.27E-178	0.417124	0.849	0.666	8.00E-174	MG-Transcı	Itm2c
Hist1h1e1	2.38E-176	0.822994	0.431	0.265	5.82E-172	MG-Transcı	Hist1h1e
Gm26532	4.96E-175	1.393827	0.25	0.123	1.21E-170	MG-Transcı	Gm26532
Ywhah1	4.53E-173	0.502325	0.75	0.585	1.11E-168	MG-Transcı	Ywhah
Usp22	2.02E-171	0.643092	0.507	0.327	4.95E-167	MG-Transcı	Usp2
Tmem50a1	1.40E-170	0.42249	0.824	0.646	3.43E-166	MG-Transcı	Tmem50a
Cd531	1.27E-168	0.432182	0.778	0.611	3.12E-164	MG-Transcı	Cd53
Grn2	1.45E-164	0.299428	0.986	0.837	3.55E-160	MG-Transcı	Grn
Cebpa1	6.42E-163	0.853346	0.481	0.326	1.57E-158	MG-Transcı	Cebpa
Hist1h1c	2.81E-162	0.992735	0.342	0.198	6.88E-158	MG-Transcı	Hist1h1c
Abi32	3.74E-160	0.42723	0.736	0.551	9.16E-156	MG-Transcı	Abi3
Nfkbiz1	9.22E-160	0.80824	0.392	0.24	2.26E-155	MG-Transcı	Nfkbiz
Mylip2	1.11E-159	0.493234	0.653	0.473	2.71E-155	MG-Transcı	Mylip
Adora31	7.07E-159	0.619959	0.425	0.258	1.73E-154	MG-Transcı	Adora3
Tcn21	6.28E-158	0.450069	0.67	0.482	1.54E-153	MG-Transcı	Tcn2
P2ry61	9.58E-158	0.412775	0.802	0.635	2.35E-153	MG-Transcı	P2ry6
Scoc1	4.81E-157	0.434419	0.63	0.44	1.18E-152	MG-Transcı	Scoc
Tex14	5.25E-157	1.006912	0.285	0.152	1.29E-152	MG-Transcı	Tex14
Zfp36l2	3.09E-156	0.524438	0.675	0.505	7.57E-152	MG-Transcı	Zfp36l2
Sirpa2	6.91E-156	0.30669	0.938	0.817	1.69E-151	MG-Transcı	Sirpa
Calm21	1.25E-155	0.404724	0.802	0.652	3.06E-151	MG-Transcı	Calm2
Cmtm61	3.14E-154	0.502622	0.605	0.435	7.68E-150	MG-Transcı	Cmtm6
1700017B(	3.95E-152	0.623564	0.498	0.333	9.67E-148	MG-Transcı	1700017B05Rik
Btg1	5.93E-151	0.46416	0.676	0.512	1.45E-146	MG-Transcı	Btg1
Rhoh2	1.00E-149	0.499504	0.571	0.396	2.46E-145	MG-Transcı	Rhoh
Man2b12	1.04E-149	0.347957	0.871	0.714	2.55E-145	MG-Transcı	Man2b1

Cfh2	3.73E-149	0.316354	0.79	0.59	9.14E-145	MG-Transcı	Cfh
Gal3st41	4.81E-148	0.522219	0.513	0.334	1.18E-143	MG-Transcı	Gal3st4
Sox41	6.07E-147	0.960113	0.322	0.182	1.49E-142	MG-Transcı	Sox4
Adrb11	2.17E-146	0.798787	0.307	0.169	5.32E-142	MG-Transcı	Adrb1
Cxxc51	1.09E-145	0.568741	0.439	0.276	2.66E-141	MG-Transcı	Cxxc5
Plekho11	4.87E-145	0.449548	0.718	0.56	1.19E-140	MG-Transcı	Plekho1
Sall3	7.31E-145	0.842113	0.32	0.181	1.79E-140	MG-Transcı	Sall3
Cd682	1.55E-143	0.288329	0.929	0.736	3.79E-139	MG-Transcı	Cd68
Csnk1e1	3.27E-143	0.601065	0.522	0.365	8.00E-139	MG-Transcı	Csnk1e
Ccl41	6.97E-143	1.125081	0.308	0.176	1.71E-138	MG-Transcı	Ccl4
Rplp11	1.52E-142	0.274275	0.993	0.911	3.72E-138	MG-Transcı	Rplp1
Golm12	2.12E-140	0.346465	0.689	0.491	5.20E-136	MG-Transcı	Golm1
Ctsa2	2.68E-140	0.307137	0.934	0.766	6.55E-136	MG-Transcı	Ctsa
Camk12	2.91E-140	0.401359	0.722	0.54	7.13E-136	MG-Transcı	Camk1
Phyhd12	4.03E-138	0.316988	0.722	0.526	9.88E-134	MG-Transcı	Phyhd1
Rad51b1	3.52E-135	0.260382	0.614	0.429	8.61E-131	MG-Transcı	Rad51b
Tsc22d3	9.49E-133	0.842355	0.424	0.285	2.32E-128	MG-Transcı	Tsc22d3
Lair12	3.10E-130	0.287847	0.881	0.718	7.59E-126	MG-Transcı	Lair1
Mat2a	5.72E-130	0.751592	0.453	0.316	1.40E-125	MG-Transcı	Mat2a
Comt1	4.77E-127	0.368811	0.719	0.553	1.17E-122	MG-Transcı	Comt
Klk8	7.20E-127	0.871479	0.257	0.139	1.76E-122	MG-Transcı	Klk8
Smad71	9.23E-127	0.538806	0.453	0.303	2.26E-122	MG-Transcı	Smad7
Pla2g151	1.16E-126	0.419547	0.612	0.447	2.84E-122	MG-Transcı	Pla2g15
Zeb2os1	9.70E-126	0.625316	0.48	0.337	2.38E-121	MG-Transcı	Zeb2os
Cd332	1.51E-124	0.376476	0.692	0.527	3.70E-120	MG-Transcı	Cd33
Ctsh1	1.15E-123	0.313845	0.926	0.77	2.82E-119	MG-Transcı	Ctsh
Mtdh2	6.82E-123	0.311589	0.819	0.673	1.67E-118	MG-Transcı	Mtdh
Tmem176b	5.51E-120	0.262681	0.801	0.606	1.35E-115	MG-Transcı	Tmem176b
Fcgr2b	3.27E-119	0.337586	0.808	0.635	8.00E-115	MG-Transcı	Fcgr2b
Rgs2	5.08E-119	0.584177	0.521	0.381	1.24E-114	MG-Transcı	Rgs2
Otulinl2	1.58E-118	0.287105	0.822	0.668	3.88E-114	MG-Transcı	Otulinl
Rnf19b	1.73E-118	0.566835	0.435	0.299	4.23E-114	MG-Transcı	Rnf19b
Smap22	2.26E-117	0.260324	0.783	0.628	5.53E-113	MG-Transcı	Smap2
Ndfip11	8.24E-115	0.405453	0.698	0.561	2.02E-110	MG-Transcı	Ndfip1
Pmepa12	1.51E-114	0.286999	0.758	0.585	3.69E-110	MG-Transcı	Pmepa1
Arsb2	2.52E-114	0.325957	0.589	0.422	6.17E-110	MG-Transcı	Arsb
Icam11	2.57E-113	0.629953	0.467	0.328	6.30E-109	MG-Transcı	Icam1
Laptm4a1	1.42E-112	0.294887	0.877	0.718	3.47E-108	MG-Transcı	Laptm4a
Orai11	3.03E-112	0.425981	0.608	0.468	7.42E-108	MG-Transcı	Orai1
Itgam2	3.80E-111	0.300652	0.774	0.621	9.31E-107	MG-Transcı	Itgam
Rps291	2.41E-110	0.321101	0.969	0.84	5.91E-106	MG-Transcı	Rps29
Tagap	2.66E-110	0.72127	0.363	0.239	6.51E-106	MG-Transcı	Tagap
Ppfia42	4.27E-110	0.419704	0.632	0.479	1.05E-105	MG-Transcı	Ppfia4
Hps42	6.29E-110	0.447208	0.48	0.336	1.54E-105	MG-Transcı	Hps4

Rps6ka11	1.22E-109	0.391361	0.615	0.472	3.00E-105	MG-Transcı	Rps6ka1
Tpst21	4.95E-109	0.361228	0.647	0.499	1.21E-104	MG-Transcı	Tpst2
Tnfaip8l21	5.93E-109	0.451197	0.59	0.45	1.45E-104	MG-Transcı	Tnfaip8l2
Fermt31	1.63E-108	0.375045	0.691	0.552	3.99E-104	MG-Transcı	Fermt3
Lrrc31	2.13E-108	0.66508	0.289	0.17	5.21E-104	MG-Transcı	Lrrc3
Daglb1	4.54E-108	0.355219	0.656	0.499	1.11E-103	MG-Transcı	Daglb
Ppp1r10	1.66E-106	0.685812	0.327	0.207	4.08E-102	MG-Transcı	Ppp1r10
Fcgr11	2.40E-106	0.315513	0.736	0.564	5.89E-102	MG-Transcı	Fcgr1
Cd341	3.82E-106	0.652853	0.311	0.189	9.35E-102	MG-Transcı	Cd34
Inka11	3.97E-106	0.712425	0.302	0.185	9.71E-102	MG-Transcı	Inka1
Limd2	5.63E-106	0.345767	0.742	0.581	1.38E-101	MG-Transcı	Limd2
Cyfip12	2.44E-105	0.28599	0.814	0.683	5.96E-101	MG-Transcı	Cyfip1
Slc3a22	2.64E-105	0.367499	0.679	0.526	6.47E-101	MG-Transcı	Slc3a2
Ntpcr1	4.90E-105	0.375514	0.628	0.475	1.20E-100	MG-Transcı	Ntpcr
Ncf1	5.71E-104	0.372785	0.671	0.53	1.40E-99	MG-Transcı	Ncf1
Tnfaip31	5.85E-104	0.783438	0.305	0.193	1.43E-99	MG-Transcı	Tnfaip3
Tmem591	1.11E-103	0.303123	0.786	0.632	2.72E-99	MG-Transcı	Tmem59
Anxa32	9.54E-102	0.296534	0.744	0.579	2.34E-97	MG-Transcı	Anxa3
Arf4	1.62E-101	0.539775	0.564	0.439	3.96E-97	MG-Transcı	Arf4
Edem21	1.87E-101	0.47984	0.494	0.362	4.58E-97	MG-Transcı	Edem2
Gm433051	2.91E-101	0.516233	0.535	0.403	7.12E-97	MG-Transcı	Gm43305
Vkorc12	1.89E-100	0.441237	0.545	0.408	4.63E-96	MG-Transcı	Vkorc1
Cd300c22	2.12E-100	0.286219	0.874	0.709	5.18E-96	MG-Transcı	Cd300c2
Mfng1	3.90E-100	0.612934	0.327	0.209	9.55E-96	MG-Transcı	Mfng
Pros12	6.18E-100	0.312463	0.613	0.456	1.51E-95	MG-Transcı	Pros1
Pmp221	6.38E-100	0.401304	0.49	0.337	1.56E-95	MG-Transcı	Pmp22
Capn32	1.55E-98	0.301486	0.487	0.333	3.80E-94	MG-Transcı	Capn3
Pdia31	9.28E-98	0.277854	0.863	0.7	2.27E-93	MG-Transcı	Pdia3
Arl4c1	5.36E-97	0.609145	0.423	0.301	1.31E-92	MG-Transcı	Arl4c
Cryba42	5.74E-96	0.572506	0.382	0.258	1.41E-91	MG-Transcı	Cryba4
Tmx4	6.61E-96	0.796253	0.295	0.185	1.62E-91	MG-Transcı	Tmx4
Mcl1	1.69E-95	0.358687	0.667	0.525	4.14E-91	MG-Transcı	Mcl1
Tspan72	4.48E-95	0.322512	0.524	0.376	1.10E-90	MG-Transcı	Tspan7
Clic11	4.97E-91	0.277852	0.896	0.741	1.22E-86	MG-Transcı	Clic1
Slc46a11	1.05E-90	0.643463	0.306	0.196	2.56E-86	MG-Transcı	Slc46a1
Bmyc1	2.12E-90	0.446773	0.493	0.368	5.18E-86	MG-Transcı	Bmyc
Ppcdc2	2.75E-90	0.328488	0.52	0.377	6.74E-86	MG-Transcı	Ppcdc
Rogdi1	1.08E-89	0.425306	0.487	0.36	2.65E-85	MG-Transcı	Rogdi
Snn1	2.96E-89	0.545784	0.304	0.192	7.24E-85	MG-Transcı	Snn
Zfp7061	3.20E-89	0.352115	0.62	0.489	7.83E-85	MG-Transcı	Zfp706
Ypel31	1.18E-88	0.29086	0.707	0.565	2.88E-84	MG-Transcı	Ypel3
Tmbim61	7.47E-88	0.261657	0.904	0.764	1.83E-83	MG-Transcı	Tmbim6
Atraid1	1.31E-87	0.419543	0.509	0.386	3.22E-83	MG-Transcı	Atraid
Commd81	2.71E-87	0.492664	0.439	0.322	6.63E-83	MG-Transcı	Commd8

Tmem86a2	2.73E-87	0.349499	0.607	0.456	6.68E-83	MG-Transcı	Tmem86a
Rab3il12	3.32E-87	0.365999	0.538	0.406	8.14E-83	MG-Transcı	Rab3il1
Tmem371	6.60E-87	0.533201	0.386	0.268	1.62E-82	MG-Transcı	Tmem37
Gpr1832	1.19E-85	0.486732	0.458	0.33	2.91E-81	MG-Transcı	Gpr183
Mzt2	5.84E-85	0.683711	0.285	0.183	1.43E-80	MG-Transcı	Mzt2
Irf1	1.75E-84	0.741434	0.427	0.318	4.28E-80	MG-Transcı	Irf1
Cebpd1	1.04E-83	0.539594	0.388	0.269	2.55E-79	MG-Transcı	Cebpd
Wsb11	1.85E-83	0.384301	0.48	0.357	4.53E-79	MG-Transcı	Wsb1
Hspa5	2.15E-83	0.302763	0.768	0.632	5.27E-79	MG-Transcı	Hspa5
Ccl31	7.08E-83	1.125167	0.278	0.182	1.73E-78	MG-Transcı	Ccl3
Cd1641	7.63E-83	0.297222	0.562	0.427	1.87E-78	MG-Transcı	Cd164
Spint12	8.62E-83	0.413989	0.444	0.314	2.11E-78	MG-Transcı	Spint1
Tpp12	9.87E-83	0.341533	0.6	0.457	2.42E-78	MG-Transcı	Tpp1
H2-M31	1.11E-82	0.45134	0.517	0.405	2.71E-78	MG-Transcı	H2-M3
Skil1	2.07E-81	0.400793	0.452	0.333	5.08E-77	MG-Transcı	Skil
Rnf131	2.47E-81	0.26061	0.7	0.569	6.05E-77	MG-Transcı	Rnf13
Irf2bp2	5.93E-81	0.429321	0.551	0.437	1.45E-76	MG-Transcı	Irf2bp2
Klf6	1.39E-80	0.447011	0.478	0.359	3.39E-76	MG-Transcı	Klf6
Snx182	1.75E-80	0.337434	0.535	0.408	4.29E-76	MG-Transcı	Snx18
Ergic32	9.56E-80	0.347869	0.586	0.463	2.34E-75	MG-Transcı	Ergic3
Fmnl32	1.05E-78	0.252195	0.541	0.404	2.56E-74	MG-Transcı	Fmnl3
Efhd21	2.12E-78	0.299192	0.74	0.604	5.19E-74	MG-Transcı	Efhd2
Asah11	2.21E-78	0.26358	0.795	0.658	5.41E-74	MG-Transcı	Asah1
Slc35b21	1.19E-77	0.472798	0.432	0.323	2.91E-73	MG-Transcı	Slc35b2
Slc12a92	1.91E-77	0.416387	0.443	0.327	4.67E-73	MG-Transcı	Slc12a9
Papss11	7.77E-76	0.543861	0.331	0.227	1.90E-71	MG-Transcı	Papss1
Klf2	1.41E-75	0.9168	0.315	0.217	3.45E-71	MG-Transcı	Klf2
Sgce1	2.12E-75	0.40718	0.354	0.242	5.18E-71	MG-Transcı	Sgce
Cep85	2.56E-75	0.622876	0.385	0.28	6.26E-71	MG-Transcı	Cep85
Gnas1	7.98E-75	0.324755	0.825	0.717	1.95E-70	MG-Transcı	Gnas
Slc16a62	7.72E-74	0.311332	0.413	0.294	1.89E-69	MG-Transcı	Slc16a6
Ang1	1.23E-73	0.471581	0.414	0.303	3.02E-69	MG-Transcı	Ang
Cirbp1	2.51E-72	0.49088	0.383	0.278	6.14E-68	MG-Transcı	Cirbp
Rpn21	2.88E-72	0.291918	0.637	0.511	7.06E-68	MG-Transcı	Rpn2
Tmed51	3.05E-72	0.407858	0.476	0.368	7.47E-68	MG-Transcı	Tmed5
Rnaset2b1	5.37E-72	0.340537	0.538	0.417	1.32E-67	MG-Transcı	Rnaset2b
Rlim1	4.23E-71	0.597911	0.338	0.242	1.03E-66	MG-Transcı	Rlim
Rab141	1.59E-70	0.303988	0.659	0.547	3.90E-66	MG-Transcı	Rab14
Phgdh1	1.81E-70	0.549869	0.273	0.179	4.44E-66	MG-Transcı	Phgdh
Adam151	2.02E-69	0.367228	0.466	0.355	4.94E-65	MG-Transcı	Adam15
H1f0	2.77E-68	1.115249	0.297	0.211	6.77E-64	MG-Transcı	H1f0
Snx171	1.54E-67	0.441231	0.433	0.336	3.77E-63	MG-Transcı	Snx17
Tuba1a1	5.11E-67	0.49981	0.339	0.241	1.25E-62	MG-Transcı	Tuba1a
Slc29a11	7.77E-67	0.443147	0.368	0.268	1.90E-62	MG-Transcı	Slc29a1

Cyb561a31	1.03E-66	0.430301	0.393	0.288	2.51E-62	MG-Transcı	Cyb561a3
C3ar11	2.86E-66	0.31657	0.542	0.412	7.01E-62	MG-Transcı	C3ar1
Rgs11	1.35E-63	0.406931	0.329	0.227	3.30E-59	MG-Transcı	Rgs1
Sipa12	1.39E-63	0.349908	0.434	0.33	3.41E-59	MG-Transcı	Sipa1
Gpr841	3.69E-63	0.559589	0.307	0.212	9.04E-59	MG-Transcı	Gpr84
Mlxipl1	4.33E-62	0.343177	0.294	0.196	1.06E-57	MG-Transcı	Mlxipl
Eif4a11	9.01E-62	0.268966	0.696	0.561	2.21E-57	MG-Transcı	Eif4a1
Ptp4a31	1.68E-61	0.396015	0.373	0.276	4.11E-57	MG-Transcı	Ptp4a3
Slc29a32	4.44E-61	0.271295	0.565	0.45	1.09E-56	MG-Transcı	Slc29a3
Kcnk62	7.36E-61	0.364252	0.348	0.247	1.80E-56	MG-Transcı	Kcnk6
Mid1ip1	3.39E-60	0.53289	0.299	0.211	8.30E-56	MG-Transcı	Mid1ip1
Pdgfb2	3.90E-60	0.36117	0.361	0.261	9.54E-56	MG-Transcı	Pdgfb
Cd821	4.94E-60	0.348997	0.469	0.368	1.21E-55	MG-Transcı	Cd82
Pip4p11	7.81E-60	0.274617	0.556	0.452	1.91E-55	MG-Transcı	Pip4p1
Pcbp11	2.27E-59	0.289641	0.631	0.516	5.56E-55	MG-Transcı	Pcbp1
Atp6ap12	4.73E-59	0.278632	0.608	0.496	1.16E-54	MG-Transcı	Atp6ap1
Bcap311	8.96E-59	0.324966	0.54	0.438	2.19E-54	MG-Transcı	Bcap31
Plod31	3.04E-57	0.401141	0.413	0.319	7.45E-53	MG-Transcı	Plod3
Fez22	4.56E-57	0.389038	0.364	0.271	1.12E-52	MG-Transcı	Fez2
Klf31	5.35E-57	0.259396	0.509	0.403	1.31E-52	MG-Transcı	Klf3
Lpcat32	5.38E-57	0.317205	0.421	0.319	1.32E-52	MG-Transcı	Lpcat3
Tnfrsf1a1	1.14E-56	0.311422	0.542	0.441	2.78E-52	MG-Transcı	Tnfrsf1a
Arl102	2.37E-56	0.417024	0.324	0.234	5.82E-52	MG-Transcı	Arl10
Ociad11	4.20E-56	0.299015	0.539	0.441	1.03E-51	MG-Transcı	Ociad1
Med12l	5.75E-56	0.437797	0.259	0.174	1.41E-51	MG-Transcı	Med12l
Gna152	6.69E-56	0.26252	0.473	0.368	1.64E-51	MG-Transcı	Gna15
H2-Oa1	6.99E-56	0.334229	0.427	0.316	1.71E-51	MG-Transcı	H2-Oa
Paqr7	1.00E-55	0.553552	0.251	0.171	2.45E-51	MG-Transcı	Paqr7
Slc11a11	1.52E-55	0.250458	0.616	0.483	3.72E-51	MG-Transcı	Slc11a1
Selenos1	1.85E-55	0.364789	0.461	0.368	4.52E-51	MG-Transcı	Selenos
Tspan42	4.48E-55	0.415225	0.33	0.24	1.10E-50	MG-Transcı	Tspan4
Tmed91	1.29E-54	0.271258	0.571	0.47	3.16E-50	MG-Transcı	Tmed9
Tnfrsf13b	2.22E-54	0.516276	0.294	0.211	5.43E-50	MG-Transcı	Tnfrsf13b
Map112	4.22E-54	0.331375	0.422	0.327	1.03E-49	MG-Transcı	Map11
Plod11	5.46E-54	0.346092	0.39	0.295	1.34E-49	MG-Transcı	Plod1
Tecr1	1.21E-53	0.263922	0.564	0.464	2.98E-49	MG-Transcı	Tecr
Iffo12	1.41E-53	0.304762	0.326	0.232	3.45E-49	MG-Transcı	Iffo1
Rel	5.30E-53	0.390083	0.371	0.281	1.30E-48	MG-Transcı	Rel
Irf5	2.14E-52	0.266012	0.565	0.461	5.25E-48	MG-Transcı	Irf5
Arhgef401	5.76E-52	0.287522	0.258	0.171	1.41E-47	MG-Transcı	Arhgef40
Il21r2	9.54E-52	0.30466	0.467	0.367	2.34E-47	MG-Transcı	Il21r
Adprh1	2.15E-51	0.435832	0.38	0.297	5.26E-47	MG-Transcı	Adprh
Parvg1	2.71E-51	0.433391	0.333	0.248	6.63E-47	MG-Transcı	Parvg
Lmo21	3.66E-51	0.267872	0.548	0.446	8.97E-47	MG-Transcı	Lmo2

Zfand51	4.03E-51	0.340062	0.424	0.334	9.88E-47	MG-Transcı	Zfand5
Srsf91	4.61E-51	0.288143	0.559	0.464	1.13E-46	MG-Transcı	Srsf9
Rnf1671	1.23E-50	0.362283	0.347	0.259	3.01E-46	MG-Transcı	Rnf167
Rasal32	2.53E-50	0.358686	0.339	0.251	6.19E-46	MG-Transcı	Rasal3
Ppp1r18	4.63E-49	0.262626	0.563	0.465	1.13E-44	MG-Transcı	Ppp1r18
Khk	1.70E-48	0.486898	0.283	0.207	4.17E-44	MG-Transcı	Khk
Twf21	2.55E-48	0.303311	0.508	0.419	6.25E-44	MG-Transcı	Twf2
Abhd61	6.35E-48	0.401154	0.294	0.213	1.55E-43	MG-Transcı	Abhd6
Naglu2	7.73E-48	0.372796	0.378	0.291	1.89E-43	MG-Transcı	Naglu
H2-Q41	2.91E-47	0.313425	0.53	0.431	7.13E-43	MG-Transcı	H2-Q4
Leprot1	4.79E-47	0.391573	0.413	0.332	1.17E-42	MG-Transcı	Leprot
Cebpz2	7.99E-47	0.369273	0.365	0.283	1.96E-42	MG-Transcı	Cebpz
Nars1	1.06E-46	0.344094	0.413	0.328	2.60E-42	MG-Transcı	Nars
Cd3021	1.39E-46	0.326473	0.426	0.339	3.40E-42	MG-Transcı	Cd302
Idh21	4.82E-46	0.361257	0.373	0.29	1.18E-41	MG-Transcı	Idh2
Lpar62	6.28E-46	0.299511	0.37	0.28	1.54E-41	MG-Transcı	Lpar6
Tubb2a1	9.46E-46	0.38256	0.33	0.248	2.32E-41	MG-Transcı	Tubb2a
H2-Q71	9.56E-46	0.252237	0.584	0.473	2.34E-41	MG-Transcı	H2-Q7
Ccng21	1.76E-45	0.415296	0.252	0.177	4.32E-41	MG-Transcı	Ccng2
Nucb12	1.79E-45	0.291175	0.461	0.372	4.38E-41	MG-Transcı	Nucb1
Gatm1	3.95E-45	0.293235	0.486	0.385	9.66E-41	MG-Transcı	Gatm
Acp21	9.34E-45	0.295494	0.429	0.34	2.29E-40	MG-Transcı	Acp2
Akr1b101	1.40E-44	0.4243	0.303	0.227	3.43E-40	MG-Transcı	Akr1b10
B4galt41	2.68E-44	0.360581	0.271	0.194	6.55E-40	MG-Transcı	B4galt4
Tifa	2.82E-44	0.40558	0.347	0.269	6.91E-40	MG-Transcı	Tifa
Srsf21	2.89E-44	0.281066	0.526	0.439	7.07E-40	MG-Transcı	Srsf2
Evi2a1	5.20E-44	0.428303	0.362	0.285	1.27E-39	MG-Transcı	Evi2a
Smox2	1.64E-43	0.287548	0.359	0.276	4.01E-39	MG-Transcı	Smox
Os92	3.54E-43	0.257691	0.51	0.42	8.67E-39	MG-Transcı	Os9
Hhex	5.50E-43	0.494884	0.263	0.192	1.35E-38	MG-Transcı	Hhex
Ccnl11	2.29E-42	0.289845	0.419	0.338	5.62E-38	MG-Transcı	Ccnl1
Slc46a31	2.78E-42	0.324117	0.304	0.225	6.80E-38	MG-Transcı	Slc46a3
Tmem1091	3.34E-42	0.446302	0.306	0.235	8.19E-38	MG-Transcı	Tmem109
Cebpzos2	4.10E-42	0.361636	0.394	0.315	1.00E-37	MG-Transcı	Cebpzos
Rab11a	4.99E-42	0.27321	0.506	0.421	1.22E-37	MG-Transcı	Rab11a
Ube2j12	6.16E-42	0.34332	0.371	0.291	1.51E-37	MG-Transcı	Ube2j1
Sun21	1.02E-41	0.322264	0.332	0.252	2.51E-37	MG-Transcı	Sun2
Ncstn1	2.26E-41	0.27817	0.484	0.398	5.53E-37	MG-Transcı	Ncstn
Alg51	6.04E-41	0.437569	0.315	0.246	1.48E-36	MG-Transcı	Alg5
Rgmb2	1.39E-40	0.342108	0.321	0.244	3.41E-36	MG-Transcı	Rgmb
Pura1	1.62E-40	0.314708	0.406	0.328	3.96E-36	MG-Transcı	Pura
H2-Q6	1.68E-40	0.309764	0.459	0.376	4.11E-36	MG-Transcı	H2-Q6
Lysmd4	3.83E-40	0.36492	0.255	0.183	9.39E-36	MG-Transcı	Lysmd4
Pon32	6.26E-40	0.327071	0.33	0.252	1.53E-35	MG-Transcı	Pon3

Abhd17c	1.58E-39	0.340116	0.26	0.189	3.88E-35	MG-Transcı	Abhd17c
Ptpro1	2.83E-39	0.296442	0.405	0.322	6.92E-35	MG-Transcı	Ptpro
Ldb12	4.08E-39	0.275037	0.377	0.299	9.98E-35	MG-Transcı	Ldb1
St6galnac4	1.63E-38	0.331664	0.373	0.297	3.98E-34	MG-Transcı	St6galnac4
Emc101	2.59E-38	0.36742	0.408	0.336	6.34E-34	MG-Transcı	Emc10
Hvcn12	3.16E-38	0.30056	0.379	0.3	7.74E-34	MG-Transcı	Hvcn1
Lactb	3.70E-38	0.415504	0.256	0.189	9.07E-34	MG-Transcı	Lactb
Pttg1ip1	4.01E-38	0.332702	0.339	0.268	9.82E-34	MG-Transcı	Pttg1ip
Clcn4	4.39E-38	0.418406	0.273	0.206	1.07E-33	MG-Transcı	Clcn4
Rnaset2a1	5.29E-38	0.316312	0.408	0.33	1.30E-33	MG-Transcı	Rnaset2a
Mboat7	2.42E-37	0.42173	0.272	0.205	5.92E-33	MG-Transcı	Mboat7
Tox41	2.89E-37	0.414519	0.333	0.265	7.08E-33	MG-Transcı	Tox4
Ddx3x	6.25E-37	0.266525	0.36	0.288	1.53E-32	MG-Transcı	Ddx3x
Man2b22	1.02E-36	0.327085	0.368	0.293	2.50E-32	MG-Transcı	Man2b2
Cops41	1.14E-36	0.381904	0.299	0.233	2.78E-32	MG-Transcı	Cops4
Mgat12	4.85E-36	0.27754	0.345	0.271	1.19E-31	MG-Transcı	Mgat1
Xbp11	9.08E-36	0.312169	0.446	0.372	2.22E-31	MG-Transcı	Xbp1
Il161	9.09E-36	0.289737	0.332	0.259	2.23E-31	MG-Transcı	Il16
Ndfip22	9.50E-36	0.290715	0.329	0.257	2.33E-31	MG-Transcı	Ndfip2
Tm2d3	4.40E-35	0.388691	0.284	0.219	1.08E-30	MG-Transcı	Tm2d3
Emc71	1.12E-34	0.286454	0.417	0.347	2.75E-30	MG-Transcı	Emc7
Cnot81	1.20E-34	0.364066	0.32	0.255	2.95E-30	MG-Transcı	Cnot8
Acox3	1.28E-34	0.349082	0.272	0.205	3.12E-30	MG-Transcı	Acox3
Tmed71	2.21E-34	0.302525	0.389	0.321	5.42E-30	MG-Transcı	Tmed7
Chka	4.30E-34	0.406773	0.258	0.194	1.05E-29	MG-Transcı	Chka
Ebi3	1.47E-33	0.377821	0.335	0.269	3.60E-29	MG-Transcı	Ebi3
Naa352	1.74E-33	0.293556	0.315	0.247	4.26E-29	MG-Transcı	Naa35
Rgs191	4.26E-33	0.301082	0.397	0.329	1.04E-28	MG-Transcı	Rgs19
Gpr146	6.15E-33	0.38769	0.277	0.214	1.50E-28	MG-Transcı	Gpr146
Tspan311	1.19E-32	0.352619	0.349	0.285	2.90E-28	MG-Transcı	Tspan31
Eng1	2.43E-32	0.251529	0.276	0.208	5.96E-28	MG-Transcı	Eng
Derl11	2.59E-32	0.272597	0.42	0.353	6.35E-28	MG-Transcı	Derl1
Sh3bp1	2.82E-32	0.306452	0.373	0.306	6.92E-28	MG-Transcı	Sh3bp1
Slc50a11	3.51E-32	0.273113	0.388	0.319	8.59E-28	MG-Transcı	Slc50a1
Hsd17b4	5.05E-32	0.285548	0.318	0.252	1.24E-27	MG-Transcı	Hsd17b4
Asb21	1.00E-31	0.331494	0.265	0.2	2.46E-27	MG-Transcı	Asb2
Mafg1	1.62E-31	0.282757	0.307	0.241	3.96E-27	MG-Transcı	Mafg
0610010K1	1.65E-31	0.396411	0.313	0.253	4.04E-27	MG-Transcı	0610010K14Rik
Mcfd21	2.23E-31	0.329404	0.34	0.275	5.47E-27	MG-Transcı	Mcfd2
Dcakd1	3.20E-31	0.281093	0.311	0.246	7.85E-27	MG-Transcı	Dcakd
Pold41	3.56E-31	0.255894	0.44	0.365	8.71E-27	MG-Transcı	Pold4
Pdia42	6.00E-31	0.275923	0.386	0.317	1.47E-26	MG-Transcı	Pdia4
Ppt2	8.29E-31	0.393875	0.307	0.248	2.03E-26	MG-Transcı	Ppt2
Pdlim42	2.92E-30	0.300546	0.321	0.257	7.15E-26	MG-Transcı	Pdlim4

Nagpa1	3.88E-30	0.344555	0.32	0.26	9.49E-26	MG-Transcı	Nagpa
Usp211	6.22E-30	0.365526	0.251	0.192	1.52E-25	MG-Transcı	Usp21
Atf4	7.71E-30	0.473323	0.263	0.207	1.89E-25	MG-Transcı	Atf4
Tmco11	1.14E-29	0.288765	0.371	0.309	2.80E-25	MG-Transcı	Tmco1
Tmem331	2.92E-29	0.314961	0.331	0.271	7.14E-25	MG-Transcı	Tmem33
Polr2a	3.78E-29	0.341992	0.322	0.262	9.26E-25	MG-Transcı	Polr2a
Txndc151	7.39E-28	0.293246	0.345	0.286	1.81E-23	MG-Transcı	Txndc15
Lat21	4.37E-27	0.272564	0.372	0.308	1.07E-22	MG-Transcı	Lat2
Taz2	4.56E-27	0.298762	0.3	0.241	1.12E-22	MG-Transcı	Taz
Vps37b	7.85E-27	0.260047	0.258	0.2	1.92E-22	MG-Transcı	Vps37b
Lman11	8.03E-26	0.306212	0.296	0.24	1.97E-21	MG-Transcı	Lman1
Bcl10	9.04E-26	0.34431	0.314	0.259	2.21E-21	MG-Transcı	Bcl10
Tmem9b2	1.17E-25	0.29102	0.343	0.286	2.87E-21	MG-Transcı	Tmem9b
Lyl11	4.41E-25	0.305207	0.291	0.235	1.08E-20	MG-Transcı	Lyl1
Ctso	6.06E-25	0.294	0.257	0.202	1.48E-20	MG-Transcı	Ctso
Vmp11	1.23E-24	0.277513	0.328	0.274	3.02E-20	MG-Transcı	Vmp1
Unc501	2.25E-24	0.339328	0.32	0.269	5.51E-20	MG-Transcı	Unc50
Ddx501	2.62E-24	0.340168	0.295	0.242	6.41E-20	MG-Transcı	Ddx50
Rela	3.67E-24	0.367488	0.271	0.221	8.99E-20	MG-Transcı	Rela
Creb31	4.29E-24	0.378084	0.298	0.249	1.05E-19	MG-Transcı	Creb3
Slc15a4	3.57E-23	0.275961	0.276	0.224	8.75E-19	MG-Transcı	Slc15a4
Puf60	3.86E-23	0.261833	0.335	0.284	9.45E-19	MG-Transcı	Puf60
Trim81	4.65E-23	0.27915	0.281	0.23	1.14E-18	MG-Transcı	Trim8
Cited2	6.16E-23	0.476631	0.341	0.29	1.51E-18	MG-Transcı	Cited2
Ptov1	1.43E-22	0.256136	0.255	0.205	3.50E-18	MG-Transcı	Ptov1
Gps21	2.00E-22	0.27147	0.325	0.275	4.90E-18	MG-Transcı	Gps2
Ccdc47	2.66E-22	0.270594	0.268	0.217	6.52E-18	MG-Transcı	Ccdc47
Maf11	3.27E-22	0.333934	0.301	0.253	8.00E-18	MG-Transcı	Maf1
Rab5a1	8.53E-22	0.277758	0.282	0.232	2.09E-17	MG-Transcı	Rab5a
Cldnd11	3.44E-21	0.324139	0.266	0.218	8.42E-17	MG-Transcı	Cldnd1
Tmem128	5.42E-21	0.283932	0.329	0.28	1.33E-16	MG-Transcı	Tmem128
Cklf1	6.11E-21	0.284303	0.295	0.245	1.50E-16	MG-Transcı	Cklf
Ccdc107	8.57E-21	0.329687	0.262	0.215	2.10E-16	MG-Transcı	Ccdc107
Ythdf22	1.07E-20	0.257269	0.294	0.245	2.62E-16	MG-Transcı	Ythdf2
Srsf61	1.60E-20	0.251495	0.333	0.284	3.92E-16	MG-Transcı	Srsf6
Emc31	2.14E-20	0.250495	0.286	0.236	5.24E-16	MG-Transcı	Emc3
2510039O1	5.47E-19	0.277801	0.263	0.218	1.34E-14	MG-Transcı	2510039O18Rik
Cyp4v31	4.51E-18	0.278656	0.284	0.239	1.10E-13	MG-Transcı	Cyp4v3
Hnrnpa0	4.53E-18	0.262057	0.325	0.282	1.11E-13	MG-Transcı	Hnrnpa0
Yif1b2	9.52E-18	0.255115	0.315	0.271	2.33E-13	MG-Transcı	Yif1b
E330020D1	9.80E-17	0.294686	0.301	0.252	2.40E-12	MG-Transcı	E330020D12Rik
Tmem50b	1.49E-16	0.263845	0.28	0.237	3.64E-12	MG-Transcı	Tmem50b
Snhg8	3.01E-16	0.346213	0.251	0.213	7.38E-12	MG-Transcı	Snhg8
Ebp1	2.49E-13	0.271786	0.291	0.257	6.09E-09	MG-Transcı	Ebp

Cmtm31	5.30E-13	0.251749	0.323	0.287	1.30E-08	MG-Transc	Cmtm3
Mfsd51	9.46E-12	0.262072	0.284	0.253	2.32E-07	MG-Transc	Mfsd5
Lag33	0	0.852273	0.756	0.52	0	MG-Phago	Lag3
Ctsl3	0	0.666515	0.929	0.734	0	MG-Phago	Ctsl
Cd813	0	0.711434	0.978	0.793	0	MG-Phago	Cd81
Trem23	0	0.616447	0.967	0.783	0	MG-Phago	Trem2
C1qa3	0	0.627974	0.998	0.861	0	MG-Phago	C1qa
C1qc3	0	0.580435	0.997	0.863	0	MG-Phago	C1qc
Ctsz1	0	0.587296	0.977	0.844	0	MG-Phago	Ctsz
C1qb3	0	0.613158	0.999	0.879	0	MG-Phago	C1qb
Grn3	0	0.569089	0.961	0.844	0	MG-Phago	Grn
Rpl211	0	0.581773	0.979	0.883	0	MG-Phago	Rpl21
Ctsd2	0	0.656477	0.996	0.901	0	MG-Phago	Ctsd
Ctsb1	0	0.584806	0.987	0.897	0	MG-Phago	Ctsb
Tyrobp1	0	0.499609	0.995	0.908	0	MG-Phago	Tyrobp
B2m1	0	0.49358	0.983	0.901	0	MG-Phago	B2m
Rplp12	0	0.543651	0.989	0.913	0	MG-Phago	Rplp1
H2-D11	0	0.55087	0.972	0.897	0	MG-Phago	H2-D1
Ly863	0	0.517272	0.979	0.907	0	MG-Phago	Ly86
Ctss3	0	0.735695	0.992	0.927	0	MG-Phago	Ctss
Psap1	0	0.443061	0.987	0.942	0	MG-Phago	Psap
Itm2b3	0	0.592501	0.997	0.952	0	MG-Phago	Itm2b
Hexb2	0	0.479911	0.998	0.969	0	MG-Phago	Hexb
Fau1	2.08E-279	0.485511	0.996	0.934	5.10E-275	MG-Phago	Fau
Lgmn2	3.62E-271	0.443491	0.976	0.893	8.85E-267	MG-Phago	Lgmn
Rpl301	1.52E-270	0.505389	0.98	0.889	3.73E-266	MG-Phago	Rpl30
Cst32	5.33E-269	0.457984	1	0.986	1.31E-264	MG-Phago	Cst3
Rps121	4.24E-260	0.550565	0.963	0.859	1.04E-255	MG-Phago	Rps12
Fcgr33	1.43E-257	0.540427	0.91	0.768	3.50E-253	MG-Phago	Fcgr3
Rps4x1	1.71E-255	0.546421	0.978	0.87	4.19E-251	MG-Phago	Rps4x
Rpl27a1	8.66E-254	0.514913	0.98	0.881	2.12E-249	MG-Phago	Rpl27a
Rps111	5.24E-248	0.488504	0.983	0.887	1.28E-243	MG-Phago	Rps11
Eef1a11	1.74E-238	0.514849	0.996	0.938	4.26E-234	MG-Phago	Eef1a1
Rpl391	1.20E-237	0.521861	0.938	0.81	2.95E-233	MG-Phago	Rpl39
Cd93	6.66E-236	0.538052	0.91	0.735	1.63E-231	MG-Phago	Cd9
Rps292	4.26E-235	0.514087	0.951	0.845	1.04E-230	MG-Phago	Rps29
Rps201	9.71E-234	0.506689	0.976	0.872	2.38E-229	MG-Phago	Rps20
Lgals3bp1	2.29E-232	0.715959	0.727	0.516	5.60E-228	MG-Phago	Lgals3bp
Lamp11	6.34E-232	0.489351	0.932	0.813	1.55E-227	MG-Phago	Lamp1
Fcer1g1	1.96E-230	0.386845	0.994	0.896	4.80E-226	MG-Phago	Fcer1g
Rps211	3.91E-222	0.509261	0.938	0.823	9.57E-218	MG-Phago	Rps21
Rpl18a1	1.93E-221	0.470615	0.982	0.892	4.72E-217	MG-Phago	Rpl18a
Rps91	4.30E-220	0.453114	0.989	0.904	1.05E-215	MG-Phago	Rps9
Olfml32	1.18E-219	0.526671	0.873	0.673	2.89E-215	MG-Phago	Olfml3

Laptm52	4.52E-215	0.385638	0.954	0.893	1.11E-210	MG-Phago	Laptm5
Rpl35a1	1.57E-214	0.448153	0.972	0.876	3.85E-210	MG-Phago	Rpl35a
Rpl321	1.68E-213	0.49551	0.972	0.856	4.11E-209	MG-Phago	Rpl32
Rps27a1	7.57E-211	0.460759	0.985	0.9	1.85E-206	MG-Phago	Rps27a
Rps241	1.49E-206	0.414472	0.989	0.923	3.64E-202	MG-Phago	Rps24
Gng102	1.33E-204	0.648675	0.78	0.617	3.25E-200	MG-Phago	Gng10
Rpl37a1	9.17E-202	0.45924	0.955	0.844	2.25E-197	MG-Phago	Rpl37a
H2-Oa2	1.91E-199	1.105739	0.495	0.311	4.69E-195	MG-Phago	H2-Oa
Sparc2	6.22E-197	0.431393	0.924	0.732	1.52E-192	MG-Phago	Sparc
Rps101	1.70E-196	0.469985	0.977	0.883	4.16E-192	MG-Phago	Rps10
H2-K11	4.69E-196	0.477476	0.937	0.875	1.15E-191	MG-Phago	H2-K1
Rpl231	4.19E-195	0.465054	0.976	0.873	1.03E-190	MG-Phago	Rpl23
Crybb12	9.22E-195	0.708879	0.69	0.493	2.26E-190	MG-Phago	Crybb1
Rpl111	2.20E-194	0.464593	0.973	0.867	5.39E-190	MG-Phago	Rpl11
Ly6e	1.56E-190	0.405527	0.962	0.855	3.83E-186	MG-Phago	Ly6e
Serpine23	3.26E-190	0.581786	0.772	0.572	7.99E-186	MG-Phago	Serpine2
Rps231	1.09E-188	0.454747	0.956	0.845	2.67E-184	MG-Phago	Rps23
Rps261	8.18E-188	0.474733	0.941	0.81	2.00E-183	MG-Phago	Rps26
Rpl131	4.93E-187	0.410911	0.988	0.913	1.21E-182	MG-Phago	Rpl13
Selenop2	8.57E-186	0.478693	0.941	0.805	2.10E-181	MG-Phago	Selenop
Rps81	1.30E-185	0.437429	0.979	0.893	3.18E-181	MG-Phago	Rps8
Rpl371	3.92E-185	0.434212	0.947	0.842	9.60E-181	MG-Phago	Rpl37
Rpl10a1	6.06E-184	0.536986	0.936	0.804	1.48E-179	MG-Phago	Rpl10a
Rpl351	1.92E-181	0.484034	0.9	0.763	4.71E-177	MG-Phago	Rpl35
Rps15a1	9.01E-181	0.446404	0.961	0.846	2.21E-176	MG-Phago	Rps15a
Rps3a11	1.14E-175	0.442314	0.972	0.875	2.79E-171	MG-Phago	Rps3a1
Ctsa3	5.14E-172	0.461203	0.888	0.775	1.26E-167	MG-Phago	Ctsa
Npc21	2.60E-170	0.432798	0.946	0.797	6.36E-166	MG-Phago	Npc2
Rps71	1.97E-169	0.427613	0.965	0.857	4.82E-165	MG-Phago	Rps7
Rpl411	1.49E-168	0.375786	0.974	0.896	3.65E-164	MG-Phago	Rpl41
Rpl191	7.42E-168	0.425016	0.981	0.881	1.82E-163	MG-Phago	Rpl19
Ssr41	1.42E-167	0.619268	0.769	0.634	3.48E-163	MG-Phago	Ssr4
Aif11	5.13E-167	0.519365	0.844	0.69	1.26E-162	MG-Phago	Aif1
Rpl61	8.45E-167	0.476092	0.937	0.818	2.07E-162	MG-Phago	Rpl6
Pld42	4.73E-164	0.468384	0.852	0.744	1.16E-159	MG-Phago	Pld4
Rplp01	2.20E-160	0.427379	0.955	0.831	5.39E-156	MG-Phago	Rplp0
Man2b13	2.02E-157	0.516808	0.823	0.723	4.95E-153	MG-Phago	Man2b1
Ldhb3	7.49E-155	0.632004	0.694	0.525	1.83E-150	MG-Phago	Ldhb
Rps131	9.29E-154	0.413486	0.951	0.839	2.27E-149	MG-Phago	Rps13
Rpl261	4.29E-152	0.436247	0.935	0.818	1.05E-147	MG-Phago	Rpl26
Rpl33	4.96E-150	0.491137	0.914	0.782	1.22E-145	MG-Phago	Rpl3
Rpl341	1.26E-149	0.387073	0.944	0.834	3.08E-145	MG-Phago	Rpl34
Rgs102	1.46E-149	0.417462	0.897	0.796	3.58E-145	MG-Phago	Rgs10
Rps51	2.16E-149	0.402111	0.962	0.842	5.28E-145	MG-Phago	Rps5

Ctsh2	4.62E-148	0.423358	0.872	0.78	1.13E-143	MG-Phago	Ctsh
Cd631	1.86E-146	0.523809	0.79	0.607	4.56E-142	MG-Phago	Cd63
Itm2c3	1.45E-143	0.474617	0.794	0.677	3.55E-139	MG-Phago	Itm2c
Rpl291	1.89E-143	0.467311	0.916	0.789	4.63E-139	MG-Phago	Rpl29
Cd741	2.64E-142	0.578062	0.884	0.763	6.46E-138	MG-Phago	Cd74
Rps161	2.08E-138	0.380661	0.97	0.873	5.11E-134	MG-Phago	Rps16
Rpl121	1.43E-135	0.522305	0.825	0.685	3.51E-131	MG-Phago	Rpl12
Serinc32	3.47E-133	0.319254	0.93	0.895	8.51E-129	MG-Phago	Serinc3
Rps271	3.10E-132	0.39169	0.887	0.773	7.59E-128	MG-Phago	Rps27
Rpl91	5.13E-132	0.377299	0.962	0.867	1.26E-127	MG-Phago	Rpl9
Rpl221	1.68E-131	0.441502	0.867	0.736	4.11E-127	MG-Phago	Rpl22
Rpl13a1	2.52E-131	0.447711	0.899	0.783	6.18E-127	MG-Phago	Rpl13a
Rplp21	1.98E-130	0.408023	0.906	0.791	4.84E-126	MG-Phago	Rplp2
Rpl361	3.79E-130	0.397357	0.899	0.784	9.27E-126	MG-Phago	Rpl36
Cd683	1.61E-129	0.410075	0.869	0.748	3.93E-125	MG-Phago	Cd68
Trf2	2.33E-129	0.414186	0.845	0.725	5.71E-125	MG-Phago	Trf
Rps281	3.48E-128	0.400892	0.874	0.746	8.52E-124	MG-Phago	Rps28
Csf1r2	2.87E-127	0.285412	0.954	0.903	7.04E-123	MG-Phago	Csf1r
Rpl151	9.09E-127	0.441721	0.908	0.775	2.23E-122	MG-Phago	Rpl15
Tmem1192	1.05E-126	0.409708	0.79	0.628	2.57E-122	MG-Phago	Tmem119
Rpl36a1	4.56E-125	0.525251	0.77	0.641	1.12E-120	MG-Phago	Rpl36a
Rpl281	5.47E-125	0.369359	0.941	0.831	1.34E-120	MG-Phago	Rpl28
Timp21	8.78E-124	0.557243	0.744	0.589	2.15E-119	MG-Phago	Timp2
H2-T231	1.13E-120	0.501637	0.799	0.707	2.76E-116	MG-Phago	H2-T23
Rpl101	1.21E-120	0.368023	0.948	0.83	2.95E-116	MG-Phago	Rpl10
Rpl171	5.58E-117	0.373469	0.933	0.831	1.37E-112	MG-Phago	Rpl17
Apoe1	1.02E-116	0.408511	0.884	0.777	2.51E-112	MG-Phago	Apoe
Rpl7a1	1.46E-116	0.458301	0.879	0.755	3.57E-112	MG-Phago	Rpl7a
Unc93b12	3.40E-116	0.31589	0.91	0.86	8.34E-112	MG-Phago	Unc93b1
Rpl241	1.52E-114	0.424151	0.9	0.779	3.72E-110	MG-Phago	Rpl24
Cd300c23	7.28E-114	0.407623	0.824	0.719	1.78E-109	MG-Phago	Cd300c2
H2-Q72	2.83E-113	0.638395	0.603	0.473	6.94E-109	MG-Phago	H2-Q7
Rps31	1.63E-111	0.397923	0.934	0.819	4.00E-107	MG-Phago	Rps3
H2-Ab11	4.74E-109	0.528003	0.778	0.634	1.16E-104	MG-Phago	H2-Ab1
H2-DMa	1.08E-108	0.475365	0.778	0.657	2.64E-104	MG-Phago	H2-DMa
Rpl181	2.89E-106	0.318817	0.944	0.829	7.08E-102	MG-Phago	Rpl18
Rps181	9.31E-106	0.397088	0.886	0.749	2.28E-101	MG-Phago	Rps18
Rpl141	1.24E-105	0.448499	0.837	0.705	3.04E-101	MG-Phago	Rpl14
Selenof2	7.08E-103	0.509117	0.715	0.617	1.73E-98	MG-Phago	Selenof
Hexa2	1.12E-100	0.418151	0.817	0.717	2.75E-96	MG-Phago	Hexa
Rpl71	1.15E-100	0.437036	0.847	0.725	2.82E-96	MG-Phago	Rpl7
Rps22	2.71E-99	0.39165	0.928	0.806	6.64E-95	MG-Phago	Rps2
Rpsa1	9.70E-96	0.307284	0.941	0.823	2.38E-91	MG-Phago	Rpsa
Rps191	1.39E-95	0.345309	0.91	0.79	3.40E-91	MG-Phago	Rps19

H2-Aa1	1.61E-95	0.416324	0.751	0.602	3.93E-91	MG-Phago	H2-Aa
Ptgs13	3.50E-95	0.402831	0.74	0.616	8.56E-91	MG-Phago	Ptgs1
Selplg2	1.65E-93	0.274208	0.882	0.8	4.04E-89	MG-Phago	Selplg
Tpt11	7.01E-93	0.291676	0.976	0.889	1.72E-88	MG-Phago	Tpt1
H2-Eb11	8.25E-93	0.421263	0.724	0.582	2.02E-88	MG-Phago	H2-Eb1
Rpl271	1.09E-90	0.342669	0.883	0.767	2.66E-86	MG-Phago	Rpl27
Clta1	7.65E-90	0.368787	0.878	0.782	1.87E-85	MG-Phago	Clta
Ltc4s2	2.61E-89	0.379645	0.747	0.612	6.39E-85	MG-Phago	Ltc4s
Pycard2	2.89E-89	0.502373	0.672	0.562	7.07E-85	MG-Phago	Pycard
Siglech3	8.73E-88	0.27339	0.807	0.648	2.14E-83	MG-Phago	Siglech
Eef1b21	3.35E-87	0.424588	0.788	0.67	8.19E-83	MG-Phago	Eef1b2
Lpcat23	1.52E-86	0.297972	0.856	0.795	3.71E-82	MG-Phago	Lpcat2
Bsg2	1.70E-86	0.384795	0.761	0.671	4.17E-82	MG-Phago	Bsg
Syngr12	1.20E-85	0.571285	0.599	0.466	2.94E-81	MG-Phago	Syngr1
Atp5g21	8.11E-85	0.449981	0.788	0.679	1.99E-80	MG-Phago	Atp5g2
Cyba1	1.93E-84	0.271024	0.956	0.852	4.74E-80	MG-Phago	Cyba
Tmbim62	1.36E-83	0.323093	0.838	0.775	3.32E-79	MG-Phago	Tmbim6
Itgb53	8.61E-82	0.269278	0.873	0.786	2.11E-77	MG-Phago	Itgb5
Ppib1	1.81E-81	0.398084	0.752	0.667	4.44E-77	MG-Phago	Ppib
Ppfia43	1.69E-80	0.528886	0.591	0.487	4.14E-76	MG-Phago	Ppfia4
Fcgr2b1	4.38E-80	0.412165	0.738	0.647	1.07E-75	MG-Phago	Fcgr2b
Pfdn51	1.23E-79	0.433664	0.778	0.683	3.00E-75	MG-Phago	Pfdn5
Rps251	2.82E-78	0.457347	0.733	0.63	6.90E-74	MG-Phago	Rps25
Asah12	6.89E-77	0.382249	0.737	0.668	1.69E-72	MG-Phago	Asah1
Pdia32	1.41E-76	0.385436	0.785	0.712	3.44E-72	MG-Phago	Pdia3
Rpl81	1.58E-74	0.323025	0.904	0.784	3.87E-70	MG-Phago	Rpl8
Gal3st42	1.82E-74	0.539966	0.461	0.344	4.45E-70	MG-Phago	Gal3st4
Rnaset2b2	2.25E-74	0.56925	0.517	0.423	5.51E-70	MG-Phago	Rnaset2b
Creg11	3.58E-74	0.367344	0.698	0.591	8.76E-70	MG-Phago	Creg1
Cst71	1.43E-73	0.840275	0.284	0.183	3.51E-69	MG-Phago	Cst7
Ckb2	1.42E-71	0.359599	0.757	0.659	3.47E-67	MG-Phago	Ckb
Rps141	2.80E-71	0.301612	0.904	0.801	6.85E-67	MG-Phago	Rps14
Adora32	6.44E-71	0.619063	0.375	0.267	1.58E-66	MG-Phago	Adora3
H2-DMb1	1.82E-67	0.41004	0.641	0.526	4.46E-63	MG-Phago	H2-DMb1
Laptm4a2	9.54E-67	0.300853	0.794	0.731	2.34E-62	MG-Phago	Laptm4a
Tcn22	1.02E-66	0.43858	0.59	0.496	2.50E-62	MG-Phago	Tcn2
Cd372	2.01E-66	0.316077	0.733	0.642	4.91E-62	MG-Phago	Cd37
Ctsf2	2.48E-66	0.506461	0.489	0.386	6.06E-62	MG-Phago	Ctsf
Adgrg12	4.74E-66	0.393665	0.503	0.381	1.16E-61	MG-Phago	Adgrg1
Spint13	4.57E-65	0.603768	0.423	0.319	1.12E-60	MG-Phago	Spint1
Pld31	1.04E-64	0.514495	0.53	0.419	2.55E-60	MG-Phago	Pld3
Sdf41	2.71E-64	0.523307	0.586	0.507	6.63E-60	MG-Phago	Sdf4
Psme11	4.27E-64	0.329078	0.716	0.595	1.04E-59	MG-Phago	Psme1
Mpeg11	4.54E-64	0.337849	0.784	0.696	1.11E-59	MG-Phago	Mpeg1

Scamp22	1.15E-63	0.318618	0.759	0.705	2.82E-59	MG-Phago	Scamp2
Pabpc11	8.48E-62	0.467325	0.701	0.617	2.08E-57	MG-Phago	Pabpc1
Vkorc13	2.04E-61	0.606968	0.5	0.416	4.98E-57	MG-Phago	Vkorc1
Rpl381	2.14E-61	0.267422	0.796	0.693	5.24E-57	MG-Phago	Rpl38
Atp6v0c1	5.72E-61	0.261686	0.878	0.767	1.40E-56	MG-Phago	Atp6v0c
Tmem50a2	8.52E-61	0.331103	0.727	0.661	2.09E-56	MG-Phago	Tmem50a
Cox4i11	1.77E-60	0.341269	0.888	0.796	4.33E-56	MG-Phago	Cox4i1
Sdf2l12	2.33E-60	0.63096	0.481	0.394	5.70E-56	MG-Phago	Sdf2l1
Rps61	5.67E-60	0.364203	0.785	0.679	1.39E-55	MG-Phago	Rps6
Naca1	2.31E-59	0.344472	0.799	0.696	5.67E-55	MG-Phago	Naca
Fcgr12	5.05E-59	0.362136	0.664	0.577	1.24E-54	MG-Phago	Fcgr1
Rack11	5.64E-59	0.363867	0.757	0.65	1.38E-54	MG-Phago	Rack1
Uba521	8.42E-59	0.481261	0.547	0.454	2.06E-54	MG-Phago	Uba52
C4b1	5.74E-58	0.623764	0.32	0.224	1.41E-53	MG-Phago	C4b
Ergic33	9.32E-58	0.4822	0.548	0.471	2.28E-53	MG-Phago	Ergic3
Tmem592	6.02E-56	0.342264	0.699	0.645	1.47E-51	MG-Phago	Tmem59
Ddost2	5.67E-55	0.451493	0.568	0.494	1.39E-50	MG-Phago	Ddost
Rpn22	5.33E-54	0.42093	0.587	0.52	1.30E-49	MG-Phago	Rpn2
Cryba43	1.18E-53	0.646554	0.354	0.264	2.90E-49	MG-Phago	Cryba4
Eef1d1	8.81E-53	0.519362	0.562	0.49	2.16E-48	MG-Phago	Eef1d
Ifngr12	1.07E-52	0.253852	0.778	0.75	2.63E-48	MG-Phago	Ifngr1
Cd342	1.59E-48	0.695776	0.276	0.196	3.90E-44	MG-Phago	Cd34
Slc25a51	2.82E-48	0.385101	0.668	0.588	6.91E-44	MG-Phago	Slc25a5
H2-Q61	5.05E-47	0.46781	0.461	0.377	1.24E-42	MG-Phago	H2-Q6
Eif3f1	5.83E-47	0.405264	0.666	0.584	1.43E-42	MG-Phago	Eif3f
Eef21	6.09E-45	0.3342	0.806	0.714	1.49E-40	MG-Phago	Eef2
Rpl36al1	1.61E-44	0.310926	0.731	0.642	3.94E-40	MG-Phago	Rpl36al
Tpp13	4.93E-44	0.409575	0.544	0.467	1.21E-39	MG-Phago	Tpp1
Uqcrh1	1.09E-43	0.405603	0.659	0.595	2.67E-39	MG-Phago	Uqcrh
Tmem86a3	9.33E-43	0.386352	0.543	0.467	2.29E-38	MG-Phago	Tmem86a
Rnasek2	2.49E-41	0.392745	0.609	0.551	6.09E-37	MG-Phago	Rnasek
Sft2d12	2.81E-40	0.26714	0.683	0.639	6.88E-36	MG-Phago	Sft2d1
Nme21	3.89E-40	0.338915	0.691	0.594	9.54E-36	MG-Phago	Nme2
Cfl11	4.13E-40	0.319331	0.86	0.755	1.01E-35	MG-Phago	Cfl1
Erp292	5.76E-40	0.268369	0.743	0.687	1.41E-35	MG-Phago	Erp29
Glmp2	7.90E-39	0.443682	0.53	0.464	1.94E-34	MG-Phago	Glmp
Hsp90b11	9.11E-39	0.261213	0.781	0.739	2.23E-34	MG-Phago	Hsp90b1
Rnaset2a2	1.55E-37	0.482208	0.402	0.333	3.80E-33	MG-Phago	Rnaset2a
Lap31	1.13E-36	0.508588	0.423	0.357	2.77E-32	MG-Phago	Lap3
Spcs22	1.66E-36	0.388205	0.553	0.5	4.06E-32	MG-Phago	Spcs2
Daglb2	5.62E-36	0.294401	0.58	0.511	1.38E-31	MG-Phago	Daglb
Saraf2	6.80E-36	0.350765	0.554	0.498	1.67E-31	MG-Phago	Saraf
Tmem1732	8.97E-36	0.342111	0.547	0.487	2.20E-31	MG-Phago	Tmem173
Ywhah2	1.24E-35	0.292021	0.653	0.6	3.03E-31	MG-Phago	Ywhah

Rpl51	3.35E-35	0.337592	0.681	0.61	8.20E-31	MG-Phago	Rpl5
Hspe11	3.68E-35	0.499731	0.483	0.424	9.02E-31	MG-Phago	Hspe1
Pomp1	7.63E-34	0.364644	0.611	0.546	1.87E-29	MG-Phago	Pomp
Rps171	1.55E-33	0.343208	0.626	0.56	3.79E-29	MG-Phago	Rps17
Camk13	6.30E-33	0.277266	0.624	0.556	1.54E-28	MG-Phago	Camk1
Rpl42	7.32E-33	0.358411	0.668	0.594	1.79E-28	MG-Phago	Rpl4
Spcs12	1.78E-32	0.369514	0.558	0.505	4.36E-28	MG-Phago	Spcs1
Tspan32	1.88E-32	0.407679	0.452	0.391	4.61E-28	MG-Phago	Tspan3
Rpl311	3.72E-32	0.368567	0.586	0.525	9.10E-28	MG-Phago	Rpl31
Naglu3	7.32E-32	0.54778	0.356	0.296	1.79E-27	MG-Phago	Naglu
mt-Atp8	9.43E-32	0.28901	0.588	0.534	2.31E-27	MG-Phago	mt-Atp8
Hebp11	2.60E-31	0.692551	0.312	0.254	6.36E-27	MG-Phago	Hebp1
Grcc101	7.49E-31	0.330101	0.631	0.583	1.83E-26	MG-Phago	Grcc10
Limd21	8.12E-31	0.278363	0.652	0.595	1.99E-26	MG-Phago	Limd2
Npm11	1.41E-30	0.376108	0.611	0.543	3.46E-26	MG-Phago	Npm1
Fcgr41	3.02E-29	0.603849	0.298	0.239	7.40E-25	MG-Phago	Fcgr4
Tmed32	1.29E-28	0.469168	0.435	0.386	3.17E-24	MG-Phago	Tmed3
Gm2a1	3.73E-28	0.365544	0.495	0.436	9.13E-24	MG-Phago	Gm2a
Comt2	4.37E-28	0.25401	0.617	0.569	1.07E-23	MG-Phago	Comt
Tspan73	6.04E-28	0.311671	0.456	0.387	1.48E-23	MG-Phago	Tspan7
Bin22	7.78E-27	0.253068	0.631	0.582	1.91E-22	MG-Phago	Bin2
Ntpcr2	1.44E-26	0.312357	0.54	0.489	3.53E-22	MG-Phago	Ntpcr
Gabarap1	5.62E-26	0.250213	0.748	0.67	1.38E-21	MG-Phago	Gabarap
Slc3a23	2.72E-25	0.271106	0.59	0.54	6.65E-21	MG-Phago	Slc3a2
Rpl22l11	2.84E-25	0.392106	0.546	0.496	6.96E-21	MG-Phago	Rpl22l1
Pla2g152	4.51E-25	0.27972	0.519	0.462	1.10E-20	MG-Phago	Pla2g15
Aup11	4.57E-25	0.302264	0.572	0.536	1.12E-20	MG-Phago	Aup1
Cebpzos3	6.36E-25	0.512122	0.369	0.32	1.56E-20	MG-Phago	Cebpzos
Slc11a12	1.26E-24	0.297202	0.552	0.494	3.09E-20	MG-Phago	Slc11a1
Lgals91	6.71E-24	0.278046	0.594	0.542	1.64E-19	MG-Phago	Lgals9
Slc35b22	5.07E-23	0.471677	0.376	0.331	1.24E-18	MG-Phago	Slc35b2
Mrpl521	4.16E-22	0.326949	0.515	0.471	1.02E-17	MG-Phago	Mrpl52
Tmed92	5.90E-22	0.308219	0.513	0.479	1.45E-17	MG-Phago	Tmed9
Tnfaip8l22	6.05E-22	0.328333	0.502	0.463	1.48E-17	MG-Phago	Tnfaip8l2
Ptms1	7.41E-22	0.355267	0.566	0.521	1.81E-17	MG-Phago	Ptms
H2-Q42	8.15E-22	0.311765	0.487	0.438	2.00E-17	MG-Phago	H2-Q4
H2-M32	1.22E-21	0.322009	0.456	0.414	2.99E-17	MG-Phago	H2-M3
Tubb51	1.26E-21	0.361176	0.516	0.467	3.10E-17	MG-Phago	Tubb5
Rps151	3.95E-21	0.275199	0.616	0.566	9.68E-17	MG-Phago	Rps15
Nsa21	7.24E-21	0.419418	0.489	0.447	1.77E-16	MG-Phago	Nsa2
Os93	1.05E-20	0.345624	0.463	0.427	2.58E-16	MG-Phago	Os9
Atp6v1f1	1.35E-20	0.331684	0.601	0.557	3.30E-16	MG-Phago	Atp6v1f
Krtcap21	1.49E-20	0.355605	0.48	0.441	3.64E-16	MG-Phago	Krtcap2
Dad12	3.84E-20	0.322027	0.564	0.533	9.39E-16	MG-Phago	Dad1

Atp6ap13	4.21E-20	0.290493	0.542	0.507	1.03E-15	MG-Phago	Atp6ap1
Gpr842	8.11E-20	0.358048	0.269	0.218	1.99E-15	MG-Phago	Gpr84
Nucb13	9.59E-20	0.392271	0.418	0.379	2.35E-15	MG-Phago	Nucb1
Ctc12	1.01E-19	0.302656	0.386	0.335	2.48E-15	MG-Phago	Ctc1
Gusb2	2.00E-19	0.306968	0.533	0.497	4.91E-15	MG-Phago	Gusb
Man2b23	2.32E-19	0.482219	0.337	0.298	5.68E-15	MG-Phago	Man2b2
Ppp1r14b2	2.75E-19	0.53115	0.406	0.371	6.74E-15	MG-Phago	Ppp1r14b
Pold42	5.21E-19	0.450948	0.407	0.371	1.28E-14	MG-Phago	Pold4
Gm118081	3.54E-18	0.506174	0.341	0.305	8.67E-14	MG-Phago	Gm11808
Atraid2	6.19E-18	0.32058	0.434	0.397	1.52E-13	MG-Phago	Atraid
Tmco12	1.22E-17	0.405558	0.351	0.313	2.99E-13	MG-Phago	Tmco1
Mif1	1.32E-17	0.316968	0.482	0.433	3.22E-13	MG-Phago	Mif
Psenen1	2.23E-16	0.326998	0.524	0.503	5.46E-12	MG-Phago	Psenen
Slc25a31	2.32E-16	0.272234	0.648	0.61	5.67E-12	MG-Phago	Slc25a3
P4hb2	6.48E-16	0.272198	0.52	0.489	1.59E-11	MG-Phago	P4hb
Ccni1	1.22E-15	0.352471	0.446	0.418	2.99E-11	MG-Phago	Ccni
Cyb561a32	1.51E-15	0.335484	0.337	0.297	3.70E-11	MG-Phago	Cyb561a3
Ndufc21	1.60E-15	0.281304	0.532	0.504	3.93E-11	MG-Phago	Ndufc2
Cmtm62	2.19E-15	0.262974	0.485	0.453	5.37E-11	MG-Phago	Cmtm6
Ndufa6	3.21E-15	0.287665	0.551	0.521	7.86E-11	MG-Phago	Ndufa6
Rogdi2	6.40E-15	0.320792	0.406	0.372	1.57E-10	MG-Phago	Rogdi
Sec11c1	9.98E-15	0.266296	0.502	0.479	2.44E-10	MG-Phago	Sec11c
Dnase2a2	1.06E-14	0.310573	0.42	0.386	2.60E-10	MG-Phago	Dnase2a
Snrpg	1.08E-14	0.336786	0.458	0.429	2.64E-10	MG-Phago	Snrpg
Ndufa4	1.72E-14	0.254744	0.57	0.543	4.21E-10	MG-Phago	Ndufa4
Pdia61	2.23E-14	0.259273	0.523	0.498	5.46E-10	MG-Phago	Pdia6
Srsf92	2.36E-14	0.287788	0.496	0.473	5.78E-10	MG-Phago	Srsf9
Lat22	2.63E-14	0.421272	0.344	0.313	6.43E-10	MG-Phago	Lat2
Cox7a2l1	3.20E-14	0.352384	0.48	0.458	7.83E-10	MG-Phago	Cox7a2l
Wdr83os1	4.73E-14	0.423947	0.41	0.389	1.16E-09	MG-Phago	Wdr83os
Higd2a2	7.31E-14	0.396202	0.435	0.415	1.79E-09	MG-Phago	Higd2a
Tmem14c2	8.27E-14	0.334416	0.463	0.442	2.03E-09	MG-Phago	Tmem14c
Bcap312	2.50E-13	0.302599	0.467	0.448	6.12E-09	MG-Phago	Bcap31
Fuca12	2.61E-13	0.314159	0.456	0.432	6.40E-09	MG-Phago	Fuca1
Zfp7062	3.58E-13	0.255175	0.523	0.503	8.76E-09	MG-Phago	Zfp706
Ang2	4.76E-13	0.278386	0.351	0.313	1.17E-08	MG-Phago	Ang
Parvg2	7.14E-13	0.39707	0.289	0.255	1.75E-08	MG-Phago	Parvg
Atp6ap22	1.64E-12	0.270958	0.502	0.478	4.02E-08	MG-Phago	Atp6ap2
Pon33	1.72E-12	0.292606	0.294	0.258	4.21E-08	MG-Phago	Pon3
Mcfd22	1.88E-12	0.401392	0.31	0.28	4.61E-08	MG-Phago	Mcfd2
Ociad12	3.03E-12	0.251276	0.473	0.451	7.42E-08	MG-Phago	Ociad1
Edem22	3.22E-12	0.30871	0.402	0.376	7.89E-08	MG-Phago	Edem2
Bmyc2	3.57E-12	0.312249	0.407	0.381	8.74E-08	MG-Phago	Bmyc
Rer11	5.40E-12	0.313075	0.434	0.415	1.32E-07	MG-Phago	Rer1

Adam152	6.32E-12	0.294858	0.394	0.366	1.55E-07	MG-Phago	Adam15
Brk1	6.35E-12	0.309039	0.502	0.487	1.56E-07	MG-Phago	Brk1
Ncstn2	5.79E-11	0.295033	0.426	0.407	1.42E-06	MG-Phago	Ncstn
Slc50a12	6.97E-11	0.351074	0.348	0.325	1.71E-06	MG-Phago	Slc50a1
Ddrgk11	8.90E-11	0.294101	0.419	0.4	2.18E-06	MG-Phago	Ddrgk1
Tmem179b	9.60E-11	0.306385	0.435	0.418	2.35E-06	MG-Phago	Tmem179b
Atp5mpl1	1.17E-10	0.313623	0.443	0.419	2.87E-06	MG-Phago	Atp5mpl
Rtraf1	3.80E-10	0.31139	0.441	0.42	9.30E-06	MG-Phago	Rtraf
Tmem9b3	1.05E-09	0.432265	0.311	0.291	2.58E-05	MG-Phago	Tmem9b
Abhd62	1.05E-09	0.307153	0.25	0.22	2.58E-05	MG-Phago	Abhd6
Eif3h1	1.25E-09	0.274698	0.508	0.488	3.06E-05	MG-Phago	Eif3h
Tmem372	2.49E-09	0.325907	0.307	0.28	6.10E-05	MG-Phago	Tmem37
Pdia43	2.52E-09	0.306739	0.347	0.323	6.17E-05	MG-Phago	Pdia4
Park71	3.35E-09	0.367516	0.421	0.412	8.20E-05	MG-Phago	Park7
Alg52	4.53E-09	0.443394	0.273	0.253	0.000111	MG-Phago	Alg5
Selenos2	9.78E-09	0.283384	0.393	0.378	0.000239	MG-Phago	Selenos
Rpn11	1.17E-08	0.332993	0.376	0.362	0.000286	MG-Phago	Rpn1
Hvcn13	1.56E-08	0.274775	0.331	0.307	0.000383	MG-Phago	Hvcn1
Fkbp21	2.23E-08	0.269493	0.422	0.407	0.000547	MG-Phago	Fkbp2
Cuta1	2.60E-08	0.343577	0.418	0.409	0.000636	MG-Phago	Cuta
St6galnac4	3.64E-08	0.320638	0.325	0.305	0.000893	MG-Phago	St6galnac4
Leprot2	5.94E-08	0.315231	0.356	0.341	0.001454	MG-Phago	Leprot
Tspan312	1.49E-07	0.351389	0.307	0.292	0.003637	MG-Phago	Tspan31
Rnf71	1.90E-07	0.347944	0.383	0.377	0.004661	MG-Phago	Rnf7
Lrpap12	2.78E-07	0.317063	0.36	0.344	0.00681	MG-Phago	Lrpap1
Plbd21	3.66E-07	0.41581	0.285	0.27	0.008958	MG-Phago	Plbd2
Akr1b102	3.94E-07	0.282876	0.257	0.234	0.009641	MG-Phago	Akr1b10
Pon21	5.32E-07	0.295546	0.332	0.317	0.013017	MG-Phago	Pon2
H2-T221	9.45E-07	0.293069	0.362	0.347	0.023137	MG-Phago	H2-T22
Yif1b3	1.45E-06	0.401711	0.288	0.275	0.035535	MG-Phago	Yif1b
Cnpy22	1.62E-06	0.324345	0.323	0.311	0.039777	MG-Phago	Cnpy2
Nagpa2	1.67E-06	0.354985	0.28	0.266	0.040777	MG-Phago	Nagpa
Ggh1	1.83E-06	0.393175	0.291	0.277	0.044783	MG-Phago	Ggh
Kdelr11	2.29E-06	0.302161	0.353	0.345	0.056136	MG-Phago	Kdelr1
Akr1b31	2.70E-06	0.407819	0.327	0.318	0.066048	MG-Phago	Akr1b3
Nudt91	3.02E-06	0.352119	0.278	0.265	0.074003	MG-Phago	Nudt9
Hsbp1	3.77E-06	0.317698	0.416	0.414	0.092407	MG-Phago	Hsbp1
Vegfb1	5.48E-06	0.422835	0.28	0.266	0.134112	MG-Phago	Vegfb
Emc102	7.45E-06	0.280593	0.351	0.344	0.182546	MG-Phago	Emc10
Arl12	1.37E-05	0.411862	0.255	0.243	0.334437	MG-Phago	Arl1
Hmgn11	1.44E-05	0.348508	0.338	0.329	0.35304	MG-Phago	Hmgn1
Clptm11	1.62E-05	0.259395	0.335	0.324	0.396135	MG-Phago	Clptm1
Tspan43	1.88E-05	0.292711	0.266	0.25	0.460271	MG-Phago	Tspan4
Tmed72	3.24E-05	0.278322	0.336	0.329	0.79329	MG-Phago	Tmed7

Tmem332	3.51E-05	0.313164	0.288	0.278	0.859488	MG-Phago	Tmem33
Mydgf2	5.23E-05	0.400503	0.277	0.269	1	MG-Phago	Mydgf
Gde11	5.43E-05	0.415017	0.257	0.246	1	MG-Phago	Gde1
Map1lc3a	9.36E-05	0.358381	0.266	0.255	1	MG-Phago	Map1lc3a
Papss12	0.000122	0.312989	0.251	0.239	1	MG-Phago	Papss1
BC004004	0.000139	0.333322	0.304	0.301	1	MG-Phago	BC004004
Gaa1	0.000168	0.363951	0.255	0.244	1	MG-Phago	Gaa
Fam173a1	0.000206	0.300985	0.342	0.341	1	MG-Phago	Fam173a
Tmem1092	0.000214	0.331484	0.252	0.242	1	MG-Phago	Tmem109
Txndc152	0.00022	0.256951	0.3	0.292	1	MG-Phago	Txndc15
Ostc1	0.000313	0.294227	0.337	0.336	1	MG-Phago	Ostc
Mrfap1	0.000335	0.298409	0.421	0.427	1	MG-Phago	Mrfap1
Tmem50b1	0.000627	0.316071	0.25	0.242	1	MG-Phago	Tmem50b
Gltp1	0.000659	0.331086	0.382	0.382	1	MG-Phago	Gltp
Aph1a1	0.001153	0.384702	0.297	0.299	1	MG-Phago	Aph1a
Ltbr1	0.001171	0.352841	0.255	0.25	1	MG-Phago	Ltbr
Ndufb51	0.001268	0.293902	0.373	0.376	1	MG-Phago	Ndufb5
Fuca21	0.001346	0.362874	0.258	0.254	1	MG-Phago	Fuca2
Tex2611	0.003174	0.384149	0.255	0.253	1	MG-Phago	Tex261
Slc35c21	0.003228	0.254985	0.28	0.276	1	MG-Phago	Slc35c2
Ppt21	0.005435	0.272882	0.258	0.255	1	MG-Phago	Ppt2
Dnajb11	0.009114	0.291207	0.288	0.288	1	MG-Phago	Dnajb11
Cybb	0	2.783781	0.927	0.197	0	Classical M	Cybb
Plbd1	0	3.132173	0.809	0.083	0	Classical M	Plbd1
Ms4a6c	0	3.003593	0.93	0.247	0	Classical M	Ms4a6c
Tgfbi	0	3.307842	0.835	0.168	0	Classical M	Tgfbi
Fxyd5	0	2.273162	0.923	0.258	0	Classical M	Fxyd5
Klra2	0	3.855956	0.69	0.042	0	Classical M	Klra2
Vim	0	2.403955	0.747	0.168	0	Classical M	Vim
Clec12a	0	3.742212	0.624	0.053	0	Classical M	Clec12a
Ccr2	0	4.413561	0.605	0.038	0	Classical M	Ccr2
Lilrb4a1	0	2.286716	0.706	0.139	0	Classical M	Lilrb4a
Lgals1	0	2.240508	0.686	0.136	0	Classical M	Lgals1
Ms4a4c	0	3.814429	0.627	0.083	0	Classical M	Ms4a4c
Emp3	0	2.196731	0.728	0.194	0	Classical M	Emp3
Cyp4f18	0	3.336454	0.603	0.073	0	Classical M	Cyp4f18
Irf7	0	2.945077	0.689	0.164	0	Classical M	Irf7
Anxa51	0	1.771644	0.801	0.278	0	Classical M	Anxa5
Ifitm3	0	2.51661	0.922	0.404	0	Classical M	Ifitm3
Mndal	0	3.304299	0.574	0.06	0	Classical M	Mndal
Lgals31	0	2.290446	0.676	0.164	0	Classical M	Lgals3
AW112010	0	2.820561	0.773	0.262	0	Classical M	AW112010
Arhgap15	0	1.138469	0.76	0.253	0	Classical M	Arhgap15
Ifi207	0	2.015186	0.656	0.152	0	Classical M	Ifi207

Apobec1	0	2.054016	0.857	0.354	0	Classical M	Apobec1
Iqgap1	0	1.786868	0.643	0.142	0	Classical M	Iqgap1
Ifi27l2a1	0	2.610848	0.861	0.36	0	Classical M	Ifi27l2a
Axl1	0	1.858718	0.739	0.241	0	Classical M	Axl
Ms4a7	0	2.509361	0.587	0.089	0	Classical M	Ms4a7
Ifitm2	0	1.757602	0.68	0.184	0	Classical M	Ifitm2
Cxcl161	0	2.028178	0.771	0.276	0	Classical M	Cxcl16
Ms4a6b	0	1.792587	0.759	0.272	0	Classical M	Ms4a6b
Ifi47	0	3.277372	0.569	0.084	0	Classical M	Ifi47
Ifi203	0	3.311283	0.526	0.052	0	Classical M	Ifi203
Slamf7	0	2.62485	0.556	0.091	0	Classical M	Slamf7
Zbp11	0	2.565437	0.614	0.156	0	Classical M	Zbp1
AB1246111	0	2.121224	0.596	0.144	0	Classical M	AB124611
Oasl2	0	2.120996	0.617	0.169	0	Classical M	Oasl2
Slfn2	0	1.682077	0.718	0.273	0	Classical M	Slfn2
Clic4	0	1.674496	0.632	0.19	0	Classical M	Clic4
Trps1	0	1.470935	0.586	0.146	0	Classical M	Trps1
Gm36161	0	3.663732	0.472	0.035	0	Classical M	Gm36161
Aoah	0	1.898829	0.542	0.106	0	Classical M	Aoah
Msrb1	0	1.368356	0.606	0.17	0	Classical M	Msrb1
Pla2g71	0	2.082209	0.575	0.139	0	Classical M	Pla2g7
Itga4	0	1.869101	0.517	0.091	0	Classical M	Itga4
Tspo1	0	1.642663	0.851	0.428	0	Classical M	Tspo
S100a11	0	0.355708	0.551	0.131	0	Classical M	S100a11
Tagln2	0	1.645574	0.612	0.193	0	Classical M	Tagln2
Slfn5	0	2.585362	0.528	0.112	0	Classical M	Slfn5
Samhd1	0	1.815889	0.787	0.372	0	Classical M	Samhd1
Wfdc17	0	1.78437	0.512	0.097	0	Classical M	Wfdc17
Crip1	0	1.94037	0.578	0.163	0	Classical M	Crip1
Gm21188	0	3.311023	0.45	0.037	0	Classical M	Gm21188
Txn1	0	1.394418	0.716	0.304	0	Classical M	Txn1
Calhm6	0	2.803504	0.569	0.158	0	Classical M	Calhm6
Gsn	0	1.81278	0.514	0.106	0	Classical M	Gsn
Clec2d	0	1.259836	0.65	0.245	0	Classical M	Clec2d
Isg15	0	2.609191	0.588	0.183	0	Classical M	Isg15
Il2rg1	0	1.815616	0.605	0.201	0	Classical M	Il2rg
CAAA0114	0	1.153599	0.784	0.382	0	Classical M	CAAA01147332.1
S100a6	0	1.152938	0.497	0.102	0	Classical M	S100a6
Rbms1	0	1.233954	0.6	0.209	0	Classical M	Rbms1
Selenow1	0	1.035846	0.83	0.442	0	Classical M	Selenow
Ms4a6d	0	1.474488	0.707	0.32	0	Classical M	Ms4a6d
Slfn1	0	3.345174	0.428	0.042	0	Classical M	Slfn1
Cstb1	0	1.536397	0.749	0.365	0	Classical M	Cstb
Pltp	0	2.085056	0.503	0.121	0	Classical M	Pltp

Mmp141	0	2.007779	0.555	0.174	0	Classical M	Mmp14
Fgl21	0	1.478916	0.631	0.254	0	Classical M	Fgl2
Rtp4	0	1.440425	0.606	0.229	0	Classical M	Rtp4
H2-DMb11	0	1.514731	0.893	0.516	0	Classical M	H2-DMb1
Bst21	0	1.278839	0.942	0.573	0	Classical M	Bst2
Ly6c2	0	4.62152	0.404	0.04	0	Classical M	Ly6c2
Rnf213	0	1.229608	0.689	0.325	0	Classical M	Rnf213
Ifi213	0	2.509746	0.442	0.079	0	Classical M	Ifi213
Crlf21	0	1.069763	0.623	0.26	0	Classical M	Crlf2
Actr3	0	1.200956	0.901	0.539	0	Classical M	Actr3
Scimp	0	2.697537	0.437	0.075	0	Classical M	Scimp
Ly6a1	0	2.513597	0.604	0.242	0	Classical M	Ly6a
Ciita	0	1.95761	0.455	0.093	0	Classical M	Ciita
Ifi211	0	2.981483	0.43	0.069	0	Classical M	Ifi211
Oas1a1	0	2.043604	0.498	0.14	0	Classical M	Oas1a
Pirb	0	1.763159	0.493	0.136	0	Classical M	Pirb
Psmb101	0	1.296443	0.722	0.365	0	Classical M	Psmb10
Sp100	0	1.218852	0.614	0.26	0	Classical M	Sp100
Cfb1	0	3.593122	0.441	0.087	0	Classical M	Cfb
C31	0	2.616441	0.482	0.128	0	Classical M	C3
Tmem106a	0	1.255267	0.563	0.212	0	Classical M	Tmem106a
Ms4a4a	0	2.318297	0.415	0.064	0	Classical M	Ms4a4a
Calm1	0	1.055899	0.925	0.576	0	Classical M	Calm1
Gpr65	0	2.61455	0.42	0.071	0	Classical M	Gpr65
Myof	0	2.916501	0.383	0.036	0	Classical M	Myof
Sh3bgrl	0	1.61457	0.463	0.118	0	Classical M	Sh3bgrl
Ifi204	0	1.762966	0.503	0.159	0	Classical M	Ifi204
Prdx5	0	1.861205	0.915	0.572	0	Classical M	Prdx5
Prdx11	0	0.76118	0.873	0.531	0	Classical M	Prdx1
Capg1	0	2.404979	0.466	0.125	0	Classical M	Capg
Psmb81	0	1.181822	0.921	0.584	0	Classical M	Psmb8
Psme21	0	1.214525	0.841	0.504	0	Classical M	Psme2
Sem1	0	1.003496	0.871	0.535	0	Classical M	Sem1
Ass1	0	3.550226	0.377	0.042	0	Classical M	Ass1
H2-Eb12	0	1.83846	0.91	0.577	0	Classical M	H2-Eb1
Sdc31	0	1.195839	0.574	0.241	0	Classical M	Sdc3
Ybx11	0	0.91442	0.886	0.556	0	Classical M	Ybx1
Lyz21	0	2.736069	0.993	0.663	0	Classical M	Lyz2
Gm34084	0	3.507437	0.36	0.03	0	Classical M	Gm34084
Ifi209	0	2.441544	0.426	0.097	0	Classical M	Ifi209
Pde7b	0	2.648193	0.369	0.041	0	Classical M	Pde7b
Igsf6	0	1.210513	0.537	0.211	0	Classical M	Igsf6
H2-Aa2	0	1.759455	0.925	0.599	0	Classical M	H2-Aa
Mcub	0	3.082977	0.358	0.037	0	Classical M	Mcub

Metrnl	0	2.146724	0.391	0.07	0	Classical M	Metrnl
Dse	0	1.114457	0.468	0.148	0	Classical M	Dse
Cd40	0	3.049279	0.367	0.047	0	Classical M	Cd40
H2afz	0	0.902025	0.878	0.558	0	Classical M	H2afz
B3gnt8	0	3.268442	0.356	0.037	0	Classical M	B3gnt8
Runx3	0	3.578718	0.349	0.031	0	Classical M	Runx3
Apoc2	0	2.645277	0.379	0.062	0	Classical M	Apoc2
Plac8	0	3.871845	0.391	0.075	0	Classical M	Plac8
Phf11b	0	1.885749	0.459	0.144	0	Classical M	Phf11b
Tmem176a	0	1.38027	0.749	0.434	0	Classical M	Tmem176a
Slamf91	0	1.56257	0.508	0.194	0	Classical M	Slamf9
Ahnak	0	1.938229	0.405	0.092	0	Classical M	Ahnak
Xaf1	0	1.432948	0.493	0.18	0	Classical M	Xaf1
Cd521	0	1.443137	0.972	0.659	0	Classical M	Cd52
Clec4a1	0	2.146194	0.385	0.076	0	Classical M	Clec4a1
Anxa41	0	1.757527	0.446	0.138	0	Classical M	Anxa4
Sirpb1c	0	3.635241	0.323	0.017	0	Classical M	Sirpb1c
Mdfic	0	1.599009	0.398	0.093	0	Classical M	Mdfic
Atox11	0	0.995848	0.884	0.579	0	Classical M	Atox1
Gbp21	0	2.177132	0.491	0.188	0	Classical M	Gbp2
Pim1	0	1.630314	0.433	0.131	0	Classical M	Pim1
Rack12	0	0.924501	0.945	0.643	0	Classical M	Rack1
Mrc1	0	0.342708	0.453	0.152	0	Classical M	Mrc1
Ifi301	0	1.562298	0.882	0.582	0	Classical M	Ifi30
H2-Ab12	0	1.711298	0.932	0.632	0	Classical M	H2-Ab1
Sap30	0	1.695057	0.411	0.113	0	Classical M	Sap30
Tmsb10	0	1.69682	0.918	0.623	0	Classical M	Tmsb10
Nampt	0	1.424412	0.478	0.183	0	Classical M	Nampt
Sema4a	0	1.876798	0.391	0.097	0	Classical M	Sema4a
Xdh	0	1.671925	0.383	0.092	0	Classical M	Xdh
Tmem51	0	2.305209	0.343	0.053	0	Classical M	Tmem51
Sirpb1b	0	2.567736	0.315	0.025	0	Classical M	Sirpb1b
Smpdl3a	0	1.45816	0.423	0.133	0	Classical M	Smpdl3a
H2-DMa1	0	0.792648	0.939	0.653	0	Classical M	H2-DMa
Fgr	0	1.576667	0.375	0.09	0	Classical M	Fgr
Adgre5	0	1.966941	0.349	0.064	0	Classical M	Adgre5
Anxa2	0	0.975505	0.411	0.126	0	Classical M	Anxa2
Ly6i	0	4.259661	0.353	0.068	0	Classical M	Ly6i
Ube2l6	0	1.262756	0.434	0.151	0	Classical M	Ube2l6
Creb5	0	1.615841	0.375	0.093	0	Classical M	Creb5
Emb	0	1.88012	0.34	0.059	0	Classical M	Emb
Rap2b1	0	1.529686	0.396	0.116	0	Classical M	Rap2b
Cd721	0	2.039428	0.403	0.124	0	Classical M	Cd72
Gbp3	0	1.841724	0.395	0.119	0	Classical M	Gbp3

Acvrl11	0	1.435997	0.407	0.132	0	Classical M	Acvrl1
Esyt1	0	1.406091	0.396	0.123	0	Classical M	Esyt1
Napsa	0	1.708383	0.325	0.055	0	Classical M	Napsa
Ctsc1	0	1.04067	0.962	0.693	0	Classical M	Ctsc
Ifit3	0	1.896065	0.377	0.108	0	Classical M	Ifit3
Atrnl1	0	1.373	0.349	0.081	0	Classical M	Atrnl1
Naca2	0	0.779801	0.958	0.691	0	Classical M	Naca
Gpx1	0	1.110073	0.954	0.688	0	Classical M	Gpx1
Gpr141	0	2.78771	0.303	0.037	0	Classical M	Gpr141
H3f3a	0	0.76417	0.925	0.66	0	Classical M	H3f3a
Cxcl9	0	2.371057	0.338	0.075	0	Classical M	Cxcl9
Serpina3g	0	3.502176	0.297	0.038	0	Classical M	Serpina3g
Usp18	0	1.937779	0.352	0.094	0	Classical M	Usp18
S100a10	0	1.558665	0.347	0.09	0	Classical M	S100a10
Ptma	0	0.953137	0.971	0.715	0	Classical M	Ptma
Arpc1b1	0	0.926484	0.947	0.691	0	Classical M	Arpc1b
Cdkn1a	0	2.220343	0.34	0.085	0	Classical M	Cdkn1a
Emilin2	0	2.298041	0.29	0.035	0	Classical M	Emilin2
Acp5	0	4.42002	0.282	0.027	0	Classical M	Acp5
Itgal	0	1.468719	0.332	0.079	0	Classical M	Itgal
Sirpb1a	0	3.288756	0.269	0.019	0	Classical M	Sirpb1a
Mgst1	0	1.428958	0.315	0.067	0	Classical M	Mgst1
Nedd9	0	0.979951	0.326	0.08	0	Classical M	Nedd9
Sh3bgrl31	0	1.010022	0.981	0.737	0	Classical M	Sh3bgrl3
Ucp21	0	0.981351	0.96	0.716	0	Classical M	Ucp2
Dnase1l1	0	1.891162	0.314	0.07	0	Classical M	Dnase1l1
Rpl72	0	0.737229	0.967	0.724	0	Classical M	Rpl7
Daxx	0	1.857556	0.344	0.103	0	Classical M	Daxx
Dram1	0	2.827485	0.278	0.04	0	Classical M	Dram1
Ifit2	0	2.163308	0.314	0.076	0	Classical M	Ifit2
S100a4	0	2.207463	0.286	0.049	0	Classical M	S100a4
Hspa81	0	1.016786	0.965	0.73	0	Classical M	Hspa8
AI662270	0	2.307065	0.282	0.047	0	Classical M	AI662270
Trerf1	0	1.986392	0.277	0.043	0	Classical M	Trerf1
Ifi206	0	2.624009	0.275	0.042	0	Classical M	Ifi206
Ccr1	0	1.583768	0.298	0.065	0	Classical M	Ccr1
Rps182	0	0.864815	0.98	0.75	0	Classical M	Rps18
Socs1	0	2.577866	0.303	0.08	0	Classical M	Socs1
Samd9l	0	2.027525	0.279	0.057	0	Classical M	Samd9l
Pfkp	0	1.973531	0.28	0.061	0	Classical M	Pfkp
Lilr4b	0	1.694762	0.266	0.048	0	Classical M	Lilr4b
Iqgap2	0	1.289681	0.287	0.07	0	Classical M	Iqgap2
Oas3	0	2.787311	0.251	0.037	0	Classical M	Oas3
Rpl7a2	0	0.733508	0.969	0.755	0	Classical M	Rpl7a

Cd742	0	1.512612	0.977	0.763	0	Classical M	Cd74
Msr1	0	1.855508	0.255	0.045	0	Classical M	Msr1
Rpl272	0	0.757541	0.977	0.767	0	Classical M	Rpl27
Dck	0	1.728925	0.269	0.061	0	Classical M	Dck
Pstpip1	0	1.58038	0.272	0.065	0	Classical M	Pstpip1
Haus8	0	1.957044	0.273	0.069	0	Classical M	Haus8
Rpl152	0	0.713933	0.979	0.777	0	Classical M	Rpl15
Rpl242	0	0.785548	0.981	0.78	0	Classical M	Rpl24
Rpl82	0	0.892427	0.985	0.785	0	Classical M	Rpl8
Rps192	0	0.821453	0.988	0.791	0	Classical M	Rps19
Rpl292	0	0.707692	0.985	0.791	0	Classical M	Rpl29
Rpl362	0	0.760759	0.976	0.784	0	Classical M	Rpl36
Rps142	0	0.815	0.988	0.801	0	Classical M	Rps14
Rps210	0	0.899166	0.993	0.808	0	Classical M	Rps2
Npc22	0	0.955411	0.985	0.802	0	Classical M	Npc2
Rps32	0	0.723686	0.992	0.821	0	Classical M	Rps3
Rpl262	0	0.676091	0.991	0.821	0	Classical M	Rpl26
Rpl62	0	0.659387	0.988	0.82	0	Classical M	Rpl6
Rpsa2	0	0.911592	0.993	0.825	0	Classical M	Rpsa
Apoe2	0	1.204716	0.945	0.778	0	Classical M	Apoe
Pfn1	0	0.801461	0.987	0.823	0	Classical M	Pfn1
Rpl172	0	0.768735	0.993	0.832	0	Classical M	Rpl17
Rpl102	0	0.837805	0.993	0.832	0	Classical M	Rpl10
Rpl182	0	0.898049	0.991	0.831	0	Classical M	Rpl18
Rpl282	0	0.777088	0.992	0.834	0	Classical M	Rpl28
Rplp02	0	0.850076	0.991	0.834	0	Classical M	Rplp0
Rpl342	0	0.69842	0.988	0.837	0	Classical M	Rpl34
Rps52	0	0.916683	0.995	0.845	0	Classical M	Rps5
Rps132	0	0.799603	0.992	0.842	0	Classical M	Rps13
Rps15a2	0	0.646669	0.992	0.85	0	Classical M	Rps15a
Rpl322	0	0.766715	0.995	0.86	0	Classical M	Rpl32
Rps72	0	0.746028	0.994	0.861	0	Classical M	Rps7
Rpl92	0	0.753443	0.997	0.87	0	Classical M	Rpl9
Rpl112	0	0.646868	0.996	0.87	0	Classical M	Rpl11
Rps202	0	0.717574	0.997	0.875	0	Classical M	Rps20
Rpl232	0	0.761385	0.998	0.876	0	Classical M	Rpl23
Rps162	0	0.807027	0.997	0.876	0	Classical M	Rps16
Rps4x2	0	0.693431	0.994	0.874	0	Classical M	Rps4x
Rps3a12	0	0.633662	0.992	0.878	0	Classical M	Rps3a1
Rpl192	0	0.776619	0.997	0.885	0	Classical M	Rpl19
Tpt12	0	0.969038	0.997	0.892	0	Classical M	Tpt1
Rps82	0	0.717147	0.998	0.896	0	Classical M	Rps8
Rpl18a2	0	0.634401	0.994	0.896	0	Classical M	Rpl18a
Rps27a2	0	0.573168	0.996	0.903	0	Classical M	Rps27a

Rps92	0	0.711677	0.996	0.907	0	Classical M	Rps9
Rpl132	0	0.723241	0.997	0.916	0	Classical M	Rpl13
Ftl11	0	0.804011	0.998	0.92	0	Classical M	Ftl1
Rps242	0	0.717586	0.999	0.925	0	Classical M	Rps24
Fau2	0	0.615034	0.999	0.937	0	Classical M	Fau
Eef1a12	0	0.626693	0.999	0.941	0	Classical M	Eef1a1
Fth11	0	0.814868	1	0.978	0	Classical M	Fth1
Rps112	6.05E-308	0.579336	0.996	0.89	1.48E-303	Classical M	Rps11
Ncf4	9.26E-307	1.106873	0.572	0.24	2.27E-302	Classical M	Ncf4
Cers6	1.38E-305	1.24778	0.5	0.192	3.37E-301	Classical M	Cers6
Flna	3.27E-305	1.13036	0.482	0.18	8.00E-301	Classical M	Flna
Stat11	3.31E-305	1.150649	0.806	0.481	8.11E-301	Classical M	Stat1
Zyx	9.71E-305	1.398275	0.567	0.241	2.38E-300	Classical M	Zyx
Psmb9	1.24E-303	0.987821	0.813	0.466	3.03E-299	Classical M	Psmb9
Il3ra	2.64E-303	1.81252	0.265	0.068	6.47E-299	Classical M	Il3ra
Csf2ra1	3.28E-303	1.012321	0.757	0.401	8.03E-299	Classical M	Csf2ra
Rps102	3.61E-301	0.584277	0.994	0.886	8.83E-297	Classical M	Rps10
Mrpl301	4.05E-301	1.041273	0.607	0.267	9.91E-297	Classical M	Mrpl30
Rps232	1.49E-300	0.623619	0.989	0.848	3.64E-296	Classical M	Rps23
Nsa22	2.64E-299	0.901356	0.799	0.431	6.46E-295	Classical M	Nsa2
H2afj	1.01E-298	0.960671	0.73	0.37	2.48E-294	Classical M	H2afj
Themis2	2.35E-297	1.128947	0.518	0.206	5.75E-293	Classical M	Themis2
Mapkapk3	2.35E-297	1.377057	0.345	0.107	5.77E-293	Classical M	Mapkapk3
Prelid11	3.89E-297	1.079392	0.661	0.32	9.51E-293	Classical M	Prelid1
Rpl35a2	2.31E-295	0.600819	0.995	0.879	5.65E-291	Classical M	Rpl35a
Slamf81	2.05E-292	1.426049	0.468	0.182	5.01E-288	Classical M	Slamf8
Tmem176b	2.95E-292	1.129777	0.84	0.618	7.22E-288	Classical M	Tmem176b
Psen21	4.72E-292	1.411013	0.378	0.126	1.16E-287	Classical M	Psen2
Nme22	3.36E-291	0.844354	0.903	0.586	8.24E-287	Classical M	Nme2
Eif3f2	2.62E-290	0.814149	0.901	0.573	6.41E-286	Classical M	Eif3f
Sp140	2.93E-290	1.313547	0.449	0.169	7.18E-286	Classical M	Sp140
Psme12	1.38E-289	0.849756	0.897	0.59	3.38E-285	Classical M	Psme1
Arpc5	3.81E-289	0.864085	0.776	0.413	9.33E-285	Classical M	Arpc5
Anp32b1	1.24E-288	0.969749	0.607	0.272	3.05E-284	Classical M	Anp32b
Il18bp1	3.48E-288	1.601492	0.391	0.137	8.52E-284	Classical M	Il18bp
Actb1	5.95E-288	0.673553	1	0.956	1.46E-283	Classical M	Actb
Plin21	1.35E-287	1.122895	0.515	0.209	3.31E-283	Classical M	Plin2
Limd1	8.26E-287	1.507825	0.255	0.065	2.02E-282	Classical M	Limd1
Eif4ebp1	3.25E-286	1.153178	0.492	0.194	7.95E-282	Classical M	Eif4ebp1
Taf101	7.33E-285	0.949469	0.698	0.35	1.79E-280	Classical M	Taf10
Rpl27a2	2.45E-284	0.559785	0.996	0.885	5.99E-280	Classical M	Rpl27a
Dok3	6.42E-283	0.981374	0.46	0.168	1.57E-278	Classical M	Dok3
Abracl	3.17E-281	1.075899	0.6	0.275	7.76E-277	Classical M	Abracl
Psma71	8.67E-280	0.984962	0.741	0.396	2.12E-275	Classical M	Psma7

Rpl222	6.47E-278	0.683644	0.968	0.736	1.58E-273	Classical M	Rpl22
Rps62	8.51E-276	0.705307	0.949	0.674	2.09E-271	Classical M	Rps6
Il1b1	2.21E-275	1.404836	0.309	0.093	5.42E-271	Classical M	Il1b
Rps262	8.49E-275	0.661922	0.982	0.814	2.08E-270	Classical M	Rps26
Btf31	2.37E-274	0.76819	0.904	0.598	5.80E-270	Classical M	Btf3
Scand1	2.98E-274	0.902668	0.749	0.4	7.29E-270	Classical M	Scand1
Shisa5	6.46E-272	0.939968	0.779	0.443	1.58E-267	Classical M	Shisa5
Vav3	1.07E-271	0.900716	0.266	0.07	2.61E-267	Classical M	Vav3
Aprt1	3.03E-271	1.11735	0.568	0.259	7.41E-267	Classical M	Aprt
Ehd1	6.88E-270	1.469078	0.316	0.098	1.69E-265	Classical M	Ehd1
Rpl10a2	2.85E-269	0.607281	0.984	0.807	6.98E-265	Classical M	Rpl10a
Arpc2	5.89E-267	0.716855	0.942	0.696	1.44E-262	Classical M	Arpc2
Nap1l1	1.26E-266	0.984836	0.515	0.214	3.09E-262	Classical M	Nap1l1
Dynll1	1.95E-266	0.966053	0.737	0.403	4.76E-262	Classical M	Dynll1
Rpl37a2	2.42E-266	0.635243	0.987	0.847	5.92E-262	Classical M	Rpl37a
Rpl310	3.34E-266	0.624764	0.979	0.784	8.19E-262	Classical M	Rpl3
Cnn2	8.92E-265	0.698395	0.356	0.113	2.18E-260	Classical M	Cnn2
Sod21	1.54E-264	1.757242	0.439	0.178	3.77E-260	Classical M	Sod2
Eif3k1	4.44E-262	0.807793	0.823	0.469	1.09E-257	Classical M	Eif3k
Nupr1	1.22E-261	1.345454	0.437	0.171	3.00E-257	Classical M	Nupr1
Rpl36al2	6.99E-260	0.765304	0.921	0.634	1.71E-255	Classical M	Rpl36al
Cebpb1	2.73E-258	1.105445	0.821	0.525	6.68E-254	Classical M	Cebpb
Myl12a	9.20E-258	0.944453	0.768	0.437	2.25E-253	Classical M	Myl12a
Mrpl33	1.17E-257	0.880481	0.564	0.251	2.86E-253	Classical M	Mrpl33
Svbp1	4.23E-257	1.077302	0.545	0.243	1.04E-252	Classical M	Svbp
Cd44	1.39E-256	0.945847	0.467	0.186	3.41E-252	Classical M	Cd44
Myl6	2.02E-256	0.688401	0.9	0.594	4.94E-252	Classical M	Myl6
H2-T222	4.11E-256	0.966645	0.66	0.33	1.01E-251	Classical M	H2-T22
Pnp1	1.32E-255	1.052164	0.776	0.465	3.23E-251	Classical M	Pnp
Gbp6	4.54E-254	1.607545	0.274	0.081	1.11E-249	Classical M	Gbp6
Phf11d	7.89E-254	1.625188	0.318	0.104	1.93E-249	Classical M	Phf11d
Cox5a1	1.12E-251	0.884909	0.776	0.447	2.75E-247	Classical M	Cox5a
Mpp1	2.00E-251	1.097698	0.388	0.14	4.91E-247	Classical M	Mpp1
Sash1	8.46E-251	0.826979	0.371	0.129	2.07E-246	Classical M	Sash1
Cd300lf1	3.88E-250	1.072364	0.374	0.133	9.51E-246	Classical M	Cd300lf
Tmem205	7.79E-250	1.417675	0.278	0.083	1.91E-245	Classical M	Tmem205
Lst1	4.24E-248	0.906253	0.815	0.523	1.04E-243	Classical M	Lst1
Fam241a	1.50E-246	1.531764	0.277	0.083	3.67E-242	Classical M	Fam241a
Eif3h2	2.68E-246	0.793705	0.806	0.471	6.57E-242	Classical M	Eif3h
Atp5l1	1.46E-244	0.766183	0.862	0.553	3.57E-240	Classical M	Atp5l
Hsp90ab11	2.10E-244	0.626181	0.962	0.729	5.14E-240	Classical M	Hsp90ab1
Birc3	7.97E-244	1.063329	0.508	0.22	1.95E-239	Classical M	Birc3
Glrx1	8.13E-244	1.250863	0.38	0.141	1.99E-239	Classical M	Glrx
Rpl142	1.39E-243	0.63	0.963	0.704	3.40E-239	Classical M	Rpl14

Cycs	7.78E-243	1.093757	0.593	0.29	1.91E-238	Classical M	Cycs
Eef22	2.26E-242	0.653596	0.953	0.71	5.53E-238	Classical M	Eef2
Nadk	9.95E-242	0.899508	0.532	0.235	2.44E-237	Classical M	Nadk
Sdc41	1.46E-241	1.579345	0.348	0.125	3.57E-237	Classical M	Sdc4
Mapkapk2	8.37E-241	0.835942	0.639	0.314	2.05E-236	Classical M	Mapkapk2
Chmp4b1	3.08E-239	0.954248	0.759	0.45	7.55E-235	Classical M	Chmp4b
Arhgdib	2.19E-237	0.647885	0.89	0.605	5.36E-233	Classical M	Arhgdib
Tap1	1.25E-236	0.900753	0.704	0.374	3.07E-232	Classical M	Tap1
Marcksl1	2.32E-236	1.379577	0.387	0.147	5.68E-232	Classical M	Marcksl1
Rpl382	3.45E-236	0.785343	0.923	0.691	8.45E-232	Classical M	Rpl38
Psma4	3.04E-235	0.981692	0.516	0.229	7.45E-231	Classical M	Psma4
Arf5	1.61E-234	0.771034	0.8	0.466	3.95E-230	Classical M	Arf5
Eif5a1	5.47E-233	0.777729	0.805	0.476	1.34E-228	Classical M	Eif5a
Epsti1	1.10E-232	0.696625	0.727	0.396	2.69E-228	Classical M	Epsti1
Esd1	1.63E-232	0.875117	0.627	0.311	4.00E-228	Classical M	Esd
Serf21	7.57E-230	0.623553	0.956	0.75	1.85E-225	Classical M	Serf2
Akr1a11	4.80E-229	0.665515	0.887	0.554	1.17E-224	Classical M	Akr1a1
Rplp22	2.42E-227	0.579737	0.978	0.793	5.92E-223	Classical M	Rplp2
Mrpl541	1.47E-226	1.005201	0.443	0.183	3.61E-222	Classical M	Mrpl54
Ppia1	7.01E-226	0.497012	0.992	0.876	1.72E-221	Classical M	Ppia
Gpi11	3.47E-225	0.774336	0.77	0.438	8.51E-221	Classical M	Gpi1
Ifnar11	5.03E-225	0.920495	0.505	0.224	1.23E-220	Classical M	Ifnar1
Rpp21	1.93E-222	1.007001	0.371	0.138	4.73E-218	Classical M	Rpp21
Diaph1	2.53E-222	0.969462	0.36	0.131	6.19E-218	Classical M	Diaph1
Cox8a1	1.23E-221	0.61606	0.964	0.775	3.01E-217	Classical M	Cox8a
Lsp1	7.76E-221	0.733299	0.456	0.192	1.90E-216	Classical M	Lsp1
Srgn	6.61E-220	1.054929	0.822	0.54	1.62E-215	Classical M	Srgn
St8sia4	4.13E-219	0.985096	0.286	0.091	1.01E-214	Classical M	St8sia4
Rps27l1	1.13E-218	0.72029	0.66	0.344	2.77E-214	Classical M	Rps27l
Jpt1	3.51E-218	0.805612	0.663	0.344	8.59E-214	Classical M	Jpt1
Ogfr1	2.40E-217	1.091351	0.441	0.187	5.87E-213	Classical M	Ogfr
Dhx58	2.42E-217	1.38653	0.276	0.089	5.91E-213	Classical M	Dhx58
Cox5b1	2.65E-217	0.746802	0.833	0.531	6.49E-213	Classical M	Cox5b
Rpl52	3.28E-217	0.672444	0.898	0.601	8.03E-213	Classical M	Rpl5
Hnrnpa3	3.78E-217	0.794075	0.754	0.439	9.25E-213	Classical M	Hnrnpa3
Rbm31	2.60E-216	0.697648	0.847	0.521	6.37E-212	Classical M	Rbm3
Cited21	3.71E-216	0.907304	0.575	0.28	9.09E-212	Classical M	Cited2
Snrpe1	6.92E-216	0.795408	0.652	0.337	1.69E-211	Classical M	Snrpe
Atp5o1	2.03E-215	0.775491	0.679	0.364	4.96E-211	Classical M	Atp5o
Tpm4	1.23E-214	0.991923	0.393	0.154	3.01E-210	Classical M	Tpm4
Nt5c	6.76E-212	0.947322	0.449	0.191	1.66E-207	Classical M	Nt5c
Siva1	1.07E-211	1.192668	0.292	0.098	2.63E-207	Classical M	Siva1
Pitpna	1.95E-211	0.788936	0.777	0.467	4.77E-207	Classical M	Pitpna
Morrbid1	1.18E-210	1.415131	0.313	0.112	2.88E-206	Classical M	Morrbid

Hpcal11	3.81E-210	0.749739	0.535	0.248	9.32E-206	Classical M	Hpcal1
Irgm1	1.14E-209	0.996553	0.512	0.234	2.78E-205	Classical M	Irgm1
Rnase6	2.97E-209	0.956423	0.425	0.173	7.27E-205	Classical M	Rnase6
Arpc31	7.55E-209	0.628049	0.906	0.627	1.85E-204	Classical M	Arpc3
Ftl1-ps11	2.10E-208	0.741256	0.474	0.208	5.14E-204	Classical M	Ftl1-ps1
Myl12b	2.38E-207	0.621969	0.897	0.621	5.83E-203	Classical M	Myl12b
Sf3b5	2.83E-206	0.76492	0.59	0.291	6.93E-202	Classical M	Sf3b5
Aldh21	3.69E-206	0.590127	0.554	0.258	9.05E-202	Classical M	Aldh2
Tapbp1	5.67E-206	0.827877	0.786	0.488	1.39E-201	Classical M	Tapbp
Rab321	1.01E-205	1.032131	0.43	0.183	2.46E-201	Classical M	Rab32
Lsm41	6.03E-203	0.748178	0.528	0.245	1.48E-198	Classical M	Lsm4
Tmem192	6.80E-203	1.008342	0.342	0.127	1.67E-198	Classical M	Tmem192
Ifih1	2.00E-202	1.195583	0.369	0.147	4.90E-198	Classical M	Ifih1
Ly6e1	4.36E-202	0.596671	0.977	0.859	1.07E-197	Classical M	Ly6e
Gmfg	3.75E-201	0.631585	0.692	0.371	9.17E-197	Classical M	Gmfg
Arl5c1	1.10E-200	1.313012	0.337	0.129	2.70E-196	Classical M	Arl5c
Rps282	2.16E-200	0.673904	0.96	0.747	5.29E-196	Classical M	Rps28
Olfm1	2.89E-200	0.879702	0.25	0.077	7.08E-196	Classical M	Olfm1
Sdcbp1	3.58E-200	0.774333	0.814	0.517	8.77E-196	Classical M	Sdcbp
Parp11	5.07E-199	1.134305	0.286	0.098	1.24E-194	Classical M	Parp11
Sec61g1	1.16E-198	0.789236	0.726	0.421	2.83E-194	Classical M	Sec61g
Sri	3.53E-196	0.704607	0.607	0.308	8.65E-192	Classical M	Sri
Rpl412	1.13E-195	0.551652	0.993	0.899	2.76E-191	Classical M	Rpl41
Coro1a	1.08E-194	0.670541	0.884	0.615	2.65E-190	Classical M	Coro1a
Parp14	1.48E-194	0.94568	0.529	0.258	3.63E-190	Classical M	Parp14
Arrdc4	1.60E-194	1.371089	0.254	0.083	3.93E-190	Classical M	Arrdc4
Dennd1a1	1.86E-194	0.464734	0.657	0.337	4.56E-190	Classical M	Dennd1a
Smdt1	7.22E-194	0.651307	0.722	0.403	1.77E-189	Classical M	Smdt1
Coro1b1	3.43E-193	0.749686	0.76	0.456	8.40E-189	Classical M	Coro1b
Trim25	4.16E-193	0.959578	0.387	0.159	1.02E-188	Classical M	Trim25
Eid1	5.05E-193	0.885475	0.368	0.146	1.24E-188	Classical M	Eid1
Atp5d1	6.39E-193	0.649179	0.821	0.514	1.56E-188	Classical M	Atp5d
Tmsb4x	2.15E-192	0.394802	1	0.966	5.27E-188	Classical M	Tmsb4x
Blvrb1	5.51E-192	0.59419	0.593	0.298	1.35E-187	Classical M	Blvrb
Mcl11	7.96E-192	0.714922	0.818	0.526	1.95E-187	Classical M	Mcl1
Psap2	8.00E-192	0.475295	0.997	0.943	1.96E-187	Classical M	Psap
Ndufb1-ps	2.72E-190	1.026923	0.548	0.287	6.67E-186	Classical M	Ndufb1-ps
Ybx3	5.02E-190	0.94967	0.304	0.109	1.23E-185	Classical M	Ybx3
Txn2	1.06E-189	0.776699	0.506	0.238	2.60E-185	Classical M	Txn2
Cd47	3.12E-189	0.703804	0.762	0.445	7.65E-185	Classical M	Cd47
Psmb41	4.33E-189	0.766481	0.549	0.269	1.06E-184	Classical M	Psmb4
Tpd52	2.22E-188	0.609155	0.732	0.412	5.45E-184	Classical M	Tpd52
Rpl302	3.21E-188	0.450769	0.996	0.893	7.87E-184	Classical M	Rpl30
Mvb12a	4.39E-188	0.865531	0.433	0.191	1.08E-183	Classical M	Mvb12a

Gabarap2	1.40E-187	0.563181	0.927	0.663	3.44E-183	Classical M	Gabarap
Iigp1	1.49E-187	0.938879	0.348	0.138	3.64E-183	Classical M	Iigp1
Bak11	2.61E-187	1.107424	0.326	0.125	6.40E-183	Classical M	Bak1
Atp5h	1.00E-186	0.579198	0.848	0.538	2.45E-182	Classical M	Atp5h
Banf11	1.17E-186	0.761046	0.532	0.256	2.85E-182	Classical M	Banf1
Rpl23a1	3.13E-186	0.666678	0.799	0.491	7.66E-182	Classical M	Rpl23a
Dazap2	5.57E-186	0.678464	0.747	0.44	1.36E-181	Classical M	Dazap2
Actg1	6.15E-186	0.628582	0.951	0.774	1.51E-181	Classical M	Actg1
Uqcrfs11	1.14E-185	0.748411	0.594	0.305	2.79E-181	Classical M	Uqcrfs1
Sulf2	2.65E-185	1.335552	0.272	0.096	6.49E-181	Classical M	Sulf2
Eif3i1	2.78E-185	0.713034	0.603	0.311	6.81E-181	Classical M	Eif3i
Cox6b11	1.06E-183	0.654896	0.826	0.544	2.59E-179	Classical M	Cox6b1
Hacd4	1.22E-182	0.744267	0.458	0.206	2.98E-178	Classical M	Hacd4
Rpl43	1.30E-182	0.614513	0.871	0.585	3.18E-178	Classical M	Rpl4
2410006H1	2.26E-182	0.768486	0.766	0.474	5.54E-178	Classical M	2410006H16Rik
Mvp1	2.95E-182	0.911301	0.41	0.179	7.23E-178	Classical M	Mvp
Hint11	1.60E-181	0.605462	0.822	0.513	3.93E-177	Classical M	Hint1
Cdk2ap2	3.53E-181	0.991446	0.557	0.299	8.65E-177	Classical M	Cdk2ap2
Tctex1d2	1.20E-180	0.937734	0.354	0.143	2.93E-176	Classical M	Tctex1d2
Psma21	2.04E-180	0.717527	0.635	0.344	5.00E-176	Classical M	Psma2
Chmp2a	5.22E-180	0.768936	0.574	0.295	1.28E-175	Classical M	Chmp2a
Npm12	1.66E-179	0.609961	0.837	0.533	4.06E-175	Classical M	Npm1
Hcls1	5.37E-179	0.726914	0.7	0.399	1.32E-174	Classical M	Hcls1
Eif3e1	8.98E-179	0.731536	0.575	0.293	2.20E-174	Classical M	Eif3e
Klf61	1.00E-178	0.681065	0.656	0.356	2.45E-174	Classical M	Klf6
Ezr	1.87E-177	0.800537	0.259	0.088	4.57E-173	Classical M	Ezr
Nmi	2.27E-177	1.047124	0.362	0.151	5.55E-173	Classical M	Nmi
Plekho21	3.26E-177	0.701861	0.436	0.195	7.98E-173	Classical M	Plekho2
Stat2	7.62E-177	1.004828	0.408	0.181	1.87E-172	Classical M	Stat2
Lrrc25	2.22E-176	0.696359	0.65	0.352	5.44E-172	Classical M	Lrrc25
Serp1	3.01E-176	0.759389	0.642	0.348	7.38E-172	Classical M	Serp1
Nop53	4.02E-176	0.880535	0.402	0.176	9.83E-172	Classical M	Nop53
Snrpg1	1.13E-175	0.641946	0.716	0.415	2.78E-171	Classical M	Snrpg
Elob1	1.37E-175	0.602699	0.813	0.51	3.36E-171	Classical M	Elob
Pgap2	8.01E-174	0.965219	0.365	0.153	1.96E-169	Classical M	Pgap2
Fos2	1.40E-173	0.538419	0.783	0.465	3.44E-169	Classical M	Fos
Lmo4	7.62E-173	0.9037	0.35	0.142	1.87E-168	Classical M	Lmo4
Tle5	7.96E-173	0.594919	0.698	0.39	1.95E-168	Classical M	Tle5
Pkm1	3.07E-172	0.750311	0.778	0.493	7.52E-168	Classical M	Pkm
Adap1	4.86E-171	0.914894	0.338	0.137	1.19E-166	Classical M	Adap1
Fam174a	7.65E-171	0.685519	0.586	0.303	1.87E-166	Classical M	Fam174a
St3gal4	1.28E-170	0.755786	0.31	0.117	3.13E-166	Classical M	St3gal4
Cast	4.45E-170	0.904882	0.29	0.108	1.09E-165	Classical M	Cast
Rps272	4.55E-170	0.562296	0.959	0.774	1.11E-165	Classical M	Rps27

Lamtor5	4.96E-170	0.685277	0.488	0.234	1.21E-165	Classical M	Lamtor5
App	1.97E-169	0.594669	0.814	0.525	4.82E-165	Classical M	App
Snrpf1	2.14E-169	0.753639	0.52	0.259	5.25E-165	Classical M	Snrpf
Rpl352	3.35E-169	0.552444	0.965	0.766	8.21E-165	Classical M	Rpl35
Znhit1	8.75E-169	0.812698	0.41	0.183	2.14E-164	Classical M	Znhit1
Ddx58	1.50E-168	0.987219	0.317	0.124	3.67E-164	Classical M	Ddx58
Arl111	1.73E-168	0.946352	0.362	0.154	4.23E-164	Classical M	Arl11
Rftn1	2.02E-168	0.520404	0.344	0.139	4.93E-164	Classical M	Rftn1
Mthfd2	8.13E-168	1.22096	0.277	0.103	1.99E-163	Classical M	Mthfd2
B4galnt1	1.75E-167	0.889679	0.375	0.162	4.28E-163	Classical M	B4galnt1
Eef1b22	4.06E-167	0.50727	0.935	0.666	9.95E-163	Classical M	Eef1b2
Gbp8	4.17E-167	1.040569	0.279	0.103	1.02E-162	Classical M	Gbp8
Myo1g	5.98E-167	0.657456	0.305	0.116	1.46E-162	Classical M	Myo1g
Dr1	7.21E-167	1.030784	0.263	0.093	1.76E-162	Classical M	Dr1
Sec61b1	9.52E-167	0.723309	0.737	0.45	2.33E-162	Classical M	Sec61b
AI4135821	1.70E-166	0.690513	0.593	0.316	4.15E-162	Classical M	AI413582
Neurl3	2.20E-166	0.681568	0.463	0.217	5.38E-162	Classical M	Neurl3
Ptpn61	4.66E-166	0.684882	0.791	0.5	1.14E-161	Classical M	Ptpn6
Tgtp2	1.12E-165	1.116511	0.274	0.101	2.75E-161	Classical M	Tgtp2
Rpl312	1.85E-165	0.591353	0.81	0.514	4.52E-161	Classical M	Rpl31
Calm3	2.54E-165	0.573918	0.604	0.32	6.21E-161	Classical M	Calm3
Tep1	2.72E-165	0.768538	0.435	0.2	6.65E-161	Classical M	Tep1
Ost4	2.85E-165	0.578225	0.786	0.484	6.98E-161	Classical M	Ost4
Capns11	3.40E-164	0.628567	0.627	0.337	8.33E-160	Classical M	Capns1
Ifi35	4.35E-164	0.864192	0.473	0.229	1.07E-159	Classical M	Ifi35
Prdx6	5.13E-164	0.754453	0.413	0.187	1.26E-159	Classical M	Prdx6
Tmem134	5.00E-163	0.70008	0.496	0.243	1.22E-158	Classical M	Tmem134
H2-T232	8.27E-163	0.533299	0.932	0.703	2.02E-158	Classical M	H2-T23
Psmb31	8.99E-163	0.63661	0.677	0.388	2.20E-158	Classical M	Psmb3
Sp110	9.34E-163	0.886892	0.475	0.234	2.29E-158	Classical M	Sp110
Atp5e1	1.38E-162	0.649774	0.873	0.64	3.38E-158	Classical M	Atp5e
Edf11	7.47E-162	0.610548	0.748	0.448	1.83E-157	Classical M	Edf1
Dbi	2.22E-161	0.392467	0.726	0.421	5.43E-157	Classical M	Dbi
Cyba2	2.83E-161	0.490316	0.987	0.855	6.92E-157	Classical M	Cyba
Pid11	4.35E-161	0.675412	0.679	0.402	1.06E-156	Classical M	Pid1
Rpl372	1.85E-160	0.48779	0.983	0.845	4.52E-156	Classical M	Rpl37
Tomm221	2.11E-160	0.628221	0.593	0.314	5.16E-156	Classical M	Tomm22
Nme11	6.31E-160	0.660461	0.506	0.25	1.54E-155	Classical M	Nme1
Cox14	1.27E-159	0.657016	0.512	0.254	3.12E-155	Classical M	Cox14
Eif1	8.69E-159	0.449513	0.978	0.826	2.13E-154	Classical M	Eif1
Clec4a31	1.08E-158	0.822364	0.529	0.276	2.64E-154	Classical M	Clec4a3
Tpi11	2.28E-158	0.939469	0.568	0.305	5.59E-154	Classical M	Tpi1
Psma51	2.47E-158	0.712843	0.561	0.296	6.04E-154	Classical M	Psma5
Gbp5	3.91E-158	1.580823	0.295	0.12	9.59E-154	Classical M	Gbp5

Batf3	4.14E-158	0.946463	0.405	0.186	1.01E-153	Classical M	Batf3
Tkt1	7.07E-158	0.627707	0.7	0.411	1.73E-153	Classical M	Tkt
Ndufb81	1.05E-157	0.581771	0.67	0.381	2.58E-153	Classical M	Ndufb8
Cox7a21	1.57E-157	0.594159	0.739	0.448	3.85E-153	Classical M	Cox7a2
Dtx3l	1.72E-157	0.975989	0.35	0.151	4.21E-153	Classical M	Dtx3l
Psma61	6.86E-157	0.689381	0.512	0.259	1.68E-152	Classical M	Psma6
Rps122	1.12E-156	0.42232	0.985	0.863	2.75E-152	Classical M	Rps12
Lamtor21	1.49E-156	0.596931	0.608	0.33	3.65E-152	Classical M	Lamtor2
Atp1a31	1.07E-155	1.053626	0.276	0.106	2.63E-151	Classical M	Atp1a3
Npm3	1.89E-155	0.982693	0.301	0.12	4.64E-151	Classical M	Npm3
Myo5a1	8.01E-155	0.416207	0.497	0.242	1.96E-150	Classical M	Myo5a
Klf131	1.43E-154	0.603822	0.568	0.299	3.50E-150	Classical M	Klf13
Snrpb	3.34E-154	0.590366	0.682	0.389	8.19E-150	Classical M	Snrpb
Psmg4	6.13E-154	0.735288	0.383	0.171	1.50E-149	Classical M	Psmg4
Uqcrq1	9.04E-154	0.643582	0.706	0.425	2.21E-149	Classical M	Uqcrq
Ldha1	3.91E-153	0.79458	0.709	0.432	9.57E-149	Classical M	Ldha
Tma7	6.31E-153	0.593805	0.677	0.394	1.55E-148	Classical M	Tma7
Tgm21	8.64E-153	2.007023	0.286	0.117	2.12E-148	Classical M	Tgm2
Srp9	6.62E-152	0.548002	0.643	0.356	1.62E-147	Classical M	Srp9
Trafd1	1.27E-151	0.710322	0.625	0.356	3.11E-147	Classical M	Trafd1
Cfl12	1.55E-151	0.461406	0.953	0.755	3.80E-147	Classical M	Cfl1
H2afy	3.67E-151	0.675027	0.642	0.365	8.98E-147	Classical M	H2afy
Csf2rb	6.12E-151	0.875251	0.484	0.247	1.50E-146	Classical M	Csf2rb
Ciao2a1	1.38E-150	0.688383	0.479	0.239	3.39E-146	Classical M	Ciao2a
Ran1	2.63E-150	0.621313	0.621	0.343	6.44E-146	Classical M	Ran
Atp5c11	4.48E-150	0.576342	0.783	0.49	1.10E-145	Classical M	Atp5c1
Cnih4	6.31E-150	0.645379	0.565	0.301	1.55E-145	Classical M	Cnih4
Ccl51	7.96E-150	0.973657	0.339	0.15	1.95E-145	Classical M	Ccl5
Milr11	1.54E-149	0.845487	0.352	0.154	3.78E-145	Classical M	Milr1
Myd88	6.55E-149	0.958567	0.309	0.127	1.60E-144	Classical M	Myd88
Ak21	1.68E-148	0.803935	0.5	0.262	4.11E-144	Classical M	Ak2
Atp6v0c2	3.09E-148	0.521467	0.958	0.767	7.57E-144	Classical M	Atp6v0c
Rab8a	3.58E-148	0.686765	0.416	0.194	8.78E-144	Classical M	Rab8a
Eno11	9.89E-148	0.69897	0.537	0.288	2.42E-143	Classical M	Eno1
Tomm71	1.08E-147	0.591462	0.652	0.373	2.63E-143	Classical M	Tomm7
Pold43	1.27E-147	0.648355	0.634	0.359	3.11E-143	Classical M	Pold4
S100a13	1.42E-147	0.63214	0.446	0.216	3.48E-143	Classical M	S100a13
1600014C1	3.57E-147	0.920427	0.377	0.173	8.75E-143	Classical M	1600014C10Rik
Bax1	6.00E-147	0.636904	0.625	0.351	1.47E-142	Classical M	Bax
Cmpk1	7.62E-147	0.630673	0.51	0.26	1.87E-142	Classical M	Cmpk1
Ndufb71	9.24E-147	0.556376	0.642	0.361	2.26E-142	Classical M	Ndufb7
Cox7b1	2.11E-146	0.63631	0.619	0.355	5.16E-142	Classical M	Cox7b
Dmac1	2.59E-146	0.857663	0.337	0.146	6.34E-142	Classical M	Dmac1
Cenpb1	2.76E-146	0.742967	0.327	0.139	6.76E-142	Classical M	Cenpb

Ppp4c	6.01E-146	0.584984	0.598	0.329	1.47E-141	Classical M	Ppp4c
Llph1	6.40E-146	0.660893	0.458	0.226	1.57E-141	Classical M	Llph
Arf6	1.84E-145	0.683811	0.5	0.257	4.52E-141	Classical M	Arf6
Bloc1s11	2.13E-145	0.825621	0.435	0.216	5.22E-141	Classical M	Bloc1s1
Mien11	2.65E-145	0.728841	0.406	0.191	6.49E-141	Classical M	Mien1
Fcgr42	2.69E-145	0.80213	0.462	0.232	6.59E-141	Classical M	Fcgr4
Fam111a	3.57E-145	0.910108	0.321	0.135	8.74E-141	Classical M	Fam111a
Parp12	5.30E-145	0.8066	0.359	0.159	1.30E-140	Classical M	Parp12
Rpl36a2	7.43E-145	0.521908	0.898	0.639	1.82E-140	Classical M	Rpl36a
Plaur1	8.29E-145	0.896001	0.364	0.166	2.03E-140	Classical M	Plaur
Rps152	1.46E-144	0.53883	0.84	0.555	3.57E-140	Classical M	Rps15
Gapdh1	2.53E-144	0.720723	0.849	0.618	6.19E-140	Classical M	Gapdh
Eif6	8.98E-144	0.725859	0.427	0.205	2.20E-139	Classical M	Eif6
C3ar12	1.12E-143	0.774692	0.664	0.414	2.74E-139	Classical M	C3ar1
Spg211	1.79E-143	0.655888	0.467	0.231	4.39E-139	Classical M	Spg21
Pgk11	2.97E-143	0.903958	0.477	0.248	7.27E-139	Classical M	Pgk1
Ppp1ca	6.25E-143	0.571091	0.732	0.451	1.53E-138	Classical M	Ppp1ca
Pilra	1.44E-142	0.754297	0.293	0.118	3.53E-138	Classical M	Pilra
Ptpn18	2.83E-142	0.515306	0.891	0.637	6.94E-138	Classical M	Ptpn18
Rpl122	2.92E-142	0.497048	0.925	0.686	7.16E-138	Classical M	Rpl12
Arid5b1	3.31E-142	0.608386	0.574	0.319	8.11E-138	Classical M	Arid5b
Oaz1	3.39E-142	0.430817	0.941	0.711	8.31E-138	Classical M	Oaz1
D8Ertd738	3.81E-142	0.521147	0.723	0.43	9.32E-138	Classical M	D8Ertd738e
Irf11	5.55E-142	0.717989	0.574	0.317	1.36E-137	Classical M	Irf1
Mbd2	5.62E-142	0.578838	0.485	0.244	1.38E-137	Classical M	Mbd2
Rras	5.66E-142	0.786509	0.315	0.133	1.39E-137	Classical M	Rras
Fam49a	1.06E-141	0.660462	0.327	0.139	2.59E-137	Classical M	Fam49a
Txndc171	1.39E-141	0.541275	0.552	0.299	3.40E-137	Classical M	Txndc17
Tifab1	2.96E-141	0.685771	0.544	0.294	7.25E-137	Classical M	Tifab
Eef1g1	3.17E-141	0.546299	0.653	0.37	7.76E-137	Classical M	Eef1g
1810037I1	3.66E-141	0.546876	0.598	0.33	8.95E-137	Classical M	1810037I17Rik
Atp5g31	5.64E-141	0.52678	0.85	0.58	1.38E-136	Classical M	Atp5g3
Nhp21	6.51E-141	0.790461	0.36	0.164	1.59E-136	Classical M	Nhp2
Sumo2	7.53E-140	0.502858	0.795	0.498	1.84E-135	Classical M	Sumo2
Rhog	7.59E-140	0.526118	0.872	0.62	1.86E-135	Classical M	Rhog
Eif3m1	1.38E-139	0.620914	0.518	0.27	3.38E-135	Classical M	Eif3m
9-Sep	1.49E-139	0.729517	0.315	0.134	3.66E-135	Classical M	9-Sep
Uqcr101	3.23E-139	0.559205	0.636	0.371	7.92E-135	Classical M	Uqcr10
Mrpl231	3.56E-139	0.545908	0.6	0.33	8.71E-135	Classical M	Mrpl23
Fam89b	3.77E-139	0.664248	0.498	0.258	9.24E-135	Classical M	Fam89b
Fam129a	6.01E-139	0.640848	0.337	0.147	1.47E-134	Classical M	Fam129a
Ndufb61	7.11E-139	0.590614	0.489	0.251	1.74E-134	Classical M	Ndufb6
Zfas11	9.22E-139	0.819994	0.386	0.182	2.26E-134	Classical M	Zfas1
Alox5ap1	1.25E-138	0.631355	0.841	0.639	3.07E-134	Classical M	Alox5ap

Atp5b1	1.66E-138	0.551517	0.799	0.522	4.07E-134	Classical M	Atp5b
Sdhb1	6.66E-138	0.568714	0.661	0.386	1.63E-133	Classical M	Sdhb
Cd2741	1.02E-137	1.124802	0.299	0.128	2.49E-133	Classical M	Cd274
Snu13	1.96E-137	0.56582	0.565	0.305	4.81E-133	Classical M	Snu13
Gm2a2	2.26E-137	0.620328	0.701	0.426	5.54E-133	Classical M	Gm2a
Ndufa61	2.61E-137	0.503564	0.801	0.507	6.38E-133	Classical M	Ndufa6
Aurkaip11	2.70E-137	0.579443	0.489	0.251	6.61E-133	Classical M	Aurkaip1
Timm10b	5.44E-137	0.599904	0.693	0.421	1.33E-132	Classical M	Timm10b
Dusp12	6.85E-137	0.650635	0.633	0.373	1.68E-132	Classical M	Dusp1
P2rx41	1.14E-136	0.550942	0.565	0.306	2.78E-132	Classical M	P2rx4
Mrps241	1.77E-136	0.563804	0.513	0.268	4.34E-132	Classical M	Mrps24
Snrpb2	2.64E-136	0.795922	0.34	0.151	6.46E-132	Classical M	Snrpb2
Ubb1	6.86E-136	0.357729	0.984	0.86	1.68E-131	Classical M	Ubb
Tomm61	1.75E-135	0.493421	0.802	0.519	4.29E-131	Classical M	Tomm6
Atp2b11	2.15E-135	0.494001	0.721	0.444	5.27E-131	Classical M	Atp2b1
Atp5j21	6.07E-135	0.546625	0.781	0.507	1.49E-130	Classical M	Atp5j2
Ap2s11	9.57E-135	0.56602	0.537	0.288	2.34E-130	Classical M	Ap2s1
Psmb11	1.27E-134	0.532401	0.672	0.39	3.11E-130	Classical M	Psmb1
Hnrnpul2	2.79E-134	0.590862	0.45	0.224	6.84E-130	Classical M	Hnrnpul2
Nop10	4.43E-134	0.561813	0.529	0.283	1.08E-129	Classical M	Nop10
Glud1	5.05E-134	0.523777	0.591	0.33	1.24E-129	Classical M	Glud1
Trim30a1	1.05E-133	0.658908	0.562	0.313	2.56E-129	Classical M	Trim30a
Sf3b6	2.93E-133	0.549813	0.562	0.308	7.18E-129	Classical M	Sf3b6
Nedd8	9.75E-133	0.500634	0.727	0.438	2.39E-128	Classical M	Nedd8
Ensa	2.04E-132	0.690791	0.318	0.138	5.01E-128	Classical M	Ensa
BC005537	2.50E-132	0.685493	0.377	0.177	6.11E-128	Classical M	BC005537
Atp5md	4.95E-132	0.565177	0.653	0.392	1.21E-127	Classical M	Atp5md
Uqcrb1	8.23E-132	0.49729	0.684	0.411	2.01E-127	Classical M	Uqcrb
Tomm51	1.91E-131	0.642981	0.4	0.192	4.69E-127	Classical M	Tomm5
Pin4	8.35E-131	0.757565	0.322	0.143	2.04E-126	Classical M	Pin4
Cacybp	1.18E-130	0.641285	0.391	0.187	2.90E-126	Classical M	Cacybp
Vamp81	1.40E-130	0.493981	0.854	0.582	3.42E-126	Classical M	Vamp8
Copz1	1.57E-130	0.605778	0.434	0.216	3.83E-126	Classical M	Copz1
Rpl392	3.15E-130	0.43652	0.973	0.814	7.72E-126	Classical M	Rpl39
Gnb2	4.43E-130	0.493665	0.846	0.57	1.08E-125	Classical M	Gnb2
Raly	4.66E-130	0.575053	0.526	0.283	1.14E-125	Classical M	Raly
Pml	8.45E-130	1.023707	0.266	0.109	2.07E-125	Classical M	Pml
Plaat31	1.13E-129	0.825908	0.401	0.199	2.76E-125	Classical M	Plaat3
Rps172	1.36E-129	0.512998	0.827	0.551	3.34E-125	Classical M	Rps17
Rpl212	1.70E-129	0.337254	0.995	0.886	4.15E-125	Classical M	Rpl21
Bcl2a1b1	3.40E-129	0.789475	0.672	0.429	8.34E-125	Classical M	Bcl2a1b
Slc9a3r1	6.00E-129	0.620795	0.363	0.168	1.47E-124	Classical M	Slc9a3r1
Rtcb1	1.13E-128	0.609221	0.543	0.297	2.78E-124	Classical M	Rtcb
Atp5g11	1.84E-128	0.480015	0.734	0.455	4.51E-124	Classical M	Atp5g1

Eif4b1	3.38E-128	0.607455	0.512	0.275	8.27E-124	Classical M	Eif4b
Tmem2561	3.94E-128	0.52997	0.558	0.307	9.65E-124	Classical M	Tmem256
Nfkb2	9.41E-128	1.005323	0.252	0.101	2.30E-123	Classical M	Nfkb2
Atp6v1b21	1.28E-127	0.578261	0.58	0.325	3.14E-123	Classical M	Atp6v1b2
Psmb51	1.97E-127	0.523961	0.587	0.328	4.83E-123	Classical M	Psmb5
Gbp7	5.07E-127	0.875447	0.379	0.183	1.24E-122	Classical M	Gbp7
Snrpd1	1.06E-126	0.656354	0.411	0.203	2.58E-122	Classical M	Snrpd1
Msn	1.08E-126	0.560327	0.832	0.58	2.65E-122	Classical M	Msn
Vasp	1.58E-126	0.636017	0.547	0.307	3.88E-122	Classical M	Vasp
Ap1s2	2.20E-126	0.607386	0.349	0.159	5.38E-122	Classical M	Ap1s2
Dapk1	3.91E-126	0.565989	0.298	0.127	9.57E-122	Classical M	Dapk1
Smim14	4.17E-126	0.515753	0.541	0.294	1.02E-121	Classical M	Smim14
Sub1	5.07E-126	0.516037	0.747	0.477	1.24E-121	Classical M	Sub1
Mrps16	7.35E-126	0.628407	0.398	0.194	1.80E-121	Classical M	Mrps16
Polr1d1	1.00E-125	0.503991	0.688	0.41	2.46E-121	Classical M	Polr1d
Mrpl522	1.83E-125	0.551034	0.728	0.46	4.47E-121	Classical M	Mrpl52
Churc1	4.70E-125	0.659598	0.339	0.156	1.15E-120	Classical M	Churc1
Eif2b2	8.44E-125	0.980043	0.259	0.107	2.07E-120	Classical M	Eif2b2
Dctn3	8.68E-125	0.536182	0.502	0.266	2.13E-120	Classical M	Dctn3
Tent5a	1.88E-124	0.591232	0.471	0.243	4.61E-120	Classical M	Tent5a
Rps252	4.95E-124	0.464759	0.883	0.626	1.21E-119	Classical M	Rps25
Arhgdia1	6.86E-124	0.46415	0.811	0.52	1.68E-119	Classical M	Arhgdia
Snx101	8.81E-124	0.558867	0.471	0.246	2.16E-119	Classical M	Snx10
Gng51	8.83E-124	0.419443	0.931	0.709	2.16E-119	Classical M	Gng5
Taldo11	1.10E-123	0.432763	0.734	0.47	2.70E-119	Classical M	Taldo1
Rpl13a2	1.12E-123	0.38118	0.975	0.784	2.74E-119	Classical M	Rpl13a
Hnrnpf	2.32E-123	0.476435	0.78	0.499	5.67E-119	Classical M	Hnrnpf
Kxd11	4.75E-123	0.584568	0.454	0.235	1.16E-118	Classical M	Kxd1
S100a12	6.15E-123	0.323529	0.371	0.173	1.51E-118	Classical M	S100a1
Pfdn52	2.82E-122	0.424947	0.92	0.679	6.90E-118	Classical M	Pfdn5
Polr2f1	6.78E-122	0.576744	0.385	0.187	1.66E-117	Classical M	Polr2f
Fcer1g2	1.29E-121	0.354129	0.997	0.9	3.16E-117	Classical M	Fcer1g
Bola21	2.33E-121	0.616519	0.429	0.219	5.70E-117	Classical M	Bola2
Pdcd6	1.87E-120	0.596996	0.397	0.197	4.57E-116	Classical M	Pdcd6
Tmem219	5.83E-120	0.539258	0.566	0.319	1.43E-115	Classical M	Tmem219
Sap18	5.98E-120	0.509731	0.517	0.28	1.47E-115	Classical M	Sap18
Mrps14	9.18E-120	0.526512	0.459	0.239	2.25E-115	Classical M	Mrps14
Abhd17a1	2.27E-119	0.595765	0.431	0.222	5.57E-115	Classical M	Abhd17a
Pomp2	2.28E-119	0.497051	0.815	0.537	5.59E-115	Classical M	Pomp
Alyref	3.67E-119	0.557772	0.405	0.201	8.99E-115	Classical M	Alyref
Rex1bd1	1.02E-118	0.528772	0.443	0.229	2.50E-114	Classical M	Rex1bd
Mrpl14	1.83E-118	0.540544	0.419	0.212	4.47E-114	Classical M	Mrpl14
Hnrnpa11	1.87E-118	0.50718	0.631	0.373	4.59E-114	Classical M	Hnrnpa1
Ptprc1	2.25E-118	0.470738	0.821	0.563	5.52E-114	Classical M	Ptprc

Mta3	2.94E-118	0.519297	0.34	0.157	7.20E-114	Classical M Mta3
Snx3	6.64E-118	0.43782	0.706	0.431	1.63E-113	Classical M Snx3
Cox4i12	1.63E-117	0.37046	0.972	0.795	3.99E-113	Classical M Cox4i1
Zmiz2	2.24E-117	0.696916	0.292	0.128	5.49E-113	Classical M Zmiz2
Cdk8	4.18E-117	0.522035	0.981	0.898	1.02E-112	Classical M Cdk8
Prr13	4.70E-117	0.343023	0.574	0.323	1.15E-112	Classical M Prr13
Ehbp1l1	1.10E-116	0.555128	0.388	0.191	2.68E-112	Classical M Ehbp1l1
Uba522	1.61E-116	0.530512	0.714	0.448	3.94E-112	Classical M Uba52
Atp5k1	1.73E-116	0.629762	0.401	0.203	4.23E-112	Classical M Atp5k
Rnh11	2.33E-116	0.574597	0.584	0.339	5.70E-112	Classical M Rnh1
Atp5g22	3.30E-116	0.420621	0.917	0.676	8.07E-112	Classical M Atp5g2
Gltp2	7.75E-116	0.480464	0.632	0.367	1.90E-111	Classical M Gltp
Chchd21	1.17E-115	0.547893	0.606	0.363	2.86E-111	Classical M Chchd2
Cct51	4.54E-115	0.541593	0.482	0.26	1.11E-110	Classical M Cct5
Atp6v1f2	8.67E-115	0.410517	0.836	0.545	2.12E-110	Classical M Atp6v1f
Nudt21	1.18E-114	0.626695	0.35	0.168	2.89E-110	Classical M Nudt21
Hnrnpab1	1.35E-114	0.497879	0.578	0.329	3.32E-110	Classical M Hnrnpab
Ubxn11	1.40E-114	0.440792	0.645	0.375	3.43E-110	Classical M Ubxn1
Impdh21	1.71E-114	0.708478	0.343	0.164	4.18E-110	Classical M Impdh2
Cyth1	1.91E-114	0.534333	0.327	0.151	4.67E-110	Classical M Cyth1
Mrps361	2.34E-114	0.623029	0.359	0.175	5.73E-110	Classical M Mrps36
Fkbp1a	3.36E-114	0.449661	0.574	0.325	8.22E-110	Classical M Fkbp1a
Cox7c1	3.94E-114	0.483381	0.81	0.563	9.65E-110	Classical M Cox7c
Set	4.54E-114	0.511568	0.545	0.308	1.11E-109	Classical M Set
Cd481	7.10E-114	0.49726	0.653	0.392	1.74E-109	Classical M Cd48
Pde4b	9.52E-114	0.737301	0.425	0.224	2.33E-109	Classical M Pde4b
Snrpd21	9.86E-114	0.504251	0.474	0.254	2.41E-109	Classical M Snrpd2
Bcl7c	1.16E-113	0.681274	0.296	0.133	2.84E-109	Classical M Bcl7c
Rbm8a	2.16E-113	0.607409	0.369	0.182	5.29E-109	Classical M Rbm8a
Dnaja1	2.49E-113	0.52601	0.511	0.283	6.09E-109	Classical M Dnaja1
Hivep2	4.00E-113	0.62253	0.263	0.112	9.79E-109	Classical M Hivep2
Rbx1	5.24E-113	0.443648	0.723	0.452	1.28E-108	Classical M Rbx1
Ndufv2	7.53E-113	0.467179	0.515	0.285	1.84E-108	Classical M Ndufv2
Tmpo	1.07E-112	0.815612	0.332	0.159	2.63E-108	Classical M Tmpo
Hmga11	1.27E-112	0.775916	0.296	0.135	3.12E-108	Classical M Hmga1
Snrpd3	2.20E-112	0.59945	0.439	0.233	5.39E-108	Classical M Snrpd3
Znrd1	2.57E-112	0.603364	0.355	0.171	6.30E-108	Classical M Znrd1
Tmem1601	4.59E-112	0.509117	0.511	0.283	1.12E-107	Classical M Tmem160
Cct41	3.85E-111	0.517033	0.53	0.297	9.44E-107	Classical M Cct4
Ndufa131	4.04E-111	0.451682	0.79	0.524	9.90E-107	Classical M Ndufa13
Ranbp11	4.41E-111	0.501581	0.48	0.26	1.08E-106	Classical M Ranbp1
Psmd81	1.01E-110	0.506538	0.546	0.307	2.48E-106	Classical M Psmd8
Sra11	1.03E-110	0.567099	0.38	0.19	2.53E-106	Classical M Sra1
Ccnd1	1.73E-110	0.836083	0.394	0.207	4.24E-106	Classical M Ccnd1

Igtp	1.99E-110	0.761174	0.403	0.211	4.87E-106	Classical M	Igtp
Eif3d	2.40E-110	0.5924	0.392	0.199	5.88E-106	Classical M	Eif3d
Mrpl281	9.06E-110	0.615487	0.307	0.142	2.22E-105	Classical M	Mrpl28
Sys11	1.49E-109	0.525554	0.518	0.292	3.64E-105	Classical M	Sys1
Plekhf2	1.92E-109	0.726846	0.351	0.174	4.69E-105	Classical M	Plekhf2
Ethe11	2.54E-109	0.813779	0.284	0.129	6.21E-105	Classical M	Ethe1
Iah11	2.64E-109	0.659048	0.305	0.141	6.47E-105	Classical M	Iah1
Dph3	3.01E-109	0.665667	0.295	0.135	7.38E-105	Classical M	Dph3
Rtraf2	3.05E-109	0.442356	0.673	0.407	7.46E-105	Classical M	Rtraf
Ube2a	4.74E-109	0.619454	0.417	0.219	1.16E-104	Classical M	Ube2a
Blvra1	1.19E-108	0.532383	0.363	0.177	2.92E-104	Classical M	Blvra
Uba1	1.49E-108	0.560023	0.391	0.198	3.65E-104	Classical M	Uba1
Polr2j	1.63E-108	0.53007	0.386	0.194	3.99E-104	Classical M	Polr2j
Naaa	5.39E-108	0.793594	0.37	0.189	1.32E-103	Classical M	Naaa
Prkcd1	6.52E-108	0.694238	0.744	0.515	1.60E-103	Classical M	Prkcd
Dnajc15	1.96E-107	0.555437	0.384	0.196	4.80E-103	Classical M	Dnajc15
Ywhab	2.64E-107	0.428481	0.584	0.337	6.46E-103	Classical M	Ywhab
Rassf4	5.12E-107	0.551366	0.615	0.375	1.25E-102	Classical M	Rassf4
Mrps21	6.36E-107	0.423819	0.579	0.334	1.56E-102	Classical M	Mrps21
1810058I2	1.94E-106	0.403768	0.471	0.253	4.76E-102	Classical M	1810058I24Rik
Acsl1	2.47E-106	0.726025	0.321	0.153	6.04E-102	Classical M	Acsl1
Mkrn1	3.24E-106	0.287713	0.52	0.29	7.93E-102	Classical M	Mkrn1
Cdc371	4.04E-106	0.437352	0.553	0.314	9.89E-102	Classical M	Cdc37
Nab1	4.16E-106	0.711949	0.277	0.125	1.02E-101	Classical M	Nab1
Ccl21	5.32E-106	0.747169	0.303	0.144	1.30E-101	Classical M	Ccl2
Gngt2	5.43E-106	0.45199	0.699	0.45	1.33E-101	Classical M	Gngt2
Uqcrh2	6.75E-106	0.40871	0.851	0.587	1.65E-101	Classical M	Uqcrh
Map1lc3b	2.81E-105	0.391956	0.576	0.335	6.87E-101	Classical M	Map1lc3b
Gas5	3.26E-105	0.458367	0.803	0.546	7.98E-101	Classical M	Gas5
Mrpl18	3.37E-105	0.52481	0.372	0.187	8.25E-101	Classical M	Mrpl18
Serbp11	5.70E-105	0.39442	0.656	0.394	1.40E-100	Classical M	Serbp1
Tor3a1	7.17E-105	0.561438	0.499	0.281	1.76E-100	Classical M	Tor3a
Ndufb21	7.66E-105	0.482044	0.443	0.237	1.88E-100	Classical M	Ndufb2
Gnai2	1.16E-104	0.377721	0.953	0.774	2.85E-100	Classical M	Gnai2
Rac21	2.07E-104	0.32542	0.708	0.445	5.07E-100	Classical M	Rac2
Eif3a1	3.43E-104	0.443042	0.571	0.332	8.41E-100	Classical M	Eif3a
Fundc21	4.71E-104	0.604551	0.32	0.154	1.15E-99	Classical M	Fundc2
Ninj11	5.16E-104	0.714402	0.534	0.32	1.26E-99	Classical M	Ninj1
Lman2	6.68E-104	0.4578	0.52	0.294	1.63E-99	Classical M	Lman2
Pole4	7.20E-104	0.556682	0.326	0.157	1.76E-99	Classical M	Pole4
Mrpl361	1.20E-103	0.561652	0.372	0.189	2.93E-99	Classical M	Mrpl36
Csnk2b1	2.37E-103	0.470973	0.56	0.326	5.80E-99	Classical M	Csnk2b
Cox6c1	3.90E-103	0.411987	0.81	0.568	9.55E-99	Classical M	Cox6c
Uqcc21	4.38E-103	0.439647	0.495	0.276	1.07E-98	Classical M	Uqcc2

Psmb61	5.99E-103	0.416033	0.62	0.367	1.47E-98	Classical M	Psmb6
Tcp11	6.12E-103	0.500592	0.447	0.241	1.50E-98	Classical M	Tcp1
Tmbim1	2.73E-102	0.803654	0.255	0.113	6.69E-98	Classical M	Tmbim1
Ndufa3	3.11E-102	0.501443	0.604	0.37	7.61E-98	Classical M	Ndufa3
Znfx1	3.79E-102	0.584344	0.472	0.265	9.29E-98	Classical M	Znfx1
Gatm2	3.93E-102	0.909925	0.591	0.386	9.62E-98	Classical M	Gatm
Snrpc	4.44E-102	0.458412	0.427	0.227	1.09E-97	Classical M	Snrpc
Mrpl57	6.24E-102	0.452678	0.397	0.205	1.53E-97	Classical M	Mrpl57
Sdhd1	1.17E-101	0.484893	0.479	0.267	2.88E-97	Classical M	Sdhd
Ndufaf8	1.24E-101	0.527067	0.346	0.172	3.05E-97	Classical M	Ndufaf8
Supt4a	1.29E-101	0.422922	0.523	0.297	3.16E-97	Classical M	Supt4a
Rexo21	1.93E-101	0.494039	0.412	0.218	4.72E-97	Classical M	Rexo2
Cct21	2.23E-101	0.461263	0.512	0.289	5.47E-97	Classical M	Cct2
Mrpl21	2.69E-101	0.639798	0.291	0.136	6.60E-97	Classical M	Mrpl21
Pfdn6	3.12E-101	0.758644	0.266	0.12	7.64E-97	Classical M	Pfdn6
Coa51	5.19E-101	0.684483	0.298	0.141	1.27E-96	Classical M	Coa5
Tagap1	5.27E-101	0.570797	0.445	0.243	1.29E-96	Classical M	Tagap
Brd2	9.43E-101	0.445112	0.466	0.255	2.31E-96	Classical M	Brd2
Ndufa71	9.89E-101	0.377996	0.744	0.484	2.42E-96	Classical M	Ndufa7
Pcbp12	1.52E-100	0.440824	0.776	0.515	3.73E-96	Classical M	Pcbp1
Eif1ax	2.80E-100	0.705366	0.27	0.124	6.86E-96	Classical M	Eif1ax
Amdhd21	6.33E-100	0.645703	0.336	0.167	1.55E-95	Classical M	Amdhd2
Nin	6.45E-100	0.494323	0.289	0.133	1.58E-95	Classical M	Nin
Ndufb3	1.10E-99	0.380165	0.522	0.295	2.69E-95	Classical M	Ndufb3
Mif2	1.57E-99	0.698123	0.662	0.424	3.84E-95	Classical M	Mif
Ndufa14	1.99E-99	0.457617	0.549	0.321	4.87E-95	Classical M	Ndufa1
Ndufb10	3.44E-99	0.369032	0.642	0.389	8.41E-95	Classical M	Ndufb10
Cct71	6.27E-99	0.494195	0.459	0.252	1.54E-94	Classical M	Cct7
Mtpn	6.29E-99	0.533886	0.41	0.22	1.54E-94	Classical M	Mtpn
Cwc15	6.97E-99	0.382096	0.529	0.301	1.71E-94	Classical M	Cwc15
Hdgf1	8.20E-99	0.566478	0.332	0.166	2.01E-94	Classical M	Hdgf
Hcfc1r11	1.14E-98	0.407549	0.466	0.256	2.80E-94	Classical M	Hcfc1r1
Psma3	1.68E-98	0.439206	0.657	0.41	4.12E-94	Classical M	Psma3
Ube2v11	2.51E-98	0.387771	0.495	0.275	6.14E-94	Classical M	Ube2v1
Bin3	2.75E-98	0.535961	0.294	0.138	6.73E-94	Classical M	Bin3
Scp2	2.80E-98	0.332727	0.567	0.328	6.86E-94	Classical M	Scp2
Nono	3.57E-98	0.446387	0.498	0.28	8.75E-94	Classical M	Nono
Spr	3.91E-98	0.621353	0.263	0.119	9.59E-94	Classical M	Spr
Rab9	5.30E-98	0.677502	0.26	0.117	1.30E-93	Classical M	Rab9
Eif3c	2.11E-97	0.437165	0.559	0.33	5.16E-93	Classical M	Eif3c
Ntan1	2.71E-97	0.42888	0.351	0.176	6.63E-93	Classical M	Ntan1
Cox6a11	3.30E-97	0.383366	0.761	0.502	8.07E-93	Classical M	Cox6a1
Card19	3.37E-97	0.499142	0.437	0.24	8.26E-93	Classical M	Card19
Rhoa	5.13E-97	0.403253	0.898	0.678	1.26E-92	Classical M	Rhoa

Nfkbiz2	5.24E-97	0.339112	0.451	0.247	1.28E-92	Classical M	Nfkbiz
Ostf1	1.18E-96	0.34399	0.749	0.482	2.88E-92	Classical M	Ostf1
Gtf2h5	1.72E-96	0.445134	0.375	0.195	4.20E-92	Classical M	Gtf2h5
Mrps18c	4.23E-96	0.471304	0.355	0.18	1.04E-91	Classical M	Mrps18c
Snx2	6.49E-96	0.375185	0.557	0.327	1.59E-91	Classical M	Snx2
Sigmar1	6.99E-96	0.633193	0.29	0.138	1.71E-91	Classical M	Sigmar1
Trmt112	1.09E-95	0.376356	0.742	0.474	2.68E-91	Classical M	Trmt112
Idnk	1.26E-95	0.491021	0.398	0.212	3.09E-91	Classical M	Idnk
5-Mar	1.63E-95	0.65055	0.423	0.234	3.99E-91	Classical M	5-Mar
Aif12	3.92E-95	0.450657	0.906	0.694	9.60E-91	Classical M	Aif1
Rwdd1	5.33E-95	0.4938	0.366	0.19	1.31E-90	Classical M	Rwdd1
Slc48a11	7.47E-95	0.37739	0.313	0.152	1.83E-90	Classical M	Slc48a1
Gm118082	1.35E-94	0.502486	0.511	0.296	3.31E-90	Classical M	Gm11808
Bccip	1.69E-94	0.483942	0.301	0.145	4.13E-90	Classical M	Bccip
Isoc1	2.19E-94	0.739769	0.264	0.122	5.36E-90	Classical M	Isoc1
Tgif11	2.63E-94	0.587048	0.326	0.162	6.45E-90	Classical M	Tgif1
Gbp9	2.83E-94	0.820337	0.255	0.116	6.92E-90	Classical M	Gbp9
Atp5f1	2.95E-94	0.401068	0.726	0.472	7.23E-90	Classical M	Atp5f1
Rasa41	8.31E-94	0.411568	0.57	0.336	2.04E-89	Classical M	Rasa4
G6pdx	9.37E-94	0.55103	0.292	0.14	2.29E-89	Classical M	G6pdx
Timm131	1.40E-93	0.41939	0.551	0.327	3.42E-89	Classical M	Timm13
Tbcb1	2.02E-93	0.447773	0.455	0.254	4.94E-89	Classical M	Tbcb
Map7d1	2.96E-93	0.487477	0.4	0.214	7.24E-89	Classical M	Map7d1
Prdx2	3.02E-93	0.334369	0.574	0.341	7.40E-89	Classical M	Prdx2
Rab5c1	3.09E-93	0.435198	0.596	0.361	7.58E-89	Classical M	Rab5c
Txndc91	4.02E-93	0.490322	0.364	0.188	9.84E-89	Classical M	Txndc9
Fbxo61	4.05E-93	0.641767	0.289	0.14	9.91E-89	Classical M	Fbxo6
Hnrnpd	5.22E-93	0.418556	0.546	0.323	1.28E-88	Classical M	Hnrnpd
BC028528	7.28E-93	0.565635	0.441	0.247	1.78E-88	Classical M	BC028528
Ndufc11	1.37E-92	0.430314	0.479	0.275	3.35E-88	Classical M	Ndufc1
Gpsm3	1.42E-92	0.369852	0.618	0.376	3.48E-88	Classical M	Gpsm3
Atp5a11	1.63E-92	0.418946	0.703	0.46	3.99E-88	Classical M	Atp5a1
Vdac3	2.09E-92	0.450351	0.431	0.237	5.12E-88	Classical M	Vdac3
Slfn8	2.64E-92	0.527417	0.369	0.193	6.47E-88	Classical M	Slfn8
Pgam11	3.20E-92	0.580516	0.559	0.339	7.83E-88	Classical M	Pgam1
Cdc42se1	6.52E-92	0.413911	0.482	0.273	1.60E-87	Classical M	Cdc42se1
Pgls1	8.50E-92	0.377762	0.602	0.366	2.08E-87	Classical M	Pgls
Grpel1	1.33E-91	0.491215	0.347	0.179	3.25E-87	Classical M	Grpel1
Cox7a2l2	2.30E-91	0.378651	0.709	0.446	5.64E-87	Classical M	Cox7a2l
Ndufs7	2.32E-91	0.33007	0.62	0.375	5.68E-87	Classical M	Ndufs7
Cmc11	2.41E-91	0.446235	0.392	0.209	5.89E-87	Classical M	Cmc1
Mrpl4	3.21E-91	0.447744	0.386	0.205	7.85E-87	Classical M	Mrpl4
Cib1	3.27E-91	0.532131	0.354	0.183	8.02E-87	Classical M	Cib1
Tpr	6.21E-91	0.348957	0.58	0.344	1.52E-86	Classical M	Tpr

Ndufs61	9.88E-91	0.400772	0.383	0.203	2.42E-86	Classical M	Ndufs6
Tmem167	1.43E-90	0.41159	0.466	0.263	3.51E-86	Classical M	Tmem167
Lsm3	1.54E-90	0.570042	0.29	0.14	3.78E-86	Classical M	Lsm3
Fis11	1.69E-90	0.388232	0.706	0.457	4.14E-86	Classical M	Fis1
Cdc42	1.80E-90	0.354327	0.922	0.721	4.41E-86	Classical M	Cdc42
Slk	1.81E-90	0.467775	0.312	0.154	4.43E-86	Classical M	Slk
Chchd11	2.03E-90	0.469042	0.372	0.196	4.96E-86	Classical M	Chchd1
Ccdc861	2.47E-90	0.761052	0.316	0.161	6.05E-86	Classical M	Ccdc86
Mrpl201	2.52E-90	0.437191	0.447	0.25	6.17E-86	Classical M	Mrpl20
Hprt	3.75E-90	0.367735	0.561	0.335	9.19E-86	Classical M	Hprt
Was	5.20E-90	0.418402	0.426	0.233	1.27E-85	Classical M	Was
Snx1	6.04E-90	0.375984	0.413	0.223	1.48E-85	Classical M	Snx1
Tcof1	9.66E-90	0.606569	0.277	0.133	2.37E-85	Classical M	Tcof1
Gsdmd	1.28E-89	0.44002	0.498	0.288	3.14E-85	Classical M	Gsdmd
Hspe12	1.31E-89	0.402516	0.665	0.416	3.22E-85	Classical M	Hspe1
H13	1.33E-89	0.371646	0.537	0.314	3.27E-85	Classical M	H13
Ndufa21	1.43E-89	0.344385	0.676	0.425	3.50E-85	Classical M	Ndufa2
Mrpl271	1.94E-89	0.527964	0.299	0.147	4.75E-85	Classical M	Mrpl27
Gyg1	2.30E-89	0.642792	0.296	0.147	5.63E-85	Classical M	Gyg
Atp5mpl2	3.08E-89	0.41218	0.646	0.408	7.55E-85	Classical M	Atp5mpl
Idh3b1	4.65E-89	0.464971	0.461	0.264	1.14E-84	Classical M	Idh3b
Lsm7	6.14E-89	0.424975	0.404	0.221	1.50E-84	Classical M	Lsm7
Ndufa121	7.25E-89	0.392022	0.448	0.252	1.77E-84	Classical M	Ndufa12
Ahsa1	7.40E-89	0.446264	0.376	0.2	1.81E-84	Classical M	Ahsa1
Ubl51	7.81E-89	0.375662	0.718	0.472	1.91E-84	Classical M	Ubl5
Mdh21	2.79E-88	0.358015	0.571	0.345	6.83E-84	Classical M	Mdh2
Aip	3.05E-88	0.488238	0.371	0.197	7.47E-84	Classical M	Aip
Aldoa1	3.10E-88	0.54734	0.809	0.585	7.59E-84	Classical M	Aldoa
Rbm421	3.18E-88	0.416031	0.44	0.246	7.79E-84	Classical M	Rbm42
Ndufb52	3.27E-88	0.366727	0.595	0.363	8.00E-84	Classical M	Ndufb5
Soat11	8.69E-88	0.402767	0.484	0.278	2.13E-83	Classical M	Soat1
Gm20001	1.11E-87	0.690484	0.262	0.125	2.71E-83	Classical M	Gm2000
Rab1b	3.16E-87	0.44226	0.448	0.254	7.73E-83	Classical M	Rab1b
Parp9	3.27E-87	0.452719	0.397	0.217	8.00E-83	Classical M	Parp9
Myo1c	3.49E-87	0.523316	0.293	0.143	8.54E-83	Classical M	Myo1c
Ube2q1	4.23E-87	0.415757	0.376	0.201	1.04E-82	Classical M	Ube2q1
Utp3	5.46E-87	0.547151	0.351	0.186	1.34E-82	Classical M	Utp3
Irf51	6.13E-87	0.418154	0.701	0.46	1.50E-82	Classical M	Irf5
Higd1a	7.36E-87	0.568043	0.366	0.196	1.80E-82	Classical M	Higd1a
Pfdn2	1.02E-86	0.322712	0.474	0.267	2.50E-82	Classical M	Pfdn2
Atp5j1	1.05E-86	0.332552	0.787	0.532	2.58E-82	Classical M	Atp5j
Cox17	1.11E-86	0.385856	0.559	0.338	2.72E-82	Classical M	Cox17
Nol7	1.23E-86	0.427556	0.446	0.252	3.00E-82	Classical M	Nol7
Luzp1	1.36E-86	0.394415	0.307	0.153	3.33E-82	Classical M	Luzp1

Adar	2.21E-86	0.525397	0.313	0.159	5.41E-82	Classical M	Adar
Fam50a1	2.23E-86	0.480586	0.334	0.173	5.47E-82	Classical M	Fam50a
Bri31	2.44E-86	0.357532	0.859	0.607	5.99E-82	Classical M	Bri3
Ndufa5	3.71E-86	0.387358	0.41	0.227	9.09E-82	Classical M	Ndufa5
Sumo1	4.32E-86	0.305755	0.536	0.315	1.06E-81	Classical M	Sumo1
Tlr21	4.49E-86	0.583676	0.454	0.268	1.10E-81	Classical M	Tlr2
Cggbp1	4.62E-86	0.404278	0.347	0.18	1.13E-81	Classical M	Cggbp1
Pgd1	7.30E-86	0.341689	0.471	0.27	1.79E-81	Classical M	Pgd
Ppdpf	1.71E-85	0.426788	0.313	0.158	4.18E-81	Classical M	Ppdpf
Cops61	1.91E-85	0.417162	0.411	0.226	4.68E-81	Classical M	Cops6
Timm23	2.85E-85	0.383951	0.462	0.263	6.98E-81	Classical M	Timm23
Szrd1	2.91E-85	0.392104	0.362	0.192	7.14E-81	Classical M	Szrd1
Gm35154	3.43E-85	0.332668	0.272	0.127	8.40E-81	Classical M	Gm35154
Ncl1	4.16E-85	0.344207	0.572	0.347	1.02E-80	Classical M	Ncl
Mrpl401	5.30E-85	0.572702	0.254	0.12	1.30E-80	Classical M	Mrpl40
Fbl1	6.71E-85	0.375356	0.445	0.249	1.64E-80	Classical M	Fbl
Rnf114	9.42E-85	0.439494	0.409	0.226	2.31E-80	Classical M	Rnf114
Gnb11	1.32E-84	0.299539	0.73	0.476	3.24E-80	Classical M	Gnb1
Psmb21	3.41E-84	0.378341	0.587	0.36	8.35E-80	Classical M	Psmb2
Rtf2	1.35E-83	0.422207	0.409	0.228	3.31E-79	Classical M	Rtf2
Ndufa81	1.38E-83	0.327115	0.528	0.314	3.38E-79	Classical M	Ndufa8
Tsc22d31	1.48E-83	0.403945	0.496	0.29	3.63E-79	Classical M	Tsc22d3
Ndufc22	2.42E-83	0.313527	0.746	0.493	5.92E-79	Classical M	Ndufc2
Btg11	3.02E-83	0.437336	0.752	0.519	7.40E-79	Classical M	Btg1
Csk1	3.16E-83	0.330743	0.572	0.348	7.73E-79	Classical M	Csk
Hck1	3.33E-83	0.371768	0.778	0.545	8.15E-79	Classical M	Hck
Cenpx1	3.48E-83	0.329575	0.472	0.271	8.53E-79	Classical M	Cenpx
Ufm11	5.54E-83	0.54662	0.281	0.139	1.36E-78	Classical M	Ufm1
Zc3hav11	7.04E-83	0.376205	0.415	0.231	1.72E-78	Classical M	Zc3hav1
Sin3b1	1.45E-82	0.38173	0.459	0.264	3.54E-78	Classical M	Sin3b
Baz2a	1.77E-82	0.425415	0.288	0.143	4.34E-78	Classical M	Baz2a
Trappc2l1	1.89E-82	0.376915	0.482	0.28	4.62E-78	Classical M	Trappc2l
N4bp11	2.00E-82	0.452764	0.413	0.232	4.91E-78	Classical M	N4bp1
Dbndd21	2.50E-82	0.441805	0.308	0.157	6.12E-78	Classical M	Dbndd2
Mob1a	2.77E-82	0.406549	0.401	0.222	6.79E-78	Classical M	Mob1a
Fdx21	4.04E-82	0.547804	0.28	0.14	9.90E-78	Classical M	Fdx2
Ndufa11	5.02E-82	0.31015	0.563	0.342	1.23E-77	Classical M	Ndufa11
Filip1l	6.31E-82	0.369735	0.698	0.469	1.54E-77	Classical M	Filip1l
Rraga	6.36E-82	0.552409	0.286	0.143	1.56E-77	Classical M	Rraga
Eef1d2	7.46E-82	0.346249	0.742	0.482	1.83E-77	Classical M	Eef1d
Leprotl1	1.36E-81	0.385199	0.463	0.268	3.32E-77	Classical M	Leprotl1
Chrac1	1.40E-81	0.394303	0.413	0.232	3.42E-77	Classical M	Chrac1
Man2a1	1.49E-81	0.334375	0.286	0.141	3.65E-77	Classical M	Man2a1
Arhgap9	1.56E-81	0.374407	0.33	0.171	3.82E-77	Classical M	Arhgap9

Snapc5	2.30E-81	0.383678	0.343	0.181	5.63E-77	Classical M	Snapc5
Myo9b1	2.59E-81	0.25082	0.512	0.299	6.35E-77	Classical M	Myo9b
Cnp	3.20E-81	0.466113	0.323	0.168	7.85E-77	Classical M	Cnp
Ramp1	3.21E-81	0.438481	0.421	0.239	7.87E-77	Classical M	Ramp1
Fam104a	4.41E-81	0.363932	0.406	0.226	1.08E-76	Classical M	Fam104a
Lsm5	4.51E-81	0.563568	0.259	0.125	1.11E-76	Classical M	Lsm5
Ube2d3	4.82E-81	0.366395	0.759	0.516	1.18E-76	Classical M	Ube2d3
Ap1s11	8.34E-81	0.42601	0.314	0.162	2.04E-76	Classical M	Ap1s1
Irf2bp21	9.50E-81	0.358673	0.675	0.437	2.33E-76	Classical M	Irf2bp2
Anxa6	1.38E-80	0.392108	0.338	0.178	3.37E-76	Classical M	Anxa6
Lcp1	1.38E-80	0.325591	0.88	0.68	3.38E-76	Classical M	Lcp1
Rbis	1.98E-80	0.379281	0.4	0.223	4.84E-76	Classical M	Rbis
Elof1	2.10E-80	0.445555	0.298	0.152	5.14E-76	Classical M	Elof1
2610507B1	2.69E-80	0.391308	0.317	0.163	6.59E-76	Classical M	2610507B11Rik
Rheb	3.36E-80	0.286793	0.506	0.298	8.24E-76	Classical M	Rheb
Tubb52	5.30E-80	0.376931	0.694	0.459	1.30E-75	Classical M	Tubb5
Slc25a32	5.39E-80	0.34529	0.851	0.6	1.32E-75	Classical M	Slc25a3
Tap2	1.02E-79	0.431461	0.596	0.38	2.49E-75	Classical M	Tap2
Cct81	2.33E-79	0.394563	0.436	0.25	5.72E-75	Classical M	Cct8
Mrpl42	2.42E-79	0.309935	0.405	0.226	5.92E-75	Classical M	Mrpl42
Ccrl21	2.97E-79	0.847802	0.285	0.147	7.28E-75	Classical M	Ccrl2
Psmd21	2.99E-79	0.386074	0.356	0.191	7.33E-75	Classical M	Psmd2
Il4ra	3.22E-79	0.395791	0.411	0.231	7.90E-75	Classical M	Il4ra
Nfkbib1	3.48E-79	0.545713	0.328	0.175	8.52E-75	Classical M	Nfkbib
Cript	3.74E-79	0.412966	0.337	0.179	9.17E-75	Classical M	Cript
Uqcrc1	5.07E-79	0.326944	0.545	0.33	1.24E-74	Classical M	Uqcrc1
1110038B1	5.33E-79	0.495994	0.35	0.19	1.30E-74	Classical M	1110038B12Rik
Mrpl121	7.79E-79	0.431237	0.361	0.198	1.91E-74	Classical M	Mrpl12
Spi1	8.06E-79	0.391545	0.902	0.695	1.97E-74	Classical M	Spi1
Dnajc19	1.74E-78	0.317399	0.464	0.269	4.25E-74	Classical M	Dnajc19
Ehd4	2.13E-78	0.361883	0.62	0.402	5.23E-74	Classical M	Ehd4
Ndufs41	2.46E-78	0.319486	0.493	0.291	6.02E-74	Classical M	Ndufs4
Rnf149	2.83E-78	0.359234	0.405	0.228	6.92E-74	Classical M	Rnf149
Med281	3.11E-78	0.294081	0.577	0.348	7.62E-74	Classical M	Med28
Atp6v1e1	4.67E-78	0.315616	0.483	0.281	1.14E-73	Classical M	Atp6v1e1
Scpep11	4.99E-78	0.340517	0.596	0.365	1.22E-73	Classical M	Scpep1
Abr1	5.46E-78	0.25946	0.504	0.305	1.34E-73	Classical M	Abr
3830406C1	7.96E-78	0.428428	0.254	0.122	1.95E-73	Classical M	3830406C13Rik
Bcl2a1a1	8.63E-78	0.894873	0.259	0.13	2.11E-73	Classical M	Bcl2a1a
Slc25a12	1.33E-77	0.384511	0.25	0.12	3.25E-73	Classical M	Slc25a12
Bloc1s2	1.36E-77	0.524536	0.287	0.146	3.34E-73	Classical M	Bloc1s2
Syngr21	1.42E-77	0.372459	0.793	0.558	3.48E-73	Classical M	Syngr2
Snw1	2.37E-77	0.352464	0.388	0.216	5.80E-73	Classical M	Snw1
Snf8	2.94E-77	0.404976	0.391	0.219	7.20E-73	Classical M	Snf8

Otub1	3.02E-77	0.389317	0.378	0.209	7.40E-73	Classical M	Otub1
Uap1l11	3.11E-77	0.50357	0.316	0.167	7.61E-73	Classical M	Uap1l1
Chmp1a	3.63E-77	0.474314	0.297	0.153	8.90E-73	Classical M	Chmp1a
Litaf1	4.16E-77	0.33193	0.607	0.385	1.02E-72	Classical M	Litaf
Snx20	4.54E-77	0.334003	0.526	0.317	1.11E-72	Classical M	Snx20
Lsm8	6.92E-77	0.466866	0.303	0.157	1.69E-72	Classical M	Lsm8
Tbca1	8.21E-77	0.290419	0.656	0.416	2.01E-72	Classical M	Tbca
Mrpl58	1.37E-76	0.454511	0.291	0.149	3.36E-72	Classical M	Mrpl58
Casp1	1.52E-76	0.447423	0.328	0.174	3.71E-72	Classical M	Casp1
Mrpl32	1.65E-76	0.526689	0.272	0.136	4.04E-72	Classical M	Mrpl32
Gm100761	1.80E-76	0.604907	0.251	0.123	4.42E-72	Classical M	Gm10076
Tlr9	2.82E-76	0.572898	0.254	0.124	6.90E-72	Classical M	Tlr9
Rbm17	2.96E-76	0.376757	0.324	0.171	7.25E-72	Classical M	Rbm17
Fcf1	3.15E-76	0.336875	0.38	0.209	7.70E-72	Classical M	Fcf1
Cyp27a1	3.53E-76	0.411186	0.269	0.133	8.66E-72	Classical M	Cyp27a1
Bnip2	4.07E-76	0.302893	0.488	0.288	9.98E-72	Classical M	Bnip2
Rab201	7.15E-76	0.645557	0.332	0.183	1.75E-71	Classical M	Rab20
Dek	7.87E-76	0.39984	0.426	0.246	1.93E-71	Classical M	Dek
Ndufb4	1.50E-75	0.294132	0.576	0.356	3.69E-71	Classical M	Ndufb4
Mrpl341	1.86E-75	0.426325	0.323	0.173	4.55E-71	Classical M	Mrpl34
Drap1	2.77E-75	0.278128	0.555	0.34	6.79E-71	Classical M	Drap1
Anapc11	3.06E-75	0.310642	0.554	0.341	7.49E-71	Classical M	Anapc11
Tmem2581	4.61E-75	0.343445	0.622	0.398	1.13E-70	Classical M	Tmem258
Tnfsf121	6.25E-75	0.421347	0.398	0.227	1.53E-70	Classical M	Tnfsf12
Rpl22l12	6.58E-75	0.317795	0.73	0.487	1.61E-70	Classical M	Rpl22l1
Coro1c1	7.82E-75	0.345872	0.348	0.19	1.91E-70	Classical M	Coro1c
Ccdc88a	1.12E-74	0.355465	0.325	0.172	2.75E-70	Classical M	Ccdc88a
Cope1	1.22E-74	0.362835	0.598	0.376	2.99E-70	Classical M	Cope
Ripk1	1.43E-74	0.431814	0.258	0.127	3.50E-70	Classical M	Ripk1
Med10	1.49E-74	0.447123	0.291	0.15	3.66E-70	Classical M	Med10
Cyb5r1	1.81E-74	0.470859	0.328	0.177	4.43E-70	Classical M	Cyb5r1
Eif3g1	1.87E-74	0.452896	0.33	0.178	4.58E-70	Classical M	Eif3g
Tln1	2.12E-74	0.322889	0.709	0.467	5.19E-70	Classical M	Tln1
Gm48099	2.64E-74	0.2982	0.45	0.266	6.46E-70	Classical M	Gm48099
Trappc4	3.20E-74	0.286307	0.431	0.247	7.84E-70	Classical M	Trappc4
Ddx24	3.24E-74	0.274779	0.521	0.313	7.95E-70	Classical M	Ddx24
Ndufb91	3.61E-74	0.26243	0.681	0.439	8.84E-70	Classical M	Ndufb9
Rgs12	3.69E-74	0.987424	0.376	0.231	9.04E-70	Classical M	Rgs1
Dynlrb1	3.80E-74	0.283306	0.518	0.313	9.30E-70	Classical M	Dynlrb1
Baz1a	3.92E-74	0.255643	0.386	0.214	9.61E-70	Classical M	Baz1a
Tor1a	4.00E-74	0.337313	0.367	0.202	9.78E-70	Classical M	Tor1a
Lat23	4.20E-74	0.325604	0.51	0.305	1.03E-69	Classical M	Lat2
Spop	5.68E-74	0.278507	0.565	0.351	1.39E-69	Classical M	Spop
Ube2s	6.70E-74	0.296866	0.43	0.247	1.64E-69	Classical M	Ube2s

Acaa1a1	6.83E-74	0.354031	0.441	0.258	1.67E-69	Classical M	Acaa1a
Snrnp27	7.16E-74	0.421777	0.298	0.155	1.75E-69	Classical M	Snrnp27
Mrps18a	7.44E-74	0.330083	0.351	0.191	1.82E-69	Classical M	Mrps18a
Klf21	1.02E-73	0.362313	0.39	0.219	2.50E-69	Classical M	Klf2
Pabpc12	1.28E-73	0.302772	0.853	0.612	3.12E-69	Classical M	Pabpc1
Slirp1	2.57E-73	0.442047	0.3	0.157	6.29E-69	Classical M	Slirp
Rnf187	3.09E-73	0.316882	0.463	0.274	7.56E-69	Classical M	Rnf187
Phb21	3.95E-73	0.317873	0.496	0.295	9.66E-69	Classical M	Phb2
Cdc341	4.40E-73	0.467939	0.27	0.137	1.08E-68	Classical M	Cdc34
Tmem50b2	5.51E-73	0.435207	0.403	0.233	1.35E-68	Classical M	Tmem50b
Parp1	7.83E-73	0.405709	0.305	0.161	1.92E-68	Classical M	Parp1
Dnajb14	8.54E-73	0.297852	0.414	0.235	2.09E-68	Classical M	Dnajb14
Nipsnap3b	1.64E-72	0.503245	0.266	0.135	4.01E-68	Classical M	Nipsnap3b
Myh9	1.76E-72	0.360614	0.803	0.575	4.30E-68	Classical M	Myh9
Ssrp11	2.03E-72	0.439605	0.303	0.16	4.97E-68	Classical M	Ssrp1
Zfp800	2.13E-72	0.527336	0.252	0.126	5.22E-68	Classical M	Zfp800
Polr2k	2.67E-72	0.392226	0.313	0.167	6.55E-68	Classical M	Polr2k
Lamtor11	3.49E-72	0.313048	0.646	0.42	8.55E-68	Classical M	Lamtor1
Ddhd11	3.52E-72	0.611409	0.264	0.136	8.62E-68	Classical M	Ddhd1
Ier31	4.97E-72	0.689589	0.278	0.146	1.22E-67	Classical M	Ier3
Ccdc121	5.38E-72	0.302828	0.676	0.442	1.32E-67	Classical M	Ccdc12
Psmc31	1.06E-71	0.274564	0.48	0.285	2.60E-67	Classical M	Psmc3
Ier3ip1	1.15E-71	0.318518	0.459	0.273	2.80E-67	Classical M	Ier3ip1
Timm17a1	1.22E-71	0.366979	0.305	0.162	3.00E-67	Classical M	Timm17a
Ppp1r9b	1.40E-71	0.284466	0.391	0.222	3.43E-67	Classical M	Ppp1r9b
Irf9	2.14E-71	0.437748	0.31	0.166	5.23E-67	Classical M	Irf9
Pcbd2	2.54E-71	0.25714	0.29	0.15	6.23E-67	Classical M	Pcbd2
Dnajc21	2.65E-71	0.393077	0.305	0.162	6.49E-67	Classical M	Dnajc2
Cndp21	3.09E-71	0.329358	0.515	0.315	7.56E-67	Classical M	Cndp2
5031439G(	3.54E-71	0.278596	0.408	0.233	8.67E-67	Classical M	5031439G07Rik
2310011J0	3.81E-71	0.50715	0.266	0.136	9.32E-67	Classical M	2310011J03Rik
Chmp5	4.63E-71	0.289459	0.382	0.215	1.13E-66	Classical M	Chmp5
Slc31a21	5.14E-71	0.487263	0.382	0.22	1.26E-66	Classical M	Slc31a2
Babam1	5.85E-71	0.428221	0.316	0.171	1.43E-66	Classical M	Babam1
Sugt1	6.03E-71	0.36176	0.361	0.203	1.48E-66	Classical M	Sugt1
Eif2ak2	9.48E-71	0.416587	0.38	0.216	2.32E-66	Classical M	Eif2ak2
Aimp11	1.08E-70	0.347328	0.396	0.228	2.65E-66	Classical M	Aimp1
Rassf1	1.25E-70	0.374027	0.349	0.192	3.05E-66	Classical M	Rassf1
Pdcd6ip	1.40E-70	0.287219	0.444	0.259	3.44E-66	Classical M	Pdcd6ip
Nfkbia2	1.65E-70	0.390045	0.699	0.479	4.04E-66	Classical M	Nfkbia
Gsto11	2.38E-70	0.555289	0.262	0.135	5.82E-66	Classical M	Gsto1
Bag11	2.56E-70	0.288075	0.495	0.3	6.27E-66	Classical M	Bag1
U2af1	2.72E-70	0.298359	0.463	0.277	6.66E-66	Classical M	U2af1
Ppp2r1a	4.06E-70	0.348888	0.398	0.229	9.93E-66	Classical M	Ppp2r1a

Ube2n	5.48E-70	0.289014	0.455	0.271	1.34E-65	Classical M	Ube2n
Asna11	5.80E-70	0.382443	0.305	0.164	1.42E-65	Classical M	Asna1
Brk11	6.28E-70	0.28463	0.72	0.475	1.54E-65	Classical M	Brk1
Prelid3b1	7.33E-70	0.476408	0.277	0.144	1.80E-65	Classical M	Prelid3b
G3bp2	9.91E-70	0.3166	0.528	0.328	2.43E-65	Classical M	G3bp2
Eif4h	1.05E-69	0.260731	0.483	0.29	2.58E-65	Classical M	Eif4h
Pfdn11	1.40E-69	0.325322	0.34	0.187	3.43E-65	Classical M	Pfdn1
Ctdnep11	1.80E-69	0.458933	0.37	0.212	4.41E-65	Classical M	Ctdnep1
Clcn71	1.84E-69	0.47622	0.28	0.146	4.50E-65	Classical M	Clcn7
Etfa	2.16E-69	0.264089	0.376	0.211	5.28E-65	Classical M	Etfa
Glrx2	4.01E-69	0.485845	0.265	0.136	9.81E-65	Classical M	Glrx2
Lypla2	4.91E-69	0.393623	0.323	0.177	1.20E-64	Classical M	Lypla2
Ilk	5.18E-69	0.274657	0.473	0.283	1.27E-64	Classical M	Ilk
Eif2s1	5.19E-69	0.347446	0.358	0.2	1.27E-64	Classical M	Eif2s1
Sf3b2	6.39E-69	0.260191	0.58	0.365	1.57E-64	Classical M	Sf3b2
Rcc2	6.82E-69	0.416908	0.285	0.149	1.67E-64	Classical M	Rcc2
Sfr1	7.98E-69	0.299435	0.43	0.252	1.96E-64	Classical M	Sfr1
Clec4a21	8.25E-69	0.408869	0.426	0.252	2.02E-64	Classical M	Clec4a2
Rfc21	9.49E-69	0.438652	0.323	0.177	2.33E-64	Classical M	Rfc2
Psmd4	1.70E-68	0.270741	0.429	0.251	4.16E-64	Classical M	Psmd4
Smarcd2	2.48E-68	0.478311	0.264	0.136	6.06E-64	Classical M	Smarcd2
Osgep	4.18E-68	0.315221	0.372	0.21	1.02E-63	Classical M	Osgep
Necap21	6.09E-68	0.331875	0.474	0.287	1.49E-63	Classical M	Necap2
Coa31	6.93E-68	0.26341	0.463	0.276	1.70E-63	Classical M	Coa3
Tmem14c3	9.15E-68	0.312292	0.651	0.432	2.24E-63	Classical M	Tmem14c
Dnaja2	1.10E-67	0.31882	0.483	0.296	2.69E-63	Classical M	Dnaja2
Zmat5	1.24E-67	0.388883	0.3	0.162	3.03E-63	Classical M	Zmat5
Polr2i	1.94E-67	0.354954	0.315	0.171	4.74E-63	Classical M	Polr2i
Max	2.60E-67	0.283072	0.433	0.255	6.36E-63	Classical M	Max
Ubald2	6.14E-67	0.295206	0.467	0.28	1.50E-62	Classical M	Ubald2
Ciao2b1	9.96E-67	0.350083	0.402	0.237	2.44E-62	Classical M	Ciao2b
Cyb5r31	1.68E-66	0.4462	0.265	0.138	4.12E-62	Classical M	Cyb5r3
Adam9	1.90E-66	0.373237	0.255	0.13	4.65E-62	Classical M	Adam9
Aph1a2	2.04E-66	0.314863	0.474	0.288	5.00E-62	Classical M	Aph1a
Syf2	2.06E-66	0.265815	0.401	0.233	5.03E-62	Classical M	Syf2
Eny2	3.12E-66	0.327191	0.288	0.153	7.64E-62	Classical M	Eny2
Arpp19	3.67E-66	0.289332	0.498	0.307	8.99E-62	Classical M	Arpp19
C1d	5.23E-66	0.321407	0.35	0.196	1.28E-61	Classical M	C1d
Smim4	5.91E-66	0.376658	0.252	0.129	1.45E-61	Classical M	Smim4
Sharpin	6.84E-66	0.361634	0.361	0.205	1.68E-61	Classical M	Sharpin
Ndufs2	7.80E-66	0.257125	0.438	0.26	1.91E-61	Classical M	Ndufs2
Ndufv31	8.23E-66	0.294101	0.569	0.365	2.02E-61	Classical M	Ndufv3
Pa2g41	2.05E-65	0.343613	0.394	0.231	5.01E-61	Classical M	Pa2g4
Commd1	2.08E-65	0.263575	0.369	0.212	5.10E-61	Classical M	Commd1

Ube2i	2.35E-65	0.252774	0.639	0.413	5.76E-61	Classical M	Ube2i
Eif4a12	5.20E-65	0.31025	0.797	0.565	1.27E-60	Classical M	Eif4a1
Herc6	6.63E-65	0.542747	0.292	0.159	1.62E-60	Classical M	Herc6
Tmem179b	7.05E-65	0.270832	0.625	0.407	1.73E-60	Classical M	Tmem179b
Ctbp1	1.41E-64	0.2629	0.405	0.238	3.45E-60	Classical M	Ctbp1
Trir	1.92E-64	0.273966	0.55	0.345	4.70E-60	Classical M	Trir
Micos10	2.18E-64	0.307386	0.517	0.327	5.34E-60	Classical M	Micos10
Flii	2.32E-64	0.293624	0.387	0.225	5.67E-60	Classical M	Flii
Rab5if1	2.57E-64	0.25961	0.602	0.387	6.28E-60	Classical M	Rab5if
Micos131	3.89E-64	0.262419	0.502	0.31	9.54E-60	Classical M	Micos13
Igsf81	4.65E-64	0.336311	0.382	0.223	1.14E-59	Classical M	Igsf8
Ngdn	5.06E-64	0.431119	0.266	0.14	1.24E-59	Classical M	Ngdn
Sh3bp11	8.63E-64	0.337907	0.489	0.304	2.11E-59	Classical M	Sh3bp1
Ubl7	1.18E-63	0.457593	0.261	0.137	2.89E-59	Classical M	Ubl7
Psmc41	1.19E-63	0.374665	0.343	0.196	2.91E-59	Classical M	Psmc4
Polr2g1	1.82E-63	0.319145	0.379	0.221	4.46E-59	Classical M	Polr2g
Pafah1b3	1.86E-63	0.533662	0.263	0.14	4.56E-59	Classical M	Pafah1b3
Lrrc591	2.02E-63	0.436148	0.266	0.141	4.93E-59	Classical M	Lrrc59
Vps26a	2.53E-63	0.291761	0.342	0.193	6.19E-59	Classical M	Vps26a
Supt5	3.62E-63	0.286564	0.297	0.161	8.86E-59	Classical M	Supt5
Trim30d	4.89E-63	0.366376	0.316	0.176	1.20E-58	Classical M	Trim30d
Hnrnpa01	6.44E-63	0.289961	0.456	0.277	1.58E-58	Classical M	Hnrnpa0
Eapp	6.99E-63	0.369571	0.279	0.149	1.71E-58	Classical M	Eapp
Bcas2	7.35E-63	0.270903	0.377	0.219	1.80E-58	Classical M	Bcas2
M6pr2	1.14E-62	0.295993	0.682	0.454	2.80E-58	Classical M	M6pr
Smim20	1.31E-62	0.350992	0.296	0.161	3.21E-58	Classical M	Smim20
Pttg1	1.75E-62	0.295431	0.352	0.202	4.29E-58	Classical M	Pttg1
Pdap1	1.97E-62	0.324191	0.282	0.152	4.83E-58	Classical M	Pdap1
Fam3c	2.29E-62	0.337013	0.318	0.178	5.60E-58	Classical M	Fam3c
Igbp11	9.52E-62	0.312432	0.374	0.219	2.33E-57	Classical M	Igbp1
Snrnp200	1.10E-61	0.347081	0.261	0.138	2.70E-57	Classical M	Snrnp200
Ebna1bp2	1.12E-61	0.461024	0.252	0.132	2.73E-57	Classical M	Ebna1bp2
Anp32a	1.19E-61	0.264372	0.54	0.342	2.93E-57	Classical M	Anp32a
Mea11	1.28E-61	0.295797	0.339	0.193	3.14E-57	Classical M	Mea1
Cmtm71	1.37E-61	0.299084	0.805	0.59	3.35E-57	Classical M	Cmtm7
Emg1	1.82E-61	0.278292	0.417	0.25	4.46E-57	Classical M	Emg1
Arpc41	2.30E-61	0.290248	0.818	0.589	5.64E-57	Classical M	Arpc4
Ccdc124	2.38E-61	0.331558	0.317	0.178	5.82E-57	Classical M	Ccdc124
Pcbp2	2.73E-61	0.251134	0.537	0.338	6.68E-57	Classical M	Pcbp2
Psma1	3.72E-61	0.277856	0.467	0.289	9.11E-57	Classical M	Psma1
Mrpl15	5.12E-61	0.376012	0.269	0.144	1.26E-56	Classical M	Mrpl15
Trappc6a1	8.97E-61	0.455966	0.262	0.141	2.20E-56	Classical M	Trappc6a
Ssna1	1.27E-60	0.264473	0.334	0.189	3.12E-56	Classical M	Ssna1
Copb2	1.56E-60	0.307627	0.338	0.193	3.82E-56	Classical M	Copb2

Tomm70a	1.88E-60	0.307923	0.301	0.167	4.60E-56	Classical M	Tomm70a
Jak2	2.28E-60	0.38672	0.267	0.142	5.59E-56	Classical M	Jak2
Got2	3.39E-60	0.328864	0.253	0.134	8.31E-56	Classical M	Got2
Azi2	3.76E-60	0.273079	0.263	0.139	9.22E-56	Classical M	Azi2
0610012G(	4.12E-60	0.286672	0.468	0.289	1.01E-55	Classical M	0610012G03Rik
Nosip	6.14E-60	0.386851	0.254	0.134	1.50E-55	Classical M	Nosip
Psmb7	1.14E-59	0.326955	0.311	0.175	2.79E-55	Classical M	Psmb7
Srp19	1.29E-59	0.271066	0.359	0.208	3.15E-55	Classical M	Srp19
Ctsh3	2.14E-59	0.269425	0.947	0.78	5.25E-55	Classical M	Ctsh
Vdac21	2.77E-59	0.294974	0.75	0.528	6.79E-55	Classical M	Vdac2
Fam32a	3.33E-59	0.278232	0.355	0.206	8.16E-55	Classical M	Fam32a
Siglece	5.23E-59	0.344516	0.313	0.175	1.28E-54	Classical M	Siglece
E2f4	8.51E-59	0.291241	0.304	0.169	2.09E-54	Classical M	E2f4
Sptssa	9.37E-59	0.265658	0.474	0.298	2.30E-54	Classical M	Sptssa
Mrpl171	1.01E-58	0.253085	0.426	0.258	2.47E-54	Classical M	Mrpl17
Rbfa	5.68E-58	0.316796	0.319	0.182	1.39E-53	Classical M	Rbfa
Med8	6.75E-58	0.313363	0.286	0.159	1.65E-53	Classical M	Med8
Eif3b1	7.02E-58	0.317077	0.295	0.165	1.72E-53	Classical M	Eif3b
Acsl5	1.40E-57	0.281905	0.354	0.206	3.42E-53	Classical M	Acsl5
Sumo3	1.60E-57	0.372935	0.288	0.16	3.92E-53	Classical M	Sumo3
Hnrnph2	1.95E-57	0.259779	0.359	0.209	4.78E-53	Classical M	Hnrnph2
Emd	2.24E-57	0.316316	0.293	0.163	5.49E-53	Classical M	Emd
Cdv3	1.06E-56	0.316775	0.256	0.137	2.60E-52	Classical M	Cdv3
Eif1a1	1.27E-56	0.315253	0.268	0.147	3.12E-52	Classical M	Eif1a
Gm15564	2.24E-56	0.288586	0.376	0.225	5.50E-52	Classical M	Gm15564
Ddt1	3.52E-56	0.325648	0.293	0.165	8.63E-52	Classical M	Ddt
Eif3l1	4.35E-56	0.270574	0.363	0.215	1.06E-51	Classical M	Eif3l
Polr2c	4.64E-56	0.290042	0.273	0.149	1.14E-51	Classical M	Polr2c
Stoml21	4.79E-56	0.319231	0.28	0.156	1.17E-51	Classical M	Stoml2
Selenoh1	4.95E-56	0.370791	0.275	0.153	1.21E-51	Classical M	Selenoh
Nubp1	5.19E-56	0.32548	0.297	0.168	1.27E-51	Classical M	Nubp1
Rps212	5.34E-56	0.251908	0.968	0.827	1.31E-51	Classical M	Rps21
Gadd45gip	5.65E-56	0.272091	0.303	0.171	1.38E-51	Classical M	Gadd45gip1
Gm16286	9.64E-56	0.275892	0.378	0.228	2.36E-51	Classical M	Gm16286
Pin1	1.04E-55	0.307915	0.267	0.147	2.54E-51	Classical M	Pin1
Psmd13	1.35E-55	0.272539	0.358	0.211	3.30E-51	Classical M	Psmd13
2210016F1	2.08E-55	0.283776	0.317	0.183	5.09E-51	Classical M	2210016F16Rik
Samm50	2.72E-55	0.266773	0.317	0.181	6.67E-51	Classical M	Samm50
Bach11	2.77E-55	0.401101	0.399	0.246	6.78E-51	Classical M	Bach1
Gm42031	4.69E-55	0.317388	0.308	0.176	1.15E-50	Classical M	Gm42031
Hif1a1	7.91E-55	0.417727	0.417	0.262	1.94E-50	Classical M	Hif1a
Gga1	8.21E-55	0.250751	0.282	0.157	2.01E-50	Classical M	Gga1
Acp1	1.04E-54	0.265518	0.266	0.146	2.54E-50	Classical M	Acp1
Snhg11	1.11E-54	0.404516	0.268	0.149	2.73E-50	Classical M	Snhg1

Hsd17b101	5.84E-54	0.377022	0.263	0.146	1.43E-49	Classical M	Hsd17b10
Med21	9.23E-54	0.330128	0.26	0.143	2.26E-49	Classical M	Med21
Hspd11	2.24E-53	0.268853	0.369	0.223	5.49E-49	Classical M	Hspd1
Keap1	2.80E-53	0.331592	0.271	0.151	6.85E-49	Classical M	Keap1
Tax1bp3	8.45E-53	0.330103	0.267	0.148	2.07E-48	Classical M	Tax1bp3
Pet100	1.83E-52	0.321711	0.274	0.154	4.48E-48	Classical M	Pet100
Dok1	1.91E-52	0.327678	0.334	0.198	4.67E-48	Classical M	Dok1
Dbnl	3.68E-52	0.283464	0.512	0.335	9.02E-48	Classical M	Dbnl
Lipa1	5.70E-52	0.267311	0.461	0.291	1.40E-47	Classical M	Lipa
Phf5a	7.06E-52	0.254465	0.302	0.175	1.73E-47	Classical M	Phf5a
Mrps12	1.76E-51	0.281466	0.27	0.152	4.31E-47	Classical M	Mrps12
Vps51	3.45E-51	0.32842	0.28	0.159	8.45E-47	Classical M	Vps51
Sf3a2	3.52E-51	0.297262	0.277	0.157	8.62E-47	Classical M	Sf3a2
Csf2rb2	5.02E-51	0.509818	0.257	0.145	1.23E-46	Classical M	Csf2rb2
Ptpn2	2.38E-50	0.272727	0.256	0.142	5.82E-46	Classical M	Ptpn2
Mif4gd	5.45E-50	0.283472	0.31	0.183	1.33E-45	Classical M	Mif4gd
Ccl9	1.47E-49	0.716153	0.251	0.144	3.61E-45	Classical M	Ccl9
Ehmt2	5.54E-49	0.301191	0.269	0.153	1.36E-44	Classical M	Ehmt2
Nsmce4a	1.87E-48	0.259821	0.278	0.16	4.57E-44	Classical M	Nsmce4a
Pkib1	7.21E-48	0.311295	0.325	0.197	1.77E-43	Classical M	Pkib
Tapbpl	9.16E-48	0.305536	0.297	0.175	2.24E-43	Classical M	Tapbpl
Zfp36l21	2.31E-47	0.288826	0.701	0.516	5.66E-43	Classical M	Zfp36l2
Mrpl111	5.51E-46	0.273159	0.266	0.154	1.35E-41	Classical M	Mrpl11
Clec5a	3.63E-45	0.327934	0.319	0.194	8.88E-41	Classical M	Clec5a
Stxbp21	1.30E-44	0.270324	0.285	0.17	3.19E-40	Classical M	Stxbp2
Lfng	2.29E-43	0.265854	0.269	0.157	5.61E-39	Classical M	Lfng
Prxl2b1	2.65E-43	0.270963	0.299	0.182	6.49E-39	Classical M	Prxl2b
Smim121	2.74E-42	0.265249	0.272	0.162	6.71E-38	Classical M	Smim12
Qars1	2.15E-39	0.251335	0.26	0.156	5.26E-35	Classical M	Qars
Cep851	2.71E-37	0.312502	0.421	0.286	6.64E-33	Classical M	Cep85
Itgax1	7.30E-32	0.300846	0.261	0.167	1.79E-27	Classical M	Itgax
Smagp	3.92E-31	0.309777	0.251	0.161	9.60E-27	Classical M	Smagp
Cd142	3.86E-28	0.334193	0.683	0.54	9.45E-24	Classical M	Cd14
Pf4	0	6.204228	0.871	0.041	0	cDC2	Pf4
F13a1	0	4.276699	0.808	0.052	0	cDC2	F13a1
Mrc11	0	4.215084	0.878	0.126	0	cDC2	Mrc1
Ms4a71	0	4.224412	0.82	0.075	0	cDC2	Ms4a7
Dab2	0	3.937219	0.772	0.089	0	cDC2	Dab2
Ccl8	0	6.13149	0.681	0.034	0	cDC2	Ccl8
Igfbp4	0	4.117339	0.748	0.117	0	cDC2	Igfbp4
Pltp1	0	3.411856	0.719	0.109	0	cDC2	Pltp
Igf11	0	3.292856	0.684	0.087	0	cDC2	Igf1
Cbr2	0	6.419066	0.577	0.015	0	cDC2	Cbr2
Blvrb2	0	2.615227	0.807	0.285	0	cDC2	Blvrb

Clec10a	0	4.894993	0.549	0.028	0	cDC2	Clec10a
Itsn1	0	2.979188	0.599	0.081	0	cDC2	Itsn1
Smagp1	0	2.993775	0.65	0.137	0	cDC2	Smagp
Stab1	0	2.928784	0.855	0.343	0	cDC2	Stab1
Ms4a4a1	0	3.62845	0.566	0.055	0	cDC2	Ms4a4a
Ifitm21	0	1.767732	0.693	0.183	0	cDC2	Ifitm2
Ms4a6c1	0	1.918093	0.76	0.257	0	cDC2	Ms4a6c
Ccl7	0	4.626384	0.545	0.047	0	cDC2	Ccl7
Aoah1	0	2.561373	0.586	0.104	0	cDC2	Aoah
Gas6	0	2.133342	0.802	0.339	0	cDC2	Gas6
Lgals11	0	2.433229	0.603	0.141	0	cDC2	Lgals1
Dse1	0	2.546334	0.598	0.14	0	cDC2	Dse
Wwp1	0	2.405482	0.609	0.154	0	cDC2	Wwp1
Clec4a11	0	2.993372	0.521	0.068	0	cDC2	Clec4a1
Vcam11	0	3.135036	0.566	0.119	0	cDC2	Vcam1
Cd163	0	5.453486	0.452	0.01	0	cDC2	Cd163
Tgfbi1	0	1.796744	0.619	0.181	0	cDC2	Tgfbi
Cybb1	0	1.235259	0.646	0.213	0	cDC2	Cybb
Pla2g72	0	2.161638	0.562	0.14	0	cDC2	Pla2g7
Aldh22	0	2.176137	0.672	0.251	0	cDC2	Aldh2
Cp	0	3.17267	0.483	0.062	0	cDC2	Cp
Ifi2071	0	2.144431	0.561	0.157	0	cDC2	Ifi207
Clec4n	0	4.147624	0.431	0.029	0	cDC2	Clec4n
Marcksl11	0	2.116414	0.539	0.138	0	cDC2	Marcksl1
Anxa52	0	1.590558	0.68	0.285	0	cDC2	Anxa5
Ninj12	0	1.897186	0.702	0.31	0	cDC2	Ninj1
Fcgrt	0	2.143313	0.765	0.375	0	cDC2	Fcgrt
Tmem106a	0	2.182533	0.598	0.209	0	cDC2	Tmem106a
Rbpj	0	2.185833	0.632	0.244	0	cDC2	Rbpj
Ifitm31	0	0.737851	0.793	0.412	0	cDC2	Ifitm3
Ms4a6b1	0	1.670881	0.659	0.278	0	cDC2	Ms4a6b
Ptgds	0	1.672982	0.613	0.235	0	cDC2	Ptgds
Mgl2	0	3.176603	0.407	0.031	0	cDC2	Mgl2
Folr2	0	4.742369	0.397	0.028	0	cDC2	Folr2
Wfdc171	0	2.961648	0.468	0.1	0	cDC2	Wfdc17
Serpinb6a	0	2.294147	0.49	0.124	0	cDC2	Serpinb6a
Hmox11	0	2.101454	0.554	0.189	0	cDC2	Hmox1
Snx21	0	1.882291	0.683	0.319	0	cDC2	Snx2
Myo5a2	0	1.510416	0.597	0.237	0	cDC2	Myo5a
Stard8	0	2.096009	0.483	0.125	0	cDC2	Stard8
Sash11	0	1.848608	0.48	0.123	0	cDC2	Sash1
Gpx3	0	3.409625	0.411	0.055	0	cDC2	Gpx3
Bst22	0	1.743651	0.922	0.574	0	cDC2	Bst2
Ccl22	0	2.531685	0.481	0.133	0	cDC2	Ccl2

Ehd41	0	1.555436	0.74	0.395	0	cDC2	Ehd4
C4b2	0	1.949806	0.558	0.214	0	cDC2	C4b
Colec12	0	3.467701	0.371	0.027	0	cDC2	Colec12
Psd3	0	2.148972	0.393	0.063	0	cDC2	Psd3
Hacd41	0	1.617708	0.53	0.201	0	cDC2	Hacd4
Pla2g2d	0	6.832982	0.332	0.004	0	cDC2	Pla2g2d
Lyz22	0	1.985297	0.99	0.663	0	cDC2	Lyz2
Iqgap21	0	2.354103	0.39	0.064	0	cDC2	Iqgap2
Ms4a6d1	0	1.476356	0.649	0.323	0	cDC2	Ms4a6d
Arhgap19	0	3.264216	0.377	0.054	0	cDC2	Arhgap19
Ftl1-ps12	0	1.775158	0.523	0.205	0	cDC2	Ftl1-ps1
Snx31	0	1.47225	0.745	0.429	0	cDC2	Snx3
Mpp11	0	2.022771	0.452	0.136	0	cDC2	Mpp1
Emp31	0	1.228286	0.521	0.206	0	cDC2	Emp3
Lrp6	0	1.508277	0.505	0.19	0	cDC2	Lrp6
Cd632	0	0.856571	0.92	0.608	0	cDC2	Cd63
CAAA0114	0	1.285418	0.699	0.388	0	cDC2	CAAA01147332.1
Sdc42	0	2.071587	0.428	0.12	0	cDC2	Sdc4
Prdx12	0	1.430979	0.84	0.533	0	cDC2	Prdx1
Lilrb4a2	0	1.316022	0.46	0.154	0	cDC2	Lilrb4a
Siglec1	0	3.898896	0.327	0.027	0	cDC2	Siglec1
Cfp	0	3.13594	0.348	0.049	0	cDC2	Cfp
Rnf150	0	1.437271	0.413	0.117	0	cDC2	Rnf150
Maf3	0	1.415377	0.743	0.448	0	cDC2	Maf
Ifi471	0	1.991695	0.388	0.094	0	cDC2	Ifi47
Arhgef3	0	1.988379	0.369	0.085	0	cDC2	Arhgef3
Ehd11	0	2.070085	0.375	0.094	0	cDC2	Ehd1
Ifi2031	0	2.086194	0.343	0.063	0	cDC2	Ifi203
2610507B1	0	1.612997	0.433	0.156	0	cDC2	2610507B11Rik
Mndal1	0	1.909028	0.349	0.073	0	cDC2	Mndal
Akr1a12	0	1.10997	0.833	0.557	0	cDC2	Akr1a1
Ap2a2	0	1.551247	0.414	0.142	0	cDC2	Ap2a2
Cd361	0	2.277531	0.376	0.105	0	cDC2	Cd36
Msr11	0	2.660169	0.308	0.042	0	cDC2	Msr1
Tbc1d4	0	1.545142	0.357	0.096	0	cDC2	Tbc1d4
Ctla2b1	0	2.418186	0.346	0.085	0	cDC2	Ctla2b
Eps8	0	1.934737	0.333	0.075	0	cDC2	Eps8
Cd4	0	5.511547	0.262	0.006	0	cDC2	Cd4
Cd38	0	3.689612	0.276	0.021	0	cDC2	Cd38
C21	0	1.843311	0.353	0.1	0	cDC2	C2
Paox1	0	1.981738	0.361	0.109	0	cDC2	Paox
Timd4	0	4.754013	0.263	0.011	0	cDC2	Timd4
Rnasel	0	2.280379	0.313	0.063	0	cDC2	Rnasel
Gstm1	0	1.787889	0.358	0.108	0	cDC2	Gstm1

Tmem176b	0	1.178061	0.865	0.616	0	cDC2	Tmem176b
Chp2	0	6.167896	0.251	0.003	0	cDC2	Chp2
Clcn5	0	1.49667	0.352	0.104	0	cDC2	Clcn5
Rap2b2	0	1.799	0.365	0.118	0	cDC2	Rap2b
Hip1	0	1.546487	0.328	0.083	0	cDC2	Hip1
Ccl24	0	2.27579	0.299	0.063	0	cDC2	Ccl24
Cd931	0	3.374535	0.259	0.026	0	cDC2	Cd93
Sh3bp5	0	1.643796	0.32	0.089	0	cDC2	Sh3bp5
Mgst11	0	1.867946	0.292	0.068	0	cDC2	Mgst1
Apoe3	0	1.69504	0.999	0.775	0	cDC2	Apoe
Pde7b1	0	1.813843	0.27	0.047	0	cDC2	Pde7b
Pxdc1	0	2.277499	0.282	0.062	0	cDC2	Pxdc1
Plekhg5	0	2.02446	0.268	0.053	0	cDC2	Plekhg5
Ccr11	0	1.958005	0.28	0.066	0	cDC2	Ccr1
Pstpip2	0	1.722969	0.268	0.057	0	cDC2	Pstpip2
Ctsc2	0	1.372292	0.899	0.697	0	cDC2	Ctsc
Selenop3	0	1.169958	0.976	0.809	0	cDC2	Selenop
Clta2	0	0.855586	0.925	0.784	0	cDC2	Clta
Ctsb2	0	0.645415	0.986	0.901	0	cDC2	Ctsb
Ftl12	0	1.594652	0.999	0.92	0	cDC2	Ftl1
Fth12	0	1.305972	1	0.978	0	cDC2	Fth1
Prxl2b2	6.27E-304	1.696113	0.447	0.174	1.53E-299	cDC2	Prxl2b
Cltc	3.17E-301	1.111579	0.796	0.572	7.77E-297	cDC2	Cltc
Iigp11	5.76E-300	1.65114	0.391	0.135	1.41E-295	cDC2	Iigp1
Nrp11	1.57E-299	1.151145	0.672	0.367	3.86E-295	cDC2	Nrp1
Gpr651	5.66E-297	1.979471	0.284	0.079	1.39E-292	cDC2	Gpr65
Vav31	1.18E-294	1.269563	0.27	0.07	2.88E-290	cDC2	Vav3
Cited22	2.14E-291	1.73354	0.575	0.28	5.24E-287	cDC2	Cited2
Slc48a12	9.40E-288	1.915645	0.395	0.147	2.30E-283	cDC2	Slc48a1
Eps151	4.01E-286	1.396257	0.51	0.222	9.81E-282	cDC2	Eps15
Cyb5r11	1.85E-282	1.627145	0.434	0.17	4.53E-278	cDC2	Cyb5r1
Snx8	7.53E-279	1.56701	0.361	0.123	1.85E-274	cDC2	Snx8
Tmem176a	3.50E-278	1.164431	0.723	0.435	8.57E-274	cDC2	Tmem176a
Blvra2	3.60E-278	1.629683	0.434	0.173	8.81E-274	cDC2	Blvra
Mtss1	9.45E-277	1.104478	0.44	0.164	2.31E-272	cDC2	Mtss1
Trf3	4.11E-276	0.918271	0.895	0.728	1.01E-271	cDC2	Trf
Nfxl1	4.86E-276	1.451507	0.358	0.12	1.19E-271	cDC2	Nfxl1
Fam234a	3.07E-265	1.671052	0.307	0.096	7.52E-261	cDC2	Fam234a
Fcgr2b2	2.35E-261	1.018647	0.834	0.645	5.75E-257	cDC2	Fcgr2b
Twf1	3.53E-261	1.55956	0.383	0.143	8.65E-257	cDC2	Twf1
Klf22	6.86E-251	1.440868	0.486	0.214	1.68E-246	cDC2	Klf2
S100a14	8.48E-245	1.409282	0.421	0.171	2.08E-240	cDC2	S100a1
Ppia2	1.05E-243	0.570604	0.981	0.876	2.58E-239	cDC2	Ppia
Bri32	1.78E-243	0.897663	0.821	0.609	4.36E-239	cDC2	Bri3

Ptpn181	1.48E-238	0.836045	0.835	0.641	3.61E-234	cDC2	Ptpn18
Lars2	4.46E-238	0.906524	0.932	0.746	1.09E-233	cDC2	Lars2
Txnip1	1.77E-237	1.258787	0.742	0.53	4.33E-233	cDC2	Txnip
Itga41	2.00E-236	0.777251	0.317	0.102	4.90E-232	cDC2	Itga4
Mdfic1	3.68E-236	1.396238	0.301	0.099	9.01E-232	cDC2	Mdfic
Clec4a22	1.15E-231	1.204328	0.521	0.246	2.82E-227	cDC2	Clec4a2
Adgre1	1.04E-230	1.012747	0.738	0.518	2.55E-226	cDC2	Adgre1
Tpt13	1.32E-223	0.542445	0.987	0.893	3.23E-219	cDC2	Tpt1
Mt11	3.79E-219	1.06971	0.759	0.493	9.28E-215	cDC2	Mt1
Fcer1g3	1.45E-217	0.507462	0.984	0.901	3.54E-213	cDC2	Fcer1g
C5ar1	2.19E-216	1.269827	0.503	0.243	5.37E-212	cDC2	C5ar1
Ybx12	2.48E-215	0.872248	0.787	0.562	6.07E-211	cDC2	Ybx1
Aplp21	1.69E-214	1.218541	0.527	0.264	4.14E-210	cDC2	Aplp2
Gabarap3	5.26E-208	0.73261	0.859	0.667	1.29E-203	cDC2	Gabarap
Ap2s12	1.99E-203	1.231621	0.539	0.288	4.88E-199	cDC2	Ap2s1
Hgsnat	1.96E-202	2.057595	0.288	0.103	4.80E-198	cDC2	Hgsnat
Dmac11	4.03E-201	1.501207	0.356	0.145	9.86E-197	cDC2	Dmac1
Fkbp1a1	1.73E-200	1.130255	0.581	0.324	4.24E-196	cDC2	Fkbp1a
Clec2d1	1.65E-199	1.115956	0.508	0.254	4.03E-195	cDC2	Clec2d
Galc	1.42E-198	1.40359	0.302	0.11	3.49E-194	cDC2	Galc
Dclre1c	3.05E-198	1.161508	0.449	0.208	7.47E-194	cDC2	Dclre1c
Etv1	1.07E-197	1.165341	0.296	0.103	2.62E-193	cDC2	Etv1
Hint12	1.53E-197	0.89086	0.759	0.516	3.74E-193	cDC2	Hint1
Ddx60	6.63E-197	1.253446	0.289	0.101	1.62E-192	cDC2	Ddx60
AB124611?	1.86E-192	1.263729	0.373	0.157	4.56E-188	cDC2	AB124611
Gm42418	1.27E-191	0.672176	1	0.981	3.12E-187	cDC2	Gm42418
St8sia41	2.26E-191	1.051024	0.273	0.092	5.53E-187	cDC2	St8sia4
Gbp71	9.96E-191	1.253108	0.407	0.181	2.44E-186	cDC2	Gbp7
Cyb5a1	1.09E-188	1.160079	0.626	0.387	2.66E-184	cDC2	Cyb5a
Klf62	4.95E-186	1.171342	0.601	0.36	1.21E-181	cDC2	Klf6
Gmfg1	5.55E-186	0.930839	0.628	0.374	1.36E-181	cDC2	Gmfg
Fam174a1	1.73E-185	1.121837	0.549	0.306	4.24E-181	cDC2	Fam174a
AW112010	1.10E-182	1.177295	0.513	0.278	2.69E-178	cDC2	AW112010
Lst11	2.72E-182	0.968221	0.732	0.528	6.67E-178	cDC2	Lst1
P2rx42	2.01E-181	1.052483	0.551	0.307	4.92E-177	cDC2	P2rx4
App1	2.98E-181	0.95303	0.73	0.53	7.30E-177	cDC2	App
Sh3bgrl1	9.72E-180	1.302283	0.319	0.126	2.38E-175	cDC2	Sh3bgrl
Dbi1	1.66E-179	0.875206	0.692	0.423	4.07E-175	cDC2	Dbi
Dusp13	1.37E-178	1.239941	0.605	0.375	3.34E-174	cDC2	Dusp1
Iqgap11	1.00E-177	0.682413	0.382	0.158	2.46E-173	cDC2	Iqgap1
Tmem2562	7.17E-177	1.083381	0.549	0.308	1.76E-172	cDC2	Tmem256
Nenf	2.05E-176	1.071352	0.527	0.285	5.02E-172	cDC2	Nenf
Hprt1	3.11E-176	1.051245	0.578	0.334	7.62E-172	cDC2	Hprt
Prkacb	4.21E-176	1.138166	0.373	0.161	1.03E-171	cDC2	Prkacb

Stk17b	5.06E-176	0.960864	0.376	0.162	1.24E-171	cDC2	Stk17b
Cyp27a11	6.30E-174	1.08195	0.326	0.129	1.54E-169	cDC2	Cyp27a1
Hpgd2	7.45E-174	0.879208	0.751	0.52	1.82E-169	cDC2	Hpgd
Laptm4a3	1.81E-173	0.607923	0.859	0.73	4.44E-169	cDC2	Laptm4a
Fosb1	3.24E-172	0.977581	0.361	0.154	7.93E-168	cDC2	Fosb
Fos3	2.97E-171	0.976741	0.691	0.471	7.27E-167	cDC2	Fos
Trps11	4.48E-170	0.536072	0.374	0.158	1.10E-165	cDC2	Trps1
Plau1	1.66E-167	1.296565	0.344	0.148	4.07E-163	cDC2	Plau
Ap1b1	3.12E-166	1.047069	0.461	0.234	7.63E-162	cDC2	Ap1b1
Tubb2a2	2.48E-164	1.057767	0.477	0.245	6.08E-160	cDC2	Tubb2a
Ubb2	2.33E-163	0.480412	0.953	0.861	5.69E-159	cDC2	Ubb
Snx6	5.77E-163	0.983148	0.609	0.384	1.41E-158	cDC2	Snx6
Gbp81	1.75E-162	1.347037	0.272	0.104	4.28E-158	cDC2	Gbp8
Hpse1	2.24E-162	1.525401	0.264	0.1	5.49E-158	cDC2	Hpse
Hfe1	1.20E-159	0.968839	0.483	0.253	2.95E-155	cDC2	Hfe
Atpif1	3.83E-156	0.797878	0.758	0.551	9.39E-152	cDC2	Atpif1
Ost41	7.88E-154	0.815756	0.705	0.489	1.93E-149	cDC2	Ost4
Adam91	5.30E-153	1.242571	0.304	0.127	1.30E-148	cDC2	Adam9
Tsc22d32	1.29E-152	1.028159	0.518	0.289	3.15E-148	cDC2	Tsc22d3
Idh22	3.55E-152	0.955192	0.518	0.287	8.70E-148	cDC2	Idh2
Cmss1	4.81E-152	0.562542	0.987	0.92	1.18E-147	cDC2	Cmss1
Ccnd11	4.93E-152	1.172783	0.413	0.205	1.21E-147	cDC2	Ccnd1
P2rx71	9.33E-151	1.33511	0.568	0.366	2.28E-146	cDC2	P2rx7
B3galnt1	2.01E-149	1.026719	0.364	0.165	4.91E-145	cDC2	B3galnt1
Tmsb4x1	6.44E-149	0.348789	0.999	0.966	1.58E-144	cDC2	Tmsb4x
Arhgap151	5.35E-148	0.324582	0.518	0.268	1.31E-143	cDC2	Arhgap15
Cstb2	2.19E-146	0.769287	0.614	0.373	5.37E-142	cDC2	Cstb
H2-Aa3	4.08E-146	0.922382	0.789	0.607	9.99E-142	cDC2	H2-Aa
Cdk81	7.71E-146	0.558502	0.975	0.898	1.89E-141	cDC2	Cdk8
Sema4a1	5.34E-144	1.333701	0.264	0.105	1.31E-139	cDC2	Sema4a
Ncoa4	3.45E-143	1.127983	0.386	0.188	8.46E-139	cDC2	Ncoa4
Cat1	8.23E-141	1.191086	0.365	0.175	2.02E-136	cDC2	Cat
Cndp22	1.11E-140	0.971245	0.533	0.314	2.71E-136	cDC2	Cndp2
Ahnak1	2.42E-139	0.754124	0.262	0.101	5.92E-135	cDC2	Ahnak
Arpc32	1.87E-138	0.652185	0.811	0.633	4.59E-134	cDC2	Arpc3
Cd143	2.30E-138	0.902969	0.712	0.539	5.64E-134	cDC2	Cd14
H2-Eb13	1.12E-137	0.997025	0.755	0.587	2.74E-133	cDC2	H2-Eb1
Ccl122	7.43E-137	1.107562	0.617	0.414	1.82E-132	cDC2	Ccl12
Tgtp21	8.76E-137	1.438029	0.253	0.102	2.15E-132	cDC2	Tgtp2
Calhm61	1.32E-136	1.25195	0.351	0.171	3.23E-132	cDC2	Calhm6
Scp21	1.66E-136	0.956981	0.55	0.33	4.07E-132	cDC2	Scp2
Npl1	2.34E-136	1.135821	0.378	0.186	5.74E-132	cDC2	Npl
Cox8a2	3.54E-136	0.501178	0.92	0.778	8.68E-132	cDC2	Cox8a
0610012G(	2.29E-134	0.980662	0.501	0.287	5.61E-130	cDC2	0610012G03Rik

Osbpl9	4.63E-133	0.830232	0.561	0.347	1.13E-128	cDC2	Osbpl9
Txn11	1.36E-132	0.638297	0.539	0.315	3.34E-128	cDC2	Txn1
Sult1a1	1.66E-132	1.022232	0.337	0.154	4.06E-128	cDC2	Sult1a1
Rnf141	1.02E-131	1.14306	0.252	0.1	2.49E-127	cDC2	Rnf141
C3ar13	3.38E-131	0.736067	0.643	0.416	8.29E-127	cDC2	C3ar1
Neat11	5.90E-130	0.57891	0.506	0.288	1.44E-125	cDC2	Neat1
AY036118	2.23E-128	0.80871	0.919	0.801	5.47E-124	cDC2	AY036118
Ly96	1.08E-125	0.991152	0.327	0.151	2.63E-121	cDC2	Ly96
Il31ra	3.96E-125	0.731465	0.803	0.607	9.69E-121	cDC2	Il31ra
mt-Co2	2.17E-124	0.420036	0.986	0.943	5.32E-120	cDC2	mt-Co2
Cebpd2	2.81E-124	0.832038	0.485	0.271	6.89E-120	cDC2	Cebpd
Nrros	7.65E-124	0.753634	0.691	0.521	1.87E-119	cDC2	Nrros
Hsd17b12	5.35E-123	0.933699	0.454	0.255	1.31E-118	cDC2	Hsd17b12
Gm49511	1.40E-120	0.802009	0.446	0.242	3.42E-116	cDC2	Gm4951
Tle51	4.21E-120	0.788173	0.607	0.395	1.03E-115	cDC2	Tle5
Nrp21	1.06E-119	0.881178	0.322	0.151	2.58E-115	cDC2	Nrp2
Use11	2.22E-119	1.057008	0.44	0.248	5.43E-115	cDC2	Use1
Gpx41	2.83E-119	0.648311	0.78	0.588	6.93E-115	cDC2	Gpx4
Hsp90ab12	8.03E-119	0.488044	0.894	0.733	1.97E-114	cDC2	Hsp90ab1
Rgl11	1.60E-118	0.744676	0.456	0.253	3.92E-114	cDC2	Rgl1
Il10rb1	9.43E-118	0.744741	0.652	0.451	2.31E-113	cDC2	Il10rb
H2afj1	2.99E-117	0.736532	0.591	0.379	7.32E-113	cDC2	H2afj
Mrpl282	7.26E-116	1.171684	0.302	0.143	1.78E-111	cDC2	Mrpl28
Arhgap18	1.04E-115	0.914259	0.302	0.14	2.54E-111	cDC2	Arhgap18
Slc40a11	9.24E-115	1.781536	0.377	0.214	2.26E-110	cDC2	Slc40a1
Man2a11	9.33E-115	0.822078	0.303	0.14	2.29E-110	cDC2	Man2a1
Calm11	1.39E-114	0.442012	0.792	0.584	3.41E-110	cDC2	Calm1
Il18bp2	2.63E-114	1.077336	0.302	0.143	6.44E-110	cDC2	Il18bp
Atp6v0c3	3.23E-114	0.417173	0.902	0.771	7.91E-110	cDC2	Atp6v0c
Rbms11	6.49E-112	0.721738	0.413	0.221	1.59E-107	cDC2	Rbms1
Vamp82	7.47E-112	0.618801	0.755	0.588	1.83E-107	cDC2	Vamp8
Lamp12	8.18E-112	0.428072	0.93	0.819	2.00E-107	cDC2	Lamp1
Tex2641	1.54E-111	0.965079	0.325	0.159	3.78E-107	cDC2	Tex264
Ly6e2	3.02E-110	0.580992	0.939	0.861	7.41E-106	cDC2	Ly6e
H2-Ab13	2.74E-109	0.828094	0.786	0.64	6.71E-105	cDC2	H2-Ab1
Ifi2041	4.02E-109	0.814981	0.339	0.169	9.85E-105	cDC2	Ifi204
Ttr1	5.37E-109	0.906622	0.714	0.5	1.32E-104	cDC2	Ttr
Clec4a32	1.16E-108	0.720531	0.482	0.279	2.83E-104	cDC2	Clec4a3
Tnfsf122	1.46E-108	0.953922	0.41	0.226	3.58E-104	cDC2	Tnfsf12
Il4ra1	1.52E-107	0.875463	0.414	0.231	3.73E-103	cDC2	Il4ra
Slfn51	2.19E-107	0.887437	0.278	0.127	5.37E-103	cDC2	Slfn5
Nfkbiz3	4.06E-107	0.737503	0.439	0.248	9.94E-103	cDC2	Nfkbiz
Psmb82	1.88E-105	0.551353	0.778	0.593	4.60E-101	cDC2	Psmb8
Nop101	3.95E-104	0.887954	0.475	0.286	9.66E-100	cDC2	Nop10

Dcxr1	4.37E-104	0.985686	0.374	0.203	1.07E-99	cDC2	Dcxr
Uap1l12	1.06E-103	0.983119	0.328	0.166	2.60E-99	cDC2	Uap1l1
Slfn21	1.96E-102	0.678094	0.483	0.287	4.81E-98	cDC2	Slfn2
Crtap1	5.13E-101	1.020007	0.278	0.133	1.26E-96	cDC2	Crtap
Vamp3	5.68E-101	0.947728	0.366	0.198	1.39E-96	cDC2	Vamp3
mt-Co1	5.87E-101	0.383823	0.987	0.962	1.44E-96	cDC2	mt-Co1
Slc9a91	6.94E-101	0.404198	0.753	0.635	1.70E-96	cDC2	Slc9a9
Txndc172	1.32E-100	0.727641	0.493	0.302	3.23E-96	cDC2	Txndc17
Atp5g32	1.33E-100	0.596382	0.776	0.584	3.26E-96	cDC2	Atp5g3
Mfsd111	1.69E-100	0.833889	0.38	0.205	4.15E-96	cDC2	Mfsd11
Prdx51	6.05E-100	0.272621	0.782	0.58	1.48E-95	cDC2	Prdx5
Sgpp1	9.72E-100	0.937393	0.253	0.114	2.38E-95	cDC2	Sgpp1
Eif11	1.14E-99	0.382237	0.932	0.829	2.78E-95	cDC2	Eif1
Swap70	1.09E-98	0.773503	0.332	0.169	2.68E-94	cDC2	Swap70
Ctnnd1	1.16E-97	0.700888	0.359	0.188	2.84E-93	cDC2	Ctnnd1
Gltp3	2.54E-97	0.786934	0.566	0.371	6.21E-93	cDC2	Gltp
Gphn	2.72E-97	0.584585	0.797	0.633	6.67E-93	cDC2	Gphn
Lrrc251	3.40E-95	0.725492	0.541	0.358	8.33E-91	cDC2	Lrrc25
Gng52	4.50E-95	0.490779	0.859	0.714	1.10E-90	cDC2	Gng5
Adipor11	6.12E-95	0.672654	0.621	0.432	1.50E-90	cDC2	Adipor1
Plin22	1.01E-94	0.830553	0.387	0.216	2.47E-90	cDC2	Plin2
Ap2m11	1.07E-94	0.770606	0.591	0.414	2.63E-90	cDC2	Ap2m1
Rpl103	1.22E-94	0.380529	0.952	0.835	3.00E-90	cDC2	Rpl10
Sh3bgrl32	1.67E-94	0.373434	0.895	0.742	4.08E-90	cDC2	Sh3bgrl3
Nagk1	1.68E-94	0.91072	0.34	0.182	4.11E-90	cDC2	Nagk
Rps213	1.87E-94	0.33714	0.95	0.811	4.58E-90	cDC2	Rps2
Tent5a1	1.57E-93	0.752619	0.425	0.246	3.84E-89	cDC2	Tent5a
Dennd1a2	1.79E-92	0.383556	0.543	0.344	4.38E-88	cDC2	Dennd1a
Mctp11	3.00E-92	0.738509	0.511	0.333	7.36E-88	cDC2	Mctp1
Sem11	3.02E-92	0.513396	0.731	0.544	7.39E-88	cDC2	Sem1
Scpep12	3.06E-92	0.696791	0.552	0.368	7.49E-88	cDC2	Scpep1
Ebi31	3.56E-92	0.818329	0.442	0.268	8.72E-88	cDC2	Ebi3
Atp6v1a1	3.65E-92	0.748457	0.439	0.261	8.95E-88	cDC2	Atp6v1a
Snx5	5.06E-92	0.516396	0.769	0.63	1.24E-87	cDC2	Snx5
Lipa2	8.82E-92	0.837012	0.469	0.291	2.16E-87	cDC2	Lipa
Atp2b12	8.90E-92	0.56609	0.63	0.449	2.18E-87	cDC2	Atp2b1
Rnf1301	1.06E-91	0.501198	0.79	0.67	2.59E-87	cDC2	Rnf130
Slc29a12	1.37E-91	0.730578	0.453	0.27	3.35E-87	cDC2	Slc29a1
Sbf21	4.00E-91	0.497135	0.544	0.34	9.79E-87	cDC2	Sbf2
H3f3a1	3.18E-90	0.333513	0.825	0.666	7.80E-86	cDC2	H3f3a
mt-Atp6	4.54E-90	0.424056	0.968	0.911	1.11E-85	cDC2	mt-Atp6
Ifi27l2a2	4.58E-90	0.267543	0.589	0.376	1.12E-85	cDC2	Ifi27l2a
Snx91	1.48E-88	0.705419	0.434	0.258	3.63E-84	cDC2	Snx9
D8Ertd738(	8.20E-88	0.651343	0.623	0.436	2.01E-83	cDC2	D8Ertd738e

Ripor2	2.17E-87	0.477507	0.263	0.124	5.31E-83	cDC2	Ripor2
Hexa3	4.78E-87	0.433917	0.848	0.72	1.17E-82	cDC2	Hexa
Cox6b12	7.55E-87	0.575538	0.728	0.55	1.85E-82	cDC2	Cox6b1
Cd743	5.54E-86	0.525149	0.888	0.769	1.36E-81	cDC2	Cd74
Igtp1	1.10E-85	0.974043	0.369	0.213	2.69E-81	cDC2	Igtp
mt-Co3	2.40E-85	0.378733	0.98	0.939	5.87E-81	cDC2	mt-Co3
Cox5a2	3.03E-85	0.655396	0.635	0.456	7.41E-81	cDC2	Cox5a
Lamtor22	8.24E-85	0.752853	0.513	0.336	2.02E-80	cDC2	Lamtor2
Psmb91	4.07E-84	0.614146	0.642	0.476	9.97E-80	cDC2	Psmb9
Pim11	1.07E-82	0.689086	0.28	0.141	2.63E-78	cDC2	Pim1
Ndufa72	1.13E-82	0.597463	0.671	0.488	2.77E-78	cDC2	Ndufa7
Rras1	1.38E-82	0.865882	0.27	0.136	3.39E-78	cDC2	Rras
Txn21	1.70E-82	0.818009	0.408	0.244	4.17E-78	cDC2	Txn2
Atp5h1	2.92E-82	0.567283	0.73	0.545	7.16E-78	cDC2	Atp5h
Sdcbp2	3.82E-82	0.534831	0.688	0.525	9.36E-78	cDC2	Sdcbp
Tln21	4.93E-82	0.460296	0.366	0.198	1.21E-77	cDC2	Tln2
Atp6v1g11	3.20E-81	0.522078	0.733	0.565	7.84E-77	cDC2	Atp6v1g1
Smdt11	1.48E-80	0.640893	0.593	0.41	3.62E-76	cDC2	Smdt1
Eid11	2.10E-80	0.899026	0.286	0.151	5.15E-76	cDC2	Eid1
Lamtor12	5.69E-80	0.658218	0.601	0.423	1.39E-75	cDC2	Lamtor1
Peak1	2.20E-79	0.590905	0.285	0.145	5.39E-75	cDC2	Peak1
Chchd7	3.74E-79	0.978821	0.252	0.126	9.15E-75	cDC2	Chchd7
Zbp12	1.98E-78	0.757045	0.317	0.174	4.85E-74	cDC2	Zbp1
Ndufs71	3.43E-78	0.677377	0.556	0.379	8.40E-74	cDC2	Ndufs7
Atp6v1e11	5.29E-78	0.735986	0.449	0.283	1.30E-73	cDC2	Atp6v1e1
Dhrs3	5.79E-78	0.642418	0.474	0.301	1.42E-73	cDC2	Dhrs3
Rplp13	6.32E-78	0.303412	0.985	0.917	1.55E-73	cDC2	Rplp1
Rtp41	2.48E-76	0.653659	0.403	0.241	6.07E-72	cDC2	Rtp4
Gm480991	8.79E-76	0.7702	0.429	0.267	2.15E-71	cDC2	Gm48099
Rex1bd2	1.45E-75	0.823461	0.386	0.232	3.55E-71	cDC2	Rex1bd
Fam89b1	3.44E-75	0.791441	0.421	0.262	8.43E-71	cDC2	Fam89b
Pfdn53	5.79E-75	0.430763	0.84	0.684	1.42E-70	cDC2	Pfdn5
Dmxl11	1.44E-74	0.512245	0.389	0.228	3.54E-70	cDC2	Dmxl1
Tpr1	1.63E-74	0.630793	0.517	0.348	3.99E-70	cDC2	Tpr
Ucp22	7.09E-74	0.359086	0.856	0.722	1.74E-69	cDC2	Ucp2
Oaz11	1.48E-73	0.420647	0.851	0.716	3.61E-69	cDC2	Oaz1
Tlr4	1.57E-73	0.579538	0.341	0.189	3.83E-69	cDC2	Tlr4
Acot131	4.47E-73	0.870659	0.295	0.161	1.09E-68	cDC2	Acot13
Enpp2	6.15E-73	0.810584	0.375	0.223	1.51E-68	cDC2	Enpp2
Rab1b1	7.36E-73	0.778753	0.412	0.257	1.80E-68	cDC2	Rab1b
Rps143	2.79E-72	0.315233	0.926	0.805	6.84E-68	cDC2	Rps14
Mien12	7.10E-72	0.802401	0.338	0.196	1.74E-67	cDC2	Mien1
B4galt6	2.41E-71	0.63229	0.271	0.141	5.90E-67	cDC2	B4galt6
Gm19951	8.84E-71	0.653297	0.527	0.358	2.16E-66	cDC2	Gm19951

Bcl7c1	1.84E-70	0.927853	0.258	0.136	4.50E-66	cDC2	Bcl7c
Pebp11	3.37E-70	0.652167	0.526	0.354	8.26E-66	cDC2	Pebp1
Dbndd22	1.30E-69	0.857485	0.287	0.159	3.19E-65	cDC2	Dbndd2
Gm155641	1.48E-69	0.786608	0.371	0.225	3.63E-65	cDC2	Gm15564
Cd3022	1.78E-69	0.559723	0.508	0.34	4.36E-65	cDC2	Cd302
Ier32	2.02E-69	0.922021	0.273	0.146	4.94E-65	cDC2	Ier3
Lyl12	6.14E-69	0.649993	0.385	0.233	1.50E-64	cDC2	Lyl1
Psmb22	1.43E-68	0.60554	0.529	0.363	3.50E-64	cDC2	Psmb2
Bank12	4.18E-68	0.260331	0.345	0.19	1.02E-63	cDC2	Bank1
Ndufa41	8.60E-68	0.535105	0.691	0.537	2.11E-63	cDC2	Ndufa4
Tomm62	1.89E-67	0.490806	0.695	0.526	4.62E-63	cDC2	Tomm6
Sf3b51	2.30E-67	0.682962	0.456	0.299	5.64E-63	cDC2	Sf3b5
Pid12	4.61E-67	0.260355	0.59	0.407	1.13E-62	cDC2	Pid1
Dazap21	7.17E-67	0.538898	0.619	0.447	1.76E-62	cDC2	Dazap2
Arl8a1	1.08E-66	0.629263	0.468	0.308	2.66E-62	cDC2	Arl8a
Mrpl272	1.28E-66	0.832845	0.272	0.149	3.14E-62	cDC2	Mrpl27
Irf71	1.63E-66	0.484543	0.325	0.185	3.99E-62	cDC2	Irf7
Atp6v1f3	2.11E-66	0.495663	0.718	0.552	5.16E-62	cDC2	Atp6v1f
Hk3	3.30E-66	0.605105	0.412	0.258	8.09E-62	cDC2	Hk3
Trappc2l2	1.36E-65	0.695074	0.437	0.283	3.33E-61	cDC2	Trappc2l
AI4135822	1.47E-65	0.662473	0.481	0.323	3.59E-61	cDC2	AI413582
Neu11	1.50E-65	0.649438	0.339	0.197	3.67E-61	cDC2	Neu1
Snap23	3.26E-65	0.588563	0.394	0.245	7.98E-61	cDC2	Snap23
Selenok1	4.49E-65	0.460847	0.732	0.583	1.10E-60	cDC2	Selenok
Atp5l2	1.42E-64	0.474352	0.735	0.561	3.49E-60	cDC2	Atp5l
Serp11	1.80E-64	0.596258	0.519	0.355	4.40E-60	cDC2	Serp1
Pmp222	2.56E-64	0.398548	0.53	0.345	6.27E-60	cDC2	Pmp22
Cox141	4.49E-64	0.711505	0.407	0.26	1.10E-59	cDC2	Cox14
Hsbp11	6.90E-64	0.645761	0.561	0.406	1.69E-59	cDC2	Hsbp1
Npc23	8.91E-64	0.265454	0.926	0.806	2.18E-59	cDC2	Npc2
Mfsd12	9.30E-64	0.599598	0.491	0.335	2.28E-59	cDC2	Mfsd1
Rps33	2.07E-63	0.282355	0.943	0.824	5.06E-59	cDC2	Rps3
Anapc111	3.44E-63	0.626819	0.505	0.344	8.44E-59	cDC2	Anapc11
Dnajc191	3.60E-63	0.656381	0.422	0.272	8.82E-59	cDC2	Dnajc19
Vegfb2	1.14E-62	0.653853	0.41	0.259	2.78E-58	cDC2	Vegfb
Churc11	1.48E-62	0.861207	0.282	0.16	3.62E-58	cDC2	Churc1
Ndufb82	1.51E-62	0.620285	0.55	0.388	3.70E-58	cDC2	Ndufb8
Rpl233	1.67E-62	0.271432	0.969	0.878	4.09E-58	cDC2	Rpl23
2810013P0	9.98E-62	0.767343	0.254	0.138	2.45E-57	cDC2	2810013P06Rik
Oasl21	1.20E-61	0.579357	0.32	0.187	2.94E-57	cDC2	Oasl2
Rbfa1	2.68E-61	0.778563	0.31	0.182	6.55E-57	cDC2	Rbfa
Ang3	4.41E-61	0.518491	0.467	0.308	1.08E-56	cDC2	Ang
Cenpb2	9.73E-61	0.9347	0.258	0.144	2.38E-56	cDC2	Cenpb
Mrpl232	2.39E-60	0.602112	0.491	0.337	5.84E-56	cDC2	Mrpl23

Sptssa1	7.28E-60	0.636327	0.45	0.299	1.78E-55	cDC2	Sptssa
Fads1	1.60E-59	0.677469	0.278	0.155	3.91E-55	cDC2	Fads1
Rab322	2.16E-59	0.81531	0.316	0.19	5.29E-55	cDC2	Rab32
Swi51	3.24E-59	0.621371	0.531	0.374	7.94E-55	cDC2	Swi5
Slc25a33	3.84E-59	0.422015	0.756	0.605	9.39E-55	cDC2	Slc25a3
Sumo31	8.63E-59	0.83923	0.279	0.161	2.11E-54	cDC2	Sumo3
Anxa42	1.77E-58	0.753951	0.265	0.149	4.33E-54	cDC2	Anxa4
Scand11	1.81E-58	0.497299	0.573	0.41	4.42E-54	cDC2	Scand1
Ndufc23	5.98E-58	0.498965	0.655	0.498	1.46E-53	cDC2	Ndufc2
Rit1	8.28E-58	0.805862	0.261	0.146	2.03E-53	cDC2	Rit1
Fkbp22	1.23E-57	0.485796	0.563	0.399	3.01E-53	cDC2	Fkbp2
Stard3nl	1.49E-57	0.560379	0.501	0.351	3.65E-53	cDC2	Stard3nl
Clic41	2.31E-57	0.598144	0.34	0.207	5.64E-53	cDC2	Clic4
Mt3	4.81E-57	0.623321	0.346	0.214	1.18E-52	cDC2	Mt3
Amdhd22	4.85E-57	0.893293	0.288	0.17	1.19E-52	cDC2	Amdhd2
Tspan33	5.47E-57	0.451227	0.55	0.388	1.34E-52	cDC2	Tspan3
Ndufb72	5.78E-57	0.547448	0.525	0.368	1.41E-52	cDC2	Ndufb7
Pcbd21	6.41E-57	0.743885	0.267	0.152	1.57E-52	cDC2	Pcbd2
Prdx41	1.52E-56	0.751441	0.306	0.182	3.71E-52	cDC2	Prdx4
Gnpda11	1.88E-56	0.795179	0.267	0.153	4.60E-52	cDC2	Gnpda1
Slc38a6	3.08E-56	0.374956	0.283	0.16	7.55E-52	cDC2	Slc38a6
Mrfap11	1.38E-55	0.519026	0.577	0.417	3.37E-51	cDC2	Mrfap1
Qdpr	1.48E-55	0.718028	0.339	0.212	3.63E-51	cDC2	Qdpr
Themis21	2.43E-55	0.552818	0.348	0.216	5.95E-51	cDC2	Themis2
Vps13c	3.36E-55	0.458548	0.338	0.205	8.23E-51	cDC2	Vps13c
H1f01	3.38E-55	0.47008	0.346	0.214	8.29E-51	cDC2	H1f0
Akr1b32	5.10E-55	0.616045	0.456	0.311	1.25E-50	cDC2	Akr1b3
Sdc32	1.01E-54	0.678892	0.387	0.252	2.48E-50	cDC2	Sdc3
Capns12	1.58E-54	0.605892	0.492	0.345	3.88E-50	cDC2	Capns1
Rps3a13	2.29E-54	0.253405	0.972	0.879	5.60E-50	cDC2	Rps3a1
Uqcr111	2.39E-54	0.530785	0.579	0.424	5.86E-50	cDC2	Uqcr11
Milr12	2.41E-54	0.708003	0.274	0.158	5.90E-50	cDC2	Milr1
Wdr41	2.55E-54	0.491212	0.27	0.153	6.24E-50	cDC2	Wdr41
Cdc42se21	2.88E-54	0.437197	0.414	0.268	7.04E-50	cDC2	Cdc42se2
Nedd81	4.36E-54	0.547119	0.602	0.446	1.07E-49	cDC2	Nedd8
Tspo2	5.55E-54	0.305539	0.617	0.442	1.36E-49	cDC2	Tspo
Ndufb101	5.92E-54	0.571953	0.546	0.395	1.45E-49	cDC2	Ndufb10
Uqcrb2	6.07E-54	0.567568	0.57	0.418	1.49E-49	cDC2	Uqcrb
Acat1	8.75E-54	0.766236	0.301	0.182	2.14E-49	cDC2	Acat1
Cox5b2	1.04E-53	0.445114	0.703	0.539	2.55E-49	cDC2	Cox5b
Cpe	1.35E-53	0.65701	0.269	0.154	3.31E-49	cDC2	Cpe
Ndufaf81	1.83E-53	0.734038	0.294	0.176	4.49E-49	cDC2	Ndufaf8
Hmox21	2.19E-53	0.514715	0.566	0.414	5.37E-49	cDC2	Hmox2
Rpl83	3.26E-53	0.261485	0.928	0.789	7.98E-49	cDC2	Rpl8

Zfp36l12	6.15E-53	0.425142	0.744	0.627	1.51E-48	cDC2	Zfp36l1
Enox2	3.16E-52	0.325976	0.278	0.158	7.74E-48	cDC2	Enox2
Apobec11	3.17E-52	0.3693	0.529	0.373	7.76E-48	cDC2	Apobec1
Slc31a11	3.36E-52	0.648192	0.324	0.201	8.22E-48	cDC2	Slc31a1
Ndufb11	4.85E-52	0.517804	0.577	0.425	1.19E-47	cDC2	Ndufb11
Ubxn12	5.70E-52	0.507568	0.537	0.382	1.40E-47	cDC2	Ubxn1
Calm31	5.84E-52	0.577583	0.474	0.328	1.43E-47	cDC2	Calm3
Mrpl141	9.43E-52	0.622985	0.344	0.216	2.31E-47	cDC2	Mrpl14
Creg12	1.44E-51	0.598134	0.722	0.594	3.52E-47	cDC2	Creg1
Trmt1121	1.53E-51	0.49915	0.63	0.48	3.74E-47	cDC2	Trmt112
Filip1l1	3.01E-51	0.580225	0.615	0.474	7.37E-47	cDC2	Filip1l
Srp141	3.99E-51	0.564647	0.507	0.361	9.78E-47	cDC2	Srp14
Nucks1	5.41E-51	0.620417	0.346	0.219	1.32E-46	cDC2	Nucks1
1810058I2	6.80E-51	0.589198	0.392	0.258	1.66E-46	cDC2	1810058I24Rik
Etfb	7.77E-51	0.654773	0.404	0.273	1.90E-46	cDC2	Etfb
Myl61	9.22E-51	0.293571	0.768	0.602	2.26E-46	cDC2	Myl6
Cuta2	9.33E-51	0.505083	0.549	0.402	2.28E-46	cDC2	Cuta
Atp6v0a11	1.23E-50	0.423121	0.493	0.344	3.00E-46	cDC2	Atp6v0a1
Atp5d2	1.29E-50	0.442709	0.682	0.523	3.16E-46	cDC2	Atp5d
Gapvd11	1.49E-50	0.415101	0.407	0.267	3.65E-46	cDC2	Gapvd1
Cenpx2	1.51E-50	0.64432	0.408	0.275	3.71E-46	cDC2	Cenpx
Anxa61	1.71E-50	0.673063	0.297	0.18	4.18E-46	cDC2	Anxa6
Atp5o2	3.54E-50	0.510564	0.525	0.374	8.68E-46	cDC2	Atp5o
Mcfd23	3.59E-50	0.534611	0.414	0.275	8.79E-46	cDC2	Mcfd2
Rftn11	4.35E-50	0.278556	0.253	0.144	1.07E-45	cDC2	Rftn1
Tmem106c	4.40E-50	0.685592	0.251	0.144	1.08E-45	cDC2	Tmem106c
Cox6a12	4.74E-50	0.455292	0.662	0.508	1.16E-45	cDC2	Cox6a1
Eif4ebp11	6.08E-50	0.581998	0.328	0.204	1.49E-45	cDC2	Eif4ebp1
Eea1	1.32E-49	0.560998	0.288	0.172	3.23E-45	cDC2	Eea1
Hcfc1r12	1.40E-49	0.644765	0.389	0.261	3.42E-45	cDC2	Hcfc1r1
Ptov11	1.83E-49	0.61117	0.327	0.204	4.48E-45	cDC2	Ptov1
Gng122	2.40E-49	0.427594	0.433	0.293	5.87E-45	cDC2	Gng12
Slc43a2	3.07E-49	0.364214	0.412	0.271	7.52E-45	cDC2	Slc43a2
Gpr160	5.92E-49	0.472715	0.291	0.173	1.45E-44	cDC2	Gpr160
Tnfaip81	5.52E-48	0.428362	0.463	0.32	1.35E-43	cDC2	Tnfaip8
Pepd1	1.71E-47	0.537894	0.377	0.248	4.18E-43	cDC2	Pepd
Elob2	2.67E-47	0.423568	0.67	0.519	6.53E-43	cDC2	Elob
Dnajc151	5.51E-47	0.624733	0.316	0.2	1.35E-42	cDC2	Dnajc15
Rasal2	5.96E-47	0.337062	0.256	0.146	1.46E-42	cDC2	Rasal2
Polr2f2	7.63E-47	0.730685	0.304	0.192	1.87E-42	cDC2	Polr2f
Sqstm11	8.81E-47	0.502479	0.653	0.509	2.16E-42	cDC2	Sqstm1
Cox7a22	9.57E-47	0.452438	0.599	0.456	2.34E-42	cDC2	Cox7a2
Necap22	1.39E-46	0.521567	0.424	0.29	3.41E-42	cDC2	Necap2
Cox4i13	1.89E-46	0.279436	0.911	0.799	4.62E-42	cDC2	Cox4i1

G3bp21	1.92E-46	0.519229	0.468	0.331	4.70E-42	cDC2	G3bp2
Lman21	1.98E-46	0.474215	0.434	0.299	4.84E-42	cDC2	Lman2
Cox6c2	2.46E-46	0.381831	0.72	0.573	6.03E-42	cDC2	Cox6c
Rragc1	3.00E-46	0.657263	0.334	0.215	7.35E-42	cDC2	Rragc
Cd482	3.61E-46	0.411494	0.549	0.398	8.85E-42	cDC2	Cd48
Stat12	5.15E-46	0.487696	0.613	0.493	1.26E-41	cDC2	Stat1
Nampt1	9.88E-46	0.47274	0.31	0.193	2.42E-41	cDC2	Nampt
Tbca2	1.19E-45	0.479787	0.567	0.421	2.90E-41	cDC2	Tbca
Ndufa132	1.67E-45	0.409342	0.674	0.531	4.09E-41	cDC2	Ndufa13
Galnt2	1.83E-45	0.324774	0.26	0.151	4.48E-41	cDC2	Galnt2
Stx12	2.98E-45	0.586698	0.302	0.189	7.30E-41	cDC2	Stx12
Mrps211	6.93E-45	0.487637	0.477	0.341	1.70E-40	cDC2	Mrps21
Sesn12	1.00E-44	0.302147	0.443	0.297	2.45E-40	cDC2	Sesn1
Crlf22	1.15E-44	0.43924	0.411	0.273	2.81E-40	cDC2	Crlf2
Dek1	1.33E-44	0.538809	0.373	0.249	3.25E-40	cDC2	Dek
H2-DMb12	1.93E-44	0.502745	0.639	0.531	4.73E-40	cDC2	H2-DMb1
Atp6v0e1	3.02E-44	0.379303	0.7	0.558	7.40E-40	cDC2	Atp6v0e
Agpat3	3.37E-44	0.487619	0.386	0.256	8.24E-40	cDC2	Agpat3
Gclm1	3.46E-44	0.741639	0.305	0.196	8.47E-40	cDC2	Gclm
Renbp1	6.80E-44	0.531233	0.399	0.268	1.66E-39	cDC2	Renbp
Nap1l11	7.18E-44	0.579351	0.343	0.224	1.76E-39	cDC2	Nap1l1
Raly1	7.32E-44	0.553681	0.418	0.289	1.79E-39	cDC2	Raly
Pea15a1	1.13E-43	0.470723	0.461	0.323	2.76E-39	cDC2	Pea15a
Cltb	1.90E-43	0.648778	0.269	0.164	4.66E-39	cDC2	Cltb
Dynll2	2.12E-43	0.623472	0.283	0.176	5.20E-39	cDC2	Dynll2
Ptma1	2.37E-43	0.252178	0.85	0.722	5.81E-39	cDC2	Ptma
Stub1	2.94E-43	0.480522	0.427	0.297	7.20E-39	cDC2	Stub1
Mvp2	3.28E-43	0.585763	0.296	0.186	8.03E-39	cDC2	Mvp
Retreg2	4.09E-43	0.592295	0.307	0.196	1.00E-38	cDC2	Retreg2
Sh3gl11	5.75E-43	0.409569	0.404	0.275	1.41E-38	cDC2	Sh3gl1
Gbp22	6.60E-43	0.253765	0.313	0.199	1.62E-38	cDC2	Gbp2
Rabac11	1.02E-42	0.374889	0.655	0.518	2.51E-38	cDC2	Rabac1
Arpc1b2	1.87E-42	0.269598	0.819	0.699	4.57E-38	cDC2	Arpc1b
Smim141	2.61E-42	0.421865	0.435	0.301	6.39E-38	cDC2	Smim14
Micos101	2.81E-42	0.47515	0.467	0.33	6.89E-38	cDC2	Micos10
Pigp	4.67E-42	0.551362	0.306	0.195	1.14E-37	cDC2	Pigp
Cd471	4.68E-42	0.351461	0.596	0.455	1.15E-37	cDC2	Cd47
Pirb1	7.52E-42	0.368019	0.252	0.15	1.84E-37	cDC2	Pirb
Plaat32	8.94E-42	0.562062	0.317	0.204	2.19E-37	cDC2	Plaat3
5031439G(	1.32E-41	0.441593	0.358	0.236	3.24E-37	cDC2	5031439G07Rik
Ndufb22	1.78E-41	0.594671	0.36	0.242	4.36E-37	cDC2	Ndufb2
Atp13a21	2.19E-41	0.444233	0.398	0.271	5.36E-37	cDC2	Atp13a2
BC005537:	3.37E-41	0.511811	0.291	0.182	8.24E-37	cDC2	BC005537
Ralbp1	3.96E-41	0.478974	0.352	0.232	9.69E-37	cDC2	Ralbp1

Timm132	4.04E-41	0.491352	0.464	0.332	9.88E-37	cDC2	Timm13
Rnf145	4.66E-41	0.455808	0.286	0.178	1.14E-36	cDC2	Rnf145
Jpt11	5.72E-41	0.452471	0.49	0.354	1.40E-36	cDC2	Jpt1
Ubl52	9.54E-41	0.41835	0.623	0.478	2.34E-36	cDC2	Ubl5
Arpc51	1.16E-40	0.367584	0.565	0.425	2.85E-36	cDC2	Arpc5
Sdf2	1.28E-40	0.479746	0.36	0.241	3.12E-36	cDC2	Sdf2
Ap3d1	1.56E-40	0.433283	0.308	0.198	3.81E-36	cDC2	Ap3d1
Ifi271	1.59E-40	0.435819	0.464	0.331	3.90E-36	cDC2	Ifi27
Pink1	1.63E-40	0.610094	0.298	0.191	3.99E-36	cDC2	Pink1
Rad23b	1.80E-40	0.483203	0.307	0.197	4.41E-36	cDC2	Rad23b
Nme12	2.00E-40	0.533374	0.378	0.258	4.89E-36	cDC2	Nme1
Rab5c2	2.27E-40	0.521606	0.493	0.368	5.57E-36	cDC2	Rab5c
Mvb12a1	3.88E-40	0.533453	0.309	0.198	9.51E-36	cDC2	Mvb12a
Aldoa2	5.48E-40	0.330134	0.731	0.59	1.34E-35	cDC2	Aldoa
1810037I1	8.06E-40	0.45528	0.469	0.337	1.97E-35	cDC2	1810037I17Rik
Mapkapk21	9.23E-40	0.383889	0.455	0.324	2.26E-35	cDC2	Mapkapk2
Spred1	9.25E-40	0.280393	0.294	0.182	2.27E-35	cDC2	Spred1
mt-Nd4	1.10E-39	0.324335	0.847	0.737	2.70E-35	cDC2	mt-Nd4
Smim11	1.27E-39	0.634177	0.291	0.187	3.12E-35	cDC2	Smim11
Rnf1871	1.29E-39	0.544387	0.398	0.278	3.16E-35	cDC2	Rnf187
Psma52	1.85E-39	0.484563	0.428	0.304	4.52E-35	cDC2	Psma5
Med282	3.08E-39	0.459796	0.486	0.354	7.53E-35	cDC2	Med28
Phf20	3.30E-39	0.318016	0.333	0.216	8.08E-35	cDC2	Phf20
Eef1d3	4.59E-39	0.388205	0.635	0.489	1.12E-34	cDC2	Eef1d
Chmp4b2	4.84E-39	0.39477	0.598	0.46	1.19E-34	cDC2	Chmp4b
Serf22	6.22E-39	0.253177	0.871	0.755	1.52E-34	cDC2	Serf2
Pnp2	6.59E-39	0.428359	0.596	0.476	1.61E-34	cDC2	Pnp
Hdgf2	1.24E-38	0.677658	0.267	0.17	3.04E-34	cDC2	Hdgf
Tmem373	1.98E-38	0.438953	0.4	0.276	4.84E-34	cDC2	Tmem37
Cpq1	2.36E-38	0.255783	0.457	0.318	5.78E-34	cDC2	Cpq
Park72	2.50E-38	0.470033	0.536	0.405	6.13E-34	cDC2	Park7
Uqcr102	4.15E-38	0.423103	0.516	0.378	1.02E-33	cDC2	Uqcr10
Sdhc	5.61E-38	0.516218	0.291	0.188	1.37E-33	cDC2	Sdhc
Ndufa51	5.74E-38	0.577855	0.341	0.231	1.40E-33	cDC2	Ndufa5
Dpp71	7.01E-38	0.60987	0.265	0.166	1.72E-33	cDC2	Dpp7
Dnajb141	7.55E-38	0.465445	0.352	0.239	1.85E-33	cDC2	Dnajb14
Adrb22	8.68E-38	0.364215	0.499	0.359	2.12E-33	cDC2	Adrb2
Pitpna1	1.04E-37	0.318935	0.613	0.477	2.54E-33	cDC2	Pitpna
Ndufa22	1.85E-37	0.449284	0.563	0.432	4.54E-33	cDC2	Ndufa2
Ntan11	2.07E-37	0.538393	0.281	0.18	5.08E-33	cDC2	Ntan1
Gpi12	2.15E-37	0.348479	0.598	0.448	5.27E-33	cDC2	Gpi1
Atp6v0d11	2.51E-37	0.404533	0.497	0.367	6.14E-33	cDC2	Atp6v0d1
Epn1	2.85E-37	0.436423	0.486	0.36	6.99E-33	cDC2	Epn1
Asah13	4.30E-37	0.283928	0.768	0.669	1.05E-32	cDC2	Asah1

Romo11	4.33E-37	0.4934	0.382	0.266	1.06E-32	cDC2	Romo1
Higd2a3	4.44E-37	0.438873	0.536	0.41	1.09E-32	cDC2	Higd2a
Psmb42	4.45E-37	0.536144	0.394	0.278	1.09E-32	cDC2	Psmb4
Tnfrsf11a1	5.96E-37	0.265245	0.506	0.372	1.46E-32	cDC2	Tnfrsf11a
Wasl	7.19E-37	0.439251	0.316	0.208	1.76E-32	cDC2	Wasl
Sumo21	7.29E-37	0.371155	0.639	0.507	1.79E-32	cDC2	Sumo2
Hmgn12	7.44E-37	0.448743	0.447	0.322	1.82E-32	cDC2	Hmgn1
Atp5j3	1.17E-36	0.364653	0.678	0.538	2.86E-32	cDC2	Atp5j
Rab7	3.41E-36	0.40938	0.518	0.39	8.35E-32	cDC2	Rab7
Snu131	4.20E-36	0.453161	0.433	0.313	1.03E-31	cDC2	Snu13
Nme23	4.51E-36	0.335537	0.743	0.596	1.11E-31	cDC2	Nme2
Cep852	4.92E-36	0.534651	0.4	0.287	1.21E-31	cDC2	Cep85
Psmg41	5.81E-36	0.572637	0.274	0.178	1.42E-31	cDC2	Psmg4
Esd2	1.14E-35	0.421375	0.445	0.322	2.78E-31	cDC2	Esd
Eif4b2	1.21E-35	0.499618	0.398	0.282	2.97E-31	cDC2	Eif4b
Sgpl11	1.80E-35	0.346694	0.449	0.323	4.41E-31	cDC2	Sgpl1
Axl2	1.86E-35	0.469708	0.378	0.263	4.56E-31	cDC2	Axl
Washc2	2.08E-35	0.44327	0.321	0.216	5.09E-31	cDC2	Washc2
Trafd11	2.47E-35	0.360786	0.49	0.364	6.06E-31	cDC2	Trafd1
Hnrnpul21	4.25E-35	0.528371	0.338	0.231	1.04E-30	cDC2	Hnrnpul2
Atp5b2	1.16E-34	0.370328	0.665	0.53	2.84E-30	cDC2	Atp5b
Commd11	1.83E-34	0.490536	0.318	0.215	4.48E-30	cDC2	Commd1
Atp5j22	2.74E-34	0.358577	0.646	0.515	6.70E-30	cDC2	Atp5j2
Mrps242	3.59E-34	0.453921	0.387	0.275	8.80E-30	cDC2	Mrps24
Mrpl421	4.15E-34	0.591026	0.332	0.231	1.02E-29	cDC2	Mrpl42
Myl12a1	4.34E-34	0.352925	0.591	0.448	1.06E-29	cDC2	Myl12a
H2-T233	5.23E-34	0.321965	0.799	0.711	1.28E-29	cDC2	H2-T23
Abhd17a2	5.52E-34	0.572905	0.332	0.228	1.35E-29	cDC2	Abhd17a
Sod22	5.89E-34	0.507767	0.284	0.187	1.44E-29	cDC2	Sod2
Lmo22	7.97E-34	0.326032	0.583	0.451	1.95E-29	cDC2	Lmo2
Chmp2a1	1.21E-33	0.431997	0.42	0.304	2.96E-29	cDC2	Chmp2a
Rps27l2	1.55E-33	0.29885	0.482	0.355	3.79E-29	cDC2	Rps27l
Rps173	1.74E-33	0.319682	0.693	0.559	4.25E-29	cDC2	Rps17
Selenow2	2.49E-33	0.296468	0.604	0.456	6.09E-29	cDC2	Selenow
Vps291	3.22E-33	0.412857	0.432	0.313	7.88E-29	cDC2	Vps29
Irgm11	3.78E-33	0.45029	0.349	0.244	9.25E-29	cDC2	Irgm1
Prpf8	3.93E-33	0.406468	0.365	0.254	9.63E-29	cDC2	Prpf8
Mxd4	4.01E-33	0.445113	0.263	0.168	9.81E-29	cDC2	Mxd4
Cdk41	4.47E-33	0.499502	0.354	0.249	1.10E-28	cDC2	Cdk4
Fcho21	6.24E-33	0.269291	0.511	0.385	1.53E-28	cDC2	Fcho2
Eif5a2	9.21E-33	0.320968	0.626	0.487	2.25E-28	cDC2	Eif5a
Hmgb1	1.23E-32	0.344463	0.633	0.51	3.01E-28	cDC2	Hmgb1
Ndufb92	1.26E-32	0.395774	0.573	0.446	3.09E-28	cDC2	Ndufb9
Atp5g23	1.31E-32	0.268857	0.826	0.682	3.20E-28	cDC2	Atp5g2

S100a131	1.43E-32	0.48335	0.326	0.223	3.50E-28	cDC2	S100a13
Zc3h7a	1.82E-32	0.48637	0.598	0.487	4.45E-28	cDC2	Zc3h7a
Ddrgk12	1.97E-32	0.350023	0.523	0.395	4.82E-28	cDC2	Ddrgk1
Prelid12	1.97E-32	0.410002	0.453	0.332	4.83E-28	cDC2	Prelid1
2210016F1	2.23E-32	0.617586	0.279	0.185	5.47E-28	cDC2	2210016F16Rik
Lmbrd11	2.49E-32	0.318768	0.376	0.261	6.09E-28	cDC2	Lmbrd1
Il2rg2	2.61E-32	0.570677	0.313	0.218	6.39E-28	cDC2	Il2rg
Pdcd61	2.65E-32	0.602881	0.298	0.203	6.50E-28	cDC2	Pdcd6
Morf4l1	3.09E-32	0.3314	0.692	0.581	7.57E-28	cDC2	Morf4l1
Atp5a12	3.20E-32	0.366869	0.593	0.467	7.83E-28	cDC2	Atp5a1
Atp5g12	4.24E-32	0.364146	0.603	0.463	1.04E-27	cDC2	Atp5g1
Jund1	9.38E-32	0.371946	0.748	0.687	2.30E-27	cDC2	Jund
Pdcd6ip1	1.20E-31	0.410481	0.371	0.264	2.95E-27	cDC2	Pdcd6ip
Pnkd	1.53E-31	0.502963	0.276	0.182	3.74E-27	cDC2	Pnkd
Rasa1	1.72E-31	0.295332	0.313	0.209	4.21E-27	cDC2	Rasa1
Arpc1a	1.91E-31	0.436558	0.389	0.281	4.67E-27	cDC2	Arpc1a
Naca3	2.40E-31	0.268034	0.83	0.699	5.88E-27	cDC2	Naca
Tmem140	4.39E-31	0.38968	0.262	0.169	1.08E-26	cDC2	Tmem140
Map1lc3b1	5.18E-31	0.37111	0.457	0.342	1.27E-26	cDC2	Map1lc3b
Cacybp1	5.32E-31	0.510016	0.287	0.193	1.30E-26	cDC2	Cacybp
Coro1b2	7.22E-31	0.342464	0.592	0.466	1.77E-26	cDC2	Coro1b
Tsen34	7.69E-31	0.415666	0.297	0.199	1.88E-26	cDC2	Tsen34
Prdx21	8.34E-31	0.397341	0.465	0.347	2.04E-26	cDC2	Prdx2
Sypl1	1.30E-30	0.402263	0.399	0.289	3.19E-26	cDC2	Sypl
Rab24	1.68E-30	0.328468	0.343	0.237	4.12E-26	cDC2	Rab24
Hmgn2	2.27E-30	0.28832	0.43	0.318	5.57E-26	cDC2	Hmgn2
Zfyve21	3.09E-30	0.501545	0.256	0.167	7.56E-26	cDC2	Zfyve21
Dpm3	4.15E-30	0.400625	0.378	0.271	1.02E-25	cDC2	Dpm3
Ufc11	4.54E-30	0.478582	0.36	0.257	1.11E-25	cDC2	Ufc1
Sdhd2	5.74E-30	0.452845	0.377	0.273	1.41E-25	cDC2	Sdhd
Psmb102	6.19E-30	0.354598	0.497	0.379	1.51E-25	cDC2	Psmb10
Gtf2a2	6.63E-30	0.411187	0.31	0.212	1.62E-25	cDC2	Gtf2a2
Szrd11	7.13E-30	0.397224	0.29	0.196	1.75E-25	cDC2	Szrd1
Dstn	7.39E-30	0.345721	0.382	0.272	1.81E-25	cDC2	Dstn
Mid1ip11	7.81E-30	0.443622	0.316	0.216	1.91E-25	cDC2	Mid1ip1
Ahsa11	8.74E-30	0.565333	0.297	0.204	2.14E-25	cDC2	Ahsa1
Hp1bp31	3.13E-29	0.338796	0.374	0.265	7.66E-25	cDC2	Hp1bp3
Trim251	3.22E-29	0.434901	0.254	0.167	7.89E-25	cDC2	Trim25
Rnaseh2c1	8.45E-29	0.465695	0.321	0.224	2.07E-24	cDC2	Rnaseh2c
Card191	1.33E-28	0.384069	0.346	0.246	3.27E-24	cDC2	Card19
Ube2s1	2.27E-28	0.411459	0.353	0.252	5.56E-24	cDC2	Ube2s
Nadk1	2.43E-28	0.33158	0.345	0.246	5.94E-24	cDC2	Nadk
Hpcal12	2.71E-28	0.299331	0.361	0.258	6.65E-24	cDC2	Hpcal1
Psma72	3.70E-28	0.279596	0.533	0.409	9.05E-24	cDC2	Psma7

Banf12	3.80E-28	0.481085	0.368	0.266	9.31E-24	cDC2	Banf1
Drap11	4.31E-28	0.397739	0.463	0.346	1.06E-23	cDC2	Drap1
Ndufv32	4.41E-28	0.368364	0.488	0.37	1.08E-23	cDC2	Ndufv3
Ndufa62	5.16E-28	0.328978	0.643	0.517	1.26E-23	cDC2	Ndufa6
Tmbim41	6.90E-28	0.292008	0.551	0.432	1.69E-23	cDC2	Tmbim4
Aif13	1.03E-27	0.263952	0.815	0.699	2.53E-23	cDC2	Aif1
Snrpd22	1.07E-27	0.431708	0.362	0.261	2.61E-23	cDC2	Snrpd2
H2afv	1.14E-27	0.343538	0.344	0.243	2.79E-23	cDC2	H2afv
Slc9a3r11	2.53E-27	0.545566	0.258	0.174	6.19E-23	cDC2	Slc9a3r1
Lsm42	3.38E-27	0.49327	0.35	0.255	8.28E-23	cDC2	Lsm4
Bcl101	4.00E-27	0.451332	0.357	0.26	9.80E-23	cDC2	Bcl10
Tmx1	4.69E-27	0.354121	0.264	0.177	1.15E-22	cDC2	Tmx1
Polr2j1	4.73E-27	0.493516	0.287	0.2	1.16E-22	cDC2	Polr2j
Skp1a	5.98E-27	0.457269	0.44	0.338	1.47E-22	cDC2	Skp1a
Nt5c1	8.00E-27	0.460535	0.29	0.201	1.96E-22	cDC2	Nt5c
Ndufab11	1.13E-26	0.378897	0.441	0.331	2.76E-22	cDC2	Ndufab1
Dctn31	1.38E-26	0.407283	0.375	0.274	3.38E-22	cDC2	Dctn3
Slc25a391	1.39E-26	0.413168	0.4	0.297	3.40E-22	cDC2	Slc25a39
Reep51	1.47E-26	0.281612	0.687	0.573	3.60E-22	cDC2	Reep5
Lamtor31	2.21E-26	0.474162	0.26	0.177	5.42E-22	cDC2	Lamtor3
Atp6v1d1	3.15E-26	0.368844	0.381	0.279	7.72E-22	cDC2	Atp6v1d
Mrpl511	3.46E-26	0.517488	0.263	0.181	8.48E-22	cDC2	Mrpl51
Lamtor41	3.60E-26	0.30961	0.559	0.44	8.82E-22	cDC2	Lamtor4
Nisch1	4.55E-26	0.260995	0.465	0.351	1.11E-21	cDC2	Nisch
Vti1b	6.39E-26	0.353425	0.392	0.289	1.56E-21	cDC2	Vti1b
Dnase2a3	6.66E-26	0.268448	0.503	0.383	1.63E-21	cDC2	Dnase2a
Igsf61	7.65E-26	0.293651	0.318	0.224	1.87E-21	cDC2	Igsf6
Glmp3	8.57E-26	0.267843	0.587	0.464	2.10E-21	cDC2	Glmp
Gm420311	8.91E-26	0.425384	0.262	0.179	2.18E-21	cDC2	Gm42031
Rbx11	8.95E-26	0.307177	0.582	0.46	2.19E-21	cDC2	Rbx1
Rab5if2	9.01E-26	0.364243	0.498	0.393	2.21E-21	cDC2	Rab5if
Jarid2	1.06E-25	0.404945	0.432	0.331	2.60E-21	cDC2	Jarid2
Lamtor51	1.24E-25	0.455758	0.335	0.243	3.04E-21	cDC2	Lamtor5
Fkbp81	1.55E-25	0.401999	0.423	0.321	3.80E-21	cDC2	Fkbp8
Timm8b1	1.74E-25	0.410514	0.317	0.226	4.27E-21	cDC2	Timm8b
St131	1.85E-25	0.364431	0.36	0.261	4.53E-21	cDC2	St13
Fam50a2	2.36E-25	0.526217	0.257	0.177	5.79E-21	cDC2	Fam50a
Tln11	2.74E-25	0.276479	0.59	0.474	6.70E-21	cDC2	Tln1
Trappc41	4.35E-25	0.386583	0.347	0.253	1.07E-20	cDC2	Trappc4
Rasa42	8.05E-25	0.278874	0.452	0.343	1.97E-20	cDC2	Rasa4
Psma31	9.94E-25	0.3106	0.533	0.418	2.43E-20	cDC2	Psma3
Zmat21	1.20E-24	0.446318	0.314	0.226	2.95E-20	cDC2	Zmat2
Mrps33	1.37E-24	0.38977	0.429	0.326	3.37E-20	cDC2	Mrps33
Ndufb41	2.06E-24	0.348536	0.471	0.362	5.04E-20	cDC2	Ndufb4

Vwa5a	2.26E-24	0.332339	0.277	0.192	5.55E-20	cDC2	Vwa5a
Aurkaip12	6.90E-24	0.397492	0.35	0.26	1.69E-19	cDC2	Aurkaip1
Atp5mpl3	8.09E-24	0.363575	0.524	0.415	1.98E-19	cDC2	Atp5mpl
Tifa1	8.54E-24	0.266653	0.376	0.272	2.09E-19	cDC2	Tifa
Snrpd11	1.02E-23	0.418902	0.294	0.21	2.50E-19	cDC2	Snrpd1
Cox7b2	1.04E-23	0.324049	0.475	0.363	2.56E-19	cDC2	Cox7b
Chchd22	1.13E-23	0.340809	0.477	0.371	2.76E-19	cDC2	Chchd2
Casp11	1.71E-23	0.481726	0.256	0.179	4.20E-19	cDC2	Casp1
Sirt21	2.54E-23	0.327797	0.413	0.312	6.22E-19	cDC2	Sirt2
Zfp106	2.64E-23	0.422229	0.299	0.215	6.48E-19	cDC2	Zfp106
Ttc3	2.91E-23	0.259839	0.27	0.187	7.11E-19	cDC2	Ttc3
Mrps161	2.91E-23	0.454468	0.282	0.201	7.13E-19	cDC2	Mrps16
Cpne3	3.21E-23	0.340466	0.297	0.21	7.87E-19	cDC2	Cpne3
Spg212	3.53E-23	0.375918	0.329	0.239	8.65E-19	cDC2	Spg21
Sf3b21	3.56E-23	0.328777	0.477	0.372	8.71E-19	cDC2	Sf3b2
Atp1b31	3.63E-23	0.270667	0.546	0.436	8.89E-19	cDC2	Atp1b3
Ube2m1	5.48E-23	0.408138	0.408	0.31	1.34E-18	cDC2	Ube2m
Dusp7	6.65E-23	0.358177	0.257	0.176	1.63E-18	cDC2	Dusp7
Rnf72	7.13E-23	0.293361	0.48	0.371	1.75E-18	cDC2	Rnf7
Nudc1	8.75E-23	0.431755	0.369	0.277	2.14E-18	cDC2	Nudc
Mea12	1.23E-22	0.497635	0.276	0.197	3.02E-18	cDC2	Mea1
Mbd21	1.24E-22	0.287511	0.345	0.252	3.04E-18	cDC2	Mbd2
Dnajc31	1.45E-22	0.298502	0.49	0.383	3.56E-18	cDC2	Dnajc3
Rrp11	1.51E-22	0.351295	0.362	0.27	3.70E-18	cDC2	Rrp1
Cln81	1.76E-22	0.31502	0.273	0.191	4.31E-18	cDC2	Cln8
Ndufs51	1.86E-22	0.389409	0.428	0.33	4.56E-18	cDC2	Ndufs5
Pfdn21	2.25E-22	0.316922	0.367	0.274	5.52E-18	cDC2	Pfdn2
Grcc102	2.30E-22	0.256325	0.699	0.581	5.64E-18	cDC2	Grcc10
Ubald21	2.46E-22	0.32409	0.379	0.285	6.03E-18	cDC2	Ubald2
Mrpl302	4.79E-22	0.354481	0.377	0.281	1.17E-17	cDC2	Mrpl30
Gm162861	5.94E-22	0.379622	0.317	0.232	1.46E-17	cDC2	Gm16286
Snapc51	6.18E-22	0.372065	0.264	0.185	1.51E-17	cDC2	Snapc5
BC005624	6.24E-22	0.332008	0.32	0.234	1.53E-17	cDC2	BC005624
Atp6ap14	8.20E-22	0.261542	0.615	0.504	2.01E-17	cDC2	Atp6ap1
Selenof3	8.54E-22	0.250013	0.723	0.621	2.09E-17	cDC2	Selenof
Ten11	1.07E-21	0.38025	0.272	0.193	2.63E-17	cDC2	Ten1
Eif3d1	1.23E-21	0.42654	0.286	0.206	3.01E-17	cDC2	Eif3d
Ech1	1.30E-21	0.311867	0.356	0.264	3.18E-17	cDC2	Ech1
Lrrc58	1.47E-21	0.352208	0.286	0.203	3.60E-17	cDC2	Lrrc58
Atp6v1b22	1.80E-21	0.278	0.435	0.334	4.42E-17	cDC2	Atp6v1b2
Cmpk11	2.03E-21	0.342169	0.359	0.269	4.98E-17	cDC2	Cmpk1
Bcas21	2.79E-21	0.386263	0.306	0.223	6.83E-17	cDC2	Bcas2
Vps26a1	3.38E-21	0.335856	0.277	0.197	8.29E-17	cDC2	Vps26a
Sec61g2	3.63E-21	0.257664	0.543	0.432	8.88E-17	cDC2	Sec61g

Ndufv21	3.66E-21	0.343127	0.383	0.292	8.97E-17	cDC2	Ndufv2
Ilk1	5.23E-21	0.333714	0.383	0.289	1.28E-16	cDC2	Ilk
Atp1a1	6.37E-21	0.255724	0.447	0.347	1.56E-16	cDC2	Atp1a1
Cfdp1	6.69E-21	0.276193	0.277	0.197	1.64E-16	cDC2	Cfdp1
Orai3	7.49E-21	0.378252	0.27	0.192	1.84E-16	cDC2	Orai3
Dtnbp12	7.82E-21	0.255973	0.383	0.285	1.91E-16	cDC2	Dtnbp1
Plekhj1	8.75E-21	0.311853	0.388	0.295	2.14E-16	cDC2	Plekhj1
2-Jun	1.03E-20	0.356168	0.695	0.635	2.52E-16	cDC2	Jun
Uba11	1.06E-20	0.322287	0.284	0.204	2.58E-16	cDC2	Uba1
Ppp1r9b1	1.36E-20	0.352231	0.31	0.226	3.32E-16	cDC2	Ppp1r9b
Ik	1.40E-20	0.354216	0.314	0.231	3.43E-16	cDC2	Ik
Anp32a1	1.41E-20	0.257548	0.449	0.347	3.46E-16	cDC2	Anp32a
Flii1	1.44E-20	0.352866	0.313	0.229	3.52E-16	cDC2	Flii
Ssu72	1.61E-20	0.273581	0.465	0.364	3.94E-16	cDC2	Ssu72
Chmp51	1.96E-20	0.47948	0.299	0.22	4.81E-16	cDC2	Chmp5
Tmem141	2.46E-20	0.436812	0.251	0.177	6.01E-16	cDC2	Tmem141
Atxn7l3b1	4.26E-20	0.264953	0.396	0.301	1.04E-15	cDC2	Atxn7l3b
Mrps362	4.30E-20	0.413468	0.253	0.181	1.05E-15	cDC2	Mrps36
Sumo11	5.22E-20	0.314556	0.417	0.323	1.28E-15	cDC2	Sumo1
Znhit11	6.13E-20	0.390437	0.266	0.192	1.50E-15	cDC2	Znhit1
Degs1	6.24E-20	0.281408	0.329	0.242	1.53E-15	cDC2	Degs1
Trappc3	7.86E-20	0.311567	0.295	0.215	1.93E-15	cDC2	Trappc3
Pfdn12	7.93E-20	0.378544	0.266	0.191	1.94E-15	cDC2	Pfdn1
Vcp1	8.13E-20	0.324106	0.483	0.384	1.99E-15	cDC2	Vcp
Edf12	1.01E-19	0.263552	0.572	0.459	2.48E-15	cDC2	Edf1
Hagh	1.07E-19	0.296505	0.266	0.19	2.63E-15	cDC2	Hagh
Eif4e	1.33E-19	0.320695	0.263	0.187	3.26E-15	cDC2	Eif4e
Tomm72	1.71E-19	0.312059	0.483	0.383	4.20E-15	cDC2	Tomm7
Kdelr12	2.17E-19	0.292953	0.435	0.34	5.30E-15	cDC2	Kdelr1
Ndufb53	2.72E-19	0.285259	0.47	0.37	6.67E-15	cDC2	Ndufb5
Arhgdia2	2.87E-19	0.268639	0.642	0.53	7.02E-15	cDC2	Arhgdia
Snrpb1	2.93E-19	0.271084	0.504	0.399	7.18E-15	cDC2	Snrpb
Ndufb31	3.02E-19	0.340571	0.391	0.303	7.40E-15	cDC2	Ndufb3
Dynlrb11	3.11E-19	0.262317	0.413	0.319	7.62E-15	cDC2	Dynlrb1
Ppp4c1	3.15E-19	0.298405	0.434	0.339	7.72E-15	cDC2	Ppp4c
Mdh1	3.61E-19	0.334906	0.479	0.387	8.84E-15	cDC2	Mdh1
Ndufa82	3.81E-19	0.322489	0.417	0.321	9.34E-15	cDC2	Ndufa8
Psma22	3.83E-19	0.285823	0.45	0.355	9.39E-15	cDC2	Psma2
Txnl11	3.94E-19	0.364901	0.329	0.248	9.65E-15	cDC2	Txnl1
Phb1	4.14E-19	0.354664	0.3	0.221	1.01E-14	cDC2	Phb
Gemin7	5.63E-19	0.384534	0.257	0.185	1.38E-14	cDC2	Gemin7
Osgep1	5.73E-19	0.345944	0.292	0.215	1.40E-14	cDC2	Osgep
Secisbp2l	6.12E-19	0.289976	0.304	0.225	1.50E-14	cDC2	Secisbp2l
Gaa2	6.56E-19	0.280542	0.325	0.241	1.61E-14	cDC2	Gaa

Coa32	7.23E-19	0.304152	0.368	0.282	1.77E-14	cDC2	Coa3
Abhd16a	7.94E-19	0.290965	0.311	0.231	1.94E-14	cDC2	Abhd16a
Mrps18a1	1.31E-18	0.396297	0.269	0.196	3.21E-14	cDC2	Mrps18a
Map7d11	1.57E-18	0.276022	0.297	0.22	3.85E-14	cDC2	Map7d1
Ighm1	1.61E-18	0.278239	0.467	0.375	3.95E-14	cDC2	Ighm
Snrpd31	1.89E-18	0.337606	0.321	0.24	4.64E-14	cDC2	Snrpd3
Pttg11	2.40E-18	0.322681	0.281	0.206	5.87E-14	cDC2	Pttg1
0610010K1	2.57E-18	0.277794	0.339	0.256	6.29E-14	cDC2	0610010K14Rik
Eif3a2	2.76E-18	0.291041	0.431	0.34	6.76E-14	cDC2	Eif3a
Ssbp42	2.92E-18	0.290462	0.407	0.319	7.14E-14	cDC2	Ssbp4
Ndufb62	2.96E-18	0.410672	0.34	0.26	7.26E-14	cDC2	Ndufb6
2-Mar	3.17E-18	0.283355	0.273	0.199	7.77E-14	cDC2	2-Mar
Prdx61	3.18E-18	0.296871	0.269	0.196	7.80E-14	cDC2	Prdx6
Ptpmt11	3.26E-18	0.313833	0.294	0.217	7.98E-14	cDC2	Ptpmt1
Sri1	3.33E-18	0.254762	0.414	0.32	8.15E-14	cDC2	Sri
Dguok	4.20E-18	0.256474	0.323	0.242	1.03E-13	cDC2	Dguok
Cops91	4.34E-18	0.338544	0.454	0.364	1.06E-13	cDC2	Cops9
Tuba1b1	4.66E-18	0.290907	0.463	0.365	1.14E-13	cDC2	Tuba1b
Naa381	5.27E-18	0.35025	0.32	0.24	1.29E-13	cDC2	Naa38
Ormdl21	6.09E-18	0.325716	0.265	0.192	1.49E-13	cDC2	Ormdl2
Cct52	6.15E-18	0.28349	0.351	0.268	1.51E-13	cDC2	Cct5
Mbp1	6.90E-18	0.308979	0.395	0.315	1.69E-13	cDC2	Mbp
Srp91	7.34E-18	0.288938	0.464	0.367	1.80E-13	cDC2	Srp9
Ly6a2	8.51E-18	1.027308	0.316	0.259	2.08E-13	cDC2	Ly6a
Taf102	9.94E-18	0.254832	0.463	0.364	2.43E-13	cDC2	Taf10
Maf12	1.06E-17	0.277804	0.335	0.254	2.59E-13	cDC2	Maf1
Soat12	1.48E-17	0.300823	0.369	0.285	3.62E-13	cDC2	Soat1
Pdcd5	1.50E-17	0.358137	0.374	0.291	3.67E-13	cDC2	Pdcd5
Rnf5	1.62E-17	0.30273	0.31	0.233	3.97E-13	cDC2	Rnf5
Cdc372	1.95E-17	0.363388	0.408	0.323	4.77E-13	cDC2	Cdc37
Naxe1	2.66E-17	0.264719	0.377	0.291	6.50E-13	cDC2	Naxe
Sin3b2	2.83E-17	0.329756	0.352	0.27	6.93E-13	cDC2	Sin3b
Aamp	3.22E-17	0.289182	0.378	0.294	7.89E-13	cDC2	Aamp
Idh3b2	3.51E-17	0.33112	0.352	0.271	8.59E-13	cDC2	Idh3b
Rab11b	3.89E-17	0.273064	0.451	0.359	9.54E-13	cDC2	Rab11b
Etfa1	4.99E-17	0.285554	0.29	0.216	1.22E-12	cDC2	Etfa
Ndufs31	7.77E-17	0.260755	0.405	0.317	1.90E-12	cDC2	Ndufs3
Pnpla2	8.01E-17	0.273714	0.286	0.213	1.96E-12	cDC2	Pnpla2
Ndufc12	9.16E-17	0.28639	0.364	0.282	2.24E-12	cDC2	Ndufc1
Ddb1	9.72E-17	0.298733	0.256	0.187	2.38E-12	cDC2	Ddb1
Gtf2h51	1.49E-16	0.336345	0.27	0.201	3.64E-12	cDC2	Gtf2h5
Tomm52	1.85E-16	0.277528	0.27	0.2	4.54E-12	cDC2	Tomm5
Cdc123	2.45E-16	0.259088	0.278	0.206	6.01E-12	cDC2	Cdc123
Cwc151	3.09E-16	0.307893	0.391	0.309	7.56E-12	cDC2	Cwc15

Ppp2r1a1	5.31E-16	0.329096	0.306	0.235	1.30E-11	cDC2	Ppp2r1a
Uqcc22	5.57E-16	0.35245	0.36	0.284	1.36E-11	cDC2	Uqcc2
Ndufa122	7.41E-16	0.318428	0.333	0.259	1.81E-11	cDC2	Ndufa12
Chchd12	9.14E-16	0.336564	0.27	0.202	2.24E-11	cDC2	Chchd1
Tmem1602	1.28E-15	0.307136	0.372	0.291	3.13E-11	cDC2	Tmem160
Trappc6b	2.51E-15	0.287723	0.316	0.243	6.15E-11	cDC2	Trappc6b
Bloc1s12	2.59E-15	0.345581	0.294	0.225	6.34E-11	cDC2	Bloc1s1
Arf3	2.82E-15	0.280748	0.301	0.228	6.90E-11	cDC2	Arf3
Cops62	4.06E-15	0.310925	0.304	0.233	9.95E-11	cDC2	Cops6
Smc1a	4.52E-15	0.261938	0.276	0.207	1.11E-10	cDC2	Smc1a
Dnaja11	1.40E-14	0.27056	0.367	0.292	3.44E-10	cDC2	Dnaja1
Atp5md1	1.47E-14	0.251284	0.496	0.401	3.61E-10	cDC2	Atp5md
Psma62	1.92E-14	0.290295	0.341	0.269	4.69E-10	cDC2	Psma6
Snrpf2	2.44E-14	0.254762	0.346	0.27	5.98E-10	cDC2	Snrpf
Micos132	3.37E-14	0.306502	0.397	0.316	8.24E-10	cDC2	Micos13
Rheb1	5.40E-14	0.251456	0.386	0.305	1.32E-09	cDC2	Rheb
Mrpl181	5.43E-14	0.412054	0.256	0.194	1.33E-09	cDC2	Mrpl18
Cuedc21	5.89E-14	0.323947	0.372	0.298	1.44E-09	cDC2	Cuedc2
Hspd12	6.15E-14	0.330678	0.295	0.228	1.51E-09	cDC2	Hspd1
Psma41	6.61E-14	0.262224	0.312	0.241	1.62E-09	cDC2	Psma4
Ndufa111	7.38E-14	0.268869	0.431	0.35	1.81E-09	cDC2	Ndufa11
Trappc11	1.03E-13	0.3195	0.268	0.204	2.51E-09	cDC2	Trappc1
Lsm71	1.55E-13	0.264039	0.294	0.227	3.79E-09	cDC2	Lsm7
Fcgr43	3.04E-13	0.317808	0.305	0.241	7.43E-09	cDC2	Fcgr4
Mrpl172	3.42E-13	0.261714	0.336	0.264	8.38E-09	cDC2	Mrpl17
Ube2v12	5.02E-13	0.302043	0.356	0.283	1.23E-08	cDC2	Ube2v1
Ptges3	5.58E-13	0.289452	0.37	0.296	1.37E-08	cDC2	Ptges3
Copz11	6.13E-13	0.30264	0.29	0.224	1.50E-08	cDC2	Copz1
Hspa91	6.37E-13	0.26555	0.264	0.202	1.56E-08	cDC2	Hspa9
Copb21	9.26E-13	0.271658	0.258	0.197	2.27E-08	cDC2	Copb2
Psmd22	1.10E-12	0.316325	0.256	0.197	2.69E-08	cDC2	Psmd2
Tex2612	1.22E-12	0.298118	0.317	0.249	3.00E-08	cDC2	Tex261
Alyref1	1.96E-12	0.268145	0.271	0.209	4.81E-08	cDC2	Alyref
Ssna11	3.88E-12	0.275024	0.252	0.194	9.49E-08	cDC2	Ssna1
Ndufs62	4.03E-12	0.337204	0.269	0.21	9.87E-08	cDC2	Ndufs6
Eif3i2	4.08E-12	0.255668	0.401	0.324	9.98E-08	cDC2	Eif3i
Pdcl3	1.80E-11	0.260145	0.25	0.193	4.40E-07	cDC2	Pdcl3
Ssr3	3.58E-11	0.259816	0.284	0.224	8.78E-07	cDC2	Ssr3
Ube2q11	6.09E-11	0.275571	0.264	0.208	1.49E-06	cDC2	Ube2q1
Rbbp7	8.11E-11	0.263702	0.271	0.213	1.99E-06	cDC2	Rbbp7
Mrpl362	1.12E-10	0.267028	0.25	0.197	2.74E-06	cDC2	Mrpl36
Mrpl122	2.32E-10	0.268236	0.258	0.204	5.69E-06	cDC2	Mrpl12
Mrps141	2.71E-10	0.268241	0.31	0.248	6.62E-06	cDC2	Mrps14
Eif3m2	2.76E-10	0.276291	0.344	0.281	6.76E-06	cDC2	Eif3m

Snrpc1	1.47E-09	0.256563	0.291	0.235	3.60E-05	cDC2	Snrpc
Hbb-bs	0	5.857383	0.622	0.138	0	Platelets	Hbb-bs
Hba-a1	0	5.681333	0.597	0.12	0	Platelets	Hba-a1
Hba-a2	0	5.690926	0.573	0.102	0	Platelets	Hba-a2
Hbb-bt	0	6.150924	0.429	0.034	0	Platelets	Hbb-bt
Ttr2	0	2.4259	0.832	0.493	0	Platelets	Ttr
Mt31	0	2.561694	0.523	0.204	0	Platelets	Mt3
Enpp21	0	2.426576	0.513	0.215	0	Platelets	Enpp2
Alas2	0	6.047113	0.301	0.015	0	Platelets	Alas2
Cpe1	0	2.733512	0.423	0.146	0	Platelets	Cpe
Slc1a2	0	2.703604	0.335	0.11	0	Platelets	Slc1a2
Lars21	0	1.40424	0.889	0.749	0	Platelets	Lars2
mt-Atp61	0	1.152173	0.954	0.912	0	Platelets	mt-Atp6
mt-Cytb1	0	1.132472	0.98	0.949	0	Platelets	mt-Cytb
mt-Co11	0	0.97225	0.986	0.962	0	Platelets	mt-Co1
Gm424181	0	1.722316	0.997	0.981	0	Platelets	Gm42418
mt-Co31	1.27E-299	0.992046	0.958	0.94	3.12E-295	Platelets	mt-Co3
AY0361181	8.15E-288	1.511184	0.885	0.803	2.00E-283	Platelets	AY036118
Cmss11	1.28E-278	0.898424	0.959	0.922	3.13E-274	Platelets	Cmss1
Clu	8.74E-275	2.459491	0.332	0.117	2.14E-270	Platelets	Clu
mt-Co21	1.66E-272	0.885394	0.962	0.945	4.08E-268	Platelets	mt-Co2
Mbp2	2.48E-272	2.100857	0.542	0.307	6.08E-268	Platelets	Mbp
Camk1d1	1.42E-269	1.297491	0.862	0.806	3.47E-265	Platelets	Camk1d
Sparcl1	9.41E-260	2.488492	0.322	0.115	2.30E-255	Platelets	Sparcl1
Pcsk1n	1.03E-248	2.548144	0.29	0.098	2.51E-244	Platelets	Pcsk1n
mt-Nd41	1.99E-244	1.19643	0.809	0.739	4.88E-240	Platelets	mt-Nd4
Aldoc	8.48E-243	2.600916	0.298	0.105	2.08E-238	Platelets	Aldoc
Ptgds1	4.14E-242	2.385116	0.481	0.243	1.01E-237	Platelets	Ptgds
Il31ra1	1.70E-239	1.522116	0.738	0.61	4.16E-235	Platelets	Il31ra
mt-Nd21	2.98E-228	1.053884	0.844	0.801	7.30E-224	Platelets	mt-Nd2
Ndrg2	8.95E-215	2.618287	0.276	0.099	2.19E-210	Platelets	Ndrg2
Mgp	5.35E-179	2.816725	0.259	0.099	1.31E-174	Platelets	Mgp
Gphn1	1.27E-141	1.17528	0.706	0.639	3.11E-137	Platelets	Gphn
mt-Nd1	5.36E-132	0.86866	0.79	0.795	1.31E-127	Platelets	mt-Nd1
Dbi2	5.36E-130	1.61153	0.544	0.432	1.31E-125	Platelets	Dbi
mt-Nd4l1	3.64E-129	0.84307	0.816	0.825	8.91E-125	Platelets	mt-Nd4l
Cdk82	2.63E-118	0.529681	0.931	0.901	6.44E-114	Platelets	Cdk8
mt-Nd5	7.52E-115	1.19675	0.651	0.608	1.84E-110	Platelets	mt-Nd5
Camk2n1	4.68E-97	1.875136	0.361	0.231	1.15E-92	Platelets	Camk2n1
Chchd10	5.20E-95	2.130074	0.253	0.132	1.27E-90	Platelets	Chchd10
Mt12	3.73E-84	1.092497	0.571	0.505	9.13E-80	Platelets	Mt1
Gm199511	8.61E-71	1.533691	0.444	0.363	2.11E-66	Platelets	Gm19951
Ckb3	3.86E-54	0.728317	0.652	0.67	9.44E-50	Platelets	Ckb
Cox8a3	7.03E-53	0.629727	0.764	0.787	1.72E-48	Platelets	Cox8a

Selenow3	1.37E-51	1.133693	0.505	0.462	3.34E-47	Platelets	Selenow
Snrpd23	1.73E-31	0.295124	0.149	0.274	4.23E-27	Platelets	Snrpd2
Oaz12	2.35E-31	0.61413	0.66	0.727	5.77E-27	Platelets	Oaz1
Ndufb32	6.40E-31	0.256924	0.179	0.315	1.57E-26	Platelets	Ndufb3
Ndufa83	2.38E-30	0.276498	0.192	0.334	5.82E-26	Platelets	Ndufa8
Uqcrfs12	2.59E-30	0.261333	0.19	0.329	6.35E-26	Platelets	Uqcrfs1
Ube2v13	7.69E-30	0.332898	0.166	0.295	1.88E-25	Platelets	Ube2v1
Aurkaip13	8.73E-29	0.327067	0.15	0.271	2.14E-24	Platelets	Aurkaip1
Mrps331	4.35E-28	0.254864	0.202	0.339	1.07E-23	Platelets	Mrps33
Eif5	5.95E-28	0.26818	0.216	0.358	1.46E-23	Platelets	Eif5
Ndufv22	1.70E-27	0.336448	0.176	0.305	4.15E-23	Platelets	Ndufv2
Ndufb42	2.69E-27	0.26407	0.229	0.376	6.59E-23	Platelets	Ndufb4
Gnas2	2.94E-27	0.496101	0.674	0.733	7.21E-23	Platelets	Gnas
Rnf73	5.05E-25	0.268441	0.238	0.385	1.24E-20	Platelets	Rnf7
Mbd31	1.23E-23	0.28582	0.151	0.259	3.00E-19	Platelets	Mbd3
Cox4i14	1.42E-23	0.396453	0.751	0.808	3.48E-19	Platelets	Cox4i1
Prkar1a	1.45E-23	0.267331	0.242	0.386	3.54E-19	Platelets	Prkar1a
Nme13	1.67E-23	0.360997	0.16	0.271	4.09E-19	Platelets	Nme1
Mdh22	1.69E-23	0.29943	0.228	0.366	4.13E-19	Platelets	Mdh2
Cyc1	4.16E-23	0.269533	0.191	0.311	1.02E-18	Platelets	Cyc1
Dnaja12	4.32E-23	0.287429	0.184	0.302	1.06E-18	Platelets	Dnaja1
Dynlrb12	5.93E-23	0.351556	0.204	0.332	1.45E-18	Platelets	Dynlrb1
Bag12	8.38E-23	0.316341	0.195	0.318	2.05E-18	Platelets	Bag1
Smim142	8.57E-23	0.284946	0.195	0.315	2.10E-18	Platelets	Smim14
Ube2s2	1.53E-22	0.251751	0.157	0.263	3.75E-18	Platelets	Ube2s
Hcfc1r13	2.51E-22	0.372188	0.164	0.274	6.14E-18	Platelets	Hcfc1r1
Scp22	5.01E-22	0.363826	0.219	0.349	1.23E-17	Platelets	Scp2
Swi52	6.36E-22	0.328907	0.25	0.39	1.56E-17	Platelets	Swi5
Mpc2	9.84E-22	0.304496	0.22	0.348	2.41E-17	Platelets	Mpc2
Ndufab12	1.40E-21	0.354918	0.215	0.344	3.43E-17	Platelets	Ndufab1
Csde11	1.40E-21	0.276422	0.172	0.28	3.43E-17	Platelets	Csde1
Uqcrb3	1.76E-21	0.29526	0.283	0.435	4.31E-17	Platelets	Uqcrb
Micos102	7.66E-21	0.374296	0.217	0.345	1.87E-16	Platelets	Micos10
Ndufb83	1.57E-20	0.348149	0.26	0.405	3.83E-16	Platelets	Ndufb8
Nfic	3.46E-20	0.250726	0.164	0.265	8.47E-16	Platelets	Nfic
Paip2	3.87E-20	0.290894	0.192	0.304	9.47E-16	Platelets	Paip2
Drap12	4.75E-20	0.335622	0.229	0.359	1.16E-15	Platelets	Drap1
7-Sep	3.57E-19	0.341734	0.211	0.328	8.73E-15	Platelets	7-Sep
Rnf1872	8.81E-19	0.367126	0.184	0.291	2.16E-14	Platelets	Rnf187
Nenf1	1.24E-18	0.423136	0.193	0.304	3.04E-14	Platelets	Nenf
Gm480992	1.37E-18	1.403149	0.305	0.275	3.35E-14	Platelets	Gm48099
Ndufb23	3.89E-18	0.551041	0.156	0.254	9.53E-14	Platelets	Ndufb2
Ndufb111	5.25E-18	0.302409	0.294	0.442	1.29E-13	Platelets	Ndufb11
Fkbp1a2	7.52E-18	0.335183	0.225	0.345	1.84E-13	Platelets	Fkbp1a

Nudc2	1.08E-17	0.414462	0.184	0.288	2.65E-13	Platelets	Nudc
Uqcc23	1.85E-17	0.485662	0.188	0.294	4.54E-13	Platelets	Uqcc2
1810037I1	3.58E-17	0.443092	0.229	0.352	8.76E-13	Platelets	1810037I17Rik
Cox7b3	8.38E-17	0.407365	0.25	0.377	2.05E-12	Platelets	Cox7b
Ube2m2	1.21E-16	0.350763	0.21	0.321	2.97E-12	Platelets	Ube2m
Mlf21	1.35E-16	0.338428	0.264	0.396	3.30E-12	Platelets	Mlf2
Mrfap12	1.60E-16	0.326234	0.289	0.434	3.93E-12	Platelets	Mrfap1
Etfb1	2.36E-16	0.566864	0.183	0.286	5.78E-12	Platelets	Etfb
Hmgn13	3.26E-16	0.40823	0.222	0.336	7.98E-12	Platelets	Hmgn1
Ndufa73	1.37E-15	0.301876	0.348	0.507	3.36E-11	Platelets	Ndufa7
Ndufa123	4.52E-15	0.567207	0.174	0.269	1.11E-10	Platelets	Ndufa12
Mtch11	4.79E-15	0.401085	0.235	0.352	1.17E-10	Platelets	Mtch1
Ndufb63	9.41E-15	0.584525	0.175	0.27	2.31E-10	Platelets	Ndufb6
Zc3h7a1	2.16E-14	0.984997	0.463	0.495	5.30E-10	Platelets	Zc3h7a
Pgk12	2.89E-14	0.548194	0.174	0.266	7.07E-10	Platelets	Pgk1
Atp5a13	3.19E-14	0.319017	0.332	0.482	7.81E-10	Platelets	Atp5a1
Ubb3	4.19E-14	0.286652	0.83	0.869	1.03E-09	Platelets	Ubb
Gm155642	5.42E-14	1.305368	0.26	0.232	1.33E-09	Platelets	Gm15564
Vdac11	1.12E-13	0.51267	0.174	0.265	2.75E-09	Platelets	Vdac1
Pgam12	1.20E-13	0.366616	0.246	0.358	2.93E-09	Platelets	Pgam1
Prrc2a	1.56E-13	0.464771	0.212	0.315	3.82E-09	Platelets	Prrc2a
Cox6c3	4.61E-13	0.782468	0.518	0.585	1.13E-08	Platelets	Cox6c
Ndufb102	4.62E-13	0.519411	0.28	0.411	1.13E-08	Platelets	Ndufb10
Gstp1	5.15E-13	0.549921	0.2	0.297	1.26E-08	Platelets	Gstp1
Eno12	7.77E-13	0.448356	0.211	0.307	1.90E-08	Platelets	Eno1
Ndufa112	8.82E-13	0.493962	0.245	0.361	2.16E-08	Platelets	Ndufa11
Dstn1	1.01E-12	0.480348	0.192	0.283	2.47E-08	Platelets	Dstn
Cox7a23	2.26E-12	0.402873	0.33	0.472	5.52E-08	Platelets	Cox7a2
Fis12	4.11E-12	0.338266	0.336	0.479	1.01E-07	Platelets	Fis1
Tax1bp1	5.15E-12	0.46588	0.249	0.361	1.26E-07	Platelets	Tax1bp1
Gm269171	4.34E-11	0.498305	0.428	0.426	1.06E-06	Platelets	Gm26917
Aplp22	8.31E-11	0.5138	0.197	0.284	2.04E-06	Platelets	Aplp2
Pea15a2	1.03E-10	0.448967	0.236	0.336	2.52E-06	Platelets	Pea15a
Mpc11	1.31E-10	0.300564	0.351	0.492	3.20E-06	Platelets	Mpc1
Uqcr103	1.81E-10	0.547759	0.276	0.393	4.43E-06	Platelets	Uqcr10
Pfdn22	2.74E-10	0.604154	0.196	0.284	6.70E-06	Platelets	Pfdn2
Mkrn11	3.27E-10	1.986169	0.298	0.303	8.00E-06	Platelets	Mkrn1
Kif5b1	4.46E-10	0.479321	0.235	0.333	1.09E-05	Platelets	Kif5b
Dynll11	5.41E-10	0.398288	0.309	0.429	1.33E-05	Platelets	Dynll1
1810058I2	7.60E-10	0.609859	0.188	0.27	1.86E-05	Platelets	1810058I24Rik
Calm12	1.19E-09	0.465356	0.548	0.598	2.92E-05	Platelets	Calm1
Ndufc13	1.64E-09	0.625989	0.204	0.291	4.02E-05	Platelets	Ndufc1
Atp5d3	1.83E-09	0.339258	0.388	0.54	4.48E-05	Platelets	Atp5d
Purb	3.79E-09	0.470402	0.227	0.315	9.27E-05	Platelets	Purb

Uqcrq2	4.94E-09	0.418066	0.322	0.448	0.000121	Platelets	Uqcrq
Smdt12	6.20E-09	0.429404	0.307	0.427	0.000152	Platelets	Smdt1
Ywhae	9.81E-09	0.321442	0.397	0.555	0.00024	Platelets	Ywhae
Eif4g2	1.17E-08	0.422988	0.328	0.457	0.000286	Platelets	Eif4g2
Ptms2	1.89E-08	0.328306	0.388	0.533	0.000462	Platelets	Ptms
Slc25a392	1.93E-08	1.400401	0.296	0.303	0.000473	Platelets	Slc25a39
Ube2h1	3.05E-08	0.302201	0.235	0.315	0.000747	Platelets	Ube2h
Tspan131	6.15E-08	0.356938	0.278	0.373	0.001507	Platelets	Tspan13
Bsg3	7.03E-08	0.53856	0.586	0.686	0.001722	Platelets	Bsg
Hist1h1e2	8.66E-08	0.291579	0.215	0.289	0.002121	Platelets	Hist1h1e
Filip1l2	1.13E-07	0.871351	0.441	0.484	0.00276	Platelets	Filip1l
Cox6b13	1.44E-07	0.271817	0.42	0.568	0.003526	Platelets	Cox6b1
Ctnnb11	2.28E-07	0.543598	0.262	0.359	0.005595	Platelets	Ctnnb1
Atp5md2	5.77E-07	0.563263	0.301	0.413	0.014121	Platelets	Atp5md
Slc38a21	8.59E-07	0.587843	0.2	0.27	0.021038	Platelets	Slc38a2
Hsp90aa1	2.19E-06	0.605131	0.248	0.336	0.053595	Platelets	Hsp90aa1
Ndufs52	3.19E-06	0.644013	0.25	0.341	0.078233	Platelets	Ndufs5
Atp5o3	3.32E-06	0.613635	0.284	0.388	0.081337	Platelets	Atp5o
Ifi272	1.24E-05	0.491809	0.261	0.343	0.304592	Platelets	Ifi27
Tjp12	1.43E-05	0.276349	0.215	0.274	0.350238	Platelets	Tjp1
Gpx42	1.73E-05	0.585524	0.519	0.604	0.423444	Platelets	Gpx4
Ghitm	2.02E-05	0.657902	0.24	0.322	0.494287	Platelets	Ghitm
Elob3	0.000134	0.428063	0.403	0.534	1	Platelets	Elob
Jarid21	0.000432	1.10675	0.314	0.338	1	Platelets	Jarid2
Uqcrh3	0.001006	0.265344	0.475	0.609	1	Platelets	Uqcrh
Map1lc3b2	0.001304	0.661075	0.273	0.353	1	Platelets	Map1lc3b
Skp1a1	0.003065	0.700221	0.269	0.348	1	Platelets	Skp1a
Prdx22	0.00402	1.179732	0.318	0.356	1	Platelets	Prdx2
Atp5g33	0.004761	0.604341	0.503	0.601	1	Platelets	Atp5g3
Bnip3l	0.005836	1.048003	0.321	0.362	1	Platelets	Bnip3l
Isg151	0	2.994082	0.789	0.186	0	TAM-Inflam	Isg15
Iigp12	0	3.408628	0.697	0.13	0	TAM-Inflam	Iigp1
Oasl22	0	2.72154	0.736	0.175	0	TAM-Inflam	Oasl2
Ifit31	0	3.944311	0.657	0.105	0	TAM-Inflam	Ifit3
Rtp42	0	2.272897	0.771	0.232	0	TAM-Inflam	Rtp4
Ccl123	0	2.934285	0.924	0.408	0	TAM-Inflam	Ccl12
Irf72	0	2.344501	0.686	0.176	0	TAM-Inflam	Irf7
Gm49512	0	2.161437	0.737	0.236	0	TAM-Inflam	Gm4951
Rnf2131	0	2.153879	0.828	0.329	0	TAM-Inflam	Rnf213
Gbp23	0	2.71215	0.682	0.188	0	TAM-Inflam	Gbp2
Parp141	0	2.274459	0.751	0.257	0	TAM-Inflam	Parp14
Ifit21	0	4.064802	0.561	0.073	0	TAM-Inflam	Ifit2
Ly6a3	0	1.9344	0.728	0.246	0	TAM-Inflam	Ly6a
Irgm12	0	2.15319	0.709	0.233	0	TAM-Inflam	Irgm1

Ifi2042	0	2.455964	0.635	0.162	0	TAM-Inflam	Ifi204
Usp181	0	3.359244	0.555	0.093	0	TAM-Inflam	Usp18
Ifitm32	0	1.153151	0.875	0.418	0	TAM-Inflam	Ifitm3
Stat21	0	2.207602	0.635	0.178	0	TAM-Inflam	Stat2
Fgl22	0	1.910941	0.716	0.26	0	TAM-Inflam	Fgl2
Zbp13	0	2.169814	0.619	0.167	0	TAM-Inflam	Zbp1
Tor3a2	0	2.133436	0.728	0.278	0	TAM-Inflam	Tor3a
Stat13	0	1.736069	0.932	0.484	0	TAM-Inflam	Stat1
Igtp2	0	2.214388	0.648	0.207	0	TAM-Inflam	Igtp
Trim30a2	0	1.774808	0.751	0.312	0	TAM-Inflam	Trim30a
Herc61	0	2.438425	0.586	0.152	0	TAM-Inflam	Herc6
Ccl23	0	2.374789	0.551	0.139	0	TAM-Inflam	Ccl2
Xaf11	0	2.083236	0.596	0.184	0	TAM-Inflam	Xaf1
Cxcl10	0	4.584197	0.454	0.049	0	TAM-Inflam	Cxcl10
Tap11	0	1.505214	0.772	0.379	0	TAM-Inflam	Tap1
Eif2ak21	0	1.793418	0.6	0.212	0	TAM-Inflam	Eif2ak2
Fcgr44	0	1.871818	0.616	0.232	0	TAM-Inflam	Fcgr4
Ifi2131	0	2.74942	0.465	0.087	0	TAM-Inflam	Ifi213
Oasl1	0	3.868355	0.422	0.049	0	TAM-Inflam	Oasl1
Lgals3bp2	0	1.429143	0.898	0.525	0	TAM-Inflam	Lgals3bp
Gbp51	0	2.824742	0.485	0.117	0	TAM-Inflam	Gbp5
Slfn52	0	2.213797	0.49	0.123	0	TAM-Inflam	Slfn5
Bst23	0	1.435396	0.944	0.582	0	TAM-Inflam	Bst2
Phf11b1	0	2.231452	0.512	0.15	0	TAM-Inflam	Phf11b
Gbp61	0	2.980627	0.44	0.079	0	TAM-Inflam	Gbp6
Phf11d1	0	2.716482	0.463	0.104	0	TAM-Inflam	Phf11d
Oas1a2	0	2.052522	0.499	0.148	0	TAM-Inflam	Oas1a
Tgtp22	0	2.371091	0.43	0.099	0	TAM-Inflam	Tgtp2
Ifi2091	0	2.176795	0.428	0.104	0	TAM-Inflam	Ifi209
Ifit1	0	3.815339	0.342	0.037	0	TAM-Inflam	Ifit1
Ifi2111	0	2.591828	0.38	0.079	0	TAM-Inflam	Ifi211
Socs11	0	2.581444	0.383	0.082	0	TAM-Inflam	Socs1
Hcar21	0	2.54649	0.386	0.093	0	TAM-Inflam	Hcar2
Gbp4	0	2.728433	0.359	0.069	0	TAM-Inflam	Gbp4
Cxcl91	0	3.251417	0.358	0.08	0	TAM-Inflam	Cxcl9
Ifi2061	0	3.061362	0.318	0.046	0	TAM-Inflam	Ifi206
Rsad2	0	3.443962	0.309	0.041	0	TAM-Inflam	Rsad2
A330040F1	0	3.002198	0.313	0.048	0	TAM-Inflam	A330040F15Rik
Ifit3b	0	4.045764	0.291	0.028	0	TAM-Inflam	Ifit3b
H2-T234	0	1.130832	0.958	0.708	0	TAM-Inflam	H2-T23
Serpina3f	0	3.22806	0.292	0.044	0	TAM-Inflam	Serpina3f
Pml1	5.79E-304	2.237887	0.402	0.108	1.42E-299	TAM-Inflam	Pml
Nlrc51	3.16E-303	1.69028	0.614	0.23	7.75E-299	TAM-Inflam	Nlrc5
H2-K12	3.40E-303	0.888687	0.987	0.877	8.33E-299	TAM-Inflam	H2-K1

Parp91	3.09E-299	1.723208	0.584	0.215	7.57E-295	TAM-Inflam	Parp9
Fcgr13	1.26E-295	1.32527	0.888	0.575	3.09E-291	TAM-Inflam	Fcgr1
Ifih11	1.42E-291	2.240315	0.467	0.148	3.48E-287	TAM-Inflam	Ifih1
Ifi472	5.08E-291	1.819004	0.386	0.101	1.24E-286	TAM-Inflam	Ifi47
Clec2d2	2.82E-286	1.664957	0.632	0.255	6.91E-282	TAM-Inflam	Clec2d
H2-Q73	3.09E-274	1.27202	0.838	0.474	7.57E-270	TAM-Inflam	H2-Q7
Cd2742	8.72E-263	2.402106	0.414	0.128	2.13E-258	TAM-Inflam	Cd274
Sp1001	1.11E-260	1.491719	0.635	0.267	2.72E-256	TAM-Inflam	Sp100
Parp121	5.22E-259	1.747818	0.475	0.16	1.28E-254	TAM-Inflam	Parp12
C4b3	1.75E-258	1.531782	0.574	0.222	4.29E-254	TAM-Inflam	C4b
Ifi2072	2.66E-252	1.650824	0.487	0.169	6.52E-248	TAM-Inflam	Ifi207
H2-Oa3	7.26E-252	1.43355	0.685	0.318	1.78E-247	TAM-Inflam	H2-Oa
Ifi27l2a3	2.17E-251	1.119028	0.758	0.375	5.32E-247	TAM-Inflam	Ifi27l2a
Slfn81	2.34E-251	1.568503	0.527	0.192	5.72E-247	TAM-Inflam	Slfn8
Oas2	6.51E-250	2.234583	0.263	0.057	1.59E-245	TAM-Inflam	Oas2
Ly6e3	7.42E-250	0.743869	0.987	0.862	1.82E-245	TAM-Inflam	Ly6e
Samhd11	5.32E-245	1.214508	0.748	0.383	1.30E-240	TAM-Inflam	Samhd1
Cd744	1.62E-242	1.14172	0.954	0.769	3.96E-238	TAM-Inflam	Cd74
Ifi351	5.32E-241	1.571696	0.569	0.232	1.30E-236	TAM-Inflam	Ifi35
Cd401	2.22E-239	2.403246	0.26	0.059	5.44E-235	TAM-Inflam	Cd40
Il18bp3	5.87E-239	1.800889	0.429	0.142	1.44E-234	TAM-Inflam	Il18bp
Ddx581	3.78E-236	1.818719	0.403	0.126	9.27E-232	TAM-Inflam	Ddx58
Dhx581	1.85E-235	2.059496	0.332	0.091	4.53E-231	TAM-Inflam	Dhx58
Mar-51	2.31E-235	1.626598	0.557	0.233	5.66E-231	TAM-Inflam	5-Mar
B2m2	1.88E-232	0.65885	0.99	0.907	4.62E-228	TAM-Inflam	B2m
Znfx11	9.59E-231	1.552448	0.597	0.265	2.35E-226	TAM-Inflam	Znfx1
H2-D12	1.20E-229	0.693638	0.987	0.901	2.94E-225	TAM-Inflam	H2-D1
Ctss4	1.21E-227	0.67483	0.999	0.931	2.97E-223	TAM-Inflam	Ctss
Slfn22	1.65E-224	1.225029	0.642	0.286	4.05E-220	TAM-Inflam	Slfn2
H2-Eb14	7.30E-223	1.052911	0.887	0.586	1.79E-218	TAM-Inflam	H2-Eb1
Carmil1	8.61E-222	1.990399	0.316	0.085	2.11E-217	TAM-Inflam	Carmil1
Trim30d1	4.33E-221	1.566621	0.478	0.173	1.06E-216	TAM-Inflam	Trim30d
H2-Ab14	1.31E-219	1.039802	0.912	0.639	3.21E-215	TAM-Inflam	H2-Ab1
Gbp72	8.78E-219	1.587625	0.487	0.184	2.15E-214	TAM-Inflam	Gbp7
H2-Aa4	7.51E-215	1.044435	0.888	0.608	1.84E-210	TAM-Inflam	H2-Aa
Trafd12	5.60E-212	1.286887	0.704	0.359	1.37E-207	TAM-Inflam	Trafd1
H2-Q43	8.99E-211	1.190082	0.767	0.432	2.20E-206	TAM-Inflam	H2-Q4
Ccl52	1.60E-210	1.593283	0.426	0.152	3.91E-206	TAM-Inflam	Ccl5
Slamf82	6.56E-205	1.59325	0.481	0.188	1.61E-200	TAM-Inflam	Slamf8
C1qa4	1.09E-204	0.611477	0.994	0.871	2.67E-200	TAM-Inflam	C1qa
Cfb2	3.08E-204	1.540779	0.332	0.099	7.54E-200	TAM-Inflam	Cfb
Cst72	6.30E-197	1.590943	0.473	0.184	1.54E-192	TAM-Inflam	Cst7
Irf12	3.81E-196	1.438125	0.648	0.32	9.34E-192	TAM-Inflam	Irf1
Lap32	6.58E-195	1.255354	0.672	0.353	1.61E-190	TAM-Inflam	Lap3

Ube2l61	3.61E-194	1.274947	0.43	0.158	8.83E-190	TAM-Inflam	Ube2l6
Parp111	3.16E-192	1.849558	0.327	0.101	7.73E-188	TAM-Inflam	Parp11
Trim34a	1.48E-191	1.753615	0.302	0.087	3.62E-187	TAM-Inflam	Trim34a
H2-Q62	2.75E-191	1.21162	0.707	0.375	6.74E-187	TAM-Inflam	H2-Q6
Gbp31	2.25E-188	1.598652	0.372	0.126	5.52E-184	TAM-Inflam	Gbp3
Il2rg3	6.99E-184	1.678293	0.491	0.214	1.71E-179	TAM-Inflam	Il2rg
Ly864	3.06E-182	0.633035	0.998	0.911	7.49E-178	TAM-Inflam	Ly86
Nampt2	2.13E-181	1.423973	0.471	0.19	5.21E-177	TAM-Inflam	Nampt
H2-T223	2.89E-180	1.206972	0.645	0.338	7.07E-176	TAM-Inflam	H2-T22
Cd522	3.83E-175	0.837017	0.905	0.668	9.38E-171	TAM-Inflam	Cd52
Fam241a1	1.03E-174	1.821568	0.291	0.087	2.53E-170	TAM-Inflam	Fam241a
Gpr843	2.60E-172	1.683547	0.492	0.214	6.37E-168	TAM-Inflam	Gpr84
Sp1101	7.44E-172	1.336859	0.526	0.238	1.82E-167	TAM-Inflam	Sp110
Tspo3	7.52E-172	1.02686	0.748	0.442	1.84E-167	TAM-Inflam	Tspo
Gbp82	2.14E-165	1.693007	0.32	0.106	5.25E-161	TAM-Inflam	Gbp8
Ctsc3	3.48E-165	0.898547	0.916	0.701	8.53E-161	TAM-Inflam	Ctsc
C1qb4	2.14E-164	0.528783	0.996	0.887	5.24E-160	TAM-Inflam	C1qb
Zup11	1.99E-161	1.255689	0.575	0.275	4.87E-157	TAM-Inflam	Zup1
Lgals92	3.46E-161	0.915485	0.818	0.538	8.48E-157	TAM-Inflam	Lgals9
Grn4	1.36E-160	0.599292	0.981	0.851	3.33E-156	TAM-Inflam	Grn
Irgm2	1.14E-156	1.398191	0.436	0.178	2.80E-152	TAM-Inflam	Irgm2
Psme13	3.31E-156	0.807139	0.859	0.599	8.12E-152	TAM-Inflam	Psme1
Dtx3l1	8.07E-154	1.471285	0.395	0.154	1.98E-149	TAM-Inflam	Dtx3l
Gbp91	8.71E-153	1.533603	0.334	0.117	2.13E-148	TAM-Inflam	Gbp9
Cd300lf2	1.17E-149	1.51099	0.366	0.139	2.86E-145	TAM-Inflam	Cd300lf
Pik3ap11	2.71E-147	0.920861	0.797	0.517	6.63E-143	TAM-Inflam	Pik3ap1
Daxx1	4.84E-147	1.645457	0.313	0.109	1.18E-142	TAM-Inflam	Daxx
C32	7.31E-145	1.692947	0.361	0.141	1.79E-140	TAM-Inflam	C3
Psme22	6.77E-144	0.850073	0.779	0.514	1.66E-139	TAM-Inflam	Psme2
Vcam12	1.88E-142	1.347117	0.359	0.137	4.60E-138	TAM-Inflam	Vcam1
Icam12	8.15E-142	1.388781	0.615	0.336	1.99E-137	TAM-Inflam	Icam1
Sdc33	2.84E-141	1.179001	0.522	0.251	6.96E-137	TAM-Inflam	Sdc3
Ddx601	3.31E-137	1.636678	0.301	0.105	8.09E-133	TAM-Inflam	Ddx60
Epsti11	2.08E-134	0.876252	0.701	0.405	5.09E-130	TAM-Inflam	Epsti1
Casp4	9.84E-132	1.346749	0.326	0.121	2.41E-127	TAM-Inflam	Casp4
Trim14	3.76E-131	1.200012	0.473	0.221	9.22E-127	TAM-Inflam	Trim14
Axl3	1.77E-130	1.07187	0.526	0.26	4.33E-126	TAM-Inflam	Axl
Zc3hav12	9.41E-130	1.11897	0.49	0.233	2.30E-125	TAM-Inflam	Zc3hav1
Psmb92	3.63E-129	0.779187	0.762	0.476	8.89E-125	TAM-Inflam	Psmb9
Ctsz2	3.79E-128	0.537867	0.973	0.853	9.27E-124	TAM-Inflam	Ctsz
Il15ra	1.48E-126	1.373353	0.293	0.104	3.62E-122	TAM-Inflam	Il15ra
C1qc4	3.24E-126	0.477401	0.993	0.872	7.93E-122	TAM-Inflam	C1qc
H2-T24	1.45E-125	1.421129	0.322	0.122	3.56E-121	TAM-Inflam	H2-T24
Apobec31	3.41E-125	0.925406	0.695	0.422	8.34E-121	TAM-Inflam	Apobec3

Psmb103	1.75E-124	0.92184	0.643	0.376	4.28E-120	TAM-Inflam	Psmb10
Cxcl162	3.63E-124	1.234644	0.546	0.296	8.88E-120	TAM-Inflam	Cxcl16
Psap3	1.03E-123	0.489605	0.996	0.944	2.51E-119	TAM-Inflam	Psap
H2-DMa2	1.43E-122	0.706055	0.879	0.662	3.51E-118	TAM-Inflam	H2-DMa
Il10ra3	3.24E-122	0.717558	0.902	0.669	7.93E-118	TAM-Inflam	Il10ra
1600014C1	1.28E-118	1.116622	0.403	0.177	3.13E-114	TAM-Inflam	1600014C10Rik
Adar1	2.77E-118	1.158433	0.375	0.16	6.78E-114	TAM-Inflam	Adar
Tor1aip11	4.89E-118	0.951022	0.684	0.42	1.20E-113	TAM-Inflam	Tor1aip1
Ms4a4c1	6.81E-116	0.863686	0.295	0.107	1.67E-111	TAM-Inflam	Ms4a4c
Cd362	2.14E-115	1.078196	0.302	0.114	5.23E-111	TAM-Inflam	Cd36
Ccdc862	3.52E-115	1.246815	0.374	0.163	8.62E-111	TAM-Inflam	Ccdc86
H2-DMb2	7.26E-115	1.533181	0.26	0.092	1.78E-110	TAM-Inflam	H2-DMb2
Gm12185	9.80E-114	1.101351	0.383	0.165	2.40E-109	TAM-Inflam	Gm12185
H2-DMb13	9.90E-113	0.674189	0.799	0.528	2.43E-108	TAM-Inflam	H2-DMb1
Arid5a	3.92E-112	1.447996	0.252	0.088	9.61E-108	TAM-Inflam	Arid5a
4930599N2	6.91E-109	1.357021	0.27	0.099	1.69E-104	TAM-Inflam	4930599N23Rik
Ifi302	4.81E-108	0.61517	0.823	0.591	1.18E-103	TAM-Inflam	Ifi30
Rps203	1.06E-107	0.532348	0.969	0.879	2.61E-103	TAM-Inflam	Rps20
Ppfia44	1.22E-106	0.74275	0.754	0.489	2.98E-102	TAM-Inflam	Ppfia4
Trim252	9.63E-106	1.18571	0.369	0.165	2.36E-101	TAM-Inflam	Trim25
Etnk1	4.62E-103	0.917967	0.495	0.254	1.13E-98	TAM-Inflam	Etnk1
Tapbp2	2.22E-99	0.722004	0.739	0.497	5.44E-95	TAM-Inflam	Tapbp
Sppl2a1	4.58E-99	0.850011	0.639	0.381	1.12E-94	TAM-Inflam	Sppl2a
Aif14	1.44E-97	0.562358	0.899	0.699	3.54E-93	TAM-Inflam	Aif1
Spint14	2.31E-95	0.820548	0.57	0.321	5.66E-91	TAM-Inflam	Spint1
Lpcat24	6.24E-95	0.489492	0.966	0.795	1.53E-90	TAM-Inflam	Lpcat2
Plaat33	4.73E-93	0.929357	0.413	0.204	1.16E-88	TAM-Inflam	Plaat3
Trim12a	1.72E-92	0.899371	0.424	0.208	4.22E-88	TAM-Inflam	Trim12a
Slc31a22	1.86E-92	1.061623	0.431	0.222	4.56E-88	TAM-Inflam	Slc31a2
Tor1aip2	4.76E-92	0.891976	0.515	0.285	1.17E-87	TAM-Inflam	Tor1aip2
Tlr3	1.80E-91	1.203779	0.262	0.102	4.41E-87	TAM-Inflam	Tlr3
Tmem184b	5.47E-91	0.939661	0.423	0.21	1.34E-86	TAM-Inflam	Tmem184b
Bcl2a1a2	2.15E-90	1.091003	0.307	0.132	5.27E-86	TAM-Inflam	Bcl2a1a
Tap21	4.75E-90	0.801386	0.62	0.384	1.16E-85	TAM-Inflam	Tap2
Tnf	3.39E-89	1.787086	0.257	0.104	8.30E-85	TAM-Inflam	Tnf
Psmb83	2.45E-88	0.577654	0.823	0.596	6.00E-84	TAM-Inflam	Psmb8
Csf11	5.91E-88	0.650972	0.322	0.14	1.45E-83	TAM-Inflam	Csf1
Fmnl21	3.04E-86	0.97079	0.523	0.296	7.45E-82	TAM-Inflam	Fmnl2
Rasa43	1.50E-85	0.766817	0.578	0.341	3.67E-81	TAM-Inflam	Rasa4
Dop1b	6.59E-85	0.929614	0.4	0.198	1.61E-80	TAM-Inflam	Dop1b
Rpl353	2.25E-84	0.512107	0.926	0.772	5.50E-80	TAM-Inflam	Rpl35
Irf81	3.60E-84	0.656983	0.814	0.599	8.82E-80	TAM-Inflam	Irf8
Sat11	9.19E-84	0.546247	0.888	0.726	2.25E-79	TAM-Inflam	Sat1
Cd472	1.83E-83	0.697461	0.691	0.455	4.48E-79	TAM-Inflam	Cd47

Ccrl22	4.01E-83	1.437025	0.319	0.149	9.81E-79	TAM-Inflam	Ccrl2
Atp6v0a21	5.32E-83	0.794722	0.527	0.299	1.30E-78	TAM-Inflam	Atp6v0a2
C22	1.03E-82	1.531311	0.257	0.109	2.51E-78	TAM-Inflam	C2
Cd814	6.91E-82	0.450428	0.972	0.806	1.69E-77	TAM-Inflam	Cd81
Cd1801	1.31E-81	0.664008	0.742	0.511	3.22E-77	TAM-Inflam	Cd180
Ogfr2	7.68E-81	0.967303	0.383	0.195	1.88E-76	TAM-Inflam	Ogfr
Adora33	1.62E-80	0.758582	0.496	0.27	3.96E-76	TAM-Inflam	Adora3
Ccl42	6.91E-80	1.708922	0.361	0.186	1.69E-75	TAM-Inflam	Ccl4
Calhm62	6.76E-79	1.113282	0.355	0.175	1.66E-74	TAM-Inflam	Calhm6
Slc31a12	1.30E-78	0.89313	0.392	0.202	3.18E-74	TAM-Inflam	Slc31a1
Hck2	3.48E-78	0.613567	0.764	0.551	8.52E-74	TAM-Inflam	Hck
Gatm3	5.32E-78	0.781522	0.613	0.39	1.30E-73	TAM-Inflam	Gatm
Tapbpl1	4.91E-76	0.951914	0.357	0.175	1.20E-71	TAM-Inflam	Tapbpl
Itm2b4	2.13E-75	0.355839	0.993	0.955	5.22E-71	TAM-Inflam	Itm2b
Rps293	3.40E-75	0.448594	0.958	0.852	8.33E-71	TAM-Inflam	Rps29
Slc1a32	7.31E-75	0.607011	0.654	0.405	1.79E-70	TAM-Inflam	Slc1a3
Npc24	1.01E-74	0.386319	0.952	0.807	2.46E-70	TAM-Inflam	Npc2
Il1a2	1.63E-73	0.920909	0.479	0.27	3.98E-69	TAM-Inflam	Il1a
Usp25	2.08E-73	0.806746	0.454	0.252	5.08E-69	TAM-Inflam	Usp25
Tmem1401	6.03E-73	0.932789	0.344	0.169	1.48E-68	TAM-Inflam	Tmem140
Naaa1	7.99E-73	0.730364	0.38	0.192	1.96E-68	TAM-Inflam	Naaa
Nmi1	1.38E-72	0.962138	0.325	0.157	3.38E-68	TAM-Inflam	Nmi
AW112010	1.73E-72	0.446108	0.498	0.284	4.25E-68	TAM-Inflam	AW112010
Pdia33	2.13E-72	0.506861	0.89	0.714	5.22E-68	TAM-Inflam	Pdia3
Il18	4.31E-72	0.878135	0.392	0.204	1.06E-67	TAM-Inflam	Il18
Tmem1733	2.60E-71	0.549653	0.716	0.485	6.37E-67	TAM-Inflam	Tmem173
Uba7	2.17E-70	0.786099	0.409	0.219	5.31E-66	TAM-Inflam	Uba7
Rpl393	2.21E-70	0.434846	0.946	0.819	5.41E-66	TAM-Inflam	Rpl39
Pycard3	2.83E-70	0.545683	0.773	0.566	6.94E-66	TAM-Inflam	Pycard
Tmbim63	9.64E-70	0.409836	0.919	0.777	2.36E-65	TAM-Inflam	Tmbim6
Lag34	1.42E-69	0.481284	0.789	0.535	3.48E-65	TAM-Inflam	Lag3
Ctsb3	3.50E-69	0.328189	0.986	0.903	8.58E-65	TAM-Inflam	Ctsb
Sp1401	1.38E-66	0.875741	0.348	0.179	3.39E-62	TAM-Inflam	Sp140
Zfas12	1.86E-66	0.964173	0.354	0.188	4.55E-62	TAM-Inflam	Zfas1
Trim12c	1.92E-66	0.980759	0.291	0.139	4.70E-62	TAM-Inflam	Trim12c
Cebpb2	5.44E-66	0.327584	0.764	0.534	1.33E-61	TAM-Inflam	Cebpb
Arid5b2	4.80E-65	0.894427	0.523	0.327	1.18E-60	TAM-Inflam	Arid5b
Icosl	5.85E-65	0.986753	0.326	0.165	1.43E-60	TAM-Inflam	Icosl
Denr1	1.08E-63	0.803604	0.403	0.224	2.65E-59	TAM-Inflam	Denr
Slc12a93	1.95E-63	0.632305	0.547	0.334	4.78E-59	TAM-Inflam	Slc12a9
Slc15a32	2.18E-63	0.73128	0.419	0.234	5.35E-59	TAM-Inflam	Slc15a3
Hspe13	1.66E-62	0.595732	0.629	0.423	4.06E-58	TAM-Inflam	Hspe1
Tmem106a	3.16E-62	0.690897	0.409	0.225	7.73E-58	TAM-Inflam	Tmem106a
Cd861	3.52E-62	0.516199	0.788	0.583	8.62E-58	TAM-Inflam	Cd86

Ccnd12	1.55E-61	0.750911	0.385	0.211	3.80E-57	TAM-Inflam	Ccnd1
Gm46224	3.16E-61	0.837972	0.317	0.159	7.74E-57	TAM-Inflam	Gm46224
Rpl413	1.31E-60	0.366241	0.967	0.902	3.21E-56	TAM-Inflam	Rpl41
Lgmn3	1.81E-60	0.342814	0.992	0.899	4.43E-56	TAM-Inflam	Lgmn
Lacc11	2.58E-60	0.634506	0.476	0.281	6.31E-56	TAM-Inflam	Lacc1
Minpp1	3.06E-60	0.830371	0.312	0.158	7.48E-56	TAM-Inflam	Minpp1
Rpl323	5.23E-60	0.347079	0.964	0.864	1.28E-55	TAM-Inflam	Rpl32
Tlr22	5.62E-60	0.856103	0.453	0.272	1.38E-55	TAM-Inflam	Tlr2
Rab202	8.37E-60	0.809593	0.349	0.185	2.05E-55	TAM-Inflam	Rab20
Lrch31	1.10E-59	0.669837	0.436	0.25	2.69E-55	TAM-Inflam	Lrch3
Mpeg12	1.64E-59	0.416237	0.89	0.698	4.01E-55	TAM-Inflam	Mpeg1
M6pr3	2.61E-59	0.584792	0.664	0.46	6.38E-55	TAM-Inflam	M6pr
Rps113	4.42E-59	0.340336	0.974	0.894	1.08E-54	TAM-Inflam	Rps11
Cmtr1	9.41E-59	0.856028	0.294	0.146	2.31E-54	TAM-Inflam	Cmtr1
Cryba44	1.20E-58	0.647839	0.457	0.267	2.95E-54	TAM-Inflam	Cryba4
Rnf1141	1.45E-58	0.681008	0.408	0.23	3.55E-54	TAM-Inflam	Rnf114
Unc93b13	1.63E-58	0.3452	0.973	0.861	4.00E-54	TAM-Inflam	Unc93b1
Rnf19b1	2.24E-58	0.56234	0.5	0.31	5.50E-54	TAM-Inflam	Rnf19b
Morc3	3.12E-58	0.637607	0.539	0.34	7.63E-54	TAM-Inflam	Morc3
Birc31	8.79E-58	0.772222	0.403	0.231	2.15E-53	TAM-Inflam	Birc3
Bbx	3.22E-57	0.886519	0.346	0.186	7.88E-53	TAM-Inflam	Bbx
Pttg12	3.91E-57	0.876226	0.366	0.205	9.57E-53	TAM-Inflam	Pttg1
Tlr121	1.11E-56	0.808478	0.291	0.145	2.71E-52	TAM-Inflam	Tlr12
Cyba3	1.88E-56	0.357037	0.958	0.859	4.60E-52	TAM-Inflam	Cyba
Sap301	5.80E-56	0.976588	0.258	0.125	1.42E-51	TAM-Inflam	Sap30
Hebp12	8.48E-56	0.661804	0.432	0.253	2.08E-51	TAM-Inflam	Hebp1
Chmp4b3	2.04E-55	0.504899	0.655	0.461	5.00E-51	TAM-Inflam	Chmp4b
Selenow4	2.83E-55	0.534357	0.652	0.457	6.93E-51	TAM-Inflam	Selenow
Ranbp2	5.97E-55	0.737365	0.377	0.214	1.46E-50	TAM-Inflam	Ranbp2
Keap11	9.16E-55	0.959358	0.295	0.153	2.24E-50	TAM-Inflam	Keap1
Tnfsf13b	1.01E-54	0.775483	0.285	0.143	2.46E-50	TAM-Inflam	Tnfsf13b
Tiam1	1.13E-54	0.711452	0.29	0.146	2.77E-50	TAM-Inflam	Tiam1
Rad51b2	1.21E-54	0.408143	0.666	0.444	2.97E-50	TAM-Inflam	Rad51b
Rps263	1.62E-53	0.363337	0.93	0.82	3.97E-49	TAM-Inflam	Rps26
P4ha12	3.01E-53	0.603894	0.564	0.369	7.37E-49	TAM-Inflam	P4ha1
Bcl2a1b2	4.73E-53	0.515845	0.637	0.436	1.16E-48	TAM-Inflam	Bcl2a1b
Mmp142	6.31E-53	0.667602	0.348	0.19	1.55E-48	TAM-Inflam	Mmp14
Themis22	9.28E-53	0.778436	0.379	0.218	2.27E-48	TAM-Inflam	Themis2
Cd722	1.36E-52	0.889506	0.27	0.135	3.33E-48	TAM-Inflam	Cd72
Ssr42	1.43E-52	0.409692	0.83	0.641	3.51E-48	TAM-Inflam	Ssr4
Cacnb21	4.07E-52	0.396399	0.546	0.337	9.96E-48	TAM-Inflam	Cacnb2
Shisa51	4.33E-52	0.495499	0.658	0.455	1.06E-47	TAM-Inflam	Shisa5
Pde4dip	4.70E-52	0.666942	0.366	0.206	1.15E-47	TAM-Inflam	Pde4dip
Rpl27a3	5.80E-52	0.319112	0.973	0.888	1.42E-47	TAM-Inflam	Rpl27a

Capza21	1.73E-51	0.418039	0.844	0.671	4.23E-47	TAM-Inflam	Capza2
Uba523	4.86E-51	0.491027	0.651	0.456	1.19E-46	TAM-Inflam	Uba52
Rufy3	6.17E-51	0.665779	0.302	0.158	1.51E-46	TAM-Inflam	Rufy3
Cybb2	6.20E-51	0.370595	0.41	0.232	1.52E-46	TAM-Inflam	Cybb
Itgax2	1.67E-49	0.848485	0.308	0.168	4.09E-45	TAM-Inflam	Itgax
Rplp14	4.86E-49	0.280686	0.979	0.919	1.19E-44	TAM-Inflam	Rplp1
Fcgr2b3	4.93E-49	0.386037	0.846	0.649	1.21E-44	TAM-Inflam	Fcgr2b
Tgs12	5.93E-49	0.604866	0.451	0.277	1.45E-44	TAM-Inflam	Tgs1
Pim12	5.52E-48	0.865825	0.273	0.144	1.35E-43	TAM-Inflam	Pim1
Sdf2l13	1.56E-47	0.559075	0.58	0.396	3.81E-43	TAM-Inflam	Sdf2l1
Sh3bp2	1.59E-47	0.64605	0.369	0.214	3.90E-43	TAM-Inflam	Sh3bp2
Rps243	5.82E-47	0.271831	0.979	0.928	1.43E-42	TAM-Inflam	Rps24
Svbp2	5.91E-47	0.561239	0.418	0.255	1.45E-42	TAM-Inflam	Svbp
Cited23	5.96E-47	0.794239	0.453	0.291	1.46E-42	TAM-Inflam	Cited2
Tmem2431	8.62E-47	0.468046	0.561	0.377	2.11E-42	TAM-Inflam	Tmem243
Cd832	8.70E-47	0.538576	0.669	0.477	2.13E-42	TAM-Inflam	Cd83
Rpl373	1.08E-46	0.335828	0.943	0.849	2.64E-42	TAM-Inflam	Rpl37
Rps283	1.13E-46	0.340686	0.894	0.754	2.77E-42	TAM-Inflam	Rps28
Slc11a13	1.30E-46	0.447775	0.695	0.493	3.19E-42	TAM-Inflam	Slc11a1
Camk2d1	3.96E-46	0.386671	0.732	0.529	9.70E-42	TAM-Inflam	Camk2d
Tmpo1	4.21E-46	0.618092	0.303	0.164	1.03E-41	TAM-Inflam	Tmpo
Rps15a3	1.96E-45	0.2939	0.951	0.854	4.80E-41	TAM-Inflam	Rps15a
Ankle2	5.01E-45	0.61698	0.352	0.202	1.23E-40	TAM-Inflam	Ankle2
Rps83	1.10E-44	0.268222	0.971	0.899	2.68E-40	TAM-Inflam	Rps8
Stard32	1.16E-44	0.523271	0.485	0.309	2.85E-40	TAM-Inflam	Stard3
Dcp2	1.61E-44	0.683886	0.258	0.133	3.93E-40	TAM-Inflam	Dcp2
Tasor2	1.76E-44	0.66237	0.293	0.157	4.30E-40	TAM-Inflam	Tasor2
Mgat13	1.78E-44	0.61398	0.437	0.275	4.36E-40	TAM-Inflam	Mgat1
Ak22	1.99E-44	0.62406	0.428	0.27	4.86E-40	TAM-Inflam	Ak2
Tmem2191	4.59E-44	0.519956	0.502	0.327	1.12E-39	TAM-Inflam	Tmem219
Abhd16a1	9.48E-44	0.580482	0.383	0.23	2.32E-39	TAM-Inflam	Abhd16a
Clec5a1	1.59E-43	0.68178	0.34	0.196	3.89E-39	TAM-Inflam	Clec5a
Batf31	6.28E-43	0.643708	0.337	0.194	1.54E-38	TAM-Inflam	Batf3
Rpl35a3	1.14E-42	0.283623	0.96	0.883	2.80E-38	TAM-Inflam	Rpl35a
Ly91	2.13E-42	0.6772	0.339	0.198	5.22E-38	TAM-Inflam	Ly9
Rps123	4.54E-42	0.281774	0.963	0.866	1.11E-37	TAM-Inflam	Rps12
Rps214	7.24E-42	0.303117	0.94	0.831	1.77E-37	TAM-Inflam	Rps21
Vps54	1.03E-41	0.507266	0.343	0.198	2.53E-37	TAM-Inflam	Vps54
Cnp1	1.55E-41	0.684808	0.302	0.172	3.79E-37	TAM-Inflam	Cnp
Il21r3	1.94E-41	0.403355	0.558	0.373	4.75E-37	TAM-Inflam	Il21r
Rpl37a3	3.65E-41	0.299487	0.955	0.852	8.94E-37	TAM-Inflam	Rpl37a
Larp11	5.81E-41	0.500697	0.477	0.309	1.42E-36	TAM-Inflam	Larp1
Laptm4a4	1.30E-40	0.329305	0.889	0.732	3.18E-36	TAM-Inflam	Laptm4a
Clic42	1.83E-40	0.638193	0.349	0.21	4.48E-36	TAM-Inflam	Clic4

Fcer1g4	1.25E-39	0.265053	0.978	0.903	3.06E-35	TAM-Inflam	Fcer1g
Lst12	1.26E-39	0.280741	0.728	0.533	3.09E-35	TAM-Inflam	Lst1
Akt3	5.40E-39	0.559174	0.316	0.182	1.32E-34	TAM-Inflam	Akt3
Gm433052	7.17E-39	0.578807	0.577	0.413	1.75E-34	TAM-Inflam	Gm43305
Tmem1281	8.48E-39	0.528469	0.435	0.281	2.08E-34	TAM-Inflam	Tmem128
Arl5c2	1.33E-38	0.740282	0.252	0.137	3.26E-34	TAM-Inflam	Arl5c
Gnl21	1.35E-38	0.653656	0.303	0.174	3.30E-34	TAM-Inflam	Gnl2
Fam167b	2.16E-38	0.577627	0.251	0.134	5.28E-34	TAM-Inflam	Fam167b
Efhd22	2.47E-38	0.377794	0.777	0.615	6.05E-34	TAM-Inflam	Efhd2
Cpq2	2.48E-38	0.395894	0.487	0.32	6.08E-34	TAM-Inflam	Cpq
Tbc1d1	2.75E-38	0.608077	0.288	0.163	6.74E-34	TAM-Inflam	Tbc1d1
Limd22	9.63E-38	0.323939	0.772	0.594	2.36E-33	TAM-Inflam	Limd2
Fnbp4	1.00E-37	0.498862	0.303	0.173	2.46E-33	TAM-Inflam	Fnbp4
Rpl193	1.17E-37	0.268252	0.964	0.889	2.87E-33	TAM-Inflam	Rpl19
Etv3	1.58E-37	0.632491	0.271	0.15	3.87E-33	TAM-Inflam	Etv3
Ascc31	1.62E-37	0.541058	0.369	0.224	3.98E-33	TAM-Inflam	Ascc3
Psma53	4.02E-37	0.491509	0.457	0.306	9.84E-33	TAM-Inflam	Psma5
Rhoh3	5.37E-37	0.301624	0.602	0.411	1.31E-32	TAM-Inflam	Rhoh
9930111J2	6.14E-37	0.616543	0.328	0.197	1.50E-32	TAM-Inflam	9930111J21Rik2
Nono1	7.20E-37	0.533426	0.436	0.287	1.76E-32	TAM-Inflam	Nono
Relb	8.37E-37	0.525799	0.29	0.166	2.05E-32	TAM-Inflam	Relb
Nfe2l21	9.19E-37	0.463466	0.657	0.489	2.25E-32	TAM-Inflam	Nfe2l2
Rpl10a3	1.09E-36	0.301079	0.922	0.814	2.66E-32	TAM-Inflam	Rpl10a
Pld43	1.42E-36	0.307122	0.912	0.75	3.47E-32	TAM-Inflam	Pld4
Atp13a1	2.29E-36	0.539735	0.281	0.159	5.60E-32	TAM-Inflam	Atp13a1
Ttc39b	2.34E-36	0.601853	0.348	0.209	5.73E-32	TAM-Inflam	Ttc39b
Etv61	2.90E-36	0.31349	0.769	0.581	7.11E-32	TAM-Inflam	Etv6
Acer31	3.56E-36	0.350661	0.642	0.463	8.72E-32	TAM-Inflam	Acer3
Npnt1	5.02E-36	0.465561	0.274	0.152	1.23E-31	TAM-Inflam	Npnt
Rpl273	6.97E-36	0.273716	0.915	0.774	1.71E-31	TAM-Inflam	Rpl27
Creb32	8.21E-36	0.529532	0.396	0.25	2.01E-31	TAM-Inflam	Creb3
Rer12	9.61E-36	0.420728	0.576	0.411	2.35E-31	TAM-Inflam	Rer1
Acsl4	1.43E-35	0.425007	0.34	0.204	3.49E-31	TAM-Inflam	Acsl4
Calr2	1.64E-35	0.412698	0.664	0.489	4.02E-31	TAM-Inflam	Calr
Sema4d1	1.71E-35	0.384369	0.619	0.437	4.18E-31	TAM-Inflam	Sema4d
Tent5a2	1.87E-35	0.524714	0.393	0.251	4.59E-31	TAM-Inflam	Tent5a
Nupr11	2.18E-35	0.580327	0.305	0.182	5.34E-31	TAM-Inflam	Nupr1
St6galnac4	4.29E-35	0.387374	0.461	0.301	1.05E-30	TAM-Inflam	St6galnac4
Akr1b33	4.71E-35	0.472233	0.461	0.314	1.15E-30	TAM-Inflam	Akr1b3
Usp212	6.47E-35	0.603415	0.323	0.195	1.59E-30	TAM-Inflam	Usp21
Hspa82	1.08E-34	0.302756	0.867	0.739	2.63E-30	TAM-Inflam	Hspa8
Slc2a11	1.39E-34	0.543138	0.283	0.164	3.41E-30	TAM-Inflam	Slc2a1
Rrbp12	1.69E-34	0.283024	0.896	0.77	4.14E-30	TAM-Inflam	Rrbp1
Rgmb3	1.90E-34	0.425532	0.395	0.248	4.66E-30	TAM-Inflam	Rgmb

Lamp21	3.07E-34	0.329561	0.82	0.65	7.51E-30	TAM-Inflam	Lamp2
Creld21	4.23E-34	0.658293	0.291	0.173	1.04E-29	TAM-Inflam	Creld2
Hsp90b12	5.10E-34	0.333355	0.872	0.738	1.25E-29	TAM-Inflam	Hsp90b1
Csf2rb21	5.70E-34	0.695249	0.258	0.147	1.40E-29	TAM-Inflam	Csf2rb2
Hspa51	7.04E-34	0.442573	0.792	0.644	1.72E-29	TAM-Inflam	Hspa5
Hk31	9.16E-34	0.484765	0.404	0.262	2.24E-29	TAM-Inflam	Hk3
Nlrp1b1	1.31E-33	0.312377	0.401	0.252	3.21E-29	TAM-Inflam	Nlrp1b
Clcn72	1.33E-33	0.526027	0.264	0.15	3.27E-29	TAM-Inflam	Clcn7
Gng103	1.96E-33	0.298124	0.808	0.628	4.80E-29	TAM-Inflam	Gng10
Rpl363	2.29E-33	0.253832	0.916	0.791	5.60E-29	TAM-Inflam	Rpl36
Rpl383	2.94E-33	0.280497	0.838	0.699	7.20E-29	TAM-Inflam	Rpl38
Lcp21	3.06E-33	0.337531	0.623	0.45	7.49E-29	TAM-Inflam	Lcp2
Grina	3.93E-33	0.276993	0.738	0.563	9.63E-29	TAM-Inflam	Grina
Idnk1	4.13E-33	0.504736	0.348	0.218	1.01E-28	TAM-Inflam	Idnk
Tmod31	5.90E-33	0.493071	0.388	0.249	1.44E-28	TAM-Inflam	Tmod3
Rab43	7.05E-33	0.514989	0.462	0.312	1.73E-28	TAM-Inflam	Rab43
Nfkbib2	1.45E-32	0.631637	0.297	0.18	3.55E-28	TAM-Inflam	Nfkbib
mt-Atp81	2.49E-32	0.333026	0.69	0.534	6.09E-28	TAM-Inflam	mt-Atp8
Mif3	3.18E-32	0.412024	0.585	0.433	7.79E-28	TAM-Inflam	Mif
Glrx4	4.61E-32	0.537504	0.261	0.151	1.13E-27	TAM-Inflam	Glrx
Slc35b1	5.74E-32	0.504083	0.329	0.204	1.41E-27	TAM-Inflam	Slc35b1
Rpl36a3	7.28E-32	0.300421	0.804	0.648	1.78E-27	TAM-Inflam	Rpl36a
B4galt42	8.29E-32	0.449759	0.326	0.199	2.03E-27	TAM-Inflam	B4galt4
Ube2e11	8.60E-32	0.4129	0.393	0.258	2.11E-27	TAM-Inflam	Ube2e1
Crybb13	1.15E-31	0.332542	0.682	0.507	2.82E-27	TAM-Inflam	Crybb1
Cops92	2.54E-31	0.31065	0.521	0.363	6.23E-27	TAM-Inflam	Cops9
Gpr1081	2.93E-31	0.459456	0.352	0.222	7.18E-27	TAM-Inflam	Gpr108
Ddx241	3.94E-31	0.400647	0.463	0.319	9.66E-27	TAM-Inflam	Ddx24
Rpl123	4.16E-31	0.285033	0.85	0.694	1.02E-26	TAM-Inflam	Rpl12
Slirp2	5.25E-31	0.584261	0.272	0.162	1.29E-26	TAM-Inflam	Slirp
Smim31	6.24E-31	0.489857	0.254	0.146	1.53E-26	TAM-Inflam	Smim3
Rpl223	8.01E-31	0.255894	0.883	0.744	1.96E-26	TAM-Inflam	Rpl22
Nme24	1.18E-30	0.27026	0.765	0.598	2.90E-26	TAM-Inflam	Nme2
Fbrsl12	1.93E-30	0.266383	0.498	0.338	4.73E-26	TAM-Inflam	Fbrsl1
Peli11	2.22E-30	0.284537	0.552	0.39	5.44E-26	TAM-Inflam	Peli1
Gsdmd1	2.45E-30	0.405588	0.436	0.295	6.00E-26	TAM-Inflam	Gsdmd
2410006H1	2.93E-30	0.294044	0.653	0.485	7.17E-26	TAM-Inflam	2410006H16Rik
Rnaset2a3	3.66E-30	0.31174	0.488	0.335	8.96E-26	TAM-Inflam	Rnaset2a
Pnp3	3.85E-30	0.342152	0.638	0.477	9.44E-26	TAM-Inflam	Pnp
Pdia62	5.33E-30	0.353559	0.661	0.495	1.30E-25	TAM-Inflam	Pdia6
Slc3a24	6.23E-30	0.307489	0.715	0.539	1.53E-25	TAM-Inflam	Slc3a2
Ctsh4	6.51E-30	0.266393	0.918	0.785	1.59E-25	TAM-Inflam	Ctsh
Gas51	6.75E-30	0.291311	0.724	0.555	1.65E-25	TAM-Inflam	Gas5
Gtpbp22	1.38E-29	0.413393	0.405	0.268	3.39E-25	TAM-Inflam	Gtpbp2

H2-M33	1.51E-29	0.316033	0.574	0.413	3.69E-25	TAM-Inflam	H2-M3
Pomp3	1.51E-29	0.343007	0.698	0.548	3.70E-25	TAM-Inflam	Pomp
Gns2	2.55E-29	0.277867	0.861	0.691	6.25E-25	TAM-Inflam	Gns
Trpm2	4.04E-29	0.45169	0.3	0.184	9.90E-25	TAM-Inflam	Trpm2
Pold44	4.19E-29	0.370527	0.521	0.369	1.03E-24	TAM-Inflam	Pold4
Eef1b23	4.88E-29	0.275095	0.82	0.677	1.19E-24	TAM-Inflam	Eef1b2
Dbnl1	5.27E-29	0.394399	0.487	0.34	1.29E-24	TAM-Inflam	Dbnl
Lair13	7.81E-29	0.257508	0.888	0.733	1.91E-24	TAM-Inflam	Lair1
Srsf72	9.58E-29	0.399756	0.587	0.43	2.35E-24	TAM-Inflam	Srsf7
Glmp4	1.01E-28	0.31509	0.635	0.465	2.47E-24	TAM-Inflam	Glmp
Map113	1.90E-28	0.298854	0.486	0.334	4.65E-24	TAM-Inflam	Map11
Gm118083	2.22E-28	0.36374	0.445	0.304	5.43E-24	TAM-Inflam	Gm11808
Rpl313	4.77E-28	0.297578	0.684	0.525	1.17E-23	TAM-Inflam	Rpl31
Dnajb111	5.31E-28	0.343015	0.422	0.284	1.30E-23	TAM-Inflam	Dnajb11
Ssr22	6.98E-28	0.378482	0.473	0.329	1.71E-23	TAM-Inflam	Ssr2
Evi2a2	7.68E-28	0.442715	0.426	0.29	1.88E-23	TAM-Inflam	Evi2a
Il15	7.87E-28	0.477737	0.3	0.187	1.93E-23	TAM-Inflam	Il15
Scpep13	8.44E-28	0.357109	0.521	0.373	2.07E-23	TAM-Inflam	Scpep1
Rtraf3	8.83E-28	0.335087	0.565	0.417	2.16E-23	TAM-Inflam	Rtraf
Gars1	1.13E-27	0.457615	0.33	0.212	2.77E-23	TAM-Inflam	Gars
Gsap1	1.20E-27	0.384741	0.425	0.289	2.93E-23	TAM-Inflam	Gsap
Scarb22	1.31E-27	0.28183	0.667	0.497	3.22E-23	TAM-Inflam	Scarb2
1110038B1	1.76E-27	0.468287	0.307	0.196	4.32E-23	TAM-Inflam	1110038B12Rik
Nudt92	1.79E-27	0.385351	0.393	0.262	4.38E-23	TAM-Inflam	Nudt9
Csf2ra2	2.13E-27	0.324467	0.571	0.416	5.22E-23	TAM-Inflam	Csf2ra
Pi4k2a	4.39E-27	0.539203	0.25	0.149	1.08E-22	TAM-Inflam	Pi4k2a
Manf1	6.04E-27	0.433276	0.55	0.401	1.48E-22	TAM-Inflam	Manf
Ppib2	6.17E-27	0.26721	0.824	0.67	1.51E-22	TAM-Inflam	Ppib
B3gnt2	8.68E-27	0.486522	0.333	0.216	2.13E-22	TAM-Inflam	B3gnt2
Nsmaf	1.17E-26	0.54165	0.259	0.157	2.86E-22	TAM-Inflam	Nsmaf
Max1	2.61E-26	0.355632	0.386	0.261	6.40E-22	TAM-Inflam	Max
Atp6v1d2	4.65E-26	0.351722	0.407	0.28	1.14E-21	TAM-Inflam	Atp6v1d
Tox42	5.97E-26	0.388102	0.396	0.269	1.46E-21	TAM-Inflam	Tox4
Pgap21	6.27E-26	0.478811	0.264	0.162	1.54E-21	TAM-Inflam	Pgap2
Pdia44	6.55E-26	0.315692	0.462	0.32	1.60E-21	TAM-Inflam	Pdia4
Cisd2	7.31E-26	0.348007	0.379	0.254	1.79E-21	TAM-Inflam	Cisd2
Hvcn14	1.02E-25	0.304591	0.443	0.305	2.49E-21	TAM-Inflam	Hvcn1
Tnfrsf1a2	1.03E-25	0.292465	0.604	0.448	2.53E-21	TAM-Inflam	Tnfrsf1a
Ddost3	1.72E-25	0.277396	0.651	0.496	4.20E-21	TAM-Inflam	Ddost
Cyb561a33	2.29E-25	0.285203	0.435	0.296	5.61E-21	TAM-Inflam	Cyb561a3
Ldha2	2.94E-25	0.332113	0.589	0.442	7.19E-21	TAM-Inflam	Ldha
Tomm70a1	3.28E-25	0.431145	0.274	0.171	8.04E-21	TAM-Inflam	Tomm70a
Arf41	3.31E-25	0.314559	0.597	0.449	8.11E-21	TAM-Inflam	Arf4
Tbc1d8	3.43E-25	0.258468	0.365	0.24	8.39E-21	TAM-Inflam	Tbc1d8

Plekho22	3.77E-25	0.372861	0.317	0.205	9.23E-21	TAM-Inflam	Plekho2
Spcs13	6.38E-25	0.28605	0.654	0.505	1.56E-20	TAM-Inflam	Spcs1
Tspan313	8.27E-25	0.347906	0.416	0.289	2.03E-20	TAM-Inflam	Tspan31
Lat24	8.94E-25	0.301271	0.447	0.312	2.19E-20	TAM-Inflam	Lat2
Ubr4	1.65E-24	0.391168	0.317	0.206	4.04E-20	TAM-Inflam	Ubr4
Ripk2	3.47E-24	0.418803	0.282	0.177	8.51E-20	TAM-Inflam	Ripk2
Atp5mpl4	3.60E-24	0.264971	0.559	0.417	8.81E-20	TAM-Inflam	Atp5mpl
Rpl23a2	6.31E-24	0.254469	0.664	0.503	1.54E-19	TAM-Inflam	Rpl23a
Mbd22	7.09E-24	0.324076	0.374	0.253	1.74E-19	TAM-Inflam	Mbd2
Rpl22l13	7.34E-24	0.268923	0.639	0.496	1.80E-19	TAM-Inflam	Rpl22l1
Mrpl523	1.02E-23	0.263287	0.625	0.47	2.49E-19	TAM-Inflam	Mrpl52
Slc29a33	1.10E-23	0.264111	0.617	0.458	2.70E-19	TAM-Inflam	Slc29a3
Tmed52	1.31E-23	0.312345	0.514	0.377	3.20E-19	TAM-Inflam	Tmed5
Milr13	1.57E-23	0.542625	0.258	0.161	3.84E-19	TAM-Inflam	Milr1
Hacd21	2.14E-23	0.346695	0.358	0.243	5.23E-19	TAM-Inflam	Hacd2
Ncf11	2.75E-23	0.284534	0.688	0.542	6.74E-19	TAM-Inflam	Ncf1
AB124611З	4.79E-23	0.60518	0.258	0.166	1.17E-18	TAM-Inflam	AB124611
Cept11	6.33E-23	0.260676	0.403	0.276	1.55E-18	TAM-Inflam	Cept1
Rnh12	9.63E-23	0.311918	0.48	0.348	2.36E-18	TAM-Inflam	Rnh1
Psma32	1.39E-22	0.260114	0.562	0.419	3.41E-18	TAM-Inflam	Psma3
Cyp4v32	1.41E-22	0.297791	0.357	0.241	3.46E-18	TAM-Inflam	Cyp4v3
Rab11a1	1.54E-22	0.268659	0.568	0.427	3.77E-18	TAM-Inflam	Rab11a
Lrrk11	1.58E-22	0.275163	0.524	0.383	3.86E-18	TAM-Inflam	Lrrk1
Trim26	2.15E-22	0.308759	0.328	0.216	5.27E-18	TAM-Inflam	Trim26
Hmox22	5.36E-22	0.33472	0.555	0.418	1.31E-17	TAM-Inflam	Hmox2
Cldnd12	6.71E-22	0.308892	0.332	0.22	1.64E-17	TAM-Inflam	Cldnd1
Tank	8.11E-22	0.407728	0.314	0.208	1.99E-17	TAM-Inflam	Tank
Dnaja21	1.12E-21	0.350179	0.421	0.303	2.74E-17	TAM-Inflam	Dnaja2
P2rx43	1.45E-21	0.32373	0.441	0.317	3.55E-17	TAM-Inflam	P2rx4
Tmem333	1.50E-21	0.283536	0.395	0.275	3.68E-17	TAM-Inflam	Tmem33
Ccnl12	1.88E-21	0.310766	0.47	0.344	4.60E-17	TAM-Inflam	Ccnl1
Csf2rb1	1.93E-21	0.292749	0.372	0.256	4.72E-17	TAM-Inflam	Csf2rb
Tagap2	2.35E-21	0.315695	0.364	0.25	5.76E-17	TAM-Inflam	Tagap
Casp12	3.06E-21	0.508831	0.275	0.18	7.49E-17	TAM-Inflam	Casp1
Psmd7	3.46E-21	0.337667	0.35	0.24	8.48E-17	TAM-Inflam	Psmd7
Nfkbia3	3.56E-21	0.332489	0.616	0.487	8.71E-17	TAM-Inflam	Nfkbia
Erap1	3.72E-21	0.375911	0.272	0.176	9.12E-17	TAM-Inflam	Erap1
9530068E0	4.00E-21	0.288385	0.384	0.267	9.79E-17	TAM-Inflam	9530068E07Rik
Sharpin1	4.15E-21	0.303058	0.316	0.211	1.02E-16	TAM-Inflam	Sharpin
Selenot	4.15E-21	0.260801	0.416	0.294	1.02E-16	TAM-Inflam	Selenot
Mif4gd1	6.22E-21	0.326613	0.285	0.187	1.52E-16	TAM-Inflam	Mif4gd
Tbc1d10a1	6.53E-21	0.339653	0.304	0.203	1.60E-16	TAM-Inflam	Tbc1d10a
Ndufs32	7.18E-21	0.304271	0.439	0.318	1.76E-16	TAM-Inflam	Ndufs3
Snhg121	8.47E-21	0.450064	0.263	0.17	2.08E-16	TAM-Inflam	Snhg12

Pdlim43	1.06E-20	0.262461	0.379	0.261	2.60E-16	TAM-Inflam	Pdlim4
Chrac11	1.74E-20	0.339821	0.346	0.238	4.25E-16	TAM-Inflam	Chrac1
Fndc3a1	1.03E-19	0.277261	0.419	0.301	2.53E-15	TAM-Inflam	Fndc3a
Gng123	1.22E-19	0.272657	0.413	0.297	2.99E-15	TAM-Inflam	Gng12
Rnase61	1.49E-19	0.368964	0.279	0.184	3.66E-15	TAM-Inflam	Rnase6
Bola22	2.23E-19	0.255515	0.334	0.227	5.46E-15	TAM-Inflam	Bola2
Ptpn62	2.98E-19	0.26362	0.649	0.512	7.31E-15	TAM-Inflam	Ptpn6
Tmem1311	3.60E-19	0.252433	0.424	0.306	8.82E-15	TAM-Inflam	Tmem131
Tnfaip32	3.85E-19	0.443504	0.299	0.203	9.43E-15	TAM-Inflam	Tnfaip3
Resf11	4.05E-19	0.292364	0.363	0.255	9.92E-15	TAM-Inflam	Resf1
Plk31	4.58E-19	0.425467	0.253	0.165	1.12E-14	TAM-Inflam	Plk3
Psmb23	4.88E-19	0.264605	0.491	0.368	1.20E-14	TAM-Inflam	Psmb2
Cep853	8.04E-19	0.37798	0.396	0.29	1.97E-14	TAM-Inflam	Cep85
Plekhf21	8.34E-19	0.392548	0.272	0.181	2.04E-14	TAM-Inflam	Plekhf2
Xrn1	8.74E-19	0.307288	0.278	0.184	2.14E-14	TAM-Inflam	Xrn1
Bloc1s13	9.60E-19	0.25079	0.329	0.225	2.35E-14	TAM-Inflam	Bloc1s1
Leprotl11	2.13E-18	0.267023	0.386	0.275	5.22E-14	TAM-Inflam	Leprotl1
Derl2	2.22E-18	0.288352	0.338	0.236	5.44E-14	TAM-Inflam	Derl2
Rubcnl2	6.30E-18	0.262324	0.345	0.239	1.54E-13	TAM-Inflam	Rubcnl
Cacybp2	1.73E-17	0.302437	0.286	0.196	4.23E-13	TAM-Inflam	Cacybp
Mvp3	2.67E-17	0.406513	0.274	0.189	6.54E-13	TAM-Inflam	Mvp
Ube2v14	4.77E-17	0.26006	0.388	0.284	1.17E-12	TAM-Inflam	Ube2v1
Utp31	1.69E-16	0.327753	0.279	0.192	4.14E-12	TAM-Inflam	Utp3
Igbp12	1.71E-16	0.279768	0.318	0.224	4.19E-12	TAM-Inflam	Igbp1
Snx201	4.94E-16	0.254597	0.437	0.325	1.21E-11	TAM-Inflam	Snx20
Elovl11	5.60E-16	0.287721	0.337	0.243	1.37E-11	TAM-Inflam	Elovl1
Ist1	9.05E-16	0.283902	0.266	0.182	2.22E-11	TAM-Inflam	Ist1
Gaa3	1.33E-15	0.257317	0.339	0.242	3.25E-11	TAM-Inflam	Gaa
Pfkl1	3.35E-15	0.365423	0.253	0.175	8.22E-11	TAM-Inflam	Pfkl
Tm9sf1	1.13E-14	0.272339	0.276	0.193	2.77E-10	TAM-Inflam	Tm9sf1
Ppp1r15b	1.19E-14	0.318747	0.258	0.179	2.92E-10	TAM-Inflam	Ppp1r15b
Dek2	1.77E-14	0.279242	0.341	0.253	4.34E-10	TAM-Inflam	Dek
Ppp1r15a2	5.08E-14	0.31884	0.268	0.19	1.24E-09	TAM-Inflam	Ppp1r15a
Psmd121	5.48E-14	0.255349	0.279	0.197	1.34E-09	TAM-Inflam	Psmd12
Unc502	2.15E-13	0.263553	0.363	0.272	5.26E-09	TAM-Inflam	Unc50
Eif2s11	4.26E-13	0.303326	0.284	0.207	1.04E-08	TAM-Inflam	Eif2s1
Srd5a3	2.08E-12	0.263561	0.252	0.179	5.10E-08	TAM-Inflam	Srd5a3
Rasgef1b1	2.73E-12	0.259469	0.292	0.213	6.69E-08	TAM-Inflam	Rasgef1b
Herpud12	3.72E-11	0.268165	0.483	0.385	9.11E-07	TAM-Inflam	Herpud1
Ccl32	1.53E-10	0.516334	0.258	0.191	3.74E-06	TAM-Inflam	Ccl3
S100a9	0	11.64108	0.981	0.033	0	Neutrophils	S100a9
S100a8	0	11.72244	0.969	0.025	0	Neutrophils	S100a8
Pglyrp1	0	7.128046	0.904	0.029	0	Neutrophils	Pglyrp1
S100a111	0	5.827837	0.939	0.133	0	Neutrophils	S100a11

Lcn2	0	11.38516	0.805	0.005	0	Neutrophils	Lcn2
Slpi	0	9.013956	0.804	0.009	0	Neutrophils	Slpi
Hp	0	6.420188	0.797	0.02	0	Neutrophils	Hp
Retnlg	0	11.28178	0.773	0.006	0	Neutrophils	Retnlg
Mmp9	0	9.100966	0.759	0.005	0	Neutrophils	Mmp9
Wfdc21	0	11.73208	0.745	0.001	0	Neutrophils	Wfdc21
Anxa1	0	6.872146	0.767	0.046	0	Neutrophils	Anxa1
Msrb11	0	4.796933	0.894	0.175	0	Neutrophils	Msrb1
Gsr	0	4.493933	0.832	0.119	0	Neutrophils	Gsr
S100a61	0	5.43693	0.803	0.105	0	Neutrophils	S100a6
Anxa21	0	3.888916	0.816	0.124	0	Neutrophils	Anxa2
Gda	0	5.122635	0.693	0.039	0	Neutrophils	Gda
Hdc	0	7.11096	0.649	0.008	0	Neutrophils	Hdc
Mxd1	0	5.142039	0.702	0.065	0	Neutrophils	Mxd1
Ifitm6	0	6.164828	0.665	0.036	0	Neutrophils	Ifitm6
Ngp	0	11.53267	0.635	0.009	0	Neutrophils	Ngp
Pygl	0	5.081928	0.668	0.065	0	Neutrophils	Pygl
Cd24a	0	4.880573	0.61	0.029	0	Neutrophils	Cd24a
Lsp11	0	2.595057	0.772	0.192	0	Neutrophils	Lsp1
Cnn21	0	3.673667	0.69	0.111	0	Neutrophils	Cnn2
Mcemp1	0	5.158537	0.616	0.042	0	Neutrophils	Mcemp1
Hmgb2	0	3.936332	0.788	0.223	0	Neutrophils	Hmgb2
Camp	0	11.79344	0.565	0.009	0	Neutrophils	Camp
Txn12	0	3.389447	0.867	0.313	0	Neutrophils	Txn1
Padi4	0	8.127643	0.54	0.004	0	Neutrophils	Padi4
Mmp8	0	9.740459	0.54	0.004	0	Neutrophils	Mmp8
Iqgap12	0	3.058074	0.691	0.156	0	Neutrophils	Iqgap1
Mgst12	0	4.078768	0.598	0.066	0	Neutrophils	Mgst1
Sorl1	0	3.497722	0.697	0.17	0	Neutrophils	Sorl1
Trem3	0	6.59806	0.533	0.008	0	Neutrophils	Trem3
Ly6c21	0	2.603516	0.572	0.047	0	Neutrophils	Ly6c2
Ly6g	0	12.6548	0.52	0	0	Neutrophils	Ly6g
Syne1	0	4.385957	0.584	0.065	0	Neutrophils	Syne1
Ifitm22	0	3.152466	0.714	0.198	0	Neutrophils	Ifitm2
Rflnb	0	6.37787	0.524	0.01	0	Neutrophils	Rflnb
Cxcr2	0	9.167674	0.509	0.002	0	Neutrophils	Cxcr2
Cd177	0	7.96176	0.511	0.006	0	Neutrophils	Cd177
Lrg1	0	9.873784	0.504	0.001	0	Neutrophils	Lrg1
Ltf	0	11.21494	0.504	0.003	0	Neutrophils	Ltf
Lgals32	0	2.015574	0.678	0.18	0	Neutrophils	Lgals3
Plp2	0	4.747997	0.535	0.037	0	Neutrophils	Plp2
Chil1	0	7.330111	0.503	0.006	0	Neutrophils	Chil1
Ripor21	0	3.585606	0.612	0.119	0	Neutrophils	Ripor2
Msra	0	3.766323	0.585	0.096	0	Neutrophils	Msra

Adpgk	0	4.875853	0.556	0.076	0	Neutrophils	Adpgk
Actn1	0	3.964289	0.527	0.062	0	Neutrophils	Actn1
Plaur2	0	3.125825	0.623	0.165	0	Neutrophils	Plaur
Fgr1	0	3.410605	0.544	0.094	0	Neutrophils	Fgr
Mrpl331	0	2.791079	0.703	0.257	0	Neutrophils	Mrpl33
Plbd11	0	2.187649	0.549	0.112	0	Neutrophils	Plbd1
Fxyd51	0	2.723901	0.721	0.284	0	Neutrophils	Fxyd5
Slfn11	0	4.236031	0.489	0.052	0	Neutrophils	Slfn1
Slfn4	0	7.488723	0.443	0.006	0	Neutrophils	Slfn4
Arhgap152	0	2.073319	0.705	0.27	0	Neutrophils	Arhgap15
Sell	0	5.405781	0.442	0.018	0	Neutrophils	Sell
Pbx1	0	2.928823	0.576	0.153	0	Neutrophils	Pbx1
Pram1	0	5.713699	0.442	0.02	0	Neutrophils	Pram1
Cd441	0	2.706703	0.608	0.191	0	Neutrophils	Cd44
Lilr4b1	0	4.077201	0.464	0.049	0	Neutrophils	Lilr4b
Anxa11	0	3.615295	0.52	0.105	0	Neutrophils	Anxa11
F630028O1	0	4.659704	0.451	0.036	0	Neutrophils	F630028O10Rik
Vim1	0	1.482748	0.603	0.189	0	Neutrophils	Vim
Rac22	0	2.864962	0.862	0.449	0	Neutrophils	Rac2
Flna1	0	2.602003	0.594	0.186	0	Neutrophils	Flna
Glipr2	0	4.084286	0.439	0.035	0	Neutrophils	Glipr2
Gmfg2	0	2.526651	0.781	0.378	0	Neutrophils	Gmfg
Ckap4	0	3.505527	0.503	0.1	0	Neutrophils	Ckap4
Svil	0	2.584539	0.599	0.196	0	Neutrophils	Svil
Taldo12	0	2.569636	0.874	0.474	0	Neutrophils	Taldo1
Prr131	0	2.828509	0.723	0.326	0	Neutrophils	Prr13
Stk17b1	0	2.944856	0.558	0.163	0	Neutrophils	Stk17b
Pirb2	0	2.995353	0.54	0.145	0	Neutrophils	Pirb
Gsn1	0	2.893813	0.511	0.119	0	Neutrophils	Gsn
Cebpd3	0	2.319922	0.665	0.273	0	Neutrophils	Cebpd
Slc2a3	0	4.795195	0.418	0.027	0	Neutrophils	Slc2a3
Nedd91	0	3.134263	0.473	0.083	0	Neutrophils	Nedd9
Chil3	0	6.154216	0.403	0.015	0	Neutrophils	Chil3
Rasgrp4	0	3.821052	0.445	0.058	0	Neutrophils	Rasgrp4
Pilra1	0	3.178848	0.504	0.118	0	Neutrophils	Pilra
Cdk2ap21	0	2.80555	0.686	0.303	0	Neutrophils	Cdk2ap2
Adam8	0	4.618874	0.42	0.038	0	Neutrophils	Adam8
Emb1	0	3.700988	0.446	0.065	0	Neutrophils	Emb
2310001H1	0	4.185314	0.436	0.06	0	Neutrophils	2310001H17Rik
Il1r2	0	6.143279	0.392	0.018	0	Neutrophils	Il1r2
Hcst	0	2.796555	0.547	0.175	0	Neutrophils	Hcst
R3hdm4	0	2.697559	0.59	0.222	0	Neutrophils	R3hdm4
Ltb	0	4.095269	0.408	0.045	0	Neutrophils	Ltb
G0s2	0	6.392222	0.373	0.011	0	Neutrophils	G0s2

Nfe2	0	5.563105	0.372	0.012	0	Neutrophils	Nfe2
Pnkp	0	3.036613	0.486	0.127	0	Neutrophils	Pnkp
Cyfip2	0	3.624693	0.404	0.046	0	Neutrophils	Cyfip2
Cd300lf3	0	2.974206	0.493	0.137	0	Neutrophils	Cd300lf
Glrx6	0	2.921063	0.499	0.145	0	Neutrophils	Glrx
Cdkn2d	0	3.967099	0.401	0.05	0	Neutrophils	Cdkn2d
Alox5	0	3.096127	0.42	0.077	0	Neutrophils	Alox5
Abtb1	0	2.959152	0.461	0.123	0	Neutrophils	Abtb1
Serpinb1a	0	6.012983	0.345	0.007	0	Neutrophils	Serpinb1a
Mapk13	0	7.456234	0.339	0.004	0	Neutrophils	Mapk13
Fpr2	0	6.291505	0.342	0.008	0	Neutrophils	Fpr2
Dgat1	0	3.527098	0.398	0.066	0	Neutrophils	Dgat1
Rnf144a	0	6.174319	0.337	0.008	0	Neutrophils	Rnf144a
Dach1	0	5.846537	0.334	0.008	0	Neutrophils	Dach1
Unc119	0	3.580769	0.37	0.049	0	Neutrophils	Unc119
Pi16	0	5.934462	0.329	0.008	0	Neutrophils	Pi16
Lbr	0	2.968696	0.407	0.086	0	Neutrophils	Lbr
Atp8b4	0	3.879825	0.357	0.037	0	Neutrophils	Atp8b4
Pstpip11	0	3.440431	0.387	0.068	0	Neutrophils	Pstpip1
Tmem154	0	4.601174	0.345	0.028	0	Neutrophils	Tmem154
Prdx52	0	2.534705	0.898	0.583	0	Neutrophils	Prdx5
Ltb4r1	0	4.711816	0.334	0.021	0	Neutrophils	Ltb4r1
Lmnb1	0	3.785538	0.382	0.07	0	Neutrophils	Lmnb1
Lrrk2	0	4.539932	0.337	0.027	0	Neutrophils	Lrrk2
Cebpe	0	10.01522	0.303	0.001	0	Neutrophils	Cebpe
B230208H1	0	6.847347	0.303	0.005	0	Neutrophils	B230208H11Rik
Mirt1	0	5.094624	0.316	0.02	0	Neutrophils	Mirt1
Pilrb2	0	4.645556	0.317	0.023	0	Neutrophils	Pilrb2
Itgb2l	0	10.7014	0.292	0	0	Neutrophils	Itgb2l
Sem12	0	1.936827	0.832	0.546	0	Neutrophils	Sem1
1110008P1	0	3.025395	0.362	0.077	0	Neutrophils	1110008P14Rik
Dhrs9	0	9.310556	0.285	0.002	0	Neutrophils	Dhrs9
AA467197	0	4.097667	0.301	0.019	0	Neutrophils	AA467197
Dock5	0	3.605569	0.313	0.032	0	Neutrophils	Dock5
Clec4e	0	5.683558	0.288	0.01	0	Neutrophils	Clec4e
Cxcr4	0	4.526063	0.306	0.03	0	Neutrophils	Cxcr4
Coro1a1	0	1.71582	0.899	0.623	0	Neutrophils	Coro1a
Il18rap	0	5.70055	0.283	0.008	0	Neutrophils	Il18rap
Tuba4a	0	3.52407	0.32	0.047	0	Neutrophils	Tuba4a
Cd523	0	1.644501	0.942	0.669	0	Neutrophils	Cd52
Rasgrp2	0	3.800042	0.297	0.029	0	Neutrophils	Rasgrp2
Abca13	0	6.383848	0.27	0.005	0	Neutrophils	Abca13
Gsdme	0	3.759643	0.299	0.038	0	Neutrophils	Gsdme
Myl62	0	1.896664	0.865	0.604	0	Neutrophils	Myl6

Gm340841	0	3.561046	0.303	0.042	0	Neutrophils	Gm34084
Megf9	0	3.591032	0.29	0.031	0	Neutrophils	Megf9
Pde2a	0	3.221954	0.297	0.039	0	Neutrophils	Pde2a
H2-Q10	0	7.651676	0.259	0.004	0	Neutrophils	H2-Q10
Cd300lb	0	4.911365	0.274	0.021	0	Neutrophils	Cd300lb
Sgms2	0	5.425062	0.259	0.007	0	Neutrophils	Sgms2
Vcl	0	3.13177	0.293	0.045	0	Neutrophils	Vcl
Pip5k1b	0	4.356689	0.268	0.021	0	Neutrophils	Pip5k1b
6430548M(	0	3.79562	0.278	0.032	0	Neutrophils	6430548M08Rik
Stx11	0	4.457732	0.27	0.024	0	Neutrophils	Stx11
Arhgdib1	0	2.449582	0.86	0.615	0	Neutrophils	Arhgdib
Alox5ap2	0	1.83513	0.888	0.644	0	Neutrophils	Alox5ap
Fpr1	0	5.57368	0.253	0.011	0	Neutrophils	Fpr1
H3f3a2	0	1.810942	0.903	0.669	0	Neutrophils	H3f3a
Clec4d	0	5.054822	0.25	0.016	0	Neutrophils	Clec4d
Rab3d	0	4.086641	0.254	0.028	0	Neutrophils	Rab3d
Pfn11	0	1.589027	0.954	0.829	0	Neutrophils	Pfn1
Tmsb4x2	0	1.066461	0.983	0.967	0	Neutrophils	Tmsb4x
Actg11	5.95E-307	1.220358	0.941	0.78	1.46E-302	Neutrophils	Actg1
Lyst	1.07E-306	2.645547	0.597	0.219	2.63E-302	Neutrophils	Lyst
Lcp11	1.28E-299	1.577319	0.891	0.685	3.14E-295	Neutrophils	Lcp1
Nudt4	7.34E-291	2.664017	0.48	0.142	1.80E-286	Neutrophils	Nudt4
Hmgn21	8.71E-290	3.084127	0.645	0.316	2.13E-285	Neutrophils	Hmgn2
Myl12b1	8.42E-285	1.589012	0.853	0.63	2.06E-280	Neutrophils	Myl12b
Zyx1	1.09E-282	2.256719	0.621	0.249	2.66E-278	Neutrophils	Zyx
Antxr2	9.45E-280	3.649158	0.265	0.047	2.31E-275	Neutrophils	Antxr2
Fam107b	2.60E-279	2.479989	0.473	0.144	6.36E-275	Neutrophils	Fam107b
Klf23	5.59E-277	1.740747	0.618	0.218	1.37E-272	Neutrophils	Klf2
Adgre51	8.78E-276	2.66159	0.336	0.073	2.15E-271	Neutrophils	Adgre5
Xylt1	1.52E-274	2.679539	0.378	0.092	3.73E-270	Neutrophils	Xylt1
Ostf11	7.23E-271	1.799429	0.776	0.49	1.77E-266	Neutrophils	Ostf1
Cmss12	2.53E-267	1.486828	0.984	0.922	6.20E-263	Neutrophils	Cmss1
B430306N(	3.52E-260	3.307287	0.266	0.05	8.62E-256	Neutrophils	B430306N03Rik
Clec12a1	2.85E-258	2.400736	0.341	0.078	6.97E-254	Neutrophils	Clec12a
Lilrb4a3	3.40E-256	2.153496	0.501	0.162	8.33E-252	Neutrophils	Lilrb4a
Arpc52	3.71E-253	1.917456	0.723	0.425	9.09E-249	Neutrophils	Arpc5
Cpne2	2.63E-251	2.930295	0.275	0.055	6.44E-247	Neutrophils	Cpne2
Ccr12	1.45E-245	2.780031	0.315	0.071	3.55E-241	Neutrophils	Ccr1
Capn1	1.26E-242	3.148259	0.306	0.07	3.08E-238	Neutrophils	Capn1
Xdh1	3.38E-240	2.664062	0.373	0.101	8.28E-236	Neutrophils	Xdh
Cyp4f181	4.72E-239	2.99356	0.357	0.096	1.16E-234	Neutrophils	Cyp4f18
Tmcc11	1.70E-238	2.202352	0.685	0.352	4.15E-234	Neutrophils	Tmcc1
Dstn2	1.24E-236	2.43147	0.576	0.27	3.04E-232	Neutrophils	Dstn
AB124611	1.32E-235	2.458464	0.47	0.161	3.23E-231	Neutrophils	AB124611

Lyz23	3.09E-234	1.198865	0.886	0.676	7.58E-230	Neutrophils	Lyz2
Vasp1	1.02E-233	2.103018	0.631	0.312	2.49E-229	Neutrophils	Vasp
Vav32	1.43E-226	2.36553	0.315	0.075	3.50E-222	Neutrophils	Vav3
Cdc42ep3	8.60E-223	2.927177	0.285	0.065	2.11E-218	Neutrophils	Cdc42ep3
Ipcef1	1.47E-222	2.415611	0.385	0.111	3.59E-218	Neutrophils	Ipcef1
Actb2	1.36E-220	0.686109	0.989	0.958	3.34E-216	Neutrophils	Actb
Slfn23	3.09E-220	2.084357	0.622	0.289	7.56E-216	Neutrophils	Slfn2
Dhrs7	2.21E-217	2.322789	0.546	0.238	5.41E-213	Neutrophils	Dhrs7
Ezr1	1.05E-214	2.503682	0.339	0.091	2.56E-210	Neutrophils	Ezr
Dok31	3.49E-214	2.207516	0.481	0.176	8.54E-210	Neutrophils	Dok3
Mettl9	1.37E-212	2.251821	0.528	0.227	3.37E-208	Neutrophils	Mettl9
Serf23	3.74E-212	1.066256	0.903	0.757	9.17E-208	Neutrophils	Serf2
Sh3bgrl33	1.12E-210	1.032058	0.898	0.746	2.73E-206	Neutrophils	Sh3bgrl3
Cytip	1.87E-207	2.124236	0.316	0.079	4.58E-203	Neutrophils	Cytip
Pgd2	1.62E-206	2.039071	0.576	0.273	3.98E-202	Neutrophils	Pgd
Pkm2	6.84E-206	1.432621	0.764	0.502	1.67E-201	Neutrophils	Pkm
Gpi13	6.68E-205	1.660554	0.703	0.45	1.64E-200	Neutrophils	Gpi1
Map4k2	8.79E-205	2.493028	0.298	0.073	2.15E-200	Neutrophils	Map4k2
Gpsm31	1.97E-202	1.923032	0.658	0.382	4.83E-198	Neutrophils	Gpsm3
Aldh23	2.43E-200	2.057911	0.558	0.266	5.96E-196	Neutrophils	Aldh2
Il1b2	1.43E-197	4.03638	0.334	0.099	3.51E-193	Neutrophils	Il1b
Cybb3	4.11E-196	2.158401	0.527	0.23	1.01E-191	Neutrophils	Cybb
Capg2	1.74E-195	2.110222	0.412	0.137	4.26E-191	Neutrophils	Capg
Gpx11	5.26E-185	0.969802	0.881	0.698	1.29E-180	Neutrophils	Gpx1
Fbxl5	1.32E-182	2.708525	0.426	0.161	3.24E-178	Neutrophils	Fbxl5
Myh91	9.17E-179	1.334607	0.784	0.583	2.25E-174	Neutrophils	Myh9
Ncf2	1.88E-176	1.648307	0.715	0.463	4.61E-172	Neutrophils	Ncf2
Ncf41	1.30E-174	2.111867	0.521	0.251	3.18E-170	Neutrophils	Ncf4
Itgal1	1.81E-173	2.487873	0.304	0.087	4.44E-169	Neutrophils	Itgal
Aldh3b1	4.24E-173	2.571991	0.3	0.085	1.04E-168	Neutrophils	Aldh3b1
Ppp2r5a	6.49E-170	1.950798	0.473	0.196	1.59E-165	Neutrophils	Ppp2r5a
Ifitm33	3.03E-169	0.830968	0.757	0.424	7.42E-165	Neutrophils	Ifitm3
Map1lc3b3	7.65E-166	2.015452	0.608	0.341	1.87E-161	Neutrophils	Map1lc3b
Lmo41	4.52E-162	2.138026	0.397	0.147	1.11E-157	Neutrophils	Lmo4
Apobr	4.95E-162	2.685983	0.256	0.067	1.21E-157	Neutrophils	Apobr
Igf1r1	4.04E-161	1.725286	0.505	0.218	9.88E-157	Neutrophils	Igf1r
Arpc21	7.76E-147	0.976015	0.835	0.707	1.90E-142	Neutrophils	Arpc2
Arpc1b3	9.42E-146	0.971491	0.855	0.701	2.31E-141	Neutrophils	Arpc1b
Diaph11	1.16E-145	2.125507	0.371	0.138	2.85E-141	Neutrophils	Diaph1
Rap1gap2	2.98E-143	2.102562	0.318	0.104	7.30E-139	Neutrophils	Rap1gap2
Ehd12	2.23E-140	2.273025	0.313	0.105	5.47E-136	Neutrophils	Ehd1
Eif12	7.82E-140	0.821205	0.931	0.832	1.92E-135	Neutrophils	Eif1
Flot2	5.80E-137	2.302761	0.256	0.075	1.42E-132	Neutrophils	Flot2
Tkt2	2.15E-135	1.713884	0.612	0.423	5.26E-131	Neutrophils	Tkt

Nadk2	6.16E-133	1.811769	0.494	0.245	1.51E-128	Neutrophils	Nadk
Triobp	1.18E-132	2.296749	0.277	0.087	2.89E-128	Neutrophils	Triobp
Ncf12	2.15E-132	1.32817	0.703	0.543	5.26E-128	Neutrophils	Ncf1
Glipr11	2.57E-132	2.63827	0.31	0.11	6.29E-128	Neutrophils	Glipr1
C5ar11	5.73E-132	1.789402	0.507	0.251	1.40E-127	Neutrophils	C5ar1
Arpc33	3.73E-130	0.992444	0.793	0.639	9.13E-126	Neutrophils	Arpc3
Hipk1	1.04E-128	1.85013	0.419	0.183	2.54E-124	Neutrophils	Hipk1
Rinl	1.04E-128	2.318177	0.302	0.104	2.55E-124	Neutrophils	Rinl
Tcp11l2	7.71E-127	2.228835	0.265	0.083	1.89E-122	Neutrophils	Tcp11l2
Cyb5r4	6.70E-124	2.014805	0.364	0.148	1.64E-119	Neutrophils	Cyb5r4
Cers61	2.40E-123	1.910162	0.444	0.203	5.88E-119	Neutrophils	Cers6
G6pdx1	3.32E-123	2.015608	0.354	0.143	8.13E-119	Neutrophils	G6pdx
Nin1	1.16E-122	1.978011	0.347	0.136	2.84E-118	Neutrophils	Nin
Gapdh2	2.64E-121	0.938274	0.781	0.627	6.47E-117	Neutrophils	Gapdh
Add3	8.44E-120	1.811601	0.357	0.14	2.07E-115	Neutrophils	Add3
Sri2	3.99E-119	1.669787	0.536	0.319	9.77E-115	Neutrophils	Sri
Gm424182	9.36E-119	0.900346	0.998	0.982	2.29E-114	Neutrophils	Gm42418
Grk6	1.71E-118	2.136526	0.276	0.092	4.18E-114	Neutrophils	Grk6
1810058I24	1.98E-117	1.74615	0.483	0.26	4.84E-113	Neutrophils	1810058I24Rik
Agpat2	1.36E-116	2.217367	0.275	0.095	3.33E-112	Neutrophils	Agpat2
Gcnt2	2.93E-116	2.879306	0.266	0.091	7.16E-112	Neutrophils	Gcnt2
Csf3r2	9.95E-116	2.160171	0.684	0.506	2.44E-111	Neutrophils	Csf3r
Adipor12	2.68E-111	1.556526	0.637	0.437	6.56E-107	Neutrophils	Adipor1
Atg7	9.20E-111	2.234332	0.321	0.125	2.25E-106	Neutrophils	Atg7
Fam129a1	7.36E-110	1.911257	0.36	0.152	1.80E-105	Neutrophils	Fam129a
Sema4a2	4.18E-109	2.219524	0.293	0.109	1.02E-104	Neutrophils	Sema4a
Grina1	6.56E-108	1.557963	0.712	0.565	1.61E-103	Neutrophils	Grina
Emp32	1.75E-107	1.286563	0.451	0.218	4.29E-103	Neutrophils	Emp3
Neat12	1.96E-105	1.50099	0.53	0.294	4.80E-101	Neutrophils	Neat1
Morrbid2	9.68E-105	1.926499	0.307	0.118	2.37E-100	Neutrophils	Morrbid
Ccpg1	1.43E-103	2.106055	0.404	0.201	3.50E-99	Neutrophils	Ccpg1
Ccnd31	7.59E-103	0.875754	0.74	0.519	1.86E-98	Neutrophils	Ccnd3
Klhl2	6.91E-102	2.346918	0.261	0.094	1.69E-97	Neutrophils	Klhl2
H2afj2	2.12E-101	1.417808	0.596	0.385	5.19E-97	Neutrophils	H2afj
Chp1	2.65E-101	1.956951	0.341	0.146	6.49E-97	Neutrophils	Chp1
Actr31	6.87E-101	0.969734	0.711	0.555	1.68E-96	Neutrophils	Actr3
Lta4h	2.79E-100	2.534768	0.324	0.144	6.83E-96	Neutrophils	Lta4h
Ptprc2	2.28E-98	1.196861	0.741	0.573	5.59E-94	Neutrophils	Ptprc
Arhgap91	7.89E-98	1.901134	0.374	0.175	1.93E-93	Neutrophils	Arhgap9
Prdx62	2.29E-95	1.662292	0.395	0.194	5.62E-91	Neutrophils	Prdx6
Rin31	4.96E-95	1.624747	0.446	0.234	1.22E-90	Neutrophils	Rin3
Ppp1r2	7.45E-95	1.751219	0.424	0.217	1.82E-90	Neutrophils	Ppp1r2
Mpc21	1.31E-94	1.52122	0.524	0.336	3.22E-90	Neutrophils	Mpc2
Fry	4.65E-93	1.584047	0.382	0.175	1.14E-88	Neutrophils	Fry

Golim4	1.05E-92	1.819292	0.292	0.115	2.57E-88	Neutrophils	Golim4
Nfam12	2.00E-92	1.492159	0.55	0.348	4.89E-88	Neutrophils	Nfam1
Card192	5.93E-91	1.773313	0.448	0.246	1.45E-86	Neutrophils	Card19
Ptpn12	1.86E-90	1.986572	0.259	0.097	4.56E-86	Neutrophils	Ptpn12
Rbms12	4.24E-90	1.503196	0.432	0.226	1.04E-85	Neutrophils	Rbms1
Atp11b	4.43E-89	1.864267	0.322	0.14	1.08E-84	Neutrophils	Atp11b
Grk2	5.92E-89	1.393983	0.542	0.342	1.45E-84	Neutrophils	Grk2
Srgn1	1.48E-88	2.44081	0.649	0.553	3.61E-84	Neutrophils	Srgn
Cap1	3.87E-88	1.151508	0.659	0.507	9.49E-84	Neutrophils	Cap1
Oaz13	5.31E-88	0.73001	0.805	0.721	1.30E-83	Neutrophils	Oaz1
Siglece1	4.53E-87	1.833598	0.368	0.177	1.11E-82	Neutrophils	Siglece
Gm267401	1.88E-86	1.562961	0.508	0.303	4.60E-82	Neutrophils	Gm26740
Ccl6	2.09E-86	2.882389	0.541	0.406	5.11E-82	Neutrophils	Ccl6
Gnai21	4.29E-86	0.713272	0.856	0.782	1.05E-81	Neutrophils	Gnai2
Slc16a3	1.99E-85	2.176888	0.272	0.11	4.88E-81	Neutrophils	Slc16a3
Gng53	1.18E-84	0.755856	0.826	0.719	2.90E-80	Neutrophils	Gng5
Fis13	5.37E-83	1.105985	0.625	0.467	1.32E-78	Neutrophils	Fis1
Atg3	8.73E-83	1.841942	0.42	0.235	2.14E-78	Neutrophils	Atg3
Lars22	3.12E-82	0.941004	0.882	0.753	7.63E-78	Neutrophils	Lars2
Ndel1	2.72E-79	1.888338	0.365	0.187	6.66E-75	Neutrophils	Ndel1
Scp23	2.79E-78	1.423739	0.499	0.338	6.83E-74	Neutrophils	Scp2
Il17ra	8.19E-78	1.583675	0.393	0.207	2.00E-73	Neutrophils	Il17ra
C33	1.75E-77	1.176642	0.32	0.143	4.29E-73	Neutrophils	C3
Ubb4	4.11E-76	0.673944	0.933	0.865	1.01E-71	Neutrophils	Ubb
Calm13	7.13E-76	0.728872	0.729	0.592	1.75E-71	Neutrophils	Calm1
Myl12a2	5.48E-75	1.021096	0.608	0.452	1.34E-70	Neutrophils	Myl12a
Vamp5	6.06E-72	1.648426	0.321	0.153	1.49E-67	Neutrophils	Vamp5
Cpne31	1.14E-71	1.69132	0.376	0.21	2.79E-67	Neutrophils	Cpne3
Rab5if3	1.15E-71	1.17956	0.555	0.394	2.82E-67	Neutrophils	Rab5if
Lamtor42	1.50E-71	1.265645	0.574	0.443	3.67E-67	Neutrophils	Lamtor4
Mfsd14b	3.00E-71	1.674113	0.31	0.145	7.35E-67	Neutrophils	Mfsd14b
Acsl11	3.21E-71	1.541338	0.328	0.158	7.87E-67	Neutrophils	Acsl1
Scand12	3.58E-71	1.19633	0.579	0.415	8.76E-67	Neutrophils	Scand1
Hacd42	7.33E-71	1.761517	0.382	0.215	1.79E-66	Neutrophils	Hacd4
Gabarap4	3.18E-70	0.811169	0.812	0.674	7.79E-66	Neutrophils	Gabarap
Ethe12	6.02E-70	1.775156	0.288	0.134	1.47E-65	Neutrophils	Ethe1
Ttr3	1.13E-69	1.342213	0.687	0.507	2.77E-65	Neutrophils	Ttr
Ier22	1.54E-69	0.812039	0.58	0.351	3.78E-65	Neutrophils	Ier2
Pgk13	2.73E-69	1.306919	0.432	0.256	6.70E-65	Neutrophils	Pgk1
Sh2d3c	3.89E-68	1.922396	0.261	0.115	9.52E-64	Neutrophils	Sh2d3c
Arap3	4.78E-67	1.464597	0.314	0.149	1.17E-62	Neutrophils	Arap3
Tspo4	1.84E-66	0.78357	0.622	0.447	4.50E-62	Neutrophils	Tspo
Rab27a	3.98E-66	1.56985	0.305	0.146	9.75E-62	Neutrophils	Rab27a
Pla2g73	1.39E-65	2.014195	0.312	0.159	3.39E-61	Neutrophils	Pla2g7

Kdm6b	1.53E-65	1.851813	0.25	0.109	3.75E-61	Neutrophils	Kdm6b
Rassf3	2.42E-65	1.733818	0.346	0.184	5.93E-61	Neutrophils	Rassf3
Gpx43	2.54E-65	0.890107	0.716	0.596	6.23E-61	Neutrophils	Gpx4
Mcl12	3.88E-65	1.353379	0.668	0.539	9.51E-61	Neutrophils	Mcl1
Ube2b	4.98E-65	1.284822	0.524	0.362	1.22E-60	Neutrophils	Ube2b
Cdc42se11	5.83E-63	1.45071	0.445	0.28	1.43E-58	Neutrophils	Cdc42se1
Igsf62	6.52E-62	1.44425	0.389	0.225	1.60E-57	Neutrophils	Igsf6
Il31ra2	7.24E-62	0.89963	0.747	0.614	1.77E-57	Neutrophils	Il31ra
Fam32a1	5.60E-61	1.704957	0.365	0.21	1.37E-56	Neutrophils	Fam32a
Ppp1r181	3.07E-60	1.036382	0.614	0.473	7.51E-56	Neutrophils	Ppp1r18
Pxn	7.90E-60	1.368413	0.383	0.217	1.94E-55	Neutrophils	Pxn
Flot1	1.24E-59	1.532346	0.33	0.175	3.04E-55	Neutrophils	Flot1
Cast1	1.14E-58	1.614634	0.253	0.115	2.79E-54	Neutrophils	Cast
Samhd12	2.61E-58	1.115585	0.554	0.391	6.39E-54	Neutrophils	Samhd1
Pim13	2.65E-57	2.227808	0.284	0.145	6.49E-53	Neutrophils	Pim1
Atp5l3	6.31E-57	0.789949	0.7	0.567	1.54E-52	Neutrophils	Atp5l
Ndufb73	1.51E-56	1.111192	0.508	0.373	3.69E-52	Neutrophils	Ndufb7
Max2	3.22E-56	1.415426	0.417	0.261	7.89E-52	Neutrophils	Max
Rab6a	8.03E-56	1.531534	0.304	0.158	1.97E-51	Neutrophils	Rab6a
Srpk2	2.87E-55	1.4489	0.34	0.186	7.03E-51	Neutrophils	Srpk2
Usp32	7.49E-55	1.741786	0.314	0.171	1.84E-50	Neutrophils	Usp32
Kdm7a1	8.99E-54	1.292548	0.413	0.257	2.20E-49	Neutrophils	Kdm7a
Ppm1m	2.11E-53	1.529662	0.265	0.13	5.16E-49	Neutrophils	Ppm1m
Atp6v1g12	3.17E-53	0.856415	0.697	0.571	7.77E-49	Neutrophils	Atp6v1g1
Jdp2	8.75E-53	1.432955	0.25	0.118	2.14E-48	Neutrophils	Jdp2
Calm32	1.05E-52	1.120055	0.477	0.332	2.57E-48	Neutrophils	Calm3
Aldoa3	1.41E-52	0.65648	0.708	0.595	3.45E-48	Neutrophils	Aldoa
Fmnl1	1.78E-52	1.179153	0.492	0.341	4.35E-48	Neutrophils	Fmnl1
Slc9a3r12	1.39E-51	1.551	0.315	0.175	3.41E-47	Neutrophils	Slc9a3r1
Eno13	2.38E-51	1.175139	0.45	0.298	5.83E-47	Neutrophils	Eno1
Riok3	2.92E-51	1.379717	0.376	0.227	7.16E-47	Neutrophils	Riok3
Litaf2	3.97E-51	1.27574	0.524	0.394	9.72E-47	Neutrophils	Litaf
Filip1l3	1.34E-49	1.110032	0.593	0.479	3.28E-45	Neutrophils	Filip1l
Cfl13	2.03E-49	0.438499	0.844	0.764	4.97E-45	Neutrophils	Cfl1
1810037I1	3.98E-49	1.180655	0.469	0.341	9.74E-45	Neutrophils	1810037I17Rik
Lasp1	5.58E-49	1.21688	0.401	0.255	1.37E-44	Neutrophils	Lasp1
Cdk11b	1.21E-48	1.346812	0.354	0.209	2.97E-44	Neutrophils	Cdk11b
Mctp12	1.27E-47	0.774144	0.51	0.338	3.12E-43	Neutrophils	Mctp1
Capza11	1.67E-47	0.987511	0.525	0.398	4.10E-43	Neutrophils	Capza1
Ap3s1	7.58E-47	1.369611	0.337	0.202	1.86E-42	Neutrophils	Ap3s1
Ptpre2	9.03E-47	1.115143	0.483	0.342	2.21E-42	Neutrophils	Ptpre
Stk38	1.31E-46	1.301564	0.365	0.222	3.21E-42	Neutrophils	Stk38
Pfkfb4	1.65E-45	1.565736	0.282	0.153	4.05E-41	Neutrophils	Pfkfb4
Enpp22	1.67E-45	1.397434	0.376	0.227	4.10E-41	Neutrophils	Enpp2

Sep-91	2.56E-44	1.396789	0.266	0.141	6.26E-40	Neutrophils	9-Sep
Smim41	9.78E-44	1.587999	0.253	0.132	2.40E-39	Neutrophils	Smim4
Hk32	1.03E-43	1.135027	0.402	0.263	2.52E-39	Neutrophils	Hk3
Sqor	4.60E-42	1.236701	0.328	0.196	1.13E-37	Neutrophils	Sqor
Sun22	1.30E-39	1.132119	0.387	0.258	3.18E-35	Neutrophils	Sun2
AY0361182	1.39E-39	0.644608	0.868	0.806	3.41E-35	Neutrophils	AY036118
Serp12	1.40E-39	1.091622	0.485	0.361	3.43E-35	Neutrophils	Serp1
Gnb21	4.64E-39	0.649811	0.662	0.584	1.14E-34	Neutrophils	Gnb2
D1Ertd622e	6.01E-39	1.479594	0.254	0.138	1.47E-34	Neutrophils	D1Ertd622e
Arhgef11	1.34E-38	1.06176	0.409	0.28	3.29E-34	Neutrophils	Arhgef1
Ppp1cb	1.04E-37	1.134302	0.31	0.184	2.56E-33	Neutrophils	Ppp1cb
Clec4a23	1.56E-37	1.01063	0.39	0.258	3.81E-33	Neutrophils	Clec4a2
Tpm41	2.58E-37	1.579938	0.276	0.165	6.32E-33	Neutrophils	Tpm4
Gnai3	1.53E-36	1.210077	0.28	0.161	3.75E-32	Neutrophils	Gnai3
Eif4ebp12	5.07E-36	1.389948	0.326	0.208	1.24E-31	Neutrophils	Eif4ebp1
Fgd4	1.09E-35	1.082944	0.321	0.193	2.68E-31	Neutrophils	Fgd4
Syk1	2.66E-35	1.114323	0.464	0.349	6.51E-31	Neutrophils	Syk
Limd23	5.48E-35	0.728294	0.662	0.599	1.34E-30	Neutrophils	Limd2
Rp9	8.59E-35	1.139689	0.419	0.302	2.10E-30	Neutrophils	Rp9
Supt4a1	9.29E-35	1.239481	0.423	0.306	2.28E-30	Neutrophils	Supt4a
H2afz1	9.37E-35	1.055008	0.617	0.575	2.30E-30	Neutrophils	H2afz
Gpcpd1	1.09E-34	1.320724	0.366	0.251	2.68E-30	Neutrophils	Gpcpd1
Cmip1	1.59E-34	0.922001	0.502	0.382	3.88E-30	Neutrophils	Cmip
Cyba4	9.22E-34	0.312138	0.904	0.861	2.26E-29	Neutrophils	Cyba
Msl1	4.44E-33	1.169951	0.307	0.191	1.09E-28	Neutrophils	Msl1
Dusp14	8.92E-33	1.12602	0.507	0.384	2.18E-28	Neutrophils	Dusp1
Cox171	1.18E-32	0.96316	0.456	0.348	2.88E-28	Neutrophils	Cox17
S100a132	1.35E-32	1.129349	0.338	0.226	3.31E-28	Neutrophils	S100a13
Tprgl	2.26E-32	1.167023	0.375	0.262	5.53E-28	Neutrophils	Tprgl
Abracl1	3.78E-32	1.085873	0.395	0.291	9.26E-28	Neutrophils	Abracl
St3gal52	1.08E-30	0.794279	0.473	0.362	2.66E-26	Neutrophils	St3gal5
Mbp3	2.49E-30	0.85805	0.44	0.316	6.09E-26	Neutrophils	Mbp
Tubb4b	2.60E-30	1.368065	0.272	0.17	6.37E-26	Neutrophils	Tubb4b
Lrrfip2	2.69E-30	1.187632	0.255	0.149	6.58E-26	Neutrophils	Lrrfip2
Mbd23	2.85E-30	1.07545	0.363	0.254	6.99E-26	Neutrophils	Mbd2
Ywhaz	3.26E-30	0.588534	0.635	0.56	7.99E-26	Neutrophils	Ywhaz
Pacs11	4.20E-30	1.097874	0.325	0.212	1.03E-25	Neutrophils	Pacs1
Tsc22d33	4.65E-30	1.057365	0.417	0.299	1.14E-25	Neutrophils	Tsc22d3
Aprt2	9.06E-30	1.089799	0.375	0.274	2.22E-25	Neutrophils	Aprt
Pde4d	1.05E-29	1.009522	0.263	0.153	2.57E-25	Neutrophils	Pde4d
Tgfbi2	1.37E-29	0.985969	0.316	0.202	3.35E-25	Neutrophils	Tgfbi
Rnaseh2c2	1.93E-29	1.171571	0.329	0.227	4.73E-25	Neutrophils	Rnaseh2c
Atxn101	2.31E-29	0.927289	0.456	0.358	5.67E-25	Neutrophils	Atxn10
Akap131	3.26E-29	0.786042	0.582	0.496	7.98E-25	Neutrophils	Akap13

Hcls11	1.51E-28	0.849598	0.511	0.413	3.71E-24	Neutrophils	Hcls1
Mdh23	2.06E-28	0.899675	0.452	0.355	5.05E-24	Neutrophils	Mdh2
Btg12	2.77E-28	1.161647	0.588	0.531	6.78E-24	Neutrophils	Btg1
Ldha3	1.19E-27	0.710537	0.524	0.445	2.92E-23	Neutrophils	Ldha
Ppp2cb	5.82E-27	1.16725	0.277	0.176	1.43E-22	Neutrophils	Ppp2cb
Baz2b1	6.27E-27	0.771767	0.491	0.384	1.54E-22	Neutrophils	Baz2b
Lst13	9.14E-27	0.720294	0.643	0.537	2.24E-22	Neutrophils	Lst1
Pgam13	1.00E-26	0.859743	0.439	0.349	2.45E-22	Neutrophils	Pgam1
Ssu721	1.07E-26	0.910422	0.458	0.367	2.63E-22	Neutrophils	Ssu72
Cript1	1.18E-26	1.166529	0.287	0.185	2.90E-22	Neutrophils	Cript
Tecr2	2.06E-26	0.76783	0.53	0.475	5.04E-22	Neutrophils	Tecr
Tle52	2.09E-26	0.694322	0.487	0.405	5.12E-22	Neutrophils	Tle5
Znhit12	2.43E-26	1.156164	0.294	0.193	5.96E-22	Neutrophils	Znhit1
Phf20l11	2.64E-26	0.931945	0.408	0.301	6.47E-22	Neutrophils	Phf20l1
Dazap22	2.83E-26	0.83588	0.536	0.455	6.93E-22	Neutrophils	Dazap2
Rnf11	3.38E-26	1.376602	0.261	0.165	8.27E-22	Neutrophils	Rnf11
Mxi1	3.73E-26	1.053062	0.29	0.185	9.13E-22	Neutrophils	Mxi1
Cpd1	5.26E-26	1.310614	0.269	0.172	1.29E-21	Neutrophils	Cpd
Atp5e2	6.37E-26	0.487807	0.724	0.651	1.56E-21	Neutrophils	Atp5e
Tyrobp2	8.09E-26	0.495868	0.956	0.916	1.98E-21	Neutrophils	Tyrobp
Ppp1r12a1	9.33E-26	0.726097	0.511	0.42	2.28E-21	Neutrophils	Ppp1r12a
Degs11	1.24E-25	1.10741	0.342	0.244	3.03E-21	Neutrophils	Degs1
Gm199512	1.43E-25	0.912387	0.462	0.365	3.50E-21	Neutrophils	Gm19951
Sp1002	1.45E-25	0.851615	0.385	0.277	3.56E-21	Neutrophils	Sp100
Rapgef61	1.46E-25	1.02251	0.383	0.281	3.58E-21	Neutrophils	Rapgef6
Ube2d31	4.40E-25	0.613424	0.591	0.528	1.08E-20	Neutrophils	Ube2d3
Klf132	4.83E-25	0.846891	0.414	0.311	1.18E-20	Neutrophils	Klf13
Cdk83	7.33E-25	0.489307	0.932	0.902	1.80E-20	Neutrophils	Cdk8
Rchy1	1.50E-24	1.259603	0.266	0.172	3.68E-20	Neutrophils	Rchy1
Ubl53	1.73E-24	0.681812	0.558	0.484	4.23E-20	Neutrophils	Ubl5
Tln12	2.14E-24	0.600903	0.551	0.478	5.25E-20	Neutrophils	Tln1
Cotl11	2.52E-24	0.593376	0.593	0.514	6.18E-20	Neutrophils	Cotl1
Gphn2	7.75E-24	0.64983	0.703	0.641	1.90E-19	Neutrophils	Gphn
Trpm21	8.09E-24	1.313864	0.274	0.185	1.98E-19	Neutrophils	Trpm2
Akna	1.06E-23	1.026437	0.311	0.213	2.59E-19	Neutrophils	Akna
Elob4	1.18E-23	0.611846	0.588	0.525	2.90E-19	Neutrophils	Elob
Prkcb1	2.64E-23	0.545922	0.599	0.517	6.46E-19	Neutrophils	Prkcb
Snap231	3.46E-23	1.564884	0.33	0.251	8.47E-19	Neutrophils	Snap23
Atp6v1e12	4.10E-23	0.909918	0.38	0.29	1.00E-18	Neutrophils	Atp6v1e1
Cdc421	4.73E-23	0.424548	0.763	0.732	1.16E-18	Neutrophils	Cdc42
Raf1	7.87E-23	1.296022	0.278	0.188	1.93E-18	Neutrophils	Raf1
Hbb-bs1	1.39E-22	0.504198	0.259	0.162	3.40E-18	Neutrophils	Hbb-bs
Tpd521	1.77E-22	1.202018	0.476	0.429	4.33E-18	Neutrophils	Tpd52
Prelid13	3.27E-22	0.815216	0.422	0.337	8.01E-18	Neutrophils	Prelid1

Sgms1	4.14E-22	1.098093	0.275	0.186	1.01E-17	Neutrophils	Sgms1
D8Ertd738e	1.30E-21	0.827238	0.512	0.445	3.17E-17	Neutrophils	D8Ertd738e
Rcsd11	1.71E-21	0.742658	0.473	0.375	4.20E-17	Neutrophils	Rcsd1
Pten2	1.96E-21	0.760185	0.517	0.445	4.80E-17	Neutrophils	Pten
Arfgef1	2.28E-21	0.949668	0.282	0.188	5.59E-17	Neutrophils	Arfgef1
Stxbp22	4.68E-21	1.175747	0.259	0.174	1.15E-16	Neutrophils	Stxbp2
Gm155643	1.15E-20	1.124538	0.319	0.231	2.81E-16	Neutrophils	Gm15564
Rbfa2	1.19E-20	1.0986	0.273	0.187	2.92E-16	Neutrophils	Rbfa
Cebpb3	2.68E-20	0.915127	0.599	0.54	6.56E-16	Neutrophils	Cebpb
Mtmr31	6.15E-20	0.755591	0.35	0.252	1.51E-15	Neutrophils	Mtmr3
Arrb21	9.99E-20	0.632831	0.564	0.53	2.45E-15	Neutrophils	Arrb2
Rhog1	2.34E-19	0.497841	0.677	0.633	5.73E-15	Neutrophils	Rhog
Ptgds2	3.33E-19	0.382038	0.35	0.254	8.16E-15	Neutrophils	Ptgds
Mapk3	3.37E-19	0.830793	0.412	0.333	8.24E-15	Neutrophils	Mapk3
Samsn12	4.21E-19	0.882764	0.335	0.245	1.03E-14	Neutrophils	Samsn1
Smim143	4.61E-19	0.814783	0.385	0.306	1.13E-14	Neutrophils	Smim14
Stim1	4.96E-19	0.990545	0.253	0.167	1.21E-14	Neutrophils	Stim1
Slc40a12	6.32E-19	0.737538	0.316	0.221	1.55E-14	Neutrophils	Slc40a1
Ap1s21	1.08E-18	0.957682	0.25	0.167	2.65E-14	Neutrophils	Ap1s2
Ndufb93	1.62E-18	0.622701	0.519	0.451	3.97E-14	Neutrophils	Ndufb9
Herc41	2.21E-18	1.00927	0.343	0.261	5.41E-14	Neutrophils	Herc4
Far1	2.37E-18	0.927106	0.279	0.193	5.81E-14	Neutrophils	Far1
Ppp1ca1	2.63E-18	0.595111	0.517	0.465	6.43E-14	Neutrophils	Ppp1ca
Mt32	3.49E-18	0.738392	0.306	0.219	8.55E-14	Neutrophils	Mt3
Hpcal13	1.59E-17	0.864895	0.342	0.262	3.88E-13	Neutrophils	Hpcal1
Spi11	5.99E-17	0.475791	0.728	0.706	1.47E-12	Neutrophils	Spi1
Ncor11	3.31E-16	0.678817	0.476	0.414	8.11E-12	Neutrophils	Ncor1
Tnrc6b1	6.12E-16	0.644119	0.444	0.365	1.50E-11	Neutrophils	Tnrc6b
Mark21	6.33E-16	0.722431	0.322	0.24	1.55E-11	Neutrophils	Mark2
Preb	1.76E-15	1.181667	0.265	0.195	4.31E-11	Neutrophils	Preb
Tut7	3.00E-15	0.876217	0.351	0.28	7.35E-11	Neutrophils	Tut7
Ypel32	3.63E-15	0.729561	0.591	0.582	8.89E-11	Neutrophils	Ypel3
Tbc1d81	3.64E-15	0.703476	0.323	0.242	8.90E-11	Neutrophils	Tbc1d8
Rock11	3.88E-15	0.758482	0.38	0.309	9.50E-11	Neutrophils	Rock1
Aurkaip14	8.50E-15	0.822008	0.335	0.263	2.08E-10	Neutrophils	Aurkaip1
Npepps	1.12E-14	0.747888	0.291	0.213	2.75E-10	Neutrophils	Npepps
Tmem1341	2.26E-14	0.884849	0.325	0.255	5.53E-10	Neutrophils	Tmem134
Bnip3l1	2.63E-14	0.655229	0.418	0.358	6.44E-10	Neutrophils	Bnip3l
Ppp3ca1	3.64E-14	0.457544	0.593	0.549	8.91E-10	Neutrophils	Ppp3ca
Lrrfip1	3.95E-14	0.690793	0.382	0.313	9.68E-10	Neutrophils	Lrrfip1
Anp32a2	5.09E-14	0.730446	0.41	0.351	1.25E-09	Neutrophils	Anp32a
Rab21	6.65E-14	0.851319	0.305	0.236	1.63E-09	Neutrophils	Rab21
Wdr11	1.02E-13	0.681389	0.421	0.366	2.49E-09	Neutrophils	Wdr1
Jarid22	1.20E-13	0.767593	0.4	0.335	2.93E-09	Neutrophils	Jarid2

Chmp2a2	4.44E-13	0.784778	0.369	0.309	1.09E-08	Neutrophils	Chmp2a
Pde4b1	4.63E-13	1.133295	0.297	0.233	1.13E-08	Neutrophils	Pde4b
Fes	8.71E-13	0.778787	0.381	0.321	2.13E-08	Neutrophils	Fes
Myo1f1	1.12E-12	0.436524	0.656	0.628	2.74E-08	Neutrophils	Myo1f
Ogfrl1	1.75E-12	0.716487	0.392	0.331	4.28E-08	Neutrophils	Ogfrl1
Vamp83	2.30E-12	0.37346	0.635	0.596	5.62E-08	Neutrophils	Vamp8
Fgfr2	2.84E-12	0.843631	0.263	0.196	6.95E-08	Neutrophils	Fgfr2
Tsc22d41	3.48E-12	0.60498	0.467	0.42	8.53E-08	Neutrophils	Tsc22d4
Map3k51	3.53E-12	0.940346	0.327	0.263	8.65E-08	Neutrophils	Map3k5
Tax1bp11	9.24E-12	0.694341	0.406	0.353	2.26E-07	Neutrophils	Tax1bp1
Gm480993	1.62E-11	0.794612	0.334	0.275	3.96E-07	Neutrophils	Gm48099
Ogt1	3.23E-11	0.966012	0.313	0.26	7.91E-07	Neutrophils	Ogt
Cklf2	3.54E-11	0.676053	0.313	0.25	8.67E-07	Neutrophils	Cklf
Ankrd121	5.03E-11	0.608543	0.317	0.252	1.23E-06	Neutrophils	Ankrd12
Ndufb112	5.74E-11	0.527794	0.468	0.433	1.41E-06	Neutrophils	Ndufb11
Sf3b22	6.80E-11	0.631106	0.42	0.376	1.67E-06	Neutrophils	Sf3b2
Oga	7.08E-11	0.833944	0.255	0.195	1.73E-06	Neutrophils	Oga
Stk4	7.98E-11	0.704333	0.35	0.291	1.95E-06	Neutrophils	Stk4
Atp5h2	1.77E-10	0.353756	0.574	0.555	4.34E-06	Neutrophils	Atp5h
Znrf11	4.06E-10	0.655295	0.286	0.222	9.95E-06	Neutrophils	Znrf1
Cox7a24	4.69E-10	0.4596	0.492	0.464	1.15E-05	Neutrophils	Cox7a2
Tmem1641	5.63E-10	0.647384	0.343	0.286	1.38E-05	Neutrophils	Tmem164
Ncoa41	6.03E-10	0.767569	0.256	0.198	1.48E-05	Neutrophils	Ncoa4
Zc3h7a2	8.01E-10	0.574594	0.514	0.493	1.96E-05	Neutrophils	Zc3h7a
Sik3	8.46E-10	0.714509	0.263	0.203	2.07E-05	Neutrophils	Sik3
Timm10b1	9.88E-10	0.699253	0.469	0.435	2.42E-05	Neutrophils	Timm10b
Ralbp11	1.22E-09	0.692064	0.296	0.238	2.99E-05	Neutrophils	Ralbp1
Sec61b2	1.54E-09	0.406111	0.507	0.465	3.78E-05	Neutrophils	Sec61b
Add11	1.86E-09	0.626368	0.301	0.243	4.56E-05	Neutrophils	Add1
Rhoa1	2.15E-09	0.294163	0.709	0.69	5.27E-05	Neutrophils	Rhoa
Gabarapl2	2.98E-09	0.56857	0.418	0.379	7.29E-05	Neutrophils	Gabarapl2
Txndc173	3.22E-09	0.546598	0.36	0.312	7.88E-05	Neutrophils	Txndc17
Mtpn1	3.43E-09	0.737667	0.283	0.229	8.40E-05	Neutrophils	Mtpn
Ndufa31	3.84E-09	0.605517	0.422	0.382	9.40E-05	Neutrophils	Ndufa3
Aip1	4.16E-09	0.749168	0.259	0.205	0.000102	Neutrophils	Aip
N4bp12	4.55E-09	0.856112	0.294	0.24	0.000112	Neutrophils	N4bp1
Bbip1	5.55E-09	0.753273	0.284	0.231	0.000136	Neutrophils	Bbip1
Rab8a1	6.08E-09	0.949391	0.253	0.205	0.000149	Neutrophils	Rab8a
Psmb32	1.38E-08	0.476789	0.436	0.403	0.000339	Neutrophils	Psmb3
Itgb21	2.07E-08	0.370234	0.577	0.59	0.000507	Neutrophils	Itgb2
Dynll12	2.43E-08	0.585576	0.448	0.421	0.000594	Neutrophils	Dynll1
Camk1d2	3.84E-08	0.286541	0.813	0.809	0.000941	Neutrophils	Camk1d
Ywhab1	8.65E-08	0.558107	0.381	0.35	0.002119	Neutrophils	Ywhab
Eif51	1.08E-07	0.604902	0.388	0.349	0.002634	Neutrophils	Eif5

Msn1	1.14E-07	0.346599	0.612	0.594	0.002796	Neutrophils	Msn
Mir142hg1	1.18E-07	0.264285	0.629	0.593	0.002894	Neutrophils	Mir142hg
Pbxip1	1.39E-07	0.870693	0.29	0.251	0.003393	Neutrophils	Pbxip1
Fgl23	1.67E-07	0.531665	0.185	0.278	0.004085	Neutrophils	Fgl2
Nsd31	2.77E-07	0.704608	0.36	0.324	0.006779	Neutrophils	Nsd3
Mkrn12	2.96E-07	0.525154	0.343	0.302	0.007254	Neutrophils	Mkrn1
Rtp43	3.94E-07	0.481891	0.171	0.252	0.009644	Neutrophils	Rtp4
Rgs192	4.94E-07	0.583086	0.366	0.337	0.012108	Neutrophils	Rgs19
Mafg2	5.30E-07	0.542758	0.297	0.248	0.012976	Neutrophils	Mafg
Ndufa15	6.46E-07	0.546746	0.372	0.332	0.015818	Neutrophils	Ndufa1
Cop11	6.62E-07	0.5898	0.263	0.214	0.016222	Neutrophils	Cop1
Ptpn63	7.42E-07	0.43904	0.533	0.516	0.018178	Neutrophils	Ptpn6
Tomm63	9.61E-07	0.356275	0.549	0.535	0.023528	Neutrophils	Tomm6
Il162	9.71E-07	0.523457	0.31	0.267	0.023778	Neutrophils	Il16
Ptbp31	1.13E-06	0.432312	0.502	0.487	0.027731	Neutrophils	Ptbp3
Csnk2b2	1.19E-06	0.508235	0.372	0.339	0.029081	Neutrophils	Csnk2b
4932438A1	1.57E-06	0.777114	0.253	0.21	0.03847	Neutrophils	4932438A13Rik
Cox5b3	2.14E-06	0.299049	0.566	0.548	0.052424	Neutrophils	Cox5b
Mrps212	2.42E-06	0.538185	0.379	0.347	0.059227	Neutrophils	Mrps21
Gng124	2.43E-06	0.57785	0.338	0.3	0.059502	Neutrophils	Gng12
Ndufa133	3.20E-06	0.324803	0.555	0.538	0.078352	Neutrophils	Ndufa13
Cited24	3.45E-06	0.304892	0.338	0.295	0.084388	Neutrophils	Cited2
Stk24	3.51E-06	0.464823	0.261	0.216	0.086023	Neutrophils	Stk24
Arf51	3.70E-06	0.336079	0.506	0.484	0.090626	Neutrophils	Arf5
Tnfaip82	4.03E-06	0.489349	0.364	0.327	0.098661	Neutrophils	Tnfaip8
Atp5j4	4.29E-06	0.329843	0.552	0.546	0.105131	Neutrophils	Atp5j
Rnf10	5.06E-06	0.591656	0.259	0.218	0.124011	Neutrophils	Rnf10
Leprotl12	5.68E-06	0.611026	0.314	0.278	0.139208	Neutrophils	Leprotl1
Mrtfa1	7.11E-06	0.445624	0.278	0.233	0.174062	Neutrophils	Mrtfa
Jak11	8.74E-06	0.515341	0.432	0.412	0.21394	Neutrophils	Jak1
Dock111	1.05E-05	0.525115	0.341	0.304	0.256078	Neutrophils	Dock11
Baz1a1	1.28E-05	0.556759	0.26	0.223	0.313952	Neutrophils	Baz1a
Rnf1491	1.54E-05	1.341908	0.257	0.237	0.377426	Neutrophils	Rnf149
Atp5f11	1.55E-05	0.361732	0.497	0.486	0.379519	Neutrophils	Atp5f1
Hcfc1r14	3.37E-05	0.533904	0.3	0.267	0.824569	Neutrophils	Hcfc1r1
Skap21	3.58E-05	0.359753	0.573	0.567	0.876629	Neutrophils	Skap2
Klf63	4.21E-05	0.406549	0.401	0.373	1	Neutrophils	Klf6
Pak21	4.54E-05	0.469341	0.423	0.405	1	Neutrophils	Pak2
Fgfr1op2	4.92E-05	0.579026	0.298	0.268	1	Neutrophils	Fgfr1op2
Asxl11	5.06E-05	0.47375	0.27	0.231	1	Neutrophils	Asxl1
Cbl1	6.22E-05	0.433198	0.396	0.372	1	Neutrophils	Cbl
Srp92	8.12E-05	0.522063	0.385	0.372	1	Neutrophils	Srp9
Dctn32	9.90E-05	0.56237	0.307	0.279	1	Neutrophils	Dctn3
Atf7ip1	0.000177	0.616245	0.304	0.276	1	Neutrophils	Atf7ip

Ndufb1-ps1	0.000228	0.491703	0.324	0.301	1	Neutrophils	Ndufb1-ps
Actr21	0.000271	0.345422	0.506	0.503	1	Neutrophils	Actr2
Hectd11	0.000277	0.689781	0.299	0.277	1	Neutrophils	Hectd1
Rab2a	0.000282	0.301074	0.521	0.524	1	Neutrophils	Rab2a
Fam104a1	0.000335	0.731907	0.261	0.235	1	Neutrophils	Fam104a
Rab241	0.000717	0.518332	0.269	0.242	1	Neutrophils	Rab24
Arhgap41	0.0008	0.408012	0.3	0.27	1	Neutrophils	Arhgap4
Lims11	0.000842	0.357259	0.461	0.467	1	Neutrophils	Lims1
Stk10	0.000972	0.423833	0.32	0.298	1	Neutrophils	Stk10
Arhgap301	0.000977	0.544822	0.345	0.33	1	Neutrophils	Arhgap30
Gdi12	0.001033	0.490387	0.269	0.244	1	Neutrophils	Gdi1
Cd473	0.00124	0.327222	0.461	0.463	1	Neutrophils	Cd47
Ubald22	0.001299	0.748966	0.303	0.29	1	Neutrophils	Ubald2
H2afv1	0.001495	0.750926	0.26	0.248	1	Neutrophils	H2afv
Tomm222	0.00157	0.487711	0.342	0.329	1	Neutrophils	Tomm22
Syf21	0.002304	0.784944	0.258	0.242	1	Neutrophils	Syf2
Osbpl91	0.002373	0.525492	0.366	0.359	1	Neutrophils	Osbpl9
Ndufs53	0.002964	0.423455	0.347	0.335	1	Neutrophils	Ndufs5
Chmp4b4	0.003529	0.394846	0.474	0.468	1	Neutrophils	Chmp4b
Tspan132	0.003683	0.411001	0.287	0.37	1	Neutrophils	Tspan13
Twf22	0.004116	0.312586	0.433	0.43	1	Neutrophils	Twf2
Ost42	0.005741	0.32265	0.501	0.501	1	Neutrophils	Ost4
Ccdc122	0.006252	0.325747	0.454	0.456	1	Neutrophils	Ccdc12
Resf12	0.006289	0.862628	0.266	0.258	1	Neutrophils	Resf1
Ap2s13	0.006698	0.401262	0.313	0.302	1	Neutrophils	Ap2s1
Ube2s3	0.006704	0.762857	0.263	0.257	1	Neutrophils	Ube2s
Vps281	0.008223	0.462844	0.405	0.408	1	Neutrophils	Vps28
Srp142	0.008975	0.378759	0.379	0.369	1	Neutrophils	Srp14
Dennd5a1	0.009534	0.467319	0.294	0.281	1	Neutrophils	Dennd5a
Plac81	0	3.809755	0.681	0.074	0	Nonclassic	Plac8
Cytip1	0	4.097823	0.648	0.068	0	Nonclassic	Cytip
Adgre52	0	4.413724	0.628	0.063	0	Nonclassic	Adgre5
Napsa1	0	3.898679	0.602	0.054	0	Nonclassic	Napsa
Nr4a1	0	4.731672	0.605	0.063	0	Nonclassic	Nr4a1
Ifitm23	0	3.048176	0.732	0.196	0	Nonclassic	Ifitm2
Ifitm61	0	3.216718	0.573	0.037	0	Nonclassic	Ifitm6
Gsr1	0	3.394842	0.655	0.122	0	Nonclassic	Gsr
Itga42	0	3.804916	0.631	0.099	0	Nonclassic	Itga4
S100a62	0	2.256788	0.639	0.108	0	Nonclassic	S100a6
Nedd92	0	4.206651	0.609	0.078	0	Nonclassic	Nedd9
Lsp12	0	2.720153	0.717	0.191	0	Nonclassic	Lsp1
Fgr2	0	3.919209	0.61	0.09	0	Nonclassic	Fgr
Iqgap13	0	2.875948	0.654	0.155	0	Nonclassic	Iqgap1
Flna2	0	3.229145	0.677	0.183	0	Nonclassic	Flna

Msrb12	0	2.940241	0.671	0.18	0	Nonclassic	Msrb1
S100a41	0	4.054429	0.538	0.048	0	Nonclassic	S100a4
Lgals33	0	2.557179	0.663	0.179	0	Nonclassic	Lgals3
Cybb4	0	2.698126	0.703	0.223	0	Nonclassic	Cybb
Fxyd52	0	2.329585	0.759	0.281	0	Nonclassic	Fxyd5
Itgal2	0	4.278309	0.551	0.079	0	Nonclassic	Itgal
Anxa22	0	2.717215	0.575	0.129	0	Nonclassic	Anxa2
Klf24	0	2.256499	0.66	0.216	0	Nonclassic	Klf2
Adgre4	0	7.218089	0.449	0.009	0	Nonclassic	Adgre4
Ace	0	6.016123	0.454	0.015	0	Nonclassic	Ace
Crip11	0	3.06673	0.611	0.173	0	Nonclassic	Crip1
Ldlrad3	0	5.131804	0.461	0.031	0	Nonclassic	Ldlrad3
Gpr1411	0	4.272373	0.466	0.039	0	Nonclassic	Gpr141
Emp33	0	2.496015	0.637	0.211	0	Nonclassic	Emp3
Ahnak2	0	3.271121	0.519	0.097	0	Nonclassic	Ahnak
S100a112	0	1.107341	0.563	0.142	0	Nonclassic	S100a11
Vim2	0	2.439866	0.608	0.188	0	Nonclassic	Vim
Plbd12	0	2.197544	0.53	0.111	0	Nonclassic	Plbd1
Gm361611	0	4.033444	0.464	0.047	0	Nonclassic	Gm36161
Smpdl3a1	0	3.304859	0.548	0.137	0	Nonclassic	Smpdl3a
Cnn22	0	2.349877	0.525	0.114	0	Nonclassic	Cnn2
Emilin21	0	4.421142	0.446	0.037	0	Nonclassic	Emilin2
Gda1	0	3.477453	0.453	0.045	0	Nonclassic	Gda
Metrnl1	0	3.874963	0.484	0.076	0	Nonclassic	Metrnl
Myo1g1	0	3.227829	0.521	0.114	0	Nonclassic	Myo1g
Apoc21	0	4.651007	0.474	0.068	0	Nonclassic	Apoc2
Arhgap153	0	1.856383	0.671	0.27	0	Nonclassic	Arhgap15
Cd442	0	2.603965	0.589	0.19	0	Nonclassic	Cd44
Zfyve9	0	3.695353	0.461	0.07	0	Nonclassic	Zfyve9
Pglyrp11	0	2.504496	0.431	0.04	0	Nonclassic	Pglyrp1
Gm15987	0	5.126451	0.411	0.021	0	Nonclassic	Gm15987
Ifitm34	0	2.63715	0.81	0.422	0	Nonclassic	Ifitm3
Rap1gap21	0	3.475628	0.485	0.098	0	Nonclassic	Rap1gap2
Trps12	0	2.562371	0.544	0.159	0	Nonclassic	Trps1
Gm211881	0	3.926041	0.433	0.049	0	Nonclassic	Gm21188
Hp1	0	3.134463	0.408	0.03	0	Nonclassic	Hp
Plaur3	0	2.577517	0.542	0.166	0	Nonclassic	Plaur
Clec4a12	0	3.175539	0.455	0.082	0	Nonclassic	Clec4a1
Ear2	0	6.425097	0.376	0.011	0	Nonclassic	Ear2
Grk5	0	4.180625	0.403	0.04	0	Nonclassic	Grk5
Stk101	0	2.478814	0.649	0.287	0	Nonclassic	Stk10
Klra21	0	3.079801	0.426	0.067	0	Nonclassic	Klra2
Cyp4f182	0	2.886849	0.447	0.092	0	Nonclassic	Cyp4f18
Rasgrp21	0	4.441368	0.372	0.026	0	Nonclassic	Rasgrp2

Vav33	0	3.122567	0.415	0.071	0	Nonclassic Vav3
Rara	0	4.006869	0.377	0.036	0	Nonclassic Rara
Fam49a1	0	2.814561	0.479	0.139	0	Nonclassic Fam49a
Add31	0	2.490953	0.476	0.136	0	Nonclassic Add3
Spn	0	5.954355	0.35	0.013	0	Nonclassic Spn
Arhgap26	0	3.102399	0.4	0.064	0	Nonclassic Arhgap26
Sirpb1c1	0	4.483057	0.357	0.025	0	Nonclassic Sirpb1c
Xylt11	0	2.644369	0.422	0.09	0	Nonclassic Xylt1
Diaph12	0	2.448145	0.463	0.134	0	Nonclassic Diaph1
Emb2	0	2.839207	0.386	0.066	0	Nonclassic Emb
Treml4	0	6.207473	0.33	0.012	0	Nonclassic Treml4
Klf4	0	2.928811	0.385	0.07	0	Nonclassic Klf4
Cd244a	0	4.170984	0.352	0.04	0	Nonclassic Cd244a
Stap1	0	4.392827	0.343	0.032	0	Nonclassic Stap1
Gm9733	0	5.001263	0.325	0.014	0	Nonclassic Gm9733
Xdh2	0	2.715088	0.406	0.099	0	Nonclassic Xdh
Sirpb1b1	0	3.704165	0.334	0.032	0	Nonclassic Sirpb1b
Hip11	0	2.937726	0.388	0.088	0	Nonclassic Hip1
Tmsb101	0	2.539408	0.927	0.631	0	Nonclassic Tmsb10
Tmem511	0	3.624394	0.357	0.061	0	Nonclassic Tmem51
Ccdc88c	0	4.64409	0.31	0.014	0	Nonclassic Ccdc88c
Unc1191	0	3.273628	0.335	0.049	0	Nonclassic Unc119
Alcam	0	2.671549	0.351	0.065	0	Nonclassic Alcam
Krt80	0	5.327095	0.298	0.015	0	Nonclassic Krt80
Ms4a4a2	0	2.342742	0.358	0.075	0	Nonclassic Ms4a4a
Ly6c22	0	2.441438	0.334	0.052	0	Nonclassic Ly6c2
Ccr21	0	2.745403	0.344	0.062	0	Nonclassic Ccr2
Arhgef37	0	6.510606	0.284	0.006	0	Nonclassic Arhgef37
Atp8b41	0	3.441126	0.314	0.037	0	Nonclassic Atp8b4
Agpat4	0	3.76128	0.322	0.045	0	Nonclassic Agpat4
Plcb1	0	4.345994	0.3	0.024	0	Nonclassic Plcb1
Sirpb1a1	0	4.06012	0.296	0.025	0	Nonclassic Sirpb1a
Cyfip21	0	3.341148	0.314	0.048	0	Nonclassic Cyfip2
Itgb7	0	3.261671	0.3	0.04	0	Nonclassic Itgb7
Dock51	0	3.868608	0.291	0.032	0	Nonclassic Dock5
Cebpb4	0	2.53806	0.791	0.534	0	Nonclassic Cebpb
Mob3b	0	3.124365	0.306	0.054	0	Nonclassic Mob3b
Hopx	0	3.028889	0.298	0.046	0	Nonclassic Hopx
St3gal1	0	3.270215	0.307	0.056	0	Nonclassic St3gal1
Ceacam1	0	3.086621	0.308	0.058	0	Nonclassic Ceacam1
Cd300ld	0	4.372222	0.271	0.021	0	Nonclassic Cd300ld
Gm5150	0	4.309054	0.258	0.012	0	Nonclassic Gm5150
Fn1	0	4.222954	0.285	0.042	0	Nonclassic Fn1
Sik1	0	3.254531	0.293	0.051	0	Nonclassic Sik1

Eno3	0	4.246505	0.254	0.032	0	Nonclassic	Eno3
Cyp4f16	0	3.192994	0.267	0.047	0	Nonclassic	Cyp4f16
Coro1a2	0	1.879182	0.844	0.624	0	Nonclassic	Coro1a
Lyz24	0	2.174841	0.884	0.675	0	Nonclassic	Lyz2
Gpx12	0	2.335836	0.868	0.698	0	Nonclassic	Gpx1
H3f3a3	0	1.788641	0.833	0.67	0	Nonclassic	H3f3a
Fam107b1	6.94E-301	2.287099	0.475	0.143	1.70E-296	Nonclassic	Fam107b
Rasgrp41	1.53E-297	2.61935	0.304	0.061	3.74E-293	Nonclassic	Rasgrp4
Klf133	4.15E-292	2.137277	0.644	0.304	1.02E-287	Nonclassic	Klf13
Mgst13	6.13E-290	2.364806	0.33	0.073	1.50E-285	Nonclassic	Mgst1
Cmss13	3.57E-289	1.260024	0.986	0.922	8.74E-285	Nonclassic	Cmss1
Rras2	2.18E-281	2.785146	0.435	0.135	5.35E-277	Nonclassic	Rras
S100a101	2.74E-274	2.620712	0.37	0.097	6.70E-270	Nonclassic	S100a10
Cdk2ap22	1.04E-273	2.289192	0.633	0.304	2.55E-269	Nonclassic	Cdk2ap2
Calm14	9.28E-273	1.467848	0.84	0.588	2.27E-268	Nonclassic	Calm1
Srgn2	5.19E-270	1.616994	0.801	0.548	1.27E-265	Nonclassic	Srgn
Arhgef10l	9.53E-259	2.694152	0.325	0.079	2.33E-254	Nonclassic	Arhgef10l
Nadk3	1.94E-252	2.260959	0.563	0.242	4.76E-248	Nonclassic	Nadk
Pfn12	2.34E-251	1.074246	0.913	0.83	5.72E-247	Nonclassic	Pfn1
Anxa12	3.33E-250	1.087268	0.281	0.059	8.14E-246	Nonclassic	Anxa1
Mrpl332	1.04E-246	2.125192	0.575	0.259	2.55E-242	Nonclassic	Mrpl33
Plec	6.69E-246	2.73267	0.252	0.051	1.64E-241	Nonclassic	Plec
Ms4a6c2	8.62E-245	1.875994	0.607	0.276	2.11E-240	Nonclassic	Ms4a6c
Dipk1a	9.53E-245	2.551881	0.339	0.088	2.33E-240	Nonclassic	Dipk1a
Samhd13	1.82E-244	1.950815	0.682	0.387	4.45E-240	Nonclassic	Samhd1
Bcl2	5.39E-244	2.966723	0.3	0.072	1.32E-239	Nonclassic	Bcl2
Atp1a32	2.19E-241	2.502803	0.372	0.108	5.37E-237	Nonclassic	Atp1a3
Cblb	7.58E-240	2.381077	0.38	0.11	1.86E-235	Nonclassic	Cblb
Grk3	1.21E-239	2.593116	0.416	0.135	2.96E-235	Nonclassic	Grk3
Ppp2r5a1	1.51E-239	2.183854	0.506	0.194	3.70E-235	Nonclassic	Ppp2r5a
Il17ra1	6.97E-239	2.119843	0.521	0.203	1.71E-234	Nonclassic	Il17ra
Stk17b2	2.96E-235	2.04848	0.469	0.165	7.26E-231	Nonclassic	Stk17b
Actg12	4.98E-228	1.205132	0.889	0.781	1.22E-223	Nonclassic	Actg1
Ptprc3	2.70E-224	1.55595	0.778	0.571	6.61E-220	Nonclassic	Ptprc
Dok32	5.57E-222	1.982725	0.475	0.175	1.36E-217	Nonclassic	Dok3
Arhgdib2	3.49E-221	1.262582	0.803	0.616	8.56E-217	Nonclassic	Arhgdib
Prr132	6.20E-221	1.942015	0.62	0.328	1.52E-216	Nonclassic	Prr13
Pla2g74	3.02E-220	1.84185	0.449	0.154	7.40E-216	Nonclassic	Pla2g7
Clec12a2	5.41E-219	2.098266	0.309	0.078	1.33E-214	Nonclassic	Clec12a
Rps27a3	5.94E-215	0.800054	0.955	0.907	1.46E-210	Nonclassic	Rps27a
Sep-92	1.50E-211	2.328287	0.401	0.136	3.68E-207	Nonclassic	9-Sep
Myl63	1.68E-211	1.237129	0.806	0.605	4.12E-207	Nonclassic	Myl6
Svil1	3.80E-209	1.948533	0.496	0.198	9.31E-205	Nonclassic	Svil
Rpl84	4.46E-204	0.906382	0.904	0.793	1.09E-199	Nonclassic	Rpl8

Sh3bgrl2	1.75E-203	2.141989	0.389	0.129	4.29E-199	Nonclassic	Sh3bgrl
Rps163	1.55E-201	0.771335	0.951	0.88	3.80E-197	Nonclassic	Rps16
Rpsa3	3.00E-200	0.831111	0.94	0.832	7.34E-196	Nonclassic	Rpsa
Gngt21	4.05E-200	2.360769	0.668	0.458	9.92E-196	Nonclassic	Gngt2
Rbms13	9.18E-200	1.870134	0.517	0.223	2.25E-195	Nonclassic	Rbms1
Msn2	3.76E-199	1.422298	0.773	0.589	9.20E-195	Nonclassic	Msn
Arpc22	4.90E-199	1.160254	0.818	0.707	1.20E-194	Nonclassic	Arpc2
Zyx2	7.20E-192	1.760959	0.539	0.25	1.76E-187	Nonclassic	Zyx
Arpc1b4	1.14E-190	1.114833	0.82	0.702	2.79E-186	Nonclassic	Arpc1b
St3gal41	9.75E-190	2.067561	0.367	0.12	2.39E-185	Nonclassic	St3gal4
Cdc42ep31	4.16E-189	2.298664	0.259	0.065	1.02E-184	Nonclassic	Cdc42ep3
Sem13	1.65E-188	1.352721	0.75	0.548	4.03E-184	Nonclassic	Sem1
Tpm42	2.21E-187	2.040963	0.424	0.16	5.42E-183	Nonclassic	Tpm4
Ucp23	8.20E-187	1.12343	0.831	0.727	2.01E-182	Nonclassic	Ucp2
Ccl61	6.87E-186	1.84541	0.651	0.402	1.68E-181	Nonclassic	Ccl6
Pot1b	5.54E-185	2.274416	0.403	0.147	1.36E-180	Nonclassic	Pot1b
Rps144	1.05E-181	0.819453	0.911	0.809	2.56E-177	Nonclassic	Rps14
Lgals12	1.13E-180	1.51472	0.436	0.158	2.78E-176	Nonclassic	Lgals1
Rps193	4.27E-179	0.870626	0.908	0.799	1.04E-174	Nonclassic	Rps19
Ywhaz1	6.59E-175	1.296068	0.753	0.556	1.61E-170	Nonclassic	Ywhaz
Pygl1	2.92E-174	1.92343	0.274	0.075	7.15E-170	Nonclassic	Pygl
Lars23	2.97E-174	1.079692	0.921	0.752	7.27E-170	Nonclassic	Lars2
Gm424183	1.75E-173	0.942266	1	0.982	4.29E-169	Nonclassic	Gm42418
Actr32	4.61E-171	1.198451	0.749	0.553	1.13E-166	Nonclassic	Actr3
Rap1b	2.68E-170	1.270821	0.807	0.703	6.56E-166	Nonclassic	Rap1b
Cers62	3.54E-170	1.92862	0.468	0.201	8.68E-166	Nonclassic	Cers6
B4galt5	3.20E-169	2.354005	0.279	0.08	7.83E-165	Nonclassic	B4galt5
Myl12b2	1.37E-164	1.150089	0.78	0.632	3.35E-160	Nonclassic	Myl12b
Ifi27l2a4	1.60E-163	1.809109	0.634	0.38	3.93E-159	Nonclassic	Ifi27l2a
Tln13	1.41E-161	1.438134	0.688	0.474	3.46E-157	Nonclassic	Tln1
Filip1l4	9.36E-160	1.452895	0.71	0.475	2.29E-155	Nonclassic	Filip1l
Rbpms	9.89E-160	2.233994	0.367	0.137	2.42E-155	Nonclassic	Rbpms
Stk241	4.92E-159	1.845962	0.47	0.21	1.20E-154	Nonclassic	Stk24
Sh3bgrl34	9.32E-158	0.915921	0.85	0.747	2.28E-153	Nonclassic	Sh3bgrl3
Tnfrsf1b2	1.35E-157	1.745264	0.596	0.348	3.31E-153	Nonclassic	Tnfrsf1b
Fmnl11	2.52E-156	1.575819	0.589	0.338	6.17E-152	Nonclassic	Fmnl1
Lilrb4a4	3.76E-155	1.545488	0.418	0.163	9.21E-151	Nonclassic	Lilrb4a
Slfn24	3.43E-154	1.530808	0.553	0.29	8.40E-150	Nonclassic	Slfn2
Ezr2	4.74E-154	2.11479	0.292	0.091	1.16E-149	Nonclassic	Ezr
Rps3a14	1.23E-153	0.642188	0.951	0.882	3.02E-149	Nonclassic	Rps3a1
Ptpre3	2.54E-153	1.748443	0.593	0.338	6.21E-149	Nonclassic	Ptpre
H2afj3	4.36E-153	1.494493	0.624	0.383	1.07E-148	Nonclassic	H2afj
Hmgb21	1.06E-152	1.597175	0.484	0.23	2.60E-148	Nonclassic	Hmgb2
Rpl183	1.09E-151	0.735257	0.911	0.838	2.67E-147	Nonclassic	Rpl18

Plin23	2.81E-151	1.845922	0.467	0.218	6.89E-147	Nonclassic	Plin2
Ptpn121	2.43E-150	2.102369	0.296	0.095	5.94E-146	Nonclassic	Ptpn12
Rpl93	1.04E-149	0.652524	0.936	0.875	2.56E-145	Nonclassic	Rpl9
Rplp03	1.35E-147	0.760159	0.921	0.841	3.31E-143	Nonclassic	Rplp0
Rps244	1.58E-146	0.584645	0.968	0.928	3.87E-142	Nonclassic	Rps24
Pirb3	3.07E-146	1.907122	0.376	0.149	7.53E-142	Nonclassic	Pirb
Mbp4	3.85E-146	1.155456	0.605	0.311	9.42E-142	Nonclassic	Mbp
Clec4a33	1.51E-144	1.754161	0.529	0.283	3.69E-140	Nonclassic	Clec4a3
Eif13	2.03E-144	0.752831	0.886	0.833	4.97E-140	Nonclassic	Eif1
Stk381	1.04E-143	1.80991	0.464	0.219	2.56E-139	Nonclassic	Stk38
Taldo13	1.97E-142	1.336139	0.662	0.479	4.83E-138	Nonclassic	Taldo1
Bri3bp	1.17E-140	2.116195	0.332	0.123	2.86E-136	Nonclassic	Bri3bp
Mndal2	1.07E-139	1.808844	0.27	0.083	2.63E-135	Nonclassic	Mndal
Rps53	7.67E-138	0.648895	0.934	0.851	1.88E-133	Nonclassic	Rps5
Serf24	9.72E-138	0.847317	0.849	0.759	2.38E-133	Nonclassic	Serf2
Fam129a2	1.05E-137	1.708354	0.378	0.15	2.56E-133	Nonclassic	Fam129a
Alox5ap3	1.54E-137	1.285807	0.744	0.648	3.77E-133	Nonclassic	Alox5ap
Tpt14	8.56E-137	0.607347	0.955	0.896	2.10E-132	Nonclassic	Tpt1
Fau3	1.80E-135	0.581186	0.976	0.939	4.40E-131	Nonclassic	Fau
Il31ra3	1.99E-135	1.104253	0.817	0.611	4.86E-131	Nonclassic	Il31ra
Jarid23	1.63E-134	1.523103	0.572	0.329	3.98E-130	Nonclassic	Jarid2
Prdx53	2.15E-134	1.11175	0.74	0.587	5.27E-130	Nonclassic	Prdx5
Wfdc172	2.93E-133	1.279032	0.321	0.114	7.17E-129	Nonclassic	Wfdc17
Fyn	7.17E-133	2.281374	0.306	0.111	1.76E-128	Nonclassic	Fyn
Neat13	8.63E-133	1.108398	0.554	0.292	2.11E-128	Nonclassic	Neat1
Nin2	1.99E-130	1.803112	0.346	0.135	4.88E-126	Nonclassic	Nin
AY0361183	5.60E-130	1.171748	0.912	0.804	1.37E-125	Nonclassic	AY036118
Rap1a1	7.30E-130	1.277064	0.7	0.532	1.79E-125	Nonclassic	Rap1a
Lst14	9.21E-129	1.490341	0.713	0.534	2.25E-124	Nonclassic	Lst1
Utrn	1.48E-128	1.74906	0.329	0.123	3.62E-124	Nonclassic	Utrn
Ikbkb1	7.48E-128	1.64425	0.468	0.233	1.83E-123	Nonclassic	Ikbkb
F13a11	9.90E-128	1.376233	0.275	0.088	2.42E-123	Nonclassic	F13a1
AB1246115	1.12E-126	1.693886	0.379	0.162	2.74E-122	Nonclassic	AB124611
Cd524	1.23E-126	0.814749	0.833	0.671	3.02E-122	Nonclassic	Cd52
Nab11	7.24E-126	1.977379	0.327	0.128	1.77E-121	Nonclassic	Nab1
Rps133	2.25E-125	0.631628	0.915	0.848	5.51E-121	Nonclassic	Rps13
Sorl11	2.85E-125	1.567362	0.402	0.177	6.98E-121	Nonclassic	Sorl1
Oaz14	1.06E-124	0.784405	0.834	0.72	2.59E-120	Nonclassic	Oaz1
Actb3	6.89E-124	0.509999	0.98	0.958	1.69E-119	Nonclassic	Actb
Cdk84	1.43E-121	0.814937	0.959	0.901	3.49E-117	Nonclassic	Cdk8
Gm199513	2.31E-121	1.332075	0.596	0.361	5.65E-117	Nonclassic	Gm19951
Camkk2	4.56E-120	1.67142	0.349	0.142	1.12E-115	Nonclassic	Camkk2
Glud11	6.44E-120	1.456772	0.554	0.338	1.58E-115	Nonclassic	Glud1
Nupr12	6.55E-118	1.679043	0.396	0.179	1.60E-113	Nonclassic	Nupr1

St8sia42	7.10E-118	1.783754	0.277	0.097	1.74E-113	Nonclassic	St8sia4
Ccnd32	2.77E-117	0.849481	0.739	0.519	6.79E-113	Nonclassic	Ccnd3
Tpd522	1.71E-116	1.298828	0.612	0.425	4.18E-112	Nonclassic	Tpd52
Ms4a4c2	2.46E-116	1.632235	0.293	0.108	6.03E-112	Nonclassic	Ms4a4c
Rps73	4.10E-116	0.586883	0.937	0.866	1.00E-111	Nonclassic	Rps7
Tagln21	1.20E-115	1.888921	0.42	0.21	2.93E-111	Nonclassic	Tagln2
Rpl133	5.92E-115	0.51489	0.966	0.919	1.45E-110	Nonclassic	Rpl13
Tgfbi3	3.62E-114	1.410093	0.418	0.199	8.85E-110	Nonclassic	Tgfbi
Foxp11	4.74E-114	1.126841	0.679	0.459	1.16E-109	Nonclassic	Foxp1
Slk1	1.13E-113	1.599529	0.363	0.157	2.77E-109	Nonclassic	Slk
Elmo2	6.13E-113	1.841563	0.304	0.118	1.50E-108	Nonclassic	Elmo2
Ap1s22	1.29E-112	1.689314	0.368	0.163	3.15E-108	Nonclassic	Ap1s2
Pilra2	6.21E-111	2.032699	0.306	0.123	1.52E-106	Nonclassic	Pilra
Cd300a1	2.16E-110	1.352931	0.622	0.436	5.28E-106	Nonclassic	Cd300a
Atp5h3	6.60E-110	0.956894	0.721	0.55	1.62E-105	Nonclassic	Atp5h
Arpc53	8.42E-110	1.242042	0.615	0.427	2.06E-105	Nonclassic	Arpc5
Cd474	4.41E-108	1.162769	0.64	0.457	1.08E-103	Nonclassic	Cd47
Prkch	4.64E-108	1.5722	0.428	0.212	1.14E-103	Nonclassic	Prkch
Dbi3	4.72E-108	1.104192	0.635	0.432	1.16E-103	Nonclassic	Dbi
Samsn13	7.64E-108	1.4772	0.462	0.241	1.87E-103	Nonclassic	Samsn1
Anxa53	7.74E-107	1.315801	0.517	0.301	1.90E-102	Nonclassic	Anxa5
Sgms11	1.44E-105	1.485214	0.395	0.182	3.52E-101	Nonclassic	Sgms1
Fis14	5.10E-104	1.066727	0.65	0.466	1.25E-99	Nonclassic	Fis1
Rpl194	1.94E-103	0.493165	0.95	0.889	4.76E-99	Nonclassic	Rpl19
Ddit4	5.42E-103	1.932957	0.299	0.12	1.33E-98	Nonclassic	Ddit4
Gstm11	7.43E-103	1.564866	0.298	0.117	1.82E-98	Nonclassic	Gstm1
Ncf21	1.28E-102	1.158156	0.631	0.465	3.13E-98	Nonclassic	Ncf2
Rps84	2.73E-102	0.521901	0.951	0.9	6.68E-98	Nonclassic	Rps8
Ppdpf1	1.09E-101	1.71945	0.352	0.161	2.66E-97	Nonclassic	Ppdpf
Bcl6	1.87E-101	1.854262	0.336	0.149	4.57E-97	Nonclassic	Bcl6
Rac23	2.59E-101	1.149767	0.631	0.454	6.35E-97	Nonclassic	Rac2
Psma73	6.42E-101	1.202436	0.595	0.41	1.57E-96	Nonclassic	Psma7
Pde4b2	1.91E-100	1.484654	0.443	0.229	4.68E-96	Nonclassic	Pde4b
Pitpna2	1.87E-98	1.088982	0.647	0.479	4.58E-94	Nonclassic	Pitpna
Ccl91	2.56E-98	1.805923	0.332	0.145	6.27E-94	Nonclassic	Ccl9
Pim14	3.75E-98	1.667411	0.328	0.143	9.19E-94	Nonclassic	Pim1
Rps34	9.45E-95	0.538005	0.905	0.828	2.32E-90	Nonclassic	Rps3
Dusp16	1.62E-94	2.446841	0.326	0.154	3.96E-90	Nonclassic	Dusp16
Rpl343	2.24E-94	0.591571	0.904	0.843	5.49E-90	Nonclassic	Rpl34
Rpl274	4.58E-94	0.623722	0.86	0.776	1.12E-89	Nonclassic	Rpl27
Atp5l4	1.10E-93	0.919879	0.715	0.566	2.70E-89	Nonclassic	Atp5l
Siva11	1.61E-93	1.843779	0.264	0.104	3.95E-89	Nonclassic	Siva1
Rhoa2	4.37E-93	0.772362	0.773	0.688	1.07E-88	Nonclassic	Rhoa
Gmfg3	1.73E-92	1.175013	0.573	0.383	4.23E-88	Nonclassic	Gmfg

Rnase62	2.32E-92	1.478791	0.376	0.181	5.69E-88	Nonclassic	Rnase6
B4galnt11	3.23E-92	1.875984	0.348	0.169	7.90E-88	Nonclassic	B4galnt1
Rpl73	6.82E-91	0.628894	0.833	0.734	1.67E-86	Nonclassic	Rpl7
Tmsb4x3	6.95E-91	0.415176	0.981	0.967	1.70E-86	Nonclassic	Tmsb4x
Herc42	9.80E-91	1.496909	0.456	0.257	2.40E-86	Nonclassic	Herc4
Snrpb3	4.99E-90	1.16337	0.579	0.4	1.22E-85	Nonclassic	Snrpb
Eif3f3	5.20E-90	0.856134	0.731	0.588	1.27E-85	Nonclassic	Eif3f
Rasa31	8.44E-89	1.358925	0.463	0.264	2.07E-84	Nonclassic	Rasa3
Eno14	8.19E-88	1.286703	0.489	0.296	2.00E-83	Nonclassic	Eno1
Ncf42	4.14E-87	1.396067	0.444	0.253	1.01E-82	Nonclassic	Ncf4
Cast2	4.81E-87	1.74134	0.273	0.113	1.18E-82	Nonclassic	Cast
Rps103	5.40E-87	0.470478	0.937	0.891	1.32E-82	Nonclassic	Rps10
Tmpo2	3.33E-86	1.630769	0.339	0.164	8.15E-82	Nonclassic	Tmpo
Fyb1	3.46E-86	0.82849	0.813	0.725	8.47E-82	Nonclassic	Fyb
Ext11	9.17E-86	1.219942	0.422	0.22	2.24E-81	Nonclassic	Ext1
Myh92	1.71E-85	0.896209	0.726	0.584	4.18E-81	Nonclassic	Myh9
Myl12a3	1.73E-85	1.05487	0.62	0.451	4.24E-81	Nonclassic	Myl12a
Smc6	1.31E-84	1.949569	0.317	0.15	3.20E-80	Nonclassic	Smc6
Ptma2	2.00E-84	0.72764	0.834	0.726	4.90E-80	Nonclassic	Ptma
Cbfa2t3	2.55E-84	1.450524	0.325	0.148	6.24E-80	Nonclassic	Cbfa2t3
Gphn3	2.70E-84	0.963764	0.789	0.638	6.61E-80	Nonclassic	Gphn
Arhgef12	3.72E-84	1.381085	0.472	0.278	9.11E-80	Nonclassic	Arhgef1
Rps15a4	1.54E-83	0.52287	0.926	0.856	3.77E-79	Nonclassic	Rps15a
Mcl13	1.78E-83	0.911029	0.673	0.539	4.36E-79	Nonclassic	Mcl1
Cfl14	2.30E-83	0.591025	0.837	0.764	5.62E-79	Nonclassic	Cfl1
Dusp15	2.35E-83	0.908366	0.599	0.381	5.75E-79	Nonclassic	Dusp1
Capzb	4.30E-83	0.721326	0.765	0.686	1.05E-78	Nonclassic	Capzb
Taf103	1.01E-82	1.197987	0.551	0.364	2.47E-78	Nonclassic	Taf10
Rack13	1.40E-82	0.693352	0.784	0.656	3.44E-78	Nonclassic	Rack1
Rpl36al3	1.80E-82	0.744021	0.759	0.647	4.40E-78	Nonclassic	Rpl36al
Ccdc88a1	2.68E-82	1.597851	0.353	0.175	6.55E-78	Nonclassic	Ccdc88a
Rpl173	3.42E-82	0.506998	0.911	0.839	8.38E-78	Nonclassic	Rpl17
Pid13	3.96E-82	0.987334	0.607	0.411	9.71E-78	Nonclassic	Pid1
Sec61b3	5.10E-82	1.100985	0.609	0.462	1.25E-77	Nonclassic	Sec61b
Scand13	1.65E-81	1.086289	0.587	0.414	4.04E-77	Nonclassic	Scand1
Sp1003	1.45E-80	1.271643	0.459	0.274	3.55E-76	Nonclassic	Sp100
Sap302	3.50E-80	1.718534	0.281	0.125	8.58E-76	Nonclassic	Sap30
Atp5e3	7.57E-80	0.774961	0.762	0.65	1.85E-75	Nonclassic	Atp5e
Rpl283	2.08E-79	0.532299	0.906	0.841	5.11E-75	Nonclassic	Rpl28
Atp1a11	2.87E-79	1.275118	0.524	0.348	7.04E-75	Nonclassic	Atp1a1
Fgd41	2.92E-79	1.515776	0.373	0.191	7.16E-75	Nonclassic	Fgd4
Atox12	3.84E-77	0.763421	0.729	0.592	9.39E-73	Nonclassic	Atox1
Nfam13	6.62E-76	1.202	0.525	0.349	1.62E-71	Nonclassic	Nfam1
Dynll13	9.59E-76	1.066754	0.586	0.417	2.35E-71	Nonclassic	Dynll1

Zc3h7a3	3.21E-75	1.145177	0.647	0.489	7.87E-71	Nonclassic Zc3h7a
Rps93	6.08E-75	0.440619	0.955	0.911	1.49E-70	Nonclassic Rps9
Ciao2a2	4.02E-74	1.356129	0.418	0.248	9.85E-70	Nonclassic Ciao2a
Mapkapk22	4.86E-74	1.167165	0.5	0.327	1.19E-69	Nonclassic Mapkapk2
Gstp11	7.03E-74	1.319788	0.464	0.286	1.72E-69	Nonclassic Gstp1
Arf52	9.31E-74	0.926393	0.626	0.48	2.28E-69	Nonclassic Arf5
Camk1d3	4.80E-73	0.544324	0.905	0.806	1.18E-68	Nonclassic Camk1d
Rpl18a3	6.14E-71	0.398526	0.949	0.9	1.50E-66	Nonclassic Rpl18a
Cmip2	2.81E-70	0.977998	0.559	0.38	6.89E-66	Nonclassic Cmip
Arpc34	5.08E-70	0.681359	0.732	0.64	1.24E-65	Nonclassic Arpc3
Eif3k2	6.94E-70	0.911172	0.628	0.484	1.70E-65	Nonclassic Eif3k
Tkt3	7.69E-70	1.059062	0.567	0.423	1.88E-65	Nonclassic Tkt
Rpl243	1.25E-69	0.512272	0.861	0.789	3.06E-65	Nonclassic Rpl24
Aldh24	1.87E-69	1.007066	0.447	0.269	4.59E-65	Nonclassic Aldh2
Ostf12	3.43E-69	0.878989	0.631	0.493	8.41E-65	Nonclassic Ostf1
Rps114	4.37E-69	0.418497	0.948	0.895	1.07E-64	Nonclassic Rps11
Scarb1	4.41E-69	1.573583	0.279	0.132	1.08E-64	Nonclassic Scarb1
Rpl37a4	7.20E-69	0.55568	0.917	0.853	1.76E-64	Nonclassic Rpl37a
Rpl35a4	1.63E-68	0.473781	0.932	0.885	3.99E-64	Nonclassic Rpl35a
Rps4x3	2.65E-68	0.400775	0.937	0.879	6.48E-64	Nonclassic Rps4x
Prdx63	2.74E-68	1.293791	0.359	0.195	6.70E-64	Nonclassic Prdx6
Tmcc12	3.84E-68	1.104323	0.531	0.356	9.40E-64	Nonclassic Tmcc1
Hspa83	3.90E-68	0.584743	0.828	0.741	9.54E-64	Nonclassic Hspa8
H2afz2	3.95E-68	0.903512	0.678	0.573	9.67E-64	Nonclassic H2afz
Ptk2b1	5.98E-68	1.180871	0.517	0.349	1.46E-63	Nonclassic Ptk2b
Plekho23	1.57E-67	1.297639	0.37	0.203	3.85E-63	Nonclassic Plekho2
Mtpn2	1.85E-67	1.282853	0.394	0.225	4.53E-63	Nonclassic Mtpn
Arhgap92	3.49E-67	1.398925	0.334	0.175	8.55E-63	Nonclassic Arhgap9
Fry1	1.92E-66	1.203976	0.344	0.175	4.70E-62	Nonclassic Fry
Rps183	1.61E-65	0.514266	0.854	0.76	3.94E-61	Nonclassic Rps18
Ier23	4.36E-65	0.513028	0.574	0.35	1.07E-60	Nonclassic Ier2
Syk2	4.94E-65	1.10071	0.515	0.347	1.21E-60	Nonclassic Syk
Rassf5	8.29E-65	1.450357	0.315	0.161	2.03E-60	Nonclassic Rassf5
Rps233	2.81E-64	0.457047	0.917	0.854	6.87E-60	Nonclassic Rps23
Selenow5	3.01E-64	0.837692	0.633	0.459	7.37E-60	Nonclassic Selenow
Grk21	1.00E-63	1.036885	0.508	0.342	2.46E-59	Nonclassic Grk2
Tomm64	1.13E-63	0.787785	0.666	0.531	2.77E-59	Nonclassic Tomm6
Cox4i15	1.29E-63	0.470887	0.877	0.803	3.17E-59	Nonclassic Cox4i1
Txn13	3.22E-63	0.659198	0.506	0.322	7.88E-59	Nonclassic Txn1
Ttr4	1.31E-62	0.812214	0.684	0.506	3.21E-58	Nonclassic Ttr
Nav1	3.10E-62	1.365927	0.283	0.137	7.59E-58	Nonclassic Nav1
Ppp1ca2	1.70E-61	0.913206	0.586	0.463	4.17E-57	Nonclassic Ppp1ca
Btf32	2.40E-61	0.70201	0.704	0.613	5.88E-57	Nonclassic Btf3
Spi12	3.44E-61	0.733208	0.746	0.706	8.43E-57	Nonclassic Spi1

Hnrnpa31	3.95E-61	0.959486	0.588	0.453	9.68E-57	Nonclassic	Hnrnpa3
Lcp12	1.14E-60	0.671704	0.774	0.688	2.79E-56	Nonclassic	Lcp1
Gnb22	1.62E-60	0.758242	0.673	0.583	3.97E-56	Nonclassic	Gnb2
Eif4ebp13	1.07E-59	1.211768	0.362	0.206	2.62E-55	Nonclassic	Eif4ebp1
Sptan1	4.10E-59	1.35385	0.305	0.16	1.00E-54	Nonclassic	Sptan1
Rps215	1.22E-58	0.418737	0.917	0.815	2.98E-54	Nonclassic	Rps2
Ybx13	1.25E-58	0.702664	0.697	0.571	3.06E-54	Nonclassic	Ybx1
Lrrfip11	1.43E-58	1.118731	0.468	0.31	3.50E-54	Nonclassic	Lrrfip1
Prkcd2	1.43E-58	0.856665	0.635	0.525	3.51E-54	Nonclassic	Prkcd
Gm267402	1.85E-58	1.062752	0.467	0.303	4.54E-54	Nonclassic	Gm26740
Ifi2073	3.07E-58	1.021527	0.33	0.175	7.51E-54	Nonclassic	Ifi207
Rpl113	3.26E-58	0.370993	0.928	0.876	7.98E-54	Nonclassic	Rpl11
Igsf63	3.32E-58	1.271232	0.377	0.225	8.12E-54	Nonclassic	Igsf6
Rps63	1.05E-57	0.583873	0.778	0.687	2.57E-53	Nonclassic	Rps6
Gm2a3	2.67E-57	0.92658	0.571	0.438	6.53E-53	Nonclassic	Gm2a
Cdc42se12	4.78E-57	1.137275	0.433	0.28	1.17E-52	Nonclassic	Cdc42se1
Adap11	1.35E-56	1.533042	0.279	0.144	3.31E-52	Nonclassic	Adap1
Gpcpd11	2.07E-56	1.281332	0.405	0.249	5.06E-52	Nonclassic	Gpcpd1
Lmo42	1.20E-55	1.502085	0.286	0.15	2.93E-51	Nonclassic	Lmo4
Ndufa63	1.54E-55	0.789248	0.645	0.52	3.77E-51	Nonclassic	Ndufa6
Rpl234	1.84E-55	0.359584	0.936	0.882	4.52E-51	Nonclassic	Rpl23
Malt1	3.47E-55	1.416597	0.275	0.139	8.49E-51	Nonclassic	Malt1
Scp24	4.87E-55	0.996905	0.485	0.337	1.19E-50	Nonclassic	Scp2
Sat12	5.67E-55	0.875494	0.774	0.73	1.39E-50	Nonclassic	Sat1
H2afy1	7.53E-55	1.077256	0.516	0.376	1.84E-50	Nonclassic	H2afy
Rpl104	1.26E-54	0.442109	0.889	0.84	3.08E-50	Nonclassic	Rpl10
Uqcrh4	1.35E-54	0.636708	0.708	0.598	3.30E-50	Nonclassic	Uqcrh
Ehbp1l11	1.73E-54	1.256956	0.342	0.198	4.25E-50	Nonclassic	Ehbp1l1
Rps273	3.12E-54	0.613778	0.844	0.782	7.65E-50	Nonclassic	Rps27
Ttc7	4.88E-54	1.097988	0.352	0.2	1.20E-49	Nonclassic	Ttc7
Esyt11	7.88E-54	1.556099	0.264	0.134	1.93E-49	Nonclassic	Esyt1
Fam111a1	1.04E-53	1.297165	0.276	0.142	2.54E-49	Nonclassic	Fam111a
Myo1f2	1.16E-53	0.729643	0.71	0.627	2.84E-49	Nonclassic	Myo1f
Cox5a3	2.62E-53	0.763973	0.607	0.461	6.41E-49	Nonclassic	Cox5a
Rpl324	3.37E-53	0.38913	0.936	0.865	8.24E-49	Nonclassic	Rpl32
Capg3	4.47E-53	1.4635	0.271	0.141	1.09E-48	Nonclassic	Capg
Ccdc123	1.75E-52	0.853536	0.576	0.452	4.28E-48	Nonclassic	Ccdc12
Smdt13	2.14E-52	0.919437	0.553	0.417	5.23E-48	Nonclassic	Smdt1
mt-Co12	3.76E-52	0.388236	0.987	0.962	9.21E-48	Nonclassic	mt-Co1
Sub11	4.68E-52	0.811417	0.604	0.489	1.15E-47	Nonclassic	Sub1
Pkm3	6.93E-52	0.660338	0.628	0.505	1.70E-47	Nonclassic	Pkm
Abracl2	7.00E-51	1.108734	0.431	0.289	1.71E-46	Nonclassic	Abracl
Hint13	1.23E-50	0.695646	0.642	0.527	3.02E-46	Nonclassic	Hint1
Btg13	1.39E-50	0.754072	0.651	0.528	3.41E-46	Nonclassic	Btg1

Nfkbiz4	3.90E-50	0.955197	0.413	0.254	9.55E-46	Nonclassic Nfkbiz
Cdkn1b	5.05E-50	1.392149	0.262	0.135	1.24E-45	Nonclassic Cdkn1b
Eif3h3	5.51E-50	0.7776	0.601	0.487	1.35E-45	Nonclassic Eif3h
Rbm32	2.37E-49	0.736006	0.639	0.536	5.80E-45	Nonclassic Rbm3
Sptbn1	2.96E-49	1.197738	0.259	0.132	7.26E-45	Nonclassic Sptbn1
Gng54	4.72E-49	0.518346	0.795	0.72	1.15E-44	Nonclassic Gng5
Ppia3	4.99E-49	0.367095	0.941	0.88	1.22E-44	Nonclassic Ppia
Cmpk12	7.20E-49	1.07897	0.409	0.27	1.76E-44	Nonclassic Cmpk1
Aprt3	7.95E-49	1.329813	0.401	0.272	1.95E-44	Nonclassic Aprt
Slc44a21	1.95E-48	1.121078	0.362	0.216	4.77E-44	Nonclassic Slc44a2
Rnf1492	3.29E-48	1.320097	0.371	0.234	8.07E-44	Nonclassic Rnf149
Dock21	3.87E-48	0.455072	0.786	0.69	9.49E-44	Nonclassic Dock2
Ppp1cb1	1.01E-47	1.229918	0.319	0.183	2.48E-43	Nonclassic Ppp1cb
Ost43	1.96E-47	0.763631	0.618	0.498	4.79E-43	Nonclassic Ost4
Nme25	2.61E-47	0.596112	0.702	0.601	6.39E-43	Nonclassic Nme2
S100a133	3.45E-47	1.181253	0.363	0.225	8.46E-43	Nonclassic S100a13
Srp93	3.87E-47	0.944127	0.5	0.368	9.48E-43	Nonclassic Srp9
Enpp23	5.15E-47	0.845573	0.379	0.227	1.26E-42	Nonclassic Enpp2
Arl5c3	5.45E-47	1.370927	0.258	0.137	1.34E-42	Nonclassic Arl5c
Kpna41	1.16E-46	1.001287	0.572	0.451	2.83E-42	Nonclassic Kpna4
Rassf31	1.33E-46	1.152861	0.318	0.184	3.26E-42	Nonclassic Rassf3
Itgb22	3.52E-46	0.746129	0.67	0.587	8.61E-42	Nonclassic Itgb2
Map3k52	3.90E-46	0.893041	0.412	0.26	9.54E-42	Nonclassic Map3k5
Eif5a3	9.08E-46	0.771847	0.609	0.491	2.22E-41	Nonclassic Eif5a
Anp32b2	1.11E-45	0.985658	0.427	0.287	2.72E-41	Nonclassic Anp32b
Naca4	1.42E-45	0.514177	0.763	0.704	3.47E-41	Nonclassic Naca
Kdm7a2	1.93E-45	1.078114	0.396	0.257	4.72E-41	Nonclassic Kdm7a
Rpl153	2.09E-45	0.40241	0.86	0.786	5.12E-41	Nonclassic Rpl15
D8Ertd738e	6.47E-45	0.778295	0.562	0.443	1.58E-40	Nonclassic D8Ertd738e
BC0055372	6.51E-45	1.124452	0.318	0.184	1.60E-40	Nonclassic BC005537
Rpl263	8.91E-45	0.362096	0.895	0.828	2.18E-40	Nonclassic Rpl26
Uqcrfs13	1.36E-44	0.936097	0.452	0.317	3.32E-40	Nonclassic Uqcrfs1
Shisa52	3.36E-44	0.857065	0.561	0.459	8.22E-40	Nonclassic Shisa5
Cox6c4	3.84E-44	0.66108	0.683	0.578	9.41E-40	Nonclassic Cox6c
Serp13	4.14E-44	0.999251	0.48	0.361	1.01E-39	Nonclassic Serp1
Tsc22d34	1.29E-43	0.823312	0.447	0.297	3.16E-39	Nonclassic Tsc22d3
Rplp23	1.51E-43	0.443786	0.867	0.801	3.70E-39	Nonclassic Rplp2
Gm480994	1.91E-43	1.098654	0.405	0.272	4.67E-39	Nonclassic Gm48099
Gm155644	5.53E-43	1.183061	0.365	0.229	1.35E-38	Nonclassic Gm15564
Rab8a2	5.80E-43	1.214889	0.329	0.203	1.42E-38	Nonclassic Rab8a
Tmem1642	7.42E-43	0.856181	0.426	0.283	1.82E-38	Nonclassic Tmem164
Sgk3	1.08E-42	1.041261	0.353	0.215	2.64E-38	Nonclassic Sgk3
Ube2d32	1.33E-42	0.68769	0.627	0.527	3.26E-38	Nonclassic Ube2d3
Gapdh3	4.17E-42	0.496722	0.723	0.628	1.02E-37	Nonclassic Gapdh

Trim253	4.80E-42	1.447079	0.288	0.169	1.18E-37	Nonclassic	Trim25
Tma71	1.20E-41	0.857938	0.527	0.406	2.95E-37	Nonclassic	Tma7
Sf3b61	1.69E-41	0.927728	0.45	0.319	4.14E-37	Nonclassic	Sf3b6
Mknk2	1.70E-41	1.36706	0.252	0.138	4.16E-37	Nonclassic	Mknk2
Rpl364	2.32E-41	0.450868	0.862	0.793	5.67E-37	Nonclassic	Rpl36
Tle53	5.96E-41	0.801797	0.527	0.404	1.46E-36	Nonclassic	Tle5
Notch21	7.66E-41	0.929032	0.46	0.325	1.88E-36	Nonclassic	Notch2
Pabpc13	7.70E-41	0.545632	0.697	0.623	1.88E-36	Nonclassic	Pabpc1
Anp32a3	8.74E-41	0.964873	0.471	0.349	2.14E-36	Nonclassic	Anp32a
Pou2f22	1.28E-40	0.905768	0.616	0.526	3.14E-36	Nonclassic	Pou2f2
Gyg2	1.33E-40	1.135604	0.268	0.152	3.27E-36	Nonclassic	Gyg
Gpx44	1.61E-40	0.607874	0.685	0.596	3.94E-36	Nonclassic	Gpx4
Rpl63	2.35E-40	0.342362	0.885	0.828	5.75E-36	Nonclassic	Rpl6
Arhgap302	3.02E-40	0.85576	0.455	0.327	7.41E-36	Nonclassic	Arhgap30
Cdk141	3.49E-40	1.139517	0.367	0.238	8.54E-36	Nonclassic	Cdk14
Dazap23	4.18E-40	0.757956	0.565	0.454	1.02E-35	Nonclassic	Dazap2
Id21	1.10E-39	0.969886	0.315	0.187	2.69E-35	Nonclassic	Id2
G6pdx2	1.26E-39	1.224034	0.259	0.145	3.08E-35	Nonclassic	G6pdx
Birc32	2.63E-39	1.004069	0.363	0.232	6.43E-35	Nonclassic	Birc3
Cox6b14	2.68E-39	0.594094	0.666	0.557	6.57E-35	Nonclassic	Cox6b1
Rps264	3.46E-39	0.380888	0.888	0.821	8.48E-35	Nonclassic	Rps26
Rftn12	3.57E-39	1.080246	0.262	0.147	8.74E-35	Nonclassic	Rftn1
Nt5c2	6.58E-39	1.06643	0.323	0.202	1.61E-34	Nonclassic	Nt5c
Cycs1	1.17E-38	0.888747	0.428	0.304	2.87E-34	Nonclassic	Cycs
Nop102	3.53E-38	1.033346	0.414	0.293	8.65E-34	Nonclassic	Nop10
Fam32a2	6.35E-38	1.064028	0.334	0.211	1.56E-33	Nonclassic	Fam32a
Zfp36l22	1.21E-37	0.726688	0.612	0.523	2.97E-33	Nonclassic	Zfp36l2
Rpl384	1.73E-37	0.603125	0.78	0.701	4.23E-33	Nonclassic	Rpl38
Hnrnpf1	1.90E-37	0.659186	0.604	0.512	4.65E-33	Nonclassic	Hnrnpf
Snrpe2	2.56E-37	0.894856	0.467	0.352	6.26E-33	Nonclassic	Snrpe
Themis23	3.34E-37	1.098182	0.338	0.22	8.17E-33	Nonclassic	Themis2
Clec2d3	5.70E-37	0.983059	0.391	0.264	1.40E-32	Nonclassic	Clec2d
Ptprj1	8.60E-37	0.574087	0.7	0.642	2.11E-32	Nonclassic	Ptprj
Ms4a6b2	1.59E-36	0.910162	0.419	0.295	3.90E-32	Nonclassic	Ms4a6b
Apobec12	2.81E-36	0.738046	0.506	0.378	6.89E-32	Nonclassic	Apobec1
Plekhm31	3.50E-36	1.013014	0.344	0.22	8.58E-32	Nonclassic	Plekhm3
Mpp12	4.96E-36	1.214268	0.26	0.151	1.22E-31	Nonclassic	Mpp1
Ndufb74	5.67E-36	0.833067	0.492	0.373	1.39E-31	Nonclassic	Ndufb7
Znhit13	5.90E-36	1.171613	0.307	0.193	1.44E-31	Nonclassic	Znhit1
Sec61g3	1.07E-35	0.834353	0.537	0.435	2.63E-31	Nonclassic	Sec61g
CAAA0114:	1.33E-35	0.68672	0.521	0.401	3.26E-31	Nonclassic	CAAA01147332.1
Psmd82	1.39E-35	0.958263	0.429	0.317	3.40E-31	Nonclassic	Psmd8
Ywhab2	2.49E-35	0.860586	0.462	0.347	6.09E-31	Nonclassic	Ywhab
Atp5g34	2.52E-35	0.533002	0.679	0.593	6.16E-31	Nonclassic	Atp5g3

Rpl414	4.38E-35	0.363586	0.934	0.903	1.07E-30	Nonclassic	Rpl41
Pdlim5	7.36E-35	0.968545	0.321	0.2	1.80E-30	Nonclassic	Pdlim5
mt-Co22	1.25E-34	0.333304	0.974	0.945	3.07E-30	Nonclassic	mt-Co2
Ppp2cb1	1.72E-34	1.11929	0.287	0.176	4.21E-30	Nonclassic	Ppp2cb
Degs12	6.64E-34	1.000395	0.357	0.243	1.63E-29	Nonclassic	Degs1
Ndufa113	9.16E-34	0.858297	0.468	0.351	2.24E-29	Nonclassic	Ndufa11
Nap1l12	2.24E-33	1.020836	0.341	0.227	5.49E-29	Nonclassic	Nap1l1
C34	4.16E-33	0.561916	0.256	0.145	1.02E-28	Nonclassic	C3
Ilrun	5.00E-33	1.040684	0.3	0.188	1.22E-28	Nonclassic	Ilrun
Nsa23	7.57E-33	0.628031	0.565	0.448	1.85E-28	Nonclassic	Nsa2
Ptpn11	8.49E-33	0.773683	0.563	0.492	2.08E-28	Nonclassic	Ptpn1
Api5	1.09E-32	1.14268	0.254	0.149	2.66E-28	Nonclassic	Api5
Hcst1	1.13E-32	1.355369	0.285	0.182	2.76E-28	Nonclassic	Hcst
Snrpg2	1.21E-32	0.757177	0.525	0.429	2.96E-28	Nonclassic	Snrpg
Cox5b4	1.54E-32	0.559864	0.638	0.545	3.78E-28	Nonclassic	Cox5b
Uqcrq3	2.20E-32	0.666437	0.547	0.438	5.39E-28	Nonclassic	Uqcrq
Stat31	2.23E-32	0.775447	0.484	0.378	5.45E-28	Nonclassic	Stat3
Hsp90ab13	4.84E-32	0.431263	0.805	0.74	1.19E-27	Nonclassic	Hsp90ab1
Zfand52	5.16E-32	0.798004	0.459	0.342	1.26E-27	Nonclassic	Zfand5
Ubl54	5.25E-32	0.584019	0.593	0.482	1.28E-27	Nonclassic	Ubl5
Tpr2	5.93E-32	0.795384	0.462	0.354	1.45E-27	Nonclassic	Tpr
Mrpl303	5.98E-32	0.924477	0.393	0.283	1.47E-27	Nonclassic	Mrpl30
Jpt12	9.17E-32	0.811085	0.468	0.359	2.24E-27	Nonclassic	Jpt1
Stat5b	1.51E-31	0.905407	0.296	0.183	3.69E-27	Nonclassic	Stat5b
Mfsd14b1	1.51E-31	1.054258	0.25	0.147	3.70E-27	Nonclassic	Mfsd14b
Supt4a2	2.33E-31	0.79071	0.42	0.306	5.70E-27	Nonclassic	Supt4a
Ppp1r12a2	3.02E-31	0.729838	0.526	0.419	7.40E-27	Nonclassic	Ppp1r12a
Usp321	3.21E-31	1.054143	0.278	0.172	7.86E-27	Nonclassic	Usp32
Prelid14	6.13E-31	0.822851	0.444	0.336	1.50E-26	Nonclassic	Prelid1
Ptpn64	1.07E-30	0.645466	0.587	0.514	2.62E-26	Nonclassic	Ptpn6
Sf3b52	1.24E-30	0.899882	0.413	0.304	3.03E-26	Nonclassic	Sf3b5
Kras1	2.20E-30	0.932527	0.348	0.24	5.38E-26	Nonclassic	Kras
Atp5d4	7.22E-30	0.573172	0.616	0.529	1.77E-25	Nonclassic	Atp5d
Mpc22	7.35E-30	0.828819	0.444	0.338	1.80E-25	Nonclassic	Mpc2
Hpcal14	7.58E-30	0.927822	0.365	0.261	1.86E-25	Nonclassic	Hpcal1
Rpl13a3	7.96E-30	0.336462	0.871	0.792	1.95E-25	Nonclassic	Rpl13a
Emd1	1.25E-29	1.141462	0.268	0.168	3.05E-25	Nonclassic	Emd
Bach12	1.67E-29	0.928418	0.358	0.251	4.10E-25	Nonclassic	Bach1
5031439G0	1.72E-29	0.904688	0.349	0.24	4.21E-25	Nonclassic	5031439G07Rik
Ppp2r5c1	2.01E-29	0.792503	0.43	0.319	4.92E-25	Nonclassic	Ppp2r5c
Nsd32	3.13E-29	0.779263	0.432	0.322	7.67E-25	Nonclassic	Nsd3
Ndel11	3.47E-29	0.983413	0.294	0.189	8.49E-25	Nonclassic	Ndel1
Polr1d2	6.22E-29	0.637628	0.525	0.423	1.52E-24	Nonclassic	Polr1d
Hipk11	7.84E-29	0.960824	0.289	0.186	1.92E-24	Nonclassic	Hipk1

Esd3	1.43E-28	0.779852	0.426	0.326	3.51E-24	Nonclassic	Esd
Gnai22	4.47E-28	0.349661	0.788	0.784	1.10E-23	Nonclassic	Gnai2
Il2rg4	6.30E-28	0.813657	0.332	0.22	1.54E-23	Nonclassic	Il2rg
Cdk17	6.51E-28	0.995826	0.269	0.169	1.59E-23	Nonclassic	Cdk17
Cript2	6.95E-28	1.059308	0.283	0.185	1.70E-23	Nonclassic	Cript
Nsf	7.87E-28	0.944791	0.289	0.186	1.93E-23	Nonclassic	Nsf
Rpl53	1.73E-27	0.461903	0.706	0.615	4.23E-23	Nonclassic	Rpl5
Chmp4b5	1.80E-27	0.606948	0.555	0.465	4.42E-23	Nonclassic	Chmp4b
Nedd82	2.41E-27	0.571314	0.551	0.451	5.90E-23	Nonclassic	Nedd8
Prdx13	2.64E-27	0.256622	0.642	0.547	6.47E-23	Nonclassic	Prdx1
Psmb52	4.40E-27	0.702584	0.441	0.339	1.08E-22	Nonclassic	Psmb5
Myadm	5.50E-27	0.967581	0.311	0.21	1.35E-22	Nonclassic	Myadm
Pgk14	8.01E-27	0.787967	0.362	0.258	1.96E-22	Nonclassic	Pgk1
Vti1a1	1.25E-26	0.696467	0.458	0.351	3.07E-22	Nonclassic	Vti1a
Cdc422	2.66E-26	0.373106	0.749	0.732	6.53E-22	Nonclassic	Cdc42
Crk	2.75E-26	1.054417	0.278	0.182	6.75E-22	Nonclassic	Crk
Psmb33	3.38E-26	0.67645	0.495	0.401	8.28E-22	Nonclassic	Psmb3
Rnf166	5.88E-26	1.03597	0.295	0.197	1.44E-21	Nonclassic	Rnf166
Map3k14	1.01E-25	1.094472	0.254	0.16	2.48E-21	Nonclassic	Map3k14
Capns13	2.61E-25	0.743549	0.447	0.351	6.38E-21	Nonclassic	Capns1
Ndufb103	3.05E-25	0.660364	0.491	0.401	7.46E-21	Nonclassic	Ndufb10
Cpe2	4.24E-25	1.011735	0.252	0.158	1.04E-20	Nonclassic	Cpe
Piezo1	6.04E-25	0.953683	0.261	0.166	1.48E-20	Nonclassic	Piezo1
Tspo5	7.22E-25	0.56359	0.534	0.45	1.77E-20	Nonclassic	Tspo
Rpl44	8.02E-25	0.417671	0.675	0.599	1.97E-20	Nonclassic	Rpl4
Rpl293	1.31E-24	0.278713	0.848	0.801	3.22E-20	Nonclassic	Rpl29
Eef23	2.33E-24	0.353089	0.769	0.722	5.70E-20	Nonclassic	Eef2
Rpl224	2.59E-24	0.314315	0.814	0.747	6.33E-20	Nonclassic	Rpl22
Mrpl142	2.97E-24	0.947068	0.313	0.221	7.28E-20	Nonclassic	Mrpl14
Atp5c12	3.77E-24	0.537232	0.574	0.505	9.24E-20	Nonclassic	Atp5c1
Ndufa42	5.21E-24	0.507806	0.611	0.544	1.28E-19	Nonclassic	Ndufa4
Elf41	5.69E-24	0.813321	0.36	0.261	1.39E-19	Nonclassic	Elf4
Csf2ra3	6.82E-24	0.652705	0.508	0.418	1.67E-19	Nonclassic	Csf2ra
Irf52	1.56E-23	0.645608	0.533	0.472	3.82E-19	Nonclassic	Irf5
Ncoa1	2.10E-23	0.794781	0.336	0.236	5.15E-19	Nonclassic	Ncoa1
1810037I1	2.36E-23	0.748291	0.434	0.342	5.77E-19	Nonclassic	1810037I17Rik
Hnrnpul22	2.71E-23	0.864565	0.327	0.234	6.64E-19	Nonclassic	Hnrnpul2
Syf22	2.75E-23	0.855634	0.332	0.24	6.74E-19	Nonclassic	Syf2
Rps27l3	3.07E-23	0.542963	0.459	0.359	7.52E-19	Nonclassic	Rps27l
Rps153	4.65E-23	0.476581	0.647	0.569	1.14E-18	Nonclassic	Rps15
Gsap2	1.46E-22	0.81073	0.384	0.291	3.58E-18	Nonclassic	Gsap
Irf73	1.70E-22	0.466392	0.289	0.19	4.16E-18	Nonclassic	Irf7
Mob1a1	1.81E-22	0.901812	0.321	0.229	4.44E-18	Nonclassic	Mob1a
Pfdn54	1.96E-22	0.336388	0.771	0.691	4.80E-18	Nonclassic	Pfdn5

Aip2	3.39E-22	0.912207	0.294	0.204	8.31E-18	Nonclassic Aip
Ndufb1-ps2	3.60E-22	0.830795	0.388	0.299	8.82E-18	Nonclassic Ndufb1-ps
Stk41	5.15E-22	0.73304	0.384	0.29	1.26E-17	Nonclassic Stk4
Spop1	1.02E-21	0.622338	0.454	0.36	2.50E-17	Nonclassic Spop
Hdgf3	1.15E-21	0.946818	0.258	0.173	2.81E-17	Nonclassic Hdgf
Eif4g31	1.20E-21	0.596517	0.506	0.417	2.93E-17	Nonclassic Eif4g3
mt-Atp62	1.28E-21	0.31746	0.929	0.914	3.13E-17	Nonclassic mt-Atp6
Ahcyl2	1.38E-21	0.843276	0.271	0.18	3.37E-17	Nonclassic Ahcyl2
BC0285281	1.45E-21	0.839623	0.342	0.255	3.56E-17	Nonclassic BC028528
Calm33	1.68E-21	0.698411	0.423	0.333	4.11E-17	Nonclassic Calm3
Ppp4c2	1.79E-21	0.718972	0.428	0.342	4.38E-17	Nonclassic Ppp4c
Etv62	1.93E-21	0.570641	0.641	0.586	4.74E-17	Nonclassic Etv6
Mrpl571	2.27E-21	0.868938	0.3	0.213	5.56E-17	Nonclassic Mrpl57
Rab5if4	2.93E-21	0.632788	0.473	0.396	7.18E-17	Nonclassic Rab5if
Tmem1671	4.29E-21	0.736135	0.361	0.272	1.05E-16	Nonclassic Tmem167
Cox6a13	5.11E-21	0.545957	0.59	0.514	1.25E-16	Nonclassic Cox6a1
Rpl23a3	5.31E-21	0.498491	0.581	0.506	1.30E-16	Nonclassic Rpl23a
Atp5f12	6.73E-21	0.581216	0.546	0.485	1.65E-16	Nonclassic Atp5f1
Klf32	7.40E-21	0.670434	0.489	0.414	1.81E-16	Nonclassic Klf3
Banf13	8.31E-21	0.779492	0.359	0.269	2.03E-16	Nonclassic Banf1
Akap132	1.12E-20	0.532695	0.557	0.497	2.73E-16	Nonclassic Akap13
Arf61	1.21E-20	0.808033	0.353	0.268	2.97E-16	Nonclassic Arf6
Mt33	1.44E-20	0.834599	0.311	0.218	3.53E-16	Nonclassic Mt3
Tent5a3	1.59E-20	0.838942	0.344	0.254	3.90E-16	Nonclassic Tent5a
Ap2s14	2.13E-20	0.713945	0.388	0.3	5.23E-16	Nonclassic Ap2s1
Lamtor23	2.40E-20	0.665883	0.426	0.343	5.88E-16	Nonclassic Lamtor2
Kif5b2	3.33E-20	0.682761	0.409	0.325	8.15E-16	Nonclassic Kif5b
Tax1bp12	3.54E-20	0.694348	0.435	0.352	8.66E-16	Nonclassic Tax1bp1
Mrtfa2	4.98E-20	0.699373	0.322	0.232	1.22E-15	Nonclassic Mrtfa
Taok31	8.40E-20	0.588701	0.457	0.369	2.06E-15	Nonclassic Taok3
Nop531	9.23E-20	1.018646	0.266	0.186	2.26E-15	Nonclassic Nop53
Ppp1r22	1.08E-19	0.784184	0.306	0.22	2.65E-15	Nonclassic Ppp1r2
Raly2	1.16E-19	0.773131	0.376	0.294	2.83E-15	Nonclassic Raly
Ube2a1	1.32E-19	0.766891	0.312	0.227	3.22E-15	Nonclassic Ube2a
Cox142	1.63E-19	0.774939	0.35	0.266	3.99E-15	Nonclassic Cox14
Hcls12	1.77E-19	0.645033	0.494	0.413	4.34E-15	Nonclassic Hcls1
Dnaja13	2.14E-19	0.702465	0.379	0.293	5.23E-15	Nonclassic Dnaja1
Baz1a2	2.36E-19	0.764923	0.311	0.221	5.77E-15	Nonclassic Baz1a
Elob5	2.92E-19	0.430495	0.599	0.525	7.15E-15	Nonclassic Elob
Psma23	3.72E-19	0.679682	0.44	0.357	9.11E-15	Nonclassic Psma2
Ywhag	4.11E-19	0.838425	0.37	0.289	1.01E-14	Nonclassic Ywhag
Rwdd11	4.60E-19	0.834583	0.28	0.197	1.13E-14	Nonclassic Rwdd1
Sod23	5.02E-19	0.925838	0.268	0.19	1.23E-14	Nonclassic Sod2
Tmem1342	6.49E-19	0.883401	0.336	0.254	1.59E-14	Nonclassic Tmem134

Cox8a4	9.50E-19	0.268169	0.837	0.784	2.33E-14	Nonclassic	Cox8a
Rps253	9.55E-19	0.408186	0.696	0.639	2.34E-14	Nonclassic	Rps25
Zfp7063	1.01E-18	0.551608	0.563	0.503	2.47E-14	Nonclassic	Zfp706
Ramp11	1.16E-18	0.732853	0.332	0.246	2.85E-14	Nonclassic	Ramp1
Tmem1603	1.19E-18	0.710678	0.374	0.294	2.92E-14	Nonclassic	Tmem160
Mkrn13	1.63E-18	0.564814	0.386	0.3	3.98E-14	Nonclassic	Mkrn1
Prdx23	2.41E-18	0.599614	0.429	0.351	5.91E-14	Nonclassic	Prdx2
Ldha4	2.51E-18	0.505355	0.527	0.445	6.14E-14	Nonclassic	Ldha
Zbp14	2.75E-18	0.723182	0.262	0.18	6.74E-14	Nonclassic	Zbp1
Gpi14	3.60E-18	0.512577	0.527	0.454	8.83E-14	Nonclassic	Gpi1
Atp5md3	3.99E-18	0.607575	0.481	0.404	9.78E-14	Nonclassic	Atp5md
Atp5o4	7.30E-18	0.631217	0.458	0.38	1.79E-13	Nonclassic	Atp5o
Rassf41	8.21E-18	0.699738	0.451	0.387	2.01E-13	Nonclassic	Rassf4
Mrps243	8.72E-18	0.778147	0.359	0.279	2.14E-13	Nonclassic	Mrps24
Ago2	9.81E-18	0.819451	0.251	0.173	2.40E-13	Nonclassic	Ago2
Raf11	1.01E-17	0.836347	0.268	0.188	2.48E-13	Nonclassic	Raf1
Serbp12	1.13E-17	0.627107	0.476	0.407	2.76E-13	Nonclassic	Serbp1
Card193	1.20E-17	0.700558	0.331	0.249	2.94E-13	Nonclassic	Card19
Mcmbp	1.71E-17	0.757999	0.252	0.172	4.20E-13	Nonclassic	Mcmbp
Ndufb94	3.66E-17	0.518911	0.517	0.451	8.96E-13	Nonclassic	Ndufb9
Cox7a25	4.25E-17	0.497424	0.533	0.462	1.04E-12	Nonclassic	Cox7a2
Bnip3l2	4.54E-17	0.539481	0.434	0.357	1.11E-12	Nonclassic	Bnip3l
Etfb2	4.69E-17	0.714745	0.353	0.278	1.15E-12	Nonclassic	Etfb
Map1lc3b4	6.28E-17	0.602479	0.42	0.346	1.54E-12	Nonclassic	Map1lc3b
Atp2b13	6.57E-17	0.5702	0.519	0.458	1.61E-12	Nonclassic	Atp2b1
Psmb43	7.35E-17	0.736907	0.358	0.282	1.80E-12	Nonclassic	Psmb4
Snrpd32	8.03E-17	0.745324	0.321	0.242	1.97E-12	Nonclassic	Snrpd3
Eif3a3	8.29E-17	0.625969	0.419	0.343	2.03E-12	Nonclassic	Eif3a
Hnrnpab2	1.07E-16	0.660322	0.416	0.341	2.63E-12	Nonclassic	Hnrnpab
Fam49b1	1.10E-16	0.287541	0.739	0.746	2.69E-12	Nonclassic	Fam49b
Hck3	1.13E-16	0.602142	0.58	0.557	2.78E-12	Nonclassic	Hck
Ndufb84	1.50E-16	0.542934	0.471	0.395	3.68E-12	Nonclassic	Ndufb8
Lsm43	1.54E-16	0.771181	0.334	0.258	3.78E-12	Nonclassic	Lsm4
Cbl2	1.73E-16	0.567851	0.447	0.37	4.24E-12	Nonclassic	Cbl
Stat6	1.78E-16	0.762091	0.286	0.209	4.35E-12	Nonclassic	Stat6
Bax2	1.80E-16	0.560265	0.441	0.364	4.40E-12	Nonclassic	Bax
Ptp4a21	2.03E-16	0.448851	0.564	0.498	4.98E-12	Nonclassic	Ptp4a2
Llph2	2.20E-16	0.814079	0.313	0.237	5.40E-12	Nonclassic	Llph
Nme14	2.43E-16	0.788192	0.338	0.262	5.95E-12	Nonclassic	Nme1
Eef1b24	3.35E-16	0.26557	0.74	0.68	8.21E-12	Nonclassic	Eef1b2
Chmp2a3	3.70E-16	0.647603	0.386	0.309	9.06E-12	Nonclassic	Chmp2a
Ccm2	3.83E-16	0.791833	0.34	0.263	9.38E-12	Nonclassic	Ccm2
Mrpl202	4.39E-16	0.722996	0.336	0.259	1.07E-11	Nonclassic	Mrpl20
mt-Nd42	4.42E-16	0.327174	0.805	0.741	1.08E-11	Nonclassic	mt-Nd4

Atp5j23	4.76E-16	0.443899	0.586	0.521	1.17E-11	Nonclassic Atp5j2
Aurkaip15	5.38E-16	0.704635	0.336	0.262	1.32E-11	Nonclassic Aurkaip1
Mrpl542	6.79E-16	0.849129	0.268	0.196	1.66E-11	Nonclassic Mrpl54
Prkce1	7.07E-16	0.442483	0.271	0.187	1.73E-11	Nonclassic Prkce
Cox7c2	7.09E-16	0.442227	0.633	0.575	1.74E-11	Nonclassic Cox7c
Lyst1	7.59E-16	0.81983	0.302	0.227	1.86E-11	Nonclassic Lyst
Eif4g21	8.94E-16	0.519012	0.519	0.448	2.19E-11	Nonclassic Eif4g2
Ube2s4	1.07E-15	0.761158	0.327	0.255	2.62E-11	Nonclassic Ube2s
Cdc42se22	1.13E-15	0.64848	0.354	0.274	2.78E-11	Nonclassic Cdc42se2
Rb11	1.51E-15	0.645079	0.3	0.221	3.70E-11	Nonclassic Rb1
Nrros1	1.68E-15	0.47544	0.576	0.529	4.13E-11	Nonclassic Nrros
Hsp90aa11	2.09E-15	0.769802	0.397	0.33	5.12E-11	Nonclassic Hsp90aa1
Ube2b1	2.21E-15	0.562726	0.438	0.364	5.42E-11	Nonclassic Ube2b
Far11	2.98E-15	0.604472	0.271	0.193	7.30E-11	Nonclassic Far1
Bola23	3.96E-15	0.930854	0.298	0.229	9.70E-11	Nonclassic Bola2
Actn4	4.57E-15	0.842498	0.252	0.183	1.12E-10	Nonclassic Actn4
Hacd43	5.05E-15	0.697318	0.292	0.217	1.24E-10	Nonclassic Hacd4
Tgfb11	5.66E-15	0.443263	0.638	0.623	1.39E-10	Nonclassic Tgfb1
Arap11	5.98E-15	0.751687	0.312	0.24	1.46E-10	Nonclassic Arap1
Aldoa4	7.00E-15	0.286718	0.661	0.596	1.71E-10	Nonclassic Aldoa
Zeb21	7.72E-15	0.318133	0.772	0.761	1.89E-10	Nonclassic Zeb2
Ndufb113	8.28E-15	0.510346	0.492	0.432	2.03E-10	Nonclassic Ndufb11
Hnrnpa2b1	8.57E-15	0.304469	0.728	0.721	2.10E-10	Nonclassic Hnrnpa2b1
Plcg21	1.04E-14	0.45301	0.509	0.449	2.54E-10	Nonclassic Plcg2
Sfr11	1.07E-14	0.719396	0.332	0.26	2.62E-10	Nonclassic Sfr1
Mier1	1.17E-14	0.67169	0.359	0.286	2.86E-10	Nonclassic Mier1
Cdc40	1.22E-14	0.821307	0.256	0.186	2.99E-10	Nonclassic Cdc40
Birc61	1.29E-14	0.602546	0.413	0.34	3.16E-10	Nonclassic Birc6
Rps284	1.31E-14	0.362559	0.794	0.758	3.21E-10	Nonclassic Rps28
Cul3	1.40E-14	0.704956	0.3	0.226	3.44E-10	Nonclassic Cul3
Atp5a14	1.85E-14	0.451084	0.528	0.472	4.52E-10	Nonclassic Atp5a1
Ankrd441	2.03E-14	0.357754	0.597	0.535	4.98E-10	Nonclassic Ankrd44
Fkbp1a3	2.25E-14	0.626004	0.409	0.337	5.51E-10	Nonclassic Fkbp1a
Ptgds3	2.86E-14	0.496853	0.336	0.254	6.99E-10	Nonclassic Ptgds
Atg31	3.87E-14	0.630524	0.309	0.237	9.48E-10	Nonclassic Atg3
Rock12	4.08E-14	0.56635	0.381	0.308	1.00E-09	Nonclassic Rock1
Mdh24	5.17E-14	0.569163	0.424	0.356	1.27E-09	Nonclassic Mdh2
Lrrc8d1	5.33E-14	0.687244	0.35	0.28	1.31E-09	Nonclassic Lrrc8d
Cwc152	5.41E-14	0.64642	0.38	0.311	1.32E-09	Nonclassic Cwc15
Fgfr1op21	5.87E-14	0.676691	0.335	0.267	1.44E-09	Nonclassic Fgfr1op2
Zc3hav13	6.13E-14	0.767545	0.308	0.239	1.50E-09	Nonclassic Zc3hav1
Cib11	6.48E-14	0.656152	0.261	0.191	1.59E-09	Nonclassic Cib1
Sri3	6.75E-14	0.541611	0.391	0.323	1.65E-09	Nonclassic Sri
Tmem1312	6.94E-14	0.700442	0.375	0.308	1.70E-09	Nonclassic Tmem131

Ndufb33	7.25E-14	0.677077	0.373	0.306	1.78E-09	Nonclassic Ndufb3
Pip4k2a1	9.26E-14	0.270233	0.697	0.655	2.27E-09	Nonclassic Pip4k2a
Atp6v1e13	1.05E-13	0.685615	0.358	0.291	2.57E-09	Nonclassic Atp6v1e1
Hnrnpd1	1.22E-13	0.579417	0.399	0.333	2.98E-09	Nonclassic Hnrnpd
Tomm223	1.44E-13	0.585187	0.393	0.328	3.53E-09	Nonclassic Tomm22
Alyref2	1.57E-13	0.764679	0.277	0.21	3.84E-09	Nonclassic Alyref
Eif3e2	1.59E-13	0.635569	0.37	0.307	3.90E-09	Nonclassic Eif3e
Mrps213	1.62E-13	0.597716	0.41	0.346	3.96E-09	Nonclassic Mrps21
Sap181	2.84E-13	0.697128	0.355	0.291	6.94E-09	Nonclassic Sap18
Ncor12	2.88E-13	0.476453	0.477	0.414	7.05E-09	Nonclassic Ncor1
Gnb12	3.62E-13	0.425755	0.544	0.489	8.87E-09	Nonclassic Gnb1
Arhgdia3	3.73E-13	0.385368	0.586	0.535	9.13E-09	Nonclassic Arhgdia
Akna1	3.93E-13	0.628124	0.285	0.214	9.62E-09	Nonclassic Akna
Dctn33	7.15E-13	0.673866	0.342	0.277	1.75E-08	Nonclassic Dctn3
1810058I2	7.18E-13	0.636046	0.332	0.264	1.76E-08	Nonclassic 1810058I24Rik
Rac1	9.05E-13	0.290756	0.683	0.664	2.22E-08	Nonclassic Rac1
Tank1	1.02E-12	0.771271	0.275	0.21	2.50E-08	Nonclassic Tank
Rpl143	1.09E-12	0.25195	0.776	0.717	2.68E-08	Nonclassic Rpl14
Rin32	1.20E-12	0.505917	0.31	0.237	2.95E-08	Nonclassic Rin3
Adipor13	1.28E-12	0.43666	0.5	0.441	3.14E-08	Nonclassic Adipor1
Eif4ebp2	1.35E-12	0.701477	0.273	0.208	3.31E-08	Nonclassic Eif4ebp2
Vamp4	1.49E-12	0.687153	0.328	0.263	3.64E-08	Nonclassic Vamp4
Rps174	1.59E-12	0.402086	0.614	0.565	3.90E-08	Nonclassic Rps17
Tab21	2.47E-12	0.59441	0.407	0.345	6.05E-08	Nonclassic Tab2
Smchd11	2.85E-12	0.641585	0.325	0.259	6.98E-08	Nonclassic Smchd1
Fam174a2	3.39E-12	0.653763	0.376	0.317	8.30E-08	Nonclassic Fam174a
Sf3b11	3.41E-12	0.46981	0.532	0.487	8.35E-08	Nonclassic Sf3b1
Atp6v0e2	3.53E-12	0.359779	0.614	0.564	8.65E-08	Nonclassic Atp6v0e
Rel1	4.17E-12	0.682526	0.351	0.29	1.02E-07	Nonclassic Rel
Ralbp12	4.18E-12	0.667214	0.302	0.237	1.02E-07	Nonclassic Ralbp1
Atp6v1f4	5.77E-12	0.313736	0.625	0.559	1.41E-07	Nonclassic Atp6v1f
Epsti12	1.13E-11	0.420833	0.468	0.413	2.78E-07	Nonclassic Epsti1
Gtf2h52	1.24E-11	0.682006	0.266	0.203	3.04E-07	Nonclassic Gtf2h5
Ndufa16	1.30E-11	0.550342	0.395	0.332	3.19E-07	Nonclassic Ndufa1
Mia21	1.31E-11	0.576993	0.399	0.34	3.21E-07	Nonclassic Mia2
Psme23	1.49E-11	0.28052	0.582	0.521	3.65E-07	Nonclassic Psme2
Sumo12	1.64E-11	0.511857	0.391	0.326	4.02E-07	Nonclassic Sumo1
Csk2	1.86E-11	0.570857	0.412	0.359	4.56E-07	Nonclassic Csk
Ncor21	2.08E-11	0.505763	0.355	0.287	5.10E-07	Nonclassic Ncor2
Lamtor52	2.26E-11	0.603115	0.306	0.246	5.53E-07	Nonclassic Lamtor5
Srp143	2.28E-11	0.523377	0.428	0.368	5.58E-07	Nonclassic Srp14
Sbno2	2.58E-11	0.625773	0.26	0.197	6.33E-07	Nonclassic Sbno2
Cox172	2.74E-11	0.609938	0.406	0.349	6.70E-07	Nonclassic Cox17
H131	3.41E-11	0.570539	0.379	0.325	8.35E-07	Nonclassic H13

Atp6v1b23	3.65E-11	0.668939	0.385	0.338	8.94E-07	Nonclassic Atp6v1b2
Tlr131	3.81E-11	0.624897	0.301	0.238	9.32E-07	Nonclassic Tlr13
Nfkbia4	3.87E-11	0.259655	0.556	0.489	9.49E-07	Nonclassic Nfkbia
Ndufa74	4.76E-11	0.39218	0.543	0.497	1.17E-06	Nonclassic Ndufa7
Sumo22	5.27E-11	0.38009	0.558	0.513	1.29E-06	Nonclassic Sumo2
Mbd24	5.50E-11	0.630618	0.315	0.255	1.35E-06	Nonclassic Mbd2
Brk12	6.07E-11	0.421845	0.528	0.487	1.49E-06	Nonclassic Brk1
Cap11	6.38E-11	0.408492	0.541	0.51	1.56E-06	Nonclassic Cap1
Ubxn13	7.29E-11	0.528169	0.439	0.389	1.78E-06	Nonclassic Ubxn1
Synj11	7.67E-11	0.56532	0.363	0.3	1.88E-06	Nonclassic Synj1
Rheb2	1.02E-10	0.547803	0.366	0.308	2.51E-06	Nonclassic Rheb
Atp5g13	1.02E-10	0.356888	0.521	0.469	2.51E-06	Nonclassic Atp5g1
Lcp22	1.05E-10	0.432752	0.497	0.455	2.56E-06	Nonclassic Lcp2
Psmd131	1.08E-10	0.698715	0.275	0.218	2.63E-06	Nonclassic Psmd13
Sra12	1.33E-10	0.697266	0.257	0.199	3.26E-06	Nonclassic Sra1
Ndufs63	1.45E-10	0.729753	0.267	0.211	3.54E-06	Nonclassic Ndufs6
Mien13	1.68E-10	0.719694	0.258	0.202	4.12E-06	Nonclassic Mien1
Map3k1	1.75E-10	0.723418	0.266	0.209	4.29E-06	Nonclassic Map3k1
Ggnbp21	1.97E-10	0.6262	0.328	0.27	4.81E-06	Nonclassic Ggnbp2
Mef2d	2.10E-10	0.683448	0.273	0.216	5.14E-06	Nonclassic Mef2d
Map2k12	2.15E-10	0.662928	0.339	0.284	5.26E-06	Nonclassic Map2k1
Fndc3a2	2.20E-10	0.587376	0.359	0.303	5.38E-06	Nonclassic Fndc3a
Srp191	2.25E-10	0.651894	0.273	0.215	5.51E-06	Nonclassic Srp19
Polr2j2	2.36E-10	0.622158	0.263	0.203	5.77E-06	Nonclassic Polr2j
Acer32	2.72E-10	0.445953	0.511	0.467	6.66E-06	Nonclassic Acer3
Chchd23	2.76E-10	0.534521	0.428	0.375	6.76E-06	Nonclassic Chchd2
Bzw1	3.27E-10	0.566949	0.33	0.273	8.01E-06	Nonclassic Bzw1
Dbnl2	3.33E-10	0.535516	0.396	0.344	8.15E-06	Nonclassic Dbnl
Capza12	4.05E-10	0.453207	0.449	0.4	9.91E-06	Nonclassic Capza1
Fam89b2	4.15E-10	0.620979	0.322	0.27	1.02E-05	Nonclassic Fam89b
Prpf40a	4.59E-10	0.52849	0.355	0.3	1.12E-05	Nonclassic Prpf40a
Slc12a61	5.33E-10	0.310141	0.464	0.395	1.30E-05	Nonclassic Slc12a6
Adcy7	5.58E-10	0.68939	0.326	0.273	1.37E-05	Nonclassic Adcy7
Ndufv23	6.63E-10	0.55954	0.354	0.296	1.62E-05	Nonclassic Ndufv2
Snf81	7.50E-10	0.649965	0.282	0.227	1.84E-05	Nonclassic Snf8
Rbx12	8.24E-10	0.408646	0.514	0.466	2.02E-05	Nonclassic Rbx1
Itgb11	8.32E-10	0.489174	0.479	0.44	2.04E-05	Nonclassic Itgb1
Copz12	8.67E-10	0.684853	0.281	0.227	2.12E-05	Nonclassic Copz1
Snx202	1.04E-09	0.54368	0.378	0.327	2.55E-05	Nonclassic Snx20
Lsm12	1.07E-09	0.697575	0.265	0.211	2.63E-05	Nonclassic Lsm12
Tprgl1	1.27E-09	0.61772	0.318	0.263	3.11E-05	Nonclassic Tprgl
Csnk2b3	1.46E-09	0.535163	0.391	0.338	3.58E-05	Nonclassic Csnk2b
Vamp84	1.60E-09	0.299549	0.631	0.596	3.93E-05	Nonclassic Vamp8
Uqcr104	1.63E-09	0.453731	0.436	0.385	3.99E-05	Nonclassic Uqcr10

Foxn31	1.68E-09	0.296878	0.719	0.725	4.10E-05	Nonclassic	Foxn3
Zcrb1	1.71E-09	0.629383	0.301	0.246	4.20E-05	Nonclassic	Zcrb1
21-Mar	1.96E-09	0.61432	0.256	0.202	4.79E-05	Nonclassic	2-Mar
Xbp12	2.00E-09	0.496462	0.426	0.379	4.89E-05	Nonclassic	Xbp1
Sema4d2	2.05E-09	0.4248	0.488	0.442	5.01E-05	Nonclassic	Sema4d
Zfp1061	2.16E-09	0.608556	0.273	0.218	5.28E-05	Nonclassic	Zfp106
Ssna12	2.37E-09	0.687298	0.25	0.196	5.80E-05	Nonclassic	Ssna1
Vps13b1	2.44E-09	0.480716	0.351	0.291	5.96E-05	Nonclassic	Vps13b
Dnajc152	2.47E-09	0.707514	0.256	0.205	6.05E-05	Nonclassic	Dnajc15
Tomm73	2.47E-09	0.511849	0.434	0.388	6.05E-05	Nonclassic	Tomm7
Calhm2	2.50E-09	0.588105	0.256	0.2	6.13E-05	Nonclassic	Calhm2
Rpl22l14	2.67E-09	0.399156	0.542	0.499	6.55E-05	Nonclassic	Rpl22l1
Wdr12	2.79E-09	0.459665	0.419	0.366	6.82E-05	Nonclassic	Wdr1
Hnrnpu	2.82E-09	0.375297	0.525	0.486	6.89E-05	Nonclassic	Hnrnpu
Snx11	2.99E-09	0.632541	0.284	0.232	7.33E-05	Nonclassic	Snx1
Dleu21	3.07E-09	0.305164	0.641	0.623	7.53E-05	Nonclassic	Dleu2
Ssu722	3.81E-09	0.466553	0.416	0.368	9.33E-05	Nonclassic	Ssu72
Pgam14	4.23E-09	0.407521	0.403	0.35	0.000104	Nonclassic	Pgam1
Sf3b23	5.10E-09	0.474354	0.424	0.376	0.000125	Nonclassic	Sf3b2
Tet2	5.36E-09	0.637453	0.257	0.204	0.000131	Nonclassic	Tet2
Snrpc2	6.00E-09	0.621726	0.289	0.237	0.000147	Nonclassic	Snrpc
Eif4a13	6.16E-09	0.313693	0.602	0.577	0.000151	Nonclassic	Eif4a1
Neurl31	6.32E-09	0.529658	0.283	0.229	0.000155	Nonclassic	Neurl3
Lyl13	6.83E-09	0.548445	0.294	0.24	0.000167	Nonclassic	Lyl1
Atp5j5	6.84E-09	0.304676	0.581	0.545	0.000168	Nonclassic	Atp5j
Cox7b4	7.91E-09	0.462549	0.421	0.368	0.000194	Nonclassic	Cox7b
Tomm53	9.18E-09	0.666185	0.252	0.202	0.000225	Nonclassic	Tomm5
Gpsm32	9.85E-09	0.419683	0.433	0.389	0.000241	Nonclassic	Gpsm3
Pdcd62	1.15E-08	0.626588	0.257	0.207	0.000282	Nonclassic	Pdcd6
Atp5b3	1.48E-08	0.320072	0.57	0.537	0.000363	Nonclassic	Atp5b
Ndufb64	1.54E-08	0.553748	0.313	0.263	0.000377	Nonclassic	Ndufb6
Zbtb7a1	1.66E-08	0.558208	0.329	0.279	0.000407	Nonclassic	Zbtb7a
Etf1	1.84E-08	0.550867	0.281	0.23	0.000452	Nonclassic	Etf1
Was1	1.89E-08	0.57343	0.293	0.242	0.000464	Nonclassic	Was
Morf4l11	1.92E-08	0.285933	0.612	0.587	0.000469	Nonclassic	Morf4l1
Pgls2	2.13E-08	0.438442	0.426	0.378	0.000522	Nonclassic	Pgls
Uqcrb4	2.49E-08	0.378157	0.474	0.425	0.000611	Nonclassic	Uqcrb
Ndufb24	3.74E-08	0.657439	0.296	0.247	0.000916	Nonclassic	Ndufb2
Ndufa32	3.84E-08	0.482882	0.427	0.382	0.000939	Nonclassic	Ndufa3
Ppig	4.10E-08	0.549358	0.296	0.246	0.001004	Nonclassic	Ppig
Edf13	4.66E-08	0.355628	0.506	0.464	0.001141	Nonclassic	Edf1
Grb2	5.49E-08	0.324359	0.553	0.517	0.001345	Nonclassic	Grb2
Xiap1	5.52E-08	0.49476	0.316	0.266	0.001352	Nonclassic	Xiap
Rnaseh2c3	5.85E-08	0.638412	0.275	0.228	0.001432	Nonclassic	Rnaseh2c

Timm133	6.86E-08	0.463848	0.382	0.338	0.001679	Nonclassic Timm13
Kmt2e1	8.14E-08	0.435163	0.439	0.401	0.001994	Nonclassic Kmt2e
Soat13	9.17E-08	0.540042	0.332	0.288	0.002246	Nonclassic Soat1
Rnh13	9.67E-08	0.474197	0.392	0.351	0.002368	Nonclassic Rnh1
Ssr31	9.83E-08	0.602435	0.273	0.226	0.002407	Nonclassic Ssr3
Sarnp	1.26E-07	0.500336	0.332	0.286	0.003096	Nonclassic Sarnp
Ythdf31	1.50E-07	0.564048	0.257	0.209	0.003681	Nonclassic Ythdf3
Hectd12	1.53E-07	0.525086	0.321	0.276	0.003747	Nonclassic Hectd1
Etfa2	1.54E-07	0.584555	0.264	0.219	0.003769	Nonclassic Etfa
Higd1a1	1.56E-07	0.552921	0.252	0.204	0.003813	Nonclassic Higd1a
Ndufb54	1.60E-07	0.422533	0.414	0.375	0.003929	Nonclassic Ndufb5
Tifab2	1.73E-07	0.499931	0.348	0.306	0.004237	Nonclassic Tifab
Rgs21	1.74E-07	0.467375	0.434	0.397	0.004267	Nonclassic Rgs2
Tcf7l21	2.00E-07	0.643669	0.27	0.227	0.004902	Nonclassic Tcf7l2
Riok31	2.01E-07	0.531567	0.275	0.23	0.004918	Nonclassic Riok3
Pfdn23	2.63E-07	0.588773	0.319	0.278	0.006433	Nonclassic Pfdn2
Abi11	2.67E-07	0.461517	0.417	0.379	0.006534	Nonclassic Abi1
Cop12	2.74E-07	0.474231	0.263	0.214	0.006712	Nonclassic Cop1
Sik31	2.76E-07	0.465112	0.253	0.203	0.006769	Nonclassic Sik3
Mrpl43	2.96E-07	0.520308	0.258	0.214	0.007254	Nonclassic Mrpl4
Psma42	3.17E-07	0.597444	0.288	0.244	0.007752	Nonclassic Psma4
Dnajc8	3.54E-07	0.556153	0.306	0.259	0.008664	Nonclassic Dnajc8
Timm231	3.60E-07	0.546918	0.317	0.273	0.008815	Nonclassic Timm23
Rab1b2	4.59E-07	0.49444	0.307	0.264	0.011248	Nonclassic Rab1b
Atf61	4.90E-07	0.488156	0.308	0.263	0.012009	Nonclassic Atf6
Ptbp32	5.86E-07	0.333131	0.518	0.487	0.014345	Nonclassic Ptbp3
Eif3i3	5.89E-07	0.532478	0.367	0.327	0.014425	Nonclassic Eif3i
Rab101	6.43E-07	0.519878	0.397	0.361	0.015749	Nonclassic Rab10
Pgd3	6.53E-07	0.413875	0.325	0.28	0.015981	Nonclassic Pgd
Psmb12	6.82E-07	0.387848	0.438	0.405	0.016694	Nonclassic Psmb1
Vps13c1	6.91E-07	0.590894	0.256	0.211	0.016921	Nonclassic Vps13c
Mapk31	8.08E-07	0.409341	0.375	0.334	0.019795	Nonclassic Mapk3
Actr22	8.61E-07	0.31796	0.523	0.503	0.021078	Nonclassic Actr2
Tcf251	8.96E-07	0.41905	0.424	0.387	0.021937	Nonclassic Tcf25
Txn22	9.79E-07	0.531998	0.295	0.252	0.023969	Nonclassic Txn2
Spag91	1.09E-06	0.490195	0.35	0.305	0.026761	Nonclassic Spag9
Timm10b2	1.18E-06	0.358757	0.466	0.435	0.028806	Nonclassic Timm10b
Uqcc24	1.28E-06	0.46689	0.328	0.287	0.031441	Nonclassic Uqcc2
Mrps142	1.63E-06	0.518794	0.293	0.25	0.039871	Nonclassic Mrps14
Dock112	1.87E-06	0.280436	0.355	0.303	0.045842	Nonclassic Dock11
Snu132	1.87E-06	0.513324	0.353	0.319	0.04585	Nonclassic Snu13
Vdac31	2.14E-06	0.481314	0.289	0.247	0.052429	Nonclassic Vdac3
Akt1	2.50E-06	0.416842	0.326	0.285	0.061319	Nonclassic Akt1
Pomp4	2.62E-06	0.252655	0.572	0.552	0.064194	Nonclassic Pomp

Wdr261	2.68E-06	0.36653	0.395	0.352	0.065628	Nonclassic	Wdr26
Uqcr112	2.69E-06	0.344792	0.468	0.432	0.065913	Nonclassic	Uqcr11
Lnpep1	3.15E-06	0.376098	0.426	0.393	0.077125	Nonclassic	Lnpep
Hfe2	3.21E-06	0.818446	0.296	0.265	0.078552	Nonclassic	Hfe
Irf21	4.13E-06	0.362156	0.465	0.439	0.101106	Nonclassic	Irf2
Kxd12	4.15E-06	0.530764	0.285	0.246	0.101517	Nonclassic	Kxd1
Osbpl81	4.46E-06	0.506232	0.296	0.256	0.10934	Nonclassic	Osbpl8
Zfp207	4.63E-06	0.469118	0.277	0.235	0.113431	Nonclassic	Zfp207
Emg11	6.07E-06	0.454267	0.297	0.258	0.148668	Nonclassic	Emg1
Flii2	6.75E-06	0.535364	0.27	0.233	0.165275	Nonclassic	Flii
Ndufa134	6.79E-06	0.279095	0.562	0.538	0.166204	Nonclassic	Ndufa13
Itch2	7.02E-06	0.350779	0.379	0.338	0.171948	Nonclassic	Itch
Cct82	7.35E-06	0.499396	0.296	0.259	0.179974	Nonclassic	Cct8
Bloc1s14	7.60E-06	0.61925	0.263	0.228	0.185995	Nonclassic	Bloc1s1
Pik3r51	7.65E-06	0.409364	0.287	0.244	0.187256	Nonclassic	Pik3r5
Atf7ip2	7.84E-06	0.412048	0.315	0.275	0.191947	Nonclassic	Atf7ip
Ube2k1	8.09E-06	0.459171	0.321	0.282	0.198199	Nonclassic	Ube2k
Hnrnph21	9.40E-06	0.607045	0.254	0.216	0.230143	Nonclassic	Hnrnph2
Cox7a2l3	9.59E-06	0.348443	0.483	0.46	0.2348	Nonclassic	Cox7a2l
Arl6ip51	9.65E-06	0.405282	0.409	0.382	0.236238	Nonclassic	Arl6ip5
Snx32	1.01E-05	0.298871	0.477	0.446	0.247554	Nonclassic	Snx3
Ndufs42	1.13E-05	0.469927	0.334	0.301	0.277603	Nonclassic	Ndufs4
Bcl102	1.50E-05	0.427622	0.302	0.264	0.367765	Nonclassic	Bcl10
H2afv2	1.54E-05	0.571387	0.284	0.248	0.377259	Nonclassic	H2afv
Hist1h1e3	1.63E-05	0.331144	0.325	0.284	0.39905	Nonclassic	Hist1h1e
Pbxip11	1.78E-05	0.586968	0.283	0.251	0.436798	Nonclassic	Pbxip1
Reep52	1.92E-05	0.271673	0.593	0.579	0.46965	Nonclassic	Reep5
Set1	2.28E-05	0.41432	0.357	0.321	0.557701	Nonclassic	Set
Smurf21	2.43E-05	0.539057	0.278	0.24	0.596205	Nonclassic	Smurf2
Atp5mpl5	2.68E-05	0.352747	0.453	0.42	0.656519	Nonclassic	Atp5mpl
Mettl91	2.87E-05	0.529042	0.269	0.234	0.701631	Nonclassic	Mettl9
Stag21	2.97E-05	0.349148	0.3	0.261	0.726704	Nonclassic	Stag2
Ube2r21	3.01E-05	0.477404	0.279	0.243	0.736539	Nonclassic	Ube2r2
Ran2	3.39E-05	0.399077	0.387	0.358	0.830764	Nonclassic	Ran
Xrn21	3.41E-05	0.426609	0.295	0.26	0.834929	Nonclassic	Xrn2
Pdcd10	4.20E-05	0.418591	0.306	0.27	1	Nonclassic	Pdcd10
Psma63	4.42E-05	0.445378	0.306	0.272	1	Nonclassic	Psma6
Rab211	4.83E-05	0.4197	0.272	0.237	1	Nonclassic	Rab21
Nipbl1	5.09E-05	0.30146	0.475	0.45	1	Nonclassic	Nipbl
Brd21	5.12E-05	0.446065	0.3	0.266	1	Nonclassic	Brd2
Ndufa23	5.12E-05	0.34933	0.467	0.438	1	Nonclassic	Ndufa2
Ogdh1	5.18E-05	0.468	0.305	0.272	1	Nonclassic	Ogdh
Limd24	5.96E-05	0.293062	0.605	0.6	1	Nonclassic	Limd2
Hnrnpm	7.11E-05	0.431579	0.329	0.299	1	Nonclassic	Hnrnpm

Hnrnpk1	7.27E-05	0.277349	0.559	0.554	1	Nonclassic	Hnrnpk
Map7d12	8.49E-05	0.488076	0.256	0.223	1	Nonclassic	Map7d1
Purb1	8.56E-05	0.441949	0.338	0.309	1	Nonclassic	Purb
Brd41	9.44E-05	0.314635	0.406	0.377	1	Nonclassic	Brd4
Eif3m3	9.45E-05	0.429292	0.314	0.283	1	Nonclassic	Eif3m
Tmed21	0.000105	0.280416	0.485	0.468	1	Nonclassic	Tmed2
Trir1	0.00011	0.380168	0.38	0.356	1	Nonclassic	Trir
Cisd21	0.000115	0.428602	0.29	0.257	1	Nonclassic	Cisd2
Atad2b1	0.00013	0.403216	0.264	0.229	1	Nonclassic	Atad2b
Csde12	0.000142	0.415332	0.304	0.273	1	Nonclassic	Csde1
Rp91	0.000148	0.409914	0.331	0.305	1	Nonclassic	Rp9
Snrpf3	0.000156	0.505236	0.3	0.273	1	Nonclassic	Snrpf
Tm6sf12	0.000158	0.257563	0.402	0.37	1	Nonclassic	Tm6sf1
Tmem14c4	0.000165	0.325544	0.462	0.444	1	Nonclassic	Tmem14c
Ppp1r9b2	0.000166	0.408866	0.263	0.23	1	Nonclassic	Ppp1r9b
U2af11	0.000173	0.375878	0.315	0.286	1	Nonclassic	U2af1
Phf20l12	0.000183	0.342274	0.336	0.303	1	Nonclassic	Phf20l1
Eif4h1	0.00019	0.423555	0.327	0.3	1	Nonclassic	Eif4h
Mapk1	0.000197	0.428508	0.3	0.268	1	Nonclassic	Mapk1
Paip21	0.000198	0.360598	0.327	0.297	1	Nonclassic	Paip2
Uqcrc11	0.000199	0.452356	0.363	0.341	1	Nonclassic	Uqcrc1
Csf2rb3	0.000222	0.541864	0.287	0.259	1	Nonclassic	Csf2rb
Prrc2c1	0.000228	0.367844	0.391	0.369	1	Nonclassic	Prrc2c
Polr2g2	0.000235	0.537368	0.256	0.229	1	Nonclassic	Polr2g
Pxn1	0.000289	0.361398	0.254	0.221	1	Nonclassic	Pxn
Swi53	0.00029	0.333147	0.406	0.382	1	Nonclassic	Swi5
Polr2a1	0.00033	0.470855	0.295	0.268	1	Nonclassic	Polr2a
Wipf11	0.000339	0.273789	0.407	0.382	1	Nonclassic	Wipf1
Cd483	0.000342	0.384982	0.42	0.406	1	Nonclassic	Cd48
Cct53	0.000351	0.459981	0.296	0.272	1	Nonclassic	Cct5
Srsf3	0.000352	0.365929	0.422	0.406	1	Nonclassic	Srsf3
Chmp52	0.000359	0.411729	0.254	0.224	1	Nonclassic	Chmp5
Aplp23	0.000373	0.387831	0.307	0.278	1	Nonclassic	Aplp2
Ier3ip11	0.000394	0.404271	0.309	0.282	1	Nonclassic	Ier3ip1
Trappc42	0.000403	0.420766	0.285	0.257	1	Nonclassic	Trappc4
Epn11	0.000456	0.361994	0.388	0.366	1	Nonclassic	Epn1
Ube2d2a	0.00048	0.303412	0.454	0.433	1	Nonclassic	Ube2d2a
Usp251	0.000492	0.307245	0.29	0.258	1	Nonclassic	Usp25
Yy11	0.000568	0.456445	0.266	0.237	1	Nonclassic	Yy1
N4bp13	0.000587	0.454007	0.267	0.241	1	Nonclassic	N4bp1
Eef1g2	0.000656	0.391094	0.405	0.385	1	Nonclassic	Eef1g
Eif52	0.000663	0.322158	0.37	0.349	1	Nonclassic	Eif5
Rexo22	0.000698	0.41496	0.256	0.228	1	Nonclassic	Rexo2
Sep-71	0.000835	0.386334	0.344	0.321	1	Nonclassic	7-Sep

Nol71	0.000856	0.485505	0.286	0.262	1	Nonclassic Nol7
Rab2a1	0.000883	0.263202	0.525	0.524	1	Nonclassic Rab2a
Tmem2582	0.001014	0.309659	0.422	0.41	1	Nonclassic Tmem258
Psmd41	0.001029	0.430307	0.287	0.261	1	Nonclassic Psmd4
Cope2	0.001061	0.338031	0.402	0.388	1	Nonclassic Cope
Gabarapl21	0.001069	0.302256	0.4	0.38	1	Nonclassic Gabarapl2
Snw11	0.001084	0.471832	0.252	0.225	1	Nonclassic Snw1
Chd41	0.001132	0.332303	0.34	0.318	1	Nonclassic Chd4
Rbm422	0.001204	0.384538	0.28	0.256	1	Nonclassic Rbm42
Drap13	0.001248	0.340083	0.372	0.352	1	Nonclassic Drap1
Sfpq	0.001291	0.298036	0.379	0.357	1	Nonclassic Sfpq
Rab11b1	0.001306	0.310113	0.38	0.363	1	Nonclassic Rab11b
Cenpx3	0.001398	0.453008	0.303	0.282	1	Nonclassic Cenpx
mt-Nd51	0.001425	0.292798	0.602	0.61	1	Nonclassic mt-Nd5
Ppp2ca	0.001433	0.324669	0.371	0.348	1	Nonclassic Ppp2ca
Ndufs21	0.001439	0.420719	0.292	0.27	1	Nonclassic Ndufs2
Atp6v0d12	0.001516	0.295108	0.395	0.374	1	Nonclassic Atp6v0d1
Csnk1a11	0.001657	0.317557	0.391	0.374	1	Nonclassic Csnk1a1
Uvrag1	0.001715	0.251906	0.461	0.447	1	Nonclassic Uvrag
Spg213	0.001779	0.417767	0.268	0.244	1	Nonclassic Spg21
Emc6	0.001802	0.407773	0.271	0.249	1	Nonclassic Emc6
Dnaja22	0.001864	0.404438	0.329	0.306	1	Nonclassic Dnaja2
Ube2l31	0.001884	0.35127	0.325	0.307	1	Nonclassic Ube2l3
Sf11	0.001973	0.409316	0.344	0.329	1	Nonclassic Sf1
Ythdc11	0.00206	0.381218	0.287	0.263	1	Nonclassic Ythdc1
Fam104a2	0.002082	0.37825	0.258	0.235	1	Nonclassic Fam104a
Dek3	0.002249	0.459291	0.278	0.255	1	Nonclassic Dek
Pdcd51	0.002251	0.377274	0.319	0.295	1	Nonclassic Pdcd5
Dpm1	0.002528	0.369799	0.333	0.312	1	Nonclassic Dpm1
Arpc1a1	0.0026	0.330825	0.308	0.286	1	Nonclassic Arpc1a
Eloc	0.002723	0.353047	0.317	0.296	1	Nonclassic Eloc
Dusp111	0.002748	0.356825	0.26	0.236	1	Nonclassic Dusp11
Rnf115	0.002809	0.296466	0.254	0.227	1	Nonclassic Rnf115
Ak23	0.00292	0.427925	0.294	0.275	1	Nonclassic Ak2
Lrrc252	0.003019	0.271194	0.386	0.368	1	Nonclassic Lrrc25
Ubxn4	0.003064	0.35451	0.319	0.298	1	Nonclassic Ubxn4
Tspan133	0.00312	0.256121	0.393	0.367	1	Nonclassic Tspan13
Pcbp21	0.003229	0.299548	0.365	0.349	1	Nonclassic Pcbp2
Psma33	0.003545	0.26539	0.433	0.424	1	Nonclassic Psma3
Skp1a2	0.003875	0.305855	0.36	0.343	1	Nonclassic Skp1a
Dynlrb13	0.004097	0.363081	0.34	0.324	1	Nonclassic Dynlrb1
Eif3c1	0.004531	0.367584	0.359	0.342	1	Nonclassic Eif3c
Lman22	0.004756	0.390499	0.323	0.306	1	Nonclassic Lman2
Cct72	0.005391	0.353939	0.281	0.263	1	Nonclassic Cct7

Plekhj11	0.005585	0.348846	0.315	0.3	1	Nonclassic	Plekhj1
Tmem179b	0.005687	0.304704	0.424	0.419	1	Nonclassic	Tmem179b
Ndufc14	0.007169	0.394315	0.304	0.286	1	Nonclassic	Ndufc1
Naip2	0.007925	0.32008	0.258	0.237	1	Nonclassic	Naip2
Prrc2a1	0.007947	0.326634	0.328	0.309	1	Nonclassic	Prrc2a
Med283	0.008344	0.285114	0.374	0.361	1	Nonclassic	Med28
Eif4b3	0.008379	0.379157	0.302	0.288	1	Nonclassic	Eif4b
Tcea1	0.008448	0.41404	0.304	0.289	1	Nonclassic	Tcea1
Napsa2	0	4.805041	0.894	0.049	0	cDC1	Napsa
Plbd13	0	4.188713	0.923	0.103	0	cDC1	Plbd1
Vim3	0	3.554854	0.942	0.181	0	cDC1	Vim
Lsp13	0	3.446778	0.942	0.188	0	cDC1	Lsp1
S100a113	0	2.422585	0.887	0.135	0	cDC1	S100a11
Crip12	0	4.052204	0.907	0.168	0	cDC1	Crip1
S100a63	0	2.614145	0.838	0.105	0	cDC1	S100a6
Lgals34	0	2.389159	0.869	0.175	0	cDC1	Lgals3
Itgb71	0	4.761754	0.724	0.03	0	cDC1	Itgb7
Slamf71	0	3.425238	0.79	0.099	0	cDC1	Slamf7
Cnn23	0	2.817151	0.788	0.109	0	cDC1	Cnn2
Fxyd53	0	2.714812	0.938	0.279	0	cDC1	Fxyd5
Anxa23	0	2.577992	0.773	0.125	0	cDC1	Anxa2
Olfm11	0	3.499536	0.716	0.07	0	cDC1	Olfm1
Gsn2	0	3.065352	0.75	0.113	0	cDC1	Gsn
Cbfa2t31	0	2.734539	0.772	0.137	0	cDC1	Cbfa2t3
Alcam1	0	3.798037	0.691	0.057	0	cDC1	Alcam
Trerf11	0	4.542121	0.671	0.04	0	cDC1	Trerf1
Plp21	0	3.962731	0.66	0.034	0	cDC1	Plp2
Cytip2	0	2.670729	0.69	0.07	0	cDC1	Cytip
Iqgap14	0	1.998252	0.766	0.155	0	cDC1	Iqgap1
Lgals13	0	2.827746	0.757	0.151	0	cDC1	Lgals1
Ccr22	0	3.09397	0.656	0.055	0	cDC1	Ccr2
Jaml	0	4.837606	0.613	0.017	0	cDC1	Jaml
Zyx3	0	2.523922	0.831	0.244	0	cDC1	Zyx
Rnase63	0	2.488205	0.752	0.172	0	cDC1	Rnase6
H2-DMb21	0	3.679993	0.651	0.083	0	cDC1	H2-DMb2
S100a42	0	3.937949	0.616	0.048	0	cDC1	S100a4
Dpp4	0	5.234956	0.577	0.013	0	cDC1	Dpp4
Ciita1	0	2.900042	0.659	0.099	0	cDC1	Ciita
Ahnak3	0	2.964876	0.654	0.095	0	cDC1	Ahnak
Tagln22	0	2.83724	0.759	0.202	0	cDC1	Tagln2
S100a102	0	3.546304	0.647	0.091	0	cDC1	S100a10
Pim15	0	2.391292	0.68	0.134	0	cDC1	Pim1
Bri3bp1	0	2.579172	0.655	0.116	0	cDC1	Bri3bp
Arhgap154	0	1.484632	0.806	0.268	0	cDC1	Arhgap15

Ass11	0	3.508532	0.573	0.047	0	cDC1	Ass1
Anxa13	0	1.589344	0.574	0.052	0	cDC1	Anxa1
Flt3	0	4.940444	0.532	0.015	0	cDC1	Flt3
Kmo	0	6.601171	0.522	0.007	0	cDC1	Kmo
Anxa54	0	1.51816	0.799	0.294	0	cDC1	Anxa5
Pfkp1	0	3.342575	0.565	0.061	0	cDC1	Pfkp
Cd209a	0	8.040779	0.506	0.005	0	cDC1	Cd209a
Slfn25	0	1.697397	0.785	0.285	0	cDC1	Slfn2
H2afy2	0	2.106111	0.867	0.367	0	cDC1	H2afy
Jak21	0	2.275248	0.637	0.137	0	cDC1	Jak2
Sh3bgrl4	0	2.202233	0.624	0.124	0	cDC1	Sh3bgrl
Itpr1	0	2.472799	0.593	0.096	0	cDC1	Itpr1
Anp32b3	0	1.591563	0.761	0.278	0	cDC1	Anp32b
St3gal42	0	2.277415	0.596	0.115	0	cDC1	St3gal4
Nap1l13	0	1.76392	0.699	0.218	0	cDC1	Nap1l1
Cfp1	0	3.40788	0.533	0.053	0	cDC1	Cfp
1700025G(	0	3.562133	0.511	0.035	0	cDC1	1700025G04Rik
Ms4a6c3	0	1.79681	0.749	0.273	0	cDC1	Ms4a6c
Klrd1	0	4.714831	0.496	0.023	0	cDC1	Klrd1
Emp34	0	2.049359	0.683	0.212	0	cDC1	Emp3
Ccnd13	0	2.118297	0.673	0.205	0	cDC1	Ccnd1
Myadm1	0	1.601386	0.662	0.201	0	cDC1	Myadm
Itga43	0	1.914196	0.562	0.103	0	cDC1	Itga4
Pkib2	0	2.099899	0.651	0.192	0	cDC1	Pkib
Bloc1s21	0	2.060154	0.6	0.142	0	cDC1	Bloc1s2
Dipk1a1	0	2.342451	0.539	0.084	0	cDC1	Dipk1a
Mndal3	0	2.528012	0.531	0.077	0	cDC1	Mndal
6-Sep	0	3.240481	0.499	0.045	0	cDC1	6-Sep
Gm2a4	0	1.856016	0.882	0.43	0	cDC1	Gm2a
Naaa2	0	2.899504	0.638	0.187	0	cDC1	Naaa
Map4k1	0	3.960358	0.481	0.031	0	cDC1	Map4k1
Tspo6	0	1.677487	0.889	0.441	0	cDC1	Tspo
Klrk1	0	4.28392	0.473	0.025	0	cDC1	Klrk1
Ifitm24	0	1.940257	0.648	0.201	0	cDC1	Ifitm2
Tpm43	0	1.804958	0.599	0.156	0	cDC1	Tpm4
Flna3	0	1.458873	0.628	0.186	0	cDC1	Flna
Zbtb46	0	3.641319	0.473	0.033	0	cDC1	Zbtb46
Prdx64	0	1.573059	0.626	0.189	0	cDC1	Prdx6
Anxa62	0	1.799097	0.612	0.176	0	cDC1	Anxa6
Grap2	0	4.42072	0.448	0.013	0	cDC1	Grap2
Sub12	0	1.88172	0.915	0.481	0	cDC1	Sub1
Eps81	0	2.350576	0.511	0.078	0	cDC1	Eps8
Sep-93	0	1.896889	0.566	0.133	0	cDC1	9-Sep
Mob3b1	0	2.95984	0.481	0.05	0	cDC1	Mob3b

Id22	0	2.643256	0.61	0.18	0	cDC1	Id2
Ms4a4c3	0	2.844447	0.531	0.102	0	cDC1	Ms4a4c
Unc1192	0	2.822229	0.472	0.047	0	cDC1	Unc119
Plac82	0	2.524914	0.506	0.082	0	cDC1	Plac8
Phf11b2	0	2.339811	0.575	0.151	0	cDC1	Phf11b
Wfdc173	0	1.880728	0.533	0.11	0	cDC1	Wfdc17
Fyn1	0	1.960895	0.528	0.106	0	cDC1	Fyn
Gpr1412	0	2.940973	0.463	0.041	0	cDC1	Gpr141
Ifi2032	0	2.60897	0.489	0.068	0	cDC1	Ifi203
Clec12a3	0	2.099989	0.492	0.074	0	cDC1	Clec12a
Rasgrp42	0	2.391472	0.473	0.058	0	cDC1	Rasgrp4
Btla	0	5.590923	0.422	0.007	0	cDC1	Btla
Ppm1m1	0	2.033901	0.534	0.123	0	cDC1	Ppm1m
Ybx31	0	2.089822	0.519	0.11	0	cDC1	Ybx3
Slfn53	0	2.117731	0.534	0.125	0	cDC1	Slfn5
Fam129a3	0	1.812612	0.555	0.147	0	cDC1	Fam129a
Coro2a	0	1.764937	0.537	0.131	0	cDC1	Coro2a
Ifi205	0	4.701144	0.431	0.025	0	cDC1	Ifi205
Runx31	0	3.218326	0.44	0.038	0	cDC1	Runx3
Dapk11	0	2.177998	0.526	0.126	0	cDC1	Dapk1
Mthfd21	0	2.293273	0.5	0.102	0	cDC1	Mthfd2
Vav34	0	2.186749	0.469	0.071	0	cDC1	Vav3
Iqgap22	0	2.396369	0.47	0.072	0	cDC1	Iqgap2
Atp8b42	0	3.087301	0.431	0.036	0	cDC1	Atp8b4
Kynu	0	3.418618	0.436	0.042	0	cDC1	Kynu
Shtn1	0	2.739681	0.458	0.065	0	cDC1	Shtn1
Gm159871	0	3.758291	0.415	0.023	0	cDC1	Gm15987
Pstpip12	0	2.358917	0.457	0.067	0	cDC1	Pstpip1
Dna2	0	2.710968	0.45	0.061	0	cDC1	Dna2
H2-DMb14	0	1.659371	0.916	0.527	0	cDC1	H2-DMb1
H2-Ob	0	2.081143	0.5	0.111	0	cDC1	H2-Ob
Sema4a3	0	1.929839	0.492	0.104	0	cDC1	Sema4a
Trappc5	0	2.298333	0.493	0.106	0	cDC1	Trappc5
Mcemp11	0	2.82386	0.432	0.047	0	cDC1	Mcemp1
Mgl21	0	4.554476	0.428	0.043	0	cDC1	Mgl2
Actr33	0	1.566111	0.929	0.55	0	cDC1	Actr3
Bcl2a1d	0	2.7971	0.436	0.057	0	cDC1	Bcl2a1d
Arhgap261	0	2.486181	0.442	0.064	0	cDC1	Arhgap26
H2-Eb15	0	1.712862	0.962	0.586	0	cDC1	H2-Eb1
Emb3	0	2.117417	0.441	0.066	0	cDC1	Emb
Tes	0	1.942439	0.461	0.089	0	cDC1	Tes
H2afz3	0	1.832723	0.935	0.567	0	cDC1	H2afz
Myo1g2	0	1.516545	0.485	0.117	0	cDC1	Myo1g
H2-Aa5	0	1.782628	0.972	0.608	0	cDC1	H2-Aa

Srebf2	0	2.015045	0.45	0.087	0	cDC1	Srebf2
Fh1	0	1.967308	0.481	0.119	0	cDC1	Fh1
AI6622701	0	2.805755	0.409	0.051	0	cDC1	AI662270
Bhlhe40	0	3.497167	0.396	0.039	0	cDC1	Bhlhe40
Calm15	0	1.359088	0.944	0.587	0	cDC1	Calm1
Mcub1	0	3.06192	0.399	0.046	0	cDC1	Mcub
Cd244a1	0	2.810067	0.392	0.04	0	cDC1	Cd244a
Adgre53	0	1.765937	0.423	0.071	0	cDC1	Adgre5
Slc1a5	0	3.128205	0.388	0.037	0	cDC1	Slc1a5
Tbrg1	0	1.948674	0.438	0.092	0	cDC1	Tbrg1
Lrrk21	0	2.917775	0.373	0.027	0	cDC1	Lrrk2
Ifi473	0	2.151731	0.447	0.102	0	cDC1	Ifi47
Traf1	0	2.999762	0.365	0.023	0	cDC1	Traf1
Tmsb102	0	3.146728	0.971	0.631	0	cDC1	Tmsb10
Ece1	0	2.706576	0.394	0.055	0	cDC1	Ece1
Lmo1	0	5.108938	0.347	0.009	0	cDC1	Lmo1
Ezr3	0	1.688369	0.427	0.089	0	cDC1	Ezr
Bcl11a	0	4.655117	0.349	0.013	0	cDC1	Bcl11a
Hepacam2	0	7.138566	0.337	0.002	0	cDC1	Hepacam2
Clec9a	0	6.195999	0.342	0.007	0	cDC1	Clec9a
Atox13	0	1.726427	0.921	0.588	0	cDC1	Atox1
Nedd93	0	1.44587	0.416	0.085	0	cDC1	Nedd9
Samd9l1	0	2.083218	0.391	0.061	0	cDC1	Samd9l
Cd24a1	0	2.733269	0.365	0.036	0	cDC1	Cd24a
Ifi303	0	1.932122	0.918	0.59	0	cDC1	Ifi30
Srgap3	0	3.31089	0.352	0.024	0	cDC1	Srgap3
Qpct	0	3.852961	0.363	0.035	0	cDC1	Qpct
H2-Ab15	0	1.741594	0.966	0.64	0	cDC1	H2-Ab1
Clec10a1	0	2.850319	0.374	0.049	0	cDC1	Clec10a
Kctd14	0	5.631836	0.33	0.006	0	cDC1	Kctd14
Ahr	0	3.579316	0.348	0.028	0	cDC1	Ahr
Rara1	0	2.596172	0.357	0.038	0	cDC1	Rara
Gpr18	0	2.849219	0.361	0.043	0	cDC1	Gpr18
Gpr132	0	3.096789	0.344	0.027	0	cDC1	Gpr132
Spint2	0	2.136207	0.353	0.039	0	cDC1	Spint2
Btf33	0	1.285484	0.921	0.608	0	cDC1	Btf3
Pqlc1	0	2.367873	0.361	0.051	0	cDC1	Pqlc1
Coro1a3	0	1.375594	0.931	0.623	0	cDC1	Coro1a
Gpr171	0	5.639825	0.314	0.006	0	cDC1	Gpr171
Ap1s3	0	4.910457	0.311	0.007	0	cDC1	Ap1s3
Mical1	0	2.261499	0.361	0.057	0	cDC1	Mical1
Zfp366	0	5.19711	0.309	0.007	0	cDC1	Zfp366
Agpat41	0	2.356122	0.348	0.046	0	cDC1	Agpat4
Card11	0	2.846539	0.338	0.037	0	cDC1	Card11

Klf41	0	1.787597	0.372	0.072	0	cDC1	Klf4
Tmem238	0	3.345365	0.321	0.021	0	cDC1	Tmem238
Slfn12	0	2.032491	0.356	0.056	0	cDC1	Slfn1
St3gal11	0	2.261497	0.356	0.056	0	cDC1	St3gal1
Nup210	0	2.776606	0.332	0.034	0	cDC1	Nup210
Ccnd2	0	2.821368	0.346	0.048	0	cDC1	Ccnd2
Cd209c	0	7.985753	0.3	0.002	0	cDC1	Cd209c
Lmnb11	0	1.914127	0.369	0.071	0	cDC1	Lmnb1
Limd11	0	2.034947	0.365	0.068	0	cDC1	Limd1
Phf11a	0	3.276302	0.332	0.036	0	cDC1	Phf11a
Ctnnd2	0	2.601575	0.351	0.055	0	cDC1	Ctnnd2
Klrb1b	0	5.764364	0.301	0.006	0	cDC1	Klrb1b
Pmaip1	0	2.531543	0.347	0.056	0	cDC1	Pmaip1
Rack14	0	1.384632	0.944	0.653	0	cDC1	Rack1
Avpi1	0	3.037312	0.316	0.026	0	cDC1	Avpi1
Afdn	0	2.469329	0.324	0.035	0	cDC1	Afdn
Kit	0	4.665797	0.296	0.007	0	cDC1	Kit
Lrrc8c	0	2.141601	0.346	0.058	0	cDC1	Lrrc8c
Net1	0	2.985667	0.3	0.016	0	cDC1	Net1
Tnip3	0	4.492939	0.296	0.014	0	cDC1	Tnip3
Cd525	0	1.647444	0.948	0.669	0	cDC1	Cd52
Cyfip22	0	1.673087	0.324	0.049	0	cDC1	Cyfip2
H3f3a4	0	1.250113	0.94	0.668	0	cDC1	H3f3a
Ffar4	0	7.919881	0.272	0.001	0	cDC1	Ffar4
Ltb4r11	0	3.154336	0.293	0.022	0	cDC1	Ltb4r1
Rps64	0	1.430178	0.948	0.683	0	cDC1	Rps6
Rnd3	0	4.63901	0.271	0.008	0	cDC1	Rnd3
Anpep	0	3.355252	0.286	0.024	0	cDC1	Anpep
Aff3	0	3.530653	0.275	0.014	0	cDC1	Aff3
Haao	0	2.03398	0.313	0.052	0	cDC1	Haao
Gpx13	0	1.414893	0.956	0.696	0	cDC1	Gpx1
Eef1b25	0	1.358519	0.934	0.675	0	cDC1	Eef1b2
Itgae	0	5.008687	0.269	0.011	0	cDC1	Itgae
Gramd3	0	2.177398	0.303	0.046	0	cDC1	Gramd3
Clec4b1	0	4.591697	0.268	0.013	0	cDC1	Clec4b1
Slco3a1	0	2.658924	0.281	0.03	0	cDC1	Slco3a1
Cldn1	0	5.004544	0.253	0.005	0	cDC1	Cldn1
Stap11	0	2.077445	0.28	0.035	0	cDC1	Stap1
Pkp3	0	4.611617	0.251	0.007	0	cDC1	Pkp3
Crybg1	0	2.312691	0.288	0.044	0	cDC1	Crybg1
Rpl144	0	1.430584	0.955	0.712	0	cDC1	Rpl14
Plscr1	0	2.483522	0.273	0.034	0	cDC1	Plscr1
Rgs18	0	3.030978	0.257	0.019	0	cDC1	Rgs18
Uck2	0	3.69071	0.253	0.015	0	cDC1	Uck2

Siglecg	0	2.08921	0.272	0.036	0	cDC1	Siglecg
Lsr	0	3.377261	0.252	0.016	0	cDC1	Lsr
Ptma3	0	1.316128	0.958	0.723	0	cDC1	Ptma
Irf4	0	3.474293	0.251	0.018	0	cDC1	Irf4
Cd745	0	1.468395	0.992	0.77	0	cDC1	Cd74
Rpl74	0	1.107851	0.952	0.732	0	cDC1	Rpl7
Sh3bgrl35	0	1.551682	0.955	0.745	0	cDC1	Sh3bgrl3
Rpl225	0	1.20988	0.953	0.744	0	cDC1	Rpl22
Rps184	0	1.418554	0.964	0.757	0	cDC1	Rps18
Actg13	0	1.893263	0.977	0.779	0	cDC1	Actg1
Rpl275	0	1.216777	0.96	0.774	0	cDC1	Rpl27
Rpl85	0	1.40769	0.971	0.792	0	cDC1	Rpl8
Rpl154	0	1.235876	0.96	0.783	0	cDC1	Rpl15
Rpl244	0	1.068341	0.962	0.787	0	cDC1	Rpl24
Rps194	0	1.395899	0.97	0.798	0	cDC1	Rps19
Rps145	0	1.257559	0.967	0.808	0	cDC1	Rps14
Rps216	0	1.311262	0.969	0.814	0	cDC1	Rps2
Rps265	0	1.212501	0.971	0.819	0	cDC1	Rps26
Rpsa4	0	1.775986	0.974	0.831	0	cDC1	Rpsa
Rpl64	0	1.048344	0.966	0.826	0	cDC1	Rpl6
Pfn13	0	1.141508	0.969	0.829	0	cDC1	Pfn1
Rpl264	0	1.039789	0.966	0.827	0	cDC1	Rpl26
Rps35	0	1.210982	0.964	0.827	0	cDC1	Rps3
Rpl174	0	1.37849	0.975	0.838	0	cDC1	Rpl17
Rpl184	0	1.506584	0.97	0.837	0	cDC1	Rpl18
Rplp04	0	1.593814	0.971	0.84	0	cDC1	Rplp0
Rpl284	0	1.29496	0.967	0.839	0	cDC1	Rpl28
Rpl344	0	1.078663	0.964	0.842	0	cDC1	Rpl34
Rps134	0	1.280979	0.969	0.847	0	cDC1	Rps13
Rps54	0	1.507182	0.967	0.851	0	cDC1	Rps5
Rps15a5	0	1.192891	0.969	0.855	0	cDC1	Rps15a
Rps74	0	1.393179	0.978	0.865	0	cDC1	Rps7
Rpl325	0	1.234008	0.972	0.865	0	cDC1	Rpl32
Rpl94	0	1.189715	0.979	0.874	0	cDC1	Rpl9
Rps204	0	1.123054	0.982	0.879	0	cDC1	Rps20
Rps4x4	0	1.126243	0.978	0.878	0	cDC1	Rps4x
Rpl114	0	1.033756	0.974	0.875	0	cDC1	Rpl11
Rps164	0	1.266471	0.976	0.88	0	cDC1	Rps16
Rpl235	0	1.092518	0.975	0.881	0	cDC1	Rpl23
Rps3a15	0	1.18919	0.974	0.882	0	cDC1	Rps3a1
Rpl195	0	1.189646	0.976	0.889	0	cDC1	Rpl19
Rps104	0	1.027765	0.976	0.89	0	cDC1	Rps10
Rps85	0	1.089804	0.982	0.899	0	cDC1	Rps8
Rpl18a4	0	1.160784	0.981	0.899	0	cDC1	Rpl18a

Tpt15	0	1.121592	0.978	0.896	0	cDC1	Tpt1
Rps115	0	1.341763	0.971	0.894	0	cDC1	Rps11
Rps94	0	1.069749	0.984	0.91	0	cDC1	Rps9
Rps27a4	0	1.100785	0.98	0.907	0	cDC1	Rps27a
Rpl134	0	1.313793	0.985	0.918	0	cDC1	Rpl13
Rps245	0	1.187977	0.983	0.928	0	cDC1	Rps24
Actb4	0	1.232638	0.993	0.958	0	cDC1	Actb
Ifitm62	4.56E-307	0.975537	0.295	0.047	1.12E-302	cDC1	Ifitm6
Rps234	6.54E-307	0.978754	0.968	0.853	1.60E-302	cDC1	Rps23
Alox5ap4	9.98E-307	1.291345	0.932	0.643	2.44E-302	cDC1	Alox5ap
4930523C(	1.90E-306	2.163414	0.302	0.051	4.66E-302	cDC1	4930523C07Rik
Rpl10a4	1.83E-304	1.016369	0.968	0.813	4.48E-300	cDC1	Rpl10a
Rpl105	3.34E-303	0.969951	0.964	0.838	8.18E-299	cDC1	Rpl10
Ccdc88a2	4.26E-301	1.663365	0.589	0.17	1.04E-296	cDC1	Ccdc88a
Eef1a13	3.37E-299	0.869636	0.99	0.943	8.24E-295	cDC1	Eef1a1
Scimp1	1.33E-297	2.09668	0.397	0.087	3.26E-293	cDC1	Scimp
Syngr22	2.00E-297	1.626784	0.909	0.562	4.89E-293	cDC1	Syngr2
Ifitm35	4.52E-296	2.225071	0.834	0.423	1.11E-291	cDC1	Ifitm3
Plec1	1.79E-295	2.15617	0.295	0.051	4.39E-291	cDC1	Plec
Wdfy41	1.28E-294	2.361543	0.705	0.282	3.14E-290	cDC1	Wdfy4
Naca5	8.54E-290	1.134895	0.94	0.7	2.09E-285	cDC1	Naca
Arpc23	3.16E-286	1.088703	0.951	0.704	7.74E-282	cDC1	Arpc2
Rpl314	8.39E-286	0.987239	0.963	0.791	2.05E-281	cDC1	Rpl3
Cd402	6.35E-285	2.399624	0.314	0.059	1.56E-280	cDC1	Cd40
Tmsb4x4	1.36E-284	0.971945	0.993	0.967	3.32E-280	cDC1	Tmsb4x
Eif3k3	1.08E-283	1.308826	0.882	0.478	2.64E-279	cDC1	Eif3k
Ctnna1	1.82E-281	1.629717	0.531	0.148	4.46E-277	cDC1	Ctnna1
Cycs2	7.78E-280	1.874384	0.723	0.296	1.91E-275	cDC1	Cycs
Psmb84	1.75E-277	1.366207	0.914	0.595	4.29E-273	cDC1	Psmb8
Rpl45	3.07E-276	1.249599	0.905	0.593	7.52E-272	cDC1	Rpl4
Clic43	5.29E-276	1.389258	0.634	0.203	1.29E-271	cDC1	Clic4
Myl64	1.88E-274	1.194001	0.929	0.603	4.60E-270	cDC1	Myl6
Eif3f4	6.93E-274	1.225338	0.915	0.583	1.70E-269	cDC1	Eif3f
Vrk1	2.92E-272	1.577516	0.64	0.216	7.14E-268	cDC1	Vrk1
Rpl35a5	1.69E-270	0.896005	0.971	0.884	4.13E-266	cDC1	Rpl35a
Rpl13a4	3.76E-269	0.948993	0.964	0.79	9.22E-265	cDC1	Rpl13a
Taldo14	5.35E-269	1.475003	0.858	0.475	1.31E-264	cDC1	Taldo1
Rpl303	1.40E-267	0.861982	0.979	0.896	3.44E-263	cDC1	Rpl30
Lsm44	1.44E-266	1.405854	0.69	0.249	3.52E-262	cDC1	Lsm4
Rpl213	2.20E-266	0.824579	0.974	0.89	5.39E-262	cDC1	Rpl21
Trps13	2.88E-266	1.459654	0.552	0.16	7.04E-262	cDC1	Trps1
Smdt14	5.93E-265	1.324005	0.849	0.409	1.45E-260	cDC1	Smdt1
Rpl36al4	1.36E-263	1.16178	0.918	0.644	3.34E-259	cDC1	Rpl36al
Siah2	1.44E-263	1.84911	0.346	0.073	3.53E-259	cDC1	Siah2

Mrpl333	2.32E-263	1.288825	0.703	0.257	5.69E-259	cDC1	Mrpl33
Serpina3g1	6.31E-263	2.943391	0.268	0.047	1.54E-258	cDC1	Serpina3g
Sec61b4	6.24E-262	1.471419	0.849	0.457	1.53E-257	cDC1	Sec61b
Fdps	1.52E-261	2.235598	0.378	0.088	3.71E-257	cDC1	Fdps
Csrp1	1.19E-260	1.642916	0.344	0.073	2.92E-256	cDC1	Csrp1
Rpl365	1.76E-260	1.074299	0.954	0.791	4.31E-256	cDC1	Rpl36
Rpl294	5.62E-260	0.942978	0.952	0.798	1.38E-255	cDC1	Rpl29
Eif5a4	3.82E-256	1.348638	0.861	0.485	9.37E-252	cDC1	Eif5a
Slk2	2.05E-255	1.448066	0.527	0.153	5.02E-251	cDC1	Slk
Mrpl304	2.50E-255	1.338368	0.72	0.275	6.12E-251	cDC1	Mrpl30
Cd475	5.98E-255	1.290981	0.874	0.452	1.46E-250	cDC1	Cd47
Snrpf4	9.20E-255	1.429298	0.694	0.263	2.25E-250	cDC1	Snrpf
Rplp24	6.30E-254	0.945303	0.965	0.799	1.54E-249	cDC1	Rplp2
Nhp22	3.16E-253	1.438725	0.542	0.165	7.73E-249	cDC1	Nhp2
Serf25	3.05E-252	0.981163	0.944	0.756	7.47E-248	cDC1	Serf2
Fau4	3.04E-251	0.775002	0.983	0.939	7.44E-247	cDC1	Fau
Rpl54	4.97E-251	1.135667	0.912	0.61	1.22E-246	cDC1	Rpl5
Rpl7a3	1.64E-250	0.955787	0.944	0.762	4.02E-246	cDC1	Rpl7a
Rpl27a4	4.81E-250	0.832993	0.97	0.889	1.18E-245	cDC1	Rpl27a
H2-DMa3	3.07E-249	1.08495	0.93	0.662	7.51E-245	cDC1	H2-DMa
Nav11	7.76E-249	1.441354	0.487	0.133	1.90E-244	cDC1	Nav1
Rpl23a4	6.28E-247	1.215289	0.878	0.498	1.54E-242	cDC1	Rpl23a
Fgr3	4.16E-246	1.389734	0.4	0.098	1.02E-241	cDC1	Fgr
Npm13	9.82E-244	1.197071	0.888	0.541	2.40E-239	cDC1	Npm1
Ly6c23	3.62E-243	1.944731	0.288	0.055	8.86E-239	cDC1	Ly6c2
Nudt211	1.81E-241	1.42537	0.542	0.169	4.44E-237	cDC1	Nudt21
Hspa84	7.88E-237	1.245166	0.943	0.738	1.93E-232	cDC1	Hspa8
St8sia43	2.29E-235	1.521647	0.39	0.095	5.60E-231	cDC1	St8sia4
Rpl415	2.28E-232	0.91908	0.981	0.902	5.59E-228	cDC1	Rpl41
Arf62	3.46E-232	1.363668	0.678	0.26	8.47E-228	cDC1	Arf6
Nsa24	2.30E-231	1.221092	0.853	0.441	5.64E-227	cDC1	Nsa2
Rpl124	1.43E-230	1.008541	0.93	0.693	3.50E-226	cDC1	Rpl12
Diaph13	1.86E-230	1.37132	0.473	0.135	4.55E-226	cDC1	Diaph1
Eif3e3	1.72E-228	1.300725	0.72	0.298	4.22E-224	cDC1	Eif3e
Pitpna3	2.13E-226	1.243553	0.867	0.474	5.21E-222	cDC1	Pitpna
Ifi2092	4.62E-226	1.609028	0.407	0.108	1.13E-221	cDC1	Ifi209
Rbm34	2.41E-222	1.174277	0.874	0.53	5.90E-218	cDC1	Rbm3
Nme26	2.75E-222	1.102272	0.905	0.596	6.74E-218	cDC1	Nme2
Cpne21	4.21E-222	1.730713	0.276	0.055	1.03E-217	cDC1	Cpne2
Arpc1b5	4.55E-222	0.95262	0.936	0.699	1.11E-217	cDC1	Arpc1b
Sri4	4.70E-222	1.232634	0.741	0.314	1.15E-217	cDC1	Sri
Mpp6	1.34E-219	1.18962	0.566	0.185	3.28E-215	cDC1	Mpp6
Ldha5	2.98E-219	1.21969	0.824	0.437	7.31E-215	cDC1	Ldha
Itgax3	6.90E-219	1.16839	0.526	0.163	1.69E-214	cDC1	Itgax

Mrpl543	3.10E-217	1.32532	0.561	0.188	7.59E-213	cDC1	Mrpl54
Fbl2	1.64E-216	1.37344	0.645	0.25	4.03E-212	cDC1	Fbl
Samhd14	6.56E-216	1.360277	0.799	0.385	1.61E-211	cDC1	Samhd1
Irf74	1.37E-215	2.04289	0.532	0.184	3.36E-211	cDC1	Irf7
Psma74	3.11E-215	1.24435	0.807	0.406	7.63E-211	cDC1	Psma7
Hsp90ab14	5.55E-214	0.905325	0.949	0.737	1.36E-209	cDC1	Hsp90ab1
Prelid15	1.62E-212	1.199419	0.755	0.328	3.97E-208	cDC1	Prelid1
Eif3h4	9.57E-211	1.112538	0.857	0.48	2.34E-206	cDC1	Eif3h
Rin33	1.60E-210	1.321956	0.626	0.229	3.93E-206	cDC1	Rin3
Plxnc1	4.22E-210	1.746993	0.268	0.054	1.03E-205	cDC1	Plxnc1
Eef1g3	1.50E-208	1.194402	0.775	0.376	3.67E-204	cDC1	Eef1g
Sfxn3	4.26E-208	1.730961	0.283	0.061	1.04E-203	cDC1	Sfxn3
Rpl394	4.82E-207	0.966589	0.952	0.819	1.18E-202	cDC1	Rpl39
Psme14	8.84E-207	1.203983	0.906	0.599	2.16E-202	cDC1	Psme1
CAAA0114	2.32E-206	1.19519	0.809	0.394	5.68E-202	cDC1	CAAA01147332.1
Ppia4	2.24E-205	0.758498	0.973	0.88	5.48E-201	cDC1	Ppia
Ifi2112	2.95E-205	1.967504	0.332	0.083	7.22E-201	cDC1	Ifi211
Set2	3.54E-205	1.266671	0.712	0.311	8.66E-201	cDC1	Set
Npm31	5.01E-205	1.440618	0.425	0.123	1.23E-200	cDC1	Npm3
Isg20	5.95E-205	2.019756	0.268	0.056	1.46E-200	cDC1	Isg20
Rtl8c	1.47E-203	2.750762	0.334	0.089	3.61E-199	cDC1	Rtl8c
Rps154	7.36E-203	1.044706	0.893	0.562	1.80E-198	cDC1	Rps15
Psma43	2.71E-202	1.302828	0.613	0.235	6.65E-198	cDC1	Psma4
Lbh	4.20E-202	1.530142	0.332	0.081	1.03E-197	cDC1	Lbh
Nol72	9.43E-202	1.242425	0.643	0.253	2.31E-197	cDC1	Nol7
Ldlr	1.92E-201	1.940621	0.272	0.058	4.69E-197	cDC1	Ldlr
H132	3.52E-201	1.208552	0.717	0.316	8.62E-197	cDC1	H13
Scand14	3.72E-201	1.13788	0.81	0.409	9.12E-197	cDC1	Scand1
Sem14	1.67E-200	0.986858	0.893	0.545	4.08E-196	cDC1	Sem1
Taf104	5.29E-200	1.145159	0.77	0.359	1.29E-195	cDC1	Taf10
Ppa1	7.95E-199	1.829413	0.29	0.066	1.95E-194	cDC1	Ppa1
Srgn3	1.10E-196	1.085419	0.885	0.547	2.70E-192	cDC1	Srgn
Psmb93	3.43E-196	1.378229	0.821	0.477	8.40E-192	cDC1	Psmb9
Ppp1r11	1.22E-195	1.413442	0.616	0.251	2.98E-191	cDC1	Ppp1r11
Esyt12	2.44E-194	1.325189	0.436	0.13	5.97E-190	cDC1	Esyt1
Siva12	4.88E-194	1.404701	0.375	0.102	1.20E-189	cDC1	Siva1
Arf53	4.95E-194	1.037496	0.859	0.475	1.21E-189	cDC1	Arf5
Rala	3.91E-193	1.202769	0.669	0.28	9.58E-189	cDC1	Rala
Myl12b3	4.04E-193	0.935307	0.909	0.629	9.89E-189	cDC1	Myl12b
Zmiz21	4.32E-193	1.420976	0.431	0.13	1.06E-188	cDC1	Zmiz2
Pirb4	6.59E-193	1.1986	0.47	0.147	1.61E-188	cDC1	Pirb
Dbnl3	7.85E-193	1.189104	0.736	0.335	1.92E-188	cDC1	Dbnl
Rpl374	2.07E-192	0.895742	0.966	0.849	5.07E-188	cDC1	Rpl37
Banf14	2.20E-192	1.196913	0.647	0.262	5.38E-188	cDC1	Banf1

Ndufa64	5.69E-192	1.044132	0.865	0.515	1.39E-187	cDC1	Ndufa6
Borcs8	1.02E-191	1.461796	0.355	0.094	2.51E-187	cDC1	Borcs8
Mrps162	1.26E-191	1.150558	0.555	0.196	3.09E-187	cDC1	Mrps16
Eif3i4	1.51E-191	1.151665	0.716	0.318	3.70E-187	cDC1	Eif3i
Snrpg3	4.31E-191	1.107078	0.806	0.422	1.05E-186	cDC1	Snrpg
Elovl5	1.60E-189	1.384366	0.383	0.106	3.91E-185	cDC1	Elovl5
Eif3m4	9.11E-189	1.125697	0.659	0.274	2.23E-184	cDC1	Eif3m
Sdad1	8.39E-188	1.502379	0.338	0.088	2.06E-183	cDC1	Sdad1
Atp5e4	1.58E-187	0.962988	0.92	0.646	3.86E-183	cDC1	Atp5e
Rps124	1.72E-187	0.709622	0.966	0.867	4.22E-183	cDC1	Rps12
Apobr1	4.87E-187	1.399955	0.289	0.067	1.19E-182	cDC1	Apobr
Akr7a5	1.20E-186	1.379628	0.383	0.108	2.95E-182	cDC1	Akr7a5
Eef24	6.42E-186	0.880688	0.928	0.718	1.57E-181	cDC1	Eef2
Arhgap181	6.88E-185	1.263768	0.45	0.141	1.69E-180	cDC1	Arhgap18
Snrpe3	1.11E-183	1.102993	0.74	0.345	2.71E-179	cDC1	Snrpe
Casp6	3.37E-183	1.568787	0.353	0.097	8.25E-179	cDC1	Casp6
Rpl385	5.87E-183	1.084445	0.93	0.698	1.44E-178	cDC1	Rpl38
Ero1lb	7.69E-183	1.626604	0.292	0.069	1.88E-178	cDC1	Ero1lb
Ranbp12	3.97E-182	1.163157	0.642	0.263	9.73E-178	cDC1	Ranbp1
Txn23	9.67E-182	1.111065	0.615	0.244	2.37E-177	cDC1	Txn2
Ifi352	1.50E-181	1.231222	0.599	0.234	3.66E-177	cDC1	Ifi35
Hnrnpa12	5.43E-181	1.134264	0.758	0.378	1.33E-176	cDC1	Hnrnpa1
Zfp593	9.99E-181	1.667507	0.285	0.068	2.45E-176	cDC1	Zfp593
Usp182	1.40E-179	1.718941	0.359	0.102	3.44E-175	cDC1	Usp18
B4galnt12	1.77E-179	1.255319	0.49	0.166	4.34E-175	cDC1	B4galnt1
Pdlim2	3.60E-179	1.395109	0.324	0.084	8.82E-175	cDC1	Pdlim2
Arpc54	4.91E-179	1.006499	0.809	0.423	1.20E-174	cDC1	Arpc5
Myl12a4	5.02E-179	0.99752	0.822	0.446	1.23E-174	cDC1	Myl12a
Znrd11	5.11E-179	1.226892	0.498	0.173	1.25E-174	cDC1	Znrd1
Aph1c	6.33E-179	1.647417	0.268	0.061	1.55E-174	cDC1	Aph1c
Serp14	6.84E-179	1.167668	0.741	0.354	1.68E-174	cDC1	Serp1
Sec61g4	9.67E-179	1.141149	0.804	0.428	2.37E-174	cDC1	Sec61g
Snrpd12	1.43E-177	1.126458	0.559	0.206	3.51E-173	cDC1	Snrpd1
Snrpd24	1.40E-176	1.124395	0.629	0.257	3.43E-172	cDC1	Snrpd2
Stk17b3	1.84E-176	1.097341	0.492	0.165	4.51E-172	cDC1	Stk17b
Pde4b3	3.91E-176	1.245065	0.583	0.226	9.58E-172	cDC1	Pde4b
Ybx14	1.06E-175	0.942305	0.888	0.566	2.61E-171	cDC1	Ybx1
Rwdd12	1.15E-175	1.140443	0.527	0.191	2.81E-171	cDC1	Rwdd1
Il2rg5	1.40E-175	1.331769	0.558	0.215	3.43E-171	cDC1	Il2rg
Hnrnpa32	4.20E-175	1.064127	0.809	0.448	1.03E-170	cDC1	Hnrnpa3
Nr4a11	3.66E-174	1.21087	0.299	0.074	8.96E-170	cDC1	Nr4a1
Hspb11	3.76E-174	1.520564	0.298	0.074	9.21E-170	cDC1	Hspb11
Cfl15	8.84E-174	0.784257	0.948	0.761	2.17E-169	cDC1	Cfl1
Psme24	2.68E-173	1.135786	0.859	0.514	6.56E-169	cDC1	Psme2

Atp5l5	6.76E-173	0.903006	0.879	0.563	1.66E-168	cDC1	Atp5l
Rps27l4	1.22E-172	1.001171	0.741	0.352	2.98E-168	cDC1	Rps27l
Hsdl2	1.69E-172	1.409548	0.268	0.062	4.14E-168	cDC1	Hsdl2
Rpl354	2.65E-172	0.907669	0.941	0.773	6.49E-168	cDC1	Rpl35
Socs12	2.71E-172	2.069676	0.319	0.087	6.65E-168	cDC1	Socs1
Abhd17a3	3.11E-172	1.130183	0.579	0.225	7.61E-168	cDC1	Abhd17a
Crlf23	4.24E-172	1.017724	0.663	0.27	1.04E-167	cDC1	Crlf2
Alyref3	8.27E-172	1.172831	0.545	0.204	2.02E-167	cDC1	Alyref
Cox7b5	3.98E-171	1.045239	0.748	0.36	9.74E-167	cDC1	Cox7b
Cast3	1.02E-170	1.288059	0.379	0.111	2.49E-166	cDC1	Cast
Snrpd33	2.47E-170	1.127531	0.594	0.235	6.05E-166	cDC1	Snrpd3
Arpc35	6.60E-170	0.889886	0.905	0.636	1.62E-165	cDC1	Arpc3
Pgls3	8.28E-169	1.037082	0.759	0.369	2.03E-164	cDC1	Pgls
1110008P1	9.17E-169	1.187855	0.306	0.079	2.24E-164	cDC1	1110008P14Rik
Pak1	2.47E-167	1.060666	0.61	0.248	6.05E-163	cDC1	Pak1
Rps274	6.57E-166	0.909734	0.944	0.78	1.61E-161	cDC1	Rps27
Vasp2	1.05E-165	1.100955	0.685	0.311	2.56E-161	cDC1	Vasp
Sp1004	1.12E-165	1.149261	0.637	0.27	2.74E-161	cDC1	Sp100
Grk31	1.29E-165	1.147576	0.427	0.136	3.15E-161	cDC1	Grk3
Cdk2ap23	8.88E-165	1.061866	0.675	0.304	2.17E-160	cDC1	Cdk2ap2
Me2	1.49E-164	1.279321	0.332	0.092	3.65E-160	cDC1	Me2
Abracl3	1.87E-164	1.045495	0.657	0.284	4.58E-160	cDC1	Abracl
Isg152	4.57E-164	1.811509	0.513	0.198	1.12E-159	cDC1	Isg15
Rpl36a4	9.77E-164	0.901278	0.909	0.647	2.39E-159	cDC1	Rpl36a
Atp5h4	8.91E-163	0.882551	0.875	0.547	2.18E-158	cDC1	Atp5h
Rraga1	1.27E-162	1.171639	0.434	0.144	3.11E-158	cDC1	Rraga
Arrdc41	7.12E-162	1.521498	0.316	0.087	1.74E-157	cDC1	Arrdc4
Tomm54	3.58E-161	1.062143	0.523	0.195	8.76E-157	cDC1	Tomm5
Naga1	4.58E-161	1.370958	0.648	0.311	1.12E-156	cDC1	Naga
Tnni2	3.95E-160	1.800648	0.276	0.07	9.67E-156	cDC1	Tnni2
Ucp24	7.48E-160	0.900993	0.929	0.724	1.83E-155	cDC1	Ucp2
Cct54	1.82E-159	1.032435	0.626	0.263	4.45E-155	cDC1	Cct5
Tle54	3.99E-159	0.942603	0.784	0.397	9.78E-155	cDC1	Tle5
Ccdc125	4.49E-159	1.011447	0.81	0.446	1.10E-154	cDC1	Ccdc12
Dynll14	1.11E-158	0.984194	0.793	0.412	2.73E-154	cDC1	Dynll1
Serbp13	4.56E-158	0.930312	0.78	0.399	1.12E-153	cDC1	Serbp1
Sigmar11	6.35E-158	1.213531	0.421	0.139	1.56E-153	cDC1	Sigmar1
Sulf21	1.71E-157	1.53543	0.337	0.099	4.18E-153	cDC1	Sulf2
Fgl24	2.46E-157	1.111453	0.618	0.267	6.02E-153	cDC1	Fgl2
Ncoa7	3.06E-157	1.225878	0.329	0.093	7.48E-153	cDC1	Ncoa7
Mvb12a2	3.70E-156	1.144202	0.517	0.196	9.07E-152	cDC1	Mvb12a
Clec2d4	5.33E-156	0.925307	0.625	0.258	1.31E-151	cDC1	Clec2d
Nop532	5.86E-156	1.131864	0.494	0.181	1.43E-151	cDC1	Nop53
Gm20513	1.80E-155	1.281358	0.261	0.063	4.40E-151	cDC1	Gm20513

Actn11	4.35E-155	1.509832	0.272	0.07	1.06E-150	cDC1	Actn1
Impdh22	9.37E-155	1.247561	0.461	0.166	2.29E-150	cDC1	Impdh2
Hmga12	1.03E-154	1.179995	0.415	0.137	2.53E-150	cDC1	Hmga1
Cnp2	9.71E-154	1.285438	0.467	0.169	2.38E-149	cDC1	Cnp
Tmem1343	1.65E-153	1.065767	0.594	0.248	4.05E-149	cDC1	Tmem134
Rps254	3.43E-153	0.827157	0.9	0.633	8.40E-149	cDC1	Rps25
Oaz15	5.25E-153	0.747178	0.936	0.718	1.29E-148	cDC1	Oaz1
Bcl3	6.33E-153	2.185245	0.295	0.083	1.55E-148	cDC1	Bcl3
Batf32	1.24E-152	1.273152	0.498	0.191	3.03E-148	cDC1	Batf3
Rbpj1	1.26E-151	0.580656	0.617	0.257	3.09E-147	cDC1	Rbpj
Fem1c	1.73E-151	1.186766	0.349	0.104	4.22E-147	cDC1	Fem1c
Llph3	1.74E-151	0.96768	0.574	0.23	4.26E-147	cDC1	Llph
Mdh25	2.41E-151	1.434897	0.712	0.349	5.89E-147	cDC1	Mdh2
Mob3a	4.49E-151	1.074371	0.401	0.129	1.10E-146	cDC1	Mob3a
Magohb	5.63E-151	1.512526	0.257	0.064	1.38E-146	cDC1	Magohb
Rps285	7.05E-151	0.935041	0.929	0.755	1.73E-146	cDC1	Rps28
Ptcd2	1.68E-150	1.39799	0.301	0.083	4.12E-146	cDC1	Ptcd2
Arpc5l	4.13E-150	1.12363	0.546	0.219	1.01E-145	cDC1	Arpc5l
Mkrn14	5.73E-150	0.777909	0.662	0.293	1.40E-145	cDC1	Mkrn1
Cox5a4	2.40E-149	0.922746	0.816	0.457	5.87E-145	cDC1	Cox5a
Gpi15	3.88E-149	0.913601	0.814	0.447	9.49E-145	cDC1	Gpi1
Eif4ebp14	3.31E-148	1.266082	0.515	0.203	8.11E-144	cDC1	Eif4ebp1
Atg32	4.08E-147	0.964028	0.567	0.231	9.99E-143	cDC1	Atg3
Cd443	4.20E-147	1.164394	0.504	0.194	1.03E-142	cDC1	Cd44
Rtraf4	6.85E-147	0.966099	0.776	0.413	1.68E-142	cDC1	Rtraf
Psmb13	7.12E-147	0.911668	0.773	0.396	1.74E-142	cDC1	Psmb1
Atp5o5	8.58E-147	0.853716	0.755	0.372	2.10E-142	cDC1	Atp5o
Atp5d5	1.26E-146	0.824098	0.867	0.523	3.08E-142	cDC1	Atp5d
Ran3	5.25E-146	0.954634	0.724	0.349	1.29E-141	cDC1	Ran
Dek4	6.34E-146	0.974249	0.591	0.247	1.55E-141	cDC1	Dek
Spata6	7.62E-146	1.370724	0.259	0.066	1.86E-141	cDC1	Spata6
Pmvk	3.23E-145	1.314976	0.319	0.094	7.92E-141	cDC1	Pmvk
Mpnd	5.45E-145	1.244535	0.347	0.107	1.34E-140	cDC1	Mpnd
Tma72	9.03E-145	0.927524	0.763	0.401	2.21E-140	cDC1	Tma7
Txn14	2.53E-144	1.050056	0.663	0.319	6.20E-140	cDC1	Txn1
Ifi27l2a5	2.54E-144	1.432777	0.707	0.38	6.22E-140	cDC1	Ifi27l2a
Tep11	1.76E-143	1.136796	0.515	0.205	4.32E-139	cDC1	Tep1
Lrrfip21	3.18E-143	0.975036	0.423	0.144	7.80E-139	cDC1	Lrrfip2
Csf2ra4	3.82E-143	0.883289	0.793	0.411	9.35E-139	cDC1	Csf2ra
Gas52	9.02E-143	1.002148	0.871	0.552	2.21E-138	cDC1	Gas5
Glipr12	1.09E-142	1.256454	0.347	0.109	2.66E-138	cDC1	Glipr1
Gltp4	1.63E-142	0.957302	0.736	0.373	3.99E-138	cDC1	Gltp
Ifi2074	1.81E-142	0.926497	0.473	0.172	4.43E-138	cDC1	Ifi207
Acadl1	3.34E-142	1.416433	0.465	0.182	8.18E-138	cDC1	Acadl

H2afx	3.95E-142	1.303661	0.253	0.064	9.67E-138	cDC1	H2afx
Ndufb65	4.72E-142	0.894888	0.604	0.256	1.16E-137	cDC1	Ndufb6
Tomm74	6.74E-142	0.923657	0.747	0.38	1.65E-137	cDC1	Tomm7
Baz1a3	8.94E-142	0.963188	0.54	0.215	2.19E-137	cDC1	Baz1a
Rpl37a5	2.83E-141	0.716938	0.966	0.852	6.93E-137	cDC1	Rpl37a
Zbp15	6.75E-141	1.44557	0.458	0.175	1.65E-136	cDC1	Zbp1
Uqcrfs14	9.14E-141	0.944876	0.67	0.312	2.24E-136	cDC1	Uqcrfs1
Nop103	9.16E-141	0.90974	0.639	0.288	2.24E-136	cDC1	Nop10
Rbms14	2.30E-140	0.837317	0.56	0.223	5.64E-136	cDC1	Rbms1
Anp32e	4.62E-140	1.003156	0.453	0.164	1.13E-135	cDC1	Anp32e
Rbm171	5.70E-140	0.994037	0.465	0.172	1.40E-135	cDC1	Rbm17
Slamf83	6.43E-140	1.157067	0.492	0.19	1.58E-135	cDC1	Slamf8
Ciao2a3	1.90E-139	0.998579	0.577	0.244	4.65E-135	cDC1	Ciao2a
Lsm72	5.37E-139	0.859648	0.553	0.222	1.31E-134	cDC1	Lsm7
Cox5b5	7.47E-139	0.814183	0.866	0.54	1.83E-134	cDC1	Cox5b
Bcl7c2	9.42E-139	1.075912	0.399	0.136	2.31E-134	cDC1	Bcl7c
2410006H1	1.08E-138	1.059459	0.817	0.482	2.64E-134	cDC1	2410006H16Rik
Selenow6	3.99E-138	0.820516	0.84	0.454	9.78E-134	cDC1	Selenow
Rab431	7.27E-138	1.021191	0.658	0.309	1.78E-133	cDC1	Rab43
Psmd83	1.57E-137	0.921713	0.673	0.311	3.83E-133	cDC1	Psmd8
Akt31	1.76E-137	0.890158	0.484	0.178	4.32E-133	cDC1	Akt3
Glud12	2.42E-137	0.877207	0.699	0.335	5.93E-133	cDC1	Glud1
Bcl61	2.71E-137	0.970898	0.424	0.148	6.63E-133	cDC1	Bcl6
Hivep21	2.98E-137	1.649938	0.351	0.114	7.29E-133	cDC1	Hivep2
Mrpl524	3.87E-137	0.920925	0.799	0.467	9.47E-133	cDC1	Mrpl52
Rnh14	7.25E-137	0.955679	0.705	0.343	1.77E-132	cDC1	Rnh1
Hint14	1.41E-136	0.782796	0.862	0.521	3.45E-132	cDC1	Hint1
Magoh	1.49E-136	1.115707	0.347	0.11	3.64E-132	cDC1	Magoh
Psmb44	2.07E-136	0.922682	0.619	0.276	5.06E-132	cDC1	Psmb4
Sap303	2.97E-136	1.141109	0.373	0.123	7.27E-132	cDC1	Sap30
Eif1ax1	2.52E-135	1.054239	0.376	0.125	6.18E-131	cDC1	Eif1ax
Rab8a3	3.60E-134	1.045219	0.501	0.199	8.83E-130	cDC1	Rab8a
Ndufb1-ps3	9.03E-133	1.014222	0.627	0.293	2.21E-128	cDC1	Ndufb1-ps
Snu133	1.02E-132	0.831822	0.673	0.31	2.49E-128	cDC1	Snu13
H2-Oa4	1.41E-132	0.808907	0.681	0.321	3.45E-128	cDC1	H2-Oa
Mbd25	2.88E-131	0.929691	0.58	0.249	7.06E-127	cDC1	Mbd2
Sh3pxd2b	3.73E-131	1.651254	0.253	0.069	9.14E-127	cDC1	Sh3pxd2b
Cct83	7.46E-131	0.949896	0.577	0.252	1.83E-126	cDC1	Cct8
Psmb34	1.05E-130	0.880201	0.746	0.395	2.57E-126	cDC1	Psmb3
Prkag2	1.63E-130	1.162878	0.276	0.077	4.00E-126	cDC1	Prkag2
Sms	2.74E-130	1.009913	0.262	0.071	6.72E-126	cDC1	Sms
Calm34	3.32E-130	0.788973	0.683	0.327	8.12E-126	cDC1	Calm3
Ppp1ca3	4.67E-130	0.823179	0.809	0.457	1.14E-125	cDC1	Ppp1ca
Nt5c3	5.02E-130	0.975818	0.496	0.198	1.23E-125	cDC1	Nt5c

Dnajc153	8.05E-130	0.899788	0.502	0.198	1.97E-125	cDC1	Dnajc15
Timm10b3	1.54E-129	0.905112	0.778	0.427	3.78E-125	cDC1	Timm10b
Mta31	2.38E-129	0.838993	0.438	0.16	5.82E-125	cDC1	Mta3
Mrps143	3.08E-129	0.877826	0.567	0.243	7.55E-125	cDC1	Mrps14
Pdcd63	4.47E-129	0.846564	0.505	0.201	1.10E-124	cDC1	Pdcd6
Arhgdib3	6.45E-129	0.723834	0.894	0.614	1.58E-124	cDC1	Arhgdib
Degs13	8.32E-129	0.886847	0.564	0.238	2.04E-124	cDC1	Degs1
Cct22	3.16E-128	0.862886	0.634	0.293	7.74E-124	cDC1	Cct2
Bak12	3.84E-128	1.097285	0.376	0.13	9.40E-124	cDC1	Bak1
Cdc42se13	6.76E-128	0.796497	0.62	0.276	1.65E-123	cDC1	Cdc42se1
Snx203	1.67E-127	0.925312	0.67	0.32	4.08E-123	cDC1	Snx20
Ndufv24	2.15E-127	0.817591	0.639	0.289	5.25E-123	cDC1	Ndufv2
Mrpl211	2.58E-127	0.960124	0.393	0.138	6.32E-123	cDC1	Mrpl21
Sla2	2.67E-127	0.893083	0.691	0.335	6.54E-123	cDC1	Sla
Pin41	5.39E-127	0.888047	0.408	0.146	1.32E-122	cDC1	Pin4
Mrpl572	5.88E-127	0.953277	0.508	0.208	1.44E-122	cDC1	Mrpl57
Emc61	9.36E-127	0.87417	0.565	0.241	2.29E-122	cDC1	Emc6
Rpl315	3.27E-126	0.840594	0.849	0.522	8.00E-122	cDC1	Rpl31
Lta4h1	5.90E-126	0.987397	0.398	0.142	1.45E-121	cDC1	Lta4h
Myd881	1.46E-125	1.142831	0.375	0.131	3.57E-121	cDC1	Myd88
U2af12	2.24E-125	0.878335	0.613	0.279	5.48E-121	cDC1	U2af1
Gpsm33	4.56E-125	0.83872	0.728	0.381	1.12E-120	cDC1	Gpsm3
Arhgap93	4.67E-125	0.984885	0.45	0.173	1.14E-120	cDC1	Arhgap9
Prr5l	1.67E-124	1.153215	0.282	0.083	4.09E-120	cDC1	Prr5l
Sf3b53	1.85E-124	0.847127	0.639	0.299	4.52E-120	cDC1	Sf3b5
Sf3b62	4.46E-124	0.805454	0.663	0.314	1.09E-119	cDC1	Sf3b6
Prdx24	5.08E-124	0.775369	0.693	0.345	1.24E-119	cDC1	Prdx2
Nedd83	1.86E-123	0.813456	0.79	0.446	4.54E-119	cDC1	Nedd8
Hint3	2.68E-123	1.019192	0.33	0.106	6.56E-119	cDC1	Hint3
Cdkn1a1	2.81E-123	1.854219	0.296	0.094	6.89E-119	cDC1	Cdkn1a
Psma54	2.85E-123	0.86418	0.646	0.302	6.97E-119	cDC1	Psma5
Hnrnpab3	4.74E-123	0.855869	0.679	0.334	1.16E-118	cDC1	Hnrnpab
Ehbp1l12	5.25E-123	0.943558	0.485	0.195	1.29E-118	cDC1	Ehbp1l1
Gnb23	3.29E-122	0.773486	0.863	0.578	8.05E-118	cDC1	Gnb2
Psmb53	9.40E-122	0.830593	0.678	0.333	2.30E-117	cDC1	Psmb5
Eif3d2	9.51E-122	0.884459	0.498	0.203	2.33E-117	cDC1	Eif3d
Lsm51	6.20E-121	0.947543	0.365	0.126	1.52E-116	cDC1	Lsm5
Hnrnpf2	2.67E-120	0.813698	0.823	0.507	6.53E-116	cDC1	Hnrnpf
Ier24	2.73E-120	0.48582	0.734	0.347	6.68E-116	cDC1	Ier2
Pafah1b31	3.28E-120	1.127955	0.384	0.14	8.03E-116	cDC1	Pafah1b3
Fam241a2	3.94E-120	1.216293	0.29	0.089	9.66E-116	cDC1	Fam241a
Pa2g42	4.54E-120	0.884098	0.539	0.232	1.11E-115	cDC1	Pa2g4
Nop16	8.17E-120	1.123884	0.315	0.101	2.00E-115	cDC1	Nop16
Ube2s5	1.44E-119	0.820579	0.568	0.249	3.54E-115	cDC1	Ube2s

Arl2bp	1.76E-119	1.03079	0.28	0.084	4.31E-115	cDC1	Arl2bp
Eif3g2	2.43E-119	1.003587	0.453	0.179	5.94E-115	cDC1	Eif3g
S100a134	5.51E-119	0.851212	0.522	0.221	1.35E-114	cDC1	S100a13
Ly6a4	5.86E-119	1.834133	0.527	0.256	1.44E-114	cDC1	Ly6a
Pomp5	6.16E-119	0.836084	0.847	0.545	1.51E-114	cDC1	Pomp
Rpp211	1.14E-118	0.902519	0.398	0.145	2.79E-114	cDC1	Rpp21
Snx33	1.82E-118	0.753248	0.784	0.438	4.46E-114	cDC1	Snx3
Got21	2.62E-118	1.028955	0.373	0.134	6.42E-114	cDC1	Got2
Srsf31	2.67E-118	0.830807	0.743	0.398	6.53E-114	cDC1	Srsf3
Gtpbp4	3.18E-118	0.969665	0.4	0.147	7.78E-114	cDC1	Gtpbp4
1810058I2	5.74E-118	0.705838	0.582	0.257	1.40E-113	cDC1	1810058I24Rik
Emg12	7.66E-118	0.762507	0.573	0.251	1.88E-113	cDC1	Emg1
Hnrnpul23	1.37E-117	0.840167	0.532	0.229	3.35E-113	cDC1	Hnrnpul2
H2afj4	1.37E-117	0.777523	0.733	0.382	3.36E-113	cDC1	H2afj
Chchd13	3.39E-117	0.831657	0.488	0.198	8.30E-113	cDC1	Chchd1
Dctn34	3.47E-117	0.723628	0.602	0.271	8.50E-113	cDC1	Dctn3
Glrx7	4.47E-117	1.031935	0.398	0.148	1.09E-112	cDC1	Glrx
Guk1	6.59E-117	0.886287	0.371	0.131	1.61E-112	cDC1	Guk1
Mcl14	1.41E-116	0.731789	0.846	0.535	3.44E-112	cDC1	Mcl1
Eif2b21	5.07E-116	1.124329	0.327	0.11	1.24E-111	cDC1	Eif2b2
Rinl1	4.45E-115	0.996748	0.318	0.104	1.09E-110	cDC1	Rinl
Sumo23	8.57E-115	0.75179	0.821	0.506	2.10E-110	cDC1	Sumo2
Grpel11	1.95E-114	0.869833	0.455	0.181	4.77E-110	cDC1	Grpel1
Mrpl151	3.52E-114	0.844797	0.393	0.145	8.62E-110	cDC1	Mrpl15
Tap12	8.30E-114	0.911134	0.714	0.384	2.03E-109	cDC1	Tap1
Pole41	9.85E-114	0.870049	0.418	0.16	2.41E-109	cDC1	Pole4
G3bp1	1.34E-113	0.830497	0.559	0.25	3.27E-109	cDC1	G3bp1
Cass4	1.40E-113	1.177827	0.318	0.106	3.42E-109	cDC1	Cass4
Ube2l62	1.52E-113	1.005031	0.411	0.161	3.73E-109	cDC1	Ube2l6
Dnajc81	2.08E-113	0.819715	0.562	0.252	5.10E-109	cDC1	Dnajc8
Polr1d3	2.82E-113	0.809947	0.747	0.417	6.90E-109	cDC1	Polr1d
Nab12	4.95E-112	0.898759	0.36	0.128	1.21E-107	cDC1	Nab1
Atp5g24	8.32E-112	0.641063	0.91	0.684	2.04E-107	cDC1	Atp5g2
Mri1	1.57E-111	0.944305	0.303	0.098	3.84E-107	cDC1	Mri1
Tomm224	6.19E-111	0.733759	0.663	0.321	1.52E-106	cDC1	Tomm22
Jpt13	6.68E-111	0.732846	0.701	0.353	1.64E-106	cDC1	Jpt1
Hnrnpd2	7.97E-111	0.714457	0.67	0.326	1.95E-106	cDC1	Hnrnpd
Nme15	1.08E-110	0.799739	0.566	0.257	2.63E-106	cDC1	Nme1
Cdc14a	1.27E-110	1.240258	0.272	0.084	3.11E-106	cDC1	Cdc14a
Rps175	2.09E-110	0.716106	0.854	0.559	5.13E-106	cDC1	Rps17
Cct73	2.61E-110	0.818829	0.561	0.256	6.40E-106	cDC1	Cct7
Uqcrq4	4.10E-110	0.768055	0.78	0.432	1.00E-105	cDC1	Uqcrq
Esd4	4.56E-110	0.69144	0.661	0.32	1.12E-105	cDC1	Esd
Ebna1bp21	6.17E-110	0.950419	0.366	0.133	1.51E-105	cDC1	Ebna1bp2

Il3ra1	6.37E-110	1.426785	0.25	0.075	1.56E-105	cDC1	Il3ra
Bcat2	8.93E-110	0.998441	0.323	0.111	2.19E-105	cDC1	Bcat2
Ccdc85b	1.53E-109	0.841306	0.353	0.125	3.74E-105	cDC1	Ccdc85b
Psma24	2.69E-109	0.76255	0.692	0.351	6.58E-105	cDC1	Psma2
Atp5f13	3.92E-109	0.714016	0.807	0.478	9.61E-105	cDC1	Atp5f1
Supt4a3	4.92E-109	0.75719	0.629	0.301	1.20E-104	cDC1	Supt4a
Rexo23	5.59E-109	0.823263	0.515	0.221	1.37E-104	cDC1	Rexo2
Tmem1604	6.64E-109	0.724151	0.618	0.287	1.63E-104	cDC1	Tmem160
Ywhaz2	9.38E-109	0.715558	0.855	0.554	2.30E-104	cDC1	Ywhaz
Nfkbie	1.12E-108	1.162886	0.299	0.099	2.74E-104	cDC1	Nfkbie
Irf53	1.56E-108	0.829084	0.778	0.465	3.83E-104	cDC1	Irf5
C1qbp1	2.19E-108	0.801396	0.582	0.27	5.35E-104	cDC1	C1qbp
Ndufb114	3.04E-108	0.755831	0.753	0.425	7.46E-104	cDC1	Ndufb11
Rtca	5.86E-108	0.951947	0.284	0.091	1.43E-103	cDC1	Rtca
Churc12	7.29E-108	0.880485	0.411	0.16	1.78E-103	cDC1	Churc1
Uba524	1.11E-107	0.867133	0.769	0.455	2.71E-103	cDC1	Uba52
Cyb5r41	1.73E-107	0.717357	0.393	0.147	4.25E-103	cDC1	Cyb5r4
Cct42	2.07E-107	0.768574	0.624	0.302	5.06E-103	cDC1	Cct4
Nop58	2.15E-107	1.046595	0.298	0.098	5.27E-103	cDC1	Nop58
Smpdl3a2	2.97E-107	1.062695	0.374	0.143	7.26E-103	cDC1	Smpdl3a
Srp94	7.97E-107	0.729549	0.702	0.363	1.95E-102	cDC1	Srp9
Polr2e	9.14E-107	0.761124	0.568	0.259	2.24E-102	cDC1	Polr2e
Pdcd2	1.60E-106	1.091593	0.283	0.091	3.91E-102	cDC1	Pdcd2
Rbm8a1	4.12E-106	0.837487	0.451	0.185	1.01E-101	cDC1	Rbm8a
Eif61	5.93E-106	0.835218	0.491	0.21	1.45E-101	cDC1	Eif6
Cdc373	6.20E-106	0.711074	0.651	0.319	1.52E-101	cDC1	Cdc37
Atp5c13	8.55E-106	0.738919	0.807	0.499	2.09E-101	cDC1	Atp5c1
Prkx	9.21E-106	1.104795	0.267	0.083	2.26E-101	cDC1	Prkx
Csnk2b4	1.21E-105	0.780784	0.655	0.331	2.97E-101	cDC1	Csnk2b
Eif4b4	1.53E-105	0.724849	0.599	0.28	3.74E-101	cDC1	Eif4b
Prmt11	1.94E-105	0.813895	0.471	0.196	4.76E-101	cDC1	Prmt1
Mtpn3	2.11E-105	0.837158	0.51	0.223	5.17E-101	cDC1	Mtpn
Eid12	3.67E-105	0.695743	0.399	0.152	8.99E-101	cDC1	Eid1
Atp5b4	4.09E-105	0.741309	0.831	0.53	1.00E-100	cDC1	Atp5b
Daxx2	4.70E-105	1.243792	0.316	0.111	1.15E-100	cDC1	Daxx
1810037I1	5.33E-105	0.761032	0.66	0.336	1.30E-100	cDC1	1810037I17Rik
Snrpb4	5.56E-105	0.708599	0.733	0.397	1.36E-100	cDC1	Snrpb
5031439G(	5.65E-105	0.751106	0.532	0.235	1.38E-100	cDC1	5031439G07Rik
Chmp2a4	5.67E-105	0.674511	0.631	0.302	1.39E-100	cDC1	Chmp2a
Eef1akmt1	5.67E-105	1.020183	0.285	0.093	1.39E-100	cDC1	Eef1akmt1
Tcp12	5.76E-105	0.778792	0.542	0.245	1.41E-100	cDC1	Tcp1
Lcp13	6.06E-105	0.67917	0.905	0.685	1.48E-100	cDC1	Lcp1
Aurkaip16	1.32E-104	0.686435	0.566	0.257	3.22E-100	cDC1	Aurkaip1
Plin24	1.96E-104	0.76019	0.502	0.219	4.79E-100	cDC1	Plin2

Cs	1.99E-104	0.849287	0.447	0.184	4.88E-100	cDC1	Cs
Cox7a2l4	2.98E-104	0.753449	0.775	0.452	7.29E-100	cDC1	Cox7a2l
Ppdpf2	3.87E-104	0.948041	0.403	0.16	9.47E-100	cDC1	Ppdpf
Prkar2a	3.88E-104	0.951074	0.259	0.08	9.50E-100	cDC1	Prkar2a
Tbca3	5.38E-104	0.763948	0.744	0.421	1.32E-99	cDC1	Tbca
Elob6	1.15E-103	0.678829	0.839	0.519	2.81E-99	cDC1	Elob
Tmpo3	1.30E-103	0.871999	0.41	0.163	3.19E-99	cDC1	Tmpo
Eef1e1	2.53E-103	0.953682	0.281	0.091	6.19E-99	cDC1	Eef1e1
Uqcrb5	3.64E-103	0.734464	0.743	0.418	8.91E-99	cDC1	Uqcrb
Irf91	5.03E-103	0.840209	0.419	0.168	1.23E-98	cDC1	Irf9
Ramp12	5.45E-103	0.746926	0.542	0.241	1.34E-98	cDC1	Ramp1
Polr2j3	6.15E-103	0.785509	0.467	0.198	1.51E-98	cDC1	Polr2j
Gm100762	7.76E-103	1.162939	0.339	0.125	1.90E-98	cDC1	Gm10076
Arhgdia4	7.87E-103	0.689086	0.839	0.529	1.93E-98	cDC1	Arhgdia
Atp5g14	1.14E-102	0.677146	0.793	0.462	2.80E-98	cDC1	Atp5g1
Selenoh2	1.16E-102	0.953422	0.391	0.154	2.84E-98	cDC1	Selenoh
Mdp1	1.20E-102	0.877527	0.395	0.154	2.95E-98	cDC1	Mdp1
H2afv3	4.48E-102	0.720501	0.537	0.241	1.10E-97	cDC1	H2afv
Cybb5	5.49E-102	0.48845	0.527	0.23	1.34E-97	cDC1	Cybb
Snx12	6.20E-102	0.870628	0.512	0.227	1.52E-97	cDC1	Snx1
Tmem123	6.46E-102	0.532086	0.374	0.143	1.58E-97	cDC1	Tmem123
Atp5md4	9.77E-102	0.742986	0.717	0.398	2.39E-97	cDC1	Atp5md
Map2k3	1.08E-101	0.875372	0.307	0.105	2.63E-97	cDC1	Map2k3
Lypla21	1.84E-101	0.759939	0.438	0.178	4.52E-97	cDC1	Lypla2
Mrpl321	1.94E-101	0.83416	0.368	0.138	4.75E-97	cDC1	Mrpl32
Exosc5	5.62E-101	0.768789	0.465	0.197	1.38E-96	cDC1	Exosc5
Eif2s2	6.13E-101	0.837237	0.65	0.334	1.50E-96	cDC1	Eif2s2
Mrpl402	2.29E-100	0.880756	0.334	0.122	5.61E-96	cDC1	Mrpl40
Anxa7	2.36E-100	0.859768	0.371	0.142	5.79E-96	cDC1	Anxa7
Acvrl12	2.39E-100	1.081312	0.365	0.142	5.86E-96	cDC1	Acvrl1
Tctex1d21	2.53E-100	0.906942	0.382	0.149	6.20E-96	cDC1	Tctex1d2
Mrpl203	4.42E-100	0.680205	0.556	0.254	1.08E-95	cDC1	Mrpl20
Birc33	5.33E-100	0.817541	0.511	0.229	1.31E-95	cDC1	Birc3
Cd484	5.35E-100	0.784252	0.726	0.398	1.31E-95	cDC1	Cd48
Polr2h	1.28E-99	1.076403	0.276	0.091	3.14E-95	cDC1	Polr2h
Ndufb85	2.04E-99	0.681518	0.724	0.389	5.00E-95	cDC1	Ndufb8
Mrps244	2.83E-99	0.670085	0.58	0.274	6.94E-95	cDC1	Mrps24
Glrx21	7.89E-99	0.810054	0.362	0.138	1.93E-94	cDC1	Glrx2
Psmb104	8.06E-99	0.826532	0.703	0.377	1.97E-94	cDC1	Psmb10
Txndc174	9.48E-99	0.618072	0.626	0.305	2.32E-94	cDC1	Txndc17
Lsm121	6.78E-98	0.722271	0.478	0.205	1.66E-93	cDC1	Lsm12
Nubp11	9.99E-98	0.798037	0.415	0.169	2.45E-93	cDC1	Nubp1
Dnaja14	1.09E-97	0.711901	0.6	0.288	2.67E-93	cDC1	Dnaja1
Rpa3	1.37E-97	0.921716	0.252	0.079	3.35E-93	cDC1	Rpa3

Hcls13	2.70E-97	0.683162	0.743	0.407	6.61E-93	cDC1	Hcls1
Ost44	4.33E-97	0.663608	0.81	0.493	1.06E-92	cDC1	Ost4
Atp5g35	5.63E-97	0.620342	0.879	0.588	1.38E-92	cDC1	Atp5g3
Hfe3	6.54E-97	1.065665	0.532	0.258	1.60E-92	cDC1	Hfe
Dnajc22	7.04E-97	0.854763	0.402	0.164	1.72E-92	cDC1	Dnajc2
Eif14	1.27E-96	0.529462	0.967	0.831	3.10E-92	cDC1	Eif1
Ust	6.92E-96	0.929001	0.343	0.127	1.69E-91	cDC1	Ust
Trappc43	1.95E-95	0.629655	0.542	0.25	4.77E-91	cDC1	Trappc4
Rbbp71	2.10E-95	0.738117	0.475	0.209	5.14E-91	cDC1	Rbbp7
Cacybp3	2.97E-95	0.722894	0.451	0.192	7.27E-91	cDC1	Cacybp
Tkt4	3.79E-95	0.684412	0.738	0.419	9.28E-91	cDC1	Tkt
Dnajc71	4.21E-95	0.892866	0.678	0.362	1.03E-90	cDC1	Dnajc7
Txnl4a	4.53E-95	0.852252	0.283	0.096	1.11E-90	cDC1	Txnl4a
Atrnl11	6.57E-95	1.355689	0.27	0.091	1.61E-90	cDC1	Atrnl1
Plekhm32	9.03E-95	0.770902	0.492	0.217	2.21E-90	cDC1	Plekhm3
Bin31	1.28E-94	0.754681	0.367	0.141	3.14E-90	cDC1	Bin3
Fam98c	1.49E-94	0.830899	0.311	0.111	3.66E-90	cDC1	Fam98c
Fmc1	1.61E-94	1.027553	0.261	0.087	3.95E-90	cDC1	Fmc1
Mrpl123	1.98E-94	0.69006	0.464	0.2	4.86E-90	cDC1	Mrpl12
Rex1bd3	1.13E-93	0.661594	0.515	0.234	2.76E-89	cDC1	Rex1bd
Sp1402	1.22E-93	0.882196	0.421	0.178	2.98E-89	cDC1	Sp140
Pold45	1.63E-93	0.748873	0.686	0.366	3.98E-89	cDC1	Pold4
Gm118084	3.56E-93	0.784183	0.598	0.301	8.71E-89	cDC1	Gm11808
Apex11	4.02E-93	0.8282	0.377	0.151	9.85E-89	cDC1	Apex1
Higd1a2	4.33E-93	0.669188	0.461	0.199	1.06E-88	cDC1	Higd1a
Pabpc14	4.88E-93	0.584956	0.893	0.618	1.20E-88	cDC1	Pabpc1
Rassf42	7.61E-93	0.762226	0.693	0.381	1.86E-88	cDC1	Rassf4
Csf2rb4	8.12E-93	0.841013	0.534	0.253	1.99E-88	cDC1	Csf2rb
Mrpl422	8.93E-93	0.656199	0.503	0.23	2.19E-88	cDC1	Mrpl42
Cox143	9.92E-93	0.678556	0.554	0.261	2.43E-88	cDC1	Cox14
Pfdn55	1.16E-92	0.591077	0.907	0.687	2.85E-88	cDC1	Pfdn5
Mea13	1.97E-92	0.734816	0.453	0.195	4.82E-88	cDC1	Mea1
Emd2	2.42E-92	0.741522	0.403	0.165	5.92E-88	cDC1	Emd
Snrpc3	4.46E-92	0.756071	0.507	0.231	1.09E-87	cDC1	Snrpc
Dr11	4.85E-92	0.983695	0.283	0.098	1.19E-87	cDC1	Dr1
Osgep2	6.66E-92	0.743523	0.479	0.213	1.63E-87	cDC1	Osgep
Cnih41	7.13E-92	0.653975	0.615	0.308	1.75E-87	cDC1	Cnih4
Exosc3	7.62E-92	0.859491	0.311	0.113	1.87E-87	cDC1	Exosc3
Hmgb22	1.25E-91	0.592425	0.506	0.231	3.07E-87	cDC1	Hmgb2
Ptprc4	2.20E-91	0.626549	0.876	0.569	5.39E-87	cDC1	Ptprc
Ddx39	3.65E-91	0.904021	0.307	0.112	8.95E-87	cDC1	Ddx39
Mvp4	4.00E-91	0.750324	0.434	0.185	9.80E-87	cDC1	Mvp
Ssrp12	4.44E-91	0.84113	0.393	0.162	1.09E-86	cDC1	Ssrp1
Marcksl12	4.86E-91	1.138127	0.374	0.155	1.19E-86	cDC1	Marcksl1

Phf5a1	5.16E-91	0.763282	0.419	0.176	1.26E-86	cDC1	Phf5a
Rheb3	6.36E-91	0.618543	0.611	0.302	1.56E-86	cDC1	Rheb
Vps36	1.22E-90	0.72563	0.338	0.128	3.00E-86	cDC1	Vps36
Ndufb104	1.41E-90	0.593368	0.728	0.395	3.46E-86	cDC1	Ndufb10
Eif3l2	1.55E-90	0.687691	0.484	0.217	3.79E-86	cDC1	Eif3l
Park73	2.23E-90	0.612775	0.731	0.404	5.47E-86	cDC1	Park7
Scp25	3.83E-90	0.607872	0.639	0.334	9.39E-86	cDC1	Scp2
Mknk21	6.17E-90	0.735757	0.351	0.136	1.51E-85	cDC1	Mknk2
Cript3	8.49E-90	0.706827	0.427	0.181	2.08E-85	cDC1	Cript
Bex3	8.93E-90	0.836221	0.288	0.102	2.19E-85	cDC1	Bex3
Uqcc25	9.12E-90	0.592477	0.581	0.281	2.23E-85	cDC1	Uqcc2
Pcbp13	1.26E-89	0.654398	0.823	0.522	3.08E-85	cDC1	Pcbp1
Hspe14	1.53E-89	0.67875	0.74	0.422	3.74E-85	cDC1	Hspe1
Ap2s15	1.54E-89	0.546957	0.61	0.294	3.78E-85	cDC1	Ap2s1
Hdgf4	4.11E-89	0.774464	0.403	0.169	1.01E-84	cDC1	Hdgf
Smim144	6.25E-89	0.595419	0.607	0.3	1.53E-84	cDC1	Smim14
Chmp4b6	8.59E-89	0.700506	0.775	0.46	2.10E-84	cDC1	Chmp4b
Mrto41	1.36E-88	0.776602	0.329	0.125	3.32E-84	cDC1	Mrto4
Copz13	2.00E-88	0.749415	0.483	0.221	4.90E-84	cDC1	Copz1
Ndufb75	4.15E-88	0.547896	0.697	0.368	1.02E-83	cDC1	Ndufb7
Myc1	4.67E-88	0.838446	0.303	0.111	1.14E-83	cDC1	Myc
Cox173	5.03E-88	0.655893	0.655	0.343	1.23E-83	cDC1	Cox17
Dctpp11	5.35E-88	0.892156	0.392	0.166	1.31E-83	cDC1	Dctpp1
Pin11	6.24E-88	0.760551	0.369	0.148	1.53E-83	cDC1	Pin1
Snhg13	1.18E-87	0.826718	0.368	0.15	2.89E-83	cDC1	Snhg1
Fbxo62	1.72E-87	0.812667	0.358	0.143	4.22E-83	cDC1	Fbxo6
Nfkb21	1.93E-87	0.94761	0.289	0.105	4.73E-83	cDC1	Nfkb2
Lsm6	1.94E-87	0.596003	0.547	0.261	4.75E-83	cDC1	Lsm6
Bccip1	2.11E-87	0.701906	0.368	0.148	5.17E-83	cDC1	Bccip
Atp5j24	2.92E-87	0.625425	0.826	0.515	7.14E-83	cDC1	Atp5j2
BC028528	3.17E-87	0.953873	0.508	0.252	7.75E-83	cDC1	BC028528
Rnf1873	3.42E-87	0.5908	0.574	0.277	8.37E-83	cDC1	Rnf187
Prr133	7.86E-87	0.517908	0.638	0.329	1.92E-82	cDC1	Prr13
Cox6a14	1.32E-86	0.584845	0.826	0.508	3.22E-82	cDC1	Cox6a1
Dpy19l1	1.71E-86	0.736562	0.399	0.166	4.20E-82	cDC1	Dpy19l1
Swi54	5.85E-86	0.640286	0.69	0.375	1.43E-81	cDC1	Swi5
Trpv2	9.37E-86	0.724077	0.407	0.173	2.29E-81	cDC1	Trpv2
Rbx13	1.75E-85	0.631046	0.773	0.459	4.29E-81	cDC1	Rbx1
Fundc1	1.80E-85	0.677248	0.365	0.147	4.41E-81	cDC1	Fundc1
Tnfaip83	2.06E-85	0.550152	0.627	0.32	5.04E-81	cDC1	Tnfaip8
Psmb62	2.84E-85	0.618999	0.693	0.373	6.96E-81	cDC1	Psmb6
Add32	3.07E-85	0.515951	0.357	0.14	7.53E-81	cDC1	Add3
Pgap22	4.02E-85	0.836089	0.381	0.16	9.84E-81	cDC1	Pgap2
Psmg42	4.57E-85	0.775399	0.41	0.177	1.12E-80	cDC1	Psmg4

Tmem1672	4.76E-85	0.610803	0.555	0.267	1.17E-80	cDC1	Tmem167
Rps217	8.04E-85	0.596497	0.953	0.832	1.97E-80	cDC1	Rps21
Metrnl2	8.42E-85	0.685549	0.25	0.084	2.06E-80	cDC1	Metrnl
Atp6v0e3	1.90E-84	0.58951	0.838	0.558	4.65E-80	cDC1	Atp6v0e
Psma34	2.80E-84	0.626451	0.732	0.416	6.86E-80	cDC1	Psma3
Pno1	4.37E-84	0.958052	0.258	0.09	1.07E-79	cDC1	Pno1
Dph31	5.50E-84	0.693532	0.348	0.138	1.35E-79	cDC1	Dph3
Snrpa1	5.78E-84	0.837008	0.293	0.108	1.42E-79	cDC1	Snrpa1
Lamtor24	5.90E-84	0.576594	0.653	0.338	1.45E-79	cDC1	Lamtor2
Def6	6.15E-84	0.871983	0.311	0.119	1.51E-79	cDC1	Def6
Shisa53	6.29E-84	0.74679	0.753	0.454	1.54E-79	cDC1	Shisa5
Arhgap303	7.64E-84	0.580652	0.631	0.323	1.87E-79	cDC1	Arhgap30
Slc9a3r13	7.94E-84	0.721413	0.403	0.173	1.95E-79	cDC1	Slc9a3r1
Kxd13	8.17E-84	0.698576	0.508	0.24	2.00E-79	cDC1	Kxd1
Ndufb55	8.34E-84	0.548374	0.693	0.368	2.04E-79	cDC1	Ndufb5
Ndufa33	9.22E-84	0.664936	0.681	0.375	2.26E-79	cDC1	Ndufa3
Ndufa24	9.28E-84	0.580831	0.751	0.431	2.27E-79	cDC1	Ndufa2
Ogfr3	1.19E-83	0.807353	0.437	0.195	2.91E-79	cDC1	Ogfr
Ubb5	1.70E-83	0.439067	0.966	0.864	4.16E-79	cDC1	Ubb
Sipa1l3	1.74E-83	0.737125	0.301	0.111	4.26E-79	cDC1	Sipa1l3
Rplp15	1.79E-83	0.450983	0.979	0.919	4.39E-79	cDC1	Rplp1
Cox6c5	1.94E-83	0.562665	0.85	0.574	4.74E-79	cDC1	Cox6c
Stat22	1.99E-83	0.736781	0.427	0.187	4.87E-79	cDC1	Stat2
Gtf2h53	2.76E-83	0.629118	0.445	0.199	6.77E-79	cDC1	Gtf2h5
Polr2f3	4.40E-83	0.545653	0.436	0.192	1.08E-78	cDC1	Polr2f
Uchl3	5.20E-83	0.7756	0.311	0.118	1.27E-78	cDC1	Uchl3
Mrps28	5.63E-83	0.596526	0.399	0.169	1.38E-78	cDC1	Mrps28
Mtmr14	1.15E-82	0.917998	0.289	0.107	2.81E-78	cDC1	Mtmr14
Gng104	1.41E-82	0.59611	0.883	0.627	3.46E-78	cDC1	Gng10
Card194	1.45E-82	0.665978	0.512	0.244	3.56E-78	cDC1	Card19
Eif3c2	2.05E-82	0.626323	0.638	0.335	5.02E-78	cDC1	Eif3c
Nmi2	2.76E-82	0.838127	0.375	0.157	6.77E-78	cDC1	Nmi
Tgfbi4	5.33E-82	0.645138	0.438	0.199	1.31E-77	cDC1	Tgfbi
Nr2c2ap	6.17E-82	0.925756	0.285	0.106	1.51E-77	cDC1	Nr2c2ap
Utrn1	8.21E-82	0.740806	0.322	0.124	2.01E-77	cDC1	Utrn
Rbm423	1.65E-81	0.604419	0.525	0.25	4.04E-77	cDC1	Rbm42
Xaf12	2.12E-81	0.886201	0.427	0.192	5.19E-77	cDC1	Xaf1
Mrpl44	3.06E-81	0.602372	0.461	0.209	7.50E-77	cDC1	Mrpl4
Ap1m1	4.43E-81	0.730463	0.343	0.138	1.09E-76	cDC1	Ap1m1
Med11	4.79E-81	0.906073	0.292	0.11	1.17E-76	cDC1	Med11
Rab1b3	5.81E-81	0.563025	0.535	0.258	1.42E-76	cDC1	Rab1b
Bola31	5.91E-81	0.66382	0.334	0.134	1.45E-76	cDC1	Bola3
Vdac32	6.17E-81	0.564787	0.511	0.241	1.51E-76	cDC1	Vdac3
Ngdn1	7.42E-81	0.73994	0.349	0.141	1.82E-76	cDC1	Ngdn

Psma64	1.24E-80	0.628685	0.539	0.266	3.04E-76	cDC1	Psma6
Sfr12	1.63E-80	0.554086	0.531	0.255	4.00E-76	cDC1	Sfr1
Cmpk13	2.09E-80	0.604139	0.547	0.267	5.11E-76	cDC1	Cmpk1
Aimp12	2.48E-80	0.671076	0.488	0.23	6.07E-76	cDC1	Aimp1
Gadd45gip	7.07E-80	0.663855	0.4	0.173	1.73E-75	cDC1	Gadd45gip1
Trabd	8.94E-80	0.949003	0.261	0.094	2.19E-75	cDC1	Trabd
Cd723	1.40E-79	1.317159	0.321	0.135	3.44E-75	cDC1	Cd72
Cdc342	1.68E-79	0.728486	0.342	0.139	4.11E-75	cDC1	Cdc34
Glrx31	2.74E-79	0.580674	0.546	0.268	6.70E-75	cDC1	Glrx3
Raly3	3.57E-79	0.633469	0.569	0.289	8.74E-75	cDC1	Raly
Prkd3	4.90E-79	0.632974	0.374	0.157	1.20E-74	cDC1	Prkd3
Sod1	7.57E-79	0.87653	0.304	0.119	1.85E-74	cDC1	Sod1
Edf14	8.14E-79	0.593192	0.776	0.457	1.99E-74	cDC1	Edf1
Ndufs81	8.34E-79	0.533214	0.595	0.3	2.04E-74	cDC1	Ndufs8
Cyb5r32	8.94E-79	0.811722	0.341	0.14	2.19E-74	cDC1	Cyb5r3
Atic	1.10E-78	0.906959	0.277	0.104	2.70E-74	cDC1	Atic
Oasl23	1.64E-78	1.004764	0.403	0.189	4.01E-74	cDC1	Oasl2
Ubxn14	3.71E-78	0.589539	0.691	0.382	9.08E-74	cDC1	Ubxn1
Cyb5a2	5.25E-78	0.523878	0.691	0.393	1.29E-73	cDC1	Cyb5a
Uqcr105	6.48E-78	0.506692	0.696	0.378	1.59E-73	cDC1	Uqcr10
Ntan12	7.00E-78	0.617994	0.407	0.18	1.71E-73	cDC1	Ntan1
Psip1	7.75E-78	0.6802	0.275	0.101	1.90E-73	cDC1	Psip1
Gtf2e2	7.88E-78	0.633132	0.264	0.095	1.93E-73	cDC1	Gtf2e2
Zfp516	1.11E-77	0.82443	0.311	0.121	2.72E-73	cDC1	Zfp516
Gyg3	1.71E-77	0.673425	0.357	0.15	4.18E-73	cDC1	Gyg
Mrpl24	4.87E-77	0.573719	0.457	0.211	1.19E-72	cDC1	Mrpl24
Cox7a26	4.92E-77	0.558294	0.77	0.456	1.20E-72	cDC1	Cox7a2
Tmem2583	5.04E-77	0.615006	0.701	0.403	1.23E-72	cDC1	Tmem258
Timm134	6.99E-77	0.515406	0.635	0.332	1.71E-72	cDC1	Timm13
Snrnp40	7.73E-77	0.685848	0.332	0.134	1.89E-72	cDC1	Snrnp40
Cox8a5	1.08E-76	0.458698	0.952	0.781	2.64E-72	cDC1	Cox8a
Oat	1.39E-76	0.669371	0.309	0.121	3.41E-72	cDC1	Oat
Arpp191	1.87E-76	0.613344	0.594	0.311	4.57E-72	cDC1	Arpp19
Prelid3b2	3.49E-76	0.741379	0.35	0.147	8.56E-72	cDC1	Prelid3b
Lmo43	3.55E-76	0.723669	0.353	0.148	8.71E-72	cDC1	Lmo4
Cnbp1	3.84E-76	0.547425	0.806	0.516	9.41E-72	cDC1	Cnbp
Spop2	4.19E-76	0.483596	0.662	0.355	1.03E-71	cDC1	Spop
Sap182	6.26E-76	0.59047	0.559	0.286	1.53E-71	cDC1	Sap18
Phb22	6.46E-76	0.581981	0.581	0.299	1.58E-71	cDC1	Phb2
Cox6b15	6.94E-76	0.559608	0.841	0.553	1.70E-71	cDC1	Cox6b1
Chchd24	7.10E-76	0.619578	0.66	0.369	1.74E-71	cDC1	Chchd2
Ptms3	8.11E-76	0.671052	0.786	0.518	1.99E-71	cDC1	Ptms
Cdk142	9.67E-76	0.917336	0.489	0.236	2.37E-71	cDC1	Cdk14
Arl3	1.03E-75	0.455745	0.336	0.136	2.51E-71	cDC1	Arl3

Ric8a	1.04E-75	0.78155	0.302	0.119	2.56E-71	cDC1	Ric8a
Nfu1	1.33E-75	0.590441	0.466	0.218	3.25E-71	cDC1	Nfu1
Trmt1122	1.99E-75	0.569806	0.785	0.481	4.86E-71	cDC1	Trmt112
Rab323	2.08E-75	0.79994	0.411	0.191	5.10E-71	cDC1	Rab32
Tomm70a2	2.19E-75	0.607945	0.387	0.169	5.36E-71	cDC1	Tomm70a
Fkbp3	2.50E-75	0.569826	0.363	0.153	6.12E-71	cDC1	Fkbp3
Ptpn122	2.94E-75	0.777931	0.264	0.097	7.19E-71	cDC1	Ptpn12
Mta2	3.02E-75	0.749979	0.352	0.149	7.39E-71	cDC1	Mta2
Cox7c3	3.29E-75	0.598726	0.837	0.57	8.07E-71	cDC1	Cox7c
Nsun2	3.84E-75	0.745464	0.325	0.132	9.41E-71	cDC1	Nsun2
Meaf6	4.08E-75	0.521126	0.369	0.156	1.00E-70	cDC1	Meaf6
D8Ertd738e	4.11E-75	0.550893	0.755	0.438	1.01E-70	cDC1	D8Ertd738e
Nsmce1	7.21E-75	0.672619	0.326	0.133	1.77E-70	cDC1	Nsmce1
Sod24	8.70E-75	0.431024	0.419	0.186	2.13E-70	cDC1	Sod2
Bax3	8.77E-75	0.612775	0.661	0.359	2.15E-70	cDC1	Bax
Bloc1s15	1.56E-74	0.650698	0.465	0.222	3.82E-70	cDC1	Bloc1s1
Cwc153	1.81E-74	0.499967	0.597	0.306	4.43E-70	cDC1	Cwc15
Tsc22d35	2.57E-74	0.519919	0.575	0.295	6.29E-70	cDC1	Tsc22d3
Stip1	2.94E-74	0.702349	0.304	0.12	7.19E-70	cDC1	Stip1
Mapkapk31	4.20E-74	0.852541	0.293	0.116	1.03E-69	cDC1	Mapkapk3
Mrpl143	7.41E-74	0.540733	0.461	0.217	1.82E-69	cDC1	Mrpl14
Eno15	8.69E-74	0.543686	0.569	0.295	2.13E-69	cDC1	Eno1
Pfdn61	9.72E-74	0.765126	0.308	0.124	2.38E-69	cDC1	Pfdn6
Spag92	1.19E-73	0.551333	0.582	0.299	2.92E-69	cDC1	Spag9
Ppp4c3	1.24E-73	0.553497	0.634	0.337	3.03E-69	cDC1	Ppp4c
Dazap24	1.38E-73	0.541081	0.758	0.449	3.37E-69	cDC1	Dazap2
Med30	1.42E-73	0.598679	0.374	0.162	3.47E-69	cDC1	Med30
Ufm12	1.69E-73	0.716853	0.338	0.142	4.13E-69	cDC1	Ufm1
Mrpl363	1.86E-73	0.567309	0.426	0.194	4.57E-69	cDC1	Mrpl36
Dbi4	2.06E-73	0.369312	0.739	0.43	5.03E-69	cDC1	Dbi
Krtcap22	2.22E-73	0.624206	0.736	0.437	5.44E-69	cDC1	Krtcap2
Fam104a3	2.22E-73	0.473681	0.485	0.229	5.45E-69	cDC1	Fam104a
Pdcd2l	2.27E-73	0.562774	0.36	0.152	5.56E-69	cDC1	Pdcd2l
Srp192	2.81E-73	0.560122	0.45	0.21	6.87E-69	cDC1	Srp19
Tbcb2	3.26E-73	0.529478	0.527	0.259	7.98E-69	cDC1	Tbcb
Otub11	3.64E-73	0.611054	0.451	0.213	8.92E-69	cDC1	Otub1
Psmd132	4.29E-73	0.541025	0.456	0.213	1.05E-68	cDC1	Psmd13
Uqcrc2	4.64E-73	0.512169	0.535	0.266	1.14E-68	cDC1	Uqcrc2
Zfp8001	5.29E-73	0.624291	0.318	0.128	1.29E-68	cDC1	Zfp800
Ndufc15	5.55E-73	0.543241	0.551	0.279	1.36E-68	cDC1	Ndufc1
Bcl2a1a3	5.60E-73	1.106838	0.315	0.133	1.37E-68	cDC1	Bcl2a1a
Mrps15	6.08E-73	0.539167	0.518	0.258	1.49E-68	cDC1	Mrps15
Hnrnpa02	7.52E-73	0.605133	0.551	0.28	1.84E-68	cDC1	Hnrnpa0
Elmo21	9.18E-73	0.732948	0.299	0.119	2.25E-68	cDC1	Elmo2

Rfx7	1.28E-72	0.568865	0.287	0.11	3.13E-68	cDC1	Rfx7
Sars1	1.48E-72	0.62481	0.444	0.209	3.62E-68	cDC1	Sars
Nfkbib3	1.81E-72	0.653512	0.396	0.178	4.42E-68	cDC1	Nfkbib
Sys12	2.04E-72	0.535841	0.576	0.298	4.99E-68	cDC1	Sys1
Gtf2f1	2.05E-72	0.748944	0.302	0.12	5.02E-68	cDC1	Gtf2f1
Micos133	3.84E-72	0.534496	0.594	0.313	9.39E-68	cDC1	Micos13
Ube2q12	4.86E-72	0.531973	0.439	0.205	1.19E-67	cDC1	Ube2q1
Gars2	5.08E-72	0.673556	0.446	0.21	1.24E-67	cDC1	Gars
Zbtb8os1	8.67E-72	0.631097	0.299	0.119	2.12E-67	cDC1	Zbtb8os
Pid14	9.79E-72	0.958479	0.683	0.41	2.40E-67	cDC1	Pid1
Ndufa124	1.26E-71	0.480076	0.519	0.257	3.10E-67	cDC1	Ndufa12
Ndufa75	1.29E-71	0.525452	0.797	0.491	3.17E-67	cDC1	Ndufa7
Fkbp1a4	1.58E-71	0.520752	0.618	0.331	3.87E-67	cDC1	Fkbp1a
Gtf3c6	1.72E-71	0.696425	0.273	0.105	4.21E-67	cDC1	Gtf3c6
Babam11	1.76E-71	0.63405	0.391	0.173	4.32E-67	cDC1	Babam1
Pgk15	1.93E-71	0.480568	0.515	0.254	4.72E-67	cDC1	Pgk1
Ndufaf82	1.94E-71	0.669627	0.391	0.177	4.74E-67	cDC1	Ndufaf8
Mob1a2	1.96E-71	0.570404	0.473	0.226	4.79E-67	cDC1	Mob1a
Cdc5l	2.04E-71	0.633791	0.353	0.151	4.98E-67	cDC1	Cdc5l
Hpcal15	2.34E-71	0.542018	0.52	0.257	5.73E-67	cDC1	Hpcal1
Eif3a4	2.40E-71	0.528652	0.634	0.337	5.88E-67	cDC1	Eif3a
Tiam11	2.68E-71	0.505343	0.348	0.145	6.57E-67	cDC1	Tiam1
Gosr2	3.21E-71	0.676224	0.326	0.136	7.86E-67	cDC1	Gosr2
Sin3b3	3.39E-71	0.570728	0.533	0.268	8.31E-67	cDC1	Sin3b
Psmc42	3.41E-71	0.562411	0.43	0.199	8.34E-67	cDC1	Psmc4
Borcs6	3.44E-71	0.718584	0.28	0.109	8.42E-67	cDC1	Borcs6
Eif2s12	3.45E-71	0.54479	0.434	0.203	8.46E-67	cDC1	Eif2s1
Sgk31	3.61E-71	0.477238	0.462	0.213	8.84E-67	cDC1	Sgk3
C1d1	4.79E-71	0.521441	0.431	0.199	1.17E-66	cDC1	C1d
Mrpl283	6.31E-71	0.639584	0.342	0.147	1.54E-66	cDC1	Mrpl28
Rpl22l15	7.63E-71	0.589215	0.79	0.493	1.87E-66	cDC1	Rpl22l1
Ubl55	8.37E-71	0.538439	0.769	0.478	2.05E-66	cDC1	Ubl5
Mrpl182	1.85E-70	0.554465	0.416	0.192	4.52E-66	cDC1	Mrpl18
Tgtp23	2.44E-70	1.056065	0.269	0.106	5.96E-66	cDC1	Tgtp2
Sep-72	2.51E-70	0.404092	0.604	0.314	6.15E-66	cDC1	7-Sep
Smarcd21	2.54E-70	0.586944	0.332	0.138	6.23E-66	cDC1	Smarcd2
Trim254	3.14E-70	0.639085	0.375	0.167	7.70E-66	cDC1	Trim25
Amz11	3.37E-70	0.57685	0.397	0.178	8.26E-66	cDC1	Amz1
Dusp22	5.38E-70	0.654571	0.298	0.119	1.32E-65	cDC1	Dusp22
Stoml22	5.57E-70	0.635982	0.359	0.157	1.36E-65	cDC1	Stoml2
Mrpl581	6.19E-70	0.701088	0.349	0.152	1.52E-65	cDC1	Mrpl58
Ubl4a	7.59E-70	0.774161	0.265	0.103	1.86E-65	cDC1	Ubl4a
Pum3	1.30E-69	0.685089	0.254	0.095	3.19E-65	cDC1	Pum3
Smim32	2.06E-69	0.545735	0.342	0.145	5.04E-65	cDC1	Smim3

Sumo13	2.49E-69	0.506093	0.604	0.321	6.09E-65	cDC1	Sumo1
Tubb53	2.51E-69	0.633936	0.749	0.465	6.15E-65	cDC1	Tubb5
Eif3b2	3.44E-69	0.617018	0.375	0.167	8.42E-65	cDC1	Eif3b
Rbck1	3.82E-69	0.512142	0.443	0.208	9.35E-65	cDC1	Rbck1
Fmnl12	4.15E-69	0.515074	0.624	0.338	1.02E-64	cDC1	Fmnl1
Mospd3	4.61E-69	0.802088	0.262	0.101	1.13E-64	cDC1	Mospd3
Gnai23	8.87E-69	0.430027	0.929	0.781	2.17E-64	cDC1	Gnai2
Dcaf12	1.22E-68	0.41672	0.411	0.186	2.98E-64	cDC1	Dcaf12
Mpc23	1.33E-68	0.492957	0.612	0.334	3.27E-64	cDC1	Mpc2
Grk61	1.81E-68	0.745042	0.25	0.093	4.43E-64	cDC1	Grk6
Mrps34	3.81E-68	0.582249	0.391	0.177	9.33E-64	cDC1	Mrps34
Cct31	4.26E-68	0.633312	0.414	0.195	1.04E-63	cDC1	Cct3
Cenpx4	4.63E-68	0.425859	0.544	0.276	1.14E-63	cDC1	Cenpx
Eef1d4	5.00E-68	0.494018	0.787	0.489	1.23E-63	cDC1	Eef1d
Capg4	5.54E-68	1.274014	0.312	0.14	1.36E-63	cDC1	Capg
Cers5	8.30E-68	0.533346	0.362	0.158	2.03E-63	cDC1	Cers5
Cops63	1.06E-67	0.553475	0.47	0.231	2.59E-63	cDC1	Cops6
Lsm81	1.22E-67	0.55163	0.365	0.16	3.00E-63	cDC1	Lsm8
Lsm2	1.26E-67	0.723469	0.284	0.114	3.09E-63	cDC1	Lsm2
Tpr3	1.42E-67	0.488868	0.639	0.35	3.47E-63	cDC1	Tpr
Mettl23	1.45E-67	0.604071	0.425	0.2	3.56E-63	cDC1	Mettl23
Etfa3	1.87E-67	0.446986	0.45	0.214	4.58E-63	cDC1	Etfa
Stk242	1.95E-67	0.38916	0.45	0.212	4.78E-63	cDC1	Stk24
Polr2m	2.36E-67	0.569381	0.326	0.139	5.78E-63	cDC1	Polr2m
Mrps23	2.71E-67	0.750815	0.292	0.119	6.65E-63	cDC1	Mrps23
Plekho24	3.62E-67	0.577559	0.426	0.203	8.87E-63	cDC1	Plekho2
Pdap11	4.20E-67	0.57791	0.349	0.154	1.03E-62	cDC1	Pdap1
Vars	4.35E-67	0.621519	0.347	0.151	1.06E-62	cDC1	Vars
Sumo32	4.37E-67	0.594776	0.363	0.162	1.07E-62	cDC1	Sumo3
Sptbn11	6.27E-67	0.523511	0.314	0.131	1.54E-62	cDC1	Sptbn1
Leprotl13	9.10E-67	0.528676	0.532	0.272	2.23E-62	cDC1	Leprotl1
Cdc423	9.84E-67	0.459741	0.914	0.728	2.41E-62	cDC1	Cdc42
Snf82	1.06E-66	0.509086	0.459	0.223	2.59E-62	cDC1	Snf8
Hes6	1.28E-66	0.752058	0.265	0.104	3.14E-62	cDC1	Hes6
Pdlim51	1.36E-66	0.469083	0.425	0.198	3.33E-62	cDC1	Pdlim5
Etfb3	1.43E-66	0.418589	0.539	0.273	3.49E-62	cDC1	Etfb
Mrpl512	1.45E-66	0.519976	0.394	0.18	3.55E-62	cDC1	Mrpl51
Hsd17b102	1.48E-66	0.676367	0.338	0.148	3.63E-62	cDC1	Hsd17b10
Gsk3a	1.73E-66	0.508912	0.326	0.138	4.25E-62	cDC1	Gsk3a
Capns14	1.98E-66	0.484384	0.635	0.346	4.86E-62	cDC1	Capns1
Mrps18c1	2.09E-66	0.563457	0.4	0.185	5.12E-62	cDC1	Mrps18c
Taok32	2.18E-66	0.471622	0.657	0.364	5.33E-62	cDC1	Taok3
Psmd42	2.75E-66	0.518398	0.504	0.255	6.74E-62	cDC1	Psmd4
Cyp27a12	3.60E-66	0.645018	0.32	0.136	8.82E-62	cDC1	Cyp27a1

Mrpl431	4.77E-66	0.542578	0.512	0.261	1.17E-61	cDC1	Mrpl43
Polr2k1	6.74E-66	0.556168	0.376	0.17	1.65E-61	cDC1	Polr2k
Exosc8	6.99E-66	0.580022	0.325	0.139	1.71E-61	cDC1	Exosc8
Csk3	9.40E-66	0.510928	0.635	0.353	2.30E-61	cDC1	Csk
Ndufa135	9.61E-66	0.489933	0.817	0.532	2.35E-61	cDC1	Ndufa13
Gclm2	1.05E-65	0.250856	0.421	0.196	2.58E-61	cDC1	Gclm
Ndufb25	1.07E-65	0.544994	0.482	0.243	2.61E-61	cDC1	Ndufb2
Fcf11	1.41E-65	0.591126	0.442	0.213	3.46E-61	cDC1	Fcf1
Sh3bp12	1.64E-65	0.662679	0.554	0.308	4.02E-61	cDC1	Sh3bp1
Eif4h2	2.33E-65	0.379588	0.571	0.294	5.71E-61	cDC1	Eif4h
Spg214	2.62E-65	0.571909	0.478	0.238	6.41E-61	cDC1	Spg21
Imp3	2.71E-65	0.596486	0.427	0.204	6.63E-61	cDC1	Imp3
Mdfic2	3.18E-65	0.65521	0.267	0.106	7.79E-61	cDC1	Mdfic
Smarcb1	3.39E-65	0.581047	0.342	0.15	8.30E-61	cDC1	Smarcb1
Irgm13	3.51E-65	0.6647	0.479	0.243	8.59E-61	cDC1	Irgm1
Pfdn24	5.21E-65	0.426073	0.535	0.272	1.28E-60	cDC1	Pfdn2
Ostf13	5.32E-65	0.464914	0.79	0.49	1.30E-60	cDC1	Ostf1
Ank	7.51E-65	0.66188	0.29	0.121	1.84E-60	cDC1	Ank
Trappc6a2	8.20E-65	0.653021	0.328	0.143	2.01E-60	cDC1	Trappc6a
Rab212	8.22E-65	0.449466	0.472	0.232	2.01E-60	cDC1	Rab21
Syf23	1.06E-64	0.486196	0.477	0.236	2.59E-60	cDC1	Syf2
Themis24	1.12E-64	0.627716	0.443	0.217	2.73E-60	cDC1	Themis2
Mak16	1.15E-64	0.567702	0.272	0.108	2.82E-60	cDC1	Mak16
Anxa43	1.19E-64	0.730215	0.34	0.15	2.91E-60	cDC1	Anxa4
Strap	1.82E-64	0.594648	0.35	0.156	4.47E-60	cDC1	Strap
Nsmce4a1	1.85E-64	0.530494	0.361	0.161	4.52E-60	cDC1	Nsmce4a
Fam162a1	1.91E-64	0.590372	0.329	0.143	4.69E-60	cDC1	Fam162a
Ube2f1	2.01E-64	0.471154	0.524	0.27	4.92E-60	cDC1	Ube2f
Rac24	2.40E-64	0.590321	0.699	0.453	5.89E-60	cDC1	Rac2
Xrn22	3.32E-64	0.448601	0.504	0.254	8.13E-60	cDC1	Xrn2
Psmb71	3.92E-64	0.525205	0.382	0.178	9.59E-60	cDC1	Psmb7
Twistnb	4.13E-64	0.549405	0.34	0.149	1.01E-59	cDC1	Twistnb
Mrpl35	4.36E-64	0.673923	0.264	0.105	1.07E-59	cDC1	Mrpl35
Ube2n1	7.28E-64	0.479508	0.527	0.275	1.78E-59	cDC1	Ube2n
Clns1a	9.04E-64	0.536581	0.338	0.149	2.21E-59	cDC1	Clns1a
Timm17b	9.73E-64	0.502202	0.353	0.157	2.38E-59	cDC1	Timm17b
Tbc1d10b	1.51E-63	0.717183	0.257	0.101	3.69E-59	cDC1	Tbc1d10b
Mrpl22	1.71E-63	0.552334	0.291	0.12	4.19E-59	cDC1	Mrpl22
Anxa111	2.18E-63	0.446874	0.276	0.112	5.33E-59	cDC1	Anxa11
Gm20002	3.73E-63	0.73805	0.3	0.128	9.13E-59	cDC1	Gm2000
Lage3	4.00E-63	0.612297	0.324	0.141	9.80E-59	cDC1	Lage3
Ndufa17	4.83E-63	0.461327	0.6	0.327	1.18E-58	cDC1	Ndufa1
Foxn2	4.95E-63	0.619314	0.268	0.107	1.21E-58	cDC1	Foxn2
Ssna13	7.17E-63	0.535219	0.404	0.192	1.75E-58	cDC1	Ssna1

Isoc11	7.17E-63	0.618504	0.297	0.126	1.76E-58	cDC1	Isoc1
Nans	7.69E-63	0.665347	0.28	0.116	1.88E-58	cDC1	Nans
Slc48a13	9.09E-63	0.380825	0.349	0.156	2.23E-58	cDC1	Slc48a1
Ube2d33	1.19E-62	0.528011	0.807	0.523	2.91E-58	cDC1	Ube2d3
Ikbkb2	1.31E-62	0.491875	0.47	0.234	3.21E-58	cDC1	Ikbkb
Polr2c1	1.46E-62	0.526802	0.342	0.151	3.59E-58	cDC1	Polr2c
Atpif11	1.72E-62	0.632058	0.782	0.557	4.21E-58	cDC1	Atpif1
1500011B0	1.73E-62	0.62959	0.253	0.1	4.24E-58	cDC1	1500011B03Rik
Tyk2	1.95E-62	0.517144	0.32	0.138	4.77E-58	cDC1	Tyk2
Oas1a3	2.16E-62	0.952856	0.334	0.156	5.29E-58	cDC1	Oas1a
Gabarapl22	2.21E-62	0.443512	0.654	0.373	5.42E-58	cDC1	Gabarapl2
Psma11	4.34E-62	0.446288	0.555	0.292	1.06E-57	cDC1	Psma1
Top2b	5.39E-62	0.352642	0.392	0.181	1.32E-57	cDC1	Top2b
Pml2	6.04E-62	0.955606	0.273	0.114	1.48E-57	cDC1	Pml
Cyb5b	6.17E-62	0.539879	0.268	0.108	1.51E-57	cDC1	Cyb5b
Psmd14	8.46E-62	0.398988	0.426	0.203	2.07E-57	cDC1	Psmd14
Fam107b2	8.78E-62	0.474203	0.337	0.148	2.15E-57	cDC1	Fam107b
Cmc12	8.89E-62	0.453952	0.439	0.214	2.18E-57	cDC1	Cmc1
Nadk4	9.57E-62	0.316672	0.494	0.245	2.34E-57	cDC1	Nadk
Mrps363	1.26E-61	0.45236	0.387	0.18	3.08E-57	cDC1	Mrps36
Znhit14	1.88E-61	0.529577	0.4	0.191	4.61E-57	cDC1	Znhit1
Ndufb95	2.04E-61	0.439964	0.759	0.445	4.98E-57	cDC1	Ndufb9
Ndufb34	2.81E-61	0.406464	0.566	0.301	6.88E-57	cDC1	Ndufb3
Sra13	3.75E-61	0.616889	0.405	0.196	9.18E-57	cDC1	Sra1
Gemin71	4.37E-61	0.590067	0.387	0.184	1.07E-56	cDC1	Gemin7
Tmem2192	4.58E-61	0.503517	0.59	0.326	1.12E-56	cDC1	Tmem219
Psmd23	8.40E-61	0.599482	0.404	0.195	2.06E-56	cDC1	Psmd2
Pcbp22	9.58E-61	0.417398	0.622	0.342	2.35E-56	cDC1	Pcbp2
Sp1102	9.92E-61	0.670003	0.468	0.242	2.43E-56	cDC1	Sp110
Nucks11	1.11E-60	0.536597	0.442	0.221	2.72E-56	cDC1	Nucks1
Suclg1	1.25E-60	0.402528	0.361	0.164	3.06E-56	cDC1	Suclg1
Polr3c	1.34E-60	0.614933	0.282	0.118	3.27E-56	cDC1	Polr3c
Dennd1b1	1.98E-60	0.477802	0.465	0.229	4.85E-56	cDC1	Dennd1b
Coro1b3	2.13E-60	0.51508	0.751	0.466	5.21E-56	cDC1	Coro1b
Zfp1062	2.29E-60	0.394877	0.439	0.214	5.62E-56	cDC1	Zfp106
Ndufs64	2.86E-60	0.466652	0.424	0.208	7.01E-56	cDC1	Ndufs6
Chrac12	3.15E-60	0.491964	0.468	0.236	7.72E-56	cDC1	Chrac1
Cers63	3.88E-60	0.60944	0.416	0.204	9.50E-56	cDC1	Cers6
Erh	4.18E-60	0.546718	0.252	0.1	1.02E-55	cDC1	Erh
Elp5	5.50E-60	0.543425	0.295	0.127	1.35E-55	cDC1	Elp5
Ccndbp1	6.93E-60	0.615508	0.299	0.13	1.70E-55	cDC1	Ccndbp1
Hat1	7.34E-60	0.687634	0.283	0.12	1.80E-55	cDC1	Hat1
Ahcyl21	9.43E-60	0.34252	0.385	0.178	2.31E-55	cDC1	Ahcyl2
Prpf40a1	1.08E-59	0.458518	0.558	0.294	2.64E-55	cDC1	Prpf40a

Cox4i16	1.47E-59	0.35791	0.952	0.801	3.59E-55	cDC1	Cox4i1
Ccng1	1.58E-59	0.557602	0.347	0.158	3.88E-55	cDC1	Ccng1
Rabgap1l	2.11E-59	0.404901	0.261	0.104	5.16E-55	cDC1	Rabgap1l
Cdkn1b1	2.74E-59	0.632523	0.307	0.134	6.70E-55	cDC1	Cdkn1b
Fdx22	3.11E-59	0.517115	0.321	0.143	7.61E-55	cDC1	Fdx2
Commd7	3.69E-59	0.478328	0.45	0.223	9.03E-55	cDC1	Commd7
Pcbd22	6.86E-59	0.508398	0.337	0.153	1.68E-54	cDC1	Pcbd2
Rnf1661	6.90E-59	0.418229	0.406	0.195	1.69E-54	cDC1	Rnf166
Bnip21	7.70E-59	0.436702	0.55	0.293	1.88E-54	cDC1	Bnip2
Adh51	7.87E-59	0.418716	0.441	0.218	1.93E-54	cDC1	Adh5
Timm8b2	8.02E-59	0.401152	0.452	0.225	1.96E-54	cDC1	Timm8b
Ssr32	8.66E-59	0.435819	0.446	0.221	2.12E-54	cDC1	Ssr3
Ola1	8.69E-59	0.453405	0.381	0.181	2.13E-54	cDC1	Ola1
Rchy11	1.36E-58	0.517509	0.365	0.17	3.33E-54	cDC1	Rchy1
Ier3ip12	1.68E-58	0.404456	0.531	0.277	4.12E-54	cDC1	Ier3ip1
Cdv31	2.17E-58	0.482959	0.315	0.14	5.32E-54	cDC1	Cdv3
Ttc71	2.27E-58	0.374451	0.415	0.199	5.55E-54	cDC1	Ttc7
Jarid24	3.77E-58	0.576236	0.592	0.33	9.23E-54	cDC1	Jarid2
Cpsf2	4.05E-58	0.578391	0.257	0.104	9.92E-54	cDC1	Cpsf2
Dusp112	4.36E-58	0.400498	0.461	0.23	1.07E-53	cDC1	Dusp11
Tomm65	4.70E-58	0.42487	0.823	0.528	1.15E-53	cDC1	Tomm6
Ensa1	6.06E-58	0.560692	0.321	0.143	1.48E-53	cDC1	Ensa
Ndufa114	6.85E-58	0.385753	0.623	0.347	1.68E-53	cDC1	Ndufa11
Trnau1ap	8.14E-58	0.676121	0.268	0.112	1.99E-53	cDC1	Trnau1ap
Rnf1501	8.97E-58	0.361155	0.301	0.129	2.20E-53	cDC1	Rnf150
Pgam15	1.09E-57	0.430644	0.615	0.345	2.68E-53	cDC1	Pgam1
Ubald23	1.28E-57	0.498487	0.531	0.284	3.14E-53	cDC1	Ubald2
Ptpn21	1.34E-57	0.464186	0.323	0.144	3.28E-53	cDC1	Ptpn2
Sgpp11	1.60E-57	0.711353	0.277	0.118	3.91E-53	cDC1	Sgpp1
Polr2g3	1.74E-57	0.42439	0.446	0.224	4.26E-53	cDC1	Polr2g
3830406C1	1.94E-57	0.467424	0.292	0.126	4.75E-53	cDC1	3830406C13Rik
Exosc7	2.30E-57	0.536855	0.258	0.105	5.63E-53	cDC1	Exosc7
Pop5	2.44E-57	0.581821	0.281	0.12	5.99E-53	cDC1	Pop5
Cope3	2.90E-57	0.495157	0.654	0.382	7.11E-53	cDC1	Cope
Xpo1	5.15E-57	0.623494	0.26	0.107	1.26E-52	cDC1	Xpo1
Eloc1	5.41E-57	0.377428	0.549	0.29	1.32E-52	cDC1	Eloc
Idh3a	5.41E-57	0.594921	0.261	0.108	1.33E-52	cDC1	Idh3a
Ccdc1241	5.74E-57	0.505902	0.377	0.181	1.40E-52	cDC1	Ccdc124
Far12	5.84E-57	0.450164	0.399	0.19	1.43E-52	cDC1	Far1
Rad23a	6.45E-57	0.40919	0.42	0.207	1.58E-52	cDC1	Rad23a
Dohh1	7.57E-57	0.652209	0.28	0.12	1.85E-52	cDC1	Dohh
Fam50a3	7.65E-57	0.484629	0.373	0.177	1.87E-52	cDC1	Fam50a
Ptges31	7.74E-57	0.433248	0.547	0.294	1.90E-52	cDC1	Ptges3
Srp144	9.17E-57	0.380815	0.647	0.362	2.25E-52	cDC1	Srp14

Spr1	1.03E-56	0.595063	0.284	0.123	2.53E-52	cDC1	Spr
Psmb24	1.08E-56	0.378153	0.649	0.365	2.65E-52	cDC1	Psmb2
Ppil3	1.25E-56	0.665619	0.25	0.103	3.07E-52	cDC1	Ppil3
Lsm1	1.26E-56	0.454325	0.282	0.12	3.10E-52	cDC1	Lsm1
Tmem2563	1.68E-56	0.424118	0.58	0.315	4.11E-52	cDC1	Tmem256
Uqcrc12	1.76E-56	0.383578	0.602	0.335	4.32E-52	cDC1	Uqcrc1
Sarnp1	1.98E-56	0.309227	0.531	0.281	4.86E-52	cDC1	Sarnp
Smarce1	2.04E-56	0.405771	0.342	0.155	5.00E-52	cDC1	Smarce1
Ppp2r1a2	2.09E-56	0.445009	0.457	0.233	5.11E-52	cDC1	Ppp2r1a
Fam89b3	3.10E-56	0.386052	0.51	0.265	7.59E-52	cDC1	Fam89b
Mrps121	3.11E-56	0.422655	0.338	0.154	7.61E-52	cDC1	Mrps12
Cct6a	3.78E-56	0.51971	0.392	0.192	9.26E-52	cDC1	Cct6a
Abce1	8.22E-56	0.519951	0.261	0.108	2.01E-51	cDC1	Abce1
Ddx46	8.87E-56	0.409385	0.332	0.152	2.17E-51	cDC1	Ddx46
Nono2	9.87E-56	0.465494	0.527	0.286	2.42E-51	cDC1	Nono
Sf3a21	1.30E-55	0.445678	0.344	0.159	3.17E-51	cDC1	Sf3a2
Atp5k2	1.46E-55	0.482864	0.417	0.209	3.58E-51	cDC1	Atp5k
Zc3hav14	1.55E-55	0.42476	0.463	0.235	3.79E-51	cDC1	Zc3hav1
Ndufs54	1.75E-55	0.321787	0.598	0.329	4.30E-51	cDC1	Ndufs5
Eny21	1.87E-55	0.445745	0.338	0.156	4.58E-51	cDC1	Eny2
Mrps26	2.06E-55	0.42203	0.319	0.143	5.05E-51	cDC1	Mrps26
Actr10	2.53E-55	0.430198	0.397	0.193	6.20E-51	cDC1	Actr10
Tmem2421	4.51E-55	0.517941	0.279	0.121	1.10E-50	cDC1	Tmem242
Tuba1a2	5.46E-55	0.254067	0.485	0.247	1.34E-50	cDC1	Tuba1a
Hdac1	5.73E-55	0.5184	0.371	0.179	1.40E-50	cDC1	Hdac1
Timm17a2	6.51E-55	0.479357	0.351	0.165	1.59E-50	cDC1	Timm17a
Mrtfa3	7.27E-55	0.352011	0.455	0.229	1.78E-50	cDC1	Mrtfa
Cisd1	8.43E-55	0.47298	0.313	0.141	2.06E-50	cDC1	Cisd1
Elof11	1.12E-54	0.509979	0.334	0.155	2.75E-50	cDC1	Elof1
Ndufs22	1.34E-54	0.362836	0.503	0.264	3.28E-50	cDC1	Ndufs2
Noc2l	1.44E-54	0.459268	0.374	0.18	3.52E-50	cDC1	Noc2l
Rcc21	1.46E-54	0.434968	0.331	0.152	3.59E-50	cDC1	Rcc2
Eif1a2	1.50E-54	0.586731	0.323	0.149	3.68E-50	cDC1	Eif1a
Srp72	1.66E-54	0.422275	0.393	0.192	4.06E-50	cDC1	Srp72
Eif4a3	2.42E-54	0.394949	0.458	0.235	5.92E-50	cDC1	Eif4a3
Atp5a15	2.54E-54	0.442452	0.745	0.467	6.22E-50	cDC1	Atp5a1
Micos103	3.15E-54	0.361216	0.593	0.331	7.71E-50	cDC1	Micos10
Malt11	3.55E-54	0.616767	0.307	0.139	8.69E-50	cDC1	Malt1
Timm232	4.08E-54	0.481565	0.5	0.268	9.99E-50	cDC1	Timm23
Ncl2	7.92E-54	0.416641	0.617	0.353	1.94E-49	cDC1	Ncl
Fam174a3	7.99E-54	0.460785	0.565	0.313	1.96E-49	cDC1	Fam174a
Arl6ip52	8.78E-54	0.404863	0.648	0.375	2.15E-49	cDC1	Arl6ip5
Spi13	1.00E-53	0.460381	0.898	0.702	2.45E-49	cDC1	Spi1
Acaa1a2	1.04E-53	0.376288	0.5	0.262	2.54E-49	cDC1	Acaa1a

1110004F1	1.30E-53	0.362095	0.431	0.216	3.18E-49	cDC1	1110004F10Rik
Rbis1	1.33E-53	0.418314	0.443	0.227	3.25E-49	cDC1	Rbis
Lman23	1.92E-53	0.382092	0.55	0.3	4.71E-49	cDC1	Lman2
Riok32	2.59E-53	0.358444	0.444	0.226	6.35E-49	cDC1	Riok3
Gstp12	2.66E-53	0.364836	0.527	0.285	6.52E-49	cDC1	Gstp1
Mrps214	2.81E-53	0.331982	0.616	0.341	6.88E-49	cDC1	Mrps21
Plaat34	3.00E-53	0.580271	0.4	0.206	7.35E-49	cDC1	Plaat3
Parl	3.18E-53	0.326373	0.315	0.142	7.79E-49	cDC1	Parl
Sptssa2	3.25E-53	0.388072	0.55	0.301	7.97E-49	cDC1	Sptssa
Aldh9a1	3.29E-53	0.5954	0.259	0.11	8.07E-49	cDC1	Aldh9a1
Ndufv1	3.97E-53	0.433052	0.377	0.184	9.72E-49	cDC1	Ndufv1
Cltb1	4.15E-53	0.384126	0.35	0.165	1.02E-48	cDC1	Cltb
Ndufa84	5.17E-53	0.387062	0.577	0.32	1.27E-48	cDC1	Ndufa8
Pip5k1c	6.06E-53	0.388158	0.368	0.177	1.48E-48	cDC1	Pip5k1c
Tomm201	6.16E-53	0.424635	0.678	0.399	1.51E-48	cDC1	Tomm20
Dock102	8.03E-53	0.451592	0.869	0.604	1.97E-48	cDC1	Dock10
Reep53	9.00E-53	0.446801	0.826	0.573	2.20E-48	cDC1	Reep5
Tor1a1	1.07E-52	0.460701	0.409	0.206	2.61E-48	cDC1	Tor1a
Wdr13	1.17E-52	0.452178	0.624	0.361	2.87E-48	cDC1	Wdr1
Hsp90aa12	1.20E-52	0.398375	0.58	0.325	2.94E-48	cDC1	Hsp90aa1
Golga7	1.26E-52	0.366886	0.385	0.188	3.09E-48	cDC1	Golga7
Tuba1c1	1.42E-52	0.599999	0.267	0.116	3.47E-48	cDC1	Tuba1c
Erg28	1.42E-52	0.51313	0.292	0.13	3.48E-48	cDC1	Erg28
Sharpin2	1.59E-52	0.397794	0.415	0.209	3.89E-48	cDC1	Sharpin
Pdcd52	1.67E-52	0.310058	0.54	0.289	4.10E-48	cDC1	Pdcd5
E2f41	1.89E-52	0.477945	0.357	0.172	4.64E-48	cDC1	E2f4
Snrpb21	2.16E-52	0.518249	0.334	0.157	5.29E-48	cDC1	Snrpb2
Lamtor53	2.25E-52	0.402277	0.465	0.242	5.52E-48	cDC1	Lamtor5
Rad23b1	2.32E-52	0.355669	0.399	0.198	5.69E-48	cDC1	Rad23b
Med101	2.48E-52	0.476886	0.329	0.154	6.06E-48	cDC1	Med10
Smc3	2.80E-52	0.336464	0.292	0.13	6.85E-48	cDC1	Smc3
Pes11	3.40E-52	0.454466	0.349	0.167	8.34E-48	cDC1	Pes1
Stk382	3.52E-52	0.371891	0.434	0.221	8.62E-48	cDC1	Stk38
Pnp4	3.76E-52	0.596739	0.728	0.476	9.20E-48	cDC1	Pnp
Stat14	3.86E-52	0.522031	0.745	0.493	9.46E-48	cDC1	Stat1
Kmt5a	4.13E-52	0.488169	0.256	0.108	1.01E-47	cDC1	Kmt5a
Rassf11	4.27E-52	0.466153	0.395	0.196	1.05E-47	cDC1	Rassf1
Ndufb43	4.53E-52	0.369186	0.631	0.361	1.11E-47	cDC1	Ndufb4
Dpy30	6.06E-52	0.390902	0.326	0.151	1.48E-47	cDC1	Dpy30
Necap23	6.69E-52	0.478649	0.527	0.291	1.64E-47	cDC1	Necap2
Tcea11	6.78E-52	0.348638	0.523	0.283	1.66E-47	cDC1	Tcea1
Rnps1	8.95E-52	0.412895	0.377	0.185	2.19E-47	cDC1	Rnps1
Mien14	9.73E-52	0.350962	0.4	0.198	2.38E-47	cDC1	Mien1
Nudc3	9.91E-52	0.36148	0.512	0.276	2.43E-47	cDC1	Nudc

Frg1	1.08E-51	0.358453	0.385	0.189	2.65E-47	cDC1	Frg1
Prpf19	1.20E-51	0.440644	0.367	0.178	2.94E-47	cDC1	Prpf19
Atp5j6	1.72E-51	0.373323	0.818	0.539	4.22E-47	cDC1	Atp5j
Msn3	2.08E-51	0.458961	0.828	0.588	5.11E-47	cDC1	Msn
Pdcd101	3.54E-51	0.422493	0.495	0.265	8.68E-47	cDC1	Pdcd10
Rassf51	4.78E-51	0.36359	0.343	0.161	1.17E-46	cDC1	Rassf5
Slc25a34	5.08E-51	0.407277	0.863	0.607	1.24E-46	cDC1	Slc25a3
Ciao2b2	5.96E-51	0.490391	0.458	0.24	1.46E-46	cDC1	Ciao2b
Drap14	6.06E-51	0.364315	0.608	0.345	1.48E-46	cDC1	Drap1
Idnk2	6.29E-51	0.456721	0.422	0.217	1.54E-46	cDC1	Idnk
Clic12	6.66E-51	0.375327	0.916	0.756	1.63E-46	cDC1	Clic1
Naa382	8.50E-51	0.335227	0.458	0.239	2.08E-46	cDC1	Naa38
Hnrnpc1	1.03E-50	0.264091	0.468	0.244	2.53E-46	cDC1	Hnrnpc
Bola24	1.17E-50	0.399303	0.435	0.226	2.87E-46	cDC1	Bola2
Pdrg1	1.42E-50	0.402455	0.284	0.126	3.48E-46	cDC1	Pdrg1
Coa33	1.55E-50	0.280796	0.527	0.28	3.79E-46	cDC1	Coa3
Pet1001	2.37E-50	0.486143	0.327	0.156	5.80E-46	cDC1	Pet100
Gdi2	2.83E-50	0.490424	0.855	0.652	6.93E-46	cDC1	Gdi2
Ube2m3	3.41E-50	0.369865	0.556	0.309	8.35E-46	cDC1	Ube2m
Prcp2	4.04E-50	0.347715	0.629	0.362	9.90E-46	cDC1	Prcp
Mybbp1a1	4.56E-50	0.427775	0.323	0.153	1.12E-45	cDC1	Mybbp1a
Tax1bp13	4.63E-50	0.332358	0.608	0.348	1.13E-45	cDC1	Tax1bp1
Ppp2r5a2	4.72E-50	0.317534	0.397	0.199	1.16E-45	cDC1	Ppp2r5a
Cbfb	4.93E-50	0.275753	0.441	0.225	1.21E-45	cDC1	Cbfb
Phactr21	5.36E-50	0.486124	0.441	0.228	1.31E-45	cDC1	Phactr2
Ddx21	6.77E-50	0.390935	0.334	0.159	1.66E-45	cDC1	Ddx21
Ndufab13	7.03E-50	0.369724	0.581	0.33	1.72E-45	cDC1	Ndufab1
Arhgap1	8.01E-50	0.483714	0.265	0.116	1.96E-45	cDC1	Arhgap1
Brix1	8.20E-50	0.527443	0.271	0.12	2.01E-45	cDC1	Brix1
Gtf3a	8.39E-50	0.488019	0.261	0.115	2.05E-45	cDC1	Gtf3a
Glod4	1.34E-49	0.49664	0.328	0.157	3.29E-45	cDC1	Glod4
Gtf2a21	1.52E-49	0.406018	0.415	0.212	3.72E-45	cDC1	Gtf2a2
Fmnl22	1.56E-49	0.469597	0.527	0.298	3.82E-45	cDC1	Fmnl2
Sf3b41	1.59E-49	0.349417	0.383	0.19	3.91E-45	cDC1	Sf3b4
Cyld	1.60E-49	0.358278	0.412	0.208	3.92E-45	cDC1	Cyld
Rsl24d1	1.67E-49	0.559059	0.289	0.132	4.08E-45	cDC1	Rsl24d1
Mrps71	1.95E-49	0.410974	0.34	0.162	4.78E-45	cDC1	Mrps7
Parp13	1.98E-49	0.50016	0.337	0.164	4.84E-45	cDC1	Parp1
Commd12	2.14E-49	0.290186	0.421	0.215	5.25E-45	cDC1	Commd1
Cpne32	2.61E-49	0.37652	0.411	0.21	6.39E-45	cDC1	Cpne3
Ndufs43	4.04E-49	0.324818	0.537	0.296	9.88E-45	cDC1	Ndufs4
Med284	6.86E-49	0.336803	0.625	0.354	1.68E-44	cDC1	Med28
Rtf21	7.47E-49	0.343945	0.444	0.232	1.83E-44	cDC1	Rtf2
Dap1	8.03E-49	0.456541	0.322	0.154	1.97E-44	cDC1	Dap

2310011J0:	9.62E-49	0.410938	0.3	0.139	2.36E-44	cDC1	2310011J03Rik
Tpm3	9.74E-49	0.390845	0.855	0.624	2.38E-44	cDC1	Tpm3
Eva1b	1.35E-48	0.580904	0.26	0.115	3.31E-44	cDC1	Eva1b
Mrpl551	1.48E-48	0.409583	0.288	0.131	3.62E-44	cDC1	Mrpl55
Anapc112	2.31E-48	0.365994	0.6	0.346	5.65E-44	cDC1	Anapc11
Ndufa101	2.42E-48	0.395666	0.43	0.224	5.92E-44	cDC1	Ndufa10
Eapp1	2.64E-48	0.414421	0.321	0.152	6.47E-44	cDC1	Eapp
Ube2k2	2.65E-48	0.297096	0.513	0.277	6.48E-44	cDC1	Ube2k
Tmem50b3	2.78E-48	0.299366	0.455	0.237	6.80E-44	cDC1	Tmem50b
Ufc12	3.51E-48	0.33057	0.478	0.257	8.59E-44	cDC1	Ufc1
Csnk1g2	3.70E-48	0.407443	0.353	0.173	9.07E-44	cDC1	Csnk1g2
Gmfg4	3.87E-48	0.317677	0.643	0.382	9.48E-44	cDC1	Gmfg
Nip7	8.82E-48	0.472229	0.259	0.115	2.16E-43	cDC1	Nip7
Tax1bp31	8.92E-48	0.486398	0.315	0.151	2.18E-43	cDC1	Tax1bp3
Tspan331	1.09E-47	0.425233	0.318	0.151	2.68E-43	cDC1	Tspan33
Zc3h15	1.67E-47	0.295655	0.378	0.189	4.08E-43	cDC1	Zc3h15
1700123O2	1.82E-47	0.38494	0.282	0.128	4.45E-43	cDC1	1700123O20Rik
Ssu723	1.84E-47	0.337531	0.621	0.363	4.50E-43	cDC1	Ssu72
Cyb561a34	1.93E-47	0.388547	0.531	0.295	4.73E-43	cDC1	Cyb561a3
Rp92	1.98E-47	0.277944	0.539	0.299	4.84E-43	cDC1	Rp9
Ddit41	2.07E-47	1.041595	0.264	0.122	5.08E-43	cDC1	Ddit4
Emc2	2.17E-47	0.332375	0.375	0.188	5.33E-43	cDC1	Emc2
Ddx54	2.36E-47	0.522125	0.265	0.12	5.79E-43	cDC1	Ddx54
Stxbp23	2.89E-47	0.47418	0.345	0.172	7.07E-43	cDC1	Stxbp2
Nelfe1	2.96E-47	0.409343	0.266	0.12	7.25E-43	cDC1	Nelfe
Eif2s3x	3.90E-47	0.481642	0.294	0.138	9.54E-43	cDC1	Eif2s3x
Bcas22	4.12E-47	0.350871	0.427	0.223	1.01E-42	cDC1	Bcas2
Strn4	4.44E-47	0.512274	0.253	0.111	1.09E-42	cDC1	Strn4
Apopt1	4.45E-47	0.406336	0.319	0.153	1.09E-42	cDC1	Apopt1
Hcfc1r15	4.77E-47	0.302864	0.486	0.262	1.17E-42	cDC1	Hcfc1r1
Ywhaq	5.21E-47	0.350097	0.315	0.149	1.28E-42	cDC1	Ywhaq
Hnrnpdl	5.61E-47	0.292908	0.527	0.289	1.37E-42	cDC1	Hnrnpdl
Lemd2	6.62E-47	0.411339	0.348	0.172	1.62E-42	cDC1	Lemd2
Gatd3a	7.19E-47	0.388818	0.276	0.126	1.76E-42	cDC1	Gatd3a
Phax	7.99E-47	0.459004	0.334	0.164	1.96E-42	cDC1	Phax
Lsm31	8.38E-47	0.40051	0.305	0.145	2.05E-42	cDC1	Lsm3
Thoc7	9.55E-47	0.357304	0.373	0.188	2.34E-42	cDC1	Thoc7
Isyna1	1.03E-46	0.384425	0.33	0.16	2.52E-42	cDC1	Isyna1
Commd41	1.15E-46	0.275581	0.466	0.248	2.82E-42	cDC1	Commd4
Ssb	1.16E-46	0.333995	0.54	0.302	2.84E-42	cDC1	Ssb
Coq10b1	1.23E-46	0.542666	0.28	0.129	3.00E-42	cDC1	Coq10b
Lipe	1.23E-46	0.382016	0.286	0.132	3.02E-42	cDC1	Lipe
Fkbp4	1.81E-46	0.361937	0.326	0.157	4.43E-42	cDC1	Fkbp4
Arl6ip4	2.42E-46	0.372858	0.395	0.204	5.93E-42	cDC1	Arl6ip4

Uqcrh5	2.57E-46	0.385379	0.845	0.595	6.28E-42	cDC1	Uqcrh
Paics	2.73E-46	0.370549	0.345	0.171	6.69E-42	cDC1	Paics
Znrd2	2.77E-46	0.388785	0.251	0.111	6.78E-42	cDC1	Znrd2
Uba12	3.13E-46	0.32627	0.396	0.204	7.68E-42	cDC1	Uba1
Slamf92	3.58E-46	0.816538	0.379	0.207	8.78E-42	cDC1	Slamf9
Kdm7a3	3.68E-46	0.376897	0.473	0.255	9.02E-42	cDC1	Kdm7a
Gapdh4	3.96E-46	0.393704	0.844	0.625	9.71E-42	cDC1	Gapdh
Chuk	4.57E-46	0.422642	0.257	0.114	1.12E-41	cDC1	Chuk
Nolc1	5.32E-46	0.435662	0.257	0.115	1.30E-41	cDC1	Nolc1
Fcgrt1	6.28E-46	0.304455	0.654	0.39	1.54E-41	cDC1	Fcgrt
Uba2	6.82E-46	0.365308	0.371	0.187	1.67E-41	cDC1	Uba2
Trp53	7.34E-46	0.301817	0.438	0.232	1.80E-41	cDC1	Trp53
Rnf220	7.92E-46	0.354445	0.37	0.186	1.94E-41	cDC1	Rnf220
Ube2l32	1.01E-45	0.347041	0.538	0.301	2.47E-41	cDC1	Ube2l3
Coro1c2	1.16E-45	0.282608	0.383	0.194	2.83E-41	cDC1	Coro1c
Ctdnep12	1.22E-45	0.400983	0.408	0.216	3.00E-41	cDC1	Ctdnep1
Rnf1142	1.37E-45	0.418236	0.432	0.231	3.35E-41	cDC1	Rnf114
Dynlrb14	2.03E-45	0.288533	0.56	0.318	4.97E-41	cDC1	Dynlrb1
Csf2rb22	2.11E-45	0.780844	0.301	0.147	5.18E-41	cDC1	Csf2rb2
Zcrb11	2.23E-45	0.262257	0.454	0.242	5.46E-41	cDC1	Zcrb1
Icosl1	3.03E-45	0.372818	0.339	0.166	7.41E-41	cDC1	Icosl
Eif4a14	3.40E-45	0.415815	0.817	0.571	8.32E-41	cDC1	Eif4a1
Twf23	3.53E-45	0.320897	0.69	0.423	8.64E-41	cDC1	Twf2
Smc61	3.60E-45	0.330203	0.313	0.151	8.82E-41	cDC1	Smc6
Hspd13	4.07E-45	0.30568	0.429	0.226	9.98E-41	cDC1	Hspd1
Baz2b2	5.51E-45	0.30671	0.643	0.38	1.35E-40	cDC1	Baz2b
Mrpl342	6.21E-45	0.30172	0.353	0.177	1.52E-40	cDC1	Mrpl34
Ndufa52	6.21E-45	0.295144	0.435	0.232	1.52E-40	cDC1	Ndufa5
Acp11	6.25E-45	0.325486	0.31	0.149	1.53E-40	cDC1	Acp1
Tap22	6.41E-45	0.468771	0.617	0.386	1.57E-40	cDC1	Tap2
Mrpl112	7.33E-45	0.364889	0.321	0.156	1.80E-40	cDC1	Mrpl11
Ube2e12	7.37E-45	0.258809	0.474	0.257	1.81E-40	cDC1	Ube2e1
Med27	8.33E-45	0.321142	0.251	0.111	2.04E-40	cDC1	Med27
Gsto12	8.76E-45	0.656507	0.288	0.138	2.14E-40	cDC1	Gsto1
Tufm1	9.83E-45	0.401243	0.294	0.14	2.41E-40	cDC1	Tufm
Aip3	1.02E-44	0.385555	0.388	0.202	2.50E-40	cDC1	Aip
Ddt2	1.09E-44	0.600021	0.333	0.168	2.68E-40	cDC1	Ddt
Mia22	1.17E-44	0.273169	0.585	0.336	2.86E-40	cDC1	Mia2
Zmat22	1.22E-44	0.339325	0.424	0.226	2.98E-40	cDC1	Zmat2
Dnajc192	1.49E-44	0.32131	0.495	0.275	3.64E-40	cDC1	Dnajc19
Cdc42se23	1.52E-44	0.258692	0.493	0.271	3.72E-40	cDC1	Cdc42se2
Rae1	1.59E-44	0.333294	0.272	0.125	3.89E-40	cDC1	Rae1
Was2	1.59E-44	0.371612	0.445	0.238	3.90E-40	cDC1	Was
Tln14	1.96E-44	0.378913	0.737	0.474	4.81E-40	cDC1	Tln1

Sdhd3	2.79E-44	0.326272	0.494	0.273	6.83E-40	cDC1	Sdhd
Hax1	2.82E-44	0.516476	0.268	0.125	6.90E-40	cDC1	Hax1
Rpl7l1	3.49E-44	0.305886	0.361	0.182	8.54E-40	cDC1	Rpl7l1
Trappc12	4.08E-44	0.373511	0.387	0.203	9.99E-40	cDC1	Trappc1
Plekhf22	4.20E-44	0.420329	0.353	0.179	1.03E-39	cDC1	Plekhf2
Igsf82	4.20E-44	0.467684	0.419	0.227	1.03E-39	cDC1	Igsf8
Snrnp271	4.70E-44	0.339255	0.324	0.159	1.15E-39	cDC1	Snrnp27
Plekhj12	5.43E-44	0.296987	0.529	0.294	1.33E-39	cDC1	Plekhj1
Spag7	5.77E-44	0.343593	0.328	0.162	1.41E-39	cDC1	Spag7
Ilkap	6.45E-44	0.279679	0.328	0.162	1.58E-39	cDC1	Ilkap
Hnrnpa2b1	6.46E-44	0.361662	0.894	0.717	1.58E-39	cDC1	Hnrnpa2b1
Eif1ad1	8.41E-44	0.421792	0.255	0.116	2.06E-39	cDC1	Eif1ad
Ube2a2	9.03E-44	0.325189	0.424	0.225	2.21E-39	cDC1	Ube2a
Ralbp13	9.58E-44	0.251293	0.438	0.234	2.35E-39	cDC1	Ralbp1
Slc8b1	1.28E-43	0.710001	0.268	0.128	3.13E-39	cDC1	Slc8b1
Samm501	1.38E-43	0.307202	0.361	0.184	3.38E-39	cDC1	Samm50
Wdr61	1.51E-43	0.411507	0.283	0.133	3.71E-39	cDC1	Wdr61
Psmc32	1.87E-43	0.309431	0.516	0.291	4.59E-39	cDC1	Psmc3
Cggbp11	1.95E-43	0.343126	0.362	0.185	4.78E-39	cDC1	Cggbp1
Gm162862	1.99E-43	0.33397	0.432	0.232	4.88E-39	cDC1	Gm16286
Zfas13	2.04E-43	0.507278	0.362	0.189	4.99E-39	cDC1	Zfas1
Ube2v15	2.88E-43	0.28489	0.505	0.282	7.06E-39	cDC1	Ube2v1
Plcb2	3.92E-43	0.296547	0.35	0.176	9.60E-39	cDC1	Plcb2
Parp81	4.09E-43	0.48539	0.523	0.296	1.00E-38	cDC1	Parp8
AI4135823	4.53E-43	0.313823	0.567	0.325	1.11E-38	cDC1	AI413582
Polb	4.61E-43	0.414637	0.307	0.15	1.13E-38	cDC1	Polb
Nampt3	5.24E-43	0.424748	0.371	0.195	1.28E-38	cDC1	Nampt
Ube2i1	5.35E-43	0.321844	0.681	0.419	1.31E-38	cDC1	Ube2i
Sbno21	5.88E-43	0.368177	0.373	0.194	1.44E-38	cDC1	Sbno2
Tbc1d91	6.71E-43	0.44135	0.616	0.373	1.64E-38	cDC1	Tbc1d9
Slirp3	7.56E-43	0.340894	0.325	0.161	1.85E-38	cDC1	Slirp
Ctdsp2	8.18E-43	0.306334	0.266	0.122	2.00E-38	cDC1	Ctdsp2
Ncaph2	9.05E-43	0.457077	0.278	0.132	2.22E-38	cDC1	Ncaph2
Gfer1	9.64E-43	0.372788	0.376	0.196	2.36E-38	cDC1	Gfer
Mrpl411	1.07E-42	0.347647	0.276	0.129	2.62E-38	cDC1	Mrpl41
Epb41l4aos	1.46E-42	0.576951	0.268	0.127	3.58E-38	cDC1	Epb41l4aos
Khdrbs11	1.63E-42	0.269433	0.612	0.364	3.99E-38	cDC1	Khdrbs1
Cetn3	1.81E-42	0.26728	0.412	0.219	4.44E-38	cDC1	Cetn3
Mrpl173	2.30E-42	0.365421	0.473	0.262	5.64E-38	cDC1	Mrpl17
Timm101	3.15E-42	0.4607	0.252	0.116	7.71E-38	cDC1	Timm10
Ostc2	3.22E-42	0.37469	0.558	0.33	7.88E-38	cDC1	Ostc
Nabp2	3.75E-42	0.328332	0.339	0.171	9.18E-38	cDC1	Nabp2
Cdk42	3.95E-42	0.407707	0.45	0.25	9.66E-38	cDC1	Cdk4
Smu1	4.30E-42	0.344613	0.343	0.174	1.05E-37	cDC1	Smu1

AB124611€	4.53E-42	0.420196	0.325	0.165	1.11E-37	cDC1	AB124611
Tcof11	5.50E-42	0.562867	0.282	0.137	1.35E-37	cDC1	Tcof1
Pcna1	7.34E-42	0.559033	0.263	0.125	1.80E-37	cDC1	Pcna
Glrx51	9.04E-42	0.317334	0.352	0.181	2.21E-37	cDC1	Glrx5
Use12	1.31E-41	0.260382	0.465	0.254	3.21E-37	cDC1	Use1
H2-T224	1.31E-41	0.375524	0.582	0.342	3.21E-37	cDC1	H2-T22
Commd2	1.34E-41	0.407956	0.293	0.143	3.28E-37	cDC1	Commd2
Ppm1g	1.37E-41	0.320091	0.436	0.236	3.36E-37	cDC1	Ppm1g
Nudt141	1.76E-41	0.437989	0.25	0.116	4.31E-37	cDC1	Nudt14
Exosc41	1.76E-41	0.553927	0.256	0.12	4.32E-37	cDC1	Exosc4
Npepl1	2.32E-41	0.376437	0.331	0.168	5.68E-37	cDC1	Npepl1
Eif2a	2.81E-41	0.287845	0.316	0.157	6.88E-37	cDC1	Eif2a
Sptan11	3.03E-41	0.301236	0.319	0.16	7.42E-37	cDC1	Sptan1
Rras3	3.03E-41	0.347557	0.288	0.14	7.42E-37	cDC1	Rras
Uqcc3	3.33E-41	0.291344	0.347	0.178	8.17E-37	cDC1	Uqcc3
Myh93	3.43E-41	0.378003	0.815	0.582	8.40E-37	cDC1	Myh9
Ghitm1	3.57E-41	0.324475	0.542	0.312	8.74E-37	cDC1	Ghitm
Brk13	3.70E-41	0.339032	0.752	0.482	9.06E-37	cDC1	Brk1
Trir2	4.35E-41	0.303174	0.597	0.35	1.06E-36	cDC1	Trir
Frmd8	5.53E-41	0.369971	0.255	0.119	1.36E-36	cDC1	Frmd8
Puf601	5.79E-41	0.262586	0.504	0.284	1.42E-36	cDC1	Puf60
Dnaja23	6.59E-41	0.258691	0.528	0.301	1.61E-36	cDC1	Dnaja2
Gnb13	6.69E-41	0.261131	0.756	0.484	1.64E-36	cDC1	Gnb1
Mdh11	7.47E-41	0.287919	0.63	0.386	1.83E-36	cDC1	Mdh1
Cyc11	1.14E-40	0.316569	0.521	0.299	2.80E-36	cDC1	Cyc1
Dcp21	1.30E-40	0.52053	0.276	0.134	3.19E-36	cDC1	Dcp2
Eif4e3	1.81E-40	0.423	0.26	0.123	4.43E-36	cDC1	Eif4e3
Utp32	1.97E-40	0.312132	0.363	0.191	4.82E-36	cDC1	Utp3
Mtch2	2.55E-40	0.260064	0.419	0.226	6.23E-36	cDC1	Mtch2
Mrpl25	2.93E-40	0.345706	0.305	0.152	7.17E-36	cDC1	Mrpl2
Hint21	3.21E-40	0.292089	0.348	0.18	7.86E-36	cDC1	Hint2
Mink1	3.42E-40	0.387134	0.274	0.132	8.37E-36	cDC1	Mink1
Ramac	4.25E-40	0.266524	0.306	0.152	1.04E-35	cDC1	Ramac
Lrmp	5.82E-40	0.262258	0.322	0.161	1.43E-35	cDC1	Lrmp
Sar1a	8.61E-40	0.342413	0.354	0.185	2.11E-35	cDC1	Sar1a
Tspyl1	8.74E-40	0.381951	0.261	0.124	2.14E-35	cDC1	Tspyl1
Gatad1	8.99E-40	0.260549	0.261	0.123	2.20E-35	cDC1	Gatad1
Hnrnpll	1.13E-39	0.385787	0.251	0.118	2.78E-35	cDC1	Hnrnpll
Nr1h2	1.25E-39	0.281807	0.365	0.192	3.05E-35	cDC1	Nr1h2
Baz2a1	1.30E-39	0.362319	0.297	0.147	3.18E-35	cDC1	Baz2a
Tapbp3	1.37E-39	0.38895	0.747	0.499	3.36E-35	cDC1	Tapbp
Tbc1d11	1.39E-39	0.423708	0.321	0.163	3.40E-35	cDC1	Tbc1d1
Lypla1	1.45E-39	0.279081	0.272	0.13	3.56E-35	cDC1	Lypla1
Pigs	1.53E-39	0.351083	0.307	0.153	3.74E-35	cDC1	Pigs

Ubl71	1.76E-39	0.383958	0.285	0.14	4.32E-35	cDC1	Ubl7
Fgfr1op22	1.92E-39	0.2764	0.468	0.263	4.70E-35	cDC1	Fgfr1op2
Zfp622	1.96E-39	0.448193	0.261	0.125	4.79E-35	cDC1	Zfp622
Trim12c1	2.38E-39	0.384993	0.286	0.141	5.83E-35	cDC1	Trim12c
Mctp13	2.72E-39	0.313232	0.573	0.337	6.65E-35	cDC1	Mctp1
Wdr83os2	2.90E-39	0.276825	0.632	0.385	7.09E-35	cDC1	Wdr83os
Pnkp1	4.07E-39	0.27498	0.276	0.133	9.97E-35	cDC1	Pnkp
Tsn	5.04E-39	0.298343	0.439	0.246	1.23E-34	cDC1	Tsn
Polr2i1	5.91E-39	0.320095	0.338	0.175	1.45E-34	cDC1	Polr2i
Evi2a3	8.02E-39	0.274297	0.507	0.289	1.97E-34	cDC1	Evi2a
Mfsd14a	8.92E-39	0.255644	0.33	0.167	2.19E-34	cDC1	Mfsd14a
Qars2	9.02E-39	0.358768	0.311	0.158	2.21E-34	cDC1	Qars
Nipsnap3b:	9.75E-39	0.369131	0.281	0.139	2.39E-34	cDC1	Nipsnap3b
Pop7	1.37E-38	0.36614	0.264	0.128	3.34E-34	cDC1	Pop7
Ech11	1.47E-38	0.270005	0.466	0.264	3.59E-34	cDC1	Ech1
Rel2	1.60E-38	0.312395	0.504	0.287	3.91E-34	cDC1	Rel
Ptpn111	1.94E-38	0.254531	0.303	0.152	4.75E-34	cDC1	Ptpn11
Coa52	2.03E-38	0.287989	0.294	0.146	4.97E-34	cDC1	Coa5
Vrk3	2.07E-38	0.300892	0.292	0.144	5.07E-34	cDC1	Vrk3
Gtf2b	2.43E-38	0.332535	0.292	0.145	5.96E-34	cDC1	Gtf2b
Acsl51	2.74E-38	0.294714	0.389	0.21	6.72E-34	cDC1	Acsl5
Med81	5.10E-38	0.272637	0.319	0.162	1.25E-33	cDC1	Med8
Atp5mpl6	6.96E-38	0.268359	0.667	0.415	1.70E-33	cDC1	Atp5mpl
Ccdc863	7.01E-38	0.440637	0.319	0.166	1.72E-33	cDC1	Ccdc86
Dda1	7.65E-38	0.317586	0.301	0.153	1.87E-33	cDC1	Dda1
Aebp2	8.96E-38	0.338593	0.253	0.121	2.19E-33	cDC1	Aebp2
Ptbp1	1.04E-37	0.270152	0.365	0.194	2.54E-33	cDC1	Ptbp1
Nutf21	1.15E-37	0.365745	0.384	0.21	2.81E-33	cDC1	Nutf2
Synj12	1.45E-37	0.286218	0.514	0.296	3.55E-33	cDC1	Synj1
Rnf181	1.65E-37	0.318878	0.284	0.141	4.04E-33	cDC1	Rnf181
Neurl32	1.85E-37	0.377171	0.403	0.226	4.52E-33	cDC1	Neurl3
Ext12	2.41E-37	0.44725	0.403	0.222	5.90E-33	cDC1	Ext1
Txndc5	2.50E-37	0.412356	0.259	0.126	6.11E-33	cDC1	Txndc5
Snx102	2.76E-37	0.271907	0.448	0.253	6.77E-33	cDC1	Snx10
Capzb1	3.02E-37	0.332764	0.884	0.683	7.38E-33	cDC1	Capzb
Sf3a3	3.58E-37	0.423732	0.264	0.13	8.76E-33	cDC1	Sf3a3
Nsmaf1	3.83E-37	0.256915	0.307	0.156	9.38E-33	cDC1	Nsmaf
Fnbp11	4.40E-37	0.420837	0.619	0.394	1.08E-32	cDC1	Fnbp1
Rpp25l	4.85E-37	0.256492	0.257	0.124	1.19E-32	cDC1	Rpp25l
Anapc5	8.90E-37	0.306884	0.415	0.233	2.18E-32	cDC1	Anapc5
Med291	1.04E-36	0.396657	0.257	0.126	2.55E-32	cDC1	Med29
Flnb	1.10E-36	0.298975	0.261	0.126	2.70E-32	cDC1	Flnb
Mrps18a2	1.22E-36	0.264385	0.365	0.196	2.99E-32	cDC1	Mrps18a
Fis15	1.50E-36	0.25241	0.742	0.464	3.68E-32	cDC1	Fis1

Dars	1.94E-36	0.296659	0.342	0.181	4.74E-32	cDC1	Dars
AI467606	2.13E-36	0.393973	0.263	0.128	5.21E-32	cDC1	AI467606
Paxbp1	3.09E-36	0.312088	0.277	0.138	7.57E-32	cDC1	Paxbp1
Hspa92	3.81E-36	0.296484	0.369	0.201	9.33E-32	cDC1	Hspa9
Bud31	4.56E-36	0.306879	0.28	0.141	1.12E-31	cDC1	Bud31
Odc11	4.62E-36	0.365512	0.256	0.125	1.13E-31	cDC1	Odc1
Ccdc115	5.67E-36	0.31129	0.253	0.122	1.39E-31	cDC1	Ccdc115
Sf3b24	7.57E-36	0.267905	0.606	0.372	1.85E-31	cDC1	Sf3b2
Lars	1.32E-35	0.277814	0.35	0.189	3.22E-31	cDC1	Lars
Tmem1921	1.47E-35	0.4074	0.271	0.136	3.60E-31	cDC1	Tmem192
Sgms12	2.23E-35	0.378567	0.344	0.184	5.45E-31	cDC1	Sgms1
Wbp11	2.36E-35	0.362462	0.256	0.126	5.78E-31	cDC1	Wbp11
Scarb11	3.33E-35	0.390845	0.266	0.133	8.15E-31	cDC1	Scarb1
Nub1	3.73E-35	0.273981	0.369	0.201	9.12E-31	cDC1	Nub1
Rnf4	5.47E-35	0.257187	0.291	0.148	1.34E-30	cDC1	Rnf4
Hnrnpu1	9.61E-35	0.306085	0.714	0.481	2.35E-30	cDC1	Hnrnpu
Foxp12	1.32E-34	0.41742	0.699	0.459	3.22E-30	cDC1	Foxp1
Dtx3l2	1.39E-34	0.464079	0.299	0.158	3.41E-30	cDC1	Dtx3l
Mtln1	1.43E-34	0.326195	0.308	0.163	3.51E-30	cDC1	Mtln
Herpud13	1.57E-34	0.388759	0.6	0.383	3.84E-30	cDC1	Herpud1
Med211	2.38E-34	0.332099	0.285	0.146	5.82E-30	cDC1	Med21
Uvrag2	3.31E-34	0.322017	0.69	0.441	8.11E-30	cDC1	Uvrag
Trim30a3	4.24E-34	0.300497	0.529	0.322	1.04E-29	cDC1	Trim30a
Rftn13	4.47E-34	0.288826	0.284	0.147	1.09E-29	cDC1	Rftn1
Atp11b1	2.06E-33	0.319774	0.276	0.141	5.06E-29	cDC1	Atp11b
Lrba2	3.41E-33	0.413544	0.363	0.197	8.35E-29	cDC1	Lrba
Aff11	3.43E-33	0.275802	0.411	0.233	8.41E-29	cDC1	Aff1
Iah12	3.88E-33	0.359397	0.284	0.147	9.50E-29	cDC1	Iah1
Prpf81	4.64E-33	0.26194	0.441	0.256	1.14E-28	cDC1	Prpf8
Snrnp2001	6.39E-33	0.395637	0.273	0.142	1.57E-28	cDC1	Snrnp200
Arpc42	2.34E-32	0.300565	0.83	0.596	5.72E-28	cDC1	Arpc4
Nfkbia5	3.54E-32	0.397567	0.715	0.485	8.67E-28	cDC1	Nfkbia
Slbp	3.57E-32	0.264179	0.281	0.146	8.73E-28	cDC1	Slbp
Ms4a6b3	5.15E-32	0.367084	0.481	0.294	1.26E-27	cDC1	Ms4a6b
Rfc1	5.35E-32	0.265973	0.268	0.138	1.31E-27	cDC1	Rfc1
Tifab3	8.67E-32	0.311455	0.497	0.303	2.12E-27	cDC1	Tifab
Zcchc17	8.71E-32	0.28216	0.261	0.133	2.13E-27	cDC1	Zcchc17
Tpgs1	1.06E-31	0.262255	0.269	0.139	2.58E-27	cDC1	Tpgs1
Lrrc592	6.98E-31	0.310848	0.276	0.145	1.71E-26	cDC1	Lrrc59
Cxcl163	9.23E-31	0.309364	0.489	0.299	2.26E-26	cDC1	Cxcl16
Nfkb11	1.49E-30	0.347129	0.683	0.467	3.65E-26	cDC1	Nfkb1
Get4	2.87E-30	0.312728	0.253	0.131	7.02E-26	cDC1	Get4
Grb21	3.45E-30	0.267457	0.771	0.511	8.44E-26	cDC1	Grb2
Relb1	3.86E-30	0.443279	0.302	0.167	9.46E-26	cDC1	Relb

Ywhah3	4.36E-30	0.280865	0.813	0.6	1.07E-25	cDC1	Ywhah
Ppib3	4.57E-30	0.263594	0.88	0.67	1.12E-25	cDC1	Ppib
Slc46a32	5.35E-30	0.272606	0.396	0.23	1.31E-25	cDC1	Slc46a3
Zfp36l23	6.76E-30	0.356	0.723	0.521	1.66E-25	cDC1	Zfp36l2
Pltp2	8.75E-30	0.584486	0.26	0.14	2.14E-25	cDC1	Pltp
Myo1c1	2.38E-29	0.31491	0.278	0.149	5.83E-25	cDC1	Myo1c
Sbds	7.69E-29	0.25617	0.351	0.199	1.88E-24	cDC1	Sbds
Trib1	8.13E-29	0.44279	0.257	0.137	1.99E-24	cDC1	Trib1
Parp122	1.02E-28	0.297799	0.303	0.167	2.50E-24	cDC1	Parp12
Ptpn65	1.03E-28	0.320047	0.739	0.51	2.53E-24	cDC1	Ptpn6
Tent5a4	1.18E-28	0.484998	0.405	0.252	2.89E-24	cDC1	Tent5a
Tbc1d82	3.06E-28	0.41873	0.4	0.24	7.49E-24	cDC1	Tbc1d8
1600014C1	6.41E-28	0.334429	0.319	0.181	1.57E-23	cDC1	1600014C10Rik
Creld22	9.73E-28	0.725281	0.302	0.173	2.38E-23	cDC1	Creld2
Casp13	1.70E-27	0.338827	0.315	0.18	4.17E-23	cDC1	Casp1
Etv31	7.01E-27	0.376181	0.272	0.151	1.72E-22	cDC1	Etv3
Slc35b11	1.29E-26	0.254811	0.349	0.204	3.16E-22	cDC1	Slc35b1
Ptpn182	1.65E-26	0.272181	0.855	0.646	4.04E-22	cDC1	Ptpn18
Ifih12	1.45E-25	0.296518	0.276	0.156	3.56E-21	cDC1	Ifih1
Rps294	4.14E-25	0.285777	0.949	0.853	1.01E-20	cDC1	Rps29
Cdk85	8.36E-24	0.383015	0.978	0.9	2.05E-19	cDC1	Cdk8
Mif4gd2	3.16E-23	0.339682	0.315	0.187	7.75E-19	cDC1	Mif4gd
Manf2	6.15E-22	0.413002	0.587	0.402	1.51E-17	cDC1	Manf
Milr14	7.58E-22	0.264501	0.276	0.162	1.86E-17	cDC1	Milr1
Arl112	5.00E-21	0.25838	0.276	0.162	1.23E-16	cDC1	Arl11
Sult1a11	6.89E-16	0.353827	0.258	0.162	1.69E-11	cDC1	Sult1a1
Gbp24	1.76E-14	0.575474	0.297	0.203	4.30E-10	cDC1	Gbp2
Sdf2l14	1.71E-12	0.374611	0.546	0.399	4.19E-08	cDC1	Sdf2l1
Nupr13	4.12E-10	0.465285	0.257	0.184	1.01E-05	cDC1	Nupr1
Ppt12	5.73E-08	1.249111	0.512	0.451	0.001403	cDC1	Ppt1
Mrc12	0	4.085687	0.815	0.157	0	BAMs	Mrc1
Dab21	0	4.341666	0.691	0.117	0	BAMs	Dab2
Arhgap155	0	3.570112	0.826	0.272	0	BAMs	Arhgap15
Aoah2	0	4.225096	0.656	0.121	0	BAMs	Aoah
F13a12	0	4.49132	0.617	0.085	0	BAMs	F13a1
Itsn11	0	4.634582	0.612	0.102	0	BAMs	Itsn1
Rnf1502	0	4.103319	0.634	0.125	0	BAMs	Rnf150
Trps14	0	3.646975	0.659	0.162	0	BAMs	Trps1
Myo5a3	0	3.511522	0.742	0.248	0	BAMs	Myo5a
Dennd1a3	0	3.215436	0.827	0.347	0	BAMs	Dennd1a
Psd31	0	4.787562	0.528	0.074	0	BAMs	Psd3
Pid15	0	2.896208	0.832	0.41	0	BAMs	Pid1
Pde7b2	0	4.603207	0.409	0.054	0	BAMs	Pde7b
Iqgap23	0	3.744203	0.425	0.076	0	BAMs	Iqgap2

Vav35	0	3.955474	0.415	0.075	0	BAMs	Vav3
Nrg2	0	4.946787	0.28	0.029	0	BAMs	Nrg2
Rbpj2	5.31E-303	4.020931	0.69	0.259	1.30E-298	BAMs	Rbpj
Malat11	7.35E-296	1.531626	0.996	0.977	1.80E-291	BAMs	Malat1
Sash12	1.47E-288	3.519767	0.524	0.136	3.60E-284	BAMs	Sash1
Cd1631	2.64E-276	3.944175	0.254	0.031	6.47E-272	BAMs	Cd163
Slc9a92	1.40E-265	2.754148	0.876	0.638	3.42E-261	BAMs	Slc9a9
Dse2	4.32E-265	3.334394	0.546	0.159	1.06E-260	BAMs	Dse
Hdac91	7.61E-265	2.80321	0.753	0.335	1.86E-260	BAMs	Hdac9
Zeb22	7.44E-264	1.970742	0.943	0.758	1.82E-259	BAMs	Zeb2
Stab11	1.60E-252	2.886114	0.753	0.365	3.93E-248	BAMs	Stab1
Ext13	2.62E-244	3.006258	0.618	0.22	6.42E-240	BAMs	Ext1
Wwp11	1.91E-243	3.346911	0.547	0.174	4.68E-239	BAMs	Wwp1
Samd4	2.37E-237	4.287651	0.265	0.04	5.80E-233	BAMs	Samd4
Ifi2075	3.31E-235	3.194903	0.546	0.174	8.11E-231	BAMs	Ifi207
Colec121	1.09E-221	4.547524	0.265	0.042	2.68E-217	BAMs	Colec12
Mtss11	1.25E-213	3.103816	0.525	0.173	3.07E-209	BAMs	Mtss1
Arhgef31	1.71E-207	3.652598	0.389	0.096	4.19E-203	BAMs	Arhgef3
Ms4a72	1.48E-198	2.034049	0.445	0.112	3.62E-194	BAMs	Ms4a7
Zswim61	5.47E-192	2.157294	0.775	0.486	1.34E-187	BAMs	Zswim6
Gnaq1	1.23E-189	2.092062	0.787	0.518	3.02E-185	BAMs	Gnaq
Frmd4b1	4.54E-189	2.012164	0.828	0.61	1.11E-184	BAMs	Frmd4b
Mbnl11	1.98E-184	1.742667	0.863	0.701	4.84E-180	BAMs	Mbnl1
Mdfic3	1.14E-181	3.296767	0.388	0.105	2.80E-177	BAMs	Mdfic
St8sia44	1.30E-180	3.234369	0.375	0.098	3.18E-176	BAMs	St8sia4
Gm269172	1.83E-171	1.972647	0.74	0.421	4.48E-167	BAMs	Gm26917
Gab3	8.56E-171	3.594256	0.31	0.071	2.10E-166	BAMs	Gab3
Msr12	2.13E-170	3.83897	0.265	0.053	5.23E-166	BAMs	Msr1
Clcn51	2.32E-170	3.25578	0.393	0.113	5.68E-166	BAMs	Clcn5
Nrp12	8.29E-170	2.555572	0.677	0.379	2.03E-165	BAMs	Nrp1
Camk1d4	1.02E-169	1.512318	0.916	0.807	2.49E-165	BAMs	Camk1d
Mndal4	4.88E-165	3.057946	0.337	0.084	1.20E-160	BAMs	Mndal
Rftn14	6.30E-160	2.973352	0.436	0.146	1.54E-155	BAMs	Rftn1
Mctp14	2.44E-159	3.049162	0.638	0.338	5.98E-155	BAMs	Mctp1
Hip12	1.17E-155	2.990868	0.344	0.093	2.85E-151	BAMs	Hip1
Cp1	4.10E-153	3.2853	0.321	0.082	1.00E-148	BAMs	Cp
Pstpip21	3.17E-152	3.507868	0.283	0.065	7.76E-148	BAMs	Pstpip2
Foxp13	1.42E-149	2.012303	0.722	0.461	3.49E-145	BAMs	Foxp1
Atrnl12	1.73E-147	3.265663	0.335	0.092	4.25E-143	BAMs	Atrnl1
Neat14	3.73E-147	2.514827	0.599	0.295	9.12E-143	BAMs	Neat1
Lrmda1	2.22E-144	1.557691	0.858	0.721	5.43E-140	BAMs	Lrmda
Immp2l1	3.92E-144	2.534428	0.53	0.228	9.60E-140	BAMs	Immp2l
Dock12	1.28E-143	2.435841	0.493	0.197	3.14E-139	BAMs	Dock1
Ptprj2	5.31E-143	1.713415	0.807	0.641	1.30E-138	BAMs	Ptprj

Snx241	1.02E-138	2.184939	0.669	0.402	2.50E-134	BAMs	Snx24
Lrp61	4.64E-138	2.665386	0.489	0.203	1.14E-133	BAMs	Lrp6
Pf41	1.28E-135	1.749395	0.327	0.083	3.14E-131	BAMs	Pf4
Stard81	3.45E-135	2.986995	0.404	0.141	8.44E-131	BAMs	Stard8
Eps82	1.14E-133	3.272186	0.311	0.086	2.78E-129	BAMs	Eps8
Fnbp12	1.61E-133	1.996438	0.658	0.395	3.95E-129	BAMs	Fnbp1
Adcy9	3.63E-133	2.612164	0.373	0.118	8.90E-129	BAMs	Adcy9
Ifi2033	2.46E-130	2.986826	0.29	0.075	6.03E-126	BAMs	Ifi203
Tbc1d51	2.99E-128	1.728117	0.722	0.495	7.33E-124	BAMs	Tbc1d5
Zbtb201	1.02E-126	1.699156	0.735	0.513	2.50E-122	BAMs	Zbtb20
Slc8a11	4.32E-126	1.521238	0.832	0.707	1.06E-121	BAMs	Slc8a1
Atxn7l11	8.05E-125	2.180366	0.55	0.273	1.97E-120	BAMs	Atxn7l1
Spred11	2.13E-124	2.473834	0.453	0.184	5.21E-120	BAMs	Spred1
Fosb2	2.35E-124	2.67784	0.427	0.161	5.76E-120	BAMs	Fosb
Rreb11	1.28E-122	1.635575	0.747	0.556	3.14E-118	BAMs	Rreb1
Dclre1c1	3.19E-122	2.527836	0.484	0.217	7.81E-118	BAMs	Dclre1c
Sbf22	1.52E-120	2.18202	0.598	0.347	3.73E-116	BAMs	Sbf2
Atp2b14	2.89E-120	1.837304	0.677	0.456	7.07E-116	BAMs	Atp2b1
Gab21	8.44E-119	1.370431	0.821	0.69	2.07E-114	BAMs	Gab2
Tmcc13	3.48E-118	1.985829	0.619	0.356	8.53E-114	BAMs	Tmcc1
Enox21	8.29E-115	2.57065	0.413	0.161	2.03E-110	BAMs	Enox2
Tbc1d41	8.77E-114	2.999588	0.33	0.107	2.15E-109	BAMs	Tbc1d4
Igf12	3.48E-113	2.379011	0.355	0.116	8.52E-109	BAMs	Igf1
Dock22	1.86E-112	1.340542	0.801	0.691	4.56E-108	BAMs	Dock2
Nfkbiz5	5.32E-112	2.30632	0.518	0.254	1.30E-107	BAMs	Nfkbiz
Smyd31	8.70E-112	2.124064	0.553	0.29	2.13E-107	BAMs	Smyd3
Zfp704	1.81E-111	2.703081	0.343	0.116	4.44E-107	BAMs	Zfp704
Arhgap182	1.54E-109	2.676966	0.382	0.145	3.77E-105	BAMs	Arhgap18
Ms4a4a3	1.66E-109	2.609726	0.284	0.08	4.07E-105	BAMs	Ms4a4a
Klra22	4.49E-109	2.874992	0.271	0.075	1.10E-104	BAMs	Klra2
Man2a12	3.82E-108	2.684924	0.381	0.145	9.37E-104	BAMs	Man2a1
Pip4k2a2	5.36E-107	1.366429	0.789	0.654	1.31E-102	BAMs	Pip4k2a
Ttr5	1.33E-106	1.222071	0.788	0.507	3.26E-102	BAMs	Ttr
Runx11	1.78E-106	1.590924	0.744	0.591	4.35E-102	BAMs	Runx1
Rbm471	2.07E-106	1.97722	0.62	0.396	5.08E-102	BAMs	Rbm47
Diaph21	2.46E-106	1.549342	0.723	0.55	6.02E-102	BAMs	Diaph2
Maml21	3.95E-106	1.836328	0.682	0.481	9.67E-102	BAMs	Maml2
Itga44	4.13E-106	2.210281	0.336	0.111	1.01E-101	BAMs	Itga4
Nfxl11	9.47E-106	2.777733	0.356	0.129	2.32E-101	BAMs	Nfxl1
Map3k53	1.37E-105	2.090983	0.516	0.26	3.36E-101	BAMs	Map3k5
Snx291	6.06E-104	1.557122	0.716	0.523	1.48E-99	BAMs	Snx29
Prkn1	6.93E-104	2.08958	0.528	0.274	1.70E-99	BAMs	Prkn
Ppm1h1	1.17E-103	1.608675	0.693	0.501	2.87E-99	BAMs	Ppm1h
Lifr1	1.11E-102	2.314929	0.451	0.202	2.71E-98	BAMs	Lifr

Ssh21	1.74E-102	1.301376	0.831	0.723	4.27E-98	BAMs	Ssh2
Wdfy31	5.34E-101	1.7749	0.641	0.441	1.31E-96	BAMs	Wdfy3
Exoc6b1	9.56E-100	2.286133	0.467	0.223	2.34E-95	BAMs	Exoc6b
Ppp3ca2	3.53E-98	1.536654	0.705	0.547	8.64E-94	BAMs	Ppp3ca
Fam172a1	2.81E-97	1.710493	0.624	0.403	6.88E-93	BAMs	Fam172a
Apobec13	2.97E-96	2.113622	0.596	0.378	7.27E-92	BAMs	Apobec1
Mitf1	1.79E-95	1.705377	0.647	0.449	4.38E-91	BAMs	Mitf
Anks11	8.30E-94	2.062166	0.486	0.249	2.03E-89	BAMs	Anks1
Tbxas11	2.08E-93	1.539351	0.727	0.594	5.08E-89	BAMs	Tbxas1
Ehd42	2.37E-93	1.921783	0.611	0.411	5.80E-89	BAMs	Ehd4
Lilrb4a5	2.63E-93	2.063192	0.404	0.167	6.44E-89	BAMs	Lilrb4a
Exoc41	4.75E-93	1.645466	0.64	0.444	1.16E-88	BAMs	Exoc4
Rnf1691	4.38E-92	1.840635	0.564	0.34	1.07E-87	BAMs	Rnf169
Ophn11	5.93E-92	1.282735	0.749	0.596	1.45E-87	BAMs	Ophn1
Cmip3	3.26E-91	1.70569	0.6	0.381	7.99E-87	BAMs	Cmip
Peak11	1.33E-90	2.43985	0.367	0.149	3.26E-86	BAMs	Peak1
Cybb6	1.55E-88	1.56437	0.496	0.233	3.81E-84	BAMs	Cybb
Ap2a21	2.15E-88	2.469857	0.368	0.154	5.26E-84	BAMs	Ap2a2
Wdfy21	3.14E-88	1.970913	0.524	0.301	7.69E-84	BAMs	Wdfy2
Rapgef21	1.17E-87	2.271365	0.433	0.207	2.85E-83	BAMs	Rapgef2
Dleu22	6.92E-87	1.226597	0.753	0.621	1.70E-82	BAMs	Dleu2
Ankrd111	5.04E-85	1.541594	0.659	0.498	1.23E-80	BAMs	Ankrd11
Etv63	7.02E-85	1.440572	0.716	0.586	1.72E-80	BAMs	Etv6
Pbx3	3.75E-84	2.445362	0.353	0.144	9.19E-80	BAMs	Pbx3
Stk17b4	2.54E-83	2.342761	0.385	0.17	6.21E-79	BAMs	Stk17b
Fli11	4.81E-83	1.370522	0.707	0.572	1.18E-78	BAMs	Fli1
Ctsc4	5.20E-82	1.276293	0.796	0.706	1.27E-77	BAMs	Ctsc
Elmo11	6.07E-82	0.929744	0.831	0.761	1.49E-77	BAMs	Elmo1
Vps13b2	1.22E-81	1.920968	0.503	0.289	2.98E-77	BAMs	Vps13b
Uvrag3	5.19E-81	1.657865	0.617	0.445	1.27E-76	BAMs	Uvrag
Dmxl12	6.39E-81	2.090231	0.449	0.233	1.57E-76	BAMs	Dmxl1
Nfatc21	1.69E-80	2.20849	0.414	0.199	4.13E-76	BAMs	Nfatc2
Osbpl92	1.78E-79	2.276901	0.538	0.356	4.36E-75	BAMs	Osbpl9
Snx81	3.00E-79	2.653519	0.328	0.133	7.34E-75	BAMs	Snx8
Ptgds4	3.01E-79	1.450538	0.501	0.252	7.38E-75	BAMs	Ptgds
Prkcb2	7.94E-79	1.485836	0.665	0.517	1.95E-74	BAMs	Prkcb
Dock81	3.09E-78	1.223858	0.746	0.635	7.57E-74	BAMs	Dock8
Parp82	3.32E-78	1.928867	0.509	0.298	8.14E-74	BAMs	Parp8
Eps152	1.61E-77	2.20645	0.441	0.235	3.95E-73	BAMs	Eps15
Rbms15	1.17E-76	2.044051	0.439	0.228	2.86E-72	BAMs	Rbms1
Tcf41	6.06E-76	1.474609	0.632	0.47	1.48E-71	BAMs	Tcf4
Arid1b1	7.03E-76	1.617119	0.562	0.367	1.72E-71	BAMs	Arid1b
Mef2a1	1.02E-75	1.059898	0.805	0.738	2.50E-71	BAMs	Mef2a
Zdhhc141	1.26E-75	2.063752	0.498	0.296	3.09E-71	BAMs	Zdhhc14

Rgl12	3.36E-75	2.336427	0.462	0.261	8.22E-71	BAMs	Rgl1
Nsmce21	3.60E-75	1.941163	0.486	0.281	8.80E-71	BAMs	Nsmce2
Tet31	3.74E-74	1.872456	0.477	0.268	9.17E-70	BAMs	Tet3
Slc38a61	5.26E-74	2.365277	0.361	0.164	1.29E-69	BAMs	Slc38a6
Prune2	6.06E-74	2.571466	0.279	0.103	1.48E-69	BAMs	Prune2
Lncpint1	7.04E-74	1.722378	0.538	0.343	1.72E-69	BAMs	Lncpint
Vps13c2	1.18E-73	2.114977	0.409	0.209	2.90E-69	BAMs	Vps13c
Fgd42	1.39E-73	2.173745	0.396	0.193	3.41E-69	BAMs	Fgd4
Tcf121	1.52E-73	1.809825	0.499	0.295	3.71E-69	BAMs	Tcf12
Ncoa21	4.12E-73	1.888378	0.492	0.286	1.01E-68	BAMs	Ncoa2
Dennd1b2	2.23E-72	2.147101	0.432	0.232	5.46E-68	BAMs	Dennd1b
Fndc3b1	2.70E-72	1.935889	0.459	0.255	6.62E-68	BAMs	Fndc3b
Slc16a7	1.23E-71	2.330213	0.324	0.135	3.00E-67	BAMs	Slc16a7
Rab8b1	2.09E-71	1.667233	0.563	0.381	5.12E-67	BAMs	Rab8b
Phactr22	3.07E-71	2.094354	0.436	0.23	7.53E-67	BAMs	Phactr2
Fyb2	2.33E-70	1.029978	0.8	0.727	5.70E-66	BAMs	Fyb
Akap101	3.46E-70	1.963461	0.43	0.231	8.47E-66	BAMs	Akap10
Adgre11	3.59E-69	1.522332	0.646	0.529	8.79E-65	BAMs	Adgre1
Prkch1	6.99E-69	1.932706	0.419	0.215	1.71E-64	BAMs	Prkch
Maf4	1.29E-68	1.481075	0.622	0.462	3.17E-64	BAMs	Maf
Ddx602	1.66E-68	2.563944	0.28	0.109	4.06E-64	BAMs	Ddx60
Gm49513	2.40E-67	2.05867	0.446	0.25	5.88E-63	BAMs	Gm4951
Skap22	5.89E-67	1.31678	0.658	0.565	1.44E-62	BAMs	Skap2
Pdlim52	7.92E-67	2.039225	0.394	0.2	1.94E-62	BAMs	Pdlim5
Notch22	8.78E-66	1.682684	0.51	0.326	2.15E-61	BAMs	Notch2
Jmjd1c1	9.12E-66	1.266655	0.664	0.54	2.23E-61	BAMs	Jmjd1c
Fbxl171	2.02E-65	1.636805	0.554	0.383	4.94E-61	BAMs	Fbxl17
Atxn11	9.55E-65	2.023461	0.413	0.221	2.34E-60	BAMs	Atxn1
Tmem1643	2.47E-64	1.844397	0.473	0.284	6.04E-60	BAMs	Tmem164
Fmnl23	5.05E-63	1.981599	0.484	0.301	1.24E-58	BAMs	Fmnl2
Kif16b	1.08E-62	2.311606	0.271	0.106	2.66E-58	BAMs	Kif16b
Zcchc71	3.81E-62	1.603336	0.504	0.324	9.32E-58	BAMs	Zcchc7
Bank13	9.82E-62	2.052109	0.385	0.195	2.41E-57	BAMs	Bank1
Fcho22	2.04E-61	1.558146	0.548	0.389	5.00E-57	BAMs	Fcho2
Btbd91	2.36E-60	1.520409	0.564	0.403	5.77E-56	BAMs	Btbd9
Inpp4a	2.45E-60	2.289991	0.308	0.137	6.00E-56	BAMs	Inpp4a
Tnfrsf11a2	5.58E-60	1.685579	0.537	0.377	1.37E-55	BAMs	Tnfrsf11a
Pik3ap12	1.29E-59	1.402752	0.634	0.525	3.16E-55	BAMs	Pik3ap1
Wwox1	1.48E-59	1.866292	0.504	0.331	3.62E-55	BAMs	Wwox
Celf21	1.56E-59	1.193077	0.686	0.606	3.81E-55	BAMs	Celf2
Picalm1	1.61E-59	1.221888	0.697	0.627	3.94E-55	BAMs	Picalm
Klf64	1.97E-59	1.798517	0.528	0.371	4.83E-55	BAMs	Klf6
mt-Co13	2.10E-59	0.807093	0.962	0.963	5.14E-55	BAMs	mt-Co1
Susd61	5.80E-59	1.836833	0.473	0.302	1.42E-54	BAMs	Susd6

Snd11	1.39E-58	1.709857	0.508	0.346	3.40E-54	BAMs	Snd1
Creb51	1.68E-58	2.875949	0.262	0.107	4.11E-54	BAMs	Creb5
Mef2c2	5.61E-58	0.96484	0.782	0.726	1.37E-53	BAMs	Mef2c
Arid5b3	1.93E-57	1.895461	0.497	0.331	4.73E-53	BAMs	Arid5b
Gm424184	2.43E-57	0.773707	0.988	0.982	5.96E-53	BAMs	Gm42418
Osbpl82	1.90E-55	1.909431	0.426	0.254	4.66E-51	BAMs	Osbpl8
Atxn71	1.53E-54	1.762512	0.401	0.224	3.75E-50	BAMs	Atxn7
Stk31	2.23E-54	1.943594	0.373	0.201	5.47E-50	BAMs	Stk3
Cd363	2.71E-54	2.712205	0.273	0.118	6.65E-50	BAMs	Cd36
Tmcc31	4.05E-54	1.236919	0.728	0.672	9.92E-50	BAMs	Tmcc3
Slc16a10	4.43E-54	2.378098	0.293	0.134	1.09E-49	BAMs	Slc16a10
Hivep22	6.31E-53	2.310307	0.271	0.117	1.55E-48	BAMs	Hivep2
Srgap21	9.35E-53	0.809313	0.815	0.771	2.29E-48	BAMs	Srgap2
Mgat51	1.00E-51	1.418033	0.525	0.368	2.45E-47	BAMs	Mgat5
Enpp24	1.71E-51	1.308419	0.424	0.228	4.20E-47	BAMs	Enpp2
Ano6	4.64E-51	1.921164	0.35	0.185	1.14E-46	BAMs	Ano6
Ncoa11	5.21E-51	1.729197	0.404	0.236	1.27E-46	BAMs	Ncoa1
App2	7.05E-51	1.353384	0.63	0.539	1.73E-46	BAMs	App
Pan31	1.84E-50	1.377152	0.547	0.409	4.50E-46	BAMs	Pan3
Fndc3a3	2.06E-50	1.664072	0.459	0.302	5.06E-46	BAMs	Fndc3a
Map4k31	2.81E-50	1.897994	0.38	0.214	6.88E-46	BAMs	Map4k3
Snx301	1.33E-49	1.893516	0.402	0.242	3.25E-45	BAMs	Snx30
Kansl11	3.71E-49	1.216113	0.607	0.498	9.09E-45	BAMs	Kansl1
Tnks1	3.74E-49	1.775969	0.355	0.19	9.16E-45	BAMs	Tnks
Fnip11	8.27E-49	1.638536	0.476	0.324	2.03E-44	BAMs	Fnip1
Gm267403	1.14E-48	1.625905	0.46	0.306	2.79E-44	BAMs	Gm26740
Lcorl1	1.85E-48	1.702585	0.388	0.222	4.53E-44	BAMs	Lcorl
mt-Atp63	2.08E-48	0.894469	0.927	0.914	5.09E-44	BAMs	mt-Atp6
Rap1gds11	2.15E-48	1.016099	0.672	0.595	5.27E-44	BAMs	Rap1gds1
Abr2	2.38E-48	1.686789	0.463	0.313	5.83E-44	BAMs	Abr
P2rx72	4.67E-48	1.978914	0.507	0.375	1.14E-43	BAMs	P2rx7
Zfand31	2.08E-47	1.295269	0.556	0.434	5.09E-43	BAMs	Zfand3
Mbnl21	2.99E-47	1.352904	0.562	0.441	7.33E-43	BAMs	Mbnl2
Stag11	6.07E-47	1.369751	0.499	0.348	1.49E-42	BAMs	Stag1
Etv11	8.38E-47	2.253203	0.252	0.111	2.05E-42	BAMs	Etv1
Dyrk1a1	1.60E-46	1.747564	0.37	0.21	3.92E-42	BAMs	Dyrk1a
Fggy	3.62E-46	2.200988	0.259	0.117	8.86E-42	BAMs	Fggy
Foxo31	3.79E-46	1.68916	0.399	0.24	9.28E-42	BAMs	Foxo3
Fos4	5.06E-46	0.857	0.679	0.479	1.24E-41	BAMs	Fos
Fto1	7.75E-46	1.587615	0.411	0.252	1.90E-41	BAMs	Fto
Tgfbi5	1.35E-45	1.307788	0.376	0.202	3.31E-41	BAMs	Tgfbi
Fer	1.85E-45	1.885469	0.309	0.155	4.54E-41	BAMs	Fer
Specc1l	2.23E-45	2.114434	0.284	0.138	5.46E-41	BAMs	Specc1l
Lyn1	2.71E-45	0.794739	0.806	0.814	6.65E-41	BAMs	Lyn

Zfp7101	4.30E-45	1.233825	0.588	0.475	1.05E-40	BAMs	Zfp710
Rabep11	4.50E-45	1.751498	0.407	0.255	1.10E-40	BAMs	Rabep1
Mtmr32	1.06E-44	1.740254	0.404	0.252	2.59E-40	BAMs	Mtmr3
Man1c11	2.37E-44	1.371633	0.551	0.436	5.81E-40	BAMs	Man1c1
Dock7	4.75E-44	1.854186	0.331	0.18	1.16E-39	BAMs	Dock7
Rasgef1b2	4.79E-44	1.976287	0.367	0.213	1.17E-39	BAMs	Rasgef1b
mt-Co32	6.00E-44	0.808729	0.95	0.941	1.47E-39	BAMs	mt-Co3
Vti1a2	1.31E-43	1.417425	0.489	0.352	3.20E-39	BAMs	Vti1a
Ankrd442	1.24E-42	1.02723	0.634	0.535	3.04E-38	BAMs	Ankrd44
Nrros2	1.38E-42	1.238532	0.606	0.529	3.39E-38	BAMs	Nrros
Utrn2	1.46E-42	1.840208	0.269	0.127	3.58E-38	BAMs	Utrn
Cdk143	1.59E-42	1.671251	0.392	0.24	3.89E-38	BAMs	Cdk14
Cdkal11	1.76E-42	1.683422	0.366	0.213	4.30E-38	BAMs	Cdkal1
Baz2b3	1.84E-42	1.455834	0.507	0.385	4.50E-38	BAMs	Baz2b
Rsrc11	2.25E-42	1.636902	0.412	0.268	5.51E-38	BAMs	Rsrc1
Zfp36l13	2.52E-42	1.173161	0.695	0.633	6.17E-38	BAMs	Zfp36l1
Vrk21	5.02E-42	1.526071	0.437	0.292	1.23E-37	BAMs	Vrk2
Inpp4b1	7.38E-42	1.222976	0.598	0.497	1.81E-37	BAMs	Inpp4b
Iqgap15	8.05E-42	1.578781	0.322	0.168	1.97E-37	BAMs	Iqgap1
Foxn32	1.03E-41	0.805988	0.749	0.724	2.51E-37	BAMs	Foxn3
Fbxo111	2.25E-41	1.627365	0.385	0.235	5.51E-37	BAMs	Fbxo11
Rasal21	3.12E-41	2.077408	0.291	0.15	7.65E-37	BAMs	Rasal2
Ptprc5	3.19E-41	0.999168	0.647	0.576	7.82E-37	BAMs	Ptprc
Fars21	3.84E-41	1.629122	0.382	0.233	9.42E-37	BAMs	Fars2
Fkbp51	7.53E-41	1.799271	0.445	0.311	1.84E-36	BAMs	Fkbp5
Rere1	1.90E-40	1.216417	0.55	0.443	4.66E-36	BAMs	Rere
Map3k11	2.59E-40	1.879225	0.35	0.208	6.34E-36	BAMs	Map3k1
Gapvd12	4.06E-40	1.694376	0.411	0.273	9.95E-36	BAMs	Gapvd1
Mlxip1	5.98E-40	1.726195	0.379	0.236	1.47E-35	BAMs	Mlxip
Cpq3	1.14E-39	1.740057	0.449	0.323	2.78E-35	BAMs	Cpq
Snx22	1.17E-39	1.72355	0.454	0.338	2.87E-35	BAMs	Snx2
Exoc6	1.52E-39	1.75462	0.374	0.232	3.71E-35	BAMs	Exoc6
Ms4a6b4	2.65E-39	1.411568	0.438	0.297	6.50E-35	BAMs	Ms4a6b
B4galt61	3.30E-39	2.034568	0.282	0.146	8.09E-35	BAMs	B4galt6
Rb12	3.35E-39	1.724191	0.362	0.221	8.21E-35	BAMs	Rb1
Med13l1	3.43E-39	1.44512	0.428	0.284	8.39E-35	BAMs	Med13l
Phf21a1	3.81E-39	1.448126	0.483	0.36	9.32E-35	BAMs	Phf21a
Herc43	5.06E-39	1.582903	0.402	0.261	1.24E-34	BAMs	Herc4
Aopep	5.57E-39	1.785378	0.33	0.187	1.36E-34	BAMs	Aopep
Abca11	5.78E-39	1.067562	0.612	0.517	1.42E-34	BAMs	Abca1
Gphn4	1.95E-38	0.82709	0.703	0.641	4.79E-34	BAMs	Gphn
Sik32	2.22E-38	1.752439	0.343	0.202	5.43E-34	BAMs	Sik3
Stard91	3.17E-38	1.447521	0.464	0.338	7.77E-34	BAMs	Stard9
Eif4g32	3.37E-38	1.300997	0.522	0.418	8.25E-34	BAMs	Eif4g3

Ambra11	3.43E-38	1.471275	0.411	0.269	8.40E-34	BAMs	Ambra1
Cyp27a13	4.33E-38	2.037027	0.271	0.138	1.06E-33	BAMs	Cyp27a1
Tnfaip84	4.95E-38	1.694871	0.444	0.326	1.21E-33	BAMs	Tnfaip8
Gng21	6.25E-38	1.502331	0.489	0.379	1.53E-33	BAMs	Gng2
Arhgap171	1.04E-37	1.192109	0.555	0.464	2.54E-33	BAMs	Arhgap17
Sgms13	3.47E-37	1.68843	0.327	0.186	8.49E-33	BAMs	Sgms1
Tbc1d22a1	6.04E-37	1.456737	0.458	0.334	1.48E-32	BAMs	Tbc1d22a
Mllt101	6.05E-37	1.509988	0.414	0.28	1.48E-32	BAMs	Mllt10
Supt3	6.27E-37	1.819677	0.331	0.193	1.54E-32	BAMs	Supt3
Tle4	8.07E-37	1.678861	0.349	0.212	1.98E-32	BAMs	Tle4
Fnip21	9.31E-37	1.727034	0.338	0.199	2.28E-32	BAMs	Fnip2
Arih11	1.55E-36	1.538593	0.424	0.293	3.79E-32	BAMs	Arih1
Nav12	1.73E-36	2.101679	0.27	0.139	4.23E-32	BAMs	Nav1
Trak1	3.80E-36	1.630854	0.337	0.2	9.32E-32	BAMs	Trak1
Bcas31	4.09E-36	1.601295	0.364	0.227	1.00E-31	BAMs	Bcas3
Cltc1	5.49E-36	1.072944	0.621	0.584	1.34E-31	BAMs	Cltc
Sfmbt11	1.05E-35	1.602351	0.362	0.224	2.57E-31	BAMs	Sfmbt1
Dock113	1.42E-35	1.43985	0.432	0.303	3.48E-31	BAMs	Dock11
Zfp4071	1.83E-35	1.724169	0.33	0.194	4.49E-31	BAMs	Zfp407
Zfhx32	3.46E-35	0.593281	0.785	0.725	8.46E-31	BAMs	Zfhx3
Dnajc131	4.51E-35	1.614125	0.388	0.261	1.10E-30	BAMs	Dnajc13
Wnk11	1.08E-34	0.984326	0.646	0.609	2.64E-30	BAMs	Wnk1
Mycbp21	1.28E-34	1.055511	0.599	0.54	3.14E-30	BAMs	Mycbp2
Epsti13	2.21E-34	1.273742	0.51	0.413	5.40E-30	BAMs	Epsti1
Dock41	2.32E-34	0.678102	0.729	0.696	5.68E-30	BAMs	Dock4
Camk2d2	3.30E-34	1.047169	0.604	0.535	8.08E-30	BAMs	Camk2d
Ankrd171	4.16E-34	1.380163	0.445	0.32	1.02E-29	BAMs	Ankrd17
Herc11	4.17E-34	1.61834	0.38	0.251	1.02E-29	BAMs	Herc1
Sgk32	4.49E-34	1.697582	0.349	0.217	1.10E-29	BAMs	Sgk3
Wipf12	5.67E-34	1.444681	0.479	0.381	1.39E-29	BAMs	Wipf1
Taok33	7.44E-34	1.298678	0.476	0.37	1.82E-29	BAMs	Taok3
Gsk3b1	1.44E-33	1.417795	0.445	0.332	3.54E-29	BAMs	Gsk3b
Lrch11	1.68E-33	1.027063	0.611	0.551	4.11E-29	BAMs	Lrch1
Vps541	2.65E-33	1.574286	0.333	0.201	6.49E-29	BAMs	Vps54
Cyth11	4.86E-33	1.943466	0.284	0.159	1.19E-28	BAMs	Cyth1
11-Mar	7.03E-33	1.224877	0.525	0.434	1.72E-28	BAMs	1-Mar
Gtdc11	8.84E-33	1.530206	0.398	0.272	2.16E-28	BAMs	Gtdc1
mt-Nd43	1.38E-32	0.804318	0.753	0.743	3.39E-28	BAMs	mt-Nd4
Abi12	1.52E-32	1.356238	0.476	0.378	3.73E-28	BAMs	Abi1
Relch	1.57E-32	1.686107	0.329	0.202	3.85E-28	BAMs	Relch
Gm50862	2.00E-32	1.444244	0.38	0.251	4.89E-28	BAMs	Gm5086
Pias11	2.74E-32	1.305732	0.486	0.393	6.71E-28	BAMs	Pias1
Cd862	3.42E-32	1.195199	0.62	0.589	8.38E-28	BAMs	Cd86
Nlrc52	4.83E-32	1.664805	0.363	0.241	1.18E-27	BAMs	Nlrc5

Mir99ahg1	5.97E-32	0.992799	0.604	0.546	1.46E-27	BAMs	Mir99ahg
Arhgef121	6.41E-32	1.629259	0.289	0.162	1.57E-27	BAMs	Arhgef12
mt-Cytb2	6.68E-32	0.714387	0.941	0.951	1.64E-27	BAMs	mt-Cytb
Atp8a11	1.22E-31	0.975761	0.58	0.506	2.98E-27	BAMs	Atp8a1
Apc1	1.25E-31	1.28157	0.456	0.341	3.07E-27	BAMs	Apc
Ccnd33	1.51E-31	1.10844	0.593	0.524	3.70E-27	BAMs	Ccnd3
Atf32	1.90E-31	1.557255	0.433	0.311	4.65E-27	BAMs	Atf3
Atf62	3.49E-31	1.529725	0.382	0.263	8.55E-27	BAMs	Atf6
Psme41	5.49E-31	1.53113	0.344	0.22	1.35E-26	BAMs	Psme4
Wdfy42	3.06E-30	1.276661	0.414	0.29	7.49E-26	BAMs	Wdfy4
Slc12a62	4.91E-30	1.24122	0.491	0.396	1.20E-25	BAMs	Slc12a6
Kmt2c1	8.25E-30	1.299016	0.425	0.311	2.02E-25	BAMs	Kmt2c
Ifnar12	8.60E-30	1.782782	0.353	0.238	2.11E-25	BAMs	Ifnar1
Mta32	1.15E-29	1.937259	0.282	0.165	2.81E-25	BAMs	Mta3
AW554918	1.47E-29	1.644823	0.296	0.173	3.60E-25	BAMs	AW554918
Lars24	2.39E-29	0.608014	0.821	0.756	5.84E-25	BAMs	Lars2
Nedd4l1	2.46E-29	1.686308	0.328	0.207	6.03E-25	BAMs	Nedd4l
Atp6v0a12	2.54E-29	1.438669	0.447	0.351	6.21E-25	BAMs	Atp6v0a1
Aff12	3.06E-29	1.621482	0.354	0.236	7.49E-25	BAMs	Aff1
Erbin1	3.29E-29	1.379509	0.418	0.306	8.06E-25	BAMs	Erbin
Cog51	5.36E-29	1.522681	0.33	0.208	1.31E-24	BAMs	Cog5
Snx131	5.68E-29	1.370665	0.412	0.301	1.39E-24	BAMs	Snx13
Ttc281	6.47E-29	1.302516	0.449	0.34	1.58E-24	BAMs	Ttc28
Kif13b1	9.33E-29	1.588551	0.342	0.225	2.28E-24	BAMs	Kif13b
Kat2b	1.71E-28	1.675674	0.278	0.161	4.18E-24	BAMs	Kat2b
Elf21	1.95E-28	1.486146	0.418	0.318	4.79E-24	BAMs	Elf2
Trio1	2.51E-28	1.345121	0.408	0.295	6.14E-24	BAMs	Trio
Strn31	4.42E-28	1.295885	0.405	0.291	1.08E-23	BAMs	Strn3
Hivep31	4.93E-28	1.246638	0.507	0.431	1.21E-23	BAMs	Hivep3
St6gal11	6.06E-28	1.587621	0.346	0.229	1.48E-23	BAMs	St6gal1
Mapk141	9.24E-28	1.141268	0.485	0.396	2.26E-23	BAMs	Mapk14
Sh3kbp11	9.97E-28	1.163839	0.521	0.446	2.44E-23	BAMs	Sh3kbp1
Myo9b2	1.64E-27	1.436068	0.408	0.309	4.01E-23	BAMs	Myo9b
Ptbp33	1.73E-27	1.114329	0.537	0.487	4.24E-23	BAMs	Ptbp3
mt-Co23	1.87E-27	0.724484	0.925	0.946	4.57E-23	BAMs	mt-Co2
Rps6ka31	2.23E-27	1.581428	0.343	0.229	5.47E-23	BAMs	Rps6ka3
Smurf22	2.43E-27	1.512066	0.351	0.239	5.94E-23	BAMs	Smurf2
Zfp1481	3.08E-27	1.43734	0.393	0.288	7.55E-23	BAMs	Zfp148
Usp341	3.45E-27	1.384231	0.382	0.271	8.45E-23	BAMs	Usp34
Crebbp1	4.48E-27	1.26529	0.431	0.33	1.10E-22	BAMs	Crebbp
Ahcyl22	1.43E-26	1.650048	0.296	0.181	3.51E-22	BAMs	Ahcyl2
Kcnq1ot1	1.90E-26	1.70293	0.252	0.141	4.66E-22	BAMs	Kcnq1ot1
Fam49a2	2.43E-26	1.783603	0.257	0.148	5.96E-22	BAMs	Fam49a
Tcf7l22	2.75E-26	1.470573	0.341	0.227	6.74E-22	BAMs	Tcf7l2

Ankhd11	2.98E-26	1.449843	0.359	0.252	7.29E-22	BAMs	Ankhd1
Cbl3	1.16E-25	1.329033	0.45	0.371	2.83E-21	BAMs	Cbl
Ppp4r1	1.67E-25	1.561923	0.271	0.16	4.09E-21	BAMs	Ppp4r1
Agps1	2.50E-25	1.283563	0.431	0.336	6.13E-21	BAMs	Agps
Babam21	2.76E-25	1.299435	0.43	0.337	6.75E-21	BAMs	Babam2
Kansl1l1	2.84E-25	1.513531	0.317	0.204	6.97E-21	BAMs	Kansl1l
Inpp5d1	3.06E-25	0.484999	0.787	0.798	7.49E-21	BAMs	Inpp5d
Pde4d1	5.67E-25	1.848318	0.264	0.155	1.39E-20	BAMs	Pde4d
Swap701	9.98E-25	1.721874	0.282	0.176	2.44E-20	BAMs	Swap70
Dip2c1	1.05E-24	1.610982	0.278	0.169	2.56E-20	BAMs	Dip2c
Ubash3b1	1.14E-24	0.952492	0.541	0.477	2.80E-20	BAMs	Ubash3b
Bcl2l1	1.52E-24	1.477688	0.334	0.226	3.72E-20	BAMs	Bcl2l1
Zmiz11	1.76E-24	1.046511	0.538	0.498	4.31E-20	BAMs	Zmiz1
Usp151	2.40E-24	1.409025	0.364	0.262	5.87E-20	BAMs	Usp15
Mbd51	3.07E-24	1.364563	0.36	0.254	7.51E-20	BAMs	Mbd5
Atf7	4.66E-24	1.476341	0.331	0.227	1.14E-19	BAMs	Atf7
Il151	6.16E-24	1.642466	0.292	0.189	1.51E-19	BAMs	Il15
Tns31	7.17E-24	0.888883	0.567	0.531	1.76E-19	BAMs	Tns3
Plek2	8.55E-24	1.249772	0.638	0.658	2.09E-19	BAMs	Plek
Tent2	1.14E-23	1.38112	0.372	0.277	2.78E-19	BAMs	Tent2
Eea11	1.21E-23	1.707606	0.28	0.177	2.97E-19	BAMs	Eea1
Ncoa31	2.08E-23	1.372814	0.415	0.333	5.10E-19	BAMs	Ncoa3
Ankrd122	2.22E-23	1.48317	0.35	0.252	5.44E-19	BAMs	Ankrd12
Atg101	2.56E-23	1.524446	0.318	0.215	6.27E-19	BAMs	Atg10
Tsc22d36	3.10E-23	1.311115	0.4	0.3	7.60E-19	BAMs	Tsc22d3
Cdc42se24	4.97E-23	1.459029	0.367	0.275	1.22E-18	BAMs	Cdc42se2
Tm6sf13	5.54E-23	1.232977	0.451	0.369	1.36E-18	BAMs	Tm6sf1
Insr1	6.01E-23	1.333357	0.297	0.19	1.47E-18	BAMs	Insr
Atp7a	6.64E-23	1.59199	0.29	0.188	1.63E-18	BAMs	Atp7a
Phip1	7.17E-23	1.470535	0.359	0.262	1.76E-18	BAMs	Phip
Slc43a21	1.21E-22	1.641107	0.366	0.277	2.96E-18	BAMs	Slc43a2
Ms4a6c4	1.35E-22	0.843947	0.408	0.283	3.30E-18	BAMs	Ms4a6c
Galnt10	1.70E-22	1.593105	0.257	0.156	4.16E-18	BAMs	Galnt10
Lpp1	2.10E-22	1.446199	0.379	0.288	5.13E-18	BAMs	Lpp
Hmbox1	2.30E-22	1.54205	0.276	0.174	5.62E-18	BAMs	Hmbox1
Nt5c21	2.83E-22	1.514578	0.309	0.211	6.94E-18	BAMs	Nt5c2
Ankfy1	2.92E-22	1.493867	0.337	0.239	7.15E-18	BAMs	Ankfy1
Msi21	4.26E-22	1.350312	0.335	0.232	1.04E-17	BAMs	Msi2
Aftph1	5.38E-22	1.198767	0.443	0.366	1.32E-17	BAMs	Aftph
Itga91	6.17E-22	1.231034	0.393	0.29	1.51E-17	BAMs	Itga9
Nsd33	6.41E-22	1.180971	0.408	0.323	1.57E-17	BAMs	Nsd3
Lcor1	7.93E-22	1.344048	0.337	0.237	1.94E-17	BAMs	Lcor
Ifi2043	1.17E-21	1.62386	0.275	0.177	2.87E-17	BAMs	Ifi204
Svil2	1.18E-21	1.443584	0.307	0.205	2.89E-17	BAMs	Svil

Dusp17	1.36E-21	0.908074	0.498	0.385	3.33E-17	BAMs	Dusp1
Arrb1	2.31E-21	1.630269	0.254	0.157	5.66E-17	BAMs	Arrb1
mt-Nd22	3.51E-21	0.64991	0.77	0.804	8.60E-17	BAMs	mt-Nd2
mt-Nd52	4.12E-21	0.908719	0.617	0.61	1.01E-16	BAMs	mt-Nd5
Ctnnd11	4.63E-21	1.600969	0.291	0.196	1.13E-16	BAMs	Ctnnd1
R3hdm1	7.32E-21	1.452456	0.283	0.183	1.79E-16	BAMs	R3hdm1
Rbm61	7.97E-21	1.328129	0.356	0.267	1.95E-16	BAMs	Rbm6
Pla2g75	1.11E-20	1.147153	0.267	0.161	2.72E-16	BAMs	Pla2g7
Trip121	1.62E-20	1.324186	0.381	0.298	3.96E-16	BAMs	Trip12
Abl2	1.84E-20	1.539902	0.298	0.203	4.50E-16	BAMs	Abl2
Coro7	2.02E-20	1.65227	0.292	0.201	4.93E-16	BAMs	Coro7
Ogt2	2.12E-20	1.357624	0.351	0.26	5.20E-16	BAMs	Ogt
Itsn21	2.55E-20	1.204837	0.4	0.322	6.24E-16	BAMs	Itsn2
Rasa11	3.03E-20	1.556955	0.307	0.214	7.42E-16	BAMs	Rasa1
Tnrc6a1	1.34E-19	1.292595	0.304	0.208	3.27E-15	BAMs	Tnrc6a
Dis3l21	2.43E-19	1.431411	0.286	0.192	5.96E-15	BAMs	Dis3l2
Nr2c2	3.24E-19	1.501317	0.254	0.162	7.93E-15	BAMs	Nr2c2
Nrf1	4.84E-19	1.525464	0.265	0.172	1.18E-14	BAMs	Nrf1
Kdm6a	5.35E-19	1.676508	0.256	0.164	1.31E-14	BAMs	Kdm6a
2610203C2	5.76E-19	1.406504	0.305	0.212	1.41E-14	BAMs	2610203C22Rik
Klf7	6.16E-19	1.595611	0.265	0.173	1.51E-14	BAMs	Klf7
Dlg1	6.90E-19	1.434473	0.277	0.184	1.69E-14	BAMs	Dlg1
Tgfbr22	7.57E-19	0.617246	0.662	0.648	1.85E-14	BAMs	Tgfbr2
Smg11	7.96E-19	1.106616	0.396	0.312	1.95E-14	BAMs	Smg1
Ash1l1	8.31E-19	1.144888	0.401	0.324	2.03E-14	BAMs	Ash1l
Arhgap251	1.27E-18	1.188095	0.388	0.313	3.11E-14	BAMs	Arhgap25
Rcsd12	1.46E-18	1.146748	0.439	0.377	3.58E-14	BAMs	Rcsd1
St3gal31	1.77E-18	1.425497	0.297	0.206	4.33E-14	BAMs	St3gal3
Ap1b11	1.87E-18	1.499367	0.328	0.246	4.57E-14	BAMs	Ap1b1
Ptpra2	2.27E-18	0.887772	0.524	0.495	5.57E-14	BAMs	Ptpra
Tmem131l	2.35E-18	1.358052	0.297	0.206	5.75E-14	BAMs	Tmem131l
Abca92	2.47E-18	0.834646	0.523	0.473	6.05E-14	BAMs	Abca9
Ppp6r31	2.49E-18	1.321189	0.325	0.238	6.09E-14	BAMs	Ppp6r3
Fam13b	2.82E-18	1.457433	0.278	0.189	6.90E-14	BAMs	Fam13b
Kidins220	3.22E-18	1.503439	0.295	0.207	7.89E-14	BAMs	Kidins220
Igf1r2	3.27E-18	1.318645	0.317	0.224	8.02E-14	BAMs	Igf1r
Luc7l21	3.75E-18	0.807763	0.578	0.578	9.19E-14	BAMs	Luc7l2
Chd21	4.07E-18	1.30763	0.351	0.269	9.98E-14	BAMs	Chd2
Sesn13	4.94E-18	1.199901	0.382	0.303	1.21E-13	BAMs	Sesn1
Arhgap311	4.97E-18	1.293324	0.387	0.311	1.22E-13	BAMs	Arhgap31
Fbxw111	5.84E-18	1.357956	0.329	0.245	1.43E-13	BAMs	Fbxw11
Kcnk131	7.12E-18	1.166705	0.394	0.321	1.74E-13	BAMs	Kcnk13
Ago3	9.36E-18	1.387212	0.302	0.216	2.29E-13	BAMs	Ago3
Bmp2k1	1.10E-17	0.631854	0.638	0.631	2.70E-13	BAMs	Bmp2k

Tnrc181	1.24E-17	1.295781	0.36	0.284	3.03E-13	BAMs	Tnrc18
Ascc32	1.28E-17	1.320901	0.315	0.228	3.13E-13	BAMs	Ascc3
Ddi21	1.79E-17	1.380956	0.336	0.253	4.39E-13	BAMs	Ddi2
Sp1005	2.01E-17	1.324962	0.355	0.278	4.93E-13	BAMs	Sp100
Gas61	2.38E-17	0.960891	0.44	0.364	5.82E-13	BAMs	Gas6
Clasp21	2.92E-17	0.970257	0.486	0.444	7.15E-13	BAMs	Clasp2
Cers64	2.97E-17	1.500592	0.293	0.208	7.27E-13	BAMs	Cers6
Ggta1	3.01E-17	1.481705	0.289	0.203	7.38E-13	BAMs	Ggta1
Vps13d	3.03E-17	1.357568	0.29	0.203	7.42E-13	BAMs	Vps13d
Epb41	4.66E-17	1.380736	0.295	0.209	1.14E-12	BAMs	Epb41
Smg61	4.75E-17	1.262266	0.338	0.255	1.16E-12	BAMs	Smg6
Arhgap122	5.60E-17	1.17221	0.348	0.267	1.37E-12	BAMs	Arhgap12
Cnot41	8.23E-17	1.177869	0.38	0.308	2.01E-12	BAMs	Cnot4
Tbl1xr11	1.01E-16	1.096241	0.383	0.312	2.47E-12	BAMs	Tbl1xr1
Phf201	1.17E-16	1.414177	0.302	0.221	2.87E-12	BAMs	Phf20
Fam49b2	1.25E-16	0.575502	0.701	0.747	3.05E-12	BAMs	Fam49b
Atp9b1	1.27E-16	1.176512	0.356	0.28	3.11E-12	BAMs	Atp9b
Gna121	1.41E-16	0.884442	0.544	0.525	3.44E-12	BAMs	Gna12
Sh3pxd2a1	1.44E-16	1.148793	0.343	0.262	3.53E-12	BAMs	Sh3pxd2a
Clic44	1.48E-16	1.767029	0.293	0.213	3.63E-12	BAMs	Clic4
Ptbp2	1.52E-16	1.331396	0.267	0.182	3.73E-12	BAMs	Ptbp2
Peli12	1.86E-16	1.11301	0.441	0.394	4.55E-12	BAMs	Peli1
Macf11	1.92E-16	0.603539	0.654	0.676	4.70E-12	BAMs	Macf1
Dock103	3.77E-16	0.811208	0.599	0.611	9.23E-12	BAMs	Dock10
Bbx1	4.25E-16	1.480065	0.271	0.19	1.04E-11	BAMs	Bbx
Nsd11	4.40E-16	1.08298	0.436	0.39	1.08E-11	BAMs	Nsd1
2610507B1	4.53E-16	1.652099	0.25	0.17	1.11E-11	BAMs	2610507B11Rik
Nipbl2	5.16E-16	0.976357	0.482	0.45	1.26E-11	BAMs	Nipbl
Tmem87b	5.94E-16	1.445058	0.25	0.168	1.46E-11	BAMs	Tmem87b
Pcca	7.14E-16	1.349253	0.263	0.178	1.75E-11	BAMs	Pcca
Map3k31	7.75E-16	1.10306	0.368	0.294	1.90E-11	BAMs	Map3k3
Zfp6381	9.68E-16	1.167691	0.348	0.272	2.37E-11	BAMs	Zfp638
Ccnl13	1.19E-15	1.079543	0.405	0.347	2.92E-11	BAMs	Ccnl1
Fam193a1	1.20E-15	1.174792	0.331	0.254	2.95E-11	BAMs	Fam193a
Mon21	1.77E-15	1.258715	0.335	0.261	4.33E-11	BAMs	Mon2
Zfand53	2.28E-15	1.382402	0.398	0.345	5.59E-11	BAMs	Zfand5
Dgkd1	2.61E-15	1.065498	0.37	0.3	6.39E-11	BAMs	Dgkd
Mdm41	2.83E-15	1.2251	0.309	0.23	6.92E-11	BAMs	Mdm4
Stx16	2.98E-15	1.223029	0.355	0.288	7.29E-11	BAMs	Stx16
Nf11	3.59E-15	1.075649	0.386	0.322	8.79E-11	BAMs	Nf1
Myo9a1	4.15E-15	1.099434	0.381	0.319	1.02E-10	BAMs	Myo9a
Acap22	4.58E-15	0.824839	0.514	0.491	1.12E-10	BAMs	Acap2
Map2k51	7.04E-15	1.275502	0.286	0.207	1.72E-10	BAMs	Map2k5
Dym	7.14E-15	1.453425	0.257	0.179	1.75E-10	BAMs	Dym

Rnf2132	1.01E-14	1.220701	0.4	0.345	2.46E-10	BAMs	Rnf213
Maml31	1.07E-14	0.637061	0.561	0.536	2.63E-10	BAMs	Maml3
Atad2b2	1.10E-14	1.278595	0.303	0.229	2.69E-10	BAMs	Atad2b
Bmpr21	1.18E-14	1.133824	0.324	0.247	2.88E-10	BAMs	Bmpr2
Phc3	1.27E-14	1.330825	0.265	0.187	3.11E-10	BAMs	Phc3
Tnrc6b2	1.40E-14	0.973041	0.422	0.366	3.42E-10	BAMs	Tnrc6b
Faf1	1.40E-14	1.258081	0.28	0.204	3.44E-10	BAMs	Faf1
Nr3c11	1.41E-14	1.040729	0.425	0.377	3.44E-10	BAMs	Nr3c1
Chd61	1.54E-14	1.184538	0.28	0.201	3.76E-10	BAMs	Chd6
Rbm251	1.69E-14	0.967712	0.436	0.396	4.14E-10	BAMs	Rbm25
Tec1	1.80E-14	1.258467	0.354	0.289	4.40E-10	BAMs	Tec
Itpkb	1.89E-14	1.518791	0.296	0.224	4.63E-10	BAMs	Itpkb
Nfkb12	2.00E-14	0.888627	0.493	0.472	4.91E-10	BAMs	Nfkb1
Arnt	2.11E-14	1.382272	0.253	0.177	5.16E-10	BAMs	Arnt
Rin21	3.47E-14	1.045283	0.441	0.405	8.50E-10	BAMs	Rin2
4932438A1	3.64E-14	1.277354	0.283	0.21	8.91E-10	BAMs	4932438A13Rik
mt-Nd11	7.53E-14	0.638392	0.762	0.795	1.84E-09	BAMs	mt-Nd1
3-Jun	9.97E-14	0.518897	0.679	0.638	2.44E-09	BAMs	Jun
Kat6a1	1.14E-13	1.137589	0.323	0.253	2.80E-09	BAMs	Kat6a
Ythdf32	1.36E-13	1.338807	0.282	0.209	3.33E-09	BAMs	Ythdf3
Ptpro2	1.56E-13	1.104154	0.388	0.332	3.83E-09	BAMs	Ptpro
Tet21	1.64E-13	1.307187	0.277	0.204	4.01E-09	BAMs	Tet2
Stxbp51	2.04E-13	1.210204	0.28	0.208	4.99E-09	BAMs	Stxbp5
Irf22	2.13E-13	0.987607	0.46	0.44	5.23E-09	BAMs	Irf2
Tbc1d141	2.17E-13	1.166867	0.342	0.281	5.31E-09	BAMs	Tbc1d14
Kdm7a4	2.30E-13	1.185201	0.325	0.26	5.63E-09	BAMs	Kdm7a
Osbpl111	3.11E-13	1.111712	0.398	0.349	7.63E-09	BAMs	Osbpl11
Slc23a2	3.79E-13	1.272489	0.298	0.23	9.28E-09	BAMs	Slc23a2
Ttc31	3.81E-13	1.378853	0.262	0.19	9.34E-09	BAMs	Ttc3
Ecpas	5.09E-13	1.24354	0.305	0.237	1.25E-08	BAMs	Ecpas
Setd51	5.21E-13	1.045724	0.38	0.327	1.28E-08	BAMs	Setd5
Tpd523	5.99E-13	1.034231	0.454	0.43	1.47E-08	BAMs	Tpd52
Tpp2	6.90E-13	1.314996	0.307	0.242	1.69E-08	BAMs	Tpp2
Hdac81	7.63E-13	1.38805	0.258	0.186	1.87E-08	BAMs	Hdac8
Ubr31	9.17E-13	1.236861	0.289	0.219	2.25E-08	BAMs	Ubr3
Tnrc6c1	9.61E-13	1.164056	0.323	0.258	2.35E-08	BAMs	Tnrc6c
Ppp1r12a3	1.10E-12	0.890408	0.45	0.422	2.68E-08	BAMs	Ppp1r12a
R3hdm21	1.22E-12	1.238378	0.309	0.246	2.99E-08	BAMs	R3hdm2
BC005537	1.32E-12	1.557235	0.254	0.187	3.22E-08	BAMs	BC005537
Ulk22	1.37E-12	0.937832	0.414	0.372	3.37E-08	BAMs	Ulk2
Nfia1	1.64E-12	0.780791	0.446	0.395	4.02E-08	BAMs	Nfia
Cacna1d1	2.68E-12	1.105046	0.334	0.271	6.57E-08	BAMs	Cacna1d
Birc62	3.00E-12	0.972007	0.389	0.341	7.35E-08	BAMs	Birc6
Cdk131	3.02E-12	1.184386	0.331	0.273	7.40E-08	BAMs	Cdk13

Slc6a61	3.16E-12	1.040195	0.415	0.379	7.74E-08	BAMs	Slc6a6
Rab311	4.58E-12	1.095628	0.382	0.339	1.12E-07	BAMs	Rab31
Synj13	4.88E-12	1.187234	0.35	0.301	1.20E-07	BAMs	Synj1
Snx92	6.33E-12	1.227501	0.322	0.267	1.55E-07	BAMs	Snx9
Chka1	1.20E-11	1.200278	0.267	0.201	2.94E-07	BAMs	Chka
Hipk21	1.55E-11	1.308511	0.338	0.289	3.80E-07	BAMs	Hipk2
Huwe11	2.01E-11	1.149822	0.322	0.265	4.92E-07	BAMs	Huwe1
Ddx3x1	2.12E-11	1.17039	0.347	0.296	5.20E-07	BAMs	Ddx3x
Gls1	2.16E-11	1.196643	0.323	0.267	5.28E-07	BAMs	Gls
Nisch2	3.03E-11	1.049672	0.391	0.357	7.42E-07	BAMs	Nisch
Spop3	3.11E-11	1.042067	0.395	0.362	7.61E-07	BAMs	Spop
Klf25	4.29E-11	0.790706	0.305	0.228	1.05E-06	BAMs	Klf2
Cop13	4.38E-11	1.230563	0.276	0.214	1.07E-06	BAMs	Cop1
Trappc8	5.08E-11	1.342851	0.265	0.203	1.24E-06	BAMs	Trappc8
Atrx1	5.69E-11	1.016834	0.36	0.314	1.39E-06	BAMs	Atrx
Tut4	6.62E-11	1.208395	0.279	0.219	1.62E-06	BAMs	Tut4
Nfat51	7.73E-11	1.163322	0.302	0.246	1.89E-06	BAMs	Nfat5
Mt34	7.73E-11	0.805185	0.295	0.22	1.89E-06	BAMs	Mt3
Adap22	8.00E-11	0.57991	0.572	0.575	1.96E-06	BAMs	Adap2
Man1a1	8.45E-11	1.119646	0.406	0.381	2.07E-06	BAMs	Man1a
Lrrc8d2	8.71E-11	1.231018	0.329	0.281	2.13E-06	BAMs	Lrrc8d
Agmo2	1.24E-10	0.928941	0.412	0.374	3.03E-06	BAMs	Agmo
Stx81	1.47E-10	1.209475	0.327	0.28	3.60E-06	BAMs	Stx8
Chchd31	1.53E-10	1.357833	0.304	0.254	3.74E-06	BAMs	Chchd3
Plcl21	1.57E-10	0.671216	0.51	0.5	3.84E-06	BAMs	Plcl2
Tut71	1.67E-10	1.162901	0.329	0.281	4.08E-06	BAMs	Tut7
Galnt1	1.79E-10	1.07899	0.354	0.314	4.38E-06	BAMs	Galnt1
Celf11	2.56E-10	1.013917	0.355	0.313	6.27E-06	BAMs	Celf1
Tcf201	2.94E-10	1.16806	0.271	0.213	7.20E-06	BAMs	Tcf20
Pacs12	2.98E-10	1.178309	0.272	0.214	7.30E-06	BAMs	Pacs1
B4galt11	4.58E-10	0.857217	0.434	0.418	1.12E-05	BAMs	B4galt1
Tsc22d2	5.14E-10	1.303555	0.259	0.201	1.26E-05	BAMs	Tsc22d2
Mrtfa4	5.16E-10	1.168167	0.29	0.233	1.26E-05	BAMs	Mrtfa
Fbxl201	5.27E-10	1.133168	0.302	0.247	1.29E-05	BAMs	Fbxl20
Rock21	5.53E-10	0.879103	0.414	0.386	1.36E-05	BAMs	Rock2
Fam117b1	5.70E-10	1.090347	0.307	0.251	1.40E-05	BAMs	Fam117b
Snx103	6.65E-10	1.275815	0.303	0.257	1.63E-05	BAMs	Snx10
Zfp2921	7.03E-10	0.969947	0.361	0.318	1.72E-05	BAMs	Zfp292
Ppp1r9a1	7.61E-10	1.00016	0.409	0.38	1.86E-05	BAMs	Ppp1r9a
Pacsin21	8.14E-10	1.153994	0.374	0.347	1.99E-05	BAMs	Pacsin2
Rnf1111	8.27E-10	1.186511	0.282	0.224	2.02E-05	BAMs	Rnf111
Stag22	8.45E-10	1.189843	0.31	0.261	2.07E-05	BAMs	Stag2
Trip11	9.45E-10	1.256676	0.259	0.204	2.31E-05	BAMs	Trip11
Lrrk12	1.03E-09	0.868988	0.412	0.388	2.53E-05	BAMs	Lrrk1

Pcm1	1.10E-09	1.159271	0.28	0.226	2.69E-05	BAMs	Pcm1
Tasor	1.25E-09	1.198101	0.259	0.202	3.06E-05	BAMs	Tasor
Thada1	1.26E-09	1.07046	0.266	0.207	3.08E-05	BAMs	Thada
Ccny1	1.28E-09	1.201904	0.293	0.243	3.14E-05	BAMs	Ccny
Usp241	1.29E-09	1.029669	0.321	0.27	3.16E-05	BAMs	Usp24
Usp47	1.79E-09	1.212389	0.251	0.194	4.38E-05	BAMs	Usp47
Tbl1x1	1.81E-09	1.144083	0.314	0.268	4.43E-05	BAMs	Tbl1x
Ranbp21	1.92E-09	1.191463	0.271	0.218	4.71E-05	BAMs	Ranbp2
Rassf22	1.94E-09	1.0465	0.36	0.321	4.74E-05	BAMs	Rassf2
Scaper	1.95E-09	1.268004	0.25	0.193	4.79E-05	BAMs	Scaper
Syk3	2.17E-09	0.960345	0.383	0.351	5.30E-05	BAMs	Syk
Cep350	2.27E-09	1.151738	0.309	0.263	5.55E-05	BAMs	Cep350
Dgkz2	2.28E-09	1.151768	0.33	0.294	5.58E-05	BAMs	Dgkz
Il31ra4	2.37E-09	0.614927	0.619	0.618	5.80E-05	BAMs	Il31ra
Asap11	2.43E-09	0.644274	0.53	0.553	5.96E-05	BAMs	Asap1
Taok11	2.85E-09	1.024311	0.328	0.282	6.98E-05	BAMs	Taok1
Arhgap221	3.27E-09	1.067689	0.369	0.329	8.01E-05	BAMs	Arhgap22
Ddx171	3.47E-09	0.950728	0.373	0.34	8.50E-05	BAMs	Ddx17
Hpcal16	4.18E-09	1.385332	0.302	0.263	0.000102	BAMs	Hpcal1
Tlr72	6.61E-09	0.994848	0.38	0.354	0.000162	BAMs	Tlr7
Mertk2	6.81E-09	0.320575	0.705	0.725	0.000167	BAMs	Mertk
Vps41	7.81E-09	1.163392	0.282	0.233	0.000191	BAMs	Vps41
Usp9x1	8.02E-09	1.114919	0.308	0.262	0.000196	BAMs	Usp9x
Map3k2	9.40E-09	1.203952	0.257	0.205	0.00023	BAMs	Map3k2
Fchsd22	1.05E-08	0.783996	0.508	0.519	0.000256	BAMs	Fchsd2
Adcy71	1.20E-08	1.102031	0.312	0.274	0.000295	BAMs	Adcy7
Tlk1	1.30E-08	1.246245	0.264	0.216	0.000319	BAMs	Tlk1
Asxl2	1.43E-08	1.129074	0.269	0.217	0.000351	BAMs	Asxl2
Pum21	1.49E-08	1.007483	0.34	0.306	0.000366	BAMs	Pum2
Chd92	1.94E-08	0.369545	0.679	0.736	0.000476	BAMs	Chd9
Larp4b1	2.41E-08	0.990382	0.368	0.346	0.00059	BAMs	Larp4b
Plekho25	2.52E-08	1.395091	0.253	0.208	0.000618	BAMs	Plekho2
Tank2	2.77E-08	1.411725	0.258	0.211	0.000679	BAMs	Tank
Prex11	3.61E-08	0.717372	0.486	0.506	0.000884	BAMs	Prex1
Tln22	3.91E-08	1.354549	0.254	0.206	0.000957	BAMs	Tln2
Nlrp1b2	3.93E-08	1.174017	0.298	0.257	0.000963	BAMs	Nlrp1b
Rapgef62	4.07E-08	0.99846	0.323	0.283	0.000996	BAMs	Rapgef6
Nek71	4.41E-08	0.700854	0.441	0.43	0.001081	BAMs	Nek7
Srrm21	5.07E-08	0.506793	0.589	0.651	0.001243	BAMs	Srrm2
Rnf2161	5.18E-08	0.888213	0.385	0.364	0.001267	BAMs	Rnf216
Mbp5	5.69E-08	0.590416	0.372	0.319	0.001393	BAMs	Mbp
mt-Nd4l2	6.96E-08	0.526138	0.766	0.826	0.001705	BAMs	mt-Nd4l
Ap3b11	8.12E-08	0.885066	0.38	0.365	0.001989	BAMs	Ap3b1
Oxr11	8.36E-08	0.970478	0.334	0.296	0.002048	BAMs	Oxr1

Mkln11	9.63E-08	0.933688	0.367	0.349	0.002358	BAMs	Mkln1
Ppp1r211	1.03E-07	0.934452	0.359	0.336	0.002513	BAMs	Ppp1r21
Lpin22	1.10E-07	0.780143	0.433	0.436	0.002705	BAMs	Lpin2
Wasf22	1.41E-07	0.526341	0.566	0.619	0.00346	BAMs	Wasf2
Mapkapk23	1.63E-07	1.215702	0.348	0.332	0.004	BAMs	Mapkapk2
Trappc91	2.59E-07	1.093721	0.288	0.246	0.00634	BAMs	Trappc9
Rab432	2.59E-07	1.079522	0.342	0.317	0.006352	BAMs	Rab43
Nktr1	2.83E-07	0.978461	0.33	0.301	0.006923	BAMs	Nktr
P2rx44	2.94E-07	1.17309	0.338	0.321	0.007192	BAMs	P2rx4
Dst1	3.23E-07	0.554572	0.57	0.596	0.007902	BAMs	Dst
Zup12	3.66E-07	1.019753	0.316	0.285	0.008956	BAMs	Zup1
Cep1701	3.72E-07	0.952873	0.312	0.278	0.009114	BAMs	Cep170
Aig1	4.69E-07	1.253306	0.311	0.285	0.011478	BAMs	Aig1
Rptor1	4.77E-07	0.617418	0.4	0.375	0.011669	BAMs	Rptor
Crlf31	4.78E-07	0.828186	0.412	0.413	0.011715	BAMs	Crlf3
Ube3a1	4.89E-07	1.018859	0.31	0.28	0.011969	BAMs	Ube3a
Rad51b3	4.97E-07	1.185133	0.445	0.452	0.012164	BAMs	Rad51b
Pik3cg2	5.04E-07	1.031781	0.321	0.293	0.01235	BAMs	Pik3cg
Mapre2	5.17E-07	1.138544	0.317	0.292	0.012657	BAMs	Mapre2
Aff41	5.87E-07	0.992854	0.316	0.284	0.014379	BAMs	Aff4
Grb22	6.73E-07	0.71649	0.488	0.519	0.016483	BAMs	Grb2
Spag93	1.12E-06	0.998617	0.33	0.306	0.027414	BAMs	Spag9
Ccm21	1.16E-06	1.025607	0.296	0.265	0.028471	BAMs	Ccm2
Polr2a2	1.18E-06	0.280772	0.169	0.271	0.028862	BAMs	Polr2a
Eml41	1.22E-06	0.932275	0.355	0.336	0.029915	BAMs	Eml4
Ubac21	1.37E-06	0.932848	0.342	0.32	0.033461	BAMs	Ubac2
Cdk191	1.42E-06	0.96514	0.291	0.253	0.034683	BAMs	Cdk19
Clec2d5	1.46E-06	1.062436	0.299	0.267	0.035637	BAMs	Clec2d
Ncor13	1.88E-06	0.736831	0.411	0.416	0.045965	BAMs	Ncor1
Sik22	1.97E-06	0.91384	0.269	0.226	0.04833	BAMs	Sik2
Clk11	2.13E-06	0.859093	0.364	0.35	0.052094	BAMs	Clk1
AU020206	2.25E-06	0.808574	0.472	0.494	0.055073	BAMs	AU020206
Zfc3h1	2.43E-06	1.016305	0.257	0.218	0.059397	BAMs	Zfc3h1
Scmh11	2.50E-06	1.077325	0.251	0.21	0.061233	BAMs	Scmh1
Kmt2e2	2.79E-06	0.766652	0.401	0.402	0.068256	BAMs	Kmt2e
Zmynd81	2.86E-06	0.946514	0.299	0.269	0.070056	BAMs	Zmynd8
Dnajc11	3.05E-06	1.069496	0.307	0.284	0.074776	BAMs	Dnajc1
Fam168a1	3.30E-06	0.956285	0.286	0.251	0.080754	BAMs	Fam168a
Jak12	3.64E-06	0.797232	0.408	0.413	0.089069	BAMs	Jak1
Wdr332	4.21E-06	0.908817	0.336	0.319	0.103206	BAMs	Wdr33
Dnmt3a1	4.40E-06	0.756781	0.375	0.36	0.107676	BAMs	Dnmt3a
Rock13	4.48E-06	1.036619	0.325	0.31	0.109713	BAMs	Rock1
Ski1	4.90E-06	0.773144	0.393	0.389	0.119969	BAMs	Ski
Ndufs44	5.36E-06	0.250186	0.196	0.304	0.131274	BAMs	Ndufs4

Fkbp151	5.55E-06	0.921713	0.346	0.336	0.136024	BAMs	Fkbp15
Phkb1	6.30E-06	1.021873	0.252	0.216	0.154248	BAMs	Phkb
Fryl	7.74E-06	1.080661	0.251	0.217	0.189553	BAMs	Fryl
Pld11	7.96E-06	0.786375	0.344	0.322	0.194928	BAMs	Pld1
Bcl103	8.23E-06	0.313616	0.17	0.267	0.201488	BAMs	Bcl10
Lrch32	8.71E-06	1.019866	0.282	0.256	0.213397	BAMs	Lrch3
Pds5a1	1.03E-05	0.94058	0.275	0.243	0.253352	BAMs	Pds5a
Cd841	1.06E-05	0.66642	0.51	0.556	0.259194	BAMs	Cd84
Ppig1	1.07E-05	0.270562	0.159	0.25	0.261657	BAMs	Ppig
Gpatch81	1.08E-05	0.95665	0.285	0.256	0.265026	BAMs	Gpatch8
Cask2	1.31E-05	0.786546	0.333	0.311	0.320064	BAMs	Cask
Pxn2	1.43E-05	1.129469	0.253	0.221	0.350684	BAMs	Pxn
Zfand61	1.65E-05	0.978595	0.322	0.306	0.404634	BAMs	Zfand6
Ccdc501	1.79E-05	0.826643	0.378	0.38	0.439439	BAMs	Ccdc50
Kpna42	1.84E-05	0.826026	0.428	0.455	0.450413	BAMs	Kpna4
Pitpna4	1.86E-05	0.683364	0.454	0.485	0.456636	BAMs	Pitpna
Glg1	2.22E-05	1.02929	0.295	0.275	0.544187	BAMs	Glg1
Axl4	2.30E-05	1.020445	0.292	0.269	0.563697	BAMs	Axl
Tbc1d231	2.36E-05	0.944171	0.279	0.252	0.5771	BAMs	Tbc1d23
Rhobtb12	2.40E-05	0.921603	0.275	0.244	0.586907	BAMs	Rhobtb1
Stk42	2.43E-05	0.887003	0.312	0.292	0.59537	BAMs	Stk4
Gsap3	2.47E-05	0.986314	0.31	0.293	0.605963	BAMs	Gsap
Wdr262	3.14E-05	0.869572	0.359	0.354	0.768549	BAMs	Wdr26
Tmod32	4.83E-05	0.32066	0.168	0.255	1	BAMs	Tmod3
Hook3	4.92E-05	0.918314	0.273	0.246	1	BAMs	Hook3
Wdr442	5.74E-05	0.928794	0.262	0.231	1	BAMs	Wdr44
Dek5	6.33E-05	0.272436	0.17	0.257	1	BAMs	Dek
Pvt11	6.94E-05	0.957403	0.278	0.253	1	BAMs	Pvt1
Washc42	8.18E-05	0.842696	0.291	0.27	1	BAMs	Washc4
Ubr51	8.34E-05	0.911651	0.262	0.236	1	BAMs	Ubr5
Jarid25	9.35E-05	0.891505	0.341	0.336	1	BAMs	Jarid2
Cnot21	9.92E-05	0.826798	0.333	0.325	1	BAMs	Cnot2
Eri31	9.95E-05	0.926488	0.267	0.245	1	BAMs	Eri3
Cept12	0.000115	0.925686	0.297	0.28	1	BAMs	Cept1
Creb11	0.000128	0.95389	0.276	0.252	1	BAMs	Creb1
Wwp21	0.000129	0.985262	0.264	0.244	1	BAMs	Wwp2
Parp142	0.000135	0.96773	0.292	0.273	1	BAMs	Parp14
Gm433053	0.000145	1.046851	0.4	0.419	1	BAMs	Gm43305
Cul31	0.000164	1.009161	0.25	0.228	1	BAMs	Cul3
Pik3cd1	0.00017	0.806416	0.379	0.39	1	BAMs	Pik3cd
Ppp2r5c2	0.00017	0.882025	0.328	0.322	1	BAMs	Ppp2r5c
Mindy21	0.000185	0.254598	0.176	0.256	1	BAMs	Mindy2
Dnajb61	0.000215	0.28938	0.227	0.327	1	BAMs	Dnajb6
Mark22	0.000228	0.911613	0.263	0.242	1	BAMs	Mark2

Asxl12	0.000231	0.909361	0.254	0.231	1	BAMs	Asxl1
Brd42	0.000235	0.722371	0.368	0.378	1	BAMs	Brd4
Lmbrd12	0.000257	0.949805	0.28	0.267	1	BAMs	Lmbrd1
Jund2	0.00027	0.283562	0.665	0.69	1	BAMs	Jund
Znfx12	0.000274	0.277226	0.193	0.278	1	BAMs	Znfx1
Ap2b1	0.000281	0.994003	0.26	0.24	1	BAMs	Ap2b1
Vav21	0.000287	0.788035	0.28	0.262	1	BAMs	Vav2
U2surp1	0.000289	0.284833	0.174	0.256	1	BAMs	U2surp
Txnip2	0.00031	0.579028	0.496	0.543	1	BAMs	Txnip
Smarca21	0.000323	0.791821	0.311	0.296	1	BAMs	Smarca2
Il4ra2	0.00033	1.192219	0.256	0.241	1	BAMs	Il4ra
Ranbp9	0.000393	0.990948	0.257	0.238	1	BAMs	Ranbp9
Arhgef6	0.0004	0.972329	0.258	0.238	1	BAMs	Arhgef6
Cux11	0.000406	0.665306	0.389	0.405	1	BAMs	Cux1
Ikzf11	0.000423	0.366383	0.57	0.63	1	BAMs	Ikzf1
Usp252	0.000424	0.9696	0.271	0.258	1	BAMs	Usp25
Tra2b1	0.00049	0.267409	0.272	0.388	1	BAMs	Tra2b
Fhit1	0.000513	0.923801	0.282	0.262	1	BAMs	Fhit
Prcp3	0.000555	0.281757	0.262	0.371	1	BAMs	Prcp
Prpf38b1	0.000558	0.316399	0.185	0.263	1	BAMs	Prpf38b
Arf42	0.000609	0.295512	0.325	0.456	1	BAMs	Arf4
Larp12	0.000639	0.944303	0.311	0.314	1	BAMs	Larp1
Mvb12b1	0.000647	0.714426	0.303	0.289	1	BAMs	Mvb12b
Pitpnc11	0.000652	0.638176	0.405	0.42	1	BAMs	Pitpnc1
Ubl31	0.000791	0.261365	0.302	0.429	1	BAMs	Ubl3
Myo1f3	0.000811	0.43646	0.556	0.63	1	BAMs	Myo1f
Mier11	0.000931	0.83311	0.296	0.288	1	BAMs	Mier1
Tmem1351	0.001131	0.543683	0.376	0.381	1	BAMs	Tmem135
Pnisr2	0.001166	0.745832	0.329	0.329	1	BAMs	Pnisr
Wac1	0.001297	0.72766	0.318	0.317	1	BAMs	Wac
St3gal63	0.001487	0.561453	0.507	0.547	1	BAMs	St3gal6
Apba11	0.001503	0.93138	0.308	0.303	1	BAMs	Apba1
Clint11	0.001613	0.587959	0.439	0.486	1	BAMs	Clint1
Pla2g4a1	0.001632	0.844907	0.262	0.246	1	BAMs	Pla2g4a
Khdrbs12	0.001635	0.289066	0.265	0.373	1	BAMs	Khdrbs1
Rlim2	0.001658	0.323175	0.182	0.255	1	BAMs	Rlim
Sh3gl12	0.001666	0.411224	0.202	0.283	1	BAMs	Sh3gl1
Brd1	0.001682	0.914688	0.26	0.249	1	BAMs	Brd1
Cd833	0.001694	0.664421	0.466	0.484	1	BAMs	Cd83
Blvrb3	0.001823	0.64816	0.318	0.315	1	BAMs	Blvrb
Cmss14	0.001838	0.262161	0.917	0.924	1	BAMs	Cmss1
Gnb14	0.001863	0.59111	0.441	0.492	1	BAMs	Gnb1
Pura2	0.001881	0.264595	0.245	0.339	1	BAMs	Pura
Plekhm21	0.001895	0.931326	0.284	0.278	1	BAMs	Plekhm2

Dync1i21	0.001962	0.312617	0.179	0.25	1	BAMs	Dync1i2
Lcp23	0.002032	0.73653	0.408	0.457	1	BAMs	Lcp2
Eif4a21	0.002055	0.286727	0.254	0.354	1	BAMs	Eif4a2
Kdm2a1	0.002063	0.845697	0.289	0.283	1	BAMs	Kdm2a
Epc1	0.002427	0.927297	0.252	0.24	1	BAMs	Epc1
mt-Nd31	0.002435	0.306261	0.191	0.261	1	BAMs	mt-Nd3
Ube2k3	0.002505	0.852966	0.285	0.283	1	BAMs	Ube2k
Slmap1	0.002679	0.722239	0.284	0.273	1	BAMs	Slmap
Actr34	0.002756	0.455779	0.507	0.56	1	BAMs	Actr3
Agpat31	0.003189	0.328838	0.192	0.265	1	BAMs	Agpat3
Sdccag81	0.003233	0.876637	0.269	0.26	1	BAMs	Sdccag8
Rbm261	0.00344	0.735969	0.304	0.302	1	BAMs	Rbm26
Trim441	0.003456	0.782112	0.258	0.244	1	BAMs	Trim44
Rasa32	0.003503	0.921261	0.275	0.27	1	BAMs	Rasa3
Abl12	0.003522	0.868293	0.267	0.257	1	BAMs	Abl1
Rapgef11	0.003783	0.722091	0.324	0.326	1	BAMs	Rapgef1
Hpgds2	0.003817	0.368781	0.567	0.622	1	BAMs	Hpgds
Snx61	0.00397	0.77742	0.372	0.397	1	BAMs	Snx6
Hnrnpm1	0.004125	0.899073	0.296	0.3	1	BAMs	Hnrnpm
Il6ra2	0.004129	0.494101	0.446	0.486	1	BAMs	Il6ra
Atf7ip3	0.004777	0.866488	0.278	0.277	1	BAMs	Atf7ip
Cables11	0.004964	0.694691	0.257	0.242	1	BAMs	Cables1
Caprin1	0.005	0.369252	0.243	0.336	1	BAMs	Caprin1
Klf134	0.005301	0.805761	0.308	0.314	1	BAMs	Klf13
Btbd11	0.005988	0.989186	0.266	0.264	1	BAMs	Btbd1
Specc11	0.006081	0.537239	0.381	0.403	1	BAMs	Specc1
Zfp6441	0.00613	0.882618	0.28	0.278	1	BAMs	Zfp644
Prrc2c2	0.006194	0.719729	0.346	0.371	1	BAMs	Prrc2c
Sf12	0.006322	0.258784	0.245	0.331	1	BAMs	Sf1
Gpcpd12	0.006367	0.768973	0.26	0.254	1	BAMs	Gpcpd1
Zmynd112	0.00664	0.788824	0.286	0.286	1	BAMs	Zmynd11
Arglu12	0.006806	0.328601	0.27	0.364	1	BAMs	Arglu1
Dnajc72	0.007089	0.258163	0.276	0.371	1	BAMs	Dnajc7
Gpr1833	0.007531	0.850847	0.336	0.346	1	BAMs	Gpr183
Tab22	0.007595	0.696544	0.336	0.347	1	BAMs	Tab2
Elf11	0.007802	0.704413	0.35	0.369	1	BAMs	Elf1
Hps31	0.008116	0.757994	0.301	0.304	1	BAMs	Hps3
Max3	0.008352	0.394473	0.196	0.267	1	BAMs	Max
Snap232	0.008441	0.953213	0.254	0.253	1	BAMs	Snap23
Xist2	0.009107	0.868788	0.355	0.379	1	BAMs	Xist
Ptprm1	0.009547	0.934505	0.25	0.239	1	BAMs	Ptprm
Cttnbp2nl2	0.009606	0.590155	0.45	0.497	1	BAMs	Cttnbp2nl
Ccr7	0	12.00069	0.938	0.002	0	pDC	Ccr7
Traf11	0	7.00519	0.921	0.025	0	pDC	Traf1

Cacnb3	0	8.332469	0.889	0.007	0	pDC	Cacnb3
Cytip3	0	4.982244	0.916	0.079	0	pDC	Cytip
Spint21	0	5.365197	0.874	0.04	0	pDC	Spint2
Tmem1231	0	5.83133	0.965	0.142	0	pDC	Tmem123
Gadd45b	0	5.771062	0.847	0.046	0	pDC	Gadd45b
Serpinb6b	0	8.104553	0.8	0.015	0	pDC	Serpinb6b
Ccl53	0	6.168013	0.936	0.154	0	pDC	Ccl5
Lsp14	0	4.059873	0.975	0.201	0	pDC	Lsp1
Socs2	0	7.016438	0.787	0.018	0	pDC	Socs2
S100a114	0	2.458054	0.916	0.148	0	pDC	S100a11
Relb2	0	4.189778	0.931	0.164	0	pDC	Relb
Nudt17	0	10.11583	0.767	0.002	0	pDC	Nudt17
Marcksl13	0	4.322354	0.918	0.154	0	pDC	Marcksl1
Cnn24	0	3.993473	0.884	0.12	0	pDC	Cnn2
Il4i1	0	5.86989	0.807	0.048	0	pDC	Il4i1
Ccl221	0	9.774122	0.757	0.003	0	pDC	Ccl22
Plxnc11	0	5.07292	0.804	0.054	0	pDC	Plxnc1
Pcgf5	0	4.744227	0.839	0.089	0	pDC	Pcgf5
Myo1g3	0	4.021591	0.859	0.12	0	pDC	Myo1g
Mmp25	0	5.990591	0.762	0.023	0	pDC	Mmp25
Itga45	0	3.673716	0.844	0.108	0	pDC	Itga4
Serpinb9	0	5.656629	0.78	0.049	0	pDC	Serpinb9
Glipr21	0	5.492154	0.757	0.04	0	pDC	Glipr2
Gpr1321	0	5.303868	0.745	0.029	0	pDC	Gpr132
Mreg	0	9.672125	0.705	0.002	0	pDC	Mreg
Rabgap1l1	0	3.962648	0.804	0.102	0	pDC	Rabgap1l
Stat4	0	6.998916	0.71	0.012	0	pDC	Stat4
Strip2	0	6.494514	0.708	0.011	0	pDC	Strip2
Zmynd15	0	6.794406	0.733	0.037	0	pDC	Zmynd15
Bcl2a1d1	0	4.829076	0.755	0.061	0	pDC	Bcl2a1d
Id23	0	3.625082	0.876	0.185	0	pDC	Id2
Net11	0	6.54662	0.705	0.018	0	pDC	Net1
Samsn14	0	3.50042	0.923	0.242	0	pDC	Samsn1
Actn12	0	4.046476	0.748	0.069	0	pDC	Actn1
Clic45	0	3.409629	0.886	0.209	0	pDC	Clic4
Etv32	0	3.624507	0.824	0.149	0	pDC	Etv3
Cblb1	0	3.696622	0.785	0.113	0	pDC	Cblb
Dusp5	0	5.809523	0.691	0.019	0	pDC	Dusp5
Anxa24	0	3.146533	0.807	0.137	0	pDC	Anxa2
Ly75	0	6.780793	0.681	0.013	0	pDC	Ly75
Tbc1d42	0	4.0805	0.772	0.105	0	pDC	Tbc1d4
Adora2a	0	7.098614	0.671	0.006	0	pDC	Adora2a
Jak22	0	3.469607	0.809	0.144	0	pDC	Jak2
Bhlhe401	0	4.616508	0.696	0.042	0	pDC	Bhlhe40

Csrp11	0	4.259265	0.725	0.074	0	pDC	Csrp1
Cd2743	0	3.506096	0.782	0.132	0	pDC	Cd274
Flt31	0	5.331462	0.661	0.023	0	pDC	Flt3
Rftn15	0	3.26183	0.782	0.145	0	pDC	Rftn1
Birc2	0	3.485553	0.748	0.117	0	pDC	Birc2
4930523C(	0	4.008892	0.673	0.052	0	pDC	4930523C07Rik
Tes1	0	3.494157	0.713	0.094	0	pDC	Tes
Gyg4	0	4.265666	0.762	0.15	0	pDC	Gyg
Il15ra1	0	3.499503	0.715	0.105	0	pDC	Il15ra
Bcl2l14	0	8.598353	0.616	0.007	0	pDC	Bcl2l14
Arl5c4	0	3.590161	0.745	0.136	0	pDC	Arl5c
Gnb4	0	6.688671	0.621	0.013	0	pDC	Gnb4
Tmtc2	0	4.769393	0.656	0.054	0	pDC	Tmtc2
Nfkb22	0	3.474434	0.705	0.105	0	pDC	Nfkb2
Pakap.1	0	6.143609	0.604	0.009	0	pDC	Pakap.1
Avpi11	0	5.03124	0.619	0.029	0	pDC	Avpi1
Nr4a3	0	6.210473	0.597	0.011	0	pDC	Nr4a3
Mxd11	0	3.186196	0.663	0.078	0	pDC	Mxd1
Ass12	0	4.00346	0.641	0.056	0	pDC	Ass1
Gypc	0	6.082794	0.587	0.011	0	pDC	Gypc
Cd403	0	4.441913	0.631	0.061	0	pDC	Cd40
Ramp3	0	9.92777	0.567	0.002	0	pDC	Ramp3
Zbtb461	0	4.227028	0.601	0.04	0	pDC	Zbtb46
Adam81	0	4.058204	0.604	0.044	0	pDC	Adam8
Ncoa71	0	4.022926	0.653	0.094	0	pDC	Ncoa7
Gm13546	0	12.39612	0.557	0	0	pDC	Gm13546
Nostrin	0	5.731279	0.579	0.025	0	pDC	Nostrin
Insl6	0	7.650396	0.559	0.006	0	pDC	Insl6
Sema7a	0	6.022855	0.559	0.008	0	pDC	Sema7a
Adcy6	0	6.391076	0.554	0.007	0	pDC	Adcy6
Pmaip11	0	3.869416	0.597	0.059	0	pDC	Pmaip1
Gramd31	0	4.169556	0.584	0.048	0	pDC	Gramd3
Ankrd33b	0	5.809859	0.54	0.009	0	pDC	Ankrd33b
Hmgn3	0	3.872783	0.567	0.04	0	pDC	Hmgn3
Cd200	0	6.639781	0.53	0.006	0	pDC	Cd200
Foxp4	0	4.60479	0.552	0.029	0	pDC	Foxp4
Sinhcaf	0	4.344486	0.545	0.03	0	pDC	Sinhcaf
Lrrc8c1	0	3.532217	0.572	0.061	0	pDC	Lrrc8c
Dpp41	0	4.658369	0.527	0.024	0	pDC	Dpp4
S100a43	0	3.528816	0.557	0.058	0	pDC	S100a4
P2ry10	0	6.278652	0.5	0.008	0	pDC	P2ry10
Ptger4	0	3.882826	0.525	0.042	0	pDC	Ptger4
Grasp	0	4.687052	0.502	0.025	0	pDC	Grasp
Poglut1	0	3.343936	0.532	0.06	0	pDC	Poglut1

Il12b	0	9.702406	0.473	0.002	0	pDC	Il12b
Adam19	0	4.258326	0.473	0.03	0	pDC	Adam19
Ccl17	0	6.799609	0.443	0.007	0	pDC	Ccl17
Slc22a23	0	5.322759	0.446	0.012	0	pDC	Slc22a23
Tmem150c	0	7.305987	0.421	0.002	0	pDC	Tmem150c
Adgrg6	0	6.812633	0.423	0.005	0	pDC	Adgrg6
Abtb2	0	4.332518	0.438	0.022	0	pDC	Abtb2
Slc4a8	0	5.609498	0.426	0.017	0	pDC	Slc4a8
Sh3bp4	0	3.822447	0.438	0.03	0	pDC	Sh3bp4
Adam23	0	5.416852	0.418	0.01	0	pDC	Adam23
Klrk11	0	3.928311	0.436	0.033	0	pDC	Klrk1
AA4671971	0	3.901109	0.423	0.023	0	pDC	AA467197
Papss2	0	5.62249	0.406	0.01	0	pDC	Papss2
Zfp3661	0	5.305611	0.403	0.011	0	pDC	Zfp366
Tnfrsf4	0	6.77839	0.396	0.005	0	pDC	Tnfrsf4
Arhgap28	0	6.789993	0.389	0.004	0	pDC	Arhgap28
Castor2	0	3.329357	0.418	0.037	0	pDC	Castor2
Apol7c	0	8.470086	0.381	0.004	0	pDC	Apol7c
Fas	0	4.058739	0.403	0.028	0	pDC	Fas
Gm47662	0	11.41583	0.369	0	0	pDC	Gm47662
Arpin	0	6.057913	0.374	0.006	0	pDC	Arpin
Tnfrsf18	0	5.451922	0.371	0.01	0	pDC	Tnfrsf18
Mir155hg	0	7.522668	0.361	0.002	0	pDC	Mir155hg
Tnip31	0	4.472967	0.374	0.018	0	pDC	Tnip3
Pdcd1lg2	0	5.273621	0.371	0.015	0	pDC	Pdcd1lg2
Slco5a1	0	9.42661	0.349	0.001	0	pDC	Slco5a1
Vopp1	0	3.967037	0.369	0.023	0	pDC	Vopp1
Gm10851	0	6.354544	0.344	0.005	0	pDC	Gm10851
Gm16685	0	7.82664	0.337	0.002	0	pDC	Gm16685
Nck2	0	4.468021	0.347	0.012	0	pDC	Nck2
Rgs121	0	3.327142	0.351	0.026	0	pDC	Rgs12
Lad1	0	10.44291	0.324	0	0	pDC	Lad1
Stx111	0	3.283373	0.351	0.028	0	pDC	Stx11
Mab21l3	0	7.907053	0.322	0.003	0	pDC	Mab21l3
Tnfrsf9	0	6.543285	0.317	0.003	0	pDC	Tnfrsf9
Nectin1	0	4.400328	0.319	0.013	0	pDC	Nectin1
Ndnf	0	5.826483	0.304	0.005	0	pDC	Ndnf
Stap2	0	6.730693	0.295	0.003	0	pDC	Stap2
Fam177a	0	5.157586	0.292	0.008	0	pDC	Fam177a
Arc	0	5.91739	0.285	0.003	0	pDC	Arc
Il2ra	0	5.694391	0.285	0.004	0	pDC	Il2ra
Dscaml1	0	6.196511	0.277	0.005	0	pDC	Dscaml1
Tm4sf5	0	7.90912	0.275	0.003	0	pDC	Tm4sf5
Vsig10	0	4.544494	0.282	0.01	0	pDC	Vsig10

Slc27a3	0	6.588198	0.272	0.006	0	pDC	Slc27a3
Synpo2	0	6.233474	0.262	0.003	0	pDC	Synpo2
Htr7	0	5.602968	0.265	0.007	0	pDC	Htr7
Hsh2d	0	4.41978	0.26	0.01	0	pDC	Hsh2d
Cx3cl1	0	4.703498	0.252	0.009	0	pDC	Cx3cl1
Gins3	0	4.02099	0.257	0.014	0	pDC	Gins3
Fgfbp3	0	5.185273	0.25	0.008	0	pDC	Fgfbp3
Tpm1	6.43E-305	3.04415	0.649	0.097	1.58E-300	pDC	Tpm1
Cpne22	2.69E-304	3.328965	0.498	0.057	6.58E-300	pDC	Cpne2
Fabp51	3.08E-303	3.859364	0.745	0.138	7.53E-299	pDC	Fabp5
Mthfd22	2.17E-299	3.200538	0.671	0.108	5.33E-295	pDC	Mthfd2
Slamf72	5.26E-299	2.962352	0.691	0.112	1.29E-294	pDC	Slamf7
Idi1	7.69E-299	3.949345	0.384	0.035	1.88E-294	pDC	Idi1
Aebp21	1.89E-297	2.91477	0.71	0.12	4.62E-293	pDC	Aebp2
Phlpp1	9.72E-296	3.178769	0.653	0.1	2.38E-291	pDC	Phlpp1
Tmem39a	4.34E-293	3.399098	0.629	0.097	1.06E-288	pDC	Tmem39a
Vim4	7.79E-291	3.221997	0.861	0.195	1.91E-286	pDC	Vim
Nabp1	5.37E-288	3.354093	0.416	0.042	1.32E-283	pDC	Nabp1
Dusp2	2.00E-286	3.237402	0.359	0.031	4.90E-282	pDC	Dusp2
Crip13	2.60E-283	2.82821	0.824	0.181	6.37E-279	pDC	Crip1
Eno31	1.78E-282	3.674228	0.381	0.036	4.36E-278	pDC	Eno3
Fscn12	2.59E-277	4.517619	0.928	0.347	6.33E-273	pDC	Fscn1
Gm159872	1.94E-272	3.082718	0.347	0.03	4.74E-268	pDC	Gm15987
Cbfa2t32	4.46E-272	2.949152	0.757	0.149	1.09E-267	pDC	Cbfa2t3
Fam49a3	1.90E-269	2.873926	0.743	0.145	4.66E-265	pDC	Fam49a
Cdc42ep32	3.75E-269	3.271914	0.51	0.067	9.19E-265	pDC	Cdc42ep3
Cxcl164	1.85E-268	2.873024	0.938	0.299	4.54E-264	pDC	Cxcl16
H2-M2	2.36E-264	3.815975	0.304	0.025	5.79E-260	pDC	H2-M2
Cdkn1a2	2.86E-263	3.168755	0.604	0.095	7.01E-259	pDC	Cdkn1a
AW112010	3.29E-263	3.583312	0.903	0.286	8.06E-259	pDC	AW112010
St8sia45	4.98E-263	2.924537	0.619	0.098	1.22E-258	pDC	St8sia4
Pim16	1.80E-262	2.693472	0.74	0.143	4.40E-258	pDC	Pim1
Pde4b4	1.84E-261	2.721831	0.884	0.23	4.51E-257	pDC	Pde4b
Mif4gd3	2.16E-258	3.064552	0.787	0.185	5.28E-254	pDC	Mif4gd
Plscr11	1.14E-255	3.579742	0.369	0.037	2.79E-251	pDC	Plscr1
Tagln23	3.96E-250	3.543391	0.814	0.212	9.70E-246	pDC	Tagln2
Lsr1	3.32E-247	3.399207	0.265	0.02	8.13E-243	pDC	Lsr
Tpm44	3.96E-246	2.662084	0.752	0.163	9.70E-242	pDC	Tpm4
Ift57	5.21E-244	3.057283	0.394	0.043	1.28E-239	pDC	Ift57
Rps27l5	3.82E-240	3.555235	0.913	0.357	9.36E-236	pDC	Rps27l
Psme25	6.53E-238	3.067007	0.965	0.519	1.60E-233	pDC	Psme2
Ccser2	1.59E-235	2.673067	0.468	0.061	3.89E-231	pDC	Ccser2
Mmd	3.58E-235	2.893023	0.48	0.066	8.76E-231	pDC	Mmd
Napsa3	8.03E-231	2.306528	0.493	0.067	1.97E-226	pDC	Napsa

Syngr23	4.20E-230	3.019377	0.953	0.568	1.03E-225	pDC	Syngr2
Bcl2a1a4	5.80E-230	2.942985	0.663	0.133	1.42E-225	pDC	Bcl2a1a
Calm16	2.31E-229	3.338434	0.98	0.592	5.65E-225	pDC	Calm1
Batf33	1.59E-227	2.769574	0.777	0.194	3.89E-223	pDC	Batf3
Pmvk1	1.61E-227	3.208021	0.562	0.096	3.94E-223	pDC	Pmvk
Mcemp12	3.47E-227	2.585096	0.431	0.054	8.49E-223	pDC	Mcemp1
Prnp	1.30E-226	2.995297	0.428	0.055	3.18E-222	pDC	Prnp
Rel3	1.22E-225	2.96347	0.876	0.287	3.00E-221	pDC	Rel
Rab11fip1	7.01E-224	3.000046	0.265	0.022	1.72E-219	pDC	Rab11fip1
Cd80	5.58E-220	3.339311	0.5	0.077	1.37E-215	pDC	Cd80
Sdhaf1	3.48E-218	2.710343	0.574	0.101	8.52E-214	pDC	Sdhaf1
Actg14	4.73E-216	2.944393	0.983	0.782	1.16E-211	pDC	Actg1
Crybg11	8.26E-215	3.042134	0.389	0.048	2.02E-210	pDC	Crybg1
Tuba1a3	9.32E-213	3.042244	0.819	0.248	2.28E-208	pDC	Tuba1a
Snhg15	9.87E-213	3.314128	0.351	0.04	2.42E-208	pDC	Snhg15
Rgs13	1.82E-212	2.917372	0.822	0.235	4.45E-208	pDC	Rgs1
Phf11a1	1.72E-210	3.560922	0.351	0.041	4.21E-206	pDC	Phf11a
Relt	2.48E-210	2.970374	0.342	0.038	6.08E-206	pDC	Relt
Btg14	4.81E-209	2.436213	0.96	0.528	1.18E-204	pDC	Btg1
Epsti14	8.58E-208	3.610504	0.918	0.411	2.10E-203	pDC	Epsti1
C130026I2	6.06E-204	3.431556	0.27	0.025	1.48E-199	pDC	C130026I21Rik
Got11	3.46E-203	3.014479	0.626	0.132	8.47E-199	pDC	Got1
Mob3a1	6.03E-203	2.606258	0.636	0.132	1.48E-198	pDC	Mob3a
Rap2b3	9.36E-203	2.880447	0.616	0.128	2.29E-198	pDC	Rap2b
Iscu1	4.16E-202	2.527902	0.936	0.468	1.02E-197	pDC	Iscu
Clec2d6	1.12E-197	2.48896	0.839	0.263	2.75E-193	pDC	Clec2d
Lactb1	4.99E-197	2.823712	0.728	0.193	1.22E-192	pDC	Lactb
Iqgap16	1.23E-196	2.067252	0.723	0.166	3.01E-192	pDC	Iqgap1
N4bp2l1	8.55E-195	2.580284	0.755	0.197	2.09E-190	pDC	N4bp2l1
Txndc175	6.86E-193	3.617543	0.842	0.309	1.68E-188	pDC	Txndc17
Kynu1	1.08E-192	2.943109	0.379	0.05	2.65E-188	pDC	Kynu
Fnbp1l	2.49E-192	2.477796	0.465	0.072	6.10E-188	pDC	Fnbp1l
Mrtfa5	1.84E-190	2.436682	0.809	0.23	4.50E-186	pDC	Mrtfa
Birc34	2.24E-190	2.401425	0.795	0.232	5.49E-186	pDC	Birc3
Tbc1d83	6.15E-190	2.636512	0.792	0.24	1.51E-185	pDC	Tbc1d8
Psme15	2.97E-189	2.226937	0.953	0.604	7.26E-185	pDC	Psme1
Nfil3	1.08E-188	3.390562	0.416	0.062	2.65E-184	pDC	Nfil3
Nr4a2	6.74E-188	3.985962	0.267	0.026	1.65E-183	pDC	Nr4a2
Rab213	8.62E-186	2.375662	0.792	0.233	2.11E-181	pDC	Rab21
Sepsecs	1.42E-185	2.753912	0.356	0.045	3.47E-181	pDC	Sepsecs
Tmsb4x5	3.66E-185	1.790485	0.988	0.968	8.97E-181	pDC	Tmsb4x
Rras21	1.74E-184	2.436147	0.446	0.068	4.26E-180	pDC	Rras2
Rasa2	2.05E-183	2.678802	0.5	0.088	5.02E-179	pDC	Rasa2
Fyn2	1.14E-182	2.400028	0.574	0.113	2.79E-178	pDC	Fyn

Rnf1151	2.58E-182	2.582568	0.765	0.223	6.31E-178	pDC	Rnf115
Rpsa5	9.61E-182	1.899932	0.978	0.834	2.35E-177	pDC	Rpsa
Gm12216	1.20E-180	2.987531	0.448	0.072	2.93E-176	pDC	Gm12216
Uap1	5.27E-179	3.127607	0.554	0.114	1.29E-174	pDC	Uap1
Tmsb103	5.80E-178	2.104221	0.975	0.637	1.42E-173	pDC	Tmsb10
Nfkbib4	1.10E-177	2.65133	0.691	0.179	2.70E-173	pDC	Nfkbib
Ctnna11	2.05E-177	2.466863	0.653	0.154	5.02E-173	pDC	Ctnna1
Casp3	4.75E-177	2.580182	0.522	0.099	1.16E-172	pDC	Casp3
CAAA0114	9.58E-177	2.38222	0.894	0.401	2.35E-172	pDC	CAAA01147332.1
Sub13	2.91E-176	2.383164	0.923	0.488	7.12E-172	pDC	Sub1
Dnajc12	3.54E-174	3.108842	0.282	0.031	8.67E-170	pDC	Dnajc12
S100a64	1.29E-173	0.824326	0.606	0.12	3.16E-169	pDC	S100a6
Actb5	1.80E-173	1.776298	0.993	0.958	4.41E-169	pDC	Actb
Smarce11	2.17E-173	2.649347	0.644	0.156	5.32E-169	pDC	Smarce1
Nuak2	2.33E-173	2.401181	0.562	0.114	5.70E-169	pDC	Nuak2
Rogdi3	3.60E-173	2.359022	0.879	0.371	8.82E-169	pDC	Rogdi
Sp1403	1.32E-172	2.925223	0.671	0.18	3.23E-168	pDC	Sp140
Rab91	2.69E-171	2.56739	0.569	0.121	6.60E-167	pDC	Rab9
Rps195	1.19E-170	1.773417	0.97	0.801	2.91E-166	pDC	Rps19
Atox14	1.55E-169	2.208531	0.95	0.593	3.80E-165	pDC	Atox1
H2afz4	3.33E-169	2.413233	0.938	0.573	8.16E-165	pDC	H2afz
Bcl2a1b3	6.28E-168	2.477802	0.889	0.439	1.54E-163	pDC	Bcl2a1b
Anxa44	1.58E-167	2.494304	0.624	0.151	3.88E-163	pDC	Anxa4
Gucd1	2.78E-167	2.633223	0.374	0.054	6.81E-163	pDC	Gucd1
Gpd2	7.75E-167	3.354234	0.371	0.055	1.90E-162	pDC	Gpd2
Ezh2	1.06E-166	2.729653	0.359	0.05	2.59E-162	pDC	Ezh2
Cd834	3.59E-166	1.890911	0.963	0.48	8.78E-162	pDC	Cd83
B4galnt13	9.30E-166	2.407914	0.661	0.17	2.28E-161	pDC	B4galnt1
Polr3c1	1.13E-165	2.412901	0.557	0.119	2.77E-161	pDC	Polr3c
Map3k141	1.75E-164	2.581364	0.644	0.159	4.28E-160	pDC	Map3k14
Spr2	8.59E-164	2.493848	0.562	0.124	2.10E-159	pDC	Spr
Ifi304	1.03E-163	2.3807	0.938	0.596	2.52E-159	pDC	Ifi30
Fam89a	2.01E-163	2.6886	0.332	0.044	4.93E-159	pDC	Fam89a
Cmc2	5.88E-163	3.139922	0.359	0.053	1.44E-158	pDC	Cmc2
Rbbp8	5.50E-161	2.491612	0.465	0.084	1.35E-156	pDC	Rbbp8
Pgap23	3.23E-158	2.333404	0.636	0.161	7.91E-154	pDC	Pgap2
Psmb85	3.35E-156	1.900432	0.941	0.601	8.20E-152	pDC	Psmb8
Shtn11	4.44E-156	2.493335	0.421	0.072	1.09E-151	pDC	Shtn1
Plaat35	6.61E-156	2.434363	0.703	0.207	1.62E-151	pDC	Plaat3
Cd476	4.36E-155	1.896233	0.918	0.459	1.07E-150	pDC	Cd47
Pfkfb3	4.68E-154	2.337115	0.75	0.241	1.15E-149	pDC	Pfkfb3
Sri5	2.00E-152	2.216561	0.817	0.321	4.90E-148	pDC	Sri
Ube2l63	4.37E-152	2.385255	0.626	0.163	1.07E-147	pDC	Ube2l6
Txn15	3.41E-151	2.301435	0.842	0.323	8.36E-147	pDC	Txn1

Srgn4	3.74E-151	2.288585	0.936	0.553	9.15E-147	pDC	Srgn
Il2rg6	5.50E-151	1.895101	0.75	0.219	1.35E-146	pDC	Il2rg
Tmem19	8.54E-151	2.553088	0.535	0.118	2.09E-146	pDC	Tmem19
Ssh1	1.54E-149	2.439273	0.401	0.067	3.78E-145	pDC	Ssh1
Vav36	2.11E-148	2.001046	0.438	0.078	5.16E-144	pDC	Vav3
Cers65	7.61E-148	2.3564	0.708	0.205	1.86E-143	pDC	Cers6
Swap702	5.85E-147	2.391158	0.644	0.174	1.43E-142	pDC	Swap70
Tspo7	9.30E-147	2.246688	0.891	0.449	2.28E-142	pDC	Tspo
Ric1	1.97E-146	2.337955	0.468	0.09	4.82E-142	pDC	Ric1
Tmem131l:	2.69E-145	2.005004	0.71	0.204	6.59E-141	pDC	Tmem131l
Nfkbia6	8.19E-143	1.90005	0.923	0.488	2.00E-138	pDC	Nfkbia
Cd1d1	7.78E-142	3.526536	0.297	0.041	1.91E-137	pDC	Cd1d1
Klf65	1.20E-141	2.104349	0.851	0.369	2.94E-137	pDC	Klf6
Ehd13	1.80E-141	2.359978	0.502	0.107	4.40E-137	pDC	Ehd1
Traf6	4.63E-141	2.135616	0.537	0.121	1.13E-136	pDC	Traf6
Sema4a4	1.33E-140	2.29228	0.505	0.11	3.25E-136	pDC	Sema4a
Sumo24	5.33E-139	1.807753	0.918	0.511	1.30E-134	pDC	Sumo2
Adap12	1.80E-138	2.237533	0.577	0.145	4.40E-134	pDC	Adap1
Emd3	1.25E-137	2.163849	0.619	0.167	3.06E-133	pDC	Emd
Gramd1b	2.62E-137	2.88434	0.515	0.121	6.40E-133	pDC	Gramd1b
Mrpl144	8.82E-137	2.143794	0.693	0.219	2.16E-132	pDC	Mrpl14
Ifi2093	1.14E-135	2.087341	0.507	0.112	2.80E-131	pDC	Ifi209
Hivep1	6.18E-135	1.896806	0.54	0.124	1.51E-130	pDC	Hivep1
Arl5a	1.66E-134	2.237002	0.577	0.146	4.07E-130	pDC	Arl5a
Zfc3h11	3.98E-134	2.157497	0.693	0.215	9.74E-130	pDC	Zfc3h1
Tubb54	6.76E-133	2.111558	0.886	0.469	1.66E-128	pDC	Tubb5
Psmg43	2.52E-132	2.333721	0.624	0.179	6.16E-128	pDC	Psmg4
Chp11	2.37E-131	2.165831	0.574	0.147	5.81E-127	pDC	Chp1
2410006H:	8.71E-131	2.669442	0.894	0.487	2.13E-126	pDC	2410006H16Rik
Tank3	9.31E-131	2.019325	0.683	0.208	2.28E-126	pDC	Tank
Cacna1s	1.53E-129	2.797857	0.275	0.038	3.75E-125	pDC	Cacna1s
Gbp52	7.99E-129	2.086047	0.53	0.126	1.96E-124	pDC	Gbp5
Atl3	1.08E-128	1.93123	0.584	0.15	2.65E-124	pDC	Atl3
Runx32	1.78E-128	2.639429	0.307	0.047	4.36E-124	pDC	Runx3
Mvp5	1.60E-127	2.033045	0.639	0.188	3.91E-123	pDC	Mvp
Eif15	7.40E-127	1.266934	0.973	0.834	1.81E-122	pDC	Eif1
Htra2	7.44E-126	1.934487	0.673	0.205	1.82E-121	pDC	Htra2
Ccdc88a3	6.39E-125	2.066704	0.624	0.177	1.57E-120	pDC	Ccdc88a
Pafah1b32	1.79E-124	2.074331	0.559	0.143	4.39E-120	pDC	Pafah1b3
Psmb94	5.67E-124	1.729697	0.899	0.482	1.39E-119	pDC	Psmb9
Triobp1	5.91E-124	2.216359	0.431	0.09	1.45E-119	pDC	Triobp
Myl65	6.95E-124	1.685764	0.931	0.609	1.70E-119	pDC	Myl6
Polb1	3.03E-123	2.123342	0.567	0.15	7.41E-119	pDC	Polb
Nfkbie1	5.46E-122	2.100833	0.46	0.101	1.34E-117	pDC	Nfkbie

Pfn14	2.50E-121	1.395797	0.96	0.831	6.11E-117	pDC	Pfn1
H2-Q63	2.72E-121	1.819305	0.851	0.382	6.66E-117	pDC	H2-Q6
H2-Q74	3.32E-121	1.581069	0.946	0.482	8.14E-117	pDC	H2-Q7
Clec2i	4.42E-121	2.973803	0.25	0.034	1.08E-116	pDC	Clec2i
Tbrg11	8.40E-121	2.074955	0.45	0.098	2.06E-116	pDC	Tbrg1
Il152	8.95E-120	2.040991	0.636	0.187	2.19E-115	pDC	Il15
Hspa85	2.64E-119	1.52417	0.96	0.741	6.47E-115	pDC	Hspa8
Rpl196	6.28E-118	1.094254	0.975	0.89	1.54E-113	pDC	Rpl19
Med111	9.53E-118	2.219714	0.473	0.112	2.33E-113	pDC	Med11
Btf34	4.26E-116	1.445856	0.936	0.613	1.04E-111	pDC	Btf3
Gfpt1	5.50E-116	2.121589	0.522	0.134	1.35E-111	pDC	Gfpt1
Tapbpl2	1.81E-115	1.987363	0.609	0.178	4.44E-111	pDC	Tapbpl
Ppp1r15a3	2.02E-115	1.739581	0.634	0.189	4.94E-111	pDC	Ppp1r15a
Ccnd21	1.47E-114	3.054069	0.312	0.054	3.60E-110	pDC	Ccnd2
Lrrk13	1.68E-114	1.668371	0.861	0.384	4.10E-110	pDC	Lrrk1
Csf2rb5	2.78E-112	1.97048	0.708	0.256	6.81E-108	pDC	Csf2rb
Nav13	5.63E-112	1.980858	0.535	0.138	1.38E-107	pDC	Nav1
Ece11	2.54E-111	2.391132	0.334	0.061	6.23E-107	pDC	Ece1
Bcl31	5.49E-111	2.315354	0.401	0.086	1.34E-106	pDC	Bcl3
Rab8b2	5.94E-111	2.011518	0.822	0.38	1.45E-106	pDC	Rab8b
Csrnp1	6.50E-111	2.363351	0.27	0.041	1.59E-106	pDC	Csrnp1
Socs13	2.51E-110	2.039892	0.413	0.09	6.16E-106	pDC	Socs1
Rps246	5.80E-110	1.061071	0.99	0.929	1.42E-105	pDC	Rps24
Arhgap312	3.03E-109	2.204214	0.755	0.309	7.41E-105	pDC	Arhgap31
Irf13	8.18E-108	1.763389	0.802	0.328	2.00E-103	pDC	Irf1
Trim35	2.72E-107	1.714231	0.631	0.192	6.65E-103	pDC	Trim35
Castor1	1.59E-106	3.004225	0.292	0.051	3.89E-102	pDC	Castor1
Rpl95	2.00E-106	1.020069	0.975	0.876	4.89E-102	pDC	Rpl9
Hmga13	4.27E-106	2.147338	0.512	0.141	1.05E-101	pDC	Hmga1
Ppia5	1.11E-105	1.004904	0.968	0.881	2.71E-101	pDC	Ppia
Slc25a10	1.27E-105	2.304054	0.314	0.057	3.11E-101	pDC	Slc25a10
Rassf32	2.44E-105	1.797012	0.604	0.185	5.97E-101	pDC	Rassf3
Ppdpf3	4.93E-105	2.130728	0.55	0.163	1.21E-100	pDC	Ppdpf
Rpl276	5.39E-105	1.216232	0.955	0.777	1.32E-100	pDC	Rpl27
Ldlr1	1.27E-104	2.253023	0.329	0.061	3.10E-100	pDC	Ldlr
Grk32	1.60E-104	1.789986	0.525	0.14	3.92E-100	pDC	Grk3
Gas53	2.14E-104	2.04145	0.889	0.558	5.25E-100	pDC	Gas5
Stard7	3.38E-104	2.016698	0.366	0.075	8.28E-100	pDC	Stard7
Ikbkb3	4.66E-104	1.634413	0.693	0.237	1.14E-99	pDC	Ikbkb
Nap1l14	4.79E-104	1.787987	0.658	0.227	1.17E-99	pDC	Nap1l1
Nub11	1.98E-103	1.671607	0.636	0.201	4.85E-99	pDC	Nub1
Phf11b3	2.97E-103	2.135353	0.542	0.159	7.28E-99	pDC	Phf11b
Samhd15	3.05E-103	1.466345	0.849	0.392	7.48E-99	pDC	Samhd1
Apobec32	3.20E-103	1.524247	0.866	0.428	7.83E-99	pDC	Apobec3

Fam129a4	3.29E-103	1.857314	0.545	0.154	8.06E-99	pDC	Fam129a
Fau5	5.05E-103	0.967503	0.985	0.94	1.24E-98	pDC	Fau
Hint31	5.44E-103	1.913378	0.448	0.109	1.33E-98	pDC	Hint3
Serpina3g2	5.86E-103	2.194593	0.295	0.051	1.43E-98	pDC	Serpina3g
Gbp25	1.07E-102	1.592128	0.639	0.201	2.62E-98	pDC	Gbp2
Dok11	7.98E-102	1.894149	0.611	0.202	1.96E-97	pDC	Dok1
Rpl245	6.06E-101	1.153925	0.958	0.79	1.48E-96	pDC	Rpl24
Tbc1d12	9.11E-101	1.940163	0.559	0.164	2.23E-96	pDC	Tbc1d1
Bcl7c3	1.77E-100	1.889435	0.507	0.139	4.32E-96	pDC	Bcl7c
Nup2101	2.07E-100	2.454631	0.252	0.04	5.07E-96	pDC	Nup210
Rpl345	3.53E-100	1.125359	0.978	0.844	8.64E-96	pDC	Rpl34
Mical3	1.18E-99	1.965547	0.329	0.063	2.90E-95	pDC	Mical3
Edf15	2.23E-99	1.494906	0.871	0.462	5.46E-95	pDC	Edf1
Inpp5b	3.48E-99	2.30452	0.354	0.074	8.52E-95	pDC	Inpp5b
Nt5c4	1.87E-98	1.809697	0.614	0.202	4.59E-94	pDC	Nt5c
Crem	2.62E-98	2.344767	0.265	0.044	6.42E-94	pDC	Crem
Erlin1	8.70E-98	2.064086	0.386	0.086	2.13E-93	pDC	Erlin1
Rbm172	1.79E-97	1.796437	0.569	0.177	4.38E-93	pDC	Rbm17
Mtmr4	7.06E-97	1.959322	0.314	0.059	1.73E-92	pDC	Mtmr4
Sh3bgrl36	7.42E-97	1.250558	0.943	0.749	1.82E-92	pDC	Sh3bgrl3
Srebf21	2.42E-96	1.786653	0.408	0.094	5.92E-92	pDC	Srebf2
Foxn21	3.35E-96	2.022706	0.433	0.109	8.21E-92	pDC	Foxn2
Fam32a3	1.15E-95	2.001654	0.609	0.211	2.83E-91	pDC	Fam32a
Mthfsl	2.48E-95	1.752424	0.567	0.178	6.08E-91	pDC	Mthfsl
Stat5a	3.12E-95	2.354011	0.285	0.051	7.64E-91	pDC	Stat5a
Atrnl13	4.63E-95	2.200376	0.399	0.093	1.13E-90	pDC	Atrnl1
Supt4a4	6.77E-95	1.648073	0.735	0.306	1.66E-90	pDC	Supt4a
B2m3	8.94E-95	0.948786	0.98	0.909	2.19E-90	pDC	B2m
Rpl185	2.18E-94	1.016011	0.963	0.839	5.34E-90	pDC	Rpl18
Zc3h12c	2.23E-94	2.593433	0.314	0.062	5.47E-90	pDC	Zc3h12c
Rrad	4.26E-94	2.518547	0.319	0.064	1.04E-89	pDC	Rrad
H3f3a5	4.52E-94	1.339133	0.921	0.673	1.11E-89	pDC	H3f3a
H2-K13	3.86E-93	0.989061	0.98	0.88	9.45E-89	pDC	H2-K1
Izumo4	7.16E-93	2.106604	0.347	0.074	1.75E-88	pDC	Izumo4
Lima1	1.79E-92	2.228877	0.406	0.1	4.39E-88	pDC	Lima1
Nup85	3.14E-92	2.005133	0.317	0.063	7.69E-88	pDC	Nup85
Tmem176a	9.12E-92	1.756901	0.819	0.448	2.23E-87	pDC	Tmem176a
Ktn1	9.80E-92	1.6728	0.624	0.209	2.40E-87	pDC	Ktn1
Icam13	1.27E-91	1.532432	0.782	0.342	3.10E-87	pDC	Icam1
Tbc1d15	2.65E-91	1.701402	0.478	0.13	6.49E-87	pDC	Tbc1d15
Prr5l1	1.50E-90	2.08126	0.371	0.086	3.68E-86	pDC	Prr5l
Ogfrl11	3.83E-90	1.778723	0.73	0.329	9.38E-86	pDC	Ogfrl1
Slc33a1	7.67E-90	1.943137	0.386	0.091	1.88E-85	pDC	Slc33a1
Tspan34	3.39E-89	2.095951	0.785	0.394	8.30E-85	pDC	Tspan3

Ddhd12	6.11E-89	1.847313	0.49	0.14	1.50E-84	pDC	Ddhd1
Rps275	1.43E-88	1.386722	0.958	0.783	3.51E-84	pDC	Rps27
Rpl175	4.99E-88	0.959273	0.963	0.84	1.22E-83	pDC	Rpl17
Spop4	6.08E-88	1.492808	0.79	0.359	1.49E-83	pDC	Spop
Tuba1c2	2.48E-87	2.117224	0.433	0.117	6.07E-83	pDC	Tuba1c
H2-Q44	4.59E-87	1.456521	0.849	0.44	1.12E-82	pDC	H2-Q4
AI6622702	8.55E-87	1.957475	0.297	0.058	2.09E-82	pDC	AI662270
Rps146	2.09E-86	0.996152	0.97	0.81	5.12E-82	pDC	Rps14
Stk43	4.37E-86	1.528292	0.71	0.289	1.07E-81	pDC	Stk4
Il7r	4.81E-86	1.788949	0.465	0.127	1.18E-81	pDC	Il7r
Sav1	1.06E-85	1.798244	0.411	0.105	2.59E-81	pDC	Sav1
Psip11	2.43E-85	1.640077	0.411	0.103	5.95E-81	pDC	Psip1
Cox174	1.26E-84	1.680249	0.748	0.348	3.10E-80	pDC	Cox17
Etv64	9.58E-84	1.351914	0.928	0.585	2.35E-79	pDC	Etv6
Dynll21	1.21E-83	1.729258	0.545	0.179	2.96E-79	pDC	Dynll2
Pkib3	1.90E-83	2.734958	0.542	0.201	4.64E-79	pDC	Pkib
Limd12	3.51E-83	1.920854	0.332	0.073	8.60E-79	pDC	Limd1
Rala1	5.08E-83	1.517842	0.705	0.286	1.24E-78	pDC	Rala
Rnaseh2b	5.12E-82	1.865491	0.361	0.087	1.25E-77	pDC	Rnaseh2b
Zfp36l14	1.25E-81	1.187601	0.938	0.632	3.06E-77	pDC	Zfp36l1
Rpl416	1.61E-81	0.96482	0.985	0.903	3.94E-77	pDC	Rpl41
Cd2bp2	3.33E-81	1.905308	0.324	0.072	8.14E-77	pDC	Cd2bp2
Zfp524	3.66E-81	2.160953	0.386	0.099	8.97E-77	pDC	Zfp524
St3gal12	4.26E-81	2.275965	0.295	0.062	1.04E-76	pDC	St3gal1
Nans1	4.35E-81	2.176689	0.418	0.117	1.07E-76	pDC	Nans
Rps55	5.28E-81	0.931779	0.973	0.853	1.29E-76	pDC	Rps5
Rps95	8.77E-81	0.867201	0.98	0.912	2.15E-76	pDC	Rps9
Ubb6	1.53E-80	0.929666	0.975	0.866	3.75E-76	pDC	Ubb
Anxa55	2.36E-80	1.313111	0.73	0.304	5.77E-76	pDC	Anxa5
Nsmce11	3.53E-80	1.759393	0.463	0.135	8.65E-76	pDC	Nsmce1
Npc25	4.36E-80	1.34782	0.96	0.811	1.07E-75	pDC	Npc2
Tapbp4	1.12E-79	1.268487	0.869	0.502	2.74E-75	pDC	Tapbp
Pde4d2	1.95E-79	2.164503	0.493	0.154	4.78E-75	pDC	Pde4d
Myl12a5	3.05E-79	1.464655	0.817	0.453	7.47E-75	pDC	Myl12a
Alkbh6	2.02E-78	1.861594	0.438	0.127	4.94E-74	pDC	Alkbh6
Rpl395	1.81E-77	1.14379	0.95	0.822	4.43E-73	pDC	Rpl39
Zmiz22	2.05E-76	1.606508	0.455	0.135	5.03E-72	pDC	Zmiz2
Psme2b	2.21E-76	2.162155	0.252	0.049	5.41E-72	pDC	Psme2b
Bex31	5.74E-76	1.592061	0.391	0.104	1.40E-71	pDC	Bex3
Coro2a1	7.96E-76	1.816105	0.46	0.139	1.95E-71	pDC	Coro2a
Hat11	8.11E-76	1.6957	0.428	0.122	1.99E-71	pDC	Hat1
Fdps1	8.30E-76	2.122831	0.361	0.094	2.03E-71	pDC	Fdps
Rpl27a5	9.28E-76	0.882621	0.97	0.89	2.27E-71	pDC	Rpl27a
Bri3bp2	1.43E-75	1.649655	0.438	0.127	3.50E-71	pDC	Bri3bp

Cox5a5	1.45E-75	1.35116	0.839	0.463	3.55E-71	pDC	Cox5a
Malt12	3.11E-75	2.066971	0.46	0.141	7.61E-71	pDC	Malt1
Rpl18a5	1.04E-74	0.839012	0.975	0.901	2.54E-70	pDC	Rpl18a
Cyp51	1.37E-74	2.071018	0.262	0.053	3.36E-70	pDC	Cyp51
Tmem1344	2.21E-74	1.462377	0.639	0.254	5.41E-70	pDC	Tmem134
Chchd25	6.63E-74	1.437293	0.76	0.373	1.62E-69	pDC	Chchd2
Trps15	8.14E-74	1.324089	0.517	0.167	1.99E-69	pDC	Trps1
Fdft1	1.15E-73	2.189315	0.267	0.056	2.81E-69	pDC	Fdft1
Serf26	2.04E-73	0.952563	0.953	0.76	4.99E-69	pDC	Serf2
Pfdn56	4.11E-73	1.021683	0.928	0.691	1.01E-68	pDC	Pfdn5
Psmb105	1.03E-72	1.256714	0.79	0.382	2.53E-68	pDC	Psmb10
Uvrag4	1.74E-72	1.339565	0.842	0.444	4.26E-68	pDC	Uvrag
Prr14	2.59E-72	1.648294	0.423	0.123	6.35E-68	pDC	Prr14
Eif3f5	6.08E-72	1.149456	0.894	0.589	1.49E-67	pDC	Eif3f
Thap2	8.65E-72	1.79887	0.262	0.054	2.12E-67	pDC	Thap2
Rnf157	1.06E-71	2.099042	0.307	0.072	2.58E-67	pDC	Rnf157
Gtpbp41	3.57E-71	1.782694	0.465	0.151	8.74E-67	pDC	Gtpbp4
Rpl7a4	3.59E-71	0.933111	0.963	0.766	8.80E-67	pDC	Rpl7a
Rplp05	9.66E-71	0.949219	0.953	0.842	2.37E-66	pDC	Rplp0
Gga2	1.23E-70	2.12966	0.309	0.075	3.01E-66	pDC	Gga2
Ostf14	1.87E-70	1.364813	0.837	0.495	4.57E-66	pDC	Ostf1
Scand15	1.96E-70	1.361231	0.802	0.416	4.80E-66	pDC	Scand1
Mfhas1	2.65E-70	1.447817	0.423	0.121	6.48E-66	pDC	Mfhas1
Map4k41	3.22E-70	1.566783	0.819	0.491	7.88E-66	pDC	Map4k4
Slc44a1	4.69E-70	1.712033	0.349	0.09	1.15E-65	pDC	Slc44a1
Asl	9.25E-70	1.94687	0.304	0.073	2.27E-65	pDC	Asl
Rbpms1	1.01E-69	1.439324	0.458	0.142	2.48E-65	pDC	Rbpms
Snrpe4	1.69E-69	1.505817	0.733	0.352	4.15E-65	pDC	Snrpe
Scpep14	2.37E-69	1.383945	0.762	0.375	5.80E-65	pDC	Scpep1
Hax11	3.25E-69	1.788331	0.421	0.127	7.95E-65	pDC	Hax1
Rhog2	5.83E-69	1.137872	0.903	0.632	1.43E-64	pDC	Rhog
Ciao2b3	9.17E-69	1.404557	0.616	0.243	2.25E-64	pDC	Ciao2b
Arpp192	1.50E-68	1.398561	0.688	0.315	3.68E-64	pDC	Arpp19
Dusp113	1.95E-68	1.341334	0.606	0.233	4.78E-64	pDC	Dusp11
Ptpn22	2.07E-68	1.404971	0.465	0.146	5.06E-64	pDC	Ptpn2
Rps15a6	2.68E-68	0.854168	0.965	0.857	6.57E-64	pDC	Rps15a
Tspan332	1.04E-67	1.493678	0.47	0.152	2.55E-63	pDC	Tspan33
Auh	1.47E-67	1.749792	0.416	0.125	3.61E-63	pDC	Auh
Ccdc6	1.08E-66	1.628025	0.327	0.082	2.64E-62	pDC	Ccdc6
Zfand62	2.03E-66	1.400153	0.686	0.303	4.97E-62	pDC	Zfand6
Arf43	4.24E-66	1.241632	0.814	0.451	1.04E-61	pDC	Arf4
Rpl115	4.93E-66	0.747619	0.965	0.877	1.21E-61	pDC	Rpl11
Stat32	5.10E-66	1.332962	0.76	0.378	1.25E-61	pDC	Stat3
Rab8a4	6.35E-66	1.405117	0.552	0.204	1.56E-61	pDC	Rab8a

Psma75	7.29E-66	1.208633	0.78	0.413	1.79E-61	pDC	Psma7
Gpbp1	9.91E-66	1.350528	0.53	0.189	2.43E-61	pDC	Gpbp1
Nsmce3	1.55E-65	1.907782	0.438	0.142	3.79E-61	pDC	Nsmce3
C9orf72	2.23E-65	1.547236	0.458	0.148	5.46E-61	pDC	C9orf72
Itgb12	4.91E-65	1.232931	0.797	0.438	1.20E-60	pDC	Itgb1
Rtraf5	2.34E-64	1.203423	0.795	0.419	5.74E-60	pDC	Rtraf
Rps75	3.94E-64	0.787948	0.97	0.867	9.65E-60	pDC	Rps7
Abracl4	4.27E-64	1.484945	0.649	0.29	1.04E-59	pDC	Abracl
Rps116	4.53E-64	0.753251	0.968	0.896	1.11E-59	pDC	Rps11
Med102	4.97E-64	1.577845	0.465	0.156	1.22E-59	pDC	Med10
Arpc36	8.35E-64	0.989467	0.891	0.641	2.04E-59	pDC	Arpc3
Taf105	1.80E-63	1.204243	0.752	0.366	4.41E-59	pDC	Taf10
Epb411	2.12E-63	1.572203	0.55	0.208	5.19E-59	pDC	Epb41
Fmnl24	2.24E-63	1.268846	0.686	0.301	5.48E-59	pDC	Fmnl2
Snhg14	2.85E-63	1.975774	0.443	0.153	6.97E-59	pDC	Snhg1
Ifrd1	4.22E-63	1.65964	0.52	0.188	1.03E-58	pDC	Ifrd1
Gnai31	4.50E-63	1.407732	0.478	0.161	1.10E-58	pDC	Gnai3
Mar-52	5.48E-63	1.427135	0.589	0.242	1.34E-58	pDC	5-Mar
Agap1	5.68E-63	1.909271	0.354	0.098	1.39E-58	pDC	Agap1
Arpc55	1.76E-62	1.1219	0.804	0.43	4.30E-58	pDC	Arpc5
Rps135	2.03E-62	0.804891	0.958	0.85	4.97E-58	pDC	Rps13
Atp6v0e4	2.60E-62	1.081773	0.889	0.563	6.37E-58	pDC	Atp6v0e
Nfe2l1	4.43E-62	1.338148	0.399	0.12	1.08E-57	pDC	Nfe2l1
Tbca4	7.35E-62	1.089123	0.804	0.426	1.80E-57	pDC	Tbca
Rps165	9.58E-62	0.742834	0.978	0.882	2.35E-57	pDC	Rps16
Nudt93	1.10E-61	1.972674	0.584	0.264	2.70E-57	pDC	Nudt9
Dnajc10	1.25E-61	1.435275	0.411	0.127	3.06E-57	pDC	Dnajc10
Kbtbd3	1.89E-61	1.961691	0.29	0.072	4.63E-57	pDC	Kbtbd3
Tubb2b	3.98E-61	2.510354	0.307	0.081	9.76E-57	pDC	Tubb2b
Crtc2	5.54E-61	1.595624	0.351	0.099	1.36E-56	pDC	Crtc2
Tap13	6.07E-61	1.20464	0.762	0.389	1.49E-56	pDC	Tap1
Rnf1811	1.31E-60	1.407307	0.438	0.143	3.21E-56	pDC	Rnf181
Ppp4r2	1.56E-60	1.464201	0.465	0.16	3.82E-56	pDC	Ppp4r2
Atp5l6	2.56E-60	0.977342	0.891	0.568	6.26E-56	pDC	Atp5l
Kxd14	5.69E-60	1.385006	0.589	0.244	1.39E-55	pDC	Kxd1
Dek6	5.70E-60	1.368609	0.599	0.253	1.39E-55	pDC	Dek
Rbm35	6.44E-60	1.082239	0.839	0.536	1.58E-55	pDC	Rbm3
Tomm75	8.07E-60	1.218653	0.748	0.386	1.98E-55	pDC	Tomm7
Mrpl544	1.40E-59	1.268103	0.525	0.195	3.42E-55	pDC	Mrpl54
Peak12	1.62E-59	1.195726	0.458	0.15	3.96E-55	pDC	Peak1
Ap1g2	1.66E-59	1.692144	0.267	0.063	4.06E-55	pDC	Ap1g2
Cfl16	1.96E-59	0.871006	0.948	0.764	4.81E-55	pDC	Cfl1
Rps218	2.45E-59	0.790127	0.97	0.817	5.99E-55	pDC	Rps2
Snn2	2.87E-59	1.454279	0.527	0.203	7.03E-55	pDC	Snn

Tuba1b2	3.02E-59	1.358679	0.71	0.368	7.39E-55	pDC	Tuba1b
Arhgap11	3.08E-59	1.487008	0.386	0.118	7.54E-55	pDC	Arhgap1
Rplp25	3.47E-59	0.873671	0.946	0.802	8.51E-55	pDC	Rplp2
Mllt6	3.84E-59	1.648478	0.28	0.068	9.41E-55	pDC	Mllt6
Zfas14	7.65E-59	1.679079	0.502	0.191	1.87E-54	pDC	Zfas1
Alcam2	1.07E-58	1.678175	0.287	0.072	2.61E-54	pDC	Alcam
Traf2	1.64E-58	1.580099	0.295	0.075	4.01E-54	pDC	Traf2
Hmgcr	6.32E-58	1.846121	0.27	0.067	1.55E-53	pDC	Hmgcr
Slc2a6	7.10E-58	1.822521	0.257	0.061	1.74E-53	pDC	Slc2a6
Rpl386	2.28E-57	1.053546	0.923	0.702	5.58E-53	pDC	Rpl38
Ggta11	2.42E-57	1.326429	0.532	0.201	5.92E-53	pDC	Ggta1
Pde6d	2.42E-57	1.396538	0.371	0.112	5.93E-53	pDC	Pde6d
Kdm4a	1.03E-56	1.913567	0.339	0.1	2.51E-52	pDC	Kdm4a
Hsp90ab15	2.55E-56	0.940423	0.933	0.741	6.25E-52	pDC	Hsp90ab1
Rps105	3.99E-56	0.649919	0.975	0.892	9.78E-52	pDC	Rps10
Arhgef402	3.99E-56	1.158467	0.505	0.179	9.78E-52	pDC	Arhgef40
Basp13	5.44E-56	0.966466	0.931	0.687	1.33E-51	pDC	Basp1
Borcs81	1.02E-55	1.615969	0.337	0.099	2.51E-51	pDC	Borcs8
Riok33	1.08E-55	1.222158	0.567	0.228	2.65E-51	pDC	Riok3
Tma73	1.31E-55	1.021871	0.78	0.407	3.21E-51	pDC	Tma7
Sec61b5	1.32E-55	1.180383	0.804	0.464	3.23E-51	pDC	Sec61b
Tpt16	3.06E-55	0.710438	0.97	0.897	7.50E-51	pDC	Tpt1
Sqstm12	4.18E-55	1.213162	0.832	0.514	1.02E-50	pDC	Sqstm1
Apool	6.56E-55	1.470946	0.28	0.072	1.61E-50	pDC	Apool
Mdfic4	1.40E-54	1.524551	0.354	0.108	3.42E-50	pDC	Mdfic
Lmnb12	3.75E-54	1.726112	0.287	0.077	9.18E-50	pDC	Lmnb1
St3gal32	4.82E-54	1.242575	0.532	0.205	1.18E-49	pDC	St3gal3
Ezr4	5.18E-54	1.248035	0.332	0.095	1.27E-49	pDC	Ezr
Stxbp3	7.34E-54	1.360886	0.448	0.156	1.80E-49	pDC	Stxbp3
Nae1	8.95E-54	1.573863	0.334	0.098	2.19E-49	pDC	Nae1
Brk14	1.02E-53	1.094357	0.817	0.486	2.49E-49	pDC	Brk1
Rpl226	1.70E-53	0.864492	0.923	0.748	4.17E-49	pDC	Rpl22
Sik33	1.82E-53	1.452268	0.52	0.202	4.46E-49	pDC	Sik3
Pus10	1.92E-53	1.328058	0.309	0.086	4.70E-49	pDC	Pus10
Tmem176b	3.22E-53	1.113002	0.859	0.628	7.88E-49	pDC	Tmem176b
Prr134	4.69E-53	0.989931	0.691	0.334	1.15E-48	pDC	Prr13
H2afv4	4.73E-53	1.150212	0.584	0.246	1.16E-48	pDC	H2afv
Eif1ax2	6.66E-53	1.485629	0.389	0.13	1.63E-48	pDC	Eif1ax
Rpl366	7.52E-53	0.885551	0.943	0.794	1.84E-48	pDC	Rpl36
Actr35	9.60E-53	0.984075	0.854	0.557	2.35E-48	pDC	Actr3
Tnfaip33	1.36E-52	1.004742	0.53	0.204	3.32E-48	pDC	Tnfaip3
Gtpbp1	1.68E-52	1.233523	0.366	0.113	4.12E-48	pDC	Gtpbp1
Sh3bgrl5	1.70E-52	1.187683	0.406	0.135	4.17E-48	pDC	Sh3bgrl
Ube2i2	2.85E-52	1.054579	0.77	0.423	6.99E-48	pDC	Ube2i

Trafd13	3.27E-52	1.077618	0.735	0.368	8.00E-48	pDC	Trafd1
Rps235	3.44E-52	0.714632	0.968	0.855	8.43E-48	pDC	Rps23
Hspb111	3.61E-52	1.857994	0.285	0.078	8.84E-48	pDC	Hspb11
Rap2a1	6.81E-52	1.239644	0.621	0.285	1.67E-47	pDC	Rap2a
Nlrc53	7.37E-52	1.223066	0.572	0.24	1.80E-47	pDC	Nlrc5
Sugt11	1.64E-51	1.234846	0.522	0.209	4.01E-47	pDC	Sugt1
Psma25	2.08E-51	1.101383	0.71	0.357	5.08E-47	pDC	Psma2
Dnajc82	2.22E-51	1.237782	0.584	0.258	5.44E-47	pDC	Dnajc8
Dazap25	2.66E-51	0.955166	0.804	0.454	6.51E-47	pDC	Dazap2
Nol73	3.13E-51	1.255109	0.587	0.26	7.67E-47	pDC	Nol7
Ghitm2	3.33E-51	1.15556	0.661	0.315	8.15E-47	pDC	Ghitm
Myadm2	3.36E-51	1.276301	0.522	0.21	8.24E-47	pDC	Myadm
Rpl236	3.52E-51	0.646431	0.97	0.882	8.63E-47	pDC	Rpl23
Arf54	4.71E-51	1.058437	0.797	0.482	1.15E-46	pDC	Arf5
Pirb5	6.91E-51	1.237704	0.431	0.153	1.69E-46	pDC	Pirb
Tmem1282	1.16E-50	1.188767	0.619	0.283	2.85E-46	pDC	Tmem128
Tnip1	1.62E-50	1.451637	0.361	0.116	3.96E-46	pDC	Tnip1
Enox22	1.70E-50	1.288313	0.448	0.163	4.17E-46	pDC	Enox2
Ubl56	2.41E-50	0.976167	0.819	0.483	5.90E-46	pDC	Ubl5
Npm14	2.95E-50	0.982745	0.869	0.547	7.23E-46	pDC	Npm1
Sec61g5	5.77E-50	1.094254	0.792	0.435	1.41E-45	pDC	Sec61g
Rps205	7.50E-50	0.722881	0.973	0.881	1.84E-45	pDC	Rps20
Ptcd21	1.30E-49	1.555539	0.3	0.087	3.19E-45	pDC	Ptcd2
Lsm52	1.44E-49	1.349356	0.386	0.13	3.52E-45	pDC	Lsm5
Cox5b6	1.72E-49	0.893655	0.861	0.545	4.22E-45	pDC	Cox5b
Ptma4	1.72E-49	0.8895	0.916	0.727	4.22E-45	pDC	Ptma
Appl1	1.86E-49	1.150393	0.351	0.11	4.57E-45	pDC	Appl1
Neat15	2.58E-49	0.788128	0.656	0.297	6.32E-45	pDC	Neat1
Akt32	2.81E-49	1.123533	0.493	0.184	6.89E-45	pDC	Akt3
Eif3m5	3.08E-49	1.174922	0.616	0.282	7.53E-45	pDC	Eif3m
Usp183	4.10E-49	1.094942	0.342	0.106	1.00E-44	pDC	Usp18
Cyth12	4.42E-49	1.029008	0.443	0.159	1.08E-44	pDC	Cyth1
Wdr14	9.24E-49	1.12075	0.71	0.365	2.26E-44	pDC	Wdr1
Il21r4	9.48E-49	1.150233	0.72	0.377	2.32E-44	pDC	Il21r
Fnbp13	1.10E-48	1.324941	0.72	0.397	2.69E-44	pDC	Fnbp1
Gabarap5	1.77E-48	0.818124	0.921	0.676	4.34E-44	pDC	Gabarap
Gpcpd13	1.83E-48	1.143519	0.582	0.251	4.48E-44	pDC	Gpcpd1
Ptms4	2.36E-48	0.941562	0.827	0.523	5.78E-44	pDC	Ptms
Lpp2	2.89E-48	1.040649	0.634	0.286	7.07E-44	pDC	Lpp
Atp5g25	2.90E-48	0.795145	0.928	0.688	7.09E-44	pDC	Atp5g2
Cdc374	3.14E-48	1.095958	0.671	0.325	7.70E-44	pDC	Cdc37
Mrpl45	3.47E-48	1.530901	0.297	0.087	8.51E-44	pDC	Mrpl45
Jpt14	4.70E-48	1.214009	0.693	0.359	1.15E-43	pDC	Jpt1
Anp32b4	7.55E-48	1.111989	0.621	0.288	1.85E-43	pDC	Anp32b

Csnk2b5	7.69E-48	1.096286	0.676	0.337	1.88E-43	pDC	Csnk2b
Eif4b5	9.32E-48	1.174964	0.614	0.286	2.28E-43	pDC	Eif4b
Eif3e4	1.69E-47	1.089212	0.639	0.306	4.13E-43	pDC	Eif3e
Mpnd1	2.10E-47	1.224997	0.347	0.112	5.13E-43	pDC	Mpnd
Plekhb2	2.43E-47	1.28492	0.396	0.14	5.94E-43	pDC	Plekhb2
Tnfaip85	2.69E-47	1.154673	0.646	0.325	6.60E-43	pDC	Tnfaip8
Eloc2	4.11E-47	1.037954	0.624	0.294	1.01E-42	pDC	Eloc
Hmg20b	4.19E-47	1.213733	0.455	0.174	1.03E-42	pDC	Hmg20b
Sbds1	5.70E-47	1.149949	0.498	0.201	1.40E-42	pDC	Sbds
Mknk22	6.82E-47	1.272101	0.396	0.139	1.67E-42	pDC	Mknk2
Pigyl	8.38E-47	1.224285	0.366	0.122	2.05E-42	pDC	Pigyl
Pgls4	9.03E-47	1.080954	0.708	0.377	2.21E-42	pDC	Pgls
Eps153	9.34E-47	1.064643	0.554	0.235	2.29E-42	pDC	Eps15
Tmpo4	1.61E-46	1.358495	0.438	0.167	3.93E-42	pDC	Tmpo
Pnrc11	1.70E-46	0.923379	0.817	0.498	4.17E-42	pDC	Pnrc1
Ubxn15	2.21E-46	1.008486	0.72	0.387	5.42E-42	pDC	Ubxn1
Banf15	2.80E-46	1.145359	0.589	0.269	6.86E-42	pDC	Banf1
Selenow7	2.92E-46	0.860875	0.817	0.461	7.16E-42	pDC	Selenow
Rbm424	5.36E-46	1.061559	0.579	0.254	1.31E-41	pDC	Rbm42
Calm35	6.18E-46	1.030191	0.671	0.333	1.51E-41	pDC	Calm3
Fam107b3	7.13E-46	1.183132	0.416	0.151	1.75E-41	pDC	Fam107b
Tbcb3	7.31E-46	1.086145	0.582	0.263	1.79E-41	pDC	Tbcb
Cdyl2	7.96E-46	1.306867	0.307	0.092	1.95E-41	pDC	Cdyl2
Gosr21	8.50E-46	1.207378	0.391	0.139	2.08E-41	pDC	Gosr2
Tax1bp32	9.87E-46	1.260186	0.416	0.153	2.42E-41	pDC	Tax1bp3
Vhl	1.07E-45	1.359401	0.327	0.105	2.61E-41	pDC	Vhl
Isy1	2.17E-45	1.496979	0.295	0.089	5.32E-41	pDC	Isy1
Rpl135	2.40E-45	0.581218	0.978	0.92	5.87E-41	pDC	Rpl13
Gnb15	2.46E-45	0.827208	0.804	0.488	6.03E-41	pDC	Gnb1
Csf2rb23	3.76E-45	1.502828	0.399	0.149	9.22E-41	pDC	Csf2rb2
Tctex1d22	4.19E-45	1.597749	0.403	0.153	1.03E-40	pDC	Tctex1d2
Pex13	4.58E-45	1.03381	0.401	0.142	1.12E-40	pDC	Pex13
Paxbp11	5.31E-45	1.142876	0.396	0.14	1.30E-40	pDC	Paxbp1
Tle55	8.00E-45	0.968584	0.735	0.405	1.96E-40	pDC	Tle5
Bloc1s22	8.84E-45	1.169027	0.413	0.152	2.16E-40	pDC	Bloc1s2
Ifitm25	8.91E-45	0.670592	0.517	0.209	2.18E-40	pDC	Ifitm2
Cox7c4	1.11E-44	0.990176	0.834	0.575	2.71E-40	pDC	Cox7c
Rpl304	1.28E-44	0.632924	0.963	0.898	3.14E-40	pDC	Rpl30
Gpr652	1.67E-44	1.620562	0.29	0.089	4.10E-40	pDC	Gpr65
Elob7	2.66E-44	0.88259	0.834	0.525	6.52E-40	pDC	Elob
Gsto13	3.52E-44	1.331434	0.384	0.14	8.61E-40	pDC	Gsto1
Uba525	4.56E-44	1.029186	0.772	0.46	1.12E-39	pDC	Uba52
Polr1d4	5.17E-44	0.984334	0.752	0.423	1.27E-39	pDC	Polr1d
Sem15	5.62E-44	0.786131	0.844	0.552	1.38E-39	pDC	Sem1

Got22	9.68E-44	1.247385	0.384	0.138	2.37E-39	pDC	Got2
Psma55	1.09E-43	1.067949	0.626	0.308	2.67E-39	pDC	Psma5
Rps266	1.48E-43	0.776304	0.943	0.822	3.62E-39	pDC	Rps26
Oaz16	2.41E-43	0.738724	0.918	0.722	5.90E-39	pDC	Oaz1
Pfkp2	4.41E-43	1.396873	0.255	0.072	1.08E-38	pDC	Pfkp
Eef1a14	5.68E-43	0.539898	0.99	0.944	1.39E-38	pDC	Eef1a1
Anxa33	7.33E-43	0.998968	0.849	0.597	1.79E-38	pDC	Anxa3
Eml6	9.26E-43	1.229461	0.411	0.152	2.27E-38	pDC	Eml6
Gbp83	1.18E-42	1.187128	0.334	0.111	2.90E-38	pDC	Gbp8
Mtf1	1.50E-42	1.575118	0.267	0.079	3.68E-38	pDC	Mtf1
Cfdp11	1.73E-42	0.980605	0.485	0.199	4.23E-38	pDC	Cfdp1
Bmt2	2.17E-42	1.221706	0.463	0.187	5.31E-38	pDC	Bmt2
Fxyd54	2.41E-42	0.404495	0.649	0.293	5.91E-38	pDC	Fxyd5
Sumo14	3.53E-42	0.914423	0.653	0.325	8.65E-38	pDC	Sumo1
Gm118085	3.77E-42	0.957579	0.636	0.306	9.22E-38	pDC	Gm11808
Rras4	5.62E-42	1.09909	0.389	0.142	1.38E-37	pDC	Rras
Tubb2a3	5.76E-42	1.307028	0.545	0.256	1.41E-37	pDC	Tubb2a
Gbp41	6.51E-42	1.425844	0.262	0.077	1.59E-37	pDC	Gbp4
Parp4	6.55E-42	1.456459	0.324	0.108	1.60E-37	pDC	Parp4
Nop533	6.80E-42	1.192914	0.46	0.187	1.66E-37	pDC	Nop53
Eif3g3	1.15E-41	1.187024	0.448	0.184	2.81E-37	pDC	Eif3g
Vdac33	1.38E-41	0.943945	0.55	0.245	3.39E-37	pDC	Vdac3
Umad1	1.97E-41	1.243382	0.381	0.14	4.83E-37	pDC	Umad1
Atp5e5	2.83E-41	0.79077	0.896	0.651	6.93E-37	pDC	Atp5e
1110038F1	2.99E-41	1.489846	0.334	0.117	7.31E-37	pDC	1110038F14Rik
Uqcrh6	4.57E-41	0.871061	0.859	0.599	1.12E-36	pDC	Uqcrh
Slc25a35	5.56E-41	0.834708	0.869	0.612	1.36E-36	pDC	Slc25a3
Myh94	5.78E-41	0.925653	0.866	0.586	1.42E-36	pDC	Myh9
Rpl375	6.30E-41	0.683147	0.953	0.852	1.54E-36	pDC	Rpl37
Rgs3	7.78E-41	1.118621	0.287	0.089	1.90E-36	pDC	Rgs3
Ndufa34	7.88E-41	0.883123	0.715	0.38	1.93E-36	pDC	Ndufa3
H2-D13	1.06E-40	0.562299	0.99	0.904	2.60E-36	pDC	H2-D1
Khnyn	1.27E-40	1.097921	0.322	0.105	3.11E-36	pDC	Khnyn
Fancl	1.28E-40	1.210115	0.282	0.087	3.12E-36	pDC	Fancl
Sar1a1	1.38E-40	1.100214	0.46	0.187	3.39E-36	pDC	Sar1a
Cyb5r33	1.49E-40	1.223623	0.384	0.144	3.64E-36	pDC	Cyb5r3
Rbis2	1.57E-40	1.207192	0.52	0.23	3.86E-36	pDC	Rbis
Tyk21	1.88E-40	1.07989	0.384	0.141	4.61E-36	pDC	Tyk2
Rpl265	3.52E-40	0.617829	0.955	0.829	8.63E-36	pDC	Rpl26
Atxn12	3.75E-40	1.615096	0.493	0.222	9.19E-36	pDC	Atxn1
Stat15	5.92E-40	0.831537	0.824	0.497	1.45E-35	pDC	Stat1
Spred12	6.09E-40	1.123031	0.458	0.186	1.49E-35	pDC	Spred1
Nsa25	6.52E-40	0.851103	0.792	0.449	1.60E-35	pDC	Nsa2
Chpt1	7.19E-40	1.24008	0.344	0.122	1.76E-35	pDC	Chpt1

Gga3	1.15E-39	1.220564	0.329	0.113	2.81E-35	pDC	Gga3
Snx204	1.21E-39	1.050328	0.636	0.326	2.97E-35	pDC	Snx20
Mrpl13	1.58E-39	1.568026	0.312	0.107	3.87E-35	pDC	Mrpl13
Eif3c3	2.03E-39	0.942462	0.658	0.34	4.98E-35	pDC	Eif3c
Rad21	3.82E-39	1.09569	0.431	0.174	9.35E-35	pDC	Rad21
Zfp217	5.26E-39	1.116836	0.364	0.132	1.29E-34	pDC	Zfp217
Traf3	6.85E-39	1.241301	0.483	0.206	1.68E-34	pDC	Traf3
Rpl37a6	6.99E-39	0.622333	0.953	0.854	1.71E-34	pDC	Rpl37a
Acadl2	7.03E-39	1.316789	0.443	0.187	1.72E-34	pDC	Acadl
Synj14	7.28E-39	1.06038	0.614	0.299	1.78E-34	pDC	Synj1
Phf6	1.28E-38	1.281354	0.262	0.08	3.14E-34	pDC	Phf6
Sod11	1.38E-38	1.238033	0.339	0.122	3.38E-34	pDC	Sod1
Tomm225	1.40E-38	0.927323	0.641	0.327	3.42E-34	pDC	Tomm22
Fbxo63	1.75E-38	1.325622	0.379	0.146	4.28E-34	pDC	Fbxo6
Rab22a	2.14E-38	0.985347	0.396	0.15	5.24E-34	pDC	Rab22a
Nrbp1	2.58E-38	0.854328	0.468	0.192	6.32E-34	pDC	Nrbp1
3830406C1	3.64E-38	1.43085	0.349	0.128	8.92E-34	pDC	3830406C13Rik
Rbbp72	3.73E-38	1.001652	0.49	0.214	9.13E-34	pDC	Rbbp7
Tmbim42	4.96E-38	1.416411	0.723	0.436	1.21E-33	pDC	Tmbim4
1810037I1	5.19E-38	0.917473	0.658	0.342	1.27E-33	pDC	1810037I17Rik
Dennd5a2	5.89E-38	0.959464	0.582	0.279	1.44E-33	pDC	Dennd5a
Tmem1673	1.09E-37	0.978613	0.569	0.272	2.68E-33	pDC	Tmem167
Mrpl38	1.14E-37	1.418727	0.332	0.121	2.78E-33	pDC	Mrpl38
Map3k12	1.57E-37	1.084726	0.483	0.208	3.84E-33	pDC	Map3k1
Akap133	2.11E-37	0.90386	0.797	0.496	5.17E-33	pDC	Akap13
Aimp13	2.68E-37	1.010067	0.52	0.235	6.56E-33	pDC	Aimp1
Akap9	3.27E-37	1.018564	0.574	0.282	8.00E-33	pDC	Akap9
Rpl326	3.91E-37	0.567631	0.973	0.867	9.57E-33	pDC	Rpl32
Fam104a4	4.38E-37	0.899697	0.517	0.234	1.07E-32	pDC	Fam104a
Uqcrfs15	5.18E-37	0.893868	0.634	0.319	1.27E-32	pDC	Uqcrfs1
H2-T235	1.01E-36	0.601765	0.943	0.714	2.48E-32	pDC	H2-T23
Cyba5	1.03E-36	0.562818	0.953	0.861	2.52E-32	pDC	Cyba
Eif3h5	1.05E-36	0.780128	0.8	0.487	2.57E-32	pDC	Eif3h
Ddt3	1.18E-36	1.613529	0.408	0.17	2.89E-32	pDC	Ddt
Arhgap304	1.46E-36	0.873573	0.639	0.328	3.58E-32	pDC	Arhgap30
Mien15	1.69E-36	0.955978	0.468	0.201	4.14E-32	pDC	Mien1
Snrpd13	1.84E-36	0.950455	0.488	0.213	4.50E-32	pDC	Snrpd1
Washc1	1.97E-36	1.157081	0.342	0.126	4.83E-32	pDC	Washc1
Ube2f2	2.15E-36	0.837689	0.577	0.274	5.26E-32	pDC	Ube2f
Psma44	2.89E-36	0.931229	0.53	0.243	7.07E-32	pDC	Psma4
Spag94	3.54E-36	0.904027	0.609	0.304	8.67E-32	pDC	Spag9
Cat2	4.58E-36	1.069171	0.433	0.183	1.12E-31	pDC	Cat
Slc9a3r14	4.69E-36	1.070477	0.423	0.177	1.15E-31	pDC	Slc9a3r1
Mrps72	4.72E-36	1.170588	0.401	0.165	1.15E-31	pDC	Mrps7

Hnrnpa33	5.71E-36	0.800281	0.765	0.454	1.40E-31	pDC	Hnrnpa3
Aplp24	7.31E-36	1.004254	0.559	0.277	1.79E-31	pDC	Aplp2
Bak13	8.98E-36	1.082394	0.356	0.135	2.20E-31	pDC	Bak1
Ppp1r111	1.17E-35	0.806215	0.554	0.258	2.86E-31	pDC	Ppp1r11
Pcyt1a	1.18E-35	1.709022	0.275	0.094	2.90E-31	pDC	Pcyt1a
Fut8	1.48E-35	1.069455	0.389	0.153	3.63E-31	pDC	Fut8
Mrpl322	1.62E-35	1.193202	0.366	0.142	3.97E-31	pDC	Mrpl32
Zyx4	2.02E-35	0.987812	0.537	0.257	4.94E-31	pDC	Zyx
Ube2n2	2.14E-35	0.808833	0.582	0.279	5.25E-31	pDC	Ube2n
Dnajc23	2.16E-35	1.096802	0.406	0.168	5.28E-31	pDC	Dnajc2
Rpl145	2.69E-35	0.686242	0.906	0.717	6.58E-31	pDC	Rpl14
Snrpg4	2.91E-35	0.917918	0.728	0.43	7.13E-31	pDC	Snrpg
Ilrun1	3.14E-35	0.936176	0.446	0.189	7.70E-31	pDC	Ilrun
Hcfc1r16	6.07E-35	0.948125	0.542	0.266	1.49E-30	pDC	Hcfc1r1
S100a135	6.91E-35	0.986237	0.493	0.227	1.69E-30	pDC	S100a13
Mink11	7.80E-35	1.032379	0.354	0.134	1.91E-30	pDC	Mink1
Ext14	1.27E-34	1.356402	0.483	0.224	3.12E-30	pDC	Ext1
Atp5md5	1.33E-34	0.740905	0.713	0.404	3.27E-30	pDC	Atp5md
Rps36	1.37E-34	0.554305	0.968	0.829	3.35E-30	pDC	Rps3
Psma35	1.54E-34	0.76376	0.738	0.421	3.77E-30	pDC	Psma3
Krit1	1.67E-34	1.021188	0.458	0.198	4.08E-30	pDC	Krit1
Pot1b1	2.58E-34	1.201501	0.379	0.153	6.32E-30	pDC	Pot1b
Ensa2	3.71E-34	1.118801	0.366	0.146	9.09E-30	pDC	Ensa
Rpl155	4.32E-34	0.602889	0.946	0.787	1.06E-29	pDC	Rpl15
Mea14	4.35E-34	0.905535	0.455	0.199	1.07E-29	pDC	Mea1
Bloc1s16	4.62E-34	0.890464	0.498	0.226	1.13E-29	pDC	Bloc1s1
Psmb14	4.81E-34	0.84579	0.708	0.403	1.18E-29	pDC	Psmb1
Tmem1313	5.81E-34	1.132413	0.589	0.307	1.42E-29	pDC	Tmem131
Cox6b16	6.07E-34	0.704507	0.861	0.558	1.49E-29	pDC	Cox6b1
Apaf1	7.04E-34	1.185154	0.287	0.099	1.72E-29	pDC	Apaf1
Arfrp1	7.57E-34	1.082306	0.356	0.139	1.85E-29	pDC	Arfrp1
Mkrn15	7.58E-34	0.792615	0.599	0.3	1.86E-29	pDC	Mkrn1
Psma65	1.06E-33	0.893494	0.557	0.271	2.59E-29	pDC	Psma6
Shb	1.13E-33	0.973645	0.265	0.087	2.77E-29	pDC	Shb
Anxa71	1.29E-33	1.1093	0.366	0.146	3.16E-29	pDC	Anxa7
H2-T225	1.41E-33	0.851142	0.653	0.346	3.46E-29	pDC	H2-T22
Aspscr1	1.57E-33	1.100533	0.351	0.137	3.85E-29	pDC	Aspscr1
Nup98	1.99E-33	0.977142	0.418	0.176	4.87E-29	pDC	Nup98
Elovl51	2.36E-33	1.515162	0.304	0.112	5.77E-29	pDC	Elovl5
Arrdc42	3.04E-33	1.367893	0.267	0.092	7.43E-29	pDC	Arrdc4
Tmem127	4.85E-33	0.812707	0.366	0.142	1.19E-28	pDC	Tmem127
Ahcyl23	6.92E-33	0.973685	0.423	0.181	1.70E-28	pDC	Ahcyl2
Rbbp4	7.25E-33	0.803232	0.572	0.282	1.78E-28	pDC	Rbbp4
Pnpla8	8.82E-33	0.809092	0.428	0.182	2.16E-28	pDC	Pnpla8

Suco	8.94E-33	0.997082	0.339	0.128	2.19E-28	pDC	Suco
Spsb1	1.08E-32	1.006742	0.401	0.168	2.65E-28	pDC	Spsb1
Mcl15	1.29E-32	0.747534	0.807	0.541	3.16E-28	pDC	Mcl1
Tsta3	1.35E-32	1.263762	0.262	0.089	3.31E-28	pDC	Tsta3
Dph5	1.49E-32	1.366732	0.265	0.09	3.65E-28	pDC	Dph5
Nhlrc2	1.52E-32	1.110137	0.386	0.159	3.72E-28	pDC	Nhlrc2
Rps185	1.88E-32	0.61557	0.918	0.761	4.60E-28	pDC	Rps18
Rpl23a5	2.74E-32	0.792665	0.79	0.506	6.72E-28	pDC	Rpl23a
Arl31	2.93E-32	1.005296	0.351	0.139	7.17E-28	pDC	Arl3
Pik3r52	3.48E-32	0.91318	0.515	0.243	8.52E-28	pDC	Pik3r5
Psmd84	3.99E-32	0.735144	0.624	0.318	9.78E-28	pDC	Psmd8
Cstb3	4.77E-32	0.762168	0.683	0.384	1.17E-27	pDC	Cstb
Eva1b1	5.82E-32	1.13614	0.312	0.117	1.43E-27	pDC	Eva1b
Llph4	5.91E-32	0.917681	0.5	0.237	1.45E-27	pDC	Llph
Otud5	7.02E-32	0.828919	0.441	0.191	1.72E-27	pDC	Otud5
Dctn35	8.57E-32	0.82568	0.554	0.277	2.10E-27	pDC	Dctn3
Ier51	1.05E-31	0.540056	0.851	0.545	2.57E-27	pDC	Ier5
Rps3a16	1.29E-31	0.50515	0.975	0.884	3.16E-27	pDC	Rps3a1
Arrdc1	1.43E-31	0.9554	0.426	0.186	3.50E-27	pDC	Arrdc1
Pbxip12	1.48E-31	0.955056	0.517	0.249	3.61E-27	pDC	Pbxip1
Sin3b4	1.53E-31	0.876418	0.545	0.273	3.76E-27	pDC	Sin3b
Kpna3	1.56E-31	0.851204	0.436	0.188	3.82E-27	pDC	Kpna3
Naca6	2.16E-31	0.593442	0.906	0.705	5.29E-27	pDC	Naca
Tmem168	2.27E-31	0.86031	0.371	0.15	5.57E-27	pDC	Tmem168
Ppp1ca4	2.40E-31	0.714742	0.762	0.464	5.87E-27	pDC	Ppp1ca
Eif2s21	2.46E-31	0.85046	0.621	0.339	6.01E-27	pDC	Eif2s2
Fbxo112	2.67E-31	0.744754	0.502	0.235	6.55E-27	pDC	Fbxo11
Pdcd64	2.79E-31	0.833614	0.453	0.206	6.82E-27	pDC	Pdcd6
Atp5h5	3.64E-31	0.684994	0.842	0.553	8.92E-27	pDC	Atp5h
Ola11	3.83E-31	0.916357	0.418	0.184	9.39E-27	pDC	Ola1
Stk19	4.04E-31	0.927004	0.356	0.143	9.90E-27	pDC	Stk19
Cd444	4.54E-31	0.780317	0.443	0.2	1.11E-26	pDC	Cd44
Plin3	5.28E-31	1.178773	0.275	0.098	1.29E-26	pDC	Plin3
Gm100763	6.55E-31	1.21995	0.327	0.129	1.60E-26	pDC	Gm10076
Rpl86	6.94E-31	0.541042	0.943	0.795	1.70E-26	pDC	Rpl8
Prelid16	7.11E-31	0.700804	0.636	0.337	1.74E-26	pDC	Prelid1
Psma12	8.67E-31	0.925924	0.579	0.297	2.12E-26	pDC	Psma1
Vwa5a1	1.30E-30	1.381075	0.421	0.195	3.18E-26	pDC	Vwa5a
Rnf19b2	1.47E-30	0.562141	0.609	0.314	3.61E-26	pDC	Rnf19b
Mdp11	2.47E-30	1.121083	0.374	0.158	6.04E-26	pDC	Mdp1
Ndufb44	2.48E-30	0.790136	0.663	0.365	6.08E-26	pDC	Ndufb4
Rassf23	3.31E-30	0.642293	0.616	0.319	8.11E-26	pDC	Rassf2
Chmp2a5	3.75E-30	0.856901	0.584	0.309	9.19E-26	pDC	Chmp2a
Rhoc1	3.94E-30	1.133461	0.29	0.108	9.66E-26	pDC	Rhoc

Arhgdia5	3.97E-30	0.646062	0.807	0.534	9.72E-26	pDC	Arhgdia
Nudt212	4.56E-30	0.861868	0.406	0.177	1.12E-25	pDC	Nudt21
Vps511	5.63E-30	0.929349	0.386	0.164	1.38E-25	pDC	Vps51
Nelfe2	6.11E-30	1.033848	0.314	0.122	1.50E-25	pDC	Nelfe
Dctn2	6.21E-30	0.764421	0.589	0.306	1.52E-25	pDC	Dctn2
Hnrnpul24	6.84E-30	0.739974	0.493	0.235	1.67E-25	pDC	Hnrnpul2
Pdlim44	6.88E-30	1.348061	0.495	0.263	1.68E-25	pDC	Pdlim4
Pgk16	6.98E-30	0.728426	0.527	0.259	1.71E-25	pDC	Pgk1
Dnajb62	7.06E-30	0.772043	0.597	0.323	1.73E-25	pDC	Dnajb6
Il4ra3	7.16E-30	0.949539	0.493	0.239	1.75E-25	pDC	Il4ra
Litaf3	1.23E-29	0.810857	0.676	0.395	3.02E-25	pDC	Litaf
Arl2bp1	1.32E-29	0.993569	0.252	0.088	3.22E-25	pDC	Arl2bp
Atp6v1f5	1.62E-29	0.655991	0.832	0.559	3.98E-25	pDC	Atp6v1f
Fis16	1.70E-29	0.675962	0.767	0.469	4.16E-25	pDC	Fis1
Arf63	1.74E-29	0.946307	0.52	0.268	4.26E-25	pDC	Arf6
Map3k8	1.81E-29	1.275766	0.327	0.133	4.43E-25	pDC	Map3k8
Laptm4b	1.90E-29	2.121414	0.302	0.125	4.66E-25	pDC	Laptm4b
Ube2d34	1.96E-29	0.676177	0.812	0.528	4.80E-25	pDC	Ube2d3
Mrpl305	2.55E-29	0.90616	0.547	0.284	6.25E-25	pDC	Mrpl30
Slc38a22	2.72E-29	0.887879	0.53	0.264	6.67E-25	pDC	Slc38a2
Vbp1	2.89E-29	1.104703	0.277	0.101	7.07E-25	pDC	Vbp1
Cript4	3.23E-29	0.759127	0.421	0.186	7.91E-25	pDC	Cript
Ciao2a4	3.59E-29	0.74241	0.512	0.251	8.79E-25	pDC	Ciao2a
Rnf1143	3.85E-29	0.851282	0.485	0.234	9.42E-25	pDC	Rnf114
Ndufv25	4.52E-29	0.700485	0.577	0.295	1.11E-24	pDC	Ndufv2
Trim255	4.93E-29	1.067498	0.389	0.17	1.21E-24	pDC	Trim25
Cd526	4.96E-29	0.687336	0.899	0.674	1.21E-24	pDC	Cd52
Pcbp23	5.16E-29	0.753049	0.629	0.347	1.26E-24	pDC	Pcbp2
Nedd84	5.45E-29	0.712817	0.735	0.452	1.33E-24	pDC	Nedd8
Nxf1	7.18E-29	0.793619	0.403	0.175	1.76E-24	pDC	Nxf1
Ell2	8.71E-29	1.1337	0.265	0.096	2.13E-24	pDC	Ell2
Hpcal17	8.87E-29	0.781035	0.517	0.262	2.17E-24	pDC	Hpcal1
Alyref4	9.12E-29	0.804736	0.453	0.21	2.23E-24	pDC	Alyref
Snrnp272	9.92E-29	0.857275	0.376	0.161	2.43E-24	pDC	Snrnp27
Idnk3	1.12E-28	0.906451	0.463	0.22	2.75E-24	pDC	Idnk
Rab102	1.16E-28	0.867448	0.631	0.359	2.84E-24	pDC	Rab10
Ube2v16	1.28E-28	0.709665	0.564	0.285	3.14E-24	pDC	Ube2v1
Rpl295	1.28E-28	0.505509	0.926	0.801	3.14E-24	pDC	Rpl29
Irf82	1.31E-28	1.245085	0.775	0.605	3.20E-24	pDC	Irf8
Psmb35	1.42E-28	0.660732	0.708	0.401	3.47E-24	pDC	Psmb3
Grb23	2.19E-28	0.645467	0.795	0.516	5.37E-24	pDC	Grb2
Ramac1	2.28E-28	0.900174	0.366	0.154	5.58E-24	pDC	Ramac
Pdcd102	2.44E-28	0.732646	0.537	0.269	5.98E-24	pDC	Pdcd10
Atp5b5	2.55E-28	0.642512	0.827	0.536	6.23E-24	pDC	Atp5b

Herc62	2.56E-28	0.885493	0.381	0.165	6.28E-24	pDC	Herc6
Tax1bp14	2.78E-28	0.652275	0.641	0.352	6.81E-24	pDC	Tax1bp1
Polr2m1	3.65E-28	0.875806	0.344	0.142	8.94E-24	pDC	Polr2m
Cct43	3.69E-28	0.727921	0.587	0.308	9.04E-24	pDC	Cct4
Oat1	4.06E-28	0.915791	0.314	0.124	9.94E-24	pDC	Oat
St3gal43	4.75E-28	0.924972	0.319	0.126	1.16E-23	pDC	St3gal4
Smarcb11	7.61E-28	0.977534	0.356	0.153	1.86E-23	pDC	Smarcb1
Arpc24	9.16E-28	0.556322	0.911	0.708	2.24E-23	pDC	Arpc2
Ncoa22	1.05E-27	0.800529	0.562	0.287	2.58E-23	pDC	Ncoa2
1810058I2	1.56E-27	0.77358	0.522	0.263	3.82E-23	pDC	1810058I24Rik
Sh3bp13	1.58E-27	0.801236	0.572	0.312	3.86E-23	pDC	Sh3bp1
Supt6	1.60E-27	0.940022	0.384	0.17	3.91E-23	pDC	Supt6
Atp5d6	1.61E-27	0.62674	0.809	0.529	3.95E-23	pDC	Atp5d
Mrpl9	1.91E-27	0.950112	0.302	0.12	4.67E-23	pDC	Mrpl9
Sdhaf2	2.74E-27	1.170467	0.257	0.095	6.70E-23	pDC	Sdhaf2
Rela1	3.05E-27	0.6906	0.47	0.225	7.47E-23	pDC	Rela
Vcp2	3.08E-27	0.826384	0.649	0.388	7.54E-23	pDC	Vcp
Myo1c2	3.10E-27	0.899605	0.354	0.15	7.59E-23	pDC	Myo1c
Krcc1	3.27E-27	0.737798	0.693	0.403	8.01E-23	pDC	Krcc1
H2-DMb22	4.01E-27	2.132703	0.25	0.097	9.81E-23	pDC	H2-DMb2
Zfp8002	5.80E-27	0.851344	0.324	0.131	1.42E-22	pDC	Zfp800
Psmd43	5.96E-27	0.68837	0.51	0.259	1.46E-22	pDC	Psmd4
Ube2l33	6.31E-27	0.76837	0.562	0.305	1.54E-22	pDC	Ube2l3
Fam241a3	6.51E-27	0.891317	0.255	0.093	1.59E-22	pDC	Fam241a
Pet1002	6.91E-27	0.908289	0.364	0.159	1.69E-22	pDC	Pet100
Raly4	1.06E-26	0.748914	0.562	0.294	2.59E-22	pDC	Raly
Rbms16	1.10E-26	0.567289	0.48	0.229	2.70E-22	pDC	Rbms1
Ube2z	1.12E-26	0.793494	0.349	0.148	2.75E-22	pDC	Ube2z
Nfat52	1.18E-26	0.708512	0.502	0.245	2.88E-22	pDC	Nfat5
Tcp13	1.31E-26	0.90006	0.493	0.251	3.20E-22	pDC	Tcp1
G3bp11	1.32E-26	0.788149	0.507	0.256	3.23E-22	pDC	G3bp1
Ak24	1.32E-26	0.881242	0.52	0.274	3.24E-22	pDC	Ak2
Suclg11	1.33E-26	0.798644	0.381	0.167	3.25E-22	pDC	Suclg1
Sbno22	1.35E-26	0.693759	0.426	0.197	3.32E-22	pDC	Sbno2
Coro1b4	1.68E-26	0.736908	0.74	0.471	4.11E-22	pDC	Coro1b
Icosl2	1.68E-26	1.284583	0.371	0.169	4.11E-22	pDC	Icosl
Mthfs1	1.78E-26	0.734121	0.537	0.277	4.35E-22	pDC	Mthfs
Bcat21	1.82E-26	1.052415	0.29	0.115	4.45E-22	pDC	Bcat2
Etf11	2.05E-26	0.749527	0.468	0.229	5.01E-22	pDC	Etf1
Cmtr11	2.11E-26	1.013715	0.347	0.15	5.17E-22	pDC	Cmtr1
Sap183	2.13E-26	0.709817	0.554	0.291	5.21E-22	pDC	Sap18
Dusp18	2.18E-26	0.361084	0.705	0.385	5.35E-22	pDC	Dusp1
Tap23	2.25E-26	0.763115	0.661	0.389	5.51E-22	pDC	Tap2
Kmt5a1	2.65E-26	1.14293	0.282	0.111	6.50E-22	pDC	Kmt5a

Nek6	2.76E-26	0.814457	0.455	0.22	6.76E-22	pDC	Nek6
Rps219	2.79E-26	0.571479	0.948	0.834	6.84E-22	pDC	Rps21
Cdc424	2.96E-26	0.568469	0.881	0.731	7.25E-22	pDC	Cdc42
Ccdc502	3.29E-26	0.696163	0.649	0.378	8.06E-22	pDC	Ccdc50
Ppp1cb2	3.50E-26	0.680433	0.411	0.186	8.58E-22	pDC	Ppp1cb
Eef1b26	3.88E-26	0.602457	0.894	0.68	9.50E-22	pDC	Eef1b2
Mrps163	4.02E-26	0.931814	0.426	0.203	9.84E-22	pDC	Mrps16
Ankib1	4.82E-26	0.825462	0.307	0.123	1.18E-21	pDC	Ankib1
Rps65	5.54E-26	0.543829	0.911	0.688	1.36E-21	pDC	Rps6
Dnm1l	5.56E-26	0.956193	0.285	0.112	1.36E-21	pDC	Dnm1l
Psmc43	5.89E-26	0.826569	0.426	0.203	1.44E-21	pDC	Psmc4
Paf1	7.03E-26	1.015317	0.295	0.119	1.72E-21	pDC	Paf1
Mrpl403	1.15E-25	0.953143	0.307	0.126	2.81E-21	pDC	Mrpl40
Coq10b2	1.16E-25	0.917589	0.317	0.132	2.84E-21	pDC	Coq10b
Glipr13	1.23E-25	1.203168	0.282	0.114	3.02E-21	pDC	Glipr1
Rpl355	1.24E-25	0.537078	0.918	0.776	3.03E-21	pDC	Rpl35
Vrk31	1.94E-25	0.798118	0.342	0.146	4.74E-21	pDC	Vrk3
Psmb45	2.15E-25	0.600878	0.552	0.282	5.26E-21	pDC	Psmb4
Zbtb18	2.39E-25	1.0245	0.267	0.102	5.85E-21	pDC	Zbtb18
Zmat23	2.82E-25	0.778487	0.458	0.229	6.91E-21	pDC	Zmat2
Cox6c6	2.88E-25	0.560905	0.822	0.579	7.05E-21	pDC	Cox6c
Lsm32	4.10E-25	0.874377	0.339	0.147	1.01E-20	pDC	Lsm3
Nampt4	4.26E-25	1.092593	0.408	0.198	1.04E-20	pDC	Nampt
Psmd71	4.76E-25	0.727139	0.483	0.242	1.16E-20	pDC	Psmd7
Tpr4	4.76E-25	0.682553	0.624	0.355	1.16E-20	pDC	Tpr
Gng55	4.86E-25	0.499913	0.926	0.72	1.19E-20	pDC	Gng5
Tprgl2	5.17E-25	0.754653	0.507	0.263	1.27E-20	pDC	Tprgl
Smim145	6.30E-25	0.612285	0.567	0.306	1.54E-20	pDC	Smim14
Mxi11	7.58E-25	1.043535	0.391	0.186	1.86E-20	pDC	Mxi1
Ahnak4	7.76E-25	0.530577	0.282	0.108	1.90E-20	pDC	Ahnak
Ccdc82	7.84E-25	0.865506	0.277	0.109	1.92E-20	pDC	Ccdc82
Mvb12a3	9.24E-25	0.834852	0.421	0.203	2.26E-20	pDC	Mvb12a
Cep3501	1.05E-24	0.744254	0.507	0.262	2.57E-20	pDC	Cep350
Psmd10	1.18E-24	1.065908	0.267	0.105	2.89E-20	pDC	Psmd10
Ran4	1.47E-24	0.581955	0.636	0.356	3.61E-20	pDC	Ran
Lsm122	1.72E-24	0.706344	0.433	0.21	4.22E-20	pDC	Lsm12
Gtf2a1	2.30E-24	0.783537	0.314	0.131	5.64E-20	pDC	Gtf2a1
Cd633	2.43E-24	0.632249	0.839	0.624	5.95E-20	pDC	Cd63
Hnrnpa13	2.59E-24	0.785356	0.639	0.386	6.33E-20	pDC	Hnrnpa1
Trim27	2.67E-24	1.1268	0.344	0.155	6.54E-20	pDC	Trim27
Ngdn2	3.31E-24	0.838634	0.337	0.145	8.09E-20	pDC	Ngdn
Cops2	3.38E-24	0.759805	0.369	0.167	8.28E-20	pDC	Cops2
Ssu724	3.42E-24	0.623324	0.644	0.367	8.37E-20	pDC	Ssu72
Nr1h21	3.45E-24	0.859825	0.406	0.194	8.44E-20	pDC	Nr1h2

Rest	3.46E-24	1.071137	0.285	0.116	8.47E-20	pDC	Rest
Ndufb1-ps4	3.99E-24	0.636888	0.559	0.3	9.77E-20	pDC	Ndufb1-ps
Stx82	4.22E-24	0.591684	0.537	0.279	1.03E-19	pDC	Stx8
S100a103	4.45E-24	1.094174	0.265	0.103	1.09E-19	pDC	S100a10
Gpx45	4.78E-24	0.638347	0.832	0.597	1.17E-19	pDC	Gpx4
Txndc92	4.80E-24	0.777945	0.408	0.197	1.18E-19	pDC	Txndc9
Isg153	5.08E-24	1.170259	0.408	0.204	1.24E-19	pDC	Isg15
Txnl12	5.09E-24	0.821835	0.478	0.25	1.25E-19	pDC	Txnl1
Meaf61	5.35E-24	0.682115	0.359	0.16	1.31E-19	pDC	Meaf6
Atxn7l12	6.07E-24	0.528864	0.535	0.276	1.49E-19	pDC	Atxn7l1
Ntan13	6.64E-24	0.650213	0.396	0.184	1.63E-19	pDC	Ntan1
Tor1aip21	8.64E-24	0.893725	0.532	0.291	2.11E-19	pDC	Tor1aip2
Stk243	9.21E-24	0.715694	0.441	0.216	2.26E-19	pDC	Stk24
Mapkapk32	1.11E-23	0.819586	0.29	0.119	2.72E-19	pDC	Mapkapk3
Psmb72	1.25E-23	0.798613	0.386	0.181	3.07E-19	pDC	Psmb7
Prdx25	1.35E-23	0.629808	0.619	0.352	3.30E-19	pDC	Prdx2
Tgif12	1.42E-23	1.05545	0.361	0.169	3.47E-19	pDC	Tgif1
Suds3	1.48E-23	0.755864	0.312	0.133	3.62E-19	pDC	Suds3
Ankrd123	1.54E-23	0.483341	0.502	0.252	3.78E-19	pDC	Ankrd12
Plekhm22	2.02E-23	0.709632	0.527	0.276	4.94E-19	pDC	Plekhm2
Pabpn11	2.88E-23	0.628765	0.616	0.351	7.05E-19	pDC	Pabpn1
Pik3r11	3.18E-23	0.812889	0.725	0.493	7.78E-19	pDC	Pik3r1
Rbx14	3.21E-23	0.598822	0.738	0.465	7.86E-19	pDC	Rbx1
Cdkn1b2	3.30E-23	0.749778	0.322	0.137	8.07E-19	pDC	Cdkn1b
Lsm82	3.43E-23	0.613565	0.364	0.164	8.39E-19	pDC	Lsm8
Atpif12	4.08E-23	0.602117	0.8	0.56	1.00E-18	pDC	Atpif1
Cmc13	4.24E-23	0.725168	0.436	0.218	1.04E-18	pDC	Cmc1
Ybx32	5.34E-23	0.830847	0.287	0.119	1.31E-18	pDC	Ybx3
Setx	5.73E-23	0.738932	0.295	0.122	1.40E-18	pDC	Setx
Rps27a5	6.29E-23	0.385852	0.978	0.908	1.54E-18	pDC	Rps27a
Snrpb22	6.40E-23	0.898926	0.349	0.16	1.57E-18	pDC	Snrpb2
Cdc343	6.43E-23	0.870882	0.324	0.143	1.57E-18	pDC	Cdc34
Eif3k4	7.72E-23	0.536436	0.775	0.486	1.89E-18	pDC	Eif3k
Smurf1	8.91E-23	0.604677	0.359	0.162	2.18E-18	pDC	Smurf1
Cdc42se14	1.03E-22	0.667855	0.525	0.283	2.53E-18	pDC	Cdc42se1
2310011J03	1.29E-22	0.69577	0.322	0.142	3.16E-18	pDC	2310011J03Rik
Clasp1	1.54E-22	0.717395	0.295	0.123	3.77E-18	pDC	Clasp1
Ube2s6	1.60E-22	0.684173	0.493	0.256	3.91E-18	pDC	Ube2s
Rpl214	2.05E-22	0.378089	0.97	0.892	5.02E-18	pDC	Rpl21
Ctdnep13	2.24E-22	0.715345	0.433	0.219	5.49E-18	pDC	Ctdnep1
Ttc9c	2.26E-22	0.789095	0.277	0.114	5.52E-18	pDC	Ttc9c
Eif4e21	2.42E-22	0.607336	0.5	0.258	5.93E-18	pDC	Eif4e2
Set3	2.50E-22	0.510921	0.587	0.32	6.12E-18	pDC	Set
Esd5	2.89E-22	0.552951	0.587	0.327	7.07E-18	pDC	Esd

Uqcrc21	3.15E-22	0.708461	0.507	0.271	7.71E-18	pDC	Uqcrc2
Rbm8a2	3.49E-22	0.562147	0.399	0.191	8.55E-18	pDC	Rbm8a
Esyt2	3.56E-22	0.845448	0.265	0.107	8.71E-18	pDC	Esyt2
Mbd26	3.94E-22	0.534381	0.49	0.255	9.65E-18	pDC	Mbd2
Necap24	4.62E-22	0.703909	0.53	0.295	1.13E-17	pDC	Necap2
Rnh15	4.95E-22	0.633097	0.616	0.35	1.21E-17	pDC	Rnh1
Gdi13	6.71E-22	0.587714	0.473	0.243	1.64E-17	pDC	Gdi1
Snrpd25	7.50E-22	0.635656	0.498	0.265	1.84E-17	pDC	Snrpd2
Cox8a6	8.12E-22	0.425625	0.953	0.784	1.99E-17	pDC	Cox8a
Coa53	8.18E-22	0.89945	0.324	0.149	2.00E-17	pDC	Coa5
Ppp4c4	8.22E-22	0.523246	0.609	0.342	2.01E-17	pDC	Ppp4c
Rpl75	8.38E-22	0.435368	0.918	0.736	2.05E-17	pDC	Rpl7
Vti1b1	9.47E-22	0.552347	0.54	0.293	2.32E-17	pDC	Vti1b
Mpc12	9.76E-22	0.618616	0.752	0.482	2.39E-17	pDC	Mpc1
Ehbp1l13	1.07E-21	0.628392	0.406	0.201	2.62E-17	pDC	Ehbp1l1
Hnrnpf3	1.08E-21	0.58329	0.787	0.513	2.66E-17	pDC	Hnrnpf
Ubl4a1	1.31E-21	0.844987	0.257	0.106	3.21E-17	pDC	Ubl4a
Chst11	1.40E-21	0.680369	0.3	0.127	3.42E-17	pDC	Chst11
Malat12	1.41E-21	0.623964	0.993	0.978	3.45E-17	pDC	Malat1
Plekhj13	1.43E-21	0.681567	0.545	0.298	3.49E-17	pDC	Plekhj1
Mtmr141	1.50E-21	0.6706	0.27	0.111	3.68E-17	pDC	Mtmr14
Rad50	1.52E-21	0.899945	0.275	0.116	3.73E-17	pDC	Rad50
Zfp6221	2.03E-21	0.810456	0.292	0.127	4.98E-17	pDC	Zfp622
Serbp14	2.07E-21	0.61263	0.661	0.407	5.07E-17	pDC	Serbp1
Ccdc1242	2.76E-21	0.709684	0.384	0.185	6.76E-17	pDC	Ccdc124
Il18bp4	2.76E-21	0.774064	0.332	0.15	6.76E-17	pDC	Il18bp
Bax4	3.11E-21	0.627082	0.626	0.364	7.62E-17	pDC	Bax
Ddx6	3.95E-21	0.639458	0.557	0.313	9.68E-17	pDC	Ddx6
Idh1	4.20E-21	0.674149	0.28	0.119	1.03E-16	pDC	Idh1
Snrpc4	4.43E-21	0.565135	0.458	0.236	1.09E-16	pDC	Snrpc
Dnajc154	4.68E-21	0.662221	0.406	0.204	1.15E-16	pDC	Dnajc15
Sra14	5.45E-21	0.626232	0.401	0.199	1.34E-16	pDC	Sra1
U2af13	5.81E-21	0.545326	0.53	0.285	1.42E-16	pDC	U2af1
Lipe1	5.88E-21	0.833826	0.304	0.134	1.44E-16	pDC	Lipe
Eef1g4	6.09E-21	0.558998	0.646	0.384	1.49E-16	pDC	Eef1g
Agpat1	6.63E-21	0.813721	0.295	0.13	1.62E-16	pDC	Agpat1
Phyh	6.84E-21	0.808873	0.359	0.175	1.68E-16	pDC	Phyh
Hsd3b7	7.24E-21	0.954459	0.317	0.146	1.77E-16	pDC	Hsd3b7
Lgals14	7.31E-21	0.265779	0.361	0.165	1.79E-16	pDC	Lgals1
Nipsnap3b:	7.35E-21	0.69891	0.314	0.141	1.80E-16	pDC	Nipsnap3b
Psmd141	7.38E-21	0.515241	0.418	0.207	1.81E-16	pDC	Psmd14
Slirp4	8.25E-21	0.781438	0.347	0.164	2.02E-16	pDC	Slirp
Rpl65	9.46E-21	0.406132	0.97	0.829	2.32E-16	pDC	Rpl6
Cct74	9.54E-21	0.496918	0.498	0.262	2.34E-16	pDC	Cct7

Ndufa115	9.84E-21	0.505507	0.611	0.352	2.41E-16	pDC	Ndufa11
Smndc1	1.16E-20	0.60812	0.458	0.24	2.85E-16	pDC	Smndc1
Phf21a2	1.34E-20	0.657499	0.614	0.36	3.28E-16	pDC	Phf21a
Nras	1.49E-20	0.742036	0.307	0.138	3.64E-16	pDC	Nras
Becn1	1.59E-20	0.618658	0.391	0.191	3.90E-16	pDC	Becn1
Cast4	1.85E-20	0.613215	0.275	0.117	4.54E-16	pDC	Cast
Atp5k3	2.05E-20	0.518393	0.423	0.213	5.03E-16	pDC	Atp5k
Fryl1	2.24E-20	0.666136	0.423	0.216	5.47E-16	pDC	Fryl
Ubl72	2.38E-20	0.57876	0.317	0.143	5.83E-16	pDC	Ubl7
Tspan134	2.57E-20	0.634147	0.611	0.366	6.29E-16	pDC	Tspan13
Sptan12	2.59E-20	0.531027	0.349	0.163	6.35E-16	pDC	Sptan1
Smchd12	2.63E-20	0.845024	0.47	0.259	6.45E-16	pDC	Smchd1
Fam98c1	3.20E-20	0.776638	0.27	0.114	7.84E-16	pDC	Fam98c
Cyb5r42	3.21E-20	0.576912	0.332	0.152	7.85E-16	pDC	Cyb5r4
Vps292	3.72E-20	0.457294	0.577	0.317	9.11E-16	pDC	Vps29
Eif62	3.73E-20	0.585416	0.426	0.216	9.13E-16	pDC	Eif6
Swi55	4.21E-20	0.507391	0.658	0.38	1.03E-15	pDC	Swi5
Galnt7	5.19E-20	0.742092	0.364	0.177	1.27E-15	pDC	Galnt7
Dda11	5.21E-20	0.794025	0.329	0.155	1.28E-15	pDC	Dda1
Smpdl3a3	6.43E-20	0.66731	0.319	0.148	1.58E-15	pDC	Smpdl3a
Tcea12	6.71E-20	0.413105	0.53	0.287	1.64E-15	pDC	Tcea1
Lypla22	6.91E-20	0.698517	0.371	0.183	1.69E-15	pDC	Lypla2
Cycs3	6.96E-20	0.531104	0.55	0.305	1.70E-15	pDC	Cycs
Eapp2	7.00E-20	0.776607	0.332	0.155	1.71E-15	pDC	Eapp
Cggbp12	7.64E-20	0.501301	0.386	0.187	1.87E-15	pDC	Cggbp1
Rab433	7.79E-20	0.574069	0.557	0.316	1.91E-15	pDC	Rab43
Dusp221	8.39E-20	0.759663	0.28	0.122	2.05E-15	pDC	Dusp22
Baz1a4	1.04E-19	0.5181	0.431	0.222	2.56E-15	pDC	Baz1a
Aurkaip17	1.06E-19	0.508311	0.49	0.263	2.59E-15	pDC	Aurkaip1
Zc3h151	1.37E-19	0.670378	0.384	0.192	3.36E-15	pDC	Zc3h15
Prelid3b3	1.42E-19	0.57039	0.324	0.15	3.47E-15	pDC	Prelid3b
Arpc1a2	1.53E-19	0.558298	0.515	0.285	3.75E-15	pDC	Arpc1a
Pdlim53	1.64E-19	0.70024	0.403	0.202	4.01E-15	pDC	Pdlim5
Gatad11	2.00E-19	0.841415	0.28	0.126	4.89E-15	pDC	Gatad1
Use13	2.02E-19	0.610093	0.478	0.257	4.96E-15	pDC	Use1
Psmd11	2.25E-19	0.698721	0.421	0.222	5.50E-15	pDC	Psmd11
Ap2s16	2.40E-19	0.480942	0.54	0.3	5.87E-15	pDC	Ap2s1
Sbno1	2.74E-19	0.46751	0.433	0.224	6.70E-15	pDC	Sbno1
Map1lc3b5	2.77E-19	0.483902	0.589	0.346	6.79E-15	pDC	Map1lc3b
Pank2	2.86E-19	0.612396	0.329	0.153	7.01E-15	pDC	Pank2
Fbxw112	3.21E-19	0.32646	0.473	0.244	7.85E-15	pDC	Fbxw11
Zdhhc3	3.41E-19	0.617725	0.292	0.132	8.35E-15	pDC	Zdhhc3
Cct23	3.97E-19	0.625066	0.527	0.3	9.71E-15	pDC	Cct2
Rack15	4.24E-19	0.426295	0.881	0.659	1.04E-14	pDC	Rack1

Nab13	4.35E-19	0.737637	0.292	0.133	1.06E-14	pDC	Nab1
Kdm6a1	4.38E-19	0.511037	0.344	0.164	1.07E-14	pDC	Kdm6a
Mob1a3	4.49E-19	0.688088	0.431	0.231	1.10E-14	pDC	Mob1a
Ginm1	4.66E-19	0.664013	0.309	0.142	1.14E-14	pDC	Ginm1
Polr2g4	5.93E-19	0.582425	0.433	0.228	1.45E-14	pDC	Polr2g
Churc13	6.71E-19	0.821069	0.337	0.165	1.64E-14	pDC	Churc1
Slc25a111	6.96E-19	0.56805	0.512	0.287	1.70E-14	pDC	Slc25a11
Mrps231	7.27E-19	0.934044	0.272	0.123	1.78E-14	pDC	Mrps23
Sfr13	7.71E-19	0.455547	0.478	0.261	1.89E-14	pDC	Sfr1
Herpud14	8.05E-19	0.70802	0.614	0.386	1.97E-14	pDC	Herpud1
Brms1	8.23E-19	0.710404	0.317	0.149	2.01E-14	pDC	Brms1
Nmi3	8.30E-19	0.6717	0.337	0.162	2.03E-14	pDC	Nmi
Cdc5l1	9.26E-19	0.670796	0.324	0.155	2.27E-14	pDC	Cdc5l
Wasl1	9.34E-19	0.525776	0.416	0.212	2.29E-14	pDC	Wasl
Bzw11	9.37E-19	0.501708	0.5	0.273	2.29E-14	pDC	Bzw1
Dapp12	9.42E-19	0.457659	0.619	0.361	2.31E-14	pDC	Dapp1
Stx121	9.69E-19	0.746424	0.379	0.194	2.37E-14	pDC	Stx12
Atf41	1.07E-18	0.589102	0.411	0.212	2.62E-14	pDC	Atf4
Cox4i17	1.49E-18	0.361538	0.955	0.804	3.65E-14	pDC	Cox4i1
Bsdc1	1.61E-18	0.798344	0.26	0.113	3.95E-14	pDC	Bsdc1
H2afy3	1.78E-18	0.462694	0.621	0.378	4.36E-14	pDC	H2afy
Ptges32	1.80E-18	0.545851	0.525	0.299	4.40E-14	pDC	Ptges3
Cwc154	1.96E-18	0.496401	0.552	0.311	4.80E-14	pDC	Cwc15
Bbx2	2.14E-18	0.72291	0.371	0.189	5.24E-14	pDC	Bbx
Mapre1	2.44E-18	0.559039	0.562	0.326	5.96E-14	pDC	Mapre1
Erg281	2.59E-18	0.688525	0.29	0.133	6.33E-14	pDC	Erg28
Kif2a	3.18E-18	0.751692	0.26	0.115	7.78E-14	pDC	Kif2a
Chchd51	3.21E-18	0.811748	0.255	0.112	7.87E-14	pDC	Chchd5
Ufm13	3.54E-18	0.605764	0.307	0.146	8.68E-14	pDC	Ufm1
Filip1l5	3.55E-18	0.405558	0.735	0.48	8.70E-14	pDC	Filip1l
Cflar1	4.24E-18	0.45976	0.681	0.435	1.04E-13	pDC	Cflar
Chmp1a1	4.41E-18	0.585917	0.329	0.159	1.08E-13	pDC	Chmp1a
Gtf2b1	4.43E-18	0.531632	0.314	0.147	1.09E-13	pDC	Gtf2b
Bmp2k2	4.48E-18	0.597613	0.834	0.63	1.10E-13	pDC	Bmp2k
Pomp6	4.77E-18	0.484618	0.787	0.551	1.17E-13	pDC	Pomp
Uba71	4.98E-18	0.621723	0.423	0.223	1.22E-13	pDC	Uba7
H2-Aa6	5.03E-18	0.519734	0.851	0.615	1.23E-13	pDC	H2-Aa
Nsmce4a2	5.12E-18	0.684701	0.337	0.165	1.25E-13	pDC	Nsmce4a
Med285	5.18E-18	0.436656	0.609	0.359	1.27E-13	pDC	Med28
Atp5c14	5.44E-18	0.583507	0.738	0.505	1.33E-13	pDC	Atp5c1
Stxbp24	5.54E-18	0.57047	0.354	0.175	1.36E-13	pDC	Stxbp2
M6pr4	5.60E-18	0.56014	0.735	0.464	1.37E-13	pDC	M6pr
Uqcrq5	5.92E-18	0.394864	0.703	0.439	1.45E-13	pDC	Uqcrq
Psmb54	6.45E-18	0.488968	0.572	0.34	1.58E-13	pDC	Psmb5

Yy12	6.62E-18	0.459722	0.443	0.236	1.62E-13	pDC	Yy1
Cnbp2	6.65E-18	0.508527	0.755	0.522	1.63E-13	pDC	Cnbp
Eif3a5	7.03E-18	0.567725	0.572	0.343	1.72E-13	pDC	Eif3a
Nono3	7.74E-18	0.52223	0.515	0.29	1.90E-13	pDC	Nono
Cirbp2	8.09E-18	0.532798	0.51	0.289	1.98E-13	pDC	Cirbp
Gclm3	8.29E-18	0.270327	0.391	0.2	2.03E-13	pDC	Gclm
Frg11	8.57E-18	0.562272	0.381	0.192	2.10E-13	pDC	Frg1
Cox7a27	8.70E-18	0.447397	0.73	0.462	2.13E-13	pDC	Cox7a2
Elp51	9.09E-18	0.7371	0.28	0.13	2.23E-13	pDC	Elp5
Fkbp82	9.34E-18	0.417754	0.559	0.324	2.29E-13	pDC	Fkbp8
Wars	9.37E-18	0.857799	0.287	0.134	2.29E-13	pDC	Wars
Mta33	1.28E-17	0.513876	0.339	0.166	3.12E-13	pDC	Mta3
Lsm11	1.35E-17	0.662278	0.27	0.123	3.30E-13	pDC	Lsm1
Mrpl113	1.41E-17	0.598141	0.327	0.159	3.46E-13	pDC	Mrpl11
Grpel12	1.55E-17	0.659416	0.369	0.187	3.79E-13	pDC	Grpel1
Mrpl241	1.58E-17	0.618584	0.403	0.216	3.86E-13	pDC	Mrpl24
Nudt142	1.58E-17	0.724655	0.262	0.118	3.88E-13	pDC	Nudt14
Snf83	1.61E-17	0.530339	0.423	0.227	3.94E-13	pDC	Snf8
Eif4a22	1.69E-17	0.552093	0.579	0.351	4.13E-13	pDC	Eif4a2
Pin12	1.81E-17	0.449905	0.319	0.152	4.43E-13	pDC	Pin1
Eid13	1.81E-17	0.530469	0.324	0.157	4.43E-13	pDC	Eid1
Rpl13a5	1.87E-17	0.378777	0.953	0.793	4.58E-13	pDC	Rpl13a
Plekha2	2.33E-17	0.51114	0.366	0.184	5.71E-13	pDC	Plekha2
Slc25a4	2.49E-17	0.904541	0.334	0.17	6.09E-13	pDC	Slc25a4
Sdc43	2.54E-17	0.490706	0.292	0.136	6.22E-13	pDC	Sdc4
Csde13	2.77E-17	0.453812	0.495	0.272	6.79E-13	pDC	Csde1
Rhoa3	3.27E-17	0.423063	0.876	0.689	8.01E-13	pDC	Rhoa
Gpr137b1	3.32E-17	0.872518	0.426	0.231	8.12E-13	pDC	Gpr137b
Cct55	3.33E-17	0.478542	0.488	0.271	8.16E-13	pDC	Cct5
Ywhaz3	3.51E-17	0.39531	0.795	0.56	8.60E-13	pDC	Ywhaz
Rdx	4.12E-17	0.442353	0.386	0.198	1.01E-12	pDC	Rdx
Gsdmd2	4.15E-17	0.512226	0.517	0.298	1.02E-12	pDC	Gsdmd
Atp5j25	4.94E-17	0.439078	0.777	0.52	1.21E-12	pDC	Atp5j2
Far13	5.47E-17	0.464121	0.381	0.194	1.34E-12	pDC	Far1
Rps4x5	5.78E-17	0.333292	0.975	0.88	1.42E-12	pDC	Rps4x
Rasal22	6.09E-17	0.44587	0.314	0.151	1.49E-12	pDC	Rasal2
Smdt15	6.28E-17	0.430787	0.673	0.419	1.54E-12	pDC	Smdt1
Rpl285	7.48E-17	0.389199	0.953	0.842	1.83E-12	pDC	Rpl28
Eif3d3	7.49E-17	0.6184	0.394	0.209	1.84E-12	pDC	Eif3d
Oip5os1	8.45E-17	0.423992	0.356	0.179	2.07E-12	pDC	Oip5os1
Anapc113	9.07E-17	0.449174	0.594	0.351	2.22E-12	pDC	Anapc11
Ythdf23	9.75E-17	0.471834	0.453	0.25	2.39E-12	pDC	Ythdf2
Psmd24	1.04E-16	0.609092	0.379	0.199	2.56E-12	pDC	Psmd2
Hras1	1.05E-16	0.624372	0.314	0.155	2.57E-12	pDC	Hras

Xpa	1.17E-16	0.788223	0.28	0.133	2.86E-12	pDC	Xpa
Rpl106	1.28E-16	0.354869	0.943	0.841	3.14E-12	pDC	Rpl10
Ndufa76	1.38E-16	0.452205	0.738	0.496	3.38E-12	pDC	Ndufa7
Psmc6	1.44E-16	0.527008	0.399	0.213	3.52E-12	pDC	Psmc6
Ccng22	1.58E-16	0.588442	0.361	0.184	3.86E-12	pDC	Ccng2
Sf3b63	1.61E-16	0.5104	0.54	0.321	3.95E-12	pDC	Sf3b6
Ifi353	1.61E-16	0.524642	0.438	0.241	3.95E-12	pDC	Ifi35
Tubb4b1	1.62E-16	0.386921	0.342	0.171	3.96E-12	pDC	Tubb4b
Ufc13	1.62E-16	0.442762	0.47	0.261	3.96E-12	pDC	Ufc1
Rps286	1.69E-16	0.448389	0.901	0.758	4.14E-12	pDC	Rps28
Ppp1r23	1.71E-16	0.343239	0.418	0.221	4.19E-12	pDC	Ppp1r2
Dcaf11	1.76E-16	0.642257	0.262	0.121	4.31E-12	pDC	Dcaf11
Ost45	1.82E-16	0.406043	0.752	0.499	4.45E-12	pDC	Ost4
Cs1	1.84E-16	0.550118	0.366	0.19	4.51E-12	pDC	Cs
Cox6a15	2.23E-16	0.357761	0.772	0.514	5.45E-12	pDC	Cox6a1
Manbal	2.58E-16	0.47372	0.364	0.186	6.32E-12	pDC	Manbal
Irf54	2.64E-16	0.496418	0.705	0.471	6.48E-12	pDC	Irf5
Srsf32	3.73E-16	0.500808	0.646	0.405	9.13E-12	pDC	Srsf3
Adprh2	3.75E-16	0.513944	0.522	0.305	9.19E-12	pDC	Adprh
Baz2b4	4.12E-16	0.411616	0.624	0.385	1.01E-11	pDC	Baz2b
Zc3hav15	4.19E-16	0.44878	0.441	0.24	1.03E-11	pDC	Zc3hav1
Eif1a3	5.41E-16	0.812192	0.302	0.152	1.33E-11	pDC	Eif1a
Cyld1	5.85E-16	0.409081	0.401	0.212	1.43E-11	pDC	Cyld
Ppp2r1a3	6.39E-16	0.501954	0.431	0.237	1.56E-11	pDC	Ppp2r1a
Kpna43	6.43E-16	0.449496	0.708	0.453	1.57E-11	pDC	Kpna4
Rrp36	7.05E-16	0.517675	0.262	0.121	1.73E-11	pDC	Rrp36
Fcf12	7.27E-16	0.573625	0.403	0.218	1.78E-11	pDC	Fcf1
Hnrnpa03	7.35E-16	0.433318	0.498	0.286	1.80E-11	pDC	Hnrnpa0
Guk11	7.70E-16	0.789498	0.28	0.136	1.89E-11	pDC	Guk1
Ube2m4	7.73E-16	0.399746	0.545	0.313	1.89E-11	pDC	Ube2m
Ddx541	8.87E-16	0.739729	0.26	0.122	2.17E-11	pDC	Ddx54
Rps155	9.03E-16	0.439766	0.797	0.569	2.21E-11	pDC	Rps15
Rassf12	1.05E-15	0.647385	0.374	0.2	2.56E-11	pDC	Rassf1
Ccndbp11	1.06E-15	0.686034	0.277	0.133	2.59E-11	pDC	Ccndbp1
Tnfrsf11a3	1.06E-15	0.453298	0.606	0.378	2.60E-11	pDC	Tnfrsf11a
Gmps	1.10E-15	0.610592	0.267	0.126	2.69E-11	pDC	Gmps
Ywhaq1	1.10E-15	0.52375	0.304	0.152	2.70E-11	pDC	Ywhaq
Map41	1.11E-15	0.475737	0.668	0.443	2.73E-11	pDC	Map4
Nup88	1.20E-15	0.571197	0.282	0.135	2.95E-11	pDC	Nup88
Yme1l1	1.46E-15	0.552221	0.324	0.163	3.57E-11	pDC	Yme1l1
Flna4	1.62E-15	0.388441	0.371	0.196	3.97E-11	pDC	Flna
Pphln1	1.68E-15	0.463949	0.282	0.134	4.12E-11	pDC	Pphln1
Mrpl204	1.70E-15	0.378603	0.465	0.26	4.16E-11	pDC	Mrpl20
Slc6a62	1.74E-15	0.560171	0.601	0.378	4.27E-11	pDC	Slc6a6

Emp35	1.93E-15	0.367884	0.411	0.222	4.74E-11	pDC	Emp3
Pdcd53	2.47E-15	0.402677	0.502	0.294	6.06E-11	pDC	Pdcd5
Cox7b6	2.62E-15	0.455004	0.599	0.368	6.41E-11	pDC	Cox7b
Rbck11	2.67E-15	0.484834	0.394	0.212	6.54E-11	pDC	Rbck1
Psmc51	3.09E-15	0.494824	0.416	0.231	7.56E-11	pDC	Psmc5
Timm135	3.14E-15	0.388551	0.567	0.338	7.70E-11	pDC	Timm13
Hnrnpdl1	3.19E-15	0.498807	0.498	0.293	7.81E-11	pDC	Hnrnpdl
Psmb63	3.21E-15	0.412827	0.611	0.379	7.87E-11	pDC	Psmb6
Rps125	3.79E-15	0.324984	0.973	0.869	9.29E-11	pDC	Rps12
Osbpl83	3.93E-15	0.472012	0.45	0.255	9.62E-11	pDC	Osbpl8
Dbnl4	4.15E-15	0.43527	0.567	0.343	1.02E-10	pDC	Dbnl
Sf13	5.39E-15	0.366772	0.554	0.328	1.32E-10	pDC	Sf1
Mcrs1	6.46E-15	0.498454	0.262	0.124	1.58E-10	pDC	Mcrs1
Ndufb76	6.81E-15	0.424983	0.604	0.375	1.67E-10	pDC	Ndufb7
Ndufa18	9.00E-15	0.479756	0.545	0.332	2.20E-10	pDC	Ndufa1
Ubxn41	9.16E-15	0.433421	0.5	0.297	2.24E-10	pDC	Ubxn4
Pdcd4	9.22E-15	0.496985	0.322	0.164	2.26E-10	pDC	Pdcd4
Dnajc14	9.25E-15	0.993058	0.46	0.283	2.26E-10	pDC	Dnajc1
Dazap1	9.63E-15	0.449586	0.374	0.199	2.36E-10	pDC	Dazap1
Slu7	1.09E-14	0.703161	0.25	0.119	2.67E-10	pDC	Slu7
Flii3	1.12E-14	0.491016	0.413	0.232	2.74E-10	pDC	Flii
Cetn31	1.16E-14	0.391798	0.403	0.223	2.83E-10	pDC	Cetn3
Tmem234	1.19E-14	0.38802	0.616	0.385	2.91E-10	pDC	Tmem234
Clu1	1.29E-14	1.192025	0.26	0.128	3.15E-10	pDC	Clu
Phf5a2	1.48E-14	0.519315	0.342	0.18	3.63E-10	pDC	Phf5a
Tmod33	1.76E-14	0.305	0.45	0.252	4.31E-10	pDC	Tmod3
Flnb1	1.86E-14	0.781756	0.262	0.129	4.56E-10	pDC	Flnb
Rpl36a5	1.96E-14	0.405154	0.864	0.652	4.81E-10	pDC	Rpl36a
Sdhd4	1.98E-14	0.430176	0.48	0.277	4.86E-10	pDC	Sdhd
Clip2	2.28E-14	0.694342	0.282	0.142	5.59E-10	pDC	Clip2
Babam12	2.34E-14	0.511364	0.334	0.178	5.72E-10	pDC	Babam1
Ube2k4	2.37E-14	0.272602	0.49	0.281	5.80E-10	pDC	Ube2k
Thoc71	2.52E-14	0.52188	0.356	0.191	6.18E-10	pDC	Thoc7
Ilkap1	2.55E-14	0.499021	0.317	0.165	6.24E-10	pDC	Ilkap
Snrnp2002	2.58E-14	0.534951	0.287	0.144	6.32E-10	pDC	Snrnp200
Rbm27	2.69E-14	0.454556	0.267	0.129	6.59E-10	pDC	Rbm27
Dnaja24	2.87E-14	0.367923	0.515	0.305	7.02E-10	pDC	Dnaja2
Morf4l12	3.04E-14	0.357177	0.842	0.585	7.43E-10	pDC	Morf4l1
Pcbp14	3.17E-14	0.426622	0.75	0.528	7.75E-10	pDC	Pcbp1
Nubp12	3.46E-14	0.537757	0.329	0.174	8.47E-10	pDC	Nubp1
Specc12	3.75E-14	0.727003	0.594	0.401	9.18E-10	pDC	Specc1
Taf12	3.92E-14	0.47071	0.25	0.119	9.61E-10	pDC	Taf12
Hnrnpc2	4.12E-14	0.405662	0.438	0.248	1.01E-09	pDC	Hnrnpc
Aip4	4.29E-14	0.38913	0.379	0.205	1.05E-09	pDC	Aip

Stk17b5	4.30E-14	0.307685	0.337	0.173	1.05E-09	pDC	Stk17b
Snu134	4.54E-14	0.406712	0.525	0.318	1.11E-09	pDC	Snu13
Csnk1d	4.55E-14	0.396925	0.399	0.22	1.11E-09	pDC	Csnk1d
Ier3ip13	4.57E-14	0.280266	0.488	0.282	1.12E-09	pDC	Ier3ip1
Specc1l1	4.60E-14	0.577119	0.28	0.139	1.13E-09	pDC	Specc1l
Csnk1g1	5.09E-14	0.562042	0.314	0.162	1.25E-09	pDC	Csnk1g1
Fibp	5.20E-14	0.421846	0.381	0.21	1.27E-09	pDC	Fibp
Samd4b	5.32E-14	0.464828	0.272	0.132	1.30E-09	pDC	Samd4b
Rspry1	5.60E-14	0.405747	0.28	0.137	1.37E-09	pDC	Rspry1
Zcrb12	5.89E-14	0.391162	0.428	0.246	1.44E-09	pDC	Zcrb1
Snap233	6.93E-14	0.488736	0.438	0.251	1.70E-09	pDC	Snap23
Myl12b4	7.03E-14	0.386851	0.819	0.635	1.72E-09	pDC	Myl12b
Sp1103	7.52E-14	0.496562	0.423	0.246	1.84E-09	pDC	Sp110
Mrpl432	7.56E-14	0.428304	0.46	0.266	1.85E-09	pDC	Mrpl43
Ube2b2	8.95E-14	0.3835	0.589	0.365	2.19E-09	pDC	Ube2b
Dlgap4	9.80E-14	0.273895	0.448	0.253	2.40E-09	pDC	Dlgap4
Micu1	1.14E-13	0.730243	0.307	0.163	2.79E-09	pDC	Micu1
Cox7a2l5	1.16E-13	0.457637	0.673	0.459	2.83E-09	pDC	Cox7a2l
Micu2	1.21E-13	0.45701	0.337	0.18	2.96E-09	pDC	Micu2
Pcnp	1.28E-13	0.354785	0.349	0.186	3.12E-09	pDC	Pcnp
Park74	1.29E-13	0.37451	0.636	0.411	3.16E-09	pDC	Park7
Lamtor54	1.36E-13	0.413437	0.431	0.246	3.32E-09	pDC	Lamtor5
Dpf2	1.37E-13	0.559283	0.295	0.152	3.36E-09	pDC	Dpf2
Chmp53	1.44E-13	0.46151	0.396	0.223	3.53E-09	pDC	Chmp5
Polr2e1	1.50E-13	0.444478	0.446	0.265	3.68E-09	pDC	Polr2e
Ccrl23	1.66E-13	0.446697	0.297	0.154	4.07E-09	pDC	Ccrl2
Sdf42	1.69E-13	0.387058	0.762	0.513	4.13E-09	pDC	Sdf4
Pdap12	1.94E-13	0.429682	0.304	0.158	4.75E-09	pDC	Pdap1
Psmb25	2.24E-13	0.405581	0.584	0.371	5.48E-09	pDC	Psmb2
Vamp85	2.80E-13	0.433746	0.79	0.596	6.85E-09	pDC	Vamp8
Yeats4	3.12E-13	0.557899	0.349	0.193	7.65E-09	pDC	Yeats4
Rwdd13	3.29E-13	0.525176	0.356	0.198	8.06E-09	pDC	Rwdd1
Rnf41	3.36E-13	0.427329	0.292	0.151	8.22E-09	pDC	Rnf4
Smarcd22	3.90E-13	0.440453	0.28	0.142	9.54E-09	pDC	Smarcd2
Add12	4.01E-13	0.258154	0.428	0.243	9.83E-09	pDC	Add1
Atrx2	4.09E-13	0.415358	0.502	0.313	1.00E-08	pDC	Atrx
Kdelr13	4.10E-13	0.494321	0.547	0.344	1.00E-08	pDC	Kdelr1
Scaper1	4.14E-13	0.254252	0.356	0.193	1.01E-08	pDC	Scaper
Atp11b2	4.22E-13	0.488104	0.282	0.144	1.03E-08	pDC	Atp11b
Rab11b2	4.37E-13	0.293432	0.582	0.362	1.07E-08	pDC	Rab11b
Tpm31	4.49E-13	0.357751	0.809	0.629	1.10E-08	pDC	Tpm3
Eif4h3	4.77E-13	0.400883	0.49	0.3	1.17E-08	pDC	Eif4h
Lypla11	4.87E-13	0.471488	0.265	0.132	1.19E-08	pDC	Lypla1
Srrm11	5.03E-13	0.310624	0.644	0.413	1.23E-08	pDC	Srrm1

Lpcat11	5.08E-13	0.344167	0.418	0.237	1.24E-08	pDC	Lpcat1
Lrrfip12	5.74E-13	0.489286	0.5	0.313	1.41E-08	pDC	Lrrfip1
Ilk2	5.80E-13	0.442262	0.483	0.292	1.42E-08	pDC	Ilk
Mcrip1	6.12E-13	0.401626	0.495	0.298	1.50E-08	pDC	Mcrip1
Dstn3	6.20E-13	0.590784	0.45	0.277	1.52E-08	pDC	Dstn
Ndufa65	6.55E-13	0.454257	0.74	0.522	1.60E-08	pDC	Ndufa6
Cpsf7	6.57E-13	0.493116	0.27	0.136	1.61E-08	pDC	Cpsf7
Mrpl212	6.94E-13	0.515985	0.28	0.144	1.70E-08	pDC	Mrpl21
Commd3	7.55E-13	0.420745	0.448	0.266	1.85E-08	pDC	Commd3
Wrn	7.63E-13	0.328645	0.25	0.122	1.87E-08	pDC	Wrn
Glod41	9.24E-13	0.612868	0.3	0.16	2.26E-08	pDC	Glod4
Tbc1d17	9.83E-13	0.45277	0.295	0.154	2.41E-08	pDC	Tbc1d17
Dynlrb15	9.99E-13	0.367002	0.525	0.323	2.45E-08	pDC	Dynlrb1
Glrx8	1.03E-12	0.773556	0.285	0.154	2.51E-08	pDC	Glrx
Ndufs45	1.13E-12	0.283573	0.502	0.3	2.77E-08	pDC	Ndufs4
Gnl22	1.18E-12	0.676914	0.322	0.177	2.88E-08	pDC	Gnl2
Selenok2	1.30E-12	0.575456	0.777	0.59	3.18E-08	pDC	Selenok
Clptm12	1.34E-12	0.34897	0.532	0.324	3.27E-08	pDC	Clptm1
Tomm34	1.41E-12	0.432131	0.349	0.191	3.45E-08	pDC	Tomm34
Piezo11	1.55E-12	0.461213	0.312	0.168	3.79E-08	pDC	Piezo1
Washc3	1.56E-12	0.607977	0.314	0.172	3.82E-08	pDC	Washc3
Puf602	1.63E-12	0.335115	0.475	0.288	3.99E-08	pDC	Puf60
Med301	1.75E-12	0.449766	0.309	0.166	4.29E-08	pDC	Med30
Pdrg11	1.84E-12	0.51444	0.255	0.129	4.50E-08	pDC	Pdrg1
Smim201	1.90E-12	0.570323	0.307	0.168	4.65E-08	pDC	Smim20
Dbndd23	2.20E-12	0.49313	0.304	0.165	5.38E-08	pDC	Dbndd2
Trappc6b1	2.20E-12	0.290412	0.423	0.245	5.39E-08	pDC	Trappc6b
B3gat3	2.26E-12	0.475099	0.309	0.166	5.52E-08	pDC	B3gat3
Mat2b	2.27E-12	0.325394	0.351	0.195	5.57E-08	pDC	Mat2b
Vps72	2.33E-12	0.277278	0.322	0.171	5.71E-08	pDC	Vps72
Hint22	2.40E-12	0.399588	0.332	0.183	5.89E-08	pDC	Hint2
Arpc5l1	2.50E-12	0.384441	0.391	0.226	6.12E-08	pDC	Arpc5l
Pnpla21	2.58E-12	0.410562	0.376	0.215	6.33E-08	pDC	Pnpla2
Rad23a1	2.77E-12	0.40192	0.371	0.211	6.79E-08	pDC	Rad23a
Trappc6a3	2.91E-12	0.513413	0.28	0.146	7.12E-08	pDC	Trappc6a
Stub11	3.06E-12	0.381834	0.495	0.302	7.49E-08	pDC	Stub1
22-Mar	3.10E-12	0.349302	0.359	0.202	7.59E-08	pDC	2-Mar
Nfya	3.20E-12	0.490466	0.302	0.16	7.83E-08	pDC	Nfya
Capns15	3.25E-12	0.285976	0.564	0.352	7.97E-08	pDC	Capns1
Psen22	3.26E-12	0.510427	0.267	0.14	7.99E-08	pDC	Psen2
Phax1	3.26E-12	0.597289	0.307	0.167	7.99E-08	pDC	Phax
Uqcrb6	3.41E-12	0.342701	0.644	0.425	8.35E-08	pDC	Uqcrb
Igbp13	3.98E-12	0.522736	0.384	0.226	9.75E-08	pDC	Igbp1
Bcl62	4.12E-12	0.652974	0.287	0.154	1.01E-07	pDC	Bcl6

Gemin72	4.46E-12	0.340987	0.339	0.188	1.09E-07	pDC	Gemin7
Trir3	4.54E-12	0.336036	0.562	0.355	1.11E-07	pDC	Trir
Eif3i5	4.68E-12	0.318124	0.525	0.326	1.15E-07	pDC	Eif3i
Snx82	4.69E-12	0.33653	0.265	0.135	1.15E-07	pDC	Snx8
Gtpbp23	5.51E-12	0.403811	0.45	0.271	1.35E-07	pDC	Gtpbp2
Pdcl31	5.56E-12	0.323048	0.351	0.195	1.36E-07	pDC	Pdcl3
Pole42	5.85E-12	0.343269	0.309	0.166	1.43E-07	pDC	Pole4
1110004F1	6.04E-12	0.310875	0.386	0.221	1.48E-07	pDC	1110004F10Rik
Hnrnpd3	6.32E-12	0.357653	0.53	0.334	1.55E-07	pDC	Hnrnpd
Dnaja15	6.47E-12	0.313065	0.478	0.294	1.59E-07	pDC	Dnaja1
Ncaph21	7.48E-12	0.458826	0.26	0.134	1.83E-07	pDC	Ncaph2
Psmc33	7.60E-12	0.325616	0.488	0.295	1.86E-07	pDC	Psmc3
Daam1	7.90E-12	0.411404	0.277	0.146	1.93E-07	pDC	Daam1
1110038B1	8.06E-12	0.645311	0.342	0.198	1.97E-07	pDC	1110038B12Rik
Atp6v1b24	8.72E-12	0.531215	0.525	0.338	2.14E-07	pDC	Atp6v1b2
Hgs	8.84E-12	0.400355	0.262	0.135	2.17E-07	pDC	Hgs
Arid4b	9.36E-12	0.41239	0.344	0.193	2.29E-07	pDC	Arid4b
Immp1l	9.47E-12	0.3434	0.317	0.175	2.32E-07	pDC	Immp1l
Cmpk14	9.64E-12	0.280266	0.455	0.273	2.36E-07	pDC	Cmpk1
Pin42	9.74E-12	0.465143	0.282	0.152	2.38E-07	pDC	Pin4
Commd6	1.00E-11	0.277606	0.329	0.181	2.46E-07	pDC	Commd6
Abcg12	1.25E-11	0.447923	0.455	0.275	3.05E-07	pDC	Abcg1
Ppp2ca1	1.27E-11	0.295901	0.547	0.347	3.10E-07	pDC	Ppp2ca
Mrps341	1.32E-11	0.570897	0.322	0.182	3.24E-07	pDC	Mrps34
Gnptab1	1.35E-11	0.559534	0.267	0.141	3.30E-07	pDC	Gnptab
Nhp23	1.53E-11	0.557487	0.312	0.174	3.74E-07	pDC	Nhp2
Trappc44	1.66E-11	0.288591	0.436	0.256	4.07E-07	pDC	Trappc4
Wbp4	1.75E-11	0.517904	0.309	0.172	4.28E-07	pDC	Wbp4
Mlf22	1.86E-11	0.352806	0.599	0.387	4.55E-07	pDC	Mlf2
Marf1	1.89E-11	0.331297	0.309	0.168	4.63E-07	pDC	Marf1
Rnf1874	1.93E-11	0.381094	0.46	0.284	4.73E-07	pDC	Rnf187
Ube2j2	2.13E-11	0.479292	0.351	0.2	5.21E-07	pDC	Ube2j2
Snw12	2.14E-11	0.338882	0.384	0.224	5.24E-07	pDC	Snw1
Zmym2	2.23E-11	0.382525	0.272	0.143	5.46E-07	pDC	Zmym2
Eif5a5	2.31E-11	0.39915	0.696	0.493	5.65E-07	pDC	Eif5a
Mrpl364	2.35E-11	0.498535	0.347	0.198	5.75E-07	pDC	Mrpl36
Rchy12	2.58E-11	0.406623	0.314	0.173	6.33E-07	pDC	Rchy1
Dynll15	2.66E-11	0.284366	0.639	0.42	6.50E-07	pDC	Dynll1
Nrbf21	2.66E-11	0.596075	0.262	0.141	6.52E-07	pDC	Nrbf2
Bfar	2.78E-11	0.429026	0.324	0.182	6.82E-07	pDC	Bfar
Mrps18c2	3.24E-11	0.33677	0.334	0.189	7.93E-07	pDC	Mrps18c
Ccni2	3.38E-11	0.334146	0.621	0.42	8.28E-07	pDC	Ccni
Mapre21	3.68E-11	0.408328	0.473	0.291	9.01E-07	pDC	Mapre2
Utp33	3.76E-11	0.484592	0.337	0.194	9.21E-07	pDC	Utp3

Mdh26	3.88E-11	0.446797	0.557	0.356	9.51E-07	pDC	Mdh2
Rpl55	3.94E-11	0.33439	0.832	0.616	9.66E-07	pDC	Rpl5
Hdac11	4.00E-11	0.396282	0.324	0.182	9.79E-07	pDC	Hdac1
Supt51	4.65E-11	0.390882	0.304	0.168	1.14E-06	pDC	Supt5
Prcp4	5.06E-11	0.384168	0.557	0.368	1.24E-06	pDC	Prcp
Micos134	5.34E-11	0.296277	0.505	0.319	1.31E-06	pDC	Micos13
Rtf22	5.36E-11	0.366866	0.399	0.236	1.31E-06	pDC	Rtf2
Tfg	5.55E-11	0.468775	0.347	0.201	1.36E-06	pDC	Tfg
Fgfr1op23	5.84E-11	0.374224	0.438	0.267	1.43E-06	pDC	Fgfr1op2
Nedd4l2	6.50E-11	0.417214	0.361	0.208	1.59E-06	pDC	Nedd4l
Mbtd11	6.65E-11	0.29965	0.396	0.234	1.63E-06	pDC	Mbtd1
Mapk1ip1l	7.24E-11	0.453552	0.277	0.151	1.77E-06	pDC	Mapk1ip1l
Nrp22	7.81E-11	0.6421	0.285	0.16	1.91E-06	pDC	Nrp2
Lrch4	8.39E-11	0.428071	0.351	0.204	2.05E-06	pDC	Lrch4
Chrac13	9.05E-11	0.319169	0.406	0.24	2.22E-06	pDC	Chrac1
Cdv32	9.23E-11	0.660951	0.262	0.143	2.26E-06	pDC	Cdv3
Rnf146	9.95E-11	0.379905	0.257	0.138	2.44E-06	pDC	Rnf146
Dap2	1.02E-10	1.20723	0.272	0.158	2.50E-06	pDC	Dap
Rps6ka5	1.07E-10	0.295873	0.309	0.171	2.63E-06	pDC	Rps6ka5
Syf24	1.08E-10	0.255508	0.406	0.241	2.64E-06	pDC	Syf2
Hnrnph32	1.13E-10	0.396629	0.433	0.261	2.77E-06	pDC	Hnrnph3
Ergic2	1.18E-10	0.287408	0.3	0.166	2.88E-06	pDC	Ergic2
Ppp1r35	1.18E-10	0.365576	0.27	0.146	2.90E-06	pDC	Ppp1r35
Eif2s13	1.20E-10	0.395498	0.361	0.208	2.95E-06	pDC	Eif2s1
Clk3	1.23E-10	0.539869	0.25	0.134	3.02E-06	pDC	Clk3
Rpl46	1.25E-10	0.303952	0.812	0.599	3.07E-06	pDC	Rpl4
Rnps11	1.32E-10	0.274103	0.332	0.189	3.22E-06	pDC	Rnps1
Ube2d2a1	1.36E-10	0.315392	0.646	0.432	3.32E-06	pDC	Ube2d2a
Rnf14	1.40E-10	0.273415	0.28	0.153	3.42E-06	pDC	Rnf14
Rab6a1	1.45E-10	0.469647	0.29	0.161	3.54E-06	pDC	Rab6a
Rpl316	1.48E-10	0.27988	0.748	0.529	3.62E-06	pDC	Rpl31
Cox144	1.52E-10	0.291323	0.436	0.267	3.72E-06	pDC	Cox14
Wsb21	1.66E-10	0.389901	0.265	0.144	4.07E-06	pDC	Wsb2
Gls2	1.68E-10	0.255842	0.438	0.266	4.11E-06	pDC	Gls
Ubac22	1.79E-10	0.281261	0.502	0.318	4.37E-06	pDC	Ubac2
Gsk3a1	2.05E-10	0.423394	0.26	0.142	5.01E-06	pDC	Gsk3a
Snrpd34	2.06E-10	0.28843	0.406	0.243	5.04E-06	pDC	Snrpd3
Snhg122	2.13E-10	0.649074	0.295	0.172	5.23E-06	pDC	Snhg12
Iah13	2.15E-10	0.377987	0.272	0.149	5.25E-06	pDC	Iah1
Ehmt21	2.19E-10	0.406328	0.282	0.159	5.37E-06	pDC	Ehmt2
Smu11	2.25E-10	0.393903	0.309	0.177	5.51E-06	pDC	Smu1
Tmem1605	2.25E-10	0.261704	0.475	0.294	5.52E-06	pDC	Tmem160
Glud13	2.39E-10	0.256593	0.537	0.343	5.85E-06	pDC	Glud1
Rps255	2.84E-10	0.280714	0.837	0.639	6.95E-06	pDC	Rps25

Pi4k2a1	2.89E-10	0.257405	0.277	0.152	7.09E-06	pDC	Pi4k2a
Elf12	3.01E-10	0.318981	0.564	0.367	7.38E-06	pDC	Elf1
Ube2r22	3.25E-10	0.255187	0.401	0.243	7.95E-06	pDC	Ube2r2
Josd2	3.36E-10	0.374019	0.277	0.154	8.24E-06	pDC	Josd2
Fundc22	3.42E-10	0.600944	0.282	0.162	8.38E-06	pDC	Fundc2
Ubald24	4.10E-10	0.421863	0.45	0.289	1.00E-05	pDC	Ubald2
AI837181	4.24E-10	0.427066	0.272	0.15	1.04E-05	pDC	AI837181
Emc8	4.42E-10	0.304767	0.304	0.174	1.08E-05	pDC	Emc8
Cnpy31	5.06E-10	0.383686	0.47	0.299	1.24E-05	pDC	Cnpy3
Suz12	5.26E-10	0.274526	0.277	0.153	1.29E-05	pDC	Suz12
Anapc2	5.39E-10	0.472497	0.282	0.158	1.32E-05	pDC	Anapc2
Atad2b3	5.93E-10	0.26998	0.384	0.229	1.45E-05	pDC	Atad2b
Ccng11	7.60E-10	0.697196	0.282	0.162	1.86E-05	pDC	Ccng1
Psmc21	7.89E-10	0.382665	0.359	0.218	1.93E-05	pDC	Psmc2
Dpy301	8.19E-10	0.300422	0.275	0.154	2.01E-05	pDC	Dpy30
Chmp4b7	8.40E-10	0.266532	0.678	0.466	2.06E-05	pDC	Chmp4b
H2-Eb16	9.17E-10	0.351766	0.795	0.594	2.25E-05	pDC	H2-Eb1
Bola32	9.81E-10	0.368333	0.25	0.138	2.40E-05	pDC	Bola3
Rex1bd4	1.13E-09	0.275285	0.396	0.24	2.78E-05	pDC	Rex1bd
Ddx51	1.14E-09	0.265614	0.926	0.786	2.79E-05	pDC	Ddx5
Fkbp1a5	1.15E-09	0.306072	0.517	0.337	2.82E-05	pDC	Fkbp1a
Eny22	1.16E-09	0.258758	0.282	0.159	2.84E-05	pDC	Eny2
Rcsd13	1.17E-09	0.285425	0.554	0.376	2.87E-05	pDC	Rcsd1
Ppp3cb	1.39E-09	0.374013	0.29	0.163	3.40E-05	pDC	Ppp3cb
Map2k2	1.60E-09	0.293131	0.552	0.366	3.92E-05	pDC	Map2k2
Rbm7	1.66E-09	0.382234	0.366	0.222	4.06E-05	pDC	Rbm7
Dph32	1.72E-09	0.357338	0.257	0.143	4.22E-05	pDC	Dph3
Lcp14	1.86E-09	0.398628	0.814	0.69	4.56E-05	pDC	Lcp1
Dctn4	2.17E-09	0.31775	0.371	0.227	5.31E-05	pDC	Dctn4
Idh3b3	3.48E-09	0.294491	0.436	0.274	8.52E-05	pDC	Idh3b
Ybx15	3.49E-09	0.251563	0.792	0.573	8.55E-05	pDC	Ybx1
Uqcc31	3.65E-09	0.275235	0.309	0.181	8.95E-05	pDC	Uqcc3
Dock104	3.66E-09	0.45741	0.752	0.61	8.96E-05	pDC	Dock10
Derl21	4.48E-09	0.254328	0.389	0.238	0.00011	pDC	Derl2
Huwe12	4.63E-09	0.277722	0.418	0.264	0.000113	pDC	Huwe1
Arid1b2	4.84E-09	0.275627	0.554	0.369	0.000118	pDC	Arid1b
Sdf21	5.73E-09	0.395304	0.389	0.247	0.00014	pDC	Sdf2
Ttyh3	6.21E-09	0.278503	0.443	0.284	0.000152	pDC	Ttyh3
Snrpf5	6.55E-09	0.301684	0.433	0.273	0.000161	pDC	Snrpf
Tmx3	6.65E-09	0.344739	0.332	0.2	0.000163	pDC	Tmx3
Uba13	6.73E-09	0.340196	0.339	0.208	0.000165	pDC	Uba1
Fbxl51	7.09E-09	0.406457	0.287	0.167	0.000174	pDC	Fbxl5
Efhd23	7.10E-09	0.277054	0.8	0.619	0.000174	pDC	Efhd2
Brd22	8.98E-09	0.348929	0.418	0.266	0.00022	pDC	Brd2

Sharpin3	9.22E-09	0.342331	0.347	0.213	0.000226	pDC	Sharpin
Capzb2	9.24E-09	0.270632	0.842	0.687	0.000226	pDC	Capzb
Arpc1b6	9.29E-09	0.263274	0.854	0.704	0.000228	pDC	Arpc1b
Rtn41	9.92E-09	0.2502	0.762	0.58	0.000243	pDC	Rtn4
Bub3	1.13E-08	0.277846	0.379	0.24	0.000276	pDC	Bub3
Aff13	1.32E-08	0.51219	0.374	0.237	0.000323	pDC	Aff1
Impdh23	1.35E-08	0.397444	0.287	0.173	0.00033	pDC	Impdh2
Lyst2	1.66E-08	0.350096	0.361	0.228	0.000407	pDC	Lyst
Gfer2	1.69E-08	0.392332	0.324	0.199	0.000415	pDC	Gfer
Spag71	1.83E-08	0.366311	0.277	0.165	0.000448	pDC	Spag7
Nabp21	2.20E-08	0.316941	0.292	0.174	0.000538	pDC	Nabp2
Adrm11	2.31E-08	0.324438	0.29	0.173	0.000566	pDC	Adrm1
Tnfrsf1b3	2.81E-08	0.29361	0.515	0.355	0.000688	pDC	Tnfrsf1b
Rpl36al5	2.95E-08	0.260362	0.839	0.649	0.000724	pDC	Rpl36al
Cers51	3.06E-08	0.365799	0.275	0.163	0.00075	pDC	Cers5
1810026B0	3.48E-08	0.328925	0.418	0.268	0.000853	pDC	1810026B05Rik
Marcks2	4.25E-08	0.260305	0.911	0.793	0.001042	pDC	Marcks
Ube2q13	4.58E-08	0.287444	0.337	0.21	0.001122	pDC	Ube2q1
Copz14	4.62E-08	0.323559	0.364	0.227	0.001132	pDC	Copz1
Mrpl46	5.25E-08	0.324776	0.339	0.215	0.001286	pDC	Mrpl4
Fxr1	6.04E-08	0.298102	0.322	0.198	0.001479	pDC	Fxr1
Hsp90aa13	7.23E-08	0.320403	0.485	0.33	0.001772	pDC	Hsp90aa1
Wnk12	8.00E-08	0.438286	0.748	0.608	0.00196	pDC	Wnk1
Anapc13	9.07E-08	0.271079	0.366	0.234	0.00222	pDC	Anapc13
Paics1	9.20E-08	0.416053	0.287	0.175	0.002252	pDC	Paics
Ddx461	9.39E-08	0.313337	0.262	0.156	0.002299	pDC	Ddx46
Bcl7b	9.93E-08	0.250462	0.27	0.161	0.002432	pDC	Bcl7b
Hdgf5	1.06E-07	0.269423	0.287	0.174	0.002589	pDC	Hdgf
Parp83	1.10E-07	0.257669	0.453	0.301	0.002706	pDC	Parp8
Add33	1.19E-07	0.298721	0.25	0.145	0.002903	pDC	Add3
Mdm2	1.22E-07	0.331385	0.295	0.178	0.002977	pDC	Mdm2
Prpsap1	1.40E-07	0.461829	0.285	0.175	0.00342	pDC	Prpsap1
Fbxw2	1.46E-07	0.294098	0.277	0.166	0.003578	pDC	Fbxw2
Usp3	2.48E-07	0.322937	0.282	0.172	0.006064	pDC	Usp3
Ppp2cb2	2.94E-07	0.343598	0.29	0.178	0.0072	pDC	Ppp2cb
Qars3	3.76E-07	0.377569	0.262	0.161	0.009218	pDC	Qars
Nagk2	4.72E-07	0.417337	0.297	0.19	0.011559	pDC	Nagk
Elof12	4.78E-07	0.317656	0.26	0.159	0.01171	pDC	Elof1
Hspe15	5.64E-07	0.308125	0.609	0.428	0.013814	pDC	Hspe1
Cdk86	8.91E-07	0.30049	0.963	0.902	0.02183	pDC	Cdk8
Rab324	1.06E-06	0.349818	0.3	0.196	0.025888	pDC	Rab32
Timm233	1.08E-06	0.323376	0.403	0.273	0.026418	pDC	Timm23
Polr2c2	2.11E-06	0.251187	0.25	0.155	0.051633	pDC	Polr2c
Smagp2	3.15E-06	0.269911	0.26	0.165	0.077248	pDC	Smagp

Resf13	5.42E-06	0.261484	0.376	0.258	0.132846	pDC	Resf1
Plaa	6.67E-06	0.331807	0.28	0.182	0.163324	pDC	Plaa
Rnaset2a4	1.51E-05	0.56705	0.453	0.339	0.370802	pDC	Rnaset2a
Rnase64	9.13E-05	0.502893	0.27	0.187	1	pDC	Rnase6
Btaf1	0.000254	0.324867	0.282	0.198	1	pDC	Btaf1

Supplementary Table 8: DEGs for Figure 5L

	p_val	avg_log2FC	pct.1	pct.2	p_val_adj	cluster	gene
Cd8b1	0	1.893148	0.558	0.23	0	CD8+ Cytot	Cd8b1
Cxcr6	0	1.285956	0.792	0.465	0	CD8+ Cytot	Cxcr6
Cd8a	0	1.709226	0.554	0.233	0	CD8+ Cytot	Cd8a
Cd3g	0	0.946278	0.972	0.678	0	CD8+ Cytot	Cd3g
Cd3e	0	0.806026	0.96	0.675	0	CD8+ Cytot	Cd3e
Cd3d	0	0.795628	0.929	0.647	0	CD8+ Cytot	Cd3d
Lat	0	0.809164	0.878	0.618	0	CD8+ Cytot	Lat
Thy1	0	1.010673	0.898	0.649	0	CD8+ Cytot	Thy1
Ms4a4b	0	0.85971	0.952	0.705	0	CD8+ Cytot	Ms4a4b
Hcst	0	0.962453	0.893	0.699	0	CD8+ Cytot	Hcst
Rpl13a	0	0.505948	0.991	0.938	0	CD8+ Cytot	Rpl13a
Rps13	0	0.480221	0.998	0.951	0	CD8+ Cytot	Rps13
Rps24	0	0.44717	1	0.973	0	CD8+ Cytot	Rps24
Cd52	2.84E-301	0.630651	0.987	0.904	6.96E-297	CD8+ Cytot	Cd52
Rps15a	2.47E-300	0.495395	0.998	0.954	6.05E-296	CD8+ Cytot	Rps15a
Ccl5	2.63E-293	1.071528	0.852	0.657	6.45E-289	CD8+ Cytot	Ccl5
Rac2	6.64E-289	0.567422	0.976	0.876	1.62E-284	CD8+ Cytot	Rac2
Rps16	7.75E-273	0.423582	0.999	0.96	1.90E-268	CD8+ Cytot	Rps16
Nkg7	3.42E-271	0.781145	0.864	0.608	8.37E-267	CD8+ Cytot	Nkg7
Rps27	3.51E-271	0.593827	0.986	0.916	8.60E-267	CD8+ Cytot	Rps27
Rpl18a	3.21E-270	0.406069	0.999	0.962	7.86E-266	CD8+ Cytot	Rpl18a
Rps10	1.10E-267	0.413364	0.999	0.957	2.69E-263	CD8+ Cytot	Rps10
Fau	9.20E-260	0.377608	1	0.971	2.25E-255	CD8+ Cytot	Fau
Rpl19	6.84E-254	0.377959	0.999	0.964	1.67E-249	CD8+ Cytot	Rpl19
Id2	3.90E-250	0.830369	0.833	0.67	9.55E-246	CD8+ Cytot	Id2
Rpl9	6.84E-239	0.383894	0.999	0.963	1.68E-234	CD8+ Cytot	Rpl9
Cxcr3	1.98E-232	0.973029	0.682	0.453	4.85E-228	CD8+ Cytot	Cxcr3
Rps7	1.23E-231	0.408892	0.998	0.956	3.00E-227	CD8+ Cytot	Rps7
Rpsa	8.11E-226	0.396763	0.999	0.966	1.99E-221	CD8+ Cytot	Rpsa
Rpl30	1.63E-221	0.39	0.998	0.959	3.99E-217	CD8+ Cytot	Rpl30
Gzmk	4.02E-221	1.7744	0.343	0.137	9.84E-217	CD8+ Cytot	Gzmk
Lck	7.50E-214	0.657431	0.864	0.673	1.84E-209	CD8+ Cytot	Lck
Ltb	1.25E-204	0.594009	0.925	0.724	3.07E-200	CD8+ Cytot	Ltb
Rpl17	1.67E-204	0.383189	0.994	0.948	4.10E-200	CD8+ Cytot	Rpl17
Rpl37a	2.65E-199	0.43754	0.994	0.945	6.49E-195	CD8+ Cytot	Rpl37a
Ms4a6b	2.58E-198	0.791326	0.753	0.589	6.32E-194	CD8+ Cytot	Ms4a6b
Themis	9.06E-197	0.617716	0.58	0.338	2.22E-192	CD8+ Cytot	Themis
Rpl18	3.10E-194	0.38374	0.999	0.952	7.60E-190	CD8+ Cytot	Rpl18
Rpl27	2.57E-193	0.392342	0.992	0.926	6.29E-189	CD8+ Cytot	Rpl27
Ptpn18	4.12E-191	0.51417	0.93	0.829	1.01E-186	CD8+ Cytot	Ptpn18
Ctsw	1.44E-189	0.635978	0.863	0.671	3.53E-185	CD8+ Cytot	Ctsw
Shisa5	7.28E-188	0.448563	0.964	0.878	1.78E-183	CD8+ Cytot	Shisa5

Rpl11	1.43E-185	0.338983	0.998	0.957	3.51E-181	CD8+ Cytot	Rpl11
Rps5	3.23E-185	0.370844	0.999	0.951	7.90E-181	CD8+ Cytot	Rps5
Rpl12	4.81E-185	0.466864	0.969	0.899	1.18E-180	CD8+ Cytot	Rpl12
Rpl27a	3.52E-184	0.342162	0.997	0.96	8.63E-180	CD8+ Cytot	Rpl27a
S100a6	4.86E-184	0.857557	0.768	0.593	1.19E-179	CD8+ Cytot	S100a6
Gimap3	3.19E-183	0.627546	0.831	0.665	7.80E-179	CD8+ Cytot	Gimap3
Ly6c2	1.40E-181	1.179154	0.535	0.316	3.43E-177	CD8+ Cytot	Ly6c2
Sh3bgrl3	1.46E-179	0.485588	0.978	0.899	3.58E-175	CD8+ Cytot	Sh3bgrl3
Rpl8	1.79E-177	0.390308	0.994	0.94	4.37E-173	CD8+ Cytot	Rpl8
Tpt1	1.51E-176	0.329257	0.999	0.962	3.70E-172	CD8+ Cytot	Tpt1
Arhgdib	3.14E-174	0.454814	0.954	0.867	7.68E-170	CD8+ Cytot	Arhgdib
Rpl13	4.42E-171	0.332342	1	0.972	1.08E-166	CD8+ Cytot	Rpl13
Rps4x	7.55E-171	0.349392	0.997	0.952	1.85E-166	CD8+ Cytot	Rps4x
Dnajc15	1.17E-169	0.929624	0.659	0.512	2.86E-165	CD8+ Cytot	Dnajc15
Ly6a	2.92E-167	0.601819	0.822	0.622	7.15E-163	CD8+ Cytot	Ly6a
Rps11	2.17E-163	0.339575	0.998	0.955	5.30E-159	CD8+ Cytot	Rps11
Gimap7	2.14E-160	0.797821	0.655	0.465	5.25E-156	CD8+ Cytot	Gimap7
Rps21	1.56E-159	0.434029	0.983	0.925	3.81E-155	CD8+ Cytot	Rps21
Plac8	4.59E-159	1.072525	0.495	0.295	1.12E-154	CD8+ Cytot	Plac8
Eef1a1	2.87E-157	0.291912	1	0.98	7.02E-153	CD8+ Cytot	Eef1a1
Rps3a1	1.26E-155	0.321411	0.997	0.958	3.09E-151	CD8+ Cytot	Rps3a1
Rpl36	2.49E-153	0.38369	0.988	0.927	6.10E-149	CD8+ Cytot	Rpl36
Rps9	2.33E-151	0.320976	0.996	0.955	5.70E-147	CD8+ Cytot	Rps9
Rpl23	2.94E-149	0.322222	0.997	0.958	7.21E-145	CD8+ Cytot	Rpl23
Itga1	3.50E-149	0.984932	0.43	0.244	8.58E-145	CD8+ Cytot	Itga1
H2-K1	1.74E-148	0.307431	0.998	0.971	4.26E-144	CD8+ Cytot	H2-K1
Slfn1	2.23E-144	0.916982	0.508	0.32	5.46E-140	CD8+ Cytot	Slfn1
Gramd3	6.90E-144	0.791484	0.508	0.318	1.69E-139	CD8+ Cytot	Gramd3
1-Sep	2.54E-143	0.540109	0.832	0.682	6.23E-139	CD8+ Cytot	1-Sep
Rps14	3.66E-143	0.324832	0.99	0.94	8.96E-139	CD8+ Cytot	Rps14
Gimap4	3.65E-142	0.546738	0.833	0.68	8.94E-138	CD8+ Cytot	Gimap4
Rps3	8.40E-140	0.322653	0.994	0.944	2.06E-135	CD8+ Cytot	Rps3
Rpl34	8.27E-139	0.336545	0.994	0.943	2.02E-134	CD8+ Cytot	Rpl34
Rpl32	1.47E-138	0.319972	0.997	0.954	3.59E-134	CD8+ Cytot	Rpl32
Rps23	1.99E-137	0.320685	0.993	0.945	4.88E-133	CD8+ Cytot	Rps23
Tmsb10	7.54E-137	0.352811	0.999	0.954	1.85E-132	CD8+ Cytot	Tmsb10
Pde7a	1.92E-131	0.614442	0.628	0.467	4.70E-127	CD8+ Cytot	Pde7a
Epsti1	4.98E-131	0.581813	0.742	0.629	1.22E-126	CD8+ Cytot	Epsti1
AW112010	9.51E-131	0.398604	0.961	0.854	2.33E-126	CD8+ Cytot	AW112010
Rps18	8.17E-129	0.348065	0.985	0.922	2.00E-124	CD8+ Cytot	Rps18
Gm44174	5.45E-127	1.391315	0.265	0.122	1.33E-122	CD8+ Cytot	Gm44174
Grap2	9.17E-127	0.466102	0.652	0.49	2.25E-122	CD8+ Cytot	Grap2
Rplp2	1.81E-123	0.337212	0.983	0.921	4.42E-119	CD8+ Cytot	Rplp2
Rpl21	3.11E-118	0.293373	0.996	0.957	7.61E-114	CD8+ Cytot	Rpl21

Gimap6	3.17E-118	0.515917	0.757	0.609	7.75E-114	CD8+ Cytot	Gimap6
Rpl35a	2.98E-117	0.323425	0.991	0.943	7.29E-113	CD8+ Cytot	Rpl35a
Rps8	3.68E-117	0.288484	0.998	0.963	9.01E-113	CD8+ Cytot	Rps8
Cnn2	2.29E-115	0.508232	0.826	0.703	5.62E-111	CD8+ Cytot	Cnn2
Fasl	6.86E-113	0.943926	0.445	0.287	1.68E-108	CD8+ Cytot	Fasl
Rpl6	3.42E-112	0.299822	0.989	0.933	8.39E-108	CD8+ Cytot	Rpl6
H2afj	5.18E-110	0.600344	0.753	0.663	1.27E-105	CD8+ Cytot	H2afj
Lsp1	9.42E-108	0.512142	0.792	0.651	2.31E-103	CD8+ Cytot	Lsp1
Hopx	3.04E-104	0.712684	0.599	0.458	7.44E-100	CD8+ Cytot	Hopx
Rpl29	7.39E-104	0.313916	0.985	0.923	1.81E-99	CD8+ Cytot	Rpl29
Fxyd5	9.55E-103	0.351002	0.939	0.847	2.34E-98	CD8+ Cytot	Fxyd5
Myl12b	1.13E-102	0.381145	0.912	0.834	2.76E-98	CD8+ Cytot	Myl12b
Cd226	1.46E-102	0.493185	0.597	0.442	3.56E-98	CD8+ Cytot	Cd226
Rpl37	5.56E-101	0.329231	0.989	0.936	1.36E-96	CD8+ Cytot	Rpl37
Rpl38	1.29E-100	0.365854	0.956	0.881	3.16E-96	CD8+ Cytot	Rpl38
Coro1a	2.22E-97	0.318691	0.96	0.882	5.43E-93	CD8+ Cytot	Coro1a
Rpl24	1.02E-95	0.293012	0.982	0.923	2.51E-91	CD8+ Cytot	Rpl24
Eif1	1.28E-95	0.262601	0.986	0.935	3.13E-91	CD8+ Cytot	Eif1
H2-Q7	1.66E-94	0.341707	0.925	0.852	4.07E-90	CD8+ Cytot	H2-Q7
S100a10	1.39E-93	0.348479	0.9	0.772	3.40E-89	CD8+ Cytot	S100a10
Zfp683	9.66E-92	1.226158	0.281	0.154	2.36E-87	CD8+ Cytot	Zfp683
Crip1	1.14E-91	0.395785	0.882	0.751	2.78E-87	CD8+ Cytot	Crip1
B4galnt1	6.02E-88	0.565905	0.673	0.57	1.48E-83	CD8+ Cytot	B4galnt1
Itk	2.32E-87	0.428071	0.65	0.52	5.68E-83	CD8+ Cytot	Itk
Rplp1	2.47E-87	0.256949	0.996	0.962	6.04E-83	CD8+ Cytot	Rplp1
Rpl39	3.42E-86	0.312303	0.981	0.922	8.38E-82	CD8+ Cytot	Rpl39
Slfn2	8.27E-83	0.505172	0.699	0.602	2.02E-78	CD8+ Cytot	Slfn2
Selenow	1.28E-82	0.339122	0.868	0.794	3.13E-78	CD8+ Cytot	Selenow
Dusp2	8.18E-82	0.622556	0.633	0.508	2.00E-77	CD8+ Cytot	Dusp2
Pfdn5	3.22E-80	0.339316	0.899	0.84	7.88E-76	CD8+ Cytot	Pfdn5
Psmb8	8.25E-80	0.32716	0.912	0.824	2.02E-75	CD8+ Cytot	Psmb8
Gmfg	4.07E-79	0.541699	0.68	0.594	9.97E-75	CD8+ Cytot	Gmfg
S100a4	9.11E-79	0.730424	0.549	0.414	2.23E-74	CD8+ Cytot	S100a4
Ppp1r18	8.57E-77	0.474055	0.727	0.657	2.10E-72	CD8+ Cytot	Ppp1r18
Txnip	3.99E-76	0.56972	0.633	0.535	9.76E-72	CD8+ Cytot	Txnip
Rplp0	6.00E-76	0.272119	0.994	0.944	1.47E-71	CD8+ Cytot	Rplp0
Rpl22	6.20E-76	0.268217	0.969	0.901	1.52E-71	CD8+ Cytot	Rpl22
Rps28	1.04E-74	0.348282	0.945	0.877	2.54E-70	CD8+ Cytot	Rps28
Eef2	2.00E-74	0.287778	0.942	0.886	4.89E-70	CD8+ Cytot	Eef2
Eif3f	4.78E-74	0.336122	0.883	0.821	1.17E-69	CD8+ Cytot	Eif3f
Jaml	9.59E-72	0.970276	0.362	0.248	2.35E-67	CD8+ Cytot	Jaml
Cd2	3.39E-71	0.310626	0.743	0.594	8.29E-67	CD8+ Cytot	Cd2
Rack1	3.82E-71	0.287008	0.942	0.87	9.35E-67	CD8+ Cytot	Rack1
Sit1	1.53E-69	1.016658	0.288	0.177	3.76E-65	CD8+ Cytot	Sit1

Tle5	1.64E-69	0.331409	0.858	0.787	4.01E-65	CD8+ Cyto	Tle5
Rps19	2.26E-68	0.293457	0.979	0.933	5.54E-64	CD8+ Cyto	Rps19
Acp5	1.02E-67	0.699711	0.46	0.35	2.49E-63	CD8+ Cyto	Acp5
Trac	6.53E-67	0.572007	0.53	0.402	1.60E-62	CD8+ Cyto	Trac
Rps29	8.81E-67	0.310986	0.971	0.914	2.16E-62	CD8+ Cyto	Rps29
Tbc1d10c	1.22E-65	0.515848	0.612	0.519	3.00E-61	CD8+ Cyto	Tbc1d10c
Pdcd4	7.11E-65	0.423751	0.663	0.572	1.74E-60	CD8+ Cyto	Pdcd4
Clec2d	9.71E-65	0.45703	0.683	0.604	2.38E-60	CD8+ Cyto	Clec2d
Rinl	6.77E-64	0.474766	0.649	0.566	1.66E-59	CD8+ Cyto	Rinl
Pabpc1	8.79E-64	0.320354	0.872	0.816	2.15E-59	CD8+ Cyto	Pabpc1
Ptprcap	1.72E-63	0.305699	0.882	0.742	4.22E-59	CD8+ Cyto	Ptprcap
Itgb1	6.96E-63	0.492261	0.672	0.604	1.70E-58	CD8+ Cyto	Itgb1
S100a11	6.84E-62	0.338766	0.829	0.72	1.67E-57	CD8+ Cyto	S100a11
Gimap1	1.71E-58	0.45585	0.635	0.547	4.20E-54	CD8+ Cyto	Gimap1
Il2rg	1.15E-57	0.315461	0.823	0.741	2.81E-53	CD8+ Cyto	Il2rg
Cd40lg	5.04E-57	0.857431	0.251	0.15	1.23E-52	CD8+ Cyto	Cd40lg
Sp100	1.09E-56	0.379582	0.706	0.64	2.68E-52	CD8+ Cyto	Sp100
Ubash3a	3.75E-56	0.660802	0.412	0.308	9.17E-52	CD8+ Cyto	Ubash3a
Stk17b	6.41E-56	0.423743	0.647	0.567	1.57E-51	CD8+ Cyto	Stk17b
Fam189b	7.09E-56	0.782142	0.384	0.284	1.74E-51	CD8+ Cyto	Fam189b
Btg1	7.61E-55	0.317902	0.817	0.76	1.86E-50	CD8+ Cyto	Btg1
Rpl36a	1.55E-54	0.29388	0.908	0.835	3.80E-50	CD8+ Cyto	Rpl36a
Sub1	2.43E-54	0.332873	0.834	0.764	5.95E-50	CD8+ Cyto	Sub1
Ifi47	3.91E-52	0.450859	0.602	0.52	9.58E-48	CD8+ Cyto	Ifi47
Rpl36al	4.50E-52	0.273396	0.907	0.834	1.10E-47	CD8+ Cyto	Rpl36al
Sema4a	9.05E-52	0.765462	0.401	0.311	2.22E-47	CD8+ Cyto	Sema4a
Uqcrh	3.21E-49	0.292661	0.85	0.793	7.87E-45	CD8+ Cyto	Uqcrh
Adam19	4.77E-49	0.380401	0.448	0.344	1.17E-44	CD8+ Cyto	Adam19
Klk8	4.76E-48	0.834464	0.369	0.285	1.17E-43	CD8+ Cyto	Klk8
Gstp1	5.67E-47	0.405795	0.66	0.6	1.39E-42	CD8+ Cyto	Gstp1
Gm11808	1.01E-45	0.522329	0.579	0.527	2.46E-41	CD8+ Cyto	Gm11808
Gpsm3	1.07E-45	0.465028	0.605	0.558	2.62E-41	CD8+ Cyto	Gpsm3
Trbc2	1.23E-45	0.516026	0.504	0.414	3.00E-41	CD8+ Cyto	Trbc2
Apol7e	2.34E-44	0.831392	0.265	0.179	5.72E-40	CD8+ Cyto	Apol7e
Tcf7	8.78E-44	0.668666	0.339	0.244	2.15E-39	CD8+ Cyto	Tcf7
Gabarapl2	9.25E-43	0.429363	0.632	0.591	2.27E-38	CD8+ Cyto	Gabarapl2
H2-T22	4.59E-42	0.375853	0.662	0.607	1.12E-37	CD8+ Cyto	H2-T22
Rabac1	1.31E-41	0.389815	0.654	0.612	3.21E-37	CD8+ Cyto	Rabac1
Fkbp3	2.85E-41	0.497865	0.552	0.496	6.99E-37	CD8+ Cyto	Fkbp3
Leprotl1	3.64E-40	0.357621	0.662	0.616	8.92E-36	CD8+ Cyto	Leprotl1
Fam78a	1.16E-38	0.90534	0.263	0.186	2.85E-34	CD8+ Cyto	Fam78a
Pstpip1	2.33E-38	0.653242	0.411	0.344	5.71E-34	CD8+ Cyto	Pstpip1
Ccnd2	3.37E-37	0.388568	0.582	0.509	8.24E-33	CD8+ Cyto	Ccnd2
Atp5h	4.61E-37	0.250807	0.849	0.792	1.13E-32	CD8+ Cyto	Atp5h

Zfp36l2	3.39E-36	0.321666	0.741	0.696	8.29E-32	CD8+ Cyto1	Zfp36l2
Gng2	3.78E-36	0.468962	0.545	0.505	9.25E-32	CD8+ Cyto1	Gng2
S100a13	7.49E-36	0.438654	0.582	0.542	1.83E-31	CD8+ Cyto1	S100a13
Ifitm10	7.62E-36	0.699822	0.358	0.282	1.87E-31	CD8+ Cyto1	Ifitm10
Ppp3cc	1.73E-35	0.382037	0.448	0.371	4.24E-31	CD8+ Cyto1	Ppp3cc
Il27ra	2.35E-35	0.523968	0.41	0.334	5.76E-31	CD8+ Cyto1	Il27ra
Gstp3	8.07E-35	0.643236	0.378	0.303	1.98E-30	CD8+ Cyto1	Gstp3
Flt3l	8.29E-35	0.629669	0.386	0.316	2.03E-30	CD8+ Cyto1	Flt3l
Gm8369	9.29E-35	0.67626	0.316	0.238	2.28E-30	CD8+ Cyto1	Gm8369
Cd6	1.19E-34	0.541899	0.378	0.297	2.93E-30	CD8+ Cyto1	Cd6
Ifng	5.89E-34	0.534825	0.294	0.215	1.44E-29	CD8+ Cyto1	Ifng
Itgb7	6.59E-34	0.343521	0.605	0.533	1.61E-29	CD8+ Cyto1	Itgb7
Rasal3	8.85E-34	0.48174	0.49	0.44	2.17E-29	CD8+ Cyto1	Rasal3
Snrpg	1.95E-33	0.278994	0.786	0.728	4.77E-29	CD8+ Cyto1	Snrpg
Saraf	2.73E-33	0.434541	0.577	0.547	6.67E-29	CD8+ Cyto1	Saraf
Ctla2a	7.30E-33	0.358609	0.471	0.385	1.79E-28	CD8+ Cyto1	Ctla2a
Izumo4	1.35E-32	0.835371	0.266	0.198	3.30E-28	CD8+ Cyto1	Izumo4
Itgal	1.52E-32	0.413048	0.544	0.496	3.72E-28	CD8+ Cyto1	Itgal
Itgb2	1.88E-32	0.317316	0.705	0.665	4.60E-28	CD8+ Cyto1	Itgb2
Vps28	2.29E-32	0.366201	0.649	0.627	5.62E-28	CD8+ Cyto1	Vps28
Adgre5	3.17E-31	0.357062	0.586	0.53	7.76E-27	CD8+ Cyto1	Adgre5
Zyx	8.44E-31	0.423375	0.572	0.525	2.07E-26	CD8+ Cyto1	Zyx
Prr13	2.06E-30	0.383067	0.615	0.587	5.05E-26	CD8+ Cyto1	Prr13
Cd48	2.26E-30	0.386926	0.61	0.576	5.54E-26	CD8+ Cyto1	Cd48
Pglyrp2	3.13E-30	0.616167	0.331	0.264	7.66E-26	CD8+ Cyto1	Pglyrp2
Nsa2	1.95E-29	0.318726	0.669	0.649	4.78E-25	CD8+ Cyto1	Nsa2
Ifi208	3.66E-29	0.585461	0.29	0.219	8.95E-25	CD8+ Cyto1	Ifi208
Il7r	3.68E-29	0.303342	0.488	0.406	9.02E-25	CD8+ Cyto1	Il7r
Ypel3	5.33E-29	0.30336	0.664	0.624	1.31E-24	CD8+ Cyto1	Ypel3
H2-Q4	9.27E-29	0.269935	0.695	0.657	2.27E-24	CD8+ Cyto1	H2-Q4
BE692007	1.28E-27	0.534288	0.266	0.197	3.14E-23	CD8+ Cyto1	BE692007
Arhgap9	4.12E-27	0.43594	0.494	0.455	1.01E-22	CD8+ Cyto1	Arhgap9
Gtf2i	4.18E-27	0.549765	0.399	0.348	1.02E-22	CD8+ Cyto1	Gtf2i
Cish	2.56E-26	0.437097	0.45	0.385	6.28E-22	CD8+ Cyto1	Cish
Eif3e	7.69E-26	0.36526	0.576	0.555	1.88E-21	CD8+ Cyto1	Eif3e
Cox5a	2.06E-25	0.268414	0.773	0.712	5.05E-21	CD8+ Cyto1	Cox5a
Ifi206	2.85E-25	0.606001	0.294	0.231	6.98E-21	CD8+ Cyto1	Ifi206
Ndufa6	1.15E-24	0.280657	0.703	0.681	2.82E-20	CD8+ Cyto1	Ndufa6
Gimap5	1.32E-24	0.328182	0.517	0.458	3.23E-20	CD8+ Cyto1	Gimap5
Uba52	1.42E-24	0.312175	0.7	0.67	3.48E-20	CD8+ Cyto1	Uba52
Tma7	2.06E-24	0.281188	0.704	0.67	5.05E-20	CD8+ Cyto1	Tma7
H2afy	9.29E-23	0.287977	0.651	0.629	2.27E-18	CD8+ Cyto1	H2afy
Zap70	1.23E-22	0.365053	0.473	0.42	3.02E-18	CD8+ Cyto1	Zap70
Grap	1.25E-22	0.514194	0.39	0.346	3.07E-18	CD8+ Cyto1	Grap

Isg20	1.45E-22	0.365563	0.409	0.351	3.54E-18	CD8+ Cyto	Isg20
Slc9a3r1	2.14E-21	0.340189	0.526	0.499	5.24E-17	CD8+ Cyto	Slc9a3r1
Cd96	2.71E-21	0.520736	0.312	0.256	6.65E-17	CD8+ Cyto	Cd96
Tmem9b	3.71E-21	0.576955	0.393	0.367	9.08E-17	CD8+ Cyto	Tmem9b
Sorl1	6.55E-20	0.315874	0.428	0.387	1.60E-15	CD8+ Cyto	Sorl1
Dgka	1.40E-19	0.401778	0.336	0.281	3.42E-15	CD8+ Cyto	Dgka
Rnf166	1.82E-19	0.544709	0.389	0.358	4.45E-15	CD8+ Cyto	Rnf166
Ech1	8.45E-19	0.465655	0.457	0.436	2.07E-14	CD8+ Cyto	Ech1
Med10	2.67E-18	0.591017	0.37	0.344	6.53E-14	CD8+ Cyto	Med10
9-Sep	4.63E-18	0.286355	0.532	0.505	1.13E-13	CD8+ Cyto	9-Sep
P2rx7	5.83E-18	0.510068	0.332	0.29	1.43E-13	CD8+ Cyto	P2rx7
Scml4	1.09E-17	0.362246	0.255	0.202	2.67E-13	CD8+ Cyto	Scml4
Anxa6	2.29E-17	0.30105	0.566	0.543	5.60E-13	CD8+ Cyto	Anxa6
Fgl2	5.41E-17	0.402641	0.459	0.426	1.33E-12	CD8+ Cyto	Fgl2
Emg1	9.13E-17	0.361421	0.509	0.499	2.24E-12	CD8+ Cyto	Emg1
Tomm7	1.61E-16	0.257998	0.63	0.61	3.93E-12	CD8+ Cyto	Tomm7
Prdx6	1.85E-16	0.264758	0.545	0.522	4.53E-12	CD8+ Cyto	Prdx6
Slamf7	2.18E-16	0.596527	0.29	0.247	5.35E-12	CD8+ Cyto	Slamf7
Abhd8	3.90E-16	0.559039	0.305	0.264	9.55E-12	CD8+ Cyto	Abhd8
Sh2d2a	4.55E-16	0.257247	0.514	0.463	1.11E-11	CD8+ Cyto	Sh2d2a
Rcbtb2	4.78E-16	0.660836	0.316	0.285	1.17E-11	CD8+ Cyto	Rcbtb2
Dnaja1	8.33E-16	0.279317	0.581	0.573	2.04E-11	CD8+ Cyto	Dnaja1
Psmb10	3.09E-15	0.266719	0.58	0.564	7.56E-11	CD8+ Cyto	Psmb10
Lpxn	4.04E-15	0.415181	0.429	0.41	9.90E-11	CD8+ Cyto	Lpxn
Twf2	4.11E-15	0.40688	0.46	0.451	1.01E-10	CD8+ Cyto	Twf2
Acap1	4.19E-15	0.36403	0.42	0.386	1.03E-10	CD8+ Cyto	Acap1
Pttg1	1.08E-14	0.506894	0.382	0.36	2.65E-10	CD8+ Cyto	Pttg1
Tprgl	1.37E-14	0.427445	0.434	0.422	3.36E-10	CD8+ Cyto	Tprgl
Trbc1	1.39E-14	0.533593	0.284	0.241	3.40E-10	CD8+ Cyto	Trbc1
Npm3	1.64E-14	0.425377	0.4	0.38	4.01E-10	CD8+ Cyto	Npm3
Sash3	2.02E-14	0.496756	0.392	0.373	4.95E-10	CD8+ Cyto	Sash3
Evl	7.79E-14	0.270477	0.509	0.487	1.91E-09	CD8+ Cyto	Evl
Scand1	2.57E-13	0.282738	0.582	0.584	6.29E-09	CD8+ Cyto	Scand1
Supt4a	3.63E-13	0.312566	0.528	0.532	8.88E-09	CD8+ Cyto	Supt4a
Pkp3	3.67E-13	0.416875	0.362	0.327	8.99E-09	CD8+ Cyto	Pkp3
Lfng	4.49E-13	0.415511	0.385	0.362	1.10E-08	CD8+ Cyto	Lfng
Rnf167	1.91E-12	0.511879	0.356	0.337	4.67E-08	CD8+ Cyto	Rnf167
Padi2	2.28E-12	0.529911	0.286	0.253	5.59E-08	CD8+ Cyto	Padi2
Ifi27	4.85E-12	0.27268	0.491	0.467	1.19E-07	CD8+ Cyto	Ifi27
Mettl23	6.70E-12	0.393619	0.434	0.43	1.64E-07	CD8+ Cyto	Mettl23
Cd69	1.09E-11	0.258253	0.407	0.372	2.67E-07	CD8+ Cyto	Cd69
Gimap9	1.45E-11	0.355895	0.374	0.345	3.56E-07	CD8+ Cyto	Gimap9
Cdc42ep3	2.24E-11	0.553572	0.263	0.232	5.48E-07	CD8+ Cyto	Cdc42ep3
6-Sep	4.65E-11	0.311759	0.418	0.402	1.14E-06	CD8+ Cyto	6-Sep

Hip1	6.02E-11	0.378616	0.323	0.297	1.47E-06	CD8+ Cytot	Hip1
1110008P1	7.06E-11	0.467187	0.353	0.334	1.73E-06	CD8+ Cytot	1110008P14Rik
AI413582	2.37E-10	0.424633	0.398	0.392	5.80E-06	CD8+ Cytot	AI413582
Fam102a	3.03E-10	0.276002	0.315	0.281	7.43E-06	CD8+ Cytot	Fam102a
Syf2	3.14E-10	0.369334	0.414	0.411	7.69E-06	CD8+ Cytot	Syf2
Aprt	4.13E-10	0.325623	0.472	0.461	1.01E-05	CD8+ Cytot	Aprt
Cbx4	5.59E-10	0.428002	0.341	0.322	1.37E-05	CD8+ Cytot	Cbx4
Ifi213	7.25E-10	0.380987	0.263	0.23	1.78E-05	CD8+ Cytot	Ifi213
Mrps16	1.69E-09	0.370429	0.433	0.44	4.14E-05	CD8+ Cytot	Mrps16
Ccdc85b	3.83E-09	0.499154	0.286	0.266	9.38E-05	CD8+ Cytot	Ccdc85b
Mxd4	4.12E-09	0.439915	0.331	0.312	0.000101	CD8+ Cytot	Mxd4
Glipr2	6.70E-09	0.44366	0.324	0.305	0.000164	CD8+ Cytot	Glipr2
Arl4c	7.30E-09	0.502898	0.319	0.304	0.000179	CD8+ Cytot	Arl4c
Ppdpf	8.26E-09	0.342947	0.424	0.422	0.000202	CD8+ Cytot	Ppdpf
Rnf187	1.01E-08	0.313479	0.472	0.482	0.000247	CD8+ Cytot	Rnf187
Il16	1.31E-08	0.371123	0.386	0.382	0.000321	CD8+ Cytot	Il16
Scamp3	1.54E-08	0.371825	0.39	0.389	0.000378	CD8+ Cytot	Scamp3
Chd3	1.90E-08	0.297517	0.383	0.366	0.000464	CD8+ Cytot	Chd3
Cd7	3.44E-08	0.285616	0.323	0.284	0.000843	CD8+ Cytot	Cd7
Sec11a	4.59E-08	0.298138	0.465	0.48	0.001123	CD8+ Cytot	Sec11a
Churc1	1.68E-07	0.446617	0.358	0.358	0.004113	CD8+ Cytot	Churc1
Rtp4	3.17E-07	0.370499	0.348	0.333	0.007759	CD8+ Cytot	Rtp4
Zbp1	4.58E-07	0.292796	0.383	0.372	0.011218	CD8+ Cytot	Zbp1
AI467606	8.12E-07	0.426296	0.295	0.28	0.019883	CD8+ Cytot	AI467606
Sh2d1a	8.36E-07	0.251118	0.32	0.295	0.020468	CD8+ Cytot	Sh2d1a
Rab8a	8.48E-07	0.361907	0.399	0.409	0.020772	CD8+ Cytot	Rab8a
Lef1	8.86E-07	0.369123	0.261	0.236	0.021706	CD8+ Cytot	Lef1
Sh2d3c	3.63E-06	0.387214	0.253	0.236	0.088941	CD8+ Cytot	Sh2d3c
Zdhhc18	1.50E-05	0.257638	0.362	0.358	0.366595	CD8+ Cytot	Zdhhc18
Cnp	7.56E-05	0.37562	0.328	0.332	1	CD8+ Cytot	Cnp
Gramd1a	9.53E-05	0.256277	0.375	0.38	1	CD8+ Cytot	Gramd1a
Thap3	0.000107	0.394844	0.266	0.258	1	CD8+ Cytot	Thap3
Def6	0.000111	0.290081	0.37	0.378	1	CD8+ Cytot	Def6
Idnk	0.000126	0.313908	0.369	0.38	1	CD8+ Cytot	Idnk
Cd27	0.000197	0.264035	0.292	0.279	1	CD8+ Cytot	Cd27
Paip2	0.000228	0.273548	0.438	0.463	1	CD8+ Cytot	Paip2
Tbrg1	0.00023	0.338045	0.338	0.344	1	CD8+ Cytot	Tbrg1
Tmem134	0.000257	0.297427	0.408	0.43	1	CD8+ Cytot	Tmem134
Lypla2	0.000268	0.342302	0.374	0.389	1	CD8+ Cytot	Lypla2
Rassf5	0.000356	0.34677	0.319	0.324	1	CD8+ Cytot	Rassf5
Sp110	0.000394	0.261862	0.385	0.401	1	CD8+ Cytot	Sp110
Dusp11	0.000504	0.297692	0.374	0.391	1	CD8+ Cytot	Dusp11
Sptssa	0.000504	0.328128	0.41	0.434	1	CD8+ Cytot	Sptssa
Cited2	0.000625	0.356961	0.344	0.35	1	CD8+ Cytot	Cited2

Emc10	0.001004	0.282237	0.412	0.439	1	CD8+ Cytot	Emc10
Maf1	0.001961	0.323453	0.36	0.379	1	CD8+ Cytot	Maf1
Cirbp	0.001987	0.356581	0.349	0.362	1	CD8+ Cytot	Cirbp
Tsc22d3	0.002678	0.304807	0.347	0.353	1	CD8+ Cytot	Tsc22d3
Uqcc2	0.004086	0.2689	0.402	0.428	1	CD8+ Cytot	Uqcc2
Fam32a	0.004392	0.325123	0.312	0.325	1	CD8+ Cytot	Fam32a
Pop5	0.00528	0.372418	0.304	0.317	1	CD8+ Cytot	Pop5
Mrpl58	0.005314	0.366044	0.338	0.357	1	CD8+ Cytot	Mrpl58
Chmp1a	0.006883	0.47194	0.252	0.257	1	CD8+ Cytot	Chmp1a
Arl2bp	0.008201	0.323971	0.302	0.311	1	CD8+ Cytot	Arl2bp
Mogs	0.00941	0.252323	0.21	0.253	1	CD8+ Cytot	Mogs
Tnfrsf4	0	3.026921	0.727	0.153	0	Treg	Tnfrsf4
Ctla4	0	2.657371	0.692	0.2	0	Treg	Ctla4
Izumo1r	0	3.071834	0.628	0.141	0	Treg	Izumo1r
Capg	0	1.97467	0.676	0.291	0	Treg	Capg
Tnfrsf18	0	1.993415	0.757	0.407	0	Treg	Tnfrsf18
Foxp3	0	3.566081	0.399	0.055	0	Treg	Foxp3
Ifi27l2a	0	1.793905	0.947	0.61	0	Treg	Ifi27l2a
Il2ra	1.47E-292	2.391758	0.432	0.133	3.60E-288	Treg	Il2ra
Icos	2.25E-284	1.909264	0.585	0.253	5.51E-280	Treg	Icos
Shisa51	3.38E-283	0.825138	0.982	0.887	8.28E-279	Treg	Shisa5
Ly6a1	4.93E-269	1.235529	0.897	0.638	1.21E-264	Treg	Ly6a
Ikzf2	1.02E-261	1.87184	0.54	0.235	2.50E-257	Treg	Ikzf2
H2-K11	3.01E-245	0.538178	0.999	0.974	7.36E-241	Treg	H2-K1
Cd5	3.80E-233	1.350245	0.665	0.344	9.32E-229	Treg	Cd5
Cd21	7.27E-228	1.123981	0.836	0.602	1.78E-223	Treg	Cd2
Cd4	2.80E-216	1.527467	0.462	0.173	6.85E-212	Treg	Cd4
Ass1	3.27E-202	1.560103	0.565	0.281	8.00E-198	Treg	Ass1
Rln3	2.80E-183	2.595502	0.262	0.072	6.86E-179	Treg	Rln3
Tigit	2.00E-179	1.714926	0.527	0.269	4.91E-175	Treg	Tigit
Ptprcap1	1.27E-177	0.747119	0.92	0.756	3.11E-173	Treg	Ptprcap
Ltb1	7.37E-177	0.82973	0.928	0.75	1.80E-172	Treg	Ltb
Srgn	5.20E-162	0.829846	0.95	0.845	1.27E-157	Treg	Srgn
Tnfrsf9	2.67E-157	2.053005	0.376	0.153	6.55E-153	Treg	Tnfrsf9
Isg201	1.77E-153	1.528553	0.575	0.337	4.34E-149	Treg	Isg20
Cd3d1	3.72E-153	0.679001	0.927	0.684	9.12E-149	Treg	Cd3d
AW112010	3.36E-147	0.632017	0.978	0.866	8.24E-143	Treg	AW112010
Cd3e1	3.73E-147	0.615857	0.95	0.713	9.14E-143	Treg	Cd3e
Glrx	8.38E-144	1.572966	0.53	0.312	2.05E-139	Treg	Glrx
Stat1	1.12E-142	1.049847	0.792	0.628	2.75E-138	Treg	Stat1
Ccr8	1.23E-140	1.789989	0.383	0.168	3.02E-136	Treg	Ccr8
H2-D1	5.90E-140	0.401652	0.998	0.969	1.44E-135	Treg	H2-D1
Iigp1	2.33E-136	1.776096	0.337	0.137	5.70E-132	Treg	Iigp1
Cd3g1	5.28E-136	0.57713	0.946	0.72	1.29E-131	Treg	Cd3g

Ndfip1	5.99E-136	0.748557	0.869	0.768	1.47E-131	Treg		Ndfip1
Samhd1	1.28E-134	1.285179	0.681	0.504	3.14E-130	Treg		Samhd1
Trac1	4.56E-132	1.015969	0.642	0.404	1.12E-127	Treg		Trac
Igflr1	2.42E-130	1.500721	0.408	0.195	5.91E-126	Treg		Igflr1
Arl5a	4.25E-127	1.943877	0.394	0.201	1.04E-122	Treg		Arl5a
Limd2	7.19E-124	0.645664	0.889	0.771	1.76E-119	Treg		Limd2
H2-T23	3.81E-122	0.638586	0.917	0.811	9.34E-118	Treg		H2-T23
Tspan32	5.90E-119	1.322683	0.458	0.247	1.44E-114	Treg		Tspan32
Rac21	8.83E-118	0.48656	0.965	0.89	2.16E-113	Treg		Rac2
Maf	7.17E-116	1.293661	0.452	0.241	1.76E-111	Treg		Maf
B2m	1.42E-115	0.385328	0.994	0.951	3.48E-111	Treg		B2m
Sdf4	3.30E-115	0.955628	0.732	0.593	8.09E-111	Treg		Sdf4
Pglyrp1	6.86E-113	1.198259	0.583	0.381	1.68E-108	Treg		Pglyrp1
Gbp6	6.53E-110	1.458286	0.426	0.231	1.60E-105	Treg		Gbp6
Rpl241	3.23E-109	0.418908	0.979	0.931	7.92E-105	Treg		Rpl24
Dusp1	3.82E-109	1.419082	0.479	0.278	9.35E-105	Treg		Dusp1
Inpp4b	9.63E-108	0.371216	0.877	0.716	2.36E-103	Treg		Inpp4b
H2-T221	1.29E-107	0.869986	0.748	0.603	3.15E-103	Treg		H2-T22
Igtp	1.92E-105	1.328421	0.536	0.352	4.69E-101	Treg		Igtp
S100a101	4.20E-103	0.630629	0.905	0.788	1.03E-98	Treg		S100a10
A430093F1	5.72E-103	1.506711	0.324	0.148	1.40E-98	Treg		A430093F15Rik
Adamts6	1.60E-101	1.543585	0.26	0.104	3.92E-97	Treg		Adamts6
Rps15a1	3.23E-99	0.350236	0.997	0.96	7.91E-95	Treg		Rps15a
Ephx1	1.07E-95	1.788101	0.259	0.108	2.61E-91	Treg		Ephx1
Rabgap1l	2.73E-94	0.689161	0.67	0.513	6.68E-90	Treg		Rabgap1l
Fxyd51	7.96E-93	0.492075	0.941	0.859	1.95E-88	Treg		Fxyd5
Cish1	2.46E-92	0.966029	0.568	0.378	6.01E-88	Treg		Cish
S100a111	2.90E-92	0.616948	0.864	0.73	7.09E-88	Treg		S100a11
Fam169b	5.52E-91	1.125603	0.352	0.176	1.35E-86	Treg		Fam169b
Rgs1	1.88E-90	0.890116	0.715	0.551	4.59E-86	Treg		Rgs1
Smpdl3a	3.60E-90	1.001616	0.549	0.373	8.81E-86	Treg		Smpdl3a
Tox	1.15E-87	0.772888	0.482	0.287	2.82E-83	Treg		Tox
Jund	4.10E-86	0.622246	0.869	0.772	1.00E-81	Treg		Jund
Tpt11	4.19E-85	0.294275	0.998	0.967	1.03E-80	Treg		Tpt1
Nfkbia	1.63E-84	1.034281	0.663	0.503	3.99E-80	Treg		Nfkbia
Pim1	2.62E-84	1.270522	0.519	0.37	6.43E-80	Treg		Pim1
Itgae	5.22E-84	1.282475	0.329	0.163	1.28E-79	Treg		Itgae
Lat1	1.15E-82	0.579264	0.827	0.659	2.82E-78	Treg		Lat
Pkp31	1.92E-82	1.037825	0.493	0.315	4.70E-78	Treg		Pkp3
Hif1a	2.00E-82	1.21358	0.471	0.306	4.91E-78	Treg		Hif1a
Cd61	3.74E-82	0.948783	0.48	0.294	9.15E-78	Treg		Cd6
Gm83691	2.83E-81	1.052222	0.413	0.235	6.93E-77	Treg		Gm8369
Gstp31	6.22E-80	1.039425	0.475	0.3	1.52E-75	Treg		Gstp3
Psmb9	1.03E-79	0.632153	0.786	0.66	2.52E-75	Treg		Psmb9

Selenow1	5.22E-79	0.507745	0.891	0.801	1.28E-74	Treg	Selenow
Lck1	1.18E-78	0.495666	0.848	0.7	2.90E-74	Treg	Lck
Psmb81	3.77E-77	0.458352	0.922	0.834	9.23E-73	Treg	Psmb8
Ly6e	4.05E-77	0.410264	0.97	0.916	9.91E-73	Treg	Ly6e
Samsn1	3.93E-76	1.249095	0.437	0.282	9.62E-72	Treg	Samsn1
Coro1a1	5.92E-76	0.390596	0.952	0.893	1.45E-71	Treg	Coro1a
Cyb5a	4.27E-75	1.006064	0.56	0.423	1.05E-70	Treg	Cyb5a
Zbp11	1.69E-72	1.127767	0.511	0.357	4.13E-68	Treg	Zbp1
Rpl391	1.91E-72	0.362153	0.984	0.93	4.69E-68	Treg	Rpl39
Rgs10	7.54E-72	0.762424	0.64	0.476	1.85E-67	Treg	Rgs10
Mndal	9.87E-71	0.843301	0.615	0.468	2.42E-66	Treg	Mndal
Gimap11	3.46E-68	0.579525	0.692	0.551	8.48E-64	Treg	Gimap1
Pdcd1	5.53E-68	1.313987	0.322	0.173	1.35E-63	Treg	Pdcd1
Ifi203	8.12E-68	0.896844	0.598	0.461	1.99E-63	Treg	Ifi203
Rps271	9.40E-67	0.31569	0.983	0.925	2.30E-62	Treg	Rps27
Serpina3g	3.27E-66	1.148369	0.459	0.306	8.01E-62	Treg	Serpina3g
Zfp36l1	4.56E-64	0.938279	0.613	0.489	1.12E-59	Treg	Zfp36l1
Slfn11	4.93E-64	0.913341	0.506	0.345	1.21E-59	Treg	Slfn1
Rps131	1.01E-63	0.252002	0.995	0.958	2.48E-59	Treg	Rps13
Rpl271	2.55E-63	0.280945	0.986	0.935	6.25E-59	Treg	Rpl27
H2-Q71	4.89E-63	0.406496	0.928	0.861	1.20E-58	Treg	H2-Q7
Rpl221	2.85E-62	0.325439	0.969	0.91	6.97E-58	Treg	Rpl22
Cd28	8.60E-62	0.837346	0.419	0.26	2.11E-57	Treg	Cd28
Rhoh	2.17E-61	0.819746	0.548	0.411	5.32E-57	Treg	Rhoh
Eva1b	3.29E-61	1.282788	0.315	0.179	8.06E-57	Treg	Eva1b
Tap1	1.49E-60	0.687532	0.643	0.523	3.65E-56	Treg	Tap1
Ubb	4.06E-60	0.278648	0.988	0.952	9.94E-56	Treg	Ubb
Smco4	4.81E-60	1.083901	0.358	0.216	1.18E-55	Treg	Smco4
Ypel31	5.02E-60	0.580383	0.731	0.62	1.23E-55	Treg	Ypel3
Plaat3	1.41E-59	0.720451	0.62	0.506	3.46E-55	Treg	Plaat3
Actg1	2.37E-59	0.401125	0.971	0.925	5.81E-55	Treg	Actg1
Btg11	3.11E-59	0.48083	0.843	0.764	7.61E-55	Treg	Btg1
BE6920071	4.41E-59	0.967581	0.341	0.196	1.08E-54	Treg	BE692007
Mif4gd	5.49E-59	1.069539	0.413	0.276	1.34E-54	Treg	Mif4gd
Rpl341	1.83E-58	0.258573	0.993	0.949	4.47E-54	Treg	Rpl34
Gm20275	2.15E-58	1.028465	0.275	0.146	5.26E-54	Treg	Gm20275
Aopep	1.83E-57	0.608855	0.605	0.469	4.48E-53	Treg	Aopep
Akap13	2.70E-56	0.525759	0.738	0.651	6.60E-52	Treg	Akap13
Rpl37a1	7.00E-56	0.25309	0.992	0.951	1.71E-51	Treg	Rpl37a
Gimap31	7.30E-56	0.467557	0.806	0.69	1.79E-51	Treg	Gimap3
Cnn21	7.55E-56	0.452407	0.82	0.72	1.85E-51	Treg	Cnn2
Mgst2	2.34E-55	1.183074	0.312	0.182	5.73E-51	Treg	Mgst2
2410006H1	6.39E-55	0.717479	0.666	0.561	1.56E-50	Treg	2410006H16Rik
Cd247	8.76E-55	0.548006	0.595	0.448	2.15E-50	Treg	Cd247

Gimap71	1.21E-53	0.666349	0.623	0.494	2.97E-49	Treg	Gimap7
Pfdn51	2.08E-53	0.329704	0.912	0.846	5.11E-49	Treg	Pfdn5
Leprotl11	2.60E-53	0.569958	0.705	0.616	6.37E-49	Treg	Leprotl1
Tnfrsf1b	3.95E-53	1.142731	0.413	0.292	9.66E-49	Treg	Tnfrsf1b
Camk4	6.21E-53	0.74458	0.452	0.31	1.52E-48	Treg	Camk4
Socs2	1.23E-52	1.203458	0.288	0.164	3.02E-48	Treg	Socs2
Socs1	1.91E-52	1.031435	0.525	0.404	4.67E-48	Treg	Socs1
Psen2	9.21E-52	1.185587	0.353	0.23	2.25E-47	Treg	Psen2
Ddit4	6.11E-51	0.945519	0.424	0.293	1.50E-46	Treg	Ddit4
Slamf1	1.05E-50	0.869545	0.305	0.175	2.58E-46	Treg	Slamf1
Vps54	4.56E-50	0.873441	0.416	0.288	1.12E-45	Treg	Vps54
Cytip	1.05E-49	0.692365	0.605	0.486	2.56E-45	Treg	Cytip
Sntb1	1.58E-49	0.648254	0.408	0.266	3.86E-45	Treg	Sntb1
Il2rg1	2.55E-49	0.401307	0.84	0.75	6.25E-45	Treg	Il2rg
Cd271	4.78E-49	1.040587	0.393	0.267	1.17E-44	Treg	Cd27
Itgb71	1.44E-47	0.611639	0.647	0.537	3.54E-43	Treg	Itgb7
Ucp2	6.56E-47	0.446518	0.875	0.821	1.61E-42	Treg	Ucp2
Eif3f1	1.25E-46	0.327795	0.883	0.829	3.07E-42	Treg	Eif3f
Isg15	1.77E-45	1.517624	0.414	0.296	4.34E-41	Treg	Isg15
Gbp7	2.35E-45	1.062503	0.39	0.275	5.75E-41	Treg	Gbp7
Phf11b	2.61E-45	1.14586	0.405	0.289	6.39E-41	Treg	Phf11b
Ifi471	3.17E-45	0.750268	0.621	0.528	7.77E-41	Treg	Ifi47
Psme2	9.33E-45	0.478039	0.823	0.741	2.28E-40	Treg	Psme2
Batf	6.03E-44	1.193633	0.325	0.212	1.48E-39	Treg	Batf
Cd37	6.78E-44	0.458894	0.78	0.713	1.66E-39	Treg	Cd37
Ptpn181	9.61E-44	0.313777	0.906	0.845	2.35E-39	Treg	Ptpn18
Arf5	1.04E-43	0.427748	0.759	0.685	2.55E-39	Treg	Arf5
Peli1	1.09E-43	0.640235	0.595	0.504	2.67E-39	Treg	Peli1
Gimap41	2.97E-43	0.38135	0.821	0.701	7.27E-39	Treg	Gimap4
Rilpl2	1.62E-42	1.369297	0.31	0.203	3.97E-38	Treg	Rilpl2
Abracl	8.07E-42	0.550376	0.646	0.557	1.98E-37	Treg	Abracl
Cdk2ap2	1.28E-41	0.544726	0.676	0.59	3.15E-37	Treg	Cdk2ap2
Ttc39c	2.97E-41	0.940658	0.296	0.183	7.27E-37	Treg	Ttc39c
Ms4a6b1	7.91E-41	0.500288	0.702	0.617	1.94E-36	Treg	Ms4a6b
Crlf2	8.41E-41	0.827883	0.472	0.368	2.06E-36	Treg	Crlf2
Pnrc1	1.35E-40	0.502771	0.682	0.605	3.30E-36	Treg	Pnrc1
Slfn21	2.04E-40	0.510809	0.701	0.614	4.99E-36	Treg	Slfn2
Odc1	2.15E-40	1.056954	0.42	0.314	5.26E-36	Treg	Odc1
Rtp41	6.92E-40	0.94693	0.435	0.324	1.70E-35	Treg	Rtp4
Cd691	7.57E-40	0.867586	0.484	0.367	1.85E-35	Treg	Cd69
Ets1	8.54E-40	0.386499	0.723	0.621	2.09E-35	Treg	Ets1
Myl12a	9.09E-39	0.362578	0.83	0.758	2.23E-34	Treg	Myl12a
Prkca	1.44E-38	0.497957	0.553	0.444	3.52E-34	Treg	Prkca
Sri	1.49E-38	0.483666	0.691	0.617	3.64E-34	Treg	Sri

Sh2d1a1	2.28E-38	0.773481	0.408	0.287	5.58E-34	Treg	Sh2d1a
Ifi209	7.57E-38	0.946737	0.395	0.286	1.85E-33	Treg	Ifi209
Prr131	3.67E-37	0.478424	0.661	0.585	8.99E-33	Treg	Prr13
Sdhaf1	3.77E-37	1.216194	0.276	0.177	9.23E-33	Treg	Sdhaf1
Prkch	8.00E-37	0.35671	0.723	0.634	1.96E-32	Treg	Prkch
Irf7	3.02E-36	0.913214	0.426	0.32	7.39E-32	Treg	Irf7
Rbm38	4.00E-36	0.896892	0.374	0.271	9.80E-32	Treg	Rbm38
Tap2	2.85E-35	0.543507	0.625	0.55	6.99E-31	Treg	Tap2
Arhgdib1	3.02E-35	0.256049	0.936	0.881	7.41E-31	Treg	Arhgdib
Bcl2a1b	9.22E-35	0.746831	0.483	0.372	2.26E-30	Treg	Bcl2a1b
Ifngr1	1.68E-34	0.648689	0.732	0.736	4.12E-30	Treg	Ifngr1
Gapdh	2.41E-34	0.342974	0.82	0.75	5.91E-30	Treg	Gapdh
Nxpe3	4.58E-34	0.776871	0.275	0.172	1.12E-29	Treg	Nxpe3
Ldha	5.71E-34	0.375818	0.776	0.699	1.40E-29	Treg	Ldha
Myl12b1	7.50E-34	0.293213	0.894	0.847	1.84E-29	Treg	Myl12b
2310001H1	1.35E-33	0.971991	0.332	0.23	3.30E-29	Treg	2310001H17Rik
Spint2	4.01E-33	0.768793	0.415	0.313	9.82E-29	Treg	Spint2
Psme1	1.31E-32	0.360669	0.837	0.781	3.21E-28	Treg	Psme1
Vim	1.38E-32	0.554618	0.775	0.711	3.37E-28	Treg	Vim
Ccnd21	1.68E-32	0.52714	0.61	0.515	4.12E-28	Treg	Ccnd2
Mcl1	1.75E-32	0.489818	0.635	0.562	4.28E-28	Treg	Mcl1
P2ry10	3.59E-32	0.797801	0.281	0.181	8.79E-28	Treg	P2ry10
Lsp11	5.09E-32	0.33221	0.753	0.674	1.25E-27	Treg	Lsp1
Coro2a	7.84E-32	0.54246	0.584	0.496	1.92E-27	Treg	Coro2a
Grap1	8.86E-32	0.671281	0.443	0.345	2.17E-27	Treg	Grap
Mmd	9.17E-32	1.188787	0.26	0.171	2.24E-27	Treg	Mmd
Lbh	9.39E-32	0.882844	0.348	0.252	2.30E-27	Treg	Lbh
Nrip1	1.20E-31	0.809758	0.411	0.317	2.94E-27	Treg	Nrip1
Ubl5	4.16E-31	0.364301	0.737	0.683	1.02E-26	Treg	Ubl5
Traf1	9.92E-31	0.678067	0.411	0.306	2.43E-26	Treg	Traf1
Tmem154	8.39E-30	0.712817	0.298	0.199	2.06E-25	Treg	Tmem154
Btf3	9.42E-30	0.260337	0.894	0.847	2.31E-25	Treg	Btf3
Aldoa	1.02E-29	0.392069	0.801	0.732	2.50E-25	Treg	Aldoa
Vamp8	3.12E-29	0.49485	0.629	0.554	7.63E-25	Treg	Vamp8
Dusp5	6.09E-29	0.738889	0.303	0.208	1.49E-24	Treg	Dusp5
Uqcrh1	1.89E-28	0.269481	0.84	0.802	4.62E-24	Treg	Uqcrh
Glcci1	2.18E-28	0.896417	0.27	0.179	5.35E-24	Treg	Glcci1
Xaf1	2.23E-28	0.870007	0.378	0.289	5.46E-24	Treg	Xaf1
Ifi2131	3.74E-28	0.862912	0.32	0.227	9.15E-24	Treg	Ifi213
Fis1	4.46E-28	0.382059	0.698	0.642	1.09E-23	Treg	Fis1
Mdfic	1.27E-27	0.5256	0.387	0.291	3.10E-23	Treg	Mdfic
Arhgef1	5.18E-27	0.387291	0.644	0.574	1.27E-22	Treg	Arhgef1
Tle51	6.21E-27	0.252026	0.848	0.798	1.52E-22	Treg	Tle5
Zap701	8.26E-27	0.479052	0.52	0.421	2.02E-22	Treg	Zap70

Gpi1	8.89E-27	0.407421	0.678	0.61	2.18E-22	Treg	Gpi1
Gbp4	1.01E-26	0.79337	0.357	0.268	2.47E-22	Treg	Gbp4
Fam3c	1.65E-26	0.994568	0.3	0.216	4.05E-22	Treg	Fam3c
Gimap61	2.10E-26	0.309171	0.719	0.634	5.15E-22	Treg	Gimap6
1110008P1	2.36E-26	0.653171	0.418	0.328	5.77E-22	Treg	1110008P14Rik
Il2rb	2.54E-26	0.27583	0.723	0.608	6.22E-22	Treg	Il2rb
Ppp1r181	4.58E-26	0.349136	0.719	0.667	1.12E-21	Treg	Ppp1r18
S100a131	4.70E-26	0.452537	0.611	0.544	1.15E-21	Treg	S100a13
Bst2	8.20E-26	0.572114	0.605	0.523	2.01E-21	Treg	Bst2
Tmem50a	1.35E-25	0.327638	0.747	0.694	3.31E-21	Treg	Tmem50a
Parp14	8.03E-25	0.83843	0.362	0.279	1.97E-20	Treg	Parp14
Junb	8.22E-25	0.377144	0.757	0.682	2.01E-20	Treg	Junb
Elob	1.20E-24	0.287824	0.829	0.767	2.94E-20	Treg	Elob
Polr1d	1.57E-24	0.353045	0.694	0.633	3.85E-20	Treg	Polr1d
Mrpl52	1.87E-24	0.412993	0.637	0.57	4.59E-20	Treg	Mrpl52
H1f0	2.39E-24	0.82083	0.291	0.208	5.84E-20	Treg	H1f0
Gstp11	2.88E-24	0.392417	0.665	0.607	7.06E-20	Treg	Gstp1
Lrp10	2.92E-24	0.524207	0.515	0.449	7.14E-20	Treg	Lrp10
Ifi2061	3.32E-24	0.80038	0.321	0.236	8.14E-20	Treg	Ifi206
Clec2d1	4.16E-24	0.384411	0.669	0.616	1.02E-19	Treg	Clec2d
Fkbp1a	1.37E-23	0.473677	0.586	0.53	3.36E-19	Treg	Fkbp1a
Rbpj	2.08E-23	0.592575	0.464	0.389	5.09E-19	Treg	Rbpj
Itgav	3.04E-23	0.924202	0.328	0.252	7.44E-19	Treg	Itgav
Jak2	3.49E-23	0.711908	0.34	0.258	8.55E-19	Treg	Jak2
Tank	4.82E-23	0.740812	0.331	0.252	1.18E-18	Treg	Tank
Coro1b	6.93E-23	0.549952	0.501	0.434	1.70E-18	Treg	Coro1b
Edf1	1.02E-22	0.343234	0.736	0.674	2.50E-18	Treg	Edf1
Ptpn7	1.24E-22	0.763907	0.342	0.262	3.04E-18	Treg	Ptpn7
Kcnn4	1.54E-22	0.652199	0.337	0.253	3.76E-18	Treg	Kcnn4
Gas5	2.52E-22	0.357033	0.721	0.672	6.17E-18	Treg	Gas5
Gimap51	3.27E-22	0.462077	0.548	0.462	8.00E-18	Treg	Gimap5
Gimap91	6.60E-22	0.567402	0.424	0.342	1.62E-17	Treg	Gimap9
Cxcr31	8.55E-22	0.355413	0.585	0.496	2.09E-17	Treg	Cxcr3
Rap1b	1.11E-21	0.289291	0.762	0.734	2.73E-17	Treg	Rap1b
Tapbp	1.27E-21	0.489474	0.605	0.555	3.11E-17	Treg	Tapbp
Hopx1	1.70E-21	0.473004	0.565	0.481	4.17E-17	Treg	Hopx
Trbc21	3.39E-21	0.420281	0.512	0.425	8.30E-17	Treg	Trbc2
Il27ra1	4.33E-21	0.505147	0.424	0.342	1.06E-16	Treg	Il27ra
S100a41	4.36E-21	0.447688	0.528	0.434	1.07E-16	Treg	S100a4
Trp53i11	5.42E-21	0.729637	0.25	0.173	1.33E-16	Treg	Trp53i11
Il7r1	8.12E-21	0.393378	0.506	0.415	1.99E-16	Treg	Il7r
Supt4a1	2.78E-20	0.426324	0.574	0.525	6.81E-16	Treg	Supt4a
Sla	4.73E-20	0.53364	0.467	0.4	1.16E-15	Treg	Sla
Pkm	5.74E-20	0.309396	0.711	0.646	1.41E-15	Treg	Pkm

Jak3	1.04E-19	0.794617	0.275	0.204	2.55E-15	Treg	Jak3
Trim30a	1.51E-19	0.657134	0.403	0.332	3.69E-15	Treg	Trim30a
Tgtp2	1.89E-19	0.895883	0.282	0.21	4.64E-15	Treg	Tgtp2
Ifi35	3.43E-19	0.713071	0.419	0.359	8.40E-15	Treg	Ifi35
Ube2b	3.66E-19	0.41745	0.559	0.51	8.95E-15	Treg	Ube2b
Chmp4b	3.73E-19	0.406459	0.623	0.575	9.13E-15	Treg	Chmp4b
Irgm1	5.68E-19	0.768414	0.357	0.294	1.39E-14	Treg	Irgm1
Tbc1d10c1	5.82E-19	0.338486	0.598	0.533	1.43E-14	Treg	Tbc1d10c
Eif3h	5.98E-19	0.27324	0.774	0.733	1.46E-14	Treg	Eif3h
Ptger4	6.52E-19	0.719094	0.279	0.207	1.60E-14	Treg	Ptger4
1600014C1	7.10E-19	0.792409	0.311	0.242	1.74E-14	Treg	1600014C10Rik
Mrps6	2.57E-18	0.756719	0.28	0.212	6.29E-14	Treg	Mrps6
Rnaset2a	3.00E-18	0.742074	0.323	0.258	7.34E-14	Treg	Rnaset2a
Dtx3l	4.99E-18	0.841325	0.272	0.206	1.22E-13	Treg	Dtx3l
Agfg1	5.26E-18	0.599984	0.353	0.285	1.29E-13	Treg	Agfg1
Rap1a	9.43E-18	0.340452	0.625	0.578	2.31E-13	Treg	Rap1a
Snx20	9.48E-18	0.697411	0.337	0.274	2.32E-13	Treg	Snx20
Ubash3a1	1.95E-17	0.442286	0.402	0.323	4.77E-13	Treg	Ubash3a
Scand11	2.35E-17	0.304235	0.624	0.579	5.76E-13	Treg	Scand1
Rapgef6	2.46E-17	0.306386	0.607	0.566	6.02E-13	Treg	Rapgef6
Sec61g	2.68E-17	0.295131	0.668	0.619	6.57E-13	Treg	Sec61g
Cdc42se2	4.34E-17	0.465534	0.486	0.437	1.06E-12	Treg	Cdc42se2
Mettl231	6.02E-17	0.453997	0.477	0.425	1.47E-12	Treg	Mettl23
Rinl1	6.04E-17	0.294753	0.631	0.58	1.48E-12	Treg	Rinl
Gm26740	7.39E-17	0.318543	0.489	0.417	1.81E-12	Treg	Gm26740
Prdx2	8.28E-17	0.351582	0.52	0.467	2.03E-12	Treg	Prdx2
Tomm71	1.15E-16	0.313452	0.659	0.609	2.81E-12	Treg	Tomm7
Itm2c	1.26E-16	0.403223	0.511	0.441	3.09E-12	Treg	Itm2c
Pigx	1.85E-16	0.645386	0.389	0.336	4.54E-12	Treg	Pigx
Map1lc3b	4.50E-16	0.404215	0.571	0.528	1.10E-11	Treg	Map1lc3b
Sh2d2a1	4.63E-16	0.310409	0.54	0.466	1.13E-11	Treg	Sh2d2a
Malt1	7.14E-16	0.528481	0.382	0.315	1.75E-11	Treg	Malt1
Cers4	7.40E-16	0.571847	0.267	0.2	1.81E-11	Treg	Cers4
Stk17b1	8.77E-16	0.265739	0.632	0.58	2.15E-11	Treg	Stk17b
Vasp	1.06E-15	0.434942	0.541	0.503	2.59E-11	Treg	Vasp
Pold4	1.54E-15	0.655756	0.36	0.304	3.76E-11	Treg	Pold4
Cd82	1.78E-15	0.447993	0.609	0.577	4.36E-11	Treg	Cd82
Rhof	1.78E-15	0.558511	0.316	0.248	4.37E-11	Treg	Rhof
Slfn8	2.03E-15	0.744917	0.297	0.236	4.96E-11	Treg	Slfn8
Prelid1	2.10E-15	0.296325	0.633	0.58	5.15E-11	Treg	Prelid1
Bmyc	2.16E-15	1.132989	0.258	0.204	5.30E-11	Treg	Bmyc
Spcs1	3.06E-15	0.365724	0.591	0.554	7.49E-11	Treg	Spcs1
Spcs2	4.12E-15	0.32134	0.631	0.59	1.01E-10	Treg	Spcs2
Sit11	4.40E-15	0.54079	0.264	0.195	1.08E-10	Treg	Sit1

Arid5b	4.59E-15	0.542452	0.395	0.34	1.12E-10	Treg	Arid5b
Prkar1a	4.97E-15	0.293375	0.628	0.591	1.22E-10	Treg	Prkar1a
Naa20	5.31E-15	0.687107	0.349	0.298	1.30E-10	Treg	Naa20
Tnfaip3	5.87E-15	0.366149	0.416	0.348	1.44E-10	Treg	Tnfaip3
Gabarap	5.94E-15	0.268939	0.72	0.674	1.45E-10	Treg	Gabarap
Selenok	6.24E-15	0.301889	0.651	0.61	1.53E-10	Treg	Selenok
Cdipt	7.30E-15	0.774434	0.298	0.242	1.79E-10	Treg	Cdipt
Arhgap31	8.64E-15	0.691352	0.282	0.223	2.12E-10	Treg	Arhgap31
Pdlim2	1.03E-14	0.591995	0.315	0.254	2.53E-10	Treg	Pdlim2
Anp32a	1.36E-14	0.336122	0.594	0.554	3.32E-10	Treg	Anp32a
Tmem258	1.79E-14	0.314332	0.617	0.575	4.38E-10	Treg	Tmem258
Srp14	1.86E-14	0.339516	0.583	0.545	4.57E-10	Treg	Srp14
Ankrd12	3.09E-14	0.406144	0.459	0.41	7.56E-10	Treg	Ankrd12
Nmi	3.28E-14	0.581341	0.385	0.333	8.04E-10	Treg	Nmi
Gbp9	3.53E-14	0.757541	0.278	0.219	8.64E-10	Treg	Gbp9
Parp9	3.83E-14	0.819133	0.281	0.226	9.39E-10	Treg	Parp9
Borcs8	5.21E-14	0.608426	0.324	0.268	1.28E-09	Treg	Borcs8
Fam129a	5.31E-14	0.48665	0.34	0.283	1.30E-09	Treg	Fam129a
Sp1001	7.90E-14	0.280205	0.675	0.653	1.94E-09	Treg	Sp100
Rtraf	9.08E-14	0.324513	0.579	0.539	2.22E-09	Treg	Rtraf
Aprt1	1.44E-13	0.386957	0.506	0.458	3.52E-09	Treg	Aprt
Rnaset2b	2.20E-13	0.524705	0.405	0.358	5.38E-09	Treg	Rnaset2b
Pmepa1	2.50E-13	0.468979	0.417	0.356	6.13E-09	Treg	Pmepa1
Pttg11	2.66E-13	0.577059	0.407	0.36	6.51E-09	Treg	Pttg1
Slc9a3r11	3.17E-13	0.406849	0.534	0.501	7.76E-09	Treg	Slc9a3r1
Stat5b	3.32E-13	0.506935	0.313	0.256	8.12E-09	Treg	Stat5b
Sep-91	3.44E-13	0.338835	0.545	0.507	8.44E-09	Treg	9-Sep
Lpxn1	3.56E-13	0.388911	0.457	0.409	8.72E-09	Treg	Lpxn
Ddx24	3.59E-13	0.418159	0.52	0.488	8.80E-09	Treg	Ddx24
Irf1	3.66E-13	0.506467	0.473	0.43	8.96E-09	Treg	Irf1
Ppdpf1	4.97E-13	0.466898	0.46	0.417	1.22E-08	Treg	Ppdpf
Zdhhc20	5.35E-13	0.414957	0.462	0.418	1.31E-08	Treg	Zdhhc20
S100a61	6.30E-13	0.339087	0.698	0.625	1.54E-08	Treg	S100a6
Gimap8	7.26E-13	0.350128	0.455	0.396	1.78E-08	Treg	Gimap8
Rnf114	1.05E-12	0.517598	0.394	0.346	2.58E-08	Treg	Rnf114
Dgka1	1.57E-12	0.328005	0.354	0.286	3.86E-08	Treg	Dgka
Rnf125	1.65E-12	0.541718	0.277	0.219	4.03E-08	Treg	Rnf125
Cyth1	1.70E-12	0.331401	0.359	0.3	4.17E-08	Treg	Cyth1
Samd9l	1.84E-12	0.598449	0.28	0.223	4.51E-08	Treg	Samd9l
Trerf1	2.16E-12	0.302168	0.389	0.326	5.28E-08	Treg	Trerf1
Ubl3	4.95E-12	0.531532	0.382	0.335	1.21E-07	Treg	Ubl3
Aup1	7.06E-12	0.357176	0.54	0.51	1.73E-07	Treg	Aup1
Itpkb	7.59E-12	0.36483	0.41	0.358	1.86E-07	Treg	Itpkb
Mea1	8.28E-12	0.533091	0.387	0.346	2.03E-07	Treg	Mea1

Ramp1	1.14E-11	0.502078	0.321	0.265	2.79E-07	Treg	Ramp1
Tspan13	1.26E-11	0.464944	0.407	0.358	3.08E-07	Treg	Tspan13
Csnk2b	1.37E-11	0.305316	0.612	0.581	3.37E-07	Treg	Csnk2b
Acp51	1.72E-11	0.309663	0.429	0.369	4.22E-07	Treg	Acp5
Rhog	2.64E-11	0.317089	0.575	0.539	6.46E-07	Treg	Rhog
Paip21	3.00E-11	0.362761	0.488	0.453	7.36E-07	Treg	Paip2
Reep5	3.12E-11	0.251256	0.667	0.625	7.65E-07	Treg	Reep5
Glipr1	4.37E-11	0.609444	0.318	0.27	1.07E-06	Treg	Glipr1
Zc3hav1	5.86E-11	0.339005	0.492	0.461	1.43E-06	Treg	Zc3hav1
Snx2	7.01E-11	0.658456	0.334	0.295	1.72E-06	Treg	Snx2
Fkbp31	7.12E-11	0.287607	0.541	0.504	1.74E-06	Treg	Fkbp3
CAAA0114	7.31E-11	0.276573	0.607	0.557	1.79E-06	Treg	CAAA01147332.1
Ighm	8.00E-11	0.938809	0.268	0.228	1.96E-06	Treg	Ighm
Hcls1	8.26E-11	0.368189	0.491	0.451	2.02E-06	Treg	Hcls1
Bnip3l	8.58E-11	0.382963	0.468	0.434	2.10E-06	Treg	Bnip3l
Chchd2	8.92E-11	0.263116	0.596	0.563	2.18E-06	Treg	Chchd2
Tes	1.04E-10	0.356603	0.468	0.426	2.54E-06	Treg	Tes
Gmfg1	1.09E-10	0.258204	0.629	0.612	2.68E-06	Treg	Gmfg
Sh3bgrl	1.43E-10	0.636616	0.282	0.237	3.51E-06	Treg	Sh3bgrl
Lrba	1.50E-10	0.336386	0.311	0.258	3.68E-06	Treg	Lrba
Mapkapk3	1.70E-10	0.49546	0.336	0.289	4.17E-06	Treg	Mapkapk3
Slc1a5	1.77E-10	0.386968	0.372	0.322	4.34E-06	Treg	Slc1a5
Tmem173	1.99E-10	0.513993	0.357	0.315	4.87E-06	Treg	Tmem173
Snw1	2.19E-10	0.534825	0.374	0.338	5.36E-06	Treg	Snw1
Gbp8	2.98E-10	0.509884	0.276	0.225	7.30E-06	Treg	Gbp8
Ebpl	3.39E-10	0.616571	0.254	0.207	8.30E-06	Treg	Ebpl
Mat2b	3.93E-10	0.44772	0.394	0.355	9.63E-06	Treg	Mat2b
Dynlrb1	4.18E-10	0.347023	0.474	0.443	1.02E-05	Treg	Dynlrb1
Ncf4	4.30E-10	0.468757	0.375	0.332	1.05E-05	Treg	Ncf4
Nt5c	6.36E-10	0.413774	0.467	0.436	1.56E-05	Treg	Nt5c
Hnrnpll	7.77E-10	0.602711	0.29	0.248	1.90E-05	Treg	Hnrnpll
Skil	8.46E-10	0.610619	0.281	0.238	2.07E-05	Treg	Skil
Mrpl54	1.09E-09	0.469288	0.381	0.343	2.68E-05	Treg	Mrpl54
Smc4	1.35E-09	0.401363	0.4	0.349	3.32E-05	Treg	Smc4
Cd53	1.37E-09	0.293628	0.587	0.569	3.36E-05	Treg	Cd53
3830406C1	1.57E-09	0.623838	0.261	0.219	3.85E-05	Treg	3830406C13Rik
Tnip1	1.75E-09	0.561918	0.257	0.212	4.29E-05	Treg	Tnip1
Tsc22d4	1.85E-09	0.294617	0.505	0.477	4.54E-05	Treg	Tsc22d4
Ifi2081	2.37E-09	0.671967	0.275	0.23	5.80E-05	Treg	Ifi208
Irf2	2.67E-09	0.33803	0.458	0.426	6.53E-05	Treg	Irf2
Med28	2.93E-09	0.294521	0.561	0.542	7.18E-05	Treg	Med28
Serpinb6a	3.72E-09	0.559571	0.266	0.222	9.11E-05	Treg	Serpinb6a
Zfp706	3.78E-09	0.30808	0.578	0.552	9.25E-05	Treg	Zfp706
Syngr2	4.41E-09	0.331567	0.444	0.402	0.000108	Treg	Syngr2

Mxd41	4.84E-09	0.438785	0.355	0.311	0.000119	Treg	Mxd4
H13	5.62E-09	0.367567	0.453	0.422	0.000138	Treg	H13
Birc3	8.20E-09	0.452425	0.318	0.275	0.000201	Treg	Birc3
Ciao2b	9.75E-09	0.525013	0.368	0.34	0.000239	Treg	Ciao2b
Wdr1	1.19E-08	0.31237	0.515	0.492	0.000291	Treg	Wdr1
Cnp1	1.30E-08	0.481726	0.362	0.327	0.000319	Treg	Cnp
Lypla21	1.88E-08	0.4002	0.411	0.382	0.000461	Treg	Lypla2
Tbrg11	2.04E-08	0.420143	0.375	0.338	0.000501	Treg	Tbrg1
Bloc1s1	2.06E-08	0.421499	0.385	0.354	0.000505	Treg	Bloc1s1
Traf3ip3	2.14E-08	0.347929	0.364	0.323	0.000525	Treg	Traf3ip3
Foxo1	2.50E-08	0.341903	0.308	0.265	0.000613	Treg	Foxo1
Sumo1	3.00E-08	0.251357	0.559	0.536	0.000735	Treg	Sumo1
Gbp2	3.89E-08	0.580882	0.27	0.228	0.000951	Treg	Gbp2
Mrps161	4.13E-08	0.381335	0.459	0.435	0.001011	Treg	Mrps16
Arpc5	5.24E-08	0.255375	0.625	0.616	0.001283	Treg	Arpc5
R3hdm4	1.23E-07	0.421594	0.362	0.332	0.003006	Treg	R3hdm4
Slc50a1	1.55E-07	0.389308	0.431	0.41	0.003794	Treg	Slc50a1
Gm2a	1.74E-07	0.53091	0.281	0.244	0.004261	Treg	Gm2a
Acap11	1.88E-07	0.270645	0.429	0.389	0.004594	Treg	Acap1
Casp8	1.97E-07	0.386676	0.366	0.335	0.004819	Treg	Casp8
Cox14	2.04E-07	0.432226	0.429	0.415	0.004988	Treg	Cox14
Cdk6	2.46E-07	0.388669	0.283	0.248	0.006016	Treg	Cdk6
Irf9	2.49E-07	0.55382	0.265	0.231	0.006088	Treg	Irf9
Eif3m	2.61E-07	0.288575	0.515	0.5	0.006388	Treg	Eif3m
Rab4b	2.69E-07	0.422001	0.408	0.383	0.006583	Treg	Rab4b
Ccng1	2.94E-07	0.496746	0.33	0.298	0.007193	Treg	Ccng1
Resf1	4.23E-07	0.576702	0.333	0.307	0.010352	Treg	Resf1
Ogfr	4.26E-07	0.620198	0.28	0.253	0.010434	Treg	Ogfr
Sgms1	4.48E-07	0.293271	0.261	0.22	0.010983	Treg	Sgms1
Ech11	4.56E-07	0.31172	0.459	0.438	0.011179	Treg	Ech1
Pld3	4.61E-07	0.251544	0.487	0.457	0.011284	Treg	Pld3
Trim14	5.52E-07	0.305457	0.364	0.329	0.013514	Treg	Trim14
Ube2v1	6.04E-07	0.3203	0.466	0.45	0.014786	Treg	Ube2v1
Hsbp1	6.18E-07	0.339307	0.429	0.411	0.015138	Treg	Hsbp1
Timp2	7.73E-07	0.369221	0.271	0.23	0.018921	Treg	Timp2
Rnf138	8.95E-07	0.413042	0.332	0.299	0.021925	Treg	Rnf138
Sec62	1.02E-06	0.291099	0.476	0.458	0.02508	Treg	Sec62
Il21r	1.15E-06	0.319909	0.39	0.362	0.028219	Treg	Il21r
1810026B0	1.21E-06	0.313053	0.424	0.402	0.029514	Treg	1810026B05Rik
Tpp2	1.22E-06	0.475822	0.336	0.31	0.029967	Treg	Tpp2
Orai1	1.62E-06	0.647863	0.321	0.295	0.03956	Treg	Orai1
Lgals9	2.10E-06	0.269282	0.545	0.521	0.051421	Treg	Lgals9
Unc119	2.16E-06	0.535233	0.255	0.222	0.052845	Treg	Unc119
Lamtor4	2.30E-06	0.296626	0.488	0.473	0.05632	Treg	Lamtor4

Arf6	2.41E-06	0.275333	0.501	0.488	0.059075	Treg	Arf6
Pglyrp21	2.84E-06	0.316845	0.314	0.275	0.069441	Treg	Pglyrp2
Kxd1	3.13E-06	0.455912	0.348	0.325	0.076722	Treg	Kxd1
Dennd1c	3.38E-06	0.370289	0.317	0.284	0.082831	Treg	Dennd1c
B3gnt2	3.93E-06	0.442438	0.318	0.292	0.096189	Treg	B3gnt2
Chic2	4.78E-06	0.32973	0.402	0.381	0.117165	Treg	Chic2
Capn1	5.11E-06	0.45541	0.266	0.235	0.125231	Treg	Capn1
Acot7	5.30E-06	0.450971	0.289	0.26	0.129865	Treg	Acot7
Fam104a	6.29E-06	0.324902	0.38	0.358	0.153955	Treg	Fam104a
Rwdd1	7.17E-06	0.27973	0.449	0.434	0.175498	Treg	Rwdd1
Ubl7	7.49E-06	0.626294	0.252	0.227	0.183504	Treg	Ubl7
Phc3	8.07E-06	0.37903	0.275	0.245	0.19754	Treg	Phc3
Nfatc1	8.20E-06	0.329342	0.372	0.348	0.200876	Treg	Nfatc1
Psmb101	8.42E-06	0.289195	0.577	0.566	0.206115	Treg	Psmb10
Lrrc8c	9.40E-06	0.267105	0.353	0.325	0.230078	Treg	Lrrc8c
Crtc3	1.01E-05	0.300574	0.275	0.242	0.24788	Treg	Crtc3
Stat3	1.11E-05	0.26683	0.427	0.406	0.271249	Treg	Stat3
Il10ra	1.14E-05	0.317559	0.381	0.35	0.278966	Treg	Il10ra
Plgrkt	1.15E-05	0.371426	0.362	0.343	0.280747	Treg	Plgrkt
Chchd10	1.27E-05	0.28116	0.277	0.241	0.309962	Treg	Chchd10
H2-M3	1.56E-05	0.388855	0.366	0.344	0.381011	Treg	H2-M3
Mthfsl	1.61E-05	0.458635	0.27	0.242	0.393529	Treg	Mthfsl
Tmem1341	2.05E-05	0.31023	0.435	0.424	0.501358	Treg	Tmem134
4930523C(	2.49E-05	0.276836	0.356	0.328	0.608677	Treg	4930523C07Rik
Trim12c	2.85E-05	0.432169	0.265	0.237	0.6983	Treg	Trim12c
Ddx58	2.93E-05	0.530433	0.262	0.235	0.718189	Treg	Ddx58
Map4k1	3.43E-05	0.387917	0.251	0.222	0.840177	Treg	Map4k1
Thap31	3.75E-05	0.3636	0.285	0.257	0.919557	Treg	Thap3
Atp11b	3.91E-05	0.362907	0.349	0.327	0.956974	Treg	Atp11b
Retreg1	4.71E-05	0.28169	0.312	0.285	1	Treg	Retreg1
Chmp2a	5.40E-05	0.302703	0.453	0.442	1	Treg	Chmp2a
Pgk1	6.02E-05	0.285173	0.438	0.416	1	Treg	Pgk1
Ncoa3	6.17E-05	0.372618	0.28	0.258	1	Treg	Ncoa3
Sys1	6.37E-05	0.315922	0.414	0.406	1	Treg	Sys1
Il161	6.59E-05	0.278834	0.397	0.381	1	Treg	Il16
Zdhhc181	9.56E-05	0.279237	0.38	0.357	1	Treg	Zdhhc18
Vmp1	0.000112	0.443297	0.327	0.311	1	Treg	Vmp1
Fgl21	0.000127	0.316017	0.444	0.433	1	Treg	Fgl2
Chd31	0.000137	0.293972	0.387	0.368	1	Treg	Chd3
Agap2	0.00014	0.277189	0.26	0.231	1	Treg	Agap2
Arf4	0.000142	0.294065	0.472	0.466	1	Treg	Arf4
Cast	0.000151	0.252759	0.357	0.336	1	Treg	Cast
Zmat5	0.000193	0.394151	0.305	0.286	1	Treg	Zmat5
AI4676061	0.000211	0.376	0.302	0.281	1	Treg	AI467606

Cyld	0.00039	0.289094	0.368	0.354	1	Treg	Cyld
Idnk1	0.000464	0.296404	0.383	0.377	1	Treg	Idnk
Supt20	0.000481	0.370434	0.289	0.27	1	Treg	Supt20
Svbp	0.000525	0.368813	0.33	0.316	1	Treg	Svbp
Rasa1	0.000533	0.307317	0.311	0.292	1	Treg	Rasa1
Tnfsf10	0.000625	0.252019	0.254	0.226	1	Treg	Tnfsf10
Sin3b	0.000683	0.326496	0.393	0.393	1	Treg	Sin3b
Psma4	0.000697	0.31882	0.386	0.377	1	Treg	Psma4
Mvb12a	0.00079	0.379874	0.31	0.298	1	Treg	Mvb12a
Nop53	0.000877	0.342115	0.362	0.356	1	Treg	Nop53
Dhrs7	0.000944	0.446611	0.251	0.235	1	Treg	Dhrs7
Tmem123	0.001057	0.371709	0.272	0.255	1	Treg	Tmem123
Fundc2	0.001154	0.330108	0.348	0.34	1	Treg	Fundc2
Dctn6	0.001169	0.366055	0.334	0.325	1	Treg	Dctn6
Susd3	0.001351	0.371219	0.316	0.301	1	Treg	Susd3
Aebp2	0.001469	0.310627	0.274	0.257	1	Treg	Aebp2
Mien1	0.001718	0.282325	0.374	0.367	1	Treg	Mien1
Lamtor5	0.001755	0.349447	0.335	0.329	1	Treg	Lamtor5
Ythdc1	0.001907	0.276807	0.362	0.355	1	Treg	Ythdc1
Tmbim4	0.003063	0.327305	0.328	0.32	1	Treg	Tmbim4
Sesn3	0.004634	0.255412	0.282	0.263	1	Treg	Sesn3
Tmem179b	0.005243	0.298371	0.44	0.449	1	Treg	Tmem179b
Yipf1	0.007369	0.426693	0.269	0.262	1	Treg	Yipf1
Txndc17	0.007436	0.289029	0.386	0.388	1	Treg	Txndc17
Zup1	0.009298	0.40705	0.259	0.249	1	Treg	Zup1
Yaf2	0.009889	0.339922	0.257	0.247	1	Treg	Yaf2
Ly86	0	5.830535	0.888	0.069	0	Mg-ACT	Ly86
Csf1r	0	5.535842	0.822	0.067	0	Mg-ACT	Csf1r
Ctsh	0	5.4843	0.779	0.032	0	Mg-ACT	Ctsh
Pld4	0	6.147356	0.746	0.017	0	Mg-ACT	Pld4
Lgmn	0	5.659654	0.809	0.089	0	Mg-ACT	Lgmn
Sirpa	0	4.893822	0.758	0.039	0	Mg-ACT	Sirpa
Spi1	0	5.736812	0.736	0.02	0	Mg-ACT	Spi1
Tyrobp	0	2.423753	0.917	0.209	0	Mg-ACT	Tyrobp
Cd68	0	5.528727	0.741	0.037	0	Mg-ACT	Cd68
Grn	0	4.495695	0.859	0.17	0	Mg-ACT	Grn
Unc93b1	0	3.85477	0.839	0.15	0	Mg-ACT	Unc93b1
C1qc	0	6.261764	0.796	0.108	0	Mg-ACT	C1qc
Lyn	0	3.491237	0.791	0.107	0	Mg-ACT	Lyn
Mpeg1	0	4.890222	0.718	0.035	0	Mg-ACT	Mpeg1
Aif1	0	5.068014	0.723	0.041	0	Mg-ACT	Aif1
C1qa	0	6.188406	0.803	0.124	0	Mg-ACT	C1qa
Marcks	0	4.227781	0.748	0.069	0	Mg-ACT	Marcks
Trem2	0	5.64278	0.71	0.041	0	Mg-ACT	Trem2

Fcer1g	0	1.947125	0.902	0.237	0	Mg-ACT	Fcer1g
Ctsz	0	4.252429	0.868	0.203	0	Mg-ACT	Ctsz
C1qb	0	6.069787	0.811	0.147	0	Mg-ACT	C1qb
Lyz2	0	5.555933	0.727	0.078	0	Mg-ACT	Lyz2
Fcgr3	0	4.612356	0.703	0.055	0	Mg-ACT	Fcgr3
H2-Ab1	0	6.513344	0.719	0.074	0	Mg-ACT	H2-Ab1
Cx3cr1	0	4.446847	0.743	0.1	0	Mg-ACT	Cx3cr1
Lpcat2	0	4.261894	0.696	0.055	0	Mg-ACT	Lpcat2
Alox5ap	0	5.788564	0.656	0.018	0	Mg-ACT	Alox5ap
Itgb5	0	4.817496	0.687	0.049	0	Mg-ACT	Itgb5
Cd300c2	0	5.343711	0.647	0.019	0	Mg-ACT	Cd300c2
Lair1	0	4.599977	0.669	0.043	0	Mg-ACT	Lair1
Gab2	0	3.74071	0.673	0.054	0	Mg-ACT	Gab2
Ctsl	0	4.676499	0.685	0.069	0	Mg-ACT	Ctsl
Slc8a1	0	4.182877	0.669	0.058	0	Mg-ACT	Slc8a1
Mef2c	0	3.910234	0.664	0.054	0	Mg-ACT	Mef2c
H2-Aa	0	6.183648	0.703	0.093	0	Mg-ACT	H2-Aa
Fcgr2b	0	5.36385	0.626	0.018	0	Mg-ACT	Fcgr2b
Hexa	0	3.323572	0.738	0.131	0	Mg-ACT	Hexa
Hck	0	5.874894	0.61	0.01	0	Mg-ACT	Hck
Trf	0	3.614329	0.666	0.069	0	Mg-ACT	Trf
Cd81	0	4.067744	0.775	0.179	0	Mg-ACT	Cd81
P2ry12	0	5.087739	0.644	0.048	0	Mg-ACT	P2ry12
H2-Eb1	0	6.214907	0.67	0.079	0	Mg-ACT	H2-Eb1
Mafb	0	5.00914	0.623	0.033	0	Mg-ACT	Mafb
Zfhx3	0	4.078067	0.638	0.05	0	Mg-ACT	Zfhx3
Basp1	0	3.901383	0.639	0.053	0	Mg-ACT	Basp1
Zeb2	0	2.676154	0.747	0.162	0	Mg-ACT	Zeb2
Cd74	0	6.036755	0.831	0.246	0	Mg-ACT	Cd74
H2-DMb1	0	5.93734	0.604	0.02	0	Mg-ACT	H2-DMb1
P2ry6	0	5.998097	0.592	0.01	0	Mg-ACT	P2ry6
Dock4	0	3.728111	0.637	0.058	0	Mg-ACT	Dock4
Olfml3	0	5.877277	0.603	0.027	0	Mg-ACT	Olfml3
Plekho1	0	3.49859	0.63	0.056	0	Mg-ACT	Plekho1
Cd9	0	3.250033	0.738	0.164	0	Mg-ACT	Cd9
Lrmda	0	3.318874	0.685	0.112	0	Mg-ACT	Lrmda
Rhob	0	3.97078	0.648	0.08	0	Mg-ACT	Rhob
Cyfip1	0	2.595199	0.705	0.141	0	Mg-ACT	Cyfip1
Selenop	0	3.107355	0.778	0.217	0	Mg-ACT	Selenop
Anxa3	0	4.415033	0.59	0.029	0	Mg-ACT	Anxa3
Epb41l2	0	2.789694	0.715	0.155	0	Mg-ACT	Epb41l2
Asah1	0	2.856556	0.662	0.103	0	Mg-ACT	Asah1
Ifi30	0	3.745081	0.724	0.168	0	Mg-ACT	Ifi30
H2-DMa	0	2.989721	0.73	0.179	0	Mg-ACT	H2-DMa

Ncf1	0	3.870184	0.592	0.043	0	Mg-ACT	Ncf1
Plxdc2	0	3.389636	0.669	0.121	0	Mg-ACT	Plxdc2
Mertk	0	4.304751	0.591	0.045	0	Mg-ACT	Mertk
Fcgr1	0	6.134459	0.548	0.008	0	Mg-ACT	Fcgr1
Tbxas1	0	4.738463	0.559	0.02	0	Mg-ACT	Tbxas1
Fgd2	0	5.179262	0.551	0.013	0	Mg-ACT	Fgd2
Gns	0	2.882388	0.633	0.096	0	Mg-ACT	Gns
Ctsc	0	3.106038	0.762	0.226	0	Mg-ACT	Ctsc
Qk	0	2.180739	0.774	0.239	0	Mg-ACT	Qk
Sparc	0	3.949489	0.662	0.128	0	Mg-ACT	Sparc
Tmem119	0	5.368331	0.557	0.024	0	Mg-ACT	Tmem119
Ptgs1	0	4.794407	0.563	0.031	0	Mg-ACT	Ptgs1
App	0	2.371704	0.621	0.092	0	Mg-ACT	App
Tanc2	0	3.508332	0.628	0.1	0	Mg-ACT	Tanc2
Skap2	0	2.59634	0.629	0.102	0	Mg-ACT	Skap2
Abhd12	0	2.668017	0.697	0.171	0	Mg-ACT	Abhd12
Cd180	0	5.085258	0.54	0.015	0	Mg-ACT	Cd180
Itgam	0	3.497758	0.566	0.041	0	Mg-ACT	Itgam
Siglech	0	5.193427	0.55	0.026	0	Mg-ACT	Siglech
Ppfia4	0	5.194649	0.535	0.013	0	Mg-ACT	Ppfia4
Ctss	0	4.014317	0.942	0.42	0	Mg-ACT	Ctss
Tmcc3	0	3.765858	0.581	0.059	0	Mg-ACT	Tmcc3
Plek	0	2.405506	0.654	0.133	0	Mg-ACT	Plek
F11r	0	5.118989	0.533	0.017	0	Mg-ACT	F11r
Kctd12	0	2.670306	0.607	0.093	0	Mg-ACT	Kctd12
Ifngr2	0	3.277261	0.552	0.04	0	Mg-ACT	Ifngr2
Rrbp1	0	1.928406	0.8	0.295	0	Mg-ACT	Rrbp1
Tmem176b	0	2.07586	0.632	0.127	0	Mg-ACT	Tmem176b
Frmd4a	0	3.254645	0.566	0.063	0	Mg-ACT	Frmd4a
Pkig	0	3.07438	0.572	0.07	0	Mg-ACT	Pkig
Lamp2	0	2.17896	0.662	0.16	0	Mg-ACT	Lamp2
Man2b1	0	2.189999	0.747	0.247	0	Mg-ACT	Man2b1
Lrp1	0	3.508192	0.546	0.046	0	Mg-ACT	Lrp1
Cd63	0	3.569125	0.586	0.088	0	Mg-ACT	Cd63
Irf8	0	1.911108	0.669	0.174	0	Mg-ACT	Irf8
Tgfbr1	0	2.462188	0.726	0.232	0	Mg-ACT	Tgfbr1
Csf2ra	0	4.040146	0.527	0.034	0	Mg-ACT	Csf2ra
Lamp1	0	2.459603	0.847	0.357	0	Mg-ACT	Lamp1
Tns3	0	3.868606	0.519	0.029	0	Mg-ACT	Tns3
Cfh	0	4.001124	0.52	0.031	0	Mg-ACT	Cfh
Ncf2	0	4.210853	0.511	0.022	0	Mg-ACT	Ncf2
Creg1	0	2.371096	0.636	0.147	0	Mg-ACT	Creg1
Irf5	0	2.902534	0.559	0.071	0	Mg-ACT	Irf5
Bmp2k	0	2.235791	0.62	0.132	0	Mg-ACT	Bmp2k

Cd86	0	2.346525	0.596	0.108	0	Mg-ACT	Cd86
Srgap2	0	2.185665	0.731	0.244	0	Mg-ACT	Srgap2
Adap2	0	4.730445	0.504	0.017	0	Mg-ACT	Adap2
Slco2b1	0	4.655854	0.508	0.022	0	Mg-ACT	Slco2b1
Prkcd	0	2.353166	0.605	0.12	0	Mg-ACT	Prkcd
Glul	0	2.164582	0.709	0.227	0	Mg-ACT	Glul
Frmd4b	0	2.805012	0.57	0.088	0	Mg-ACT	Frmd4b
Lst1	0	3.838987	0.532	0.051	0	Mg-ACT	Lst1
Hpgds	0	3.545778	0.52	0.042	0	Mg-ACT	Hpgds
Camk1	0	3.970011	0.51	0.034	0	Mg-ACT	Camk1
Gatm	0	4.587374	0.501	0.027	0	Mg-ACT	Gatm
Apoe	0	4.533021	0.775	0.302	0	Mg-ACT	Apoe
Serpine2	0	3.770901	0.521	0.049	0	Mg-ACT	Serpine2
Ophn1	0	3.86898	0.507	0.035	0	Mg-ACT	Ophn1
Acer3	0	2.73736	0.538	0.067	0	Mg-ACT	Acer3
Rnf130	0	1.517992	0.694	0.223	0	Mg-ACT	Rnf130
Jun	0	2.338159	0.651	0.183	0	Mg-ACT	Jun
Ckb	0	1.601895	0.655	0.19	0	Mg-ACT	Ckb
Gusb	0	2.347861	0.598	0.133	0	Mg-ACT	Gusb
Cd14	0	5.98058	0.473	0.008	0	Mg-ACT	Cd14
Ltc4s	0	4.621624	0.49	0.026	0	Mg-ACT	Ltc4s
Rassf4	0	5.001721	0.471	0.011	0	Mg-ACT	Rassf4
Scamp2	0	1.731353	0.73	0.27	0	Mg-ACT	Scamp2
Slc11a1	0	6.001622	0.466	0.008	0	Mg-ACT	Slc11a1
Zfp36	0	2.292623	0.743	0.285	0	Mg-ACT	Zfp36
Fcrls	0	5.053998	0.488	0.031	0	Mg-ACT	Fcrls
Adgre1	0	5.173375	0.467	0.011	0	Mg-ACT	Adgre1
Nrros	0	1.807378	0.615	0.16	0	Mg-ACT	Nrros
Plcg2	0	2.689656	0.511	0.058	0	Mg-ACT	Plcg2
Pik3ap1	0	2.257384	0.55	0.098	0	Mg-ACT	Pik3ap1
Cd33	0	4.86057	0.465	0.013	0	Mg-ACT	Cd33
Cd83	0	3.530942	0.502	0.05	0	Mg-ACT	Cd83
Ptprj	0	1.658596	0.65	0.2	0	Mg-ACT	Ptprj
Ifitm3	0	2.26118	0.621	0.174	0	Mg-ACT	Ifitm3
Etv6	0	1.548968	0.665	0.22	0	Mg-ACT	Etv6
Erp29	0	1.828053	0.769	0.324	0	Mg-ACT	Erp29
Sat1	0	1.825157	0.774	0.333	0	Mg-ACT	Sat1
Daglb	0	3.026733	0.496	0.056	0	Mg-ACT	Daglb
Stx7	0	1.815878	0.571	0.131	0	Mg-ACT	Stx7
Dst	0	2.645741	0.529	0.09	0	Mg-ACT	Dst
Rapgef5	0	3.684634	0.487	0.05	0	Mg-ACT	Rapgef5
Gpr34	0	3.315672	0.538	0.103	0	Mg-ACT	Gpr34
Chd9	0	1.391265	0.752	0.317	0	Mg-ACT	Chd9
Lmo2	0	3.744558	0.454	0.02	0	Mg-ACT	Lmo2

Rnase4	0	2.952619	0.496	0.063	0	Mg-ACT	Rnase4
Gnaq	0	1.79442	0.563	0.13	0	Mg-ACT	Gnaq
Crybb1	0	5.46803	0.447	0.016	0	Mg-ACT	Crybb1
Atp6v0b	0	1.505215	0.802	0.372	0	Mg-ACT	Atp6v0b
Slc29a3	0	3.1839	0.469	0.039	0	Mg-ACT	Slc29a3
Pnp	0	1.913385	0.629	0.199	0	Mg-ACT	Pnp
Fcgrt	0	4.135473	0.451	0.022	0	Mg-ACT	Fcgrt
Zfp710	0	2.870924	0.481	0.053	0	Mg-ACT	Zfp710
Cttnbp2nl	0	4.063747	0.448	0.021	0	Mg-ACT	Cttnbp2nl
Blnk	0	4.552476	0.441	0.014	0	Mg-ACT	Blnk
Il6ra	0	2.609042	0.475	0.05	0	Mg-ACT	Il6ra
Mef2a	0	1.441302	0.721	0.296	0	Mg-ACT	Mef2a
E230029C(	0	3.655427	0.461	0.037	0	Mg-ACT	E230029C05Rik
Tcirg1	0	1.879531	0.548	0.124	0	Mg-ACT	Tcirg1
Cd300a	0	5.415441	0.429	0.006	0	Mg-ACT	Cd300a
Dleu2	0	1.461342	0.649	0.227	0	Mg-ACT	Dleu2
Entpd1	0	1.5882	0.609	0.187	0	Mg-ACT	Entpd1
Ctsa	0	2.014689	0.766	0.345	0	Mg-ACT	Ctsa
Maml3	0	3.260279	0.474	0.054	0	Mg-ACT	Maml3
Ntpcr	0	2.630387	0.501	0.081	0	Mg-ACT	Ntpcr
Tcn2	0	3.035031	0.467	0.048	0	Mg-ACT	Tcn2
Gna12	0	2.410079	0.503	0.084	0	Mg-ACT	Gna12
Arhgap5	0	2.357171	0.503	0.084	0	Mg-ACT	Arhgap5
Pou2f2	0	2.038863	0.518	0.1	0	Mg-ACT	Pou2f2
Hpgd	0	4.453463	0.437	0.02	0	Mg-ACT	Hpgd
Scpep1	0	2.956898	0.472	0.056	0	Mg-ACT	Scpep1
Rreb1	0	1.877982	0.525	0.113	0	Mg-ACT	Rreb1
Ccl12	0	6.95397	0.428	0.016	0	Mg-ACT	Ccl12
Plxnb2	0	3.798584	0.435	0.023	0	Mg-ACT	Plxnb2
Fos	0	2.649153	0.554	0.143	0	Mg-ACT	Fos
Csf3r	0	4.718351	0.421	0.01	0	Mg-ACT	Csf3r
Wdfy3	0	3.614909	0.437	0.026	0	Mg-ACT	Wdfy3
Bin1	0	1.69218	0.691	0.28	0	Mg-ACT	Bin1
Ldhb	0	1.778272	0.561	0.151	0	Mg-ACT	Ldhb
Phyhd1	0	4.256291	0.43	0.021	0	Mg-ACT	Phyhd1
Pros1	0	3.534744	0.442	0.034	0	Mg-ACT	Pros1
Tcf4	0	2.137968	0.485	0.077	0	Mg-ACT	Tcf4
Timp21	0	2.158841	0.568	0.16	0	Mg-ACT	Timp2
Ctsb	0	3.051466	0.913	0.506	0	Mg-ACT	Ctsb
Diaph2	0	1.425461	0.615	0.208	0	Mg-ACT	Diaph2
Tpd52	0	2.301963	0.486	0.08	0	Mg-ACT	Tpd52
1-Mar	0	3.708638	0.428	0.022	0	Mg-ACT	1-Mar
Tmem86a	0	4.308144	0.424	0.02	0	Mg-ACT	Tmem86a
Rbm47	0	3.048872	0.441	0.038	0	Mg-ACT	Rbm47

Gm2a1	0	1.700099	0.578	0.175	0	Mg-ACT	Gm2a
Cebpb	0	1.808554	0.63	0.228	0	Mg-ACT	Cebpb
Rad51b	0	2.541493	0.49	0.089	0	Mg-ACT	Rad51b
Nfe2l2	0	1.668162	0.557	0.157	0	Mg-ACT	Nfe2l2
Bri3	0	1.309782	0.72	0.32	0	Mg-ACT	Bri3
Il10ra1	0	1.139357	0.679	0.281	0	Mg-ACT	Il10ra
Abi3	0	1.683878	0.567	0.17	0	Mg-ACT	Abi3
Inpp5d	0	1.424774	0.803	0.407	0	Mg-ACT	Inpp5d
Gngt2	0	2.033757	0.518	0.122	0	Mg-ACT	Gngt2
Rnf13	0	1.334515	0.586	0.19	0	Mg-ACT	Rnf13
Nav2	0	2.516542	0.511	0.117	0	Mg-ACT	Nav2
Pip4k2a	0	1.113441	0.697	0.303	0	Mg-ACT	Pip4k2a
Cxcl16	0	5.081274	0.408	0.014	0	Mg-ACT	Cxcl16
Pip4p1	0	1.409251	0.539	0.146	0	Mg-ACT	Pip4p1
Comt	0	1.367	0.609	0.216	0	Mg-ACT	Comt
Pla2g15	0	4.071741	0.414	0.021	0	Mg-ACT	Pla2g15
Pid1	0	4.032712	0.418	0.026	0	Mg-ACT	Pid1
Atp6ap2	0	1.418963	0.586	0.194	0	Mg-ACT	Atp6ap2
Mir99ahg	0	3.160696	0.441	0.05	0	Mg-ACT	Mir99ahg
Rgs101	0	1.281763	0.815	0.424	0	Mg-ACT	Rgs10
Slc7a8	0	3.843365	0.412	0.021	0	Mg-ACT	Slc7a8
Tmem176a	0	1.712004	0.479	0.089	0	Mg-ACT	Tmem176a
Prcp	0	2.342067	0.444	0.057	0	Mg-ACT	Prcp
Ccl6	0	5.356255	0.399	0.012	0	Mg-ACT	Ccl6
Ehd4	0	2.064678	0.484	0.098	0	Mg-ACT	Ehd4
Lap3	0	2.594477	0.473	0.087	0	Mg-ACT	Lap3
Syngr1	0	4.367251	0.409	0.024	0	Mg-ACT	Syngr1
Lag3	0	1.925581	0.515	0.131	0	Mg-ACT	Lag3
Gm43305	0	1.803076	0.554	0.17	0	Mg-ACT	Gm43305
Abca1	0	2.942078	0.437	0.054	0	Mg-ACT	Abca1
Ptms	0	1.2875	0.67	0.288	0	Mg-ACT	Ptms
Cltc	0	1.11967	0.665	0.283	0	Mg-ACT	Cltc
Tnfrsf1a	0	1.274735	0.552	0.17	0	Mg-ACT	Tnfrsf1a
Rogdi	0	3.013945	0.423	0.042	0	Mg-ACT	Rogdi
Gng10	0	1.516971	0.712	0.331	0	Mg-ACT	Gng10
Gpx1	0	2.128136	0.827	0.446	0	Mg-ACT	Gpx1
Capza2	0	1.142628	0.783	0.405	0	Mg-ACT	Capza2
Mtus1	0	3.942749	0.395	0.017	0	Mg-ACT	Mtus1
C3ar1	0	5.228785	0.386	0.009	0	Mg-ACT	C3ar1
Ogfrl1	0	3.005436	0.412	0.037	0	Mg-ACT	Ogfrl1
Rasa4	0	3.104213	0.404	0.029	0	Mg-ACT	Rasa4
Scarb2	0	1.246347	0.571	0.196	0	Mg-ACT	Scarb2
Scoc	0	2.286143	0.455	0.081	0	Mg-ACT	Scoc
Nckap1l	0	1.26395	0.55	0.177	0	Mg-ACT	Nckap1l

Man1c1	0	2.812179	0.413	0.041	0	Mg-ACT	Man1c1
Mitf	0	2.7035	0.417	0.045	0	Mg-ACT	Mitf
Ppt1	0	1.744805	0.524	0.153	0	Mg-ACT	Ppt1
Rnf180	0	4.376268	0.386	0.015	0	Mg-ACT	Rnf180
Mt1	0	1.2321	0.574	0.203	0	Mg-ACT	Mt1
Specc1	0	2.705234	0.412	0.042	0	Mg-ACT	Specc1
Slc7a7	0	4.491082	0.38	0.01	0	Mg-ACT	Slc7a7
Nfam1	0	4.91105	0.377	0.007	0	Mg-ACT	Nfam1
Rab3il1	0	4.295764	0.383	0.013	0	Mg-ACT	Rab3il1
Laptm4a	0	1.116215	0.795	0.426	0	Mg-ACT	Laptm4a
Apobec1	0	2.595808	0.433	0.064	0	Mg-ACT	Apobec1
Lrrc25	0	4.436028	0.378	0.009	0	Mg-ACT	Lrrc25
Tbc1d9	0	2.837056	0.411	0.042	0	Mg-ACT	Tbc1d9
Syk	0	2.603071	0.403	0.034	0	Mg-ACT	Syk
Ms4a6c	0	3.084595	0.442	0.074	0	Mg-ACT	Ms4a6c
Numb	0	1.554008	0.551	0.184	0	Mg-ACT	Numb
Vsir	0	1.230484	0.682	0.317	0	Mg-ACT	Vsir
Snx29	0	1.914423	0.467	0.102	0	Mg-ACT	Snx29
Rin2	0	3.407191	0.391	0.026	0	Mg-ACT	Rin2
Tnfrsf11a	0	4.246421	0.377	0.013	0	Mg-ACT	Tnfrsf11a
Cadm1	0	3.800337	0.394	0.03	0	Mg-ACT	Cadm1
Tifab	0	5.281152	0.37	0.006	0	Mg-ACT	Tifab
Soga1	0	4.393912	0.375	0.013	0	Mg-ACT	Soga1
Sall1	0	4.362319	0.377	0.015	0	Mg-ACT	Sall1
Cd2ap	0	1.623015	0.457	0.095	0	Mg-ACT	Cd2ap
Fkbp2	0	1.315717	0.58	0.218	0	Mg-ACT	Fkbp2
Fes	0	2.974548	0.391	0.031	0	Mg-ACT	Fes
Clasp2	0	1.318695	0.508	0.148	0	Mg-ACT	Clasp2
Asph	0	3.407891	0.388	0.029	0	Mg-ACT	Asph
Gm10790	0	4.482616	0.373	0.014	0	Mg-ACT	Gm10790
Psap	0	3.052342	0.963	0.606	0	Mg-ACT	Psap
Golm1	0	1.669981	0.514	0.157	0	Mg-ACT	Golm1
H2-Oa	0	3.263716	0.417	0.06	0	Mg-ACT	H2-Oa
Cybb	0	4.437353	0.38	0.023	0	Mg-ACT	Cybb
Adam15	0	3.165406	0.385	0.029	0	Mg-ACT	Adam15
Tmed3	0	1.349028	0.538	0.184	0	Mg-ACT	Tmed3
Ighm1	0	0.956046	0.522	0.168	0	Mg-ACT	Ighm
Rasgrp3	0	4.030166	0.37	0.016	0	Mg-ACT	Rasgrp3
Arhgap17	0	1.159292	0.529	0.176	0	Mg-ACT	Arhgap17
Tpp1	0	1.689196	0.485	0.132	0	Mg-ACT	Tpp1
0610040J0	0	4.598189	0.364	0.012	0	Mg-ACT	0610040J01Rik
Dnase2a	0	1.820885	0.445	0.093	0	Mg-ACT	Dnase2a
Abca9	0	3.881923	0.369	0.017	0	Mg-ACT	Abca9
Havcr2	0	2.785712	0.393	0.042	0	Mg-ACT	Havcr2

Wdfy4	0	4.757247	0.36	0.01	0	Mg-ACT	Wdfy4
Rgs2	0	1.311373	0.462	0.113	0	Mg-ACT	Rgs2
Atp8a1	0	1.143849	0.525	0.176	0	Mg-ACT	Atp8a1
Snx18	0	1.490356	0.462	0.115	0	Mg-ACT	Snx18
Fmnl2	0	3.475484	0.375	0.028	0	Mg-ACT	Fmnl2
Zeb2os	0	4.76176	0.354	0.008	0	Mg-ACT	Zeb2os
Vkorc1	0	1.38294	0.515	0.169	0	Mg-ACT	Vkorc1
Sdf2l1	0	1.483268	0.551	0.206	0	Mg-ACT	Sdf2l1
Spint1	0	6.342076	0.348	0.004	0	Mg-ACT	Spint1
Rab31	0	3.302623	0.369	0.025	0	Mg-ACT	Rab31
Cmtm6	0	1.312701	0.481	0.138	0	Mg-ACT	Cmtm6
Slc1a3	0	3.014303	0.377	0.036	0	Mg-ACT	Slc1a3
Csf2rb	0	5.253806	0.346	0.005	0	Mg-ACT	Csf2rb
Cux1	0	1.036386	0.515	0.175	0	Mg-ACT	Cux1
Ivns1abp	0	1.300986	0.502	0.162	0	Mg-ACT	Ivns1abp
Rnpep	0	1.267688	0.496	0.157	0	Mg-ACT	Rnpep
Cd302	0	4.228287	0.35	0.012	0	Mg-ACT	Cd302
Gng12	0	1.677536	0.434	0.096	0	Mg-ACT	Gng12
Gm2629	0	3.47095	0.365	0.027	0	Mg-ACT	Gm2629
Mfsd1	0	1.404405	0.461	0.127	0	Mg-ACT	Mfsd1
Cebpa	0	4.640198	0.343	0.009	0	Mg-ACT	Cebpa
Gtf2h2	0	1.837839	0.436	0.102	0	Mg-ACT	Gtf2h2
Lrrk1	0	1.210851	0.469	0.135	0	Mg-ACT	Lrrk1
Heatr5a	0	2.013959	0.399	0.068	0	Mg-ACT	Heatr5a
Ms4a6d	0	2.952755	0.368	0.037	0	Mg-ACT	Ms4a6d
Lat2	0	1.523174	0.431	0.1	0	Mg-ACT	Lat2
P2ry13	0	4.956183	0.337	0.007	0	Mg-ACT	P2ry13
Large1	0	1.592734	0.384	0.054	0	Mg-ACT	Large1
Csnk1e	0	1.817868	0.405	0.075	0	Mg-ACT	Csnk1e
Zfp385a	0	3.936215	0.343	0.013	0	Mg-ACT	Zfp385a
Bmyc1	0	0.95467	0.48	0.15	0	Mg-ACT	Bmyc
Pak1	0	3.70437	0.347	0.017	0	Mg-ACT	Pak1
Cpq	0	1.883851	0.397	0.068	0	Mg-ACT	Cpq
Cst3	0	4.650593	0.997	0.668	0	Mg-ACT	Cst3
Snx24	0	4.180149	0.344	0.015	0	Mg-ACT	Snx24
Lhfpl2	0	4.31885	0.342	0.014	0	Mg-ACT	Lhfpl2
Colgalt1	0	1.326131	0.446	0.119	0	Mg-ACT	Colgalt1
Dapp1	0	1.154441	0.469	0.143	0	Mg-ACT	Dapp1
Slc12a9	0	2.321749	0.369	0.044	0	Mg-ACT	Slc12a9
Atf3	0	4.630988	0.338	0.014	0	Mg-ACT	Atf3
BC028528	0	5.210325	0.329	0.005	0	Mg-ACT	BC028528
Gal3st4	0	5.570276	0.329	0.005	0	Mg-ACT	Gal3st4
Plcl1	0	2.180695	0.406	0.083	0	Mg-ACT	Plcl1
Hps4	0	2.206214	0.38	0.057	0	Mg-ACT	Hps4

Rnf216	0	1.292008	0.436	0.116	0	Mg-ACT	Rnf216
St3gal5	0	2.769724	0.361	0.041	0	Mg-ACT	St3gal5
Nav3	0	3.290757	0.372	0.052	0	Mg-ACT	Nav3
Tnfrsf21	0	4.106758	0.333	0.013	0	Mg-ACT	Tnfrsf21
Apba1	0	4.084235	0.335	0.015	0	Mg-ACT	Apba1
Rp2	0	2.2831	0.37	0.05	0	Mg-ACT	Rp2
Arhgap39	0	1.718481	0.411	0.091	0	Mg-ACT	Arhgap39
Cacnb2	0	3.973238	0.341	0.022	0	Mg-ACT	Cacnb2
Acp2	0	2.73302	0.356	0.037	0	Mg-ACT	Acp2
Sdc3	0	3.207813	0.344	0.025	0	Mg-ACT	Sdc3
Slc6a6	0	1.08992	0.46	0.143	0	Mg-ACT	Slc6a6
Aldh2	0	2.509183	0.369	0.052	0	Mg-ACT	Aldh2
Ptpro	0	4.54761	0.325	0.008	0	Mg-ACT	Ptpro
Naga	0	1.701162	0.406	0.09	0	Mg-ACT	Naga
Idh2	0	1.49058	0.427	0.111	0	Mg-ACT	Idh2
Sipa1l2	0	2.794839	0.355	0.039	0	Mg-ACT	Sipa1l2
Klf3	0	0.763072	0.44	0.125	0	Mg-ACT	Klf3
Plod3	0	2.710685	0.354	0.039	0	Mg-ACT	Plod3
4933406I1ߦ	0	1.685894	0.451	0.137	0	Mg-ACT	4933406I18Rik
Egr1	0	2.410701	0.39	0.076	0	Mg-ACT	Egr1
Ctsf	0	4.034295	0.327	0.013	0	Mg-ACT	Ctsf
Cmtm3	0	2.36699	0.355	0.042	0	Mg-ACT	Cmtm3
Leprot	0	1.151726	0.444	0.132	0	Mg-ACT	Leprot
Hdac9	0	3.006049	0.347	0.035	0	Mg-ACT	Hdac9
Blvrb	0	1.991531	0.367	0.057	0	Mg-ACT	Blvrb
Cyb561a3	0	2.014471	0.372	0.062	0	Mg-ACT	Cyb561a3
Tor3a	0	1.30186	0.417	0.108	0	Mg-ACT	Tor3a
Lacc1	0	4.027961	0.321	0.012	0	Mg-ACT	Lacc1
Stmn1	0	1.264466	0.459	0.151	0	Mg-ACT	Stmn1
Plod1	0	2.944952	0.337	0.029	0	Mg-ACT	Plod1
Hk3	0	4.556776	0.316	0.008	0	Mg-ACT	Hk3
Atp6v0c	0	1.384232	0.847	0.539	0	Mg-ACT	Atp6v0c
P2rx4	0	2.050368	0.363	0.056	0	Mg-ACT	P2rx4
Arhgap22	0	4.400354	0.318	0.011	0	Mg-ACT	Arhgap22
Stard9	0	3.711736	0.321	0.015	0	Mg-ACT	Stard9
Extl3	0	1.814824	0.389	0.083	0	Mg-ACT	Extl3
Ints6l	0	2.018932	0.367	0.061	0	Mg-ACT	Ints6l
Itga6	0	1.718782	0.395	0.091	0	Mg-ACT	Itga6
Tifa	0	3.432923	0.323	0.019	0	Mg-ACT	Tifa
Pacsin2	0	1.232513	0.425	0.122	0	Mg-ACT	Pacsin2
Fmnl3	0	1.392857	0.418	0.115	0	Mg-ACT	Fmnl3
Cnpy3	0	1.069385	0.424	0.121	0	Mg-ACT	Cnpy3
Mknk1	0	1.356877	0.405	0.103	0	Mg-ACT	Mknk1
Fscn1	0	4.212083	0.317	0.015	0	Mg-ACT	Fscn1

Ebi3	0	1.927239	0.358	0.056	0	Mg-ACT	Ebi3
Ccdc88a	0	2.77722	0.332	0.031	0	Mg-ACT	Ccdc88a
Sypl	0	1.231611	0.406	0.105	0	Mg-ACT	Sypl
Snx10	0	1.699215	0.372	0.071	0	Mg-ACT	Snx10
Mgat4a	0	1.458628	0.401	0.101	0	Mg-ACT	Mgat4a
Ctc1	0	2.428076	0.351	0.051	0	Mg-ACT	Ctc1
Nf1	0	1.578569	0.384	0.084	0	Mg-ACT	Nf1
[truncated: 515,230 more chars]
