# Supplementary figures and images for: Pediatric glioma immune profiling identifies TIM3 as a therapeutic target in BRAF fusion pilocytic astrocytoma
Source: J Clin Invest. 2024 Aug 13;134(19):e177413. doi: 10.1172/JCI177413 (PMC11444160; doi:10.1172/JCI177413)

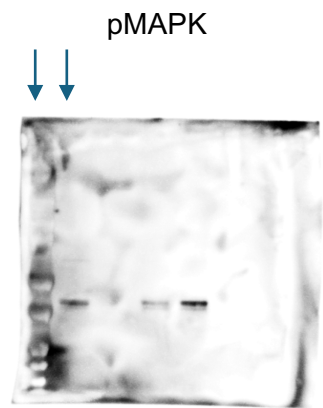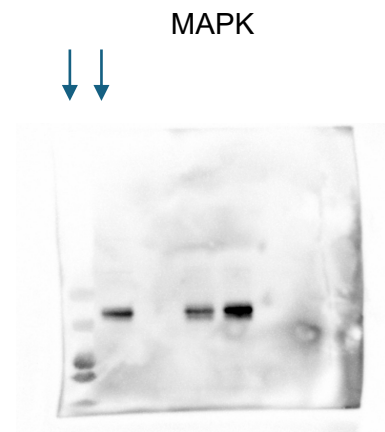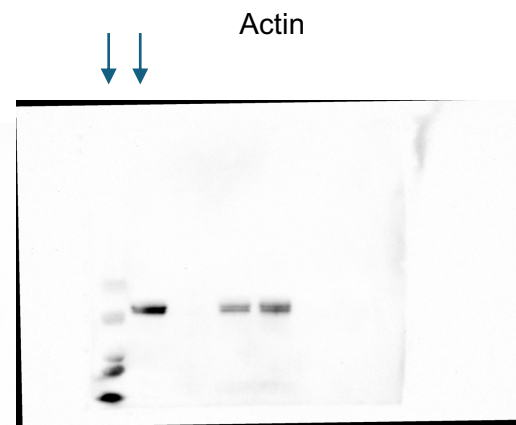

Supplement: Unedited blot and gel images [file jci-134-177413-s014.pdf]
